# TAFA4-IL-10 axis potentiate immunotherapy for airway allergy by induction of specific regulatory T cells

**DOI:** 10.1038/s41541-022-00559-w

**Published:** 2022-10-31

**Authors:** Shuyao Qiu, Xiangqian Luo, Lihua Mo, Shuang Zhang, Yun Liao, Li Guan, Liteng Yang, Qinmiao Huang, Dabo Liu, Pingchang Yang

**Affiliations:** 1 Department of Pediatric Otolaryngology, Shenzhen Hospital, Southern Medical University, Shenzhen, China; 2 The Third School of Clinical Medicine, Southern Medical University, Guangzhou, China; 3 Guangdong Provincial Regional Disease Key Laboratory, Shenzhen, China; 4 Institute of Allergy & Immunology of Shenzhen University, State Key Laboratory of Respiratory Diseases Allergy Division at Shenzhen University, Shenzhen, China; 5 Department of Allergy & Respirology, Third Affiliated Hospital of Shenzhen University, Shenzhen, China

**Keywords:** Immunological disorders, Immunological disorders

## Abstract

Allergen-specific immunotherapy (AIT) is the main treatment for allergic diseases. The therapeutic efficacy of AIT has to be improved. Neuropeptides, such as TAFA4, have immune-regulating features. The objective of this study is to promote the efficacy of AIT in experimental allergic rhinitis (AR) by the concurrent use of TAFA chemokine as a family member 4 (TAFA4). In this study, an AR mouse model was developed using ovalbumin (OVA) as the specific antigen. The AR response was assessed in mice after treatment with AIT or/and TAFA4. We found that exposure to TAFA4 activated dendritic cells (DCs) in the airway tissues. Activation of DC by TAFA4 resulted in the expression of IL-10. TAFA4 also promoted the activities of c-Maf inducing protein. The FPR1-MyD88-AKT signal pathway was associated with the TAFA4-induced *Il10* expression in the DCs. Co-administration of AIT/TAFA4 attenuated the AR response in mice by inducing antigen-specific Tr1 cells. In conclusion, TAFA4 induces the expression of IL-10 in DCs. Acting as an adjuvant, TAFA4 significantly improves AIT’s therapeutic efficacy against AR by inducing antigen-specific Tr1 cells.

## Introduction

Airway allergy is an adverse response to innocent airborne antigens by the nervous system in airway tissues. The pathogenesis is still not fully understood. Allergen-specific immunotherapy (AIT) is an etiologic therapy tool for allergic diseases^1^. By introducing small doses of specific antigens into the body, AIT aims to re-establish specific immune tolerance to competent immune antigens against allergic diseases^2^. However, although AIT has been applied in allergy clinics for many years, the incidence of allergic diseases continues to rise worldwide^3^. It therefore appears necessary to improve the therapeutic efficacy of AIT.

Regulatory immune cells, such as regulatory T cells (Treg) and regulatory B cells (Breg), are canonical contributors to immune tolerance^4^. Type 1 regulatory T-cells (Tr1 cells) are one of the major immune regulatory cell fractions. Tr1 cells play an important role in the suppression of aberrant immune response^5^. By releasing IL-10, one of the major mediators of immune regulation, Tr1 cells inhibit other immune cell activities or induce apoptosis of immune cells that act^6^. This characteristic confers the ability of Tr1 cells to suppress the abnormal immune response, such as allergic rhinitis (AR)^7^. Therefore, the promotion of Tr1 cell generation can enhance the effectiveness of AIT. Tr1 cell production methods have to be refined.

The expression of IL-10 in CD4 T cells is the key point in the generation of Tr1 cells. Numerous substances have been tested to induce IL-10. For instance, lipopolysaccharide, CpG and flagellin are inducers of IL-10^8–10^. A number of neuropeptides, such as substance P, CGRP and TAFA chemokine like family member 4 (TAFA4), have immune regulatory functions^11^. TATA4 is also called FAM19A4, can be produced by non-peptidergic C-fiber sensory neurons expressing the Gαi-interacting protein^12^. Because TAFA4 has the capacity to induce IL-10 expression in immune cells^12,13^, we speculate that TAFA4 could contribute to immune regulation by favoring tolerogenic dendritic cells. Thus, in this study, a new therapeutic tool combining AIT and TAFA 4 was developed. The application of AIT/TAFA4 was shown to have better effects on inhibition of experimental AR than the use of AIT alone.

## Results

### TAFA4 activates dendritic cells (DCs) in the airway tissues

**Fig. 1:**
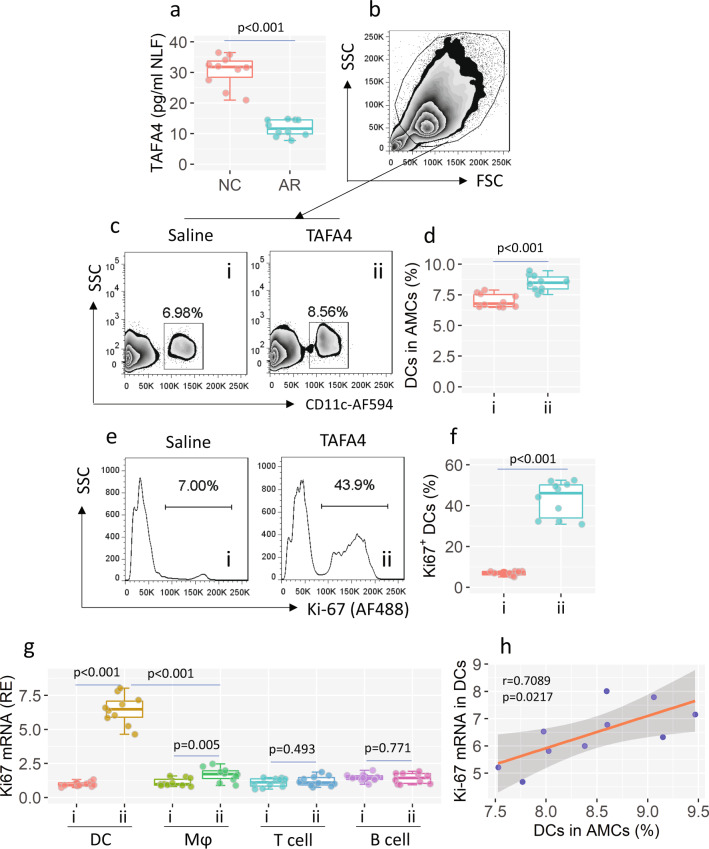
TAFA4 increases and activates DCs in the airway tissues. **a** TAFA4 levels in nasal lavage fluids (NLF). **b**–**h** Mice (10 mice per group) were treated with nasal instillation (containing TAFA4) daily for one week. AMCs were prepared with the airway tissues, and analyzed by FCM. **b** The FCM FSC/SSC plots. **c**, **d** Gated FCM plots show DC counts. Boxplots show DC frequency in AMCs. **e** Gated histograms show Ki-67^+^ DCs in panel **c**. f, boxplots show Ki-67^+^ DC frequency. **g** RNA was extracted from AMC-purified DCs, Mϕs, T cells, B cells, and analyzed by RT-qPCR. Boxplots show the levels of IL-10 mRNA in DCs. **h** Positive correlation between DC counts and Ki-67 mRNA in DCs. Group labels of boxplots are the same as that of FCM plots on the left side. Each dot in boxplots presents data obtained from one sample. Statistics: Two-tail Student’s-*t* test. TAFA4 TAFA chemokine like family member 4, AMC airway mononuclear cell, FCM flow cytometry, DC dendritic cell, Mϕ macrophage. The group labels **a** and **b** of boxplots are the same as those in panel **c**. The data of boxplot are presented as median (middle line inside box), 75% value (upper bound of box), 25% value (lower bound of box), max (upper whisker), and min (lower whisker).

We examined whether TAFA4 could improve immunotherapy against AR. A mouse AR model was first developed (Supplementary Fig. 1 in supplemental materials, data of the AR response are not shown). We found that TAFA4 could be detected in nasal lavage fluid (NLF). The TAFA4 levels in the NLF of AR mice were significantly lower than that of the naive control (NC) mice (Fig. 1a). Naïve mice were then treated with TAFA4 by daily nasal instillations for one week. Airway mononuclear cells (AMCs) were prepared, and analyzed using flow cytometry (FCM). We found that exposure to TAFA4 greatly increased the frequency of DCs in the airway tissues (Fig. 1b–d), and induced considerable activation (increase in Ki-67 expression) of DCs (Fig. 1e–g). Moderate activation was detected in macrophages, but not T cells and B cells, in AMCs (Fig. 1g). A positive correlation was detected in the data between the DC frequency in AMCs and the Ki-67 mRNA levels in DC (Fig. 1h). The results demonstrate that TAFA4 can recruit and activate DCs in the airway tissues.

### TAFA4 induces IL-10 expression in DCs

**Fig. 2:**
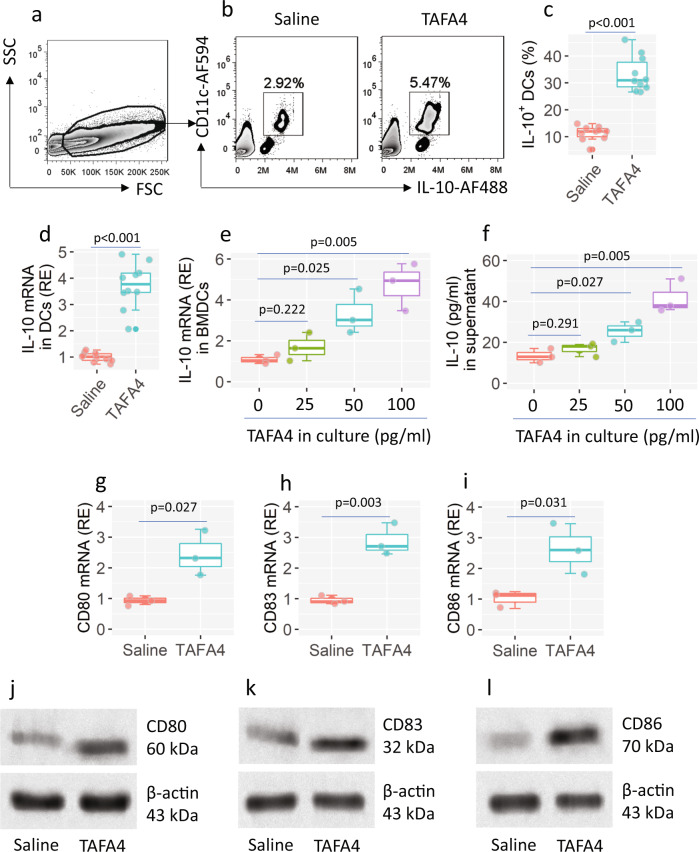
TAFA4 induces IL-10 expression in DCs. **a**–**d** Mice (10 mice per group) were treated with TAFA4 through nasal instillation daily for one week. AMCs were prepared with the airway tissues, and analyzed by FCM. **a** The FSC/SSC plots. **b** Gated FCM plots show IL-10^+^ DC counts. **c** Boxplots show IL-10^+^ DC frequency. **d** Boxplots show IL-10 mRNA levels in purified DCs. **e**, **f** BMDCs were exposed to TAFA4 in culture at indicated concentrations overnight. **e** The IL-10 mRNA levels in BMDCs. **f** The IL-10 levels in culture supernatant. **g**–**i** BMDCs were cultured overnight in the presence of TAFA4 (100 pg/ml). Boxplots show the mRNA levels of CD80, CD83 and CD86 in BMDCs. **j**–**l** The protein levels of CD80, CD83 and CD86 in BMDCs. Statistics: Two-tail Student’s-*t* test (**c**, **d**, **g**–**i**) or ANOVA + Dunnett’s test (**e**, **f**). TAFA4 TAFA chemokine like family member-4, FCM flow cytometry, BMDC bone marrow-derived dendritic cell. The data of boxplot are presented as median (middle line inside box), 75% value (upper bound of box), 25% value (lower bound of box), max (upper whisker), and min (lower whisker). Each dot in boxplot presents data obtained from one sample.

Upon activation, some immune cells, like DCs, express IL-10. Based on the results in Fig. 1, we inferred that TAFA4 might induce the expression IL-10 in the DCs. To test this, we treated naive mice with TAFA4 via nasal instillations daily for one week. The airway tissues were excised upon the sacrifice. AMCs were isolated from the tissues, and analyzed by FCM. The results showed that TAFA4 considerably increased the number of CD11c^+^ IL-10^+^ cells in the airway tissues (Fig. 2a–c), indicating that TAFA4 can induce the expression of IL-10 in DCs. CD11c^+^ DCs were isolated from AMCs, and analyzed by RT-qPCR. Results showed that IL-10 mRNA levels were higher in DCs isolated from mice treated with TAFA4 (Fig. 2d). The data were verified by an in vitro cell culture experiment. Bone marrow-derived DCs (BMDCs) were stimulated with TAFA4 in culture overnight. The IL-10 mRNA levels were increased in BMDCs by TAFA4 on a dose-dependent basis (Fig. 2e). The increase in IL-10 was also found in the culture supernatant (Fig. 2f). In addition, exposure of BMDC to TAFA4 resulted in a moderate increase in the expression of CD80, CD83, and CD86 (Fig. 2g–l). The results demonstrate that TAFA4 can activate DCs and induce the expression of IL-10 in DCs.

### TAFA4 promotes *Il10* expression-related gene activities in DCs

**Fig. 3:**
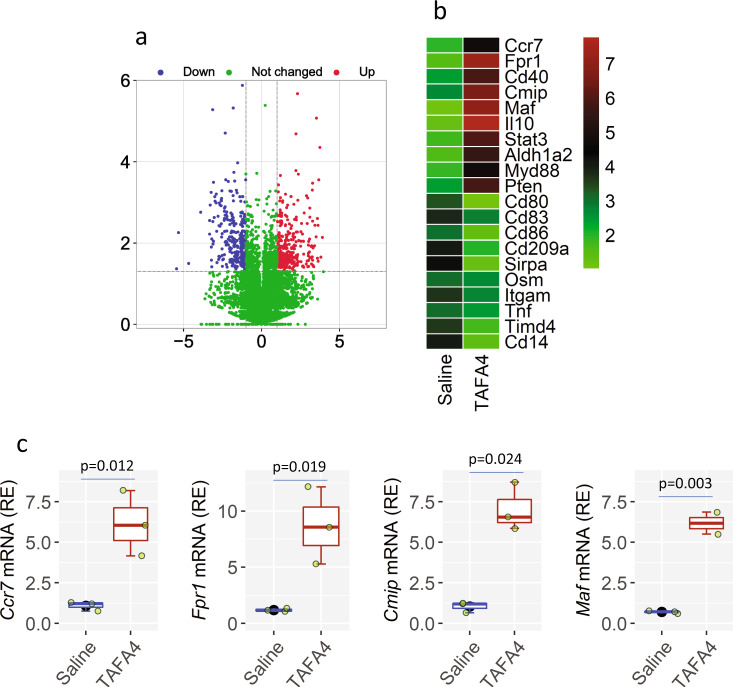
TAFA4 induces the IL10 expression-related gene activation in DCs. **a**, **b** Mice were treated with TAFA4 or saline through nasal instillation daily for 6 days. DCs were isolated from the airway tissues, and analyzed by RNAseq. **a** Volcano plot shows the gene profiles. **b** Heatmap shows the 20 most differentially expressed genes (DEGs). **c** BMDCs were stimulated with TAFA4 in culture overnight, and analyzed by RT-qPCR. Statistics: Two-tail Student’s-*t* test. TAFA4 TAFA chemokine like family member 4, BMDC bone marrow-derived dendritic cell, DEG differentially expressed gene, RNAseq RNA sequencing. The data of boxplot are presented as median (middle line inside box), 75% value (upper bound of box), 25% value (lower bound of box), max (upper whisker), and min (lower whisker). Each dot in boxplot presents data obtained from one sample.

We then looked at the genetic profile of DC after exposure to TAFA4. By RNAseq assay, we found in 15,587 detected genes, 225 genes were upregulated and 185 genes were downregulated (Fig. 3a, b). Genes linked to the expression *Il10*, including *Cmip*, *Maf* and *Il10*, were most active. The activities of *Fpr1*, the receptor of TAFA4, were also markedly up regulated. The results demonstrate that, when exposed to TAFA4, the genetic activities related to *Il10* expression can be regulated. The results were verified by an in vitro experiment. BMDCs were stimulated with TAFA4 in culture overnight. The gene activities of *Fpr1*, *Il10*, *Cmip*, and *Maf* were significantly increased (Fig. 3c).

### The FPR1-MyD88-AKT signal pathway is associated with the TAFA4-induced *Il10* expression

**Fig. 4:**
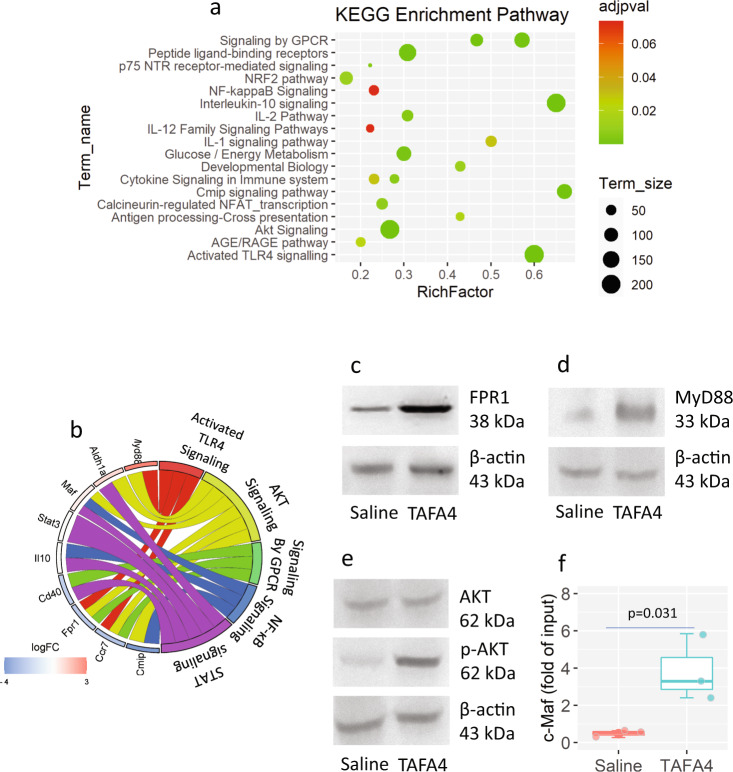
Assessment of TAFA4-activated signal pathway in DCs. Mice were treated with TAFA4-containing nasal instillation daily for one week. DCs were isolated from the airway tissues, and analyzed by RNAseq and Western blotting. **a** 20 most enriched signaling pathways in DCs. **b** GO analysis results show DEGs and signaling pathway mapping. **c**–**e** Immunoblots show protein levels of FPR1, MyD88 and AKT, in DCs. **f** Boxplots show the levels of c-Maf in the *Il10* promoter in DCs. Statistics: Two-tail Student-*t* test. The data represent six independent experiments. The data of boxplot are presented as median (middle line inside box), 75% value (upper bound of box), 25% value (lower bound of box), max (upper whisker), and min (lower whisker). Each dot in boxplot presents data obtained from one sample.

Through the DEG signal pathway enrichment approach with the KEGG database, we found that exposure to TAFA4 activated several signaling pathways associated with IL-10 expression in DCs. The most enriched pathways include, sequentially, IL-10 signaling, CMIP signaling, TLR4 signaling, and signaling by GPCR (G protein-coupled receptor) (Fig. 4a). The gene ontology (GO) analysis showed the mapping routes between the DEGs and the enriched signaling pathways. *Myd88*, *Fpr1,* and *Ccr7* mapped to the activated TLR4 pathway. *Cmip*, *Ccr7*, *Fpr1*, *Maf*, and *Myd88* mapped to the AKT pathway. *Cd40*, *Ccr7,* and *Cmip* mapped to the GPCR pathway. Maf, Il10, and Cmip mapped to the NF-κB pathway. *Aldh1a*, *Maf*, *Stat3*, *Il10,* and *Cd40* mapped to the STAT pathway (Fig. 4b). The protein levels of FPR1, MyD88, p-AKT in airway DCs were significantly upregulated (Fig. 4c–e). By ChIP assay, we observed that the levels of c-Maf in the *Il10* promoter were significantly upregulated by TAFA4 (Fig. 4f). The results demonstrate that exposure to TAFA4 can activate the *Il10* expression-related signaling pathway in DCs.

### TAFA4 reduces AR response in mice

**Fig. 5:**
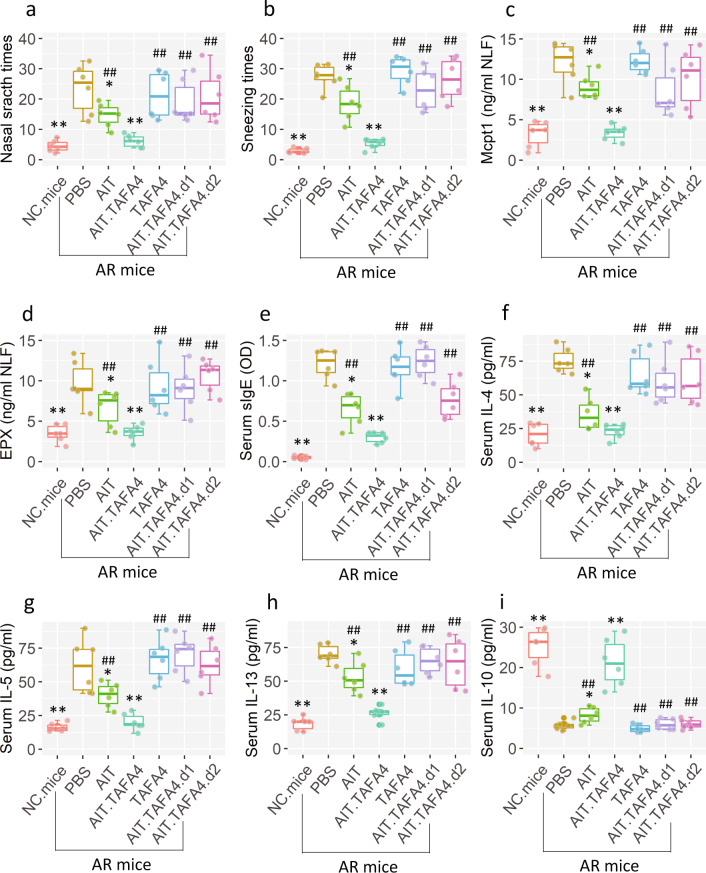
AR response assessment. **a**, **b** AR-like clinical response records (nasal itch and sneezing). **c**, **d** Allergic mediators (Mcpt1 and EPX) in NLF. **e** Serum sIgE levels. **f**–**h** Serum Th2 cytokine levels. **i** Serum IL-10 levels. The data are presented as median (IQR) from 6 mice per group. Mcpt1 mast cell protease-1, EPX eosinophil peroxidase, NLF nasal lavage fluids, AIT specific allergen immunotherapy. The “d1” stands for “mice carry the *Fpr1*-deficient DCs”. The “d2” stands for “mice carry the *Il10rb*-deficient CD4^+^ T cells”. TAFA4: TAFA chemokine like family member 4. Statistics: ANOVA + Bonferroni test. *(*p* < 0.05), **(*p* < 0.01), compared with the AR mice/PBS group. ## (*p* < 0.01), compared with the AR mice/AIT.TAFA4 group. The data of boxplot are presented as median (middle line inside box), 75% value (upper bound of box), 25% value (lower bound of box), max (upper whisker), and min (lower whisker). Each dot in boxplot presents data obtained from one sample.

Since IL-10 can counteract immune inflammation^14^, we inferred that administration of TAFA4 may limit the AR response. To test this, the *Fpr1*^*∆*DC^ mice (this mouse strain carries the *Fpr1*-deficient DCs. The total number of DC in the airways and spleen was not significantly different between WT mice and *Fpr1*^*∆*DC^ mice; Supplementary Fig. 2 in supplemental materials) and wild-type (WT; C57/B6) mice were treated with the AR mouse model development protocol^15^. AIT (with or without the TAFA4 in the AIT vaccine) was conducted in AR mice. In a specific antigenic challenge, AR mice showed an AR response, including AR-like clinical symptoms (nasal itching and sneezing) (Fig. 1a, b), allergic mediators (Mcpt1 and EPX) (Fig. 1c, d) in nasal secretions, high levels of sIgE and Th2 cytokines, and lower IL-10 levels in the serum (Fig. 5e–i). Treating AR mice with AIT alone moderately (*p* < 0.05) suppressed the AR response. While simultaneous administration of TAFA4 and AIT significantly reduced the AR response (*p* < 0.001). In particular, although the *Fpr1*^*∆*DC^ mice could be induced typical AR response (Supplementary Fig. 3), treatment with TAFA4 alone did not enhance the therapeutic effects of AIT in *Fpr1*^*∆*DC^ mice (Fig. 5). The results indicate that TAFA4 can promote the AIT effects on inhibiting AR response.

### Tr1 cells induced by TAFA4-primed DCs are the effector cells for suppressing AR response

**Fig. 6:**
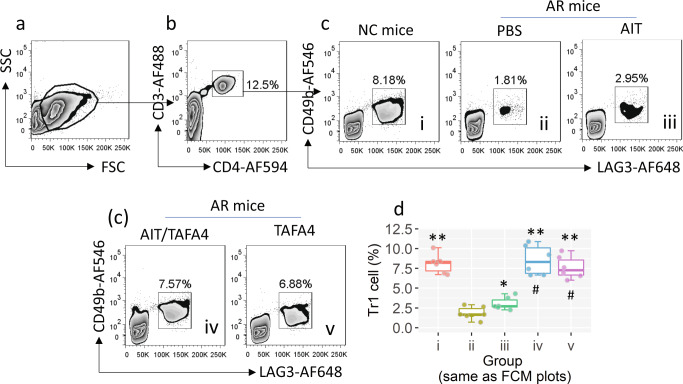
Assessment of Tr1 cells in the airway tissues. NC mice and AR mice were treated with the procedures as denoted above each FCM subpanel. AMCs were prepared with the airway tissues, and analyzed by FCM. **a** The FSC/SSC plots. **b** The FSC/SSC plots. **c** Gated plots show Tr1 cell counts. **d** Boxplots show Tr1 frequency in AMCs from 6 mice per group. Statistics: ANOVA + Bonferroni test. *(*p* < 0.05), **(*p* < 0.01), compared with group ii. #(*p* < 0.05), compared with group iii. NC naive control, AR allergic rhinitis, FCM flow cytometry, AMC airway mononuclear cell, Tr1 cell type 1 regulatory T cell, TAFA4 TAFA chemokine like family member 4, AIT allergen specific immunotherapy. The data of boxplot are presented as median (middle line inside box), 75% value (upper bound of box), 25% value (lower bound of box), max (upper whisker), and min (lower whisker). Each dot in boxplot presents data obtained from one sample.

**Fig. 7:**
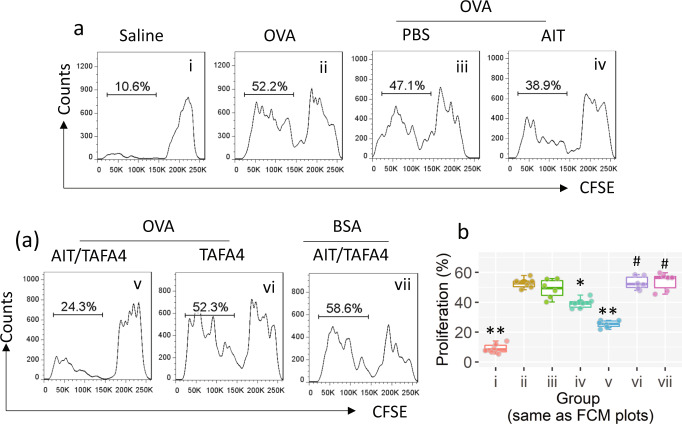
Assessment of Tr1 cell’s immune suppressive function. Tr1 cells were isolated from AMCs of mice treated with procedures denoted above each subpanel. DCs and CD4^+^ CD25¯ T cells (Teffs; isolated from DO11.10 mice; labeled with CFSE) were isolated from the spleen. DC:Teff:Tr1 cells were cultured at a ratio of 1:5:1 in the presence of ovalbumin (OVA, 1 µg/ml; BSA was used as an irrelevant antigen), gated plots show proliferating Teffs (**a**). Boxplots show proliferating Teff frequency from six independent experiments (**b**). *(*p* < 0.05), **(*p* < 0.01), compared with group b. #(*p* < 0.05), compared with group d. TAFA4 TAFA chemokine like family member 4, AIT allergen-specific immunotherapy. The data of boxplot are presented as median (middle line inside box), 75% value (upper bound of box), 25% value (lower bound of box), max (upper whisker), and min (lower whisker). Each dot in boxplot presents data obtained from one sample.

**Fig. 8:**
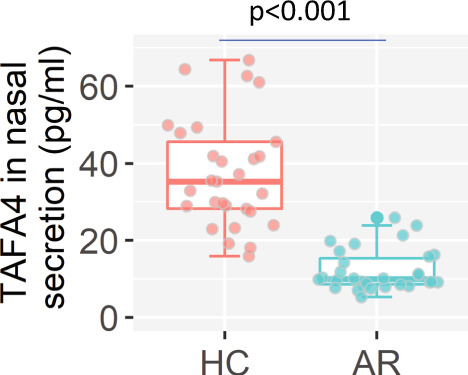
Assessment of TAFA4 levels in nasal secretions of AR patients. Nasal secretions were collected from HC subjects (*n* = 30) and AR patients (*n* = 30). The TAFA4 levels in nasal secretions were determined by ELISA. Boxplots show TAFA4 levels from 30 nasal secretion sample per group. Statistics: Mann–Whitney test. AR allergic rhinitis, HC health control. The data of boxplot are presented as median (middle line inside box), 75% value (upper bound of box), 25% value (lower bound of box), max (upper whisker), and min (lower whisker). Each dot in boxplot presents data obtained from one sample.

It is known that IL-10^+^ DCs can induce Tr1 cells^16^. We then assessed the Tr1 cell frequency in the airway tissues of AR mice after TAFA4 therapy. We found that administration of either AIT or/and TAFA4 could induce Tr1 cells in the airway tissues. Treatment with AIT/TAFA4 or TAFA4 alone induced more Tr1 cells than AIT alone (Fig. 6). However, those Tr1 cells induced by the TAFA4 alone did not show antigen-specific immune suppressive effects. Both AIT and AIT/TAFA4 therapy could induce antigen-specific Tr1 cells. AIT/TAFA4 treatment was more effective for inducing antigen-specific Tr1 cells than treatment with AIT alone (Fig. 7). The results suggest that the therapeutic effects by AIT or AIT/TAFA4 are attributed to the antigen-specific Tr1 cells. To verify this, we constructed a mouse strain, which carried the *Il10rb-*deficient CD4^+^ T cells (*Il10rb*^*Δ*Cd4tc^; the mice still had the normal number of CD4^+^ T cells as compared to WT mice; Supplementary Fig. 4). WT mice and *Il10rb*^*Δ*Cd4tc^ AR mice were treated with the AIT/TAFA4 therapy. We found that although the AR response could be induced in *Il10rb*^*Δ*Cd4tc^ mice (Supplementary Fig. 5), which did not respond to the TAFA4 therapy or AIT/TAFA4 therapy (Fig. 5). Additionally, we also found TAFA4 in human nasal secretions. The levels of TAFA4 in nasal secretions were significantly lower in AR patients than that in healthy controls (Fig. 8).

## Discussion

In this study, we found that TAFA4, a neuropeptide, could substantially enhance the therapeutic efficacy of AIT. Upon exposure to TAFA4, DC became the tolerogenic DC (tDC) after gaining the immune tolerogenic capacity by expressing IL-10. tDCs further induced Tr1 cell development. A combined AIT and TAFA4 therapy induced antigen-specific Tr1 cells. Better inhibitory effects on AR response were achieved with the AIT/TAFA4 therapy.

The data show that TAFA4 can promote immunotherapy. According to the definition of immune adjuvant, TAFA4 can be a candidate of immune adjuvant as it can markedly improve immune response. The mechanism behind this effect of TAFA4 treatment is endorsed by it induces DC to produce IL-10. Others also found this feature of TAFA4. Hoeffel et al observed that TAFA4 induced the production of IL-10 in macrophages, which reduced inflammation and promoted tissue regeneration^12^. TAFA4 is produced by sensory neurons expressing the Gαi-interacting protein (GINIP)^12,17^. We also detected TAFA4 in the NLF, the amounts of which were much lower in AR mice as compared with NC mice. Since TAFA4 is an anti-inflammatory factor^12^, the results suggest that insufficient TAFA4 may be associated with the pathogenesis of AR. How TAFA4 is reduced in AR subjects requires further study.

We found that the addition of exogenous TAFA4 recruited DCs in the airway tissues. DCs are cells to begin an immune reaction^18^. The data may be considered as DCs are recruited to the airway tissues to induce a given immune response. Another sign induced by TAFA4-containing nasal instillation is that DCs recruited by TAFA4 are activated. This is indicated by the expression of Ki-67 in DCs. Ki-67 is a marker that mirrors cell activation. This demonstrates another aspect that TAFA4 can activate DCs. Following the TAFA4 exposure, we found that the DCs expressed IL-10. IL-10 is the pillar of tolerogenic DCs (tDC). Since IL-10 is an anti-inflammatory cytokine as well as an immune regulatory cytokine, the TAFA4-primed DCs meet the definition of tDC. This is also confirmed by the results of the costimulatory molecules assessment of BMDCs. BMDCs exposure to TAFA4 in culture resulted in a moderate increase in the expression of CD80, CD83 and CD86.

The data show that airway DCs express FPR1. The main ligand of FPR1 is TAFA4. TAFA4 can be released from the mitochondria of dying cells and nervous terminations. FPR1 is expressed through a variety of immune cells, including macrophages, natural killer cells, dendritic cells and neutrophils^19^. TAFA4 has chemotactic property^19^. In response to the signal from TAFA4, immune cells move towards the sources of TAFA4. Our data show that upon treating mice with TAFA4, the number of DC is increased in the airway tissues. Previous studies indicate that TAFA4 has anti-inflammatory properties. Hoeffel et al reported that TAFA4 could induce macrophages to produce IL-10 to mitigate the sunburn-induced skin inflammation^12^. Delfini et al. report that TAFA4 can reduce inflammatory activities^20^. Current data show that the TAFA4-recruited DCs express IL-10. This demonstrates that TAFA4 can benefit the airway tissues by promoting immune tolerance.

We found the interaction of TAFA4 and FPR1 activated the PI3K-AKT signaling pathway in DCs. The PI3K-AKT pathway links to the IL-10 expression^21^. With respect to the relationship between FPR1 and PI3k, our data show that exposure to TAFA4 improves PI3K phosphorylation and c-Maf phosphorylation induced in DC. c-Maf is the transcription factor of IL-10. These data support the proposed concept of TAFA4 being able to induce IL-10 expression in DCs. The notion was further verified by blocking experiments. The presence of inhibitors of PI3k or AKT abolished the TAFA4-induced expression of IL-10 in DCs. It is known that the IL-10-expressing DCs have immune tolerogenic properties, and can be called the tolerogenic DCs (tDCs). Inducing tDC is an important milestone in the AIT course^22^. The data show that administration of either AIT or TAFA4 can induce tDC in the airway tissues. Such an effect can be substantially promoted by a combination of AIT and TAFA4.

The data show that administration of either AIT/TAFA4 or TAFA4 could induce Tr1 cells in the airway tissues. However, only those AR mice treated with AIT/TAFA4 achieved therapeutic effects on AR. The administration of TAFA4 alone did not result in appreciable effects on the suppression of AR. The results suggest that the AIT/TAFA4 therapy induces antigen specific Tr1 cells. The latter can be activated by exposing to specific antigens. Additional in vitro experiments confirmed this inference. Tr1 cells isolated from the AIT/TAFA4-treated AR mice were activated by specific antigens in culture. Activated Tr1 cells exhibited immunosuppressive effects on antigen-specific T cell activation.

In addition, epithelial cells can also play a part in therapeutic effects on airway allergy. Previous reports show that IL-4 and IFN-γ orchestrate epithelial activities in the airways, such as IFN-γ can antagonize the effect of IL-24 by IL-4-primed epithelial cells^23^. Immunotherapy can suppress nasal epithelial cell-secreted secretoglobin1A1 and IL-24^24^. Whether TAFA4 has any effects on regulating the activities of epithelial cells, or regulates the effects of periostin or CCL26^25^, during immunotherapy is an interesting topic and may be further investigated.

TGF-β1 is a major immunoregulatory molecule. A fraction of the regulatory T cells expresses TGF-β1 which suppresses the activities of other immune cells to avoid self-injury during the immune reaction. However, TGF-β1 is also involved in inflammation. For example, TGF-β/Smad signaling is a major pathway leading to kidney disease^26^. It remains to be examined whether TAFA4 also interacts with TGF-β in the tissues of the airways.

IL-37 is a member of the IL-1 family that can regulate allergic inflammation by counterbalancing pro-inflammatory IL-1 and IL-33 in murine models as well as in patient samples^27^. In addition, during AIT, much more immune cells are involved in therapeutic effectiveness. Such as Tregs, Bregs, Th1 cells, and Th2 cells^1^. The prostaglandin EP3 receptor is a selective target in AIT^28^. If TAFA4 also interacts with these immune components during AIT can be studied in more detail.

The data show that the levels of TAFA4 in nasal secretions are lower in patients with AR than in healthy controls. Further research is required to elucidate the importance of TAFA4 in maintaining immunologic homeostasis in nasal tissues.

In summary, current data show that TAFA4, acting as an immune adjuvant, substantially improves the efficacy of TIA. The findings suggest that AIT/TAFA4 therapy has the potential to translate for the treatment of allergic diseases.

## Methods

### Reagents

Mouse TAFA4 ELISA kit was purchased from Kanglang Biotech (Shanghai, China). Anti-mouse FPR1 (PAB0284) Ab were purchased from Kemi Biotech (Shanghai, China). Anti-mouse TAFA4 Ab (PA569319) was purchased from Shanghai Saimofeishier Biotech (Shanghai, China). Recombinant mouse TAFA4 protein was purchased from CUSABio (Wuhan, China). MyD88 (sc-74532, E-11), AKT (sc-81434, 5C10; sc-514032, C-11), CD11c (sc-398708, D-8, AF594), IL-10 (sc-57245, 2G101H7, AF488), CD3 (sc-20047, PC3/188 A, AF488), CD4 (sc-19641, MT310, AF594), CD49b (sc-53353, HAS-4, AF546), LAG3 (sc-32750, C9B7W, AF648) were purchased from Santa Cruz Biotech (Santa Cruz Biotech (Santa Cruz, CA). Ki-67 Ab (ab16667, SP6, AF488) was purchased from abcam (Cambridge, MA). ELISA kits of sIgE, Mcpt1, EPX, IL-4, IL-5, IL-10, IL-13 were purchased from CRK Pharma (Wuhan, China). The ChIP kit was purchased from Sigma Aldrich (St. Louis., MO). Reagents and materials for RT-qPCR and Western blotting were purchased from Invitrogen (Carlsbad, CA).

### Mice

C57/B6 mice were purchased from the Guangdong Provincial Experimental Animal Center (Guangzhou, China). By employing the genetic engineering approach, we constructed *Fpr1*^f/f^*Itgax*-Cre mice (here we called *Fpr1*^ΔDC^ mice), that deleted the *Fpr1* gene in DCs, expressing Cre recombinase from the *Itgax* promoter (*Itgax*-Cre mice), were crossed with mice with loxP-flanked *Fpr1* exons 1 and 2 (*Fpr1*^f/f^ mice). To initiate the Fpr1 gene ablation, *Fpr1*^ΔDC^ mice were gavage-fed with tamoxifen (200 mg/kg in corn oil) daily for 5 consecutive days before experiments. DCs in the airway tissues of *Fpr1*^ΔDC^ mice did not show detectable *Fpr1* expression. The frequency of DC in the airway tissues of *Fpr1*^ΔDC^ mice was not significantly different between wild type (WT) mice and *Fpr1*^ΔDC^ mice. In the same approach, mice carrying *Il10rb*-deficient CD4^+^ T cells (*Il10rb*^f/f^*Cd4*-Cre mice; here we call *Il10rb*^ΔCd4tc^ mice, that deleted the *Il10rb* gene in CD4^+^ T cells, expressing Cre recombinase from the *Cd4* promoter, the *Cd4*-Cre mice) were crossed with mice with loxP-flanked *Il10rb* exons 1 and 6 (*Il10rb*^f/f^ mice). To initiate the *Il10rb* gene ablation, *Il10rb*^ΔCd4tc^ mice were gavage-fed with tamoxifen (200 mg/kg in corn oil) daily for 5 consecutive days before experiments. CD4^+^ T cells in the airway tissues of *Il10rb*^ΔCd4tc^ mice did not show detectable *Il10rb* expression. The frequency of DC in the airway tissues of *Il10rb*^ΔCd4tc^ mice was not significantly different between wild-type (WT) mice and *Il10rb*^ΔCd4tc^ mice. *Cd4*-Cre mice, *Itgax*-Cre mice and DO11.10 mice were purchased from Jackson Laboratory (Bar Harbor, ME). Mice were maintained in a specific pathogen-free facility with accessing water and food freely.

### Enzyme-linked immunosorbent assay (ELISA)

Levels of cytokines and sIgE (OVA specific IgE) in the serum or NLF were determined by ELISA with commercial reagent kits following the manufacturer’s instruction.

### Flow cytometry (FCM)

To stain molecules on the cell surface, cells were incubated with fluorescence-labeled Abs (detailed in figures) or isotype IgG (Abs were diluted into 1 µg/ml) for 30 min at 4 °C. After washing with PBS, cells were analyzed with a flow cytometer (BD FACSCanto II). To stain the intracellular molecules, cells were fixed with 1% paraformaldehyde (containing 0.05% Triton X-100) for 1 h. Cells were then processed with the procedures of surface staining. The data were processed with a software package (Flowjo, TreeStar Inc., Ashland, OR). The data obtained from isotype IgG staining were used as gating references.

### Real-time RT-PCR (RT-qPCR)

RNA was extracted from cells obtained from relevant experiments. The RNA samples were converted to cDNA with a reverse transcription kit following the manufacturer’s instruction. The cDNA samples were then amplified in a qPCR device (Bio-Rad CFX96) with the SYBR Green Master Mix in the presence of primers of *Ki67* (gccataacccgaaagagcag and ccagtttacgctttgcaggt), *Il10* (ataactgcacccacttccca and gggcatcacttctaccaggt), *Cd80* (ttatcatcctgggcctggtc and gtgtctgcagatgggtttcc), *Cd83* (gctctcctatgcagtgtcct and actctgtagcttccttgggg), *Cd86* (gcacgtctaagcaaggtcac and catatgccacacaccatccg), *Ccr7* (gggctggtgatactgacgta and acacaggtagacgccaaaga), Fpr1 (tcagcactcccatgtccatt and tcacggacttggattgtgga), *Cmip* (cctacagcgccattgaagac and ggcttgggttactcaggact), *Maf* (gtcccctggccatggaatat and tagtagtcttccaggtgcgc). The results were calculated with the 2^-∆∆Ct^ method, and presented as relative expression.

### Western blotting

DCs were isolated from AMCs. Proteins were extracted from DCs, fractioned by SDS-PAGE, and transferred onto a PVDF membrane. After blocking with 5% skim milk for 30 min, the membrane was incubated with the primary Abs (detailed in figures; diluted to 200 ng/ml) for 2 h at room temperature, washed with TBST (Tris-buffered saline containing 0.05% Tween 20) 3 times, incubated with HRP-labeled secondary Abs (diluted to 20 ng/ml) for 2 h at room temperature, washed with TBST 3 times. Immunoblots on the membrane were developed with the enhanced chemiluminescence, and photographed in an imaging station.

### Chromatin immunoprecipitation (ChIP)

DCs were prepared and fixed in 1% formalin for 15 min. The cells were lysed, and followed by sonication to shear the DNA into small pieces. Samples were precleared by incubating with protein G agarose beads for 2 h. The beads were removed by centrifugation. Supernatant was incubated with anti-c-Maf Ab for 2 h, and followed by incubating with protein G agarose beads for 2 h. Proteins on the beads were eluted; DNA was extracted from the samples with a DNA extracting reagent kit following the manufacturer’s instruction, and analyzed by qPCR in the presence of *Il10* promoter primers (ctgtgccaacgaagatcctc and aacattcgcctagagtcccc). The results were presented as fold change against the input. The uncropped gel graphs are presented in supplemental materials.

### RNA-sequencing (RNAseq)

DCs were isolated from AMCs. Total RNA was extracted from DCs using the TRIzol reagents. The RNA samples were analyzed by professional staff in a biotech company (BGI, Shenzhen, China). Briefly, the library was constructed first (RNA-Seq Library Prep Kit; Illumina), followed by analyzing with an Illumina platform (HiSeq 2500, Illumina, San Diego, CA). Gene expression was assessed using the DESeq R package (version 1.18.0). Multiple adjustment tests were performed to calculate the adjusted *P*-value (adjpval). Genes with corrected *P* values of less than 0.05 and log2 (fold change, FC) of 1 or greater between two groups were considered to have significantly differential expressions. The gene significantly differential expression between the two groups was determined when *p* < 0.05 and log2 (fold change, FC) of 1 or greater. The differentially expressed genes (DEGs) were analyzed by signaling pathway enrichment assay with the KEGG database, and further by ontology analysis. The raw gene data are presented in Table S1.

### Human subjects

Patients (*n* = 30, male = 15, female = 15) with perennial allergic rhinitis (AR) were enrolled into the present study from May 2021 to May 2022 in our hospital. The using human samples in the present study was reviewed and approved by the Human Ethics Committee at our hospital (Approve number: SMUSZHE2021003). A written informed consent was obtained from each human subject. The diagnosis of AR and collection of nasal secretion were carried out by doctors of our hospital based on our routine procedures that can be found elsewhere^29^. AR patients had AR history at least for 2 years, serum specific IgE (sIgE) positive, skin prick test positive. Health control (HC) subjects (*n* = 30, male = 15, female = 15) were also enrolled into the present study. HC subjects did not have any appreciable diseases, serum sIgE negative and skin prick test negative.

### Development of an AR mouse model

The Animal Experimental Protocol was reviewed and approved by the Animal Ethics Committee at Southern Medical University (Approve#: SMUAEC0222019). As depicted in Supplementary Fig. 1 (in supplemental materials), mice were sensitized by subcutaneous injection of ovalbumin (OVA, 100 µg/mouse, mixed in 0.1 ml Alum) in the back skin on day 1 and day 7, respectively. Mice were boosted by nasal instillation (20 µl/nostril, 5 mg/ml) daily from day 9 to day 22. On day 23, mice were challenged with the specific antigen (OVA) by nasal instillation (20 µl/nostril, 50 mg/ml).

### Assessment of the AR response in mice

A clinical response of AR (nasal scratches and sneezes) was recorded in each mouse within a 30-minute period after the nasal antigen challenge. Under general anesthesia (60 mg/kg pentobarbital, ip), blood samples were taken from each mouse using the eyeball drawing method. After that, the trachea was exposed. With a syringe, 1 ml of saline was injected into the trachea (in the direction of the nose; the head was put in the lower position). The saline solution was taken from the nostrils with an Eppendorf tube and used as a nasal wash fluid (NLF) in other experiments.

### Preparation of airway mononuclear cells (AMCs)

The nasal tissues and the lungs were excised from the mice on the sacrifice. The tissues were cut into small pieces, incubated with collagenase IV for 20 min at 37 °C with light shaking. Single cells were filtered through a cell strainer (70 µm first, then 40 µm). AMCs were further isolated from single cells with the Percoll gradient density centrifugation.

### Immune cell isolation

DCs and CD4^+^ CD25¯ T cells were isolated from mouse spleen cells using commercial reagent kits (Miltenyi biotech) as per the manufacturer’s instructions. For Tr1 cell isolation, AMCs were labeled with Abs of CD3 (FITC), CD4 (AF594), LAG3 (AF546), CD49b (AF647). The Tr1 cells (CD3^+^ CD4^+^ LAG3^+^ CD49b^+^) were isolated from AMCs by FCM. The purified immune cells were checked by FCM. If purity did not reach or exceed 95%, purification would be repeated.

### Assessment of immune suppressive function of Tr1 cells

Tr1 cells were isolated from AMCs of AR mice treated with the AIT or/and TAFA4 therapies. DCs and CD4^+^ CD25¯ T cells (Effector T cells or Teffs; labeled with CFSE) were isolated from the DO11.10 mouse spleen cells. Tr1 cell:DC:Teff cells were co-cultured at a ratio of 1:1:5 in the presence of OVA (1 µg/ml) or BSA (used as an irrelevant antigen, negative control) for 3 days. Cells were harvested on day 3, and analyzed by FCM, the CFSE-dilution assay. Teff proliferation was assessed and used as an indicator for Teff activities.

### Statistics

The difference between two groups was determined by the two-tail Student’s *t* test. Multiple comparisons were carried out with ANOVA, then with the Dunnett test or the Bonferroni test for groups of more than two groups. *p* < 0.05 was set as a significant criterion.

### Reporting summary

Further information on research design is available in the Nature Research Reporting Summary linked to this article.

## Supplementary information

Supplementary data

Supplemental materials

REPORTING SUMMARY

## Data Availability

All the data are included in this paper and the online supplemental materials.
