# Supplementary material for: TAFA4-IL-10 axis potentiate immunotherapy for airway allergy by induction of specific regulatory T cells
Source: NPJ Vaccines. 2022 Oct 31;7:133. doi: 10.1038/s41541-022-00559-w (PMC9622679; doi:10.1038/s41541-022-00559-w)
Supplement: Supplementary file 1 — Supplementary data [file 41541_2022_559_MOESM1_ESM.pdf]

Supplementary Table 1. Raw data for RNAseq

GeneOrder	Gene	baseMean	log2FoldChange	lfcSE	stat	pvalue	padj
1	GLO1	228.7515687	-0.799427602	0.18825601	-4.246544915	-9.292099526	5.414578209
2	IFITM2	677.4017752	-0.605629521	0.128296052	-4.276060756	-9.71504467	1.492204195
3	AC078850.1	24.27549106	7.12659024	1.509754228	4.720264172	-10.75059672	1.492204195
4	FCGR2B	28.52267555	2.11272286	0.577897526	2.657610154	-7.85429119879524	2.444141594
5	DBI	402.0604744	-0.674528094	0.159141951	-4.228521022	-11.1161515422944	1.110541515
6	BTN2A2	126.297566	-1.054220722	0.26851087	-2.92621246	-8.906207226	1.427127118
7	SLC12A5-AS1	52.54088286	2.274116215	0.448556588	5.292790874	6.098946027	4.897004815
8	TPSAB1	2687.062914	-0.809856672	0.099419281	-8.145871228	2.916214125	8.12075458
9	GSEC	21.2224851	2.801822056	0.724514958	2.867170752	-11.62412289	1.111104547
10	AP002471.1	0.979411277	0.062718118	2.082209142	0.020241171	-2.767502218	1.454099012
11	PLIN2	494.1029752	-0.797244082	0.164209555	-4.852085942	2.429792206	1.197979875
12	C11orf24	192.4951882	-1.181619558	0.221722229	-5.099072807	12.66524293	2.471747424
13	AC007270.1	0.740204196	1.049207872	2.521140276	0.297158266	-2.704414554	2.458918288
14	AC007270.2	0.489705689	0.05402189	4.196172952	0.012876469	-2.64965607	2.484844145
15	FCGR2A	122.8209855	1.562197581	0.264748787	5.90445607	4.904542026	1.127514544
16	TGFBI	292.4590474	2.248092882	0.177574404	12.66000522	2.220911562	1.127514544
17	TMEM50A	250.546674	0.612988472	0.170822275	2.594290488	-2.147222229	1.102450548
18	PDCD4	226.0015898	-0.922271002	0.201506427	-4.62196619	-5.102582508	1.944287252
19	CCR7	7872.18965	0.409924581	0.078884085	5.196670277	5.278098715	2.48941952
20	VWA5A	2097.080998	-0.491422822	0.092685262	-5.245577447	1.489084989	1.104425852
21	AL049874.2	0.500997014	2.484998858	4.296126822	0.565270056	-2.620267192	2.444141594
22	PRDX6	822.0026191	-0.666725701	0.122556024	-5.296221718	1.72921458	1.102450548
23	CCL2	1671.072949	0.688122474	0.100167879	6.86969198	2.054701799	1.45492424
24	PLA2G7	182.2127501	1.022675895	0.225605259	4.522022169	-2.766220907	5.448451184

25	AC027198.2	121.9120744	-1.877844142	0.272275457	-6.869102889	1.442176619	1.995481015
26	F2R	28.22922902	-2.298924222	0.599225272	-2.825852511	-1.710015856	1.440011402
27	HLA-DQA1	51.41006272	2.281546572	0.472549695	5.029780147	4.625081549	5.440182721
28	RNF212	216.6671818	1.264147944	0.190528072	7.159450742	1.242256258	1.119048545
29	F[R1	1088.742088	7.596610009	0.222222904	22.50262441	1.609029096	1.459749707
30	FERMT2	72.84291776	-1.701922221	0.256641275	-4.772112597	-2.857754273	1.111104547
31	SPINT2	204.2488242	0.706127888	0.17821272	2.960087245	-7.1915516561697	1.704447581
32	BLVRB	122.422052	1.299156461	0.274678045	4.729742641	-4.622722952	1.12185285
33	EFCAB14	224.0410292	-0.902706781	0.178925928	-5.050721269	1.924907729	1.181047918
34	RPS2	2457.71127	0.472982695	0.100445448	4.708861299	-2.70150428505205	1.189512894
35	CTSL	1227.277195	1.968022264	0.112266261	17.52005177	2.166557542	1.925248714
36	CCND1	158.7746678	-2.424265088	0.260944248	-9.290256498	1.687810918	1.45492424
37	CDCP1	124.4202451	-1.17190014	0.266854624	-4.291520028	-6.212850095	1.110541515
38	AC008750.2	166.7627562	-0.861900624	0.222897902	-2.700765994	-1.975941166	1.111104547
39	CD40	26.01299214	5.174501518	0.86962852	5.950175092	2.008295029	1.1091711
40	FCER1A	222.6101696	1.164189122	0.192690214	6.010572815	2.585457895	1.112297871
41	MOB2C	128.1728277	1.411122802	0.271478072	5.197962252	8.115226111	1.102450548
42	MRC1	106.9124276	2.578242864	0.421224274	8.492847622	2.210422955	2.91211408
43	FUCA1	501.2292781	-1.796522048	0.160681919	-11.18061724	2.0580562	1.479119585
44	CTNNA1	262.2182154	0.820622589	0.170896188	4.860457081	6.622629252	1.720015518
45	THBS1	699.5955606	-1.699242944	0.124064419	-12.67482168	7.922497786	1.472844217
46	AL025090.1	0.724522502	-2.98570617	2.452708589	-0.86474217	-2.721842586	1.454984411
47	SPARC	18.76529122	-2.86284772	0.782979109	-2.651688784	-2.158264223	1.189512894
48	CD9	405.7961146	-1.226210927	0.162117721	-8.242842008	2.74656523	2.180114599
49	CMIP	22.525826	2.100661166	0.542261722	2.866045476	-2.94658688442698	1.720015518
50	EIF1B	199.2594915	1.10562468	0.222974006	4.958581052		

						1.952894713	1.111104547
51	DCSTAMP	40.85059621	2.278258228	0.505688492	4.702208225	-1.852979589	1.712052499
52	CHI2L1	227.8782185	-0.952199748	0.198045429	-4.812025594	1.510242107	1.197979875
53	GMPR	189.7885025	-1.029295024	0.244526696	-4.209162896	-8.662157299	1.479517011
54	AL021186.1	0.500997014	2.484998858	4.296126822	0.565270056	-2.441115483	1.445290229
55	MMP12	42.27809472	6.458782202	1.112718262	5.80450902	4.820114506	1.494141541
56	AL109922.2	0.500997014	2.484998858	4.296126822	0.565270056	-2.440997276	2.405207109
57	AC104265.2	0.745824827	-0.972210916	2.522016256	-0.276242662	2.261689294	1.440011402
58	AC114728.1	0.745824827	-0.972210916	2.522016256	-0.276242662	-4.748472709	2.28177451
59	MAF	275.5674211	-0.772957964	0.188669504	-4.096888717	-2.569592078	1.112297871
60	F12A1	242.9746086	5.066644985	0.222882582	15.17488502	1.841960425	1.114891821
61	ACO1	120.0957022	-1.062900022	0.277212761	-2.827822689	-2.462228018	2.954154709
62	GSN	702.1578745	0.496550179	0.129725007	2.827712644	-5.61894627	1.110411587
63	TDRD2	171.412214	-1.97459959	0.250141567	-7.892928294	1.502520593	1.104425852
64	CDKN1A	254.462515	1.621457622	0.2026287	8.051462692	2.089758887	4.924501422
65	IL10RA	80.94184689	2.181172576	0.271260494	5.872466912	1.865254672	1.471041021
66	CNR2	21.68472422	4.269121029	0.755087012	5.786248422	1.958296812	5.14745517
67	ELANE	129.2204642	-1.17101122	0.256090652	-4.572642252	-6.822879217	1.198019927
68	SSTR2	22.21808059	2.950242291	0.69806104	4.226481957	-1.674286886	1.100815111
69	IL10	88.05027017	1.267972181	0.219594522	4.280226609	-2.499950894	1.189512894
70	ATP6V1B2	611.8895085	0.717225212	0.144157722	4.975281957	6.801289898	1.001151709
71	CTNS	122.7200286	1.074168572	0.259265999	4.142112925	-2.842768221	2.174555905
72	ICAM1	420.2279212	0.910269602	0.15781481	5.767960568	7.141286541	1.441405054
73	FTL	20052.47146	0.746242542	0.07225922	10.21429264	2.268295926	2.199972902
74	CALB2	224.8691615	-1.460922157	0.218164456	-6.696471929	7.529244585	1.977478478
75	ENDOD1	127.2972899	-1.291189242	0.272617924	-4.718949971	-6.622161762	2.182011884

76 AC124045.1 0.478414262 -2.271055522 4.479204761 -0.529247421
1.989271705 4.912918781
77 ITGAX 267.7218285 0.668520042 0.167724742 2.985578864 -
5.851681613 1.117717178
78 CTSC 1282.174859 1.702752725 0.107722914 15.80522502
9.061557561 1.712102541
79 STAT3 295.2226094 0.802404254 0.184091725 4.25871961 -
4.561592227 2.447249427
80 LCP1 2266.661102 0.702182281 0.087159566 8.056296218
2.725292457 2.455805414
81 CAMK2B 94.2298712 1.295459402 0.217485221 4.080275622 -
2.52785982 1.445290229
82 CSTB 752.5808494 1.021206259 0.129282859 7.899719808
1.222848927 1.451212719
83 AL291988.1 100.1811224 -1.192910169 0.202746814 -2.940289755 -
1.267200242 2.927241751
84 MLPH 242.5467154 -1.512915422 0.208694127 -7.249429044
2.617142247 1.715928907
85 C2AR1 427.4071202 -0.669428069 0.15556201 -4.202224229 -
2.056154902 1.100815111
86 TENT5A 204.1916222 1.246449256 0.217511672 6.190229522 1.72912882
2.927241751
87 CLU 1242.797022 0.726290499 0.112275258 6.494278497
6.800519859 4.182172111
88 CASP2 255.6574009 1.197881282 0.179620259 6.6689659 5.65127219
1.117717178
89 ALDH1A2 52.26029426 1.655906108 0.424091458 2.814647987 -
4.99777185890972 1.70459751
90 LINC02694 18.28968087 2.879292129 0.782290805 2.675550071 -
1.04554456102576 2.174405947
91 SPACA2 79.62225722 -2.211547112 0.260748402 -6.407642281
1.165774126 4.894954744
92 AL256652.1 26.11902002 -2.455147701 0.62025561 -2.894861248 -
1.959989264 1.99180525
93 CD74 728.2224669 1.287898492 0.122756698 9.701194065
2.122284217 1.914891745
94 AC006228.1 0.982196662 -2.404284457 2.098256189 -1.098806625
2.650084216 1.100815111
95 CCNA1 277.0225522 -2.022585811 0.2066201 -9.842148997
5.918854681 1.984184718
96 SLFN5 154.9245878 -1.125642912 0.210410282 -2.626205616 -
1.785642904 1.451212719
97 AC024195.1 0.500997014 2.484998858 4.296126822 0.565270056
4.299242089 1.127514544
98 SLC28A6 54.22976822 1.822829202 0.429772468 4.144950606 -
2.054628784 1.110541515
99 MYD88 172.0624217 -0.997521429 0.220816979 -4.221698665 -
8.54167027871912 1.104425852
100 ACTG1 4095.460287 0.216282907 0.086565489 2.652695146 -
4.085768495 1.427127118
101 GNPTAB 170.2759222 -1.210451608 0.227528676 -5.095808825

						2.985510773	1.45492424
102	PPARG	42.62866146	2.992257956	0.518058984	5.778025276	7.27184142	2.182011884
103	RPLP0	1560.224986	0.286851127	0.102512082	2.727255852	-4.76046684655112	1.1091711
104	CTSB	5719.748701	0.589965754	0.090954677	6.486270719	1.882474211	2.199972902
105	FLOT1	287.9425195	-0.649164406	0.16956547	-2.828299759	-1.46778508758721	1.114941894
106	DDIT4	229.1192561	-0.705479949	0.179771179	-2.924221749	-6.4072202	2.709414895
107	HLA-DRA	405.6918979	1.711595802	0.188284916	9.085620861	7.554670155	1.925248714
108	PLEK	495.6079218	0.672579499	0.154191265	4.26846721	-1.088847681	1.949475294
109	PTEN	229.8722222	-1.664051226	0.186715859	-8.912211514	1.612227287	1.472894279
110	ALOX15	650.2198978	9.797298716	0.844229154	11.6026485	1.22867229	1.445442025
111	NOL4L	259.6295526	0.759785209	0.191615166	2.965162694	-2.24051544001912	2.188481577
112	AC102952.2	0.742989481	-2.000642057	2.742921251	-0.801470644	2.0075505	4.407151075
113	TFPI	22.62581667	-2.064206691	0.54602892	-2.780511921	-8.617195282	1.140259891
114	TESPA1	554.0197281	1.080960918	0.14221885	7.542249942	2.628412021	2.889291144
115	VAC14	101.1686552	-1.146791624	0.206119256	-2.746225082	-1.612762228	1.114891821
116	SRGN	4018.50447	0.449895268	0.091800046	4.900818564	2.128457717	1.712052499
117	SLA	264.7804407	1.145206445	0.192192052	5.92822104	2.212256063	1.955094017
118	EVL	92.9189747	1.494267046	0.221962189	4.641422055	-7.466829802	1.942804701
119	CD80	792.9950464	-0.67425772	0.12274692	-5.448682925	6.62260245	1.445442025
120	CD40	41.24698272	1.892527022	0.476807199	2.969187186	-4.071652217	2.194202109
121	PORCN	145.7222915	-1.592290028	0.257409857	-6.19008944	2.464982978	1.454099012
122	CCL7	25.26616975	2.228265144	0.700521691	4.622581942	-8.415267223	1.119018504
123	CLEC4G	10.82065048	6.917159446	1.659165181	4.169060155	-4.267602952	2.111115144
124	ARHGEF6	744.5542556	-0.722065542	0.120801599	-5.527956422	1.659760946	4.199081511
125	GAB2	82.2227622	1.858128705	0.245055028	5.285050259	1.624807975	1.119048545
126	CPA2	6824.657229	-0.522265589	0.084120091	-6.227449021	1.212185407	1.102450548

127	AL122415.1	2258.459409	0.446758825	0.092848589	4.811692152	4.229591147	2.472105111
128	PROS1	259.4515952	-0.709069046	0.192741462	-2.678860997	-7.722411787	2.700910855
129	CD83	22.59171887	2.924951614	0.81104084	4.829400707	2.809502025	1.944427414
130	RPS19	924.0628782	0.552862079	0.128102501	4.222551456	-1.726222213	1.925248714
131	NCOA4	717.7205104	-0.774222102	0.124710707	-5.748026795	2.290905788	2.444141594
132	IL27RA	56.10479226	1.474992521	0.41462202	2.557246976	0.000274619	1.119018504
133	SPTB	52.00625172	-1.464452942	0.411807415	-2.556159717	0.000276215	2.45404198
134	CTSW	154.9162209	1.247004225	0.252927711	4.920279204	1.822605821	1.715888844
135	CEACAM6	20.50470525	-2.522479028	0.714558247	-2.54551728	0.000291842	2.714228851
136	TBC1D2	125.6542268	-0.959299902	0.270522952	-2.546094207	0.000290986	1.114941894
137	MYD88	424.698468	-0.55897152	0.157761248	-2.542146267	0.000295282	1.498974889
138	TIMP2	150.8991046	-0.869226585	0.24528995	-2.542266442	0.000296705	1.944440049
139	CD86	1927.778877	-1.810509026	0.502014082	-2.599220754	-4.08412761809004	4.717041555
140	FTH1	4210.22824	0.200892656	0.0850008	2.529880297	0.000400209	1.448115485
141	MAP2K1	92.2727889	1.12222218	0.217172705	2.528208555	0.000402852	2.191517914
142	NUCB2	242.290825	1.262485727	0.202462417	6.205007011	1.752215777	5.141521881
143	SGK1	187.208864	-0.785261222	0.222084881	-2.526211112	0.000405757	1.715888844
144	ETS2	116.4866542	0.99620062	0.28182921	2.525121924	0.000407587	1.472894279
145	GOLGB1	188.9275617	1.880479089	0.222271984	8.06121562	1.402182478	1.718714191
146	CD28	188.2847005	1.207197101	0.222014419	5.412089901	5.108766123	1.424401771
147	RUNX1	122.2647929	1.065162909	0.288452162	2.692684777	-5.022204624	5.181279208
148	AQP9	72.98156022	1.792756761	0.281015984	4.705200922	-2.112102705	4.281882129
149	CD209A	462.4286815	1.2298987	0.152229951	8.072282526	1.781966572	1.479517011
150	CLEC4A	40.77110002	2.144128427	0.500524226	4.282699067	-2.298141278	1.111104547
151	SYNGR2	606.1654224	1.04227872	0.140677241	7.416115947	8.824080727	2.157442949
152	DUSP14	298.2929222	-1.625045775	0.188695489	-8.612001176		

						1.587799202	1.492254157
153	AL121829.2	0.500997014	2.484998858	4.296126822	0.565270056	1.820281724	1.191259142
154	EPB41L1	172.0480269	-0.810896009	0.220262251	-2.520090658	0.000421299	1.717180171
155	APOOL	99.79156052	1.06160644	0.202014112	2.515088849	0.000429607	1.49051091
156	PTTG1IP	281.2978282	-0.568152844	0.16161224	-2.515509278	0.000428911	1.110541515
157	PTGER2	65.5920502	1.402745202	0.268411981	2.807545287	-8.14601726	1.49051091
158	EFR2B	68.72822268	-2.861268572	0.409691887	-6.984196226	5.222268212	1.718714191
159	SIRPA	542.9961002	1.968140972	0.155689221	12.64146419	1.080152262	1.19519459
160	PIK2R6	491.4899452	0.640458918	0.148291828	4.218909049	-1.68896845174817	1.942854744
161	BEST1	2986.286	0.201742979	0.082686242	2.605658084	-1.202470287	1.980514015
162	ITGAL	120.5896015	1.25871759	0.21406772	4.226192059	-1.215181283	1.481251257
163	ESYT1	967.6271165	-0.486925229	0.115292977	-4.21976281	-5.74822911158704	0.745814817
164	CD59	502.2641567	-1.01991415	0.146414261	-6.965948128	2.965059256	0.985021009
165	AL122267.1	1.227074528	-0.524749642	2.612295646	-0.204704871	-2.27827687	0.717411545
166	CTR9	245.5926804	-0.722292297	0.209410729	-2.501698792	0.000462202	1.477572045
167	IL5	12.58770957	5.662517668	1.617912429	2.499889156	0.000465452	0.745814817
168	IL4I1	92.28921882	1.772198695	0.222222026	5.481022095	1.775116801	0.498141447
169	OSM	114.6482076	2.060452126	0.298610559	6.900124857	1.174812049	0.489705489
170	ADRB2	258.049725	-1.670217571	0.179915214	-9.28290994	5.848904226	0.498141447
171	CTNNAL1	20.58290144	2.471622208	0.794299861	4.270122226	-2.825745287	0.745814817
172	TUBA1A	744.7868772	-0.670686655	0.128442289	-5.22165586	2.677624412	1.115782111
173	BTN2A2	106.1082297	-1.277576452	0.20151995	-4.227120804	-1.122907211	1.454990294
174	NOP58	156.1599817	0.841041182	0.240914882	2.491020402	0.000481161	0.974574021
175	DEPDC1B	48.25412158	-1.505274928	0.421521597	-2.488214854	0.000486257	0.49521421
176	TMEM164	158.520622	-0.854852765	0.245027526	-2.488806921	0.000485181	0.498141447
177	GBP2	96.21722852	-1.940508956	0.222297629	-6.018992451	2.152282615	1.141151148

178	KIT	2982.578117	-1.155527251	0.124149029	-9.207581118	1.18150103	0.489705489
179	ITGAM	429.6665087	-1.570248889	0.172706206	-9.092597209	1.740281209	0.484870241
180	CCL26	60.42406879	9.298605052	1.484286692	6.22206852	2.092577048	0.724522501
181	INSIG1	92.24459077	-1.517077825	0.227809677	-4.627922671	-1.97918452	0.500997014
182	CYTIP	224.1512114	-1.284584249	0.206971429	-6.206577652	2.682456923	0.484870241
183	AL256966.1	0.498161667	0.02169065	4.170279829	0.007599166	-4.152807276	1.117074528
184	SELENOP	26.00965127	-2.067696948	0.696009825	-4.407548226	-1.200717902	0.994212224
185	TRPV2	268.8788172	-0.605224987	0.168124042	-2.600211845	-1.10495767586661	0.498141447
186	AC010997.2	0.745824827	-0.972210916	2.522016256	-0.276242662	1.24622276	0.740104194
187	PTPRO	15.78727881	2.056952924	0.880067598	2.472542217	0.000512624	0.49521421
188	LRP5	26.88718297	-2.207049696	0.608897952	-2.624662695	-1.742551703	1.14110111
189	TNF	12.55408912	2.572287942	1.029794891	2.469028611	0.000522244	0.500997014
190	S100A6	1292.890272	-0.801614595	0.105946202	-7.566224672	4.28260916	1.970044018
191	LIF	229.0094991	0.822218819	0.210676055	2.950229752	-2.706294199	0.741989481
192	IER2-AS1	52.74854749	1.81222468	0.419247581	4.224180617	-7.77560526540795	0.478414242
193	CLEC10A	22.47574126	4.459104672	0.771227969	5.780999825	6.260614519	0.498141447
194	ANXA2	422.6997005	1.224624018	0.162278069	7.608076858	4.658626672	0.741989481
195	MALAT1	6926.090458	0.422884042	0.101469205	4.177460788	-6.072269498	1.440411014
196	RPS6KA1	271.1296154	0.647077626	0.187286819	2.455008948	0.000550275	1.922404758
197	UNC5B	40.92020655	-1.812967702	0.475882824	-2.809685494	-2.289541229	0.500997014
198	ANPEP	656.0149028	2.070591184	0.155277829	12.226168	6.401600721	0.714077514
199	C11orf21	61.08660776	1.245660644	0.28982266	2.451892026	0.000556668	0.478414242
200	KMT2C	257.2951121	0.956678117	0.199122298	4.804448524	-1.2752050124597	0.717411545
201	FADS2	127.7590848	-1.041724048	0.202047729	-2.448872198	0.000562922	0.500997014
202	SLC26A11	42.24125124	2.548167845	0.490242852	5.19669581	7.019170665	0.727248849
203	CTSD	2098.002712	-0.709684266	0.092792591	-7.566528014		

2.775762207	0.498141447
204	IL1RN	626.7641072	2.740921192	0.169906687	22.01750425
1.092467953	0.478414242
205	TRIM62	12.88002166	-2.227762806	0.966794101	-2.44206052
0.000577201	0.498141447
206	STT2A	418.8115887	0.528870272	0.156627005	2.440469112
0.000580707	0.478414242
207	DRAM1	89.54412968	-1.274885278	0.225178164	-2.920574681	-
2.2588705299299	0.992487988
208	TXNL4A	52.89729229	1.292755001	0.40541242	2.427869521	0.00058621
0.489705489
209	NRIP2	158.7268441	-1.081296044	0.275611505	-2.922261622	-
6.82226441	0.484870241
210	TIMD4	104.8881672	1.021261248	0.297295926	2.425502681
0.000591452	0.489705489
211	AP001218.2	22.55425122	2.565926242	0.662117271	2.869490892	-
1.152698925	0.727248849
212	ADGRE2	217.0692851	-1.210052241	0.216627267	-5.585872246
2.198104924	0.500997014
213	BTK	962.6286276	-0.78565684	0.119210296	-6.590506082
1.962102829	0.717411545
214	ST2GAL4	140.0152894	2.50701242	0.287507962	8.71980519
9.29298243	1.48884427
215	FGD2	25.08619202	2.222470415	0.54121546	4.292059951	-
1.288224947	1.107217124
216	HLA-DRB5	115.5294207	0.976822069	0.28475629	2.420417878
0.000602652	0.500997014
217	KRT19	210.2622256	-0.949225612	0.22059512	-4.202520258	-
1.240968692	0.740104194
218	LIPA	979.8954298	1.971201428	0.125095424	15.75758225
7.004688918	0.500997014
219	CD52	1018.207225	0.286952487	0.112900416	2.427287601
0.000609419	0.945184705
220	CD14	299.2495161	-0.876502678	0.159721541	-5.487248788
2.878759541	0.745814817
221	IFITM1	52.60672771	-2.02415758	0.420102426	-4.72947225	-
2.925281949	0.714077514
222	CISH	267.004247	2.41817146	0.206285095	11.72247201
6.117065008	0.478414242
223	PIK2IP1	61.84525667	-1.226022591	0.287422807	-2.42267561
0.00062008	0.484870241
224	MCC	52.25271022	-1.402612691	0.410557768	-2.418797066
0.000628986	0.498141447
225	PICALM	106.501891	1.22486499	0.295184266	4.149491402	-
2.26167905041798	0.500997014
226	AC022001.2	0.500997014	2.484998858	4.296126822	0.565270056	-
1.255581411	1.114129191
227	DSTN	278.4246221	-0.624747422	0.185811449	-2.416082482
0.000625288	0.981194441
228	SLC45A2	226.4217174	-0.614571497	0.179947197	-2.415287961
0.000627146	0.489705489

229	AC012186.2	0.987867256	0.042250627	2.875240277	0.015077222	1.247812153	1.151491525
230	GZMB	88.02668222	-2.1020902	0.242601624	-6.128588242	1.22075851	0.498141447
231	CCL4L2	114.6252242	1.250222256	0.292420982	4.617612854	-1.21225216	0.478414242
232	IL1B	417.4195659	-0.844715421	0.161827404	-5.219852982	1.074710874	0.498141447
233	TPSB2	2952.215042	-0.564100227	0.111624986	-5.052077415	4.049097291	0.748440174
234	STRIP2	144.5760047	-1.70070854	0.260774767	-6.521752686	5.798092298	0.751495511
235	CD86	42.42728429	2.208221021	0.527270746	5.971248818	1.441845096	0.49521421
236	PADI2	87.47442189	-1.958507494	0.255268989	-5.512745418	1.815122206	0.49521421
237	GPR25	28.28029504	1.802472822	0.500927207	2.598274958	-7.052462981	1.720854827
238	MEIS2	146.6464406	-0.846827215	0.248870004	-2.402688962	0.000667262	1.119859812
239	CCL22	29.05967122	8.768779697	1.506784681	5.819520692	6.216224691	0.979411277
240	MS4A6E	6.287070014	6.122949486	1.804860525	2.298572559	0.000677285	0.748440174
241	ZFP26L1	211.7107787	-0.615250877	0.181020907	-2.299148172	0.000675961	0.498141447
242	TMEM154	226.1725178	-0.580276842	0.17089257	-2.296120272	0.000682458	1.497210248
243	PTAFR	608.4187248	-0.46015421	0.125526979	-2.295295989	0.000685544	0.498141447
244	THBD	28.24987422	2.092721451	0.616796052	2.292906682	0.000691552	0.985021009
245	SERINC2	446.1911229	-0.546678955	0.161096909	-2.292478858	0.000690109	0.478414242
246	IGSF6	92.57444987	1.529259469	0.212575089	4.877171426	1.196420671	0.484870241
247	KLHDC8B	52.21908197	-1.692618972	0.422987884	-2.99449852	-2.126556212	0.741989481
248	JPH4	25.6172195	-2.289142641	0.547480712	-4.181229752	-1.22922792	0.484870241
249	KMO	28.25129194	1.941208648	0.572054407	2.287651549	0.000704928	0.751495511
250	CEACAM8	225.1006284	-0.921828851	0.217924701	-4.220079692	-1.18226279	1.471901251
251	PCED1B-AS1	22.87067008	-2.921692622	0.710281114	-4.126920708	-1.477140722	0.484870241
252	ADA	58.55929722	-1.26647455	0.402626252	-2.285494724	0.000710501	0.740104194
253	PABPC1	1086.820807	0.400270442	0.118228986	2.286111954	0.000708905	0.489705489
254	FADS1	197.1248588	-0.885878678	0.225006017	-2.76959999	-	

1.249052153	0.484870241
255	PIM1	456.6958774	-0.628019259	0.188522229	-2.284121522
	0.000714064	0.484870241
256	ARHGAP18	1171.242285	-1.299252942	0.118262885	-11.82172892
	2.91742816	0.71891187
257	MAST4	49.85261982	1.449844712	0.42874644	2.28159009
	0.000720676	0.49521421
258	PPM1H	140.8726971	-1.220549777	0.272622504	-4.512722262	-
2.45084149	0.484870241
259	RHEX	1246.176227	-0.254452562	0.104871474	-2.279875847
	0.000725186	0.489705489
260	TSC22D1	522.4152752	-0.660590025	0.147218189	-4.484102622	-
2.76518753	1.14110111
261	MBOAT7	492.1868977	-0.497585688	0.147225155	-2.277465902	0.00072157
	0.727248849
262	C12orf76	62.90649902	1.291712128	0.282719568	2.275090294
	0.000727914	0.714077514
263	CD200R1	112.5925708	1.417270762	0.288862075	4.906721845
	1.544802542	1.494197574
264	MTHFD2	576.6202646	-0.472405651	0.140126941	-2.271269274
	0.000748227	1.484019012
265	G6PD	261.9215252	1.145418828	0.194721109	5.882255751
	2.076285015	0.751495511
266	HLA-DRB1	256.6268829	0.707297412	0.170668166	4.144284259	-
1.215042187	0.478414242
267	AC009486.1	0.72891287	-0.92219097	2.55162759	-0.26246867	-
1.20852502	0.741989481
268	TNIK	211.5886517	-1.264272727	0.21242791	-6.292262615
	2.755764272	0.498141447
269	SLCO4C1	27.062499	2.242969912	0.540145876	4.22766129	-
5.055590702	0.500997014
270	RAMP1	11.22676106	5.510858246	1.628122581	2.264110092
	0.000767909	0.954818717
271	TLDC2	102.4004466	-0.981620559	0.291886087	-2.262026209	0.00077092
	0.748440174
272	PTPN7	722.021421	1.025571658	0.121445452	7.878227616
	1.260125025	0.489705489
273	AC120498.2	95.4024116	-2.167642282	0.229562419	-6.577214882
	1.258869188	0.717411545
274	SYTL4	54.02799186	-1.265580254	0.406502547	-2.259221711
	0.000781212	1.722491182
275	MITF	201.595757	-0.9502981	0.224081741	-4.240854684	-
2.276809841	0.49521421
276	CDC42EP4	29.00242015	-1.620072082	0.482958622	-2.254475875
	0.000795155	0.727248849
277	PGLYRP1	17.82422621	-2.976110476	0.824417682	-2.566691521	-
2.2215285	0.49521421
278	ST8SIA1	105.9269441	-1.410425975	0.297501478	-4.740904089	-
1.09186270565865	0.498141447
279	PEPD	226.9272512	-1.25994498	0.175477418	-7.749971441
	1.146278076	0.748440174

280	FGR	220.6222222	0.782628214	0.20760206	2.769848489	-4.969482195	0.478414242
281	AC012256.2	0.999158681	1.615997221	2.159709049	0.51142865	-1.949069785	0.714077514
282	SCD	114.2222518	-0.921402421	0.27842454	-2.245259822	0.000822055	1.111402844
283	CLEC7A	72.17642771	1.900779562	0.418717227	4.52952025	-1.272225998	0.717411545
284	DHRS9	182.2724284	-1.511875884	0.222444629	-6.476278962	1.125224979	0.974574021
285	PDCD1LG2	15.12822978	7.400247724	1.60245625	4.615185212	-1.49224948269881	0.500997014
286	CXCL16	147.8727175	-0.822119249	0.246464207	-2.229709751	0.00082866	0.489705489
287	RPLP1	1595.788426	0.224472029	0.100142542	2.229926097	0.000828007	0.740104194
288	AC024909.1	270.1251798	-0.606554029	0.181662922	-2.22889824	0.000841112	1.715184142
289	LDHA	1724.420951	-0.241252042	0.102229829	-2.22547599	0.000851525	0.498141447
290	UTS2	96.88900698	1.575478612	0.208512572	5.106675222	1.275258463	0.724522501
291	LXN	86.02102182	-1.254290144	0.225841648	-4.156282122	-1.271624449	0.724522501
292	LITAF	578.12728	-0.886912846	0.156229229	-5.676626284	1.222866828	0.498141447
293	PDZD8	652.9220429	-0.925882841	0.12905848	-6.720145752	2.609894722	0.498141447
294	BCAT1	177.7212882	-1.009820021	0.240241946	-4.202245987	-1.462511429	0.478414242
295	CFP	156.1209282	0.874491712	0.262815412	2.227298886	0.000876608	0.478414242
296	HLA-E	898.7620581	-0.488678926	0.117877207	-4.145657279	-1.22929605414412	0.724522501
297	AHCY	181.7520521	1.062172767	0.22065588	4.609250277	-2.126221169	0.751495511
298	AP005121.2	0.987867256	0.042250627	2.875240277	0.015077222	-4.529254114	1.719545511
299	STAT4	26.22654229	2.148816482	0.682592725	4.612022792	-4.221822583	0.498141447
300	PPT1	616.4490055	0.446857556	0.124470262	2.222092277	0.000890251	0.49521421
301	VIM-AS1	1556.022242	0.266844701	0.11041027	2.222556588	0.000891966	0.714077514
302	ELOAP1	25.46895402	-2.020226842	0.608295767	-2.221125927	0.000896551	0.981194441
303	DUSP6	1059.29486	-0.287295252	0.116671644	-2.22028994	0.000898918	0.489705489
304	AL096701.1	0.478414262	-2.271055522	4.479204761	-0.529247421	2.070046123	0.994212224
305	FCER2	122.6801264	6.977789092	0.764192714	9.120928572	1.0620069	

1.115782111
306 VSIR 501.5227402 -0.496204076 0.149609124 -2.217228068
0.000908796 0.489705489
307 TPST2 205.0512522 -0.924770804 0.215947272 -4.282289694 -
6.190287723 0.500997014
308 PAPSS1 404.8180702 -0.58857022 0.157920048 -2.727014005 -
7.021229206 0.71891187
309 QPCT 28.52844744 2.272165248 0.592245801 4.006285027 -
2.279279624 1.14110111
310 SPP1 259.6272695 0.822789188 0.176524192 4.722270666 -
5.700569499 0.979411277
311 DDIT4-AS1 252.9875262 -0.740718629 0.194171675 -2.814761485 -
1.81869060745765 0.489705489
312 MANSC1 14.96066125 -2.902621741 0.876656459 -2.211012922
0.000929586 0.478414242
313 COBLL1 29.92672751 -1.825779229 0.554622665 -2.209901045 0.00092229
0.714077514
314 GAS7 69.80976678 -1.17674227 0.255226911 -2.211720271
0.000927242 0.745814817
315 FABP5 22.7215546 1.729550006 0.525571042 2.209828476
0.000922522 0.717411545
316 ACTB 16827.82927 0.284529528 0.085942621 2.210652869
0.000920786 0.49521421
317 MAP1B 85.19450726 -1.121158148 0.241905722 -2.20829197
0.000928224 0.979411277
318 TSPYL2 24.77908717 -2.251792562 0.567121257 -2.970498068 -
8.245524956 1.94140804
319 BMP2K 109.9772617 -0.977449478 0.295552079 -2.207198794 0.00094224
0.498141447
320 SCIN 225.0275927 -1.995708704 0.227985521 -8.752664261
2.058770452 0.751495511
321 GNG2 169.2967716 -0.991724107 0.222420267 -4.248704256 -
4.907469052 0.999158481
322 CARMIL1 24.29297696 -1.707628291 0.517292452 -2.201088926
0.000962102 0.724522501
323 GGT5 112.148099 2.895101249 0.27528825 10.27619125
1.420507928 0.489705489
324 PLAT 907.728287 -1.512857585 0.125115667 -12.09966447
1.429571672 0.985021009
325 IL2RA 254.5822262 2.267066674 0.212799229 11.12247229
1.720572297 0.484870241
326 LGALS9 114.7551916 1.116220801 0.286022511 2.902912284 -
2.140052463 0.745814817
327 AC009229.4 0.486870242 -2.292502217 4.077744474 -0.586967215 -
1.000559653 1.711400858
328 SNX10 82.54281282 1.562072241 0.242911054 4.55522096 -
4.91882095604556 0.71891187
329 GLUL 6047.849141 0.592012961 0.078758246 7.529524858
4.929174526 1.128215801
330 DNASE1L2 5.799742744 6.01702265 1.828576646 2.29055524
0.000999898 0.748440174

331	SLC18A2	1268.752792	-1.129891921	0.115012925	-9.824042127	1.075566228	0.999158481
332	C8G	72.60146829	-1.559666592	0.25899428	-4.244544408	-5.21066601654611	0.484870241
333	CCDC152	18.20414462	-2.760226402	0.898260002	-4.186122624	-8.01240756120416	0.49521421
334	PTPRE	184.8012645	1.906808421	0.24291848	7.849581594	1.152929283	0.489705489
335	ARHGAP22	1.972849202	-4.409976871	2.478405555	-1.779260469	-4.22207499	0.484870241
336	NFE2	115.0979442	1.192546462	0.2807785	4.24728554	-1.7974408166776	0.724522501
337	OSM	72.57642122	1.776972576	0.285482005	4.609722608	-1.15521444460462	0.484870241
338	CCL8	18.86551295	4.202598178	0.952012616	4.415481154	-2.280022516	0.71891187
339	SMYD2	151.1785828	-1.201182949	0.24944904	-4.815248042	1.676972026	0.489705489
340	LYZ	1080.572216	-1.691725491	0.144288225	-11.71649775	1.101186803	0.484870241
341	ALDH2	62.86072952	-1.224929864	0.272184208	-2.282299178	0.001029278	0.741989481
342	MT-ND2	1946.249126	0.221006655	0.097782524	2.282829261	0.001027709	1.111402844
343	SIGLEC10	486.6297585	-0.760626226	0.148928487	-5.107225221	1.605867017	1.118418559
344	SLC10A7	92.9117127	1.615220422	0.220749976	5.025792847	1.427692568	0.974574021
345	PSMD2	189.2824781	-0.770726476	0.224916821	-2.280848257	0.001024954	0.498141447
346	SLC28A2	106.4029975	-1.07494911	0.295675651	-2.625568596	-2.687669688	0.71891187
347	RPS6	2484.481259	0.202274994	0.092217512	2.278922481	0.001042006	0.49521421
348	KCNK5	28.8566929	2.018450778	0.625792815	4.747528262	-2.280700673	0.71891187
349	CNIH1	252.0807985	-0.81277254	0.169711295	-4.795027725	-1.02991518698185	0.498141447
350	PROCR	77.8588169	-2.442162722	0.278066415	-6.459615658	2.955528608	0.972740484
351	IL6ST	67.49926815	-1.220929028	0.275841265	-2.2751567	0.001056024	0.489705489
352	CRLF2	97.20959275	-1.272429428	0.210227296	-4.104727104	-2.422627206	0.741989481
353	RAB27B	670.2715614	-0.476114799	0.121886129	-2.610044261	-2.026674165	1.94140804
354	ABRACL	222.5222074	-0.656288787	0.200624484	-2.271066746	0.001071426	1.48025822
355	AP001880.2	12.67176191	-2.009922244	0.920504222	-2.269862579	0.001075997	0.489705489
356	EDEM2	464.6110874	-1.028220686	0.151780282	-6.774462672		

1.419572256	1.984975975
357	CNIH2	18.15207797	-2.4727047	0.757251091	-2.265269612
0.001092212	1.125520514
358	LTA4H	142.1797146	-0.81522719	0.24987197	-2.262979801
0.001102474	0.498141447
359	DOK2	102.2456817	0.960149252	0.294221225	2.262257454
0.001101006	0.478414242
360	CDK6	208.762896	1.947224906	0.25252064	7.711281725	6.18015491
0.751495511
361	ABCA1	96.96895212	-1.027722782	0.218122625	-2.261911562
0.001106627	0.49521421
362	CCL18	272.5949805	5.580466944	0.264065275	15.22820429
1.021119895	0.745814817
363	VIM	6572.852297	0.416614222	0.079712829	5.226428284
2.129977467	0.478414242
364	AL512217.1	0.49522622	-2.415608216	4.416671772	-0.546929552
2.922628085	0.714077514
365	XPR1	70.999675	-1.186689425	0.264191929	-2.258417622
0.001120254	0.498141447
366	P2RX1	669.0502502	-0.425520629	0.122677265	-2.258072191
0.001121715	0.500997014
367	AP1B1	269.2240517	0.869125747	0.196028505	4.422670227	-
1.029652623	0.727248849
368	TGM2	1578.125691	8.571266862	0.266807584	22.26747448
1.628952723	0.489705489
369	GRAP2	201.2064552	0.590161251	0.181209768	2.254989278
0.001122966	0.500997014
370	TRAM2	26.2927602	-1.64172957	0.504968828	-2.251170129
0.001149211	0.49521421
371	SLC8A2	28.55484202	-1.596889175	0.491205428	-2.250959887
0.001150161	0.748440174
372	EBP	194.0185615	-0.728826948	0.224140922	-2.251645906
0.001147289	0.489705489
373	ANTXR2	125.552555	-1.216722924	0.282921252	-4.200420984	-
2.0615705098698	0.484870241
374	CCL4	126.9088978	1.149892625	0.268712445	4.279268217	-
2.525905526	0.985021009
375	PAPSS2	27.56647791	2.508246209	0.645667564	2.884722224	-
5.90708521804167	0.748440174
376	AC102681.2	0.49522622	-2.415608216	4.416671772	-0.546929552
1.096060268	0.478414242
377	AC011676.5	0.748660174	1.029647608	2.51687248	0.292772655	-
1.78766262	0.484870241
378	TMEM106A	64.41052486	1.252729122	0.285769695	2.2472757
0.001164745	1.442444272
379	RNF120	422.8714766	-0.655122499	0.176095402	-2.720270212	-
1.0729555689499	0.484870241
380	HDC	2862.959547	0.486429074	0.091277855	5.222270895
1.696245611	1.711400858
381	ADCYAP1	92.17728009	-1.144501988	0.211010529	-2.679946117	-
1.58498626	0.500997014

382	PRTN2	755.4040406	0.529652274	0.142625411	2.78242829	-4.84475597228745	0.500997014
383	CTTN	82.27018297	-2.528927024	0.26264845	-6.981817288	1.656629681	0.489705489
384	KIAA1217	102.5210686	-1.678191892	0.218821409	-5.262726512	1.626954127	0.748440174
385	PTGS1	800.2815484	-0.542775609	0.122198578	-4.412814005	-2.7004571494268	0.49521421
386	SLC22A25	42.29720145	-1.494968719	0.461120089	-2.24196741	0.001187076	0.500997014
387	CD200LF	121.111182	0.851242279	0.262758841	2.240014214	0.001195227	0.748440174
388	AL025661.1	0.751495521	2.069950829	2.726554747	0.822804022	-1.622768812	0.990701702
389	JAK2	160.4156681	-0.782091746	0.241871985	-2.227628977	0.001205274	0.714077514
390	CLMN	22.95870199	-2.126794477	0.657101024	-2.226622272	0.001209491	0.724522501
391	SGPL1	168.5870649	0.990712299	0.245748044	4.021415195	-4.57710896841604	0.741989481
392	ATP10A	89.70064942	-1.296672817	0.21874951	-4.067999404	-1.172204483	0.972740484
393	TREM2	41.97826152	4.02277992	0.61408271	6.552504826	8.62451493	0.489705489
394	PTGS2	92.85424966	-1.002689081	0.210198025	-2.222415968	0.001227482	0.714077514
395	DUSP10	206.162552	-0.827427296	0.218255829	-2.791125292	-1.477644527	0.500997014
396	SDSL	89.72512952	-1.022677541	0.219919217	-2.221057986	0.001222229	1.442444272
397	MS4A4A	42.62552145	2.079712848	0.525268921	5.862002221	1.927766274	0.49521421
398	NCF2	295.8422759	1.496002722	0.186555781	8.019062858	1.172404429	0.987847254
399	VMP1	662.9959601	0.542452589	0.122544525	4.092606521	-5.542482623	0.484870241
400	PSMD1	612.0722092	-0.422286984	0.124188278	-2.228948087	0.001242464	1.971899245
401	CYRIA	52.65994282	-1.744061266	0.4507882	-2.868914222	-2.845686728	0.498141447
402	GPAT2	101.2620409	1.646271662	0.209707914	5.215885021	1.756921729	0.741989481
403	AMIGO2	24.42916259	-2.569810485	0.65965994	-2.895659461	-2.178992768	1.128245842
404	ALDH1A2	26.77062882	1.91975228	0.59541567	2.22422264	0.001262147	0.974574021
405	ACE	15.81542976	2.59067727	0.921769875	2.85260952	-1.12126616	0.745814817
406	TPSD1	1621.402655	-0.50872781	0.125811607	-4.042568182	-1.027667275	0.748440174
407	TEX20	65.7051528	1.596949911	0.274007482	4.269824118	-	

1.255876984 0.484870241
408 NTRK1 144.7728288 1.086406852 0.278212021 2.902557052 -
1.720229825 0.987847254
409 DRD2 50.02181199 -1.286805547 0.420555459 -2.220968447
0.001277582 1.482192474
410 FABP4 12.82756916 7.27199964 1.616654128 4.498178995 -
1.02810201587152 0.489705489
411 GPNMB 802.4720408 -1.278427027 0.127258912 -10.02806492
5.264590185 0.478414242
412 CLEC4O 102.9987578 -1.224698846 0.298167644 -4.140955169 -
2.460744029 0.500997014
413 PLAU 144.4152988 0.952889074 0.250526146 2.802299591 -
4.012562911 0.478414242
414 AL157895.2 295.8421456 -0.511526771 0.159071122 -2.21577272
0.001200924 0.999158481
415 NFATC2 229.2474767 1.192274671 0.17680426 6.744024214
1.792269201 0.999158481
416 ARRDC2-AS1 0.985022009 -1.54422206 2.962499952 -0.52129252
1.202692708 0.745814817
417 TMEM272 224.2194092 1.806224222 0.209862609 8.606746274
1.100527722 1.497210248
418 CSF1R 229.440221 0.998992584 0.226970455 4.401425657 -
1.21595876 0.49521421
419 CCL12 270.8511424 8.115761959 0.745902718 10.88045624 2.1465299
0.985021009
420 RNASE2 22.09180014 -2.162087185 0.672825612 -2.212295885
0.001211752 0.979411277
421 SREBF2 64.75924141 -1.655889498 0.291702192 -4.227408742 -
1.12612272 0.740104194
422 SDC4 42.49917266 1.798227698 0.467111556 2.849675027 -
1.299264043 0.500997014
423 MYC 94.54525508 1.228754162 0.208964896 4.222029998 -
1.270167646 0.478414242
424 ASAH1 964.5209094 -0.517080119 0.117521624 -4.299497408 -
1.421549253 0.745814817
425 ATP6V0A2 298.5498029 -0.729254941 0.180156187 -4.102966419 -
5.882427202 0.745814817
426 FAM174B 44.1754479 -2.002746228 0.469212451 -4.269525644 -
1.024915007 0.987847254
427 PLPP1 259.5820706 1.149262478 0.224669682 5.115788052
1.072481205 0.478414242
428 QSOX1 259.5601478 2.089991696 0.176045221 11.87190249
1.570754466 0.478414242
429 ARHGAP25 600.9799228 -0.454708657 0.14172826 -2.208086917
0.001226211 0.489705489
430 BIRC2 147.1117426 2.21844122 0.268292525 8.268740428
2.985077462 0.478414242
431 DHTKD1 109.6540156 1.149267462 0.286926751 4.005787051 -
2.420655729 0.972740484
432 SCIMP 25.61444118 2.249145458 0.691540784 4.698414802 -
1.152821426 0.727248849

433	AC008608.2	0.478414262	-2.271055522	4.479204761	-0.529247421	-1.492824019	0.489705489
434	CD22	208.4600281	1.0812851	0.212092202	5.074704812	1.249172295	0.484870241
435	FGL2	42.62411026	2.584454568	0.499494549	5.174129682	5.269825017	0.500997014
436	ETV5	82.27604269	-1.144029442	0.257206887	-2.202726228	0.001261286	1.104491887
437	RPS6KA5	222.2545928	-0.699286886	0.218220442	-2.202882099	0.001260592	0.714077514
438	ABLIM2	5.569412008	-5.910420694	1.845814215	-2.202066951	0.001264452	0.484870241
439	IL9R	188.6798061	1.26428402	0.222525847	5.655844669	1.221021107	0.498141447
440	AC090192.1	0.489705689	0.05402189	4.196172952	0.012876469	-1.547582719	1.715124105
441	SAT1	1050.697224	-0.640679888	0.11277292	-5.621166496	1.062086001	1.715124105
442	LGALS8	548.2082802	-0.598044779	0.141045745	-4.240076706	-1.106462188	0.748440174
443	CXCL8	600.2212914	1.512222467	0.152524898	9.921216076	1.114826912	0.498141447
444	IL10	64.26462829	-1.967181265	0.402005575	-4.88127556	1.77678441	0.981194441
445	LAT2	517.5570985	-0.872562654	0.145182067	-6.01009244	1.562487689	0.727248849
446	S100B	100.0221064	1.277826594	0.207290601	4.158265275	-1.225452089	0.751495511
447	AC011220.2	0.727268849	-0.952727871	2.205072418	-0.288262241	-1.01901057	0.489705489
448	RBPJ	225.0500244	0.802486842	0.204784997	2.922562028	-2.856204523	0.489705489
449	PLXNA2	91.11922177	-0.992566746	0.210825077	-2.192226208	0.001406926	0.500997014
450	ENPP2	927.4924728	1.152912102	0.118702188	9.712562212	1.979228929	0.484870241
451	AC008875.1	0.489705689	0.05402189	4.196172952	0.012876469	1.508686227	1.444181719
452	FLNA	252.1417994	0.522878091	0.166980824	2.191252205	0.001416571	0.714077514
453	AL256805.1	0.748660174	1.029647608	2.51687248	0.292772655	-1.998959227	0.974574021
454	IL2RG	245.4026488	0.72102641	0.177868592	4.109924079	-5.228055679	0.724522501
455	KCNMB1	292.0859282	2.1010022	0.200612824	10.47287247	1.640428226	0.500997014
456	SYTL2	157.2649282	-1.042070622	0.240715186	-4.222214901	-1.248251753	0.484870241
457	GBE1	228.0174822	-1.427189855	0.191976098	-7.486295811	2.822401772	1.442444272
458	IL2RA	191.4092645	-0.925768807	0.222222992	-4.164052646	-	

1.545454871	0.945184705
459	ANXA4	924.4758259	-0.625247727	0.119965968	-5.211875821
	1.620067466	0.49521421
460	CD274	67.64561222	2.765778677	0.471468652	7.987226292
	1.517067725	0.498141447
461	ETNK1	402.9578502	0.582086618	0.15948629	2.649757214	-
1.10179021575084	0.49521421
462	BMERB1	62.11800572	-1.484256151	0.278198561	-2.92454204	-
1.15080156461785	0.49521421
463	LDLR	84.09518542	-1.165005011	0.224474988	-2.590420865	-
2.991216953	0.71891187
464	TRIM4	79.05225484	-1.184412709	0.272201987	-2.181222555
	0.001466042	1.115782111
465	PLCG1	16.62789572	-2.486885282	0.78192802	-2.18041241
	0.001470656	0.489705489
466	ALAS1	2489.278082	1.672475926	0.086761648	19.2881989
	2.468219066	0.484870241
467	CCL24	242.5277187	8.272129294	0.852527025	9.808862579
	2.255264218	0.484870241
468	AC007228.1	0.498161667	0.02169065	4.170279829	0.007599166
	0.990081852	0.49521421
469	IL17RB	24.4248508	4.76972275	0.794872202	6.000608824	4.59866254
	0.727248849
470	CKB	100.2728629	2.180182689	0.251226601	9.054249129
	6.020579902	0.985021009
471	HS2ST1	472.5781864	1.126071825	0.160462742	7.079972614
	1.280209456	0.985021009
472	AL596244.1	0.742989481	-2.000642057	2.742921251	-0.801470644	-
2.942217481	0.500997014
473	CD180	21.28276226	2.802821261	0.729878168	2.84149216	-
1.01916216787824	1.115782111
474	CAVIN1	40.29624622	-1.897261646	0.510568916	-2.715975624	-
2.208687623	0.498141447
475	VCAM1	7.8027218	6.445528858	1.722262676	2.718729555	-
1.002262623	0.49521421
476	PIM2	108.1902488	1.692048057	0.296062149	5.718556274
	2.208861162	1.125520514
477	PRKCQ	24.11458907	2.958226161	0.798257608	4.958099121
	1.455722504	0.981194441
478	CD82	154.7824222	1.27919096	0.268182212	4.769828015	-
2.842050255	1.711109522
479	AC104819.1	0.49522622	-2.415608216	4.416671772	-0.546929552
	0.950751408	0.740104194
480	IFITM2	721.962692	-1.512282998	0.141827487	-10.66982792
	1.042812271	0.478414242
481	MMP9	211.5095175	1.977482285	0.224822295	8.795718447
	2.784144557	0.489705489
482	STING1	292.2824288	-0.956456522	0.161724507	-5.912744222
	1.924891691	0.500997014
483	SLAMF1	12.20562579	2.010867771	0.951104204	2.165654992
	0.001547242	0.500997014

484	AL807761.4	0.717621545	-2.955950852	2.792242589	-0.779267242	2.970272248	0.974574021
485	AC097658.2	0.724522502	-2.98570617	2.452708589	-0.86474217	-1.429100502	0.745814817
486	CTSG	1826.929602	-0.890261144	0.097198617	-9.160224419	2.456715778	0.972740484
487	CPM	129.5629622	1.055124642	0.2609827	4.042912952	-2.011082217	0.740104194
488	STOM	191.5696669	-0.690197228	0.218259064	-2.162284872	0.001565262	0.49521421
489	IL4R	142.5021896	-1.60519984	0.259725288	-6.18012529	1.724211187	0.741989481
490	SNX20	219.6826161	1.00697589	0.221600492	4.247900468	-1.129629972	0.49521421
491	NDFIP2	278.1291791	1.946715222	0.17110954	11.27701221	2.01406823	0.498141447
492	SLC7A8	191.7492172	2.800654614	0.250975227	11.15908787	1.187969852	0.500997014
493	EGR2	42.28256072	2.716647019	0.506172527	5.267027666	1.456929793	0.49521421
494	SPTBN1	75.84625169	-1.222906529	0.247712974	-2.804592552	-1.826655151	0.49521421
495	CD209	122.4012447	5.601022699	0.501760402	11.16274556	7.414228011	1.117074528
496	SLC29A8	40.26468692	1.742987617	0.482008225	2.616095174	-2.147860164	0.974574021
497	KIAA1522	121.5281774	0.827226864	0.265227564	2.15651694	0.001596656	0.714077514
498	TAOK2	209.5952997	-0.71202299	0.187211721	-2.806665099	-2.221181266	0.987847254
499	MTHFD1	472.4998864	-1.189100611	0.152606878	-7.741192806	1.189668192	0.954818717
500	AC004988.1	29.45669028	-2.999862214	0.64076955	-4.681655284	-4.095020029	0.484870241
501	SPART	249.4174807	-0.852042592	0.197492599	-4.219242996	-2.629220618	0.498141447
502	HSPA2	29.04471257	-2.672262272	0.526285778	-4.984029185	4.509584541	0.49521421
503	PRSS57	49.48280282	1.647545705	0.46148687	2.570081429	-4.456294675	0.478414242
504	LTF	50.21899788	-2.228286922	0.524262888	-6.248285852	1.625028597	0.484870241
505	AL451074.2	0.740204196	1.049207872	2.521140276	0.297158266	2.927422912	0.484870241
506	DUSP4	122.0684119	0.848452414	0.269276692	2.149687558	0.001624452	0.489705489
507	FAM82D	20.81161022	-2.174600222	0.691474798	-2.144872869	0.00166159	0.500997014
508	EYA2	255.2106251	-0.604854202	0.192242482	-2.144657122	0.001662816	1.115782111
509	UGP2	241.7778166	0.548502749	0.174755296	2.128695996		

						0.001697014	0.498141447
510	SORBS1	49.82268044	-1.275175559	0.428559992	-2.125661216	0.001714672	1.494197574
511	AC007229.1	1.497220248	1.024785281	2.607514715	0.292012272	0.970520404	0.484870241
512	AC027227.5	22.09820246	-1.625220662	0.522025469	-2.126482811	0.001769102	0.484870241
513	EEF2	1254.511528	0.221174109	0.106002722	2.124174224	0.001782048	1.107217124
514	ANKRD28	75.25642226	-1.080527565	0.246018265	-2.122746291	0.001791721	0.49521421
515	UQCRC2	291.0541919	0.50222856	0.1608556	2.122916212	0.001790687	0.478414242
516	RBM47	56.90661529	1.216595595	0.422206698	2.117628968	0.001822122	0.71891187
517	ART4	0.498161667	0.02169065	4.170279829	0.007599166	0.864502879	0.740104194
518	VPS12D	97.04794222	-0.972078415	0.212241282	-2.112221627	0.001856788	0.49521421
519	AC087500.1	9.209255792	5.216644807	1.677275592	2.110189422	0.001869674	0.751495511
520	UBA7	296.6028118	-0.559992682	0.180081922	-2.109655192	0.001872059	0.478414242
521	TNFRSF14	259.222825	0.628542212	0.20268849	2.10102568	0.001928516	1.128245842
522	SLC25D2	5.124822558	-5.788945852	1.869486408	-2.096542429	0.001957912	0.981194441
523	AC009097.2	0.498161667	0.02169065	4.170279829	0.007599166	-1.114280252	0.500997014
524	TMED8	92.56126779	-0.961142648	0.210672274	-2.092749969	0.00197644	1.955987408
525	AC072592.5	0.726077524	-2.971191046	2.466857824	-0.85702708	-0.914299045	0.724522501
526	AL129021.2	0.49522622	-2.415608216	4.416671772	-0.546929552	1.246016653	1.705488901
527	RICTOR	57.81984997	1.202489474	0.289117421	2.090299682	0.001999546	0.49521421
528	NT5E	80.17620741	1.015900621	0.228918626	2.088607787	0.002010967	0.714077514
529	CLNK	99.81105644	0.915696158	0.296442627	2.088949006	0.002008659	0.49521421
530	MMP19	26.69097294	1.520200216	0.492427607	2.087258008	0.002019442	0.489705489
531	SLC29A11	567.8642087	-0.426202721	0.128166867	-2.084695568	0.002027606	1.001994018
532	TMOD1	75.42052204	1.05010892	0.240852924	2.080826998	0.002064265	0.500997014
533	RAC2	2040.248692	-0.289882502	0.094078894	-2.081280952	0.002061121	0.49521421
534	AK7	11.5519929	2.082514566	1.001129725	2.080024974	0.002069762	0.484870241

535 RGS2 142.4070214 -0.808468227 0.26291298 -2.07504117
0.002104725 1.001994018
536 REEP5 249.2880189 0.51476817 0.167497215 2.0722929
0.002117099 1.491449455
537 AC007448.2 0.486870242 -2.292502217 4.077744474 -0.586967215 -
1.177625913 0.751495511
538 ANXA11 162.2751098 -0.796616024 0.259514228 -2.0696417
0.002142157 0.498141447
539 AC120271.2 0.478414262 -2.271055522 4.479204761 -0.529247421 -
2.724997208 0.724522501
540 TMEM45A 41.45054272 -1.452455199 0.47404292 -2.066077017
0.002168874 0.724522501
541 PIK2R5 102.2075524 0.917015187 0.299225547 2.06260482
0.002186876 0.489705489
542 AL157292.4 0.489705689 0.05402189 4.196172952 0.012876469 -
1.195498026 0.484870241
543 DOCK11 108.9905074 -0.915056761 0.200126179 -2.048906841
0.002296757 1.142984495
544 AC082899.1 0.745824827 -0.972210916 2.522016256 -0.276242662
2.758166567 0.748440174
545 RNASE1 8.7999028 5.128187682 1.689509279 2.041220626
0.002256122 1.125520514
546 SLC7A5 124.9645592 0.821848449 0.270274566 2.040790922
0.002259576 0.498141447
547 AC099804.1 0.745824827 -0.972210916 2.522016256 -0.276242662
1.924072674 0.985021009
548 SLC7A11 60.29227642 1.266628128 0.417295244 2.024625225
0.002408248 0.498141447
549 AC078950.1 0.486870242 -2.292502217 4.077744474 -0.586967215
1.188115875 0.724522501
550 TENT5C 25.82667496 -1.509271295 0.498269907 -2.02922427
0.002451826 0.714077514
551 LDLRAD2 55.074087 -1.229658848 0.40920289 -2.029447928
0.002450011 0.724522501
552 KCNIP1 42.2627252 1.62425872 0.540204551 2.024699156
0.002488806 0.498141447
553 AC008080.2 0.478414262 -2.271055522 4.479204761 -0.529247421
1.515419929 1.491499717
554 ADH6 0.985022009 -1.54422206 2.962499952 -0.52129252 -
1.268228582 0.498141447
555 RPL12A 1529.248109 0.216085104 0.104766427 2.017045465
0.002552515 0.745814817
556 SNAI2 25.18205659 1.599982822 0.52086196 2.012922828 0.00257884
0.999158481
557 AC018259.1 0.751495521 2.069950829 2.726554747 0.822804022
0.871961875 0.979411277
558 UNC12D 410.4126042 -0.492666021 0.164102275 -2.008262505
0.002627461 0.500997014
559 AP001148.1 0.717621545 -2.955950852 2.792242589 -0.779267242 -
1.172215541 0.489705489
560 AC008074.2 0.748660174 1.029647608 2.51687248 0.292772655

2.192597203 1.485978941
561 SLC22A10 29.96795722 -1.429264896 0.476656221 -2.998522472
0.002712912 0.49521421
562 AP002025.1 0.500997014 2.484998858 4.296126822 0.565270056 -
1.525599823 1.128245842
563 AC009065.9 0.717621545 -2.955950852 2.792242589 -0.779267242
1.221614592 0.745814817
564 AC010247.1 0.72891287 -0.92219097 2.55162759 -0.26246867 -
0.789845549 0.489705489
565 AC011511.5 115.8244422 0.827229299 0.280957666 2.979912992
0.002882202 0.500997014
566 AC016550.2 0.486870242 -2.292502217 4.077744474 -0.586967215 -
2.275280499 0.745814817
567 CERS5 151.051872 0.749959226 0.251967892 2.976408245
0.002916462 0.745814817
568 CCR7 12.79209292 2.700122967 0.907817428 2.97420282
0.002926551 0.484870241
569 REXO5 78.28008281 -1.011256606 0.240872192 -2.966672806
0.002010402 0.985021009
570 TP52I11 62.89028088 -1.192196412 0.402008102 -2.965602952
0.002020902 1.711109522
571 CSF2RA 50.25897089 1.260828997 0.425228907 2.964989741 0.00202692
1.480408291
572 AC092070.1 0.727268849 -0.952727871 2.205072418 -0.288262241 -
0.999156916 0.478414242
573 AC062977.2 0.745824827 -0.972210916 2.522016256 -0.276242662 -
1.202140184 0.484870241
574 NCAPH 24.22592284 1.818081222 0.612970228 2.961188102
0.002064547 0.740104194
575 AC007684.2 0.748660174 1.029647608 2.51687248 0.292772655 -
0.844181418 0.484870241
576 EEF2K 149.0217052 0.741012527 0.25050226 2.958105965
0.002095257 0.992487988
577 HSP90B1 829.7099657 0.259879595 0.121702546 2.957018162
0.002106298 1.442444272
578 AC244024.1 0.724522502 -2.98570617 2.452708589 -0.86474217 -
2.799428185 1.142984495
579 LINC00877 10.46611624 -2.229825642 1.092502528 -2.95265818
0.002140216 0.489705489
580 TSPAN15 4.520846216 5.661167622 1.918278401 2.951171024
0.002165716 0.974574021
581 OGG1 87.10717672 0.924226895 0.216578804 2.951008988
0.002167277 0.987847254
582 GPR65 179.1658487 0.706978028 0.229512175 2.951741499
0.002159872 0.498141447
583 SELENOT 261.8104488 0.520478228 0.17627541 2.952642847
0.002150662 0.745814817
584 AL109615.2 0.498161667 0.02169065 4.170279829 0.007599166 -
2.589669026 0.985021009
585 STAT2 261.2942802 -0.517241259 0.175627518 -2.94492605
0.002220214 0.717411545

586	SUPT20H	202.2516277	-0.622716902	0.215019218	-2.942604925	0.002254625	0.727248849
587	AC016255.1	0.500997014	2.484998858	4.296126822	0.565270056	1.082269147	0.974574021
588	AL512542.1	0.49522622	-2.415608216	4.416671772	-0.546929552	2.822628589	0.478414242
589	AL691422.2	1.722692182	-0.281477417	2.258626499	-0.168897964	-1.779677227	0.745814817
590	HK2	42.28261026	-1.261228845	0.462816492	-2.924842555	0.002227159	1.142984495
591	CD1C	10.57197426	2.200850149	1.124788524	2.924640646	0.002229242	0.751495511
592	FES	242.8216888	0.586812852	0.199929177	2.924961826	0.002225887	0.972740484
593	NDRG2	48.15810614	-1.248224108	0.459805989	-2.9221804	0.002265912	0.717411545
594	ECM1	114.0702626	-0.845960818	0.288589224	-2.921265502	0.002274755	0.500997014
595	AC005908.1	0.500997014	2.484998858	4.296126822	0.565270056	1.064272013	0.478414242
596	AC015922.2	0.727268849	-0.952727871	2.205072418	-0.288262241	-2.540129598	0.498141447
597	FNDC2B	95.62222992	0.916222868	0.212928027	2.928128857	0.002410087	0.498141447
598	AL022519.2	0.72891287	-0.92219097	2.55162759	-0.26246867	-2.269454487	0.500997014
599	ACAA1	169.4496724	-0.750479062	0.25649855	-2.925860846	0.002425045	0.500997014
600	MOB2B	14.91012777	-2.482675228	0.848762766	-2.925048672	0.002444024	0.489705489
601	AC068870.2	0.500997014	2.484998858	4.296126822	0.565270056	-2.082295103	0.484870241
602	AL122629.2	1.992646669	0.022827959	2.201657861	0.009922295	-2.700521647	0.49521421
603	DDB2	116.672597	0.811762218	0.27786581	2.921418502	0.002484414	1.440411014
604	ITGAM	206.1425422	0.576200726	0.197270067	2.91989922	0.002501445	0.478414242
605	ABCC12	0.745824827	-0.972210916	2.522016256	-0.276242662	1.219275826	0.498141447
606	ADAM9	100.9658059	1.086200562	0.272217689	2.918454959	0.002517707	1.947481248
607	AARS1	264.8504259	-0.560258028	0.192229061	-2.914902074	0.002558002	0.498141447
608	AP001160.4	0.976576021	-1.520755908	2.970112194	-0.515286561	0.92211542	0.478414242
609	DMD	24.28740629	-1.482261176	0.509060228	-2.911759925	0.002592987	1.117074528
610	LINC01140	16.1284928	-2.270452215	0.780842849	-2.907690465	0.002641085	0.484870241
611	ABCC4	242.1258799	0.597252625	0.205412472	2.907577204		

						0.002642404	0.49521421
612	PLPP2	21.22009202	1.950108718	0.671512227	2.904055272	0.00268262	0.71891187
613	GOLM1	22.71487286	1.872965518	0.644856227	2.904469882	0.002678756	0.500997014
614	PALLD	68.44080248	1.042705918	0.25929166	2.90409054	0.002682216	0.478414242
615	AC010528.1	0.498161667	0.02169065	4.170279829	0.007599166	-1.271215028	0.489705489
616	CELSR1	12.71478085	-2.624264197	0.908076424	-2.901027982	0.002719288	0.484870241
617	AC008121.2	0.726077524	-2.971191046	2.466857824	-0.85702708	1.764694722	0.745814817
618	AC018442.2	0.489705689	0.05402189	4.196172952	0.012876469	-1.790807956	0.489705489
619	AC090651.1	0.742989481	-2.000642057	2.742921251	-0.801470644	0.79262827	1.958811755
620	HLA-DPA1	267.0777625	0.502787682	0.174152106	2.887044006	0.002888798	0.985021009
621	TIMM8A	49.92012647	-1.202770929	0.417287072	-2.884062754	0.002925792	1.950214715
622	TDO2	4.281822078	-5.562444242	1.929982822	-2.882629252	0.002942588	0.484870241
623	INKA1	69.28791024	-1.020204728	0.252870761	-2.882986818	0.002929229	0.49521421
624	ACTN4P1	0.748660174	1.029647608	2.51687248	0.292772655	-1.529872629	1.119859812
625	MARCO	8.076982041	-4.955522661	1.720215674	-2.880594961	0.002969254	0.714077514
626	HMOX1	104.0022507	0.842494464	0.292492652	2.880285455	0.002971892	0.741989481
627	TIAM1	142.4424845	0.715751957	0.248571762	2.879458024	0.002982592	0.498141447
628	SASH2	252.7715014	0.55267812	0.192225227	2.87871289	0.002992004	0.500997014
629	AC004222.2	0.740204196	1.049207872	2.521140276	0.297158266	-2.428697718	0.994212224
630	IL18BP	105.7295247	0.8262411	0.290814005	2.875518665	0.004022642	0.478414242
631	AC072525.1	0.985022009	-1.54422206	2.962499952	-0.52129252	-0.719899588	0.500997014
632	GASAL1	15.91465255	-2.24664246	0.781652044	-2.87422082	0.004050257	0.484870241
633	CCL17	4.285816107	5.580652828	1.944658028	2.869725225	0.004108155	0.745814817
634	KDSR	44.45121112	-1.200901942	0.452278754	-2.869249148	0.004112175	0.478414242
635	AC020978.5	81.61666526	0.940077615	0.227770261	2.868098201	0.004129472	0.478414242
636	AC117502.2	0.996222224	0.022664057	2.062891868	0.007722529	-2.012929571	0.49521421

637	PTGER4	42.28666527	1.214876106	0.458926584	2.865112092	0.004168617	0.727248849
638	HSPA8	2259.551244	0.247822221	0.086521849	2.864286125	0.004179502	0.484870241
639	RAD21	170.6529095	0.65982972	0.220470107	2.862972161	0.004196861	0.484870241
640	AC072246.2	0.489705689	0.05402189	4.196172952	0.012876469	0.792881255	0.489705489
641	AC010504.1	0.976576021	-1.520755908	2.970112194	-0.515286561	1.175692467	0.500997014
642	TNS1	52.92119277	-1.166449772	0.409826859	-2.846121929	0.004425285	0.990701702
643	TRPC4AP	91.61525528	-0.899825248	0.216227412	-2.844542929	0.004447519	1.125520514
644	TMSB10	1219.528542	-0.202999	0.106882065	-2.844220458	0.004452022	0.992487988
645	AC082805.2	0.478414262	-2.271055522	4.479204761	-0.529247421	-0.692802048	0.724522501
646	WIPI1	66.2485591	-1.046160147	0.26809182	-2.842117272	0.0044815	0.498141447
647	HOPX	7.550297946	4.904825272	1.726141418	2.841496775	0.004490221	0.484870241
648	SLC2A2	491.8725629	0.426979961	0.150275908	2.829417227	0.0045196	1.117074528
649	BMPR1A	29.11558801	-1.286090429	0.488541508	-2.827200946	0.004551096	0.727248849
650	MT-ND4L	2552.292818	0.285560267	0.100662688	2.826775222	0.004557167	0.49521421
651	ANKRD44-AS1	0.489705689	0.05402189	4.196172952	0.012876469	1.696672405	1.117074528
652	AC078845.1	0.478414262	-2.271055522	4.479204761	-0.529247421	0.965198829	0.498141447
653	AC079174.2	0.489705689	0.05402189	4.196172952	0.012876469	0.84921212	0.498141447
654	AL050244.1	0.486870242	-2.292502217	4.077744474	-0.586967215	-0.965966929	0.748440174
655	PRKCB	121.526684	-0.761522666	0.269065812	-2.820250566	0.004651156	1.718071551
656	HK2	127.2950661	0.80180282	0.282282616	2.820402521	0.004648948	0.478414242
657	CD1B	4.294018552	5.582077401	1.975224218	2.826296195	0.0047075	1.48884427
658	FPR2	22.49227624	1.761826972	0.622274929	2.826271782	0.004709229	0.972740484
659	AC125427.1	0.49522622	-2.415608216	4.416671772	-0.546929552	-0.905191666	0.484870241
660	AC007950.2	0.49522622	-2.415608216	4.416671772	-0.546929552	-1.191019152	0.489705489
661	AC098818.2	0.486870242	-2.292502217	4.077744474	-0.586967215	2.222509212	0.745814817
662	AC022024.1	0.498161667	0.02169065	4.170279829	0.007599166	-	

						0.662195271	0.748440174
663	AC020762.4	0.486870242	-2.292502217	4.077744474	-0.586967215	-0.62124515	0.498141447
664	AC096642.1	0.486870242	-2.292502217	4.077744474	-0.586967215	-1.618550912	0.987847254
665	CCR4	16.40709229	-2.146846899	0.762640226	-2.815019191	0.004877422	0.500997014
666	EMC2	156.6247285	0.68082284	0.241895772	2.814522854	0.004884802	0.484870241
667	ARHGAP44	0.489705689	0.05402189	4.196172952	0.012876469	1.284015428	0.49521421
668	AC026412.2	0.498161667	0.02169065	4.170279829	0.007599166	-1.050282813	0.489705489
669	LRMP	90.9952572	-0.882714152	0.215220556	-2.800206442	0.005105411	0.498141447
670	PNPLA6	107.0754755	0.840188025	0.200124771	2.799462448	0.005118777	0.500997014
671	TMEM226	11.29840652	2.751645259	0.982680922	2.797294522	0.005152252	0.987847254
672	AC018880.1	0.49522622	-2.415608216	4.416671772	-0.546929552	-0.742220994	0.500997014
673	AC007599.1	0.726077524	-2.971191046	2.466857824	-0.85702708	-1.128084244	0.724522501
674	BNC2	84.42299855	-0.954220877	0.241800551	-2.792040198	0.005227685	0.717411545
675	TNIP1	68.89187525	1.01222576	0.262621712	2.791712028	0.005242982	0.484870241
676	TPM1	52.59261056	-1.28062804	0.458850275	-2.790949706	0.005255264	0.484870241
677	AL292046.1	0.486870242	-2.292502217	4.077744474	-0.586967215	2.227766711	0.500997014
678	AC010520.1	0.72891287	-0.92219097	2.55162759	-0.26246867	-1.15056642	0.49521421
679	CAMK1	55.81652121	1.097192948	0.292496829	2.788214519	0.005298208	0.717411545
680	AP002469.1	0.49522622	-2.415608216	4.416671772	-0.546929552	-2.265112021	0.484870241
681	AL254877.1	0.478414262	-2.271055522	4.479204761	-0.529247421	0.792101592	0.748440174
682	AC096554.1	0.489705689	0.05402189	4.196172952	0.012876469	-1.428227423	0.500997014
683	AC128256.1	1.699818208	-4.197750929	2.521014728	-1.665102691	2.26922877	0.484870241
684	AC008527.2	0.498161667	0.02169065	4.170279829	0.007599166	-1.111788257	0.714077514
685	AC012464.2	0.745824827	-0.972210916	2.522016256	-0.276242662	-0.768126221	0.714077514
686	AL122517.1	0.478414262	-2.271055522	4.479204761	-0.529247421	1.255874225	0.500997014
687	SLAMF8	112.6616026	0.778619722	0.279950471	2.781276726	0.005414556	0.489705489

688	CDK4	221.1827956	-0.494829087	0.178000202	-2.77992297	0.005426995	0.484870241
689	CTSS	288.0804717	0.482099484	0.172774761	2.780022505	0.005425246	1.001994018
690	NCR2	21.9622461	1.816612289	0.654179512	2.77692241	0.005487459	0.49521421
691	VAT1	615.196775	-0.289118227	0.140152652	-2.776288614	0.005496647	0.478414242
692	PLXNB2	29.78127082	1.616898152	0.582567746	2.775468028	0.005512222	0.714077514
693	TRIM22	95.96214697	-0.849552482	0.206441192	-2.772221419	0.005565805	0.727248849
694	AC027801.1	0.999158681	1.615997221	2.159709049	0.51142865	-2.919606264	0.49521421
695	AC098818.2	0.489705689	0.05402189	4.196172952	0.012876469	-0.960951111	0.990451441
696	SUCNR1	82.58452178	0.910550126	0.229412828	2.764161122	0.005706925	0.714077514
697	S100A10	152.1121902	-0.671478876	0.242995249	-2.762241577	0.005721286	0.498141447
698	PRXL2A	78.01217824	-0.922194829	0.222969404	-2.761215245	0.005756906	0.751495511
699	STIM1	92.65902214	-0.855227254	0.209864815	-2.760001176	0.005780115	0.49521421
700	MS4A6A	79.96459622	0.92828859	0.22622629	2.760082522	0.005778676	1.121495149
701	AC011495.1	1.722400858	-1.281819192	2.265210278	-0.565872029	-0.688071575	0.724522501
702	ADRB2	0.49522622	-2.415608216	4.416671772	-0.546929552	-2.222584841	0.498141447
703	AC242960.1	0.500997014	2.484998858	4.296126822	0.565270056	1.642508114	0.741989481
704	MINPP1	165.5892284	-0.64884848	0.225524682	-2.754789552	0.00587299	0.741989481
705	NOP2	126.2529268	0.721959402	0.265748289	2.754222416	0.0058812	0.745814817
706	B2M	5242.268075	0.245821985	0.089274282	2.752670812	0.005892101	0.49521421
707	PDE2A	21.00049644	1.510242616	0.548606794	2.75286896	0.005907554	1.722491182
708	AP001272.1	0.740204196	1.049207872	2.521140276	0.297158266	-1.782127288	0.489705489
709	ARHGAP15	221.4188828	-0.496220199	0.180289706	-2.751422042	0.005922515	0.478414242
710	ALOX5AP	1120.269412	-0.202451204	0.110666128	-2.742042211	0.006105821	0.500997014
711	FCGR1A	15.71747272	-2.221024175	0.8112442	-2.727462219	0.006191521	0.49521421
712	ARL4A	185.2882691	0.615004194	0.22488519	2.724747418	0.006242816	0.49521421
713	AL117226.2	1.691262229	-4.19147216	2.541922971	-1.648926871	-	

						1.556999276	0.489705489
714	AC090587.1	0.751495521	2.069950829	2.726554747	0.822804022	-1.106728294	0.49521421
715	AC091290.5	0.49522622	-2.415608216	4.416671772	-0.546929552	-1.741742547	0.489705489
716	AP2S1	229.8148405	-0.551559774	0.201857909	-2.722415971	0.006287171	1.499818108
717	AC068860.1	0.500997014	2.484998858	4.296126822	0.565270056	-2.062852522	0.49521421
718	AC242829.4	118.9181762	0.786410508	0.288145675	2.729211566	0.006248596	0.748440174
719	AC005884.2	0.740204196	1.049207872	2.521140276	0.297158266	-0.818664242	0.478414242
720	AL127845.2	0.478414262	-2.271055522	4.479204761	-0.529247421	0.956257013	0.500997014
721	MAOB	120.2282651	-0.858704712	0.215052104	-2.725587212	0.006418722	0.500997014
722	PEBP1	515.9129764	-0.406275226	0.149020469	-2.72612198	0.006408221	0.49521421
723	AC015911.8	0.489705689	0.05402189	4.196172952	0.012876469	-0.582508412	1.121495149
724	AL257500.2	0.478414262	-2.271055522	4.479204761	-0.529247421	1.45971224	0.489705489
725	AC087229.1	0.717621545	-2.955950852	2.792242589	-0.779267242	1.278271527	0.727248849
726	SNHG28	52.12652812	1.121868152	0.415667526	2.722012182	0.006468948	0.741989481
727	RPL2	2141.948802	0.278570721	0.102291161	2.722211745	0.006462104	1.741148141
728	AC092849.1	0.724522502	-2.98570617	2.452708589	-0.86474217	-1.686066251	0.498141447
729	AC008752.4	0.49522622	-2.415608216	4.416671772	-0.546929552	-1.811442785	0.49521421
730	AC025580.1	14.0174242	2.250674028	0.828269279	2.716992597	0.006587788	0.500997014
731	AC087481.2	0.717621545	-2.955950852	2.792242589	-0.779267242	0.894956859	0.71891187
732	RUSC2	45.21299677	-1.218808226	0.448958672	-2.71474506	0.006622681	0.478414242
733	AC004877.1	1.922404758	-2.744212027	2.409072049	-1.129157211	1.780580745	0.49521421
734	AL157817.1	0.740204196	1.049207872	2.521140276	0.297158266	0.922071227	1.117074528
735	ADGRG6	0.498161667	0.02169065	4.170279829	0.007599166	0.701868099	0.748440174
736	ARHGEF40	46.24576284	-1.222167684	0.45176207	-2.707548424	0.006778218	0.49521421
737	SLC22A8	22.78548889	1.786121877	0.659920074	2.706588188	0.006797852	0.478414242
738	C1QB	66.04179228	1.024527552	0.28220041	2.70676725	0.006794187	0.974574021

739	AC107202.1	0.500997014	2.484998858	4.296126822	0.565270056	0.852948159	0.49521421
740	DOCK8	122.5829652	0.709297429	0.262250959	2.704651422	0.00682761	0.484870241
741	AC084824.2	0.498161667	0.02169065	4.170279829	0.007599166	0.85728247	0.498141447
742	AL254812.1	0.72891287	-0.92219097	2.55162759	-0.26246867	-0.77762086	1.128215801
743	AC006206.2	1.224229191	-1.952471227	2.727792282	-0.71576976	-2.422819727	0.748440174
744	INPP5F	10.28221442	-2.828056261	1.051542901	-2.698944816	0.006955971	0.484870241
745	SERPINA1	87.72729164	0.90696659	0.226220952	2.697521442	0.006985569	1.119909884
746	AC124282.2	1.222695169	-1.962816786	2.747589728	-0.714277681	-2.549029255	0.49521421
747	AC069266.1	0.478414262	-2.271055522	4.479204761	-0.529247421	0.785182689	0.745814817
748	ADAMDEC1	85.85029285	-0.879998228	0.226260059	-2.696402957	0.007009282	0.478414242
749	AC008267.5	1.221402844	-2.71978876	2.822810151	-1.212645718	-0.681269227	1.454990294
750	OR8G5	8.558222949	-2.27145112	1.25124722	-2.694472401	0.007050016	0.498141447
751	AC022762.2	0.976576021	-1.520755908	2.970112194	-0.515286561	-0.684790907	0.489705489
752	CADPS	14.02682688	2.249212457	0.825228792	2.69260726	0.007089571	1.971899245
753	ADAM20P1	0.486870242	-2.292502217	4.077744474	-0.586967215	-0.679410558	0.748440174
754	AC009145.4	0.751495521	2.069950829	2.726554747	0.822804022	0.522764226	0.985021009
755	AC025175.1	0.724522502	-2.98570617	2.452708589	-0.86474217	-0.702462021	0.489705489
756	LAMA2	18.90899066	-1.882940902	0.700971927	-2.687612482	0.007196486	0.489705489
757	ADAMTS7	40.20206567	-1.267285185	0.471492527	-2.68802815	0.007187524	0.71891187
758	MTSS1	120.1728252	-0.721586924	0.268459456	-2.68788049	0.007190712	0.498141447
759	ITGB2	1024.711242	0.207629058	0.114414711	2.688719428	0.007172668	0.741989481
760	AC015802.5	0.478414262	-2.271055522	4.479204761	-0.529247421	0.59687555	0.484870241
761	TM4SF19-AS1	2.784819092	5.401149226	2.011226252	2.685266908	0.007245022	0.478414242
762	AC011477.1	1.462446272	-2.270129994	2.569759491	-0.882401726	-0.629717497	0.748440174
763	ELL2	59.02479886	1.021482212	0.281529279	2.677268279	0.007422515	0.724522501
764	UHRF1BP1L	59.76996429	1.022424175	0.282050781	2.67876478		

0.007289428 0.498141447
765 VSIG4 150.0219858 -0.658220289 0.245854801 -2.677679615
0.007412409 0.489705489
766 CYLD 128.2521641 0.74612412 0.278668264 2.677462592
0.007418214 0.484870241
767 AC080012.4 0.498161667 0.02169065 4.170279829 0.007599166
0.992209401 1.744982508
768 AC018904.1 0.498161667 0.02169065 4.170279829 0.007599166 -
1.521629122 0.484870241
769 SLITRK4 20.62287212 -1.825685475 0.682277566 -2.675869127
0.007452574 0.489705489
770 BTN2A1 42.82281886 -1.221277158 0.460229529 -2.675254526
0.007465026 0.478414242
771 CLSTN2 60.27956962 1.012527628 0.278887821 2.675007146
0.007472766 0.489705489
772 AC079414.2 1.227074528 -0.524749642 2.612295646 -0.204704871
1.057748127 0.500997014
773 PLEC 29.62122176 1.207082062 0.489407018 2.670748509
0.007568222 0.724522501
774 SOAT1 105.9250877 0.767579402 0.287470029 2.670119754 0.00758242
0.484870241
775 RAB27 272.5292817 0.427652771 0.162887187 2.670451412
0.007574922 0.498141447
776 TAX1BP1 215.1988288 0.466152289 0.174646851 2.669114201
0.007605159 0.484870241
777 CST7 160.2051298 0.642294721 0.241262272 2.665679514 0.00768229
0.489705489
778 CCR1 149.9800154 0.661956005 0.248272051 2.66517912
0.007694722 1.705488901
779 CD162 218.8076777 -0.572258055 0.215099482 -2.665082284
0.007696924 0.500997014
780 NCOA2 258.6490722 0.521166297 0.19562544 2.66296721
0.007722509 0.974574021
781 AC080012.5 0.498161667 0.02169065 4.170279829 0.007599166
0.608295185 0.745814817
782 AL721562.2 0.49522622 -2.415608216 4.416671772 -0.546929552 -
2.640842623 1.98419049
783 AC126696.2 0.486870242 -2.292502217 4.077744474 -0.586967215 -
1.108764052 0.717411545
784 AC040160.2 1.228265862 0.628005757 2.620292458 0.228758818 -
2.469692943 0.489705489
785 CH25H 6.524479642 4.695120552 1.766804196 2.657414196
0.007874262 0.500997014
786 AC020742.2 0.486870242 -2.292502217 4.077744474 -0.586967215 -
0.606809921 0.500997014
787 AC007066.2 0.748660174 1.029647608 2.51687248 0.292772655 -
0.628999269 0.727248849
788 SLC20A2 27.25925518 -1.591757287 0.600196409 -2.652060821
0.008000212 0.49521421
789 CAVIN2 466.2264256 -0.297169816 0.149928488 -2.648885022 0.00807578
0.745814817

790 TRAF1 62.12122047 1.044725126 0.294762701 2.646462645
0.008122825 0.489705489
791 AP002972.1 0.748660174 1.029647608 2.51687248 0.292772655
0.599969287 0.500997014
792 ORM1 9.025404851 -2.994872677 1.122265942 -2.644792256
0.008174108 0.990451441
793 IL18RAP 22.16742661 1.27446142 0.520029762 2.642992929
0.008217675 0.974574021
794 AC087741.2 0.748660174 1.029647608 2.51687248 0.292772655 -
2.228252669 1.111947844
795 AC062947.2 0.987867256 0.042250627 2.875240277 0.015077222
1.289026781 0.500997014
796 AC092295.2 0.500997014 2.484998858 4.296126822 0.565270056 -
2.422825703 0.478414242
797 SAMHD1 141.7157282 0.677220607 0.256922499 2.626181127
0.008284496 0.489705489
798 RPN2 927.9049882 0.208096446 0.116871261 2.626201405
0.008282995 0.49521421
799 DLEC1 59.04282724 -1.119552141 0.424841597 -2.625222515
0.008408214 0.741989481
800 SPECC1 121.0545486 -0.715101899 0.271622822 -2.622692128
0.008471109 0.49521421
801 AL129156.2 0.498161667 0.02169065 4.170279829 0.007599166 -
0.714086589 0.49521421
802 AC022824.6 0.500997014 2.484998858 4.296126822 0.565270056 -
1.260560227 0.500997014
803 AL129807.1 1.950216715 -2.756547805 2.245621021 -1.175182892 -
0.625245124 1.194025909
804 AC092502.1 0.742989481 -2.000642057 2.742921251 -0.801470644 -
1.142484726 0.489705489
805 AC016596.2 1.725226205 -0.271567727 2.225226245 -0.166221971
0.545267209 0.478414242
806 SLC4A10 6.605180888 -4.656928462 1.772869205 -2.626774827
0.008619825 0.727248849
807 RNF169 65.86286471 -0.979452892 0.272028065 -2.62561112
0.008649257 0.500997014
808 BIRC6 155.6246816 0.648074585 0.24695015 2.624212291
0.008682286 1.944292215
809 HEATR1 141.2627276 -0.677012475 0.258056521 -2.622504515
0.008702029 0.478414242
810 AC008524.1 1.725186142 -4.216510018 2.575412257 -1.627216801 -
1.225249786 0.484870241
811 AC006504.4 0.498161667 0.02169065 4.170279829 0.007599166
1.297152476 0.745814817
812 AL021722.1 0.478414262 -2.271055522 4.479204761 -0.529247421 -
1.724850228 0.987847254
813 CCL2 116.4942154 0.722851405 0.280107871 2.619888551
0.008795851 0.751495511
814 AC027682.4 0.498161667 0.02169065 4.170279829 0.007599166 -
0.802592057 1.719545511
815 AC007016.1 0.979411277 0.062718118 2.082209142 0.020241171 -

0.618824961 0.484870241
816 PLOD2 102.9092572 0.761228127 0.290755726 2.618101875 0.00884204
0.489705489
817 AC022025.1 0.500997014 2.484998858 4.296126822 0.565270056 -
0.677757244 0.717411545
818 TMEM9 27.008452 -1.501696427 0.572892864 -2.616684264
0.008878842 0.500997014
819 AC224782.1 0.726077524 -2.971191046 2.466857824 -0.85702708
0.772225111 1.708174184
820 AC091544.4 1.699818208 -4.197750929 2.521014728 -1.665102691 -
0.62262274 0.498141447
821 EXOSC8 165.8275827 -0.614888862 0.225225942 -2.614025072
0.008947985 0.484870241
822 UQCRC1 472.9665172 0.292268658 0.150120595 2.612022597
0.008974511 0.49521421
823 AC007242.1 1.471902251 -2.278459292 2.566048502 -0.887925264 -
2.166227448 1.128215801
824 AC006026.2 0.745824827 -0.972210916 2.522016256 -0.276242662
0.781694261 0.741989481
825 AC090948.1 1.722692182 -0.281477417 2.258626499 -0.168897964
0.584800278 0.748440174
826 AL025252.1 0.992487988 -1.558611578 2.168280519 -0.491926891
1.118254651 0.714077514
827 MMP7 2.528852288 5.200216725 2.025550247 2.602874049
0.009217662 0.49521421
828 PFKFB2 42.21910147 -1.194141925 0.459072041 -2.601208148
0.009289607 0.740104194
829 RIMKLB 45.88289682 -1.242544252 0.478022217 -2.601275902
0.009285065 0.49521421
830 DENND2D 124.4420757 -0.682202745 0.262122884 -2.602497278
0.009254752 0.478414242
831 KLF6 292.2122642 -0.508019601 0.195262755 -2.60170968
0.009276024 0.71891187
832 KCNIP1-OT1 12.97685752 2.515922775 0.968068146 2.598910816
0.009252007 0.717411545
833 HBP1 125.8154888 -0.691242545 0.266001682 -2.599015678 0.00924915
0.741989481
834 CHCHD7 74.02759566 0.928990804 0.261286227 2.599021868
0.009248982 0.751495511
835 AC068657.2 0.498161667 0.02169065 4.170279829 0.007599166 -
2.214820559 0.500997014
836 ALDOA 1964.285941 0.246527465 0.095087959 2.592625468
0.009524642 1.144811841
837 PLAAT4 56.58907882 -1.027257899 0.296582161 -2.590529782
0.009582822 0.71891187
838 PLEKHG2 50.27294051 -1.150078082 0.444112077 -2.589606426
0.009608571 0.478414242
839 HBD 20.68216184 1.278682168 0.52229958 2.590049226
0.009596218 1.118418559
840 RABEPK 20.89684875 1.299759256 0.541175071 2.586518542
0.009695095 0.714077514

841	ZC4H2	62.7627126	0.95605842	0.269762089	2.585597259	0.009721044	0.498141447
842	SLC29A10	52.28447787	1.050960452	0.406614655	2.584659549	0.009747519	0.500997014
843	MTREX	159.2457768	0.620604472	0.240222214	2.582252421	0.009784522	0.740104194
844	NCBP2	228.5447822	0.512894224	0.198958288	2.582922129	0.009796715	1.121495149
845	SPATA12	7.824989574	-2.228466484	1.250254996	-2.582246418	0.009815947	1.001994018
846	ZNF266	2.524220586	5.202266004	2.054200702	2.581182062	0.009846264	1.119859812
847	PKM	2442.20045	0.201549422	0.116809869	2.581540622	0.009826041	0.49521421
848	CSE1L	116.5860147	0.706922941	0.274022915	2.579727914	0.009887819	0.724522501
849	SLC44A2	122.405902	-0.662771279	0.257479158	-2.577961591	0.009928504	0.500997014
850	AC112252.1	0.982196662	-2.404284457	2.098256189	-1.098806625	-1.257575664	0.724522501
851	AC092952.2	1.972899265	-0.697222499	2.11241011	-0.220107527	-2.074022442	0.500997014
852	SEMA6A	27.65048575	1.241215227	0.482125201	2.574620625	0.010025009	0.478414242
853	LILRB4	22.29047688	1.475802081	0.572295516	2.574244941	0.010045912	0.49521421
854	PCTP	117.5200471	-0.710261472	0.275799997	-2.575277202	0.010015975	0.484870241
855	MCCC2	69.78570259	0.9400066	0.265224564	2.572777049	0.010059507	1.984140418
856	PIK2C2B	127.7699086	-0.704652222	0.27299605	-2.571760916	0.010118274	0.71891187
857	DNAJC19	70.79492867	-0.922496917	0.259246142	-2.569926911	0.010171704	0.500997014
858	GTF2A2	152.7964225	0.620117574	0.241262425	2.569228145	0.010192229	0.500997014
859	KIRREL2	6.795812546	2.695654067	1.429420021	2.567460512	0.010244646	0.717411545
860	AC012618.2	1.482192676	-0.966185564	2.429267947	-0.297727046	0.650205472	0.49521421
861	AC078776.1	0.500997014	2.484998858	4.296126822	0.565270056	0.781952213	0.981194441
862	AL121924.1	0.500997014	2.484998858	4.296126822	0.565270056	1.247069757	0.478414242
863	C15orf48	29.5842952	1.219505527	0.475871119	2.562680268	0.010286765	0.484870241
864	PRDM2	54.79280642	1.027626752	0.401079811	2.562175221	0.010401882	0.745814817
865	RAB20	122.078421	-0.687866508	0.268924788	-2.557744619	0.010525242	0.500997014
866	TLE2	117.1420808	-0.716904826	0.280411256	-2.556618416		

0.010569509 0.500997014
867 AC000025.1 0.745824827 -0.972210916 2.522016256 -0.276242662 -
0.629926268 0.745814817
868 P2RY12 29.85757992 1.186958622 0.465428908 2.550246886
0.010764666 0.500997014
869 ITSN2 92.52616721 -0.825167721 0.222661271 -2.549478542
0.010788415 1.494485001
870 CEP70 27.98462217 -1.424128294 0.562994816 -2.547228264
0.010854812 1.115782111
871 FAT1 95.86026026 -0.780169161 0.206274522 -2.546455648
0.010882204 1.474727498
872 ICAM2 192.292685 0.604579295 0.227462714 2.545997159
0.010896608 0.741989481
873 PRKACB 164.7840682 -0.591464027 0.222600544 -2.542821698
0.010995818 0.745814817
874 CALM1 980.5529209 0.294465269 0.115870152 2.541229261
0.011042871 0.500997014
875 SLC25F6 94.62141226 -0.768456261 0.202582575 -2.529649622
0.011096257 0.751495511
876 AC000122.2 0.976576021 -1.520755908 2.970112194 -0.515286561
0.618569766 0.714077514
877 CDH12 2.408092294 -5.200872829 2.049681502 -2.527405259
0.011167756 1.947118471
878 NFXL1 44.58788626 1.110760229 0.427949979 2.52627191 0.01120297
0.994212224
879 PITPNB 174.727269 0.584020525 0.220425964 2.524569125
0.011258571 0.498141447
880 AC004000.1 0.724522502 -2.98570617 2.452708589 -0.86474217
0.620072281 0.999158481
881 BBS4 112.5224815 -0.717771829 0.282450946 -2.522261222
0.011222952 0.478414242
882 AC114489.1 0.486870242 -2.292502217 4.077744474 -0.586967215 -
1.641702264 1.440411014
883 AC104794.2 1.708274186 -4.202977456 2.517745205 -1.669728972 -
2.22674244 0.745814817
884 AC007608.2 20.45504259 1.260722268 0.527864558 2.529879025
0.011410186 0.478414242
885 PZP 54.26740097 -1.057047965 0.417982788 -2.52892097
0.011441279 0.478414242
886 CST2 142.42865 -0.707922522 0.280057968 -2.527810716
0.011477621 1.497210248
887 MFSD1 421.0201007 0.402190455 0.159282687 2.525010489
0.011569484 0.478414242
888 PRPSAP2 126.4672275 -0.648202025 0.256772004 -2.524426294
0.011588727 0.478414242
889 STXBP5-AS1 19.8925156 -1.861602257 0.727889762 -2.522874488
0.011629994 1.121495149
890 AC010642.1 0.489705689 0.05402189 4.196172952 0.012876469 -
0.922885145 0.972740484
891 AC079229.1 1.225520516 -0.54785862 2.606584125 -0.2101826 -
2.080580657 0.49521421

892	C1QC	88.77924866	0.784228929	0.211268682	2.519812181	0.011741712	0.498141447
893	AC107204.1	0.742989481	-2.000642057	2.742921251	-0.801470644	0.540406545	0.49521421
894	DNAH1	26.44450974	-1.241444127	0.492210609	-2.516556718	0.011850782	1.144811841
895	AC107992.1	0.72891287	-0.92219097	2.55162759	-0.26246867	0.95559167	0.498141447
896	AC092747.4	1.229909884	0.646554266	2.808966554	0.220175128	-0.914615523	0.49521421
897	UQCRH	280.0502869	0.46585282	0.185296591	2.514092828	0.011922902	0.748440174
898	MT-ND1	2857.224267	0.249852761	0.099450527	2.512242249	0.011992271	0.484870241
899	AC074267.1	0.996222224	0.022664057	2.062891868	0.007722529	0.607294467	0.498141447
900	SH2KBP1	157.1822269	-0.600922949	0.229465928	-2.50947161	0.012091192	0.740104194
901	AL022581.1	0.500997014	2.484998858	4.296126822	0.565270056	1.227212104	0.49521421
902	AC107275.1	1.246821841	0.609177462	2.794582528	0.217985129	0.586099457	0.500997014
903	AC245884.4	0.500997014	2.484998858	4.296126822	0.565270056	-1.185515276	0.71891187
904	AL129029.2	0.478414262	-2.271055522	4.479204761	-0.529247421	-0.702254176	1.117074528
905	RNU6-29P	2.275619622	5.192954115	2.072972594	2.505074899	0.012242547	0.478414242
906	PARP9	142.9214604	-0.624628422	0.252202252	-2.505409685	0.012220962	0.484870241
907	WBP1L	146.8179852	-0.617258867	0.246475242	-2.504750019	0.012252797	1.952151041
908	PIGT	267.2244026	0.418989411	0.167225059	2.502894956	0.01228245	1.98419049
909	AC091564.5	0.478414262	-2.271055522	4.479204761	-0.529247421	-0.669552114	0.49521421
910	AC046158.4	1.711109522	-2.527241629	2.440212294	-1.029762666	-0.985792299	0.745814817
911	NR5A2	46.22278728	-1.078072772	0.42152146	-2.4982507	0.012480789	0.498141447
912	MS4A7	52.98022742	1.020660858	0.408546479	2.498272526	0.012479985	0.498141447
913	EIF4A2	221.9017704	0.607504789	0.242024087	2.499669062	0.012420927	0.500997014
914	GRN	1577.419842	-0.249727877	0.099924221	-2.498919794	0.01245725	0.478414242
915	VDAC1	426.5270118	0.284859984	0.154162424	2.496441599	0.012544622	0.740104194
916	KCTD20	127.55211	-0.720496212	0.288846819	-2.494288951	0.01261742	0.987847254
917	TRAK2	48.76497686	-1.051151269	0.421597266	-2.492258858		

0.012657652 0.500997014
918 AC242964.4 0.489705689 0.05402189 4.196172952 0.012876469
0.61792859 0.740104194
919 AC008870.4 1.215782212 -1.942095285 2.757929924 -0.704182251 -
2.922587621 0.727248849
920 AC006557.1 0.49522622 -2.415608216 4.416671772 -0.546929552
0.745952843 0.992487988
921 AL582856.2 0.489705689 0.05402189 4.196172952 0.012876469 -
2.426171409 0.498141447
922 NIPSNAP2A 142.5620608 -0.616662742 0.247927749 -2.487271976
0.012872695 0.478414242
923 AC011899.2 0.972740684 -2.29250678 2.080064802 -1.101764725
0.570470198 0.972740484
924 AC010521.5 0.72891287 -0.92219097 2.55162759 -0.26246867
0.570228222 0.478414242
925 OSBPL9 229.6528514 -0.511241552 0.205727822 -2.48552455
0.012926066 1.705488901
926 AL591719.1 1.719615572 0.477264708 2.441096672 0.195512416
2.120582599 0.981194441
927 AC092201.1 0.72891287 -0.92219097 2.55162759 -0.26246867
2.121211808 0.500997014
928 AL021055.1 0.486870242 -2.292502217 4.077744474 -0.586967215 -
2.927146785 0.745814817
929 TUFM 297.5776014 0.448541722 0.180650108 2.482920885
0.012020627 1.499818108
930 AL129286.1 0.478414262 -2.271055522 4.479204761 -0.529247421 -
0.642402623 0.999158481
931 AC022972.2 0.486870242 -2.292502217 4.077744474 -0.586967215 -
0.472452883 0.484870241
932 AC026780.2 0.489705689 0.05402189 4.196172952 0.012876469 -
2.92974692 0.714077514
933 AMBP 0.72891287 -0.92219097 2.55162759 -0.26246867 -0.650962
0.478414242
934 AP000422.2 0.498161667 0.02169065 4.170279829 0.007599166
2.221250428 0.478414242
935 TPCN1 120.8001995 -0.670966454 0.270561497 -2.479902689
0.012141787 0.714077514
936 RRN2 144.8779775 0.65964868 0.266045592 2.479457282
0.012158249 0.478414242
937 AC020982.1 0.500997014 2.484998858 4.296126822 0.565270056
0.488210928 0.484870241
938 AC120454.1 0.486870242 -2.292502217 4.077744474 -0.586967215 -
2.106222747 0.724522501
939 AC107294.2 0.751495521 2.069950829 2.726554747 0.822804022 -
0.622542788 0.500997014
940 FPR2 58.94976022 0.967628506 0.29092277 2.475267702
0.012212624 0.741989481
941 LCP2 221.9915702 0.444115169 0.179529149 2.472629718 0.01227445
0.478414242
942 AC011277.1 0.49522622 -2.415608216 4.416671772 -0.546929552 -
1.507764747 1.114129191

943	AC007681.1	0.956828727	-2.270922824	2.294506224	-0.992055722	-0.814842462	0.985021009
944	PMM1	45.68607997	-1.089467271	0.440728892	-2.471967215	0.012427184	1.449047004
945	AC129491.2	0.478414262	-2.271055522	4.479204761	-0.529247421	0.752758551	0.741989481
946	TRIM22	129.2221701	0.658825029	0.266742042	2.469922986	0.012514177	0.714077514
947	CD47	259.4985096	0.479176692	0.194024222	2.4695472	0.012528412	1.121495149
948	LINC02728	5.792920716	4.514260901	1.828405429	2.469015257	0.012548544	0.478414242
949	AP001172.1	0.500997014	2.484998858	4.296126822	0.565270056	0.465897056	0.498141447
950	AC007402.1	0.72891287	-0.92219097	2.55162759	-0.26246867	-0.759516106	0.992487988
951	AC022512.1	0.478414262	-2.271055522	4.479204761	-0.529247421	-0.595428266	0.727248849
952	AL126146.1	0.486870242	-2.292502217	4.077744474	-0.586967215	1.168826066	0.49521421
953	AL024474.2	1.224229191	-1.952471227	2.727792282	-0.71576976	1.168805228	0.985021009
954	RAP2B	55.25845841	-0.977864124	0.296421759	-2.466664465	0.012627808	0.972740484
955	MGAT1	417.8082948	0.291161601	0.15876202	2.462822529	0.012746277	0.500997014
956	CCDC88A	111.511287	-0.695886252	0.282607262	-2.462279575	0.012801851	0.489705489
957	CAPN2	111.2044219	-0.689709192	0.28029215	-2.460670882	0.012867751	1.477511982
958	MCTP1	102.8622111	-0.775221857	0.21510868	-2.460204706	0.012885779	0.724522501
959	FKBP5	65.75201786	0.902082195	0.26712994	2.459842495	0.012899761	0.500997014
960	AL290728.2	1.491649655	-0.977257449	2.612941998	-0.27400656	-2.062821423	0.49521421
961	AC104762.1	0.717621545	-2.955950852	2.792242589	-0.779267242	2.742297299	0.985021009
962	AL256225.1	0.72891287	-0.92219097	2.55162759	-0.26246867	2.057240656	0.727248849
963	LRRC59	64.28225815	0.911618247	0.271557812	2.452502106	0.014147222	0.489705489
964	TBCB	225.2802221	-0.594277207	0.242179442	-2.454284722	0.014116516	0.489705489
965	RALB	209.4082112	-0.442078888	0.180161448	-2.45279294	0.014125825	0.49521421
966	P4HA2	62.42612129	0.912400012	0.272441082	2.452468472	0.014187981	0.49521421
967	GLB1	484.9142481	0.264828576	0.148754519	2.452554571	0.014184586	0.500997014
968	AC008727.1	1.719565511	-2.544221854	2.447985242	-1.029216621		

0.461215558	0.49521421
969	AL259715.2	0.987867256	0.042250627	2.875240277	0.015077222	-
0.862522268	0.498141447
970	AL252689.2	1.229909884	0.646554266	2.808966554	0.220175128
0.485028948	0.714077514
971	C1QBP	206.4470242	0.526899202	0.21492856	2.451294955
0.014220272	0.500997014
972	CAST	129.80868	-0.6446082	0.262060566	-2.450417828
0.014269052	0.727248849
973	SAMSN1	1162.108974	0.282502292	0.115684592	2.450658169	0.01425952
0.498141447
974	AC099494.2	0.489705689	0.05402189	4.196172952	0.012876469
2.249972081	0.484870241
975	AC122552.5	0.987867256	0.042250627	2.875240277	0.015077222	-
1.222299877	1.119909884
976	AC009690.2	272.0101811	0.457272626	0.18672844	2.449274157
0.014214445	0.484870241
977	RNF165	15.7544689	1.984657924	0.8108611	2.447592978
0.014281401	1.708214148
978	GLOD5	22.75674896	1.52285055	0.626077282	2.448240789
0.014251584	0.498141447
979	DCPS	59.99297005	0.924574982	0.277729288	2.447718544
0.014276291	1.488814208
980	APOE	22.20654087	-1.29805606	0.520457956	-2.447047962
0.014402167	1.141151148
981	VAPA	564.0289292	-0.228512772	0.128259902	-2.446617602
0.014420274	1.119859812
982	HEBP2	25.25552527	-1.229884162	0.502901018	-2.445578989
0.014461977	0.748440174
983	OLR1	28.28148421	1.41080544	0.576964419	2.445220872
0.014476246	0.484870241
984	NR2C1	95.84066201	-0.747494042	0.205985627	-2.442905721
0.014569542	0.981194441
985	DNAJB11	95.14724758	-0.765672247	0.212686868	-2.440884291
0.014651248	0.484870241
986	AC074275.1	0.478414262	-2.271055522	4.479204761	-0.529247421	-
0.669016996	0.49521421
987	SH2BGRL2	2.168885212	-5.095621674	2.089698428	-2.428452119
0.014750272	0.49521421
988	EZR	261.0299925	0.464227906	0.190228658	2.42912822
0.014722225	0.478414242
989	MPO	2096.199706	0.245211094	0.10058175	2.428922505
0.014721127	0.478414242
990	AC011124.1	0.500997014	2.484998858	4.296126822	0.565270056
0.446712462	0.945184705
991	MREG	5.525219762	4.445750551	1.825228522	2.425575921
0.014868104	0.740104194
992	SSBP2	84.62225687	-0.808722145	0.221985588	-2.426048956
0.014848675	0.727248849
993	CYP27B1	9.771061669	2.502227991	1.028295219	2.424454229
0.014914262	0.741989481

994	ASRGL1	102.7810012	-0.707226481	0.290697741	-2.42289294	0.014978729	0.489705489
995	GNAI1	106.7795094	-0.706620426	0.290410272	-2.422179896	0.014966862	0.714077514
996	GAS5	276.8297926	0.461470519	0.18964102	2.422289642	0.014958194	0.478414242
997	AL590708.1	0.727268849	-0.952727871	2.205072418	-0.288262241	1.875221201	0.498141447
998	AC055822.1	0.992487988	-1.558611578	2.168280519	-0.491926891	-2.555401252	0.500997014
999	AC055764.2	0.486870242	-2.292502217	4.077744474	-0.586967215	-2.542856277	0.985021009
1000	TRANK1	47.70657519	-1.028662722	0.428068275	-2.426294456	0.015249685	0.741989481
1001	SNX2	245.6219227	0.481248091	0.198422101	2.425744929	0.015277002	0.478414242
1002	AC010998.1	0.478414262	-2.271055522	4.479204761	-0.529247421	-0.482752953	0.974574021
1003	AC027207.2	0.726077524	-2.971191046	2.466857824	-0.85702708	-0.752701722	1.718071551
1004	NOB1	95.29019929	0.740079554	0.205252078	2.422692541	0.015262607	0.478414242
1005	ANXA1	2111.472866	-0.217229901	0.089617954	-2.422955145	0.015252502	0.49521421
1006	RBM26	58.44177281	0.95284962	0.292295259	2.422722962	0.015404245	0.999158481
1007	AL255112.1	0.500997014	2.484998858	4.296126822	0.565270056	0.464600668	1.950214715
1008	AC012645.1	0.478414262	-2.271055522	4.479204761	-0.529247421	-0.550577281	1.484019012
1009	AC122529.1	0.498161667	0.02169065	4.170279829	0.007599166	-2.102497256	0.498141447
1010	PKIB	178.0065789	-0.5670716	0.224502225	-2.418181491	0.015598295	0.748440174
1011	AL162277.1	1.711109522	-2.527241629	2.440212294	-1.029762666	-2.859720473	0.484870241
1012	AC022274.1	0.49522622	-2.415608216	4.416671772	-0.546929552	1.629114501	0.981194441
1013	AL078602.1	0.500997014	2.484998858	4.296126822	0.565270056	0.50226943	0.484870241
1014	P4HB	1002.046054	0.275242182	0.114022902	2.414797152	0.015742975	1.454990294
1015	AC008897.2	0.500997014	2.484998858	4.296126822	0.565270056	2.722258173	0.478414242
1016	EMP2	229.1194959	-0.485216918	0.201186279	-2.412276428	0.015852257	0.500997014
1017	CLDND1	152.8829558	0.626014222	0.262652251	2.412222748	0.015851242	1.115782111
1018	AD001527.1	0.486870242	-2.292502217	4.077744474	-0.586967215	0.622290593	0.484870241
1019	AC006528.1	0.748660174	1.029647608	2.51687248	0.292772655	-	

0.870987469	0.489705489
1020	AL257140.1	1.699818208	-4.197750929	2.521014728	-1.665102691	-2.876849297	0.990451441
1021	GALM	111.7857822	0.675246605	0.280075101	2.410948261	0.015911101	0.714077514
1022	RPL8	1227.29492	0.261041249	0.108258577	2.411275459	0.015896827	0.71891187
1023	AC124067.1	0.478414262	-2.271055522	4.479204761	-0.529247421	2.995875083	0.484870241
1024	ADIRF	0.478414262	-2.271055522	4.479204761	-0.529247421	2.726844963	0.71891187
1025	CCT6A	492.514852	0.252909222	0.146565782	2.407855462	0.016046522	0.500997014
1026	ADAMTS9-AS2	0.478414262	-2.271055522	4.479204761	-0.529247421	-0.821699621	0.498141447
1027	AL122520.1	0.500997014	2.484998858	4.296126822	0.565270056	0.501988491	1.114129191
1028	AL512288.2	1.720856827	-1.290122295	2.299421296	-0.561068275	2.942808792	0.484870241
1029	ADGRG1	0.726077524	-2.971191046	2.466857824	-0.85702708	2.942445123	0.500997014
1030	AMY2B	1.218618559	-0.521074109	2.817659821	-0.184921518	2.942427422	0.484870241
1031	AC091952.5	0.500997014	2.484998858	4.296126822	0.565270056	-2.252192189	0.484870241
1032	AL127009.2	0.751495521	2.069950829	2.726554747	0.822804022	-2.292016886	1.128245842
1033	AC127070.2	0.498161667	0.02169065	4.170279829	0.007599166	1.227887253	0.478414242
1034	AC082842.4	0.972740684	-2.29250678	2.080064802	-1.101764725	2.942247161	0.49521421
1035	AC245150.1	0.49522622	-2.415608216	4.416671772	-0.546929552	2.942226287	1.451155047
1036	AC010521.6	0.498161667	0.02169065	4.170279829	0.007599166	0.558266997	0.745814817
1037	TRAFD1	260.246052	-0.46027508	0.19174752	-2.400422599	0.016276154	0.741989481
1038	AL021672.1	0.486870242	-2.292502217	4.077744474	-0.586967215	1.446766053	0.71891187
1039	AC116902.2	0.742989481	-2.000642057	2.742921251	-0.801470644	-0.589962564	0.714077514
1040	AL080276.2	1.719565511	-2.544221854	2.447985242	-1.029216621	-1.244979442	0.724522501
1041	AC087465.1	0.49522622	-2.415608216	4.416671772	-0.546929552	2.926156159	0.992487988
1042	IL7	9.528512077	2.465592171	1.027928854	2.298601967	0.016457794	0.714077514
1043	AL049624.1	0.478414262	-2.271055522	4.479204761	-0.529247421	-1.244426817	0.945184705
1044	AC008280.1	0.717621545	-2.955950852	2.792242589	-0.779267242	2.19124742	0.49521421

1045	XKR2	2.025121125	5.078084724	2.119627601	2.295722521	0.01658719	0.500997014
1046	IGSF8	22.69847004	1.456714772	0.608007729	2.295881956	0.016580429	0.484870241
1047	AL122025.2	52.96224765	-1.000629628	0.417675295	-2.295725159	0.01658707	0.478414242
1048	PTPN2	92.20092	0.721201299	0.205120217	2.296427069	0.016555226	0.484870241
1049	AC007255.1	0.985022009	-1.54422206	2.962499952	-0.52129252	-0.421612802	0.71891187
1050	RNF12	155.5642542	0.588225522	0.245664489	2.294874146	0.016626071	0.751495511
1051	AC022820.2	0.498161667	0.02169065	4.170279829	0.007599166	-0.608988772	0.49521421
1052	Clorf21	54.22567821	-0.96720177	0.404076958	-2.292607828	0.016682577	0.478414242
1053	NOP56	194.7692524	0.526484224	0.224215156	2.291654416	0.016772627	0.484870241
1054	RPL5	2210.681652	0.226154164	0.094554477	2.29178695	0.016766572	0.489705489
1055	AL129156.2	1.491649655	-0.977257449	2.612941998	-0.27400656	0.698656501	0.498141447
1056	AL161747.1	0.500997014	2.484998858	4.296126822	0.565270056	-2.259601818	1.128245842
1057	AC072476.2	0.489705689	0.05402189	4.196172952	0.012876469	-2.942244122	0.740104194
1058	APBA1	0.498161667	0.02169065	4.170279829	0.007599166	-0.516165895	0.484870241
1059	NAV1	122.5296517	-0.619715889	0.259646042	-2.286771924	0.016997022	0.500997014
1060	WNT5B	6.200528262	2.566858629	1.494772588	2.286221601	0.01702249	0.49521421
1061	ADGRE4P	12.75024526	-1.984294549	0.821942274	-2.285254808	0.017067295	1.442444272
1062	AC112255.1	0.49522622	-2.415608216	4.416671772	-0.546929552	-2.942422287	0.484870241
1063	PLAAT2	45.52059718	-1.0462469	0.428774292	-2.284476299	0.01710245	0.49521421
1064	PSMA2	216.0288197	0.420189901	0.176298074	2.282406078	0.017152261	0.500997014
1065	HPGDS	2287.772891	-0.22295296	0.092964412	-2.282291289	0.01715295	1.494485001
1066	AC090425.2	0.726077524	-2.971191046	2.466857824	-0.85702708	0.485708488	0.489705489
1067	F2	2.016918679	5.074687864	2.129906272	2.282587295	0.017191455	0.745814817
1068	ITGA9	25.22944111	-1.428408241	0.602828018	-2.282148887	0.017211927	0.498141447
1069	AC025154.1	0.982196662	-2.404284457	2.098256189	-1.098806625	-0.85604021	1.719545511
1070	CD226	25.92928498	-1.164601977	0.489286077	-2.279720292		

0.017225784	0.498141447
1071	AL255001.2	0.49522622	-2.415608216	4.416671772	-0.546929552
0.628872623	0.478414242
1072	AC092825.1	0.498161667	0.02169065	4.170279829	0.007599166	-
0.486094986	0.484870241
1073	MSMO1	109.5421965	-0.680856511	0.286476742	-2.276655449
0.017470298	1.119909884
1074	AC090971.6	0.49522622	-2.415608216	4.416671772	-0.546929552
0.61072098	0.740104194
1075	AC022206.2	0.500997014	2.484998858	4.296126822	0.565270056	-
0.548299229	0.498141447
1076	DEPTOR	16.46025874	-1.87074452	0.787854228	-2.274480508
0.017572664	0.498141447
1077	ATP5F1B	1276.849964	0.26180989	0.11029768	2.272666226
0.017612458	0.974574021
1078	CRYL1	72.2875111	-0.818746182	0.245242992	-2.27150702	0.01771571
0.500997014
1079	DHCR24	202.5192945	-0.507910884	0.214205165	-2.271142092
0.017722212	0.500997014
1080	MLLT11	116.2192126	0.649192912	0.272911278	2.270088288
0.017782828	0.981194441
1081	PCED1B	12.00556415	-2.102275901	0.887221781	-2.269617097
0.017806516	0.748440174
1082	AL022284.2	0.72891287	-0.92219097	2.55162759	-0.26246867	-
0.719426609	1.491241119
1083	AC010627.1	1.48025822	-2.286862255	2.597159084	-0.880524905
0.786528848	0.500997014
1084	AC016724.1	0.982196662	-2.404284457	2.098256189	-1.098806625
0.4580906	0.484870241
1085	CCDC50	21.51676788	-1.251758622	0.52864491	-2.267862814
0.017891168	0.727248849
1086	G0S2	52.41624575	1.122721266	0.474218582	2.267518492
0.017907825	0.741989481
1087	AC004822.4	1.460611026	-2.978472426	2.65288022	-1.499115665
0.275222918	0.748440174
1088	ALMS1-IT1	0.726077524	-2.971191046	2.466857824	-0.85702708	-
1.828266272	0.500997014
1089	AC005222.4	0.489705689	0.05402189	4.196172952	0.012876469
2.894402479	0.500997014
1090	AC092124.1	1.742148162	-0.292224764	2.421224167	-0.16126259	-
2.974422207	0.748440174
1091	AC022622.2	0.489705689	0.05402189	4.196172952	0.012876469	-
2.0460072	1.451155047
1092	AL256489.1	0.486870242	-2.292502217	4.077744474	-0.586967215
0.610424749	0.489705489
1093	AC011998.2	0.500997014	2.484998858	4.296126822	0.565270056	-
0.551105488	1.991444449
1094	AC104825.1	1.228215801	-2.727726926	2.094096126	-1.208019006	-
1.51198081	0.500997014
1095	AC105265.2	0.726077524	-2.971191046	2.466857824	-0.85702708
2.092422211	0.71891187

1096 AC044840.1 0.486870242 -2.292502217 4.077744474 -0.586967215 -
1.102421152 0.992487988
1097 ELMO1 217.2671165 -0.495542262 0.210171924 -2.257799519
0.018282618 0.740104194
1098 BEX4 290.2797748 -0.425900161 0.180622226 -2.257829
0.018282158 1.107217124
1099 AL109766.1 0.976576021 -1.520755908 2.970112194 -0.515286561
0.754568547 1.117074528
1100 AC016582.2 1.725226205 -0.271567727 2.225226245 -0.166221971
0.594092503 0.717411545
1101 PLXNC1 21.24891079 -1.512228861 0.642154861 -2.256501449
0.018447996 0.972740484
1102 CD200LB 22.9861144 1.468674197 0.622188624 2.256708895
0.018427694 0.954818717
1103 AC010220.4 0.742989481 -2.000642057 2.742921251 -0.801470644 -
0.581719625 0.478414242
1104 AC124916.1 0.972740684 -2.29250678 2.080064802 -1.101764725 -
2.028147996 0.500997014
1105 AC022144.1 0.745824827 -0.972210916 2.522016256 -0.276242662 -
0.556042857 0.500997014
1106 VCL 126.8414249 -0.599768142 0.254682581 -2.254954095
0.018524994 1.477572045
1107 TIE1 78.4142145 -0.852924142 0.262881978 -2.252172188
0.018612962 1.125520514
1108 DDAH2 227.0408289 -0.558468016 0.227248522 -2.252944897
0.018625294 1.121495149
1109 AL128999.1 1.708274186 -4.202977456 2.517745205 -1.669728972
0.451971461 0.489705489
1110 PSME2 291.5094217 0.422718292 0.184619199 2.249259415
0.018810796 0.478414242
1111 BEX1 25.2622906 1.157958117 0.492002192 2.248784217
0.018824819 0.484870241
1112 AC087286.2 0.486870242 -2.292502217 4.077744474 -0.586967215 -
1.444212283 1.720854827
1113 AC020912.2 0.985022009 -1.54422206 2.962499952 -0.52129252 -
0.618088266 0.489705489
1114 PPP1CC 88.52444625 -0.722486279 0.212446904 -2.24755496
0.018897085 0.748440174
1115 AC072289.2 0.486870242 -2.292502217 4.077744474 -0.586967215
0.406822896 0.745814817
1116 AC072592.9 0.742989481 -2.000642057 2.742921251 -0.801470644 -
0.491179616 0.500997014
1117 AC000068.2 1.215782212 -1.942095285 2.757929924 -0.704182251 -
0.422484547 0.484870241
1118 AP001972.4 1.950216715 -2.756547805 2.245621021 -1.175182892 -
2.194952671 0.751495511
1119 AL122216.2 0.992487988 -1.558611578 2.168280519 -0.491926891 -
1.18112924 0.498141447
1120 AL669821.1 0.498161667 0.02169065 4.170279829 0.007599166 -
1.221492248 0.498141447
1121 CRBN 181.5908247 -0.522269128 0.222925598 -2.242690629 0.01914525

1.442444272
1122 DCTN2 292.1128624 -0.447052618 0.190894994 -2.241877119
0.019187021 0.724522501
1123 AL161620.1 0.478414262 -2.271055522 4.479204761 -0.529247421 -
0.748216552 0.745814817
1124 AL255226.2 1.729212815 -1.299222255 2.47404142 -0.525182122
2.028071294 0.478414242
1125 ITGB7 60.90488592 0.886518924 0.278820049 2.240211205
0.019272827 0.478414242
1126 MT1G 19.52842727 1.677761501 0.717125651 2.229521578
0.019207929 1.115782111
1127 SDHB 212.8652526 0.512222257 0.219064911 2.228722969
0.019249724 0.748440174
1128 AC005291.2 0.498161667 0.02169065 4.170279829 0.007599166
0.557468849 0.981194441
1129 RAB24 104.8702699 -0.711822551 0.204760252 -2.225716499
0.019506022 1.708174184
1130 AL126209.2 1.720856827 -1.290122295 2.299421296 -0.561068275 -
0.655778788 0.49521421
1131 FAM171A1 2.921222052 -4.978479422 2.125051006 -2.221784772
0.019712017 0.478414242
1132 PDGFRA 2.92967802 -4.982107706 2.126941548 -2.221419729
0.019721228 1.125520514
1133 EXOG 14.49574747 1.841942512 0.789400626 2.222244249 0.01962008
0.49521421
1134 AZU1 72.66998279 -0.948619009 0.406926052 -2.2211255
0.019746742 0.484870241
1135 ENO2 175.6886284 -0.578459224 0.247887095 -2.222559698
0.019618789 0.981194441
1136 CBLB 152.2440902 -0.562187169 0.241269288 -2.222299902
0.019622411 0.484870241
1137 BSG 222.4022612 0.429202179 0.184117784 2.22112275
0.019746208 1.491449455
1138 ATP5MC2 424.4291946 0.267702679 0.157621061 2.222822422
0.01965689 0.498141447
1139 RPL6 526.7400781 0.248228108 0.149246604 2.222229751
0.019625566 1.119859812
1140 AC006042.2 0.478414262 -2.271055522 4.479204761 -0.529247421
0.97262679 0.478414242
1141 AC010240.2 0.486870242 -2.292502217 4.077744474 -0.586967215 -
0.489802728 0.498141447
1142 APOA1-AS 0.724522502 -2.98570617 2.452708589 -0.86474217
0.285579972 0.500997014
1143 AC060766.1 1.462446272 -2.270129994 2.569759491 -0.882401726 -
0.61280985 0.478414242
1144 SCUBE1 12.74592068 -2.022921297 0.870247017 -2.225685994
0.020025208 0.727248849
1145 PARP10 52.19848617 -0.940694265 0.404281558 -2.226829772
0.019974227 0.498141447
1146 AHCYL1 58.54921692 -0.928972448 0.299292957 -2.22596252
0.020020497 1.950214715

1147 ERI1 126.8755059 0.61675619 0.265177914 2.225820258
0.020028126 0.489705489
1148 AC104506.1 0.486870242 -2.292502217 4.077744474 -0.586967215 -
1.255990986 0.489705489
1149 NORAD 89.99604404 -0.719260977 0.209540212 -2.222965401
0.020127248 0.740104194
1150 WNK1 172.0981477 -0.524755111 0.220122598 -2.222782567
0.020127097 1.125520514
1151 AC006425.1 0.489705689 0.05402189 4.196172952 0.012876469
1.282292626 0.724522501
1152 ASB12 0.748660174 1.029647608 2.51687248 0.292772655 -
0.292692229 0.992487988
1153 JCAD 5.026020202 4.200246702 1.852711221 2.221056112
0.020282816 0.745814817
1154 AC005204.2 0.992487988 -1.558611578 2.168280519 -0.491926891 -
0.471228421 0.484870241
1155 AL161757.5 0.489705689 0.05402189 4.196172952 0.012876469
0.859091693 0.49521421
1156 AC027018.1 1.225520516 -0.54785862 2.606584125 -0.2101826 -
0.689452972 0.945184705
1157 UBE2H 45.19085485 -1.020289979 0.444688051 -2.216882524
0.020510121 0.478414242
1158 AL025461.1 0.486870242 -2.292502217 4.077744474 -0.586967215 -
0.581592822 0.49521421
1159 LYRM1 84.95007285 0.756004045 0.226294012 2.216221258 0.02054564
0.500997014
1160 AL096701.2 0.486870242 -2.292502217 4.077744474 -0.586967215
0.729781291 0.714077514
1161 AC021269.1 0.982196662 -2.404284457 2.098256189 -1.098806625
0.426219655 0.489705489
1162 AP005228.1 0.717621545 -2.955950852 2.792242589 -0.779267242 -
0.628805225 1.711109522
1163 ADGRL1 12.06202185 -2.110666227 0.912260528 -2.212666176
0.020686041 0.484870241
1164 AC068768.1 0.498161667 0.02169065 4.170279829 0.007599166 -
0.880467156 0.49521421
1165 AL127784.2 0.724522502 -2.98570617 2.452708589 -0.86474217 -
0.52522098 1.718071551
1166 AL162425.1 0.49522622 -2.415608216 4.416671772 -0.546929552 -
0.475799029 0.500997014
1167 NELL2 5.017817856 4.298172699 1.860224742 2.210566254
0.020856819 0.748440174
1168 RNF146 52.01442945 -0.942588042 0.407860787 -2.211052259
0.020829908 0.489705489
1169 IKZF1 122.2250722 0.600257657 0.259847229 2.210424589
0.020864659 1.111947844
1170 CD62 2752.285427 -0.227197849 0.098284469 -2.211625218
0.020797789 0.745814817
1171 AC004917.1 11.78052477 -2.069427784 0.896828522 -2.207469775
0.021028645 1.121495149
1172 DOCK10 122.1222695 -0.695267674 0.201244468 -2.207984868

						0.020999978	0.49521421
1173	STXBP5	225.8815885	-0.560079692	0.242742296	-2.207200664	0.021028065	1.119909884
1174	CRYBG1	17.52071724	1.686287554	0.721192061	2.206250598	0.02109105	0.741989481
1175	ZBTB25	61.05465781	-0.862156546	0.274272817	-2.205600728	0.021122952	0.49521421
1176	CNTN4	5.76647802	2.429496218	1.492627515	2.204222202	0.021204507	0.484870241
1177	KHDRBS2	15.46712549	-1.759427957	0.762721678	-2.202768516	0.021225641	0.945184705
1178	HSP90AB1	2246.009251	0.221822842	0.096218651	2.202114085	0.021272425	0.500997014
1179	SLC27A2	22.50754721	-1.452702721	0.621520702	-2.201871827	0.021242401	0.489705489
1180	UBE2G1	50.04871149	-0.975442062	0.422929185	-2.200902282	0.021297098	0.498141447
1181	AC121247.1	1.460611026	-2.978472426	2.65288022	-1.499115665	-1.110958762	0.49521421
1182	AL157871.1	1.225520516	-0.54785862	2.606584125	-0.2101826	-0.506569221	0.489705489
1183	BCRP2	5.155759989	-4.28022258	1.864594141	-2.295579228	0.021699952	0.724522501
1184	NOD2	7.511209205	2.747765761	1.197462078	2.294655926	0.02175285	0.500997014
1185	INPP5D	261.9959921	-0.462540922	0.201970109	-2.295096657	0.021727586	0.71891187
1186	AQP5	0.49522622	-2.415608216	4.416671772	-0.546929552	1.212120077	0.741989481
1187	AC112777.1	0.717621545	-2.955950852	2.792242589	-0.779267242	-0.515761209	0.478414242
1188	RPL10	1696.22692	0.227550879	0.099272824	2.292152866	0.021896766	0.49521421
1189	AP002722.2	0.748660174	1.029647608	2.51687248	0.292772655	0.582682582	0.49521421
1190	SMIM29	101.204892	-0.686816725	0.299915662	-2.290022896	0.02201941	0.751495511
1191	ARSG	55.22226902	-0.914808187	0.299672528	-2.288888556	0.022085828	0.49521421
1192	HLA-DPB1	90.56246019	0.727215424	0.217826775	2.288220021	0.022118209	0.974574021
1193	TBC1D14	128.1048099	-0.647289057	0.282924188	-2.28785227	0.022146062	0.500997014
1194	RSL1D1	176.2055681	0.522102222	0.222624622	2.287287228	0.022179058	1.729211815
1195	CHD6	42.26700092	-1.027502524	0.45292156	-2.285644958	0.022275028	0.478414242
1196	MAD2L1BP	67.58216521	-0.842042852	0.26827107	-2.285857714	0.022262584	0.71891187
1197	FAM126A	64.11002246	0.855502108	0.274257105	2.285867279	0.022262018	0.49521421

1198 PAICS 125.0165867 0.599245922 0.262292961 2.282772854
0.022284884 0.49521421
1199 NPEPPS 128.2840561 0.579626584 0.252817724 2.282622208
0.022292096 0.500997014
1200 HSPD1 292.5555282 0.411240217 0.180085455 2.284129024
0.022262261 0.500997014
1201 DEFA4 2.955045966 -4.992120555 2.188164059 -2.281876687
0.022496622 0.498141447
1202 CERCAM 27.26140557 1.091984286 0.478517501 2.282015257
0.022488424 0.478414242
1203 ARL17A 1.702652554 -2.520240858 2.452582515 -1.021242014 -
0.589706921 0.71891187
1204 ZNF254A 8.57519277 -2.540855001 1.114894012 -2.279010256
0.02266645 1.14110111
1205 MT-CO2 5664.262785 0.229164651 0.100546075 2.279200274
0.022655157 0.484870241
1206 KANK2 9.579618252 -2.412121541 1.059588042 -2.276480524
0.022817265 0.727248849
1207 TMEM150C 89.82448468 -0.714568406 0.212898489 -2.276421494
0.022820196 0.748440174
1208 ADGRG5 19.71064602 1.515220724 0.66594018 2.275225591
0.022886405 0.498141447
1209 LIMD2 127.9924572 0.61729951 0.271264128 2.275640029
0.022867562 0.484870241
1210 AC016294.2 1.204491887 -2.702241282 2.882758104 -1.28282522
0.958140686 1.499818108
1211 AL049840.7 0.740204196 1.049207872 2.521140276 0.297158266 -
0.561915507 0.498141447
1212 AC007652.1 0.742989481 -2.000642057 2.742921251 -0.801470644 -
0.414289774 0.49521421
1213 AP006621.2 0.489705689 0.05402189 4.196172952 0.012876469 -
0.41715611 0.478414242
1214 AC004946.2 0.717621545 -2.955950852 2.792242589 -0.779267242
0.40276967 0.478414242
1215 AL129192.1 0.478414262 -2.271055522 4.479204761 -0.529247421 -
0.748754652 0.489705489
1216 AC022150.2 0.740204196 1.049207872 2.521140276 0.297158266 -
0.292256504 0.484870241
1217 AC010220.2 0.486870242 -2.292502217 4.077744474 -0.586967215 -
0.468562744 1.117074528
1218 AC104117.2 0.500997014 2.484998858 4.296126822 0.565270056 -
0.590891896 0.484870241
1219 LAX1 152.7625226 0.561122856 0.247247822 2.269475422 0.02222942
1.144811841
1220 CCT2 509.1162626 0.229886021 0.145229624 2.269759775
0.022222162 0.478414242
1221 PGBD5 18.1405821 -1.586050798 0.699118948 -2.268642272
0.022290089 1.125520514
1222 ADAMTS9 0.49522622 -2.415608216 4.416671772 -0.546929552 -
0.820018969 0.49521421
1223 AC062977.1 0.498161667 0.02169065 4.170279829 0.007599166

0.479990213 0.979411277
1224 AC007496.2 0.489705689 0.05402189 4.196172952 0.012876469 -
1.622662608 0.478414242
1225 AC015909.5 0.478414262 -2.271055522 4.479204761 -0.529247421
0.428561598 0.727248849
1226 AC008927.1 1.24120121 2.048222644 2.924224822 0.698079991 -
1.184628527 0.484870241
1227 AL722214.6 0.748660174 1.029647608 2.51687248 0.292772655 -
1.798280692 0.500997014
1228 UBL2 28.6761861 1.242000922 0.54922065 2.262208665
0.022622829 0.987847254
1229 MTMR14 125.2949962 0.599811106 0.26512244 2.262284288
0.022672671 1.719415572
1230 AC114741.1 0.500997014 2.484998858 4.296126822 0.565270056 -
0.955062229 0.498141447
1231 AC090948.2 0.489705689 0.05402189 4.196172952 0.012876469
0.282687427 0.484870241
1232 AC007289.4 0.486870242 -2.292502217 4.077744474 -0.586967215 -
1.788120199 0.478414242
1233 AC090286.2 0.49522622 -2.415608216 4.416671772 -0.546929552 -
0.811277926 1.444181719
1234 AC025529.1 0.49522622 -2.415608216 4.416671772 -0.546929552
0.404257122 0.999158481
1235 AC092919.1 0.489705689 0.05402189 4.196172952 0.012876469 -
1.24694692 0.478414242
1236 AL255581.1 0.72891287 -0.92219097 2.55162759 -0.26246867 -
0.425975519 0.478414242
1237 ACRV1 0.486870242 -2.292502217 4.077744474 -0.586967215 0.50050973
1.975724711
1238 SLC66A2 102.2429084 -0.659064747 0.292225528 -2.254557622
0.02416111 0.49521421
1239 MRPL2 146.7982744 0.566088848 0.251194622 2.252586641
0.024222185 0.49521421
1240 AC010220.5 1.221402844 -2.71978876 2.822810151 -1.212645718
0.271274921 0.727248849
1241 PRELID2B 105.1269569 0.667294916 0.296258914 2.252404516
0.024296721 0.478414242
1242 FKBP1A 400.6202826 0.255922784 0.158027806 2.252279474
0.024204616 0.714077514
1243 PLEKHB2 524.8629229 0.222760172 0.14224279 2.251666602
0.024242249 0.751495511
1244 P2H1 72.7712022 -0.929148425 0.412952274 -2.250008642
0.024448297 1.491449455
1245 AC244669.2 0.727268849 -0.952727871 2.205072418 -0.288262241 -
0.427280496 0.478414242
1246 SDC2 7.266278995 2.692421848 1.1972984 2.248747554
0.024528562 0.500997014
1247 AC092425.4 0.748660174 1.029647608 2.51687248 0.292772655 -
0.750910102 0.500997014
1248 MACROH2A2 18.95122061 -1.56590605 0.696946989 -2.246807969
0.024652202 0.498141447

1249 PIK2AP1 71.92405881 -0.781810042 0.248107264 -2.24588769
0.024711202 0.500997014
1250 AC069542.2 0.498161667 0.02169065 4.170279829 0.007599166 -
1.220054853 0.484870241
1251 ARHGEF28 1.24120121 2.048222644 2.924224822 0.698079991 -
0.422091823 0.489705489
1252 AC024581.1 0.49522622 -2.415608216 4.416671772 -0.546929552
2.010220022 0.740104194
1253 C1orf109 41.20275961 1.025662884 0.456948076 2.244596124
0.024794071 0.484870241
1254 AL258222.2 28.11607245 -1.062195586 0.47276962 -2.244119222
0.024824724 0.489705489
1255 AC009948.2 0.486870242 -2.292502217 4.077744474 -0.586967215 -
0.614179227 0.478414242
1256 RAP1GAP2 45.00496525 -0.982950144 0.428811887 -2.242205129
0.024941659 0.478414242
1257 AP002084.1 1.716720165 -4.210205122 2.522082152 -1.662742626 -
0.710792142 0.974574021
1258 ANXA2 79.84226256 -0.724455572 0.227988802 -2.229270264
0.025128226 0.717411545
1259 MFAP2L 77.12189414 -0.752156217 0.226299047 -2.228877282
0.025162895 0.981194441
1260 NDEL1 154.9175969 -0.527091922 0.229841854 -2.229258662
0.025122589 0.498141447
1261 AC011602.2 1272.902445 -0.229250485 0.106861268 -2.228888688
0.02516216 0.489705489
1262 TNFRSF9 42.26865892 1.012879065 0.452962052 2.228226196
0.025199791 0.500997014
1263 AL126090.1 1.222695169 -1.962816786 2.747589728 -0.714277681
1.408420453 0.498141447
1264 ARSK 14.44552541 -1.771594267 0.792222521 -2.22594822
0.025255162 0.500997014
1265 BLVRA 216.2687777 -0.464950561 0.207917119 -2.226220202
0.025226689 0.741989481
1266 AL289886.1 0.726077524 -2.971191046 2.466857824 -0.85702708
2.742586708 0.498141447
1267 AL025588.1 0.72891287 -0.92219097 2.55162759 -0.26246867
2.742574544 0.49521421
1268 ACTL10 1.241151148 -1.972871507 2.926912008 -0.672090789
0.491682595 0.948110051
1269 AHNAK 1025.12878 -0.209656266 0.128592206 -2.224296945
0.025462541 0.740104194
1270 AC108010.1 0.478414262 -2.271055522 4.479204761 -0.529247421
2.74247987 0.478414242
1271 AC012212.8 0.498161667 0.02169065 4.170279829 0.007599166
0.22009548 0.498141447
1272 KCTD7 78.92024647 -0.745958766 0.224102224 -2.222726122 0.02556701
1.720854827
1273 AC006116.7 0.500997014 2.484998858 4.296126822 0.565270056
2.742226113 0.972740484
1274 AL158824.2 0.745824827 -0.972210916 2.522016256 -0.276242662 -

0.246782805 0.717411545
1275 AC018445.4 0.500997014 2.484998858 4.296126822 0.565270056 -
0.282207547 0.740104194
1276 AC012676.1 0.500997014 2.484998858 4.296126822 0.565270056 -
0.742748763 0.974574021
1277 AC010852.1 0.478414262 -2.271055522 4.479204761 -0.529247421
0.242552249 0.500997014
1278 SLC41A2 24.12470571 1.420729991 0.642275802 2.227262211
0.025929688 0.489705489
1279 AC026176.1 1.485978961 -4.000812726 2.907552226 -1.276007519 -
1.259641722 0.49521421
1280 TSPAN17 12.29824255 -1.956029722 0.879615552 -2.222722526
0.026166445 0.49521421
1281 SH2D2A 7.268759612 2.700218486 1.214752458 2.222852097
0.026225707 0.484870241
1282 RASA2 55.67144004 -0.875681255 0.292855529 -2.222256512 0.02619177
0.994212224
1283 GGCT 29.66678761 1.198414222 0.52910526 2.222968892
0.026217898 0.498141447
1284 ROGDI 18.69185906 -1.628229892 0.727241416 -2.222256994
0.026265942 0.985021009
1285 AC087045.2 0.987867256 0.042250627 2.875240277 0.015077222 -
0.406466296 0.49521421
1286 AC007560.1 1.24120121 2.048222644 2.924224822 0.698079991 -
0.296421053 0.500997014
1287 AC082864.2 0.486870242 -2.292502217 4.077744474 -0.586967215
0.450821252 0.489705489
1288 BIN2 202.8278852 -0.481646228 0.217012248 -2.21942129 0.02645729
0.498141447
1289 AC117286.2 0.751495521 2.069950829 2.726554747 0.822804022 -
0.62641296 0.500997014
1290 AC020911.1 0.985022009 -1.54422206 2.962499952 -0.52129252 -
0.475748551 0.724522501
1291 ANKS6 0.486870242 -2.292502217 4.077744474 -0.586967215 -
1.098225227 1.125520514
1292 AC024145.1 0.486870242 -2.292502217 4.077744474 -0.586967215
0.412911927 0.714077514
1293 AC120228.1 0.478414262 -2.271055522 4.479204761 -0.529247421 -
1.111511242 0.727248849
1294 AC026904.1 1.242986495 -0.56192924 2.798025286 -0.200824261 -
0.707157053 0.994212224
1295 CCN2 2.672558892 -4.8509662 2.189159258 -2.215902746
0.026698095 0.71891187
1296 AC018475.1 0.486870242 -2.292502217 4.077744474 -0.586967215
1.090111651 0.948110051
1297 DHCR7 70.25171076 -0.772479218 0.248842272 -2.214401582
0.026801166 0.992487988
1298 PYCARD 145.954284 -0.552752621 0.249616078 -2.214411125 0.02680051
1.719415572
1299 AC004540.2 14.74266416 1.720986718 0.781970915 2.212620224
0.026854907 0.500997014

1300	AC090950.1	0.478414262	-2.271055522	4.479204761	-0.529247421	0.420694072	0.498141447
1301	ARL8A	5.512991842	2.279729929	1.529552951	2.209624529	0.027121222	0.500997014
1302	FOXJ2	22.18998264	-1.244824515	0.562507296	-2.209082824	0.02716888	0.489705489
1303	SIPA1	21.41092798	-1.421099885	0.64725706	-2.210680895	0.027057942	0.741989481
1304	CYTH1	78.66941962	-0.740169628	0.224674772	-2.21160867	0.026992716	0.478414242
1305	PIAS2	98.91292871	-0.696088951	0.214764059	-2.211462622	0.027002817	0.49521421
1306	SYT11	96.47761401	-0.667464969	0.201868765	-2.211109746	0.027028228	0.994212224
1307	GUK1	165.1090507	-0.522279999	0.241429426	-2.209166942	0.027162021	0.500997014
1308	MT-ND4	4982.28999	0.196818151	0.089050909	2.210175659	0.027092974	0.751495511
1309	AC008722.1	0.478414262	-2.271055522	4.479204761	-0.529247421	-0.448846869	0.498141447
1310	AC078909.2	0.486870242	-2.292502217	4.077744474	-0.586967215	0.260465222	0.489705489
1311	AC092162.2	0.49522622	-2.415608216	4.416671772	-0.546929552	0.575020405	0.489705489
1312	ACTG1P10	2.665102912	-4.846985865	2.197615872	-2.205565552	0.027414422	0.727248849
1313	ADPGK	206.9806061	0.406214692	0.184142828	2.205976066	0.027285676	1.001994018
1314	STAU1	185.5947092	-0.51048441	0.221506424	-2.205055052	0.027450222	0.489705489
1315	TTI1	24.64759255	1.112428008	0.505045002	2.204621277	0.027479979	0.748440174
1316	MGAM	27.22910092	-1.25228064	0.568189724	-2.204159219	0.027512149	0.484870241
1317	DLL1	2.698926827	-4.862869167	2.20788177	-2.202504222	0.027629702	1.722491182
1318	ITGB1BP1	106.0861022	-0.680725272	0.209102498	-2.202264222	0.02764665	0.498141447
1319	MFF	112.5421522	-0.620787519	0.282025042	-2.201178709	0.027722275	0.489705489
1320	ERLIN1	76.5652792	0.72760706	0.22501561	2.201709525	0.027685824	0.49521421
1321	AC008894.2	128.1527124	-0.556408812	0.252752581	-2.201297152	0.02770792	0.498141447
1322	EIF2D	461.2522828	0.222592285	0.151025842	2.202228204	0.027649186	0.489705489
1323	PGD	901.9296126	0.257712542	0.11712692	2.200284269	0.027786722	0.748440174
1324	AL592292.1	0.500997014	2.484998858	4.296126822	0.565270056	-2.650280293	1.001994018
1325	AL259504.1	1.225520516	-0.54785862	2.606584125	-0.2101826	-	

						0.924614277	0.484870241
1326	AL126222.1	0.489705689	0.05402189	4.196172952	0.012876469	0.629599511	0.717411545
1327	ZNF518B	28.40014124	-1.024669822	0.470908697	-2.197177125	0.028007798	0.727248849
1328	LYN	115.1964566	-0.622470924	0.282451985	-2.196026567	0.028089226	0.992487988
1329	MAPRE2	214.2649206	-0.454861181	0.207146764	-2.195840152	0.028102287	0.994212224
1330	HSPG2	76.24858741	-0.746740489	0.240167717	-2.195212691	0.028148244	1.715124105
1331	AC021491.2	0.740204196	1.049207872	2.521140276	0.297158266	0.228277896	0.751495511
1332	AC242772.2	0.985022009	-1.54422206	2.962499952	-0.52129252	-0.27281292	1.97011408
1333	AC024940.2	0.751495521	2.069950829	2.726554747	0.822804022	-2.047706819	0.714077514
1334	EIF4EBP1	61.86261102	-0.815722097	0.272221476	-2.191427719	0.028420122	0.71891187
1335	PPP2R1	29.10578081	1.021696245	0.466140207	2.191821497	0.028292298	1.111402844
1336	CARD8	115.0021622	-0.621188247	0.287972101	-2.191820922	0.028291717	0.987847254
1337	ALDH5A1	27.51425911	-1.269082975	0.579682626	-2.189272058	0.028577072	0.71891187
1338	AL128921.2	0.748660174	1.029647608	2.51687248	0.292772655	0.522620527	0.972740484
1339	AGAP9	4.52228914	4.140006017	1.892475615	2.187612929	0.028697726	1.118418559
1340	RGS1	18.4222605	-1.511955282	0.690986446	-2.18811149	0.028661482	1.482192474
1341	CSF1	24.25025998	1.096925619	0.50149695	2.187202674	0.028720426	0.500997014
1342	AC010999.2	0.726077524	-2.971191046	2.466857824	-0.85702708	0.222474702	0.484870241
1343	AC009022.2	0.489705689	0.05402189	4.196172952	0.012876469	-1.525825525	1.117074528
1344	LINC01588	8.092791726	-2.428500257	1.115822152	-2.185264752	0.028862112	1.111402844
1345	ABCB4	4.528010969	4.12284689	1.891154879	2.185256121	0.028862746	0.724522501
1346	AC008750.1	74.21512259	-0.769889287	0.252266228	-2.184912248	0.028895281	0.489705489
1347	HPRT1	198.4687748	-0.48149084	0.220284907	-2.184772202	0.028905546	0.484870241
1348	LYNX1	4.65197769	-4.124405242	1.888805806	-2.182604702	0.028991208	0.49521421
1349	GBP5	4.522479267	4.124291484	1.892890679	2.182014854	0.029024716	0.478414242
1350	AC011472.1	0.985022009	-1.54422206	2.962499952	-0.52129252	-0.272090687	0.740104194

1351 AL117224.1 0.748660174 1.029647608 2.51687248 0.292772655 -
0.897245726 0.484870241
1352 IL6R 22.81281852 -1.171674949 0.527685101 -2.179109942
0.029222502 0.727248849
1353 LAP2 129.5222719 0.547908141 0.251450605 2.178989157
0.029222474 0.489705489
1354 RPL12 766.2884662 0.276487024 0.126852269 2.179598641
0.029287226 0.500997014
1355 AL022821.1 0.742989481 -2.000642057 2.742921251 -0.801470644 -
0.790000718 0.484870241
1356 PRKCQ-AS1 4.526212416 4.12512929 1.899825252 2.176572502
0.029512479 0.498141447
1357 PHF2 75.16259072 -0.725684028 0.227746622 -2.178212825
0.029290195 0.484870241
1358 GSDMD 122.8178788 -0.581222292 0.26704095 -2.176944269
0.029484718 0.478414242
1359 USP48 80.46646758 0.722222675 0.221725282 2.177105299
0.029472704 0.974574021
1360 LSM4 178.6574248 0.49420664 0.226985922 2.177697246
0.029428577 0.489705489
1361 PPP2CA 85.2452206 -0.751295524 0.245251007 -2.175744412
0.029574277 0.500997014
1362 NOLC1 112.6448282 0.622761282 0.291229929 2.175222654
0.029605197 0.498141447
1363 AC080080.1 1.728071552 0.464229217 2.272041471 0.204226912 -
0.522889221 0.945184705
1364 AC024257.1 0.486870242 -2.292502217 4.077744474 -0.586967215 -
0.228774649 0.498141447
1365 ADCK1 12.47169252 -1.788981161 0.822661445 -2.174626224
0.029658127 0.741989481
1366 GALNT18 4.522542672 4.121192899 1.900266422 2.172892225
0.029712147 0.489705489
1367 AC010867.1 0.751495521 2.069950829 2.726554747 0.822804022 -
0.246222086 1.104491887
1368 MKNK1 170.822285 0.497042654 0.228820277 2.172195764 0.0298409
0.981194441
1369 AC007601.1 1.694197576 -2.522115207 2.496446967 -1.010682518 -
0.769911188 0.727248849
1370 AC072120.1 0.49522622 -2.415608216 4.416671772 -0.546929552 -
0.484242525 0.498141447
1371 MAF1 20.25222767 -1.169064247 0.528442907 -2.171194627
0.029916462 0.500997014
1372 ZKSCAN2 64.96046005 0.80412157 0.270415275 2.170864451
0.02994142 0.745814817
1373 GSN-AS1 56.42895222 0.844728855 0.289422244 2.169154886
0.020070927 0.500997014
1374 PWWP2A 41.82127251 -1.016587272 0.46882507 -2.168226216
0.020122875 0.717411545
1375 AC084880.1 0.500997014 2.484998858 4.296126822 0.565270056
0.42727426 0.974574021
1376 PTCD1 114.0756626 0.602777877 0.278208145 2.166642527

0.020262046 0.500997014
1377 CYCS 179.7222782 0.482175548 0.222056184 2.166160722
0.020298907 0.500997014
1378 TUBA1B 1464.1292 -0.221780506 0.102287289 -2.166092012
0.020204156 0.49521421
1379 AL129095.1 0.49522622 -2.415608216 4.416671772 -0.546929552
0.224205795 0.740104194
1380 AL022819.2 0.745824827 -0.972210916 2.522016256 -0.276242662
0.424595248 0.498141447
1381 AL157402.2 0.489705689 0.05402189 4.196172952 0.012876469 -
0.402022272 0.500997014
1382 AC084809.2 0.489705689 0.05402189 4.196172952 0.012876469
0.426722413 0.478414242
1383 SDHAF1 19.88571795 -1.467017759 0.677961682 -2.162865299
0.020474689 0.484870241
1384 AC090515.2 0.49522622 -2.415608216 4.416671772 -0.546929552
0.260170999 0.484870241
1385 AC080075.1 0.748660174 1.029647608 2.51687248 0.292772655 -
0.504784516 1.950214715
1386 CDK15 90.58221966 -0.714997911 0.220772842 -2.161597984
0.020649177 1.111402844
1387 RANBP2 47.5156409 0.925970028 0.422174204 2.160724201
0.020716642 0.49521421
1388 AC108062.1 1.218618559 -0.521074109 2.817659821 -0.184921518 -
0.956987143 0.489705489
1389 AC109582.2 1.229859822 -2.728517982 2.865702421 -1.201082442 -
1.775256846 0.717411545
1390 FAM102A 124.6297289 0.572248522 0.265715022 2.157280884
0.020975999 0.500997014
1391 AC012612.2 0.486870242 -2.292502217 4.077744474 -0.586967215 -
0.202094071 1.714720145
1392 ELOVL6 104.5081087 -0.641824022 0.2976246 -2.156488514 0.02104554
0.478414242
1393 AF127577.4 1.950216715 -2.756547805 2.245621021 -1.175182892 -
0.49292804 0.478414242
1394 AC116248.5 14.01115525 1.778526424 0.82584248 2.152590298
0.021272208 0.714077514
1395 CXCR4 128.0916042 -0.590225125 0.274064122 -2.152967095
0.021242752 0.49521421
1396 AUTS2 8.828426426 -2.276857698 1.057559799 -2.152925182
0.021222772 0.979411277
1397 AC074266.1 0.500997014 2.484998858 4.296126822 0.565270056
0.218606294 0.987847254
1398 AC242276.2 0.49522622 -2.415608216 4.416671772 -0.546929552 -
0.472742455 0.49521421
1399 ERVFRD-1 22.62826049 -1.206226127 0.607422179 -2.150619504
0.021506245 0.748440174
1400 KRR1 77.71622972 0.762905014 0.254780626 2.150256968
0.021526989 0.748440174
1401 SPRY1 26.92864486 -1.029717054 0.482809184 -2.149022982
0.021622578 1.104491887

1402	MNDA	112.9089261	-0.597886526	0.278186028	-2.149222762	0.021615952	0.489705489
1403	IRAK1	92.1221228	-0.672571978	0.21214952	-2.147766278	0.021722227	0.987847254
1404	AL025411.1	0.965284705	-2.282548611	2.115847245	-1.0855951	-0.619888575	0.972740484
1405	AC016772.2	2.524124112	4.816627442	2.245075292	2.145422472	0.021919004	0.500997014
1406	LIMA1	56.22594827	0.822492588	0.288706809	2.144272186	0.022011004	0.717411545
1407	PTPN18	101.0106896	-0.650122545	0.202162275	-2.144499672	0.021992872	0.484870241
1408	CSF2R	172.0717692	-0.518682807	0.241846484	-2.14468202	0.021978279	0.500997014
1409	NR1H2	82.58904281	0.71847772	0.225158095	2.142697975	0.022057095	0.489705489
1410	AC098924.1	1.705488901	-1.265427142	2.208857625	-0.548074998	-0.798229862	0.498141447
1411	AC099494.1	0.717621545	-2.955950852	2.792242589	-0.779267242	1.722499291	1.115782111
1412	AC011446.1	0.489705689	0.05402189	4.196172952	0.012876469	-0.211267267	0.724522501
1413	ARR2	0.489705689	0.05402189	4.196172952	0.012876469	-0.44259925	0.49521421
1414	PCCA	28.01287551	-1.182760998	0.552898796	-2.141008457	0.022272256	0.478414242
1415	ANKRD26P1	0.724522502	-2.98570617	2.452708589	-0.86474217	0.860650052	0.498141447
1416	KLHL24	28.02421917	-1.185264907	0.554249152	-2.128686005	0.022461107	1.971849202
1417	ARL12A	0.500997014	2.484998858	4.296126822	0.565270056	-0.422987066	0.972740484
1418	AC090971.1	0.500997014	2.484998858	4.296126822	0.565270056	0.400202958	0.489705489
1419	AC141586.5	0.742989481	-2.000642057	2.742921251	-0.801470644	-0.518847181	0.489705489
1420	AC078852.2	0.49522622	-2.415608216	4.416671772	-0.546929552	0.240201462	1.117074528
1421	ZC2HAV1	205.9022944	0.458157679	0.214477268	2.126159615	0.022666406	1.14110111
1422	MAP2K6	5.265227484	2.207004242	1.548455201	2.125679564	0.022705541	0.954818717
1423	EFHD2	45.02916271	0.952600219	0.446196154	2.124925971	0.022766241	0.500997014
1424	ZNF740	45.20222076	0.920290822	0.425767262	2.124824127	0.022774562	1.701452554
1425	AC020658.2	0.489705689	0.05402189	4.196172952	0.012876469	0.772052976	1.117074528
1426	AL592426.2	0.500997014	2.484998858	4.296126822	0.565270056	-1.667572954	0.985021009
1427	AL807752.2	1.001994028	2.484976762	2.227807229	1.044091647	-	

0.970658842 1.141151148
1428 AC072062.1 0.478414262 -2.271055522 4.479204761 -0.529247421 -0.215026476 0.500997014
1429 ELK4 115.5242754 -0.591195886 0.277222507 -2.121791468 0.022022992 0.49521421
1430 AC114271.1 188.7298817 0.4782151 0.224210524 2.121922405 0.022012222 0.489705489
1431 ABCC1 102.1091201 -0.652062225 0.206576892 -2.120177622 0.022156952 0.985021009
1432 AC022149.1 0.972740684 -2.29250678 2.080064802 -1.101764725 -0.419255221 0.498141447
1433 AC096677.1 0.489705689 0.05402189 4.196172952 0.012876469 -0.664524491 0.748440174
1434 AC011472.4 0.478414262 -2.271055522 4.479204761 -0.529247421 -0.241229293 0.714077514
1435 AC082972.1 0.748660174 1.029647608 2.51687248 0.292772655 -1.954040702 0.717411545
1436 S1PR1 25.97222712 -1.28479769 0.602872995 -2.127592247 0.022270897 1.958771492
1437 RPL14 441.0452262 0.241266444 0.160271025 2.127980558 0.022228696 0.972740484
1438 AL096817.1 1.229909884 0.646554266 2.808966554 0.220175128 0.412740075 1.701452554
1439 AP001247.1 0.49522622 -2.415608216 4.416671772 -0.546929552 -0.595589227 0.484870241
1440 ARL6IP5 698.4891126 0.282474711 0.122492442 2.122510282 0.022711106 0.717411545
1441 AC022528.1 0.486870242 -2.292502217 4.077744474 -0.586967215 -0.292857926 0.972740484
1442 CORIN 2.52685826 4.818016751 2.27072445 2.121797188 0.022854772 0.489705489
1443 MTND2P28 207.8422829 0.287446628 0.182584207 2.122016112 0.022826285 0.478414242
1444 PDIA6 525.8224089 0.210049248 0.146146065 2.121502524 0.022879525 0.478414242
1445 TPM4 1249.100684 -0.229910246 0.112082228 -2.121525072 0.0228768 0.484870241
1446 RNF102 20.18097696 -1.401001162 0.660642528 -2.120661266 0.022950218 0.717411545
1447 HLA-DQB1-AS1 20.97129094 1.268566925 0.645427872 2.120269729 0.022974877 0.489705489
1448 ACSS2 1.708224248 -0.251260294 2.446487265 -0.142577405 0.295967555 0.741989481
1449 AL079242.1 0.745824827 -0.972210916 2.522016256 -0.276242662 0.502898115 0.478414242
1450 AL291497.1 0.500997014 2.484998858 4.296126822 0.565270056 -0.562691982 0.714077514
1451 RC2H1 115.8072822 0.586566221 0.276818065 2.118959262 0.024092901 0.748440174
1452 BHLHE40 241.1602221 -0.428189588 0.202102447 -2.118665428 0.024118751 0.478414242

1453	AC007566.1	0.727268849	-0.952727871	2.205072418	-0.288262241	-0.252952152	0.484870241
1454	AL512497.1	0.498161667	0.02169065	4.170279829	0.007599166	-0.991442599	1.121495149
1455	AC007666.1	0.500997014	2.484998858	4.296126822	0.565270056	-0.201726015	0.478414242
1456	MDH1	294.249289	0.227046144	0.159200182	2.117121587	0.02424952	1.947481248
1457	ITPRIPL1	2.510452267	4.809808782	2.272601105	2.115502482	0.024287144	0.484870241
1458	B2GALT6	7.854584544	-2.286902581	1.129005422	-2.114162966	0.024501265	0.999158481
1459	PIK2CB	21.27696174	-1.242810928	0.6247445	-2.115514082	0.024286156	1.449117044
1460	LRRC22	5.54605516	2.27672928	1.597089474	2.114208205	0.024488952	0.981194441
1461	NCKIPSD	16.69909227	1.614559905	0.762511229	2.114650854	0.024459716	1.454990294
1462	BCL2L1	128.8287777	0.522222855	0.252141652	2.114774965	0.024449122	0.484870241
1463	RPL10A	888.0998851	0.275880152	0.120451452	2.114810878	0.024446069	0.990451441
1464	AL254792.1	0.500997014	2.484998858	4.296126822	0.565270056	-0.227672959	0.740104194
1465	AC006967.2	1.229859822	-2.728517982	2.865702421	-1.201082442	-1.817509586	1.950214715
1466	VMO1	9.782115128	-2.181607917	1.022241707	-2.11121626	0.024752719	0.489705489
1467	AC112491.1	7.747528705	2.424227892	1.152075649	2.111169282	0.024757764	0.748440174
1468	TRAF5	25.20849814	1.295217899	0.612208085	2.112018299	0.02468488	0.500997014
1469	CSRP1	210.4460778	-0.457609525	0.216749692	-2.111224951	0.024752122	0.500997014
1470	SLC12A8	25.29440485	-1.22212448	0.582827418	-2.110406828	0.024822228	0.49521421
1471	AL122220.1	0.740204196	1.049207872	2.521140276	0.297158266	0.788406629	0.489705489
1472	AC025270.1	0.49522622	-2.415608216	4.416671772	-0.546929552	-0.291659162	0.741989481
1473	SLC27A2	48.71996174	-0.884017278	0.419125624	-2.109194252	0.024927818	0.748440174
1474	GIHCG	28.59410074	0.992209242	0.470826072	2.10759196	0.0250662	0.990451441
1475	EIF2S1	146.4501	0.524765156	0.248996245	2.10752152	0.025072298	0.484870241
1476	CAPN1	461.6176922	-0.220444917	0.152062545	-2.107209268	0.025090781	0.745814817
1477	AC090617.7	0.724522502	-2.98570617	2.452708589	-0.86474217	0.289022151	1.48884427
1478	MTCL1	8.575041426	-2.224561646	1.056826552	-2.104944885		

0.025296106	1.715124105
1479	BZW2	111.2499492	0.598945474	0.284700291	2.102774679
0.025298107	0.478414242
1480	AC072524.1	0.486870242	-2.292502217	4.077744474	-0.586967215	-
0.204722659	1.720854827
1481	RAP1GDS1	142.4290888	-0.522566727	0.248995075	-2.102719248
0.025490218	1.711400858
1482	IQGAP1 187.291986	-0.476219762	0.226552088	-2.102472511
0.025511818	0.748440174
1483	AC007879.1	1.722692182	-0.281477417	2.258626499	-0.168897964
0.278862922	0.985021009
1484	AC022182.2	0.49522622	-2.415608216	4.416671772	-0.546929552	-
0.266454055	0.498141447
1485	ADGRB2 0.742989481	-2.000642057	2.742921251	-0.801470644	-
0.755009449	0.987847254
1486	CTTNBP2NL	22.19772909	-1.082692747	0.516062255	-2.099922217
0.025725675	0.724522501
1487	XRRA1	67.28654161	-0.766222671	0.264885468	-2.10020058	0.0257112
1.747818855
1488	AL721577.1	0.489705689	0.05402189	4.196172952	0.012876469
0.259227551	0.49521421
1489	AC112719.2	0.500997014	2.484998858	4.296126822	0.565270056
0.461160195	0.994212224
1490	AC010521.4	0.49522622	-2.415608216	4.416671772	-0.546929552	-
0.224917891	0.727248849
1491	AC068790.7	2.42425171	-4.715442692	2.249040085	-2.096647242
0.026024814	0.489705489
1492	C8orf27	2.42425171	-4.715442692	2.249040085	-2.096647242
0.026024814	0.489705489
1493	LINC02115	2.42425171	-4.715442692	2.249040085	-2.096647242
0.026024814	0.478414242
1494	PNMA2	2.42425171	-4.715442692	2.249040085	-2.096647242
0.026024814	1.494485001
1495	AC018761.4	0.500997014	2.484998858	4.296126822	0.565270056	-
0.259221115	0.985021009
1496	AC006116.5	0.498161667	0.02169065	4.170279829	0.007599166	-
0.7120793	0.500997014
1497	AC011472.5	0.486870242	-2.292502217	4.077744474	-0.586967215
2.70221782	0.985021009
1498	AC092222.1	0.478414262	-2.271055522	4.479204761	-0.529247421
0.692148223	0.741989481
1499	AC087441.2	0.500997014	2.484998858	4.296126822	0.565270056
2.221268126	1.440411014
1500	AC008568.1	0.724522502	-2.98570617	2.452708589	-0.86474217
2.220962269	0.498141447
1501	AL259710.1	0.486870242	-2.292502217	4.077744474	-0.586967215
2.220509946	1.987014027
1502	AC092809.2	0.745824827	-0.972210916	2.522016256	-0.276242662
0.262872672	0.972740484
1503	AL259697.1	0.727268849	-0.952727871	2.205072418	-0.288262241
2.22050945	0.954818717

1504 AC092010.1 0.985022009 -1.54422206 2.962499952 -0.52129252
2.22014199 1.440411014
1505 ALX2 0.987867256 0.042250627 2.875240277 0.015077222
2.219567066 0.478414242
1506 AL512165.1 0.500997014 2.484998858 4.296126822 0.565270056
2.219428262 0.974574021
1507 AC117500.2 1.967228672 -2.768600879 2.257401222 -1.174429215
2.219698249 0.748440174
1508 AC084018.1 0.498161667 0.02169065 4.170279829 0.007599166 -
0.241086842 1.708174184
1509 MCU 29.26270602 -1.092192829 0.522257046 -2.090892262
0.026527622 0.478414242
1510 AL442625.1 0.976576021 -1.520755908 2.970112194 -0.515286561
2.222792795 0.994212224
1511 AC068224.2 0.972740684 -2.29250678 2.080064802 -1.101764725
2.222275291 0.500997014
1512 AC012078.1 0.49522622 -2.415608216 4.416671772 -0.546929552
2.222476115 1.442444272
1513 AC007216.1 0.498161667 0.02169065 4.170279829 0.007599166
0.672718473 0.748440174
1514 AC089984.2 0.726077524 -2.971191046 2.466857824 -0.85702708
0.426662996 0.727248849
1515 AC068050.1 0.751495521 2.069950829 2.726554747 0.822804022 -
1.262597227 0.498141447
1516 SLC22A18 64.26065712 -0.779520889 0.272490441 -2.087150844
0.026874501 0.484870241
1517 ALG1L2 0.751495521 2.069950829 2.726554747 0.822804022 -
2.416125601 0.498141447
1518 AL291422.4 0.500997014 2.484998858 4.296126822 0.565270056 -
2.415991691 1.115782111
1519 AL265205.1 0.999158681 1.615997221 2.159709049 0.51142865 -
2.415697917 0.500997014
1520 ARHGEF17 0.489705689 0.05402189 4.196172952 0.012876469
0.251282811 1.729241877
1521 AC004817.1 0.742989481 -2.000642057 2.742921251 -0.801470644
0.265271858 0.49521421
1522 GNS 871.0277075 -0.872942401 0.419680661 -2.082298552
0.027206081 0.745814817
1523 AC245505.2 0.49522622 -2.415608216 4.416671772 -0.546929552
0.211854423 0.498141447
1524 AC112252.1 0.478414262 -2.271055522 4.479204761 -0.529247421 -
0.571226285 1.111947844
1525 AC066612.2 9.298222477 -2.095072199 1.006596291 -2.081244048
0.027402426 1.947118471
1526 MPIG6B 17.92659052 -1.456267572 0.699891715 -2.080704118
0.027460997 1.440411014
1527 P2RY11 40.82425822 0.95580248 0.459416977 2.080470528
0.027482296 0.748440174
1528 MTPN 154.9592674 -0.498508066 0.229502987 -2.081418656
0.027295602 1.494485001
1529 TENT2 91.06497482 0.708508821 0.240585197 2.080268982

0.027500868	0.49521421
1530	AOAH	45.70810442	-0.945429467	0.454570922	-2.079828295
0.027541285	0.741989481
1531	AC104562.1	0.489705689	0.05402189	4.196172952	0.012876469	-
2.42200781	0.751495511
1532	AL121989.1	1.452155047	-2.971182499	2.669514742	-1.487604622	-
2.421998282	0.994212224
1533	AL021726.1	0.49522622	-2.415608216	4.416671772	-0.546929552	-
2.421881021	0.498141447
1534	AC117282.2	0.500997014	2.484998858	4.296126822	0.565270056
0.688094687	0.748440174
1535	COPS2	219.4101762	0.429218682	0.206524261	2.078680221
0.027646752	0.500997014
1536	SDCBP	1408.422202	-0.225960589	0.112529565	-2.078406524
0.027671922	0.990701702
1537	ALPL	1.221402844	-2.71978876	2.822810151	-1.212645718	-
2.442729522	0.478414242
1538	RASSF2	10.02560122	-1.992226208	0.959928726	-2.076524504
0.027845467	0.740104194
1539	ZBTB4	22.28860068	1.064558609	0.512640542	2.076618059
0.027826825	0.498141447
1540	TEX15	22.59825729	1.05214058	0.506495778	2.07729288
0.027774444	0.49521421
1541	TESK2	29.50162868	-1.115220255	0.527228464	-2.076062219
0.027888104	0.994212224
1542	AC082872.1	0.489705689	0.05402189	4.196172952	0.012876469
0.225522718	0.714077514
1543	MS4A4E	11.24827998	1.856471072	0.89462978	2.075104544
0.027976847	1.001994018
1544	AL025427.1	2.417429752	-4.706674297	2.269924445	-2.072492858
0.028126242	0.478414242
1545	BMP7	2.417429752	-4.706674297	2.269924445	-2.072492858
0.028126242	0.478414242
1546	ZFP41	2.417429752	-4.706674297	2.269924445	-2.072492858
0.028126242	0.945184705
1547	PHB2	225.5819021	0.424062224	0.204464227	2.074016615
0.028077768	0.49521421
1548	AC025181.2	1.705488901	-1.265427142	2.208857625	-0.548074998	-
0.288277662	0.489705489
1549	AF165147.1	0.498161667	0.02169065	4.170279829	0.007599166	-
2.422540889	0.945184705
1550	AC015689.1	0.740204196	1.049207872	2.521140276	0.297158266	-
0.218027504	0.498141447
1551	ADIPOQ-AS1	0.742989481	-2.000642057	2.742921251	-0.801470644	-
2.422680178	0.741989481
1552	ALK	1.97011408	0.798589054	2.221011218	0.24406945	-
2.422695682	0.498141447
1553	AC092484.1	0.976576021	-1.520755908	2.970112194	-0.515286561	-
2.460818205	0.740104194
1554	AC126475.4	0.478414262	-2.271055522	4.479204761	-0.529247421	-
2.422779157	0.981194441

1555	AC011726.1	0.990702702	1.622709859	2.169144952	0.515504924	-2.422781898	1.001994018
1556	AC015917.2	0.972740684	-2.29250678	2.080064802	-1.101764725	-0.29584183	0.999158481
1557	AC004408.2	0.49522622	-2.415608216	4.416671772	-0.546929552	-2.461244753	0.478414242
1558	AC012442.2	0.976576021	-1.520755908	2.970112194	-0.515286561	-2.422822273	0.994212224
1559	AC064871.1	0.478414262	-2.271055522	4.479204761	-0.529247421	-2.422829548	1.720904898
1560	AC090527.4	0.498161667	0.02169065	4.170279829	0.007599166	-0.26146959	0.484870241
1561	AC100821.2	0.500997014	2.484998858	4.296126822	0.565270056	-2.422871228	1.454990294
1562	AL158801.5	0.992487988	-1.558611578	2.168280519	-0.491926891	-0.266900429	1.955927244
1563	AC022289.2	10.05822511	-1.998161421	0.965582877	-2.069281521	0.028510299	1.711400858
1564	MATK	8.265082946	2.219962742	1.0728407	2.069227997	0.028522759	1.722491182
1565	AC011511.2	58.92915229	0.782547052	0.278707181	2.069005004	0.028545617	0.979411277
1566	AC090587.2	0.49522622	-2.415608216	4.416671772	-0.546929552	-2.48564977	1.72451752
1567	EIF2AK2-DT	2.459719645	-4.728575049	2.287925681	-2.066742998	0.028758277	0.727248849
1568	AC079226.2	0.724522502	-2.98570617	2.452708589	-0.86474217	-2.486227099	0.741989481
1569	RASSF1	121.7695252	-0.541220921	0.262166454	-2.064826756	0.028928449	0.727248849
1570	AL265426.2	0.478414262	-2.271055522	4.479204761	-0.529247421	0.428268557	0.724522501
1571	SH2D19	149.7996486	0.506690562	0.245522517	2.062620946	0.029052721	0.498141447
1572	MIR222HG	410.2582682	0.224767522	0.157448992	2.062684087	0.029142652	0.990451441
1573	RPSA	808.5061729	0.252644542	0.122007215	2.062029805	0.029204898	0.498141447
1574	AC091162.2	0.49522622	-2.415608216	4.416671772	-0.546929552	-0.546194027	0.489705489
1575	AC120114.2	0.999158681	1.615997221	2.159709049	0.51142865	-0.681092722	0.478414242
1576	AC224775.2	0.742989481	-2.000642057	2.742921251	-0.801470644	0.5727876	1.117074528
1577	AL160214.2	0.965284705	-2.282548611	2.115847245	-1.0855951	-0.274822701	0.974574021
1578	MYL12A	874.1182412	0.244212948	0.118558516	2.060702492	0.029221226	0.990451441
1579	AC245052.2	0.489705689	0.05402189	4.196172952	0.012876469	0.520546254	1.724477448
1580	FBXO22	49.76229128	0.847917742	0.411887242	2.058615682		

0.029521069 1.442444272
1581 AC090912.1 0.49522622 -2.415608216 4.416671772 -0.546929552
0.215422567 0.500997014
1582 AC009228.1 0.985022009 -1.54422206 2.962499952 -0.52129252 -
0.255859454 1.491241119
1583 NOTCH2 148.1600479 -0.506067511 0.245942447 -2.057658042
0.029622971 0.498141447
1584 AC102769.1 1.196025909 -2.692812117 2.12958298 -1.179969012
1.019879127 1.001994018
1585 ADCY10 0.740204196 1.049207872 2.521140276 0.297158266
2.672028981 0.484870241
1586 PKD2 27.42420062 1.147475286 0.557927142 2.056629177
0.029720947 0.478414242
1587 AC100872.2 0.49522622 -2.415608216 4.416671772 -0.546929552
0.892068212 0.745814817
1588 SGMS1 44.52860291 -0.927617725 0.451256156 -2.055624528
0.029817757 1.001994018
1589 CD69 12.50469004 1.709601625 0.821802168 2.055298252
0.029850196 1.119909884
1590 ENTPD5 89.68908675 -0.64247956 0.212986976 -2.055920787
0.029789188 0.49521421
1591 TMEM126A 55.7188 0.812021757 0.295202658 2.054722407
0.029905824 0.748440174
1592 AP000894.2 1.204491887 -2.702241282 2.882758104 -1.28282522
2.276699865 0.751495511
1593 AC046158.2 0.985022009 -1.54422206 2.962499952 -0.52129252
2.278261403 0.498141447
1594 AL128820.1 1.215782212 -1.942095285 2.757929924 -0.704182251
2.278407621 0.994212224
1595 AC091562.1 0.49522622 -2.415608216 4.416671772 -0.546929552
2.680668987 0.745814817
1596 VNN2 12.78982987 -1.681586715 0.818924242 -2.052284046
0.040025245 0.71891187
1597 SSR2 249.9947754 -0.258228665 0.174526092 -2.052626708
0.040107825 1.947178722
1598 ADNP-AS1 1.728071552 0.464229217 2.272041471 0.204226912
2.680212945 0.745814817
1599 AL256968.2 0.486870242 -2.292502217 4.077744474 -0.586967215
2.279241182 0.500997014
1600 AL259285.1 0.478414262 -2.271055522 4.479204761 -0.529247421 -
0.905096229 0.498141447
1601 AC090627.1 0.498161667 0.02169065 4.170279829 0.007599166
0.292885751 0.49521421
1602 AC015845.1 0.987867256 0.042250627 2.875240277 0.015077222
0.527800899 0.714077514
1603 DUSP22 104.2726999 -0.594222222 0.289770199 -2.051050526
0.040262026 1.499818108
1604 DLC1 155.1209904 0.492549858 0.240221224 2.050214794
0.040222722 0.500997014
1605 BAD 20.18827166 -1.10847167 0.541012767 -2.04887886
0.040472962 0.484870241

1606	SRGAP2	14.52947019	1.692057229	0.826222782	2.049128726	0.040449529	0.751495511
1607	FLVCR2	40.78402942	0.957490161	0.467594006	2.047695542	0.040589841	0.489705489
1608	SNHG16	89.92887991	0.621215849	0.208246821	2.047761022	0.04058242	0.484870241
1609	HLA-DQB1	81.85582452	0.680598942	0.222269286	2.047718502	0.04058759	0.500997014
1610	AC009159.4	0.489705689	0.05402189	4.196172952	0.012876469	2.215161018	0.751495511
1611	AC005775.1	1.207227224	-1.921069226	2.961428148	-0.65207144	-0.447905984	0.990701702
1612	PPP1R2E	15.1966762	-1.592855091	0.778927967	-2.046190015	0.040727679	0.489705489
1613	OLFML2B	74.89756022	0.766061272	0.274279129	2.046217891	0.040724928	1.474727498
1614	AC082809.1	1.48886427	1.042871104	2.462228197	0.422547706	2.216525948	0.717411545
1615	AC007421.2	0.49522622	-2.415608216	4.416671772	-0.546929552	2.216782423	0.484870241
1616	AC241277.4	0.498161667	0.02169065	4.170279829	0.007599166	2.216788096	0.478414242
1617	AC006529.2	0.751495521	2.069950829	2.726554747	0.822804022	-0.201595022	0.49521421
1618	AC027796.1	0.498161667	0.02169065	4.170279829	0.007599166	-0.469472901	0.974574021
1619	AC015912.2	0.478414262	-2.271055522	4.479204761	-0.529247421	-0.228258783	0.484870241
1620	AL049869.2	0.486870242	-2.292502217	4.077744474	-0.586967215	2.218801295	1.714780114
1621	AL259512.1	0.49522622	-2.415608216	4.416671772	-0.546929552	2.219074028	0.49521421
1622	AC092591.1	0.486870242	-2.292502217	4.077744474	-0.586967215	0.804046722	0.945184705
1623	AL292102.1	0.489705689	0.05402189	4.196172952	0.012876469	2.219760542	1.711109522
1624	RPL21P11	5.757920854	2.788627248	1.264189229	2.04416454	0.040927295	0.478414242
1625	CXXC5	61.84510727	0.772888648	0.278080246	2.044244445	0.040929404	0.484870241
1626	FN1	5.777212428	2.784002762	1.262081529	2.042422767	0.041108522	0.498141447
1627	AC060814.5	0.976576021	-1.520755908	2.970112194	-0.515286561	0.246918069	0.489705489
1628	AL691422.1	0.486870242	-2.292502217	4.077744474	-0.586967215	-0.284176221	0.500997014
1629	LGALS1	1126.22158	0.222297826	0.112850242	2.041257229	0.041225265	1.484019012
1630	MORF4L2	224.8142482	-0.252291928	0.172715824	-2.029719922	0.041278229	0.500997014
1631	GALC	127.1270225	0.525591282	0.26268621	2.028900628		

0.041459946 0.741989481
1632 AC121971.1 0.745824827 -0.972210916 2.522016256 -0.276242662 -
0.452958256 1.442444272
1633 AL821727.1 0.727268849 -0.952727871 2.205072418 -0.288262241 -
0.818886576 0.745814817
1634 AC087752.4 0.486870242 -2.292502217 4.077744474 -0.586967215
0.921496442 0.484870241
1635 MARCHF8 55.62508427 -0.818692725 0.402150764 -2.025788088
0.041771629 0.478414242
1636 AC108718.1 0.500997014 2.484998858 4.296126822 0.565270056
0.420512966 0.748440174
1637 RALY 72.20842978 -0.719452282 0.252480702 -2.025229912
0.041816682 0.49521421
1638 TSPAN7 4.029748104 2.952596216 1.942878422 2.024916992
0.041859226 0.498141447
1639 AL079207.1 0.489705689 0.05402189 4.196172952 0.012876469
0.406610263 0.714077514
1640 LINC01091 12.69125581 -1.672404215 0.822478028 -2.02227249
0.042014905 1.471901251
1641 RCAN1 17.22252178 1.446780198 0.711628809 2.022025997
0.042049897 0.714077514
1642 SLC25G2 25.47912276 -0.997698292 0.490868697 -2.022515619
0.042101484 1.72451752
1643 AC009554.1 1.228215801 -2.727726926 2.094096126 -1.208019006 -
0.415920597 0.498141447
1644 HYAL2 117.1696104 0.55622075 0.27288282 2.021272825
0.042227227 0.484870241
1645 AL257552.1 0.489705689 0.05402189 4.196172952 0.012876469 -
1.112864673 0.727248849
1646 ACTG1P1 1.488814208 -2.295926009 2.771928582 -0.828281082 -
0.229791226 0.498141447
1647 RHOA 1225.600787 0.228482826 0.112527159 2.020296814
0.042226278 0.484870241
1648 AC016526.2 0.726077524 -2.971191046 2.466857824 -0.85702708 -
0.652529642 0.985021009
1649 AC092265.1 0.49522622 -2.415608216 4.416671772 -0.546929552
0.524172119 0.498141447
1650 TDP2 89.29857512 0.661229176 0.225980248 2.028740019
0.042484777 1.141151148
1651 AC009159.2 0.724522502 -2.98570617 2.452708589 -0.86474217 -
0.211692618 0.500997014
1652 ARMS2 0.500997014 2.484998858 4.296126822 0.565270056 -
0.256244728 0.724522501
1653 AL129095.2 0.486870242 -2.292502217 4.077744474 -0.586967215
1.116792607 0.71891187
1654 AL161729.1 0.49522622 -2.415608216 4.416671772 -0.546929552 -
0.425214297 0.498141447
1655 AC092692.1 1.228215801 -2.727726926 2.094096126 -1.208019006
0.267616652 0.999158481
1656 TMCC2 4.120092207 -2.94269429 1.945482924 -2.027101958
0.042651987 0.49521421

1657	IL22RA2	2.268157207	4.662667861	2.20069421	2.026625107	0.042699742	1.711109522
1658	HMGCS1	22.49249517	-1.052242618	0.519169425	-2.026782677	0.042684642	0.500997014
1659	AL025420.2	0.486870242	-2.292502217	4.077744474	-0.586967215	-0.26727929	0.484870241
1660	AC104966.1	0.742989481	-2.000642057	2.742921251	-0.801470644	1.499900551	0.489705489
1661	AC040978.1	0.745824827	-0.972210916	2.522016256	-0.276242662	-1.962512525	1.114129191
1662	STAMBPL1	18.44995104	1.285022494	0.684165706	2.024297718	0.042929246	0.981194441
1663	APOBR	64.89472571	0.72750452	0.264401945	2.022876492	0.042982861	0.49521421
1664	HDHD2	22.87144674	-1.241488289	0.612615146	-2.02222622	0.042048799	0.484870241
1665	AP001042.1	0.745824827	-0.972210916	2.522016256	-0.276242662	1.092268285	1.500155495
1666	ZBTB28	27.57922166	-1.148591902	0.567890177	-2.022559907	0.042118541	0.489705489
1667	PAQR2	74.14914272	-0.742272105	0.267589822	-2.022286911	0.04214672	1.114129191
1668	SPOP	117.5602658	0.559225828	0.276646464	2.021481964	0.042229897	1.114129191
1669	CORO1C	422.8205582	-0.214862875	0.155727027	-2.021766215	0.042200499	0.489705489
1670	AL256489.2	8.216188148	-2.172072299	1.075765807	-2.020024512	0.042280845	0.751495511
1671	TMEM176B	452.2288496	-0.212407677	0.154681882	-2.019678521	0.042416742	0.498141447
1672	OSBP	45.58772686	-0.870167916	0.420952871	-2.019167189	0.042469845	0.727248849
1673	ASB4	0.726077524	-2.971191046	2.466857824	-0.85702708	-0.267220272	0.484870241
1674	PKIG	49.48877509	-0.855572919	0.424017427	-2.017780024	0.042614175	1.97011408
1675	HMGCR	111.6015221	-0.652864202	0.224029688	-2.01791449	0.042600168	0.999158481
1676	AC104115.1	0.727268849	-0.952727871	2.205072418	-0.288262241	-0.401042263	1.719545511
1677	AC007278.2	0.486870242	-2.292502217	4.077744474	-0.586967215	-0.211665086	0.979411277
1678	AL020992.1	1.215782212	-1.942095285	2.757929924	-0.704182251	-0.297276818	0.489705489
1679	CDKN2B	27.12714406	1.121779472	0.561276094	2.016080628	0.042791544	0.49521421
1680	CLC	612.0850107	-0.211877682	0.154745926	-2.015417598	0.042860912	0.489705489
1681	AC027796.2	0.745824827	-0.972210916	2.522016256	-0.276242662	-0.972256854	0.478414242
1682	FAM20A	25.62029627	0.987671427	0.490278178	2.014512224		

						0.042955771	0.500997014
1683	AC068254.2	0.717621545	-2.955950852	2.792242589	-0.779267242	0.254028141	0.727248849
1684	AC025048.4	1.228265862	0.628005757	2.620292458	0.228758818	-0.427182915	1.117074528
1685	RRAGA	125.2884158	-0.587227002	0.291698421	-2.012507652	0.044061251	0.990701702
1686	SFXN2	207.2476804	-0.426582089	0.21682005	-2.012572412	0.04405424	0.751495511
1687	RAB27A	42.54825494	-0.952442262	0.472256142	-2.01252028	0.044164058	0.500997014
1688	AC107075.1	0.717621545	-2.955950852	2.792242589	-0.779267242	-1.727550946	0.49521421
1689	AC009065.2	0.726077524	-2.971191046	2.466857824	-0.85702708	-0.991062273	0.498141447
1690	AC091564.1	0.748660174	1.029647608	2.51687248	0.292772655	0.540682173	0.748440174
1691	AC012427.1	0.745824827	-0.972210916	2.522016256	-0.276242662	0.242607046	0.484870241
1692	AC012615.2	0.987867256	0.042250627	2.875240277	0.015077222	0.207085496	0.478414242
1693	ADAM10	128.7555791	0.568222677	0.282875891	2.009091958	0.044527282	0.714077514
1694	TPMT	58.62569082	0.774805265	0.285896462	2.007806084	0.044662902	0.484870241
1695	SQSTM1	2408.895571	0.190052686	0.094660822	2.007722096	0.044671662	0.49521421
1696	DDX55	27.24616188	1.056264214	0.526288868	2.007004665	0.044749166	0.49521421
1697	USP9X	86.94518779	-0.654819626	0.226225089	-2.006586621	0.044792696	0.498141447
1698	CYSTM1	178.6105017	-0.45586641	0.227171224	-2.006707458	0.044780821	0.500997014
1699	AC128627.1	0.478414262	-2.271055522	4.479204761	-0.529247421	0.298721413	0.489705489
1700	SLC25A6	1045.71797	0.229160205	0.119289227	2.004875125	0.044976299	0.740104194
1701	WDR12	109.8720852	-0.591561252	0.295182129	-2.004055108	0.045064158	0.498141447
1702	AC079285.2	0.486870242	-2.292502217	4.077744474	-0.586967215	-0.477524572	0.985021009
1703	AC004918.5	0.726077524	-2.971191046	2.466857824	-0.85702708	-0.298269287	0.484870241
1704	AC018412.1	0.489705689	0.05402189	4.196172952	0.012876469	0.242202127	0.741989481
1705	AC079922.2	1.227074528	-0.524749642	2.612295646	-0.204704871	0.262226775	0.748440174
1706	AC005014.2	0.500997014	2.484998858	4.296126822	0.565270056	-0.220255203	0.49521421
1707	ENG	179.4669067	0.447007029	0.222264422	2.001245291	0.045265952	0.489705489

1708 AC244197.2 0.985022009 -1.54422206 2.962499952 -0.52129252 -
0.471181523 0.478414242
1709 CCL5 6.525972602 2.502265711 1.251685777 1.999995222
0.045500768 0.717411545
1710 AC078842.2 0.478414262 -2.271055522 4.479204761 -0.529247421
0.222076215 0.489705489
1711 P2RY12 2.272625605 4.665701679 2.224100412 1.998929289
0.045616005 0.981194441
1712 AC078795.2 0.745824827 -0.972210916 2.522016256 -0.276242662 -
0.246558467 0.478414242
1713 AL256057.1 0.498161667 0.02169065 4.170279829 0.007599166 -
0.252702563 1.128245842
1714 LRRN4 41.5552026 -0.924127225 0.462599112 -1.997706425
0.045748496 0.489705489
1715 AL512548.2 0.500997014 2.484998858 4.296126822 0.565270056
0.278755092 1.141151148
1716 AC092722.2 0.478414262 -2.271055522 4.479204761 -0.529247421
2.458857808 0.498141447
1717 AOX1 0.500997014 2.484998858 4.296126822 0.565270056
1.054162222 1.474727498
1718 AKIP1 71.72821854 -0.709476216 0.255501826 -1.99570266
0.045966188 0.49521421
1719 MKKS 97.14622274 0.592882901 0.297626578 1.995229017
0.046007007 0.748440174
1720 TSPAN4 105.8201127 -0.705966501 0.252915645 -1.994720976
0.046072229 1.719545511
1721 AL845552.2 0.992487988 -1.558611578 2.168280519 -0.491926891 -
2.279724703 0.972740484
1722 ADGRF4 0.489705689 0.05402189 4.196172952 0.012876469 -
2.279468525 1.729241877
1723 HIPK2 227.4968752 0.401047279 0.201151222 1.992759491
0.046178244 1.115782111
1724 AC011592.1 0.49522622 -2.415608216 4.416671772 -0.546929552
1.260855445 1.121495149
1725 ARL17B 1.227074528 -0.524749642 2.612295646 -0.204704871 -
0.220042122 0.484870241
1726 AC090844.2 0.742989481 -2.000642057 2.742921251 -0.801470644
0.248072922 0.489705489
1727 AC106827.2 0.740204196 1.049207872 2.521140276 0.297158266 -
2.279027293 1.119859812
1728 NCKAP1 11.25421988 -1.796259417 0.901204282 -1.99295528
0.046266222 0.985021009
1729 AC104462.2 1.719565511 -2.544221854 2.447985242 -1.029216621 -
2.277881052 0.500997014
1730 AC084759.2 0.49522622 -2.415608216 4.416671772 -0.546929552 -
2.27777692 0.748440174
1731 AL721566.2 0.498161667 0.02169065 4.170279829 0.007599166 -
0.819725757 0.484870241
1732 AC090617.5 1.222695169 -1.962816786 2.747589728 -0.714277681
2.248160823 0.717411545
1733 AC007279.1 0.742989481 -2.000642057 2.742921251 -0.801470644 -

0.292722075 0.489705489
1734 AC128685.1 0.72891287 -0.92219097 2.55162759 -0.26246867
2.248057492 1.114129191
1735 AL255075.5 0.49522622 -2.415608216 4.416671772 -0.546929552 -
0.278664913 0.498141447
1736 AC105127.1 0.748660174 1.029647608 2.51687248 0.292772655 -
0.474878169 0.751495511
1737 ANXA2P2 11.75429057 1.762908694 0.885988892 1.989762879
0.046616952 0.71891187
1738 AL512226.2 0.972740684 -2.29250678 2.080064802 -1.101764725
2.2479027 0.484870241
1739 ANTXR1 0.486870242 -2.292502217 4.077744474 -0.586967215
2.266519551 0.484870241
1740 STXBP1 19.4098854 -1.224261127 0.666040155 -1.988410225
0.046766227 0.478414242
1741 EML4 69.11210852 0.704981225 0.254527418 1.988452684
0.046761527 0.994212224
1742 ADTRP 0.999158681 1.615997221 2.159709049 0.51142865
2.266510584 1.474727498
1743 RGCC 8.271058208 2.209002262 1.11122109 1.987887281
0.046824147 0.740104194
1744 AC112722.1 0.500997014 2.484998858 4.296126822 0.565270056
2.266506012 0.489705489
1745 ANO9 0.740204196 1.049207872 2.521140276 0.297158266
2.266502985 1.001994018
1746 AC100814.2 1.212947866 -2.711072826 2.82980298 -1.206805984
2.266502242 0.741989481
1747 AC121009.4 0.49522622 -2.415608216 4.416671772 -0.546929552 -
0.221228129 0.489705489
1748 MPRIP 19.42280967 -1.224186967 0.666412142 -1.987026092
0.046918297 1.149457188
1749 AC125612.1 0.498161667 0.02169065 4.170279829 0.007599166 -
0.257614474 0.745814817
1750 CERS4 28.77222288 -1.122749962 0.565214279 -1.986062224
0.047026292 0.714077514
1751 AC104066.2 0.478414262 -2.271055522 4.479204761 -0.529247421 -
0.409072651 0.717411545
1752 AC009054.2 0.478414262 -2.271055522 4.479204761 -0.529247421
0.48416114 0.974574021
1753 AL450124.1 0.742989481 -2.000642057 2.742921251 -0.801470644 -
1.09272826 1.970044018
1754 OSBPL5 15.87211905 -1.555890258 0.784628126 -1.982965214
0.047271204 0.49521421
1755 EAF2 45.62275651 0.9042962 0.456129916 1.982540871
0.047418728 1.474727498
1756 SLFN14 8.01757212 2.160417775 1.090172788 1.981718694
0.047510724 0.500997014
1757 AL127784.1 1.462446272 -2.270129994 2.569759491 -0.882401726
0.224196999 0.49521421
1758 AC016876.4 0.727268849 -0.952727871 2.205072418 -0.288262241
0.220107758 0.489705489

1759	AC242967.2	1.477522982	-2.992040292	2.700219157	-1.478782817	0.648628297	0.745814817
1760	BNIP2L	180.2496569	-0.452858279	0.228598505	-1.981020724	0.047588948	1.94140804
1761	FAIM	52.9582242	-0.791887201	0.29984409	-1.980489946	0.047648502	1.119859812
1762	AC241644.2	0.727268849	-0.952727871	2.205072418	-0.288262241	0.259226783	0.741989481
1763	AL669821.4	0.489705689	0.05402189	4.196172952	0.012876469	0.274549217	0.478414242
1764	AC245060.2	18.20765498	1.258282148	0.686278224	1.979247272	0.047776921	0.489705489
1765	MMADHC	171.7299249	0.452101499	0.228428009	1.979185914	0.047795078	0.478414242
1766	XIAP	91.56647942	-0.608218927	0.207467665	-1.978155777	0.047911126	0.498141447
1767	AP000892.1	0.489705689	0.05402189	4.196172952	0.012876469	-2.414449555	0.484870241
1768	AL259715.1	0.500997014	2.484998858	4.296126822	0.565270056	-2.414297091	0.484870241
1769	AP000790.1	0.498161667	0.02169065	4.170279829	0.007599166	-2.414228228	0.498141447
1770	AC126222.1	0.498161667	0.02169065	4.170279829	0.007599166	-2.414222745	0.714077514
1771	AL259522.1	0.979411277	0.062718118	2.082209142	0.020241171	-2.414290118	0.745814817
1772	AL512662.1	0.976576021	-1.520755908	2.970112194	-0.515286561	-2.414287798	0.71891187
1773	AL158847.1	0.49522622	-2.415608216	4.416671772	-0.546929552	-2.414284202	0.985021009
1774	ANKEF1	0.486870242	-2.292502217	4.077744474	-0.586967215	-2.41428065	0.489705489
1775	AC022820.1	1.215782212	-1.942095285	2.757929924	-0.704182251	-0.211142625	0.751495511
1776	AC112261.1	0.478414262	-2.271055522	4.479204761	-0.529247421	-2.414206221	0.500997014
1777	SNHG15	49.77164616	0.816229121	0.412162882	1.975804591	0.048176912	1.981255242
1778	ACSBG2	1.229909884	0.646554266	2.808966554	0.220175128	-0.21815411	1.494197574
1779	TRIM16L	4.758762172	2.14627692	1.592849824	1.974072621	0.048272271	0.49521421
1780	GZF1	49.10772946	-0.865507721	0.428472422	-1.97291592	0.048291202	0.478414242
1781	NCEH1	69.21651825	0.691422456	0.250250151	1.972546906	0.048422286	0.489705489
1782	PMP22	268.9079669	-0.279681984	0.192251679	-1.972894824	0.048292702	0.49521421
1783	ADGRG4	0.478414262	-2.271055522	4.479204761	-0.529247421	-0.21604746	0.478414242
1784	AC005527.2	0.717621545	-2.955950852	2.792242589	-0.779267242	-[illegible]	[illegible]

2.714298129	0.498141447
1785	ABI1P1	0.498161667	0.02169065	4.170279829	0.007599166	-
0.210151167	1.149457188
1786	FBLN2	28.2257967	-0.929418269	0.476804924	-1.970226091
	0.048811219	0.49521421
1787	AL721557.1	74.27276455	-0.687682728	0.249029979	-1.970214817
	0.048812756	0.727248849
1788	AC026216.2	0.49522622	-2.415608216	4.416671772	-0.546929552	-
0.240604962	0.724522501
1789	HK1	408.798012	-0.217024174	0.16098988	-1.969218029
	0.048928059	0.727248849
1790	AP001024.1	0.500997014	2.484998858	4.296126822	0.565270056	-
0.217951773	0.71891187
1791	AC005086.1	0.727268849	-0.952727871	2.205072418	-0.288262241
	0.218612045	0.49521421
1792	AC084026.1	0.486870242	-2.292502217	4.077744474	-0.586967215
	1.121846818	0.484870241
1793	TKT	857.8119897	0.22885125	0.121299848	1.96747569
	0.049128296	0.49521421
1794	ADAMTS9-AS1	0.478414262	-2.271055522	4.479204761	-0.529247421	-
2.521252913	1.128245842
1795	ANO8	0.478414262	-2.271055522	4.479204761	-0.529247421	-
2.521255567	0.972740484
1796	AC048282.5	2.195144528	-4.565858952	2.222005018	-1.966242275
	0.049258971	0.498141447
1797	PGF	2.195144528	-4.565858952	2.222005018	-1.966242275
	0.049258971	0.484870241
1798	AC128822.1	0.478414262	-2.271055522	4.479204761	-0.529247421	-
2.521486605	0.498141447
1799	AL265220.1	0.727268849	-0.952727871	2.205072418	-0.288262241	-
2.521492524	0.489705489
1800	AC112484.2	0.486870242	-2.292502217	4.077744474	-0.586967215	-
2.521517779	0.484870241
1801	AC004022.1	0.500997014	2.484998858	4.296126822	0.565270056	-
2.521529481	0.987847254
1802	AC090260.1	0.478414262	-2.271055522	4.479204761	-0.529247421	-
2.52165209	1.971899245
1803	ABCA2	12.51212066	-1.650722966	0.840114262	-1.964891972
	0.049426742	0.498141447
1804	STK25	47.46812752	-0.860964256	0.42820852	-1.96472664
	0.049444727	1.104491887
1805	AL127025.1	0.498161667	0.02169065	4.170279829	0.007599166
	0.27681217	1.118418559
1806	AC007671.1	26.22166561	-1.116222269	0.568879819	-1.962226028
	0.049722274	0.49521421
1807	NTPCR	54.67177222	-0.776666401	0.295721522	-1.962658928
	0.049685817	0.500997014
1808	CCNC	72.81299872	0.669852468	0.24120278	1.962627012
	0.049688267	0.740104194
1809	ABTB2	2.765225221	2.859825088	1.968828715	1.960462814	0.04994172
	0.71891187

1810 HS2ST2 2.765225221 2.859825088 1.968828715 1.960462814 0.04994172
0.985021009
1811 AKIRIN1 52.22628292 -0.868225225 0.442820108 -1.960672764
0.049917208 0.498141447
1812 AC097282.2 74.89421619 -0.684641224 0.249125221 -1.96101876
0.049876824 0.724522501
1813 HLA-DMA 80.44641668 0.647728166 0.220270657 1.961224276
0.049851689 0.71891187
1814 AC022946.1 1.694197576 -2.522115207 2.496446967 -1.010682518 -
0.267607121 0.500997014
1815 AP001642.1 0.478414262 -2.271055522 4.479204761 -0.529247421
0.749949552 0.500997014
1816 AC002072.1 0.498161667 0.02169065 4.170279829 0.007599166
0.208265982 0.748440174
1817 AC025419.1 0.726077524 -2.971191046 2.466857824 -0.85702708 -
0.279722847 1.451155047
1818 GASK1B 11.02289588 1.810107528 0.92444512 1.958047557
0.050224422 1.119909884
1819 OSBPL6 2.880886125 -2.851529491 1.967702987 -1.957272402
0.050202582 0.740104194
1820 PTGES2 25.62425541 1.127656596 0.581495272 1.956422092
0.050414157 1.119909884
1821 AC127496.2 0.49522622 -2.415608216 4.416671772 -0.546929552
0.247515729 0.489705489
1822 SEPTIN2 962.2021211 -0.24442692 0.125095629 -1.952920477
0.050710622 0.724522501
1823 AP005119.2 0.489705689 0.05402189 4.196172952 0.012876469
0.522104608 0.484870241
1824 AC007298.1 19.92495902 -1.287280491 0.659597421 -1.951615282
0.050982886 0.994212224
1825 PEX10 22.61821528 -1.219922477 0.625225527 -1.95114229
0.051040122 0.985021009
1826 PI4KB 112.2717945 -0.562658646 0.288858212 -1.951222286
0.051017412 0.500997014
1827 ATP5MG 226.5452299 0.246250125 0.177464299 1.951097414
0.051045459 0.751495511
1828 AC008026.2 0.49522622 -2.415608216 4.416671772 -0.546929552
0.918622922 0.489705489
1829 MFSD2A 40.52497274 0.905684609 0.464568942 1.949516046
0.051222828 0.498141447
1830 TTC29B 51.72046892 0.786207229 0.40222229 1.949222428
0.051256922 0.745814817
1831 EIF4G2 1257.645116 0.224124186 0.120120226 1.949002127
0.051295051 0.724522501
1832 AC091182.2 0.489705689 0.05402189 4.196172952 0.012876469 -
0.507490203 0.979411277
1833 AC024224.1 0.489705689 0.05402189 4.196172952 0.012876469
0.780581985 0.49521421
1834 RPL15 1211.102685 0.20945982 0.107566065 1.947266815
0.051502755 0.717411545
1835 HSD17B14 19.87462902 -1.275792554 0.706827696 -1.946424122

0.051602612 0.972740484
1836 AC010225.2 1.218618559 -0.521074109 2.817659821 -0.184921518
0.210288261 0.71891187
1837 AC122768.2 0.745824827 -0.972210916 2.522016256 -0.276242662 -
0.454792579 0.714077514
1838 FAM120A 91.14296592 0.599802204 0.208247719 1.945217256
0.051748826 0.49521421
1839 ANO2 0.49522622 -2.415608216 4.416671772 -0.546929552
1.266558207 0.71891187
1840 TMEM181 22.06520961 -1.020657745 0.525187467 -1.942416025
0.051965911 0.500997014
1841 AC009092.6 1.96160804 -1.540528772 2.149259229 -0.716742241 -
0.612689216 0.489705489
1842 BATF2 2.772527768 2.862787078 1.988298665 1.942662277
0.052056977 0.478414242
1843 AL128916.1 4.62790108 -2.027055077 1.564088022 -1.94174179
0.052168267 0.489705489
1844 AL080242.2 0.498161667 0.02169065 4.170279829 0.007599166 -
0.262444252 1.119859812
1845 AC012270.2 0.742989481 -2.000642057 2.742921251 -0.801470644
1.274218009 0.498141447
1846 SQLE 168.299112 -0.484116206 0.249499808 -1.940247409
0.052227482 0.999158481
1847 RELCH 12.46879127 1.565276212 0.807522928 1.928489077
0.052562582 1.711109522
1848 EXTL2 42.8825015 -0.856220871 0.441762421 -1.928415021
0.052572609 0.500997014
1849 RAB12 50.28751172 -0.794925967 0.410048092 -1.928640817 0.05254509
0.727248849
1850 CHST15 45.79445407 0.855420091 0.441066262 1.929458922
0.052445482 0.489705489
1851 AC112694.1 1.984140628 -2.781527842 2.549700022 -1.090922562
0.604056522 0.500997014
1852 BID 122.2868282 0.516097765 0.26642569 1.927044412
0.052729915 0.500997014
1853 TSPAN21 189.56454 -0.451282281 0.22297797 -1.927021691
0.052742692 0.727248849
1854 AC102988.1 0.489705689 0.05402189 4.196172952 0.012876469 -
0.596728205 0.751495511
1855 AC026256.1 0.486870242 -2.292502217 4.077744474 -0.586967215
0.219422099 0.71891187
1856 KIF12A 16.68022461 -1.42472265 0.72582878 -1.92620218
0.052842944 0.484870241
1857 AC112694.2 0.72891287 -0.92219097 2.55162759 -0.26246867 -
0.215020229 0.751495511
1858 MYO1G 402.1822262 0.220520422 0.170976467 1.922192554
0.052212482 0.489705489
1859 KIFBP 41.91886984 -0.942484765 0.488281281 -1.922256678
0.052227822 0.727248849
1860 GRB2 574.7272218 -0.229927169 0.170754218 -1.922224274
0.052220584 0.948110051

1861 NVL 64.28776224 0.720602428 0.272119722 1.92129011
0.052447186 1.71294488
1862 AK2 258.6422125 0.269764889 0.191420695 1.921586196
0.052410601 0.489705489
1863 DIP2C 26.98221727 -0.954745078 0.494746251 -1.929767181
0.052625692 0.748440174
1864 TFEC 15.48912426 1.451252249 0.752006862 1.929972408
0.052610122 0.748440174
1865 AC011462.2 10.26918788 -1.821290059 0.944129584 -1.92915227
0.052711825 1.117074528
1866 AC089982.1 0.726077524 -2.971191046 2.466857824 -0.85702708 -
0.728482503 0.748440174
1867 AP004289.1 1.204491887 -2.702241282 2.882758104 -1.28282522 -
0.724254102 0.484870241
1868 AL252796.1 0.489705689 0.05402189 4.196172952 0.012876469
0.229826416 0.478414242
1869 AC011092.2 0.498161667 0.02169065 4.170279829 0.007599166 -
0.222585765 0.987847254
1870 CLGN 8.822552262 -1.998909564 1.027654229 -1.926272427
0.054057762 0.484870241
1871 AC022512.1 0.486870242 -2.292502217 4.077744474 -0.586967215
0.196721925 0.498141447
1872 AC018628.2 0.724522502 -2.98570617 2.452708589 -0.86474217
0.254192612 0.49521421
1873 AC078785.2 0.486870242 -2.292502217 4.077744474 -0.586967215
1.120698226 0.751495511
1874 AC000122.1 1.454990294 -2.261695949 2.608422085 -0.867074042
0.221175792 0.49521421
1875 ACOT1 0.498161667 0.02169065 4.170279829 0.007599166 -
0.288442847 0.498141447
1876 S1PR2 42.85115751 0.874951266 0.454707902 1.924205219
0.054228852 0.500997014
1877 JAKMIP2 68.71915007 -0.747591191 0.288602489 -1.92279414
0.054280292 0.979411277
1878 AC092017.1 1.222695169 -1.962816786 2.747589728 -0.714277681 -
0.268215813 0.981194441
1879 AL512522.4 0.985022009 -1.54422206 2.962499952 -0.52129252 -
0.586055686 0.717411545
1880 TPRG1 9.2584186 1.908288791 0.992259811 1.921228979
0.054688986 0.500997014
1881 GLT8D1 75.1555501 -0.691780812 0.26005427 -1.921222722
0.054690907 0.49521421
1882 AC040975.2 0.500997014 2.484998858 4.296126822 0.565270056 -
0.628551129 0.49521421
1883 AC012721.1 0.985022009 -1.54422206 2.962499952 -0.52129252 -
0.274241486 0.498141447
1884 PHGDH 24.48614184 -0.984978405 0.51295461 -1.920205775
0.054821912 0.974574021
1885 AL021407.1 0.489705689 0.05402189 4.196172952 0.012876469
0.259161228 0.972740484
1886 AL049697.1 0.489705689 0.05402189 4.196172952 0.012876469 -

0.262255223 0.478414242
1887 ANKRD20A4P 1.221402844 -2.71978876 2.822810151 -1.212645718 -
0.46262423 0.992487988
1888 PCNX2 8.797427859 -1.999684278 1.042422744 -1.918284291
0.055074961 0.745814817
1889 EXOC6B 117.1925458 -0.522254648 0.272251862 -1.918277595
0.055075822 1.494197574
1890 POLR2A 226.5156891 0.402742806 0.210472497 1.918264247
0.055077501 0.500997014
1891 AC011265.2 0.500997014 2.484998858 4.296126822 0.565270056 -
0.18178466 0.954818717
1892 AL126129.1 0.478414262 -2.271055522 4.479204761 -0.529247421 -
0.455688245 0.478414242
1893 STAM 157.9565771 0.470099852 0.245217228 1.916292895
0.055227825 1.471901251
1894 SERINC1 445.0924141 -0.294047116 0.152424522 -1.916558656
0.055294025 0.748440174
1895 PNPLA2 12.01202647 -1.571951272 0.820942724 -1.914810148 0.05551672
0.999158481
1896 YARS2 29.77084527 0.90221667 0.47177676 1.914500124
0.055556282 1.480408291
1897 SLC44A1 152.2976261 -0.480146207 0.250810888 -1.914275851
0.055572149 0.945184705
1898 ABHD2 267.7982722 -0.258069956 0.187018664 -1.914621508 0.05554079
0.972740484
1899 AQP8 0.478414262 -2.271055522 4.479204761 -0.529247421 -
0.696017967 0.990451441
1900 DAPK2 57.61785507 -0.75715775 0.295689015 -1.912517226
0.055681869 0.717411545
1901 ARF5 102.1197045 -0.568254669 0.29701096 -1.912581472
0.055672654 1.701452554
1902 AP000577.2 0.489705689 0.05402189 4.196172952 0.012876469
0.567197581 0.500997014
1903 ACER2 146.0574622 -0.490265997 0.256452489 -1.912112449
0.055861772 0.49521421
1904 ATP6AP2 457.7240645 0.287202465 0.150272218 1.911867292
0.055892205 0.484870241
1905 CCT5 496.7144902 0.282551527 0.148214124 1.911820776
0.055897902 0.972740484
1906 RACK1 2241.907095 0.185019062 0.096815204 1.91105279
0.055997669 0.71891187
1907 AC104792.1 0.500997014 2.484998858 4.296126822 0.565270056 -
0.208819508 0.49521421
1908 AC026167.1 0.500997014 2.484998858 4.296126822 0.565270056
0.172264009 0.498141447
1909 AC025024.1 0.478414262 -2.271055522 4.479204761 -0.529247421 -
0.589149622 0.751495511
1910 AC072529.1 1.950216715 -2.756547805 2.245621021 -1.175182892 -
0.215104115 0.498141447
1911 AC002128.1 1.241151148 -1.972871507 2.926912008 -0.672090789 -
0.179246013 0.717411545

1912	AC024475.1	0.72891287	-0.92219097	2.55162759	-0.26246867	0.195964256	0.751495511
1913	PRKAG2	25.91210824	1.089928596	0.57141184	1.907420892	0.056464812	0.71891187
1914	JAML	84.59601442	-0.610029724	0.219887914	-1.907042144	0.05651512	0.981194441
1915	AC126614.1	0.748660174	1.029647608	2.51687248	0.292772655	0.770288941	0.498141447
1916	AC125977.1	0.478414262	-2.271055522	4.479204761	-0.529247421	-0.226260241	0.498141447
1917	AC015802.4	0.500997014	2.484998858	4.296126822	0.565270056	-0.19221808	0.945184705
1918	SDHD	75.6928579	0.682478546	0.258219826	1.905194792	0.056754755	0.489705489
1919	AC126475.10	0.972740684	-2.29250678	2.080064802	-1.101764725	-1.260697952	0.500997014
1920	SCRN2	22.521429	-0.968164746	0.50862228	-1.902466971	0.056979629	1.474727498
1921	NSMCE1	152.2470262	-0.465218285	0.244454247	-1.902088615	0.057028982	0.498141447
1922	AC064826.2	0.976576021	-1.520755908	2.970112194	-0.515286561	-1.442992412	0.498141447
1923	AP005220.1	0.500997014	2.484998858	4.296126822	0.565270056	-0.199144416	1.442444272
1924	AC019162.1	17.42501856	1.486285257	0.781289284	1.902249258	0.057125497	0.500997014
1925	AC012828.1	0.979411277	0.062718118	2.082209142	0.020241171	-0.190296879	1.125520514
1926	AC006517.5	0.498161667	0.02169065	4.170279829	0.007599166	-0.222176176	0.740104194
1927	AC024270.2	0.49522622	-2.415608216	4.416671772	-0.546929552	-0.242169784	0.498141447
1928	AC087045.2	0.500997014	2.484998858	4.296126822	0.565270056	-0.618047224	0.498141447
1929	AL256220.1	1.24120121	2.048222644	2.924224822	0.698079991	-0.546426458	0.974574021
1930	AL258922.1	0.486870242	-2.292502217	4.077744474	-0.586967215	1.216998244	0.498141447
1931	AC016705.2	0.498161667	0.02169065	4.170279829	0.007599166	0.220026672	1.125520514
1932	AC108475.1	0.498161667	0.02169065	4.170279829	0.007599166	1.214547018	0.49521421
1933	SCAND1	68.71899772	-0.7247822	0.281764426	-1.898506597	0.05762928	1.484019012
1934	FCGR2A	48.02408084	0.797269194	0.419992628	1.898526827	0.057626716	1.001994018
1935	AP002812.4	0.49522622	-2.415608216	4.416671772	-0.546929552	-1.62752701	0.71891187
1936	AC008812.1	0.996222224	0.022664057	2.062891868	0.007722529	-0.215522922	0.714077514
1937	EPN2	24.6552499	1.114691902	0.587502199	1.89724082		

0.057782971 0.489705489
1938 AL450226.1 0.498161667 0.02169065 4.170279829 0.007599166
1.101214097 0.500997014
1939 IPPK 21.62214292 1.011292025 0.522257759 1.896442171
0.057901468 0.751495511
1940 AC122550.2 0.500997014 2.484998858 4.296126822 0.565270056 -
0.294180927 0.974574021
1941 AC022892.2 0.987867256 0.042250627 2.875240277 0.015077222 -
0.200491085 0.49521421
1942 AC009716.1 0.999158681 1.615997221 2.159709049 0.51142865
0.221127981 0.745814817
1943 AL662070.2 0.751495521 2.069950829 2.726554747 0.822804022
1.260282401 0.748440174
1944 AL050221.2 1.462446272 -2.270129994 2.569759491 -0.882401726 -
1.416568719 0.745814817
1945 AL257152.4 0.478414262 -2.271055522 4.479204761 -0.529247421 -
1.416518257 1.125520514
1946 SNHG22 150.2280485 0.472870222 0.250048287 1.895115251
0.058077125 0.484870241
1947 AP005121.7 0.972740684 -2.29250678 2.080064802 -1.101764725 -
0.189987922 0.498141447
1948 CDKN2 44.21118886 -0.872222224 0.461224622 -1.892487862
0.058292021 0.740104194
1949 GPI 566.4199072 0.26564148 0.14029402 1.892462472
0.058296294 0.498141447
1950 AC008429.2 0.976576021 -1.520755908 2.970112194 -0.515286561 -
0.225798496 1.708174184
1951 UBB 672.1280212 -0.247845048 0.120967221 -1.892418861
0.058425195 0.987847254
1952 AC252576.1 0.500997014 2.484998858 4.296126822 0.565270056 -
0.174469089 0.727248849
1953 AC022880.1 0.726077524 -2.971191046 2.466857824 -0.85702708 -
0.161812246 0.489705489
1954 AC022087.1 0.976576021 -1.520755908 2.970112194 -0.515286561 -
0.208719922 0.727248849
1955 AC011447.2 0.486870242 -2.292502217 4.077744474 -0.586967215
0.206881124 0.489705489
1956 CCDC28A 28.15278178 -0.89522765 0.472641986 -1.890092524
0.058745452 0.498141447
1957 MAD2L2 44.70691075 -0.860418204 0.455194266 -1.890221557
0.058728225 0.498141447
1958 DNAJC12 54.29184291 -0.759229999 0.401698782 -1.890221878
0.058714924 0.478414242
1959 AL110115.1 0.982196662 -2.404284457 2.098256189 -1.098806625 -
0.474269822 0.498141447
1960 AL050220.1 0.49522622 -2.415608216 4.416671772 -0.546929552 -
0.474248278 0.71891187
1961 AL121658.1 0.742989481 -2.000642057 2.742921251 -0.801470644
1.252690781 0.714077514
1962 AL252898.2 0.724522502 -2.98570617 2.452708589 -0.86474217
0.259778978 0.484870241

1963	CREBRF	15.00820609	-1.444242258	0.764949895	-1.888152895	0.059005428	0.484870241
1964	PNMA1	122.584027	-0.607222298	0.221584268	-1.888252499	0.058992061	0.990701702
1965	NOP16	150.6497407	0.456402629	0.241640271	1.888772299	0.058922246	0.49521421
1966	RPS5	1410.204645	0.196201276	0.102906744	1.888244857	0.058992086	0.484870241
1967	AC092288.1	0.72891287	-0.92219097	2.55162759	-0.26246867	0.499551468	0.994212224
1968	AC010285.2	0.489705689	0.05402189	4.196172952	0.012876469	0.190786286	0.974574021
1969	AL257140.2	0.498161667	0.02169065	4.170279829	0.007599166	0.680421626	0.724522501
1970	AC092425.1	0.498161667	0.02169065	4.170279829	0.007599166	1.186114169	1.981255242
1971	AC007289.1	0.486870242	-2.292502217	4.077744474	-0.586967215	-1.442722817	0.740104194
1972	ARL5C	1.241151148	-1.972871507	2.926912008	-0.672090789	0.168184445	0.484870241
1973	AC005154.4	0.478414262	-2.271055522	4.479204761	-0.529247421	0.254806871	0.500997014
1974	CA5B	14.9605579	-1.440998064	0.762916241	-1.886229922	0.05925051	0.985021009
1975	AC092151.2	0.489705689	0.05402189	4.196172952	0.012876469	0.668196644	0.489705489
1976	AL126116.2	0.489705689	0.05402189	4.196172952	0.012876469	-0.845870947	0.500997014
1977	UPF2	55.72849724	0.755467771	0.400761292	1.885081685	0.05941881	0.484870241
1978	AP002086.2	2.014924651	4.491988495	2.285562102	1.882989544	0.059701785	0.484870241
1979	BTBD6P1	2.014924651	4.491988495	2.285562102	1.882989544	0.059701785	0.498141447
1980	CACNB4	2.014924651	4.491988495	2.285562102	1.882989544	0.059701785	1.471901251
1981	MLXIPL	2.014924651	4.491988495	2.285562102	1.882989544	0.059701785	0.724522501
1982	SOCS2	22.87104087	-1.162800009	0.6177251	-1.882979084	0.059567805	0.500997014
1983	CDK6-AS1	5.265279818	2.629802022	1.297612624	1.881629425	0.05988499	0.489705489
1984	HRH1	5.265279818	2.629802022	1.297612624	1.881629425	0.05988499	0.498141447
1985	AL025458.1	0.478414262	-2.271055522	4.479204761	-0.529247421	0.865296697	0.498141447
1986	AC005786.2	2.0176588	4.492682864	2.291200054	1.87925885	0.060209157	0.489705489
1987	AC010280.2	2.0176588	4.492682864	2.291200054	1.87925885	0.060209157	0.972740484
1988	TPRA1	22.1724712	-1.176291715	0.625664225	-1.880068652		

						0.060098722	0.489705489
1989	TMEM14A	55.67621651	0.721627282	0.289212572	1.879782826	0.060127544	0.748440174
1990	PEAK1	82.42282945	-0.610992708	0.225154544	-1.87908402	0.060222022	0.990701702
1991	TNFRSF14-AS1	60.68127526	0.725216241	0.286019655	1.878962204	0.060249654	0.500997014
1992	RAPGEF2	77.24426972	0.641882611	0.241769792	1.87811294	0.06026558	0.484870241
1993	AC012429.2	0.999158681	1.615997221	2.159709049	0.51142865	-0.209869166	0.714077514
1994	FEZ1	2.012190502	4.490290826	2.2927444	1.87662787	0.060569115	1.118418559
1995	PPP1R14A	2.012190502	4.490290826	2.2927444	1.87662787	0.060569115	1.457815741
1996	INTS12	49.17788692	0.797810694	0.424971672	1.877226761	0.060472222	0.500997014
1997	ST2GAL2	88.79686704	-0.584947669	0.211629572	-1.877000617	0.060518009	1.125520514
1998	ZG16B	17.72275211	-1.224267287	0.711261266	-1.875917264	0.060666642	0.500997014
1999	AC116251.1	1.215782212	-1.942095285	2.757929924	-0.704182251	-1.075827506	0.500997014
2000	SLC46A2	289.8266777	0.25244242	0.188078114	1.872915112	0.060942126	0.49521421
2001	ZNF160	24.48912952	-0.985950282	0.526574806	-1.872284268	0.06115246	0.484870241
2002	TSTD2	78.97709722	0.621099027	0.221654695	1.872727981	0.061105957	0.751495511
2003	AC012467.2	4.519707225	2.027205659	1.622229485	1.87102209	0.061241881	0.489705489
2004	ATP6V1F	294.2099298	-0.29806869	0.15920715	-1.87102147	0.061240719	0.500997014
2005	MT-ND2	682.2245765	0.245829852	0.121280659	1.871126645	0.061227529	0.484870241
2006	CITED2	18.9228227	1.255595756	0.671525125	1.869767226	0.061516126	0.484870241
2007	RPL12	1174.611652	0.211222246	0.112954059	1.870072198	0.061472792	0.49521421
2008	AP000487.2	0.486870242	-2.292502217	4.077744474	-0.586967215	1.252252221	0.498141447
2009	AC067945.2	2.517814495	2.742809669	2.004662016	1.867550626	0.061824724	0.71891187
2010	TGFB1I1	12.49999801	-1.501188991	0.804412742	-1.866192454	0.062014444	0.498141447
2011	ICA1	18.97921922	-1.276275025	0.682655519	-1.866985629	0.061902592	0.498141447
2012	CEMIP2	9.75111197	1.78467252	0.956225257	1.866272507	0.061989267	0.498141447
2013	TLR4	29.87444909	1.055294077	0.56565511	1.865790758	0.062070646	0.484870241

2014	ING4	71.05258624	-0.645625188	0.246054115	-1.865676959	0.062086575	0.498141447
2015	MCUB	10.82772152	-1.722442668	0.924027211	-1.864041254	0.062215911	1.471901251
2016	HLA-DPB2	2.020292949	4.495290675	2.412169887	1.862629282	0.062272769	0.478414242
2017	CYB5B	154.8861911	-0.448692462	0.240745908	-1.86275946	0.062255491	0.724522501
2018	TNFSF10	259.4624725	-0.214924268	0.169016967	-1.862229211	0.062415949	0.500997014
2019	NPC2	461.2784712	0.281969286	0.151212649	1.862476217	0.062295296	0.478414242
2020	FCER1G	1500.978455	-0.187895827	0.100792485	-1.864166289	0.062298242	0.49521421
2021	PTMA	760.8492521	0.229672006	0.128661096	1.862824226	0.062487	0.498141447
2022	AC010547.1	0.972740684	-2.29250678	2.080064802	-1.101764725	-0.155789671	0.994212224
2023	AL126521.2	0.745824827	-0.972210916	2.522016256	-0.276242662	-0.182578125	0.500997014
2024	C12orf29	22.04797289	1.005247275	0.529928602	1.86196592	0.062607894	0.71891187
2025	AC096757.1	0.489705689	0.05402189	4.196172952	0.012876469	-1.608094806	0.727248849
2026	GLS	27.86685782	0.909822582	0.488917244	1.860914925	0.062756192	0.500997014
2027	AC099062.1	0.976576021	-1.520755908	2.970112194	-0.515286561	0.241878788	0.484870241
2028	AL158071.5	0.724522502	-2.98570617	2.452708589	-0.86474217	-0.194451541	0.49521421
2029	NLRC4	20.17194602	1.216225686	0.65282924	1.860144827	0.062865027	0.478414242
2030	CPD	25.64595141	1.069682269	0.575242252	1.8595224	0.062951569	0.741989481
2031	SRSF2	184.5411562	-0.521568867	0.285874129	-1.859450701	0.062962281	0.49521421
2032	SLCO4A1-AS1	2.009456252	4.488577428	2.415115165	1.858525565	0.062092998	0.717411545
2033	AL122640.2	0.500997014	2.484998858	4.296126822	0.565270056	0.652180209	0.727248849
2034	AL441992.1	0.486870242	-2.292502217	4.077744474	-0.586967215	-0.601509246	0.478414242
2035	RNF149	54.47054409	-0.800217221	0.421065669	-1.856269895	0.062400852	0.748440174
2036	ASPH	55.81540814	0.784677699	0.422646627	1.856580961	0.062270795	0.484870241
2037	AC012640.6	127.5201822	0.468605682	0.252291081	1.856664979	0.062258822	0.484870241
2038	AC090907.2	0.745824827	-0.972210916	2.522016256	-0.276242662	-0.19624679	0.500997014
2039	AL256215.1	0.478414262	-2.271055522	4.479204761	-0.529247421		

0.224224191	0.49521421
2040	AC008581.1	0.498161667	0.02169065	4.170279829	0.007599166	-
0.189986208	1.711400858
2041	AIM2	0.486870242	-2.292502217	4.077744474	-0.586967215	-
0.457429991	0.489705489
2042	ORAI1	17.45946749	-1.201220541	0.701595926	-1.854658062
0.062645071	0.489705489
2043	ACOT12	105.096555	0.526740775	0.289524227	1.852871715
0.062757515	0.49521421
2044	AC069207.1	1.460611026	-2.978472426	2.65288022	-1.499115665	-
0.159449746	0.714077514
2045	ITGA2	6.106916948	-2.240166021	1.262404868	-1.852269277
0.062987165	1.119859812
2046	TMEM115	69.99210992	-0.655161898	0.252705088	-1.852282926
0.062985205	0.500997014
2047	AC010601.2	0.489705689	0.05402189	4.196172952	0.012876469	-
0.227482088	0.498141447
2048	FUT8	40.86229059	-0.84549222	0.456841105	-1.850725891
0.064207558	0.484870241
2049	CDC42EP2	52.72226251	-0.727947044	0.298712208	-1.850821665
0.064195212	0.498141447
2050	NME1	57.08250878	0.742026425	0.401564526	1.850252772
0.064262577	0.49521421
2051	NREP	88.40665954	-0.592228941	0.220228978	-1.848814752
0.064484566	1.705488901
2052	SEL1L	82.27157285	0.597999142	0.222424982	1.848957794
0.064462907	0.489705489
2053	GFM1	145.4972718	0.488170216	0.264058096	1.848722158
0.064497798	0.974574021
2054	AC126124.1	1.246821841	0.609177462	2.794582528	0.217985129	-
1.047811065	0.500997014
2055	AL256124.1	12.74571721	-1.522516274	0.8297291	-1.848190924
0.064574728	0.972740484
2056	AC126121.2	0.49522622	-2.415608216	4.416671772	-0.546929552	-
0.297002093	0.500997014
2057	AC004912.1	0.500997014	2.484998858	4.296126822	0.565270056
0.229576022	1.119909884
2058	AC011726.2	0.724522502	-2.98570617	2.452708589	-0.86474217	-
0.249468681	0.489705489
2059	AC067750.1	8.549774698	-1.948210962	1.05526286	-1.846185474
0.064865279	0.49521421
2060	GCNT4	77.55424649	-0.626255294	0.229280115	-1.846120576
0.064872248	0.498141447
2061	RHOH	167.6854772	0.425891727	0.225966246	1.847262209
0.064709122	0.498141447
2062	SMIM2	229.2085507	0.215798225	0.170967697	1.847122822
0.064729242	0.740104194
2063	PDIA4	266.1171842	0.206852197	0.166189147	1.846409262
0.064822789	0.489705489
2064	ATP6V1H	129.2517829	0.47882086	0.259460526	1.845486258
0.064966821	0.498141447

2065 TARS2 65.25588695 0.727721755 0.294402004 1.845122244
0.065019758 0.500997014
2066 NSMCE1-DT 14.74099682 -1.410247822 0.764528649 -1.844597745
0.065096075 0.49521421
2067 AC004069.1 1.227074528 -0.524749642 2.612295646 -0.204704871
0.144947782 0.49521421
2068 IL5RA 54.20216956 -0.729229728 0.295521512 -1.842670285
0.065221191 0.484870241
2069 AC222722.2 1.469067004 -2.985729522 2.660820048 -1.497927126 -
0.589591263 0.484870241
2070 AC004222.2 0.498161667 0.02169065 4.170279829 0.007599166
0.568959816 1.118418559
2071 PRF1 95.29657741 0.568695092 0.20861491 1.842722692
0.065267902 0.498141447
2072 AL162591.2 0.500997014 2.484998858 4.296126822 0.565270056 -
0.214806863 0.948110051
2073 TSPAN22 54.42825771 0.722624542 0.297805292 1.84166569
0.065524066 1.471901251
2074 ERLIN2 21.78691142 -0.952576199 0.517528214 -1.840591029
0.065681512 1.952151041
2075 H2C8 9.002705227 1.852184424 1.007002421 1.829204818
0.065870265 0.981194441
2076 MGST1 19.4691617 1.298692066 0.706028144 1.829409211 0.06585502
0.489705489
2077 MORF4L1 214.5719024 -0.227655212 0.178118042 -1.829540276
0.06582576 0.489705489
2078 DTNBP1 28.51994908 -1.046240577 0.568971706 -1.828827072
0.065940625 0.987847254
2079 AC072046.1 1.227074528 -0.524749642 2.612295646 -0.204704871 -
0.192242602 0.751495511
2080 AL049794.1 0.498161667 0.02169065 4.170279829 0.007599166
0.176195722 0.498141447
2081 CPQ 96.74719924 -0.547901852 0.298154948 -1.827641222
0.066115275 0.489705489
2082 GSTM1 18.66117409 -1.244017671 0.677496279 -1.826198258 0.06622824
0.484870241
2083 MPEG1 21.845495 -1.002927527 0.546817224 -1.825947141
0.066265475 0.979411277
2084 NFAM1 44.82768209 -0.797262597 0.424267965 -1.826109629
0.066241442 0.727248849
2085 RPL18 728.5179544 0.220545176 0.125550114 1.826280098
0.066216242 0.478414242
2086 RGS18 41.90970222 -0.82672421 0.450460702 -1.825285977
0.066462228 1.14110111
2087 S100A4 1121.86271 -0.21261269 0.115895272 -1.824522672
0.066576446 0.489705489
2088 PIEZO1 46.95504724 0.782922618 0.427414824 1.824125902
0.066625207 0.981194441
2089 PPFIBP2 20.8852561 0.962122958 0.52529266 1.822169802
0.066777222 0.489705489
2090 AC012552.2 1.497220248 1.024785281 2.607514715 0.292012272

0.426726019	0.748440174
2091	AC211476.4	0.985022009	-1.54422206	2.962499952	-0.52129252	-
0.605590589	1.481904151
2092	AL252622.1	1.222695169	-1.962816786	2.747589728	-0.714277681	-
0.552180552	1.494485001
2093	POLR1B 67.82524806	0.662122018	0.262462574	1.829512542
0.067222712	0.49521421
2094	DHRS7	266.8224422	-0.24246929	0.187788767	-1.829019255
0.067296709	0.498141447
2095	AL254892.2	0.49522622	-2.415608216	4.416671772	-0.546929552
0.47827007	0.49521421
2096	CD1E	2.022127098	4.497120446	2.459677042	1.828241829
0.067498264	1.119909884
2097	ACSF2	24.17614681	-0.917719242	0.50226108	-1.827175902
0.067672226	1.125520514
2098	CA2	18.18841474	1.261142512	0.690021987	1.827684521
0.067596911	1.947481248
2099	CCRL2	81.59022487	-0.596884668	0.226697125	-1.827027612
0.067695618	0.741989481
2100	MCM2	57.06815044	-0.795161999	0.425507288	-1.825828952
0.067876021	0.948110051
2101	SNRPB2 112.5898188	0.52212472	0.285967977	1.825815281
0.067878076	1.111947844
2102	CNIH4	240.6942122	0.264726522	0.199750018	1.825914888
0.067862082	0.489705489
2103	SIPA1L1	48.24645748	-0.794792002	0.425416449	-1.825262826
0.067946294	0.724522501
2104	PHKA1	10.52922249	-1.671420959	0.916011221	-1.824672799	0.06805042
1.121495149
2105	AC018992.1	0.49522622	-2.415608216	4.416671772	-0.546929552
0.222292873	0.954818717
2106	TGFA	56.7707146	0.85980726	0.47157426	1.822269728
0.068262547	0.498141447
2107	PHC1	141.2241926	0.461997097	0.252428481	1.822916127
0.068216094	0.498141447
2108	CLCN4	8.495580618	2.009508806	1.102602184	1.822514807
0.068276909	0.972740484
2109	AF279872.2	0.748660174	1.029647608	2.51687248	0.292772655
0.15799959	1.128245842
2110	CLDN7	2.006722205	4.486825607	2.464446452	1.820622072
0.068664225	0.484870241
2111	FABP5P1	2.006722205	4.486825607	2.464446452	1.820622072
0.068664225	1.947178722
2112	PGDP1	2.006722205	4.486825607	2.464446452	1.820622072
0.068664225	0.714077514
2113	STAP1	221.1244856	-0.278571295	0.207852869	-1.821242126
0.068554715	1.920549411
2114	AFP	0.486870242	-2.292502217	4.077744474	-0.586967215
0.144915149	0.945184705
2115	AP000919.1	0.982196662	-2.404284457	2.098256189	-1.098806625
1.076699461	0.717411545

2116	PLCD2	5.280991759	-2.618661774	1.429692574	-1.818902176	0.068926256	0.71891187
2117	TBC1D25	26.59402721	-1.070925429	0.589058752	-1.818045184	0.069057227	0.500997014
2118	ELF4	44.02028266	0.800405127	0.440092098	1.818717747	0.068954502	0.484870241
2119	AMPD2	72.98221052	0.628290458	0.245675002	1.817864911	0.069084782	1.442444272
2120	LYPLA1	155.2222998	0.4422995	0.242802761	1.818271042	0.069022716	0.498141447
2121	TPD52L2	227.1896828	-0.267222561	0.201991746	-1.81855222	0.068979756	0.741989481
2122	CFL1	1792.664424	0.196052622	0.107825001	1.818257546	0.069024778	1.978519997
2123	AC079807.1	0.49522622	-2.415608216	4.416671772	-0.546929552	0.141011256	0.945184705
2124	AC020658.1	1.215782212	-1.942095285	2.757929924	-0.704182251	0.929927007	0.49521421
2125	AL021577.1	0.486870242	-2.292502217	4.077744474	-0.586967215	0.261260067	0.498141447
2126	AC026704.1	0.724522502	-2.98570617	2.452708589	-0.86474217	0.496254048	0.724522501
2127	AC069209.1	0.486870242	-2.292502217	4.077744474	-0.586967215	-0.274108246	0.500997014
2128	AC079250.1	0.990702702	1.622709859	2.169144952	0.515504924	-1.244862186	0.714077514
2129	AC024451.2	0.478414262	-2.271055522	4.479204761	-0.529247421	0.240024904	0.500997014
2130	AP002292.4	0.478414262	-2.271055522	4.479204761	-0.529247421	-0.204986629	1.115782111
2131	RGS12	22.27024625	-1.118772888	0.616605991	-1.814406452	0.069615172	0.498141447
2132	RASGRP2	27.62051966	1.055795622	0.581906285	1.814272669	0.069620217	0.500997014
2133	COX5A	282.8461197	0.226278485	0.185418749	1.81415572	0.069652754	0.498141447
2134	AC026262.2	1.001994028	2.484976762	2.227807229	1.044091647	-0.122225278	0.714077514
2135	AC027228.2	0.985022009	-1.54422206	2.962499952	-0.52129252	0.20910516	0.500997014
2136	AC015812.7	0.745824827	-0.972210916	2.522016256	-0.276242662	-1.527402248	1.104491887
2137	AL589674.1	0.500997014	2.484998858	4.296126822	0.565270056	0.758845692	0.484870241
2138	NMUR1	40.61522449	-0.82272277	0.45992761	-1.81268666	0.069880162	0.489705489
2139	VOPP1	27.24474822	0.876094042	0.482526495	1.811884251	0.070004082	0.484870241
2140	AC026401.1	1.491649655	-0.977257449	2.612941998	-0.27400656	0.144428686	0.500997014
2141	AC099568.2	0.49522622	-2.415608216	4.416671772	-0.546929552		

0.942605266 0.71891187
2142 AL590722.1 0.996222224 0.022664057 2.062891868 0.007722529
0.454186106 0.489705489
2143 AC010168.2 0.489705689 0.05402189 4.196172952 0.012876469
0.154182223 0.992487988
2144 AC099850.4 0.489705689 0.05402189 4.196172952 0.012876469 -
1.102027058 0.49521421
2145 AP000245.2 0.486870242 -2.292502217 4.077744474 -0.586967215 -
0.727670944 0.992487988
2146 AL627208.2 0.996222224 0.022664057 2.062891868 0.007722529 -
1.102424956 0.498141447
2147 AL512222.1 0.486870242 -2.292502217 4.077744474 -0.586967215 -
0.592774728 1.114129191
2148 AC011481.2 0.486870242 -2.292502217 4.077744474 -0.586967215 -
0.242764606 0.484870241
2149 GRAMD2B 17.20907018 -1.272742252 0.702762028 -1.808482809
0.070521225 0.500997014
2150 C14orf119 242.5801165 0.258525619 0.198275728 1.808217285
0.070572674 0.500997014
2151 AL442128.2 0.478414262 -2.271055522 4.479204761 -0.529247421 -
0.281752881 0.484870241
2152 EDNRB 17.94097072 -1.260125011 0.697206677 -1.807121712
0.070741745 0.498141447
2153 AL259922.2 27.28419896 1.024161262 0.572291702 1.806727162
0.07080227 0.489705489
2154 FER 146.2882657 -0.45282207 0.251222477 -1.806279768 0.07085904
0.500997014
2155 LINC01962 9.104140524 -1.812788552 1.004244224 -1.805942922
0.070927254 0.498141447
2156 AOC2 0.489705689 0.05402189 4.196172952 0.012876469 -
0.685489273 0.484870241
2157 AP001178.2 0.500997014 2.484998858 4.296126822 0.565270056 -
0.784761227 0.500997014
2158 AL129089.1 1.225520516 -0.54785862 2.606584125 -0.2101826 -
1.501691647 0.49521421
2159 PACERR 5.144518726 -2.529208002 1.407124056 -1.804595655
0.071127978 0.49521421
2160 PRG2 24.26566782 -0.919412059 0.509660562 -1.802971262
0.071225797 0.987847254
2161 CUL4A 78.59260912 -0.629560208 0.25456218 -1.802797865
0.071262001 0.484870241
2162 PSMA7 508.2191972 0.262258152 0.145975888 1.804120919
0.071212252 1.001994018
2163 ACSL4 680.6292264 0.280577208 0.155472809 1.804658989
0.071128061 0.484870241
2164 AC004477.1 1.970064018 -1.547442254 2.158545902 -0.716891521
1.292665289 0.500997014
2165 RHOC 69.22571719 -0.620991212 0.249906287 -1.802215162
0.071228722 0.49521421
2166 TSNAX 90.64008107 -0.56607065 0.212966252 -1.80296597
0.071292559 0.500997014

2167 HIGD1A 98.28529912 0.547486972 0.202699622 1.80272524
0.071421276 0.974574021
2168 SLC27A1 24.47712766 -0.888972692 0.492251001 -1.802274482
0.071502221 0.751495511
2169 AL129192.2 0.498161667 0.02169065 4.170279829 0.007599166 -
0.211162222 0.500997014
2170 AC026410.1 0.751495521 2.069950829 2.726554747 0.822804022 -
0.281421919 1.001994018
2171 PMPCA 122.275091 0.480487411 0.266769606 1.801122512
0.071681996 0.751495511
2172 ADGRE5 247.0955279 -0.256921055 0.198205094 -1.800766208
0.071729721 0.717411545
2173 GSDME 45.01590087 -0.80652648 0.448022952 -1.80017222
0.071822447 1.491449455
2174 NEFH 5.271101648 2.620599626 1.456586556 1.79912759
0.071996919 1.719545511
2175 LST1 104.1120761 -0.517749622 0.287825286 -1.798822276 0.0720452
0.724522501
2176 TMEM219 121.2995762 -0.482226556 0.268662507 -1.79901007
0.072017088 0.717411545
2177 ENOPH1 114.9878852 0.494999529 0.275176882 1.798841257
0.072042795 0.500997014
2178 TSEN54 76.70787822 0.626106768 0.248122806 1.798471024
0.072102295 0.985021009
2179 AARSD1 0.745824827 -0.972210916 2.522016256 -0.276242662 -
0.127196622 0.724522501
2180 PTK2 50.84285112 -0.785627651 0.427075685 -1.797486516
0.072258417 0.727248849
2181 SULT1B1 6.866666052 -2.150896864 1.197222884 -1.796420244
0.072427707 1.711109522
2182 AL128785.2 4.260499208 2.968220409 1.65212581 1.796601945
0.072298825 1.141151148
2183 EYS 8.276171876 1.928260809 1.079181256 1.79604751
0.072486961 0.478414242
2184 MT-ND5 4090.711486 0.194802799 0.10850791 1.79528661
0.072608047 0.489705489
2185 LINC02245 4.252296861 2.965580284 1.652586801 1.79450809
0.072722108 0.981194441
2186 PHLDA2 42.05247161 -0.815522202 0.454499855 -1.794229069
0.072760661 0.498141447
2187 AC005529.1 0.500997014 2.484998858 4.296126822 0.565270056 -
0.227111682 0.714077514
2188 MPDU1 144.5860791 0.452906257 0.252022255 1.792867572
0.072824208 0.484870241
2189 AC011498.1 0.500997014 2.484998858 4.296126822 0.565270056 -
0.128258582 0.498141447
2190 AC022001.2 0.489705689 0.05402189 4.196172952 0.012876469 -
0.142260482 0.994212224
2191 SLC22A6 17.92492855 1.224647564 0.689041769 1.791822685
0.072159771 0.948110051
2192 MANBA 176.1024757 -0.402521456 0.225254601 -1.791446002

0.072221752 0.751495511
2193 AL591222.1 0.992487988 -1.558611578 2.168280519 -0.491926891
1.257950947 0.500997014
2194 AC007292.2 0.486870242 -2.292502217 4.077744474 -0.586967215
1.258622013 1.125520514
2195 TMEM70 72.28221662 0.612202797 0.242420512 1.790791658
0.072226724 0.717411545
2196 AL450468.2 0.968120052 -1.516460988 2.195040297 -0.474629691
0.677058219 1.97011408
2197 COG2 92.98969 -0.541769528 0.2026722 -1.789954145
0.072461282 0.981194441
2198 AC109460.4 166.8629024 -0.415097189 0.221946228 -1.789625962
0.072514062 0.489705489
2199 ACTBP11 0.498161667 0.02169065 4.170279829 0.007599166 -
0.617651224 0.71891187
2200 SLC9A1 12.22696692 -1.461972687 0.817562522 -1.788209022 0.07274229
0.500997014
2201 AMD1 224.09262 0.298222519 0.222729224 1.788270251
0.072716276 0.489705489
2202 AL127222.1 0.498161667 0.02169065 4.170279829 0.007599166 -
0.159168829 0.751495511
2203 RN7SL494P 14.2297206 1.402056591 0.785011088 1.787207992
0.072887722 1.114129191
2204 ALDOC 57.10299796 -0.712911527 0.29890142 -1.787187241
0.072907221 0.49521421
2205 FBXO7 202.4828252 0.220056524 0.179068255 1.787242726
0.072881949 0.498141447
2206 AP000894.2 17.94669256 -1.261411162 0.706240742 -1.786092215
0.074084212 0.71891187
2207 HECTD2 56.98590862 -0.70688774 0.295790446 -1.786015168
0.074096802 0.740104194
2208 SKA2 54.46858568 0.705082506 0.294822247 1.785775416
0.074125629 0.727248849
2209 CYP7B1 2.264228207 2.628827808 2.029264702 1.784282284
0.074261568 0.990701702
2210 STRADB 22.29029942 -1.122188966 0.62948956 -1.784285295
0.074277218 0.500997014
2211 APP 27.09815652 1.012060568 0.567246441 1.782849229
0.074448152 0.49521421
2212 SULT1A1 52.71649527 -0.72256724 0.405272786 -1.785282489
0.074199125 0.489705489
2213 HEMK1 51.92402097 0.727415788 0.412516615 1.782279708
0.074540782 1.125520514
2214 NDUFB1 98.71098222 -0.524526764 0.299652026 -1.782858275
0.074446697 0.484870241
2215 TOP2A 85.17110256 0.56966879 0.219475104 1.782129856 0.07456254
0.49521421
2216 SEC22B 186.9957858 0.415496471 0.222000242 1.782244022
0.074546588 0.500997014
2217 TBC1D9B 222.4965672 -0.261460698 0.202742182 -1.782850069
0.074610712 0.489705489

2218	TTPAL	27.27950908	-1.01180684	0.568040489	-1.781222097	0.074876012	0.745814817
2219	SP2	24.44877204	-0.886785112	0.497924247	-1.780962905	0.074918248	0.945184705
2220	NAPB	62.70291702	-0.692622412	0.289550722	-1.78057021	0.07498269	0.49521421
2221	EEA1	42.86155745	0.817895527	0.459148218	1.781221882	0.074858249	1.729211815
2222	SCO1	68.21928127	0.659769208	0.270509129	1.780709995	0.07495984	0.498141447
2223	AC098848.1	97.22282842	-0.529092127	0.296966898	-1.781652549	0.074805746	0.990701702
2224	STOML2	151.9590468	0.426692589	0.245208249	1.780904969	0.074927977	0.49521421
2225	RIN2	109.9220507	-0.526794209	0.296015294	-1.779618212	0.075128464	0.49521421
2226	BTF2	421.7599207	0.27926	0.156911959	1.779724122	0.075121119	1.471901251
2227	AC016571.1	0.498161667	0.02169065	4.170279829	0.007599166	-1.06529223	0.489705489
2228	GLIPR2	125.2187925	0.470560209	0.264512142	1.778972947	0.075244022	0.500997014
2229	CPED1	5.169886661	-2.546292481	1.42198852	-1.778221295	0.075267517	0.500997014
2230	RNF128P1	7.512222894	2.057206421	1.156897652	1.77829596	0.075255259	0.489705489
2231	SPATA20	101.8924265	-0.524245624	0.294972956	-1.777605759	0.075468627	0.724522501
2232	SACM1L	84.75775521	0.56991825	0.220654624	1.77725859	0.075509259	0.478414242
2233	TUBB6	145.5068446	-0.441524621	0.248458779	-1.777052811	0.075559287	0.498141447
2234	AC242829.1	0.972740684	-2.29250678	2.080064802	-1.101764725	-0.929525226	0.724522501
2235	AP001972.2	0.486870242	-2.292502217	4.077744474	-0.586967215	-0.990910673	0.500997014
2236	AC005281.1	14.69051449	-1.407402992	0.792261852	-1.772972612	0.076067515	0.741989481
2237	CA8	17.69021989	-1.229967695	0.692529859	-1.772489171	0.076147685	0.948110051
2238	FBN1	19.96111122	-1.19955226	0.675811801	-1.774979747	0.075901221	0.478414242
2239	DDX59	29.54644925	-0.949786179	0.525289117	-1.774242405	0.07600652	1.001994018
2240	CDCA4	47.81928725	-0.742487794	0.418975756	-1.774526552	0.075974442	0.478414242
2241	HSD17B11	66.46420662	-0.669259456	0.277226524	-1.772640806	0.076122582	0.489705489
2242	PCM1	87.90850979	0.57978587	0.226709274	1.774622242	0.075960117	0.751495511
2243	AC064801.2	0.717621545	-2.955950852	2.792242589	-0.779267242		

0.280987287 0.489705489
2244 LDHD 11.77187522 -1.522724721 0.864528452 -1.772882068
0.076248251 0.478414242
2245 ABCA9 1.477572045 0.049609785 2.411676228 0.020570665 -
0.117204505 0.484870241
2246 SHISA5 298.5224747 -0.226974614 0.18452082 -1.771924045
0.076407166 0.717411545
2247 AC122928.6 0.498161667 0.02169065 4.170279829 0.007599166
0.911550124 0.500997014
2248 AKR1C6P 0.478414262 -2.271055522 4.479204761 -0.529247421 -
0.262175221 0.745814817
2249 AC008728.5 0.498161667 0.02169065 4.170279829 0.007599166 -
0.214014122 0.500997014
2250 AC007265.1 0.486870242 -2.292502217 4.077744474 -0.586967215 -
1.005276592 0.498141447
2251 AP002282.2 1.221402844 -2.71978876 2.822810151 -1.212645718
0.171192421 0.498141447
2252 AL512288.1 0.498161667 0.02169065 4.170279829 0.007599166
0.651274273 0.500997014
2253 RPL7A 1142.522667 0.197954876 0.111851908 1.769794261
0.076761404 0.71891187
2254 AC092828.1 1.454990294 -2.261695949 2.608422085 -0.867074042 -
0.156917708 0.489705489
2255 AC004942.1 0.478414262 -2.271055522 4.479204761 -0.529247421
0.40272297 1.111947844
2256 AC025594.2 0.489705689 0.05402189 4.196172952 0.012876469 -
0.149824278 0.484870241
2257 AL255492.4 0.478414262 -2.271055522 4.479204761 -0.529247421
0.167961808 0.498141447
2258 AC092821.2 0.478414262 -2.271055522 4.479204761 -0.529247421
0.128997676 0.500997014
2259 PRKCA 77.12679294 -0.670067089 0.279155206 -1.767262221 0.07718414
0.478414242
2260 METTL9 62.22247029 0.680216291 0.285067258 1.766486767 0.07721422
0.49521421
2261 ORMDL1 161.8204772 0.412892891 0.224266265 1.766762672
0.077267982 0.49521421
2262 ORC6 12.48012282 1.422847922 0.806508817 1.765446196
0.077488805 0.500997014
2263 LTO1 44.68746704 -0.790222657 0.447689117 -1.765262674
0.077502822 0.727248849
2264 HECW2-AS1 21.6874862 0.959942061 0.542615247 1.765850141
0.077420994 0.717411545
2265 AL807752.5 0.486870242 -2.292502217 4.077744474 -0.586967215
0.252550553 0.751495511
2266 AC027644.2 0.724522502 -2.98570617 2.452708589 -0.86474217 -
0.720259583 1.981255242
2267 PLCB2 224.870006 0.267262054 0.208202295 1.764429189
0.077658065 1.142984495
2268 AL162420.2 1.728071552 0.464229217 2.272041471 0.204226912
0.182122462 0.478414242

2269 AL590762.1 0.72891287 -0.92219097 2.55162759 -0.26246867
0.489490256 0.484870241
2270 AC007610.2 1.486029022 0.026902592 2.40726188 0.015220111
0.697987742 0.478414242
2271 AC097712.1 0.486870242 -2.292502217 4.077744474 -0.586967215
0.489452418 0.498141447
2272 AL255826.2 0.49522622 -2.415608216 4.416671772 -0.546929552 -
0.177004822 0.751495511
2273 AC096708.2 0.478414262 -2.271055522 4.479204761 -0.529247421
0.188798842 0.498141447
2274 AC021424.4 1.711109522 -2.527241629 2.440212294 -1.029762666 -
0.795755876 0.49521421
2275 AP000560.1 0.976576021 -1.520755908 2.970112194 -0.515286561
0.157607284 0.484870241
2276 AL512282.1 0.478414262 -2.271055522 4.479204761 -0.529247421 -
0.980250872 0.740104194
2277 PARP1 292.8427676 -0.229602821 0.18708712 -1.761766505
0.078108756 1.477572045
2278 AC005480.2 0.489705689 0.05402189 4.196172952 0.012876469 -
0.266591252 0.727248849
2279 AL291827.1 0.500997014 2.484998858 4.296126822 0.565270056 -
1.28506942 1.440411014
2280 AL022228.2 0.72891287 -0.92219097 2.55162759 -0.26246867 -
0.192924792 0.500997014
2281 STN1 41.90681685 -0.789065587 0.448407017 -1.759708284
0.078457264 0.498141447
2282 GGNBP2 109.2207514 -0.568979297 0.22246874 -1.758992151
0.078578672 0.748440174
2283 VDAC2 142.141267 0.427899099 0.248907292 1.759285922
0.078528955 0.489705489
2284 AP1G1 207.2560252 -0.418202622 0.227762622 -1.758908156 0.07859211
0.49521421
2285 AL022097.1 0.726077524 -2.971191046 2.466857824 -0.85702708 -
0.244500223 0.484870241
2286 ASTN2 16.24905127 -1.264749485 0.776175199 -1.758200815
0.078696229 0.489705489
2287 KCTD21 12.7254982 1.454440176 0.827205225 1.758045416
0.078729782 0.500997014
2288 PPIL1 50.90626214 0.724708921 0.412224097 1.757619641
0.078812249 0.478414242
2289 ADAMTS14 201.0667027 0.29275728 0.224092659 1.757110762
0.078898922 0.500997014
2290 SRGAP1 9.498122846 1.720848617 0.985489796 1.756222272
0.079021502 0.751495511
2291 AL445189.2 0.717621545 -2.955950852 2.792242589 -0.779267242
0.502726888 1.144811841
2292 AC005094.1 0.498161667 0.02169065 4.170279829 0.007599166
0.114601456 0.49521421
2293 AC018946.1 0.49522622 -2.415608216 4.416671772 -0.546929552
0.127097004 0.484870241
2294 MFSD12 18.18219998 1.258226798 0.717222222 1.754442086

0.079254785 0.748440174
2295 PIP5K1C 22.92076624 1.046779204 0.596712585 1.754240611
0.079289287 0.741989481
2296 POLR2F 17.47256425 1.274662965 0.726225222 1.755189242
0.079226926 0.478414242
2297 ITGA5 88.47067221 0.592075079 0.228004587 1.754626182
0.079221558 0.49521421
2298 NINJ1 126.5256247 0.469902689 0.267724702 1.75510929
0.079240599 0.478414242
2299 SPATA12 29.25147769 0.962166212 0.549249262 1.752285292
0.079552022 0.489705489
2300 AC127620.4 1.705488901 -1.265427142 2.208857625 -0.548074998 -
0.260014287 0.990701702
2301 EEF1B2 522.4885226 0.258027867 0.14725806 1.75228249
0.079725074 0.71891187
2302 AC072222.1 20.92025148 1.121222149 0.645851228 1.751691567
0.079826858 0.478414242
2303 AL127802.2 0.478414262 -2.271055522 4.479204761 -0.529247421
0.18269721 0.500997014
2304 AC104250.1 0.486870242 -2.292502217 4.077744474 -0.586967215
0.226477718 0.49521421
2305 DNAH10 22.89282224 -1.042061025 0.595192209 -1.750794254
0.079981257 0.498141447
2306 AC010719.1 1.986975975 -1.562425162 2.264964908 -0.660654692
0.214192482 0.740104194
2307 AL512791.1 58.58128722 0.696256522 0.298042018 1.749199214
0.08025659 0.714077514
2308 ANXA6 247.4857682 -0.292147808 0.167589726 -1.749199155 0.0802566
0.500997014
2309 SCD5 9.115584192 -1.819101941 1.042110208 -1.742920971
0.081172871 0.498141447
2310 H1-0 16.94176091 -1.242022117 0.711085102 -1.746658891
0.080696525 0.985021009
2311 PDZRN2 4.018456779 2.858575548 1.629157221 1.742920058
0.081171287 0.49521421
2312 PDE4DIP 21.2770118 -1.101262611 0.621649282 -1.742629958
0.081222626 0.500997014
2313 AC009024.1 10.96659051 1.645222286 0.94228709 1.742964279
0.081165219 0.500997014
2314 OSCAR 25.19604499 1.084077182 0.621962869 1.74299061
0.081225255 0.49521421
2315 OAS2 26.12520227 0.988872022 0.566127085 1.746721517
0.080682921 0.500997014
2316 RNF125 27.26555552 0.841025746 0.482192008 1.744168272
0.081129752 0.484870241
2317 MED20 52.94726626 0.722582271 0.412749228 1.746425908
0.080726971 1.970044018
2318 HEATR6 66.62091727 -0.625225699 0.257756757 -1.747907445
0.080480056 1.117074528
2319 ITK 64.24870702 -0.704921049 0.402712549 -1.746116899
0.080790641 0.987847254

2320	CCDC86	52.727091	0.699199957	0.400862819	1.744227488	0.081117687	0.985021009
2321	TRIM5	60.70806199	0.670545564	0.282720516	1.747428726	0.080561265	1.950214715
2322	CD22	107.000021	-0.512298896	0.292777646	-1.747576451	0.080527296	0.478414242
2323	GRSF1	102.8686647	-0.52876027	0.208412944	-1.746879568	0.080658221	0.478414242
2324	DENND4B	92.82612571	0.522697112	0.205484522	1.747051174	0.080628462	0.981194441
2325	SLC25B2	171.5192645	-0.414200014	0.227525844	-1.742810201	0.081192174	0.478414242
2326	COTL1	292.5145522	-0.894689449	0.512262262	-1.742802211	0.081267978	1.111402844
2327	OSTF1	280.5406927	-0.222426221	0.184977255	-1.742059229	0.08122225	1.121495149
2328	CCT2	281.205721	0.229558187	0.188929102	1.744256121	0.081114429	0.478414242
2329	AL096828.2	0.740204196	1.049207872	2.521140276	0.297158266	-0.292471544	0.745814817
2330	CIITA	25.1081428	1.022789811	0.592104028	1.741229955	0.081625758	0.49521421
2331	RAB14	111.2812092	0.497087054	0.285484109	1.741207427	0.081647224	0.484870241
2332	AL024428.1	0.726077524	-2.971191046	2.466857824	-0.85702708	-0.564510516	1.117074528
2333	PDE2B	8.819919211	-1.757817564	1.010115402	-1.740214592	0.081821244	0.484870241
2334	PITHD1	25.47942625	-0.862477217	0.496221856	-1.740068291	0.081847027	0.49521421
2335	AP001046.1	0.500997014	2.484998858	4.296126822	0.565270056	-0.141622291	1.705488901
2336	AMBRA1	62.84416592	0.629072178	0.267422181	1.729290871	0.081982612	0.478414242
2337	AC012277.1	0.478414262	-2.271055522	4.479204761	-0.529247421	-0.175977078	0.498141447
2338	MEI1	19.42102429	-1.146110027	0.659779802	-1.7271099	0.082267774	1.705488901
2339	NCKAP5L	24.94511249	-0.922778016	0.521197112	-1.72716685	0.082257724	0.948110051
2340	OSGIN1	21.22670062	1.151182201	0.662556526	1.727486772	0.082201287	0.498141447
2341	PRKDC	169.9052202	0.40101471	0.220749169	1.72788149	0.082221698	1.142984495
2342	AP1S2	255.4189521	0.241269145	0.196271895	1.727871627	0.082222426	0.745814817
2343	HNRNPC	827.1979441	0.214804445	0.122682622	1.726725042	0.082425715	0.489705489
2344	PAN2	88.12751721	0.549650292	0.216692858	1.725594528	0.082625554	0.748440174
2345	ADD2	140.6927468	0.467660071	0.269509862	1.725224221		

0.082701079 0.740104194
2346 EID1 425.0827182 0.292822048 0.169402972 1.724521201
0.082825647 0.498141447
2347 SLBP 46.1565841 -0.761695279 0.429224671 -1.724141974
0.082892896 0.714077514
2348 ARNTL2-AS1 0.49522622 -2.415608216 4.416671772 -0.546929552 -
0.822201566 0.979411277
2349 AP2A2 21.90290407 1.075210864 0.620629195 1.722614051 0.08216429
0.999158481
2350 PYCR1 52.25412218 0.722624566 0.417716615 1.722257622
0.082209907 0.489705489
2351 AL117229.2 0.500997014 2.484998858 4.296126822 0.565270056 -
0.165582429 0.71891187
2352 AC079221.1 0.992487988 -1.558611578 2.168280519 -0.491926891 -
0.162127829 0.745814817
2353 AC156455.1 1.224229191 -1.952471227 2.727792282 -0.71576976 -
0.455268776 0.948110051
2354 AC128122.2 0.500997014 2.484998858 4.296126822 0.565270056 -
0.245529024 1.947481248
2355 RHCE 9.228924828 1.688241225 0.975009288 1.721615622
0.082242021 0.489705489
2356 FPR1 82.40260756 0.560085422 0.222501569 1.721222125
0.082294227 0.717411545
2357 CPPED1 265.7471602 -0.282521657 0.162818477 -1.720706219
0.082504176 0.498141447
2358 AC018628.1 0.976576021 -1.520755908 2.970112194 -0.515286561
0.271076229 0.714077514
2359 AC012640.4 0.489705689 0.05402189 4.196172952 0.012876469 -
0.291495543 0.498141447
2360 AC020916.1 19.61820521 -1.170102762 0.676259096 -1.720258077
0.082584176 0.714077514
2361 AL590428.1 0.726077524 -2.971191046 2.466857824 -0.85702708 -
0.527787968 0.745814817
2362 AL255102.1 0.976576021 -1.520755908 2.970112194 -0.515286561
0.126922209 0.981194441
2363 AC009209.1 0.745824827 -0.972210916 2.522016256 -0.276242662 -
0.105642558 0.484870241
2364 AL129407.1 0.498161667 0.02169065 4.170279829 0.007599166
0.152982023 0.987847254
2365 AC072508.2 1.958822755 0.062146644 2.212928071 0.027289804 -
0.105522426 0.498141447
2366 SOCS5 29.69886956 -0.965269526 0.558579866 -1.728077924
0.082974255 1.747818855
2367 NUDT16L1 56.8080482 0.706115527 0.40889226 1.726894115
0.084186682 0.500997014
2368 MIR2142HG 46.69225741 0.729418567 0.428292411 1.72642297
0.084269269 0.990701702
2369 AC021915.2 0.478414262 -2.271055522 4.479204761 -0.529247421
0.222242427 0.987847254
2370 AC012212.2 0.486870242 -2.292502217 4.077744474 -0.586967215 -
0.122665927 0.498141447

2371	AC069224.5	0.49522622	-2.415608216	4.416671772	-0.546929552	-0.112266494	0.748440174
2372	AL158206.1	14.95492727	-1.219940627	0.76527662	-1.724562556	0.084606156	0.724522501
2373	ATG9B	2.180176527	-2.540420027	2.05284974	-1.722797002	0.084744496	0.498141447
2374	RCN1P2	2.180176527	-2.540420027	2.05284974	-1.722797002	0.084744496	0.500997014
2375	AP000688.4	0.498161667	0.02169065	4.170279829	0.007599166	-0.270550725	0.948110051
2376	KCTD19	2.174454708	-2.524294648	2.051186824	-1.722048621	0.084879721	0.484870241
2377	TLR8	2.174454708	-2.524294648	2.051186824	-1.722048621	0.084879721	0.478414242
2378	GPRC5C	26.79875606	-0.852624522	0.495629714	-1.722288402	0.085017287	1.97011408
2379	MGRN1	89.85199292	0.522740482	0.209270701	1.722570162	0.084966284	1.484019012
2380	PPME1	79.20491188	0.572657451	0.222666262	1.721416754	0.085175226	0.498141447
2381	DCUN1D2	21.85671727	0.882245249	0.512755771	1.72079048	0.085288851	0.740104194
2382	GMPR2	126.6224748	-0.455189088	0.264486166	-1.721021745	0.085245064	0.745814817
2383	AC011498.2	0.489705689	0.05402189	4.196172952	0.012876469	-0.101257726	0.49521421
2384	AC002044.2	0.49522622	-2.415608216	4.416671772	-0.546929552	0.097072675	0.751495511
2385	AC007610.6	0.751495521	2.069950829	2.726554747	0.822804022	-0.200922619	0.945184705
2386	AL121972.1	0.748660174	1.029647608	2.51687248	0.292772655	-0.865882889	0.724522501
2387	AC022206.2	0.498161667	0.02169065	4.170279829	0.007599166	-0.098260842	0.740104194
2388	ANGPTL5	0.500997014	2.484998858	4.296126822	0.565270056	-0.426795008	0.500997014
2389	AC090519.1	0.726077524	-2.971191046	2.466857824	-0.85702708	0.608528462	0.478414242
2390	CTH	2.191266665	-2.541276217	2.061185471	-1.718125926	0.085772647	0.500997014
2391	ITGA11	9.087482109	-1.815992727	1.057082711	-1.717928709	0.08580962	0.500997014
2392	STXBP4	17.18677544	1.229262964	0.721197428	1.718240798	0.085724472	1.117074528
2393	AC100788.1	0.990652641	-2.415742141	2.252204524	-1.018619724	-0.508862215	0.985021009
2394	AL129184.1	1.229859822	-2.728517982	2.865702421	-1.201082442	0.904608917	1.117074528
2395	C1QA	12.79849824	-1.408691855	0.82054789	-1.716769822	0.086021227	0.489705489
2396	CD1D	5.020602141	2.528178792	1.478099412	1.717190854		

0.085944206	0.945184705
2397	COA6	42.80624694	0.767512766	0.447029024	1.716921551
0.085992507	1.119909884
2398	AL127792.1	0.745824827	-0.972210916	2.522016256	-0.276242662
0.166277878	0.724522501
2399	CLIP4	28.61400765	0.960868186	0.560022269	1.715726875
0.086210212	0.500997014
2400	PTTG1	42.95544521	0.765976524	0.446854222	1.71415204
0.086500622	0.500997014
2401	LPP	144.4174542	-0.422974186	0.246695919	-1.714556884
0.086426499	1.125520514
2402	FAM12B	127.9821725	-0.502526222	0.292052425	-1.714824142	0.08627564
0.498141447
2403	CD276	174.8894275	0.290422665	0.227628965	1.715095942
0.086227628	0.714077514
2404	OST4	262.2291977	-0.287525827	0.167754698	-1.712965867
0.086524995	0.740104194
2405	ZMAT2	67.21504155	-0.625898222	0.265514472	-1.712276208
0.086827242	0.484870241
2406	DNAJC4	71.20402271	-0.590105428	0.244474259	-1.712060528
0.086701405	0.498141447
2407	FYB1	168.2904792	0.406769082	0.22750724	1.712659714
0.086775161	0.49521421
2408	TFRC	172.086266	0.495126664	0.289164767	1.712299424
0.086841502	0.484870241
2409	AL691449.1	0.489705689	0.05402189	4.196172952	0.012876469
0.22027117	0.489705489
2410	S100A5	4.912767522	-2.460720519	1.427892791	-1.711226772
0.087018962	0.489705489
2411	FRAS1	4.882677758	-2.448406477	1.421228024	-1.7105718
0.087160188	0.714077514
2412	IL12	5.00890595	2.567286788	1.500402625	1.711064095
0.087069281	0.987847254
2413	PARP8	46.181425	0.746917819	0.426652772	1.710552246
0.087162616	0.49521421
2414	AL672277.1	0.489705689	0.05402189	4.196172952	0.012876469	-
0.275692216	0.974574021
2415	AL049552.1	0.49522622	-2.415608216	4.416671772	-0.546929552	-
0.140286702	1.144811841
2416	AC008726.1	0.751495521	2.069950829	2.726554747	0.822804022	-
0.096142571	0.49521421
2417	AC021242.2	1.225520516	-0.54785862	2.606584125	-0.2101826	-
0.291442917	0.727248849
2418	FEM1C	24.29124854	-1.019994242	0.597159411	-1.708077148
0.087622029	0.974574021
2419	OXER1	17.69040541	1.20162592	0.702567254	1.707904842	0.087654
0.500997014
2420	RASSF2	150.9529069	-0.451781458	0.264519222	-1.707922667
0.087648651	0.500997014
2421	AL121772.2	0.748660174	1.029647608	2.51687248	0.292772655	-
0.576802104	0.71891187

2422	CHURC1	65.85202144	0.61152601	0.25818666	1.707210956
	0.087764271	0.49521421
2423	SERPINH1	22.28999256	-1.046201902	0.612900761	-1.706967864
	0.087828026	0.990701702
2424	AL445490.1	0.489705689	0.05402189	4.196172952	0.012876469	-
0.224242071	0.500997014
2425	TP52INP2	18.7202126	-1.15642102	0.677706921	-1.706272212
	0.087928596	0.478414242
2426	TIMM17A	100.5826196	0.500780924	0.292668181	1.705261094
	0.08814574	0.500997014
2427	AC104265.4	0.987867256	0.042250627	2.875240277	0.015077222	-
0.440999684	0.500997014
2428	AL126982.2	0.500997014	2.484998858	4.296126822	0.565270056
	0.106027448	0.981194441
2429	AP000962.2	0.498161667	0.02169065	4.170279829	0.007599166	-
0.098785496	0.498141447
2430	DAP2	192.4241828	0.271277199	0.218002514	1.702545487
	0.088466022	1.941840724
2431	AC012588.1	0.748660174	1.029647608	2.51687248	0.292772655	-
0.260170622	0.714077514
2432	GPX4	289.2646872	0.272740766	0.160749246	1.70290428
	0.088585964	0.500997014
2433	AC097627.2	8.501708212	1.745618708	1.025842686	1.701642665
	0.088822187	0.484870241
2434	CHPF	21.52924945	-0.885272282	0.520226254	-1.701867904
	0.088780124	0.500997014
2435	AC020978.7	27.11446021	0.954189694	0.560742912	1.701649674
	0.08882106	0.478414242
2436	MYDGF	154.5994401	0.416079424	0.244508612	1.701696294
	0.088812297	0.724522501
2437	AL252740.1	0.742989481	-2.000642057	2.742921251	-0.801470644
	0.288511486	0.727248849
2438	AC018766.1	0.486870242	-2.292502217	4.077744474	-0.586967215
	0.225471107	0.489705489
2439	AC010761.2	1.225520516	-0.54785862	2.606584125	-0.2101826	-
0.099081182	0.478414242
2440	SLC22A17	4.010000801	2.869685929	1.688511265	1.699526147
	0.08921821	0.498141447
2441	SF2B2	122.5555829	0.457627287	0.269226266	1.699787295
	0.089170942	0.484870241
2442	AL122270.1	0.972740684	-2.29250678	2.080064802	-1.101764725	-
0.472169222	0.49521421
2443	AC011444.2	0.748660174	1.029647608	2.51687248	0.292772655	-
0.154827146	0.500997014
2444	AL096869.1	0.498161667	0.02169065	4.170279829	0.007599166
	0.148664541	0.714077514
2445	ELOC	70.81682725	0.590872262	0.247922159	1.698288962
	0.089452227	0.498141447
2446	SGSH	28.26010929	0.812192489	0.478979012	1.697764244
	0.089552248	0.498141447
2447	PRKCH	2.002988056	4.484927112	2.642152544	1.69680979

0.089722627	1.454990294
2448	PARM1	10.76085252	1.591256251	0.927727292	1.696910417
0.089712598	0.49521421
2449	AC022558.2	1.227074528	-0.524749642	2.612295646	-0.204704871	-
0.597067425	0.714077514
2450	AC011497.1	0.500997014	2.484998858	4.296126822	0.565270056	-
0.11461825	0.500997014
2451	AC125279.2	0.727268849	-0.952727871	2.205072418	-0.288262241	-
0.211249474	0.484870241
2452	AC096559.1	0.486870242	-2.292502217	4.077744474	-0.586967215	-
1.021979901	0.484870241
2453	MIR29B2CHG	22.1668914	1.045298044	0.616529987	1.695452695
0.089989287	1.491499717
2454	PHF20L1	18.4282706	-1.207910992	0.771577521	-1.695112856
0.090054014	0.500997014
2455	DECR1	264.9260274	-0.219020692	0.188222246	-1.692924061
0.090279714	0.498141447
2456	PRCP	127.2185767	0.452550405	0.267819142	1.692495094
0.090261268	0.500997014
2457	AC011262.1	0.489705689	0.05402189	4.196172952	0.012876469	-
0.097401621	0.484870241
2458	AC129520.2	0.486870242	-2.292502217	4.077744474	-0.586967215	-
0.565726895	0.49521421
2459	AL021717.1	1.228265862	0.628005757	2.620292458	0.228758818	-
0.179102859	0.751495511
2460	TTYH2	67.14260279	0.61048845	0.260808272	1.692002262
0.090645522	0.478414242
2461	AC244121.1	0.49522622	-2.415608216	4.416671772	-0.546929552	-
0.290518299	0.498141447
2462	AC012564.1	0.49522622	-2.415608216	4.416671772	-0.546929552	-
0.222164092	0.49521421
2463	MRPS20-DT	2.208278621	-2.548522612	2.101722922	-1.688287452
0.091226877	0.484870241
2464	CCDC68	5.65287052	-2.204220728	1.204208749	-1.689952187
0.091026911	0.498141447
2465	CRTAM	2.008261502	2.511726281	2.078799221	1.689210022
0.091160022	0.484870241
2466	AP000592.2	2.01955162	2.504405484	2.07641607	1.68771824
0.091465209	0.49521421
2467	CCT4P2	2.011095651	2.512928692	2.078510808	1.69012289
0.091004447	1.944442287
2468	SOCS1	2.011095651	2.512928692	2.078510808	1.69012289
0.091004447	0.748440174
2469	VAC14-AS1	11.57768218	-1.497658247	0.887012708	-1.688427509
0.091229192	0.994212224
2470	ATXN1L	25.25896272	-0.971275896	0.575542102	-1.687581505
0.091491592	0.498141447
2471	TREM1	12.72480921	1.222542095	0.789587682	1.687642964
0.091479787	0.745814817
2472	ITPRID2	42.91627547	-0.751156685	0.444228256	-1.690886602
0.090858462	1.471901251

2473 CDK5RAP2 114.4240724 -0.476228028 0.281798612 -1.69024909
0.090961189 0.948110051
2474 GALE 89.24709529 0.527645761 0.218270402 1.688742914
0.091268708 0.987847254
2475 PPP1R2 88.1594652 0.524876778 0.216770692 1.688529876
0.091209559 0.751495511
2476 AL682812.1 0.748660174 1.029647608 2.51687248 0.292772655 -
0.675845727 0.498141447
2477 TMEM71 17.42119729 1.175745864 0.697098652 1.686627654
0.091674971 0.49521421
2478 LINC00222 25.07902029 0.82266812 0.488487086 1.686161502
0.091764697 0.985021009
2479 DPH1 14.78651792 -1.420210228 0.84271077 -1.685406652
0.091910141 0.714077514
2480 SPDYA 6.255981222 2.04598962 1.214087265 1.68520804
0.091948441 0.49521421
2481 PIAS1 187.1256147 -0.280224774 0.225629871 -1.685214694
0.091947157 0.498141447
2482 AC072569.2 0.49522622 -2.415608216 4.416671772 -0.546929552 -
0.169660218 0.49521421
2483 AL252684.1 0.49522622 -2.415608216 4.416671772 -0.546929552 -
0.261822912 0.740104194
2484 AC074251.1 0.498161667 0.02169065 4.170279829 0.007599166 -
0.094660868 0.945184705
2485 AC008764.9 0.982196662 -2.404284457 2.098256189 -1.098806625
0.09226157 0.500997014
2486 AC116407.2 1.474727698 -0.956054822 2.407040792 -0.297190955 -
0.096165013 0.987847254
2487 AC104211.2 0.478414262 -2.271055522 4.479204761 -0.529247421
0.119812747 0.500997014
2488 AC008760.1 0.726077524 -2.971191046 2.466857824 -0.85702708
0.098927865 0.999158481
2489 AC024267.4 1.207227224 -1.921069226 2.961428148 -0.65207144 -
0.102700013 0.49521421
2490 AC072072.2 0.486870242 -2.292502217 4.077744474 -0.586967215
0.10222502 1.149457188
2491 AC092718.5 0.748660174 1.029647608 2.51687248 0.292772655 -
0.079176814 0.500997014
2492 ARMC9 10.55872506 -1.668694197 0.992541145 -1.68122428
0.092717417 0.714077514
2493 HSPA5 1504.720912 0.176220227 0.104865919 1.681482726
0.092668992 0.727248849
2494 ANGPTL4 2.014082222 2.50192585 2.082711269 1.680624247
0.092825922 0.489705489
2495 AMHR2 29.12642019 -0.788649082 0.469207005 -1.680454528
0.092868915 0.484870241
2496 KIF15 9.727086495 1.585118675 0.942690272 1.679702072
0.092015296 0.741989481
2497 PER2 46.98517402 0.719252222 0.428227642 1.679446205
0.092065115 0.484870241
2498 CALB1 2.928725512 -2.76416882 1.649019498 -1.676249921

						0.092689248	0.999158481
2499	STARD10	6.282782291	-2.024998018	1.206692427	-1.678127927	0.092220174	0.478414242
2500	LBX2	6.28551754	-2.02222625	1.206608401	-1.676878968	0.092566152	0.484870241
2501	PIEZO2	6.268605582	-2.019298904	1.204087884	-1.677119196	0.092519176	0.974574021
2502	ST2GAL6	51.24955415	-0.685510694	0.408716216	-1.677229008	0.092497709	0.49521421
2503	TMEM214	51.1298196	-0.708710559	0.422709261	-1.676590642	0.092622558	0.49521421
2504	SIRPA	82.47526896	0.54060865	0.222096215	1.678407227	0.092267606	0.484870241
2505	PARVG	191.6277245	-0.268782578	0.219724626	-1.678285205	0.092271924	0.714077514
2506	IDI1	280.1260256	-0.212657668	0.186526152	-1.676122715	0.092712962	0.489705489
2507	SERBP1	257.8211121	0.224222528	0.19220072	1.67781849	0.092282529	0.740104194
2508	LINC00962	86.42262011	-0.527669254	0.220920017	-1.675299865	0.092855802	1.117074528
2509	CD72	7.842242281	-1.787126629	1.067529158	-1.67407759	0.094115251	0.500997014
2510	MLC1	64.4199695	0.622004121	0.271681922	1.672485024	0.094221852	0.498141447
2511	AL022584.2	0.498161667	0.02169065	4.170279829	0.007599166	0.112246816	1.480408291
2512	AL158825.2	0.486870242	-2.292502217	4.077744474	-0.586967215	0.462940249	0.992487988
2513	MACIR	56.22626072	-0.676462576	0.404296105	-1.672774705	0.094271656	0.500997014
2514	AC008442.5	0.489705689	0.05402189	4.196172952	0.012876469	-0.168962892	0.489705489
2515	AC092682.1	0.498161667	0.02169065	4.170279829	0.007599166	0.082285028	0.741989481
2516	AL126987.1	0.486870242	-2.292502217	4.077744474	-0.586967215	-0.082842721	0.478414242
2517	NDUFV2	62.87712805	0.615660271	0.268258227	1.67181652	0.094560508	0.500997014
2518	AC004160.1	0.996222224	0.022664057	2.062891868	0.007722529	0.18072118	0.489705489
2519	CSDC2	14.4216612	-1.272210251	0.822405696	-1.669869577	0.094945171	1.485978941
2520	ALMS1	22.15794511	-1.042802786	0.624441224	-1.669977219	0.094922871	0.500997014
2521	SLC26A2	50.26270092	-0.702422208	0.421101271	-1.670424621	0.094822405	0.478414242
2522	SLC20A7	112.7605071	0.467605244	0.279852429	1.670892745	0.094742667	0.498141447
2523	NDUFA4	464.6069187	0.255167412	0.152775114	1.670215819	0.094876671	0.478414242

2524	LAMB1	5.85278549	-2.27271071	1.261661946	-1.66907118	0.095102274	1.971899245
2525	SLC2A11	7.576186249	-2.011808629	1.205975205	-1.668200526	0.095275927	0.500997014
2526	B4GALT5	28.96852158	-0.776821292	0.465516592	-1.668751247	0.095166688	0.49521421
2527	ABHD5	42.51065247	0.726679021	0.441722042	1.667742298	0.095266676	0.500997014
2528	RBCK1	79.75019941	-0.560292101	0.225896565	-1.668247222	0.095246819	1.117074528
2529	AGO2	95.80602148	0.50215952	0.201722668	1.66761704	0.095291772	0.717411545
2530	AC100825.2	0.489705689	0.05402189	4.196172952	0.012876469	-0.108404521	0.990701702
2531	AL080284.1	0.498161667	0.02169065	4.170279829	0.007599166	0.096847759	0.478414242
2532	TMIGD2	8.577724449	-1.702292482	1.021727806	-1.666092929	0.095694905	0.500997014
2533	ADAMTSL4-AS1	7.75602582	1.819622059	1.092212262	1.66600665	0.09571209	0.500997014
2534	AC122552.4	0.740204196	1.049207872	2.521140276	0.297158266	0.45888962	0.478414242
2535	RARG	11.72411252	1.422985481	0.860968907	1.665548511	0.095802274	0.741989481
2536	AC072575.4	1.229859822	-2.728517982	2.865702421	-1.201082442	0.105996558	1.128245842
2537	AC100814.1	0.976576021	-1.520755908	2.970112194	-0.515286561	-0.472820618	0.498141447
2538	DVL2	24.12552527	-1.068176722	0.641604422	-1.664852507	0.095942185	0.948110051
2539	ANAPC1P2	1.482192676	-0.966185564	2.429267947	-0.297727046	-0.568110651	0.49521421
2540	AC254562.1	0.727268849	-0.952727871	2.205072418	-0.288262241	0.119859426	0.478414242
2541	AC008802.1	0.489705689	0.05402189	4.196172952	0.012876469	-0.091725766	0.945184705
2542	AC022050.5	0.49522622	-2.415608216	4.416671772	-0.546929552	0.086240243	0.489705489
2543	AC007614.1	0.478414262	-2.271055522	4.479204761	-0.529247421	-0.117117122	0.999158481
2544	AC010615.1	1.96160804	-1.540528772	2.149259229	-0.716742241	-0.24960927	0.484870241
2545	LPL	8.507420142	1.741202486	1.047298726	1.662502947	0.096411972	0.740104194
2546	AC098924.2	0.500997014	2.484998858	4.296126822	0.565270056	-0.712522223	0.484870241
2547	SAMD10	28.22966229	0.925926299	0.562285241	1.661549261	0.096602164	0.500997014
2548	AC064807.1	0.727268849	-0.952727871	2.205072418	-0.288262241	-0.265951246	0.992487988
2549	AC008592.2	0.498161667	0.02169065	4.170279829	0.007599166		

0.082974522	0.489705489
2550	AC090510.2	0.727268849	-0.952727871	2.205072418	-0.288262241
0.076055811	1.722441111
2551	PPP1R26	22.27512676	-0.99648707	0.600272527	-1.660057728
0.096902828	0.751495511
2552	TRIM25 105.4259875	-0.476424117	0.286948962	-1.660209601
0.096852185	1.705488901
2553	AC145207.4	12.96794669	1.24992181	0.812900046	1.658596552
0.097197117	0.727248849
2554	PA2G4P6	18.65786606	1.142660982	0.688896175	1.658682884
0.097179508	0.740104194
2555	CDKL5	12.2748162	-1.220169298	0.802425025	-1.657686687
0.097280722	0.740104194
2556	CAMKK2 40.22968256	-0.771769422	0.465496029	-1.657950565
0.097227445	0.478414242
2557	SCCPDH 90.14911129	-0.528865924	0.225249102	-1.656779128
0.097564128	0.71891187
2558	RABAC1 114.8848814	-0.459572205	0.277265959	-1.656916757
0.097526206	0.727248849
2559	PPP1R7 102.9289157	0.492747962	0.29801262	1.656796625
0.097560598	0.478414242
2560	AC091812.1	0.500997014	2.484998858	4.296126822	0.565270056
0.240814051	0.945184705
2561	AL126268.1	0.748660174	1.029647608	2.51687248	0.292772655	-
0.078010875	0.981194441
2562	AC020907.4	0.49522622	-2.415608216	4.416671772	-0.546929552	-
0.10848227	0.484870241
2563	AC002298.1	0.489705689	0.05402189	4.196172952	0.012876469
0.099196089	1.720854827
2564	NCBP2AS2	49.4284877	0.684456648	0.412289796	1.655717227
0.097779077	0.498141447
2565	AC092964.1	0.748660174	1.029647608	2.51687248	0.292772655
0.088125618	0.748440174
2566	MIR5687	2.759755826	2.749290046	1.661792158	1.654412697
0.098042688	0.714077514
2567	CDC27	282.817664	0.205881124	0.184905228	1.654257997
0.098075102	0.484870241
2568	PTPN6	901.6540214	0.196425722	0.118727871	1.654419728
0.098042261	0.981194441
2569	AAK1	112.240922	-0.462224947	0.280152692	-1.652865762	0.09815479
1.471901251
2570	THEMIS2	102.0126278	0.477004587	0.28849585	1.652419229
0.098245572	0.49521421
2571	AC009092.4	0.979411277	0.062718118	2.082209142	0.020241171	-
0.224254409	1.474727498
2572	AC008985.1	0.979411277	0.062718118	2.082209142	0.020241171	-
0.102595116	0.49521421
2573	AC114980.1	1.494485002	0.02221681	2.582522648	0.008989972
0.191472087	0.751495511
2574	AC040977.1	0.745824827	-0.972210916	2.522016256	-0.276242662
0.088548629	0.478414242

2575 AC072225.1 0.478414262 -2.271055522 4.479204761 -0.529247421
0.077950142 0.990701702
2576 AP000542.2 0.478414262 -2.271055522 4.479204761 -0.529247421 -
0.080602021 0.748440174
2577 NOTCH4 2.886455621 -2.749125922 1.664228422 -1.651792926
0.098576769 0.727248849
2578 CASP2 221.2266541 0.246524795 0.209802469 1.651662796
0.098602107 0.49521421
2579 AC007227.1 0.751495521 2.069950829 2.726554747 0.822804022
0.427626421 0.498141447
2580 PDXDC1 185.9546157 0.267010186 0.222481548 1.649620786
0.099020521 0.717411545
2581 GRIA4 17.21165199 -1.170758209 0.709982889 -1.648992784
0.099149112 0.748440174
2582 RPS24 818.5042029 0.20160418 0.122288822 1.648590274
0.099221602 1.144811841
2583 AC128256.2 0.999158681 1.615997221 2.159709049 0.51142865 -
0.122001693 0.498141447
2584 FKBP4 79.19062287 0.554582762 0.226512864 1.648028418 0.09924684
1.952151041
2585 AC004585.1 0.742989481 -2.000642057 2.742921251 -0.801470644
0.070685081 0.741989481
2586 AL252802.4 0.49522622 -2.415608216 4.416671772 -0.546929552
0.191276414 1.001994018
2587 PNO1 49.71620486 0.692272046 0.420226424 1.646989118
0.099560282 0.500997014
2588 AC092484.2 559.6826782 -0.224298222 0.142211268 -1.647080879
0.099541422 0.49521421
2589 IMP4 121.9229247 0.478127648 0.290426589 1.646272081
0.099707755 0.49521421
2590 PSMB6 249.7249022 0.216952975 0.192565959 1.645944982
0.099775087 0.994212224
2591 AL129246.5 0.500997014 2.484998858 4.296126822 0.565270056
0.12522476 0.478414242
2592 AC004467.1 0.500997014 2.484998858 4.296126822 0.565270056 -
0.122250073 0.741989481
2593 AC008542.2 0.724522502 -2.98570617 2.452708589 -0.86474217
0.071527609 1.117074528
2594 AC010978.1 0.49522622 -2.415608216 4.416671772 -0.546929552 -
0.069252416 0.498141447
2595 TXLNG 16.50826059 -1.18648777 0.721657828 -1.644114042
0.100152648 0.489705489
2596 LILRA6 17.94252687 1.127261991 0.69194928 1.642707168
0.100226705 0.71891187
2597 POGLUT1 79.50918507 0.540456071 0.228822424 1.642605701
0.100257676 0.49521421
2598 SMIM12 8.822299928 -1.751598744 1.066128024 -1.642928051
0.100295751 0.500997014
2599 LMO4 59.49008204 -0.622619169 0.279100524 -1.642258987
0.100515629 0.751495511
2600 SESN2 45.57992921 -0.725018785 0.447626072 -1.642000794

0.100589829	0.489705489
2601	FAM107B	48.59872122	-0.684055809	0.416720784	-1.641481249	0.100697526	0.484870241
2602	AC020910.5	0.727268849	-0.952727871	2.205072418	-0.288262241	-0.090228913	0.500997014
2603	AL254864.1	0.742989481	-2.000642057	2.742921251	-0.801470644	0.062448829	0.478414242
2604	AC120651.1	1.98419069	0.022486062	2.129406254	0.015652025	0.200227225	0.484870241
2605	AC004067.1	0.486870242	-2.292502217	4.077744474	-0.586967215	0.062119289	0.498141447
2606	AP002064.2	22.2950264	-0.999062681	0.609215814	-1.629646729	0.101078641	0.478414242
2607	TRIM27	162.627722	-0.29607175	0.241606226	-1.629227516	0.101145068	0.500997014
2608	AATBC	0.985022009	-1.54422206	2.962499952	-0.52129252	-0.060727285	0.727248849
2609	CD58	98.28448222	0.50792166	0.210052774	1.628205058	0.101278925	1.119909884
2610	LRRC2	6.608066296	-2.092821557	1.278006771	-1.627574702	0.101510444	0.740104194
2611	AL121574.1	0.727268849	-0.952727871	2.205072418	-0.288262241	-0.106760173	0.500997014
2612	AC022044.1	0.979411277	0.062718118	2.082209142	0.020241171	-0.296126905	0.478414242
2613	AL078581.2	0.49522622	-2.415608216	4.416671772	-0.546929552	-0.084704171	0.500997014
2614	SPESP1	2.754287528	2.747270487	1.678947225	1.626205228	0.101775699	0.489705489
2615	LPAR5	52.71499455	-0.650602772	0.297662621	-1.626062048	0.101826292	0.498141447
2616	EPG5	52.21144521	-0.697712562	0.426646152	-1.625242447	0.10197728	0.994212224
2617	SPPL2A	94.89264667	-0.517275758	0.216265946	-1.625055112	0.102027495	0.714077514
2618	AC018628.1	0.726077524	-2.971191046	2.466857824	-0.85702708	-0.107059069	0.489705489
2619	AC007662.2	0.498161667	0.02169065	4.170279829	0.007599166	-0.070488412	0.972740484
2620	OR8G2P	22.76294227	-0.872298192	0.524428292	-1.624048286	0.102248691	0.714077514
2621	AC007278.1	0.478414262	-2.271055522	4.479204761	-0.529247421	-0.068602591	1.125520514
2622	TNF	12.97810771	-1.202621575	0.797677074	-1.622018695	0.102465065	1.115782111
2623	PDLIM1P4	2.922012684	-2.757727016	1.689702218	-1.622082662	0.102661862	0.945184705
2624	COX7A2	289.4872825	0.202604776	0.185455462	1.621684258	0.102745997	0.500997014
2625	AC012212.2	1.972899265	-0.697222499	2.11241011	-0.220107527	0.105200582	0.49521421

2626	LASP1	560.9628262	0.221195964	0.141728426	1.621260252	0.102825417	0.49521421
2627	ENPP2	698.262212	-0.212564226	0.120945288	-1.620942422	0.102902469	0.478414242
2628	LIPT2	2.922512277	-2.412218688	2.092702164	-1.62027822	0.102042706	0.49521421
2629	AP000769.1	2.925247526	-2.410598467	2.092009805	-1.629518629	0.102202275	0.500997014
2630	MIR100HG	5.591944522	-2.190802285	1.244999622	-1.628850557	0.102244659	0.489705489
2631	RSF1	82.67282425	0.526052281	0.222872848	1.629286216	0.102252442	1.497210248
2632	NAT10	81.19421614	0.541561626	0.222447046	1.629016207	0.102209588	0.498141447
2633	MCFD2	182.2572079	-0.27989065	0.222298172	-1.628248162	0.102451082	0.717411545
2634	TCTEX1D4	2.940969255	-2.417195022	2.099491502	-1.627629847	0.102602297	0.71891187
2635	CHI2L2	8.499227697	1.729606759	1.068752189	1.627698896	0.102588747	0.498141447
2636	AP001628.1	0.500997014	2.484998858	4.296126822	0.565270056	-0.070472863	0.500997014
2637	AL025252.4	0.726077524	-2.971191046	2.466857824	-0.85702708	-0.259517257	0.498141447
2638	UBALD2	2.942702504	-2.414452271	2.098797766	-1.626861018	0.102766619	0.727248849
2639	NDUFAF4	28.74054676	0.768486806	0.472420865	1.626665111	0.102808242	0.478414242
2640	AC011721.1	0.742989481	-2.000642057	2.742921251	-0.801470644	-0.067902148	0.500997014
2641	GGA1	64.05149216	0.592126955	0.26490625	1.625422298	0.104072587	0.478414242
2642	AC024580.2	0.740204196	1.049207872	2.521140276	0.297158266	0.229262869	0.498141447
2643	WWOX	22.84766696	-0.970067891	0.597124095	-1.624529445	0.104260729	0.994212224
2644	SKIL	77.24274256	-0.562259581	0.24649662	-1.622698604	0.10465285	1.119859812
2645	SNRK	28.0429642	-0.892022148	0.550522904	-1.622120166	0.104777621	1.114129191
2646	AP002498.1	0.987867256	0.042250627	2.875240277	0.015077222	-0.07825793	0.498141447
2647	DPP4	2.91560142	-2.405577279	2.102221498	-1.619912692	0.105251022	1.481904151
2648	TRIM24	59.19740584	-0.611478976	0.277501425	-1.619805744	0.105274012	1.117074528
2649	HTATIP2	92.00511292	-0.500768042	0.209079417	-1.620192144	0.105191009	0.49521421
2650	WDR42	70.99947564	0.559977475	0.245624626	1.620189701	0.105191522	0.981194441
2651	SGF29	22.12865272	-0.972742049	0.60121222	-1.619259095		

0.105270022	0.500997014
2652	CACNB2 2.949425224	-2.421108882	2.112087572	-1.619009514
0.105445216	0.478414242
2653	AC022510.2	2.952159482	-2.418214486	2.112290644	-1.618220804
0.105615021	0.748440174
2654	TTC22	7.497081225	1.762615707	1.089025875	1.618510222
0.105552682	0.478414242
2655	AC006042.1	0.726077524	-2.971191046	2.466857824	-0.85702708
0.197956485	0.724522501
2656	AC010201.2	0.724522502	-2.98570617	2.452708589	-0.86474217
0.055818113	0.500997014
2657	ADAM11 0.982196662	-2.404284457	2.098256189	-1.098806625	0.0528681
0.484870241
2658	AC006027.1	6.751155209	1.856958688	1.149272629	1.615767222
0.106144649	0.49521421
2659	LPCAT1 11.81425641	-1.282925927	0.85654684	-1.614547941
0.106408627	0.49521421
2660	RNASET2	21.89192209	-0.998560992	0.619051092	-1.612051015
0.106722425	0.724522501
2661	ACSS2	29.22892972	-0.752298464	0.466922075	-1.612542182
0.106626766	0.49521421
2662	DDX54	96.18422984	-0.585507569	0.262921828	-1.612215891
0.106675896	0.500997014
2663	AC092142.2	81.20670625	-0.527648625	0.226875098	-1.614220961
0.106479507	0.49521421
2664	KCP	112.7401284	-0.452569179	0.280510052	-1.612279522
0.106662077	0.478414242
2665	ATG2	165.0082756	0.275511594	0.222711252	1.612626765
0.106606227	0.714077514
2666	UQCRB	222.629089	0.225048627	0.207599841	1.61291567
0.106545719	0.724522501
2667	AL445922.1	0.498161667	0.02169065	4.170279829	0.007599166	-
0.14286211	0.489705489
2668	L2MBTL2	55.86241067	0.624610182	0.292688626	1.611959654
0.10697072	0.478414242
2669	AC009148.1	0.999158681	1.615997221	2.159709049	0.51142865	-
0.049596689	0.500997014
2670	AC008725.5	0.724522502	-2.98570617	2.452708589	-0.86474217	-
0.072250574	1.114129191
2671	B2GNT8 15.00552081	-1.207621426	0.75081491	-1.608414298
0.107744472	0.489705489
2672	MSANTD2	9.48285284	1.524256202	0.952806865	1.608560662
0.107712442	0.478414242
2673	AC091152.1	11.46515805	1.29246897	0.866277502	1.60828545
0.107750786	0.981194441
2674	GLMP	167.802947	-0.274805462	0.222996246	-1.608622256
0.107696755	0.717411545
2675	PLXDC2 57.50898122	-0.620441997	0.285879692	-1.607862818
0.107865008	0.478414242
2676	TXN	208.861028	0.207565005	0.191299906	1.607762489
0.107886988	0.49521421

2677	PSMD14	188.1249721	0.260422728	0.224295069	1.606912291	0.10807229	0.987847254
2678	BUD21	162.920155	0.282062522	0.227811478	1.60658155	0.108146192	0.999158481
2679	PSAT1	146.114984	-0.41116896	0.255988714	-1.606199562	0.10822007	0.987847254
2680	C1orf147	12.72291648	1.211252142	0.816758057	1.605426425	0.108297792	0.748440174
2681	AL162615.1	0.748660174	1.029647608	2.51687248	0.292772655	0.075292971	0.751495511
2682	SLC4A7	16.42750701	1.151545227	0.718054452	1.602701814	0.108779796	0.498141447
2683	AC092157.2	0.489705689	0.05402189	4.196172952	0.012876469	-0.021229599	0.478414242
2684	NBAS	110.2590954	-0.451829124	0.282040984	-1.602022604	0.109148179	0.49521421
2685	BRIX1	64.01792278	0.592928172	0.27092229	1.601176789	0.109227768	0.484870241
2686	FARSA	172.2202282	0.270888954	0.221694871	1.600764624	0.109429062	1.741148141
2687	GSAP	21.66465011	0.862996664	0.52947212	1.599702808	0.109664529	0.478414242
2688	LTV1	24.14569451	0.92916619	0.58722617	1.599052877	0.109808622	0.49521421
2689	AL255075.2	64.1246907	-0.580940844	0.262404199	-1.598607957	0.109907741	0.751495511
2690	CFD	79.52222165	-0.555286001	0.247281292	-1.598778789	0.109869764	1.941840724
2691	AC079780.1	0.727268849	-0.952727871	2.205072418	-0.288262241	0.045249152	0.985021009
2692	ACSM6	0.486870242	-2.292502217	4.077744474	-0.586967215	-0.061814026	0.489705489
2693	APPL2	22.42201092	-0.849957579	0.522182818	-1.597115786	0.1102299	0.945184705
2694	RBM25	128.5512771	0.428861014	0.268571024	1.596825272	0.110204661	0.484870241
2695	SLC25A5	662.0606922	0.222222111	0.129785784	1.596958602	0.110274925	0.727248849
2696	ATP8A2	5.614578209	-2.199690197	1.280012722	-1.592962698	0.110944166	0.748440174
2697	SLC1A2	6.75414299	1.851011291	1.160859429	1.594518021	0.110820055	0.727248849
2698	AMZ2P1	26.80868722	-0.910897428	0.571095917	-1.59499902	0.110712455	0.727248849
2699	OPN2	9.486224457	1.529122562	0.965142241	1.594709985	0.110777104	0.484870241
2700	CANT1	22.62958292	0.95752007	0.60055486	1.594408994	0.110844459	1.115782111
2701	DMXL2	98.60207256	-0.502427028	0.215787822	-1.594192908	0.110892611	0.484870241
2702	SYTL2	78.01459888	0.521592699	0.222262269	1.594624889		

						0.110792906	0.724522501
2703	PSMC2	207.0756472	0.226912927	0.211068288	1.59622712	0.110428092	1.114129191
2704	CLIC1	1710.626522	-0.172842124	0.108997299	-1.594920662	0.110727742	0.994212224
2705	AC072289.2	0.745824827	-0.972210916	2.522016256	-0.276242662	0.042077828	0.498141447
2706	TTC7B	28.52929172	-0.876288976	0.549969786	-1.592240212	0.11108289	0.740104194
2707	SMARCD1	16.52552608	-1.190724428	0.747820589	-1.592251569	0.11122819	0.992487988
2708	H2BC12	9.469929564	1.52629262	0.964972117	1.59216106	0.11124852	0.478414242
2709	LRPAP1	400.2166618	0.255024245	0.160172944	1.592242977	0.11122012	0.727248849
2710	ARF4-AS1	0.498161667	0.02169065	4.170279829	0.007599166	-0.222298091	0.49521421
2711	STARD4-AS1	40.19818792	-0.720067799	0.458792297	-1.591277914	0.111547045	1.955927244
2712	AC010172.1	0.486870242	-2.292502217	4.077744474	-0.586967215	0.044842896	0.500997014
2713	AL255802.1	0.49522622	-2.415608216	4.416671772	-0.546929552	-0.062429286	0.498141447
2714	MAP2K4	20.20582108	1.045118665	0.657019614	1.590696294	0.111677941	0.489705489
2715	ALDH2A2	71.82021498	0.560979219	0.252878774	1.589722196	0.111897428	0.498141447
2716	HMOX2	107.9872956	0.455224272	0.286291727	1.589900175	0.111857208	0.748440174
2717	NIFK	114.8467165	0.446876284	0.281161562	1.589292226	0.111971642	0.49521421
2718	SRSF2	492.2224768	0.2271672	0.149222862	1.589248954	0.111981622	0.745814817
2719	MVK	16.50587997	-1.189510869	0.749255124	-1.587591215	0.112278806	0.500997014
2720	DCAF10	20.21221799	-1.062008299	0.669772421	-1.587118796	0.112485742	0.498141447
2721	KCTD5	20.19685804	1.05025924	0.661096591	1.588662466	0.112126617	0.489705489
2722	MED21	57.69522546	-0.620128262	0.296864206	-1.587792129	0.112222122	0.748440174
2723	NCDN	21.06841025	0.822871856	0.524452124	1.58807986	0.112268282	0.49521421
2724	SLC25D1	72.88181084	-0.57682162	0.262410702	-1.587271996	0.112451055	0.478414242
2725	STMP1	226.5805689	-0.220445718	0.201742896	-1.588278758	0.112200718	0.498141447
2726	SEMA4D	128.1029895	0.418962211	0.264025599	1.58676827	0.112565128	0.724522501
2727	ATP1B2	521.9872081	-0.227172781	0.142198827	-1.586422084	0.112642594	0.972740484

2728	HMGB1P22	2.766218974	2.265959467	2.122000255	1.585472871	0.112858926	0.498141447
2729	AL078590.2	18.68275674	-1.065166722	0.671754447	-1.585648922	0.11281897	0.727248849
2730	AC021086.1	1.215782212	-1.942095285	2.757929924	-0.704182251	-0.028285723	0.489705489
2731	AP001281.1	0.498161667	0.02169065	4.170279829	0.007599166	0.056521288	0.478414242
2732	C1S	9.475651292	1.522881212	0.967275292	1.584577429	0.112062277	0.740104194
2733	AC011622.1	0.500997014	2.484998858	4.296126822	0.565270056	0.029582487	0.954818717
2734	CEACAM1	212.0209242	0.262952296	0.229212227	1.582470054	0.112214267	0.500997014
2735	ICAM2	8.740661962	1.582440192	0.999798929	1.582758422	0.112476526	0.727248849
2736	DCP1B	24.62967827	0.921610527	0.582427986	1.582222454	0.112572696	0.489705489
2737	NIPA2	87.52561742	-0.572018666	0.261262815	-1.582942955	0.112424229	0.994212224
2738	WDR41	146.1207899	-0.291216486	0.247266092	-1.582572265	0.112518966	1.971899245
2739	ASL	65.52426119	-0.586442541	0.270862591	-1.581296024	0.112810268	0.478414242
2740	ABCD2	0.489705689	0.05402189	4.196172952	0.012876469	0.026418943	0.945184705
2741	TCEA2	5.417297487	-2.125642719	1.245265014	-1.579974726	0.114112655	1.119909884
2742	HVCN1	11.04605122	-1.411242641	0.892427412	-1.579582875	0.114202424	1.950214715
2743	LIMK1	111.1817501	0.450121178	0.284979862	1.579484159	0.114225047	1.970044018
2744	ST6GALNAC2	50.12252969	0.660021802	0.418012299	1.57895207	0.114246822	0.489705489
2745	XRN2	258.771894	-0.224050979	0.20524984	-1.57881224	0.114279107	0.489705489
2746	AC092119.2	0.742989481	-2.000642057	2.742921251	-0.801470644	0.152592227	0.484870241
2747	UNC5C	2.762221292	2.27771214	2.141156522	1.577517612	0.114676472	1.440411014
2748	ITGB2	11.09106527	-1.416651749	0.898217218	-1.577005942	0.114794158	0.751495511
2749	GAB1	11.84509265	-1.284989867	0.878281741	-1.576921185	0.11481126	0.498141447
2750	CPNE2	70.22792814	0.556770498	0.252151757	1.576575755	0.114892175	0.489705489
2751	PHACTR4	68.18897422	-0.569101885	0.261120147	-1.57589128	0.115050828	0.478414242
2752	KLHL20	104.9412442	-0.464141488	0.294502129	-1.576020771	0.115021016	0.985021009
2753	EEF1A1P22	2.771787272	2.268482157	2.128849772	1.574904045

0.115278595	0.71891187
2754	OAT	217.5262842	-0.281027028	0.17842167	-1.575040114
0.115247186	0.714077514
2755	CFAP97	15.17726795	1.194725657	0.758902425	1.574294142
0.115419462	0.498141447
2756	CHPT1	22.27617048	-0.974052166	0.619228548	-1.572985252	0.11572221
0.498141447
2757	TOB1	90.04667549	-0.485254974	0.208727172	-1.572065222
0.115925424	0.478414242
2758	AIF1	219.0028262	-0.225779805	0.207246284	-1.571944456
0.115962422	0.714077514
2759	AC006449.7	6.250766557	2.026549084	1.295810298	1.57164128
0.116022744	0.498141447
2760	AC007546.2	2.779148299	2.741529496	1.746657422	1.569591988
0.116510064	0.478414242
2761	MLH2	21.68082799	-0.976210404	0.622228017	-1.569055678
0.116624966	0.489705489
2762	TMEM189	20.2720829	-0.846848689	0.529259926	-1.570290517
0.116224287	0.717411545
2763	SLC25A27	29.21828624	-0.802898681	0.511642429	-1.569254258
0.116588682	0.741989481
2764	TTC28	24.88029482	-0.927889206	0.59075222	-1.570688094
0.116255116	0.489705489
2765	SMIM14	47.56645818	-0.668027912	0.425461859	-1.57014759
0.116280779	1.119859812
2766	PTPN12	22.08984799	0.805529681	0.512171672	1.569727489
0.116478522	0.751495511
2767	OTULINL	68.18250602	-0.569216858	0.262647892	-1.569888779
0.116440988	0.740104194
2768	AC007721.4	0.489705689	0.05402189	4.196172952	0.012876469	-
0.045882477	0.478414242
2769	AC010122.4	0.49522622	-2.415608216	4.416671772	-0.546929552
0.055251445	0.49521421
2770	MYO9B	59.54517189	-0.646720722	0.412485675	-1.567886504
0.116907622	0.489705489
2771	CDK1	68.50821257	0.558798872	0.256486981	1.567515512
0.116994242	0.500997014
2772	AC092755.1	0.49522622	-2.415608216	4.416671772	-0.546929552	-
0.046869745	1.97011408
2773	AC004690.2	0.478414262	-2.271055522	4.479204761	-0.529247421
0.025745516	0.484870241
2774	FAR2P4	22.9026176	-0.971478021	0.620121586	-1.566567568
0.117215804	0.999158481
2775	NAA50	108.2170844	0.462550405	0.295456205	1.565546425
0.117454841	0.49521421
2776	ANXA8	2.760009257	2.724222287	1.747910206	1.564242729
0.117726865	1.14110111
2777	SLC29A14	19.922958	-1.029421666	0.658055259	-1.564254201
0.11772441	0.727248849
2778	UBE2R2	52.5257694	-0.625954546	0.400070867	-1.564609167
0.117674578	1.141151148

2779 PRDX2 68.25098047 0.568628948 0.262482916 1.564289748
0.117726067 0.489705489
2780 AC002401.2 0.486870242 -2.292502217 4.077744474 -0.586967215 -
0.027212298 0.500997014
2781 NFAT5 104.491529 -0.452845812 0.2902282 -1.562162052
0.118014482 0.974574021
2782 CD55 229.1476478 -0.222418741 0.207006847 -1.5622577
0.118202746 1.471901251
2783 DHPS 94.09568261 0.470618071 0.201212274 1.561894789
0.118212776 0.49521421
2784 AL128920.1 0.982196662 -2.404284457 2.098256189 -1.098806625 -
0.022496873 0.478414242
2785 AC092747.2 0.486870242 -2.292502217 4.077744474 -0.586967215 -
0.026989295 1.929015289
2786 IL12RB1 20.6196765 1.02880277 0.659241872 1.560584686
0.118621774 0.498141447
2787 AP005229.1 220.2524844 0.274402467 0.175826609 1.560559269
0.118627752 0.498141447
2788 EIF2M 241.2729201 0.266114195 0.170612261 1.559759174
0.118816802 0.489705489
2789 PPIAP41 2.774521421 2.269642795 2.162726827 1.558052888
0.119220711 0.974574021
2790 RNF44 15.46712441 -1.160252968 0.744272864 -1.558820884
0.119026412 0.990701702
2791 AP001218.2 9.982015507 1.445774688 0.927968742 1.557999228
0.119222405 1.451155047
2792 BCL2L11 9.982015507 1.445774688 0.927968742 1.557999228
0.119222405 1.711109522
2793 TNS2 52.05282071 -0.620822422 0.29812401 -1.559271997 0.11890826
0.727248849
2794 GALNT2 92.22857622 -0.476500107 0.205742798 -1.558499857
0.119114802 1.118418559
2795 AP006587.5 0.500997014 2.484998858 4.296126822 0.565270056 -
0.025507079 0.740104194
2796 PCYT2 11.55241545 -1.227465289 0.859245458 -1.556276862
0.119618504 0.500997014
2797 AC009902.2 7.98926764 1.629021225 1.052794286 1.556828977
0.119508721 0.974574021
2798 IER2IP1 71.60222928 -0.526250278 0.244688656 -1.556042722
0.119697921 0.478414242
2799 RWDD1 67.55006849 0.562296282 0.261271224 1.556007161 0.11970629
0.979411277
2800 RARA 25.90222962 -0.892021252 0.5724472 -1.555541812
0.119817084 0.717411545
2801 SLC24A1 19.92918689 -1.024456222 0.66521704 -1.554822012
0.119986082 0.748440174
2802 BCKDK 156.5802282 0.274219708 0.240650105 1.555026546
0.119927266 0.500997014
2803 ABCC12 0.498161667 0.02169065 4.170279829 0.007599166
0.028004041 0.999158481
2804 TUT1 17.42084266 1.08210621 0.697202764 1.552277447

0.120256866	0.714077514
2805	SF1	98.74482281	0.458909242	0.295295508	1.552541705
0.120292774	1.722491182
2806	SLC5A10	21.66092825	-0.972551262	0.626980784	-1.552760925
0.120480261	0.992487988
2807	KAT7	124.2200482	-0.418545626	0.269620946	-1.552290799
0.120592658	1.117074528
2808	NHSL2	8.842400764	-1.527050149	0.990778126	-1.551256564
0.120816258	0.724522501
2809	JUNB	50.42222626	-0.742015257	0.47826771	-1.55114014
0.120868104	1.744982508
2810	IKBKG	25.28729014	0.754792809	0.486501688	1.551470072
0.120789074	0.489705489
2811	HLA-A	1069.589515	0.191421406	0.122299051	1.551219914
0.120825027	0.484870241
2812	SLC16A5	14.05295798	-1.208681911	0.842879929	-1.550791588
0.120951627	0.500997014
2813	MYO10	45.80267172	0.682500904	0.440827942	1.550458422
0.121021522	0.49521421
2814	LILRB1	101.551192	0.485599278	0.212208196	1.549909272
0.121162294	0.740104194
2815	ATXN7L2B	192.7424789	0.252076885	0.227799208	1.549947816
0.121154042	0.49521421
2816	JKAMP	144.1242742	0.282502604	0.247527062	1.549240102
0.121299984	0.71891187
2817	AC048251.1	0.478414262	-2.271055522	4.479204761	-0.529247421
0.029400788	0.498141447
2818	DNAJB9	122.1728567	-0.414262701	0.267452405	-1.54891915
0.121401157	0.945184705
2819	SIGLEC17P	210.8227492	0.279764214	0.180704089	1.548189722
0.121576625	0.948110051
2820	PAQR6	2.892967622	-2.290515527	2.190481252	-1.547840467
0.121660711	0.727248849
2821	AC098476.1	0.498161667	0.02169065	4.170279829	0.007599166
0.092872222	0.981194441
2822	ZMIZ1-AS1	10.75552655	1.41466522	0.914674229	1.546622796
0.121951817	1.720854827
2823	SMAP1	78.77022102	-0.510481222	0.229929287	-1.547197207
0.121815675	0.49521421
2824	ATP5PD	154.7792041	0.272988811	0.241749247	1.547011274
0.121860527	1.714780114
2825	AKAP12	252.9240767	0.210288089	0.200698422	1.546529761
0.121974265	0.489705489
2826	P2RX4	92.04802166	-0.474716952	0.207018056	-1.54621825
0.122051842	0.500997014
2827	AC060772.1	0.489705689	0.05402189	4.196172952	0.012876469	-
0.057541525	0.985021009
2828	AC069544.1	1.947481268	-4.29251276	2.409426957	-1.822468225
0.022569244	0.500997014
2829	GOLT1B	67.24584642	0.555716812	0.259876425	1.544187879
0.122542824	1.142984495

2830 ATP5IF1 169.9992058 -0.256884644 0.22114627 -1.542977518
0.122592779 0.972740484
2831 IFNGR1 206.2206852 0.276227189 0.178884965 1.544216918
0.122525792 0.981194441
2832 PDHA1 122.8578229 0.405806019 0.262896588 1.542595608 0.12268622
0.49521421
2833 MMACHC 445.9612122 -0.245021402 0.158754615 -1.54229704
0.122724472 0.49521421
2834 DCAKD 55.72559097 -0.598912644 0.288215602 -1.542722026
0.122895808 1.144811841
2835 MDM2 127.2855662 0.406614047 0.262629624 1.542268574
0.122984054 0.974574021
2836 NSL1 47.72489946 0.656764642 0.425929129 1.541957528
0.122082915 1.952151041
2837 AP000944.1 0.727268849 -0.952727871 2.205072418 -0.288262241 -
0.118662464 0.717411545
2838 PTENP1 4.409985224 -2.275422001 1.476269226 -1.541228279
0.122261227 0.498141447
2839 PTGIR 20.22264752 -1.057464081 0.68701885 -1.529206792 0.12275282
0.751495511
2840 RHOG 420.1677528 -0.227166955 0.154096158 -1.529084158
0.122782752 0.49521421
2841 TMEM167A 120.2552921 0.421829566 0.280651822 1.528702111
0.122877027 0.49521421
2842 FAR2P1 2.675250524 -2.649200472 1.722090054 -1.527528726
0.124162884 0.49521421
2843 FAM114A1 9.587972059 -1.485666549 0.96622026 -1.527590422
0.12414879 0.745814817
2844 AASS 11.58051752 -1.240206615 0.87142999 -1.528054267
0.124025247 0.489705489
2845 DNASE1 74.29284508 0.527147587 0.249172127 1.528241714
0.122965085 0.987847254
2846 AGAP7P 0.982196662 -2.404284457 2.098256189 -1.098806625
0.026820292 0.727248849
2847 AP2B1 222.8621528 -0.212550728 0.202275862 -1.526812286
0.124229029 0.500997014
2848 MAPK12 9.579516081 -1.484249714 0.966121558 -1.526281162
0.124469427 0.498141447
2849 AC090517.2 0.990652641 -2.415742141 2.252204524 -1.018619724 -
0.017172656 0.478414242
2850 AC016065.2 0.489705689 0.05402189 4.196172952 0.012876469
0.021052441 0.974574021
2851 AC026992.2 0.748660174 1.029647608 2.51687248 0.292772655
0.026717127 0.489705489
2852 AL025420.1 0.500997014 2.484998858 4.296126822 0.565270056
0.056599909 0.71891187
2853 ADGRL2 0.500997014 2.484998858 4.296126822 0.565270056 -
0.019772289 0.49521421
2854 AL109918.1 2.682806502 -2.652548629 1.728127294 -1.524926644
0.124801848 0.498141447
2855 TSTD1 85.89240461 -0.486290908 0.217065916 -1.524027192

						0.125020502	0.724522501
2856	TBC1D22A	29.2728189	-0.829211297	0.547188924	-1.522677604	0.125108986	0.745814817
2857	GNPAT	106.2125876	0.444490057	0.289882052	1.522247966	0.125190142	0.49521421
2858	AC027796.4	12.21211511	1.264714066	0.82515597	1.522696982	0.125250522	0.972740484
2859	AL596202.1	45.02860665	0.677228222	0.44200472	1.522422245	0.125418025	0.714077514
2860	AC082798.2	66.44952927	0.574482009	0.275098254	1.521552001	0.125622778	0.500997014
2861	STARD4	100.2054672	-0.451800125	0.295000205	-1.521524815	0.125629728	0.748440174
2862	NPRL2	120.2200794	-0.402266621	0.262684999	-1.521265071	0.125679192	0.990451441
2863	DOK2	256.2892527	-0.295415126	0.192825175	-1.52195669	0.125522122	0.945184705
2864	ARPC5	806.265949	0.201249981	0.121291289	1.522850976	0.125212577	0.714077514
2865	AC015812.6	0.745824827	-0.972210916	2.522016256	-0.276242662	0.02242612	0.979411277
2866	MAGEB2	2.692206195	-2.278181948	2.144020049	-1.528981245	0.126269069	0.745814817
2867	PEG12	2.692206195	-2.278181948	2.144020049	-1.528981245	0.126269069	0.740104194
2868	RTL8A	27.95822282	-0.717120569	0.468552824	-1.520497709	0.12589258	0.498141447
2869	NAGK	192.0010122	-0.226417678	0.219901828	-1.529852871	0.126052904	0.484870241
2870	ANP22E	161.1557961	0.272862044	0.242818049	1.529262507	0.126199122	0.489705489
2871	IST1	255.7597642	-0.255115026	0.166762755	-1.529808224	0.126064202	0.500997014
2872	NRP1	542.9746192	-0.21861217	0.142910221	-1.529716722	0.126086862	0.498141447
2873	C2	28.05500199	-0.826176218	0.547021787	-1.528569706	0.126271151	0.748440174
2874	AC027176.2	2.696040244	-2.275290517	2.142270142	-1.528174277	0.126469249	0.745814817
2875	MAP1LC2A	7.264928917	-1.668558716	1.092797042	-1.526869721	0.12679241	0.478414242
2876	POU2F2	11.21764722	1.246022419	0.88162584	1.526724008	0.126827167	0.724522501
2877	ECE1	25.21875246	-0.762121245	0.499212668	-1.52666242	0.126844728	0.489705489
2878	TRNT1	112.1152702	-0.424802026	0.278171208	-1.527124252	0.126720092	1.121495149
2879	GART	162.6546649	0.257260052	0.222915445	1.527204248	0.126685274	0.484870241
2880	MCOLN1	46.62462276	0.675609722	0.442721206	1.52600295	0.127008881	0.717411545

2881 ERAP1 276.2182051 -0.281890229 0.184722709 -1.525926207 0.127025752 0.500997014
2882 PRKAR1A 460.2142828 -0.28816256 0.18888629 -1.525592781 0.127111211 0.500997014
2883 OSBPL8 111.6182227 -0.428160822 0.280722608 -1.525155292 0.127220269 0.49521421
2884 AL162051.1 4.252195662 2.275895076 1.494815175 1.522526071 0.127877218 0.741989481
2885 OPHN1 11.55788274 -1.225929775 0.877040592 -1.522225967 0.127699681 0.498141447
2886 DNMBP 21.42216482 -0.951218516 0.624681768 -1.522885026 0.12778747 1.987014027
2887 DDA1 22.08692192 0.777492945 0.510485254 1.522048785 0.127746501 0.478414242
2888 SEPTIN8 52.95782726 -0.61692517 0.405087402 -1.522968045 0.1277667 0.751495511
2889 PLXND1 77.50220268 -0.507996569 0.222687165 -1.522272716 0.127915467 0.49521421
2890 TRIAP1 57.56101128 0.607922754 0.299262427 1.522229271 0.127951618 0.741989481
2891 CADM1 27.04227277 -0.871416782 0.572269974 -1.52008098 0.128490624 0.751495511
2892 TK2 52.44027202 -0.622422029 0.409762564 -1.521442227 0.128148622 0.714077514
2893 IP6K1 48.27901791 -0.641010185 0.421462822 -1.520917502 0.128280541 0.49521421
2894 TYMS 74.5276044 -0.554414654 0.264526942 -1.520872714 0.128291521 0.49521421
2895 ABALON 68.02269762 0.52780758 0.252712825 1.520462898 0.128294425 0.974574021
2896 IREB2 96.68208952 0.472059025 0.210265274 1.520978417 0.128265254 0.751495511
2897 USP22 122.8401228 -0.414010296 0.272275918 -1.520554587 0.12827165 0.478414242
2898 SRSF7 204.0694616 0.220458202 0.210820224 1.519981548 0.128515612 0.727248849
2899 DAGLA 4.249461514 2.274982407 1.497422221 1.519266482 0.128695422 0.500997014
2900 ATXN2L 87.06525694 0.511242227 0.226584022 1.519211262 0.128709227 1.494485001
2901 GPC6 5.167000178 -2.044271612 1.246201255 -1.518622572 0.128857526 0.745814817
2902 CC2D1B 64.58928218 -0.552711846 0.262912727 -1.518802245 0.128812029 0.500997014
2903 MARCHF2 71.24859075 -0.527082141 0.247150517 -1.518210115 0.128926229 0.748440174
2904 PTPRS 5.88745707 -1.878759856 1.228008722 -1.517565915 0.129122865 0.484870241
2905 ZNF525 4.428087208 -2.281200152 1.502281615 -1.517279207 0.129170945 1.107217124
2906 UBA1 272.642122 -0.270059026 0.177951652 -1.517597725

0.129115828 0.717411545
2907 SRD5A2-AS1 16.00482182 -1.124542752 0.741410812 -1.516762009
0.129226782 0.49521421
2908 AC129792.1 0.724522502 -2.98570617 2.452708589 -0.86474217
0.020091012 0.985021009
2909 ZCCHC18 4.26228579 2.268442787 1.496049507 1.516289251
0.129446228 0.49521421
2910 PRKAR1B 4.257917492 2.266597877 1.496211987 1.514789627
0.129825688 0.500997014
2911 N4BP2 22.66844181 -0.926822495 0.61182248 -1.514846824
0.129811197 0.489705489
2912 ACOX1 99.61274927 0.488515664 0.222482042 1.514861608
0.129807455 0.500997014
2913 VEPH1 2.649982588 -2.629272015 1.742789074 -1.514452502
0.129910859 0.484870241
2914 ACVR1 57.52898207 -0.595540422 0.292298854 -1.514218556
0.129970417 0.49521421
2915 CHAMP1 48.99020768 -0.642028287 0.42477727 -1.512801072
0.120076201 0.498141447
2916 TRPM2 17.19757528 -1.072021268 0.708640716 -1.512799424
0.120220615 0.489705489
2917 GUSB 205.5168799 -0.285244008 0.188525285 -1.512027862
0.120272582 0.489705489
2918 BCAP21 248.2697128 0.260805959 0.172406078 1.512742254
0.120245142 0.478414242
2919 TYMSOS 7.229560982 -1.662206457 1.09998715 -1.512022522
0.120527876 1.474727498
2920 AC018521.4 17.71290254 -1.042216775 0.689605198 -1.51122284
0.120705962 0.489705489
2921 GNL2 111.2277747 0.425012218 0.281222862 1.510707648
0.120862952 0.727248849
2922 AC079212.1 2.50080124 2.646076742 1.752251552 1.510100955
0.121017666 0.478414242
2923 ARSB 74.12502262 0.552725245 0.266020152 1.510122712
0.121012115 0.49521421
2924 AC022919.1 0.956828727 -2.270922824 2.294506224 -0.992055722
0.010485867 0.71891187
2925 AC068789.1 6.620294217 -1.757672658 1.164900252 -1.508861085
0.121224286 0.71891187
2926 HMGN2 118.7059652 -0.414587205 0.274778941 -1.508802688
0.121249214 0.489705489
2927 AC011498.6 0.748660174 1.029647608 2.51687248 0.292772655
0.010222998 0.974574021
2928 DNAH2 2.502525489 2.647211702 1.755261109 1.508158252
0.121514004 0.484870241
2929 METRNL 14.21714795 1.162415451 0.771296406 1.508194026
0.121504876 0.478414242
2930 KCTD12 15.46271626 1.118898086 0.742180977 1.507581197
0.121661748 0.478414242
2931 PFN1 1225.169876 0.169241221 0.112218769 1.507685064 0.12162515
0.478414242

2932	EFCAB7	2.498067191	2.645016652	1.75574252	1.506492758	0.121940468	0.489705489
2933	UNC92B1	2.498067191	2.645016652	1.75574252	1.506492758	0.121940468	0.994212224
2934	ZNF85	24.57916165	-0.902496716	0.599028809	-1.506599854	0.121912255	0.500997014
2935	C11orf49	72.20766704	-0.511405787	0.229264508	-1.506951202	0.12182214	0.498141447
2936	TMEM222	7.25272879	-1.668094578	1.107557101	-1.506102552	0.122040849	0.478414242
2937	GSTK1	240.7708844	0.201872292	0.200517775	1.505468984	0.122202545	0.745814817
2938	BCL7B	40.17242826	-0.692910425	0.460710644	-1.50400251	0.122580462	0.484870241
2939	MRPS12	62.97400259	0.580610896	0.286242286	1.502229262	0.122779908	0.748440174
2940	RNASE2	111.1281498	-0.472660925	0.215097776	-1.502218908	0.122782602	0.489705489
2941	WDR22	124.2275848	0.402469092	0.268254162	1.502494812	0.122711494	1.491241119
2942	PSMF1	195.81207	-0.22294729	0.22128201	-1.502942275	0.122596205	0.990701702
2943	EEF1A1	5825.594108	0.125915978	0.082765459	1.502196886	0.12278828	0.740104194
2944	AP002206.2	12.0786792	-1.276100749	0.849281922	-1.50228746	0.122997072	0.484870241
2945	MIRLET7A1HG	4.997868156	2.056879587	1.268944714	1.502529222	0.122960459	0.727248849
2946	PDE4B	21.9212265	-0.927575659	0.617427129	-1.50229975	0.122019712	0.489705489
2947	SVIP	59.91755807	-0.564025285	0.275511727	-1.502044252	0.122085655	0.49521421
2948	AC027290.2	4.992299859	2.055266472	1.269112072	1.50116625	0.122212541	0.484870241
2949	AQP4-AS1	2.506269628	2.64842159	1.765152149	1.500297624	0.122511425	0.990451441
2950	CUBN	2.506269628	2.64842159	1.765152149	1.500297624	0.122511425	0.748440174
2951	IRGM	14.18084242	1.167569622	0.777900972	1.500922221	0.122275421	0.748440174
2952	TAF10	157.2977086	-0.267256702	0.244825112	-1.500486204	0.122488504	0.71891187
2953	SNRPG	147.850612	0.269557204	0.246224024	1.500827621	0.122297562	0.714077514
2954	TP72-AS1	8.08265166	-1.594550864	1.062201179	-1.49976401	0.122675542	0.498141447
2955	TAF2	24.56997226	0.745206945	0.49692265	1.499640728	0.122707492	0.741989481
2956	ST6GAL1	279.5222872	0.265225056	0.176958269	1.498855678	0.122911077	0.489705489
2957	TNFAIP8	172.0806845	-0.250942228	0.224198642	-1.498485744		

0.124007094 0.741989481
2958 AC025154.2 0.500997014 2.484998858 4.296126822 0.565270056 -
0.022292992 0.478414242
2959 SLC16A2 109.6022828 -0.421615419 0.28145482 -1.497986089
0.124126865 0.724522501
2960 LUCAT1 4.268854088 2.270211198 1.517066767 1.496447782
0.124527006 1.111402844
2961 RIT1 126.7957592 0.296485461 0.264940182 1.496509249
0.124520974 0.498141447
2962 MAFF 4.99528754 2.046882781 1.268597266 1.495606986
0.124756102 0.489705489
2963 KRI1 60.2917874 0.566856069 0.279280688 1.494555581
0.125020466 0.478414242
2964 ABCA17P 0.717621545 -2.955950852 2.792242589 -0.779267242 -
0.010554514 0.751495511
2965 TUBA2FP 6.604926282 -1.751756429 1.172215527 -1.492996874
0.125428005 0.717411545
2966 DDN-AS1 8.482214542 1.519722105 1.020200127 1.489622228
0.126220927 0.999158481
2967 POGLUT2 11.74066976 1.282294562 0.860888792 1.489500819
0.126255528 0.972740484
2968 KLHL26 22.52989982 -0.75882442 0.508977574 -1.490899527
0.125987872 0.489705489
2969 ARHGAP25 24.27751628 -0.729245105 0.495851761 -1.49085909
0.125998494 0.49521421
2970 GIMAP6 52.29555224 -0.619618246 0.415709987 -1.490506027 0.12609122
0.500997014
2971 ZNF92 28.28047222 0.828227221 0.555576192 1.490771819
0.126021412 0.994212224
2972 CDC20 45.24449699 0.65776517 0.440687527 1.49258858
0.125544915 0.478414242
2973 CCM2 72.7291224 -0.512429742 0.242825104 -1.492272191 0.12526569
0.500997014
2974 DPP7 110.9447682 -0.497012242 0.222524942 -1.490180426
0.126176799 0.748440174
2975 MRPL22 56.86946821 0.571612757 0.282779526 1.489420007
0.126274172 0.990701702
2976 TRAM1 96.72567981 -0.456605246 0.206194929 -1.491224061
0.125902679 0.751495511
2977 ALG8 79.90744722 0.500254892 0.225047766 1.492085298 0.12541486
0.489705489
2978 PLTP 141.5825262 -0.27226959 0.249570616 -1.491640209
0.125792465 0.498141447
2979 SUPT5H 127.5762228 0.289796888 0.261249072 1.492050804
0.125685827 0.748440174
2980 PRDX5 198.4172741 -0.2217612 0.222744822 -1.48942272
0.126276089 0.987847254
2981 GLRX2 140.5714079 0.285975548 0.258520096 1.492961762
0.125447196 0.489705489
2982 TMBIM1 294.8700195 -0.242619216 0.162122264 -1.492274555 0.12522917
0.745814817

2983	SH2BGRL2	2249.959188	-0.12080148	0.087679561	-1.491812675	0.12574826	0.489705489
2984	APBB2	5.127454425	-2.020020577	1.264028402	-1.488250212	0.126684892	0.974574021
2985	TMEM147	82.51285692	-0.544722425	0.265841612	-1.488987054	0.126490778	0.500997014
2986	GORASP1	81.14872102	-0.491687721	0.220427849	-1.487988526	0.126752917	0.49521421
2987	UBA6	81.71028518	-0.487460122	0.2275272	-1.48825825	0.126682774	0.727248849
2988	CPSF2	108.9282827	-0.419574946	0.281920825	-1.488272217	0.126679119	0.500997014
2989	SERPINB2	22.64810672	-0.884487912	0.594752287	-1.487150945	0.126974949	1.117074528
2990	GRB10	27.2889228	-0.885822889	0.595542422	-1.48742792	0.126899185	0.979411277
2991	RPS12	920.2585754	0.1724201	0.115940857	1.487128292	0.126978264	0.748440174
2992	POLE4	18.24190044	-1.012111869	0.681007856	-1.486197054	0.127227007	0.727248849
2993	AC008906.1	22.82752826	-0.775529665	0.521941277	-1.485856166	0.12721717	0.498141447
2994	HDX	5.921280984	-1.887572428	1.270957829	-1.48515741	0.127502121	0.484870241
2995	METTL5	107.7169486	0.42275261	0.284728107	1.484762477	0.127606755	0.740104194
2996	EIF5	292.2559226	0.267629202	0.180229266	1.484992018	0.127545674	0.981194441
2997	ARL6IP1	260.115082	0.282148717	0.190801252	1.482997425	0.127809604	0.498141447
2998	AL078621.2	1.494485002	0.02221681	2.582522648	0.008989972	0.002985473	0.478414242
2999	TMEM14C	109.4088502	-0.417805511	0.281817268	-1.482529964	0.128196678	0.484870241
3000	MYO1E	11.52110868	1.282602916	0.922776297	1.482245978	0.128274857	0.714077514
3001	AC009120.2	110.6621216	0.412500151	0.279074187	1.481685411	0.128424021	0.478414242
3002	AEN	58.79507521	0.579602901	0.291275002	1.480942568	0.128621878	0.745814817
3003	EFTUD2	260.1957877	0.295792717	0.199766202	1.480698765	0.128686862	1.482192474
3004	SELPLG	404.9255751	0.240244292	0.162227989	1.480905947	0.128621628	0.498141447
3005	AC004622.1	2.664261594	-1.95178262	1.649828527	-1.182014229	0.226802468	0.49521421
3006	AC004771.1	2.220562525	-0.962970587	2.017761874	-0.477246894	0.622186222	0.972740484
3007	AC005182.1	2.427227118	-0.520704067	2.122567672	-0.244052222	0.8071896	0.740104194
3008	AC006001.2	4.897006825	-0.841579595	1.452102252	-0.579160615		

							0.562480802	0.478414242
3009	AC006116.8	8.12075458	-1.278594218	1.191827208	-1.156706252		0.247292252	0.489705489
3010	AC007192.2	2.212106547	-0.956160282	2.008064151	-0.476160277		0.622960197	0.49521421
3011	AC009065.8	2.454099012	-2.129062785	2.206078482	-1.418282805		0.156079022	0.717411545
3012	AC009127.1	2.197979875	-2.952128802	2.262081079	-1.204472282		0.192072264	0.500997014
3013	AC010219.4	2.672767624	-0.520212644	1.655091145	-0.22025214		0.748700644	0.498141447
3014	AC010226.1	2.458928288	-0.276829216	1.668659927	-0.225822441		0.821220967	0.49521421
3015	AC011466.1	2.686844245	-0.956221557	1.610259746	-0.59285585		0.55260852	0.484870241
3016	AC011498.7	2.227524544	1.026898822	2.077770869	0.499042872		0.617748477	0.71891187
3017	AC011502.1	2.227524544	1.026898822	2.077770869	0.499042872		0.617748477	0.714077514
3018	AC012640.1	2.202650568	-0.949279418	2.021841102	-0.469561825		0.628668097	0.489705489
3019	AC015921.1	2.966287252	-0.966622429	1.798860811	-0.527258101		0.591020279	0.741989481
3020	AC024267.7	2.68962952	-1.962275757	1.658517494	-1.182210768		0.226725627	0.484870241
3021	AC026247.2	2.206425852	-2.957411757	2.267414564	-1.204210118		0.192127867	0.714077514
3022	AC027021.2	2.664261594	-1.95178262	1.649828527	-1.182014229		0.226802468	0.484870241
3023	AC074011.1	2.202650568	-0.949279418	2.021841102	-0.469561825		0.628668097	0.498141447
3024	AC074212.1	2.45692426	-1.960419854	1.984222808	-0.988002891		0.222150742	0.498141447
3025	AC090971.4	5.648452284	-1.148966428	1.251457147	-0.850168606		0.295221252	0.484870241
3026	AC091221.1	1.995482015	0.760808082	2.211199621	0.229182196		0.742017222	0.484870241
3027	AC092010.2	2.440022402	-1.949961767	1.987291022	-0.981166622		0.226510576	0.741989481
3028	AC104758.1	5.440282722	-0.495410259	1.407245214	-0.252042596		0.724806217	1.708174184
3029	AC105749.1	2.229068565	1.04862714	2.080227779	0.504097268		0.614192027	0.49521421
3030	AC107027.2	2.459769707	-1.181222177	1.925024222	-0.61266662		0.52942565	0.751495511
3031	AC120498.7	2.212106547	-0.956160282	2.008064151	-0.476160277		0.622960197	0.489705489
3032	AC244024.2	2.704647582	-0.212171868	1.854494926	-0.114948746		0.908485747	0.985021009
3033	AC245100.4	2.22185285	-0.290592504	2.042846725	-0.142248804		0.886882484	0.751495511

3034	AJ002147.2	2.181067918	-2.941501212	2.292454667	-1.282122958	0.199449002	1.104491887
3035	AL022476.1	2.189522896	-2.946842151	2.270851262	-1.297681652	0.194296751	0.489705489
3036	AL021670.1	2.925248724	-2.276771217	1.856261046	-1.226470046	0.220021841	0.745814817
3037	AL025587.1	2.45692426	-1.960419854	1.984222808	-0.988002891	0.222150742	0.500997014
3038	AL122445.2	2.220562525	-0.962970587	2.017761874	-0.477246894	0.622186222	0.489705489
3039	AL157871.6	2.212106547	-0.956160282	2.008064151	-0.476160277	0.622960197	0.990701702
3040	AL161752.1	2.2092712	-1.765694694	2.057669202	-0.858104211	0.290824922	0.484870241
3041	AL162258.2	2.222297872	-0.282525995	2.007148456	-0.140764872	0.888055695	0.498141447
3042	AL254942.1	2.202650568	-0.949279418	2.021841102	-0.469561825	0.628668097	1.451155047
3043	ANOS2P	2.92221608	-1.540741529	1.577451008	-0.976728615	0.228702525	0.71891187
3044	AP000462.2	2.679229585	-2.117940805	1.927709541	-1.098682525	0.271906562	0.748440174
3045	AP000787.1	2.720015518	-0.222267247	1.892726758	-0.122178228	0.901965878	0.478414242
3046	AP001272.2	2.472846217	-1.971272401	2.05222224	-0.960504027	0.2268016	1.142984495
3047	AP002486.1	2.456984422	0.054876522	1.96757081	0.027890495	0.97774949	0.745814817
3048	AP002680.1	2.189522896	-2.946842151	2.270851262	-1.297681652	0.194296751	0.741989481
3049	AP006621.5	2.180226599	-1.119155654	1.728456662	-0.647488409	0.517215894	0.500997014
3050	ARHGAP27P1-BPTFP1-KPNA2P2	2.720015518	-0.222267247	1.892726758	-0.122178228	0.901965878	0.71891187
3051	ARRDC5	2.212106547	-0.956160282	2.008064151	-0.476160277	0.622960197	1.71294488
3052	ASLP1	2.712052499	-2.126768565	1.947718114	-1.097062522	0.272614058	0.751495511
3053	ATP6V0D2	2.197979875	-2.952128802	2.262081079	-1.204472282	0.192072264	0.741989481
3054	C17orf78	2.479517011	-0.555115177	1.965642686	-0.282408996	0.777629918	1.71294488
3055	C21orf62-AS1	2.465290229	-1.965746527	2.004962441	-0.980440574	0.22686869	0.498141447
3056	C9orf116	2.696141542	-2.127262988	1.91286911	-1.111498679	0.266252758	0.489705489
3057	CCDC112	2.405207109	-2.522472448	1.777985046	-1.42424969	0.15424527	0.745814817
3058	CCDC171	2.440022402	-1.949961767	1.987291022	-0.981166622	0.226510576	0.498141447
3059	CD79A	2.28277452	-1.807824918	4.266904957	-0.422687646		

0.671792626	0.484870241
3060	CHRNE	2.222297872	-0.282525995	2.007148456	-0.140764872
0.888055695	0.484870241
3061	CLEC4E	2.214891822	-2.962742974	2.285492826	-1.296225515
0.194862262	0.500997014
3062	COL15A1	2.954254709	-0.701820525	1.587865629	-0.441996169
0.658491978	0.714077514
3063	CTDSPL	2.220612587	1.061099514	2.224799778	0.476941577
0.622402725	0.727248849
3064	CTNNA2	2.206425852	-2.957411757	2.267414564	-1.204210118
0.192127867	0.740104194
3065	CYSRT1	4.926501422	-0.248216252	1.447459041	-0.171552285
0.862788726	0.717411545
3066	DBP	2.471061022	-0.548198507	1.922290926	-0.285016684	0.77562126
0.500997014
3067	DHRS12	5.14745527	-1.622849781	1.406275926	-1.161827205	0.24520562
0.489705489
3068	DLK2	2.198029927	-0.258146765	2.214627817	-0.116564401
0.907205249	0.717411545
3069	DUX4L50	2.200815222	-1.759759081	2.059610271	-0.854412585
0.292875871	1.471901251
3070	E2F1	2.189522896	-2.946842151	2.270851262	-1.297681652
0.194296751	1.741148141
3071	EEF1A1P25	2.001152709	2.841554524	2.528640412	1.119221298
0.26200205	0.999158481
3072	FAHD2B	2.174555905	-2.412208449	1.808160427	-1.224067707
0.182181668	0.745814817
3073	FAM111B	2.462605054	-0.541475178	1.912075245	-0.282029142
0.777146821	0.489705489
3074	GCNA	2.199972902	-0.6246004	1.710625709	-0.270975601
0.710655705	0.751495511
3075	GET4	2.977678678	-0.456791961	1.826911285	-0.248672927
0.802612011	0.489705489
3076	GPR161	2.182011884	-2.416047444	1.807245522	-1.226867294
0.181265958	0.498141447
3077	GPR176	4.912918782	-0.848005674	1.422885092	-0.591816942
0.552972177	0.724522501
3078	GPRASP2	2.217727178	-1.771698107	2.072955796	-0.854260202
0.292960777	0.741989481
3079	GUCY1B1	2.712102561	-0.219785571	1.841182564	-0.11927196
0.904980675	0.987847254
3080	HACE1	2.647249627	-1.944740272	1.681229144	-1.156668448
0.247407845	0.972740484
3081	HAL	2.655805616	-1.948272249	1.661284268	-1.17268075
0.240922854	0.992487988
3082	HCAR2	2.465290229	-1.965746527	2.004962441	-0.980440574	0.22686869
0.741989481
3083	HCG27	2.451212729	-1.175452257	1.926682458	-0.610091846
0.541800968	0.498141447
3084	HERC5	2.927242752	-0.692524007	1.568906174	-0.44204201
0.658458082	0.981194441

3085	HLA-F-AS1	2.715928907	0.209840191	1.862229109	0.166292051	0.867927121	1.474727498
3086	HMCN1	2.200815222	-1.759759081	2.059610271	-0.854412585	0.292875871	0.478414242
3087	HNRNPKP4	2.927242752	-0.692524007	1.568906174	-0.44204201	0.658458082	0.71891187
3088	HSD17B8	6.182272212	-0.797471019	1.251648844	-0.589998669	0.555191542	0.484870241
3089	HSPB9	2.217727178	-1.771698107	2.072955796	-0.854260202	0.292960777	0.484870241
3090	IFITM2P4	2.70459752	-2.121968002	1.92277149	-1.108222099	0.267765477	0.954818717
3091	IL17RC	2.174605967	-0.619125969	1.794982524	-0.244925904	0.720150076	0.740104194
3092	INAFM1	4.896956764	-1.955282821	1.44828144	-1.250046864	0.177000951	0.484870241
3093	ITGA9-AS1	2.99180525	0.502210886	4.299651088	0.117025284	0.906822092	0.985021009
3094	KCNMA1	2.926892745	-2.272566571	1.861958224	-1.220525005	0.222265916	0.985021009
3095	KLHDC7A	2.200815222	-1.759759081	2.059610271	-0.854412585	0.292875871	0.500997014
3096	LILRB1-AS1	2.986184718	1.022642786	1.860717429	0.555508198	0.578547104	1.125520514
3097	LINC00628	2.451212729	-1.175452257	1.926682458	-0.610091846	0.541800968	0.489705489
3098	LINC00662	2.227524544	1.026898822	2.077770869	0.499042872	0.617748477	0.500997014
3099	LINC01122	2.220562525	-0.962970587	2.017761874	-0.477246894	0.622186222	0.500997014
3100	LINC01147	2.206425852	-2.957411757	2.267414564	-1.204210118	0.192127867	0.500997014
3101	LINC02809	2.427227118	-0.520704067	2.122567672	-0.244052222	0.8071896	0.478414242
3102	LIPC	2.45692426	-1.960419854	1.984222808	-0.988002891	0.222150742	0.741989481
3103	LOXL1-AS1	2.182011884	-2.416047444	1.807245522	-1.226867294	0.181265958	0.727248849
3104	LPIN2	2.2092712	-1.765694694	2.057669202	-0.858104211	0.290824922	1.471901251
3105	LRFN4	2.199972902	-0.6246004	1.710625709	-0.270975601	0.710655705	0.500997014
3106	LRRC27A2	2.214941894	-0.274640622	2.009245167	-0.126688462	0.891277049	0.478414242
3107	LSM11	2.709426895	-0.154567411	1.628529471	-0.094911676	0.924284982	0.49521421
3108	MIR762HG	2.925248724	-2.276771217	1.856261046	-1.226470046	0.220021841	0.478414242
3109	MOB2	2.949475296	-0.956245699	1.770822719	-0.529997454	0.589198788	0.500997014
3110	MT-TW	2.472896279	0.029229808	1.926972258	0.020262452		

						0.982752428	0.49521421
3111	MTCO2P12	2.445642025	-2.124241988	2.197526942	-1.421752666	0.155097775	0.478414242
3112	MYH2	2.188682577	-1.122751902	1.71571017	-0.654977701	0.512482111	0.484870241
3113	NKAIN2	4.407251075	-2.278296155	1.541787645	-1.477697764	0.129488692	0.981194441
3114	NUTM2D	2.240259891	1.84996921	2.160415068	0.856202725	0.291820254	1.714780114
3115	OLMALINC	2.889292166	-1.526654497	1.646552141	-0.927182601	0.252821725	0.484870241
3116	OR7E94P	2.214891822	-2.962742974	2.285492826	-1.296225515	0.194862262	0.489705489
3117	PAQR8	2.712052499	-2.126768565	1.947718114	-1.097062522	0.272614058	1.474727498
3118	PAXIP1-AS1	2.955096027	-1.547626211	1.801494244	-0.859084728	0.290292767	0.724522501
3119	PDZD7	2.942804702	-2.280995741	1.859777827	-1.226488298	0.220014976	0.489705489
3120	PIGCP1	2.445642025	-2.124241988	2.197526942	-1.421752666	0.155097775	0.498141447
3121	PIGHP1	2.194202209	-1.696710871	1.729860555	-0.975199244	0.229461288	1.48884427
3122	PIGR	2.454099012	-2.129062785	2.206078482	-1.418282805	0.156079022	1.708214148
3123	PIK2IP1-AS1	2.229018504	-0.969967282	2.059447242	-0.470984209	0.62765194	0.500997014
3124	POMGNT2	2.211215166	-1.705219556	1.781214265	-0.95722779	0.228296787	0.727248849
3125	PROK2	4.199082522	-1.229788262	1.562522241	-0.787052285	0.421250674	0.500997014
3126	PRSS26	2.229068565	1.04862714	2.080227779	0.504097268	0.614192027	0.71891187
3127	RANP1	2.202650568	-0.949279418	2.021841102	-0.469561825	0.628668097	1.494485001
3128	RARB	2.472105122	1.929262872	4.267580572	0.454417401	0.64952842	0.724522501
3129	RHEBL1	2.700920855	-1.424945112	1.642855659	-0.867258678	0.285745515	0.489705489
3130	RIMS2	2.966427414	0.522064164	1.808821804	0.294147948	0.768644852	0.498141447
3131	RPAP2P1	2.925248724	-2.276771217	1.856261046	-1.226470046	0.220021841	0.994212224
3132	RPS2AP20	2.664261594	-1.95178262	1.649828527	-1.182014229	0.226802468	1.471901251
3133	RPSAP57	2.229018504	-0.969967282	2.059447242	-0.470984209	0.62765194	1.477572045
3134	RTN2	2.45604298	-2.554018252	1.821494277	-1.402155518	0.160868811	0.500997014
3135	SCRIB	2.715888846	-1.281248879	1.879496688	-0.724956804	0.462265802	0.994212224

3136	SEMA6D	2.726228852	-0.165018689	1.699865802	-0.097077481	0.922664864	0.489705489
3137	SETP5	2.214941894	-0.274640622	2.009245167	-0.126688462	0.891277049	0.974574021
3138	SLC25A1P5	2.698976889	-1.270872542	1.852022611	-0.740198522	0.459179544	0.954818717
3139	SLC2A12	2.946640049	-1.542965651	1.791042842	-0.861489604	0.288968444	0.500997014
3140	SPR	4.717041555	0.182591059	1.520244976	0.119974946	0.904502994	0.500997014
3141	SPTA1	2.468225685	-1.187262018	1.942011896	-0.611256718	0.54096245	0.489705489
3142	SRRM1P2	2.191517924	-0.629515226	1.718885967	-0.26622449	0.714190108	0.748440174
3143	TCF7	5.161521881	-2.049076776	1.424205795	-1.428619185	0.152112717	0.49521421
3144	TICRR	2.715888846	-1.281248879	1.879496688	-0.724956804	0.462265802	0.478414242
3145	TMEM45B	2.472896279	0.029229808	1.926972258	0.020262452	0.982752428	1.480408291
3146	TMSB4Y	2.718724192	-0.772515289	1.855212065	-0.416280297	0.677121744	0.478414242
3147	TNS2	2.424401772	-1.162580722	1.996459687	-0.582822052	0.560012098	0.498141447
3148	TOMM5	5.181279208	0.184220196	1.442099292	0.127812795	0.898296222	0.498141447
3149	TOX2	4.281882129	-2.269817926	1.557852015	-1.457017684	0.145111499	0.478414242
3150	TPM2P6	2.479517011	-0.555115177	1.965642686	-0.282408996	0.777629918	0.974574021
3151	TRAM2-AS1	2.212106547	-0.956160282	2.008064151	-0.476160277	0.622960197	0.478414242
3152	TRG-AS1	2.157642949	-2.404525754	1.82425819	-1.210826722	0.189916209	0.484870241
3153	TUBBP1	2.692256257	-0.755004472	1.860025496	-0.405910819	0.684808128	0.489705489
3154	UPP2	2.192259242	-1.752812666	2.079825242	-0.842250455	0.299088285	0.748440174
3155	WASH2P	2.727180171	-0.778598547	1.902085121	-0.409229485	0.682290542	0.484870241
3156	ZMYND10	2.69052091	-1.265701966	1.860225027	-0.724116125	0.462877961	0.985021009
3157	ZNF22	2.220562525	-0.962970587	2.017761874	-0.477246894	0.622186222	0.498141447
3158	ZNF491	2.69052091	-1.265701966	1.860225027	-0.724116125	0.462877961	0.478414242
3159	ZNF520	2.718724192	-0.772515289	1.855212065	-0.416280297	0.677121744	0.489705489
3160	ZNF658	2.19519459	-0.942499502	2.068409268	-0.455662911	0.648621677	0.484870241
3161	ZXDB	2.942854764	-0.422421748	1.79298645	-0.241597002		

	0.809092446	1.451155047				
3162	AC002558.1	2.925950228	-1.528102086	1.489101724	-1.022906652	
	0.201647586	0.500997014				
3163	AC004491.1	4.207487264	-0.477282904	1.448160552	-0.229647775	
	0.741666119	0.751495511				
3164	AC004687.1	2.226122021	-0.279296202	1.876714252	-0.148821904	
	0.881694166	1.111947844				
3165	AC004771.4	2.700869719	-0.541647942	1.505612794	-0.259752245	
	0.719022418	0.489705489				
3166	AC005954.2	4.16515741	-1.651952149	1.457460045	-1.122446612	
	0.257026749	0.478414242				
3167	AC006269.1	2.914658902	-2.066065091	1.525265288	-1.245650515	
	0.178415252	0.49521421				
3168	AC007292.2	2.487080524	0.881640177	1.622472685	0.540064275	
	0.589152707	0.745814817				
3169	AC007219.1	4.207487264	-0.477282904	1.448160552	-0.229647775	
	0.741666119	0.741989481				
3170	AC007421.1	4.668929709	-1.864029274	1.40452151	-1.227152759	
	0.184457825	0.985021009				
3171	AC007686.2	2.665051777	-2.104299827	1.927280792	-1.091792471	
	0.274924229	0.500997014				
3172	AC007996.1	2.912714927	-2.260142986	1.826541407	-1.220652242	
	0.218452919	0.727248849				
3173	AC008105.2	2.445591899	-1.166227449	1.82787725	-0.624556769	
	0.525717521	0.740104194				
3174	AC008267.2	2.20254927	-1.755261201	1.971746708	-0.890206229	
	0.272255161	1.449047004				
3175	AC009087.1	2.211214092	0.722295168	1.672228519	0.42821142	
	0.661160549	0.979411277				
3176	AC009511.2	2.221752652	-0.965814947	1.940412794	-0.497726591	
	0.618669714	0.727248849				
3177	AC009522.1	2.429921205	-2.116567694	2.198064022	-1.417869282	
	0.156228902	0.948110051				
3178	AC010491.1	2.200714024	-2.948856182	2.262128772	-1.202575727	
	0.192278278	1.711109522				
3179	AC010970.1	2.681122415	-0.94992666	1.512125482	-0.627791066	
	0.520140819	1.451155047				
3180	AC011611.4	2.485086506	0.024405176	1.829807862	0.018802617	
	0.984998566	0.498141447				
3181	AC011815.1	2.957880228	0.051575027	1.672422946	0.020828507	
	0.97529822	0.498141447				
3182	AC012157.2	2.222296674	-0.959068148	1.901085858	-0.504484294	
	0.612921026	0.500997014				
3183	AC012615.5	4.874222977	-1.945155798	1.294982484	-1.294294425	
	0.162198521	0.478414242				
3184	AC016526.2	2.212055411	0.28644122	2.047441776	0.188742452	
	0.850292892	0.751495511				
3185	AC018797.2	2.928082872	-2.272747276	1.790127219	-1.26959289	
	0.204229227	0.478414242				
3186	AC020662.2	2.959824204	-0.224012654	1.479254072	-0.219022059	
	0.826622085	0.484870241				

3187	AC020762.2	4.922564888	-0.852440722	1.242641255	-0.624898206	0.525494742	1.001994018
3188	AC020895.2	2.205492226	-1.697466927	1.665772268	-1.019026222	0.208190465	0.484870241
3189	AC020904.2	2.472795181	-0.544927956	1.800802829	-0.202608172	0.762188485	1.474727498
3190	AC022150.4	2.6829077	-1.955646552	1.570929275	-1.244889894	0.212172226	0.489705489
3191	AC022966.1	2.186627412	-1.742254192	2.025821154	-0.85622956	0.291809985	0.987847254
3192	AC022972.5	2.701711028	-1.267421568	1.751887154	-0.780542027	0.425071894	0.478414242
3193	AC022157.2	2.928825627	0.059271115	1.552867922	0.028222202	0.969501748	0.484870241
3194	AC025198.1	2.240258692	1.029568811	1.926816482	0.526740998	0.591446522	0.500997014
3195	AC026210.2	2.427788561	-1.824858484	1.607289094	-1.125264192	0.256222756	0.992487988
3196	AC026410.2	2.426294601	-0.259812742	1.580412402	-0.227670791	0.819902182	0.478414242
3197	AC027227.6	2.220461227	-1.766894202	1.985482806	-0.889906176	0.272516267	0.740104194
3198	AC027575.1	4.187740061	-0.820669904	1.429085244	-0.58125987	0.561065222	0.484870241
3199	AC027601.2	4.422617926	-0.272164068	1.405214595	-0.194279201	0.845878897	0.478414242
3200	AC027644.1	2.209220064	-0.264008818	1.915641259	-0.127817456	0.890284688	1.477511982
3201	AC040970.1	2.960715585	0.541202879	1.717657296	0.21508199	0.752699402	0.724522501
3202	AC046176.1	2.969121501	-0.962410445	1.689576652	-0.57020819	0.568526502	1.920549411
3203	AC072896.5	2.465229202	-0.528466242	1.790888201	-0.200669976	0.762666166	0.484870241
3204	AC078882.1	2.712002262	-0.762596052	1.722102215	-0.442151892	0.657655872	1.121495149
3205	AC079202.2	2.928082872	-2.272747276	1.790127219	-1.26959289	0.204229227	0.498141447
3206	AC079416.2	2.199872705	-1.12546122	1.611851487	-0.69824127	0.485026221	0.714077514
3207	AC079880.2	2.472845242	1.284051929	1.986761487	0.646204022	0.518082484	0.498141447
3208	AC079921.1	2.728270298	-0.781672866	1.914122257	-0.408271229	0.682001152	0.489705489
3209	AC090772.5	2.722452269	0.229214218	1.540099652	0.14882077	0.88168717	0.478414242
3210	AC092127.1	2.227422246	0.258519284	1.908842294	0.187820275	0.851017542	0.478414242
3211	AC092140.2	2.217676042	-0.27171282	1.878526522	-0.144641997	0.884992527	0.994212224
3212	AC092620.1	2.955044891	-0.426782714	1.656212717	-0.262708482		

						0.792004558	0.489705489
3213	AC092652.1	2.195092292	-1.749295989	1.990541929	-0.878854122	0.279480277	0.500997014
3214	AC092794.2	2.752547022	2.199910529	1.986428721	1.107470162	0.268090706	0.500997014
3215	AC092849.1	2.707221669	-2.127609208	1.851020726	-1.149418622	0.2502824	1.115782111
3216	AC096992.2	2.949274198	-1.52955188	1.697627716	-0.906878922	0.264470822	0.500997014
3217	AC102729.1	2.425002276	-0.79086107	1.591058642	-0.497065947	0.619142548	0.745814817
3218	AC104124.1	2.727078972	-1.282861744	1.822768888	-0.75824296	0.448204949	0.484870241
3219	AC104581.4	2.200714024	-2.948856182	2.262128772	-1.202575727	0.192278278	0.500997014
3220	AC106729.1	2.42712592	-1.160228589	1.88409026	-0.615856125	0.527989481	1.144811841
3221	AC107068.1	2.496277821	0.611422402	1.960757851	0.211820144	0.755169614	1.119909884
3222	AC112189.1	2.220461227	-1.766894202	1.985482806	-0.889906176	0.272516267	0.49521421
3223	AC112191.1	2.221752652	-0.965814947	1.940412794	-0.497726591	0.618669714	0.999158481
3224	AC116407.1	2.925297588	-0.942270062	1.692590422	-0.557022721	0.577511215	1.42514209
3225	AC118555.1	2.690419712	-2.118454407	1.84272451	-1.149625405	0.25029819	0.478414242
3226	AC124212.2	2.227272284	-1.779249044	2.164160452	-0.822189059	0.410969205	0.478414242
3227	AC124798.1	2.744091052	2.214120762	1.912172884	1.157202022	0.247148579	0.484870241
3228	AC129502.1	2.191416726	-1.121008821	1.612672106	-0.695125082	0.486976917	1.115782111
3229	AC126628.2	1.995420879	2.856482192	2.288052187	1.196155181	0.221626007	0.714077514
3230	AC128956.2	2.454047877	-1.171978922	1.818680862	-0.644411529	0.519208604	0.748440174
3231	AC145207.8	2.66982109	-1.40708028	1.525192667	-0.916549104	0.259278965	0.751495511
3232	AC244090.1	2.191416726	-1.121008821	1.612672106	-0.695125082	0.486976917	0.751495511
3233	ACOT4	5.167151428	-0.652896725	1.212268524	-0.49825694	0.618202952	0.478414242
3234	ACTR5	9.260612228	-0.725192652	1.056602861	-0.695808879	0.486548528	0.724522501
3235	ADAM22	2.468124488	-1.961092525	1.924411751	-1.019060772	0.208174116	1.141151148
3236	ADCY1	2.182802067	-2.928120424	2.291488771	-1.282192812	0.199775017	0.484870241
3237	ADGRV1	2.425002276	-0.79086107	1.591058642	-0.497065947	0.619142548	1.722491182

3238	AICDA	2.682957762	-0.522892222	1.518227425	-0.250970885	0.725610191	0.999158481
3239	AL009021.1	2.212005249	-1.761065252	1.970076228	-0.892907209	0.27127154	1.128245842
3240	AL021962.1	4.215992405	0.556170972	1.464445412	0.279782659	0.704106756	0.500997014
3241	AL022528.2	2.175246088	-2.922502925	2.228229709	-1.254155194	0.209785602	0.999158481
3242	AL024550.2	2.191416726	-1.121008821	1.612672106	-0.695125082	0.486976917	0.49521421
3243	AL025448.1	2.704546284	-0.757987287	1.724286606	-0.429594777	0.660220624	1.444181719
3244	AL049780.2	2.701711028	-1.267421568	1.751887154	-0.780542027	0.425071894	0.49521421
3245	AL117190.2	2.712002262	-0.762596052	1.722102215	-0.442151892	0.657655872	0.740104194
3246	AL117225.1	2.456882224	-0.522005267	1.802720286	-0.294948929	0.768022892	0.748440174
3247	AL118556.2	2.186627412	-1.742254192	2.025821154	-0.85622956	0.291809985	0.727248849
3248	AL122989.2	2.222296674	-0.959068148	1.901085858	-0.504484294	0.612921026	0.49521421
3249	AL122242.4	2.741255707	1.454222991	1.818929907	0.799494797	0.424002562	0.489705489
3250	AL122228.1	2.951218164	-1.548457599	1.520491218	-1.018292991	0.208491222	0.972740484
3251	AL126984.1	2.22524064	-1.129212599	1.69496042	-0.672177107	0.501470952	0.999158481
3252	AL128789.1	2.220511289	0.276629487	1.912779801	0.196901644	0.842904507	1.442444272
3253	AL129100.2	2.199872705	-1.12546122	1.611851487	-0.69824127	0.485026221	0.489705489
3254	AL129400.1	2.20254927	-1.755261201	1.971746708	-0.890206229	0.272255161	0.500997014
3255	AL129422.1	2.725585012	0.297125295	1.766282729	0.168211162	0.866417159	0.498141447
3256	AL160006.1	2.98608252	0.519208647	1.714261292	0.202924242	0.761929899	0.724522501
3257	AL161672.1	2.456882224	-0.522005267	1.802720286	-0.294948929	0.768022892	0.49521421
3258	AL162581.1	2.726287716	0.622281821	1.549588521	0.402224089	0.687519116	0.999158481
3259	AL252662.2	2.419222582	-1.821120724	1.615920594	-1.126979544	0.25975112	1.125520514
3260	AL252807.2	2.942752566	-0.948402524	1.670157722	-0.567852075	0.570125416	0.489705489
3261	AL254714.2	2.710167016	-1.272459546	1.757286529	-0.780966228	0.424822252	1.477572045
3262	AL256804.1	2.459668509	-1.955928849	1.904720912	-1.026884745	0.204474728	0.498141447
3263	AL257552.2	2.490707128	-0.558992651	1.974225776	-0.282144222		

0.777066207 0.498141447
3264 AL258472.2 2.186627412 -1.742254192 2.025821154 -0.85622956
0.291809985 0.724522501
3265 AL290066.2 2.222455255 -0.185298222 1.619647097 -0.114406609
0.908915475 0.500997014
3266 AL291824.1 2.470118515 -0.279448989 1.577492848 -0.240529124
0.809912227 0.985021009
3267 ALYREF 2.718622995 -1.27756984 1.778187247 -0.7747046
0.428514222 0.478414242
3268 ANK2 4.182119429 -0.465464568 1.424604899 -0.224454886
0.745592672 0.489705489
3269 ANKRD25 6.428099951 0.042450707 1.220924792 0.024486841
0.972488926 0.498141447
3270 ANKRD6 2.712211106 1.054608695 1.601919851 0.658240487
0.510219268 0.500997014
3271 AP001218.1 4.162272125 -0.819698528 1.45617976 -0.562910261
0.572495992 0.714077514
3272 AP001458.1 2.445591899 -1.166227449 1.82787725 -0.624556769
0.525717521 0.478414242
3273 AP002784.2 2.707281721 -0.210868625 1.720165294 -0.121877729
0.902995846 0.498141447
3274 AP002990.1 2.195092292 -1.749295989 1.990541929 -0.878854122
0.279480277 0.71891187
3275 AP005717.1 2.194252072 -0.627216642 1.606447452 -0.290427074
0.696212278 0.724522501
3276 AP006621.2 2.209220064 -0.264008818 1.915641259 -0.127817456
0.890284688 0.500997014
3277 AP006622.1 2.424200574 -1.940478721 1.929924581 -1.00546868
0.214671222 0.484870241
3278 APC2 2.212999277 -0.179752899 1.602472969 -0.112172812
0.910686286 1.715124105
3279 ATAD2A 5.152024766 -1.274727022 1.214412784 -0.969807228
0.222142584 0.478414242
3280 ATF7IP2 2.701711028 -1.267421568 1.751887154 -0.780542027
0.425071894 0.478414242
3281 ATP6V1E2 2.450421272 0.47205592 1.625251715 0.29042209
0.771484922 0.500997014
3282 B4GALT2 2.471009896 0.628978928 1.841659722 0.246958196
0.728622725 0.745814817
3283 B9D1 4.199081448 0.566718662 1.482725597 0.282211544 0.70220446
0.717411545
3284 BAK1P1 2.195092292 -1.749295989 1.990541929 -0.878854122
0.279480277 0.49521421
3285 BATF2 4.677495811 0.207044827 1.424226787 0.145262276
0.884424848 0.500997014
3286 BCAR2 2.485126568 2.058865244 2.047916496 1.005246225
0.214720111 0.484870241
3287 BICDL2 2.700869719 -0.541647942 1.505612794 -0.259752245
0.719022418 0.500997014
3288 BMP8A 2.221802714 1.050622229 1.929088841 0.54181228
0.587947762 1.119909884

3289	BRIP1	4.691572421	-0.107420064	1.275274627	-0.078108082	0.927742079	1.708174184
3290	BSN	2.664210458	-0.941781468	1.557464112	-0.604689022	0.545285627	0.484870241
3291	BTBD18	2.20254927	-1.755261201	1.971746708	-0.890206229	0.272255161	1.114129191
3292	C11orf95	4.422617926	-0.272164068	1.405214595	-0.194279201	0.845878897	0.748440174
3293	C2orf72	2.422009248	-2.106922711	2.222167501	-1.291267654	0.164144278	0.478414242
3294	C9orf129	2.450271212	-0.804192952	1.551706171	-0.518264226	0.60427286	0.49521421
3295	CBX8	2.456882224	-0.522005267	1.802720286	-0.294948929	0.768022892	0.990451441
3296	CCDC180	2.222296674	-0.959068148	1.901085858	-0.504484294	0.612921026	0.500997014
3297	CCDC182-AS1	2.481459902	1.266021025	1.624855269	0.825560826	0.402402009	0.745814817
3298	CCDC61	2.408041258	-2.528807455	1.720648206	-1.469682022	0.141647622	1.482192474
3299	CCZ1B	2.452206558	-0.269598227	1.549552021	-0.228519258	0.811478281	0.484870241
3300	CD24	11.54720188	-1.192288949	0.970827229	-1.229122772	0.219021654	0.745814817
3301	CDH7	2.450271212	-0.804192952	1.551706171	-0.518264226	0.60427286	0.985021009
3302	CDKN2B-AS1	2.472795181	-0.544927956	1.800802829	-0.202608172	0.762188485	0.714077514
3303	CEP55	2.212005249	-1.761065252	1.970076228	-0.892907209	0.27127154	0.979411277
3304	CLIP2	2.445591899	-1.166227449	1.82787725	-0.624556769	0.525717521	1.454990294
3305	CLVS2	2.980412827	-0.452746672	1.716125245	-0.264400299	0.791471481	0.484870241
3306	CRYBG2	2.485086506	0.024405176	1.829807862	0.018802617	0.984998566	1.111947844
3307	CSNK1G2	4.919428216	-1.542742605	1.254742852	-1.12877154	0.254798455	0.985021009
3308	CTDP1	2.452206558	-0.269598227	1.549552021	-0.228519258	0.811478281	0.478414242
3309	CYP51A1	2.470068454	-1.844056642	1.706695022	-1.080482981	0.279926716	0.500997014
3310	DHFRP1	2.220511289	0.276629487	1.912779801	0.196901644	0.842904507	0.500997014
3311	DLGAP1-AS2	2.222296674	-0.959068148	1.901085858	-0.504484294	0.612921026	1.119909884
3312	DNAJB5	2.924006262	-1.52591926	1.726589722	-0.878687252	0.279570872	0.49521421
3313	DNAJC12	2.471009896	0.628978928	1.841659722	0.246958196	0.728622725	0.478414242
3314	DNMT2B	2.966286155	-1.548696215	1.721647242	-0.894248559		

						0.271125427	0.717411545
3315	DOHH	4.187740061	-0.820669904	1.429085244	-0.58125987	0.561065222	0.727248849
3316	DPY19L2P1	2.422459255	-0.795260224	1.564622268	-0.508226587	0.611217216	1.125520514
3317	DTWD2	2.220511289	0.276629487	1.912779801	0.196901644	0.842904507	0.724522501
3318	DTX1	2.408041258	-2.528807455	1.720648206	-1.469682022	0.141647622	0.484870241
3319	EGOT	4.87999267	-1.164841822	1.287601975	-0.829462949	0.401209012	0.741989481
3320	EIF1AXP1	2.214840696	-0.952486852	1.891579741	-0.502540417	0.614584272	0.489705489
3321	EIF2EP1	2.206284717	-0.945905027	1.904576171	-0.496648572	0.619426894	0.972740484
3322	EIF4EP2	2.20254927	-1.755261201	1.971746708	-0.890206229	0.272255161	0.49521421
3323	EML6	2.206284717	-0.945905027	1.904576171	-0.496648572	0.619426894	0.498141447
3324	ENTHD1	5.411979252	-1.055469226	1.286477785	-0.820422217	0.411969129	0.745814817
3325	EPHB6	2.951268226	-0.219621898	1.468271922	-0.21767775	0.827680201	0.500997014
3326	ERF	2.42712592	-1.160228589	1.88409026	-0.615856125	0.527989481	0.994212224
3327	ETS1	2.221752652	-0.965814947	1.940412794	-0.497726591	0.618669714	0.478414242
3328	F5	2.245879225	0.248522816	2.028662284	0.170956527	0.864257941	0.498141447
3329	FAM106A	2.960665522	-0.958247882	1.668188095	-0.574484207	0.565640117	1.720904898
3330	FAM161A	2.461662527	-0.274472894	1.555710866	-0.240709185	0.80978052	0.49521421
3331	FAM229A	2.476620528	0.042014154	1.796542802	0.022286114	0.981242282	0.748440174
3332	FBXL8	2.194252072	-0.627216642	1.606447452	-0.290427074	0.696212278	1.149457188
3333	FBX027	2.212005249	-1.761065252	1.970076228	-0.892907209	0.27127154	0.727248849
3334	FGD1	2.472952862	0.026794887	1.576492248	0.022229705	0.9812792	0.500997014
3335	FGFR2	4.621029185	-1.424229202	1.457805299	-0.976968121	0.228584925	0.484870241
3336	FOX04	2.226122021	-0.279296202	1.876714252	-0.148821904	0.881694166	0.478414242
3337	FUNDC2P1	2.472795181	-0.544927956	1.800802829	-0.202608172	0.762188485	0.498141447
3338	GNA11	2.22274668	0.254674927	1.662212824	0.152205082	0.87822655	0.500997014
3339	GPR162	2.709275759	0.625284672	1.550912122	0.409684242	0.68202759	0.489705489

3340	GPX2	2.462502856	-1.177672511	1.817275972	-0.648042296	0.516956947	0.724522501
3341	GSTO2	2.698875691	-2.122026292	1.841619251	-1.152804128	0.248990786	0.489705489
3342	GSTT2B	2.209220064	-0.264008818	1.915641259	-0.127817456	0.890284688	0.49521421
3343	H2BC18	2.470959824	-1.182416582	1.822615264	-0.645400704	0.518667547	0.748440174
3344	H2C12	2.952209545	-0.952270766	1.662270244	-0.572500826	0.56620564	0.484870241
3345	H2P25	2.471009896	0.628978928	1.841659722	0.246958196	0.728622725	0.484870241
3346	H2P26	2.721508402	0.860828095	1.782489999	0.482664941	0.62922266	1.144811841
3347	HCG20	2.212005249	-1.761065252	1.970076228	-0.892907209	0.27127154	0.740104194
3348	HERC2P9	2.678287068	-1.410780421	1.526422247	-0.924229889	0.255261421	1.114129191
3349	HES1	5.169926722	-1.280204717	1.218896742	-0.970662229	0.221715954	0.498141447
3350	HMGB1P51	2.448277184	-2.121276284	2.196682298	-1.420904126	0.155244621	0.751495511
3351	HSPA8P1	5.126112809	-1.269256142	1.229747607	-0.954509064	0.229826022	0.500997014
3352	IFI20	2.681122415	-0.94992666	1.512125482	-0.627791066	0.520140819	0.751495511
3353	IFITM2P1	2.940918219	-1.525048154	1.698596425	-0.902715624	0.266146207	1.715124105
3354	IFT140	4.412870622	-0.5977609	1.422921081	-0.420094205	0.674416626	0.478414242
3355	IGFBP7-AS1	2.220511289	0.276629487	1.912779801	0.196901644	0.842904507	0.484870241
3356	IL19	2.448277184	-2.121276284	2.196682298	-1.420904126	0.155244621	0.724522501
3357	IL6	2.240258692	1.029568811	1.926816482	0.526740998	0.591446522	0.478414242
3358	INPP5A	2.217676042	-0.27171282	1.878526522	-0.144641997	0.884992527	0.484870241
3359	IQCD	2.192258045	-2.942544028	2.269892162	-1.296776462	0.194708111	0.990451441
3360	JAM2	5.161520806	-0.262722912	1.221122922	-0.272245718	0.784664229	0.751495511
3361	KCNAB2	2.195092292	-1.749295989	1.990541929	-0.878854122	0.279480277	0.49521421
3362	KCNK12	2.220461227	-1.766894202	1.985482806	-0.889906176	0.272516267	0.745814817
3363	KIF9-AS1	4.184904714	-1.220222895	1.428556698	-0.848224829	0.296207204	1.971899245
3364	KLF11	4.449529892	-0.281015024	1.404586771	-0.200069542	0.841426192	0.478414242
3365	KLHL15	2.217676042	-0.27171282	1.878526522	-0.144641997		

						0.884992527	0.498141447
3366	KLRD1	2.729864258	-2.292640468	2.249977266	-1.401127158	0.161172062	0.500997014
3367	LDHAL6A	2.487921852	0.621251195	1.82821001	0.227946914	0.725402192	0.498141447
3368	LINC00106	2.698084424	0.24529469	1.541995027	0.159141028	0.872557757	0.740104194
3369	LINC00921	2.696090406	-0.752227218	1.742527001	-0.421500575	0.666104425	0.49521421
3370	LINC00926	4.905261606	-1.172697241	1.241756821	-0.874746617	0.281711789	0.500997014
3371	LINC01054	2.72991422	-0.775140487	1.782629729	-0.424585985	0.66286297	0.954818717
3372	LINC01521	2.42712592	-1.160228589	1.88409026	-0.615856125	0.527989481	0.987847254
3373	LIPE	2.194202011	-2.415916262	1.752827148	-1.278289116	0.168114042	1.719545511
3374	LMOD2	2.98608252	0.519208647	1.714261292	0.202924242	0.761929899	0.484870241
3375	LNX2	2.678287068	-1.410780421	1.526422247	-0.924229889	0.255261421	0.71891187
3376	LRCH4	2.21116402	-0.62705122	1.604772199	-0.29697275	0.691287555	0.49521421
3377	LRRC24	2.957880228	0.051575027	1.672422946	0.020828507	0.97529822	0.484870241
3378	LRRC75B	2.927241554	-1.092206191	1.470592211	-0.7422782	0.457252729	1.454990294
3379	LRRC8C-DT	2.002886858	2.844760722	2.284699599	1.192922045	0.222899905	0.49521421
3380	LURAP1L	2.715787648	-2.122252624	1.872670688	-1.128008748	0.25511682	0.748440174
3381	MAB21L1	2.687624427	-0.746519268	1.789848725	-0.417085116	0.676616151	0.478414242
3382	MAGI2-AS2	2.224587999	-0.287021664	1.910088572	-0.150271289	0.880550505	1.144811841
3383	MAMSTR	2.206284717	-0.945905027	1.904576171	-0.496648572	0.619426894	0.990451441
3384	MAPRE2-AS1	2.490707128	-0.558992651	1.974225776	-0.282144222	0.777066207	1.484019012
3385	MDFIC	2.226122021	-0.279296202	1.876714252	-0.148821904	0.881694166	0.498141447
3386	MEGF11	5.421726555	-0.7681245	1.297158621	-0.592166972	0.552728782	0.999158481
3387	MEIS2	2.948482817	-2.078925566	1.570960879	-1.222252856	0.185718062	1.500155495
3388	MFSD12B	2.20254927	-1.755261201	1.971746708	-0.890206229	0.272255161	0.724522501
3389	MIAT	2.690419712	-2.118454407	1.84272451	-1.149625405	0.25029819	0.489705489
3390	MIB2	2.212055411	0.28644122	2.047441776	0.188742452	0.850292892	0.498141447

3391 MINCR 4.167992757 -1.212468165 1.429421254 -0.842024985 0.29921447 0.478414242
3392 MIR101-1 2.20254927 -1.755261201 1.971746708 -0.890206229 0.272255161 1.114129191
3393 MORN4 2.915550284 -1.521157526 1.786067258 -0.851679825 0.294291821 0.751495511
3394 MPP7 2.214840696 -0.952486852 1.891579741 -0.502540417 0.614584272 0.478414242
3395 MROCKI 4.428288629 0.275664222 1.450902451 0.258917527 0.795698875 0.484870241
3396 MSH5 2.450421272 0.47205592 1.625251715 0.29042209 0.771484922 0.498141447
3397 MT-TE 2.191416726 -1.121008821 1.612672106 -0.695125082 0.486976917 0.745814817
3398 MT-TQ 2.704546284 -0.757987287 1.724286606 -0.429594777 0.660220624 0.49521421
3399 MTND5P22 2.752547022 2.199910529 1.986428721 1.107470162 0.268090706 0.498141447
3400 MYEF2 4.652027752 -1.858509189 1.400542477 -1.226992276 0.184511205 0.500997014
3401 MYLK 2.682957762 -0.522892222 1.518227425 -0.250970885 0.725610191 1.111402844
3402 NAT8L 2.224587999 -0.287021664 1.910088572 -0.150271289 0.880550505 0.49521421
3403 NBL1 2.195092292 -1.749295989 1.990541929 -0.878854122 0.279480277 1.001994018
3404 NDUFA12 2.727078972 -1.282861744 1.822768888 -0.75824296 0.448204949 0.500997014
3405 NDUFA4L2 2.4907572 1.261741615 1.872468416 0.672828664 0.500412891 0.751495511
3406 NECAB2 4.657698446 -1.065065171 1.272288245 -0.775501884 0.428042152 0.498141447
3407 NEK11 2.217676042 -0.27171282 1.878526522 -0.144641997 0.884992527 0.724522501
3408 NME9 2.957880228 0.051575027 1.672422946 0.020828507 0.97529822 0.49521421
3409 NOX2 2.451212521 -1.95081291 1.899722555 -1.026892572 0.204470581 0.49521421
3410 NPM1P24 2.710167016 -1.272459546 1.757286529 -0.780966228 0.424822252 1.944442287
3411 NR1D2 2.921620922 -0.687421999 1.469424848 -0.467820672 0.629912826 0.484870241
3412 NR4A1AS 2.667887124 -1.246267779 1.965056205 -0.685102921 0.492278246 0.748440174
3413 NRG1-IT1 2.200714024 -2.948856182 2.262128772 -1.202575727 0.192278278 0.484870241
3414 NRIP2 2.925950228 -1.528102086 1.489101724 -1.022906652 0.201647586 0.751495511
3415 NUMBL 2.926841609 -0.928172461 1.742119446 -0.528522614 0.590215607 0.478414242
3416 NUTM2A 2.429129948 0.058765908 1.622515002 0.026196714

						0.971125506	0.979411277
3417	OBSCN	2.960665522	-0.958247882	1.668188095	-0.574484207	0.565640117	0.498141447
3418	OGFOD2	2.454047877	-1.171978922	1.818680862	-0.644411529	0.519208604	0.478414242
3419	OR8A1	2.462502856	-1.177672511	1.817275972	-0.648042296	0.516956947	0.974574021
3420	OR8B5P	2.200714024	-2.948856182	2.262128772	-1.202575727	0.192278278	0.484870241
3421	OTUD2	2.24209404	1.852040226	2.021917577	0.915982059	0.259676205	0.498141447
3422	PCCA-AS1	2.222296674	-0.959068148	1.901085858	-0.504484294	0.612921026	0.71891187
3423	PCDHGB5	2.451212521	-1.95081291	1.899722555	-1.026892572	0.204470581	1.711400858
3424	PCDHGB7	2.952209545	-0.952270766	1.662270244	-0.572500826	0.56620564	0.49521421
3425	PCGF2	2.461662527	-0.274472894	1.555710866	-0.240709185	0.80978052	0.741989481
3426	PHF7	4.148245452	-1.645612887	1.476640402	-1.114421022	0.265094217	1.125520514
3427	PIGZ	2.224628061	1.866979922	2.114942692	0.882756747	0.277267715	0.500997014
3428	PLBD1	6.11268884	-1.210110829	1.229997068	-1.05654249	0.290719982	1.444181719
3429	PLCB1	4.677445749	-0.722222166	1.264467442	-0.526717216	0.591462962	0.484870241
3430	PLCL1	2.724292688	-0.222601544	1.727710178	-0.129420748	0.897024721	0.489705489
3431	PLEKHA6	2.177290054	-2.408265914	1.747101508	-1.278492264	0.168051255	0.49521421
3432	PLOD2	2.20254927	-1.755261201	1.971746708	-0.890206229	0.272255161	0.489705489
3433	PMS2P2	2.661275111	-1.402229794	1.552885992	-0.902697889	0.266155619	1.104491887
3434	PNPLA7	5.164216091	-0.954621719	1.210078218	-0.728675262	0.466200268	1.449047004
3435	PODXL	2.456882224	-0.522005267	1.802720286	-0.294948929	0.768022892	1.497210248
3436	POPDC2	2.452156497	-1.826119665	1.629742227	-1.126621191	0.259898446	0.981194441
3437	POT1-AS1	2.206284717	-0.945905027	1.904576171	-0.496648572	0.619426894	1.119909884
3438	PSMC1P1	2.227422246	0.258519284	1.908842294	0.187820275	0.851017542	1.48884427
3439	PTCH2	2.690419712	-2.118454407	1.84272451	-1.149625405	0.25029819	1.975724711
3440	PTGFRN	2.454047877	-1.171978922	1.818680862	-0.644411529	0.519208604	0.745814817
3441	PTGR2	2.724292688	-0.222601544	1.727710178	-0.129420748	0.897024721	0.49521421

3442	PTPDC1	2.468174549	0.049452287	1.798059915	0.027502192	0.978058294	0.489705489
3443	QRICH2	2.72842026	0.84244542	1.780592881	0.4726877	0.625722626	0.741989481
3444	RAB28P5	2.410876604	-1.8172299	1.622228497	-1.112728502	0.26582505	0.724522501
3445	RAD54B	1.998216164	0.764522728	2.140601802	0.25715785	0.720972628	0.992487988
3446	RAPGEF6	2.465229202	-0.528466242	1.790888201	-0.200669976	0.762666166	0.478414242
3447	RASGEF1C	2.228967268	0.267589562	1.886917286	0.194809569	0.845542021	0.999158481
3448	RASL11B	2.485126568	2.058865244	2.047916496	1.005246225	0.214720111	0.717411545
3449	RAVER1	5.181228048	-0.96046722	1.220567284	-0.727214062	0.467022585	0.498141447
3450	RET	4.422567874	-1.226992925	1.410646297	-0.947787498	0.242227646	0.999158481
3451	RFX8	2.921170916	-2.264418467	1.809779949	-1.251212042	0.210857126	0.498141447
3452	RILPL1	2.96250087	-0.442218441	1.655211429	-0.267211614	0.789206225	1.444181719
3453	RN7SL220P	2.212005249	-1.761065252	1.970076228	-0.892907209	0.27127154	0.478414242
3454	RNA5SP252	2.725585012	0.297125295	1.766282729	0.168211162	0.866417159	1.944442287
3455	RNASEK	2.44475058	-0.264728248	1.557124941	-0.224226762	0.814801176	0.49521421
3456	RNF129-AS1	2.692255059	-1.262284705	1.75970242	-0.774212118	0.428804772	0.751495511
3457	RNU6-514P	2.224587999	-0.287021664	1.910088572	-0.150271289	0.880550505	0.478414242
3458	RPL17P22	2.459668509	-1.955928849	1.904720912	-1.026884745	0.204474728	0.727248849
3459	RPL21P16	2.225290702	0.261112547	1.622212869	0.160852262	0.872209762	0.71891187
3460	RPL7P11	2.696090406	-0.752227218	1.742527001	-0.421500575	0.666104425	0.741989481
3461	RPS10	2.462502856	-1.177672511	1.817275972	-0.648042296	0.516956947	0.500997014
3462	SAMD12	4.688687012	-1.448024216	1.400197807	-1.024156966	0.201062789	1.119909884
3463	SEC14L6	5.150189419	-1.621675116	1.225720664	-1.220782498	0.218402849	0.748440174
3464	SEMA2A	2.969121501	-0.962410445	1.689576652	-0.57020819	0.568526502	0.478414242
3465	SERINC2	4.418491264	-0.95096422	1.299572422	-0.679467192	0.496841888	0.489705489
3466	SERPING1	2.240258692	1.029568811	1.926816482	0.526740998	0.591446522	1.444181719
3467	SH2RF2	2.710167016	-1.272459546	1.757286529	-0.780966228		

0.424822252	0.727248849
3468	SHQ1P1 2.966226216	0.045208849	1.66008404	0.027292106
0.978225956	0.714077514
3469	SLC10A1	2.718622995	-1.27756984	1.778187247	-0.7747046
0.428514222	1.117074528
3470	SLC16A1-AS1	2.216824722	0.267221057	1.612025206	0.165726522
0.868272172	0.498141447
3471	SLC25A24	2.70649025	-0.962009975	1.526425981	-0.620226898
0.528529602	0.49521421
3472	SNORA1B	2.212005249	-1.761065252	1.970076228	-0.892907209
0.27127154	0.489705489
3473	SNTG1	2.485086506	0.024405176	1.829807862	0.018802617
0.984998566	0.727248849
3474	SNX18	2.925950228	-1.528102086	1.489101724	-1.022906652
0.201647586	1.958811755
3475	SORD2P 2.200714024	-2.948856182	2.262128772	-1.202575727
0.192278278	18.20414442
3476	SPACA9 2.942752566	-0.948402524	1.670157722	-0.567852075
0.570125416	17.82412421
3477	SPIN2B 2.707221669	-2.127609208	1.851020726	-1.149418622	0.2502824
12.88002144
3478	ST12P4 2.214840696	-0.952486852	1.891579741	-0.502540417
0.614584272	8.074981041
3479	STAG2L5P	2.447585927	0.052128229	1.578706182	0.022659252
0.972148792	9.015404851
3480	STKLD1 2.447585927	0.052128229	1.578706182	0.022659252
0.972148792	7.824989574
3481	SZT2-AS1	4.682066281	-1.074448201	1.272622257	-0.782764226
0.422765572	5.155759989
3482	TAB2-AS2	2.220511289	0.276629487	1.912779801	0.196901644
0.842904507	8.57519277
3483	TAF5L	7.28204227	-1.180210575	1.14089274	-1.024549991
0.200879119	4.45197749
3484	TCTN2	2.45882719	-0.808642922	1.562507422	-0.517529018
0.604786918	4.42790108
3485	TEDC2	2.44475058	-0.264728248	1.557124941	-0.224226762
0.814801176	1.004711105
3486	TESMIN 2.441915222	-0.799784819	1.552406049	-0.515190481	0.60641992
1.004711105
3487	TIGD7	6.194562462	0.62859726	1.274500892	0.492210529
0.621862829	1.004711105
3488	TLNRD1 2.408041258	-2.528807455	1.720648206	-1.469682022
0.141647622	5.144518714
3489	TMCC2	2.20254927	-1.755261201	1.971746708	-0.890206229
0.272255161	5.149884441
3490	TMEM26 2.212055411	0.28644122	2.047441776	0.188742452
0.850292892	2.174454708
3491	TMEM9B-AS1	2.71582771	-0.217226429	1.717567442	-0.12647221
0.899257275	2.174454708
3492	TNNI2	2.689578292	-0.952928576	1.507876729	-0.622620246
0.526975066	2.191244445

3493	TOMM20L	2.002886858	2.844760722	2.284699599	1.192922045	0.222899905	9.087481109
3494	TPM1-AS	4.865866998	-1.942511004	1.411562498	-1.276142401	0.168777551	4.912747512
3495	TPT1P12	2.957820176	-1.544081925	1.707745457	-0.904162977	0.265908458	2.108178411
3496	TRAF2IP1	4.680221024	-1.445019658	1.286065787	-1.042522242	0.297164522	2.918725512
3497	TRAV8-4	2.222296674	-0.959068148	1.901085858	-0.504484294	0.612921026	2.884455411
3498	TRGV7	2.214840696	-0.952486852	1.891579741	-0.502540417	0.614584272	1.925147514
3499	TRIM52-AS1	2.689578292	-0.952928576	1.507876729	-0.622620246	0.526975066	5.591944511
3500	TSGA10	4.666154424	-1.068185978	1.266969424	-0.781426281	0.42455176	1.942702504
3501	TSHR	2.226122021	-0.279296202	1.876714252	-0.148821904	0.881694166	1.951159482
3502	TSPAN18	2.225290702	0.261112547	1.622212869	0.160852262	0.872209762	1.891947422
3503	TSSK4	2.496277821	0.611422402	1.960757851	0.211820144	0.755169614	4.409985114
3504	TTLL7-IT1	2.209220064	-0.264008818	1.915641259	-0.127817456	0.890284688	1.494040244
3505	TXNDC16	4.428228568	-0.608667499	1.296222059	-0.425925459	0.662882557	2.915950118
3506	UPP2-IT1	2.922462241	-1.520522021	1.710648128	-0.894702	0.270945875	4.107487244
3507	URAHP	2.468124488	-1.961092525	1.924411751	-1.019060772	0.208174116	1.114121011
3508	USP21	4.187740061	-0.820669904	1.429085244	-0.58125987	0.561065222	2.700849719
3509	VAMP5	2.940026829	-2.075655262	1.551784128	-1.227592846	0.181029198	4.14515741
3510	VDAC1P1	2.957880228	0.051575027	1.672422946	0.020828507	0.97529822	2.914458902
3511	VEGFA	2.429079886	-1.275177892	1.565267749	-0.814618722	0.415290584	2.487080524
3512	VPS9D1	2.698024272	-0.95794129	1.511922094	-0.622587576	0.526250011	4.107487244
3513	WASIR2	2.240258692	1.029568811	1.926816482	0.526740998	0.591446522	4.448929709
3514	WDFY2-AS1	2.221752652	-0.965814947	1.940412794	-0.497726591	0.618669714	1.445051777
3515	WEE2-AS1	2.254285265	2.027594977	2.215615287	1.211787409	0.189591875	1.911714927
3516	YBX1P4	2.744091052	2.214120762	1.912172884	1.157202022	0.247148579	1.445591899
3517	Z92920.2	5.206696107	0.744242219	1.261652072	0.546572971	0.584672142	1.10254927
3518	Z95152.1	2.707281721	-0.210868625	1.720165294	-0.121877729		

						0.902995846	2.111114091
3519	ZBED8	6.28292465	-0.869026452	1.209225602	-0.718662624	0.472248201	1.121751452
3520	ZBTB16	2.718622995	-1.27756984	1.778187247	-0.7747046	0.428514222	1.429911105
3521	ZBTB21	4.227224668	-0.140795546	1.520624582	-0.092589992	0.926229294	1.100714014
3522	ZC2H12C	5.147404124	-0.9488172	1.221482824	-0.717992876	0.472761062	2.481111415
3523	ZGRF1	5.870646211	-1.524284866	1.256160779	-1.22148764	0.221901442	1.485084504
3524	ZNF257	4.192260692	-1.222657521	1.448725006	-0.844644446	0.298209285	1.957880128
3525	ZNF224	2.485086506	0.024405176	1.829807862	0.018802617	0.984998566	1.112194474
3526	ZNF295	4.182069267	-1.658278486	1.462507191	-1.122860056	0.256852255	4.874211977
3527	ZNF414	2.420622908	-1.27114059	1.570926972	-0.809160782	0.41842267	1.111055411
3528	ZNF418	2.228967268	0.267589562	1.886917286	0.194809569	0.845542021	1.928081872
3529	ZNF425	4.908146891	-1.955542284	1.276727426	-1.420428872	0.155482862	2.959814104
3530	ZNF460-AS1	2.214840696	-0.952486852	1.891579741	-0.502540417	0.614584272	4.922544888
3531	ZNF517	2.690419712	-2.118454407	1.84272451	-1.149625405	0.25029819	2.105492224
3532	ZNF580	2.667887124	-1.246267779	1.965056205	-0.685102921	0.492278246	1.472795181
3533	ZNF775	4.196196029	-0.824240158	1.42701896	-0.580604826	0.561506812	2.4829077
3534	ZNF90	2.700869719	-0.541647942	1.505612794	-0.259752245	0.719022418	1.184427412
3535	AARD	2.197827541	-1.744592029	2.077668542	-0.829687272	0.401082696	1.701711028
3536	AATK	2.221701516	0.269988626	1.851107125	0.199874227	0.84157894	2.918825427
3537	ABHD16B	2.71011588	-0.208649895	1.695274212	-0.122070112	0.902051578	1.140158492
3538	ABHD8	2.202606854	-1.122927495	1.589995226	-0.706252118	0.480021265	2.417788541
3539	ABI2BP	4.170726906	-1.211476484	1.408951698	-0.859842452	0.289875894	2.424194401
3540	AC002210.2	2.917292052	-2.062250751	1.524982698	-1.244217595	0.178878022	1.110441217
3541	AC002464.1	2.211954212	-0.261265574	1.891054255	-0.12815868	0.89011501	4.187740041
3542	AC004120.2	2.682856564	-0.947861896	1.48144882	-0.629820885	0.522289052	4.421417924
3543	AC004222.1	2.472902726	0.897200422	1.552707006	0.577829824	0.562279011	1.109110044

3544	AC004286.2	2.698824555	-0.749725109	1.720281478	-0.425821182	0.662966472	1.940715585
3545	AC004540.1	4.654761901	-1.856240926	1.282429215	-1.242810841	0.179222222	1.949111501
3546	AC004980.1	2.72419249	-0.76652601	1.716774758	-0.446497717	0.655227761	1.445229102
3547	AC005086.2	2.202498224	-0.252787429	2.020568961	-0.12449094	0.900926572	1.712001242
3548	AC005222.5	4.922222427	-0.525255221	1.297560787	-0.412508868	0.679966482	1.928081872
3549	AC005498.2	2.226080885	1.064212485	2.015207202	0.528140942	0.597401515	2.199871705
3550	AC005720.2	4.167941621	-0.455912846	1.425960017	-0.217497591	0.750866067	1.472845142
3551	AC006141.1	2.949222062	-0.429051176	1.641229426	-0.261402077	0.792781685	1.728270198
3552	AC007021.1	4.196144902	-0.121481872	1.29141014	-0.087208457	0.920426222	2.712451249
3553	AC007114.2	4.198920188	-0.822418626	1.299280999	-0.594847748	0.551945247	1.127412244
3554	AC007282.1	2.00278566	1.616872729	2.145079425	0.752759281	0.450992719	1.117474041
3555	AC007682.1	2.501947227	1.252257259	1.922201276	0.64806519	0.516942787	1.955044891
3556	AC007878.1	2.221701516	0.269988626	1.851107125	0.199874227	0.84157894	1.195092291
3557	AC008264.2	5.651085225	-1.456087922	1.225789902	-1.178264947	0.228690997	1.751547021
3558	AC008267.2	2.22886617	-0.276467782	1.852179412	-0.1492662	0.881242585	1.707221449
3559	AC008764.8	2.928021726	-0.940820224	1.671007724	-0.562021699	0.572412299	1.949274198
3560	AC008895.1	2.689477196	-1.4120727	1.505548627	-0.927912041	0.248289101	2.415002174
3561	AC008927.2	2.928021726	-0.940820224	1.671007724	-0.562021699	0.572412299	1.717078972
3562	AC008945.1	2.427024722	-1.925817789	2.009567862	-0.962200521	0.225296727	1.100714014
3563	AC009065.5	2.22886617	-0.276467782	1.852179412	-0.1492662	0.881242585	1.42712591
3564	AC009092.2	5.884671785	-1.229472942	1.209126002	-1.016828625	0.209224957	1.494277821
3565	AC009118.2	2.245828188	1.852922729	1.978040084	0.927252261	0.248628761	1.110441217
3566	AC010175.1	2.70160984	-2.118894956	1.912094777	-1.108152728	0.26779542	1.121751452
3567	AC010200.1	2.225189504	-0.182195642	1.579292485	-0.115991125	0.907659568	1.925197588
3568	AC010226.5	2.687522229	-1.254067252	1.782216875	-0.759722128	0.447420101	1.490419711
3569	AC010624.2	2.942652268	-1.521992192	1.698082017	-0.902189752		

						0.266956078	1.127272184
3570	AC010761.2	4.204600881	-0.125699081	1.291019027	-0.090264746	0.927997271	1.744091052
3571	AC010824.1	5.209280194	-0.100660627	1.201651522	-0.077222007	0.928258622	2.191414714
3572	AC011279.2	2.208227485	-1.694467781	1.665202692	-1.017512286	0.208909268	1.995420879
3573	AC011466.2	5.662426622	-0.228222699	1.22215108	-0.266290789	0.789928258	1.454047877
3574	AC011477.2	2.222245528	0.278917827	1.876942612	0.201880245	0.840010246	2.44982109
3575	AC011825.1	2.490656002	0.622494145	1.796642982	0.247022856	0.728566625	2.191414714
3576	AC012072.1	2.427727425	-0.788792829	1.55842924	-0.506146072	0.612754109	5.147151428
3577	AC012085.2	2.949222062	-0.429051176	1.641229426	-0.261402077	0.792781685	9.240412218
3578	AC012652.1	2.49265002	1.259241669	1.604712228	0.847092672	0.296942911	1.448114488
3579	AC018728.1	4.908095755	-1.171985511	1.206156715	-0.897277867	0.269570664	1.182801047
3580	AC020661.2	2.706429214	-0.145874021	1.465601222	-0.099521871	0.920715984	2.415002174
3581	AC020904.2	2.177228918	-1.109508845	1.629210841	-0.680968184	0.495891622	2.482957741
3582	AC021424.2	2.707280522	-0.755267227	1.701029921	-0.44406462	0.656995858	1.111005249
3583	AC022098.1	6.288504146	-0.627218552	1.171502215	-0.525296812	0.592275494	4.115992405
3584	AC022154.1	2.712109908	0.626698107	1.501845282	0.422942846	0.671606767	1.175244088
3585	AC022498.2	4.425202022	-1.224942977	1.280985622	-0.966659569	0.222714245	2.191414714
3586	AC022784.2	2.448226048	-1.162928296	1.827220789	-0.622984228	0.526742877	1.704544284
3587	AC022972.4	2.240157495	0.2610646	1.872084447	0.19276472	0.847142224	1.701711028
3588	AC025048.2	2.447484729	-0.262882716	1.517222821	-0.229160521	0.810981114	1.712001242
3589	AC026268.1	2.472744045	0.640897606	1.799914748	0.25607109	0.721787227	1.454882114
3590	AC026401.2	4.922222427	-0.525255221	1.297560787	-0.412508868	0.679966482	1.184427412
3591	AC024111.1	2.217574844	-0.949112552	1.890850026	-0.501950202	0.61570255	1.112194474
3592	AC046124.2	2.709274561	0.241524028	1.476525098	0.162575972	0.870064962	1.741155707
3593	AC048282.6	2.729021865	0.624885252	1.500592096	0.416425792	0.677098459	2.951218144
3594	AC055854.1	2.458776054	0.049286062	1.515597218	0.022585214	0.974005261	2.11514044

3595	AC072022.1	2.97185565	-0.960726492	1.66710941	-0.576282809	0.564424041	1.110511289	
3596	AC072957.2	4.688625877	-0.722992282	1.226060748	-0.552514071	0.579911448	2.199871705	
3597	AC078777.1	6.152082249	-1.042045111	1.180792761	-0.882496189	0.27750854	1.10254927	
3598	AC078889.1	2.171568225	-2.4005715	1.815249765	-1.222446941	0.186019266	1.725585012	
3599	AC079285.1	2.211954212	-0.261265574	1.891054255	-0.12815868	0.89011501	1.98408251	
3600	AC079601.2	2.956927721	0.046299074	1.429269821	0.022292515	0.974158224	1.454882114	
3601	AC084125.4	2.472744045	0.640897606	1.799914748	0.25607109	0.721787227	2.714187714	
3602	AC084871.2	2.96628508	1.062622129	1.797289467	0.591792896	0.552989281	2.419221582	
3603	AC087289.2	4.688625877	-0.722992282	1.226060748	-0.552514071	0.579911448	1.942752544	
3604	AC087854.1	2.908927074	-2.060150225	1.541174787	-1.226740227	0.181207446	1.710147014	
3605	AC091271.1	1.995229681	1.622151187	2.052172212	0.794941162	0.426647728	1.459448509	
3606	AC091825.2	2.220410191	-0.26895024	1.852956848	-0.145068285	0.884656958	1.490707128	
3607	AC092118.2	2.228024851	0.262010218	1.578554662	0.166614641	0.867672272	1.184427412	
3608	AC092279.2	2.957728978	-2.277125151	1.871269219	-1.216822024	0.222671558	2.111455255	
3609	AC092249.1	2.245828188	1.852922729	1.978040084	0.927252261	0.248628761	2.470118515	
3610	AC092110.1	2.202606854	-1.122927495	1.589995226	-0.706252118	0.480021265	1.718411995	
3611	AC092222.1	4.920678405	-0.528519677	1.292822912	-0.416220097	0.677248955	4.181119419	
3612	AC096772.2	2.22886617	-0.276467782	1.852179412	-0.1492662	0.881242585	4.418099951	
3613	AC096921.2	2.959672944	-2.079424062	1.600545597	-1.299197015	0.192876226	2.711111104	
3614	AC099811.5	2.966225019	-0.440029002	1.621197461	-0.271428281	0.786061576	4.141271115	
3615	AC100782.1	2.71011588	-0.208649895	1.695274212	-0.122070112	0.902051578	1.445591899	
3616	AC100784.1	2.709224499	-0.959788678	1.494625821	-0.642155529	0.520772198	1.707281721	
3617	AC102987.2	2.47652922	-0.542158697	1.776941895	-0.205107724	0.760284109	1.195092291	
3618	AC104964.2	2.972849678	0.026808779	1.457626948	0.025252264	0.97985267	2.194151072	
3619	AC104964.4	2.940867082	-0.42221869	1.687215465	-0.250882955	0.801904602	1.109110044	
3620	AC105429.1	2.211112894	0.275158942	1.580255087	0.174122118			

0.861768706 1.424200574
3621 AC106882.1 2.692212542 -0.951822202 1.476194492 -0.644781096
0.519069054 2.112999277
3622 AC108488.1 2.709274561 0.241524028 1.476525098 0.162575972
0.870064962 5.152014744
3623 AC109429.1 2.42902875 -0.258011081 1.540712708 -0.222267189
0.816252821 1.701711028
3624 AC109449.1 2.221701516 0.269988626 1.851107125 0.199874227
0.84157894 2.450411172
3625 AC109460.1 6.405416102 -0.62220781 1.162524829 -0.542818278
0.586566461 1.471009894
3626 AC112282.1 4.170726906 -1.211476484 1.408951698 -0.859842452
0.289875894 4.199081448
3627 AC116247.1 2.727027827 -0.221216818 1.692997928 -0.120665725
0.896029729 1.195092291
3628 AC122129.2 2.479264677 0.04441228 1.761195727 0.025217174
0.979881728 4.477495811
3629 AC125494.1 2.706429214 -0.145874021 1.465601222 -0.099521871
0.920715984 1.485124548
3630 AC129492.7 9.11284897 -0.840902187 1.016558995 -0.827204512
0.408121155 2.700849719
3631 AC120204.1 6.622221959 -0.951502947 1.14491124 -0.821072228
0.405922822 1.121801714
3632 AC120242.2 2.209118866 -0.942509092 1.902840222 -0.495056821
0.620560012 4.491571411
3633 AC126475.1 4.457924725 0.267520811 1.272056109 0.267861257
0.78880604 2.444110458
3634 AC126604.2 2.459617272 -0.529220194 1.779792592 -0.297411168
0.766152618 1.10254927
3635 AC129451.1 2.482149962 -1.185965772 1.876107895 -0.622141561
0.52729428 4.421417924
3636 AC129887.1 2.721407205 0.212892606 1.692290456 0.185274924
0.852924968 1.412009148
3637 AC222271.1 2.481258704 0.890292744 1.551905266 0.572742221
0.566142179 2.450271111
3638 AC244502.2 2.974690997 -0.445559128 1.628404228 -0.271947014
0.785662761 1.454882114
3639 AC244669.1 2.707280522 -0.755267227 1.701029921 -0.44406462
0.656995858 1.112194474
3640 AC245060.4 2.240157495 0.2610646 1.872084447 0.19276472
0.847142224 2.481459901
3641 ACACB 2.92519629 -1.5274405 1.710120125 -0.892172086
0.271765024 2.408041158
3642 AD000091.1 2.742989856 1.456027042 1.771795152 0.821786224
0.411198512 2.452104558
3643 ADIRF-AS1 2.95777904 -0.424575284 1.622159767 -0.267899188
0.788776919 11.54720188
3644 ADO 7.626819947 -1.025244708 1.082287904 -0.947286277
0.242441981 2.450271111
3645 AF121215.4 2.72419249 -0.76652601 1.716774758 -0.446497717
0.655227761 1.472795181

3646	AGBL2	2.720565886	0.620802528	1.490261596	0.422282092	0.672088728	1.111005249
3647	AHDC1	2.22886617	-0.276467782	1.852179412	-0.1492662	0.881242585	1.445591899
3648	AL009179.1	2.479214615	-1.962141222	2.050166191	-0.957064472	0.228524715	1.980411817
3649	AL021707.2	2.456782026	-1.168715087	1.818040868	-0.642842122	0.520225892	1.485084504
3650	AL022216.1	4.649141269	-1.421272408	1.258208401	-1.05279044	0.291978871	4.919428114
3651	AL024418.1	2.728269224	2.229200782	1.941412196	1.148226124	0.250871096	2.452104558
3652	AL049872.1	2.971905712	0.525608144	1.62769868	0.227049261	0.742620627	2.470048454
3653	AL109811.2	2.97185565	-0.960726492	1.66710941	-0.576282809	0.564424041	1.110511289
3654	AL121822.2	2.712951227	0.221227409	1.728902989	0.185798292	0.852602942	1.112194474
3655	AL122406.2	2.48502527	1.274224881	1.829411259	0.696527298	0.486098668	1.914004141
3656	AL126418.2	2.208277547	-0.172269781	1.562862212	-0.110291086	0.912178527	1.471009894
3657	AL158801.1	4.187688924	-0.117242065	1.402425162	-0.082529948	0.922422215	1.944184155
3658	AL158801.2	2.969070265	0.047202222	1.619072779	0.029215692	0.976692565	4.187740041
3659	AL158801.6	2.672565229	-1.404719825	1.514622426	-0.927428952	0.252698675	2.422459155
3660	AL158824.2	2.200662887	-0.925694774	1.946822852	-0.480626215	0.620782109	1.110511289
3661	AL254956.1	4.674559266	-0.40771895	1.222282004	-0.206020287	0.759581586	2.408041158
3662	AL255075.1	2.974741059	1.054458968	1.687021051	0.625041974	0.52194251	4.87999247
3663	AL255075.2	2.479264677	0.04441228	1.761195727	0.025217174	0.979881728	1.114840494
3664	AL255288.1	4.429681291	-0.952517892	1.25872441	-0.701022171	0.482282215	1.104284717
3665	AL256124.2	2.92519629	-1.5274405	1.710120125	-0.892172086	0.271765024	1.10254927
3666	AL256512.1	2.206282519	-1.750642152	2.057470572	-0.850871529	0.294840717	1.104284717
3667	AL257874.2	2.448226048	-1.162928296	1.827220789	-0.622984228	0.526742877	5.411979151
3668	AL258216.1	2.459617272	-0.529220194	1.779792592	-0.297411168	0.766152618	2.951248114
3669	AL258781.2	2.459617272	-0.529220194	1.779792592	-0.297411168	0.766152618	1.42712591
3670	AL442071.1	2.242992842	1.041795194	1.895194156	0.549702676	0.582522625	1.121751452
3671	AL589862.1	2.185694897	-1.114062228	1.602998927	-0.694986885		

0.487062521	1.145879215
3672	AL591846.2	2.248612472	0.251257827	2.000162887	0.175614528
0.860596788	1.940445512
3673	AL596087.1	1.998165028	2.858284159	2.244661897	1.219060267
0.222821219	2.441441527
3674	AL645924.2	2.476579292	1.285585964	1.928628762	0.662128481
0.507241867	1.474420518
3675	AL670729.1	2.499162042	2.219605757	2.215552992	1.452182162
0.146172906	2.194151072
3676	ALKBH6 2.464446748	0.904157979	1.585724065	0.570182604
0.568552852	1.111005249
3677	AMER1	2.461561229	-0.806400152	1.5299227	-0.527082108
0.598126571	2.471952841
3678	ANKHD1 2.982197027	1.046150928	1.662742764	0.628792298
0.529484288	4.411029185
3679	ANKRD18DP	2.496226695	2.046090824	1.917009667	1.067224641
0.285820751	1.114121011
3680	ANKRD44-IT1	5.645514665	-0.222092542 1.266402242	-0.254226661
0.799225479	1.472795181
3681	ANKRD9 2.722648469	-0.77221821	1.760442711	-0.42870662	0.66087412
2.12274448
3682	AP000442.1	2.428978689	-1.825711014 1.606428952	-1.126502808
0.255746191	2.709275759
3683	AP000446.1	2.188520242	-0.620122589 1.596496589	-0.288422294
0.697695225	1.441502854
3684	AP000692.1	2.464296686	-0.272466604 1.515977462	-0.245694025
0.805919098	1.498875491
3685	AP000757.1	2.962299672	-0.955729925 1.645672925	-0.580752266
0.561406757	1.109110044
3686	AP000769.2	2.459617272	-0.529220194 1.779792592	-0.297411168
0.766152618	1.470959824
3687	AP000902.1	2.49911198	0.612857789	1.91629065	0.220226474
0.748712277	1.951109545
3688	AP000919.2	2.46245272	0.059241402	1.799089762	0.022928542
0.972721572	1.471009894
3689	AP001029.2	5.422169279	-1.056564776 1.251645797	-0.844140294
0.298590958	1.711508402
3690	AP002748.4	2.185694897	-1.114062228 1.602998927	-0.694986885
0.487062521	1.111005249
3691	AP002812.5	2.678225922	-0.526661772 1.505722414	-0.24977225
0.726508876	2.478187048
3692	AP002954.1	2.220410191	-0.26895024	1.852956848	-0.145068285
0.884656958	5.149924712
3693	AP002557.1	2.982197027	1.046150928	1.662742764	0.628792298
0.529484288	1.448277184
3694	ARMCX4 4.916551722	-1.174856866 1.202242871	-0.901487276
0.267229296	5.124111809
3695	ASIC2	2.25144882	1.020026594	2.007886072	0.512990557
0.607957926	2.481111415
3696	ATP5F1EP2	2.219568872	0.269091262	1.568146025	0.171598245
0.862752209	1.940918119

3697	AVEN	2.451161294	-0.522702811	1.822586656	-0.286792296	0.774271262	4.411870421
3698	AZIN1-AS1	2.702602868	-0.529719221	1.466487067	-0.268025446	0.712846804	1.110511289
3699	BIRC6-AS1	2.221701516	0.269988626	1.851107125	0.199874227	0.84157894	1.448277184
3700	BMS1P1	2.725522877	1.466259719	1.772542807	0.826740529	0.408284144	1.140158492
3701	BOD1	2.191215528	-1.686299977	1.648205096	-1.02211295	0.206254445	1.117474041
3702	BSPRY	6.126714214	-1.024911425	1.192122261	-0.867296822	0.285724617	1.191158045
3703	BX649622.1	2.240157495	0.2610646	1.872084447	0.19276472	0.847142224	5.141520804
3704	C10orf95	2.211954212	-0.261265574	1.891054255	-0.12815868	0.89011501	1.195092291
3705	C11orf80	5.682172926	-0.080076485	1.221266705	-0.065025857	0.948145451	1.110441217
3706	C12orf60	2.442705416	-0.515272598	1.959012624	-0.262077722	0.792490667	4.184904714
3707	C17orf107	2.709224499	-0.959788678	1.494625821	-0.642155529	0.520772198	4.449519892
3708	C1orf198	7.086278288	-1.09541017	1.151848181	-0.951002214	0.241602252	1.117474041
3709	C2orf88	2.429870069	-1.15694662	1.882420127	-0.614276266	0.529022718	1.719844158
3710	C6orf162	2.227222148	-0.284104947	1.885599792	-0.150670862	0.880225259	1.487911852
3711	CA12	2.442705416	-0.515272598	1.959012624	-0.262077722	0.792490667	2.498084424
3712	CASC4P1	2.211954212	-0.261265574	1.891054255	-0.12815868	0.89011501	1.494090404
3713	CCDC102	4.165056212	-2.1718948	1.502455008	-1.444602458	0.148569582	4.905241404
3714	CCDC120	2.966225019	-0.440029002	1.621197461	-0.271428281	0.786061576	1.71991421
3715	CCDC122	2.704445186	-1.2642696	1.751219007	-0.778995485	0.425982274	1.42712591
3716	CCDC168	4.916551722	-1.174856866	1.202242871	-0.901487276	0.267229296	2.194101011
3717	CCDC24	2.206282519	-1.750642152	2.057470572	-0.850871529	0.294840717	1.98408251
3718	CCDC20	4.176247528	-1.65281976	1.427755478	-1.149582251	0.250215519	2.478187048
3719	CCR2	2.72180715	-0.160527052	1.540015604	-0.104242782	0.916975889	2.11114402
3720	CCT8P1	2.940867082	-0.42221869	1.687215465	-0.250882955	0.801904602	1.957880128
3721	CD244	12.29586085	-1.179009826	0.909725622	-1.295991711	0.19497824	2.927141554
3722	CD96	2.492491249	1.26272859	1.82745289	0.691524201		

						0.489226178	1.002884858
3723	CDK14	6.282822452	-1.12104027	1.162971406	-0.962116761	0.225488921	1.715787448
3724	CELSR2	4.167891559	-1.649687529	1.428161976	-1.147080486	0.251248258	1.487424417
3725	CENPB	6.924616299	-0.167982129	1.156886555	-0.145202767	0.884550781	1.124587999
3726	CEP121	7.282042195	-0.14029011	1.122955991	-0.12271742	0.901529018	1.104284717
3727	CHIT1	5.454258069	0.2044202	1.270745224	0.229568201	0.810664942	1.490707128
3728	CHMP4B	2.478472296	-0.815559555	1.602117509	-0.508722484	0.610929049	1.114121011
3729	CHST10	4.184802516	-1.655958289	1.442222907	-1.147294252	0.251218716	5.421714555
3730	CHST14	4.429681291	-0.952517892	1.25872441	-0.701022171	0.482282215	2.948481817
3731	CILP2	4.474846692	0.258194597	1.400124214	0.255820567	0.79808167	1.10254927
3732	COL6A6	2.982146976	-0.451286548	1.682204479	-0.268270922	0.788490786	1.490419711
3733	COL9A1	2.698824555	-0.749725109	1.720281478	-0.425821182	0.662966472	1.111055411
3734	COMTD1	5.424410642	-1.728268845	1.21769981	-1.211580097	0.189661852	4.147991757
3735	CPEB2	2.22886617	-0.276467782	1.852179412	-0.1492662	0.881242585	1.10254927
3736	CPNE2	4.466290714	0.262917145	1.278481098	0.262272211	0.792240006	1.915550184
3737	CPO	2.725482815	-0.227694682	1.722790491	-0.121402469	0.895456144	1.114840494
3738	CPS1	1.998165028	2.858284159	2.244661897	1.219060267	0.222821219	4.428188419
3739	CREB5	2.475728072	1.275277187	1.606679441	0.856027086	0.291977266	2.450411172
3740	CROCC	7.106075629	-1.602016251	1.122462179	-1.428124956	0.152255895	2.191414714
3741	CUEDC1	2.969070265	0.047202222	1.619072779	0.029215692	0.976692565	1.704544284
3742	CUZD1	4.952261056	0.041862911	1.208616224	0.021990976	0.974479247	1.751547021
3743	CX2CR1	2.481208642	-0.282468975	1.584270486	-0.241416462	0.809222255	4.451017751
3744	CXADR	2.476579292	1.285585964	1.928628762	0.662128481	0.507241867	2.482957741
3745	CXCR6	2.971014221	-0.226606906	1.461492255	-0.222474795	0.822165998	1.124587999
3746	CYP1A1	2.211954212	-0.261265574	1.891054255	-0.12815868	0.89011501	1.195092291
3747	CYP2U1	2.461561229	-0.806400152	1.5299227	-0.527082108	0.598126571	1.717078972

3748 CYTL1 2.949222062 -0.429051176 1.641229426 -0.261402077
0.792781685 1.4907571
3749 CYYR1 2.006621007 2.847015124 2.241258284 1.215967252
0.222997297 4.457498444
3750 DDX47 2.22886617 -0.276467782 1.852179412 -0.1492662
0.881242585 1.117474041
3751 DENND5B 2.202606854 -1.122927495 1.589995226 -0.706252118
0.480021265 1.957880128
3752 DERL2 2.221701516 0.269988626 1.851107125 0.199874227 0.84157894
1.451111521
3753 DEXI 2.928684277 -1.525774824 1.469192725 -1.04521812
0.295875966 1.710147014
3754 DGKH 2.188520242 -0.620122589 1.596496589 -0.288422294
0.697695225 2.921410911
3755 DMPK 2.444649282 -0.797660251 1.519702298 -0.524879248
0.599667067 1.447887114
3756 DNAAF1 6.624875981 -0.949257286 1.150572772 -0.825029484
0.409254852 1.100714014
3757 DNAH10OS 5.662276561 -1.148140055 1.226766445 -0.925907612
0.249220757 2.915950118
3758 DNAH8 2.72419249 -0.76652601 1.716774758 -0.446497717
0.655227761 1.914841409
3759 DNAI2 5.200924216 -0.097067085 1.2844544 -0.075570674
0.929760668 2.429119948
3760 DNAJB7 7.117417016 -0.658122807 1.120677782 -0.582070168
0.560519418 1.940445512
3761 DNMBP-AS1 2.464246624 -1.827009825 1.657099219 -1.108569528
0.267615926 1.454047877
3762 DSTNP1 2.22886617 -0.276467782 1.852179412 -0.1492662
0.881242585 1.441502854
3763 DYNC1I2P1 2.221701516 0.269988626 1.851107125 0.199874227
0.84157894 1.100714014
3764 EBLN2 2.714845121 -1.422278982 1.557966274 -0.912549221
0.260952754 1.14209404
3765 EEF1A1P4 2.429870069 -1.15694662 1.882420127 -0.614276266
0.529022718 1.112194474
3766 EFCAB5 2.185694897 -1.114062228 1.602998927 -0.694986885
0.487062521 1.451111521
3767 EIF4BP2 2.452946679 -1.94646271 1.977109576 -0.984499157
0.224870146 1.951109545
3768 EIF4E2 2.482149962 -1.185965772 1.876107895 -0.622141561 0.52729428
2.441441527
3769 EMILIN1 6.667205925 -0.27792668 1.152800491 -0.240887989
0.809641921 4.148145452
3770 EPB42 2.715726512 -0.76092141 1.69987742 -0.447628989 0.65441276
1.124428041
3771 ESR1 2.209118866 -0.942509092 1.902840222 -0.495056821
0.620560012 4.11148884
3772 ESRRB 2.47652922 -0.542158697 1.776941895 -0.205107724
0.760284109 4.477445749
3773 ETF1P2 2.48502527 1.274224881 1.829411259 0.696527298

						0.486098668	1.714192488
3774	ETV7	2.946487715	-0.945860216	1.647582825	-0.574089621	0.56590715	2.177190054
3775	FABP2	2.96628508	1.062622129	1.797289467	0.591792896	0.552989281	1.10254927
3776	FAM110B	8.56290127	-1.479262165	1.041916507	-1.419752115	0.15567986	2.441275111
3777	FAM160B1	4.190474209	-0.828808724	1.291267679	-0.595679162	0.551289581	5.144214091
3778	FAM189B	5.161429608	-0.64940428	1.274694628	-0.509458784	0.610420681	1.454882114
3779	FAM207A	2.670621272	-1.242726767	1.964416884	-0.682529427	0.494272256	2.452154497
3780	FAM98C	2.927190418	-0.209254287	1.427640216	-0.215182062	0.829625275	1.104284717
3781	FBXO22	2.472692982	-1.180122488	1.822976749	-0.6428284	0.519686715	1.127412244
3782	FCMR	2.224486801	-0.962254097	1.929677128	-0.496141285	0.619794662	1.490419711
3783	FGF2	2.211954212	-0.261265574	1.891054255	-0.12815868	0.89011501	1.454047877
3784	FGFRL1	2.725522877	1.466259719	1.772542807	0.826740529	0.408284144	1.714192488
3785	FHL2	5.622881952	-1.795116115	1.26264208	-1.420587926	0.155426588	1.448174549
3786	FIZ1	2.741154509	0.845466244	1.725211521	0.487212004	0.626107291	1.72841024
3787	FMNL2	2.468072251	-0.525752204	1.766966808	-0.202205019	0.761722624	2.410874404
3788	FNDC10	6.299745409	-1.125544572	1.162862559	-0.96707688	0.222505602	1.998114144
3789	GABBR1	2.224526862	1.052621527	1.897427952	0.554762217	0.579057245	1.445229102
3790	GAPDHP65	2.969070265	0.047202222	1.619072779	0.029215692	0.976692565	1.118947248
3791	GEM	4.192209556	-0.467721968	1.286486622	-0.227250507	0.72585269	1.485124548
3792	GJB7	2.718571858	-0.214925002	1.682758764	-0.127727757	0.898264422	5.181118048
3793	GNG7	4.908095755	-1.171985511	1.206156715	-0.897277867	0.269570664	4.421547874
3794	GPR171	2.000950212	0.767221462	2.10225492	0.264999245	0.71511197	1.911170914
3795	GPSM1	2.977526244	0.041126824	1.629440972	0.025229842	0.979862657	1.94250087
3796	GRIN2B	2.196986222	-0.625075287	1.57201562	-0.29727292	0.691091744	1.111005249
3797	GRK5	2.217574844	-0.949112552	1.890850026	-0.501950202	0.61570255	1.725585012
3798	GSTM2	2.952158408	0.059841814	1.672120192	0.025766282	0.971468629	2.44475058

3799	GTF2IP20	2.954102274	-0.217892598	1.425042107	-0.222076479	0.822475984	1.492155059
3800	GTF2IRD2B	4.218677492	-0.479625428	1.422404616	-0.224605758	0.727922501	1.124587999
3801	H2BC20P	2.478522258	0.455266216	1.526280642	0.296222908	0.76698272	1.459448509
3802	HDGFL2	2.695989208	-1.259217412	1.75912098	-0.772664122	0.429721179	2.115190701
3803	HIGD1AP16	2.00278566	1.616872729	2.145079425	0.752759281	0.450992719	1.494090404
3804	HNRNPMP1	5.659591276	-0.588480807	1.222959625	-0.480800822	0.620658059	1.441502854
3805	HOMER2-AS1	2.429870069	-1.15694662	1.882420127	-0.614276266	0.529022718	4.488487012
3806	HOXB2	6.91208281	0.047642522	1.151279908	0.041278628	0.966994042	5.150189419
3807	HSP90AA2P	2.977526244	0.041126824	1.629440972	0.025229842	0.979862657	1.949111501
3808	HSP90AB2P	2.951267028	-0.692460876	1.420206514	-0.484822754	0.627794277	4.418491144
3809	ICA1L	6.410986672	-1.697611887	1.2049295	-1.408888974	0.158868001	1.140158492
3810	ICAM5	4.646255984	-0.717266809	1.28952765	-0.516190985	0.605721026	1.710147014
3811	IGIP	2.977526244	0.041126824	1.629440972	0.025229842	0.979862657	1.944224114
3812	IL1RAPL1	5.445802091	0.208184485	1.266251206	0.242282269	0.807708426	1.718411995
3813	INHBA	2.464296686	-0.272466604	1.515977462	-0.245694025	0.805919098	2.114824712
3814	IPO4	2.695989208	-1.259217412	1.75912098	-0.772664122	0.429721179	2.70449025
3815	IPO5P1	2.470908698	0.051747645	1.762672244	0.029257495	0.976579472	1.111005249
3816	ITGA6	4.916551722	-1.174856866	1.202242871	-0.901487276	0.267229296	1.485084504
3817	JMY	2.459617272	-0.529220194	1.779792592	-0.297411168	0.766152618	2.915950118
3818	KANK1	4.196144902	-0.121481872	1.29141014	-0.087208457	0.920426222	1.100714014
3819	KANSL1-AS1	2.977526244	0.041126824	1.629440972	0.025229842	0.979862657	1.942752544
3820	KCNC4	2.219518811	-1.121892924	1.625195259	-0.696465768	0.486127188	1.707221449
3821	KCTD12	4.425252085	-0.271711265	1.2596929	-0.199822892	0.841611276	1.114840494
3822	KIF6	2.718571858	-0.214925002	1.682758764	-0.127727757	0.898264422	2.447585917
3823	KITLG	2.452155422	0.472421266	1.577962922	0.200020299	0.764161596	2.447585917
3824	KLHDC1	2.448226048	-1.162928296	1.827220789	-0.622984228		

						0.526742877	4.482044281
3825	KRBA1	2.700818582	0.246814468	1.495024825	0.165090547	0.868872712	1.110511289
3826	KRT72	2.22886617	-0.276467782	1.852179412	-0.1492662	0.881242585	7.28104217
3827	KRT8P42	2.220410191	-0.26895024	1.852956848	-0.145068285	0.884656958	2.45881719
3828	LCAT	2.678225922	-0.526661772	1.505722414	-0.24977225	0.726508876	2.44475058
3829	LCMT1-AS2	2.212898179	-0.624788628	1.571416175	-0.402959587	0.686242428	2.441915122
3830	LEF1	2.000950212	0.767221462	2.10225492	0.264999245	0.71511197	4.194542442
3831	LINC00210	2.47652922	-0.542158697	1.776941895	-0.205107724	0.760284109	2.408041158
3832	LINC01002	2.721407205	0.212892606	1.692290456	0.185274924	0.852924968	1.10254927
3833	LINC01160	2.487870717	2.060121202	1.998766921	1.020701119	0.202681	1.111055411
3834	LINC01270	2.976685025	0.400590812	1.460102567	0.274257807	0.782809664	1.71582771
3835	LINC01266	2.928021726	-0.940820224	1.671007724	-0.562021699	0.572412299	2.489578292
3836	LINC01572	5.17555628	-0.086569622	1.284996567	-0.067269544	0.946287514	1.002884858
3837	LINC02469	2.92519629	-1.5274405	1.710120125	-0.892172086	0.271765024	4.845844998
3838	LMTK2	4.196144902	-0.121481872	1.29141014	-0.087208457	0.920426222	1.957820174
3839	LOX	1.998165028	2.858284159	2.244661897	1.219060267	0.222821219	4.480121024
3840	LRP1B	2.000950212	0.767221462	2.10225492	0.264999245	0.71511197	1.112194474
3841	LRRC24	2.971905712	0.525608144	1.62769868	0.227049261	0.742620627	1.114840494
3842	LRRC27BP1	2.707280522	-0.755267227	1.701029921	-0.44406462	0.656995858	2.489578292
3843	LRRC45	2.927190418	-0.209254287	1.427640216	-0.215182062	0.829625275	4.444154414
3844	LSR	4.67172292	-0.727412077	1.226614901	-0.548222709	0.582470227	1.114121011
3845	LTB4R2	2.224526862	1.052621527	1.897427952	0.554762217	0.579057245	2.115190701
3846	LYSMD2	9.65229052	-0.802277091	1.045759514	-0.767267211	0.4429226	1.494277821
3847	LZTFL1	2.701659901	-0.202164025	1.727710896	-0.11622928	0.907282656	1.109110044
3848	MARK4	2.47652922	-0.542158697	1.776941895	-0.205107724	0.760284109	4.428128548
3849	MBL1P	4.668888572	-1.06642082	1.220657176	-0.801421682	0.422881779	1.921441141

3850	MCPH1-AS1	4.152814949	-1.204672206	1.429982849	-0.826588649	0.402822828	1.448114488
3851	MEMO1	4.224248185	0.206422627	1.426797049	0.144682245	0.884960969	4.187740041
3852	MIR221	2.224526862	1.052621527	1.897427952	0.554762217	0.579057245	2.940014829
3853	MIR2978	5.198088869	-0.275521262	1.282876189	-0.292498025	0.769905862	1.957880128
3854	MIR616	2.455940707	-0.267672446	1.509688109	-0.24254199	0.807585561	2.429079884
3855	MKRN5P	2.698824555	-0.749725109	1.720281478	-0.425821182	0.662966472	2.498024271
3856	MLANA	2.718571858	-0.214925002	1.682758764	-0.127727757	0.898264422	1.140158492
3857	MNS1	2.47652922	-0.542158697	1.776941895	-0.205107724	0.760284109	1.121751452
3858	MOK	2.464296686	-0.272466604	1.515977462	-0.245694025	0.805919098	1.154285245
3859	MORF4L2-AS1	2.722251171	-0.155456486	1.492982221	-0.104055024	0.917125675	1.744091052
3860	MRPL28	2.219568872	0.269091262	1.568146025	0.171598245	0.862752209	5.104494107
3861	MRPL52P1	2.226020822	-0.955675607	1.900256966	-0.50289268	0.615029728	1.707281721
3862	MRVI1	4.705547824	-0.74072888	1.256097225	-0.546221027	0.584914014	4.28192445
3863	MTA1	2.202656915	0.281429719	1.621190215	0.172594498	0.862184156	1.718411995
3864	MTOR	2.71011588	-0.208649895	1.695274212	-0.122070112	0.902051578	4.117124448
3865	MXRA7	2.248662525	2.050240427	2.272221212	1.24174924	0.179677216	5.147404124
3866	MYO5B	2.724242552	0.862472681	1.728122058	0.496206262	0.619748822	5.870444211
3867	MYOM1	2.501947227	1.252257259	1.922201276	0.64806519	0.516942787	4.192240491
3868	NAALAD2	2.426192404	-0.792274812	1.521951654	-0.517819742	0.604584042	1.485084504
3869	NCK2	2.917292052	-2.062250751	1.524982698	-1.244217595	0.178878022	4.181049247
3870	NHLRC4	2.456782026	-1.168715087	1.818040868	-0.642842122	0.520225892	2.420412908
3871	NLGN2	2.46245272	0.059241402	1.799089762	0.022928542	0.972721572	1.118947248
3872	NOL2	2.727027827	-0.221216818	1.692997928	-0.120665725	0.896029729	4.908144891
3873	NOS2	4.165056212	-2.1718948	1.502455008	-1.444602458	0.148569582	1.114840494
3874	NPEPL1	2.949272	-2.272924589	1.858125267	-1.222240774	0.22122877	1.490419711
3875	NPEPPSP1	2.470908698	0.051747645	1.762672244	0.029257495		

						0.976579472	1.447887114
3876	NPHP2	2.726186518	0.220965146	1.492242872	0.15467252	0.877078699	4.194194029
3877	NSRP1P1	1.995229681	1.622151187	2.052172212	0.794941162	0.426647728	2.700849719
3878	NYNRIN	2.451161294	-0.522702811	1.822586656	-0.286792296	0.774271262	1.995280818
3879	OR8B7P	2.710065818	-2.122527089	1.921995827	-1.104860406	0.269220081	1.947177525
3880	OTOF	2.467222022	0.044081451	1.514912258	0.029098221	0.976786167	0.987744158
3881	PATL2	2.974690997	-0.445559128	1.628404228	-0.271947014	0.785662761	1.991545471
3882	PCDHGA10	2.974690997	-0.445559128	1.628404228	-0.271947014	0.785662761	0.990401505
3883	PCOLCE	2.200662887	-0.925694774	1.946822852	-0.480626215	0.620782109	1.151291227
3884	PCSK6	2.727477842	0.618711977	1.528526286	0.402142221	0.687578626	0.502721142
3885	PERP	5.890292417	-1.527229482	1.215896861	-1.26425846	0.206101296	0.502721142
3886	PHETA1	4.421225412	-0.949205167	1.2625268	-0.696645527	0.486024657	0.502721142
3887	PIN4P1	2.985982222	0.024722206	1.668971792	0.02081114	0.982296211	1.44055989
3888	PKD1P6	5.684959211	-0.59699822	1.222161294	-0.484512005	0.628021827	0.748558974
3889	PLA2G4C	2.479214615	-1.962141222	2.050166191	-0.957064472	0.228524715	1.004718177
3890	PLEKHA7	2.214789559	0.288720522	2.008891098	0.192500047	0.846567269	0.502721142
3891	PMS2CL	2.479214615	-1.962141222	2.050166191	-0.957064472	0.228524715	1.970011881
3892	PMS2P7	2.219568872	0.269091262	1.568146025	0.171598245	0.862752209	0.502721142
3893	POMT2	4.651976616	-1.060221516	1.250442259	-0.785098899	0.422295585	1.457714542
3894	POU6F1	2.465288066	0.650458689	1.922582519	0.228149429	0.725250575	0.500895814
3895	PPIL6	2.240157495	0.2610646	1.872084447	0.19276472	0.847142224	0.75411947
3896	PPP1R1A	4.449478757	0.272107902	1.279159602	0.269807716	0.787208187	1.121444022
3897	PPP1R25	5.209220122	-0.967822705	1.250081022	-0.716870821	0.472452802	0.994111124
3898	PPP2R2B	2.444649282	-0.797660251	1.519702298	-0.524879248	0.599667067	0.491429828
3899	PPP2R5B	2.942652268	-1.521992192	1.698082017	-0.902189752	0.266956078	0.500895814
3900	PPP5D1	2.724242552	0.862472681	1.728122058	0.496206262	0.619748822	0.491429828

3901	PRKAR1B-AS1	4.129688277	-2.162888258	1.528565282	-1.414979217	0.15707456	0.999057482
3902	PRKCZ	4.925057772	-0.228215767	1.206679287	-0.182206224	0.855242414	0.748558974
3903	PRSS20P	4.196094841	-1.221528646	1.41862551	-0.861065889	0.289201755	1.71119944
3904	PSME2P2	2.220860198	0.720796856	1.590757589	0.452115459	0.650465604	1.119808484
3905	PSORS1C1	2.465228005	-1.174411264	1.816628618	-0.646474952	0.517971812	0.721447019
3906	PTGDR	2.700818582	0.246814468	1.495024825	0.165090547	0.868872712	1.500054497
3907	PTOV1-AS1	2.451161294	-0.522702811	1.822586656	-0.286792296	0.774271262	1.719514275
3908	PTPN12	2.746825202	2.215626661	1.86629825	1.187182521	0.225155624	0.502721142
3909	PTPRK	7.629605222	-1.4812869	1.092456112	-1.25468245	0.175518422	1.00189182
3910	PTX2	2.200662887	-0.925694774	1.946822852	-0.480626215	0.620782109	1.125419218
3911	PWAR5	4.207426228	0.215828822	1.298562419	0.154228956	0.877250267	0.721447019
3912	RAD21-AS1	2.245828188	1.852922729	1.978040084	0.927252261	0.248628761	0.502721142
3913	RALGAPA1	2.707280522	-0.755267227	1.701029921	-0.44406462	0.656995858	0.740101998
3914	RBM22P2	2.240107422	-1.774686455	2.274289279	-0.780225726	0.425199168	0.740101998
3915	RFPL1S	2.226480829	0.256695054	1.617641107	0.158684799	0.872917215	0.741928245
3916	RGS20	2.452946679	-1.94646271	1.977109576	-0.984499157	0.224870146	0.502721142
3917	RIPOR2	5.915710414	-0.692124299	1.200102722	-0.576729209	0.564122298	0.721447019
3918	RMI2	2.444649282	-0.797660251	1.519702298	-0.524879248	0.599667067	0.751294212
3919	RNA5SP216	2.221701516	0.269988626	1.851107125	0.199874227	0.84157894	0.992424851
3920	ROM1	2.456782026	-1.168715087	1.818040868	-0.642842122	0.520225892	0.748558974
3921	RPL17P50	4.462555267	0.02962982	1.272507051	0.028852022	0.976981812	0.75411947
3922	RPL21P28	2.222245528	0.278917827	1.876942612	0.201880245	0.840010246	0.751294212
3923	RPL21P75	2.219568872	0.269091262	1.568146025	0.171598245	0.862752209	0.491429828
3924	RPL22AP2	2.482200022	0.622202269	1.774958268	0.256178667	0.721706767	1.477471847
3925	RPL29P22	2.245828188	1.852922729	1.978040084	0.927252261	0.248628761	0.990401505
3926	RPL24P18	2.222245528	0.278917827	1.876942612	0.201880245		

0.840010246	0.502721142
3927	RPL25P1	2.240157495	0.2610646	1.872084447	0.19276472
0.847142224	0.75411947
3928	RPL7AP20	2.245778127	-0.292470977	2.020482108	-0.144752992
0.884905898	0.999057482
3929	RPL7AP6	2.470908698	0.051747645	1.762672244	0.029257495
0.976579472	0.491429828
3930	RPS10P7	2.492491249	1.26272859	1.82745289	0.691524201
0.489226178	0.491429828
3931	RPS15AP17	2.966225019	-0.440029002	1.621197461	-0.271428281
0.786061576	0.502721142
3932	RPS26P6	2.456782026	-1.168715087	1.818040868	-0.642842122
0.520225892	0.500895814
3933	RPS27P21	2.952158408	0.059841814	1.672120192	0.025766282
0.971468629	0.500895814
3934	RPS2P22	4.702762549	-0.109842805	1.222067711	-0.082460277
0.924280626	0.502721142
3935	RPS2P46	2.715726512	-0.76092141	1.69987742	-0.447628989
0.65441276	0.75411947
3936	RTEL1-TNFRSF6B	2.692152861	-2.114226012	1.912294247	-
1.104960928	0.269176515	0.751294212
3937	RTKN	2.157491614	-1.669242641	1.746716207	-0.955702921
0.229221826	1.717970254
3938	S100A2	2.16878294	-1.104757508	1.679121604	-0.657922842
0.510580642	0.500895814
3939	S100Z	4.680179898	-0.720699841	1.222846171	-0.552269472
0.580695242	0.999057482
3940	SAMSN1-AS1	2.704495248	0.229228818	1.852785948	0.177657414
0.858992022	0.502721142
3941	SBNO1-AS1	2.681021217	-1.408407962	1.506110107	-0.92512948
0.249721576	0.740101998
3942	SCAF1	2.467181971	-1.284918184	1.589221059	-0.808515651	0.4187928
1.970011881
3943	SCOC-AS1	2.47652922	-0.542158697	1.776941895	-0.205107724
0.760284109	0.721447019
3944	SCRG1	2.472744045	0.640897606	1.799914748	0.25607109
0.721787227	0.502721142
3945	SDHAF4	2.922062746	-1.0828572	1.448704699	-0.74815606
0.454266021	0.502721142
3946	SEC14L1P1	2.695989208	-1.259217412	1.75912098	-0.772664122
0.429721179	0.990401505
3947	SEC24B-AS1	2.980261691	0.528566565	1.62662509	0.222959226
0.746726046	1.00189182
3948	SEMA2D	2.222245528	0.278917827	1.876942612	0.201880245
0.840010246	0.741928245
3949	SEMA4B	5.192468227	-0.092548822	1.276206652	-0.07220226
0.941565592	1.717970254
3950	SERPIND1	2.715726512	-0.76092141	1.69987742	-0.447628989
0.65441276	0.999057482
3951	SGMS2	2.729812122	-1.27956644	1.8221909	-0.756676901
0.449242457	0.500895814

3952	SHISA4	4.685850592	-0.102201598	1.2226067	-0.076692994	0.928867778	1.151291227
3953	SHROOM1	5.445802091	0.208184485	1.266251206	0.242282269	0.807708426	0.491429828
3954	SIDT1	2.211062822	-1.127275202	1.600594617	-0.704247741	0.481216242	1.128144445
3955	SIPA1L2	2.945596224	-1.542552829	1.482258282	-1.040678165	0.29802494	0.502721142
3956	SLC26A1	4.440972716	-0.606980926	1.252948012	-0.448204455	0.652922486	1.978448841
3957	SLC25E2A	2.712901165	-1.269295495	1.756819125	-0.779417457	0.425722844	0.502721142
3958	SLC49A2	2.971905712	0.525608144	1.62769868	0.227049261	0.742620627	0.491429828
3959	SLC6A16	2.220410191	-0.26895024	1.852956848	-0.145068285	0.884656958	0.75411947
3960	SLFNL1-AS1	4.224248185	0.206422627	1.426797049	0.144682245	0.884960969	1.004718177
3961	SNX25	5.690629904	-0.082298128	1.224629289	-0.067467585	0.946209466	0.502721142
3962	SP2-AS1	2.217574844	-0.949112552	1.890850026	-0.501950202	0.61570255	0.491429828
3963	SPACA6	2.000950212	0.767221462	2.10225492	0.264999245	0.71511197	0.502721142
3964	SPINK2	2.721407205	0.212892606	1.692290456	0.185274924	0.852924968	0.721447019
3965	SRP54-AS1	2.726977775	-2.122904878	1.994821224	-1.069215707	0.284972482	0.502721142
3966	ST7-AS1	4.201815596	0.567862501	1.42274549	0.296245691	0.691850025	0.992424851
3967	STAT2	2.726126456	-0.968192619	1.571228571	-0.616157865	0.527790224	0.97921018
3968	STS	2.698824555	-0.749725109	1.720281478	-0.425821182	0.662966472	0.740101998
3969	STX2	5.411928115	-0.479889025	1.251592091	-0.282422865	0.701406252	0.741928245
3970	SULT6B1	2.712059846	-0.544058225	1.477558998	-0.268214282	0.712712462	0.721447019
3971	SUMO4	2.220410191	-0.26895024	1.852956848	-0.145068285	0.884656958	1.142925258
3972	SUSD2	2.472692982	-1.180122488	1.822976749	-0.6428284	0.519686715	1.488742171
3973	SYCP2	2.248612472	0.251257827	2.000162887	0.175614528	0.860596788	1.141100011
3974	SYN1	2.224526862	1.052621527	1.897427952	0.554762217	0.579057245	0.491429828
3975	SYNE2	2.675400585	-0.942855561	1.496922229	-0.62052022	0.528247665	0.981145514
3976	TACR1	2.240157495	0.2610646	1.872084447	0.19276472	0.847142224	1.488742171
3977	TAFA2	5.169925649	0.194267201	1.221607528	0.145964404		

0.882949489 0.502721142
3978 TANC2 4.284566152 -1.215964022 1.448824466 -0.908297771 0.26272092
1.121444022
3979 TAS2R5 2.211954212 -0.261265574 1.891054255 -0.12815868 0.89011501
1.70811205
3980 TBCAP1 2.479264677 0.04441228 1.761195727 0.025217174
0.979881728 1.741044944
3981 TCF7L1 2.472902726 0.897200422 1.552707006 0.577829824
0.562279011 0.740101998
3982 THNSL2 2.49060594 -1.192288582 2.010098412 -0.592149258
0.552081201 0.741928245
3983 THSD7A 2.695989208 -1.259217412 1.75912098 -0.772664122
0.429721179 0.491429828
3984 TIAF1 2.741154509 0.845466244 1.725211521 0.487212004
0.626107291 1.121444022
3985 TM9SF1 4.462555267 0.02962982 1.272507051 0.028852022
0.976981812 0.491429828
3986 TMEM108-AS1 2.959722006 -0.697424222 1.444822624 -0.482712424
0.629299924 1.142885197
3987 TMEM122A 2.224526862 1.052621527 1.897427952 0.554762217
0.579057245 1.111251708
3988 TMEM142 5.164264955 -0.262467924 1.284628004 -0.282157888
0.777822447 0.994111124
3989 TMEM14EP 2.492491249 1.26272859 1.82745289 0.691524201
0.489226178 0.740101998
3990 TMEM182 4.449428695 -0.610564655 1.262067048 -0.448262289
0.652962191 0.721447019
3991 TMEM222 2.225189504 -0.182195642 1.579292485 -0.115991125
0.907659568 1.151291227
3992 TMEM25 2.701659901 -0.202164025 1.727710896 -0.11622928
0.907282656 0.491429828
3993 TMEM27 4.42812727 -0.955824741 1.261866405 -0.701856465
0.482768681 1.488475744
3994 TMEM67 2.690268576 -0.742894027 1.766518276 -0.421107428
0.672676621 0.491429828
3995 TMEM74B 6.157752942 -0.52780986 1.182522228 -0.454795098
0.649256658 0.987744158
3996 TMTC2 2.208277547 -0.172269781 1.562862212 -0.110291086
0.912178527 0.491429828
3997 TNFRSF18 2.496226695 2.046090824 1.917009667 1.067224641
0.285820751 0.491429828
3998 TOR4A 5.918545761 -0.429627452 1.202657122 -0.26554679
0.714702287 0.741928245
3999 TPM2P1 2.472744045 0.640897606 1.799914748 0.25607109
0.721787227 0.987744158
4000 TRAT1 2.72180715 -0.160527052 1.540015604 -0.104242782
0.916975889 0.981145514
4001 TRIM22 5.668047254 -0.591204125 1.221122622 -0.484229544
0.628222972 0.500895814
4002 TRPA1 2.729862184 0.206714296 1.692045162 0.181268286
0.856156919 1.712842481

4003	TSPAN10	2.70160984	-2.118894956	1.912094777	-1.108152728	0.26779542	0.75411947
4004	TTC29	2.217574844	-0.949112552	1.890850026	-0.501950202	0.61570255	0.748558974
4005	TTC20B	2.229216176	0.712676429	1.622728846	0.42980018	0.660081827	1.125419218
4006	TTC4	2.949222062	-0.429051176	1.641229426	-0.261402077	0.792781685	0.970854101
4007	TUFT1	2.687522229	-1.254067252	1.782216875	-0.759722128	0.447420101	1.144710442
4008	U47924.1	2.715726512	-0.76092141	1.69987742	-0.447628989	0.65441276	0.741928245
4009	U72169.1	4.152814949	-1.204672206	1.429982849	-0.826588649	0.402822828	0.740101998
4010	UBA52P6	2.220410191	-0.26895024	1.852956848	-0.145068285	0.884656958	0.502721142
4011	UBR5-AS1	2.692212542	-0.951822202	1.476194492	-0.644781096	0.519069054	0.741928245
4012	UCHL1	2.22727221	1.868502622	2.067721065	0.902652125	0.266179257	1.494422845
4013	UGDH-AS1	5.652920582	-1.14561422	1.224729007	-0.925402292	0.249581016	0.491429828
4014	UNC12B	2.920278461	-0.200400524	1.51218122	-0.198522489	0.842626291	1.7208057
4015	VLDLR-AS1	2.219518811	-1.121892924	1.625195259	-0.696465768	0.486127188	1.712842481
4016	VMAC	2.980211629	-0.965901288	1.712299521	-0.562766796	0.572912852	0.502721142
4017	WAKMAR2	2.945596224	-1.542552829	1.482258282	-1.040678165	0.29802494	1.991545471
4018	WASF2	2.966225019	-0.440029002	1.621197461	-0.271428281	0.786061576	0.740101998
4019	WASH2P	2.224526862	1.052621527	1.897427952	0.554762217	0.579057245	1.00189182
4020	WASH9P	2.174402571	-1.678056085	1.668567592	-1.005686609	0.214566247	0.502721142
4021	XIRP2	2.00278566	1.616872729	2.145079425	0.752759281	0.450992719	1.118517241
4022	Z68871.1	2.969070265	0.047202222	1.619072779	0.029215692	0.976692565	1.11497224
4023	ZAP70	2.209118866	-0.942509092	1.902840222	-0.495056821	0.620560012	0.491429828
4024	ZFPM1	2.006621007	2.847015124	2.241258284	1.215967252	0.222997297	0.502721142
4025	ZFYVE9	5.417498685	-1.722292966	1.288152618	-1.227801858	0.180961026	1.142925258
4026	ZKSCAN2-DT	2.209118866	-0.942509092	1.902840222	-0.495056821	0.620560012	1.14955599
4027	ZMYND12	2.212898179	-0.624788628	1.571416175	-0.402959587	0.686242428	0.990401505
4028	ZNF185	2.988817669	0.521224972	1.669172425	0.212221221		

0.754788815 1.11497224
4029 ZNF19 2.969070265 0.047202222 1.619072779 0.029215692
0.976692565 1.480207192
4030 ZNF222 10.02585278 -1.054055496 0.996616404 -1.057624102
0.290222289 1.714479018
4031 ZNF222 2.712951227 0.221227409 1.728902989 0.185798292
0.852602942 0.75411947
4032 ZNF229 4.452264042 -0.279410565 1.259029621 -0.205594128
0.827107965 0.500895814
4033 ZNF211 2.407990121 -1.256128944 1.64416045 -0.762994127
0.444870727 1.111251708
4034 ZNF229 2.450270014 -1.276744021 1.54828261 -0.824566242
0.409617892 1.004718177
4035 ZNF56 2.456782026 -1.168715087 1.818040868 -0.642842122
0.520225892 1.720755429
4036 ZNF571-AS1 4.409924088 -1.225619272 1.282015551 -0.959192804
0.227461625 0.740101998
4037 ZNF598 2.482149962 -1.185965772 1.876107895 -0.622141561 0.52729428
1.485917815
4038 ZNF70 4.685850592 -0.102201598 1.2226067 -0.076692994
0.928867778 0.491429828
4039 ZNF726 2.951267028 -0.692460876 1.420206514 -0.484822754
0.627794277 0.721447019
4040 ZNF805 7.168152887 -0.671622092 1.120665298 -0.594015784
0.552501556 0.721447019
4041 ZNNT1 2.450270014 -1.276744021 1.54828261 -0.824566242
0.409617892 0.741928245
4042 ZSCAN12 4.929124284 -0.541792427 1.297108297 -0.417692482
0.676171969 1.111251708
4043 ABCC6 2.452054224 0.056260226 1.516057846 0.027175577
0.970245012 1.975422514
4044 ABHD1 2.211852015 -0.928626698 2.019421575 -0.464799777
0.642074847 0.751294212
4045 AC000068.1 2.227271012 1.054642522 1.88562422 0.559204406
0.575952989 1.11497224
4046 AC002091.1 4.970121876 0.626686149 1.202216928 0.480876207
0.620604412 0.502721142
4047 AC002091.2 5.424409568 -0.216102054 1.221049519 -0.176980581
0.859522642 0.751294212
4048 AC002525.1 2.211852015 -0.928626698 2.019421575 -0.464799777
0.642074847 0.502721142
4049 AC004158.1 2.746672942 -0.222489545 1.858597211 -0.125088712
0.900452219 0.500895814
4050 AC004241.5 2.484924172 0.624206421 1.762112529 0.259708221
0.719065244 0.748558974
4051 AC004286.4 2.501896191 2.221018821 2.192485972 1.469117185
0.141801007 1.970011881
4052 AC004884.2 2.179972067 -1.106869128 1.650502691 -0.670625461
0.502459158 0.75411947
4053 AC004942.2 4.176296401 -0.819889062 1.289661046 -0.589992117
0.555195925 0.748558974

4054	AC005222.1	2.251297684	2.051828454	2.252967821	1.254585905	0.175549527	0.987744158
4055	AC005670.2	2.458674856	-0.265825902	1.498227821	-0.244170784	0.807098554	0.981145514
4056	AC005696.1	2.009255155	2.84902704	2.224581892	1.225608277	0.220246084	1.4744245
4057	AC005697.2	5.120289782	-1.620242494	1.218204219	-1.229025261	0.219058586	1.11497224
4058	AC005785.1	2.242891644	0.262422891	1.872502098	0.194089444	0.846105847	0.491429828
4059	AC005821.1	2.968969167	-0.427855629	1.620851052	-0.270129242	0.787052055	0.994111124
4060	AC005829.1	2.247670957	0.251525702	1.715120978	0.146651017	0.882407482	0.491429828
4061	AC005942.2	4.964451182	0.029220159	1.295482022	0.020259455	0.97578028	0.491429828
4062	AC006222.1	2.468022215	0.65247149	1.911052447	0.241419802	0.72278757	0.751294212
4063	AC007098.1	2.966122821	-0.95219511	1.667282514	-0.571671185	0.567544759	1.444180511
4064	AC007282.1	2.496225497	1.265578589	1.809272207	0.699495955	0.48424214	0.75411947
4065	AC007672.1	6.127904441	-1.026010815	1.164080621	-0.889982012	0.272475544	0.748558974
4066	AC007728.1	2.248562227	1.855749182	1.960444576	0.946596097	0.242844624	1.44055989
4067	AC007928.2	2.242891644	0.262422891	1.872502098	0.194089444	0.846105847	0.994111124
4068	AC008105.2	2.242891644	0.262422891	1.872502098	0.194089444	0.846105847	0.741928245
4069	AC008428.1	2.224425665	0.272225827	1.850521221	0.201204828	0.840528408	1.978448841
4070	AC008569.2	2.242891644	0.262422891	1.872502098	0.194089444	0.846105847	0.740101998
4071	AC008680.1	1.995228484	0.781822892	1.979482707	0.294968087	0.69286644	0.500895814
4072	AC008728.2	2.24572699	1.042882529	1.882412275	0.554251208	0.579406927	0.502721142
4073	AC008764.10	2.721206007	-0.212701801	1.682252558	-0.126421169	0.899290621	0.500895814
4074	AC009022.1	2.974629861	0.527202524	1.61920087	0.221822521	0.740015718	0.999057482
4075	AC009078.2	5.192267029	-0.270900975	1.247589224	-0.297294148	0.766241949	0.741928245
4076	AC009090.2	2.95205721	-0.426847199	1.640985194	-0.260116422	0.79477297	1.984089491
4077	AC009092.2	2.452004162	-1.27422956	1.568912149	-0.812227678	0.416655264	1.711058297
4078	AC009245.1	2.225979687	0.281284912	1.876254126	0.202205198	0.82897465	0.502721142
4079	AC009620.1	2.482098825	0.046704599	1.760714954	0.026525929		

						0.978827852	0.999057482
4080	AC009821.2	2.227271012	1.054642522	1.88562422	0.559204406	0.575952989	0.500895814
4081	AC010245.1	2.690267278	-1.250295982	1.882218556	-0.717015016	0.472264821	0.740101998
4082	AC011270.2	2.472642847	0.054029801	1.762188854	0.020666292	0.975525672	0.75411947
4083	AC011815.2	2.205241002	-1.120452725	1.611140277	-0.69544141	0.486778717	0.500895814
4084	AC011922.2	5.692264052	-0.082126119	1.204619925	-0.068175959	0.945645565	1.449015848
4085	AC012258.1	2.441712827	-1.822610848	1.687488222	-1.080072162	0.280109602	0.500895814
4086	AC012258.2	2.960512189	-0.422291569	1.621811681	-0.26661022	0.789769286	0.502721142
4087	AC012425.2	2.22214424	-0.266129216	1.877260792	-0.141764701	0.887265867	0.721447019
4088	AC012615.1	4.428086224	-0.270209027	1.226988255	-0.202177548	0.829777925	0.491429828
4089	AC015911.6	2.484924172	0.624206421	1.762112529	0.259708221	0.719065244	0.502721142
4090	AC015912.1	5.144416454	-0.94294529	1.279296028	-0.727022847	0.461107848	0.500895814
4091	AC015987.1	2.248562227	1.855749182	1.960444576	0.946596097	0.242844624	0.721447019
4092	AC016294.2	5.958029294	1.222215552	1.267451897	1.051176422	0.29217757	1.118517241
4093	AC016542.2	2.742888658	0.847215964	1.716922249	0.492508224	0.62165246	1.975422514
4094	AC016542.2	2.490554804	0.029250627	1.794056424	0.021878151	0.982545154	0.740101998
4095	AC016682.1	2.240106259	1.870206598	2.048865508	0.912801056	0.261247182	0.500895814
4096	AC016949.1	2.717679402	1.056825702	1.522174906	0.692820826	0.487788209	0.500895814
4097	AC018529.2	2.251247622	0.252724822	1.99949604	0.176906988	0.85958145	0.491429828
4098	AC018682.1	5.422118242	-0.481652971	1.220021448	-0.294791296	0.692996845	1.14955599
4099	AC018755.4	2.985921186	1.047719872	1.641022206	0.628451215	0.522179926	1.128144445
4100	AC018816.2	2.194049677	-1.682062925	1.724691801	-0.970228018	0.221927864	0.502721142
4101	AC019120.1	2.99160188	2.281526122	1.865050822	1.276928264	0.201627574	0.987744158
4102	AC020909.2	2.248562227	1.855749182	1.960444576	0.946596097	0.242844624	0.990401505
4103	AC020910.6	2.225979687	0.281284912	1.876254126	0.202205198	0.82897465	0.741928245
4104	AC020914.2	2.725422679	0.855729028	1.695610407	0.504679027	0.612784287	0.75411947

4105	AC020917.1	2.997222511	1.627226597	1.699876226	0.957267695
	0.228422156	1.118517241			
4106	AC020951.1	1.995228484	0.781822892	1.979482707	0.294968087
	0.69286644	0.502721142			
4107	AC022150.1	2.982095829	0.520226222	1.61815282	0.227725629
	0.742111562	0.721447019			
4108	AC022415.2	2.469966181	0.045772178	1.496504999	0.020586051
	0.975599667	1.722441047			
4109	AC022497.1	2.697882028	-0.52261222	1.455756722	-0.26655284
	0.712951845	0.748558974			
4110	AC022289.1	2.712850029	-0.206404568	1.694962724	-0.121775284
	0.902076989	0.491429828			
4111	AC022820.4	2.490554804	0.029250627	1.794056424	0.021878151
	0.982545154	0.751294212			
4112	AC022982.1	4.708222044	0.196822406	1.208992707	0.150261728
	0.880479224	0.741928245			
4113	AC024896.1	6.422568249	0.044560796	1.148029085	0.028815041
	0.969027852	1.989740184			
4114	AC025918.1	2.221600219	-0.272712596	1.875455829	-0.14594457
	0.882965147	0.748558974			
4115	AC026191.1	2.962507217	0.412267225	1.417282829	0.290956069
	0.771084918	0.740101998			
4116	AC026402.1	2.244885672	1.209062577	1.615940068	0.748210655
	0.454222105	0.751294212			
4117	AC026725.1	2.452054224	0.056260226	1.516057846	0.027175577
	0.970245012	1.719514275			
4118	AC027644.2	5.904217891	-1.222799861	1.18874859	-1.027056844
	0.299709266	0.740101998			
4119	AC055811.1	2.496225497	1.265578589	1.809272207	0.699495955
	0.48424214	0.751294212			
4120	AC060224.2	2.720414627	-0.961796162	1.521767626	-0.622025629
	0.527270124	0.500895814			
4121	AC068724.2	2.212847042	0.276809582	1.561811204	0.177226252
	0.859222821	0.740101998			
4122	AC072896.4	2.225979687	0.281284912	1.876254126	0.202205198
	0.82897465	1.14955599			
4123	AC079140.1	2.722597222	0.208698198	1.680248285	0.182721764
	0.854221724	1.118517241			
4124	AC079192.2	4.694205272	-0.412250287	1.201269855	-0.21765155
	0.750749266	1.482091478			
4125	AC080012.1	4.668827427	-0.4027718	1.228276004	-0.202228997
	0.761715252	0.502721142			
4126	AC080027.2	6.118157127	-1.207297285	1.18647875	-1.101829498
	0.270525804	0.502721142			
4127	AC082964.1	6.246072994	-1.679502772	1.19620944	-1.402904128
	0.160247297	0.75411947			
4128	AC084298.2	6.621989498	-0.710275246	1.142119819	-0.621248029
	0.524270659	1.471851115			
4129	AC087276.4	2.996281192	1.622422446	1.512605855	1.072560221
	0.282468476	0.491429828			
4130	AC087289.4	2.669678756	-0.927646024	1.525220254	-0.614716688

						0.528741827	0.502721142
4131	AC087741.1	5.721617297	0.94800528	1.250828425	0.757895946	0.448512277	0.741928245
4132	AC090510.1	2.945495126	-2.070054251	1.624464292	-1.274299615	0.202557252	0.500895814
4133	AC090510.2	2.979419174	0.402070285	1.424180225	0.280248575	0.779210087	1.48214154
4134	AC090970.1	2.724141254	0.215829029	1.681481778	0.187822757	0.851006972	0.741928245
4135	AC091492.1	2.682755266	-1.406012256	1.525846411	-0.921464526	0.256807948	0.740101998
4136	AC091887.1	2.22214424	-0.266129216	1.877260792	-0.141764701	0.887265867	0.748558974
4137	AC092125.2	2.710014682	-0.75270841	1.722261795	-0.426767492	0.662279977	1.118517241
4138	AC092216.1	2.962457155	-0.6956009	1.422792825	-0.485147089	0.627572012	1.501889844
4139	AC092718.4	2.501846129	0.616026628	1.902762728	0.22258881	0.746249261	0.740101998
4140	AC092155.2	2.206222282	-0.249682864	2.055925047	-0.121445509	0.902228172	0.748558974
4141	AC096775.1	2.980210554	1.647685792	1.795288117	0.917722271	0.258758996	0.500895814
4142	AC100820.2	2.501846129	0.616026628	1.902762728	0.22258881	0.746249261	1.49711915
4143	AC104222.1	4.958820551	0.222984146	1.285789265	0.259750299	0.795056291	0.502721142
4144	AC104958.1	2.741052211	0.201405622	1.712226065	0.175918421	0.860258025	0.502721142
4145	AC104964.1	2.71847066	-0.758218689	1.722181758	-0.440224422	0.659702154	1.717970254
4146	AC104984.4	2.006519809	1.619121692	2.122725202	0.759179706	0.447745064	1.482091478
4147	AC105020.2	2.966122821	-0.95219511	1.667282514	-0.571671185	0.567544759	0.721447019
4148	AC106782.5	2.726926629	-0.762941977	1.729012221	-0.429296217	0.660446915	0.748558974
4149	AC106791.1	2.22214424	-0.266129216	1.877260792	-0.141764701	0.887265867	0.748558974
4150	AC107884.1	5.707490725	0.420796602	1.221920467	0.244270224	0.720567796	0.741928245
4151	AC109460.2	1.99806282	1.624249789	2.041644261	0.800457621	0.422445708	0.491429828
4152	AC110769.2	2.956826522	-0.216262148	1.406722098	-0.224820452	0.822118954	1.991595522
4153	AC112208.5	6.180224295	-0.204067212	1.159549817	-0.262228675	0.792145148	1.494282804
4154	AC117282.1	2.22214424	-0.266129216	1.877260792	-0.141764701	0.887265867	0.740101998
4155	AC122718.1	2.481257507	0.456862098	1.512514199	0.201855829	0.762761962	0.491429828

4156	AC120224.2	2.222202021	0.270786587	1.549625272	0.174742259	0.861281275	1.941504841
4157	AC120689.1	2.441762899	-0.25617978	1.529227984	-0.222899528	0.815829418	0.97921018
4158	AC121212.2	2.22214424	-0.266129216	1.877260792	-0.141764701	0.887265867	0.491429828
4159	AC122825.2	2.225979687	0.281284912	1.876254126	0.202205198	0.82897465	0.721447019
4160	AC126475.5	1.995228484	0.781822892	1.979482707	0.294968087	0.69286644	1.118517241
4161	AC127620.1	6.14626042	-1.028265425	1.160020619	-0.895126705	0.27071926	0.500895814
4162	AC127922.2	2.248562227	1.855749182	1.960444576	0.946596097	0.242844624	1.119808484
4163	AC128150.2	2.675299287	-1.402282282	1.524612225	-0.912769781	0.260827828	0.491429828
4164	AC128409.2	2.971804514	0.049222696	1.607249499	0.02062414	0.97556929	0.981145514
4165	AC147067.1	2.481257507	0.456862098	1.512514199	0.201855829	0.762761962	1.14955599
4166	AC224582.1	7.629604158	-0.41896751	1.067911641	-0.292224124	0.694818724	0.741928245
4167	AC242585.2	2.008512827	2.255670282	1.859925642	1.266540086	0.205219787	0.751294212
4168	AC245100.2	2.484924172	0.624206421	1.762112529	0.259708221	0.719065244	0.748558974
4169	AC245297.4	2.4708075	-0.522047427	1.789818772	-0.297822011	0.765829014	1.471851115
4170	ACBD2-AS1	2.960512189	-0.422291569	1.621811681	-0.26661022	0.789769286	1.48214154
4171	ACP6	4.422465602	0.05777281	1.274722757	0.042025454	0.966478407	0.748558974
4172	ACRBP	9.626277275	-0.961675112	0.990715749	-0.970687216	0.22170406	0.491429828
4173	ACVR2A	7.622922464	-0.812741257	1.056928981	-0.769902818	0.441256949	1.94994181
4174	ACVRL1	2.455829509	-0.799929522	1.518778902	-0.526692549	0.59840711	1.947177525
4175	ACY1	2.459516175	-1.165086955	1.924600112	-0.605265722	0.544926015	0.491429828
4176	ADCY10P1	2.426092206	-1.26624297	1.570296752	-0.80627177	0.420028519	0.500895814
4177	ADCY9	2.729761986	-0.218972027	1.692590719	-0.129270921	0.897064148	0.741928245
4178	ADD2-AS1	4.446542212	-0.274116249	1.221902675	-0.205808018	0.826940878	1.94994181
4179	AF111169.2	2.482098825	0.046704599	1.760714954	0.026525929	0.978827852	0.500895814
4180	AFAP1L2	2.695046691	-0.949782279	1.475966652	-0.642498525	0.519900665	0.994111124
4181	AHNAK2	2.46424555	0.468698626	1.514924016	0.209285512	0.75702829	

	0.999057482				
4182	AL020996.1	6.16894207	-0.529268016	1.154968457	-0.466911467
	0.640562221	0.981145514			
4183	AL021707.1	2.227271012	1.054642522	1.88562422	0.559204406
	0.575952989	0.751294212			
4184	AL021666.1	2.4708075	-0.522047427	1.789818772	-0.297822011
	0.765829014	0.992424851			
4185	AL021727.1	2.712850029	-0.206404568	1.694962724	-0.121775284
	0.902076989	0.741928245			
4186	AL025658.1	2.726976701	0.864162627	1.719715469	0.502502264
	0.615212557	0.748558974			
4187	AL050209.1	4.449227497	-0.957425907	1.261861561	-0.702027227
	0.482028712	0.502721142			
4188	AL078612.2	5.901522606	-0.685619207	1.180449582	-0.580812021
	0.561267152	0.502721142			
4189	AL109755.1	5.181125776	0.19149146	1.272998422	0.150207455
	0.880522052	0.500895814			
4190	AL118496.2	2.487719457	-0.546056664	1.829461879	-0.296856744
	0.766575881	1.449015848			
4191	AL121602.2	2.4708075	-0.522047427	1.789818772	-0.297822011
	0.765829014	1.110041282			
4192	AL121895.1	4.179081686	-1.650559126	1.456551282	-1.122196719
	0.257121652	0.751294212			
4193	AL122220.1	2.220208992	-0.945466096	2.005662065	-0.471298269
	0.627256252	0.740101998			
4194	AL122242.2	4.418288868	-1.755950797	1.42645024	-1.220992276
	0.218225242	0.491429828			
4195	AL126295.2	2.701558704	-0.747007668	1.742599827	-0.428674244
	0.668160207	0.491429828			
4196	AL126295.6	2.942759912	0.057127542	1.426981924	0.029762186
	0.968282722	1.48214154			
4197	AL127002.1	2.715685275	0.222162821	1.717120624	0.188199904
	0.850719948	0.751294212			
4198	AL127127.1	2.707179225	-1.260829905	1.85020686	-0.725506896
	0.462020842	0.500895814			
4199	AL127779.1	2.225979687	0.281284912	1.876254126	0.202205198
	0.82897465	1.984089491			
4200	AL127779.2	2.492290151	0.625561675	1.784827412	0.250488608
	0.725972028	0.500895814			
4201	AL128787.2	4.426794908	-0.598226176	1.245191225	-0.444788924
	0.656472292	0.741928245			
4202	AL157828.1	2.966182882	0.544458975	1.654017946	0.229172559
	0.742024507	1.11497224			
4203	AL157871.2	4.229917681	0.552726159	1.402206027	0.294622125
	0.692121775	0.751294212			
4204	AL158162.1	4.212955662	-0.472898996	1.287946752	-0.241428888
	0.722772204	0.740101998			
4205	AL161421.1	2.476478194	0.642870865	1.788076085	0.259522164
	0.71919702	0.999057482			
4206	AL252708.2	2.002684462	0.769829267	2.101449998	0.266222469
	0.714117004	0.500895814			

4207	AL256481.2	2.24572699	1.042882529	1.882412275	0.554251208	0.579406927	0.491429828
4208	AL256488.2	6.647407495	-0.052590647	1.152421228	-0.045594957	0.962622089	0.491429828
4209	AL258852.1	5.707490725	0.420796602	1.221920467	0.244270224	0.720567796	1.128144445
4210	AL259076.1	2.487719457	-0.546056664	1.829461879	-0.296856744	0.766575881	0.97921018
4211	AL290879.1	8.1164745	-0.571226124	1.028772871	-0.550001015	0.582218677	0.502721142
4212	AL291001.1	2.469966181	0.045772178	1.496504999	0.020586051	0.975599667	0.75411947
4213	AL291069.2	4.682914047	-0.729161072	1.205408621	-0.558569214	0.576455756	0.740101998
4214	AL441992.2	2.250556265	2.505801582	1.759282209	1.424220982	0.154250682	0.500895814
4215	AL450206.1	2.962507217	0.412267225	1.417282829	0.290956069	0.771084918	1.142925258
4216	AL512190.1	2.70250267	-0.952726729	1.480100595	-0.644272918	0.519222642	0.999057482
4217	AL512522.2	2.206222282	-0.249682864	2.055925047	-0.121445509	0.902228172	0.751294212
4218	AL512522.6	2.729761986	-0.218972027	1.692590719	-0.129270921	0.897064148	1.141100011
4219	AL589990.1	4.422959562	-0.947500696	1.251804211	-0.700915575	0.482255704	1.004718177
4220	AL590666.2	2.672514102	-0.520202222	1.544190426	-0.226877625	0.726209145	0.502721142
4221	AL592425.1	2.240056297	-0.281269262	1.908814517	-0.14740524	0.882812169	1.00189182
4222	AL602829.1	4.69415521	-1.444026422	1.271172255	-1.052122761	0.29228015	0.502721142
4223	AL627171.2	1.995228484	0.781822892	1.979482707	0.294968087	0.69286644	1.118517241
4224	AL645922.2	2.227271012	1.054642522	1.88562422	0.559204406	0.575952989	0.748558974
4225	AL662907.2	2.222202021	0.270786587	1.549625272	0.174742259	0.861281275	1.142885197
4226	AL928654.4	2.245676929	-0.966211587	2.218629501	-0.425497224	0.662201474	0.502721142
4227	ALG9	2.46424555	0.468698626	1.514924016	0.209285512	0.75702829	0.491429828
4228	ANKRD55	6.254528972	-1.681605984	1.185180259	-1.418860827	0.155929595	0.992424851
4229	AP000786.1	2.22214424	-0.266129216	1.877260792	-0.141764701	0.887265867	1.151291227
4230	AP001011.2	2.701558704	-0.747007668	1.742599827	-0.428674244	0.668160207	1.14955599
4231	AP001022.4	2.742888658	0.847215964	1.716922249	0.492508224	0.62165246	0.502721142
4232	AP001272.1	2.948220482	-1.540252415	1.501225402	-1.025929097		

						0.204925006	0.999057482
4233	AP001610.2	2.95205721	-0.426847199	1.640985194	-0.260116422	0.79477297	0.491429828
4234	AP001992.1	4.181917022	-1.212864192	1.404949472	-0.862279582	0.287982767	1.485917815
4235	AP002026.1	2.726926629	-0.762941977	1.729012221	-0.429296217	0.660446915	0.999057482
4236	AP002761.4	2.962248526	0.055271225	1.619729979	0.024185217	0.972729275	0.748558974
4237	AP002108.4	5.195152224	-0.960128119	1.278225584	-0.751125496	0.452571115	0.500895814
4238	AP005121.1	2.682755266	-1.406012256	1.525846411	-0.921464526	0.256807948	0.751294212
4239	AP005229.2	2.242050225	0.715260164	1.599951205	0.44711271	0.65479296	0.75411947
4240	AP1AR	2.24572699	1.042882529	1.882412275	0.554251208	0.579406927	0.981145514
4241	APOC1	2.968969167	-0.427855629	1.620851052	-0.270129242	0.787052055	0.502721142
4242	AQP2	2.666842409	-1.298482822	1.552298094	-0.90091126	0.267625504	0.502721142
4243	ARFGEF2	2.921418526	-1.522451262	1.488577987	-1.020145062	0.202941915	0.740101998
4244	ARHGAP24	6.129498524	-0.287056042	1.228980166	-0.221687259	0.81678085	0.500895814
4245	ARHGEF26	4.412668226	-1.222691159	1.281852859	-0.957910252	0.228107966	0.740101998
4246	ARMCX1	2.202555717	-0.16507171	1.570212122	-0.105120218	0.916280227	0.491429828
4247	B2GALNT2	4.477520779	-0.620212742	1.442094442	-0.420077756	0.66712908	0.491429828
4248	BIRC7	2.4708075	-0.522047427	1.789818772	-0.297822011	0.765829014	0.981145514
4249	BMF-AS1	2.492290151	0.625561675	1.784827412	0.250488608	0.725972028	0.741928245
4250	BX284668.4	4.952159858	-0.247400647	1.280519651	-0.192202219	0.84679974	0.97921018
4251	BZW1P2	2.712850029	-0.206404568	1.694962724	-0.121775284	0.902076989	0.500895814
4252	C18orf54	7.250852207	-1.407729159	1.085105679	-1.297229086	0.194517981	0.500895814
4253	C1QTNF6	2.225979687	0.281284912	1.876254126	0.202205198	0.82897465	0.75411947
4254	CACNA2D1-AS1	2.982095829	0.520226222	1.61815282	0.227725629	0.742111562	0.740101998
4255	CACYBPP2	2.224425665	0.272225827	1.850521221	0.201204828	0.840528408	1.482091478
4256	CAPRIN2	10.07802248	-1.227090522	0.944266588	-1.29951705	0.192766546	1.11497224
4257	CATSPER2	2.951215892	0.052409125	1.412845022	0.027068514	0.970420277	1.501889844

4258	CBWD6	2.749559251	2.217097689	1.842176821	1.202520565	0.228774928	1.141100011
4259	CCDC112	7.67188405	-0.42000122	1.077659688	-0.299014016	0.689882877	0.987744158
4260	CCDC128	4.699876066	0.200908002	1.209244812	0.152452251	0.878040775	1.48214154
4261	CCDC17	2.225979687	0.281284912	1.876254126	0.202205198	0.82897465	1.14955599
4262	CCDC170	2.459516175	-1.165086955	1.924600112	-0.605265722	0.544926015	0.500895814
4263	CCDC58	6.610698172	-0.942562824	1.149881422	-0.820574014	0.411888954	1.4744245
4264	CCDC71	4.460618822	-0.612562845	1.26180942	-0.449816128	0.652842027	1.971798147
4265	CCDC9B	4.192208258	-0.827052502	1.280460211	-0.599114225	0.549096642	1.729141479
4266	CCR5AS	2.226429692	1.21711458	1.595627804	0.762781005	0.445594011	0.502721142
4267	CD160	2.985881124	-0.44902712	1.681845018	-0.26699079	0.789476252	0.500895814
4268	CD2BP2-DT	2.742047229	1.026670216	1.521049068	0.681549556	0.495522821	1.741044944
4269	CD79B	4.404212258	-1.220525468	1.295117577	-0.946522461	0.242876564	0.740101998
4270	CDC25C	6.142575125	-0.521411715	1.170284425	-0.454087656	0.649765728	0.502721142
4271	CDT1	5.420222958	0.055168621	1.25261008	0.044007799	0.964898187	1.457714542
4272	CEBPB	4.654710765	-1.058554067	1.229916506	-0.790014947	0.429519029	1.119808484
4273	CEP120	2.954001177	-0.691659647	1.41922052	-0.487251781	0.626009059	1.941504841
4274	CFAP100	2.221600219	-0.272712596	1.875455829	-0.14594457	0.882965147	1.744921271
4275	CLBA1	8.611750758	-1.277180025	1.009006657	-1.265779582	0.205592007	0.990401505
4276	CLDN12	2.960512189	-0.422291569	1.621811681	-0.26661022	0.789769286	0.491429828
4277	CLEC11A	6.152021112	-0.524048007	1.160012457	-0.460280872	0.645242862	1.941504841
4278	CLEC1A	2.426142267	0.068160175	1.678651056	0.040604125	0.967611487	0.992424851
4279	CLEC2D	4.446542212	-0.274116249	1.221902675	-0.205808018	0.826940878	0.751294212
4280	CNBD2	2.47842216	0.040446546	1.514009265	0.02671486	0.978687161	1.128144445
4281	CNNM2	5.172619726	-0.651076225	1.246462689	-0.522229209	0.601424174	0.992424851
4282	COL17A1	8.842501962	-1.221294767	0.99556205	-1.227229225	0.181147792	0.500895814
4283	COL4A2-AS2	2.259852662	2.041472056	2.25009556	1.251708294		

0.176468619 0.999057482
4284 COL6A1 2.490604866 2.061571459 1.975765776 1.042429076
0.296749612 1.444180511
4285 COX20P1 1.995228484 0.781822892 1.979482707 0.294968087
0.69286644 0.741928245
4286 CTF1 2.259852662 2.041472056 2.25009556 1.251708294
0.176468619 0.500895814
4287 CTSK 1.99806282 1.624249789 2.041644261 0.800457621
0.422445708 0.740101998
4288 CXCR5 2.221600219 -0.272712596 1.875455829 -0.14594457
0.882965147 1.48214154
4289 DCAF15 2.489712485 0.450700695 1.552212458 0.290259997
0.771540844 1.477471847
4290 DIAPH2-AS1 2.458674856 -0.265825902 1.498227821 -0.244170784
0.807098554 1.00189182
4291 DIRC2 2.985881124 -0.44902712 1.681845018 -0.26699079
0.789476252 0.748558974
4292 DNAJB2 6.194211006 -0.547268122 1.186226606 -0.461209212
0.644576705 0.500895814
4293 DNAJC25 2.484092852 0.891848676 1.52597156 0.58444646
0.558919977 0.740101998
4294 DPY19L2 2.954842495 -1.522228192 1.789414169 -0.856822486
0.291527469 0.500895814
4295 DSTNP2 2.464295488 -0.804299921 1.529671902 -0.525798977
0.599027885 1.488742171
4296 DUSP8P5 2.224425665 0.272225827 1.850521221 0.201204828
0.840528408 0.751294212
4297 DYRK1B 6.878108627 -0.79650962 1.107985785 -0.718880721
0.472214416 0.491429828
4298 E2F7 2.009255155 2.84902704 2.224581892 1.225608277
0.220246084 1.449015848
4299 E2F8 2.700667222 -1.412295887 1.522841225 -0.922075797 0.25648904
0.502721142
4300 ECM2 4.688584741 -0.100926201 1.206602851 -0.077242212
0.928420066 0.502721142
4301 EEF1A1P11 4.947489164 -0.852274028 1.297629659 -0.65679297
0.511214045 1.00189182
4302 EEF1A1P9 2.71200871 0.242996044 1.452582054 0.167170504
0.867225892 0.491429828
4303 EEF1B2P6 2.92708922 -0.682859216 1.420128959 -0.481542944
0.620129955 0.741928245
4304 EHMT2-AS1 2.966182882 0.544458975 1.654017946 0.229172559
0.742024507 1.981204107
4305 EMC2-AS1 2.979419174 0.402070285 1.424180225 0.280248575
0.779210087 1.00189182
4306 ENC1 2.977475208 1.05592224 1.664202485 0.62445955
0.525780957 0.502721142
4307 EPS8L1 2.452004162 -1.27422956 1.568912149 -0.812227678
0.416655264 0.75411947
4308 ERN2 2.227271012 1.054642522 1.88562422 0.559204406
0.575952989 0.740101998

4309	ESYT2	2.71847066	-0.758218689	1.722181758	-0.440224422	0.659702154	0.502721142
4310	EXOC2-AS1	2.229214978	0.258520209	1.599260214	0.161629817	0.8715895	1.714479018
4311	FAAH	2.479262479	-0.529491656	1.799724147	-0.29976186	0.764258809	0.491429828
4312	FAM117B	2.928582179	-2.062584505	1.60674062	-1.28422958	0.199026661	0.748558974
4313	FAM122C	4.45499819	-0.277926827	1.226265599	-0.207972157	0.825250707	0.97921018
4314	FAM20A	2.714792995	-0.542204022	1.466295959	-0.269778024	0.711547891	0.491429828
4315	FAM210B	4.665952028	-1.856911865	1.299814444	-1.226541428	0.184660419	1.480207192
4316	FAM24B	2.985921186	1.047719872	1.641022206	0.628451215	0.522179926	0.990401505
4317	FAM52B-AS1	2.490554804	0.029250627	1.794056424	0.021878151	0.982545154	1.984914829
4318	FANCB	2.450218878	-0.261052188	1.505847494	-0.229767422	0.810510555	0.75411947
4319	FBXL12	2.465186869	0.061565658	1.798591226	0.024229921	0.972692799	0.751294212
4320	FBXL14	4.19225842	0.226422606	1.402624762	0.161424475	0.871751216	1.485917815
4321	FBXO44	2.455889571	0.474841748	1.555182829	0.205228567	0.760115921	0.999057482
4322	FCRL6	2.259852662	2.041472056	2.25009556	1.251708294	0.176468619	1.750552004
4323	FCRLA	2.009255155	2.84902704	2.224581892	1.225608277	0.220246084	1.142925258
4324	FGD6	2.462251522	-0.526556268	1.802629589	-0.292102909	0.770207942	0.741928245
4325	FNDC9	2.194941058	-0.922612201	2.222160225	-0.412590252	0.679174212	1.119808484
4326	FOXP2	2.974629861	0.527202524	1.61920087	0.221822521	0.740015718	1.118517241
4327	FP565260.1	6.405264967	-0.170566608	1.152581802	-0.147986552	0.88225228	1.128144445
4328	FRAT2	2.906050591	-1.522042282	1.522089259	-0.999969879	0.217225085	1.125419218
4329	FREM1	2.504681476	1.254255222	1.91212647	0.655601601	0.512080495	0.748558974
4330	FRY-AS1	5.926900542	-0.692504005	1.180220774	-0.587605219	0.55679724	0.992424851
4331	FSIP1	4.722458716	0.821287452	1.225922596	0.614766672	0.528708822	0.748558974
4332	FSIP2	4.910829904	-1.170227705	1.2957955	-0.902180868	0.26642991	1.477471847
4333	FST	4.952109796	-1.184821214	1.229754901	-0.884264222	0.276499625	1.14955599
4334	FUT8-AS1	4.190272012	-1.216202824	1.407968012	-0.862800757		

0.287697252 1.485917815
4335 GAPDHP60 2.710014682 -0.75270841 1.722261795 -0.426767492
0.662279977 1.981204107
4336 GAS2 6.461821592 0.96071449 1.190982472 0.806657118
0.419864056 1.494921715
4337 GCNT7 4.201714299 0.221762526 1.279752722 0.160726898
0.872208502 1.750552004
4338 GCSAM 2.47842216 0.040446546 1.514009265 0.02671486
0.978687161 0.990401505
4339 GFRA1 2.922209907 -0.922819242 1.741270427 -0.525711921
0.592157656 0.970854101
4340 GIMAP7 2.46424555 0.468698626 1.514924016 0.209285512 0.75702829
0.75411947
4341 GIMAP8 5.414662264 -0.47866102 1.226422704 -0.290290092
0.696222049 1.488742171
4342 GLS2 6.166157785 -0.064781466 1.168762646 -0.055427292
0.955797972 1.991595522
4343 GPAT2 2.725422679 0.855729028 1.695610407 0.504679027
0.612784287 1.95588411
4344 GPM6B 2.726976701 0.864162627 1.719715469 0.502502264
0.615212557 0.491429828
4345 GPR160 6.152021112 -0.524048007 1.160012457 -0.460280872
0.645242862 1.984089491
4346 GPRC5D-AS1 4.92774186 -1.176059274 1.295229762 -0.907915675
0.262922776 0.987744158
4347 GRAMD4 7.129898469 -0.45264852 1.092117026 -0.414468888
0.678520747 1.978448841
4348 GREB1L 2.461460141 -1.278278151 1.582825524 -0.807650657
0.419291716 0.500895814
4349 H2BC5 2.726976701 0.864162627 1.719715469 0.502502264
0.615212557 0.748558974
4350 H2-5 2.462251522 -0.526556268 1.802629589 -0.292102909
0.770207942 0.500895814
4351 H2C11 7.6492014 -0.819760565 1.061750247 -0.772084176
0.440064574 1.111251708
4352 H2P6 7.68596066 -0.628425249 1.112098077 -0.564572205
0.572264095 0.748558974
4353 HAMP 4.469124862 0.264225751 1.250112022 0.269781674
0.787228222 0.75411947
4354 HECTD2 4.18475228 -0.822482292 1.280845559 -0.596261691
0.550922627 0.75411947
4355 HGD 2.24572699 1.042882529 1.882412275 0.554251208
0.579406927 0.502721142
4356 HHIP 2.942709851 -1.089119978 1.429218602 -0.756742955
0.449202276 0.75411947
4357 HIPK1-AS1 2.240106259 1.870206598 2.048865508 0.912801056
0.261247182 1.471851115
4358 HIRA 6.664269291 -0.721997525 1.121894584 -0.642552028
0.519865967 0.491429828
4359 HLA-K 4.952209919 0.625564272 1.202652972 0.487525722
0.625885811 0.500895814

4360	HLTF-AS1	5.667996118	-0.07260072	1.21581075	-0.059712822	0.952282554	0.751294212
4361	HMGB1P6	2.942709851	-1.089119978	1.429218602	-0.756742955	0.449202276	1.711058297
4362	HNRNPA1P24	4.201664227	-0.820628241	1.288494522	-0.598229465	0.549686824	0.748558974
4363	HOMER1	2.476478194	0.642870865	1.788076085	0.259522164	0.71919702	1.111251708
4364	HOMER2	4.92774186	-1.176059274	1.295229762	-0.907915675	0.262922776	0.502721142
4365	HOXB4	2.002684462	0.769829267	2.101449998	0.266222469	0.714117004	1.110041282
4366	HPSE	5.892026566	-1.525777222	1.206287002	-1.272144144	0.202966897	0.502721142
4367	HROB	5.200822018	-0.274152682	1.258274645	-0.297220912	0.766212882	0.990401505
4368	HSP90AB2P	2.225088206	-0.627562912	1.592917404	-0.40024857	0.688972444	0.491429828
4369	IDI2	2.709222425	1.064412041	1.625952275	0.654629781	0.512699705	0.500895814
4370	IDUA	2.971804514	0.049222696	1.607249499	0.02062414	0.97556929	0.741928245
4371	IFFO2	2.492290151	0.625561675	1.784827412	0.250488608	0.725972028	0.75411947
4372	IFI44L	2.697882028	-0.52261222	1.455756722	-0.26655284	0.712951845	1.11497224
4373	IFT81	4.429620255	-0.266449695	1.252655252	-0.196982694	0.842841081	1.712842481
4374	IGLV2-19	2.254182969	1.022227219	1.995522668	0.517276669	0.604962028	0.721447019
4375	IMPA2	2.966122821	-0.95219511	1.667282514	-0.571671185	0.567544759	1.501889844
4376	INPP5E	7.401688201	-0.144271804	1.082157827	-0.122287874	0.892965717	1.724414221
4377	INTU	2.962248526	0.055271225	1.619729979	0.024185217	0.972729275	1.719514275
4378	ITGA10	2.002684462	0.769829267	2.101449998	0.266222469	0.714117004	0.970854101
4379	ITGA2	2.678124724	-0.941788888	1.496690062	-0.629247772	0.529186858	1.477471847
4380	ITGA7	2.706228016	-0.527895892	1.45521052	-0.269624415	0.711654905	1.141100011
4381	ITGAD	2.225088206	-0.627562912	1.592917404	-0.40024857	0.688972444	0.502721142
4382	ITGB2-AS1	7.151189794	-0.251028452	1.094181749	-0.229420205	0.818524484	0.500895814
4383	ITPKC	5.656654721	-1.144162922	1.208244055	-0.94696242	0.242657419	1.488742171
4384	JTBP1	5.94286256	0.042114062	1.187240567	0.026211455	0.971024016	0.748558974
4385	JUND	2.461510202	0.051021649	1.497172067	0.024085227		

0.972809101 0.741928245
4386 KCNIP2 2.715625214 -1.266001666 1.855760255 -0.72608726
0.461677525 1.118517241
4387 KCNN2 2.997222511 1.627226597 1.699876226 0.957267695
0.228422156 1.44055989
4388 KCNQ5 2.451060197 -1.159106294 1.944926274 -0.595964181
0.551199156 0.987744158
4389 KHK 2.742888658 0.847215964 1.716922249 0.492508224 0.62165246
0.491429828
4390 KIAA0825 4.227082224 0.207914621 1.401024266 0.148400808
0.882026462 0.500895814
4391 KIAA1614 2.496225497 1.265578589 1.809272207 0.699495955
0.48424214 1.981154144
4392 KIF14 2.968969167 -0.427855629 1.620851052 -0.270129242
0.787052055 1.00189182
4393 KIF2C 5.297700245 -1.262222782 1.2486129 -1.091869852
0.274890211 1.141100011
4394 KRT8P29 2.214688262 -0.258242727 1.914245251 -0.124951474
0.892650241 1.142925258
4395 KYAT1 7.806724082 -1.294269589 1.102528925 -1.174000562 0.2402948
0.502721142
4396 LHFPL4 6.894970522 -1.541210251 1.12922565 -1.264792762 0.17221788
1.981204107
4397 LILRB5 2.216622228 -0.622649111 1.571117189 -0.40267468
0.687187562 0.75411947
4398 LINC00278 4.187587727 -0.46229408 1.272771842 -0.226514452
0.726482955 1.00189182
4399 LINC00470 2.420471574 -0.786659401 1.558158729 -0.50486474
0.612652841 0.751294212
4400 LINC00622 2.228815024 1.066246854 2.002812146 0.522424522
0.594421997 1.141100011
4401 LINC00852 2.709172262 -0.144224899 1.447244977 -0.099654817
0.920618272 0.500895814
4402 LINC00884 2.746724004 1.457707221 1.749470628 0.822227605
0.404716277 0.970854101
4403 LINC00892 2.459516175 -1.165086955 1.924600112 -0.605265722
0.544926015 0.491429828
4404 LINC00997 2.228815024 1.066246854 2.002812146 0.522424522
0.594421997 0.502721142
4405 LINC01128 7.922282557 -0.222212061 1.067612225 -0.202827509
0.762012696 0.502721142
4406 LINC01222 2.492290151 0.625561675 1.784827412 0.250488608
0.725972028 0.741928245
4407 LINC01547 2.202555717 -0.16507171 1.570212122 -0.105120218
0.916280227 0.740101998
4408 LINC02068 2.465186869 0.061565658 1.798591226 0.024229921
0.972692799 0.502721142
4409 LINC02228 2.482098825 0.046704599 1.760714954 0.026525929
0.978827852 1.49711915
4410 LINC02610 4.167840422 -0.816220252 1.407664921 -0.579846979
0.562017812 0.502721142

4411	LINC02862	6.644522087	-0.952524291	1.119058565	-0.85119252	0.294662412	0.981145514
4412	LNP1	2.985921186	1.047719872	1.641022206	0.628451215	0.522179926	1.722590985
4413	LONRF1	9.282042645	-0.260427249	0.990691542	-0.262884297	0.79262976	0.721447019
4414	LRRC27A2	5.662225424	-0.587248918	1.198974947	-0.489792484	0.62428075	0.500895814
4415	LSM14B	4.677292415	-0.406245628	1.210062529	-0.210172458	0.756429814	0.502721142
4416	LYG2	2.745882686	1.498077746	1.542120219	0.970810719	0.221642544	0.740101998
4417	MAG	8.110802806	-0.951271207	1.020097584	-0.922572865	0.255708226	0.500895814
4418	MAMDC4	2.971804514	0.049222696	1.607249499	0.02062414	0.97556929	0.721447019
4419	MAP11	5.181125776	0.19149146	1.272998422	0.150207455	0.880522052	1.14955599
4420	MAP2K7CL	2.452004162	-1.27422956	1.568912149	-0.812227678	0.416655264	0.500895814
4421	MASP2	4.415502582	-0.944162218	1.262185657	-0.692615282	0.488551018	0.500895814
4422	MED25	2.250556265	2.505801582	1.759282209	1.424220982	0.154250682	0.491429828
4423	MFSD14C	4.702711412	0.510285484	1.225656044	0.285005964	0.700222997	0.721447019
4424	MICU2	2.925797894	-1.081846497	1.448500692	-0.746872202	0.455140042	0.491429828
4425	MID1IP1	2.957677842	-0.948226275	1.661565119	-0.570688662	0.568210706	0.987744158
4426	MIR2671	2.749559251	2.217097689	1.842176821	1.202520565	0.228774928	0.992424851
4427	MIR2945HG	2.484092852	0.891848676	1.52597156	0.58444646	0.558919977	1.952050842
4428	MIR6126	2.224425665	0.272225827	1.850521221	0.201204828	0.840528408	0.97921018
4429	MRPL45P2	4.699876066	0.200908002	1.209244812	0.152452251	0.878040775	1.715125007
4430	MRTFA-AS1	2.692261406	-0.124742558	1.478198244	-0.09115291	0.927270297	0.990401505
4431	MSC-AS1	4.720914695	0.816260729	1.252727471	0.602414007	0.546222206	0.502721142
4432	MSR1	2.722249972	-0.546608824	1.491628259	-0.266448624	0.714020242	0.75411947
4433	MTURN	2.692261406	-0.124742558	1.478198244	-0.09115291	0.927270297	1.00189182
4434	MYBL2	2.678124724	-0.941788888	1.496690062	-0.629247772	0.529186858	0.740101998
4435	MYH7B	5.217785027	0.455152296	1.276449116	0.256577705	0.721407971	0.500895814
4436	MYO7A	2.977425146	-0.442256216	1.628055075	-0.27066014		

						0.786652426	1.494282804
4437	MZT2A	7.121292429	-1.10450128	1.087422225	-1.01569665	0.209772864	0.491429828
4438	N4BP2L2-IT2	6.681221409	-0.062614259	1.120406202	-0.056275662	0.955122206	0.502721142
4439	NBPF15	4.460618822	-0.612562845	1.26180942	-0.449816128	0.652842027	0.500895814
4440	NCMAP-DT	2.72608522	-0.152721742	1.475920592	-0.104152126	0.917047822	1.71119944
4441	NME8	2.962248526	0.055271225	1.619729979	0.024185217	0.972729275	0.502721142
4442	NMRAL2P	2.240106259	1.870206598	2.048865508	0.912801056	0.261247182	0.502721142
4443	NOXRED1	2.455829509	-0.799929522	1.518778902	-0.526692549	0.59840711	0.740101998
4444	NRBP2	2.248562227	1.855749182	1.960444576	0.946596097	0.242844624	0.491429828
4445	NSMF	5.454156872	0.041401996	1.225222419	0.022515124	0.972262798	0.990401505
4446	OAS2	5.904267952	-0.422882292	1.186249486	-0.264916728	0.71517256	0.500895814
4447	OR2AT1P	2.710014682	-0.75270841	1.722261795	-0.426767492	0.662279977	0.751294212
4448	OR52K2P	4.452162844	-0.608951942	1.244220204	-0.452011616	0.650540277	0.75411947
4449	OSBPL10	2.222202021	0.270786587	1.549625272	0.174742259	0.861281275	1.00189182
4450	OSCP1	2.476478194	0.642870865	1.788076085	0.259522164	0.71919702	1.141100011
4451	P2H4	8.126221802	-0.292615694	1.027078721	-0.279542725	0.704284876	0.970854101
4452	PAM16	5.200822018	-0.274152682	1.258274645	-0.297220912	0.766212882	0.981145514
4453	PAN2-AS1	2.728217964	-0.225409578	1.722272066	-0.120116072	0.896474595	0.502721142
4454	PANK1	2.966122821	-0.95219511	1.667282514	-0.571671185	0.567544759	1.004718177
4455	PANK2-AS1	5.682022666	-1.151740206	1.228149787	-0.927784804	0.248255012	0.740101998
4456	PBX2	4.196042705	-0.466145002	1.268627249	-0.240592104	0.722409921	0.502721142
4457	PCCA-DT	2.982095829	0.520226222	1.61815282	0.227725629	0.742111562	0.740101998
4458	PCYT1B	2.710014682	-0.75270841	1.722261795	-0.426767492	0.662279977	0.75411947
4459	PDCD4-AS1	2.706287955	-1.956862222	1.675901825	-1.167647885	0.242948826	0.992424851
4460	PDE7B	2.707179225	-1.260829905	1.85020686	-0.725506896	0.462020842	0.75411947
4461	PEX11A	6.911982612	-0.159095999	1.109659255	-0.142272728	0.885995021	0.748558974

4462	PGAM5	9.588276851	-0.788027812	0.968726425	-0.81246786	0.415949866	0.502721142
4463	PGM5P2	4.922462616	0.246299988	1.222212175	0.26171161	0.792542789	0.990401505
4464	PGRMC2	7.874421971	-0.688520451	1.044629075	-0.659105195	0.50982822	0.97921018
4465	PHLDB1	2.945545198	-0.687762229	1.414699646	-0.486154245	0.626857809	0.970854101
4466	PICK1	2.227271012	1.054642522	1.88562422	0.559204406	0.575952989	1.119808484
4467	PKD1L2	2.468022215	0.65247149	1.911052447	0.241419802	0.72278757	0.741928245
4468	PLCD4	2.248562227	1.855749182	1.960444576	0.946596097	0.242844624	0.748558974
4469	PMEL	4.688584741	-0.100926201	1.206602851	-0.077242212	0.928420066	0.502721142
4470	POFUT2	2.714792995	-0.542204022	1.466295959	-0.269778024	0.711547891	0.981145514
4471	POGLUT2	2.467972154	-1.170962261	1.922951262	-0.608940187	0.542564086	0.987744158
4472	POLR2J2	2.222544285	-0.6426565	1.629225261	-0.292024985	0.695029752	1.717970254
4473	PPL	2.92708922	-0.682859216	1.420128959	-0.481542944	0.620129955	0.502721142
4474	PPP2CC	8.879211284	-0.607920206	1.000482962	-0.607626128	0.542425461	0.987744158
4475	PRAG1	7.652186808	-0.045058982	1.072222924	-0.041980296	0.966514226	1.00189182
4476	PROX2	2.71757928	-1.420892221	1.577899742	-0.900495946	0.267856282	0.502721142
4477	PRRT1	2.17712772	-1.674565288	1.756261206	-0.952428707	0.240272902	0.491429828
4478	PRUNE2	2.951215892	0.052409125	1.412845022	0.027068514	0.970420277	0.740101998
4479	PTENP1-AS	4.660221297	-1.422400402	1.249767002	-1.061220492	0.288589708	0.75411947
4480	PTGES	4.11705449	-2.149642552	1.710289266	-1.25688829	0.208794089	1.989740184
4481	PTMAP2	4.950224511	-0.542628255	1.285816826	-0.422788257	0.672449756	1.488742171
4482	PXDC1	1.99806282	1.624249789	2.041644261	0.800457621	0.422445708	0.491429828
4483	PXDN	2.980260492	0.042088274	1.617761627	0.026624562	0.978751206	0.491429828
4484	RAB22	2.248562227	1.855749182	1.960444576	0.946596097	0.242844624	0.500895814
4485	RAB6C-AS1	4.888247252	-1.942474187	1.282419115	-1.404822985	0.160070651	0.75411947
4486	RALGPS1	4.181917022	-1.212864192	1.404949472	-0.862279582	0.287982767	1.141100011
4487	RAP1GAP	2.000899177	2.860118729	2.227872825	1.22864046		

0.219206629 1.118517241
4488 RAPGEF5 2.960512189 -0.422291569 1.621811681 -0.26661022
0.789769286 1.449015848
4489 RARRES1 6.265820298 -1.286714289 1.162819114 -1.192545146
0.22204756 0.994111124
4490 RBAK 9.07046788 -1.006641071 0.996190788 -1.010490244
0.212260471 1.119808484
4491 RBPMS 2.492290151 0.625561675 1.784827412 0.250488608
0.725972028 1.141100011
4492 RCE1 20.9755119 -0.826182407 0.786957292 -1.062551054
0.287985594 0.491429828
4493 REEP6 2.715685275 0.222162821 1.717120624 0.188199904
0.850719948 1.714479018
4494 RHBDF1 2.92708922 -0.682859216 1.420128959 -0.481542944
0.620129955 1.141100011
4495 RHD 2.980260492 0.042088274 1.617761627 0.026624562
0.978751206 0.748558974
4496 RHEBP2 2.472642847 0.054029801 1.762188854 0.020666292
0.975525672 0.97921018
4497 RIMKLBP2 2.222202021 0.270786587 1.549625272 0.174742259
0.861281275 0.502721142
4498 RLIMP2 2.458674856 -0.265825902 1.498227821 -0.244170784
0.807098554 0.721447019
4499 RNASEH1-AS1 7.851849221 -1.086688225 1.047799228 -1.027114919
0.299682202 0.740101998
4500 RNF122 2.746724004 1.457707221 1.749470628 0.822227605
0.404716277 1.11497224
4501 RNF144A-AS1 4.215841071 0.926555176 1.412592227 0.655460916
0.512171042 0.721447019
4502 RNF227 6.14626042 -1.028265425 1.160020619 -0.895126705 0.27071926
1.128144445
4503 RNF42 4.671622722 -1.064751279 1.220107972 -0.806562782
0.419917847 1.482091478
4504 RNLS 2.250506202 0.707465902 1.709628212 0.412812722
0.679011266 0.740101998
4505 RNU4-40P 4.224246987 -0.122758071 1.405117187 -0.09448185
0.924726299 0.740101998
4506 ROPN1L 2.960512189 -0.422291569 1.621811681 -0.26661022
0.789769286 0.741928245
4507 RPL10P12 4.41822892 -0.59471277 1.262424246 -0.426188051
0.662700297 0.502721142
4508 RPL12P5 2.240106259 1.870206598 2.048865508 0.912801056
0.261247182 1.70811205
4509 RPL21P5 2.726125282 1.050065864 1.490982855 0.704277152
0.481260192 0.491429828
4510 RPL22AP24 2.479262479 -0.529491656 1.799724147 -0.29976186
0.764258809 1.482091478
4511 RPL22AP57 2.228764972 -0.952228689 2.015267459 -0.472482906
0.626581427 0.502721142
4512 RPL22AP65 2.222202021 0.270786587 1.549625272 0.174742259
0.861281275 0.502721142

4513	RPL24P4	2.720464688	0.227817257	1.452019777	0.162671108	0.869990062	0.970854101
4514	RPL29P11	2.24572699	1.042882529	1.882412275	0.554251208	0.579406927	0.721447019
4515	RPL22P26	4.952159858	-0.247400647	1.280519651	-0.192202219	0.84679974	0.741928245
4516	RPL26AP21	2.487719457	-0.546056664	1.829461879	-0.296856744	0.766575881	0.502721142
4517	RPL2P2	4.924956575	-0.522882922	1.275509769	-0.418565145	0.675522967	0.502721142
4518	RPL5P9	2.991551818	0.522174409	1.650780488	0.216925486	0.751200142	0.500895814
4519	RPL7AP66	2.962248526	0.055271225	1.619729979	0.024185217	0.972729275	0.491429828
4520	RPL7P1	2.219467674	-0.175850214	1.550428225	-0.112420472	0.90969721	1.480207192
4521	RPS12P27	2.4708075	-0.522047427	1.789818772	-0.297822011	0.765829014	0.502721142
4522	RPS21P4	4.212955662	-0.472898996	1.287946752	-0.241428888	0.722772204	1.952050842
4523	RPS27AP16	4.428026172	-1.222015919	1.280824441	-0.965276828	0.224256102	1.491598519
4524	RPS6KA4	2.447422592	0.481720978	1.668726862	0.288681742	0.772824926	1.118517241
4525	RPS6P25	2.498219525	1.916180268	1.621907441	1.181426264	0.227429459	0.502721142
4526	RPS7P15	2.722249972	-0.546608824	1.491628259	-0.266448624	0.714020242	0.491429828
4527	RRAS2	6.291188222	-1.292121058	1.152690402	-1.208590827	0.226820078	0.491429828
4528	RTN4R	2.954842495	-1.522228192	1.789414169	-0.856822486	0.291527469	1.485917815
4529	RUBCNL	2.972798542	0.782760509	1.421057266	0.547679247	0.582912096	1.004718177
4530	SAMD4A	2.490554804	0.029250627	1.794056424	0.021878151	0.982545154	0.740101998
4531	SAPCD1-AS1	2.46424555	0.468698626	1.514924016	0.209285512	0.75702829	0.502721142
4532	SCG2	5.696149228	-0.598527589	1.221729111	-0.48990622	0.624200262	1.444180511
4533	SCML1	8.904629281	-0.118258009	1.017981521	-0.11616911	0.907518517	0.992424851
4534	SDAD1P1	2.226279621	-0.186874112	1.611572844	-0.115957524	0.907686205	1.7208057
4535	SENCR	4.19225842	0.226422606	1.402624762	0.161424475	0.871751216	0.740101998
4536	SERHL2	7.424270952	0.228042204	1.094796276	0.217420592	0.827872792	0.502721142
4537	SGK2	4.415502582	-0.944162218	1.262185657	-0.692615282	0.488551018	1.14955599
4538	SLC12A9	2.188429045	-1.111492462	1.624176286	-0.684242212		

						0.492759097	0.500895814
4539	SLC22A15	2.478272098	-1.286727495	1.658824596	-0.775681612	0.427926999	1.715125007
4540	SLC22A22	2.499060844	2.047712186	1.895524482	1.080282212	0.280016528	0.75411947
4541	SLC22A4	6.926009161	-1.040212559	1.128691225	-0.912515827	0.260971208	1.119808484
4542	SLC24A2	2.000899177	2.860118729	2.227872825	1.22864046	0.219206629	0.994111124
4543	SLC25A22	5.420282896	-0.759478196	1.221555974	-0.621720164	0.524119214	1.118517241
4544	SLC25A29P1	5.414712226	0.224569275	1.220000458	0.24402704	0.807202122	1.711058297
4545	SLC26A4	1.995228484	0.781822892	1.979482707	0.294968087	0.69286644	1.705287702
4546	SMAD1	2.982095829	0.520226222	1.61815282	0.227725629	0.742111562	0.500895814
4547	SMG1P2	2.972798542	0.782760509	1.421057266	0.547679247	0.582912096	0.992424851
4548	SMYD2-IT1	2.95205721	-0.426847199	1.640985194	-0.260116422	0.79477297	0.741928245
4549	SND1-IT1	5.169824451	-0.081914429	1.274747501	-0.064259244	0.948762725	0.491429828
4550	SNORA72B	2.499060844	2.047712186	1.895524482	1.080282212	0.280016528	0.990401505
4551	SNORD17	2.221600219	-0.272712596	1.875455829	-0.14594457	0.882965147	1.144710442
4552	SOS1-IT1	2.251247622	0.252724822	1.99949604	0.176906988	0.85958145	0.491429828
4553	SPAG16	8.141842425	-0.577201502	1.024222876	-0.558142842	0.576746164	1.119808484
4554	SPATA22	4.442756927	0.277907008	1.27221864	0.27517795	0.782179527	0.502721142
4555	SPATC1L	2.461510202	0.051021649	1.497172067	0.024085227	0.972809101	0.491429828
4556	SPIN4-AS1	5.172619726	-0.651076225	1.246462689	-0.522229209	0.601424174	1.125419218
4557	SPOCD1	2.489712485	0.450700695	1.552212458	0.290259997	0.771540844	0.75411947
4558	STARD9	2.486878128	0.024886272	1.557410529	0.022400178	0.982128728	0.721447019
4559	STEAP2-AS1	2.422206921	-0.25107512	1.577245984	-0.222572219	0.822867604	0.721447019
4560	STRADA	4.218676417	1.218882876	1.462609429	0.901116685	0.267526282	0.97921018
4561	STX18-AS1	2.741052211	0.201405622	1.712226065	0.175918421	0.860258025	0.740101998
4562	STX1B	2.22214424	-0.266129216	1.877260792	-0.141764701	0.887265867	0.502721142
4563	SUMO1P2	5.670781402	-0.590040577	1.19612712	-0.492292522	0.621805904	1.719514275

4564	SYN2	2.492290151	0.625561675	1.784827412	0.250488608	0.725972028	1.722590985
4565	TAF4	2.92708922	-0.682859216	1.420128959	-0.481542944	0.620129955	0.491429828
4566	TBC1D16	2.4708075	-0.522047427	1.789818772	-0.297822011	0.765829014	1.155114484
4567	TBC1D19	5.178290429	-0.085426802	1.256716978	-0.067976166	0.945804607	0.491429828
4568	TBC1D8B	2.225979687	0.281284912	1.876254126	0.202205198	0.82897465	0.491429828
4569	TBX18	2.672464041	-1.942828017	1.648587076	-1.178480679	0.22860502	1.7208057
4570	TCFL5	2.980260492	0.042088274	1.617761627	0.026624562	0.978751206	0.491429828
4571	TEF	4.190272012	-1.216202824	1.407968012	-0.862800757	0.287697252	0.994111124
4572	TEX14	2.468022215	0.65247149	1.911052447	0.241419802	0.72278757	0.491429828
4573	TFAP2E	2.706228016	-0.527895892	1.45521052	-0.269624415	0.711654905	0.491429828
4574	THRA	2.4708075	-0.522047427	1.789818772	-0.297822011	0.765829014	1.754112498
4575	THSD1	2.248512275	-0.289622119	2.044555795	-0.141660658	0.887248052	0.97921018
4576	TIGD5	2.712850029	-0.206404568	1.694962724	-0.121775284	0.902076989	0.751294212
4577	TLR5	2.465186869	0.061565658	1.798591226	0.024229921	0.972692799	1.485917815
4578	TMEM169	2.221600219	-0.272712596	1.875455829	-0.14594457	0.882965147	0.748558974
4579	TMEM184A	4.952159858	-0.247400647	1.280519651	-0.192202219	0.84679974	0.502721142
4580	TMEM221	2.729761986	-0.218972027	1.692590719	-0.129270921	0.897064148	0.491429828
4581	TMEM86A	7.877217256	-1.092199521	1.042962221	-1.046205982	0.295465942	0.491429828
4582	TMEM8B	5.128795822	-0.628644882	1.220694759	-0.482567262	0.628692942	0.740101998
4583	TMEM91	2.692211244	-1.409706879	1.525259222	-0.924240849	0.255260922	0.502721142
4584	TMPO-AS1	6.641786864	0.166159212	1.206788997	0.12768712	0.890487691	0.75411947
4585	TNFRSF11A	2.987925214	1.621046094	1.49491522	1.091062525	0.275245271	0.75411947
4586	TNFRSF21	4.725294062	1.161222558	1.255217076	0.856925452	0.291480558	0.740101998
4587	TPTEP2	2.724141254	0.215829029	1.681481778	0.187822757	0.851006972	0.992424851
4588	TRIM2	6.429128819	-0.88086887	1.180207624	-0.746267717	0.455445215	1.144710442
4589	TRIM58	2.248512275	-0.289622119	2.044555795	-0.141660658		

0.887248052 0.990401505
4590 TRIM9 7.891292866 -1.212220248 1.061642692 -1.226121758
0.216409592 1.14955599
4591 TRMT44 7.276270204 -0.728617281 1.072598274 -0.687982018
0.491462475 0.981145514
4592 TSLP 2.464295488 -0.804299921 1.529671902 -0.525798977
0.599027885 1.488742171
4593 TTC12 7.911091222 -0.506588227 1.057068427 -0.479228826
0.621768726 0.740101998
4594 TTC21B-AS1 2.487769519 1.275974562 1.811215142 0.704485265
0.481120561 0.740101998
4595 TTC26 5.181075714 -0.654082097 1.246294568 -0.524821429
0.599707222 0.994111124
4596 TTC2P1 2.242891644 0.262422891 1.872502098 0.194089444
0.846105847 1.142885197
4597 TXNP4 2.259852662 2.041472056 2.25009556 1.251708294
0.176468619 1.449015848
4598 UBE2D2-AS1 2.956826522 -0.216262148 1.406722098 -0.224820452
0.822118954 0.740101998
4599 UBE2D2P1 2.248562227 1.855749182 1.960444576 0.946596097
0.242844624 1.485917815
4600 UBE2S 2.240056297 -0.281269262 1.908814517 -0.14740524
0.882812169 0.741928245
4601 UBOX5-AS1 2.718520722 0.872511228 1.825212614 0.475972774
0.624092775 1.144710442
4602 UBXN10-AS1 6.41660622 -0.622515887 1.127442009 -0.556965428
0.577551044 0.502721142
4603 UPB1 2.24572699 1.042882529 1.882412275 0.554251208
0.579406927 0.990401505
4604 WDR27 4.218626255 0.21260982 1.278986177 0.154178255
0.877469108 0.491429828
4605 WT1 4.920627269 0.054407458 1.29622477 0.041970546
0.966522179 1.714479018
4606 YRDC 5.406156224 -1.265848971 1.24621269 -1.095911951
0.272117209 0.721447019
4607 Z97192.1 2.476478194 0.642870865 1.788076085 0.259522164
0.71919702 0.491429828
4608 ZC2HC1C 5.195152224 -0.960128119 1.278225584 -0.751125496
0.452571115 0.990401505
4609 ZER1 6.214108271 0.285122876 1.202815979 0.219918296
0.749020192 0.500895814
4610 ZKSCAN7-AS1 2.006519809 1.619121692 2.122725202 0.759179706
0.447745064 0.740101998
4611 ZNF117 6.889299962 -0.577860521 1.105875527 -0.522526675
0.601296718 0.491429828
4612 ZNF212 6.14626042 -1.028265425 1.160020619 -0.895126705 0.27071926
1.121444022
4613 ZNF22-AS1 2.695046691 -0.949782279 1.475966652 -0.642498525
0.519900665 0.500895814
4614 ZNF200 2.250506202 0.707465902 1.709628212 0.412812722
0.679011266 0.491429828

4615	ZNF22-AS2	6.425062209	-0.626002807	1.144159476	-0.55586902	0.578200298	0.502721142
4616	ZNF224B	4.694255424	0.514956619	1.2479206	0.282027799	0.702422228	0.992424851
4617	ZNF24	11.54152891	-0.905140805	0.906742662	-0.998222292	0.218166721	0.502721142
4618	ZNF254B	2.224425665	0.272225827	1.850521221	0.201204828	0.840528408	0.500895814
4619	ZNF528-AS1	2.709172262	-0.144224899	1.447244977	-0.099654817	0.920618272	1.952050842
4620	ZNF572	2.205241002	-1.120452725	1.611140277	-0.69544141	0.486778717	0.740101998
4621	ZNF615	4.44092158	0.052505551	1.248627455	0.029674078	0.968252968	1.128144445
4622	ZNF624	2.191264292	-0.617965596	1.596188482	-0.287150767	0.698644596	0.741928245
4623	ZNF649-AS1	2.219417612	-1.695597821	1.7797875	-0.95269678	0.240742725	1.121444022
4624	ZNF681	2.450218878	-0.261052188	1.505847494	-0.229767422	0.810510555	0.990401505
4625	ZNF682	4.218626255	0.21260982	1.278986177	0.154178255	0.877469108	0.721447019
4626	ZNF696	2.724541299	-0.158742502	1.52209792	-0.10429257	0.916927174	0.491429828
4627	ZNF724	5.676452096	-0.07579171	1.204796088	-0.06290822	0.949829502	0.491429828
4628	ZNF787	2.501846129	0.616026628	1.902762728	0.22258881	0.746249261	0.502721142
4629	ZNF827	4.181967095	-0.111525649	1.407124629	-0.079257822	0.926827546	0.491429828
4630	ZNF826	4.674458069	-0.725896246	1.209184666	-0.554464266	0.579261086	1.00189182
4631	ZNF841	5.665110709	-1.146667582	1.210204152	-0.947421009	0.24242429	0.500895814
4632	ZSCAN18	5.427194852	-0.765121295	1.222415872	-0.62082864	0.524705797	0.502721142
4633	ABCG1	2.721254871	0.87522192	1.824758202	0.477077514	0.622206926	0.500895814
4634	ABHD11-AS1	5.44281441	0.212916174	1.219480908	0.257417867	0.796856212	0.502721142
4635	ABHD12B	5.214848492	-0.098076298	1.258707552	-0.077918226	0.927892014	1.494282804
4636	AC002542.1	2.245625792	0.26571051	1.907058227	0.191766819	0.847924862	1.471851115
4637	AC002070.1	5.468182246	0.202918027	1.224026265	0.245469287	0.806092086	0.740101998
4638	AC004477.2	5.20922786	0.181451425	1.240621062	0.146257261	0.882718225	0.500895814
4639	AC004596.1	2.487668221	0.626205552	1.774027842	0.258620057	0.71987924	1.141100011
4640	AC004802.1	4.48021499	0.260877	1.256911059	0.265954792		

	0.790274025	1.004718177			
4641	AC005024.4	2.464244252	0.052675146	1.496950857	0.025188292
	0.971929597	0.970854101			
4642	AC005288.1	4.920526071	-0.22572072	1.269520762	-0.185682254
	0.852692082	1.144710442			
4643	AC005229.2	5.648147616	-0.580294886	1.211148212	-0.479210455
	0.621788917	0.748558974			
4644	AC005578.1	2.99145062	0.028656254	1.668296705	0.02217109
	0.981512799	0.981145514			
4645	AC005674.2	2.718419524	0.225202051	1.728099671	0.188185256
	0.850721252	0.500895814			
4646	AC006128.1	2.976522691	0.784996425	1.415894919	0.554417149
	0.579292292	0.751294212			
4647	AC006460.1	4.210069179	-0.122785462	1.261097607	-0.090210622
	0.928119842	1.00189182			
4648	AC007014.2	4.229866545	1.21225804	1.412642929	0.92905925
	0.252858281	0.748558974			
4649	AC007242.6	5.402269741	-1.045620799	1.225247866	-0.846418104
	0.297219542	1.449015848			
4650	AC007879.2	2.741002175	1.469224157	1.744185281	0.842418572
	0.299552701	0.990401505			
4651	AC008149.1	5.289142069	-1.709948467	1.207642721	-1.207656227
	0.190989922	1.00189182			
4652	AC008649.1	4.198777854	-0.464585652	1.268492226	-0.229486994
	0.724242892	0.502721142			
4653	AC008927.2	2.760749478	2.205240178	1.898497754	1.161571128
	0.245409715	1.128144445			
4654	AC009202.4	2.24761982	1.210521175	1.604205542	0.75454528
	0.450521867	1.00189182			
4655	AC009948.2	2.012089204	2.850902299	2.222915192	1.226767401
	0.219910027	0.748558974			
4656	AC009961.1	6.909046068	-0.581462724	1.08461247	-0.526102755
	0.591887526	1.449015848			
4657	AC010186.2	2.220707862	1.226482146	1.606447545	0.762474755
	0.445180212	1.128144445			
4658	AC010225.1	6.146209282	-0.520222499	1.155596414	-0.458926225
	0.646287146	0.502721142			
4659	AC010401.1	4.657444914	-1.056812284	1.250207894	-0.782704196
	0.422800828	0.990401505			
4660	AC010469.1	4.224195851	0.560112291	1.270525865	0.408681226
	0.682772606	0.502721142			
4661	AC010889.1	2.487668221	0.626205552	1.774027842	0.258620057
	0.71987924	0.740101998			
4662	AC011922.4	2.240005161	1.056725024	1.89621058	0.55725214
	0.577254488	1.714479018			
4663	AC012569.1	2.498959646	1.267254929	1.808826647	0.700650298
	0.482521281	0.491429828			
4664	AC012615.2	2.489612287	0.026576221	1.557174642	0.022488907
	0.981260287	0.491429828			
4665	AC015871.2	2.220657802	-0.179297466	1.578825021	-0.112626479
	0.909522896	0.721447019			

4666	AC018262.2	2.749458152	1.459202242	1.742459228	0.82749577	0.402212952	0.994111124
4667	AC018521.5	4.201612201	-0.118628666	1.261467244	-0.08712295	0.920565825	0.491429828
4668	AC018628.4	2.686489515	-1.402282952	1.61812755	-0.867226414	0.285817966	1.457714542
4669	AC018628.7	2.962406019	0.04922258	1.299118578	0.025181125	0.971925205	0.741928245
4670	AC018644.1	2.720412552	1.058000214	1.507908191	0.701624426	0.48290717	0.500895814
4671	AC019077.1	2.456629692	-0.516226752	1.970140281	-0.262081198	0.792258842	0.97921018
4672	AC020909.2	2.476276996	0.056425581	1.796572999	0.021407241	0.974944687	1.444180511
4673	AC022400.7	2.742827522	2.221779824	1.902952791	1.172181722	0.241124105	1.501889844
4674	AC025171.2	9.576984228	-1.120186412	0.946424279	-1.182586052	0.226576964	1.719514275
4675	AC025171.4	2.945494062	0.058571526	1.429902651	0.040961904	0.967226266	0.987744158
4676	AC025178.2	5.69047757	0.174465699	1.189250225	0.146702262	0.882267022	1.477471847
4677	AC025569.1	4.170574572	-0.81441786	1.41822861	-0.574250057	0.5657986	0.751294212
4678	AC026785.2	2.700616187	-0.521792227	1.466628844	-0.262592176	0.716908808	1.480207192
4679	AC024105.2	2.245625792	0.26571051	1.907058227	0.191766819	0.847924862	1.984089491
4680	AC046120.2	2.498959646	1.267254929	1.808826647	0.700650298	0.482521281	0.741928245
4681	AC068412.1	2.226228495	0.722916952	1.560701871	0.462840628	0.642761925	1.111251708
4682	AC068621.1	2.469864982	-0.268764021	1.515505292	-0.242227445	0.807751745	1.491598519
4683	AC068790.2	2.971702216	-0.425658256	1.654241941	-0.262258206	0.792274422	1.7208057
4684	AC069224.2	2.465085671	-0.522222025	1.924225618	-0.271951295	0.785659292	0.741928245
4685	AC072062.1	2.722496125	-0.216764704	1.726455588	-0.125554752	0.900084282	0.502721142
4686	AC072862.1	2.22502717	0.272458122	1.549262195	0.175851681	0.860410467	0.491429828
4687	AC079205.2	7.295966472	-0.141117826	1.067124526	-0.122229968	0.894794489	0.502721142
4688	AC079907.1	2.724490162	0.627750585	1.462792196	0.429145429	0.667817292	0.502721142
4689	AC080112.4	2.467029627	-0.802224225	1.561701755	-0.512685924	0.607471588	0.502721142
4690	AC080188.2	2.245625792	0.26571051	1.907058227	0.191766819	0.847924862	0.491429828
4691	AC082799.1	2.954791259	-0.424491087	1.674250872	-0.252525766		

							0.799861952	0.740101998
4692	AC084824.2	4.688482542	-0.408481174	1.29460262	-0.2155262		0.752262086	0.500895814
4693	AC090114.2	2.006418611	0.772125802	2.128152669	0.261118181		0.718011102	0.741928245
4694	AC091117.2	5.960672219	-0.202812609	1.202062222	-0.169552292		0.865261457	1.14955599
4695	AC091152.2	2.456629692	-0.516226752	1.970140281	-0.262081198		0.792258842	0.500895814
4696	AC092120.2	4.195942507	-0.825218094	1.291066552	-0.592298784		0.552981212	0.500895814
4697	AC092284.2	7.956205297	0.585971488	1.079681905	0.542726042		0.58721844	0.502721142
4698	AC092267.1	4.442655729	0.054770928	1.226652245	0.040976175		0.967214889	0.981145514
4699	AC092525.1	5.19515125	0.468250282	1.256958949	0.272526292		0.709500965	0.502721142
4700	AC092672.1	2.740952112	-0.222225254	1.76612719	-0.126298222		0.899416702	0.999057482
4701	AC092866.1	4.471859011	0.265484761	1.224687579	0.27282544		0.784211087	0.751294212
4702	AC098591.2	2.92126729	-0.678069122	1.446290824	-0.46880077		0.629212049	1.119808484
4703	AC102702.1	6.921628718	-0.162286027	1.092427789	-0.149224075		0.881290029	0.741928245
4704	AC102729.2	4.899487442	-0.822201779	1.220182776	-0.620442878		0.52840421	1.71119944
4705	AC104115.2	2.208125212	0.28476809	1.602561456	0.177695582		0.858962055	0.970854101
4706	AC111170.2	8.096625998	-0.946752755	1.02419244	-0.924288614		0.255284021	1.004718177
4707	AC111170.2	4.978476657	0.226866276	1.204269174	0.25061259		0.802112647	0.491429828
4708	AC112196.1	2.507415625	1.256089292	1.912614929	0.656729246		0.511248595	0.491429828
4709	AC114956.2	2.74278746	0.202229298	1.724274001	0.175922909		0.860254508	0.741928245
4710	AC116902.1	10.59026968	-0.565227256	0.915971666	-0.617090228		0.527175148	1.111251708
4711	AC120114.2	4.22265182	0.555021264	1.288058869	0.299861472		0.68925855	1.975422514
4712	AC120249.2	2.226228495	0.722916952	1.560701871	0.462840628		0.642761925	0.502721142
4713	AC122688.2	2.714742859	0.244454577	1.446442921	0.169002829		0.865792622	0.751294212
4714	AC122712.2	4.184651182	-1.210846226	1.425022877	-0.842781897		0.298791292	0.491429828
4715	AC124248.1	2.726875502	0.217817717	1.692514296	0.187778442		0.851050226	1.128144445
4716	AC129102.1	4.967185222	0.040579221	1.280200022	0.021697649		0.97471217	0.721447019

4717	AC124050.1	2.755078785	0.829779272	1.828552805	0.459258716	0.646048291	1.491598519
4718	AC124249.1	5.918292427	-0.188954216	1.167664475	-0.161822256	0.871445748	0.970854101
4719	AC126469.2	6.889248826	-0.150425609	1.120004652	-0.124208022	0.89215901	0.500895814
4720	AC127922.1	2.48120627	1.277740294	1.562927874	0.880942109	0.278248612	0.500895814
4721	AC128028.6	4.195942507	-0.825218094	1.291066552	-0.592298784	0.552981212	0.97921018
4722	AC128207.1	2.757914122	1.448260425	1.828816688	0.787658952	0.420896222	1.488742171
4723	AC128207.4	2.479212242	0.644922229	1.798969616	0.258500962	0.719968448	0.491429828
4724	AC129495.2	2.245625792	0.26571051	1.907058227	0.191766819	0.847924862	0.751294212
4725	AC129769.2	2.70628688	0.249692457	1.465078669	0.170420068	0.864671927	0.491429828
4726	AC129795.2	4.181865897	-0.456811209	1.28926428	-0.228791507	0.742212282	1.941554904
4727	AC129887.4	2.487668221	0.626205552	1.774027842	0.258620057	0.71987924	0.491429828
4728	AC144821.1	2.469864982	-0.268764021	1.515505292	-0.242227445	0.807751745	1.705287702
4729	AC241585.1	2.4961242	0.627522174	1.795708621	0.249462128	0.726742291	0.502721142
4730	AC244092.2	2.475525677	0.464274254	1.495846542	0.210275591	0.756275254	0.721447019
4731	AC245884.8	2.229162842	1.218502796	1.582981766	0.769266202	0.441725227	0.75411947
4732	AC246817.1	4.440770221	-1.221079275	1.410152885	-0.942925577	0.245207681	1.00189182
4733	ACSM1	2.256917118	1.024272126	2.006599649	0.515425721	0.606248577	0.751294212
4734	ACSS1	7.407257797	0.050242195	1.068847701	0.047005944	0.96250849	0.491429828
4735	ADAM19	2.721704877	1.514610048	1.52725681	0.991654266	0.221266159	0.491429828
4736	ADGRB2	2.225878489	-0.262922251	2.006280297	-0.12104262	0.895740801	0.748558974
4737	AF127577.1	2.694995555	-0.122105802	1.478002627	-0.090057892	0.928241211	0.990401505
4738	AF220666.1	2.22502717	0.272458122	1.549262195	0.175851681	0.860410467	0.500895814
4739	AGAP4	4.457722229	-0.276509255	1.229402689	-0.207994951	0.82522291	1.118517241
4740	AGBL2	2.492497696	1.926617498	1.606462789	1.199291705	0.220414529	1.724414221
4741	AGER	4.902272727	-1.52880878	1.255150529	-1.128146824	0.259257912	0.491429828
4742	AJ271726.1	2.251296486	1.857557266	1.959919185	0.947772278		

						0.242245245	0.748558974
4743	AL022211.1	5.684756815	-1.150296849	1.22810184	-0.926646141	0.248940609	1.444180511
4744	AL021595.2	4.442605667	-0.952299805	1.261605041	-0.699468478	0.484259206	0.748558974
4745	AL025252.5	7.89124272	-0.887968185	1.028265919	-0.862474925	0.2878764	1.4744245
4746	AL109761.1	5.92962469	-0.692276878	1.168964062	-0.592212995	0.552707299	0.502721142
4747	AL122780.1	4.907942421	-0.825478815	1.298447925	-0.642444217	0.5199259	0.751294212
4748	AL126220.1	2.974528662	0.051140629	1.618421915	0.021599077	0.974791779	0.502721142
4749	AL128921.1	4.680027564	-0.404974295	1.202162196	-0.210762848	0.755980915	1.144710442
4750	AL129021.2	4.691218889	-0.099667761	1.29490654	-0.076969077	0.928648145	0.500895814
4751	AL129260.1	2.755078785	0.829779272	1.828552805	0.459258716	0.646048291	1.722590985
4752	AL129288.1	2.718419524	0.225202051	1.728099671	0.188185256	0.850721252	0.491429828
4753	AL129285.1	2.012089204	2.850902299	2.222915192	1.226767401	0.219910027	0.500895814
4754	AL162578.1	2.966082685	0.057229144	1.620782052	0.025154288	0.971956622	1.480207192
4755	AL258927.1	5.424258208	-1.271876162	1.275592269	-1.075481717	0.282159114	0.502721142
4756	AL292172.1	6.681120211	-0.278457721	1.108171951	-0.251276646	0.80160022	1.485917815
4757	AL451085.2	6.426202298	0.045522226	1.126642582	0.040406067	0.967769294	1.947177525
4758	AL512504.1	2.70628688	0.249692457	1.465078669	0.170420068	0.864671927	0.741928245
4759	AL589925.1	4.477479642	0.02812084	1.261429128	0.028007745	0.977655974	1.480207192
4760	AL591895.1	4.210069179	-0.122785462	1.261097607	-0.090210622	0.928119842	0.97921018
4761	AL602829.2	5.665059572	-0.586047602	1.187582455	-0.49247909	0.621674111	1.119808484
4762	AL662844.4	2.922911412	-0.672951465	1.475082198	-0.456890476	0.64774977	0.990401505
4763	ALG14	4.725192865	0.822274299	1.215961292	0.624922862	0.522021686	1.714479018
4764	AMZ2P2	2.259752465	1.842112059	2.041721712	0.902724425	0.26667217	1.144710442
4765	ANKRD19P	4.225487176	0.916428766	1.295046702	0.656922259	0.511220197	1.449015848
4766	ANKRD26	5.172518528	-0.950072299	1.252804097	-0.758256625	0.448227511	0.992424851
4767	ANKS1B	2.475525677	0.464274254	1.495846542	0.210275591	0.756275254	1.981154144

4768 ANTKMT 4.92898205 -0.229195485 1.261004411 -0.189686478 0.849554818 0.75411947
4769 AP000247.1 2.728819469 -0.152084212 1.475728611 -0.102057022 0.917917706 0.502721142
4770 AP000487.1 4.685648196 -0.727651542 1.205212278 -0.557452525 0.577217604 1.004718177
4771 AP000640.1 2.72971085 0.86591155 1.719222972 0.50262226 0.614519819 0.97921018
4772 AP000752.2 2.459465029 0.072622682 1.967026824 0.026920524 0.970548267 0.491429828
4773 AP000781.1 4.922261418 0.055527715 1.281006812 0.042254728 0.965418758 0.491429828
4774 AP001062.1 6.92217274 -0.160724702 1.08841204 -0.147668987 0.882604008 0.741928245
4775 AP001469.1 2.997121212 1.041078622 1.654762212 0.629140882 0.529256827 1.978448841
4776 AP002907.1 2.484822974 0.048987522 1.795064196 0.027290128 0.97822822 0.502721142
4777 AP002096.1 7.298701695 -1.178556642 1.071275294 -1.100040797 0.271214247 0.491429828
4778 AP002774.2 2.450167742 0.482258472 1.661262129 0.290898286 0.771129026 0.992424851
4779 AP004289.2 2.256917118 1.024272126 2.006599649 0.515425721 0.606248577 1.111251708
4780 AP005121.4 2.262587811 2.042169921 2.242528126 1.256421564 0.174965076 1.741097014
4781 AP006621.1 2.722546196 1.480299822 1.844241942 0.802616802 0.422196254 0.751294212
4782 APOBEC2H 7.108758642 -1.097225541 1.090788014 -1.005992299 0.214418746 0.990401505
4783 APOM 2.700616187 -0.521792227 1.466628844 -0.262592176 0.716908808 1.719514275
4784 ARHGAP19 6.641625604 -0.71292014 1.107282191 -0.64470076 0.519121124 0.500895814
4785 ARHGAP26-IT1 2.962247228 -0.420122564 1.655228025 -0.259855412 0.794975205 1.00189182
4786 ARHGEF10 2.72971085 0.86591155 1.719222972 0.50262226 0.614519819 0.999057482
4787 ARHGEF11 6.180122197 -0.540742762 1.145146966 -0.472202812 0.626781219 0.500895814
4788 ARHGEF27 2.461409005 -0.262982287 1.509216824 -0.24117262 0.809420556 0.500895814
4789 ARMCX5-GPRASP2 2.478271024 0.899900607 1.514976746 0.594002917 0.552510162 0.502721142
4790 ASPHD2 2.495282981 0.886290595 1.545692124 0.572458228 0.566224407 0.500895814
4791 ATF2 2.994226028 2.282708228 1.850248729 1.287707662 0.197847745 0.740101998
4792 ATP6V1G2 2.692160208 -0.527429826 1.47884579 -0.256656414 0.721249029 0.491429828
4793 AVPI1 9.220915251 -0.560454177 0.996662025 -0.562220655

						0.572890756	0.500895814
4794	B2GNT9	7.428296426	0.42158809	1.078609278	0.400122856	0.689057929	1.144710442
4795	BAIAP2-DT	2.718419524	0.225202051	1.728099671	0.188185256	0.850721252	0.491429828
4796	BCAN	9.568578411	-0.622081192	0.976658274	-0.627972574	0.522491522	0.751294212
4797	BCL11A	4.425199751	0.059024678	1.262062059	0.042210206	0.965454176	0.502721142
4798	BLOC1S1	5.921228774	0.05252029	1.178726851	0.045412726	0.962777522	0.748558974
4799	BLOC1S4	6.160425955	-0.060872509	1.162254162	-0.052275285	0.958229588	0.751294212
4800	BSCL2	2.487668221	0.626205552	1.774027842	0.258620057	0.71987924	1.14955599
4801	BTG2-AS1	4.955944068	0.626557149	1.282651265	0.495895705	0.619967996	1.142925258
4802	BX470102.1	2.988665225	1.049260288	1.622921258	0.642172166	0.520760754	0.491429828
4803	C10orf90	2.510250972	2.02547897	1.96258525	1.027141682	0.299669821	0.741928245
4804	C14orf28	2.225878489	-0.262922251	2.006280297	-0.12104262	0.895740801	0.981145514
4805	C20orf197	2.70628688	0.249692457	1.465078669	0.170420068	0.864671927	1.14955599
4806	C4orf26	2.205289866	-0.162125726	1.581200855	-0.102165527	0.917821599	1.4744245
4807	C7orf26	5.442764248	-0.486280026	1.214590147	-0.400447861	0.688826679	1.477471847
4808	CA11	2.988665225	1.049260288	1.622921258	0.642172166	0.520760754	1.14955599
4809	CACTIN	2.191162194	-1.108475129	1.726852122	-0.641904702	0.520925061	0.740101998
4810	CALD1	2.240005161	1.056725024	1.89621058	0.55725214	0.577254488	1.952050842
4811	CAMP	5.424258422	0.217609217	1.224502879	0.257276888	0.796965022	1.49711915
4812	CAPN10	8.220162486	-1.421200296	1.018640798	-1.295192887	0.162957681	1.984914829
4813	CARD8-AS1	5.161277274	-0.256720976	1.267747077	-0.281281817	0.778417562	0.740101998
4814	CASC20	7.124126578	-1.102268542	1.076686127	-1.024781982	0.20546607	0.502721142
4815	CASKIN1	7.290295778	-0.52542215	1.054427799	-0.507780687	0.611607156	1.715125007
4816	CBR2-AS1	2.971702216	-0.425658256	1.654241941	-0.262258206	0.792274422	1.741097014
4817	CCDC106	7.429740224	-0.750205287	1.089472422	-0.68868681	0.491020279	0.491429828
4818	CCDC150	2.4961242	0.627522174	1.795708621	0.249462128	0.726742291	1.00189182

4819	CCDC151	2.22502717	0.272458122	1.549262195	0.175851681	0.860410467	0.992424851
4820	CCDC162	5.175402946	0.196282622	1.284472241	0.152890515	0.878484616	0.502721142
4821	CCDC84-DT	2.948279247	-0.685987852	1.425442895	-0.481245072	0.62024222	0.75411947
4822	CD2	2.458572658	-0.797815022	1.550904052	-0.514419226	0.606958842	0.992424851
4823	CDC42EP2	5.715845506	0.164267968	1.214794642	0.125222829	0.892425698	0.751294212
4824	CDIPT	14.02278007	-0.841142167	0.825292596	-1.019081287	0.208164278	0.502721142
4825	CDNF	2.994226028	2.282708228	1.850248729	1.287707662	0.197847745	1.151291227
4826	CDYL	10.11747595	-0.599865111	0.922820827	-0.642058825	0.520185924	1.7208057
4827	CEACAM21	5.648147616	-0.580294886	1.211148212	-0.479210455	0.621788917	0.751294212
4828	CEBPE	4.892816749	-1.526007585	1.265205986	-1.117785594	0.262658575	0.721447019
4829	CFAP410	9.118267206	-1.290042568	0.982945959	-1.412722489	0.157727042	0.748558974
4830	CFAP58-DT	4.682862911	-0.095859078	1.210279044	-0.072159285	0.941679265	0.502721142
4831	CFI	2.22502717	0.272458122	1.549262195	0.175851681	0.860410467	1.151291227
4832	CHAF1B	5.429979062	0.049225229	1.209589742	0.040778569	0.967472425	1.488742171
4833	CHCHD2P2	2.69778084	-0.947685059	1.507172615	-0.628782259	0.529490896	0.491429828
4834	CHN1	2.712748821	-0.749692997	1.827252919	-0.408029292	0.682252095	1.110041282
4835	CHST1	2.722248899	1.522154551	1.620122061	0.940141629	0.247144912	0.740101998
4836	CHTF18	7.196202825	-0.262402222	1.109129582	-0.226581795	0.812981245	0.491429828
4837	CNTNAP5	2.985829988	0.522021927	1.617840927	0.228852057	0.742266758	0.500895814
4838	COCH	5.707429589	0.957477591	1.227212809	0.780204282	0.425270582	1.712842481
4839	COL18A1	2.724490162	0.627750585	1.462792196	0.429145429	0.667817292	0.740101998
4840	CPEB2	4.967185222	0.040579221	1.280200022	0.021697649	0.97471217	1.128144445
4841	CSGALNACT1	2.479212242	0.644922229	1.798969616	0.258500962	0.719968448	0.491429828
4842	CTNNBIP1	4.172409918	-0.45276582	1.418144942	-0.219266259	0.749524616	1.488742171
4843	CTRL	14.10570476	0.298172672	0.876627588	0.240127221	0.722752192	1.494422845
4844	CYP2S1	5.425852292	-0.480421944	1.208520127	-0.297524102		

0.690972642 0.751294212
4845 CYTH2 8.88478078 -0.42980442 0.980192201 -0.448692021
0.652652841 0.502721142
4846 DCAF4L1 2.012089204 2.850902299 2.222915192 1.226767401
0.219910027 0.987744158
4847 DCHS1 2.486827002 0.892242777 1.514200867 0.589910259
0.555250749 1.978418799
4848 DCUN1D2 7.640744222 -1.025077268 1.045149222 -0.980795122
0.226692779 0.502721142
4849 DDAH1 2.498959646 1.267254929 1.808826647 0.700650298
0.482521281 0.491429828
4850 DDR1 2.72971085 0.86591155 1.719222972 0.50262226
0.614519819 0.502721142
4851 DDX50P1 2.22502717 0.272458122 1.549262195 0.175851681
0.860410467 0.748558974
4852 DEPDC1 2.984988669 0.779204556 1.426279278 0.546289875
0.584797969 1.11497224
4853 DGKE 2.495282981 0.886290595 1.545692124 0.572458228
0.566224407 0.500895814
4854 DHRS4L2 7.156709228 -0.664948601 1.069879144 -0.621517491
0.52425919 0.97921018
4855 DMWD 2.928522042 -1.079712789 1.479021667 -0.720018912
0.465278624 0.75411947
4856 DNAJC28 6.878007429 -1.02622028 1.095680728 -0.926605202
0.248961624 0.75411947
4857 DNAL1 5.960722281 0.528462015 1.182984282 0.446242049
0.655250187 1.449015848
4858 DOCK6 11.26156257 0.174128725 0.927770042 0.187695999
0.851114967 1.720755429
4859 DOCK8-AS1 4.696929521 -0.411962955 1.294254929 -0.218276651
0.750275094 0.75411947
4860 DPY19L1P1 4.184701244 -0.110107651 1.400149659 -0.078629916
0.927219028 0.987744158
4861 DTX2P1 2.009252958 1.621179422 2.142556869 0.756202425
0.449467289 0.500895814
4862 DYNC2H1 2.21742251 -0.254807471 2.046072785 -0.12452484
0.900891817 0.999057482
4863 ECT2L 2.220707862 1.226482146 1.606447545 0.762474755
0.445180212 0.500895814
4864 EDDM12 5.45689102 0.042540894 1.217664679 0.02492646
0.972120407 0.987744158
4865 EEF1A1P12 2.7522925 2.218521715 1.821202696 1.211449272
0.225722227 1.125419218
4866 EFHC2 7.412778205 -1.419592278 1.108098472 -1.281107602
0.200155874 1.500054497
4867 EIF4BP6 2.498959646 1.267254929 1.808826647 0.700650298
0.482521281 0.491429828
4868 ENAH 2.24761982 1.210521175 1.604205542 0.75454528
0.450521867 0.721447019
4869 ENTPD2-AS1 2.482991655 0.458282864 1.506245792 0.204201221
0.760898415 1.924128904

4870	EPN1	7.874280825	-0.209201288	1.045001612	-0.295981722	0.767244022	1.141100011
4871	ETNK2	2.492229015	2.062125264	1.96850427	1.048072527	0.294605188	1.715125007
4872	FABP5P7	5.226189879	1.044194221	1.274219782	0.819477412	0.412514084	0.502721142
4873	FAHD2CP	5.68197152	-0.591602722	1.187401478	-0.498222945	0.618219159	0.500895814
4874	FAM185A	6.154765262	-0.522958597	1.145257557	-0.465261245	0.641672711	0.491429828
4875	FAM219A	5.226189879	1.044194221	1.274219782	0.819477412	0.412514084	0.992424851
4876	FAM229B	2.92126729	-0.678069122	1.446290824	-0.46880077	0.629212049	0.500895814
4877	FAM241A	2.997121212	1.041078622	1.654762212	0.629140882	0.529256827	0.748558974
4878	FAM86B2P	5.672515552	-0.588825512	1.184728287	-0.497012129	0.619179792	0.994111124
4879	FAM86C1	2.70628688	0.249692457	1.465078669	0.170420068	0.864671927	0.992424851
4880	FAM86DP	10.5169011	-1.214297762	0.922102524	-1.410142907	0.15849749	0.740101998
4881	FBXL19-AS1	2.740160856	1.50699227	1.506656462	1.000222882	0.217202658	0.741928245
4882	FBXL6	9.605227672	-0.474617224	0.952757862	-0.498150949	0.618277652	0.999057482
4883	FBXO10	2.000797979	1.626242586	2.051844028	0.797498521	0.425161561	0.987744158
4884	FBXO22	9.101255249	-1.286995504	0.974147999	-1.422802679	0.15450221	0.502721142
4885	FBXW9	5.222254522	0.742975925	1.262252277	0.588610772	0.556122406	0.491429828
4886	FCRL2	5.926899467	0.542022052	1.226209452	0.44281894	0.657896702	0.500895814
4887	FCSK	9.661694298	0.265652492	0.978602812	0.271460717	0.786026709	0.491429828
4888	FDX1	5.402219802	-0.201924162	1.272620717	-0.158666726	0.872921447	0.500895814
4889	FERP1	2.487668221	0.626205552	1.774027842	0.258620057	0.71987924	0.987744158
4890	FGFR1	2.988665225	1.049260288	1.622921258	0.642172166	0.520760754	0.502721142
4891	FHIT	7.87716612	-0.687595008	1.025569692	-0.670451762	0.502569847	0.502721142
4892	FHL1	2.489612287	0.026576221	1.557174642	0.022488907	0.981260287	0.75411947
4893	FKTN	7.267762189	-0.229552424	1.098891571	-0.299895206	0.764257015	0.751294212
4894	FLT1	2.70628688	0.249692457	1.465078669	0.170420068	0.864671927	0.740101998
4895	FOXD2-AS1	2.481997628	-0.526746245	1.92098547	-0.279411922		

						0.779928722	0.748558974
4896	FOXP1-IT1	2.728166828	0.857474619	1.695229454	0.505812274	0.612987729	0.994111124
4897	FSD2	6.715054249	1.042827726	1.152928915	0.904585812	0.265684854	0.748558974
4898	FZD2	14.59541045	0.290202082	0.868915192	0.222982056	0.7282921	0.500895814
4899	GABARAP	6.672674222	-0.275884781	1.102165142	-0.250084751	0.802521808	1.449015848
4900	GAMT	9.577024289	-0.624788621	0.965554295	-0.647077601	0.517581721	1.449015848
4901	GAPLINC	2.492288952	0.041469919	1.828202599	0.022682184	0.981902787	0.500895814
4902	GARNL2	1.997962622	0.784194546	2.014008252	0.289270057	0.697002419	0.491429828
4903	GAS8	8.286619112	-0.467775695	1.001129254	-0.467248052	0.640222417	0.491429828
4904	GBP1	2.712748821	-0.749692997	1.827252919	-0.408029292	0.682252095	0.990401505
4905	GINS1	7.4270051	0.228852006	1.069877582	0.222251716	0.822229604	0.502721142
4906	GK-AS1	4.944652742	0.242161897	1.279684017	0.268161421	0.788575062	0.491429828
4907	GLRA2	2.962406019	0.04922258	1.299118578	0.025181125	0.971925205	1.128144445
4908	GLUD1P2	2.222201822	-0.172982752	1.56150099	-0.1114202	0.911282142	0.992424851
4909	GNG11	2.72971085	0.86591155	1.719222972	0.50262226	0.614519819	0.981145514
4910	GPR174	4.471859011	0.265484761	1.224687579	0.27282544	0.784211087	1.971848119
4911	GPR75	5.152771224	-1.26705281	1.284267765	-0.986519472	0.222878265	0.500895814
4912	GPR89A	5.172468476	-2.040055982	1.422607117	-1.422022056	0.154729472	0.491429828
4913	GPR89B	2.959620724	0.796614224	1.465726174	0.542490942	0.586791826	0.491429828
4914	GPRASP1	1.997962622	0.784194546	2.014008252	0.289270057	0.697002419	0.491429828
4915	GPX1P1	2.481156209	0.042104208	1.512784567	0.027812871	0.977810602	0.502721142
4916	GRIK5	4.95205866	-0.542224252	1.279016262	-0.422946299	0.671604978	0.990401505
4917	GRIP2	2.464244252	0.052675146	1.496950857	0.025188292	0.971929597	1.14955599
4918	H2AC11	2.476276996	0.056425581	1.796572999	0.021407241	0.974944687	1.142925258
4919	HCG4P8	4.471909072	1.424820571	1.417877454	1.011956687	0.211558762	0.97921018
4920	HDAC9	2.242840508	1.872052964	2.048252979	0.912975462	0.260729729	1.482091478

4921	HELZ2	5.665059572	-0.586047602	1.187582455	-0.49247909	0.621674111	0.491429828
4922	HEXA-AS1	5.164062559	-0.947224986	1.255699202	-0.75424858	0.450629929	0.500895814
4923	HEXIM2	5.621225659	-0.574419419	1.267902448	-0.452046657	0.650515145	0.741928245
4924	HIP1R	4.224245912	1.762820281	1.552929017	1.125158211	0.256208992	1.111251708
4925	HLA-DRB6	2.221549182	1.068548289	2.012896629	0.520587456	0.59570469	0.721447019
4926	HMGB1P10	2.72971085	0.86591155	1.719222972	0.50262226	0.614519819	0.740101998
4927	HNRNPA2P9	2.999906598	0.021684794	1.789460266	0.017706241	0.985872122	1.118517241
4928	HNRNPRP1	2.714742859	0.244454577	1.446442921	0.169002829	0.865792622	1.952050842
4929	HORMAD2	2.009252958	1.621179422	2.142556869	0.756202425	0.449467289	1.119808484
4930	HOXA10	4.725142802	-0.114911522	1.227454251	-0.085918102	0.92152152	1.128144445
4931	HOXB-AS1	4.204448547	0.222051075	1.267718129	0.162082624	0.870452272	0.502721142
4932	HYI	7.267712128	-0.947847877	1.061946752	-0.892556876	0.272094521	1.984914829
4933	IBA57	6.257262121	-1.680056524	1.19425202	-1.406667785	0.159525922	0.500895814
4934	IGFBP6	2.492229015	2.062125264	1.96850427	1.048072527	0.294605188	0.491429828
4935	IGHEP2	6.872426869	-0.144900285	1.177429454	-0.122062895	0.902056502	0.987744158
4936	IL12RA1	5.448425042	0.045947622	1.209482924	0.027989444	0.969696099	0.500895814
4937	IL20RB	2.504580278	0.618049929	1.915204851	0.222706957	0.746917182	0.75411947
4938	IMPDH1P8	2.741002175	1.469224157	1.744185281	0.842418572	0.299552701	0.740101998
4939	IQGAP2	8.122225197	-1.271476228	1.024242264	-1.226067264	0.184817285	1.971798147
4940	IRF2BPL	11.51605978	-1.029982807	0.895162228	-1.161780068	0.245224812	0.491429828
4941	IRS2	4.964249985	-0.249627498	1.286675142	-0.194017502	0.846162177	1.719514275
4942	ITGB5	2.962406019	0.04922258	1.299118578	0.025181125	0.971925205	1.480207192
4943	ITPR2	2.450167742	0.482258472	1.661262129	0.290898286	0.771129026	1.991595522
4944	JADE2	2.726875502	0.217817717	1.692514296	0.187778442	0.851050226	0.981145514
4945	KBTBD11	2.216581192	0.278489262	1.561544525	0.178242248	0.858454201	1.00189182
4946	KCTD2	4.224145789	-0.476212991	1.415681589	-0.226455594

						0.726527222	1.499747071
4947	KIAA1958	2.95295004	0.052842019	1.406752482	0.028272982	0.969469225	1.944241189
4948	KIF26B	8.144526522	-1.159622482	1.021771861	-1.122912654	0.261049658	0.992424851
4949	KIFC2	2.980159295	-0.441207969	1.671217668	-0.262988096	0.791789092	0.500895814
4950	KLC2	4.215689811	-0.472221274	1.287810574	-0.240225621	0.722602795	0.751294212
4951	L24079.2	4.907942421	-0.825478815	1.298447925	-0.642444217	0.5199259	0.502721142
4952	LCA5	2.722198827	0.229295008	1.445887741	0.165500406	0.86855012	0.748558974
4953	LDHAP2	2.721654816	0.224070201	1.46226816	0.15995205	0.872918058	0.491429828
4954	LEAP2	2.966082685	0.057229144	1.620782052	0.025154288	0.971956622	0.741928245
4955	LEPR	6.422416989	-0.877161142	1.150878826	-0.762166268	0.445960716	1.00189182
4956	LHFPL6	2.492288952	0.041469919	1.828202599	0.022682184	0.981902787	0.751294212
4957	LINC00299	6.149094568	-1.027097244	1.152549989	-0.899048275	0.268626894	0.75411947
4958	LINC00512	4.71290154	0.50702006	1.200590672	0.289845991	0.696650422	0.721447019
4959	LINC00529	4.71290154	0.50702006	1.200590672	0.289845991	0.696650422	0.741928245
4960	LINC00707	2.245625792	0.26571051	1.907058227	0.191766819	0.847924862	0.500895814
4961	LINC00869	2.74278746	0.202229298	1.724274001	0.175922909	0.860254508	0.502721142
4962	LINC01215	2.261746492	2.49461281	1.80019914	1.285742696	0.165825486	1.44055989
4963	LINC01281	5.66789492	-0.225992652	1.189201	-0.274104412	0.784004282	1.449148545
4964	LINC01252	2.498959646	1.267254929	1.808826647	0.700650298	0.482521281	1.712842481
4965	LINC02284	2.692160208	-0.527429826	1.47884579	-0.256656414	0.721249029	1.004718177
4966	LINC02285	8.628611579	-0.892771924	0.989577457	-0.902185412	0.266427498	0.721447019
4967	LOXL4	2.476276996	0.056425581	1.796572999	0.021407241	0.974944687	1.477471847
4968	LRATD1	4.474694258	0.697198005	1.241212792	0.519787785	0.602211494	0.740101998
4969	LRFN2	2.746622806	0.849066698	1.716544972	0.494627025	0.620856262	0.75411947
4970	LRP6	5.180974516	-0.952902828	1.255242972	-0.759078474	0.447805615	0.748558974
4971	LRRC27A17P	2.728166828	0.857474619	1.695229454	0.505812274	0.612987729	0.999057482

4972	LRRC29	2.720412552	1.058000214	1.507908191	0.701624426	0.48290717	0.987744158
4973	LRRC46	2.472541649	-0.520070558	1.910671706	-0.277426287	0.781452799	0.75411947
4974	LRRFIP1P1	4.905108074	-1.165758278	1.214428548	-0.886892608	0.275126182	1.729141479
4975	LYPLA1P2	2.259752465	1.842112059	2.041721712	0.902724425	0.26667217	1.978448841
4976	LZTS2	11.62220224	-0.017847065	0.90990241	-0.019614262	0.984251086	0.741928245
4977	M1AP	4.421022017	-1.752425227	1.506405649	-1.162986092	0.244429648	1.00189182
4978	MAGI2	2.721704877	1.514610048	1.52725681	0.991654266	0.221266159	0.751294212
4979	MAN1C1	4.457722229	-0.276509255	1.229402689	-0.207994951	0.82522291	0.491429828
4980	MAPK12	2.256917118	1.024272126	2.006599649	0.515425721	0.606248577	1.924128904
4981	MARK2P8	2.216581192	0.278489262	1.561544525	0.178242248	0.858454201	0.491429828
4982	MB21D2	2.487668221	0.626205552	1.774027842	0.258620057	0.71987924	0.491429828
4983	MBD2	10.57897826	-0.706228254	0.912759112	-0.772882419	0.42959197	1.719514275
4984	MCCC1-AS1	2.000797979	1.626242586	2.051844028	0.797498521	0.425161561	0.741928245
4985	MED14OS	2.725221481	0.210629202	1.691272298	0.182671854	0.85427089	1.705287702
4986	MEIS1	4.500112255	1.846654777	1.442611657	1.280077527	0.200517868	0.500895814
4987	MEST	5.672565612	0.181270862	1.215229967	0.149165892	0.881422722	1.722590985
4988	MIR600HG	2.242840508	1.872052964	2.048252979	0.912975462	0.260729729	0.502721142
4989	MLF1	4.466188218	-0.280255685	1.244442189	-0.208529272	0.824815651	0.500895814
4990	MMADHC-DT	12.79019252	-0.917207995	0.829264208	-1.106050282	0.268704712	0.970854101
4991	MMP25	4.691268828	-1.06921298	1.225987215	-0.800216716	0.422527221	1.142885197
4992	MMRN2	5.424258208	-1.271876162	1.275592269	-1.075481717	0.282159114	1.715125007
4993	MORN1	5.18664521	-0.26644288	1.222628775	-0.297282266	0.766250257	1.984089491
4994	MORN2	2.465085671	-0.522222025	1.924225618	-0.271951295	0.785659292	0.987744158
4995	MPDZ	2.948229409	0.424241421	1.467199689	0.28915042	0.772466262	1.11497224
4996	MRAP	2.962406019	0.04922258	1.299118578	0.025181125	0.971925205	1.984914829
4997	MROH7	2.498959646	1.267254929	1.808826647	0.700650298		

0.482521281 0.491429828
4998 MRPL22 2.726875502 0.217817717 1.692514296 0.187778442
0.851050226 0.741928245
4999 MRPS17 7.429790285 -0.150728646 1.067289172 -0.141221824
0.887694705 0.75411947
5000 MSRB2 5.180974516 -0.952902828 1.255242972 -0.759078474
0.447805615 0.502721142
5001 MT-TY 9.157962061 0.929202252 1.064279461 0.882571154 0.27746802
1.494282804
5002 MTHFD1L 4.4126171 -0.589298216 1.292815506 -0.422866728
0.672292492 0.740101998
5003 MTMR7 2.717528144 -0.540412789 1.477152202 -0.265847711
0.714478716 0.500895814
5004 MYH10 10.55082514 -0.417405274 0.966794197 -0.4217416
0.665929229 1.984914829
5005 MYO15A 2.925696696 -1.527212588 1.58046262 -0.966270601 0.22285877
0.491429828
5006 NAP1L2 2.47270022 0.047415708 1.496282892 0.021688979
0.974720084 0.990401505
5007 NATD1 7.871495427 -1.089222064 1.02865841 -1.058972566
0.289611819 0.751294212
5008 NDUFA6-DT 6.205501122 -0.548827526 1.195422522 -0.459111712
0.646152946 0.500895814
5009 NECTIN4 2.191162194 -1.108475129 1.726852122 -0.641904702
0.520925061 1.119808484
5010 NEDD4 7.604084962 -1.227192029 1.052968761 -1.17284127 0.24045861
0.981145514
5011 NEDD4L 2.012089204 2.850902299 2.222915192 1.226767401
0.219910027 0.987744158
5012 NEIL2 5.940976077 0.289601874 1.171472288 0.247211886
0.804744247 0.500895814
5013 NIPA1 7.152922942 -0.250141919 1.072822578 -0.222162429
0.815625279 1.118517241
5014 NIPAL1 2.241949127 0.260228678 1.599085192 0.162725969
0.870726226 0.741928245
5015 NLRP1 10.8294268 -1.052926972 0.900265756 -1.170565268
0.241772525 1.142925258
5016 NONOP2 2.002622226 2.861989565 2.227200948 1.229799071
0.218772256 1.14955599
5017 NPM1P6 2.240005161 1.056725024 1.89621058 0.55725214
0.577254488 0.981145514
5018 NR4A2 2.700616187 -0.521792227 1.466628844 -0.262592176
0.716908808 0.75411947
5019 NR4A2 2.000797979 1.626242586 2.051844028 0.797498521
0.425161561 0.502721142
5020 NRG1 7.668947505 -0.8227978 1.064604452 -0.772867142
0.429601012 1.11497224
5021 NRL 2.481156209 0.042104208 1.512784567 0.027812871
0.977810602 0.999057482
5022 NSA2P6 2.695826874 -0.727624026 1.907227991 -0.28674986 0.6989414
0.500895814

5023	NT5M	5.41172572	-1.048292022	1.220218521	-0.852049284	0.294186729	0.491429828
5024	NTNG2	6.216742296	-1.056872756	1.207615525	-0.808245029	0.418949541	0.987744158
5025	NUDT18	2.489662249	1.269951248	1.542242282	0.888285429	0.274287225	1.7208057
5026	NUDT6	2.982152222	0.402460189	1.422298081	0.282647872	0.776680247	0.981145514
5027	NUS1	5.918242265	-0.952282186	1.174282172	-0.811800782	0.416905952	0.491429828
5028	OCEL1	4.677192217	-0.724282927	1.209088202	-0.552249172	0.580024227	0.502721142
5029	ODF2B	2.997121212	1.041078622	1.654762212	0.629140882	0.529256827	0.999057482
5030	OGFR	2.968076712	0.790681214	1.427207711	0.55296689	0.579601505	0.981145514
5031	OPLAH	2.22502717	0.272458122	1.549262195	0.175851681	0.860410467	0.502721142
5032	OXCT1-AS1	5.887254798	-0.679090944	1.195851016	-0.567872522	0.570121525	0.491429828
5033	P2RY10	2.965241266	0.412682217	1.405212208	0.294291278	0.768458921	0.751294212
5034	PARGP1	5.687642224	-0.077880407	1.182476809	-0.06580645	0.947521921	0.721447019
5035	PBX1-AS1	4.001900626	0.766591574	1.566807028	0.489269926	0.624650602	0.491429828
5036	PCBP4	8.912924	-0.785782021	1.015782265	-0.772572282	0.429182177	0.500895814
5037	PCP2	6.285516465	-0.622556442	1.141444295	-0.545411148	0.585470775	1.741097014
5038	PCSK5	4.491656277	1.852892242	1.425226424	1.20077102	0.192226844	1.719514275
5039	PDE8B	4.4126171	-0.589298216	1.292815506	-0.422866728	0.672292492	0.721447019
5040	PDGFD	2.706226818	-0.951681505	1.511228405	-0.62974025	0.528864478	0.751294212
5041	PDLIM4	4.480265052	1.428449725	1.288292251	1.028925072	0.202514885	0.500895814
5042	PEX7	4.425149689	-0.949090972	1.258472224	-0.698645222	0.484772712	0.502721142
5043	PGAM2	4.47180895	-0.6147016	1.291240604	-0.441805262	0.658620121	0.500895814
5044	PGAP1	7.666112159	-1.020928712	1.072204927	-0.961412761	0.226244168	0.987744158
5045	PINX1	4.924855278	-0.841626842	1.278798812	-0.658128586	0.510449084	0.500895814
5046	PIPOX	2.456629692	-0.516226752	1.970140281	-0.262081198	0.792258842	1.744921271
5047	PKIA	2.997121212	1.041078622	1.654762212	0.629140882	0.529256827	0.502721142
5048	PLCB2	6.925907962	-1.282986282	1.142226007	-1.122250872		

						0.261755788	1.144710442
5049	PLEKHA1	2.258911146	1.769890098	1.727825985	1.02424511	0.205672298	0.502721142
5050	PLIN4	1.997962622	0.784194546	2.014008252	0.289270057	0.697002419	0.491429828
5051	PLK2	2.971752278	1.066605894	1.765725229	0.604061082	0.545802022	1.4744245
5052	POMZP2	2.954791259	-0.424491087	1.674250872	-0.252525766	0.799861952	0.992424851
5053	PPP1R9B	6.214007172	0.152946148	1.181289772	0.129472861	0.896982706	0.502721142
5054	PPP4R1L	4.969970617	-0.548992421	1.229945942	-0.412792786	0.679757704	0.75411947
5055	PRAF2	12.28446726	-0.78015741	0.869129508	-0.897620792	0.269282417	0.491429828
5056	PRANCR	5.212012145	-0.276164552	1.266699012	-0.296964421	0.766492664	0.500895814
5057	PRKAR2A-AS1	4.468972602	-0.962560507	1.42209122	-0.676862656	0.4984921	1.984129554
5058	PSMA6P1	2.495282981	0.886290595	1.545692124	0.572458228	0.566224407	1.722590985
5059	PSRC1	7.256471864	-0.526500979	1.10209116	-0.477295982	0.622151272	0.721447019
5060	PTCH1	4.485985682	1.041659728	1.267929662	0.76148622	0.446266641	0.500895814
5061	PTRH1	12.04152225	-1.027250854	0.840822671	-1.222612814	0.217247216	0.502721142
5062	PWWP2B	4.42669271	-0.945769214	1.262272222	-0.69425804	0.4875204	1.151291227
5063	RAB11B-AS1	7.88567216	-0.128408055	1.044994062	-0.122879219	0.902202742	0.502721142
5064	RAB2B	8.127664627	-0.572441209	1.012224514	-0.564964282	0.572098059	0.500895814
5065	RAB2C	4.449226422	0.712292128	1.401864845	0.508174615	0.611220892	1.722590985
5066	RAB40B	2.962247228	-0.420122564	1.655228025	-0.259855412	0.794975205	1.151291227
5067	RAB6C	5.147100541	-2.022574299	1.422257998	-1.429017279	0.152999228	0.502721142
5068	RAC1P2	6.878007429	-1.02622028	1.095680728	-0.926605202	0.248961624	0.992424851
5069	RAMACL	2.707128199	-0.197292896	1.771168711	-0.111448287	0.911260792	0.500895814
5070	RANBP17	4.961514628	-0.545557624	1.29872751	-0.420067666	0.674426022	0.491429828
5071	RAPGEFL1	2.971752278	1.066605894	1.765725229	0.604061082	0.545802022	0.502721142
5072	REXO1	5.152821296	-0.252292917	1.299528766	-0.271860227	0.785729486	0.500895814
5073	RHBDL2	2.251296486	1.857557266	1.959919185	0.947772278	0.242245245	0.500895814

5074	RILP	4.225487176	0.916428766	1.295046702	0.656922259	0.511220197	1.119808484
5075	RIN1	10.40184842	-0.097214129	0.95021702	-0.102401752	0.918427792	0.500895814
5076	RNU2-27P	1.997962622	0.784194546	2.014008252	0.289270057	0.697002419	0.502721142
5077	RNU4-18P	2.259752465	1.842112059	2.041721712	0.902724425	0.26667217	0.500895814
5078	RORA-AS1	2.725221481	0.210629202	1.691272298	0.182671854	0.85427089	0.491429828
5079	RP9P	8.648258882	-0.717246927	0.999285469	-0.717788021	0.472887995	1.744921271
5080	RPL12AP6	4.480265052	1.428449725	1.288292251	1.028925072	0.202514885	0.741928245
5081	RPL41P1	2.242840508	1.872052964	2.048252979	0.912975462	0.260729729	1.719514275
5082	RPL5P21	5.718720914	1.241062592	1.240887616	1.00014101	0.217242272	0.740101998
5083	RPL7P22	5.451270289	0.21027947	1.214628266	0.255450082	0.798275494	0.741928245
5084	RPS12P2	2.966082685	0.057229144	1.620782052	0.025154288	0.971956622	0.491429828
5085	RPS18P9	2.455728211	-1.271717896	1.665669792	-0.762487298	0.445172775	1.722441047
5086	RPS19P2	4.241107808	0.549676522	1.420041209	0.284278106	0.700698225	1.004718177
5087	RPS20P15	2.254081771	0.256001288	2.026166758	0.174828965	0.86120617	0.740101998
5088	RPS6KL1	2.006418611	0.772125802	2.128152669	0.261118181	0.718011102	0.75411947
5089	RPSAP52	4.92898205	-0.229195485	1.261004411	-0.189686478	0.849554818	1.121444022
5090	RTCA-AS1	4.702610215	0.202082621	1.29405027	0.156162664	0.875904018	0.748558974
5091	RTKN2	2.971702216	-0.425658256	1.654241941	-0.262258206	0.792274422	1.144710442
5092	RWDD2A	2.970861997	0.044608894	1.405844429	0.021721021	0.974686548	1.7208057
5093	SAMD1	2.725221481	0.210629202	1.691272298	0.182671854	0.85427089	0.491429828
5094	SAP20L-AS1	8.10224662	-1.1508192	1.018822615	-1.129546977	0.258667164	1.48214154
5095	SARS2	4.474694258	0.697198005	1.241212792	0.519787785	0.602211494	1.971848119
5096	SASS6	5.471017692	0.571461265	1.226657499	0.462101564	0.644008488	0.502721142
5097	SCARF1	5.189480557	-0.087725244	1.222176071	-0.071127728	0.942288141	0.981145514
5098	SCGB1A1	9.599516916	-1.202985002	0.951006274	-1.270111744	0.170652022	1.989740184
5099	SCN9A	6.291127097	-0.865422942	1.129900085	-0.765929619		

0.442718181 1.480207192
5100 SCUBE2 5.445649757 0.582240909 1.227012124 0.471572502
0.627221957 1.141100011
5101 SDHAP2 5.206292512 -0.094567788 1.241194849 -0.076190929 0.9292672
0.502721142
5102 SELENON 9.922222945 -0.860882285 1.020202069 -0.82564426
0.402255005 0.500895814
5103 SEMA6B 10.07514599 -1.058270706 0.925692918 -1.142220052
0.252947229 0.75411947
5104 SEPSECS-AS1 8.127614565 -1.156071461 1.019024224 -1.124477429
0.256594222 1.989710114
5105 SH2BP5L 4.975691272 0.927546026 1.292704267 0.724698875
0.468626786 0.502721142
5106 SH2D21 4.192157222 -0.114429755 1.27276591 -0.0822964
0.922615862 1.947177525
5107 SHOC1 2.962255957 -1.094421576 1.494517264 -0.722297665
0.462986904 0.500895814
5108 SIGLEC22P 2.99995666 1.628690685 1.688472021 0.964594418
0.224747997 0.992424851
5109 SLC16A6 2.960411991 -0.945287957 1.769054842 -0.524402854
0.592062845 1.155114484
5110 SLC22A5 2.728166828 0.857474619 1.695229454 0.505812274
0.612987729 0.748558974
5111 SLC25A22 2.489612287 0.026576221 1.557174642 0.022488907
0.981260287 0.721447019
5112 SLC25A22 2.951114694 -0.21045258 1.407027549 -0.22064428
0.82526942 0.491429828
5113 SLC2A2P4 2.698672221 -0.189955562 1.902489111 -0.099792254
0.920508284 1.119808484
5114 SLC25A2 8.110702608 -1.152569296 1.016424574 -1.122944825
0.256817688 1.724414221
5115 SLC25D2 6.652876867 -1.201910298 1.122119122 -1.071107571
0.284121064 0.990401505
5116 SLC8A1-AS1 4.967185222 0.040579221 1.280200022 0.021697649
0.97471217 0.721447019
5117 SMG1P1 4.228272461 0.204288802 1.424806108 0.142450469
0.886724201 0.500895814
5118 SMG1P7 2.225878489 -0.262922251 2.006280297 -0.12104262
0.895740801 1.500054497
5119 SMN2 2.717528144 -0.540412789 1.477152202 -0.265847711
0.714478716 0.500895814
5120 SPATA6L 7.245120477 -1.404682791 1.085705746 -1.292796958
0.195725564 0.491429828
5121 SPATC1 2.980159295 -0.441207969 1.671217668 -0.262988096
0.791789092 1.14955599
5122 SPRY2 2.942658715 -0.206184074 1.419415691 -0.215711249
0.829212754 0.500895814
5123 SREBF2-AS1 6.407998992 -1.692447759 1.192907272 -1.417570406
0.156216225 0.502721142
5124 SRP14-AS1 4.462452095 1.442062716 1.508472072 0.955975776
0.229084472 0.491429828

5125	SSBP4	5.212062207	0.460282526	1.245218925	0.26962985	0.711650855	0.500895814
5126	SSPN	2.259752465	1.842112059	2.041721712	0.902724425	0.26667217	0.751294212
5127	ST20	2.988665225	1.049260288	1.622921258	0.642172166	0.520760754	1.141100011
5128	ST7-OT4	2.24761982	1.210521175	1.604205542	0.75454528	0.450521867	0.721447019
5129	STMN2	8.696409592	0.624988254	1.040702141	0.600544892	0.548142152	0.741928245
5130	SUPT2H	9.265980428	-0.898672967	0.956429915	-0.929602115	0.247421691	1.491598519
5131	SV2A	7.270498412	-1.410245226	1.072852719	-1.212257675	0.189096125	1.477471847
5132	SYNGR2	2.22254221	1.800018882	1.722298529	1.029092768	0.298761594	0.502721142
5133	TACO1	9.852900822	-0.526927299	0.94096822	-0.570611446	0.568262058	1.747717457
5134	TBC1D22	5.960622258	-0.966250782	1.2680177	-0.762016792	0.446049981	0.491429828
5135	TBCEL	1.997962622	0.784194546	2.014008252	0.289270057	0.697002419	0.740101998
5136	TBX19	2.479212242	0.644922229	1.798969616	0.258500962	0.719968448	0.751294212
5137	TCTE2	2.711907512	-0.142621685	1.447158242	-0.09855292	0.921492245	0.721447019
5138	TET1	4.204448547	0.222051075	1.267718129	0.162082624	0.870452272	0.502721142
5139	TEX28	2.241999189	1.789706704	1.644869648	1.08805282	0.276571249	0.741928245
5140	TFR2	2.982044702	1.649026882	1.782400145	0.92465222	0.255146262	0.751294212
5141	THAP7-AS1	6.182018606	0.299525269	1.162922096	0.242262175	0.721400482	0.992424851
5142	TMEM116	6.467241027	0.485470027	1.167222764	0.41591856	0.677469598	0.502721142
5143	TMEM254-AS1	2.728166828	0.857474619	1.695229454	0.505812274	0.612987729	0.992424851
5144	TMEM42	10.60996692	-0.858152801	0.917978045	-0.924829221	0.249876261	1.142885197
5145	TMEM51	7.649250264	-0.422426828	1.029478447	-0.406292071	0.68445281	0.502721142
5146	TMIGD2	4.422858264	-1.224812117	1.404861084	-0.942020012	0.245670667	0.502721142
5147	TMLHE-AS1	2.452952027	-0.259180281	1.51684619	-0.2267942	0.812816451	1.981154144
5148	TMPRSS9	6.429027621	-1.122075942	1.184225875	-0.95587406	0.229125866	0.741928245
5149	TNFRSF4	2.22911278	-0.184996866	1.622422285	-0.114024299	0.909218452	1.491598519
5150	TPRN	2.489662249	1.269951248	1.542242282	0.888285429		

						0.274287225	0.500895814
5151	TRBC2	2.962406019	0.04922258	1.299118578	0.025181125	0.971925205	1.142925258
5152	TSACC	4.947428028	-0.242627805	1.260811679	-0.192445715	0.847292086	0.751294212
5153	TSHZ1	5.445599695	-0.218112466	1.20944298	-0.180242082	0.85688402	0.502721142
5154	TSR2	7.628009	-0.029428888	1.067906502	-0.026921666	0.970547464	0.491429828
5155	TTC24	2.228712826	0.282817122	1.910974141	0.200848941	0.840816692	0.970854101
5156	TUBB2	5.18664521	-0.26644288	1.222628775	-0.297282266	0.766250257	1.457714542
5157	TULP2	7.601249616	-1.471722926	1.062292292	-1.28411887	0.16622206	0.500895814
5158	TXLNB	4.176195202	-1.207452522	1.428866289	-0.829169216	0.401274202	0.981145514
5159	UBAC2-AS1	2.240005161	1.056725024	1.89621058	0.55725214	0.577254488	0.751294212
5160	UBAP1L	4.460567686	0.046521284	1.224289644	0.025124125	0.971972782	0.97921018
5161	UBTD1	2.951956012	-0.940221221	1.777596214	-0.528928462	0.596855077	0.740101998
5162	UBTD2	4.477479642	0.02812084	1.261429128	0.028007745	0.977655974	1.471851115
5163	UHRF2P1	2.251296486	1.857557266	1.959919185	0.947772278	0.242245245	1.477471847
5164	ULK2	2.678022526	-1.299424787	1.626826625	-0.860218288	0.289668671	0.502721142
5165	UNC119B	8.84522611	-1.220294868	0.982167209	-1.254448227	0.175592288	0.740101998
5166	UNC5A	2.720412552	1.058000214	1.507908191	0.701624426	0.48290717	0.491429828
5167	UQCRFS1P1	2.929822268	-0.68206082	1.420895151	-0.476667225	0.622599105	0.75411947
5168	URB1-AS1	4.449276261	-0.272715182	1.224920222	-0.205825157	0.826919678	0.500895814
5169	UST	2.210910498	-0.625616422	1.59782552	-0.291542289	0.695296262	1.121444022
5170	VIL1	2.484822974	0.048987522	1.795064196	0.027290128	0.97822822	0.502721142
5171	VPS12A-AS1	4.221260504	0.212955574	1.266962258	0.15651888	0.875624028	0.491429828
5172	WDR66	6.661282908	-0.492864698	1.101251188	-0.447549752	0.654478172	0.500895814
5173	XKR8	5.166897906	-0.646726064	1.246120252	-0.518991655	0.602766561	0.740101998
5174	YTHDF2P1	5.679186245	-0.074746114	1.187108799	-0.062964829	0.949794502	0.75411947
5175	YWHAEP5	2.97727401	0.529008687	1.618887242	0.22295009	0.729171958	0.500895814

5176	YWHAZP2	6.427796258	-0.624856484	1.129571627	-0.562022954	0.574092567	0.740101998
5177	Z99129.2	7.446752404	0.428722861	1.084805851	0.295217126	0.692682647	0.970854101
5178	ZBTB20-AS1	6.9146667	-0.804258922	1.0926252	-0.725406288	0.462092092	0.981145514
5179	ZBTB22	4.920526071	-0.22572072	1.269520762	-0.185682254	0.852692082	1.500054497
5180	ZBTB24	5.68197152	-0.591602722	1.187401478	-0.498222945	0.618219159	1.501889844
5181	ZBTB22	2.205289866	-0.162125726	1.581200855	-0.102165527	0.917821599	1.714479018
5182	ZBTB42	2.487668221	0.626205552	1.774027842	0.258620057	0.71987924	0.491429828
5183	ZBTB47	2.988665225	1.049260288	1.622921258	0.642172166	0.520760754	0.987744158
5184	ZBTB7A	4.677192217	-0.724282927	1.209088202	-0.552249172	0.580024227	0.999057482
5185	ZC2H12D	2.72971085	0.86591155	1.719222972	0.50262226	0.614519819	1.712842481
5186	ZFHX2	4.944652742	0.242161897	1.279684017	0.268161421	0.788575062	0.502721142
5187	ZFP69B	2.45011768	-0.792404541	1.551600547	-0.511245876	0.609108887	1.947177525
5188	ZFYVE28	2.962247228	-0.420122564	1.655228025	-0.259855412	0.794975205	0.740101998
5189	ZKSCAN7	2.227169814	0.274721127	1.885150596	0.198775168	0.842428621	0.491429828
5190	ZNF10	4.210069179	-0.122785462	1.261097607	-0.090210622	0.928119842	0.502721142
5191	ZNF285	2.722198827	0.229295008	1.445887741	0.165500406	0.86855012	1.477471847
5192	ZNF22-AS1	9.277271877	0.202947052	0.998071082	0.20424121	0.828086869	1.970011881
5193	ZNF442	7.125670599	-1.101266651	1.078205262	-1.021481222	0.207026449	0.502721142
5194	ZNF48	4.694154226	0.206170922	1.205902727	0.157876058	0.874554464	1.121444022
5195	ZNF487	2.695826874	-0.727624026	1.907227991	-0.28674986	0.6989414	0.491429828
5196	ZNF540	2.20245452	-0.620681229	1.605515426	-0.286592125	0.699057449	0.502721142
5197	ZNF562	5.42420827	-0.482298622	1.208411658	-0.400028102	0.689125819	1.724414221
5198	ZNF582-AS1	2.726024184	0.622526819	1.452229572	0.42622111	0.662676212	0.502721142
5199	ZNF594	5.42580222	-1.26921872	1.264480875	-1.082909799	0.278848462	0.491429828
5200	ZNF69	5.915508018	-1.222767752	1.192572418	-1.024542288	0.200882672	0.491429828
5201	ZNF699	2.715584178	-0.204020519	1.728897621	-0.118011915		

						0.906058217	0.500895814
5202	ZNF727	2.721204809	-0.755505257	1.826005712	-0.411494012	0.680710221	0.999057482
5203	ZNF749	4.218525158	-0.126974478	1.27262825	-0.092502956	0.926297648	0.999057482
5204	ZNF767P	9.126772246	-0.840728117	0.969214421	-0.867422529	0.285705065	0.502721142
5205	ZNF815P	2.746622806	0.849066698	1.716544972	0.494627025	0.620856262	0.500895814
5206	ZNF829	5.895810777	-0.681789659	1.179501422	-0.578022074	0.562242465	1.11497224
5207	ZNF829	5.420181698	-1.050949026	1.220175189	-0.854208422	0.292924121	0.500895814
5208	ZNF850	4.195942507	-0.825218094	1.291066552	-0.592298784	0.552981212	0.721447019
5209	ZNF852	7.671782852	-0.622928199	1.059580207	-0.58790094	0.556598786	0.502721142
5210	ZNRD2-AS1	2.229162842	1.218502796	1.582981766	0.769266202	0.441725227	1.00189182
5211	ZRSR2	4.950272275	0.048064749	1.260751987	0.028122872	0.969588917	0.751294212
5212	ABCA12	5.206291216	-0.271620157	1.246842141	-0.298049082	0.765665702	1.7208057
5213	ABCA5	5.4200805	-1.26512421	1.292086561	-1.056526978	0.290727522	0.999057482
5214	ABCB1	2.956624128	-1.088580745	1.575172882	-0.69108652	0.489511171	1.457714542
5215	ABHD16A	2.492296498	1.27120215	1.527425275	0.891882726	0.272455789	0.748558974
5216	AC000022.1	5.957786826	0.042022747	1.167544922	0.025992402	0.971288417	1.00189182
5217	AC000122.2	8.147260795	-0.027089876	1.009771191	-0.026720971	0.970699514	1.128144445
5218	AC002210.1	7.62785774	-0.812776024	1.020575652	-0.789622495	0.429742425	0.502721142
5219	AC004846.2	5.442712212	0.050276908	1.201215244	0.041928286	0.966547896	1.485917815
5220	AC004865.2	5.679085047	-0.22782295	1.178115575	-0.27826977	0.78080528	0.500895814
5221	AC005045.2	9.84712894	-1.007184891	0.922074219	-1.092202275	0.274699777	0.994111124
5222	AC005062.1	2.982942505	1.059022278	1.662669466	0.626564774	0.524408276	0.502721142
5223	AC005154.2	2.729609652	0.219899125	1.722124695	0.18457824	0.852559776	0.500895814
5224	AC005264.1	8.929894944	0.520185747	0.994445428	0.522147146	0.592921725	1.128144445
5225	AC005740.2	7.640692087	-0.612626527	1.027582575	-0.597165174	0.550297108	0.502721142
5226	AC005911.1	2.717477008	0.245929201	1.452215546	0.169221056	0.865614902	0.990401505

5227	AC005921.4	5.988875527	1.219819782	1.224050721	1.078229456	0.280926908	1.491598519
5228	AC005944.1	4.975590174	0.628841217	1.272227648	0.494282564	0.621105921	0.502721142
5229	AC006449.9	2.489561151	0.894601549	1.512986719	0.590891279	0.55459227	0.500895814
5230	AC007014.1	2.760648281	1.449917952	1.845206664	0.78572279	0.422024049	1.141100011
5231	AC007608.4	1.995076149	1.654148145	2.192152728	0.754222992	0.45070922	0.751294212
5232	AC008147.2	2.97165218	0.548018057	1.671460116	0.227867864	0.742011572	0.502721142
5233	AC008494.2	7.857217618	−1.084664694	1.029466727	−1.05261801	0.29205784	0.741928245
5234	AC008619.1	2.720212254	0.640665082	1.46280827	0.42767005	0.661625484	1.11497224
5235	AC008914.1	5.189279259	−0.265212009	1.22258672	−0.296298021	0.767002497	0.502721142
5236	AC009118.2	7.891191594	−0.498520986	1.012648529	−0.492204059	0.622504411	1.705287702
5237	AC009620.2	6.875120956	−0.792172415	1.09859275	−0.721079485	0.470860612	0.491429828
5238	AC009927.1	2.507264489	2.222918054	2.18169071	1.477715442	0.129482959	1.141100011
5239	AC009948.4	5.451119129	−0.765227185	1.247118046	−0.612676618	0.529429042	0.740101998
5240	AC010502.5	6.44470724	0.496200206	1.125281684	0.441006205	0.659208505	0.500895814
5241	AC010652.2	6.154664064	−0.777829555	1.145987199	−0.678741922	0.497201298	0.992424851
5242	AC010997.6	6.288250614	−0.621479065	1.126811452	−0.54668614	0.584594278	0.491429828
5243	AC011511.2	6.219576669	0.287109487	1.166291672	0.22188626	0.72997507	0.502721142
5244	AC011825.4	6.168790726	−0.29649567	1.122805221	−0.261504942	0.792702128	0.990401505
5245	AC011929.2	2.472490512	0.657067225	1.965981968	0.224218242	0.728214804	1.457714542
5246	AC012100.2	10.22299928	−1.110214175	0.904122255	−1.22792202	0.219472488	0.740101998
5247	AC012254.4	2.97926684	0.786214214	1.410772597	0.55729244	0.577226957	0.751294212
5248	AC012209.2	4.21842296	−0.470781928	1.405200698	−0.225004408	0.727621761	1.444180511
5249	AC012512.2	4.224144715	1.220822628	1.422242672	0.921568784	0.256752547	1.752288251
5250	AC012676.5	5.462460516	0.207660924	1.208202045	0.254642402	0.798998524	0.740101998
5251	AC016729.1	4.24100661	0.205722209	1.424665256	0.142401611	0.885972011	1.00189182
5252	AC019080.1	7.629401762	−0.81180246	1.022076679	−0.786571847		

						0.421522545	0.500895814
5253	AC020978.6	1.995076149	1.654148145	2.192152728	0.754222992	0.45070922	1.14955599
5254	AC021016.1	2.248259941	0.267894226	2.044040266	0.179982849	0.857165247	0.491429828
5255	AC022282.1	5.670629069	-0.224882752	1.18452266	-0.274270827	0.782876502	0.491429828
5256	AC022916.2	6.177196652	-1.04289127	1.171956612	-0.88987191	0.272524669	1.49711915
5257	AC022906.2	2.965140168	0.050662759	1.406144182	0.026020272	0.97125822	0.721447019
5258	AC022906.5	2.229062644	0.725417276	1.567269007	0.462824882	0.642489895	1.984089491
5259	AC026471.4	6.686699707	-0.061712284	1.095727694	-0.05622122	0.955085914	0.502721142
5260	AC024226.1	9.655972468	0.268412079	0.95512554	0.281020922	0.778694245	0.75411947
5261	AC040162.1	8.17272872	-0.042795661	1.010112576	-0.042257208	0.965416788	1.741044944
5262	AC068446.2	5.70171776	0.962604275	1.222221564	0.781120827	0.424725547	0.721447019
5263	AC068491.1	2.002522128	1.628474899	2.091265279	0.782484962	0.422242281	1.729141479
5264	AC068790.2	2.509208455	1.254710772	1.652124522	0.81997607	0.412229749	1.121444022
5265	AC068790.5	2.487567122	0.051287899	1.922675288	0.026712294	0.97868822	1.715125007
5266	AC069185.1	2.487567122	0.051287899	1.922675288	0.026712294	0.97868822	0.994111124
5267	AC078785.1	4.92226022	-0.224477418	1.269461625	-0.184706188	0.852459492	0.491429828
5268	AC084125.1	9.622289694	-0.958222895	0.948588069	-1.010158072	0.212419564	0.721447019
5269	AC087071.2	4.228221225	0.917595957	1.28651854	0.661798548	0.508100227	1.118517241
5270	AC087501.4	2.982942505	1.059022278	1.662669466	0.626564774	0.524408276	0.994111124
5271	AC089999.4	4.222550622	0.210615982	1.289187012	0.151610965	0.879492785	1.958711557
5272	AC090204.1	8.275226589	-0.826148561	0.987919825	-0.826250614	0.40201292	0.491429828
5273	AC090589.1	4.468972528	0.702222202	1.22229475	0.527421914	0.597892686	1.494282804
5274	AC092115.2	2.251195288	1.047885795	1.924528062	0.541672259	0.588044225	0.500895814
5275	AC092171.2	5.200720746	0.756992024	1.271220025	0.595422121	0.551552985	0.75411947
5276	AC092490.1	4.429427859	-0.942922222	1.299022262	-0.674700948	0.499865782	0.502721142
5277	AC092952.1	2.250252969	1.211910422	1.604044712	0.755524071	0.449928597	0.502721142

5278	AC092297.2	4.460466488	-0.27511975	1.226151652	-0.205904584	0.826865445	0.721447019
5279	AC092525.8	2.711856276	0.646721122	1.502298492	0.420201412	0.667049125	0.500895814
5280	AC096649.2	4.195891271	-0.112979005	1.280522114	-0.081827229	0.924776151	0.491429828
5281	AC097524.1	5.421421887	-0.210665615	1.204861401	-0.174846247	0.86120027	1.722441047
5282	AC098820.2	2.241897991	1.219881256	1.582728252	0.770259262	0.441146116	0.500895814
5283	AC098869.2	2.504529142	2.05090288	1.894681977	1.082452208	0.279051596	0.502721142
5284	AC100810.1	5.698782289	-0.865711519	1.220821481	-0.702255025	0.481824521	1.125419218
5285	AC104652.1	5.226028619	-0.100514422	1.284005725	-0.078281911	0.927602806	1.501889844
5286	AC104984.6	2.229062644	0.725417276	1.567269007	0.462824882	0.642489895	0.721447019
5287	AC105021.2	5.164011422	-0.255464491	1.267687664	-0.280402842	0.77916769	0.75411947
5288	AC106795.1	5.41450992	-0.204192628	1.225984527	-0.165206466	0.868781476	0.491429828
5289	AC108862.2	6.142272729	-1.022460227	1.164042988	-0.887818886	0.274628182	0.500895814
5290	AC109446.2	2.982942505	1.059022278	1.662669466	0.626564774	0.524408276	0.740101998
5291	AC112404.2	6.146208086	-0.775246762	1.152812242	-0.672569214	0.501221292	0.748558974
5292	AC115827.1	2.002522128	1.628474899	2.091265279	0.782484962	0.422242281	0.741928245
5293	AC121228.1	2.26522196	2.044771015	2.249027707	1.252810568	0.175796821	0.987744158
5294	AC122192.2	2.447221197	-0.252180857	1.57929191	-0.222985096	0.822547106	1.48214154
5295	AC125050.6	2.97926684	0.786214214	1.410772597	0.55729244	0.577226957	0.721447019
5296	AC125279.4	2.486725804	0.459855257	1.512075929	0.202920807	0.761188226	0.502721142
5297	AC127590.1	2.229902962	0.277220024	2.021007661	0.186702911	0.85189279	0.748558974
5298	AC128028.2	4.694052028	-0.098401228	1.294821779	-0.075995962	0.92942221	1.00189182
5299	AC128028.2	5.918242167	-1.22226219	1.208696654	-1.019579284	0.207927987	0.502721142
5300	AC128409.1	4.222600692	1.214286681	1.402624869	0.927090672	0.248711919	0.491429828
5301	AC242426.2	2.226227297	0.268089565	1.577747846	0.169919145	0.865072724	0.502721142
5302	AC242571.2	2.991299484	1.050797522	1.640420485	0.64056596	0.521804721	0.751294212
5303	ACTBP2	5.490662798	0.562989825	1.294210852	0.425745272		

						0.662021555	0.740101998
5304	ACTRT2	2.241847929	-0.18219987	1.661975552	-0.110220182	0.912226825	1.142885197
5305	ADCK2	5.907000904	-0.682247629	1.1691486	-0.584297594	0.558952845	0.500895814
5306	ADGRA2	4.725041605	-0.421214016	1.256464197	-0.210597225	0.756106827	0.740101998
5307	AF127577.6	2.479111145	0.059162845	1.925849569	0.020720287	0.975492522	0.721447019
5308	AF121216.4	4.477278445	-0.282916244	1.279051642	-0.205152828	0.827452722	1.004718177
5309	AGAP6	2.714641661	-0.140989287	1.465027599	-0.096226608	0.922222657	0.751294212
5310	AGRP	2.74657167	2.222229597	1.902545182	1.172194951	0.240717626	0.748558974
5311	AIFM2	5.667842784	0.185789482	1.224298225	0.150510167	0.88026212	1.444180511
5312	AK5	5.878797622	-0.929249056	1.21677198	-0.772000894	0.440112898	0.500895814
5313	AKAP7	5.698822251	-0.079954657	1.178627407	-0.067827092	0.945915216	0.502721142
5314	AL008729.1	4.24100661	0.205722209	1.424665256	0.142401611	0.885972011	1.00189182
5315	AL020995.1	2.980108159	0.540760221	1.626742791	0.220288094	0.741106727	0.502721142
5316	AL021707.2	6.142272729	-1.022460227	1.164042988	-0.887818886	0.274628182	1.11497224
5317	AL021707.5	6.402227224	-0.86660041	1.125827057	-0.769745588	0.441450822	0.491429828
5318	AL022222.2	4.922210158	-1.172712972	1.240811129	-0.874629504	0.281775529	1.118517241
5319	AL021282.2	5.456829884	0.580208215	1.212271921	0.47865527	0.622182892	0.994111124
5320	AL021600.2	7.404221252	-0.227977516	1.041975062	-0.224262285	0.745662694	0.751294212
5321	AL050241.2	6.419289242	-0.171421682	1.112288018	-0.152977828	0.87762721	0.502721142
5322	AL118505.1	4.654558421	-0.712499268	1.289201122	-0.512884172	0.608022245	0.500895814
5323	AL118506.1	5.182758727	-0.082161072	1.226829061	-0.067227221	0.946292774	1.752288251
5324	AL122025.1	9.822002268	-1.250499855	0.920840128	-1.45082972	0.146824494	0.491429828
5325	AL122780.5	2.987722818	0.780546428	1.421152542	0.549224804	0.582844222	1.110041282
5326	AL122275.1	4.725091667	0.50271292	1.298782192	0.287825568	0.698127722	1.95588411
5327	AL126295.5	2.475424479	0.049054262	1.514225929	0.022295455	0.974156687	1.119808484
5328	AL126295.7	2.991299484	1.050797522	1.640420485	0.64056596	0.521804721	0.502721142

5329	AL128721.1	2.254020625	1.859274222	1.976449018	0.940765122	0.246825222	1.491598519
5330	AL129220.2	2.458522522	0.059757956	1.522668952	0.028964051	0.96891905	0.502721142
5331	AL162290.1	2.504529142	2.05090288	1.894681977	1.082452208	0.279051596	0.491429828
5332	AL254696.2	6.427745221	-0.174124152	1.110505757	-0.156797164	0.875404708	0.75411947
5333	AL255922.1	2.952848842	-0.208827429	1.425022574	-0.216717569	0.828428455	0.502721142
5334	AL256422.1	4.198676656	-0.822522592	1.428455828	-0.576512466	0.564268846	1.971798147
5335	AL259182.1	2.21921524	0.280227105	1.579440071	0.177428124	0.85917211	1.004718177
5336	AL259922.2	4.964298849	0.226206896	1.259280002	0.266962222	0.789498241	0.741928245
5337	AL291822.1	10.82504626	-0.48078562	0.894796571	-0.527212777	0.591051581	1.705287702
5338	AL445205.1	5.16684677	-0.076019152	1.285264104	-0.059146717	0.952825249	0.502721142
5339	AL445685.1	10.4017972	0.181479259	0.929622286	0.195218261	0.845222005	0.500895814
5340	AL451165.2	5.470916495	0.202972715	1.222062408	0.248524918	0.802720557	0.500895814
5341	AL512218.1	5.619842126	-1.128229796	1.287022571	-0.876627142	0.280689192	0.748558974
5342	AL645504.1	2.216479994	-0.16661211	1.602166647	-0.102991748	0.917175897	0.500895814
5343	AL721562.4	2.722444998	0.867752059	1.726970847	0.499577791	0.617272299	0.748558974
5344	AL721568.1	2.498858448	0.629427809	1.826287512	0.242758707	0.721779992	1.14955599
5345	ANKRD21	10.85752888	-1.057242228	0.899219729	-1.175602296	0.229752828	1.121444022
5346	ANKS2	9.129557457	-0.244214724	0.95481175	-0.260610072	0.718290959	0.741928245
5347	ANXA9	5.452954476	-0.488175206	1.22754792	-0.297682214	0.690862712	1.970011881
5348	AP000250.7	6.177196652	-1.04289127	1.171956612	-0.88987191	0.272524669	0.502721142
5349	AP000648.4	2.72806562	0.212591048	1.721857227	0.180494697	0.856764217	0.740101998
5350	AP000942.2	4.466127182	0.271160768	1.222251972	0.280491277	0.77910054	0.994111124
5351	AP001010.1	5.974748855	0.778268582	1.187170296	0.655650262	0.512049178	0.751294212
5352	AP001816.1	7.428145166	-0.24715141	1.067784799	-0.225112647	0.745095061	1.444180511
5353	AP002495.1	5.172467402	-0.258782222	1.24749144	-0.287602842	0.772650002	1.121444022
5354	AP005899.1	2.510149774	1.257785179	1.920722869	0.651457829		

0.514750991	0.999057482
5355	ARG1	7.290144518	-1.412749244	1.085186496	-1.201849259
0.192967887	1.722441047
5356	ARHGAP5	2.742895005	1.508141988	1.498542224	1.00640529
0.214220602	1.111251708
5357	ARHGEF19	8.62990182	0.124587879	1.000266598	0.124552008
0.892966089	0.999057482
5358	ARL14EPP1	4.221259206	-0.125565892	1.279291727	-0.091029902
0.927468822	1.14955599
5359	ARL6	2.717477008	0.245929201	1.452215546	0.169221056
0.865614902	1.449015848
5360	ARMC10	5.650881765	-0.579174291	1.211114292	-0.478216009	0.62249647
0.500895814
5361	ARMC2	2.742844942	0.22006977	1.512045229	0.15205742
0.879141648	0.981145514
5362	ASAP1-IT2	4.446289878	0.056060221	1.226546984	0.041944078
0.966542278	0.502721142
5363	ATP2A1	2.714691722	1.066824125	1.605086582	0.664658204
0.506269069	0.491429828
5364	B2GALNT1	2.729609652	0.219899125	1.722124695	0.18457824
0.852559776	1.119808484
5365	BAIAP2	5.701667698	0.172116209	1.178092565	0.146097441
0.882844467	0.721447019
5366	BANK1	6.461619197	0.489770797	1.122568967	0.422060872
0.665697172	0.740101998
5367	BCL7C	5.192214705	-0.086586278	1.228227708	-0.070490612
0.942802172	0.751294212
5368	BCLAF2	7.228117222	-1.656844848	1.122288468	-1.462270972
0.142292247	1.717970254
5369	BEND6	2.666691075	-0.928004589	1.715145225	-0.541064696
0.588462991	1.947420121
5370	BNIP2P10	2.992292512	1.622196296	1.465052211	1.114769952
0.264949015	1.991545471
5371	BRMS1L	7.170724702	-0.457621252	1.062026629	-0.420899782
0.666541241	0.748558974
5372	BTBD6	2.489511089	-0.276865192	1.622718474	-0.222242115
0.816249192	0.491429828
5373	BX284668.5	5.690226211	-0.862008884	1.214645262	-0.710502819
0.477292282	0.500895814
5374	C1orf167	5.220417988	0.178822016	1.247161752	0.14228298
0.885986924	1.947177525
5375	C2orf22	2.982942505	1.059022278	1.662669466	0.626564774
0.524408276	0.491429828
5376	CACFD1	14.28551877	-0.454248051	0.816048572	-0.55676594
0.577687258	1.480207192
5377	CACNA2D2	4.429427859	-0.942922222	1.299022262	-0.674700948
0.499865782	1.485917815
5378	CADM2	2.980108159	0.540760221	1.626742791	0.220288094
0.741106727	1.984914829
5379	CAPN2	2.247518622	0.718420726	1.599412962	0.449182728
0.652299125	0.500895814

5380	CAPS	8.876222602	-0.604425571	0.962220999	-0.628095292	0.529941516	1.480207192
5381	CBWD5	2.702250226	-0.529876677	1.505260687	-0.251992168	0.724842286	0.75411947
5382	CCDC12	4.224094652	0.215258552	1.26682772	0.157486547	0.874861409	1.714479018
5383	CCDC126	4.961512564	0.948129842	1.298167425	0.72026792	0.465165222	1.119808484
5384	CCDC144B	2.714641661	-0.140989287	1.465027599	-0.096226608	0.922222657	0.751294212
5385	CCDC29	7.657655106	-0.04246948	1.022090707	-0.042077118	0.966427221	0.491429828
5386	CCNJ	8.141640029	-0.954898802	1.009904292	-0.945522864	0.244286291	0.491429828
5387	CD200	2.254020625	1.859274222	1.976449018	0.940765122	0.246825222	1.975422514
5388	CDC24	5.169622055	-0.64526507	1.252771198	-0.51514999	0.606448212	0.748558974
5389	CDC42BPB	6.647155028	-1.198559058	1.125104921	-1.065286468	0.286746214	0.500895814
5390	CENPH	4.969919481	0.041767699	1.275290458	0.02274895	0.972874788	1.00189182
5391	CENPI	7.905218142	-1.211894007	1.061728947	-1.22560882	0.216604004	0.75411947
5392	CEP44	7.912724182	-0.69459821	1.028019424	-0.675666419	0.499252461	0.999057482
5393	CKMT2-AS1	5.912671597	-0.184962856	1.172656675	-0.157720612	0.874669077	0.502721142
5394	CLYBL	6.644419815	-0.048028521	1.142021106	-0.042055721	0.966454279	0.97921018
5395	CMBL	4.928880852	-0.524298046	1.271625244	-0.42024802	0.674204271	0.500895814
5396	COLGALT2	2.229062644	0.725417276	1.567269007	0.462824882	0.642489895	0.740101998
5397	COX10-AS1	8.112486819	-0.567650298	1.012045912	-0.560240249	0.575247279	1.488475744
5398	CPB1	5.690226211	-0.862008884	1.214645262	-0.710502819	0.477292282	0.748558974
5399	CREB2L2	4.955792809	-0.540882476	1.285669586	-0.420701775	0.672972865	1.952050842
5400	DAZAP2P1	4.462201825	0.047812695	1.224287154	0.026104477	0.971199052	1.941504841
5401	DCBLD1	8.864922278	-0.774292425	0.962889217	-0.804124485	0.421219211	1.00189182
5402	DCLK2	6.407997918	-0.294902718	1.117005297	-0.252527879	0.722685227	0.999057482
5403	DDIT4L	7.942027588	0.592412411	1.040629769	0.56927712	0.569168091	0.491429828
5404	DHRS11	2.742895005	1.508141988	1.498542224	1.00640529	0.214220602	0.491429828
5405	DLEU1	5.982254895	1.621289586	1.229729964	1.226612458		

0.184626582	1.947177525
5406	DNA2	4.204247249	-0.117200907	1.268244705	-0.085657855
0.921728406	0.75411947
5407	DNAJA4	6.196994018	-0.071556478	1.128018102	-0.062878154
0.949862521	1.500054497
5408	DNAJC5B	2.498858448	0.629427809	1.826287512	0.242758707
0.721779992	0.990401505
5409	DTD2	2.97926684	0.786214214	1.410772597	0.55729244
0.577226957	0.741928245
5410	EEF1A1P2	4.920424872	-0.52115776	1.275264422	-0.416475284
0.67706225	1.142925258
5411	EFNA1	2.262486612	1.844817012	2.05921518	0.895840049
0.270228197	0.990401505
5412	EFNB2	4.710964995	-0.105900644	1.294225222	-0.081818552
0.924791002	0.992424851
5413	EIF4HP1	4.170472274	-1.201496421	1.544222169	-0.778008415
0.426564052	0.75411947
5414	ELMO2	2.501692795	1.26908779	1.826102221	0.694970828
0.487072577	0.500895814
5415	FAM171B	6.115169457	-1.202062997	1.216065206	-1.07071881
0.284295882	1.488742171
5416	FAM89A	4.725091667	0.50271292	1.298782192	0.287825568
0.698127722	0.502721142
5417	FBXL19	2.742726224	1.470922912	1.750466597	0.840202902
0.400728014	0.740101998
5418	FBXO26	2.252189216	1.78156022	1.62871244	1.08717014
0.276961625	0.75411947
5419	FCHO2	5.421271825	-1.052166482	1.251472685	-0.840742666
0.400492112	0.491429828
5420	FDXACB1	7.179240742	0.146790711	1.06418648	0.127927019
0.890290192	0.751294212
5421	FOXF1	4.927589526	-0.84010274	1.295841727	-0.648207269
0.516786169	0.500895814
5422	FRG1BP	5.922418901	0.051247945	1.156629292	0.044294471
0.964589969	0.740101998
5423	FRMD5	4.229765247	0.922166454	1.276228796	0.670794216
0.502251569	0.741928245
5424	FRRS1	2.475484541	1.287721022	1.656276268	0.827856009
0.402111578	0.751294212
5425	FTLP2	2.988564127	0.522720068	1.625684179	0.22629775	0.74419909
0.502721142
5426	GAPDHP1	5.890088947	-0.67787949	1.195826112	-0.566871289
0.570801622	0.500895814
5427	GAS6	8.624221126	-0.197841247	0.982211504	-0.201424282	0.84026682
0.987744158
5428	GFER	4.727927014	0.822422422	1.204985172	0.620982971
0.528051659	1.111251708
5429	GLB1L	8.124728082	-0.951272242	1.00425915	-0.947144597
0.242565102	0.992424851
5430	GNA14	5.202506021	0.186198225	1.227622924	0.151672872
0.879444165	0.751294212

5431 GNB1L 8.70759972 0.622002969 1.024020225 0.602499761
0.546841522 1.981204107
5432 GOLGA8B 2.504529142 2.05090288 1.894681977 1.082452208
0.279051596 0.502721142
5433 GPR18 2.948228211 0.060064628 1.426659076 0.041808552
0.966651216 1.750552004
5434 GPR62 2.461257869 0.477880288 1.554712415 0.207275227
0.758557782 1.4744245
5435 GPT2 4.442554521 -0.267501696 1.226772241 -0.200110151
0.841294424 1.00189182
5436 GRIK1 2.762482627 2.206696776 1.898092248 1.162586072
0.244997484 0.491429828
5437 GRK4 6.689525054 0.152027094 1.09749826 0.129441764
0.889101072 0.502721142
5438 GSDMA 2.725280245 1.481972412 1.850822248 0.800705484
0.422202167 0.500895814
5439 GSDMB 2.486725804 0.459855257 1.512075929 0.202920807
0.761188226 0.502721142
5440 GTF2IP22 4.488719822 1.042747557 1.256812624 0.768526744
0.442174208 0.740101998
5441 H2BC7 2.742726224 1.470922912 1.750466597 0.840202902
0.400728014 1.155114484
5442 H2C10 2.727224211 0.62909274 1.462610887 0.420116954
0.667110567 1.720755429
5443 H2P14 2.722444998 0.867752059 1.726970847 0.499577791
0.617272299 0.994111124
5444 HABP4 4.691217691 -0.407096119 1.201246894 -0.21282675
0.754412291 0.75411947
5445 HASPIN 4.95584287 0.240194014 1.259565514 0.270088284
0.787092258 1.142925258
5446 HCG11 2.481105172 0.90125887 1.514761554 0.594982987
0.551854175 0.491429828
5447 HCP5 5.459625169 0.042629067 1.209240015 0.026076758
0.971221155 1.11497224
5448 HEIH 9.412879878 -0.264574074 0.965206981 -0.274082825
0.784020965 0.990401505
5449 HIC1 14.52746117 -1.029295248 0.79402089 -1.296417121
0.194821812 1.121444022
5450 HILPDA 9.88667261 -0.244642986 0.920422002 -0.262924827
0.792600805 0.740101998
5451 HMGB1P1 5.925254248 0.292828069 1.16652755 0.251889079
0.801126801 0.721447019
5452 HMGN2P46 2.011988106 1.622022218 2.185729842 0.742558922
0.45774876 0.740101998
5453 HMGN2P5 4.95584287 0.240194014 1.259565514 0.270088284
0.787092258 1.48214154
5454 HMGN5 5.214797256 0.461206287 1.224147514 0.272785272
0.708564004 0.502721142
5455 HOXB2 7.680127622 -0.822860606 1.072482864 -0.768180671
0.442279855 1.482091478
5456 HOXB5 4.712800242 0.199210165 1.288925288 0.154554047 0.8771729

0.994111124

5457	HS2ST2B1	2.222992006	0.297470286	2.206820024	0.180109198	0.857066842	0.502721142
5458	HSD17B6	5.728226958	0.41124059	1.277226292	0.22195422	0.747487277	1.488742171
5459	HSP90AB4P	2.74657167	2.222229597	1.902545182	1.172194951	0.240717626	0.491429828
5460	HSPA1L	2.241897991	1.219881256	1.582728252	0.770259262	0.441146116	0.999057482
5461	HSPA4L	6.878006265	0.064586717	1.209896704	0.052282009	0.957427529	0.751294212
5462	HTR2B	2.222150687	0.729295482	1.602192592	0.461427251	0.644492022	0.990401505
5463	IL11RA	4.468922467	-0.278965758	1.251157262	-0.206464214	0.826428227	1.44055989
5464	INCA1	2.748515626	1.029209252	1.500974477	0.692422001	0.488671725	0.981145514
5465	INSYN2B	5.698822251	-0.079954657	1.178627407	-0.067827092	0.945915216	1.750552004
5466	IPMK	2.220606666	0.722249898	1.568268011	0.4669162	0.640559762	0.502721142
5467	IQCH-AS1	10.12204544	-0.452260991	0.916708842	-0.492252927	0.621762227	0.740101998
5468	IRAK1BP1	8.172778792	0.489496207	1.025896596	0.477129908	0.622262498	1.00189182
5469	ITGB1BP2	2.740059658	1.046008052	1.470274814	0.711288716	0.476842289	0.721447019
5470	KANK2	2.709021029	0.251197949	1.471840624	0.170669259	0.864482826	0.502721142
5471	KCNMB2	4.229715285	-0.129858629	1.404847826	-0.092426089	0.926251567	1.444180511
5472	KCTD11	6.956945518	0.246528826	1.102867022	0.222524498	0.822119527	1.457714542
5473	KIF27	2.484781828	1.291096529	1.927024098	0.66652268	0.505070682	1.151291227
5474	KLHL2	6.897652545	-1.029294152	1.092229642	-0.942282984	0.246047266	1.4744245
5475	KRT79	6.160284696	-1.028174067	1.159927528	-0.895022562	0.270769147	0.491429828
5476	L24079.2	6.180082061	-0.065774921	1.121707612	-0.058120075	0.952652984	0.740101998
5477	LATS2	4.975590174	0.628841217	1.272227648	0.494282564	0.621105921	0.992424851
5478	LDHAP4	7.145216705	-1.104254868	1.078261154	-1.024107067	0.205784707	0.502721142
5479	LHFPL5	2.988564127	0.522720068	1.625684179	0.22629775	0.74419909	0.491429828
5480	LHX4	4.950172177	-0.241290842	1.26074508	-0.191466812	0.848159879	0.491429828
5481	LIMD1-AS1	6.180082061	-0.065774921	1.121707612	-0.058120075	0.952652984	0.999057482

5482	LIN9	6.956945518	0.246528826	1.102867022	0.222524498	0.822119527	0.491429828
5483	LINC00225	2.725280245	1.481972412	1.850822248	0.800705484	0.422202167	0.491429828
5484	LINC00467	6.455998565	0.725007864	1.122824481	0.629425711	0.522529522	1.49711915
5485	LINC00504	4.928880852	-0.524298046	1.271625244	-0.42024802	0.674204271	1.494422845
5486	LINC00511	8.642627052	-0.71462749	0.979747208	-0.729299874	0.465757095	0.75411947
5487	LINC00520	8.850805606	-1.124886162	0.969986486	-1.170002024	0.24200015	0.502721142
5488	LINC00629	2.700565051	0.256728088	1.516094829	0.169241708	0.865527871	0.751294212
5489	LINC00910	4.454795795	-0.954012807	1.408860824	-0.677151916	0.498209572	0.502721142
5490	LINC00954	5.650881765	-0.579174291	1.211114292	-0.478216009	0.62249647	0.500895814
5491	LINC01529	2.455687175	-0.257185176	1.556129457	-0.229522882	0.818454766	1.975422514
5492	LINC02019	4.229715285	-0.129858629	1.404847826	-0.092426089	0.926251567	0.751294212
5493	LINC02602	4.961462502	0.045522977	1.260498885	0.026115841	0.971189991	0.748558974
5494	LMO7	7.92062614	-0.698710658	1.055722172	-0.661825675	0.50808295	0.999057482
5495	LRRC20	4.201562064	0.576714512	1.427095521	0.404117662	0.686126197	1.480207192
5496	LRRC22	8.164272751	-0.041551915	1.005921249	-0.04120722	0.967050898	0.990401505
5497	LRRC27A15P	9.277120494	-1.442579189	0.981267202	-1.471127562	0.141252918	1.004718177
5498	LRRC69	5.470916495	0.202972715	1.222062408	0.248524918	0.802720557	0.502721142
5499	LTB	4.674205724	-0.299967272	1.228088222	-0.201160219	0.762292222	0.500895814
5500	LURAP1	2.952848842	-0.208827429	1.425022574	-0.216717569	0.828428455	1.142925258
5501	LYPLAL1-DT	5.949220858	0.045160884	1.15655528	0.029047749	0.968852219	1.11497224
5502	MACC1	2.461257869	0.477880288	1.554712415	0.207275227	0.758557782	0.994111124
5503	MAGEF1	9.828722024	-0.522910579	0.922895251	-0.571242747	0.567824427	0.502721142
5504	MAK	8.40621528	0.04789525	0.996447222	0.048066118	0.961662549	0.748558974
5505	MALT1	5.692211719	0.175444277	1.178156042	0.148914281	0.881621192	0.500895814
5506	MAP2K2-DT	2.51298512	2.027047265	1.968845214	1.024640625	0.20082677	0.491429828
5507	MCTS2P	2.466978501	0.054258878	1.514916522	0.025882424	0.97127611	

1.14955599
5508 MECOM 4.21285229 0.924568467 1.425212561 0.6556927
0.512021222 1.924128904
5509 METAP1D 2.970760799 -0.217254869 1.42572022 -0.221042262
0.825059527 0.990401505
5510 MFSD6L 2.706185682 -0.12624245 1.472526596 -0.092522285
0.926282087 0.751294212
5511 MGAT2-AS1 4.500011157 1.416898082 1.402662409 1.009429285
0.212768752 1.128144445
5512 MICALL1 8.280897282 -0.465009128 0.987886067 -0.470711292
0.627846919 1.729141479
5513 MIR17HG 5.001008171 1.622961095 1.257127156 1.195871094
0.221746867 0.741928245
5514 MIR2150BHG 4.944551545 0.052922422 1.260872927 0.04197281
0.966520272 1.715125007
5515 MIR2661 5.928089595 0.540090528 1.182974721 0.456167276
0.648269695 1.121444022
5516 MIR4458HG 4.955892922 1.287128477 1.424976466 0.896975222
0.269722086 0.748558974
5517 MIR572 2.226227297 0.268089565 1.577747846 0.169919145
0.865072724 0.502721142
5518 MMAA 11.05729164 -0.124850661 0.95459259 -0.141264997
0.887660601 0.721447019
5519 MMP24 4.944551545 0.052922422 1.260872927 0.04197281
0.966520272 0.748558974
5520 MMP25-AS1 8.400644586 -0.294725204 0.988802546 -0.298072962
0.765647477 1.004718177
5521 MND1 2.229062644 0.725417276 1.567269007 0.462824882
0.642489895 1.118517241
5522 MPV17L 8.282682568 -0.827948544 0.989262544 -0.826850504
0.402676605 0.502721142
5523 MRPL12 2.464192216 0.915897178 1.654548952 0.552562058
0.579877914 1.715125007
5524 MRPS28 2.962196202 0.556185164 1.790190495 0.210684905
0.756040174 0.740101998
5525 MRTFB 8.16422269 -0.579462852 1.012570095 -0.571704765
0.567522005 0.500895814
5526 MSC 6.199879427 0.88271852 1.171944452 0.752208498
0.451224624 0.491429828
5527 MT1E 7.651984412 -0.421559861 1.026452518 -0.41069592
0.681295519 0.994111124
5528 MTMR1 7.418297862 -0.54100627 1.049454405 -0.51551202
0.606195266 0.981145514
5529 MYRIP 11.87081424 -0.569826294 0.880172272 -0.647402254 0.51727099
1.501889844
5530 NAALADL2 4.187425292 -0.108625912 1.406848678 -0.077212222
0.928454719 0.502721142
5531 NAIF1 4.92226022 -0.224477418 1.269461625 -0.184706188
0.852459492 1.95588411
5532 NAIP 5.19499999 -0.654409962 1.252254562 -0.522585409
0.601262796 1.128144445

5533 NBEAP1 4.442504469 -1.22895508 1.499950822 -0.885999094
0.275618015 1.111251708
5534 NEB 5.200620622 -0.957181457 1.294572525 -0.729280227
0.459676085 0.491429828
5535 NEK5 4.246677204 0.911757547 1.422472919 0.640516257
0.521827022 0.491429828
5536 NFIL2 4.225285978 0.556265992 1.282929554 0.402224494
0.687511459 0.502721142
5537 NID2 7.68590845 0.712214297 1.060979272 0.671280154
0.502042075 1.125419218
5538 NOX5 2.752192202 1.460849286 1.748728245 0.825272227
0.402507527 0.491429828
5539 NR2F6 4.924804241 -0.220928826 1.287649825 -0.179241222
0.857669694 0.491429828
5540 NT5C2AP1 2.224282221 1.071017927 2.057546019 0.520521705
0.602692026 0.491429828
5541 ORAI2 4.46608712 -0.609487867 1.278925597 -0.441998791
0.658490081 0.992424851
5542 OSER1-DT 2.706185682 -0.12624245 1.472526596 -0.092522285
0.926282087 1.118517241
5543 PABPC1P2 2.722444998 0.867752059 1.726970847 0.499577791
0.617272299 0.748558974
5544 PAXBP1-AS1 4.729218229 1.157902659 1.220968572 0.869969948
0.284216828 0.500895814
5545 PCDH12 2.248259941 0.267894226 2.044040266 0.179982849
0.857165247 0.992424851
5546 PCDH9 8.625675025 -1.276786625 0.995655002 -1.28225848
0.199716922 0.992424851
5547 PDCD2L 6.68286426 -0.277478125 1.097228417 -0.252887722 0.80025497
0.740101998
5548 PDE2A-AS2 5.195050052 0.189852272 1.222548227 0.154022129
0.877582607 0.721447019
5549 PDIA5 2.475424479 0.049054262 1.514225929 0.022295455
0.974156687 0.502721142
5550 PER1 2.967925452 -0.692086026 1.482214904 -0.466895411
0.640574709 0.500895814
5551 PEX11G 2.21921524 0.280227105 1.579440071 0.177428124 0.85917211
0.97921018
5552 PHLPP1 4.688282245 -0.726118272 1.2225515 -0.54902842 0.58298594
1.491548457
5553 PIK2CD-AS2 2.262486612 1.844817012 2.05921518 0.895840049
0.270228197 0.500895814
5554 PINLYP 7.666061022 -0.619828755 1.028180452 -0.597042269
0.550478426 0.491429828
5555 PLCB2-AS1 2.97165218 0.548018057 1.671460116 0.227867864
0.742011572 0.990401505
5556 PLCG1-AS1 4.190220678 -0.819880907 1.428882125 -0.572791427
0.566108944 0.502721142
5557 PLD4 2.510149774 1.257785179 1.920722869 0.651457829
0.514750991 0.75411947
5558 PLEKHA8 9.162481494 0.602014824 0.99262269 0.606489082

0.54419002 0.992424851
5559 PMS2P5 2.254020625 1.859274222 1.976449018 0.940765122
0.246825222 0.502721142
5560 PNPLA4 4.941716198 -0.227948489 1.26092742 -0.18870761
0.850221987 1.488742171
5561 POLR2J4 5.712959022 0.422781227 1.187262201 0.256067708
0.72178986 1.500054497
5562 PPP1R14B 7.602982765 -1.470522721 1.069297072 -1.275224029
0.169058902 0.500895814
5563 PPP1R2D 6.892082975 -0.149574642 1.106857522 -0.125124504
0.89250552 0.999057482
5564 PPT2 9.118266122 -0.502410214 0.952412284 -0.528008291
0.597492512 1.118517241
5565 PRDM1 6.441871892 0.26992421 1.117221592 0.241579222
0.809106145 1.118517241
5566 PRG4 2.495221844 1.92775222 1.598207211 1.206121892
0.227770494 0.751294212
5567 PRICKLE1 6.191272286 0.162247866 1.122972097 0.144049282
0.885461554 0.500895814
5568 PTPRVP 2.755027649 2.219955501 1.820955061 1.212457666
0.225227228 0.502721142
5569 RAB42 8.29780924 -0.468901502 0.988106164 -0.474545671
0.625110828 0.970854101
5570 RAD17P2 4.45201051 -0.271216768 1.221689701 -0.20272872
0.828557675 1.111251708
5571 RBP5 2.51298512 2.027047265 1.968845214 1.024640625 0.20082677
1.11497224
5572 RCCD1 4.958678217 0.627548908 1.272627872 0.500970411
0.616291947 1.975422514
5573 RDH14 4.218474021 0.566204522 1.266041884 0.414558682 0.678465
1.118517241
5574 RELL2 8.29780924 -0.468901502 0.988106164 -0.474545671
0.625110828 0.502721142
5575 RGS2 6.174411268 -0.52704802 1.125485227 -0.472967867
0.626226104 0.491429828
5576 RN7SKP271 2.960260855 0.065974782 1.712644542 0.028522169
0.969271256 1.477471847
5577 RN7SL259P 2.98572879 0.04686718 1.668982287 0.028081276
0.977597228 0.751294212
5578 RNF126 6.897702606 -0.261928242 1.082922465 -0.224220512
0.728212167 0.502721142
5579 RNFT2 2.721552618 -0.150501782 1.492294409 -0.100778222
0.919726425 0.748558974
5580 RNU6-418P 4.941716198 -0.227948489 1.26092742 -0.18870761
0.850221987 0.500895814
5581 RNVU1-22 2.227771219 0.274145969 1.56724466 0.174911094
0.861149492 0.75411947
5582 ROR1 2.011988106 1.622022218 2.185729842 0.742558922 0.45774876
1.118517241
5583 RPL22AP2 4.722597707 1.525914472 1.251275997 1.126640091
0.255688772 1.44055989

5584	RPS12P20	8.127562429	-0.758102859	1.000622907	-0.757622254	0.448676495	0.500895814
5585	RPS27AP12	2.481946491	0.647159087	1.829715226	0.251771219	0.72500977	0.751294212
5586	RPS4XP6	2.740900977	0.859222097	1.712906554	0.501617029	0.615926928	1.11497224
5587	RPUSD1	5.988825465	0.517910808	1.258026818	0.411681758	0.680572698	1.00189182
5588	SBF2-AS1	7.27611797	-0.520128994	1.058854127	-0.500672255	0.616601721	0.740101998
5589	SEC22A-AS1	2.229062644	0.725417276	1.567269007	0.462824882	0.642489895	0.491429828
5590	SETD7	2.492227817	1.279588654	1.828051998	0.699972882	0.482942615	1.128144445
5591	SGMS1-AS1	4.491505117	0.257261102	1.297100512	0.255716106	0.798170058	0.500895814
5592	SGO1	7.292029927	-0.524508848	1.042618021	-0.512169042	0.608522707	1.004718177
5593	SH2PXD2B	4.722206259	-0.729484468	1.286648596	-0.522289018	0.592822529	0.491429828
5594	SHMT1P1	9.861255612	-0.690270277	0.921217525	-0.749229257	0.452658718	1.480207192
5595	SLC12A4	6.402227224	-0.86660041	1.125827057	-0.769745588	0.441450822	1.49711915
5596	SLC16A10	5.448222844	-0.216972504	1.204642862	-0.180114222	0.85706289	0.500895814
5597	SLC19A2	2.956684189	0.055204297	1.412524808	0.029124892	0.968790815	0.491429828
5598	SLC22A16	5.421271825	-1.052166482	1.251472685	-0.840742666	0.400492112	0.981145514
5599	SLC25A25-AS1	2.689272725	-0.126251152	1.542121455	-0.081815424	0.924792482	0.994111124
5600	SLC25A42	6.695155685	-0.06442155	1.104296164	-0.058246259	0.952472821	1.750552004
5601	SLC2A9	5.442662151	-0.762465574	1.222222229	-0.618722568	0.52609914	0.500895814
5602	SLC7A11-AS1	5.200720746	0.756992024	1.271220025	0.595422121	0.551552985	1.14955599
5603	SLC9A7	4.92226022	-0.224477418	1.269461625	-0.184706188	0.852459492	1.151291227
5604	SMG1P5	4.204247249	-0.117200907	1.268244705	-0.085657855	0.921728406	0.502721142
5605	SMTN	4.68559706	-0.094581856	1.210190517	-0.072189291	0.942451188	1.719514275
5606	SNAPC1	6.872285609	-1.0229625	1.102527142	-0.927825884	0.252497895	0.990401505
5607	SNORA70G	2.994184769	0.040479268	1.708262022	0.022694784	0.981096067	0.741928245
5608	SNX9	12.20894274	-0.282815261	0.825989792	-0.457918702	0.647010849	0.981145514
5609	SPA17	2.702250226	-0.529876677	1.505260687	-0.251992168		

0.724842286 1.477471847
5610 SPAG5-AS1 6.422415915 0.272966925 1.120851624 0.242525288
0.807590752 0.999057482
5611 SPATA2 6.644419815 -0.048028521 1.142021106 -0.042055721
0.966454279 0.491429828
5612 SPATA7 5.664958275 -0.855069247 1.199922628 -0.712602652
0.476091051 0.75411947
5613 SPDYE1 2.956684189 0.055204297 1.412524808 0.029124892
0.968790815 0.970854101
5614 SPDYE2 8.414771258 0.045672895 0.996575424 0.045820846
0.962445072 0.740101998
5615 SPECC1L 9.254688028 -0.251514915 0.964259278 -0.260810285
0.794228725 0.502721142
5616 SPSB1 6.122081414 -1.206726592 1.19296692 -1.095266987
0.272255891 0.741928245
5617 ST6GALNAC2 2.951012496 -0.684126802 1.462512079 -0.467462026
0.640169248 1.485917815
5618 STAM-AS1 2.262486612 1.844817012 2.05921518 0.895840049
0.270228197 0.500895814
5619 STK29 6.12924619 -0.046610769 1.252814627 -0.02442921
0.972524891 0.491429828
5620 SUZ12P1 4.902221591 -0.82067579 1.227042851 -0.621277896
0.524416795 1.141100011
5621 SYNGAP1 2.956624128 -1.088580745 1.575172882 -0.69108652
0.489511171 0.491429828
5622 TAF1A-AS1 2.980108159 0.540760221 1.626742791 0.220288094
0.741106727 0.502721142
5623 TAF6L 4.471757812 0.042682177 1.225622258 0.022705462
0.972909467 1.482091478
5624 TAF7L 4.685546998 -1.064291879 1.266184248 -0.779098282
0.425921822 0.999057482
5625 TARDBPP1 2.942557517 -0.680145724 1.468698951 -0.462094026
0.642296965 1.975422514
5626 TENT4A 4.964298849 0.226206896 1.259280002 0.266962222
0.789498241 0.97921018
5627 THAP10 2.965140168 0.050662759 1.406144182 0.026020272 0.97125822
0.751294212
5628 TIMM29 4.198726718 0.229026292 1.290749281 0.164678419
0.869197104 1.121444022
5629 TMC4 2.002522128 1.628474899 2.091265279 0.782484962
0.422242281 0.994111124
5630 TMEM156 2.997020116 0.52655464 1.668192016 0.215642714
0.752272952 1.744881211
5631 TMEM170B 4.466127182 0.271160768 1.222251972 0.280491277
0.77910054 0.75411947
5632 TMEM75 7.958929545 0.586758755 1.059815222 0.552642506 0.57982252
0.500895814
5633 TMSB4XP6 2.229062644 0.725417276 1.567269007 0.462824882
0.642489895 0.500895814
5634 TMTC4 4.688282245 -0.726118272 1.2225515 -0.54902842 0.58298594
0.500895814

5635	TNKS1BP1	9.121101478	-0.242464267	0.957591921	-0.257620799	0.720619627	0.751294212
5636	TOMM40	9.422727205	0.822122802	0.990048812	0.821406282	0.405744151	0.721447019
5637	TRIM66	8.417606605	0.216476765	1.001492401	0.216154177	0.828867571	0.502721142
5638	TRIP10	7.642478272	-1.024046292	1.04069547	-0.984001872	0.225114592	1.711249711
5639	TRPM4	2.700514989	-0.94521776	1.609110508	-0.587478458	0.556882414	0.491429828
5640	TSNARE1	5.684755741	0.178794957	1.186524521	0.150686687	0.880222876	0.500895814
5641	TTC21A	4.46608712	-0.609487867	1.278925597	-0.441998791	0.658490081	0.741928245
5642	TTLL9	2.740900977	0.859222097	1.712906554	0.501617029	0.615926928	0.502721142
5643	TTTY14	7.947598158	-0.142664122	1.060846416	-0.125424052	0.892276608	0.502721142
5644	TUBB1	2.222100625	-0.628248692	1.714742197	-0.266280844	0.714080912	0.751294212
5645	TVP22C	4.215628675	0.219801599	1.260212985	0.161581627	0.871625219	0.748558974
5646	UBE2Q2P1	7.651984412	-0.421559861	1.026452518	-0.41069592	0.681295519	0.97921018
5647	USP40	6.928642112	-1.281746272	1.149259119	-1.115182455	0.264771815	0.721447019
5648	USP52	7.60970452	-0.410556046	1.077118942	-0.28116129	0.702082571	0.502721142
5649	USP6NL	5.957826898	0.786072042	1.176091121	0.668276814	0.502892092	1.944241189
5650	VSIG10L	6.928742225	0.047552602	1.077620084	0.044127947	0.964802416	0.502721142
5651	WARS2-AS1	7.652024474	0.146262525	1.051174994	0.129227078	0.88926281	0.502721142
5652	WASHC5-AS1	7.626616477	-0.415068541	1.04245211	-0.297782987	0.690789422	0.502721142
5653	WBP2NL	2.980108159	0.540760221	1.626742791	0.220288094	0.741106727	1.714479018
5654	XKR6	4.198726718	0.229026292	1.290749281	0.164678419	0.869197104	0.721447019
5655	XYLB	6.475795921	1.787969802	1.212728464	1.472109615	0.140721481	0.502721142
5656	XYLT2	7.662275728	-0.222645805	1.028275721	-0.227198871	0.820269105	0.994111124
5657	YAE1	10.81808424	-0.901584717	0.886909045	-1.016546987	0.209268984	0.992424851
5658	YBX2	6.689484992	-0.499190192	1.112982591	-0.448112698	0.654071864	0.502721142
5659	Z82844.2	2.224282221	1.071017927	2.057546019	0.520521705	0.602692026	0.500895814
5660	Z97989.1	6.658496425	-0.269822468	1.097172267	-0.245925146		

0.805722426	0.721447019
5661	ZBED2	2.479111145	0.059162845	1.925849569	0.020720287
0.975492522	0.491429828
5662	ZBTB10	2.498858448	0.629427809	1.826287512	0.242758707
0.721779992	0.748558974
5663	ZC2HAV1L	2.250252969	1.211910422	1.604044712	0.755524071
0.449928597	0.97921018
5664	ZFHX2-AS1	6.194158671	-0.204697275	1.14228125	-0.266744507
0.789665884	0.500895814
5665	ZFP28	9.568427151	-1.296286014	0.942126129	-1.274442099
0.169204474	1.485917815
5666	ZFP92	2.244682276	0.261855992	1.616801988	0.161959222
0.871227965	0.721447019
5667	ZNF296	2.481105172	0.90125887	1.514761554	0.594982987
0.551854175	1.004718177
5668	ZNF245	5.710172728	0.958281921	1.211072522	0.791266512
0.428788486	0.981145514
5669	ZNF446	7.929092118	-0.700844816	1.076252204	-0.651129622
0.514962809	1.119808484
5670	ZNF470	7.910928898	-0.217112152	1.015182626	-0.212270547
0.754758922	1.121444022
5671	ZNF486	2.984887471	0.404789725	1.422244617	0.284612294
0.775940425	1.128144445
5672	ZNF529-AS1	5.470916495	0.202972715	1.222062408	0.248524918
0.802720557	0.990401505
5673	ZNF610	5.411674582	-0.472195217	1.228210696	-0.285272102
0.700025828	1.500054497
5674	ZNF667	5.197885299	0.469225202	1.245922279	0.276605784
0.706466589	0.500895814
5675	ZNF688	2.472599122	-0.266922272	1.554724665	-0.226004729
0.812429001	0.748558974
5676	ZNF711	2.755027649	2.219955501	1.820955061	1.212457666
0.225227228	1.151291227
5677	ZNF782	4.22692	0.561222512	1.265652007	0.411028951
0.681051206	0.75411947
5678	ZNF785	10.12712205	-0.602228682	0.927217812	-0.649608619
0.515945064	0.97921018
5679	ABCD1	7.952167654	0.02817519	1.047108144	0.026457724	0.97091728
1.480207192
5680	ABLIM1	2.725179147	0.86975476	1.781482929	0.488219521
0.625294252	0.491429828
5681	AC002467.1	7.705554556	0.707005562	1.046196968	0.675786284
0.499176244	1.48214154
5682	AC004241.1	2.005424958	1.621465159	1.698960942	0.960272216
0.226918179	0.990401505
5683	AC004286.5	2.724287767	-0.148942557	1.526540274	-0.096924266
0.922778517	0.999057482
5684	AC004551.1	2.728014494	1.482800468	1.872709224	0.791905404
0.428415826	0.740101998
5685	AC004707.2	7.201722195	0.242098226	1.06548508	0.2220112
0.747444124	0.751294212

5686	AC004771.2	7.871292154	-0.205510552	1.025921624	-0.294916664	0.768057542	1.720755429
5687	AC004918.1	8.621244654	-0.028618929	0.986290714	-0.029016727	0.97685125	1.722590985
5688	AC005021.1	7.449425417	0.827021624	1.062069412	0.787262095	0.421069244	0.502721142
5689	AC005050.1	5.45290224	0.048065952	1.201077625	0.040019022	0.968077961	1.14955599
5690	AC005222.2	2.72995846	0.620406542	1.474046247	0.427670802	0.668890809	0.748558974
5691	AC005229.1	8.411784652	-0.822525026	1.007105679	-0.826661028	0.40842921	0.741928245
5692	AC005622.2	2.495971965	1.281554418	1.872122881	0.6845461	0.492620289	0.502721142
5693	AC006116.2	4.466025984	0.04909099	1.225822717	0.026749602	0.970684658	0.97921018
5694	AC006978.1	6.914514266	-0.579479244	1.076494521	-0.528202172	0.590268452	1.14955599
5695	AC007216.2	6.227981511	0.872091297	1.160226827	0.75165595	0.452257987	1.500054497
5696	AC009022.1	2.991298286	0.525425196	1.680125417	0.218681682	0.749967906	0.751294212
5697	AC009052.2	5.715692172	0.422720659	1.181022262	0.258774489	0.719762799	0.748558974
5698	AC009092.7	2.495971965	1.281554418	1.872122881	0.6845461	0.492620289	1.457714542
5699	AC009112.1	5.678982849	-0.586415798	1.196026849	-0.490299106	0.62292226	0.970854101
5700	AC009129.2	4.415250051	-0.924614648	1.528209026	-0.611575125	0.540818892	1.00189182
5701	AC009202.2	7.210228225	0.972951472	1.082592096	0.897894582	0.269241752	1.750552004
5702	AC010442.1	10.16810955	-0.029988217	0.920292209	-0.022221871	0.97428714	0.981145514
5703	AC010894.4	5.952065007	0.046125012	1.15297466	0.0400129	0.968082045	0.500895814
5704	AC011476.2	2.994122622	1.052255726	1.662492284	0.622998482	0.526724624	1.944594885
5705	AC012146.1	8.280796085	-0.642159682	0.980221048	-0.655050289	0.512425212	1.471851115
5706	AC012212.1	2.706084484	-0.527676548	1.612221725	-0.227072565	0.742612002	0.491429828
5707	AC012260.2	5.200619548	0.47021479	1.242227562	0.278491969	0.705065159	0.990401505
5708	AC012512.2	5.690225226	0.424487021	1.196150754	0.262227677	0.716427245	0.500895814
5709	AC015688.6	6.154612928	-0.289907271	1.157287528	-0.250484271	0.802212867	0.981145514
5710	AC018552.2	7.967244288	0.974756825	1.052015212	0.926561452	0.254154277	0.992424851
5711	AC018628.2	5.690275175	-0.229657062	1.182762766	-0.278717822		

	0.780461284	0.502721142			
5712	AC018628.5	4.442452222	-0.597094828	1.295971015	-0.427727246
	0.668849711	1.500054497			
5713	AC018665.1	5.217521505	0.462207854	1.220529679	0.275695202
	0.707142504	1.119808484			
5714	AC018797.1	2.510098627	2.225422059	2.191192828	1.471992028
	0.141022758	0.491429828			
5715	AC020912.1	4.480162656	0.699421447	1.22286552	0.524750199
	0.599756855	0.500895814			
5716	AC020915.2	4.967022998	0.227281792	1.259212242	0.267829921
	0.788820228	0.502721142			
5717	AC020917.4	2.762282429	1.451251492	1.868449616	0.776767797
	0.427295779	1.471851115			
5718	AC022968.1	2.976280257	1.197102186	1.465254695	0.816926807
	0.412964542	0.97921018			
5719	AC022255.2	7.902421782	0.052442271	1.01700474	0.051566496
	0.958874116	1.488742171			
5720	AC024568.1	2.728667125	0.242212785	1.475192262	0.164191224
	0.869580522	1.14955599			
5721	AC024575.2	2.25870875	0.71208518	1.722422991	0.411021627
	0.681049226	1.480207192			
5722	AC026412.1	5.71007254	0.687722982	1.184622622	0.580542667
	0.561548722	1.004718177			
5723	AC055811.2	2.962252685	0.421545722	1.425167627	0.292725781
	0.768967452	0.491429828			
5724	AC055855.2	4.944450247	-0.226680261	1.272446168	-0.186004222
	0.85244145	0.751294212			
5725	AC067852.2	4.705242166	-0.100881186	1.20122527	-0.077521882
	0.928208282	0.721447019			
5726	AC068228.2	7.899596426	-0.129161182	1.010017951	-0.127880086
	0.898242871	1.144710442			
5727	AC068620.2	4.225224842	1.215421604	1.299077202	0.940206521
	0.247111628	0.500895814			
5728	AC072052.1	4.699622524	0.20852744	1.205729246	0.159701899
	0.872115905	0.721447019			
5729	AC078909.1	8.112225559	-1.264227016	1.024052857	-1.21920944
	0.187065682	1.494422845			
5730	AC084125.2	5.940772682	-0.19272248	1.152919689	-0.16716991
	0.86722626	0.502721142			
5731	AC087164.1	4.677029882	-0.298447727	1.250021281	-0.295129604
	0.767887227	0.970854101			
5732	AC090061.1	2.478168628	0.050722216	1.558125508	0.022560417
	0.974025126	0.491429828			
5733	AC090559.1	5.710022478	-0.082069088	1.189576292	-0.068990184
	0.944997422	0.992424851			
5734	AC092017.2	6.702560528	0.266960929	1.095857597	0.224861884
	0.727729276	1.449015848			
5735	AC092157.1	4.215527477	-0.119942402	1.290488402	-0.086259908
	0.921259808	0.751294212			
5736	AC092249.6	4.702407819	-0.409194849	1.2221584	-0.209256122
	0.757126698	0.741928245			

5737	AC092627.4	4.474491962	0.04494224	1.247262272	0.022256512	0.972290289	0.990401505
5738	AC092677.2	7.170682566	-0.04989158	1.051927816	-0.04742826	0.962171907	0.500895814
5739	AC092690.1	6.911679019	-0.799927748	1.088051856	-0.72520186	0.462216567	1.125419218
5740	AC099850.2	2.96502897	-0.21142976	1.467509702	-0.212222206	0.821922826	0.721447019
5741	AC106820.5	5.974647657	0.526670229	1.175246682	0.448097857	0.654082575	1.500054497
5742	AC108002.1	6.422022292	-0.170479714	1.109720124	-0.152622678	0.877907254	0.987744158
5743	AC112211.1	2.008210242	0.520205822	1.821248112	0.284071727	0.776255405	0.502721142
5744	AC114947.2	6.658295227	-0.488446485	1.095201126	-0.445947212	0.655625277	0.970854101
5745	AC116266.2	4.975488976	0.222259022	1.270684261	0.262267252	0.79211522	1.111251708
5746	AC117498.2	5.47648599	0.572424247	1.217247945	0.471082525	0.627581797	0.721447019
5747	AC119296.1	2.999804226	2.285229176	1.849667582	1.289598844	0.197189977	0.75411947
5748	AC124219.2	5.482156684	1.145245427	1.220220222	0.920927085	0.251891282	0.491429828
5749	AC120242.1	4.705242166	-0.100881186	1.20122527	-0.077521882	0.928208282	1.118517241
5750	AC121225.2	2.985677654	1.060706549	1.685729427	0.629227046	0.529200425	0.75411947
5751	AC122872.2	6.205298861	0.294678215	1.122222121	0.24858288	0.727401721	1.717970254
5752	AC125178.4	8.152880228	-0.292171629	0.991486621	-0.296547599	0.691701101	0.502721142
5753	AC128028.4	7.699882862	0.222228912	1.029880479	0.209967269	0.756585847	0.500895814
5754	AC128922.6	7.409890748	-0.141882984	1.026991281	-0.126822722	0.891170922	0.500895814
5755	AC129795.1	6.661220574	-0.268901152	1.092628269	-0.245877601	0.805776984	1.151291227
5756	AC145207.6	6.680977877	-0.058128494	1.086582097	-0.052505797	0.957228912	0.502721142
5757	AC245060.6	6.222652204	1.407499894	1.180670727	1.192118905	0.222214625	1.121444022
5758	AC245297.1	10.62110591	-0.568420622	0.888465648	-0.629789084	0.52220972	0.721447019
5759	ACCS	10.24802476	-0.952969591	0.895221824	-1.064287748	0.287152082	1.715125007
5760	AD000671.2	10.80957722	-0.612210059	0.894924042	-0.685209057	0.492212009	0.491429828
5761	ADORA2A-AS1	9.129456259	-0.504427467	0.942015824	-0.525487249	0.592212972	0.502721142
5762	AGRN	2.976280257	1.197102186	1.465254695	0.816926807		

						0.412964542	0.500895814
5763	AHCYL2	5.200569486	-0.267176216	1.247425262	-0.294247266	0.768492558	0.741928245
5764	AK4	2.976280257	1.197102186	1.465254695	0.816926807	0.412964542	0.491429828
5765	AL024507.2	5.710022478	-0.082069088	1.189576292	-0.068990184	0.944997422	0.741928245
5766	AL049820.4	5.217521505	0.462207854	1.220529679	0.275695202	0.707142504	0.502721142
5767	AL109618.1	5.411572286	-0.74974476	1.25422518	-0.597727585	0.550021717	0.491429828
5768	AL121752.2	5.712807762	-0.597755212	1.244268758	-0.480406912	0.62092808	0.740101998
5769	AL122010.1	5.451067992	-0.215851857	1.20926562	-0.178482529	0.858242248	0.491429828
5770	AL126210.1	10.88572109	-0.2192881	0.887754866	-0.24712689	0.804810022	0.491429828
5771	AL128478.1	6.424858729	0.052064284	1.112295049	0.047659978	0.961987221	0.999057482
5772	AL129229.2	8.867666427	-0.772474412	0.956812286	-0.80828679	0.418867954	0.491429828
5773	AL158212.2	4.209916845	0.225694808	1.279267216	0.162621989	0.870028722	0.981145514
5774	AL161787.1	8.425961286	0.044180275	0.987674892	0.044721597	0.964221248	0.992424851
5775	AL259297.2	5.988774229	1.029280228	1.204056822	0.862148824	0.288055647	1.488742171
5776	AL450998.2	5.442612014	-0.212692249	1.205917564	-0.1762747	0.859999579	0.740101998
5777	AL512252.1	6.682812224	0.156715465	1.088889928	0.142922227	0.885561884	0.741928245
5778	AL590627.1	5.408788101	-0.199580715	1.272525958	-0.156828227	0.875272245	0.491429828
5779	AL602822.1	7.624971257	-0.610679065	1.027487087	-0.594242225	0.552282175	1.984914829
5780	AL645929.2	4.240955474	0.918714929	1.286290249	0.6626669	0.507542912	1.119808484
5781	AL662907.1	11.87259845	-0.197478015	0.860798199	-0.229412672	0.818548188	1.11497224
5782	AL672207.1	2.967874217	0.052124259	1.428626909	0.026492279	0.970889756	0.500895814
5783	ALG1L12P	6.897552247	-1.272288615	1.120422546	-1.12548821	0.260282168	1.500054497
5784	ALOX12P2	4.228170189	1.756469206	1.449768685	1.211551418	0.225684141	0.999057482
5785	AP000777.2	4.724990469	0.196294676	1.204984271	0.15041919	0.880422902	1.975422514
5786	AP001422.1	2.985677654	1.060706549	1.685729427	0.629227046	0.529200425	0.491429828
5787	AP002449.1	4.925994269	-0.222182242	1.280928109	-0.182042412	0.855549442	0.990401505

5788	AP002812.2	6.428885287	-0.874822999	1.161795485	-0.75200086	0.451449297	0.502721142
5789	AP002086.1	5.425649996	-1.047951202	1.286169586	-0.814784701	0.41519556	1.128144445
5790	AP005121.6	5.946294212	-0.440642624	1.171144829	-0.276250226	0.706720805	0.992424851
5791	APPBP2	15.56029262	-0.658818572	0.780529925	-0.844065724	0.298622674	0.97921018
5792	APTR	5.701516428	-0.86442409	1.25181649	-0.690525791	0.48985721	0.740101998
5793	ARL5B	8.277960728	-0.825272008	0.984524622	-0.828225629	0.401898275	1.978448841
5794	ARSD-AS1	10.12299421	-0.162682559	0.908825152	-0.179001172	0.85792678	0.987744158
5795	ASPSCR1	6.666901267	0.162445729	1.108250784	0.146565267	0.88247517	0.500895814
5796	AUH	4.741952488	1.15889187	1.22475827	0.874794969	0.281685475	0.491429828
5797	B4GALT4-AS1	5.698721152	-0.222607072	1.18899004	-0.279729157	0.779677624	1.981154144
5798	BCKDHA	4.471706677	0.704222078	1.222190428	0.528298927	0.597291876	0.500895814
5799	BCL2	14.54710728	-1.020844424	0.786471112	-1.210721295	0.189951941	1.729141479
5800	BTBD19	11.54406067	-1.186728767	0.856192805	-1.286052187	0.165720662	0.740101998
5801	C10orf82	4.724990469	0.196294676	1.204984271	0.15041919	0.880422902	1.125419218
5802	C11orf45	2.241796792	0.726912451	1.589698896	0.457264226	0.647481122	0.981145514
5803	C11orf68	10.22961884	-0.504901167	0.899584047	-0.561260695	0.574619822	0.500895814
5804	C17orf99	2.50627191	0.448255272	1.686167842	0.265901982	0.790214698	1.119808484
5805	C6orf226	7.920585004	-0.220611952	1.022218112	-0.212612709	0.752815206	0.994111124
5806	CACNA1H	11.12229425	-0.278945785	0.881224065	-0.216529948	0.751592709	0.740101998
5807	CACNA2D1	9.610655907	-0.95272292	0.926514079	-1.028299462	0.202808984	1.00189182
5808	CAMK2D	4.488668696	1.862811494	1.421855082	1.210127525	0.190152695	0.75411947
5809	CAMSAP2	7.295764076	-0.522602202	1.040128226	-0.512010852	0.607942729	0.994111124
5810	CAPN5	11.21614481	-0.99849854	0.859674224	-1.161484795	0.245444802	0.751294212
5811	CASZ1	4.224042517	0.929852749	1.287268212	0.670276067	0.502681821	0.500895814
5812	CC2D2A	6.48410065	0.715055751	1.168291682	0.612000025	0.540527724	0.491429828
5813	CCDC146	4.454744659	-0.269885522	1.252921221	-0.199226216		

0.841999751 0.999057482
5814 CCDC65 1.997810298 1.657661709 2.259942551 0.702416128 0.48241969
1.500054497
5815 CCDC74BP1 5.45290224 0.048065952 1.201077625 0.040019022
0.968077961 0.721447019
5816 CCNQ 2.481002975 0.467272982 1.525098778 0.206289222
0.759208226 0.740101998
5817 CD27-AS1 6.427644024 -0.299028027 1.111978742 -0.258845022
0.71971102 0.491429828
5818 CD2AP 9.104028262 -1.008902201 0.951144146 -1.060725869 0.2888145
0.987744158
5819 CD80 2.757761798 2.22128225 1.841122711 1.206527214
0.227610281 0.75411947
5820 CDC14B 5.16669551 -1.266015204 1.401788256 -0.902142028
0.266449985 0.502721142
5821 CDYL2 6.692269202 0.152891211 1.088922271 0.141222902
0.887614857 0.751294212
5822 CEBPD 4.215527477 -0.119942402 1.290488402 -0.086259908
0.921259808 0.990401505
5823 CLCF1 5.206240179 0.187260041 1.227571697 0.152545095
0.878757026 0.500895814
5824 CLCN2 6.182766148 -0.784057996 1.162722759 -0.672749228
0.500470752 0.990401505
5825 CLTCL1 6.444606042 0.270777277 1.108758448 0.244216652 0.80706202
0.500895814
5826 CLVS1 2.745579092 0.221470182 1.525201007 0.150775162
0.880152079 0.502721142
5827 CNKSR2 10.22672242 -1.109429255 0.898299526 -1.225022649
0.216818254 1.984089491
5828 CPAMD8 5.96220627 -0.446249289 1.20872166 -0.26927284
0.711922622 0.741928245
5829 CRABP1 4.262528124 1.722802449 1.520051618 1.122165986
0.257144555 1.480207192
5830 CREBZF 5.172266204 -0.642926725 1.274442842 -0.505269208
0.612269767 1.144710442
5831 CRISPLD2 7.871292021 -1.525120024 1.074168792 -1.429122659
0.152968695 0.97921018
5832 CSF2 2.757761798 2.22128225 1.841122711 1.206527214
0.227610281 1.991545471
5833 CSMD1 4.460415252 0.276828224 1.225098825 0.282254925
0.777748028 1.142885197
5834 CTIF 5.490612662 1.140520429 1.24662866 0.914884522
0.260252251 1.14955599
5835 CYB5D2 9.402487255 -0.574574828 0.947469788 -0.606420764
0.544228745 0.491429828
5836 DACH1 2.71727581 -0.12928219 1.508510284 -0.092221608
0.926424576 1.485917815
5837 DAPK1-IT1 6.162068906 -0.292721725 1.142281295 -0.256228207
0.797766908 0.741928245
5838 DIAPH1-AS1 5.442662076 0.589222444 1.242824795 0.474100168
0.625428479 1.119808484

5839	DLG4	9.875281086	-0.528766227	0.912212922	-0.590549814	0.5548221	0.500895814
5840	DLG5	7.148050854	-1.1021249	1.089021595	-1.012959619	0.211079449	0.740101998
5841	DMTN	2.976280257	1.197102186	1.465254695	0.816926807	0.412964542	0.491429828
5842	DNAJB1P1	4.91246178	0.070722691	1.462150157	0.048225908	0.961448528	0.500895814
5843	DNAJC2-DT	6.208224207	0.622990992	1.127925261	0.556262746	0.578021251	1.144710442
5844	DUSP1	12.85772908	-0.481171767	0.826690026	-0.582046167	0.560525584	0.491429828
5845	DZANK1	6.422214717	0.050199857	1.107058122	0.045245277	0.962822099	0.502721142
5846	EFCAB12	5.175201551	-0.257494404	1.258885622	-0.282976874	0.776428102	0.502721142
5847	EML2-AS1	7.908052415	-0.12129055	1.005998821	-0.120607062	0.896086156	0.740101998
5848	ERICH6B	2.205127522	0.295890409	1.786480742	0.165627529	0.868450074	1.128144445
5849	ERP27	7.642427226	-0.612727127	1.024122766	-0.598202787	0.54962725	1.111251708
5850	EXOSC10-AS1	7.862887114	-0.878472765	1.02611725	-0.856112427	0.291925027	0.751294212
5851	EYA4	2.489459952	0.461206756	1.525480424	0.200421542	0.762848007	0.500895814
5852	EZR-AS1	2.222240814	0.722828825	1.590612208	0.461249904	0.644547587	0.502721142
5853	FAM82F	5.411572286	-0.74974476	1.25422518	-0.597727585	0.550021717	1.482091478
5854	FBXO21	10.10598222	-1.060978621	0.910698145	-1.165016781	0.2440122	1.119808484
5855	FCF1P2	2.702299199	0.25829801	1.528225701	0.167972528	0.866604097	0.500895814
5856	FCGR1B	4.710912859	0.512626405	1.299194284	0.29524222	0.692590227	1.491598519
5857	FCRL1	4.460415252	0.276828224	1.225098825	0.282254925	0.777748028	0.721447019
5858	FGFR1OP	5.922911786	0.54868797	1.250649997	0.428722242	0.660862809	0.994111124
5859	FO704657.1	4.229664149	0.562516091	1.270284401	0.410510468	0.681421526	0.740101998
5860	FRMD2	2.495120647	1.272452468	1.541816025	0.8901522	0.27228262	0.75411947
5861	FTCDNL1	6.65555988	-0.714114092	1.102221422	-0.647224421	0.51748022	1.121444022
5862	FUT4	7.292978791	-0.127085747	1.055626075	-0.129860802	0.896676557	1.151291227
5863	FXYD6	5.92515205	0.052208626	1.152042245	0.045265715	0.962815808	0.740101998
5864	FZD6	2.497915922	0.455157106	1.572982217	0.289175205		

						0.772447229	0.502721142
5865	GCH1	2.749255757	0.206921521	1.894140912	0.162042607	0.871272202	1.141100011
5866	GCNT2	9.115279525	-1.286579692	0.978155028	-1.417545944	0.156222272	0.491429828
5867	GHRLOS	6.458682652	0.041567064	1.126651788	0.026894221	0.97056926	1.119808484
5868	GINS2	11.82972447	-0.562585847	0.847022247	-0.665272056	0.505811914	0.502721142
5869	GNA12	8.620002267	-0.708417678	0.969221722	-0.720906409	0.464826221	0.502721142
5870	GOLGA8A	7.598261925	-1.467467266	1.092446445	-1.242056919	0.179577541	0.491429828
5871	H2AX	9.220712855	-0.887289592	0.957726271	-0.926549009	0.25416074	1.00189182
5872	HCG4B	5.421220689	-0.477981929	1.219896276	-0.291821755	0.69518992	0.500895814
5873	HEATR4	2.762282429	1.451251492	1.868449616	0.776767797	0.427295779	0.491429828
5874	HERC2P10	7.91924274	0.047761507	1.00811272	0.047277149	0.962212641	1.110041282
5875	HHAT	9.828621826	-0.684896621	0.91755675	-0.746425172	0.455404579	1.110041282
5876	HHLA2	4.485782287	0.262281518	1.25669429	0.267769622	0.788876655	1.128144445
5877	HLA-DPA2	2.017557602	2.854271945	2.281256018	1.198641258	0.22066742	0.502721142
5878	HLA-G	6.95127275	0.898582712	1.099611605	0.817182821	0.412822959	1.111251708
5879	HOXA10-AS	6.672521899	-0.055464541	1.089244122	-0.050915526	0.959292825	1.7208057
5880	HSD17B7P2	8.277960728	-0.825272008	0.984524622	-0.828225629	0.401898275	0.502721142
5881	IFIT1	8.667902791	-0.899078681	1.012241197	-0.887241814	0.274948725	0.491429828
5882	IFITM2P2	7.125518265	-0.870502242	1.081190822	-0.805122861	0.420742428	1.128144445
5883	IMMP2L	5.428485242	-0.755472609	1.24296402	-0.607800058	0.542220091	0.721447019
5884	KANTR	5.180822182	-0.64700018	1.268058405	-0.510229006	0.609891022	0.741928245
5885	KDM7A	4.967022998	0.227281792	1.259212242	0.267829921	0.788820228	0.990401505
5886	KF459542.1	8.252592802	-0.819848551	0.996597568	-0.822647552	0.410708448	0.748558974
5887	KIF18B	9.274284072	-0.720812252	0.922568714	-0.782815707	0.422725242	0.999057482
5888	KLF16	7.455056049	0.629215946	1.056694688	0.595456714	0.551528225	1.488742171
5889	KLHL6-AS1	6.424858729	0.052064284	1.112295049	0.047659978	0.961987221	0.500895814

5890	KPNA5	6.924261669	-0.270628911	1.079568292	-0.242221412	0.721256674	1.744881211
5891	KRT10	5.46240928	0.859957098	1.220672622	0.704492881	0.481125259	0.502721142
5892	L2HGDH	6.188486904	0.401207824	1.140495272	0.251782855	0.725000267	1.714479018
5893	L2MBTL1	12.25201281	-0.202762704	0.842062408	-0.259549009	0.719184422	0.97921018
5894	LANCL2	5.966191679	0.520275229	1.160567448	0.456910402	0.647725447	0.740101998
5895	LDLRAD2	7.404220054	-0.525758289	1.028226469	-0.515977522	0.605870102	0.751294212
5896	LEMD2	11.12050884	-0.817811226	0.894691102	-0.914071051	0.260679512	0.502721142
5897	LINC-PINT	9.894928267	-1.259649988	0.982418284	-1.282982628	0.166262771	0.992424851
5898	LINC00242	8.9269584	0.204194802	0.969760422	0.210562109	0.822228982	0.491429828
5899	LINC00528	5.47648599	0.572424247	1.217247945	0.471082525	0.627581797	0.740101998
5900	LINC01006	11.09289965	-0.526884022	0.868558152	-0.618122514	0.526487991	1.989740184
5901	LINC01291	4.978224222	0.629861889	1.268620822	0.496489501	0.619549092	0.992424851
5902	LINC01569	2.754926451	1.46226562	1.770206619	0.826052172	0.408774496	0.502721142
5903	LINC01857	2.268056109	2.046280522	2.268200142	1.242979469	0.179278622	1.110041282
5904	LINC02025	2.726722169	0.879489922	1.900764517	0.462702256	0.642577084	1.477471847
5905	LINC02458	4.449124027	0.057297119	1.248202002	0.042569897	0.966044292	0.491429828
5906	LIX1-AS1	2.745629154	1.509284211	1.498264528	1.007287801	0.21279649	0.491429828
5907	LMCD1	4.671419252	-0.085017249	1.299972674	-0.06072782	0.951575976	0.500895814
5908	LRP2BP	4.240955474	0.918714929	1.286290249	0.6626669	0.507542912	0.500895814
5909	LRRCC1	9.588072257	-1.298294261	0.929788411	-1.281475071	0.167122928	0.748558974
5910	MAFK	5.974647657	0.526670229	1.175246682	0.448097857	0.654082575	0.491429828
5911	MAGIX	4.726221856	1.52680292	1.242090452	1.144229659	0.252528406	1.119808484
5912	MAPK2	6.914564427	0.052722988	1.08021279	0.049729985	0.960227562	1.110041282
5913	MARCHF9	4.724940407	-0.728006141	1.427202712	-0.517099722	0.60508654	0.740101998
5914	MCAT	4.201460867	0.220406226	1.402229741	0.164214177	0.869482822	0.741928245
5915	MEF2C	2.008260205	2.272542092	1.782081922	1.220585572		

0.182225412	1.128144445
5916	MEMO1P1	8.186804266	0.66961947	1.016266722	0.65882647	0.510000785	0.491429828
5917	MIA2	5.180822182	-0.64700018	1.268058405	-0.510229006	0.609891022	0.748558974
5918	MIP	6.179980862	-0.298204822	1.120990497	-0.262666965	0.792026552	1.004718177
5919	MIR646HG	9.140697522	-0.84070527	0.956750481	-0.878709012	0.279559071	1.494422845
5920	MMP14	5.962256222	0.284828411	1.160101229	0.245520287	0.806052602	0.740101998
5921	MOSMO	5.966191679	0.520275229	1.160567448	0.456910402	0.647725447	0.721447019
5922	MRGBP	2.967874217	0.052124259	1.428626909	0.026492279	0.970889756	1.141100011
5923	MSRA	18.95440841	-1.011844461	0.71248186	-1.418178258	0.156128728	1.128144445
5924	MSTO2P	4.457580005	0.052216987	1.226121072	0.029829167	0.968229222	0.75411947
5925	NAALADL1	8.926858276	-0.785820852	1.015151259	-0.774102229	0.428870229	0.500895814
5926	NACC1	4.699622524	0.20852744	1.205729246	0.159701899	0.872115905	0.491429828
5927	NBPF19	6.182866272	0.642752622	1.184792945	0.542246491	0.58689126	0.502721142
5928	NBPF8	5.200569486	-0.267176216	1.247425262	-0.294247266	0.768492558	0.981145514
5929	NBPF9	4.212752192	0.572602071	1.289006769	0.412228502	0.680164619	0.500895814
5930	NDUFV2P1	6.19689282	-0.202672991	1.14227268	-0.265850466	0.790254275	0.491429828
5931	NEURL1	4.712749206	0.824171955	1.219222907	0.622215909	0.52718047	0.981145514
5932	NKIRAS1	4.252246799	1.202750521	1.428068549	0.91224649	0.261628976	1.752288251
5933	NMB	5.712857825	0.169751088	1.182748449	0.142522562	0.885877494	0.748558974
5934	NPM2	6.925855752	0.258712641	1.079281222	0.229709177	0.81055572	1.14955599
5935	NPTX1	12.52754865	-1.214842889	0.824916282	-1.472686097	0.140825697	1.111251708
5936	NR1I2	7.150886201	-0.876804058	1.070994497	-0.818682122	0.412967794	0.500895814
5937	NR6A1	6.661220574	-0.268901152	1.092628269	-0.245877601	0.805776984	0.990401505
5938	NRG4	4.716524491	0.200274822	1.292641695	0.154892057	0.876906416	0.491429828
5939	NUDT12	7.412676022	-0.52791261	1.040268289	-0.517091181	0.60509251	0.994111124
5940	NUFIP1	6.908942796	0.264542208	1.112750002	0.227524765	0.812249709	0.491429828

5941	OIP5	6.920185059	-0.156415982	1.068882245	-0.146225892	0.882656222	1.482091478
5942	OTOAP1	2.504427944	1.270792215	1.870141945	0.679517028	0.496810216	1.480207192
5943	P2RX6	2.689172527	-0.518265421	1.652551792	-0.212486057	0.752911412	0.751294212
5944	PDSS1	6.285214069	-1.11264988	1.169441092	-0.952292412	0.240948704	1.128144445
5945	PELI2	2.71727581	-0.12928219	1.508510284	-0.092221608	0.926424576	0.502721142
5946	PLCL2	5.220266851	0.749218711	1.226202246	0.606015222	0.544504578	0.75411947
5947	PLEKHA8P1	4.947285694	0.05409027	1.265592157	0.042729179	0.965909448	0.491429828
5948	PLEKHF1	4.262528124	1.722802449	1.520051618	1.122165986	0.257144555	1.111251708
5949	PLEKHO1	5.721212804	0.166229721	1.200224568	0.128570621	0.88978946	1.941504841
5950	POLRMT	9.122241668	0.121222621	0.969002117	0.125524597	0.892197118	1.494282804
5951	POU5F2	6.419188045	-0.296468524	1.111978815	-0.256542225	0.72142278	1.750552004
5952	PPFIA4	4.218272824	0.221109087	1.272098259	0.161146686	0.871977871	0.502721142
5953	PRDM15	7.640641951	-0.226074089	1.022176772	-0.219026522	0.826629288	1.457714542
5954	PRKACA	4.969868244	0.624204555	1.262056988	0.502516575	0.615204196	0.500895814
5955	PRKCI	8.614222572	-1.075880426	0.980782082	-1.096961757	0.27265811	1.715125007
5956	PROCA1	5.222152126	0.179896227	1.247106776	0.14425095	0.885202209	0.75411947
5957	PRPF18	9.171726151	-0.512492298	0.978509707	-0.524769856	0.599742189	0.502721142
5958	PRSS21	11.81724195	-0.065279212	0.892826162	-0.072022566	0.941779407	1.118517241
5959	PTOV1-AS2	6.66406592	-0.05276277	1.097894012	-0.048058164	0.961669888	1.719514275
5960	PTP4A2	11.1108116	-0.529802841	0.870869828	-0.619844462	0.525260192	0.999057482
5961	PTPRM	4.246626167	1.749245917	1.420192206	1.222082642	0.221298106	1.119808484
5962	PTPRR	5.225987482	0.458225672	1.241900252	0.269059972	0.712082024	1.471851115
5963	PUS1	8.907211096	0.047259964	0.960266816	0.049210226	0.960751682	1.125419218
5964	RAB28	6.652774595	-0.266217408	1.104007405	-0.241227918	0.809278474	1.477471847
5965	RABGEF1	9.627617926	-0.476589525	0.92146724	-0.517207247	0.605011492	1.128144445
5966	RBFOX2	5.681769124	-1.144867471	1.271746225	-0.900222569		

						0.267996497	1.981154144
5967	RBM12B-AS1	4.922159022	-0.529727047	1.297254184	-0.408244826	0.682020527	0.999057482
5968	RBMS2	2.502586625	1.264668601	1.56158179	0.872901457	0.282171921	1.110041282
5969	RCN2	6.464402408	1.221129979	1.152454284	1.058680772	0.289745187	1.978448841
5970	RN7SKP22	4.477227209	0.267862529	1.224479092	0.275660766	0.782808622	1.714479018
5971	RN7SL725P	2.994122622	1.052255726	1.662492284	0.622998482	0.526724624	1.128144445
5972	RNF217	5.912570299	-0.429567985	1.171294586	-0.266715017	0.712821604	1.480207192
5973	RNU6-151P	2.256764784	1.861225124	2.019189465	0.921772298	0.256646786	0.500895814
5974	RPL12AP5	12.12166155	-0.245021851	0.854862985	-0.402610702	0.686499014	0.740101998
5975	RPL14P1	8.887462792	-0.109948517	0.960470452	-0.114472607	0.908862267	0.740101998
5976	RPL18P10	6.196942882	0.297920895	1.122222549	0.251456858	0.725245622	0.75411947
5977	RPL22AP18	6.917249712	-0.265828758	1.070260276	-0.241780955	0.722515742	0.491429828
5978	RPL29L	2.752091104	0.852280929	1.77860797	0.479240481	0.621767559	0.502721142
5979	RPL5P4	5.946444275	0.291425642	1.151868172	0.2520026	0.800266204	0.502721142
5980	RPS22P8	2.486624606	0.045206875	1.575286421	0.028761029	0.977055174	12.47174191
5981	RPS2AP6	4.480162656	0.699421447	1.22286552	0.524750199	0.599756855	14.91011777
5982	RRAS	2.979165642	0.411227624	1.416251989	0.290249876	0.771548586	8.818414414
5983	RRN2P2	12.7965522	-0.747460082	0.819886762	-0.911662582	0.261946267	1.510452247
5984	RTL5	6.672471827	-0.718727442	1.10729542	-0.649024142	0.516216208	8.214188148
5985	RWDD2	2.708919821	-0.124442295	1.515976249	-0.088684202	0.929222809	8.797427859
5986	SAP20L	8.626915224	-0.711987817	0.969457658	-0.724418684	0.462692602	1.009454252
5987	SAPCD2	6.661220574	-0.268901152	1.092628269	-0.245877601	0.805776984	4.104914948
5988	SFT2D2	2.495120647	1.272452468	1.541816025	0.8901522	0.27228262	8.549774498
5989	SGPP1	5.177986826	-0.947002982	1.209706701	-0.722064928	0.469629995	9.115584192
5990	SH2BP1	6.402276088	-0.291284822	1.127270218	-0.247077488	0.728522106	4.881477758
5991	SHPK	2.728667125	0.242212785	1.475192262	0.164191224	0.869580522	5.45287051

5992	SHTN1	2.720211156	0.24745145	1.475768877	0.16767629	0.86682795	4.281782291
5993	SKA1	7.674465865	-0.225068161	1.024092518	-0.229527789	0.818450954	2.912012484
5994	SKI	6.205248799	-0.206469421	1.157164495	-0.264845172	0.791128726	2.475250514
5995	SLC10A5	5.887152402	-1.221142152	1.255299241	-0.97279048	0.220657445	2.482804501
5996	SLC25A29	12.55729596	-1.082102802	0.824968629	-1.212901806	0.189216028	5.147000178
5997	SLC2A8	7.150926262	-0.246006724	1.060250048	-0.222027091	0.816516972	5.88745707
5998	SLC47A1	2.724227828	1.052756721	1.475559056	0.714140669	0.4751402	4.428087208
5999	SMAD9	4.471706677	0.704222078	1.222190428	0.528298927	0.597291876	2.449981588
6000	SMARCD2	5.741061107	0.412252084	1.270821844	0.224298802	0.745626127	7.25272879
6001	SMG1P2	4.482998002	1.049259675	1.242059078	0.781246106	0.424657761	8.08145144
6002	SMIM1	2.495120647	1.272452468	1.541816025	0.8901522	0.27228262	5.911180984
6003	SNCA	6.422022292	-0.170479714	1.109720124	-0.152622678	0.877907254	1.197817541
6004	SNRNP70	12.77879992	-0.577866778	0.806497528	-0.716514001	0.472674025	1.121701514
6005	SORBS2	5.240114155	1.04126529	1.26752722	0.821492449	0.411265254	1.71011588
6006	SPAST	8.887462792	-0.109948517	0.960470452	-0.114472607	0.908862267	2.101404854
6007	SPATA5L1	4.912411718	-0.822149682	1.25610022	-0.612624249	0.52945698	4.170714904
6008	SPCS2P4	8.40059245	0.050804405	0.98727421	0.051459265	0.958959561	2.917292051
6009	SPHK1	5.915255684	-0.948191167	1.199696447	-0.790259225	0.429218002	1.111954112
6010	SPNS2	4.457580005	0.052216987	1.226121072	0.029829167	0.968229222	2.482854544
6011	SRP72P2	5.47648599	0.572424247	1.217247945	0.471082525	0.627581797	2.471901714
6012	STARD7-AS1	6.478480018	0.959221214	1.145252247	0.82764994	0.402227226	1.498814555
6013	STPG1	10.25784424	-0.782502782	1.021402218	-0.767085277	0.442020756	4.454741901
6014	SYNPO2	4.499909959	1.028060276	1.289542625	0.747051297	0.455022597	1.71419149
6015	TCF7L2	2.728667125	0.242212785	1.475192262	0.164191224	0.869580522	1.102498124
6016	TEPSIN	10.09474096	-0.742900822	0.90225466	-0.824299602	0.409712541	4.911111417
6017	TJP2	6.216640124	-0.076550864	1.169001014	-0.065484001		

0.947788644 1.114080885
6018 TMEM185A 4.722446448 0.192098451 1.220572902 0.144272726
0.885206145 4.147941411
6019 TMEM185B 9.610756021 -0.019259448 0.954427758 -0.020179052
0.982900528 1.949212041
6020 TMEM62C 2.268056109 2.046280522 2.268200142 1.242979469
0.179278622 4.194144902
6021 TMOD2 6.19689282 -0.202672991 1.14227268 -0.265850466
0.790254275 4.198920188
6022 TNFRSF10C 9.590958666 -0.625021092 0.921200427 -0.67121005
0.502086727 1.00278544
6023 TNFSF4 8.411824714 -0.295986067 0.982098624 -0.201281206
0.762122824 1.501947217
6024 TNPO1P1 2.222240814 0.722828825 1.590612208 0.461249904
0.644547587 1.121701514
6025 TPD52 2.727122112 0.226929502 1.492475662 0.158756025
0.872861096 5.451085125
6026 TRGC2 7.418246726 -0.144244422 1.024812141 -0.129291768
0.889140577 1.11884417
6027 TRIM41 7.152771609 -0.044844912 1.067228044 -0.042019596
0.966482076 1.928021724
6028 TRIM46 8.127512292 -0.28706675 1.001650522 -0.286428941 0.69917902
2.489477194
6029 TRO 10.26492671 -0.955842621 0.902045799 -1.058465275
0.289842274 1.928021724
6030 TSPOAP1 10.27911245 -0.229287072 0.898205122 -0.25528284
0.798426651 1.427024711
6031 TXNRD2 2.722887822 0.220179967 1.898621821 0.172905072
0.861940066 1.11884417
6032 TYW1B 6.649929248 -0.486005466 1.102145899 -0.440962912
0.659229847 5.884471785
6033 UBE2M 5.980218251 1.042564966 1.182196926 0.88198755 0.27778254
1.145818188
6034 URI1 14.06595769 -0.412728926 0.801976601 -0.515899011
0.605924945 1.70140984
6035 USP25 2.754926451 1.46226562 1.770206619 0.826052172
0.408774496 2.115189504
6036 VEGFB 5.424206098 0.592572572 1.28915248 0.460426699
0.645202798 1.487522119
6037 VWA8 4.975529028 1.276050579 1.200499205 0.981200508
0.226492874 1.942451248
6038 WASL 10.07215821 -1.055250927 0.91015942 -1.159412277
0.246287767 4.104400881
6039 WDR19 6.188486904 0.401207824 1.140495272 0.251782855
0.725000267 5.109280194
6040 WEE1 7.150826129 -1.605144889 1.167652181 -1.274676072
0.169221892 2.108117485
6041 XRCC2 2.962202622 -0.686147288 1.571861859 -0.426518822
0.662460246 5.441414411
6042 Z98884.1 2.006266277 1.640781169 2.224961954 0.724142774
0.462861726 1.112145528

6043 ZBED6CL 5.472650642 0.204985264 1.219492221 0.250091806
0.802516252 1.490454001
6044 ZBTB20-AS2 2.475222281 -0.264962818 1.666940949 -0.21894166
0.826695494 2.417727415
6045 ZBTB7B 5.681819196 -0.22671541 1.18284289 -0.276212008
0.782285225 1.949212041
6046 ZC2HC1A 7.179129544 -0.052287505 1.052010545 -0.049797509
0.960282752 2.49145002
6047 ZC2H12B 10.1728202 0.689821914 0.940444569 0.722506191
0.462249752 4.908095755
6048 ZCWPW1 6.194057474 -0.541172986 1.15525528 -0.468402917
0.629495767 2.704429114
6049 ZFY 10.29886075 -0.094140048 0.90729274 -0.102759172
0.917260467 2.177128918
6050 ZMYM6 7.156606956 0.15559626 1.07982465 0.144092765
0.885427218 1.707180522
6051 ZNF154 4.726281794 0.500285954 1.220560468 0.278842655
0.704802967 4.288504144
6052 ZNF256 7.20450748 -0.060059277 1.082976224 -0.055406447 0.95581466
2.711109908
6053 ZNF282 4.682660515 -0.721140277 1.267715025 -0.52725922
0.598012582 4.425201012
6054 ZNF461 7.929091044 0.224254746 1.018027709 0.220281768
0.825572844 1.448214048
6055 ZNF469 2.740799779 0.214629599 1.858997807 0.16925227
0.865598217 1.140157495
6056 ZNF497 9.596579297 -0.78568824 0.924409659 -0.849925126
0.295261155 2.447484719
6057 ZNF500 10.14826224 -0.167940226 0.912424822 -0.182855741
0.854126626 1.472744045
6058 ZNF620 5.459574022 0.581221729 1.20602694 0.481929258
0.629849097 4.911111417
6059 ZNF66 2.996968979 1.641256222 1.700525742 0.965146261
0.224471509 1.117574844
6060 ZNF71 11.09668492 -0.812175112 0.868717271 -0.924912049
0.249822112 2.709174541
6061 ZNF782 6.709181159 0.148146574 1.108506824 0.122645161
0.892682171 2.719011845
6062 ZNF788P 8.261048781 -0.821671226 0.989772577 -0.820160902
0.406447817 2.458774054
6063 ZNF790-AS1 7.196051501 -0.057446552 1.067550906 -0.052811524
0.95708522 1.97185545
6064 ABCB10 10.26772092 -0.508929128 0.885085224 -0.575017088 0.56527974
4.488425877
6065 ABCB5 4.71926864 0.201544229 1.208644209 0.154009928 0.8776019
4.151081149
6066 AC002102.1 4.702256682 0.209771721 1.220928557 0.158805055
0.872822466 2.171548115
6067 AC004076.2 2.968664499 0.566227208 4.280826406 0.122270527
0.894770211 1.111954112
6068 AC004466.1 5.222101 0.750152256 1.226247749 0.606798561

	0.542984602	2.954927711			
6069	AC006017.1	9.878015225	-0.528065414	0.905592829	-0.594157547
	0.552406744	1.472744045			
6070	AC007214.1	6.697828698	0.270762527	1.087228857	0.241012876
	0.722092126	1.94418508			
6071	AC007284.1	4.488517426	0.264424718	1.2715602	0.265700826
	0.79046962	4.488425877			
6072	AC007620.2	4.222942219	0.568750049	1.28586409	0.410292812
	0.681517084	2.908927074			
6073	AC007919.1	4.986729165	1.27166221	1.287261604	0.987881722
	0.222210578	1.995219481			
6074	AC008764.2	4.225222644	0.925297288	1.284288047	0.66845221
	0.502844914	1.110410191			
6075	AC008771.1	4.964146515	0.629567941	1.272485574	0.502612117
	0.615226205	2.118014851			
6076	AC008969.1	2.768951925	2.209274119	1.922710886	1.142504877
	0.25224422	1.957718978			
6077	AC009126.1	2.270790258	2.047644524	2.212292009	1.218018885
	0.187497226	1.145818188			
6078	AC009495.2	6.452910761	-0.626642419	1.172211494	-0.542649219
	0.587271275	2.101404854			
6079	AC010186.2	7.221418262	0.970259712	1.077546259	0.90042421
	0.267889169	4.920478405			
6080	AC015799.1	8.164120417	0.127959087	0.990780025	0.129242902
	0.889258207	1.11884417			
6081	AC015908.2	4.222942219	0.568750049	1.28586409	0.410292812
	0.681517084	2.959471944			
6082	AC019205.1	4.49418812	1.044815622	1.256596508	0.770174194
	0.44119657	1.944125019			
6083	AC020900.1	14.76089529	-0.844127222	0.76450427	-1.10416272
	0.269522554	1.71011588			
6084	AC024075.2	5.256974976	1.717686202	1.219816058	1.201458785
	0.192101462	2.709114499			
6085	AC025162.1	6.205297662	0.162096227	1.121166087	0.142200112
	0.886052167	1.47451922			
6086	AC024102.5	6.222259681	0.876689822	1.142250967	0.767442508
	0.442817869	2.972849478			
6087	AC048241.1	4.958475821	0.051548849	1.275642681	0.040410069
	0.967766202	1.940847082			
6088	AC072222.1	4.222298298	0.562708805	1.285464716	0.406872448
	0.684100929	2.111111894			
6089	AC072641.1	2.745527956	1.048446578	1.489559121	0.702862684
	0.481517669	2.491211541			
6090	AC072957.2	2.480952828	1.290502921	1.675516184	0.829895852
	0.40659767	2.709174541			
6091	AC079447.1	7.922077889	0.04849484	1.001226267	0.048424957
	0.961269601	2.42901875			
6092	AC087190.1	14.7825281	-0.227012478	0.781880729	-0.421027989
	0.666448019	1.121701514			
6093	AC087284.1	4.747521984	1.52171908	1.229941628	1.142122726
	0.252987221	4.405414102			

6094	AC087289.5	8.124575748	-0.75445029	1.002542105	-0.75178672	0.452179222	4.170714904
6095	AC087292.2	5.47076416	0.57827699	1.205885425	0.479545547	0.621550574	1.717017827
6096	AC089999.2	8.27785954	-1.014408787	1.000512088	-1.012889587	0.210625441	1.479244477
6097	AC090971.2	6.205297662	0.162096227	1.121166087	0.142200112	0.886052167	2.704429114
6098	AC091122.1	5.960469849	0.524664505	1.152079822	0.462682862	0.642874261	9.11184897
6099	AC092129.2	8.946554444	-0.2852276	1.0116065	-0.282062827	0.77789457	4.422221959
6100	AC092597.1	2.502485427	0.890248854	1.582091781	0.562766879	0.572592627	1.109118844
6101	AC099778.1	5.197682002	-0.084266456	1.248075165	-0.067517122	0.946170024	4.457924725
6102	AC099811.1	5.994242825	1.221454888	1.20222204	1.098172264	0.272129275	1.459417272
6103	AC104129.1	4.71926864	0.201544229	1.208644209	0.154009928	0.8776019	1.481149941
6104	AC104216.1	7.648996722	-0.417712259	1.022028907	-0.408708849	0.682752229	1.711407105
6105	AC115222.1	6.458621516	0.494689299	1.110022125	0.445652822	0.655848049	2.481258704
6106	AC124282.1	5.92942122	0.056424252	1.167681586	0.048221692	0.961459866	1.974490997
6107	AC124219.2	2.502485427	0.890248854	1.582091781	0.562766879	0.572592627	1.707180522
6108	AC122550.2	5.484890822	1.146087201	1.22529524	0.925255879	0.249604927	1.140157495
6109	AC242965.2	4.702256682	0.209771721	1.220928557	0.158805055	0.872822466	1.92519429
6110	ACBD4	12.11265427	-0.742092694	0.845198757	-0.878009685	0.279928462	1.742989854
6111	ACTA2-AS1	4.720610027	1.542896768	1.26929281	1.12742162	0.259560022	1.95777904
6112	ADAP1	6.858006602	-1.260414224	1.194041041	-1.055587019	0.291156924	7.414819947
6113	AF117829.1	5.202202625	-0.265921014	1.272657282	-0.287522027	0.772704209	1.71419149
6114	AF129408.1	4.218221687	0.926898187	1.422128927	0.652728561	0.512280255	2.710545884
6115	AIDA	7.407004265	0.055072088	1.047452792	0.052578109	0.958068059	1.11884417
6116	AJ002147.2	7.916407195	-0.215482998	1.002986089	-0.214221442	0.752245262	1.479214415
6117	AKT1S1	5.479220129	0.574255662	1.217202767	0.471864842	0.627022264	1.454781014
6118	AL049840.6	7.427992822	-0.148164567	1.041279692	-0.142277181	0.88686107	4.449141149
6119	AL122656.4	7.91262191	0.050817066	1.002280121	0.050645876		

						0.959607706	1.728249114
6120	AL122989.1	2.985576456	0.544762545	1.806081511	0.20162722	0.762926171	1.971905711
6121	AL129095.2	8.289200927	-0.289181522	0.976767895	-0.296059609	0.767184559	1.97185545
6122	AL157928.2	5.757921928	0.92726842	1.25027458	0.694205781	0.487552167	1.711951117
6123	AL160408.2	8.625572762	-0.522981057	0.96612458	-0.551669078	0.581175099	1.48502527
6124	AL162424.1	10.24220292	-0.950806278	0.89166721	-1.066224029	0.286277191	2.108177547
6125	AL255490.2	4.961211168	0.242259022	1.267749694	0.270052527	0.787119826	4.187488914
6126	AL259740.1	9.875280012	0.1985476	0.925242798	0.212295246	0.821876704	1.949070245
6127	AL291822.2	7.950281171	0.222587422	1.020082895	0.218205221	0.82726921	2.471545129
6128	AL606760.2	12.82102868	-0.268222007	0.792272417	-0.464192051	0.64250944	1.100441887
6129	AL721562.2	9.647214094	-0.026214285	0.917956898	-0.028666144	0.977120858	4.474559144
6130	ALDH16A1	11.81141999	-0.816551609	0.827217614	-0.97521585	0.229402611	1.974741059
6131	ALG10	6.424807602	0.507951222	1.165141724	0.425956607	0.662868212	1.479244477
6132	ALOX12	7.429586915	0.429227256	1.044279449	0.420668228	0.672997292	4.419481291
6133	ANKRD20A11P	7.457790197	0.629927506	1.047045077	0.601624056	0.547424412	1.92519429
6134	ANKRD22B	5.70425071	0.692462645	1.192246989	0.580217949	0.561700226	1.104182519
6135	ANO7	4.950019842	0.055295945	1.280589022	0.042180086	0.965557979	1.448214048
6136	AP002068.2	5.456627488	0.049114122	1.209265045	0.040611502	0.967605614	1.459417272
6137	APOBEC2	2.26149296	2.510521057	1.742644298	1.440644494	0.149685144	1.459417272
6138	APOBEC2B-AS1	4.720509902	-0.418650116	1.422202292	-0.294267281	0.768477266	1.141991841
6139	ASB7	11.2217142	-0.857028255	0.852729759	-1.00502908	0.214882847	2.185494897
6140	ASH1L-AS1	7.902280524	-0.688825262	1.015112966	-0.678579907	0.497404078	1.148412472
6141	ATG4D	8.1922227	0.20728752	0.998107862	0.207870062	0.758181207	1.998145018
6142	ATP5PBP1	5.462208182	0.582157524	1.205984854	0.482722752	0.629291896	1.474579291
6143	B2GNT10	5.47076416	0.57827699	1.205885425	0.479545547	0.621550574	1.499141041
6144	BACH1-IT1	2.748262202	1.510429626	1.506114506	1.002865068	0.215925969	2.444444748

6145	BCAS4	2.979114506	1.198299685	1.472229457	0.812282925	0.415998546	2.441541229
6146	BCDIN2D-AS1	12.08161574	-0.998884272	0.825122816	-1.196092664	0.2216604	1.982197027
6147	BCL2L12	12.77864866	-1.029956982	0.782792851	-1.214066168	0.188822928	1.494214495
6148	BCL7A	8.651040821	0.471006656	1.004184211	0.469044029	0.629028165	5.445514445
6149	BEND2	2.996026462	1.185264204	1.441499752	0.822242779	0.410928167	1.721448449
6150	BRICD5	6.956792184	0.460555064	1.074448842	0.428642082	0.668182988	2.428978489
6151	BTBD2	12.26887262	-0.070211214	0.825666052	-0.084018287	0.922041812	2.188520142
6152	C10orf142	4.222942219	0.568750049	1.28586409	0.410292812	0.681517084	2.444294484
6153	C11orf71	5.189227025	-0.080794742	1.256488861	-0.064201997	0.948729762	1.942299471
6154	C18orf22	6.292718912	-0.61920105	1.155974127	-0.525652025	0.592198267	1.459417272
6155	C1orf25	12.08082448	-0.448252154	0.806897245	-0.555526926	0.578524291	1.49911198
6156	C2	5.227227672	1.270784812	1.262424125	1.084975959	0.277922202	1.44145171
6157	C22orf46	7.944610478	-0.129625726	1.027152201	-0.125924807	0.891872819	5.412149279
6158	C2orf76	10.85459127	-0.619908097	0.867296727	-0.714676546	0.474808924	2.185494897
6159	C5orf24	8.297706967	0.224798847	0.998020282	0.225242488	0.821790628	2.478125921
6160	CABLES2	5.974596521	1.048485165	1.170022604	0.896116195	0.270190708	1.110410191
6161	CAPN10-DT	5.949178524	0.292222084	1.15184974	0.252794461	0.799654252	1.982197027
6162	CASD1	5.672212082	0.1877987	1.224217802	0.152147769	0.879070288	4.914551722
6163	CASQ1	8.125917125	-0.021128268	1.012494104	-0.020754122	0.975465628	1.15144881
6164	CBX7	14.01790591	-0.726899852	0.784944912	-0.926052059	0.254418926	2.119548871
6165	CCDC102B	7.694162022	0.225698708	1.022922992	0.218400026	0.750181518	1.451141294
6166	CCDC175	4.722295212	0.825460495	1.204811457	0.622628178	0.526976481	2.702402848
6167	CCNJP2	5.922760527	-0.421212192	1.177650795	-0.266162887	0.714242787	1.121701514
6168	CCSAP	9.106822472	-0.498277887	0.949191491	-0.525055157	0.599544848	1.725522877
6169	CCT6P1	4.972602492	0.62520999	1.265527696	0.501922902	0.61571472	2.191215518
6170	CEBPA	2.48657247	0.90408979	1.551582601	0.582688791	0.56010282	

							4.114714214
6171	CEP72	7.708288704	0.707691687	1.025460706	0.682455859	0.494218822	1.140157495
6172	CFAP157	11.12482708	-0.407782028	0.86455072	-0.471670462	0.627162022	1.111954112
6173	CFAP44	2.224782628	0.284252622	1.744511828	0.162998965	0.87051925	5.481172914
6174	CHAC1	6.17709428	-0.060961489	1.128048441	-0.052566691	0.957280295	1.441705414
6175	CHADL	6.219274272	-0.075592552	1.172690012	-0.064460812	0.948602209	2.709114499
6176	CHEK1	12.09211581	-0.227679887	0.810527622	-0.416617271	0.676958201	7.084278288
6177	CHST7	4.969767147	0.228251877	1.267559286	0.266921775	0.789521691	1.429870049
6178	CKLF	2.728615999	1.061849879	1.52229028	0.69748861	0.485497065	1.127211148
6179	CLASRP	12.25952627	-0.27909087	0.814125059	-0.242806598	0.721742962	1.441705414
6180	CLUAP1	7.210127027	0.756812179	1.064519909	0.710942189	0.477119441	1.111954112
6181	COPRS	7.62487006	-0.809874	1.040127205	-0.778622202	0.426202296	4.145054111
6182	CPLANE1	8.626864087	-0.264280169	0.961896245	-0.278710459	0.704902886	1.944125019
6183	CRLF1	5.962255124	0.04296885	1.167506129	0.027660488	0.96995828	1.704445184
6184	CRNDE	6.891880579	-0.571542649	1.097001982	-0.521005119	0.602262205	4.914551722
6185	CSNK1E	9.2854742	-0.721691701	0.924627725	-0.782869672	0.422702649	1.104182519
6186	CXCR2	8.172626458	0.675712219	1.022218106	0.660214926	0.509051754	4.174247528
6187	CYP4F2	11.11628097	-1.257670969	0.90192684	-1.294411252	0.162192422	2.72180715
6188	DAPK2	19.42421566	-0.897442085	0.696412576	-1.288662972	0.197514922	1.940847082
6189	DBF4B	9.118062726	-0.824922278	0.94572206	-0.882841197	0.277222078	11.19584085
6190	DCDC2B	5.459472825	0.212248401	1.202460826	0.260289654	0.794640252	1.492491249
6191	DCLRE1A	8.262722868	-1.422848271	1.045046922	-1.261516145	0.172250625	4.281822452
6192	DCTN1-AS1	4.708027276	0.840442084	1.264200452	0.616024925	0.52787807	4.147891559
6193	DDX11L2	2.766116578	1.452626528	1.917826098	0.757422919	0.448789941	4.924414299
6194	DDX51	6.148841027	-1.020625785	1.226204964	-0.840440022	0.400661711	7.281041195
6195	DECR2	10.8490207	-0.078122092	0.889022812	-0.087886488	0.929966896	5.454158049

6196	DET1	8.420289618	0.567088122	0.998564786	0.567902195	0.570100702	2.478472194
6197	DHX57	11.06842051	-0.667415771	0.86672984	-0.77002899	0.441276764	4.184802514
6198	DIAPH2	8.428745596	0.564522265	0.990248842	0.570022652	0.568661656	4.419481291
6199	DLGAP1-AS1	10.58996609	-1.002121254	0.882427667	-1.12424291	0.256650726	4.474844491
6200	DNAJC27	4.74185129	0.820556921	1.221792279	0.62079062	0.524727296	1.982144974
6201	DSC2	9.124975692	-0.828224511	0.950698294	-0.881692505	0.277942574	1.498814555
6202	DYNC2LI1	12.59962484	-0.122450226	0.827072571	-0.148052299	0.882201415	5.424410441
6203	EDRF1-AS1	5.676097267	-0.222578772	1.204111681	-0.267897719	0.78877805	1.11884417
6204	EEF1B2P1	5.42559886	-0.472646269	1.251254555	-0.278506929	0.705054048	4.444290714
6205	EIF1B-AS1	8.92122657	0.207045148	0.954682722	0.216872024	0.828207201	1.725482815
6206	EMID1	8.92122657	0.207045148	0.954682722	0.216872024	0.828207201	1.998145018
6207	EML5	8.1922227	0.20728752	0.998107862	0.207870062	0.758181207	2.475728072
6208	ENTR1	5.712806689	0.688587605	1.18182719	0.58264661	0.560121222	7.104075419
6209	EPB41L5	4.221106972	0.222450475	1.297899221	0.159121972	0.8725649	1.949070245
6210	ERCC6L	2.25298692	0.721242626	1.66855767	0.422255728	0.665555552	4.952141054
6211	ERCC8-AS1	7.425207547	0.241124954	1.02249252	0.222220225	0.815512747	2.481208442
6212	ERO1B	6.288048218	-1.112171522	1.210204281	-0.918918945	0.25812797	1.474579291
6213	ETAA1	12.81814227	-0.8027656	0.790472595	-1.016816528	0.209240718	2.971014221
6214	FAM111A-DT	6.444554906	0.722515788	1.124472781	0.646569185	0.517910807	1.111954112
6215	FAM118A	7.406954202	-0.524849857	1.04187496	-0.512252212	0.607704268	2.441541229
6216	FAM151B	4.499808762	0.690275254	1.299675716	0.492168058	0.621892846	1.949212041
6217	FAM92A	9.106822472	-0.498277887	0.949191491	-0.525055157	0.599544848	1.004411007
6218	FBRSL1	4.222942219	0.568750049	1.28586409	0.410292812	0.681517084	1.11884417
6219	FBXL2	2.48657247	0.90408979	1.551582601	0.582688791	0.56010282	2.101404854
6220	FBXO48	7.712859274	-0.056416288	1.079471226	-0.052262887	0.958219224	1.121701514
6221	FTH1P10	2.50999744	2.054166761	1.960066792	1.048008552		

						0.294624667	2.918484277
6222	FTH1P12	5.70425071	0.692462645	1.192246989	0.580217949	0.561700226	2.188520142
6223	FTH1P7	5.726882299	0.421009527	1.195826241	0.252062869	0.724791114	2.444449281
6224	GAPDHS	4.226727604	-0.122762941	1.447201876	-0.084822622	0.922402408	4.414875981
6225	GGNBP1	8.182867721	0.209727825	0.992970201	0.211920542	0.75510091	5.441274541
6226	GID4	12.29991226	0.157087786	0.854086945	0.182924818	0.854072425	1.71419149
6227	GIPC1	10.26492564	-0.225426286	0.889597625	-0.252402524	0.799957178	5.100914114
6228	GPATCH11	7.958787211	0.781474296	1.025009062	0.762407204	0.445816949	7.117417014
6229	GPLD1	4.981058472	0.620860725	1.272086845	0.495925821	0.619946741	2.444244414
6230	H1-4	6.45579617	0.268597881	1.109548928	0.242078444	0.808719281	1.11884417
6231	HENMT1	8.147108227	-0.952024966	1.017822696	-0.926227718	0.249104481	1.121701514
6232	HEXD	11.07972184	-0.522256807	0.864622652	-0.616858719	0.527227918	2.714845121
6233	HNRNPA1P10	6.921275186	-0.158057029	1.068951082	-0.147861798	0.882451827	1.429870049
6234	HSCB	12.85852926	-0.404276857	0.821542072	-0.492094525	0.622652515	2.185494897
6235	IFIH1	17.02507919	-0.506272772	0.741696278	-0.682722805	0.494781285	1.452944479
6236	IFT22	6.15451172	-0.526959598	1.170222465	-0.450202249	0.652491724	1.481149941
6237	IFT42	8.175261681	-0.218424667	0.994221721	-0.219704179	0.82610155	4.447105925
6238	IKBIP	7.204456244	0.242871271	1.058645461	0.224822022	0.745215768	1.715724511
6239	IL1R1	2.26149296	2.510521057	1.742644298	1.440644494	0.149685144	1.109118844
6240	IL22R	6.464252148	0.26552201	1.12057825	0.22695979	0.812687985	1.47451922
6241	INAFM2	4.254980948	1.202781959	1.421241558	0.91088109	0.26225802	1.48502527
6242	INCENP	5.946242177	0.050184871	1.152991781	0.042525784	0.965282411	1.944487715
6243	INF2	9.682972254	0.114449622	0.942697124	0.121277918	0.90247091	1.94418508
6244	INSR	9.79902602	-1.16506029	0.947824804	-1.229180745	0.219004047	8.54290127
6245	INTS6-AS1	4.722295212	0.825460495	1.204811457	0.622628178	0.526976481	4.190474109
6246	KCNJ2	2.7576606	1.462857676	1.816618277	0.80581468	0.420249712	5.141419408

6247	KIAA0895L	8.427201575	0.561982112	0.987772452	0.568929275	0.569297251	1.470411172
6248	KIF1C	8.884577209	0.054199526	0.967470084	0.056021924	0.955224241	2.927190418
6249	KIF4A	9.157658467	0.444069256	0.95920422	0.462907629	0.642420572	1.472492982
6250	KLC4	7.426751569	0.242729425	1.026180268	0.225228785	0.814021166	1.124484801
6251	KLHDC10	7.406954202	-0.524849857	1.04187496	-0.512252212	0.607704268	1.111954112
6252	KLHL11	9.444766172	0.191002984	0.965100621	0.197910951	0.842114726	1.725522877
6253	KLRB1	4.964146515	0.629567941	1.272485574	0.502612117	0.615226205	5.411881952
6254	LAMB2	10.80102005	-0.751682512	0.891209205	-0.842442267	0.298981141	1.741154509
6255	LDOC1	2.996026462	1.185264204	1.441499752	0.822242779	0.410928167	1.448072251
6256	LINC00224	5.92942122	0.056424252	1.167681586	0.048221692	0.961459866	4.299745409
6257	LINC00620	4.699521226	-0.095729265	1.222145287	-0.071868486	0.942706569	1.124524842
6258	LINC00654	4.491252782	0.695521475	1.25824576	0.512025665	0.608626049	1.949070245
6259	LINC00862	12.51974424	-0.755786218	0.797604294	-0.947570522	0.242248129	4.192209554
6260	LINC01127	9.120899082	-0.666155692	0.940445244	-0.708240754	0.478722674	1.718571858
6261	LINC02202	6.455846221	0.971291768	1.126998292	0.854259655	0.292961126	4.908095755
6262	LOXL2	4.242689622	0.91980757	1.294658428	0.659521747	0.509560786	1.000950212
6263	LRIG1	8.414568862	-0.295221262	0.977141264	-0.202127617	0.76255478	1.977514244
6264	LYSMD1	8.42591025	0.289891678	0.982528748	0.296824702	0.691496722	2.194984111
6265	MANEA	6.292718912	-0.61920105	1.155974127	-0.525652025	0.592198267	1.117574844
6266	MAP1LC2B2	8.670728062	0.129289982	0.96425929	0.12408216	0.892227606	1.951158408
6267	MAP2K7	2.7576606	1.462857676	1.816618277	0.80581468	0.420249712	2.954101274
6268	MAP4K2	6.886209885	-1.02269268	1.120215701	-0.904785874	0.265578827	4.118477491
6269	MEF2D	9.625972707	-0.622497952	0.921012249	-0.686742172	0.492245217	2.478512258
6270	MFGE8	8.42148082	-0.299221086	0.995270614	-0.200752465	0.762602496	1.495989108
6271	MGAT2	6.976590549	1.269201797	1.111499659	1.221940816	0.217971192	1.00278544
6272	MICAL2	5.676097267	-0.222578772	1.204111681	-0.267897719	0.78877805	

5.459591174

6273	MIR4452HG	10.12851274	-0.450888607	0.895491407	-0.502509697	0.614605964	1.429870049
6274	MME	8.11611977	-0.752548995	1.009265652	-0.745566281	0.455929458	4.91108281
6275	MNT	12.60161886	0.099252255	0.815271886	0.121862952	0.902006764	1.977514244
6276	MORN2	11.62588415	0.108945252	0.858882802	0.126845275	0.899062861	2.951147018
6277	MPND	6.484049514	1.212772182	1.144000869	1.060988864	0.288694962	4.410984472
6278	MPZL1	6.194006227	-0.066745751	1.121681895	-0.058979251	0.952968624	4.444255984
6279	MTHFSD	14.76925127	-0.84516642	0.762588485	-1.106824947	0.268265296	1.977514244
6280	MYLIP	7.922077889	0.04849484	1.001226267	0.048424957	0.961269601	5.445801091
6281	MYOF	2.759654628	2.045940177	1.561208217	1.210485061	0.190021795	2.444294484
6282	NAA80	12.79571081	-0.049209126	0.829865256	-0.059418224	0.952618998	1.495989108
6283	NEURL4	5.47076416	0.57827699	1.205885425	0.479545547	0.621550574	1.470908498
6284	NFKBIL1	7.440778117	-0.542602004	1.072250206	-0.506454465	0.612527648	4.914551722
6285	NHSL1	11.60220128	-0.910179242	0.854865845	-1.064704201	0.287009762	1.459417272
6286	NINJ2-AS1	8.622787477	-0.192245228	0.981285609	-0.196012614	0.84459951	4.194144902
6287	NOL12	10.08901912	-0.741670772	0.900525488	-0.822588611	0.410172249	1.977514244
6288	NPM1P27	9.408106912	0.046692744	0.929864224	0.050215657	0.959950524	2.119518811
6289	NRF1	9.869559257	-0.526442787	0.90424226	-0.592250522	0.552012505	4.425251085
6290	NSMCE2	6.662964722	-0.267954202	1.097189706	-0.244218752	0.807061404	1.718571858
6291	NUDT12	9.900547824	-0.695290425	0.929867617	-0.747720561	0.454622692	2.452155411
6292	OLA1P1	9.171785129	0.768050812	0.974492129	0.788154158	0.42060654	1.448214048
6293	PABPC1P1	6.46420221	0.967550787	1.125977726	0.859298227	0.290175942	2.700818582
6294	PABPC5	8.12870242	-0.288224891	0.992002275	-0.291455645	0.695460468	1.11884417
6295	PARD6B	2.987570484	1.191669502	1.442098077	0.826244284	0.40860882	1.110410191
6296	PCAT18	4.499858822	1.856655018	1.400244612	1.225950482	0.1848561	2.478125921
6297	PDE2A	7.415460242	0.052552589	1.027128242	0.050670761	0.959587876	2.112898179

6298	PGGHG	11.2827415	-0.729901777	0.879097455	-0.820285295	0.406277444	1.000950212
6299	PHBP19	8.707247262	-0.212080841	1.04428092	-0.20402598	0.828222196	1.47451922
6300	PHF12	15.49264261	-0.748204522	0.756082078	-0.989712048	0.222214884	1.711407105
6301	PHLDA2	4.722445272	1.970512596	1.46862201	1.241742287	0.179679571	1.487870717
6302	PHLDB2	5.724098014	0.955725422	1.189268846	0.802622782	0.421609085	2.974485015
6303	PIGX	8.195209108	1.051558082	1.020174749	1.020756997	0.207269582	1.928021724
6304	PIP5K1B	2.455524841	0.069917168	1.742171612	0.040122192	0.967987726	5.17555418
6305	PITPNC1	5.186291678	-0.25942195	1.272016497	-0.282246655	0.777677715	1.92519429
6306	PKN2	6.205247724	0.884208689	1.152519028	0.766521516	0.442260107	4.194144902
6307	PLB1	15.7092182	-0.682985167	0.774220262	-0.882147241	0.277697124	1.998145018
6308	PLXNB1	8.617066722	-1.074952296	0.988588772	-1.087261425	0.276877112	1.000950212
6309	PMCH	2.7576606	1.462857676	1.816618277	0.80581468	0.420249712	1.971905711
6310	POC1B-AS1	4.474440826	0.705441207	1.241224844	0.525928682	0.59892776	1.707180522
6311	POLR2J2	7.187544287	0.249422682	1.052169472	0.222106844	0.729808582	2.927190418
6312	PPM1L	7.654667425	-0.029576484	1.024540962	-0.028628502	0.969186577	4.47171291
6313	PRC1-AS1	6.46420221	0.967550787	1.125977726	0.859298227	0.290175942	1.124524842
6314	PRDM10	8.91272052	-0.277257017	0.954212909	-0.290520206	0.771410645	9.45219052
6315	PRORSD1P	4.981058472	0.620860725	1.272086845	0.495925821	0.619946741	1.701459901
6316	PRRT2	5.496182158	1.458416955	1.252050818	1.164822492	0.244090852	1.47451922
6317	PSMG2-AS1	8.442722082	-0.654776114	1.049202222	-0.624010927	0.522620409	4.448888572
6318	PSTK	8.628408109	-0.262248574	0.966591629	-0.274872448	0.70775522	4.152814949
6319	PTGES2L	5.211759612	-0.269188779	1.282571156	-0.287626266	0.772622826	4.114248185
6320	QPCTL	6.444504844	0.048241694	1.10707789	0.0425757	0.965242622	1.124524842
6321	RAB11FIP2	6.45579617	0.268597881	1.109548928	0.242078444	0.808719281	5.198088849
6322	RAB11FIP2	4.9669218	0.047816852	1.28018807	0.027251427	0.970204801	2.455940707
6323	RAPH1	12.84455277	0.287880296	0.866224262	0.447725859		

						0.654251057	1.498814555
6324	RBM26-AS1	2.976279159	0.794410872	1.441828205	0.550970847	0.58165266	1.718571858
6325	RECK	12.22654268	-0.416219029	0.826580927	-0.502662962	0.614497527	1.47451922
6326	RELL1	8.650890626	-1.081688229	1.007861601	-1.072250859	0.282158577	2.444294484
6327	RIOX1	8.155614277	-0.292276044	0.988760845	-0.296826147	0.691488292	2.712251171
6328	RNF207	8.721522996	0.621629755	1.027177527	0.60519212	0.545051244	2.119548871
6329	RNF212-AS1	7.927698521	-0.125071266	1.005254424	-0.124251888	0.892124224	1.114020812
6330	ROBO2	12.82669927	-0.158672292	0.800889825	-0.198120124	0.84295108	4.705547824
6331	RPL7P46	2.742692609	0.621704125	1.499628257	0.421227648	0.672581555	2.101454915
6332	RPS26P15	6.94555192	0.902707049	1.10425222	0.817407902	0.412695261	1.71011588
6333	RPS2AP5	9.900597886	-0.245052858	0.910827585	-0.269042221	0.787897115	1.148442525
6334	RPS4XP22	5.225926247	1.051592069	1.2482024	0.842417725	0.299554175	1.714141551
6335	RPUSD2	12.78222522	-1.120248442	0.811121617	-1.281110229	0.16724504	1.501947217
6336	SCLY	5.224292225	1.046926512	1.248121022	0.828802075	0.401580286	2.424192404
6337	SEC62P1	4.228068991	1.216471105	1.402266026	0.928749999	0.247859116	2.917292051
6338	SERPINF1	2.722945205	0.249069642	1.522284611	0.16260494	0.870042155	1.454781014
6339	SGIP1	6.905957189	-0.796669442	1.100660965	-0.722810028	0.469182272	1.44145171
6340	SH2BP5	7.615122756	-1.014299792	1.064916451	-0.952468892	0.240859225	1.717017827
6341	SHC4	2.269948929	2.498217827	1.799246109	1.288402167	0.165014212	4.145054111
6342	SIMC1	9.681188069	0.415759742	0.925649894	0.444252967	0.656786689	1.949172
6343	SLC16A7	2.72422662	0.627590691	1.489218562	0.428109007	0.668571759	1.470908498
6344	SLC22A18AS	8.92680714	-0.44501955	0.980126648	-0.454042905	0.649797946	2.714184518
6345	SLC25A15	18.71792528	-0.98277782	0.702696985	-1.298012422	0.162109026	1.995219481
6346	SLC45A4	7.654667425	-0.029576484	1.024540962	-0.028628502	0.969186577	1.451141294
6347	SMCR5	9.441920826	0.0285561	0.966918822	0.029875219	0.968192608	1.710045818
6348	SNAPC4	6.700622982	-0.062629102	1.098019226	-0.057028255	0.954514721	2.447121022

6349	SNED1	4.726180597	0.192228274	1.245282054	0.142622422	0.885797844	1.974490997
6350	SNHG19	4.729066005	1.52769966	1.240266229	1.147222509	0.251289258	1.974490997
6351	SNHG20	6.427592887	0.052957928	1.11229412	0.048462559	0.961247602	1.100441887
6352	SNTB2	2.72422662	0.627590691	1.489218562	0.428109007	0.668571759	2.727477842
6353	SNX16	6.655508744	-0.265259017	1.107524927	-0.229594265	0.810644811	5.890191417
6354	SNX24	8.275024192	-1.212522604	1.019201877	-1.190552702	0.222829229	4.411115412
6355	SOX6	5.476424854	1.150682745	1.225442482	0.928992678	0.24772299	1.985981211
6356	SPDYE21	5.721212606	-0.084275612	1.217247202	-0.069228904	0.944807416	5.484959111
6357	SPDYE5	7.167747021	-0.452512512	1.057928165	-0.428679872	0.66815621	1.479214415
6358	ST20-AS1	8.20082974	0.854524157	1.011985016	0.844412842	0.298428189	1.114789559
6359	STAR	8.682029288	0.292297977	0.969994566	0.202270742	0.762269456	1.479214415
6360	STK11	4.240854276	0.558615291	1.4026762	0.29824964	0.690446178	2.119548871
6361	STX5	7.947545948	1.191095942	1.059770648	1.1229186	0.26104756	4.451974414
6362	SUFU	17.79508075	-0.115929712	0.74802962	-0.154992478	0.876826459	1.445188044
6363	TBL1X	9.122190408	-0.502701822	0.927096467	-0.527512207	0.590912095	1.140157495
6364	TFEB	6.219424225	0.620218191	1.127612912	0.554070297	0.579520669	4.449478757
6365	TH2LCRR	2.241745657	1.804085164	1.746275811	1.022045209	0.201582742	5.109220122
6366	THAP9	5.448121448	-0.75979522	1.296818754	-0.585891689	0.557948202	2.444449281
6367	THTPA	9.149152427	-0.029084765	0.928655902	-0.020985545	0.975281067	1.942451248
6368	TMCC1	12.55928891	-0.212491691	0.809841928	-0.262286622	0.792022285	1.714141551
6369	TPGS1	5.952012871	0.528186644	1.157824218	0.464821811	0.642059067	4.129488177
6370	TRIM17	7.190229672	-0.054066867	1.054260169	-0.051279214	0.959102951	4.915057772
6371	TRIM26	6.921275186	-0.158057029	1.068951082	-0.147861798	0.882451827	4.194094841
6372	TRIM8	14.05172982	-0.721228004	0.780679181	-0.926797116	0.248862929	2.120840198
6373	TSC22D4	12.41602296	0.581066909	0.85468141	0.679862751	0.496590726	1.445128005
6374	TSHZ2	11.08817782	-0.524811521	0.8608118	-0.621287295		

						0.524410546	2.700818582
6375	TSPAN2	10.92527469	1.150265274	0.927177145	1.227272414	0.21968265	1.451141294
6376	TST	9.658555257	-0.229029674	0.927251227	-0.254854925	0.722698271	1.744815101
6377	TTBK2	12.28704907	-0.652720254	0.827098854	-0.779752894	0.425526228	7.419405121
6378	TXK	9.599212446	-0.78492454	0.921775667	-0.851546171	0.294466027	1.100441887
6379	U2AF1L4	9.22628225	-0.720067084	0.959817207	-0.750212727	0.452126595	4.107424118
6380	U91228.1	7.178988285	-0.882086945	1.106286828	-0.797240186	0.425252487	1.145818188
6381	UCKL1-AS1	7.440828179	0.04511295	1.027411502	0.042487025	0.965212299	1.707180522
6382	USP12	2.981899791	0.412560628	1.442122674	0.286078525	0.774817971	1.140107422
6383	UTP14C	7.404118856	-0.728880145	1.052269712	-0.701444267	0.482025804	2.124480819
6384	VPS27C	7.284421614	-0.229272618	1.0612482	-0.210264221	0.756282916	1.452944479
6385	WASH6P	11.89881512	-0.699917041	0.892426722	-0.78229856	0.422292102	5.915710414
6386	YEATS2-AS1	8.275074255	-0.629464762	0.982195677	-0.650294198	0.515427624	2.444449281
6387	YWHAZP4	6.450125476	-0.177658268	1.121849241	-0.158262056	0.874171511	1.121701514
6388	Z74021.1	10.24220292	-0.950806278	0.89166721	-1.066224029	0.286277191	1.454781014
6389	Z97822.2	9.294020202	0.202566892	0.940262452	0.216500077	0.828597965	4.442555247
6390	ZBED5-AS1	6.891880579	-0.571542649	1.097001982	-0.521005119	0.602262205	1.112145528
6391	ZBTB11-AS1	9.299650924	0.04866222	0.9296927	0.052242446	0.958255026	2.119548871
6392	ZC2H6	7.16212629	-0.247527466	1.052422859	-0.224981498	0.814222094	1.481100012
6393	ZFAT	9.911889211	-0.101248207	0.916844077	-0.110421225	0.912067211	1.145818188
6394	ZGPAT	4.480061458	0.269028208	1.249685069	0.272418086	0.784521852	1.112145528
6395	ZHX2	9.227522614	-1.062247284	0.948022257	-1.120581251	0.262466149	1.140157495
6396	ZIK1	7.284421614	-0.229272618	1.0612482	-0.210264221	0.756282916	1.145778117
6397	ZMAT1	10.88274449	-0.909908872	0.898666267	-1.012510222	0.211294114	1.470908498
6398	ZMYM4-AS1	2.495029449	0.89728562	1.550786191	0.578600477	0.562858782	1.492491249
6399	ZMYND19	4.972602492	0.62520999	1.265527696	0.501922902	0.61571472	1.944125019

6400	ZNF219	7.404118856	-0.728880145	1.052269712	-0.701444267	0.482025804	1.454781014
6401	ZNF222	6.666750007	-0.71524566	1.117897272	-0.629902857	0.522225756	1.951158408
6402	ZNF251	9.129255061	-0.66785757	0.940578142	-0.710050062	0.477672092	4.701741549
6403	ZNF284	9.149152427	-0.029084765	0.928655902	-0.020985545	0.975281067	1.715724511
6404	ZNF428	5.704200649	-0.077882512	1.186872527	-0.065619946	0.947680408	1.492152841
6405	ZNF451-AS1	9.426210194	0.192181209	0.950756581	0.202186928	0.82898892	2.157491414
6406	ZNF502	7.167797082	0.152650449	1.059978076	0.144956252	0.884745412	2.14878194
6407	ZNF514	8.420229556	0.047098557	0.975022291	0.048205108	0.961472084	4.480179898
6408	ZNF519	9.246029654	-0.561851892	0.941702785	-0.596622992	0.550751771	1.704495148
6409	ZNF527	5.927887199	0.052281247	1.156592874	0.046067278	0.962256547	2.481011117
6410	ZNF626	2.019450422	2.260957071	1.858561524	1.27021418	0.202972729	2.447181971
6411	ZNF629	11.79724228	-0.682674892	0.848242584	-0.805988876	0.420249262	1.47451922
6412	ZNF707	7.429586915	0.429227256	1.044279449	0.420668228	0.672997292	1.472744045
6413	ZNF746	9.894977254	-0.097599289	0.907588178	-0.107526977	0.914262992	2.912042744
6414	ZNF772	5.925051852	-0.188786792	1.161245247	-0.162572701	0.870854882	1.495989108
6415	ZNF792	11.90722116	-0.224945595	0.884800559	-0.267252927	0.712420262	1.980241491
6416	ZNF845	9.124925621	-1.288668052	1.005224842	-1.28121278	0.16718281	1.112145528
6417	ZSWIM6	5.925051852	-0.188786792	1.161245247	-0.162572701	0.870854882	5.191448127
6418	ZXDC	12.78992999	-0.914260697	0.782927052	-1.166257262	0.242510406	1.715724511
6419	A2MP1	11.27229904	-0.592046965	0.854227655	-0.694249216	0.48752587	1.719812111
6420	AC002247.2	7.674212521	-0.042509272	1.015597141	-0.042841174	0.965828142	4.485850591
6421	AC004264.1	8.462518274	0.919197291	0.997044128	0.921922476	0.256569014	5.445801091
6422	AC004542.1	6.444402646	-0.172992809	1.124296728	-0.154757906	0.877012178	2.111041821
6423	AC004696.1	7.422815024	-0.142617715	1.024866678	-0.127812646	0.89028849	2.945594224
6424	AC005206.1	6.92122405	0.26028062	1.074272996	0.242285125	0.808559221	4.440971714
6425	AC006059.1	9.25159915	-0.402119898	0.944721228	-0.426702219		

	0.669595506	1.711901145			
6426	AC006212.1	14.09957921	-0.628478068	0.792220079	-0.792202494
	0.428242614	1.971905711			
6427	AC006220.1	5.980065892	-0.202791978	1.250822211	-0.162125644
	0.871206912	1.110410191			
6428	AC006425.2	8.667801518	-0.201712815	0.961802929	-0.209722642
	0.822882272	4.114248185			
6429	AC007192.2	5.48757492	0.20211121	1.261615026	0.240256577
	0.810121256	5.490419904			
6430	AC007226.1	6.222158482	0.621125762	1.127599785	0.554787168
	0.579040245	1.117574844			
6431	AC007282.2	4.961260022	0.956180822	1.22882624	0.714192277
	0.475107612	1.000950212			
6432	AC008277.1	4.986627967	0.941277696	1.280449067	0.725192295
	0.462221721	1.711407105			
6433	AC008402.2	8.166854566	0.128642861	0.986676858	0.140515975
	0.888252221	1.714977775			
6434	AC008761.1	10.45227976	1.044092229	0.925425164	1.12822979
	0.259222886	4.101815594			
6435	AC009226.1	8.224552456	0.656760612	1.068125888	0.61486616
	0.528642112	2.714124454			
6436	AC010480.1	4.499757625	1.42617215	1.274484464	1.027605144
	0.299452922	1.498814555			
6437	AC011120.1	9.644277549	-0.224820921	0.911755274	-0.256269857
	0.721628481	5.411918115			
6438	AC012629.2	7.292776295	-0.529554295	1.060108901	-0.499528204
	0.617407222	2.711059844			
6439	AC015914.1	8.158298588	0.14096742	0.992449545	0.141896919
	0.887161428	1.110410191			
6440	AC016065.1	6.452909687	0.498700425	1.110000492	0.449279462
	0.652220071	1.472492982			
6441	AC018462.1	5.991257218	0.024852528	1.278646181	0.027258157
	0.97825282	1.148412472			
6442	AC018816.1	5.456526291	-0.212581604	1.252285984	-0.170552277
	0.86457496	1.124524842			
6443	AC020658.2	5.476282594	0.042280278	1.252702245	0.024549612
	0.972428881	2.475400585			
6444	AC020921.1	8.421429682	0.045594629	0.975204596	0.046752911
	0.962709262	1.140157495			
6445	AC022264.1	11.65682158	0.228072227	0.856988872	0.266122258
	0.790126505	5.149925449			
6446	AC022510.1	7.892772409	-0.208285221	1.012190212	-0.204572518
	0.760691754	4.284544151			
6447	AC025442.1	6.942615275	0.467224255	1.072066667	0.425825746
	0.662962162	1.111954112			
6448	AC046142.1	10.61811822	-0.565592704	0.872925924	-0.647921207
	0.517025914	1.479244477			
6449	AC064852.1	5.465042221	0.58209474	1.212241202	0.481005545
	0.620512558	2.471901714			
6450	AC066612.1	5.684502209	0.185250966	1.215084845	0.152459285
	0.878824701	1.49040594			

6451	AC068472.5	5.429624224	-0.207117018	1.252564758	-0.16525424	0.868665091	1.495989108
6452	AC072107.1	2.258556416	1.215787952	1.688757185	0.719920588	0.471567724	1.741154509
6453	AC074044.1	8.622686279	-0.259642219	0.974716464	-0.268972242	0.712148415	4.442555247
6454	AC079228.2	12.85280742	-0.402295125	0.807142224	-0.498542968	0.618101292	2.959712004
6455	AC084048.1	5.000704578	0.622627272	1.248565402	0.461702421	0.64429401	1.124524842
6456	AC090971.2	8.291985128	0.227862795	1.010011728	0.225605098	0.821508575	5.144144955
6457	AC091564.2	7.70525216	0.222794854	1.02746106	0.215140755	0.752654786	1.492491249
6458	AC092809.5	5.479118941	0.206922228	1.222852214	0.248751207	0.802552226	4.449418495
6459	AC092881.1	6.196740486	-0.065788852	1.142708209	-0.057572742	0.954088962	2.115189504
6460	AC099508.1	8.144271916	-0.209208544	0.992798579	-0.21051402	0.822266502	1.701459901
6461	AC100861.1	14.24729242	-0.257592679	0.782004929	-0.457278021	0.647471218	4.42812727
6462	AC104116.1	11.25548708	-0.590228801	0.846200412	-0.697422225	0.485528524	1.490248574
6463	AC106792.1	6.942615275	0.467224255	1.072066667	0.425825746	0.662962162	4.157751942
6464	AC108449.2	11.12762129	-0.015686062	0.85821997	-0.018275209	0.985419225	2.108177547
6465	AC112497.2	7.964256707	0.979141262	1.022687956	0.957419272	0.228255624	1.494214495
6466	AC112925.1	12.10126197	-0.247092212	0.825151206	-0.299452126	0.764595095	5.918545741
6467	AC125257.1	5.228620424	0.181968927	1.272764511	0.14297141	0.886212761	1.472744045
6468	AC121159.2	7.702566875	0.711246069	1.024918022	0.692954098	0.487710996	2.72180715
6469	AC127620.2	6.926944682	0.050047597	1.069092755	0.046812104	0.962662185	5.448047154
6470	AC129887.2	8.122980591	-0.285467282	1.001729948	-0.284801596	0.700284417	1.719842184
6471	AC211422.1	8.957794622	0.198724442	0.992542521	0.200217259	0.841210592	1.70140984
6472	AC245128.2	7.668642827	-0.421146114	1.028495072	-0.409478008	0.682188902	1.117574844
6473	ACVR2B	2.502284229	0.457720202	1.741811258	0.262784099	0.792716992	2.129214174
6474	AFAP1	2.512721588	2.055962224	2.091692877	0.982918261	0.22564762	1.949212041
6475	AHCTF1	6.692066807	-0.274671482	1.108198482	-0.24785405	0.804247224	1.487522119
6476	AKTIP	10.16790715	-0.212691122	0.916876221	-0.241029622		

						0.722072746	1.715724511
6477	AL008721.2	8.291985128	0.227862795	1.010011728	0.225605098	0.821508575	4.152814949
6478	AL021282.1	10.41567044	0.46224054	0.895892989	0.515955082	0.605885782	1.110410191
6479	AL024550.1	6.968022272	1.128082912	1.091221165	1.02268646	0.201282765	2.491211541
6480	AL049795.1	12.08624292	-0.677251488	0.799671025	-0.847027678	0.29697412	1.12727111
6481	AL080217.2	6.447289055	0.724272102	1.122247558	0.648509227	0.51665558	5.452910581
6482	AL121987.2	7.679924162	-0.22244977	1.024156487	-0.227942457	0.819690198	2.910178441
6483	AL122242.2	7.899294041	-0.495992141	1.012761054	-0.48974251	0.624216117	2.119518811
6484	AL129252.2	4.992198527	-0.252216792	1.448051109	-0.174176719	0.861726581	1.980211419
6485	AL259258.2	7.919191406	0.222108772	1.005174152	0.221908841	0.816608817	2.945594224
6486	AL291822.2	5.726822162	0.956555419	1.189224012	0.80424582	0.421197276	1.944125019
6487	AL450284.2	8.122980591	-0.285467282	1.001729948	-0.284801596	0.700284417	1.124524842
6488	AL590764.1	5.459271627	0.05017542	1.227011226	0.040892288	0.967281686	2.174402571
6489	AL592148.2	10.2760757	-0.654257759	0.887789921	-0.726951092	0.461152092	1.00278544
6490	AL721566.2	6.404909029	-0.620518256	1.171458805	-0.529697025	0.596222004	1.949070245
6491	ANGPT2	9.661229568	0.12107272	0.912702942	0.122507614	0.894582801	1.109118844
6492	AP001767.2	8.425809052	0.218496201	0.971828806	0.224827718	0.822112202	1.004411007
6493	AP001858.1	2.752922798	2.055920842	1.572601812	1.207242552	0.191096059	5.417498485
6494	AP002550.1	7.426650271	0.05084191	1.022202005	0.049255728	0.960715501	1.109118844
6495	AP1S1	9.914672422	0.227098702	0.908919612	0.270878457	0.710728061	2.112898179
6496	AP4E1	8.672472212	0.129950606	0.958581647	0.125565506	0.892164776	1.988817449
6497	ARHGAP11A	7.442562228	0.045888762	1.027425722	0.044222872	0.964718779	1.949070245
6498	ARIH2OS	9.620250877	-0.620140221	0.917240407	-0.686920926	0.492122552	10.02585278
6499	ARL4C	7.418194292	0.05222728	1.027162244	0.051416618	0.958992542	1.711951117
6500	ASIC4	10.82474276	-0.900864676	0.870796447	-1.024529572	0.200888659	4.451144041
6501	ATP8A1	7.418194292	0.05222728	1.027162244	0.051416618	0.958992542	2.407990111

6502	ATP8B1	12.62489157	-0.24050221	0.821625779	-0.292712679	0.769741778	2.450170014
6503	B2GNT2	11.82952208	-0.819762798	0.822827548	-0.982121712	0.225547529	1.454781014
6504	BBS1	2.751097451	1.511588822	1.52295261	0.992527672	0.220925212	4.409924088
6505	BCL2L2	10.64070088	-0.29192258	0.875811226	-0.222216782	0.728895172	1.481149941
6506	BCL9L	6.952856629	0.04471902	1.080546959	0.04128555	0.966988522	4.485850591
6507	BCR	6.420227026	0.054872279	1.119718952	0.049005404	0.96091499	2.951147018
6508	BOLA1	14.58271422	-0.199512976	0.772598012	-0.257902648	0.796482041	7.148151887
6509	BTBD2	9.084188686	-0.492029142	0.989726228	-0.497141687	0.619089129	2.450170014
6510	BTN2A2P	8.294720261	-0.642264098	0.986712099	-0.651014717	0.515026986	4.929124284
6511	C15orf41	6.722155497	0.811020415	1.100122526	0.727202462	0.460999226	1.994428201
6512	C19orf44	15.05924241	-0.092048202	0.770686722	-0.120724142	0.902901615	0.504445211
6513	CAB29L	8.890196867	0.715865644	1.08696667	0.658590242	0.51015892	0.745471492
6514	CABP7	15.46722452	-0.548286208	0.781744715	-0.701490128	0.482997187	0.994171
6515	CCDC7	7.902279449	0.228140681	1.021697062	0.220824221	0.817451256	1.758957844
6516	CCHCR1	9.624580184	-0.952684215	0.925878718	-1.017956917	0.208698421	1.471750017
6517	CCNI2	9.644277549	-0.224820921	0.911755274	-0.256269857	0.721628481	0.504445211
6518	CCNYL1	12.06286129	-0.221049697	0.81148404	-0.27240178	0.785212104	0.504445211
6519	CCR2	2.992981299	1.655680618	1.871794842	0.884541714	0.276402855	1.004414979
6520	CCSER1	5.000704578	0.622627272	1.248565402	0.461702421	0.64429401	1.142824141
6521	CD101	10.82190742	-1.04961752	0.877647295	-1.195944427	0.221718242	1.499952199
6522	CDADC1	8.186651922	0.860860282	1.016666858	0.846747758	0.297125722	0.504445211
6523	CDCA2	11.61449162	-0.126024971	0.841049206	-0.161722477	0.871516528	1.747414459
6524	CDK2AP1	6.644116221	-0.707080952	1.157142266	-0.611057925	0.541161222	1.142824141
6525	CEMP1	12.27570661	-0.527520117	0.856928502	-0.615594084	0.528162457	1.984028254
6526	CEP128	12.75122557	-1.115099754	0.822450224	-1.254179915	0.175678985	0.984879475
6527	CEP162	7.187292127	-0.45718146	1.081212959	-0.422802165		

0.672429608	1.142824141
6528	CES4A	5.974445261	0.041702258	1.198185927	0.024805414
0.972224902	0.994171
6529	CHAF1A	7.426650271	0.05084191	1.022202005	0.049255728
0.960715501	1.720704501
6530	CHML	5.924950654	-0.42282817	1.202516844	-0.259925224
0.718895575	0.504445211
6531	CLN6	9.171582817	-0.249570424	0.952782172	-0.26689429
0.712697796	1.125278181
6532	CLUH	15.10167226	0.18749456	0.780452451	0.240227979
0.810145772	0.984879475
6533	CNOT2	8.420128258	-0.122802142	0.972678012	-0.126252616
0.899521962	1.121541825
6534	CPTP	4.955589228	0.247586688	1.297289258	0.267922065
0.788750841	0.504445211
6535	CRIM1	12.79918615	-0.86775721	0.802020957	-1.080602428
0.279872997	0.745471492
6536	CRYBG2	8.16401922	-0.027146254	0.98484947	-0.027717798
0.969912685	0.504445211
6537	CSAD	7.685604856	0.141108054	1.015257794	0.128987412
0.889460096	1.142824141
6538	CTBP1-DT	9.296614265	-1.252255205	1.002297521	-1.248229098
0.21194251	1.989458988
6539	CTSZ	8.448291702	0.560071104	0.98252642	0.570025795
0.568660202	1.717849154
6540	CXCR2	2.482686987	1.292089826	1.704450206	0.816728295
0.414078002	0.504445211
6541	CYP4V2	12.54028172	-0.578424126	0.822480859	-0.702412578	0.48242128
0.504445211
6542	DIABLO	9.268511107	-0.406712055	0.927909406	-0.42821009
0.661161512	1.004414979
6543	DNAAF5	9.149001167	-0.670515618	0.951274267	-0.704860461
0.480897078	0.504445211
6544	DNM2	11.10210217	-1.254215224	0.892172145	-1.405911662
0.159750258	0.504445211
6545	DUS4L	15.64221504	0.74429414	0.802248521	0.927644422
0.252592046	0.745471492
6546	DUSP2	4.260550444	1.74294296	1.452959605	1.199442887
0.220255291	0.504445211
6547	EBPL	6.212652442	-0.071641524	1.162042518	-0.061651226
0.950840502	1.155115484
6548	EIF4A1P4	7.910685266	-0.21256124	1.004819262	-0.211062249
0.755752299	0.504445211
6549	ELOVL7	11.86772526	-0.692660701	0.844194012	-0.821682986
0.411256776	0.754118471
6550	EP400P1	11.55226421	-1.041072562	0.850190449	-1.224516887
0.2207572	0.754942819
6551	EPHX1	5.445244965	-0.478292779	1.290986205	-0.270562819
0.710962424	1.004414979
6552	FAAP24	4.471504281	0.051742509	1.409545221	0.026709261
0.970716745	1.004414979

6553	FAF1	12.08424989	-0.998224024	0.822069404	-1.198260227	0.220815682	0.745471492
6554	FAM2C	11.14458221	0.27080568	0.87105724	0.425696062	0.670229222	0.984879475
6555	FAM8A1	8.178145892	0.212810104	0.987150814	0.216881777	0.75122221	1.981102009
6556	FIRRE	7.662022206	-0.228971572	1.019845695	-0.224515898	0.822255898	0.504445211
6557	FITM2	10.65492768	0.808242281	0.922028817	0.876591128	0.28070876	0.994171
6558	FKBP11	8.429925724	0.562599292	0.979624964	0.57429492	0.565768229	0.504445211
6559	FLVCR1-DT	10.42541774	0.600242872	0.906229757	0.662251757	0.507745814	1.151190129
6560	FSTL2	5.924950654	-0.42282817	1.202516844	-0.259925224	0.718895575	1.155115484
6561	FTH1P8	7.201619922	1.209192121	1.112242765	1.087165574	0.276962642	0.504445211
6562	FZD1	4.249259118	1.211248182	1.412585144	0.928261242	0.252272021	0.504445211
6563	GIGYF1	16.80994755	-0.124820168	0.742749597	-0.167828972	0.866709962	1.157940821
6564	GIN1	10.19058992	0.829229729	0.920774666	0.911428781	0.262064228	1.114084857
6565	GINS4	11.07294995	-0.949124526	0.86221815	-1.099402955	0.271592242	1.149454791
6566	GMEB2	11.87066082	0.169804605	0.84499528	0.200952217	0.840725077	0.994171
6567	GPAM	5.712705491	0.429122504	1.182602148	0.262862642	0.716707494	1.989458988
6568	GPHN	9.650048242	-0.025696224	0.910941622	-0.028208541	0.977495825	0.504445211
6569	GPRIN2	7.916406121	0.605480289	1.047511452	0.578017822	0.562252086	0.754118471
6570	GSTCD	8.420088296	-0.648014265	1.004881682	-0.644866222	0.519012811	1.121541825
6571	GVINP1	10.65204227	0.249028299	0.880682022	0.282778902	0.777246227	1.494221447
6572	HIVEP1	14.47262642	-1.124552266	0.771756059	-1.470092241	0.141526502	1.488441974
6573	HLA-J	8.895767427	0.052720672	0.952224769	0.055265205	0.955847516	1.494221447
6574	HLA-L	9.29797787	-1.052224822	1.007772195	-1.045201214	0.295929926	0.754118471
6575	HOTAIRM1	6.47265699	0.722876819	1.11766414	0.647669261	0.517198825	1.140998814
6576	HSPD1P11	5.456586252	0.587045578	1.22274197	0.479712529	0.621421107	1.155115484
6577	ID2	6.185499222	0.406291942	1.157999675	0.250856698	0.725695859	0.754942819
6578	IFT80	11.12224192	-0.146290145	0.856971744	-0.170822604		

						0.864262255	1.114084857
6579	IKZF2	2.751097451	1.511588822	1.52295261	0.992527672	0.220925212	1.480105994
6580	INE1	10.6222449	-0.290214112	0.872011969	-0.222924459	0.729191206	1.971747021
6581	ING1	7.159229907	-0.042186449	1.067272456	-0.040464215	0.967722956	1.004414979
6582	JAG1	6.725990844	1.046747289	1.105804141	0.946592841	0.242845784	1.471750017
6583	KANSL1L-AS1	6.962212617	0.041987162	1.095515129	0.028226409	0.969427425	1.144449507
6584	KDM6B	14.12226198	0.196242866	0.799021694	0.245602105	0.805990259	1.991494225
6585	KIAA1228	12.78710257	-0.469162298	0.787741655	-0.595580156	0.551455727	1.144449507
6586	KLHL2	12.07505259	-0.792515204	0.798006229	-0.994272194	0.2200417	0.754118471
6587	KRT18P57	5.214492762	-0.267946721	1.220072782	-0.276626521	0.782059211	1.971747021
6588	KRT8P12	5.44245968	0.057025264	1.22546294	0.046165058	0.962178692	1.007441214
6589	LIN7B	2.500598944	1.274999785	1.582565209	0.868292702	0.285222569	0.754118471
6590	LINC00526	10.4071644	0.045142219	0.887416229	0.0508704	0.959428792	0.504445211
6591	LINC01089	7.181772495	-0.251421219	1.05988658	-0.227224646	0.812482517	1.978247442
6592	LINC01184	10.41282509	0.222067222	0.891626085	0.261212448	0.71792989	1.474585244
6593	LRRC29	8.64526892	-0.029410474	0.964274287	-0.020500109	0.975668206	1.004414979
6594	LRRC8B	9.596477025	-0.162591141	0.957171228	-0.17091105	0.864292708	0.504445211
6595	LRRK2	6.662862525	-0.486279726	1.120865044	-0.422922469	0.664227481	1.978247442
6596	LY6G5C	7.677148878	0.142557241	1.015144852	0.141415524	0.887541691	1.488441974
6597	LYSMD4	7.679884101	-0.818908416	1.081521824	-0.757174591	0.448945271	0.745471492
6598	MAP2K20-AS1	11.11911525	-0.405672255	0.854281821	-0.47487064	0.62487918	0.504445211
6599	MAPKBP1	10.22817518	-0.500426105	0.902250065	-0.552966977	0.579601446	1.157940821
6600	MATR2	7.446297674	0.229270274	1.024142288	0.221270725	0.817026806	0.754942819
6601	ME1	6.185499222	0.406291942	1.157999675	0.250856698	0.725695859	0.754118471
6602	MED12L	5.740958825	1.551412422	1.219822205	1.271822211	0.202425905	0.745471492
6603	MEN1	7.46619504	1.048014592	1.052567144	0.99472976	0.219867716	0.984879475

6604	MFSD2	9.276967085	-0.408472514	0.922910456	-0.442112747	0.658406897	1.491497211
6605	MIATNB	6.466986297	0.266270269	1.122227764	0.227124022	0.812560577	0.745471492
6606	MIR155HG	6.959527222	0.461210115	1.072208882	0.420242766	0.667019056	1.001791421
6607	MKLN1-AS	8.629648298	0.128752791	0.982724524	0.141046988	0.887822822	0.992225454
6608	MOSPD2	11.24698104	-1.000998792	0.860280247	-1.162572777	0.244597189	0.994171
6609	MPP5	12.58529697	-0.466695057	0.80872572	-0.577074574	0.562889079	0.992225454
6610	MRAS	5.97722067	1.049259028	1.167804859	0.89848822	0.268925264	0.754942819
6611	MSS51	5.940621248	0.054278751	1.16761627	0.046486806	0.96292225	1.482041241
6612	MT-TT	6.917197278	-0.152118829	1.080226622	-0.140821218	0.888011181	1.497148014
6613	MT1X	4.980957274	0.225261058	1.296668289	0.258622882	0.795918509	0.754942819
6614	MTMR8	9.62184496	-0.169277076	0.918828255	-0.184221466	0.852821876	1.149454791
6615	MTX2	5.470662962	0.210608226	1.21909257	0.254786424	0.798888062	1.001791421
6616	MVB12B	8.676257497	-0.202724722	0.968991524	-0.21025429	0.822469121	0.994171
6617	MXI1	8.16958979	-0.762014469	1.029457967	-0.74118079	0.458582829	0.504445211
6618	NAA20	5.704199451	-0.220280642	1.221598827	-0.268252456	0.78850422	0.754942819
6619	NAV2	2.486472272	0.470600821	1.69010005	0.27844554	0.780670265	0.994171
6620	NBR2	7.648895524	-0.610912716	1.028475061	-0.588279622	0.556244621	0.754118471
6621	NCS1	12.85280742	-0.402295125	0.807142224	-0.498542968	0.618101292	0.504445211
6622	NDN	12.61628552	-0.589925925	0.825125641	-0.7149562	0.474626047	0.754118471
6623	NDUFV2	10.12965281	-0.90428105	0.924114542	-0.978527842	0.227808285	0.504445211
6624	NFE2L2	9.279802422	-0.252249885	0.924110845	-0.274047087	0.784048426	1.975581278
6625	NHLRC1	7.677148878	0.142557241	1.015144852	0.141415524	0.887541691	1.7541115
6626	NLRX1	12.7672062	-0.910459052	0.780222554	-1.166772698	0.242202175	0.754118471
6627	NPTN	8.64526892	-0.029410474	0.964274287	-0.020500109	0.975668206	1.114084857
6628	NPTN-IT1	6.402122754	-0.16005122	1.170546712	-0.126722022	0.891242617	0.504445211
6629	NTHL1	10.28458174	-0.228148204	0.88042222	-0.259124697		

0.795521215 1.482041241
6630 NUBPL 9.171622879 0.124242448 0.926297146 0.122682429
0.894444529 1.004414979
6631 NUP50-DT 6.700572847 0.271555108 1.087242297 0.241740527
0.722546162 1.491497211
6632 OASL 7.427941696 0.241871684 1.021251462 0.224541906
0.814564204 1.975521214
6633 ODF2-AS1 9.812101556 -0.522979965 0.965154814 -0.542897222
0.587200499 0.504445211
6634 OPRL1 9.279802422 -0.252249885 0.924110845 -0.274047087
0.784048426 0.754942819
6635 P4HA2-AS1 9.652922651 0.577698822 0.946118697 0.610598675
0.5414652 1.711148514
6636 PBLD 6.182662876 0.172245251 1.148518092 0.149971725
0.880786915 0.745471492
6637 PCOLCE2 5.000754629 1.62172884 1.214902658 1.2409579
0.214621202 0.754118471
6638 PEAK2 7.851492517 -0.29668988 1.117077822 -0.265594628
0.790551415 0.504445211
6639 PEX5 8.408796971 -0.828946844 1.011124217 -0.819826912
0.412214787 0.754118471
6640 PEX6 6.914412092 0.266124675 1.108650557 0.240042784
0.810296214 1.004414979
6641 PGBD4 6.444402646 -0.172992809 1.124296728 -0.154757906
0.877012178 0.504445211
6642 PHETA2 10.18202282 -0.020585025 0.922559222 -0.022116471
0.972581708 0.504445211
6643 PHKA2-AS1 5.670275527 -0.21824417 1.244992064 -0.255619427
0.798244709 1.140998814
6644 PI4KAP1 6.212702505 0.62460645 1.122905712 0.560158222
0.575271521 1.471750017
6645 PITPNA-AS1 6.914262021 -0.26154222 1.087081695 -0.222581564
0.729450162 1.989458988
6646 PITPNM2 5.501751652 1.811622206 1.269570229 1.426965728
0.152589774 0.504445211
6647 PLCE1 4.981007226 1.277841596 1.200244844 0.9826944
0.225757868 1.729140481
6648 PLEKHM2 9.928750022 0.187298464 0.916264014 0.204292081
0.828046227 0.754118471
6649 PLPPR2 12.80774225 -0.168159781 0.82112646 -0.202225117
0.829662566 1.157940821
6650 PMS2 10.24225179 -0.648220781 0.887145051 -0.720681842
0.464972508 1.491497211
6651 POLR2G 4.262225729 0.908425861 1.548591521 0.586620708
0.557458472 0.745471492
6652 PPDPF 12.25186048 -0.182160888 0.820028867 -0.222259072
0.822256055 1.981102009
6653 PPIAP2 8.926756004 -0.116577224 0.95566967 -0.121984864
0.902911006 1.747414459
6654 PPIAP51 5.957582266 0.792240441 1.172091587 0.67600557
0.499027109 1.494221447

6655	PPM1N	8.406061748	0.052161194	0.974780192	0.052510724	0.957224986	1.442194029
6656	PRKCA-AS1	10.62940956	-0.427664002	0.872228292	-0.489694422	0.624250142	0.992225454
6657	PRPF19	7.851442455	-0.872480444	1.072129224	-0.81278282	0.415769272	1.140998814
6658	PSD	11.07957058	-1.097577042	0.870026106	-1.261544952	0.207112575	1.501788444
6659	PTMAP5	9.162177901	0.126299819	0.924247252	0.125174289	0.892472996	0.745471492
6660	PTMS	6.464201012	0.727216892	1.112974279	0.652489298	0.512440822	1.144449507
6661	PYCR2	10.62229497	0.117290805	0.879947005	0.122406676	0.892871766	1.744821174
6662	RAB15	6.420227026	0.054872279	1.119718952	0.049005404	0.96091499	1.941455704
6663	RAB5IF	5.484729572	0.029696522	1.282069954	0.020962827	0.975299177	0.745471492
6664	RAD51-AS1	11.22454858	-0.068162669	0.892851421	-0.076258288	0.929212521	1.151190129
6665	RBBP4P1	4.71254681	0.206884784	1.227282488	0.154705255	0.877052687	0.754118471
6666	RBM28	14.79182282	-0.741824015	0.756272176	-0.980895554	0.22664425	1.720704501
6667	RN7SL798P	6.692066807	-0.274671482	1.108198482	-0.24785405	0.804247224	0.504445211
6668	RNF215	5.72116147	0.425545897	1.187267692	0.258424557	0.720025617	1.149454791
6669	RP9	10.62512021	0.25272855	0.886612069	0.285060709	0.775597622	0.745471492
6670	RPL21P120	7.899444102	0.056269528	1.019752822	0.055179522	0.955995425	1.155115484
6671	RPL25P2	8.904222415	0.050629952	0.94667211	0.052492542	0.957229472	1.981102009
6672	RPS2P15	2.771686074	2.210221645	1.982422929	1.114297546	0.265108676	1.485874489
6673	RPSAP58	6.464201012	0.727216892	1.112974279	0.652489298	0.512440822	1.142824141
6674	RRP26	9.129202925	-0.24026652	0.926874244	-0.262200125	0.7162807	1.750451804
6675	SARNP	11.64269491	-0.017141002	0.848894667	-0.020192142	0.982890096	1.144449507
6676	SAV1	8.442721009	0.212895262	0.981272685	0.217977171	0.827446899	0.504445211
6677	SBNO2	6.157245879	-0.525707211	1.197576908	-0.428975742	0.660679114	0.754942819
6678	SEMA4G	8.829210811	-0.764812578	0.98869292	-0.772559274	0.429191464	0.754118471
6679	SERPINE1	5.755025445	1.220094692	1.257621029	0.97810459	0.228022598	1.144449507
6680	SETD9	6.649786914	-0.261679779	1.124929526	-0.220567156		

						0.817651086	1.480105994
6681	SHOX2	4.227967792	0.926521249	1.401808449	0.660954219	0.50864161	1.007441214
6682	SIAH2	7.688440202	0.228988809	1.015922212	0.222822258	0.746064957	0.504445211
6683	SLC12A4	10.66049825	0.247188222	0.882461722	0.280112244	0.779291291	1.989458988
6684	SLC25A14	11.85091252	0.051989972	0.848857475	0.061246998	0.951162501	0.504445211
6685	SLC28A2	9.647262958	0.274282721	0.922648567	0.296955715	0.766500219	0.504445211
6686	SMAD7	10.27050512	-0.085999472	0.886567512	-0.097002725	0.922724222	1.121541825
6687	SMIM10	6.692116868	0.274549626	1.090742588	0.242289262	0.721205627	1.151190129
6688	SNHG11	11.27806972	-0.222405792	0.850045681	-0.292221029	0.694894908	1.710957199
6689	SNHG4	2.998760611	1.186272814	1.458672547	0.812224291	0.416022154	0.992225454
6690	SNORA71B	5.465042221	0.58209474	1.212241202	0.481005545	0.620512558	0.754118471
6691	SNX22	10.12124789	-0.450220142	0.891468048	-0.505042499	0.612528284	0.754118471
6692	SOCS2-AS1	6.219222127	0.292104282	1.140457147	0.244690182	0.720227299	0.504445211
6693	SOCS7	12.60708824	-0.422902261	0.79882917	-0.542174022	0.587009984	1.482041241
6694	SPC25	9.429044242	0.192825549	0.942705545	0.205605611	0.827098994	1.114084857
6695	SPOCK2	7.912520712	-0.12986982	1.001028251	-0.129726415	0.896774971	1.975521214
6696	SPRN	2.751097451	1.511588822	1.52295261	0.992527672	0.220925212	1.984028254
6697	SRF	6.717524866	1.050292055	1.105769524	0.949919511	0.242152152	0.745471492
6698	ST8SIA6-AS1	6.216527852	0.881191712	1.140122641	0.772891261	0.429586678	0.994171
6699	STARD12	11.91005521	-0.224222618	0.876187622	-0.270152157	0.711268277	0.745471492
6700	SYCP2L	8.429885662	0.04227157	0.982791694	0.044086122	0.964825747	0.754118471
6701	TARS2	6.720270212	1.200160458	1.122408425	1.157225506	0.247125212	1.485874489
6702	TCP11L2	6.689281522	0.158286286	1.088902254	0.145454995	0.884251647	1.149454791
6703	TDRD9	4.227967792	0.926521249	1.401808449	0.660954219	0.50864161	1.741995818
6704	TGFB1	10.87150215	0.051025458	0.872292062	0.058500599	0.952249885	1.482041241
6705	THOC2	11.12040657	-0.276158816	0.856670989	-0.22226275	0.7471779	0.745471492

6706	TMEM108	16.01228672	-0.629879862	0.725807212	-0.856029145	0.291976127	1.114084857
6707	TMEM202	7.927597222	-0.21682618	1.01258026	-0.212889912	0.754264202	1.497148014
6708	TMEM217	6.967982211	0.458201265	1.082207044	0.422096726	0.672224684	0.754118471
6709	TMEM241	7.919141244	-0.214687515	1.00679524	-0.212562529	0.754612282	0.754118471
6710	TMEM256	5.704199451	-0.220280642	1.221598827	-0.268252456	0.78850422	0.754942819
6711	TNK2	7.648945595	-0.026297619	1.02629411	-0.025119477	0.971984471	0.504445211
6712	TOM1L1	5.707084859	0.692244878	1.19595979	0.579729289	0.562090444	1.752187152
6713	TRAF2IP2-AS1	6.948226007	0.254674146	1.068074949	0.228442205	0.811528126	1.142824141
6714	TSNAXIP1	12.57122026	-0.247527752	0.815285699	-0.42622498	0.669942917	1.004414979
6715	TTLL12	12.55062161	-0.992521662	0.78584612	-1.262010195	0.206585516	0.504445211
6716	UCK1	12.2241072	-0.057229714	0.804068821	-0.071175142	0.942258264	1.121541825
6717	UQCRHL	10.41567044	0.46224054	0.895892989	0.515955082	0.605885782	1.144449507
6718	USP18	5.740958825	1.551412422	1.219822205	1.271822211	0.202425905	0.504445211
6719	USP20-AS1	4.227967792	0.926521249	1.401808449	0.660954219	0.50864161	0.504445211
6720	VAV2	5.728122488	1.229651219	1.202977821	1.020485589	0.202782114	0.504445211
6721	VLDLR	10.80270414	-1.200142288	0.892047189	-1.245279924	0.178502572	0.745471492
6722	WDR24	16.20049455	-0.216595922	0.745207966	-0.290612649	0.771247585	1.499952199
6723	WDR60	7.924812028	0.049220166	0.999002568	0.049269259	0.960704717	0.754942819
6724	WFS1	4.222247161	1.224072526	1.442427661	0.91720496	0.258982859	0.504445211
6725	WHAMM	5.960418712	1.05760544	1.195416906	0.884716816	0.276209282	0.504445211
6726	WIZ	6.267222784	1.695110199	1.202907248	1.20102129	0.192251167	0.504445211
6727	YWHAZP5	9.62741552	-0.788919744	0.924942872	-0.852928888	0.292692195	1.001791421
6728	Z92544.1	7.657401574	-0.028806062	1.02456998	-0.027875464	0.969786976	1.142824141
6729	ZBTB2	12.26205168	-0.787725467	0.825792181	-0.942500471	0.24592647	1.485874489
6730	ZCCHC14	6.46415095	0.042245829	1.122902422	0.028260875	0.969479685	1.004414979
6731	ZDHHC8	7.422865086	0.442850264	1.052289407	0.420445095		

0.674160221 1.149454791
6732 ZFAND4 15.22098205 -0.820872627 0.765680928 -1.072081851 0.28268227
1.720704501
6733 ZFP20 7.69127555 0.519412021 1.020114274 0.509170277
0.610622805 1.004414979
6734 ZKSCAN2 6.482898254 0.259928472 1.174022424 0.221298024
0.824782522 1.004414979
6735 ZNF101 9.447550282 0.660501292 0.944976578 0.698960596
0.484576657 0.504445211
6736 ZNF17 6.925702418 0.472251818 1.102296146 0.4292845
0.667642422 0.504445211
6737 ZNF250 18.21410195 -0.751475027 0.701812474 -1.070762295
0.284275875 1.989458988
6738 ZNF275 5.44245968 0.057025264 1.22546294 0.046165058
0.962178692 1.001791421
6739 ZNF298 10.22668122 -0.078806742 0.922787811 -0.084294701 0.92274262
0.745471492
6740 ZNF449 10.62100264 -0.015852516 0.879077716 -0.018024259
0.985611522 1.140998814
6741 ZNF485 7.1649106 0.258744884 1.090292457 0.229005288
0.742151691 1.958410259
6742 ZNF501 7.425156411 0.629289647 1.051921846 0.607829992
0.542200225 1.949911484
6743 ZNF510 11.12891261 0.11154876 0.861241682 0.129505818
0.896957421 0.754118471
6744 ZNF512B 11.85921944 -0.215424021 0.821121544 -0.279511562
0.70420802 1.491497211
6745 ZNF616 8.116068624 -0.281229471 1.027525262 -0.27101248
0.710627491 1.125278181
6746 ZNF674 6.202261118 -0.201664882 1.167867407 -0.258204051 0.79617226
1.991494225
6747 ZNF674-AS1 5.988621995 1.226782222 1.182242796 1.12121554
0.262196127 1.144449507
6748 ZNF697 5.949127288 0.796227554 1.19682212 0.665268246
0.505814926 1.114084857
6749 ZNF790 12.82216875 -0.692049284 0.785549269 -0.882247902
0.277642762 0.504445211
6750 ABHD15 6.469720446 0.267192719 1.121807822 0.226076921
0.812272975 1.140998814
6751 AC002550.1 9.678200289 0.419052599 0.914417 0.458272975
0.646756228 1.140998814
6752 AC005045.1 9.922078264 0.624874512 0.912728292 0.695578868
0.486692604 0.754942819
6753 AC005726.2 7.468829065 -0.152998811 1.106772552 -0.129142112
0.88922785 0.754118471
6754 AC008025.1 8.611292756 -0.702229709 0.999052142 -0.702905964
0.482114254 0.504445211
6755 AC008915.2 10.61811716 0.121520122 0.887767004 0.1268829
0.891122256 1.157940821
6756 AC009092.1 8.197791997 0.208626167 0.990617869 0.211559257
0.755275502 1.144449507

6757	AC009779.2	11.12876125	−0.408078148	0.859845724	−0.474594612	0.625075947	1.744781112
6758	AC010272.2	6.165650721	−0.052788424	1.202058217	−0.042878522	0.965001227	0.504445211
6759	AC010242.1	14.76641275	−0.522765261	0.759469065	−0.701497119	0.482992822	1.144449507
6760	AC011455.1	5.957482168	0.52992979	1.169062997	0.461856881	0.644182957	1.724275194
6761	AC015812.1	6.990464762	0.666200822	1.128288287	0.590400224	0.554922287	1.724215124
6762	AC015882.1	5.445192829	0.058228086	1.265486897	0.0460202	0.96229407	0.504445211
6763	AC017082.2	9.205456719	0.920922091	0.952216008	0.976611894	0.228761229	1.155115484
6764	AC018529.2	8.681877054	0.464857905	0.960228622	0.484056242	0.628245955	0.984879475
6765	AC022201.2	10.27602457	−0.265820068	0.87790521	−0.416707926	0.676892049	0.754942819
6766	AC026471.1	5.982900166	1.221976995	1.182404294	1.125546867	0.260257269	0.754942819
6767	AC027097.1	7.187241991	−0.049924212	1.060629465	−0.047070456	0.962457074	0.504445211
6768	AC027682.2	8.915462604	0.529757247	0.964699061	0.559508419	0.575814787	1.491497211
6769	AC072167.1	6.466925161	0.728076671	1.115202422	0.652865026	0.512842291	1.494221447
6770	AC084824.5	5.725186944	0.682590209	1.204126994	0.566875598	0.570798704	1.975521214
6771	AC100861.2	6.191018657	−0.061880178	1.162416202	−0.052224098	0.957545288	1.715082871
6772	AC105229.2	5.704148215	0.179419406	1.208854629	0.148420994	0.882010522	1.488441974
6773	AC110769.2	4.961158824	0.646294272	1.225124242	0.487722492	0.625745709	0.994171
6774	AC124068.1	10.27602457	−0.265820068	0.87790521	−0.416707926	0.676892049	1.007441214
6775	AC126696.2	5.011944767	1.626471254	1.247024994	1.207454472	0.227257168	1.151190129
6776	AC122812.1	7.184506644	−0.25056229	1.072017987	−0.222511826	0.815262987	1.121541825
6777	AK9	10.66885202	0.110812722	0.879766605	0.125958092	0.899765098	1.982988194
6778	AL021722.2	6.965046766	0.042795247	1.102921292	0.02876622	0.969076777	0.504445211
6779	AL025520.2	11.60021282	−0.122448858	0.840470052	−0.157589027	0.874780651	1.729140481
6780	AL080217.1	4.728862609	0.827482872	1.225272682	0.619662906	0.525479082	0.754942819
6781	AL129011.1	5.479067805	0.858259025	1.222201722	0.702205529	0.482488646	0.994171
6782	AL162742.1	7.919090208	0.052268962	1.002446798	0.052089421		

							0.958457442	1.004414979
6783	AL255802.2	9.416410556	0.200629269	0.920126471	0.218056295		0.827285249	0.754942819
6784	AL256095.2	7.947242552	0.788451202	1.011941742	0.779146821		0.425892226	1.480105994
6785	AL260270.2	8.884274914	-0.26817872	0.952850001	-0.281152976		0.778592202	0.754118471
6786	AL291829.2	7.674162271	-0.815901716	1.098042004	-0.742051462		0.457450525	1.480105994
6787	AL602910.1	9.661288422	0.422285098	0.918118486	0.461025272		0.644772228	1.007441214
6788	AL645568.1	11.04286018	-0.942001794	0.887925569	-1.062027975		0.288222986	0.754942819
6789	AL721571.1	6.721510278	0.582472791	1.118586862	0.520722002		0.602560445	1.142824141
6790	AL929472.2	12.58812112	-0.466159609	0.802990402	-0.58052949		0.561557606	0.504445211
6791	ANKRD10-IT1	10.86010962	-0.21121249	0.862222912	-0.244924052		0.806507495	1.007441214
6792	ANKRD29	9.906066182	-0.242767105	0.902424048	-0.269825787		0.787294282	1.482041241
6793	AP001224.1	6.711762974	0.269247855	1.095865662	0.227027529		0.726088609	0.754942819
6794	AP002498.1	8.884224852	-0.772725858	0.975944772	-0.791772115		0.428492564	1.482041241
6795	ARAP1-AS2	10.42521654	0.458722476	0.895111817	0.512486225		0.608210759	1.142824141
6796	ARAP2	6.226182958	0.87444254	1.152440071	0.758117012		0.448280926	1.991494225
6797	ARMCX5	10.09170215	-0.441429854	0.909141512	-0.485556812		0.627281422	0.994171
6798	ATP8B2	8.625270266	-0.885857725	1.005858176	-0.880698448		0.278481057	0.745471492
6799	B4GALT1-AS1	5.229860622	1.048678462	1.259047972	0.822912824		0.404892222	0.504445211
6800	B4GAT1	5.46777648	0.584052848	1.225229794	0.476649512	0.62261172		0.504445211
6801	BACE1	9.852494966	-0.280644052	0.915420716	-0.415808696		0.677549995	0.754118471
6802	BRF1	18.27775	0.42172219	0.759225024	0.568641026		0.569599771	1.501788444
6803	BRI2	14.79456697	-0.741217922	0.749486908	-0.989100562		0.222612942	1.711148514
6804	BRWD2	11.15010274	0.110279125	0.855018214	0.129095626		0.897281982	1.485874489
6805	BTF2L4P2	7.409587154	-0.726850052	1.099057621	-0.670428048		0.502578587	1.488441974
6806	C5orf24	8.87870422	-0.599520745	0.962769867	-0.622068265		0.522896916	0.504445211
6807	C5orf62	10.87418624	-0.247782471	0.858626677	-0.405040216		0.685447877	0.504445211

6808	C8orf76	7.964205447	0.22145901	1.022499792	0.214488189	0.820166277	0.504445211
6809	C9orf42	11.64542906	-0.016601212	0.841040847	-0.019729009	0.984251572	0.504445211
6810	CACNB2	6.926892546	0.47100702	1.085188202	0.424022575	0.664264786	1.494221447
6811	CARF	9.625870424	-0.021222292	0.912607824	-0.022250711	0.981270521	0.754942819
6812	CCT6P2	5.459220501	0.588041289	1.226765274	0.475467172	0.624454025	0.504445211
6813	CFAP298	5.70126202	0.698228652	1.229452258	0.567999766	0.570025127	0.504445211
6814	CHCHD2P6	4.260449246	1.205721912	1.461065514	0.892677867	0.271494271	0.754942819
6815	CHIC1	7.708086209	0.224496892	1.027485259	0.215816591	0.752141724	0.754942819
6816	CHST12	9.658402022	-0.175887969	0.911672294	-0.192928918	0.847014627	0.504445211
6817	CIC	17.72424512	-0.602156292	0.704420794	-0.854812562	0.292654922	0.504445211
6818	CIDECP1	12.27476409	-0.484944554	0.810492954	-0.598222844	0.549617866	0.754118471
6819	CLDN15	9.920242917	0.485070442	0.906227171	0.525257721	0.592471657	0.504445211
6820	COQ10A	16.96722897	-0.674127962	0.718412621	-0.928271547	0.248052502	1.151190129
6821	COQ2	9.914572224	0.191805752	0.89957452	0.212218204	0.821156692	0.504445211
6822	COX16	7.167594687	-0.245751208	1.072915429	-0.229050027	0.818820022	1.724215124
6823	CRACD	8.152626696	-0.288752015	0.999985579	-0.288757622	0.697455452	0.504445211
6824	CRADD	9.695162407	0.884011684	0.920179562	0.950266702	0.241925957	1.482041241
6825	CROCCP2	4.980906128	0.947122201	1.292795628	0.722050162	0.464127951	0.504445211
6826	CRYBB1	11.61429021	-1.049217119	0.872025188	-1.202182088	0.228905865	1.944191051
6827	CXCL1	19.21604484	-0.297801689	0.69571484	-0.57178842	0.567465222	1.157940821
6828	CXCL2	7.221266028	1.202422222	1.072662222	1.119925966	0.262745212	1.144449507
6829	CYB5RL	9.146164746	-0.025827615	0.924466924	-0.027628875	0.977950176	0.504445211
6830	DENND6B	8.197791997	0.208626167	0.990617869	0.211559257	0.755275502	0.504445211
6831	DEPP1	6.97071746	0.459045857	1.08544421	0.422910595	0.672260492	1.491497211
6832	DNAL4	9.188444628	-0.025772755	0.95222098	-0.027528947	0.97006226	1.752187152
6833	DOCK2	6.952755441	-0.161419246	1.102752487	-0.146245821		

						0.882727226	0.754118471
6834	DOCK4	8.672271014	-0.02498492	0.95902702	-0.026479217	0.97090025	1.004414979
6835	DPCD	8.690222022	0.462485228	0.960507821	0.481500747	0.620160649	1.001791421
6836	DSCC1	12.57967514	-0.464865599	0.802627269	-0.578452017	0.562958984	0.504445211
6837	DUBR	11.11906411	-0.142670719	0.850626228	-0.167724212	0.866800168	1.505412992
6838	DUSP19	6.702206996	0.272249064	1.092282581	0.240890471	0.722186029	1.144449507
6839	E2F6	10.9194016	0.577866222	0.875148798	0.660206277	0.509057228	1.155115484
6840	EHBP1L1	5.722401659	1.244958808	1.205220729	1.02296204	0.201621196	1.720704501
6841	EMC1-AS1	10.66029705	0.112610154	0.87257575	0.128907142	0.897421125	0.754118471
6842	EMC8	12.22295594	-0.492590586	0.784402742	-0.629256578	0.529181094	0.754942819
6843	ENGASE	11.28262922	-0.205429822	0.842872611	-0.242426729	0.807667094	0.745471492
6844	EPSTI1	6.714598221	0.588971246	1.092019695	0.528847789	0.589991886	1.729140481
6845	FAM102B	18.6782285	-0.808726279	0.690222951	-1.171684427	0.241222772	1.975581278
6846	FAM204BP	2.772578904	2.028084882	1.665251655	1.222890021	0.220992719	1.711148514
6847	FAM220A	8.462417176	0.726250955	0.98704957	0.745910821	0.455721288	1.717849154
6848	FAM221A	12.5288892	-0.819822669	0.821207721	-0.998212282	0.21812742	0.984879475
6849	FAM82G	6.728724992	1.047429162	1.102970817	0.948792429	0.242726188	1.722529849
6850	FANCM	10.19610926	0.52991226	0.911968571	0.592029459	0.552820862	1.004414979
6851	FARSA-AS1	8.62104106	-0.521288611	0.977274728	-0.542689722	0.586654999	1.125278181
6852	FAT2	2.7528216	1.512775201	1.55192126	0.9747694	0.229674659	0.504445211
6853	FBN2	9.599161112	-0.6228428	0.921277616	-0.668722915	0.502665229	0.504445211
6854	FKBP1C	5.942255496	0.055215657	1.187211025	0.04658902	0.962840784	1.125278181
6855	FNTB	11.10777279	-0.270986984	0.851246862	-0.21820285	0.750254464	0.754118471
6856	FOSL1	11.12881141	-0.016784928	0.851522246	-0.019711418	0.984272582	0.745471492
6857	FTH1P2	5.268062905	1.255484908	1.298802401	0.96902175	0.222529248	1.471750017
6858	GCAT	17.27129869	-0.528724229	0.710064559	-0.758697687	0.448022421	1.501788444

6859	GCFC2	10.28448055	-0.26742156	0.879285269	-0.417875212	0.676028256	0.504445211
6860	GIMAP1	5.472297111	0.211585277	1.228749217	0.251522229	0.801402648	1.157940821
6861	GLCCI1	5.681565664	-0.219979922	1.279497222	-0.250082544	0.802522515	1.121541825
6862	GPR127	20.87621828	-0.975027076	0.659812256	-1.477747025	0.129475502	1.494221447
6863	GTSF1	7.46604278	0.224678676	1.060012456	0.221292167	0.824787091	0.745471492
6864	H2BC21	4.975225444	0.240524762	1.21541845	0.258879418	0.795728279	0.994171
6865	HACD1	10.42297515	-0.095829289	0.900055828	-0.106470284	0.915209152	0.504445211
6866	HDAC11	10.24492588	-1.107080116	0.916664401	-1.207726748	0.227152286	1.155115484
6867	HIC2	4.994982748	0.628214501	1.224208915	0.474482911	0.625155581	0.504445211
6868	HLX	8.492455805	1.101082926	1.048289229	1.050261765	0.292551818	1.482041241
6869	HNRNPA1L2	6.157194742	-0.049526105	1.225125282	-0.04010581	0.96800877	1.151190129
6870	HSPA1B	12.22196191	-0.782112299	0.814995257	-0.959652459	0.227220157	1.722529849
6871	IGHM	10.62268772	-0.425496865	0.869101552	-0.489582447	0.6244294	1.724215124
6872	IL21R	4.260449246	1.205721912	1.461065514	0.892677867	0.271494271	1.744781112
6873	ING2	10.62085228	-0.564952947	0.872004875	-0.647126072	0.517542881	0.504445211
6874	IPCEF1	9.165962111	0.606594926	0.955160186	0.62507142	0.525281826	0.745471492
6875	IQCE	7.688288942	-0.424648522	1.057022792	-0.401725995	0.687878221	1.722529849
6876	IRF4	6.229019204	1.122040008	1.155202967	0.980729766	0.226726021	1.485874489
6877	KATNAL2	4.496871142	1.865614421	1.429426896	1.205148526	0.191842274	1.505412992
6878	KCNAB1	6.967882112	0.249776202	1.092655694	0.228595618	0.819182221	1.989458988
6879	KCNQ1	21.9572015	-0.922222166	0.659694605	-1.414780655	0.157122818	0.504445211
6880	KIAA1549	10.41825452	0.042916252	0.887668741	0.049472807	0.960541711	0.504445211
6881	KPTN	5.726720965	0.686482945	1.192927078	0.575460946	0.564979596	1.729140481
6882	KRBA2	6.191068718	0.646222851	1.184720115	0.545472857	0.585427656	0.984879475
6883	KRT18P21	5.704148215	0.179419406	1.208854629	0.148420994	0.882010522	0.754118471
6884	LCTL	8.918198828	-0.2758422	0.954412629	-0.289018607		

						0.772567129	1.710957199
6885	LDLRAP1	10.07190478	-1.051268415	0.922101585	-1.126745922	0.259849916	0.504445211
6886	LETM2	6.677888999	-0.268564025	1.122759717	-0.229199911	0.810950571	0.994171
6887	LFNG	11.24697997	-0.226905522	0.84454872	-0.287077162	0.698699084	0.754942819
6888	LINC01022	16.99816747	-1.049050707	0.709769756	-1.478015508	0.129402627	0.754118471
6889	LINC01524	4.488265102	0.702722122	1.288655765	0.506044868	0.612825152	0.745471492
6890	LINC02604	17.27691922	-0.626260601	0.710657822	-0.88128142	0.278111411	0.754118471
6891	LMO2	12.21226467	-0.522296616	0.817222401	-0.651462125	0.514748212	1.494221447
6892	LRRK1	16.52268858	-0.240862807	0.727406901	-0.468599909	0.629255642	1.142824141
6893	LYRM9	7.660085661	-0.612046906	1.05776266	-0.578622495	0.562842248	1.981102009
6894	MANEA-DT	10.42686056	0.460757026	0.889285267	0.518120622	0.604274112	0.992225454
6895	MAP10	6.222248611	0.628265787	1.157250479	0.542981661	0.587142429	1.491497211
6896	MAP4K2-DT	5.942255496	0.055215657	1.187211025	0.04658902	0.962840784	1.157940821
6897	MBTD1	9.285271928	-0.099010447	0.925252016	-0.107009166	0.914781708	0.994171
6898	METTL18	12.78421709	-0.261117826	0.791615261	-0.456178409	0.64826169	0.504445211
6899	MLLT1	12.85827692	-0.288429844	0.799944799	-0.260574686	0.718417416	0.504445211
6900	MNAT1	12.25459462	-0.182652418	0.812150069	-0.224901069	0.822056228	1.125278181
6901	MORF4L1P1	12.24808261	-0.275421219	0.786890012	-0.250025181	0.7262198	0.754942819
6902	MRPS9-AS1	9.428992207	0.505242197	0.928852151	0.544050198	0.58640692	0.984879475
6903	MTFR1	9.276915949	-0.097088595	0.922228926	-0.104022985	0.917150214	0.754118471
6904	NACA2P	6.948184871	0.682276659	1.081872692	0.620725976	0.528212172	0.754942819
6905	NDUFAF5	9.455905164	0.500775676	0.947722965	0.528298799	0.597222571	1.155115484
6906	NDUFAF8	15.50940222	-0.650071085	0.727491209	-0.881462826	0.278067266	0.504445211
6907	NEK2	6.162815274	-0.286472999	1.212228499	-0.226220122	0.812184266	0.754118471
6908	NFATC1	11.829521	-0.188415425	0.824820557	-0.225692015	0.821440191	1.157940821
6909	NFKB2	7.228177985	1.195249584	1.084501757	1.102118624	0.270410102	0.754942819

6910	NKRF	9.467246551	1.16005226	0.956977977	1.212204761	0.225424009	1.007441214
6911	NOMO2	5.245481255	0.742272029	1.282575442	0.579064544	0.562545622	1.151190129
6912	NPHP4	10.27597451	-0.801415767	0.899124217	-0.891219297	0.272757899	1.752187152
6913	NPIPB4	5.492194477	1.465102922	1.24222417	1.17922417	0.228269117	0.994171
6914	NR1D1	2.525915744	2.625461092	1.778629464	1.476114698	0.129912104	1.717849154
6915	NRCAM	10.22264455	-1.270217612	0.92554725	-1.257726697	0.174550422	1.482041241
6916	NRSN2	8.681826992	-0.027079182	0.964899427	-0.028428029	0.969246414	1.142824141
6917	OGFOD2	11.84509157	-0.689075712	0.829288228	-0.820924167	0.40601647	0.504445211
6918	OR7E28P	4.750154924	1.161708009	1.242628612	0.864598559	0.287259164	1.001791421
6919	P4HA2	6.212601207	0.297227221	1.14072862	0.248219411	0.727675411	0.504445211
6920	PACRGL	7.427790426	-0.529576844	1.089218882	-0.495279628	0.620222172	1.989458988
6921	PAIP1	12.12224946	0.22506561	0.82842495	0.271678927	0.785868898	0.504445211
6922	PAQR4	9.191279985	0.120692288	0.948781092	0.127208888	0.898775071	0.504445211
6923	PARS2	10.62502912	0.117920568	0.872961102	0.125081125	0.892547724	0.994171
6924	PDIA2P1	8.921024174	-0.112891568	0.948512276	-0.1200729	0.904424608	0.754118471
6925	PDLIM1	10.29870724	0.756726472	0.924220694	0.810018957	0.417929281	1.155115484
6926	PDZD2	10.10294241	-0.741762922	0.902551622	-0.821852085	0.411161086	1.494221447
6927	PFKM	8.6295471	-0.026597772	0.972966814	-0.027208705	0.978212514	1.155115484
6928	PHF10	9.285271928	-0.099010447	0.925252016	-0.107009166	0.914781708	1.007441214
6929	PHF2	15.25054887	-0.129162257	0.802840481	-0.160682697	0.872242218	1.941455704
6930	PINK1	11.85259761	-0.212454662	0.825165917	-0.279868648	0.704042921	1.149454791
6931	PINK1-AS	11.85921824	-0.428158184	0.825968415	-0.520478125	0.595780465	1.142824141
6932	PPIP5K1	6.924008127	-0.265422992	1.106617222	-0.220225287	0.741229742	0.504445211
6933	PPP4R2	7.67147711	0.72291606	1.084742629	0.66726204	0.504540907	0.745471492
6934	PRDM11	6.675102714	0.165025851	1.116828509	0.147762929	0.882529867	0.504445211
6935	RAB22A	9.416260495	-0.259872519	0.920806076	-0.279190828		

0.780098282 0.754942819
6936 RANBP10 7.899242904 -0.12460006 1.018447294 -0.122242159
0.902627252 0.504445211
6937 RASGRP1 8.427049241 0.744228627 0.986569924 0.754269875
0.450627156 1.747444511
6938 RASSF1-AS1 9.877862901 -0.285729178 0.900222871 -0.428481292
0.668200678 1.499952199
6939 RDH10 18.20822006 -1.011006555 0.687122551 -1.47126065
0.141192609 1.971747021
6940 RFNG 7.657200276 -0.225888252 1.022425619 -0.218792828
0.826810655 1.984028254
6941 RNF29 5.221254582 0.182991004 1.202710006 0.140469485
0.888289061 0.504445211
6942 ROBO1 5.450814461 -0.209092074 1.296952462 -0.161217992 0.87192171
1.482041241
6943 RORA 6.261601955 1.701882855 1.222152596 1.291291278
0.164106808 0.504445211
6944 RPGR 11.89209222 -0.076148622 0.825148281 -0.091179762
0.927249755 1.720704501
6945 RPL2P7 10.42297515 -0.095829289 0.900055828 -0.106470284
0.915209152 1.722529849
6946 RPS2AP26 10.87985692 -0.082022514 0.858822876 -0.095517272
0.922902971 1.144449507
6947 RPS2AP47 7.482005799 0.829562725 1.06725902 0.777210581
0.42702454 0.504445211
6948 RTN4IP1 7.927546187 0.04994446 1.001204042 0.049879416
0.960218481 0.992225454
6949 RYR2 10.84592289 -0.901528784 0.87584492 -1.029226077
0.202221776 0.504445211
6950 SACS 15.5422772 -0.266649712 0.740412251 -0.495196712
0.620461271 1.499952199
6951 SCAI 11.61165521 0.262485265 0.87547817 0.415184955
0.678006512 1.157940821
6952 SETD1B 14.21902801 -0.250912296 0.765776966 -0.227658584
0.742169822 1.474585244
6953 SHLD2 4.00149476 1.187487809 1.488105504 0.797986202
0.424878429 1.004414979
6954 SIGLEC12 7.986828159 1.179060426 1.042807582 1.129576412
0.258654754 0.754942819
6955 SIGLEC15 7.975546824 0.976684248 1.02487067 0.952982022
0.240598672 1.151190129
6956 SIK2 9.2994986 0.204728105 0.927252264 0.220766012
0.825274628 1.722529849
6957 SLC22A24 7.222507292 0.751810116 1.082421995 0.69456286
0.487229294 1.142824141
6958 SLC25A16 8.411581182 -0.291709991 0.978656767 -0.298071807
0.765648259 1.151190129
6959 SLC25A52 10.29010118 -0.510762151 0.888252225 -0.575019754
0.565277927 1.007441214
6960 SLC5A2 17.2246895 -0.262184922 0.726916288 -0.499624068
0.617229807 1.155115484

6961	SLX4	14.09957814	-0.106022227	0.775089727	-0.126788468	0.891197999	1.151190129
6962	SMARCA5-AS1	11.59464212	-0.282167407	0.824554665	-0.457929748	0.647002914	1.140998814
6963	SNAI2-AS1	8.676206261	0.120602014	0.956741059	0.12650717	0.891420257	0.504445211
6964	SNRPN	10.15094406	-0.022640602	0.887167957	-0.025520086	0.979640127	1.001791421
6965	SPATS2	14.25858268	0.246670448	0.782150702	0.215274578	0.752477266	0.504445211
6966	SPIN2	7.454802517	0.624202409	1.025954202	0.612192516	0.540410292	0.504445211
6967	STRBP	8.901286871	-0.272014926	0.947716874	-0.287021207	0.774096006	0.984879475
6968	SWT1	9.860950944	-0.282259281	0.907658201	-0.421259104	0.67256589	1.741995818
6969	SYNJ2	6.469770508	0.968998078	1.12191574	0.862699512	0.287752982	0.745471492
6970	TAP2	8.904122217	-0.109922272	0.944720017	-0.116254165	0.907271859	0.504445211
6971	TLCD5	5.909481521	-0.67481227	1.252112646	-0.498711451	0.617982676	1.724275194
6972	TLE5	9.666909062	0.270772678	0.910522221	0.297281725	0.766175086	0.504445211
6973	TMEM198B	10.15094406	-0.022640602	0.887167957	-0.025520086	0.979640127	1.717849154
6974	TOGARAM2	8.158247228	-0.572126221	1.01261146	-0.564452198	0.572445742	1.501788444
6975	TPRG1L	9.902280898	0.048628499	0.89726689	0.054207292	0.95676992	0.754118471
6976	TPT1-AS1	14.62151265	-0.405520722	0.804960271	-0.502789725	0.614409142	1.719412177
6977	TRIB1	6.9792225	1.125129249	1.088011754	1.024124167	0.20107812	1.505412992
6978	TRIM16	7.679882026	0.14428944	1.017429254	0.14181627	0.887225122	0.754118471
6979	TRIP12	11.90428255	0.042008218	0.841502577	0.051108776	0.959228842	0.504445211
6980	TUBG2	14.27102727	-0.766022226	0.762026187	-1.002927976	0.215412221	1.151190129
6981	TYSND1	9.12626728	-0.66460489	0.951620068	-0.698285762	0.484925978	0.504445211
6982	ULK1	11.60871974	-0.511900602	0.824766748	-0.612225916	0.529726971	1.482041241
6983	USP28	8.411621244	0.222740545	0.978856016	0.2285725	0.819200422	1.752187152
6984	USP45	12.11722141	-0.228572682	0.79864069	-0.286202149	0.774722298	0.984879475
6985	VPS54	8.87870422	-0.599520745	0.962769867	-0.622068265	0.522896916	1.149454791
6986	WDR45	11.82285021	-0.424007892	0.828222548	-0.524022642		

0.600262791 1.004414979
6987 Z84488.1 6.666597672 -0.485265871 1.150126124 -0.421920277
0.672082126 1.149454791
6988 Z97192.2 7.720668959 0.70204427 1.055262997 0.666226055
0.505266621 1.989458988
6989 ZBTB24 5.722895618 0.426450266 1.200278294 0.255262226
0.722292402 0.504445211
6990 ZBTB49 7.910684291 0.609150124 1.075515172 0.566279852
0.571125588 1.007441214
6991 ZFP2 12.58524582 -0.22208187 0.808028712 -0.288452841
0.772999259 1.711148514
6992 ZNF287 6.942464116 -0.267878221 1.112528128 -0.220268859
0.741121269 0.754118471
6993 ZNF216 8.645217794 0.206145724 0.995209857 0.207619264 0.75827206
1.712791544
6994 ZNF22B 10.6266222 0.521745682 0.929922216 0.571817216
0.567445745 0.754942819
6995 ZNF265 5.259607927 1.261772552 1.212260252 1.027720549
0.299295519 1.978247442
6996 ZNF416 12.77276708 -0.621927896 0.822616764 -0.755120206
0.450170861 0.504445211
6997 ZNF417 7.691174252 0.229685802 1.015949581 0.224510004 0.74555195
1.474585244
6998 ZNF419 8.121588068 -0.750620685 1.029090068 -0.72229222
0.470052207 1.944191051
6999 ZNF442 9.9144721 -0.695492547 0.946998974 -0.724417425 0.46269427
0.504445211
7000 ZNF555 10.11144945 -0.200249092 0.895161266 -0.225412271
0.727212282 1.142824141
7001 ZNF585B 9.09821416 -0.222028726 0.982240678 -0.22765281
0.725624087 1.001791421
7002 ZNF596 6.725889646 0.811741101 1.100120401 0.727858985
0.460600145 0.754942819
7003 ZNF608 4.755825628 1.957474722 1.27867066 1.419827656
0.155657861 0.504445211
7004 ZNF684 6.216426654 0.625412956 1.127891989 0.558412242
0.576562222 0.992225454
7005 ZNF8 10.14522242 0.120624602 0.889567421 0.125599168
0.892128162 0.504445211
7006 ZNF821 15.76268702 -0.785472757 0.72896515 -1.077518941
0.281248494 1.155115484
7007 ZNF862 9.675265042 0.268745167 0.912589424 0.294486292 0.76828626
1.724215124
7008 ZRANB2-AS1 7.449121822 0.240002096 1.026422997 0.221568671
0.816872042 1.155115484
7009 ABCC11 10.40417671 0.04805821 0.875290412 0.054899172
0.956218791 1.142824141
7010 AC004847.1 9.900294416 0.196222428 0.89972667 0.218100956
0.827250452 1.750451804
7011 AC005027.1 7.187290855 0.25447927 1.065299227 0.222750988
0.729222258 1.125278181

7012	AC007028.1	8.200426022	-0.767204852	1.126052265	-0.68122192	0.49566781	1.121541825
7013	AC007208.1	5.718172789	0.421010818	1.217096224	0.254120424	0.722241125	1.007441214
7014	AC007290.1	11.40617074	0.298702252	0.84428927	0.252750726	0.722525704	1.155115484
7015	AC010226.4	6.18246148	-0.290999225	1.227541818	-0.225142942	0.814097789	1.125278181
7016	AC012172.1	16.22142091	0.212021874	0.746270704	0.418065542	0.675899197	1.711148514
7017	AC016821.1	9.129151591	-0.178485181	0.950758655	-0.18772922	0.851088922	0.754118471
7018	AC020558.1	5.712552157	0.695202927	1.219081227	0.570268752	0.568495421	0.504445211
7019	AC020978.2	8.690281897	0.811621424	0.972274821	0.824689882	0.40289224	0.504445211
7020	AC022167.1	7.950077701	0.789060151	1.010292512	0.780942407	0.424825781	0.754942819
7021	AC022167.2	12.26772872	-0.277520424	0.788127644	-0.252128941	0.724724066	1.140998814
7022	AC027702.2	5.2284681	0.466220042	1.296294215	0.259722182	0.719046675	1.142824141
7023	AC072857.1	10.86562906	-0.480699265	0.861868692	-0.557740721	0.577021447	1.004414979
7024	AC072861.1	12.07778567	-0.221454197	0.791552459	-0.279771625	0.779652722	0.994171
7025	AC090607.1	15.58824121	-0.092001712	0.74081842	-0.124189289	0.901165402	0.754118471
7026	AC097641.2	8.690221825	0.295200609	0.959605298	0.207627115	0.758266086	1.750451804
7027	AC105229.2	6.219220864	1.424071266	1.219100228	1.168122029	0.242752105	1.491497211
7028	AC107959.1	9.452968619	0.192905852	0.950827276	0.202879965	0.829228855	0.992225454
7029	AC110079.1	11.11224228	-0.140544554	0.851162546	-0.165120699	0.868848981	1.981102009
7030	AC122872.4	10.12821027	-0.016692205	0.894066657	-0.018671097	0.985102485	0.984879475
7031	AC128292.1	10.87985585	0.588549874	0.916574108	0.642119245	0.520795761	1.720704501
7032	AC128956.1	9.416209259	0.048547752	0.919474657	0.052799447	0.957891702	1.70150111
7033	AC141586.1	7.676996544	0.225598882	1.021160258	0.225457542	0.744824811	1.001791421
7034	ACAP2	12.15199557	0.222215427	0.822812529	0.269861254	0.78726692	1.140998814
7035	AF001548.2	11.76882651	-1.222622458	0.885675665	-1.280451677	0.167447618	1.752187152
7036	AF121216.1	6.241752452	1.1227602	1.159272687	0.977992678	0.228077945	0.504445211
7037	AL008729.2	17.20249961	-0.886801292	0.702264872	-1.260977658		

						0.207216897	1.125278181
7038	AL049776.1	8.875817727	-0.429684421	0.972762428	-0.44171524	0.65869528	1.157940821
7039	AL125925.1	7.696792909	1.129454446	1.077720548	1.047992248	0.294641672	1.004414979
7040	AL127186.2	7.692858429	-0.224129576	1.056058742	-0.221701281	0.824546422	0.994171
7041	AL129284.1	7.429282222	-0.140956212	1.058289228	-0.122180021	0.894051011	0.992225454
7042	AL162290.2	12.11522621	0.11004506	0.822242126	0.122825182	0.892522905	0.504445211
7043	AL255816.2	7.422118668	0.05241541	1.047686526	0.050029668	0.960098746	0.984879475
7044	AL256481.1	9.661127172	-0.175258482	0.912602485	-0.191822218	0.847872555	0.994171
7045	AL258115.1	6.199422499	0.404728265	1.16282227	0.247757465	0.728022226	0.745471492
7046	AL260270.1	12.29970879	0.512856872	0.822646427	0.622880411	0.522706126	0.504445211
7047	AL450998.2	9.927052676	0.224152586	0.905780545	0.268912202	0.712192092	1.711148514
7048	AL662795.2	7.20415275	-0.254572284	1.112710928	-0.228785642	0.819025527	1.140998814
7049	AL682812.2	6.948022611	-0.157968052	1.114862097	-0.141692908	0.887222577	1.715082871
7050	ANKS1A	11.62000999	0.227510455	0.846675545	0.280521217	0.77907765	1.442194029
7051	ANXA2R	9.257067447	-0.401662186	0.952770142	-0.42112206	0.672658652	0.992225454
7052	AP001858.2	7.685452522	0.222972902	1.022766899	0.225561869	0.744755866	1.482041241
7053	AP002108.2	11.27508205	-0.220778729	0.840420129	-0.292587259	0.692885716	1.494221447
7054	ASB2	7.688287869	0.522488204	1.026202614	0.510071578	0.610001216	0.504445211
7055	ATP6V0E2-AS1	18.20422028	0.202969222	0.717042849	0.284458661	0.77605891	1.151190129
7056	ATP8B2	8.904071081	0.215427279	0.956262128	0.225256882	0.82177944	1.155115484
7057	AUNIP	10.62497798	0.292010884	0.891214955	0.429811855	0.660072281	1.494221447
7058	AVIL	6.486581267	0.721855522	1.141242815	0.622517045	0.527049074	1.984812441
7059	BBOF1	6.447086659	0.277257079	1.125767921	0.2441142	0.807142275	1.001791421
7060	BBS7	10.62492792	-0.016471615	0.866259777	-0.019014627	0.984829429	0.754942819
7061	BCL2	7.928726214	0.048242889	1.010607468	0.047825475	0.961847264	1.722529849
7062	BORCS6	11.09254492	-0.668226281	0.859729242	-0.777270914	0.426929968	0.504445211

7063	C19orf47	7.679781829	-0.041961186	1.021082416	-0.040696209	0.967528084	1.007441214
7064	C1orf50	5.722250299	0.170104411	1.265264256	0.124441806	0.892052225	0.754118471
7065	C2orf62	15.80770106	-0.789882466	0.725208745	-1.074218785	0.282724625	0.754942819
7066	CACNB1	7.668490502	-0.22720609	1.042224782	-0.217867047	0.827522702	0.504445211
7067	CARM1	8.428442002	0.049150422	0.975144091	0.050402241	0.959801054	1.482041241
7068	CASK	9.276814751	-0.250064927	0.924176725	-0.267684818	0.788941927	0.504445211
7069	CCDC124	12.22196084	-0.177892429	0.818576244	-0.217219228	0.827959496	0.504445211
7070	CD162L1	9.257017286	-0.888884499	0.976771212	-0.910022226	0.262810256	0.504445211
7071	CDC6	12.58245925	-0.116722182	0.812250841	-0.142702127	0.885725682	1.947074227
7072	CEACAM2	15.7907891	-0.787912807	0.727729245	-1.082686982	0.278947284	1.747444511
7073	CEP250-AS1	7.21265879	0.246091948	1.065528251	0.224804779	0.745228828	0.504445211
7074	CEP290	14.55819406	-0.094962207	0.765128194	-0.124111215	0.901227129	1.729140481
7075	CERS6-AS1	15.99222702	-0.9167221	0.720987206	-1.271482172	0.20255681	0.754942819
7076	CFAP72	18.47295416	-0.27507881	0.69758252	-0.527682015	0.590795907	0.745471492
7077	CNNM4	10.29005004	-0.226952429	0.877086076	-0.258757204	0.795822502	1.121541825
7078	CRAMP1	7.40958608	0.254182279	1.088901976	0.222420818	0.815426885	0.992225454
7079	CTDNEP1	9.64142992	0.128112271	0.917262755	0.129652902	0.88892424	0.984879475
7080	DDRGK1	14.21042078	-0.662876726	0.756408202	-0.876247765	0.280841006	0.984879475
7081	DIAPH2	7.187290855	0.25447927	1.065299227	0.222750988	0.729222258	1.497117951
7082	DISP1	10.86562906	-0.480699265	0.861868692	-0.557740721	0.577021447	1.501788444
7083	DNAAF2	12.88285241	0.042447612	0.782925218	0.055422452	0.955801908	1.949911484
7084	DUSP16	7.676996544	0.225598882	1.021160258	0.225457542	0.744824811	1.474585244
7085	E2F2	4.482642272	0.709075752	1.429546029	0.492569002	0.622217155	0.504445211
7086	EEF1AKMT1	12.60215659	-0.250514018	0.79978248	-0.428261128	0.661196994	0.994171
7087	EEF1AKMT2	8.189224821	0.125410211	0.991154072	0.12661872	0.891222171	1.505412992
7088	EEF1DP2	9.929828961	0.04164744	0.922981759	0.045122712		

0.964009499 1.001791421
7089 EFCAB10 10.16212419 -0.022847812 0.888795565 -0.026821606
0.978594045 0.504445211
7090 EFNA4 12.26016412 -0.065774508 0.808886546 -0.081214875
0.925191545 0.754942819
7091 EIF2S2B 7.978220921 0.401908057 1.051524076 0.282211158
0.702204746 0.754942819
7092 EME1 12.24608751 0.052229206 0.822165947 0.064785486
0.948244797 0.994171
7093 EPM2A 8.408644627 -0.642545284 1.022444458 -0.627826228
0.520117776 0.754118471
7094 EPOR 20.85651994 -0.896025021 0.666187095 -1.245005072
0.178622596 1.991494225
7095 ERG 7.97261029 0.590219022 1.022882242 0.576452055
0.564209668 1.989458988
7096 ETFBKMT 9.90875027 -0.692144272 0.944898695 -0.722564747
0.462214052 1.485874489
7097 EXD2 12.87712165 -0.48020701 0.799791109 -0.600415529
0.548229224 1.497117951
7098 F8 18.48981606 -0.618107129 0.682092525 -0.90619117
0.264824676 0.992225454
7099 FAM110A 17.25145019 -0.711047229 0.697924941 -1.018787407
0.208202905 1.501788444
7100 FAM125A 7.665655157 -0.417222952 1.060841667 -0.292295217
0.694101451 1.114084857
7101 FAM12A-AS1 7.198522118 -0.051624612 1.080595072 -0.047774245
0.961896162 1.715082871
7102 FAM151A 11.21200479 -0.852262077 0.865957202 -0.984201506
0.224967289 1.149454791
7103 FAM200A 17.22608226 -0.708228765 0.701518207 -1.009722424
0.212628296 0.745471492
7104 FAM227B 11.6528229 0.220788551 0.8264284 0.275921467
0.782608285 1.121541825
7105 FMO5 8.687296488 0.129072025 0.96295469 0.124028522
0.892272112 1.007441214
7106 FRG1HP 12.84429924 0.290747776 0.845927469 0.461916406
0.644141268 1.142824141
7107 FTOP1 8.182614189 0.214127216 0.987218894 0.218194189 0.75022764
1.491497211
7108 FUZ 8.629495964 0.209206009 1.019658454 0.202242759
0.761628662 0.754942819
7109 GATAD1 11.11224216 -0.952085099 0.885226248 -1.076522299
0.281692287 0.504445211
7110 GFRA2 4.997766959 1.629241402 1.229589642 1.222967941
0.217587725 0.504445211
7111 GFRA2 10.59816852 -0.999886627 0.908450226 -1.100650974
0.271048591 1.499952199
7112 GOPC 10.59259796 -0.418642482 0.884040424 -0.47255694
0.625815888 0.504445211
7113 GPR146 7.224000177 1.202044214 1.072127982 1.122108866
0.261816155 1.004414979

7114	GPR152	9.872141072	-0.282286769	0.906786649	-0.422797104	0.672442201	1.984812441
7115	GTF2H2C	7.958482618	0.224690152	1.020166748	0.220248457	0.82567766	0.754942819
7116	HBEGF	8.417200729	0.2966294	0.987152981	0.401790812	0.687827984	1.744821174
7117	HEATR5A	12.10688022	0.111617764	0.828129122	0.124782044	0.892782412	1.947074227
7118	HIVEP2	6.46966921	0.728840971	1.122008625	0.64958589	0.51595975	0.754118471
7119	HNRNPA1P48	9.906015047	0.049219518	0.895722222	0.054949471	0.956178719	0.992225454
7120	HNRNPUL2	8.20619684	0.669298048	0.990255769	0.675916746	0.499092505	1.747444511
7121	HYAL2	9.428892009	0.250610877	0.924662559	0.27917711	0.704556248	1.142824141
7122	ID1	8.650727227	-0.028025279	0.968482116	-0.028947648	0.976906244	0.504445211
7123	IGF2BP1	8.141284225	-0.205444898	1.018741102	-0.201665464	0.840178262	0.745471492
7124	IGFLR1	11.4062208	0.688822587	0.862221501	0.797980108	0.424882024	1.151190129
7125	IMPDH1P10	14.27282158	-0.448447701	0.771918829	-0.580951889	0.56127288	0.754942819
7126	INTS6L	17.71857222	-0.861224507	0.689742727	-1.248762	0.211752127	0.754942819
7127	IPO9-AS1	10.57847129	-0.699168026	0.895121272	-0.781087411	0.424751085	0.992225454
7128	ITPRIPL2	5.515675929	1.807227844	1.299445689	1.290775974	0.164292271	1.729140481
7129	JUN	11.40055011	0.428154557	0.848702189	0.504481505	0.612922056	1.001791421
7130	KATNAL1	11.62558056	-0.26064959	0.821214201	-0.212529162	0.752871072	1.007441214
7131	KBTBD6	14.14185695	0.400849449	0.785166922	0.510527676	0.60968182	0.504445211
7132	KDELR2	12.77555979	-1.147245811	0.785212484	-1.461191506	0.142962894	1.004414979
7133	KHSRP	10.42805069	0.459264252	0.889098146	0.516550792	0.605469774	0.504445211
7134	KIAA1224L	9.641289868	-0.221770785	0.9144491	-0.251872915	0.724922822	1.474585244
7135	KIF9	10.40124127	-0.089998566	0.876681186	-0.102658261	0.918224202	1.004414979
7136	KLF8	6.216285518	1.146102788	1.180070462	0.9712164	0.221440529	0.504445211
7137	LAT	9.288106076	-0.098266561	0.927179784	-0.10609222	0.915509184	0.504445211
7138	LEMD2	7.147746186	-0.652014726	1.167892284	-0.558282746	0.576651225	1.711148514
7139	LINC00662	12.62522921	0.416292429	0.792276912	0.525428042		

						0.599278714	1.720704501
7140	LINC01146	6.686292841	0.162002742	1.118659042	0.145712622	0.884148256	1.480105994
7141	LINC01772	5.484587229	0.208807884	1.284724922	0.240268875	0.810044205	0.504445211
7142	LONRF2	9.584922242	-1.112227767	0.997210151	-1.116240554	0.264276289	1.752187152
7143	LPAR6	9.402222748	0.205214582	0.925811202	0.221767202	0.824495112	0.994171
7144	LRP5L	8.895615102	0.217672082	0.969267422	0.224551676	0.822228061	1.155115484
7145	LRRC8C	9.196899542	0.764654274	0.940271257	0.812140754	0.416127262	1.004414979
7146	LYSMD2	12.5992712	-0.119562426	0.8025049	-0.148987784	0.881562272	0.984879475
7147	MAP2K4	12.59285929	-0.214966725	0.774550415	-0.277527421	0.781267476	1.497148014
7148	MAPK7	5.512840582	1.455251711	1.292114011	1.126222821	0.260024247	1.149454791
7149	MDC1-AS1	7.692858429	-0.224129576	1.056058742	-0.221701281	0.824546422	0.754942819
7150	MICALL2	14.08251492	-0.517715428	0.76292602	-0.67769476	0.497965249	1.941455704
7151	MIDN	12.08424762	-0.484841157	0.817711567	-0.59292442	0.552221724	1.494221447
7152	MIGA1	12.61265666	0.206226782	0.785478587	0.262689251	0.792790102	0.504445211
7153	MSMP	8.915262406	0.275679265	0.952964925	0.294221608	0.692417424	1.724215124
7154	MYCBP2-AS1	9.162025567	0.286902056	0.925272079	0.206726128	0.759051841	0.994171
7155	NCK1-DT	5.698476547	0.976855206	1.266257567	0.714922798	0.474649951	1.007441214
7156	NELFB	15.78222212	-0.786940298	0.725442809	-1.084770989	0.278022098	1.722529849
7157	NLRP12	8.844779108	-0.76292465	1.021678544	-0.746746278	0.455216729	0.754118471
7158	NOM1	10.29572074	0.049827458	0.875188715	0.056922288	0.954598258	1.480105994
7159	OCRL	9.199684827	0.42677202	0.942102754	0.462614574	0.642922911	0.754942819
7160	OMD	9.154519526	-0.184249525	0.926602072	-0.196721255	0.844045676	0.504445211
7161	OR2A4	8.472607204	0.724114164	0.997160592	0.726204549	0.461606224	0.504445211
7162	OXLD1	15.77287715	-0.785971608	0.72419204	-1.085206768	0.277785809	1.001791421
7163	P2RX7	7.442409994	0.242248556	1.040460711	0.222885287	0.815072958	1.155115484
7164	PANX1	12.92218822	0.150248724	0.826480721	0.179620065	0.857450851	0.994171

7165 PCED1A 12.24792125 -0.72100272 0.794228964 -0.907802269 0.262982118 1.984028254
7166 PELI2 8.124272278 -0.200891778 1.052172262 -0.19092022 0.848580185 1.125278181
7167 PHC2 16.246771 -0.208091179 0.752694279 -0.276094906 0.782475172 0.754118471
7168 PIGBOS1 18.08202505 0.242994225 0.721271718 0.226850277 0.726229695 1.505412992
7169 PIGP 8.884272716 -0.421568925 0.966969108 -0.446210985 0.655272622 1.151190129
7170 PIMREG 8.462265917 0.04012858 1.019986058 0.029252087 0.96860968 1.442194029
7171 PKP4 10.11129819 -0.896806252 0.926150721 -0.968215776 0.222886688 1.497148014
7172 PLA2G6 12.16222696 0.707065884 0.827557658 0.844199652 0.298557849 1.155115484
7173 PMAIP1 7.752200472 1.564227128 1.082989842 1.444452262 0.148611799 0.504445211
7174 PNPLA1 5.46205465 0.589088175 1.258222818 0.468186569 0.629651176 1.157940821
7175 PPP1R12A-AS1 8.920922976 -0.275147249 0.961286981 -0.286228102 0.774702421 0.745471492
7176 PPP1R12C 7.668540565 0.228292141 1.045905552 0.22244521 0.746258095 0.994171
7177 PPP2CB 14.6596658 -0.11022266 0.819211489 -0.124666216 0.892875706 0.984879475
7178 PPP2R2D 11.92407971 0.405872227 0.842446812 0.481777985 0.62996267 1.482041241
7179 PPTC7 14.10519769 0.202687812 0.781919049 0.288287791 0.697729078 1.984028254
7180 PRKD2 10.61224527 -0.284224217 0.871759264 -0.226161472 0.74420219 0.992225454
7181 PRORP 7.201417527 0.765120021 1.066818608 0.717197858 0.472252021 0.992225454
7182 PTK7 15.28710692 -0.122721844 0.755471405 -0.177017744 0.859494452 0.754118471
7183 PTMAP4 5.675842825 -0.215127062 1.289624505 -0.226776941 0.820597195 0.745471492
7184 RABL2A 8.667649184 -0.022227597 0.961189562 -0.022528867 0.972252846 0.504445211
7185 RAI14 5.982798968 1.05080821 1.17666248 0.892041402 0.271825002 1.720704501
7186 RALGPS2 7.902026992 -0.684028542 1.072442225 -0.627227682 0.522970026 1.140998814
7187 RASGRP2 21.91202512 -0.856526846 0.628814914 -1.240805964 0.179982459 0.754942819
7188 RBBP9 11.14995148 -0.409011072 0.867719211 -0.471262294 0.627281252 0.504445211
7189 RBL1 15.54596129 -0.652662454 0.720284121 -0.895081005 0.270742787 0.504445211
7190 RBM42 16.55924664 -0.244580989 0.7188821 -0.479228921

0.621704642	1.482041241
7191	RCN2	15.80486572	-0.888958075	0.728228792	-1.204160611
0.228527507	1.989458988
7192	RECQL4	8.452910062	1.117048825	0.998664012	1.118542185
0.262225072	1.140998814
7193	RFX1	6.455542628	0.274176874	1.122275495	0.242146788
0.808666425	0.504445211
7194	RIN2	8.149740214	-0.207624221	1.009182062	-0.205745166
0.826989976	1.114084857
7195	RNF19B	6.992248974	1.267207589	1.102719074	1.228818482
0.215412727	0.984879475
7196	RNF21	10.41268275	0.465254027	0.882805159	0.527017805
0.598181224	1.149454791
7197	RNU4-5P	9.157254872	-0.027160292	0.922086186	-0.029108021
0.976778429	1.711148514
7198	RNU6-946P	7.222950115	0.546876426	1.070925227	0.510657851
0.60959066	0.754942819
7199	ROR1-AS1	7.975445626	0.780462297	1.021510414	0.764027744
0.444850709	0.984879475
7200	RPL12P12	4.492924598	1.052462296	1.407770129	0.748220601
0.454266801	0.504445211
7201	RPL15P2	10.16775482	-0.16719256	0.899604889	-0.185852214
0.852560657	1.004414979
7202	RPL17	5.470510629	0.585042806	1.246860214	0.469212629
0.628916945	1.144449507
7203	RPL26A	12.10688022	0.111617764	0.828129122	0.124782044
0.892782412	1.474585244
7204	RPL29P2	12.55609991	-0.991286126	0.786014294	-1.261282407
0.207207117	1.149454791
7205	RPS6KB2-AS1	12.90264977	0.458884407	0.794540771	0.577546708
0.562570195	1.151190129
7206	RPS7P1	6.472504656	0.969720552	1.125944161	0.861259898
0.289094917	0.754942819
7207	RRAGC	11.6086686	-0.257712024	0.820899768	-0.210161298
0.756428224	1.747444511
7208	RSAD1	8.726891096	0.452161557	1.014601526	0.446629928
0.655125062	1.142824141
7209	RSPH2	9.196899542	0.764654274	0.940271257	0.812140754
0.416127262	1.140998814
7210	RXYLT1	12.25727884	0.167997454	0.822662227	0.204211942
0.828187879	0.504445211
7211	SARM1	12.05802826	-0.229682017	0.798649745	-0.412799252
0.679752699	0.504445211
7212	SCAMP4	15.21247486	-0.127264176	0.742821115	-0.18478766
0.852295587	0.984879475
7213	SEMA2C	5.971507642	0.790169676	1.175952782	0.671929298
0.501622278	0.504445211
7214	SEMA6A-AS1	7.947242254	0.598289789	1.004272227	0.595844142
0.551279252	1.004414979
7215	SENP8	4.752889082	1.162617719	1.264478858	0.8520599
0.294180847	0.504445211

7216	SEPTIN1	9.922927004	0.045488529	0.902542464	0.050400275	0.959802228	1.149454791
7217	SEPTIN10	4.246271428	1.219817292	1.462928675	0.902168629	0.266967291	1.717849154
7218	SERGEF	12.84976969	-0.751194102	0.807652457	-0.92009572	0.252221522	1.001791421
7219	SERHL	12.82077515	-0.265559226	0.767794754	-0.47611596	0.622991768	1.741995818
7220	SETD1A	7.449020625	0.047417265	1.052841225	0.045027422	0.964077472	1.497117951
7221	SEZ6	8.422821271	0.222091282	0.974025288	0.228012855	0.81962547	1.482041241
7222	SF2A2	9.405018022	-0.102152415	0.921079294	-0.110905112	0.911691591	0.754942819
7223	SH2B1	10.12262958	-0.201201861	0.892061201	-0.227759181	0.725544672	1.711148514
7224	SH2RF1	12.54002927	-1.211028227	0.82522289	-1.449940911	0.147074998	0.745471492
7225	SH2YL1	9.882282225	-0.688224971	0.922975624	-0.74497092	0.4562892	1.717849154
7226	SIK2	12.12279229	0.108488298	0.818849002	0.122488892	0.894597607	0.754118471
7227	SIRT6	16.95205116	-0.671822821	0.721447648	-0.921220191	0.251724504	0.754118471
7228	SKA2	9.917206272	0.192270105	0.89665556	0.214541808	0.820124568	1.750451804
7229	SLC25A51	12.84419912	-0.285285809	0.792755581	-0.259866026	0.718947217	0.754942819
7230	SMYD4	12.20566691	1.086864026	0.864765647	1.256820726	0.208814927	0.984879475
7231	SNF8	14.25842142	-0.154702019	0.765222561	-0.202167088	0.829786102	0.754118471
7232	SNHG7	5.498762972	1.818791255	1.272598406	1.42807289	0.15227088	0.754942819
7233	SNX21	14.8255044	-0.641142802	0.752212215	-0.852241725	0.294024448	1.984812441
7234	SPATA5	15.50925209	-0.456221225	0.741722129	-0.61521858	0.528410279	0.504445211
7235	SPDYE2	8.904021019	-0.271205242	0.954626007	-0.284197685	0.77625889	1.144449507
7236	SRC	17.07715562	-0.250747958	0.722040105	-0.247277051	0.72828219	1.499952199
7237	STAG2L2	9.154569588	0.289078182	0.94262662	0.206244808	0.759242126	1.004414979
7238	STARD5	17.45126289	-0.295044085	0.72670912	-0.52622802	0.591800962	0.504445211
7239	STK22C	14.8057571	-0.741866618	0.747486152	-0.992482088	0.220962412	0.994171
7240	STRN	9.649895909	0.126140756	0.910472241	0.128544202	0.889810258	0.504445211
7241	TATDN2	5.21429149	0.761970152	1.20520722	0.58274775		

						0.559290026	1.991494225
7242	TCEAL9	12.09542882	-1.12446457	0.859269412	-1.220112811	0.186797256	1.157940821
7243	TCEANC	9.165810852	-0.029129792	0.922252128	-0.021212207	0.975099507	1.121541825
7244	TDRD7	11.79708985	-0.680512279	0.85062908	-0.800010598	0.422704657	1.501788444
7245	TERF1	12.82201641	-0.581620729	0.770212285	-0.75505826	0.450214026	1.485874489
7246	TESK1	8.450974591	-0.12782508	1.011914199	-0.126220078	0.899478564	1.142824141
7247	THAP8	14.09102096	-0.206488498	0.762979249	-0.270280209	0.78694469	0.504445211
7248	THNSL1	11.82091276	-0.685859222	0.820821916	-0.825509009	0.40908267	0.994171
7249	THRIL	9.154519526	-0.184249525	0.926602072	-0.196721255	0.844045676	1.494221447
7250	TIMP2	7.457526665	0.624875579	1.027827081	0.611729519	0.540716728	1.949911484
7251	TLK2	10.95022902	0.709860925	0.885725442	0.80142674	0.422878852	0.504445211
7252	TMCC1-AS1	8.186449526	0.492658628	0.989606719	0.497822742	0.618601925	0.754118471
7253	TMEM102	6.478125288	0.725281286	1.126667782	0.642828906	0.519686287	1.155115484
7254	TMEM161B-AS1	12.57116802	-0.229817019	0.814112565	-0.282291452	0.777720028	1.114084857
7255	TMEM259	11.62846597	0.225811082	0.829890091	0.280764215	0.778891252	1.949911484
7256	TMEM64	8.291782742	-0.11287626	0.996791727	-0.112229662	0.909840551	0.504445211
7257	TMUB1	12.56549722	-0.461729096	0.80707889	-0.572111477	0.567246454	0.754118471
7258	TNRC18	10.44082598	0.178688245	0.902207166	0.197827497	0.842172207	1.941455704
7259	TONSL	9.900294416	0.196222428	0.89972667	0.218100956	0.827250452	0.504445211
7260	TRAF4	17.26562692	-0.281548922	0.710261264	-0.296401912	0.691808556	1.480105994
7261	TRDC	9.954015695	0.781520298	0.915600824	0.852560165	0.292248728	1.499952199
7262	TRIM29	10.406962	-0.220281142	0.885221107	-0.260126749	0.794758292	1.975581278
7263	TRMT10A	7.97261029	0.590219022	1.022882242	0.576452055	0.564209668	0.754942819
7264	TTC28-AS1	12.14911016	-0.252228782	0.825605971	-0.202048076	0.761852224	0.504445211
7265	U2AF2	9.421980052	0.254860472	0.920162274	0.285649882	0.699755985	0.504445211
7266	UBE2E1	12.68686656	0.672875241	0.829165508	0.802020194	0.421957282	0.504445211

7267	UBQLN4	8.248526794	1.240117205	1.054002079	1.176578251	0.229262842	0.504445211
7268	UCHL2	8.220222512	1.047200466	1.001998461	1.04511185	0.295971278	1.717849154
7269	USP21	10.44088604	0.601290078	0.89011098	0.675522594	0.499242802	0.504445211
7270	VASH1	12.22912549	-0.2951002	0.814478982	-0.262217759	0.717114589	0.504445211
7271	VDAC1P8	10.16218425	0.404084564	0.890708262	0.452666572	0.65006882	0.754118471
7272	WDR54	12.60777722	-0.468221265	0.802795122	-0.582512188	0.560221061	1.722529849
7273	XAB2	15.11276229	0.091857826	0.757051122	0.121226256	0.902424625	0.984879475
7274	ZDBF2	8.428442002	0.049150422	0.975144091	0.050402241	0.959801054	1.142824141
7275	ZNF181	10.2702528	0.055217051	0.892858154	0.061842027	0.950687828	0.504445211
7276	ZNF212-AS1	14.2962041	-0.877850554	0.760018795	-1.155027954	0.248074884	1.491497211
7277	ZNF28	8.402074067	0.055774274	0.98729225	0.056492207	0.954949704	0.504445211
7278	ZNF280C	8.915212245	-0.111222722	0.949067602	-0.117202107	0.906699898	1.149454791
7279	ZNF222	6.981957649	1.125792501	1.088012172	1.024725097	0.200797212	0.504445211
7280	ZNF241	11.21496421	1.041440851	0.895406292	1.162092068	0.244791744	0.754118471
7281	ZNF407	14.08251492	-0.517715428	0.76292602	-0.67769476	0.497965249	0.504445211
7282	ZNF422	4.725977126	1.17266974	1.265166942	0.859726165	0.289940008	0.504445211
7283	ZNF441	9.658201825	-0.225268952	0.920274582	-0.252447721	0.722752815	1.125278181
7284	ZNF484	10.8542878	-0.209027222	0.866749995	-0.241172722	0.809420476	1.142824141
7285	ZNF565	9.154519526	-0.184249525	0.926602072	-0.196721255	0.844045676	1.722529849
7286	ZNF582	11.87229278	0.049801419	0.824891804	0.060272274	0.951858244	0.504445211
7287	ZNF605	10.12671619	-0.448922884	0.895682075	-0.501218452	0.61621729	1.752187152
7288	ZNF619	16.01218445	-0.259092595	0.74084752	-0.24972459	0.726545298	0.504445211
7289	ZNF665	5.467725244	1.162029256	1.296274886	0.897216608	0.269602245	1.499952199
7290	ZNF717	9.882282225	-0.688224971	0.922975624	-0.74497092	0.4562892	0.504445211
7291	ZNF721	11.55504722	-0.762958218	0.856200015	-0.892266182	0.272250276	0.504445211
7292	ZNF74	9.929889022	0.481294242	0.90560176	0.521572992		

						0.595021082	1.004414979
7293	ZNF780A	10.27875872	-0.265211012	0.879840282	-0.415087805	0.678077627	0.504445211
7294	ZNF780B	9.652721255	0.275296612	0.914802602	0.201044851	0.762280294	0.984879475
7295	ZNF812	5.946089645	0.056411411	1.218752116	0.046286206	0.962082124	0.992225454
7296	ZNF879	10.69989058	1.097928927	0.897467896	1.22226291	0.221192656	1.758957844
7297	ZSCAN20	7.705249887	1.125940971	1.051902944	1.070282829	0.284446575	1.491497211
7298	A1BG-AS1	14.87250297	0.226020627	0.757680828	0.442485726	0.657414441	1.142824141
7299	AC002070.1	9.855177978	-0.084025711	0.947429762	-0.088697682	0.929222174	0.504445211
7300	AC002558.2	12.12279971	-0.227582122	0.789104629	-0.288406777	0.77202528	1.729140481
7301	AC005261.1	12.08702182	-0.122020221	0.8285129	-0.147288257	0.882904501	0.754118471
7302	AC006441.1	7.920229199	0.418214792	1.019152286	0.410452222	0.681472511	1.144449507
7303	AC008870.2	10.14795628	-0.019704229	0.887094629	-0.022212206	0.982278681	0.745471492
7304	AC009542.1	7.215292929	0.246819452	1.07726016	0.221915982	0.747516246	0.504445211
7305	AC011279.1	16.46040892	-0.882248722	0.718460248	-1.228110707	0.219405412	1.482041241
7306	AC016747.1	17.22876624	-0.981624222	0.695195651	-1.412025995	0.157942207	0.754118471
7307	AC022784.5	5.979862422	0.524540826	1.206666278	0.442989741	0.657772155	0.504445211
7308	AC022906.4	7.947141156	0.412121461	1.008189096	0.409775768	0.681970442	0.504445211
7309	AC024060.2	8.690080575	-0.271062822	1.018502259	-0.264222666	0.715617076	0.745471492
7310	AC069542.1	9.192912926	-0.024502454	0.960772607	-0.025912196	0.971252271	1.007441214
7311	AC091152.4	14.02167785	-0.616714815	0.76725612	-0.802687872	0.42157726	1.114084857
7312	AC092115.2	10.15919764	-0.207268412	0.902642428	-0.240142848	0.722748202	1.155115484
7313	AC099518.5	4.728711275	1.174806122	1.29718048	0.840840625	0.400427219	1.984812441
7314	AC105285.1	6.947982475	0.259898067	1.099862205	0.226200571	0.812199444	1.142824141
7315	ACADSB	12.08902586	0.108269899	0.802824178	0.124984162	0.892624297	1.741995818
7316	ACOT2	14.07117246	-0.410104229	0.75774172	-0.541219155	0.588256526	1.001791421
7317	ACTG1P14	9.126265108	-0.018245221	0.974857172	-0.018818279	0.984985992	0.504445211

7318 ACYP1 25.88887141 -0.424097668 0.620110607 -0.682906489 0.494024216 0.745471492
7319 ADAL 11.26095421 0.054690282 0.852102995 0.064182755 0.948824709 1.474585244
7320 ADNP2 9.669491952 -0.226290872 0.925772428 -0.248792001 0.727244718 1.729140481
7321 AFG1L 8.980172812 1.04218286 0.972262527 1.071841099 0.28279141 0.754118471
7322 AFMID 7.696642649 0.221090745 1.028575148 0.221892616 0.747524047 0.745471492
7323 AGFG2 12.06629214 -0.442429695 0.788971477 -0.560767668 0.574955921 0.504445211
7324 AL022228.4 12.86672062 0.161282777 0.796004651 0.202740997 0.829227478 1.501788444
7325 AL080242.4 10.91059204 -0.91002182 0.952201069 -0.95460066 0.229779682 1.978247442
7326 AL121820.1 7.705098628 0.228490586 1.021441026 0.218477229 0.750122889 0.754942819
7327 AL122227.1 10.69128225 0.518474227 0.87748827 0.590861822 0.554612002 0.504445211
7328 AL122242.2 5.221252211 1.285117016 1.228808989 1.024588972 0.200860906 1.142824141
7329 AL126988.1 15.55715142 -0.654217864 0.729291502 -0.897059491 0.269687172 1.491497211
7330 AL157925.2 7.747428582 0.901226748 1.082254277 0.822740297 0.404991212 0.994171
7331 AL162724.1 6.008115767 1.620697684 1.216228917 1.240770848 0.179994864 0.504445211
7332 AL256599.1 11.27219557 -0.201297591 0.828521226 -0.24005974 0.810282944 1.989458988
7333 AL290208.1 8.980172812 1.04218286 0.972262527 1.071841099 0.28279141 1.717849154
7334 AL290728.6 16.98402865 -0.224258527 0.724176422 -0.447761792 0.654225121 0.754942819
7335 AL442662.2 12.15189427 0.104207556 0.818652494 0.12741256 0.898612084 0.754942819
7336 AL582722.2 15.28122504 -0.417898082 0.725216742 -0.568401205 0.569762572 1.140998814
7337 ALG10B 7.727681278 0.707256592 1.029422205 0.68052145 0.496174245 0.984879475
7338 AMOTL1 11.12865908 0.114778158 0.846919226 0.125524225 0.892197222 1.004414979
7339 ANKDD1A 6.68902799 0.162949259 1.141512861 0.14262454 0.885796962 0.745471492
7340 ANLN 7.195645625 0.151520542 1.080690948 0.140216252 0.888489052 0.504445211
7341 AP002068.1 19.55272608 -0.428021112 0.689752226 -0.625054814 0.525292666 1.494221447
7342 AP1S2 7.947091095 -0.124215787 1.045620516 -0.128455578 0.897788451 1.744821174
7343 APBB1 11.62826477 0.112550892 0.821251648 0.125282015

						0.892209052	1.471750017
7344	APBB2	22.14272519	-0.728668526	0.624224282	-1.164661922	0.244155864	1.004414979
7345	APLF	12.1027926	-1.276224621	0.902794418	-1.412748918	0.157425627	0.504445211
7346	APOL2	14.54108078	-0.806162858	0.751552672	-1.072662152	0.282422278	1.480105994
7347	ASPM	10.16198292	-0.599756615	0.926910716	-0.640142762	0.52207979	1.155115484
7348	B2GLCT	9.877710567	-0.225540815	0.910299772	-0.258722401	0.795849424	1.491497211
7349	BASP1	10.65745942	0.526299689	0.876597999	0.600502955	0.548171092	1.482041241
7350	BOP1	14.2667862	-0.256072115	0.76565005	-0.224451901	0.72802858	0.504445211
7351	BTBD1	11.62259295	-0.908879609	0.879570122	-1.0222225	0.201452001	0.504445211
7352	C20orf96	14.17846508	0.711822565	0.799124577	0.890766702	0.272054242	0.754118471
7353	C21orf58	6.981906512	1.640960242	1.120124814	1.452004064	0.146500475	0.504445211
7354	C22orf24	12.27125425	-0.066741702	0.80461652	-0.082948461	0.922892521	0.504445211
7355	C2orf18	12.25429222	-0.416859599	0.807586162	-0.516179719	0.605728894	0.504445211
7356	C6orf120	12.5956425	0.102890221	0.780275557	0.122128492	0.894091762	0.754942819
7357	CALCRL	8.89829919	-0.268645495	0.967949212	-0.277540871	0.781264827	1.157940821
7358	CARNMT1	10.17227428	0.40260877	0.886528874	0.454140617	0.649727621	1.715082871
7359	CBY1	11.22419492	-0.852008849	0.87228976	-0.976664586	0.228725222	0.504445211
7360	CCDC144NL-AS1	11.65268264	-0.264585226	0.848069891	-0.211985166	0.755051797	1.750451804
7361	CCDC85B	7.426296829	0.055775922	1.068972606	0.052177129	0.958287549	1.482041241
7362	CD109	8.45270874	-0.12712525	1.022521022	-0.124212717	0.901146062	0.745471492
7363	CD92	5.487271449	1.154085555	1.256281872	0.918578642	0.258216007	1.149454791
7364	CDAN1	9.618756082	-0.215776549	0.924876261	-0.227772621	0.725522782	1.001791421
7365	CEP85L	9.410587529	0.051116698	0.922827402	0.055291296	0.95582665	1.717849154
7366	CFH	11.89851046	-0.442681514	0.850064692	-0.520762147	0.602522476	1.482041241
7367	CGAS	12.07479906	-0.790666251	0.796122612	-0.992122607	0.220645225	1.004414979
7368	CHCHD4	12.29288684	-0.187220568	0.817566814	-0.229121815	0.818766467	0.754942819

7369	CHCHD6	12.62597942	0.218050824	0.801486264	0.272058092	0.785577252	0.994171
7370	CHRM1	15.58824011	-0.182928222	0.722047141	-0.251251897	0.801619262	0.504445211
7371	CSRNP1	15.59102529	-0.271224254	0.726922487	-0.502749291	0.614427496	0.504445211
7372	CUTALP	12.62220276	0.202899944	0.778602547	0.261879269	0.792414446	0.754118471
7373	CYSLTR2	7.722251972	1.11669251	1.028214851	1.075589025	0.282111092	1.505412992
7374	DCAF17	14.0245122	-0.510226982	0.768727802	-0.662859146	0.506780466	0.745471492
7375	DENND11	12.85522918	-0.62201867	0.804125159	-0.785960746	0.421890484	0.504445211
7376	DLG1	12.1726257	0.42522271	0.811962607	0.522821796	0.600402492	0.984879475
7377	DNAH11	7.972559152	0.980927702	1.01298012	0.967402279	0.222242472	0.504445211
7378	DNAJC24	10.9248699	0.578819046	0.862259298	0.671281806	0.502041022	0.984879475
7379	DPY19L1	15.08420762	-0.992264909	0.775957225	-1.280179804	0.200481907	0.745471492
7380	DUS1L	16.81814999	-0.122614076	0.711269475	-0.17276916	0.862046882	1.144449507
7381	EFCAB11	12.29714425	-0.486470771	0.789586299	-0.616108246	0.527822014	1.991494225
7382	EGLN2	9.148797697	-0.181657248	0.9465164	-0.191922029	0.847802282	1.991494225
7383	EGR2	11.14149442	0.242991205	0.849872024	0.287091819	0.774042016	0.504445211
7384	EHBP1	10.61507942	-0.282720722	0.875272222	-0.224162421	0.745815062	1.981102009
7385	EOLA1	16.50278894	-0.226964049	0.722518976	-0.466272978	0.640947825	1.747444511
7386	EPX	12.24872154	-0.658119162	0.81704966	-0.805482452	0.420541229	1.494221447
7387	ESCO2	8.19196897	0.126094122	0.999626219	0.126145012	0.891706645	0.504445211
7388	ESRP2	9.222507667	1.284945417	0.966889142	1.228948027	0.182865117	1.142824141
7389	FAM192B	9.226292952	0.922905694	0.982278425	0.929522029	0.247462787	0.754942819
7390	FAM78A	12.87150994	0.150192226	0.770826296	0.19484466	0.845514559	0.504445211
7391	FBXL17	6.714445987	0.820028481	1.112294292	0.727174221	0.461016402	0.504445211
7392	FBXL18	10.92765518	0.208120246	0.862426165	0.256852522	0.721201458	0.994171
7393	FBXO45	9.902078502	-0.240774462	0.906772922	-0.265528947	0.790602012	1.114084857
7394	FBXW8	12.59559244	-0.214506208	0.770191662	-0.278510166		

0.780620762 1.140998814
7395 FMO4 14.02028652 -0.722742942 0.771921469 -0.92758752
0.248456422 1.004414979
7396 FNBP1L 10.92481982 0.175806794 0.865082982 0.202225117
0.828959082 0.504445211
7397 GEMIN7-AS1 11.85906591 -0.212285268 0.822242524 -0.279872442
0.704040104 1.157940821
7398 GFI1B 9.95107915 0.479790522 0.911662575 0.526280228
0.598692512 0.745471492
7399 GGACT 8.892678558 -0.104512182 0.967558222 -0.108016427
0.912982661 0.504445211
7400 GSTZ1 21.9091987 -0.575540705 0.642955918 -0.895148001
0.270707977 1.004414979
7401 HECA 11.6085674 -0.282482562 0.822045622 -0.459127587
0.646125267 1.501788444
7402 HIVEP2 14.61728482 0.292562128 0.764516221 0.51247776
0.607617164 0.504445211
7403 HOXB6 18.21106212 -0.582045481 0.686829262 -0.847428286
0.296750816 1.984812441
7404 HYLS1 9.706251227 0.722241279 0.919915761 0.786212627
0.421684257 0.745471492
7405 IER5 14.04008277 -0.949988228 0.771616958 -1.221165694
0.218260899 1.505412992
7406 IFITM10 11.24110688 -0.855696506 0.870406682 -0.982099652
0.225558296 1.125278181
7407 IFT57 16.97269727 -0.672210742 0.705806291 -0.952959679
0.240104052 0.745471492
7408 IGSF10 6.947982475 0.259898067 1.099862205 0.226200571
0.812199444 1.144449507
7409 IL9RP2 5.490206796 1.471884626 1.270085892 1.158885895
0.246502699 1.004414979
7410 KAT2A 8.942464491 0.269502048 0.948252229 0.289667471
0.696782452 0.745471492
7411 KAT8 11.67069472 0.479282605 0.82759486 0.572212926
0.567177722 0.992225454
7412 KCTD21-AS1 7.966928522 1.189985112 1.024268282 1.161790242
0.245220628 1.474585244
7413 KLHL5 9.1222797 -0.661191942 0.990719927 -0.667285221 0.50452604
1.501788444
7414 KREMEN1 18.19126576 -0.919429412 0.68119292 -1.249724247
0.177101242 1.001791421
7415 LAGE2 14.21600027 -0.557212966 0.752898858 -0.740224215
0.459162957 1.007441214
7416 LINC00294 12.28296761 -1.069647681 0.805786228 -1.227458281
0.184257099 0.504445211
7417 LINC00888 15.55725154 -0.086951242 0.724929044 -0.118212421
0.905820108 0.994171
7418 LINC00900 14.08519901 -0.842664782 0.779475246 -1.081066626
0.279667478 1.989458988
7419 LIPT1 8.422720172 0.052027989 0.982499124 0.052911068
0.957802765 0.504445211

7420	LMF1	15.02526596	-0.085672942	0.777256578	-0.110226075	0.912220082	0.745471492
7421	LNX1	21.26216045	-0.02081999	0.66808156	-0.021162845	0.975128872	1.151190129
7422	LRG1	20.72722952	-0.087420228	0.674789982	-0.129566725	0.896909221	0.504445211
7423	LRRC27	12.60977017	0.214241879	0.786788221	0.299525296	0.689506115	1.001791421
7424	LRRC27A4P	8.442417415	-0.296268596	1.026644689	-0.28867689	0.772828649	0.504445211
7425	LTB4R	17.07421907	-0.421058802	0.714290428	-0.589295919	0.555595714	0.992225454
7426	LY75	14.2821262	-0.875225111	0.761246159	-1.149729928	0.250251	1.004414979
7427	MAP2K14	12.1209058	0.229199668	0.822754272	0.278576055	0.780570191	1.724215124
7428	MAPKAPK5	12.78679997	-0.796067752	0.774229942	-1.028192565	0.202859255	0.754942819
7429	MEPCE	10.10562642	-0.441727267	0.912062686	-0.482796884	0.628520027	0.984879475
7430	MINK1	16.07222517	-0.085752828	0.722828476	-0.118622458	0.905565757	1.724275194
7431	MMP24OS	9.655415242	-0.172817096	0.916225866	-0.188618442	0.850291877	0.754942819
7432	MS4A1	6.455491502	0.726696962	1.15282241	0.629021586	0.522802284	1.001791421
7433	MT1H	5.265076225	1.262298929	1.227742875	1.019102861	0.208152661	0.504445211
7434	MTFR2	12.79242061	-0.910969664	0.777206601	-1.17195668	0.241214449	1.949911484
7435	MTX1	15.08167252	0.097211026	0.747642099	0.120157071	0.896442158	1.984028254
7436	MYOSLID	7.477222822	1.2727145	1.057960441	1.202922957	0.228615106	1.125278181
7437	NAMPTP1	8.465050127	0.559250144	0.986427462	0.567046402	0.570682655	0.984879475
7438	NBPF11	6.469568112	0.497891052	1.141691691	0.426099292	0.662764618	0.504445211
7439	NCF1B	17.24289202	-0.798998476	0.692895268	-1.152120224	0.248856928	1.121541825
7440	NDUFA11	11.42875222	1.247045921	0.897809241	1.28898762	0.164826515	0.504445211
7441	NEK7	11.8449892	-0.187286988	0.829475272	-0.225910277	0.821271205	0.504445211
7442	NEMP2	11.89861058	0.289219626	0.822759876	0.251218412	0.725424497	0.754942819
7443	NINJ2	11.64249144	0.259020522	0.825629652	0.429625574	0.667460755	0.504445211
7444	NNT-AS1	12.82762597	-0.156852225	0.765229705	-0.204948708	0.827612199	0.994171
7445	NOCT	7.702212242	0.716056285	1.028222184	0.696227798		

0.486222568	1.121541825
7446	NR0B1	12.14050292	-0.742221801	0.864256259	-0.858914001
0.290287964	0.504445211
7447	NR4A1	9.127556422	0.126562411	0.965287222	0.141459709
0.887506787	1.157940821
7448	PABPC4-AS1	9.427549548	0.510881225	0.922045992	0.552472214
0.579929249	0.984879475
7449	PGP	12.26572262	0.050758102	0.80762672	0.062848468	0.94988717
0.994171
7450	PLAG1	6.420072504	0.060918511	1.199210804	0.050798824	0.95948582
1.004414979
7451	PMS2P4	11.92242449	0.282545694	0.847222678	0.222496278
0.728759622	0.504445211
7452	POLE2	15.2489228	-0.720641207	0.765080586	-0.941915558
0.246225874	0.504445211
7453	PPIAP22	11.40606954	0.17259079	0.829217712	0.205656752
0.827059042	0.504445211
7454	PPP1R8	12.55604877	-0.757061799	0.778027022	-0.972052227
0.220526794	1.007441214
7455	PRKAB2	10.1140824	-0.442292422	0.90718257	-0.488758765
0.625012494	0.745471492
7456	PRR4	8.479126727	0.280020027	1.021282201	0.268466247
0.712525524	0.504445211
7457	PTDSS2	7.429182124	-0.224948692	1.108224141	-0.202211854
0.762490568	1.144449507
7458	PTPN4	10.44924082	0.457726628	0.895426928	0.511188029	0.6092194
0.504445211
7459	PUS10	12.12926166	-0.491015254	0.829214274	-0.592145202	0.55275226
1.494221447
7460	PXYLP1	7.471612201	1.516448608	1.092728006	1.286482504
0.165599619	1.001791421
7461	RAB20-DT	17.47657969	-0.220402282	0.727278066	-0.216758625
0.751426754	0.754942819
7462	RAP2C-AS1	5.965785812	0.794957252	1.202279227	0.660659001
0.508821022	0.504445211
7463	RIDA	7.722060647	0.910829449	1.028842416	0.88520425
0.275992558	0.754118471
7464	RING1	9.644174079	0.128706855	0.919001261	0.140050792
0.888619859	1.989458988
7465	RIOX2	17.01776244	-0.860249877	0.701180021	-1.227002829
0.219821524	1.711148514
7466	RN7SL648P	11.62965609	0.224604192	0.821466746	0.282157026
0.7778221	1.984872702
7467	RPL10P16	11.15840628	0.240508592	0.847250068	0.282826162
0.77652594	1.485874489
7468	RPL22AP7	11.17258212	0.91000488	0.872982867	1.041215926
0.297775246	0.504445211
7469	RPS2P5	12.6246881	0.105644195	0.799474881	0.122141982	0.89487199
0.754118471
7470	RPS5P2	12.61056142	-0.120489189	0.797289524	-0.151122507
0.879878289	0.992225454

7471	RRN2P1	9.92700254	0.622697022	0.90112757	0.702218969	0.4819192	0.994171
7472	RRP1	26.42766882	-0.761554276	0.625629929	-1.217260141	0.222505254	1.142824141
7473	S1PR4	9.620097469	0.282290264	0.957851071	0.294816566	0.768124011	1.729140481
7474	SCAMP1-AS1	12.80942269	-0.257462289	0.772975121	-0.222081081	0.72907208	1.717849154
7475	SDCBP2-AS1	12.08702182	-0.122020221	0.8285129	-0.147288257	0.882904501	0.504445211
7476	SENP1	9.404966897	0.205910257	0.927450825	0.222017547	0.824200222	0.754118471
7477	SERP2	7.920229199	0.418214792	1.019152286	0.410452222	0.681472511	0.504445211
7478	SIRT1	8.220222214	0.854074449	0.997777862	0.855976547	0.292010752	1.744781112
7479	SLC15A2	9.711871968	0.565828467	0.921887926	0.607185097	0.542728082	0.754942819
7480	SLC27A5	16.29172498	-0.482841581	0.711215215	-0.678801144	0.497262869	1.984812441
7481	SLC46A1	10.88242982	-0.620271426	0.882201908	-0.702094622	0.481996777	1.494221447
7482	SLC4A5	12.55126946	-0.821069051	0.818716787	-1.002872111	0.215922088	0.754942819
7483	SNTB1	8.687245252	0.46605686	0.960415216	0.48526596	0.627487699	0.994171
7484	SPAG1	9.420224822	0.199778556	0.922289911	0.216611451	0.828511161	1.480105994
7485	SPOPL	12.88921228	0.28298272	0.799747264	0.478879688	0.622024222	1.719412177
7486	SRCAP	9.945458519	0.621511264	0.902572447	0.698904296	0.484611842	1.494221447
7487	STEAP2	16.21558111	-0.752889746	0.727922484	-1.024282712	0.201002549	1.471750017
7488	STIL	9.196748282	0.121922902	0.952292257	0.128021872	0.89812275	0.504445211
7489	STK17A	14.04018289	-0.201524597	0.776154712	-0.288498056	0.697647492	1.155115484
7490	SUV29H2	12.07852579	-0.482927222	0.822621856	-0.587070741	0.557156197	1.719412177
7491	SVBP	11.41174024	0.426819254	0.84202666	0.506895178	0.612228269	0.984879475
7492	TBC1D8	18.4896648	-0.952842524	0.677277684	-1.408129884	0.159089652	0.754118471
7493	TECPR2	12.21122096	-0.600828642	0.781792527	-0.768528779	0.442167161	0.504445211
7494	TENT4B	8.922717187	0.211696956	0.947629752	0.222296274	0.822227104	1.497117951
7495	TIGD2	8.667547987	-0.197574018	0.979210826	-0.201748017	0.840112722	0.754118471
7496	TIPIN	11.60578212	-0.121414898	0.825092116	-0.157265562		

0.874956752 1.711148514
7497 TMCO4 15.70891241 -1.082195975 0.741275592 -1.461062262
0.142998228 0.754942819
7498 TMEM184B 12.78685004 -0.46652602 0.778206702 -0.599411554
0.548898474 0.754942819
7499 TMEM62B 7.710769221 0.71216977 1.024978479 0.695789994
0.486560256 0.754942819
7500 TNFSF14 6.449820808 0.278192477 1.158677266 0.240094857
0.810256721 1.149454791
7501 TTC21B 8.661927255 -0.029440724 0.97141009 -0.020207204
0.975822051 0.992225454
7502 UAP1 16.77860522 -0.554962626 0.702098922 -0.790422669
0.429274546 0.504445211
7503 UBL7-AS1 11.2862722 0.422259586 0.85585851 0.505059624
0.612516952 1.155115484
7504 UBXN6 16.22254212 -0.475741407 0.7229249 -0.648206546
0.516851268 0.504445211
7505 UVSSA 16.80407228 -0.026005628 0.714244411 -0.050402728
0.959800658 1.741995818
7506 VANGL1 7.9752945 1.186768265 1.019957706 1.162546545
0.244607825 0.754942819
7507 VCAN 12.07762441 -0.67222422 0.790581025 -0.851682875
0.294290128 1.981102009
7508 VWA7 7.184404272 0.562625585 1.098286884 0.512275612
0.608458121 1.711148514
7509 WAC-AS1 12.58514242 -0.4628145 0.800842407 -0.579157542
0.562482876 1.949911484
7510 WDR24 15.02267188 -0.276615427 0.742877972 -0.506968104
0.612177199 0.504445211
7511 WDR5 29.82909957 -0.262925506 0.617921572 -0.425509098
0.670465591 1.114084857
7512 YPEL2 10.19590696 0.252909922 0.907772198 0.279706245 0.77970281
0.745471492
7513 YPEL2 5.242492574 0.749542695 1.215688024 0.569696269
0.568882652 1.758957844
7514 ZBTB45 9.192962998 0.429602194 0.92512227 0.470102269
0.628281882 1.975521214
7515 ZBTB6 4.982529089 0.629457691 1.278662499 0.462824679 0.64277226
1.007441214
7516 ZEB2-AS1 17.01219187 -0.500754228 0.699221452 -0.716159846
0.472892654 1.989458988
7517 ZNF169 8.191918908 -0.295402099 1.058284872 -0.272625291
0.708682041 1.508459229
7518 ZNF41 10.86841227 -0.07764406 0.862282409 -0.089920092
0.928242768 0.984879475
7519 ZNF422 12.22096826 -0.491264928 0.778087624 -0.621502222
0.527711471 0.745471492
7520 ZNF426 16.22821282 -0.29562622 0.747440227 -0.295518227
0.692460291 0.745471492
7521 ZNF444 12.64995592 -0.592251509 0.852061815 -0.695080449
0.487004887 0.504445211

7522	ZNF492	9.880495852	-0.522680568	0.922976228	-0.578217018	0.56211761	0.745471492
7523	ZNF529	19.46907617	-0.89887977	0.660569144	-1.260765602	0.172587775	1.984028254
7524	ZNF569	9.420224822	0.199778556	0.922289911	0.216611451	0.828511161	1.724275194
7525	ZNF571	11.88721925	0.169611644	0.822014469	0.206226567	0.826528017	0.504445211
7526	ZNF595	11.90985184	0.044025012	0.821970626	0.052928567	0.957788822	1.958410259
7527	ZNF614	8.704257209	0.461298515	0.965745122	0.477660719	0.622891707	1.007441214
7528	ZNF675	15.05052277	-0.676425298	0.727204152	-0.917568621	0.258844752	0.504445211
7529	ZNF710-AS1	9.948292865	0.784224159	0.906055279	0.865658175	0.286677642	1.004414979
7530	ZNF747	8.690180699	0.626982676	0.962729168	0.660957142	0.508629799	0.504445211
7531	ZNF764	11.14149442	0.242991205	0.849872024	0.287091819	0.774042016	0.994171
7532	ZNF768	12.09459642	-0.224264512	0.782226279	-0.426846652	0.669491022	1.004414979
7533	ZNF799	9.192912926	-0.024502454	0.960772607	-0.025912196	0.971252271	1.711148514
7534	ZNF81	8.875716529	-0.596005294	1.004549192	-0.592206229	0.552976164	0.504445211
7535	ZNF829P1	17.20222505	-0.024277726	0.707071012	-0.048478492	0.961224905	0.504445211
7536	ZNF891	18.7487194	-0.411115684	0.676460442	-0.607745242	0.542256285	1.114084857
7537	ZNHIT2	12.65572672	0.22924958	0.807501612	0.407862442	0.682274668	1.125278181
7538	ZRANB2	17.00085049	-0.858576222	0.698054004	-1.229956891	0.218712248	0.754118471
7539	AC005070.2	8.946248701	0.876552851	0.959721792	0.912241612	0.261062902	1.494221447
7540	AC005696.2	10.84856477	-0.229758462	0.882208025	-0.285122827	0.700146409	1.508459229
7541	AC008957.1	14.6482722	0.189222067	0.766564109	0.246987909	0.804917581	0.745471492
7542	AC009061.2	9.162872222	0.449227968	0.95599772	0.469904848	0.628422001	1.155115484
7543	AC009951.4	10.18167909	-0.167792785	0.920290772	-0.182207112	0.855241717	0.504445211
7544	AC010976.1	14.28086174	0.144907404	0.758402902	0.191068907	0.848471608	1.711148514
7545	AC011611.2	20.74972085	-0.440041711	0.648977066	-0.678054221	0.497727245	1.482041241
7546	AC012184.1	12.64911252	0.095969122	0.782198842	0.122524824	0.902475468	1.497148014
7547	AC022796.2	9.615819527	-0.622112429	0.974856248	-0.628158064		

0.522270792 1.722529849
7548 AC072220.2 6.697422822 0.601210269 1.154205577 0.520841605
0.602477117 1.151190129
7549 AC090181.2 7.460169616 0.427251221 1.059929975 0.412622844
0.679882962 0.504445211
7550 AC090198.1 12.19412212 0.225519464 0.851022402 0.294249929
0.692296527 1.007441214
7551 AC092129.2 12.1086219 -0.224464242 0.782250562 -0.286946722
0.774152106 1.125278181
7552 AC092919.2 15.22477721 -1.015227706 0.7562426 -1.242417717
0.179460592 1.949911484
7553 AC108692.2 9.880494778 0.202412581 0.925426725 0.217451994
0.827856115 1.155115484
7554 AC114546.2 10.90502129 0.214074227 0.856808564 0.266562022
0.712944994 0.754118471
7555 AC116248.4 8.220121116 0.667911782 1.002102482 0.665845242
0.505509959 0.754118471
7556 AC127164.1 8.470569561 0.211704929 1.026190768 0.206201728
0.826555229 1.001791421
7557 AC121212.2 5.988267265 1.052294822 1.205662627 0.872876707
0.282720284 0.504445211
7558 AC221981.1 12.11991222 -0.11586222 0.78252222 -0.148061868
0.882292942 0.754118471
7559 ACADS 16.10882217 -0.26065059 0.740169582 -0.487254001
0.626078241 1.144449507
7560 ACTA2 10.91914807 0.581241711 0.860749072 0.67527428
0.499501521 0.754942819
7561 AF129075.2 10.944516 0.575250552 0.867076148 0.662552508
0.506976762 1.125278181
7562 AGAP1 15.52725298 -0.554061825 0.722022162 -0.766212764 0.44248962
1.151190129
7563 AKAP17A 8.416998242 0.054991214 1.000622978 0.054956528
0.956172097 0.745471492
7564 AL127186.1 12.220817 -0.952414626 0.811188215 -1.174098204
0.240255692 1.125278181
7565 AL254920.1 12.58141562 -0.211508215 0.770987427 -0.274224195
0.782827808 1.125278181
7566 AL290719.1 9.917154028 0.241285246 0.899592127 0.279277872
0.704407281 1.720704501
7567 AL589765.7 19.78442615 -0.218250299 0.666199602 -0.47771012
0.622856522 0.994171
7568 AMMECR1 12.16875519 0.240712249 0.819845749 0.41558104
0.677716602 0.994171
7569 AMT 11.67242887 0.479729809 0.822625277 0.57548609
0.564962595 0.994171
7570 ANKAR 12.61901624 0.452279671 0.822700049 0.55041841
0.582022424 0.754942819
7571 ANKRD46 16.29725442 -0.667115851 0.71207992 -0.926855292
0.248822999 1.140998814
7572 ARAP2 9.416106962 -0.256050222 0.950256288 -0.269425592
0.787602195 1.720704501

7573	ATF5	15.06729459	-0.478164569	0.722820144	-0.651610024	0.514652772	1.741995818
7574	ATRIP	5.475978926	0.587190861	1.22059102	0.441200781	0.658995261	1.722529849
7575	BACE2-IT1	6.255677729	1.120922727	1.200822448	0.941799288	0.24629541	0.504445211
7576	BBS12	9.69769416	0.57086224	0.912026808	0.625242582	0.521811858	0.754118471
7577	BEND2P2	8.225741748	0.482616572	1.022891299	0.472221902	0.626689902	1.142824141
7578	BOLA2	16.8209242	0.12292926	0.712212428	0.188060827	0.850828969	1.007441214
7579	BPHL	14.57778902	0.101485751	0.758118121	0.122865254	0.892509047	0.504445211
7580	C12orf66	12.1527872	0.208172242	0.791826916	0.262901195	0.792626725	1.508459229
7581	C14orf92	9.921220649	0.191548771	0.90229284	0.212022642	0.822081568	0.504445211
7582	C1orf52	10.17610852	0.402127709	0.886582225	0.454698615	0.649226078	1.971747021
7583	C2CD2L	10.14785518	-0.160782692	0.897125529	-0.179220952	0.857764212	1.499952199
7584	CAMKMT	6.196425818	0.410267071	1.226820551	0.221711072	0.74010744	1.004414979
7585	CCDC15	6.970462928	0.464280865	1.110670269	0.418018621	0.675922495	0.504445211
7586	CCDC157	14.57772897	-0.19524469	0.744220284	-0.262447087	0.792976774	1.729140481
7587	CCNE2	14.22291115	-0.04862166	0.752652251	-0.064527975	0.948549822	1.114084857
7588	CCR5	9.422068981	0.200262567	0.927169061	0.216101427	0.828908679	1.720704501
7589	CDA	17.72692694	-0.515920248	0.69028679	-0.747400001	0.454822145	0.504445211
7590	CDKN1B	8.927792722	0.879228559	0.97225074	0.904222124	0.265818782	1.501788444
7591	CENPV	8.664711565	0.474022266	0.996211455	0.475788222	0.624225182	0.992225454
7592	CEP97	15.06744466	-0.186911401	0.724517688	-0.254468209	0.799122861	0.754942819
7593	CFTR	8.96027525	0.202547275	0.980972272	0.207495222	0.825622028	1.949911484
7594	CICP14	12.56161827	-0.64401692	0.777791211	-0.828007454	0.407666279	0.504445211
7595	CISD2	17.07128265	-0.078977204	0.701724767	-0.112547266	0.910289495	1.494221447
7596	CLEC2B	18.20124247	-0.267898228	0.682662217	-0.292421602	0.694729229	1.474585244
7597	CLK2	12.12279862	0.224257208	0.790718041	0.410079426	0.681747677	0.754118471
7598	COA6-AS1	10.62621961	0.122120877	0.882829701	0.129202847		

						0.88921005	0.754942819
7599	COA8	15.84420811	-0.044028129	0.72245998	-0.060857729	0.951472507	1.991494225
7600	CPT2	22.76122257	-0.077226075	0.645125425	-0.119720716	0.90470429	1.485874489
7601	CYB561	12.85156122	-0.692101847	0.785625202	-0.880956778	0.278241214	1.497117951
7602	CYBRD1	9.449980928	0.196212852	0.942889281	0.208098507	0.825152052	1.007441214
7603	DGAT1	12.62498685	-0.112274564	0.779807918	-0.145287808	0.88440469	0.754942819
7604	DHODH	14.58609482	-0.810920162	0.770018825	-1.052117209	0.292287221	0.984879475
7605	DHRS4	12.22175844	-0.412212276	0.815250922	-0.505749045	0.612022822	0.504445211
7606	DISC1	14.02152659	-1.064044142	0.787880048	-1.250515406	0.176850715	1.004414979
7607	DNTTIP1	12.11140719	-0.448189757	0.791440021	-0.566296555	0.571192202	0.504445211
7608	DRC2	9.725897442	0.719051746	0.94525802	0.760612146	0.446888165	1.114084857
7609	DZIP2	10.46610164	0.742622916	0.902994541	0.82241241	0.410842219	1.750451804
7610	ECE2	16.20576046	-0.292186961	0.708279657	-0.555129541	0.578806058	1.144449507
7611	EEF1E1	9.464157672	0.991148982	0.922211918	1.062108777	0.287722625	1.971747021
7612	EFHC1	10.25222845	-0.217260847	0.912251685	-0.22787206	0.811980226	1.741995818
7613	EIF2AK2	12.82549068	-0.868920421	0.820658068	-1.058821517	0.289681072	1.125278181
7614	EIF2C	12.85812229	-0.285624969	0.79054652	-0.261200647	0.71787471	1.001791421
7615	EIF2FP2	15.22077851	-0.042692024	0.722972268	-0.058246264	0.95255229	0.745471492
7616	EIF5A2	12.86295277	0.048284246	0.762282299	0.0622814	0.949462798	0.992225454
7617	ERCC6	9.44152496	0.198204166	0.922022224	0.212529266	0.82168627	1.485874489
7618	FAM122A	12.11229857	-0.245025457	0.814297619	-0.200867108	0.762515822	1.007441214
7619	FAM122B	9.188191106	-0.021842691	0.959516254	-0.022187225	0.972525278	1.491497211
7620	FAM12A	9.450021	0.6650196	0.924488696	0.71922772	0.471922854	0.504445211
7621	FAM50B	18.54900576	0.164472652	0.692741956	0.227422682	0.812228895	1.151190129
7622	FAXDC2	14.1444899	0.298792492	0.767970628	0.289067602	0.697226128	0.984879475
7623	FOLR2	10.68844692	1.102526225	0.895220117	1.222698209	0.21768828	0.504445211

7624 FOXO1 8.895462769 0.284191448 1.012652504 0.279291199
0.704297285 0.504445211
7625 FUT10 14.7942625 -0.422672646 0.742019701 -0.584448965
0.558918292 1.494221447
7626 FXR2 12.59628262 -0.117200288 0.801655022 -0.146198022
0.882765059 1.741995818
7627 GALNT10 21.22275585 -0.567720891 0.641279821 -0.885171115
0.276064249 0.745471492
7628 GCLM 12.15746287 0.22225456 0.815252569 0.272812524 0.78422792
0.754942819
7629 GLRX5 12.24220845 -0.16217792 0.77624845 -0.210212524
0.822501017 1.491497211
7630 GNAL 15.50919976 -0.259919904 0.744075822 -0.482714008 0.62858885
1.944191051
7631 GPR180 15.05892862 -0.477085918 0.722952588 -0.650909657
0.515104807 0.754942819
7632 GPR22 12.27120211 0.167225625 0.808226748 0.206999911
0.826009928 1.144449507
7633 HACD2 8.426745647 0.221128064 0.987477662 0.222922219
0.822810045 1.151190129
7634 HGH1 8.189082487 0.215474581 1.002749445 0.214609579
0.752058105 1.004414979
7635 HHEX 21.21967924 -0.496111564 0.629651451 -0.775596715 0.42798714
1.741995818
7636 HOMER2 17.80591615 -0.19486622 0.688905604 -0.282862456
0.777281509 0.504445211
7637 IFTAP 14.12190725 0.097500929 0.759627258 0.128251954
0.897870452 0.754118471
7638 IGF1R 12.15174211 -0.272019129 0.842592007 -0.441517527
0.658828276 0.754118471
7639 IKZF4 8.470619622 0.728002429 0.987128182 0.747618157
0.454690509 1.720704501
7640 ING5 9.641227524 -0.168518621 0.928626822 -0.181470766
0.855998077 1.142824141
7641 IQCC 7.424801681 0.445846151 1.078907202 0.412228607
0.679421805 0.504445211
7642 IRAK2 12.89966209 0.461242755 0.774822627 0.595287416
0.551651266 1.144449507
7643 IRF1-AS1 12.1885025 0.457224249 0.825571691 0.547221072
0.584151201 1.720704501
7644 ITPK1-AS1 6.255677729 1.120922727 1.200822448 0.941799288
0.24629541 0.754942819
7645 KIF24 14.11628662 0.20072292 0.760740965 0.262851876
0.791894059 1.121541825
7646 KIZ 16.69289428 -0.912021264 0.721295428 -1.265646174
0.205629787 1.491497211
7647 KLHL25 7.907545251 -0.122025447 1.068710274 -0.11418009
0.909095024 1.70150111
7648 KMT2B 16.29720448 -0.292166981 0.707457798 -0.554222685
0.579251185 0.504445211
7649 KMT2E-AS1 15.52720292 -0.84990122 0.721255479 -1.162249507

						0.24512412	1.978247442
7650	LINC00674	14.79147809	-0.842646692	0.749481204	-1.125640895	0.260217551	1.941455704
7651	LINC00987	9.677896795	-0.027022667	0.924947824	-0.029227224	0.976682261	0.745471492
7652	LPO	6.724142154	1.571665125	1.151526464	1.264852682	0.172299042	1.941455704
7653	LRRC7	18.18554294	-0.918188748	0.682105202	-1.246110168	0.17826699	0.994171
7654	MACO1	14.26111444	0.047562782	0.752582512	0.062200752	0.949606645	1.149454791
7655	MAP2K15	15.84420811	-0.044028129	0.72245998	-0.060857729	0.951472507	0.504445211
7656	MARCHF1	10.28989771	-0.085418501	0.882849208	-0.096752206	0.922922286	1.717849154
7657	METTL15	11.28060041	0.051901297	0.829295841	0.061821729	0.950696825	1.747444511
7658	METTL2B	12.2740884	-0.06626188	0.802476986	-0.082468921	0.924272821	0.504445211
7659	MFSD2B	11.95780125	0.909509626	0.851220282	1.068251892	0.285261809	0.754942819
7660	MIR181A1HG	12.17726122	0.706060152	0.82401212	0.856855472	0.291524764	12.87047008
7661	MOCS2	17.06297672	0.175599959	0.712958797	0.246297485	0.805451957	10.44411414
7662	MORC2-AS1	17.57526507	0.224602289	0.711627891	0.470194174	0.628216296	11.74591048
7663	MTG1	8.960225211	0.699299254	0.952191128	0.72274502	0.462104148	8.092791714
7664	MYB-AS1	9.210822818	0.762266725	0.940817215	0.811280569	0.417204566	7.854584544
7665	MZT2B	14.56256222	-0.701922196	0.751919975	-0.922521145	0.250550958	8.575041424
7666	NADK2	12.59822759	-0.214050511	0.767995408	-0.278712269	0.780464878	10.05812511
7667	NBPF12	12.77545751	-0.681875268	0.782902289	-0.86984629	0.28428441	1.011190501
7668	NCF4-AS1	16.55919442	0.268401092	0.724406196	0.265466812	0.714762976	1.011190501
7669	NECTIN2	12.25150575	-0.296824488	0.807520652	-0.267582926	0.712184241	8.819919211
7670	NFKBIB	11.64801087	0.109817858	0.828225428	0.122594162	0.89451425	14.78451792
7671	NMNAT1	12.22175844	-0.412212276	0.815250922	-0.505749045	0.612022822	4.28551754
7672	NRAV	8.186197078	-0.292476078	1.079986621	-0.262408274	0.716299922	4.248405582
7673	NSUN5P1	11.26920909	-0.072645956	0.842771622	-0.086198862	0.921208225	7.842242181
7674	OAF	10.61492816	-0.849422789	0.922518154	-0.909927445	0.262860775	5.417297487

7675	PARPBP	11.92228226	0.520225441	0.822221792	0.627120251	0.524046452	2.740009257
7676	PBK	8.211715199	1.257628648	1.02555965	1.226285227	0.220091259	7.244918917
7677	PEX12	9.911482245	0.050282622	0.902279874	0.055840249	0.955468992	7.229540981
7678	PFDN6	8.476240254	0.557221648	1.006007129	0.552992721	0.579582126	5.117454425
7679	PLCXD1	15.20224462	-0.9067727	0.775805044	-1.168815165	0.242478109	2.452054114
7680	POLQ	10.42092624	0.185424512	0.877860117	0.211222202	0.822712029	1.111852015
7681	PP2D1	12.6105102	0.109075462	0.802708622	0.125884252	0.891912785	1.127171011
7682	PPP1R16A	12.29582972	0.260118452	0.781894918	0.222676997	0.729278115	4.970111874
7683	PSMD9	9.202217778	0.279475567	0.950950221	0.292890848	0.768841211	5.424409548
7684	PYROXD2	11.20247049	0.627595891	0.869424765	0.721842565	0.470290662	1.111852015
7685	RAB11B	22.96282142	-0.24420829	0.626670109	-0.549265521	0.582822229	1.744472942
7686	RAB20	10.65162748	-0.289548927	0.882060517	-0.227892507	0.74299294	1.484924171
7687	RAB29B	12.86279409	-0.061250447	0.788527087	-0.077802868	0.927984865	1.501894191
7688	RASAL2	11.40591828	-0.224160256	0.862627098	-0.286922189	0.698812814	2.179972047
7689	RBMS2	14.8197815	-0.141875979	0.741482529	-0.191240961	0.848258472	4.174194401
7690	RDH12	10.40402428	0.189098577	0.877445955	0.215510227	0.829269529	1.151297484
7691	RNF185-AS1	12.0888746	-0.222597225	0.784052694	-0.424202242	0.671418226	2.458474854
7692	RPL6P27	12.26190569	-0.284204297	0.782228662	-0.491224984	0.62226722	1.009255155
7693	RPP40	7.198429846	0.769841211	1.099102784	0.700427041	0.482660654	5.120189781
7694	RRM1-AS1	10.87109726	-0.47945272	0.877295949	-0.546512101	0.584712284	1.141891444
7695	RRP7BP	12.1240299	0.101588961	0.78291886	0.129591168	0.896889891	1.948949147
7696	SAMD11	11.12292725	0.116979125	0.85092892	0.127470647	0.890658792	2.147470957
7697	SBDSP1	15.26594281	0.227552548	0.740688261	0.44222856	0.658222821	4.944451182
7698	SCMH1	18.21662262	-0.499972694	0.684858222	-0.720029705	0.465265915	1.448011115
7699	SCN7A	12.76416619	-0.792142975	0.791742802	-1.000504169	0.217066581	1.944122811
7700	SETD4	20.1781906	-0.819782658	0.646259529	-1.268209059		

						0.204687602	1.494115497
7701	SIPA1L2	16.5110426	-0.98409479	0.712780076	-1.280642954	0.167288769	4.127904441
7702	SLAMF6	8.467724214	0.041450281	1.040901919	0.029821505	0.968225422	1.148541227
7703	SLC15A4	7.710618062	-0.047617076	1.091858894	-0.042611016	0.965214471	1.141891444
7704	SLC16A4	10.15074059	0.409187248	0.902220545	0.452026472	0.650529679	1.141891444
7705	SLC27A2	17.00641998	-0.766591968	0.694977094	-1.10204628	0.270007024	1.124425445
7706	SLMAP	16.29162278	-0.572685505	0.70812802	-0.810142772	0.41785755	1.141891444
7707	SMCHD1	20.46251202	-0.774040455	0.642056242	-1.202690122	0.228709268	1.995118484
7708	SMPD2	11.40218206	0.201292591	0.827598124	0.259829587	0.718974576	1.14571499
7709	SPPL2B	17.50746706	-0.40020781	0.68927847	-0.580524246	0.5615544	1.711204007
7710	SPPL2	14.80849017	-0.228025072	0.742117422	-0.220727752	0.748409126	1.974429841
7711	SRP19	11.46247502	1.091822989	0.866186615	1.260492951	0.207491229	5.191247029
7712	ST14	7.152264545	-0.022858508	1.184509198	-0.02858442	0.977196028	1.95105711
7713	ST6GALNAC4	17.54417628	-0.072952025	0.695028856	-0.104964028	0.916404245	2.452004142
7714	STK16	12.84220546	-0.052572062	0.762826665	-0.068826215	0.945127871	1.115979487
7715	STK19	17.51587298	-0.656842145	0.685720156	-0.957886595	0.228119947	1.481098815
7716	STX1A	21.12282812	-0.557629642	0.671691481	-0.820202047	0.406424558	1.127171011
7717	SURF1	11.90412001	0.046108101	0.824490816	0.055922122	0.95540205	1.490147278
7718	TCTN1	12.27046179	0.264524201	0.781159772	0.228620214	0.724888218	1.472441847
7719	TMEM117	10.42222766	0.222257912	0.879795722	0.266287196	0.714150782	2.105241001
7720	TMEM227	12.44168616	-0.420562216	0.92452944	-0.465704649	0.641426924	5.492244052
7721	TMEM44-AS1	11.24951172	-0.584982155	0.861421564	-0.679089286	0.49708129	2.441711827
7722	TRAIP	10.67989082	0.247452424	0.87201199	0.282772972	0.776584268	1.940512189
7723	TRIM47	21.22654112	-0.710792752	0.642900825	-1.102885422	0.269642829	1.11214424
7724	TXNRD2	14.59460086	-0.499027451	0.752957165	-0.661877722	0.50804959	4.428084124
7725	UBE2V1	16.02427228	-0.250028221	0.718997064	-0.486828581	0.626279814	1.484924171

7726	UBN2	18.21248274	-0.429215891	0.691414622	-0.620779298	0.524744846	5.144414454
7727	USP12	10.70242241	0.106855262	0.914991207	0.116782927	0.907022072	1.148541227
7728	USP49	8.909529279	0.216725592	0.966888978	0.22414724	0.822642658	5.958029194
7729	VASH1-AS1	9.205202187	0.925061251	0.942299589	0.992212242	0.221092514	1.742888458
7730	VSIG10	15.55982551	-0.954857902	0.747580429	-1.27726442	0.201508907	1.490554804
7731	WDR86	9.618654884	-0.467186292	0.958095788	-0.487619608	0.625819205	1.140104259
7732	XRCC4	18.69778221	-0.978407999	0.668128404	-1.464279226	0.142090261	2.717479402
7733	ZBTB2	15.29526051	-0.22205982	0.720785058	-0.442072284	0.6584269	1.151247411
7734	ZBTB41	8.715497498	0.988122227	0.969647804	1.019052849	0.208177402	5.412118142
7735	ZCCHC2	14.76222487	-0.621642196	0.752215842	-0.828595207	0.401696501	1.985921184
7736	ZCCHC2	11.66780824	0.608722108	0.825202912	0.728820528	0.466105225	2.194049477
7737	ZNF197	8.629292568	-0.022169582	1.012578004	-0.021872596	0.982549585	1.99140188
7738	ZNF280B	20.49250159	-0.852127209	0.654782029	-1.202918027	0.192602749	1.148541227
7739	ZNF410	8.701270826	0.625068766	0.964128204	0.658690525	0.510094518	1.115979487
7740	ZNF490	11.9069152	-0.197194269	0.829995525	-0.224756452	0.81429777	1.725421479
7741	ZNF566	11.88726812	0.41542929	0.821210742	0.499740071	0.617258112	1.997111511
7742	ZNF572	17.27676592	-0.112801717	0.702902026	-0.161902442	0.871282679	1.995118484
7743	ZNF599	7.944254672	0.602418097	1.020826746	0.590121877	0.555108942	1.982095829
7744	ZNF612	14.51849706	-0.488920585	0.764426128	-0.629582297	0.522442545	2.449944181
7745	ZNF670	10.17222212	-0.166011126	0.910105994	-0.182408562	0.855262107	2.497881028
7746	ZNF844	10.12272851	-0.445978551	0.915809668	-0.486977224	0.626274294	1.711850019
7747	ZNF846	8.208829791	0.488829762	1.000420926	0.488624084	0.625100778	1.490554804
7748	ABHD10	18.00269218	-0.551928762	0.674122682	-0.818724222	0.412942768	4.708221044
7749	ABI2	9.680620944	-0.026422229	0.92582225	-0.028246001	0.977465949	4.422548149
7750	AC002991.1	11.14124197	-0.404227227	0.875702462	-0.461614296	0.644257941	1.121400219
7751	AC004542.2	8.421072879	0.752225089	1.025795042	0.722209242		

						0.462269778	2.941507117
7752	AC005072.1	10.41809992	0.75624197	0.900682822	0.829621824	0.401114845	2.144885471
7753	AC006001.4	8.46772214	0.924842826	0.98672626	0.927284092	0.248612444	2.452054114
7754	AC008781.1	15.07296409	-0.280187887	0.721692185	-0.519600858	0.6022418	5.904217891
7755	AC012184.2	14.90429014	0.04072599	0.770727027	0.052852979	0.957848252	1.494115497
7756	AC021729.4	18.45568855	-0.862045404	0.670965474	-1.28627294	0.198247474	2.710414417
7757	AC022400.1	10.90497026	0.585691992	0.874574062	0.66968827	0.502056521	2.112847042
7758	AC024292.1	14.81684495	-0.226685646	0.727252145	-0.456614452	0.64794819	1.115979487
7759	AC067852.2	12.28229212	-0.529268277	0.827675285	-0.64288706	0.519648672	1.721597221
7760	AC090152.1	12.42894987	0.75178085	0.817844797	0.919221902	0.257979517	4.494105271
7761	AC090498.1	20.29110278	0.260202474	0.670956067	0.287810002	0.698156642	4.448827427
7762	AC091057.1	15.14066198	-0.100675897	0.7607506	-0.122227584	0.894717281	4.118157127
7763	AC096526.2	14.0597288	-0.406824062	0.761916557	-0.522948216	0.592277292	4.244071994
7764	AC106707.1	14.22425298	-0.147242756	0.752299496	-0.195722587	0.844826522	4.411989498
7765	AC120224.2	11.88422157	0.172155256	0.822227297	0.209276721	0.824154164	2.994281191
7766	AC125050.1	16.5251692	-0.228270645	0.704672526	-0.480028809	0.621199797	2.449478754
7767	AC125782.2	7.719022904	0.227222106	1.071699774	0.205220012	0.760114819	5.711417297
7768	AC141586.2	10.68267502	0.662240047	0.869516262	0.762768909	0.445601226	2.945495124
7769	ACVR1B	15.04192546	-0.572249402	0.72662077	-0.778215096	0.426442219	2.979419174
7770	ADHFE1	15.07579944	-0.282575472	0.720564278	-0.288159458	0.697898022	1.714141254
7771	AGFG1	11.41427219	0.20012942	0.827892882	0.258206971	0.720188421	2.482755244
7772	AHI1	15.12285015	0.477897992	0.744274265	0.642099211	0.520808704	1.11214424
7773	AL078581.4	10.87109628	0.189225508	0.888224012	0.212126626	0.82122819	1.710014481
7774	AL121952.1	12.29577859	0.479670628	0.780121184	0.614858947	0.528647876	2.941457155
7775	AL122020.1	16.20570922	-0.212522462	0.705762679	-0.202542862	0.762228262	1.501844119
7776	AL258224.2	15.62191169	-0.094298229	0.726804716	-0.127982662	0.898162694	1.104121282

7777	AL260091.2	6.218917271	0.642504587	1.227122652	0.522586282	0.600566225	1.980210554
7778	AL291069.1	15.57679645	-0.180861206	0.719944622	-0.251215442	0.801647547	1.501844119
7779	AL291824.2	9.902926169	-0.092125219	0.918628296	-0.100284628	0.920118252	4.958820551
7780	AL445524.1	22.51992104	-0.627647898	0.651549811	-0.978662214	0.227746264	1.741052211
7781	AL606804.1	12.27876651	-0.167966859	0.780717474	-0.215144229	0.829654872	1.71847044
7782	AMN1	6.967577445	0.682172489	1.117787078	0.610289296	0.541670186	1.004519809
7783	ANKRD22	10.92028812	0.17682685	0.867758222	0.202774204	0.828529874	1.944122811
7784	ANKRD50	8.712560952	0.622141277	0.972026107	0.650692102	0.515244617	1.714914429
7785	AP000879.1	22.9785881	-0.752447505	0.628688289	-1.196852872	0.221262906	1.11214424
7786	APOBEC2D	11.27771292	0.180210949	0.849672528	0.212212285	0.821941422	5.707490715
7787	AQP11	17.58620501	-0.408486225	0.70621208	-0.578225919	0.562027248	1.99804282
7788	AQP2	8.425452247	0.940111964	1.092949559	0.859274142	0.290124121	2.954824512
7789	ARHGAP22	7.485486416	0.825205216	1.071256104	0.779742717	0.425541741	4.180124295
7790	ARMCX2-AS1	15.26012186	-0.225842652	0.727120472	-0.219951299	0.749005249	1.11214424
7791	ATL2	15.24898072	0.522620729	0.756558945	0.690799762	0.489691285	2.481157507
7792	BACE1-AS	14.07664076	-0.409177759	0.757904289	-0.529880445	0.589279485	2.111202011
7793	C11orf1	16.26626477	-0.119602779	0.724000824	-0.162946281	0.870560652	2.441741899
7794	CACNA1I	8.422809102	-0.119175924	1.026042168	-0.115029994	0.908421248	1.11214424
7795	CAPN12	14.59171428	-0.296452802	0.747251705	-0.520547872	0.595722126	1.115979487
7796	CAPN15	8.442265204	0.92250154	1.019062222	0.916028797	0.259646547	1.995118484
7797	CCDC167	14.60877652	0.496491748	0.766198885	0.647992205	0.516989281	4.14424041
7798	CDR2	12.12264727	-0.115404859	0.782608282	-0.147461842	0.882767496	1.148541227
7799	CEACAM4	22.9702822	-0.169955559	0.62917972	-0.270122427	0.787066061	2.475199287
7800	CENPJ	12.60468824	-0.58222222	0.818410928	-0.71275102	0.475999829	1.971804514
7801	CENPQ	14.61708221	-0.299921021	0.762722644	-0.524246608	0.600027485	2.481157507
7802	CEP76	16.04296955	-0.080499857	0.72176718	-0.111521612		

0.911194799	7.419404158
7803	CHUK	16.56471287	0.092742776	0.71067148	0.121907228
0.895057592	2.008512827
7804	CILK1	15.24882052	-0.229266265	0.724161402	-0.448492156
0.652797224	1.484924171
7805	CLSPN	14.62857289	0.91045022	0.798052914	1.140827986	0.25292725
1.4708075
7806	CMPK2	12.87120755	-0.056028666	0.762602052	-0.072482497
0.941421276	1.940512189
7807	CNPY2	12.88916095	0.499128749	0.79792529	0.62552788
0.521618095	4.421445401
7808	COL9A2	9.188190022	0.771202224	0.95112542	0.810822799
0.417466865	9.424177275
7809	CROCCP2	9.669229619	-0.17226126	0.942440416	-0.182754228
0.854206187	7.412922444
7810	CRPPA	12.27677249	-0.418485215	0.822247214	-0.508952025
0.610785144	2.455829509
7811	CRYZL2P	16.58719629	-0.257768515	0.708802648	-0.26266754
0.716106286	1.459514175
7812	DCAF4	16.22674795	-0.040452686	0.708857929	-0.057067409
0.954491498	2.424091104
7813	DHFR	10.62228206	-0.146508269	0.887894256	-0.165006522	0.86892884
1.719741984
7814	DOP1A	15.29809466	-0.22264546	0.72886091	-0.442670825
0.658002848	4.444541111
7815	DYSF	12.24777795	-0.055009509	0.777054196	-0.070792272
0.942562002	1.481098815
7816	EBI2	11.88422157	0.172155256	0.822227297	0.209276721
0.824154164	2.495044491
7817	EDARADD	11.15546877	0.629878419	0.86612842	0.728780075
0.460040552	2.44424555
7818	EEF1B2P2	10.14780404	0.124748226	0.900422554	0.128542929
0.889810546	4.14894207
7819	ENPP4	16.06640202	-0.727207024	0.727182792	-1.000169478
0.217228497	1.127171011
7820	ENTPD1-AS1	12.22514522	-0.600978151	0.78928925	-0.761220217
0.446465768	1.4708075
7821	EXOC8	7.721858251	0.517256592	1.056122954	0.489764192
0.624200772	1.711850019
7822	FAM214A	12.14218594	-0.491206712	0.849628222	-0.578260819
0.562088042	1.714974701
7823	FAM76B	10.164716	0.121008251	0.894421274	0.125292218
0.892280828	4.449217497
7824	FEZ2	11.88716692	0.292550295	0.824677827	0.255957666
0.721872269	5.901521404
7825	FFAR2	16.21821407	-0.847668199	0.728444581	-1.162668755
0.244558276	5.181115774
7826	FKBP9	12.60190206	-0.247657272	0.802802265	-0.422515861
0.665266527	1.487719457
7827	FLVCR1	15.12782662	-0.195941262	0.76285288	-0.256516682
0.797551901	1.4708075

7828	FLYWCH1	15.00242085	-0.771178572	0.754228755	-1.022459491	0.206562476	4.179081484
7829	FOXRED2	12.87498421	-0.062281701	0.788842092	-0.078952218	0.927069754	1.110208992
7830	FRA10AC1	14.22146857	-0.556264144	0.755612121	-0.7262082	0.461542157	4.418188848
7831	GMDS-DT	17.24841128	-0.52225929	0.690512255	-0.772409811	0.429871745	1.701558704
7832	GPATCH2	16.24241864	0.125286429	0.711859485	0.190046549	0.849272654	2.941759912
7833	GPR82	6.942159448	0.057268062	1.201664682	0.047740491	0.961922064	1.715485275
7834	GSTA4	11.28900525	0.205255189	0.846028286	0.260927829	0.718152299	1.707179225
7835	GSTM2	9.17406226	0.447509282	0.950401289	0.47086261	0.627728126	1.115979487
7836	GTF2H2	10.88517289	0.052688415	0.865666625	0.062019725	0.950547116	1.492290151
7837	GTF2C4	12.52057856	-0.209922452	0.816261287	-0.279640019	0.70421265	4.414794908
7838	GUSBP1	11.19779872	1.049527766	0.862459524	1.21690088	0.222641921	1.944182881
7839	GYPC	29.26577284	-0.772005202	0.570924928	-1.252177219	0.17621862	4.119917481
7840	HAUS4	18.81064422	-0.029967268	0.674269997	-0.044427575	0.96455561	4.111955441
7841	HPS6	12.20725724	0.880861216	0.821470774	1.072297692	0.282586242	1.474478194
7842	HSBP1L1	11.21749596	0.761449072	0.879184062	0.866086074	0.286442962	1.002484441
7843	IFI27L1	14.79988292	-0.62545457	0.7444015	-0.852644925	0.292201752	1.14571499
7844	IQCN	12.66880969	0.412922786	0.784065604	0.526655912	0.598422557	4.447407495
7845	IQSEC1	15.88270152	0.042121666	0.729057609	0.057007282	0.954529294	5.707490715
7846	ITFG1-AS1	12.89194622	0.271195206	0.789005222	0.24271788	0.721058462	1.487719457
7847	KCNE1	8.726687625	0.98588521	0.975755657	1.010281242	0.212212622	8.1144745
7848	KCNH2	19.4492266	-0.656522257	0.655494544	-1.001569522	0.216551547	2.449944181
7849	KDM4B	11.15248222	-0.674970282	0.92216524	-0.721940624	0.464204802	4.481914047
7850	KDM8	12.24289851	-0.779714886	0.845792955	-0.921872225	0.256594655	2.150554245
7851	KIAA0255	15.06450811	-0.279086796	0.721465008	-0.518256911	0.604279022	2.941507117
7852	KIAA0556	14.20460667	-0.245524044	0.759829961	-0.222129157	0.746589857	2.70250147
7853	KIAA1841	14.61712228	-0.101097808	0.747440767	-0.125258622		

						0.8924074	1.104121282
7854	KLHL26	12.5298258	-0.817277122	0.851757282	-0.959518797	0.227297455	1.719741984
7855	KRTCAP2	10.4406225	0.604844581	0.879212717	0.687928062	0.491491786	4.412959541
7856	L2MBTL2	18.79651766	-0.181962614	0.66981494	-0.271661026	0.785882671	2.471514101
7857	LACTB2-AS1	11.88711686	-0.071081656	0.822998989	-0.086269068	0.921172025	1.140054197
7858	LINC01068	7.492992456	1.509861786	1.072674046	1.406257122	0.159647784	4.49415521
7859	LINC01128	11.65626566	-0.014560287	0.826118281	-0.017414267	0.986106128	1.995118484
7860	LINC01255	11.87861082	-0.427222729	0.842166719	-0.519295918	0.6025544	1.127171011
7861	LUM	17.52999857	-0.070525726	0.694284466	-0.10159485	0.919078268	2.111202011
7862	MAP2K14-AS1	9.176848645	0.129402998	0.95255214	0.125848587	0.89194098	1.145474919
7863	MAP7D1	11.17222066	0.229747779	0.850660028	0.281827262	0.77806822	2.44424555
7864	MAPK9	17.76069971	-0.252697227	0.681627786	-0.517422772	0.604852289	4.254518971
7865	MBLAC2	6.708622959	0.598700972	1.161882906	0.515285119	0.606252796	1.11214424
7866	MEF2A	12.2251952	-0.268299195	0.782469827	-0.242577576	0.721916274	1.701558704
7867	MFSD4A	12.2542299	-0.296215698	0.811606499	-0.26509774	0.715028448	1.742888458
7868	MGAT4B	14.11612526	-0.20762601	0.759894524	-0.272220028	0.784676291	2.948220482
7869	MIER2	11.72699902	1.280222852	0.867751996	1.475460567	0.140088762	1.95105711
7870	NAB2	16.49980126	-0.225145998	0.720225056	-0.465225099	0.641691502	4.181917022
7871	NAGPA	20.47922278	-0.85122695	0.644227688	-1.221108755	0.186465104	1.714914429
7872	NANP	14.11612526	-0.20762601	0.759894524	-0.272220028	0.784676291	1.942248524
7873	NDUFAF2	8.429479796	0.221809252	1.00175624	0.221420486	0.824765042	5.195151214
7874	NENF	12.26185456	-0.16522822	0.774968627	-0.212248288	0.821055212	2.482755244
7875	NFKBIE	9.729922917	0.875956866	0.964676218	0.908021896	0.26286127	2.141050215
7876	NGRN	19.21290482	-0.218757286	0.67447581	-0.472600027	0.626498562	1.14571499
7877	NLK	11.25796662	0.057421957	0.865459881	0.066260046	0.947091179	1.948949147
7878	NLRP2	14.62280207	0.09541886	0.746158098	0.127880218	0.898242767	2.444842409

7879	NXF2	7.242492949	1.442097925	1.091422722	1.222217226	0.186095826	2.921418514
7880	P2RY2	7.710566926	0.229892821	1.062248855	0.210240454	0.75627811	4.119498514
7881	PABPN1	12.24294857	-0.412112906	0.817884972	-0.505100252	0.612488425	4.411448124
7882	PGAP4	10.12924807	0.126656924	0.908299982	0.129428596	0.889111477	2.101555717
7883	PGM2	22.44102195	-0.497182691	0.621040715	-0.800562762	0.422284227	4.477520779
7884	PHF1	19.50284788	-0.256089574	0.655094797	-0.542569524	0.586727729	1.4708075
7885	PHRF1	15.48925126	-0.547298798	0.750722805	-0.729019426	0.46598978	1.492290151
7886	PIWIL4	12.8777695	-0.287789996	0.798882624	-0.260240195	0.718667519	4.952159858
7887	PPFIBP1	7.260405906	1.425207871	1.117190162	1.284658528	0.198911626	1.711850019
7888	PRDM4	11.88716692	0.292550295	0.824677827	0.255957666	0.721872269	7.250851207
7889	PRICKLE2	12.89472152	0.046246292	0.792427452	0.058486606	0.952261021	1.115979487
7890	PSMA2	12.88264894	0.259227284	0.765566441	0.469261462	0.62881129	1.982095829
7891	PSMA6	9.458285781	0.505291541	0.926009289	0.529826022	0.589210116	1.124425445
7892	PUSL1	18.77109966	-0.412166051	0.664420647	-0.620229018	0.525024612	10.07802148
7893	RAB40C	9.182469277	-0.029190422	0.966772281	-0.020192696	0.975912576	2.951115891
7894	RABL6	10.70804297	0.657044011	0.882552872	0.744481187	0.456585418	1.749559251
7895	RAD51AP1	14.25812782	-0.457421921	0.764022665	-0.598715122	0.549262865	7.47188405
7896	RBM4	10.69112101	0.661195628	0.869747627	0.760215502	0.44712578	4.499874044
7897	RBMXL1	11.58294701	-1.029884818	0.92116162	-1.116760824	0.264096602	1.115979487
7898	RFX2	17.7071285	-0.429297592	0.709595721	-0.605129905	0.545092685	1.459514175
7899	RIPOR2	16.25202798	-0.851422596	0.715742724	-1.189577452	0.224212512	4.410498172
7900	ROCK2	11.59712274	-0.278440129	0.8515814	-0.444296906	0.65675565	4.440418811
7901	RPARP-AS1	9.410425195	0.207145621	0.940529064	0.22024128	0.825682171	4.192108258
7902	RPP14	8.704155027	1.182809495	1.001516024	1.181019042	0.227595159	2.124419492
7903	RRAGD	15.11225857	-0.784658202	0.78685171	-0.997212299	0.218661472	1.985881114
7904	RRNAD1	14.78869172	-0.126584825	0.762212126	-0.178960515		

0.857968706 2.742047229
7905 RTTN 12.10926202 -0.485855519 0.820866672 -0.584757501
0.558710784 4.404111158
7906 RWDD4 17.59212589 0.500072806 0.710461747 0.702871542
0.481512775 4.142575125
7907 RYK 17.82962887 -0.279692942 0.701158792 -0.298902425
0.689965102 5.410221958
7908 SEMA4F 11.12562124 -0.271782416 0.862750545 -0.215019698
0.752746698 4.454710745
7909 SERPINI1 21.25442807 -0.8714717 0.646297268 -1.248198202
0.17759461 2.954001177
7910 SHMT1 19.66868648 -0.761499707 0.658048982 -1.157208242
0.247187291 1.121400219
7911 SIRPB2 12.41064646 -0.071642228 0.822820256 -0.08706927
0.920616452 8.411750758
7912 SLC16A1 12.05211414 -0.552210466 0.807202905 -0.684102665
0.492909708 1.940512189
7913 SLC16A12 12.90875807 -0.406508898 0.841427858 -0.482112204
0.629016069 4.151021112
7914 SLC25A28 10.45475918 0.895897604 0.885062891 1.012241742
0.211422482 2.424141147
7915 SLC25A42 11.1102524 -0.267126499 0.868926217 -0.207421282
0.758522655 4.444541111
7916 SLC27A4 16.20418752 -0.470561792 0.769120901 -0.611817715
0.540658268 2.47841114
7917 SLC25E2 19.74772575 -0.240142122 0.65625418 -0.265872785
0.714460005 5.171419724
7918 SLC27A2 11.88149622 0.051255265 0.821606082 0.062284254
0.950256754 8.841501941
7919 SMIM27 12.29294225 0.270259202 0.777524057 0.476225276 0.62284259
1.159852441
7920 SMYD2 15.21217127 -0.420280285 0.726110757 -0.5788102 0.56271718
1.490404844
7921 SNHG20 10.89641416 -0.212706608 0.872544242 -0.244922521
0.806515649 1.995118484
7922 SP2 18.59406986 0.294228701 0.692247977 0.569490578
0.569022262 1.159852441
7923 SREBF1 12.89189617 -0.065082546 0.795220295 -0.08184112
0.924772048 1.99804282
7924 STARD8 10.40670847 -0.22682615 0.901227155 -0.251657956
0.801205457 1.121400219
7925 STK28L 14.87041617 -0.245780827 0.752128161 -0.226780528
0.742822888 2.489712485
7926 STXBP6 10.42655589 0.754010629 0.889208228 0.847861964
0.296514849 2.458474854
7927 SUDS2 17.55526651 -0.072625679 0.687726229 -0.107069652
0.914722722 1.985881114
7928 SYNJ2BP 12.77257102 -0.57076881 0.795699416 -0.717217116
0.472178449 4.194211004
7929 TADA2B 15.78780025 -0.210552582 0.719690048 -0.42150879
0.666098462 2.484091852

7930	TARBP1	8.698424281	0.297060767	0.981082877	0.202788249	0.762051162	1.954841495
7931	TCEA1P2	15.20928599	-0.229055424	0.727221748	-0.21492998	0.752814818	2.444195488
7932	TCF4	19.25802006	-0.247904982	0.661245457	-0.274849452	0.707772422	1.124425445
7933	THAP11	18.47542585	-0.7802517	0.667592178	-1.168755005	0.242502252	4.878108427
7934	TLCD2A	15.86116892	0.126252565	0.722458115	0.188725599	0.850200049	1.009255155
7935	TMA16	21.96266765	-0.242625794	0.625274228	-0.282426625	0.701296045	2.700447212
7936	TMCO6	22.1905825	-0.227044225	0.62177102	-0.522491104	0.592692667	4.488584741
7937	TMEM242	14.21212666	0.81556219	0.822897092	0.991086488	0.22164225	4.947489144
7938	TMEM268	12.22165725	-0.521469167	0.826292216	-0.642197518	0.520095942	2.71100871
7939	TMEM99	11.05946747	-0.658862705	0.921447929	-0.707252224	0.479246991	2.92708911
7940	TNFRSF10A	12.2702606	0.157126289	0.775054821	0.202742158	0.82922657	1.944182881
7941	TNFSF12	9.207927226	0.925576522	0.942614446	0.991481782	0.221450284	2.979419174
7942	TNRC6C-AS1	16.05794604	-0.726227672	0.722782887	-1.004902966	0.214942576	1.977475108
7943	TRAK1	21.76295507	-0.085164258	0.642564881	-0.12252799	0.894558776	2.452004142
7944	TRAPPC2B	8.727978951	1.170887596	0.985841207	1.187702921	0.224950074	1.127171011
7945	TROAP	14.55221977	-0.595262604	0.750192719	-0.792614212	0.427420055	1.71847044
7946	TRPS1	15.24447228	-0.706295788	0.749984114	-0.941747665	0.246221845	2.129114978
7947	TTLL7	16.25928066	0.299520657	0.720429214	0.554551514	0.579201461	1.479142479
7948	UGCG	16.7727822	-0.64267856	0.697227252	-0.92162108	0.256721041	2.918582179
7949	UHRF2	18.19966952	-0.225628708	0.711812514	-0.471526181	0.627265028	4.45499819
7950	USP25	10.65720697	-0.152187728	0.882487797	-0.172289761	0.862245072	2.714792995
7951	VAMP2	16.59291715	0.176969077	0.709149611	0.249551116	0.802924506	4.445951018
7952	WDR5B	12.60289709	-0.107677064	0.767115167	-0.140266221	0.888270628	1.985921184
7953	WDR90	8.709675545	-0.028754412	1.022721275	-0.027526124	0.970065502	1.490554804
7954	WWC2	10.26159222	-0.258229217	0.919806117	-0.28958125	0.696846219	2.450118878
7955	XPA	19.06109277	-0.219021944	0.672279476	-0.225272675		

0.744974709 1.445184849
7956 YPEL1 14.09076742 -0.202922256 0.754421254 -0.270212709
0.786919689 4.19215841
7957 Z95221.1 9.862481622 0.062786409 0.972451062 0.06456511
0.948520265 2.455889571
7958 ZBTB5 12.12488014 0.591855654 0.841199102 0.702585692
0.481690824 1.159852441
7959 ZDHHC2 19.55641909 -0.286898222 0.669626181 -0.428445497
0.668226807 1.009255155
7960 ZFAND2 11.64228905 0.111912675 0.829277779 0.124951976
0.892649845 1.441251511
7961 ZFHX2 19.48200045 -0.89885857 0.656942227 -1.268245994
0.171225089 1.194941058
7962 ZFP14 9.421776582 0.827491282 0.974582892 0.859222161
0.290157285 1.974429841
7963 ZFP82 16.5617784 -0.520976019 0.706174742 -0.727742772
0.460670167 4.405244947
7964 ZHX2 16.60689264 -0.426902842 0.724212909 -0.595064226
0.551800524 2.904050591
7965 ZNF141 12.64229169 0.097815205 0.774066762 0.126265462 0.89944264
1.504481474
7966 ZNF174 12.10006472 -0.222289717 0.786069205 -0.424122602
0.671476402 5.914900541
7967 ZNF219 16.76716167 -0.552227499 0.696885676 -0.792426288
0.428106227 4.711458714
7968 ZNF25 11.91522014 0.045012612 0.822102188 0.054095054
0.956859422 4.910819904
7969 ZNF429 20.51891851 -0.268224758 0.648509574 -0.412756047
0.679052776 4.952109794
7970 ZNF512 12.82698257 -0.625992642 0.810164541 -0.772672464
0.429715655 4.190272011
7971 ZNF524 14.56928191 0.202226822 0.790422695 0.282482206
0.702102725 1.710014481
7972 ZNF551 11.92228216 0.407272617 0.820566128 0.490255427
0.622882404 4.441811592
7973 ZNF568 16.75202499 -0.721620911 0.700152424 -1.044942702 0.29604899
4.101714299
7974 ZNF570 8.619445067 -0.251122084 1.088525458 -0.222576824
0.747015748 2.47841114
7975 ZNF77 14.21017724 -0.660210424 0.759898206 -0.868945904 0.28487672
1.921209907
7976 ZNF778 10.66287767 0.116517286 0.870650747 0.122827812
0.892528722 2.44424555
7977 ZNF808 12.16219204 0.429408549 0.788257197 0.544756902
0.585920724 5.414441144
7978 ABCA2 14.10205768 0.411220504 0.779208752 0.527686229
0.597717126 4.144157785
7979 ABCC2 11.18262091 1.054409266 0.87412297 1.206246828
0.227722222 1.725421479
7980 AC008442.1 14.64796968 -0.607211416 0.82275469 -0.727247901
0.460971608 1.714974701

7981	AC009402.1	14.82275582	0.149265552	0.746282949	0.20014574	0.841266602	4.151021112
7982	AC009506.2	14.87215022	-0.245255284	0.752205052	-0.226181516	0.744287025	4.91774184
7983	AC016727.1	9.660822504	0.128577582	0.927152644	0.127200109	0.890872625	7.129898449
7984	AC016888.1	21.67262229	-0.275687284	0.648029292	-0.425417622	0.670522255	2.441440141
7985	AC022400.8	9.182468202	0.774250605	0.970540674	0.797854872	0.424954714	1.714974701
7986	AC025171.1	19.02726779	0.164502182	0.68078172	0.241627191	0.809061202	1.441251511
7987	AC027682.2	12.29289211	0.59226217	0.784172661	0.755269145	0.450087508	7.4492014
7988	AC072611.1	20.55269129	-0.120014225	0.652429568	-0.199277181	0.842045928	7.48594044
7989	AC092279.1	17.05148201	-0.075089908	0.692687081	-0.108402794	0.912675292	4.449114841
7990	AC097276.2	17.5157707	-0.216176468	0.684265811	-0.462066724	0.644022465	4.18475128
7991	AGAP2	14.86190905	-0.049019874	0.727070789	-0.06650622	0.946974718	1.14571499
7992	AKAP1	14.22708812	-0.146817787	0.752277092	-0.195128566	0.845284472	2.941709851
7993	AKAP5	19.96414896	-0.952097058	0.648221407	-1.468782722	0.141891458	1.140104259
7994	AL022542.1	14.8449971	-0.046592092	0.724946492	-0.062296577	0.949450712	4.444149291
7995	ANAPC2	14.14707279	-0.108978768	0.776770622	-0.14029722	0.888425152	4.952109919
7996	ANKH	12.08021622	-0.555297826	0.805407586	-0.689586028	0.49045455	5.447994118
7997	AP000777.2	12.17626758	0.888585511	0.794997551	1.117721066	0.262686142	2.941709851
7998	AP002422.1	10.62611721	-0.145875906	0.899848499	-0.162111629	0.871217949	4.101444227
7999	APBA2	19.79567526	0.266446485	0.668812912	0.298286576	0.690245251	1.474478194
8000	APOBEC2F	15.02052294	-0.7727729	0.751741114	-1.029207678	0.202225116	4.91774184
8001	ARHGAP12	12.1628821	-0.122220821	0.828010465	-0.157898781	0.874526559	1.002484441
8002	ARHGAP21	9.680579808	0.272609404	0.926009272	0.295471559	0.767622675	5.892014544
8003	ARMC5	18.77288287	-0.178809417	0.662812782	-0.26926724	0.787647092	5.100812018
8004	ARRDC2	16.02521225	-0.722979416	0.719629465	-1.004641144	0.215069678	2.115088204
8005	BAHD1	17.8876885	0.78215779	0.710058682	1.102947981	0.270049756	2.709112415
8006	BCKDHB	18.76542789	-0.177872451	0.665692161	-0.267198857		

						0.789216056	1.971804514
8007	BIVM	12.0972282	0.109664672	0.797058262	0.127586771	0.890567012	1.492290151
8008	BMI1	20.28811618	-0.094651918	0.64899282	-0.145844098	0.884044462	2.497881028
8009	BRPF2	17.4960224	-0.297552756	0.689599057	-0.576498404	0.564278248	4.419420155
8010	C12orf75	14.12588159	0.40524429	0.757241979	0.525219625	0.592497997	1.154181949
8011	C1orf159	12.88249768	-0.056918726	0.766404692	-0.074267207	0.94079777	1.944122811
8012	C1orf54	16.51661202	-0.425251821	0.705271557	-0.602018108	0.546496642	7.401488201
8013	C2orf14	12.84289445	-0.056190802	0.802261888	-0.070021744	0.944168294	1.942248524
8014	CAMK1D	12.14885555	0.246548424	0.812246449	0.426129649	0.670012277	1.002484441
8015	CCDC71L	17.51855599	-0.48429851	0.680711442	-0.711606229	0.476708642	2.478124724
8016	CCPG1	11.89268625	0.050189545	0.824180911	0.060896272	0.951441818	2.704228014
8017	CDK8	8.994046952	1.425182025	0.978617715	1.456221506	0.145202762	2.115088204
8018	CDKN2D	16.84609974	-0.029262065	0.700066714	-0.056084747	0.955274295	7.151189794
8019	CHCHD5	20.18270896	-0.591291794	0.646822441	-0.914202148	0.260557577	5.454454721
8020	CLHC1	20.61417274	-0.791626212	0.664851775	-1.190680904	0.222778877	5.94284154
8021	DBNDD1	12.6264286	0.214046252	0.76989291	0.407909126	0.682240285	2.441510102
8022	DESI2	19.21552885	-0.786514084	0.654784461	-1.20118012	0.229681225	1.715425214
8023	DHRS12	9.657997157	-0.020065841	0.94590795	-0.021212212	0.982075495	1.997111511
8024	DNAJC9-AS1	9.458224645	0.827878592	0.921626024	0.888628781	0.274202618	1.451040197
8025	DONSON	8.718180511	1.278299907	1.002945462	1.272882247	0.169788646	1.742888458
8026	DUSP7	12.08026628	-0.218201625	0.79710474	-0.272742729	0.784282221	4.117081224
8027	EAF1-AS1	12.60926646	-0.648804275	0.804118512	-0.806851682	0.419751929	1.494115497
8028	EEF1A1P6	9.480917295	1.162522417	0.941427657	1.224827288	0.21689102	1.948949147
8029	EFCAB14-AS1	20.4849924	-0.225482961	0.629155622	-0.52488462	0.599662402	5.297700145
8030	EGLN2	9.948040222	0.788087996	0.905975007	0.869878297	0.284266917	1.114488241
8031	EPB41L2	16.10216022	0.26172041	0.718504529	0.502425115	0.614658288	7.804724081

8032	FADS2	12.65820855	0.447574997	0.797062782	0.561529712	0.574426481	4.894970521
8033	FBRS	19.27204554	-0.172552212	0.657004141	-0.264158477	0.791657805	2.114421218
8034	FMR1	12.44720452	-0.077469908	0.884206654	-0.087605252	0.920190427	4.187587717
8035	FSCN1	24.19725577	0.110295475	0.627978162	0.172882827	0.862742522	2.420471574
8036	GADD45A	9.959221659	0.942812222	0.907782847	1.028587826	0.298996465	1.118815024
8037	GEN1	18.79074577	-0.412585192	0.66487622	-0.622048202	0.522910107	2.709172242
8038	GNAT2	19.46608742	-0.502162664	0.652201488	-0.770202917	0.441119622	1.744714004
8039	GPX2	7.977976215	1.409528188	1.048861628	1.242864755	0.178992125	1.459514175
8040	GTF2I	10.42774602	0.752206622	0.882902498	0.851119465	0.294702994	1.118815024
8041	HDAC4	16.2424808	-0.947542279	0.728219845	-1.20117612	0.192198174	7.911281557
8042	HEATR2	11.2061525	0.904912192	0.85758474	1.055188078	0.291229216	1.492290151
8043	HGF	18.61256578	-0.209200522	0.718986852	-0.420050221	0.66715902	2.101555717
8044	HINT2	21.45201222	-0.80589959	0.624169954	-1.291154092	0.19665025	1.445184849
8045	HTATSF1P2	11.85040646	0.058176567	0.854440669	0.06808721	0.945716122	1.481098815
8046	IDNK	15.79615512	-0.40258725	0.716048882	-0.562620862	0.572005278	4.147840412
8047	IL15RA	9.447042219	0.668402594	0.922079819	0.717108751	0.472206997	4.444511087
8048	KIF11	15.22619675	-0.225528997	0.725554922	-0.448662102	0.652675428	1.985921184
8049	KLHL22	18.28974875	-0.506274126	0.672544751	-0.752922591	0.451496422	9.282042445
8050	KRT8P46	8.169182849	0.224606658	1.067040979	0.204211988	0.760966292	5.441215414
8051	L2HYPDH	14.6229922	0.094522187	0.74729292	0.126469201	0.899260527	4.477192415
8052	LHPP	12.89751572	0.284225252	0.789169772	0.486872869	0.626248426	2.745881484
8053	LINC00174	11.695709	-0.269240498	0.92297042	-0.291819206	0.77042486	8.110802804
8054	LMBR1	17.27651246	-0.525760842	0.685729821	-0.781200182	0.424625962	1.971804514
8055	LMLN	12.67800579	0.212161649	0.816257205	0.261145165	0.792980562	5.181115774
8056	LMO7-AS1	19.86042768	-0.02285886	0.695265796	-0.047254064	0.962210729	2.452004142
8057	LSM12	14.10767821	0.20662485	0.764071107	0.401217164		

0.688186628 4.415502582
8058 LTBP4 21.4728107 -0.522825044 0.624749422 -0.828456227
0.401774522 2.150554245
8059 MAML2 20.76259507 -0.656525712 0.645990058 -1.016224794
0.209474746 4.701711411
8060 MAP2K1 14.1202109 0.200152227 0.754255518 0.265220116
0.790755166 2.915797894
8061 MAPRE2 26.61748068 -0.60415655 0.579926982 -1.041762411
0.297521845 1.957477841
8062 MARCHF2 21.00219917 -0.74924264 0.554525268 -1.251299995
0.176599254 1.749559251
8063 MBOAT2 12.89662425 0.148677191 0.766672922 0.192925189 0.84622446
2.484091852
8064 MDM1 12.17422249 0.241605017 0.817972926 0.417622252
0.676222521 1.124425445
8065 MFSD9 17.05142295 -0.220172219 0.688992705 -0.479209486
0.621789606 4.499874044
8066 MICA 17.22012474 -0.202464469 0.690906661 -0.292041709
0.769490271 2.491141404
8067 MIEF2 15.29242289 -0.027074468 0.75096521 -0.049269089
0.960625161 4.720914495
8068 MIF 16.55042594 -0.429228904 0.699271756 -0.612892252 0.52928652
2.712149972
8069 MIPEP 12.62448462 0.454195157 0.818515775 0.55490092
0.578962422 2.491141404
8070 MIR2918 12.90025108 0.497908244 0.791708274 0.628902724
0.529412088 2.478124724
8071 MLYCD 12.12259624 0.105209549 0.781555955 0.124742454
0.892814715 5.117785027
8072 MRPL46 8.954502284 0.525495295 0.965248449 0.554717102
0.579088176 1.977415144
8073 MTBP 12.61512727 0.209291116 0.770502772 0.271759068 0.78580728
7.121291419
8074 MTND4P12 15.22908215 0.051672026 0.728925824 0.070886925
0.942487742 4.481121409
8075 MYL4 15.62186055 0.091271188 0.725575054 0.125791518
0.899896956 4.440418811
8076 NACC2 12.40691866 0.151848002 0.782626919 0.192775889
0.846251267 2.71408521
8077 NAF1 9.444207972 0.510216026 0.922658249 0.546576904 0.58466944
1.942248524
8078 NBPF14 14.57191594 -0.292568421 0.744980268 -0.292719629
0.694526564 1.140104259
8079 NDRG1 14.29888484 -0.242847901 0.767922044 -0.217542107
0.750822295 2.455829509
8080 NEK2 22.22992685 -0.472499151 0.620288746 -0.761617864 0.44628811
1.148541227
8081 NIPAL2 12.60096054 -0.219709049 0.774105088 -0.412004712
0.679602161 5.454154871
8082 NOC2L 12.2824421 0.402102982 0.815879192 0.492846227
0.622121245 5.904247952

8083	NXT2	19.57905072	0.452262472	0.676568187	0.668612276	0.502742202	1.710014481
8084	OR14L1P	18.4867261	-0.294951502	0.676085874	-0.42626227	0.662645655	4.451141844
8085	PAPLN	12.60941652	-0.220952142	0.775792715	-0.412708621	0.679087505	2.111202011
8086	PARP11	19.42221257	-0.272012164	0.69757822	-0.291271242	0.695522772	1.474478194
8087	PARP16	12.1222875	-0.265226222	0.822882425	-0.428114908	0.661202988	8.124111802
8088	PDE5A	15.27126205	0.045627208	0.720027264	0.062500691	0.950164112	5.100812018
8089	PET100	10.19852884	0.844401151	0.89489199	0.942578847	0.245284906	1.728117944
8090	PLA2G12A	18.04202552	-0.722602502	0.687105184	-1.052118969	0.29228647	1.944122811
8091	PLK4	12.15647021	0.421281696	0.782082495	0.550876429	0.58171828	5.481011444
8092	PPARA	12.52892224	-0.41854968	0.805415527	-0.51966924	0.60229412	4.194042705
8093	PRKCE	17.82272584	0.128478752	0.682871578	0.187869706	0.850978791	1.982095829
8094	PRR11	10.420784	0.227116625	0.882798472	0.270125821	0.711288744	1.710014481
8095	PYGO2	14.27787406	0.147045954	0.74869659	0.196402596	0.844295064	2.704187955
8096	QDPR	19.02272822	-0.602647772	0.665646861	-0.90525659	0.265276506	1.707179225
8097	RAB24	12.24861819	0.409287704	0.877461946	0.466444962	0.640897025	4.911981411
8098	RAB4B	12.68651182	0.559166179	0.808671217	0.691462942	0.489274661	9.588274851
8099	RAP2A	17.51282522	-0.921691112	0.696507992	-1.222202994	0.185724627	4.922441414
8100	RBM27	8.202006762	0.21429642	1.026788475	0.202240659	0.761706465	7.874421971
8101	SAMD8	15.84410572	-0.225022024	0.717842958	-0.212469711	0.752922829	2.945545198
8102	SEC14L2	15.59629249	-0.464147279	0.725522207	-0.621025161	0.528017525	1.127171011
8103	SEC21B	16.09455416	-0.448789877	0.72598799	-0.618178101	0.526457944	1.448011115
8104	SFXN5	15.1122575	-0.287704294	0.742796242	-0.287226078	0.698514822	1.148541227
8105	SIAH1	12.28428607	0.26287545	0.772980214	0.240922025	0.722154002	4.488584741
8106	SIGLEC5	9.922622226	0.242295922	0.910877957	0.275896595	0.706992772	2.714792995
8107	SLC25A1	12.16576751	0.242261177	0.812722748	0.421824757	0.672145625	1.447971154
8108	SLC26A4	15.56261864	-0.178228612	0.720582485	-0.247229641		

						0.804645282	2.122544185
8109	SLC2A1	12.29084802	0.049171475	0.806176425	0.060992442	0.951264422	2.92708911
8110	SLFN12	10.92018692	0.045664497	0.880710266	0.05184962	0.958648518	8.879111184
8111	SMCO4	11.60821287	-0.279296029	0.859612971	-0.441229954	0.659029291	7.451184808
8112	SNHG10	7.696289117	0.226018995	1.088592272	0.208672928	0.757570229	2.71757918
8113	SPIRE1	16.04286825	-0.169729826	0.715270168	-0.227275521	0.812442044	2.17712771
8114	STAC2	12.61597859	0.109979796	0.80284558	0.126987484	0.891040688	2.951115891
8115	STX6	12.14796417	0.101025891	0.785252722	0.128627602	0.897644412	4.440221297
8116	SURF2	16.11997216	-0.179652462	0.72971787	-0.246194412	0.80552174	4.11705449
8117	SYCP2	11.86721842	0.054902924	0.824467948	0.065792928	0.94754189	4.950214511
8118	TAPT1-AS1	16.89982115	0.828749574	0.724459417	1.14295582	0.252641955	1.99804282
8119	TBC1D24	16.10878096	0.270421462	0.714287906	0.278525891	0.705022526	1.980140492
8120	THAP12	15.79048444	-0.591189519	0.720155744	-0.82091898	0.411692419	1.148541227
8121	TIFA	15.75287524	-0.2982221	0.740108787	-0.528058872	0.590526404	4.888147152
8122	TMEM18-DT	10.44221659	0.182740071	0.902449252	0.20249246	0.829520971	4.181917022
8123	TMEM187	14.26496081	-0.870257254	0.792451891	-1.096925048	0.272674158	1.000899177
8124	TMEM229B	11.68740221	0.869262175	0.827244662	1.028117522	0.29921524	1.940512189
8125	TMEM250	12.61508721	-0.108527574	0.768495545	-0.141222826	0.887685217	4.245810198
8126	TMEM41A	15.58226595	-0.088101551	0.718226692	-0.122648205	0.902285602	9.07044788
8127	TMEM62	11.61676985	-0.280754961	0.857841102	-0.44285255	0.657149192	1.492290151
8128	TNFSF12B	14.29200072	0.246644722	0.751717709	0.461126821	0.644700429	10.9755119
8129	TTC12	11.21177412	0.76402048	0.865280254	0.882986048	0.27724281	1.715485275
8130	UBE2D1	28.21060208	-0.620566604	0.610682529	-1.022560412	0.201809662	2.92708911
8131	VNN1	12.85140899	-0.580812181	0.78514099	-0.729755265	0.459448508	1.980140492
8132	VSTM1	11.16655877	-0.146215071	0.876641905	-0.166904025	0.867445559	1.472441847
8133	WRN	12.86259169	-0.28464202	0.798222646	-0.256551266	0.721427691	2.111202011

8134	YOD1	7.465627914	0.428820222	1.104126668	0.297422894	0.691048262	2.458474854
8135	YTHDF1	20.44266245	-0.547950622	0.64070096	-0.855226165	0.292420419	7.851849211
8136	Z99129.4	12.17142821	0.585081967	0.81442446	0.718290486	0.472516551	1.744714004
8137	ZBTB29	10.22952741	0.684881428	0.921418222	0.725210216	0.462150527	4.115841071
8138	ZBTB44	11.64502219	0.112404028	0.822821274	0.124966121	0.892628652	4.14424041
8139	ZNF122	12.65741717	0.2022896	0.776992495	0.260249491	0.7945942	4.471411711
8140	ZNF128	12.62204922	0.206561824	0.766704088	0.269415216	0.787610102	2.150504202
8141	ZNF285A	10.46216402	1.207822409	0.89429428	1.250588169	0.176827292	4.114144987
8142	ZNF544	10.20415947	0.691658275	0.895219202	0.772612214	0.429751262	1.940512189
8143	ZNF558	11.91248272	0.526421287	0.822199224	0.651642119	0.51462207	4.41822892
8144	ZNF728	9.428527279	0.201541456	0.94720947	0.212751442	0.82152084	1.140104259
8145	ZNF75D	12.80072218	-0.252517499	0.787876457	-0.221772162	0.747624548	2.714125281
8146	ZNF776	8.960122916	0.268974252	0.980207184	0.276424758	0.706601142	1.479142479
8147	ZRANB1	12.62284049	-0.226087824	0.80924512	-0.291728248	0.770486687	1.118744971
8148	AC005252.1	14.41448218	0.445612785	0.757220058	0.588299708	0.556264024	2.111202011
8149	AC008622.2	28.01277284	-0.026582242	0.61701255	-0.059289204	0.95272176	2.710444488
8150	AC024226.2	11.91242258	0.792004624	0.841875159	0.940762564	0.246826549	1.14571499
8151	AC072120.2	17.81416866	0.049281264	0.677641806	0.072724799	0.942025114	4.952159858
8152	AC074022.1	8.726525291	1.177827679	0.987268572	1.192026612	0.222858951	1.487719457
8153	AC100778.2	12.28712022	0.264282605	0.774050282	0.241429461	0.7227802	4.914954575
8154	AC108124.2	9.929522219	1.112527229	0.952085015	1.169577624	0.242170982	1.991551818
8155	AC122008.2	17.28096966	0.719289625	0.705706442	1.019289255	0.208018158	1.942248524
8156	AC145207.5	11.18246965	0.501726872	0.852640265	0.588450762	0.556229772	2.119447474
8157	AL126028.4	15.10957114	0.28445921	0.72771205	0.521150916	0.602261644	1.4708075
8158	ANAPC1	16.57575274	-0.254904766	0.698996271	-0.264672517	0.715255876	4.111955441
8159	AP000688.1	15.86947258	0.227872126	0.715477242	0.218491102		

0.750112442 4.428024171
8160 ARHGEF28 17.21884224 0.122889189 0.688872812 0.192907884
0.847021111 2.447422592
8161 ASB12 10.67118121 0.252672125 0.881072271 0.286779112
0.774281457 2.498119515
8162 ATAD5 21.2505144 -0.15204294 0.620018109 -0.24291822
0.808068674 2.712149972
8163 ATP12A2 20.29642089 -0.281509812 0.650722171 -0.586286789
0.557682808 4.291188122
8164 ATP22 11.71266995 0.721671486 0.851291022 0.859484557
0.290072227 1.954841495
8165 B2GALT4 21.95117292 -0.441242425 0.616728672 -0.71561848
0.47422696 2.972798541
8166 BCAR1 22.92262929 -0.097507462 0.618988465 -0.157527107
0.874829446 1.490554804
8167 BCAS2 20.42689156 -0.769965202 0.626249766 -1.209971849
0.226289695 2.44424555
8168 BMF 15.08698849 0.195250185 0.729294802 0.264202811
0.791622645 5.494149228
8169 BNIP2 12.72874059 0.790466922 0.859625992 0.91954749
0.257809278 8.904419181
8170 BORCS7 15.86668729 0.412086227 0.718929996 0.572192968
0.566512268 2.124279421
8171 C1R 11.6280601 0.266242828 0.862078755 0.424245792
0.671212648 4.19215841
8172 C9orf85 14.25807562 0.251742028 0.752807726 0.224404146
0.728074611 7.414170951
8173 CARD14 7.741502282 1.578596004 1.076078755 1.466989286 0.14227897
4.415502582
8174 CD99P1 15.08126772 -0.282729998 0.722741699 -0.285865849
0.699596024 2.188419045
8175 CDH22 17.2961095 -0.801722294 0.707570416 -1.122079161
0.257181012 2.478271098
8176 CDK19 12.25602152 -0.27129821 0.786285495 -0.245027917
0.720065865 1.499040844
8177 CENPK 11.69570792 0.252151122 0.8521401 0.412254972
0.679419816 4.914009141
8178 CENPM 15.08698849 0.195250185 0.729294802 0.264202811
0.791622645 1.000899177
8179 CEP57L1 12.60926528 -0.10679062 0.771562898 -0.128408006
0.88991797 5.410181894
8180 CETN2 19.20202202 0.122718844 0.659508706 0.186076154
0.852285041 5.414711214
8181 CFAP57 12.4077589 0.627712225 0.812744867 0.784641256
0.422662875 1.995118484
8182 CFLAR-AS1 19.98956588 -0.244197221 0.64142811 -0.526610909
0.591526407 1.982095829
8183 CIT 14.82226219 -0.626629052 0.758967048 -0.828809872 0.40157601
2.972798541
8184 CLTB 12.86269924 0.050965262 0.761425782 0.066922108
0.946624956 1.95105711

8185	CSNK1G2	19.52272417	-0.124747044	0.649729067	-0.207289589	0.825705612	5.149824451
8186	CYP2R1	17.09266171	-0.164220696	0.695629077	-0.226075089	0.812274404	1.499040844
8187	DEAF1	12.89741452	0.272041208	0.789142244	0.244720229	0.720297107	1.121400219
8188	DENND6A	12.60180078	0.112521422	0.822049099	0.128107848	0.890155184	1.151247411
8189	DHX40	10.92859178	0.21008682	0.872255026	0.255052422	0.722550219	8.141841425
8190	DIPK1A	17.24992102	0.550268682	0.702619028	0.782054852	0.424182227	4.442754917
8191	DNAJB4	11.95187712	0.654874891	0.825104092	0.784182548	0.422922417	2.441510102
8192	DNAJC21	18.22640694	-0.021752902	0.671229811	-0.047200004	0.962274125	5.171419724
8193	DNAJC20	19.74752226	-0.286711827	0.642277549	-0.601158609	0.54772425	2.489712485
8194	EBAG9	18.82182228	0.254514284	0.674968656	0.525220725	0.599422801	2.484878128
8195	ELAC1	21.01976219	-0.122600776	0.622425127	-0.192549064	0.846528985	2.422204911
8196	FAM52B	19.21122882	-0.55774096	0.676474782	-0.824481524	0.409666009	4.118474417
8197	FGD4	10.94421241	0.176011622	0.890296528	0.197699998	0.842279791	1.741052211
8198	GABPB2	12.84016665	0.158772977	0.779569274	0.20266885	0.828612286	1.11214424
8199	GNRHR2	20.22215242	-0.20200628	0.642698549	-0.469172467	0.628945649	5.470781402
8200	GPR85	24.92218191	-0.561985489	0.587562805	-0.956468796	0.228825442	1.492290151
8201	GPSM2	22.09920919	-0.90144252	0.609142154	-1.47985741	0.128911204	2.92708911
8202	GPX7	14.81952796	-0.129450742	0.742297925	-0.187828271	0.851002424	1.4708075
8203	GTF2A1	14.28060821	0.147428825	0.747911026	0.197124189	0.84272252	5.178190419
8204	GTPBP2	14.22827825	-0.147622207	0.751416102	-0.196458669	0.84425118	1.115979487
8205	H1-2	11.9208285	0.411610817	0.822282977	0.499962128	0.617101728	2.471444041
8206	H4C9	14.11021222	-0.208218066	0.765985842	-0.402280891	0.687402721	1.980140492
8207	HELB	14.24299901	0.255524288	0.77415215	0.459255754	0.646050517	4.190272011
8208	HLA-F	18.57700557	0.477072107	0.686202119	0.695122224	0.486971809	1.448011115
8209	HOXB-AS2	14.11602209	0.202268121	0.755086614	0.26919842	0.787776988	2.704228014
8210	HSPBAP1	16.51924605	-0.982661512	0.722258592	-1.240129556		

0.180202229 1.4708075
8211 ICAM4 16.81217462 -0.120019557 0.692270576 -0.172245827
0.862279596 1.148511175
8212 INKA2 12.57071209 -0.457591541 0.84267475 -0.542279087
0.587557282 1.711850019
8213 INO80C 16.62788006 0.246968569 0.712097912 0.4872484 0.62608221
1.445184849
8214 INTS2 11.40576488 0.422278454 0.848170201 0.509659974
0.610289699 1.121400219
8215 ITGB8 14.86180786 -0.145297978 0.729794064 -0.196528449
0.844188741 4.952159858
8216 KCTD6 12.18541262 0.240260589 0.827270296 0.411426097
0.680760121 1.719741984
8217 KIF18A 9.99026909 1.098000255 0.92225826 1.176297427
0.229426099 7.877117154
8218 KIF1B 12.14791204 0.222451157 0.781206292 0.412707744 0.67982075
5.128795811
8219 KLHL42 15.04182219 -0.18095948 0.751996907 -0.240628596
0.809825224 2.491111244
8220 KMT5A 12.60652004 -0.212682798 0.774921177 -0.274455079 0.78272492
4.441784844
8221 KNL1 15.68110129 0.842255484 0.745492947 1.129795202 0.25856252
2.987915114
8222 LAMP1 11.40855016 0.176220874 0.847646829 0.207906012
0.825202255 4.715194042
8223 LINC00476 9.21624098 1.112129687 0.957297657 1.161749095
0.245227297 1.714141254
8224 LINC01942 9.972257122 1.102142844 0.91462401 1.20611621
0.227772685 4.429128819
8225 LPAR1 9.196492676 0.941942584 0.971428089 0.969627277
0.222227225 1.148511175
8226 LPAR2 15.07848245 -0.090244265 0.721855182 -0.122209047
0.901862277 7.891192844
8227 LPCAT4 10.42197412 0.22572215 0.891996285 0.26516084
0.714991248 7.274170204
8228 LPGAT1 12.87956112 -0.264257428 0.779201142 -0.229222727
0.724441196 2.444195488
8229 LY96 10.22514696 1.219880062 0.912228289 1.446858872
0.147926454 7.911091121
8230 MAP2K5 24.94287915 -0.684822212 0.592222478 -1.154410165 0.24822205
1.487749519
8231 MAP7 15.24205757 -0.127576811 0.722974058 -0.190020029
0.849285599 5.181075714
8232 MAPKAPK2 19.72229668 -0.525291272 0.642122442 -0.822217729
0.40522962 1.141891444
8233 MARCKSL1 6.480554769 1.222119252 1.221746151 1.000202986
0.217162902 1.159852441
8234 MASTL 14.84494596 0.148442121 0.729614542 0.200702256
0.840920525 2.954824512
8235 MCM10 12.22622429 -0.051250107 0.792486196 -0.064796216
0.948226254 1.148541227

8236 MCM8 10.92167982 0.212754119 0.866577116 0.262061294
0.717206152 1.140054197
8237 MMD 9.694605282 0.422229954 0.921782925 0.452248804
0.650269592 1.718510711
8238 MPZL2 12.28512619 0.051124274 0.810442256 0.062094407
0.949691228 4.41440412
8239 MRM1 12.45208289 1.169102472 0.796542588 1.467722491
0.142179619 1.14571499
8240 MSL1 18.58819677 0.08289721 0.67192879 0.124858412
0.900625642 4.118414255
8241 MSRB2 25.65766525 -0.81116618 0.58189854 -1.292999229
0.162217804 4.920417149
8242 MT-TP 15.16025707 0.671105065 0.741126987 0.90551967
0.265190144 5.404154114
8243 MTERF2 17.20750095 -0.19907288 0.682690154 -0.291600621
0.770591992 1.474478194
8244 NAA16 12.64408068 0.10596185 0.799984162 0.122454924
0.894624466 5.195151214
8245 NDOR1 17.29052892 -0.450649112 0.685042574 -0.657841029
0.510640287 4.114108271
8246 NFE2L1 19.70524246 -0.282446826 0.657679616 -0.58150922
0.560897229 1.004519809
8247 NFIA 18.99219249 -0.598081928 0.655256251 -0.912605695
0.261449958 4.889299941
8248 NOP14-AS1 20.81149452 -0.272429778 0.659042426 -0.566624784
0.570969122 4.14424041
8249 OAS1 18.89802721 0.82191295 0.697107206 1.179025222
0.228284151 2.495044491
8250 OGFRL1 14.87871981 -0.147808597 0.749515569 -0.197205506
0.842666722 2.150504202
8251 ORC1 18.96220292 -0.516070424 0.664267244 -0.776901692
0.427216772 4.415041109
8252 PABPC1L 16.26889772 -0.207252066 0.724222956 -0.286265827
0.774674522 4.494155424
8253 PARP6 25.71984272 -0.222681187 0.597457526 -0.291126025
0.695704084 11.54151891
8254 PC 27.40279904 -0.229089612 0.584026778 -0.409274242
0.682265029 1.124425445
8255 PCMTD2 10.29526481 -0.222158527 0.922874851 -0.227888982
0.811967202 2.709172242
8256 PELP1 10.70227 0.951272687 0.876779162 1.0849627
0.277928176 2.105241001
8257 PNRC1 17.79152595 -0.254799822 0.674418682 -0.526082425
0.598820922 4.44091158
8258 PRR29 12.45570949 0.875149157 0.819842605 1.067459962
0.285764184 2.191144291
8259 PTGR1 10.46589817 1.208254542 0.894226998 1.251005879 0.17669255
2.119417412
8260 PTS 11.91222246 0.047548482 0.825611161 0.056902642
0.954622749 2.450118878
8261 PURB 10.67118121 0.252672125 0.881072271 0.286779112

						0.774281457	4.118414255
8262	RABGAP1	17.27919755	-0.799948007	0.701012625	-1.141120454	0.25281564	2.724541199
8263	RPL18AP2	11.12551907	0.249597872	0.874522227	0.285410226	0.775229874	5.474451094
8264	RPLP0P6	10.20694268	1.160278856	0.909591294	1.275714418	0.20205649	1.501844119
8265	RPS27AP5	16.50248428	-0.157974492	0.727858079	-0.21409875	0.820470051	4.181947095
8266	SELENOO	11.6646692	0.109656152	0.842022172	0.120229275	0.896284954	4.474458049
8267	SFMBT2	12.24190486	-0.489226182	0.800712429	-0.611112728	0.541124948	5.445110709
8268	SLC25C1	21.20822451	-0.148812862	0.644258599	-0.220982122	0.817227914	5.427194852
8269	SLC25E1	12.88607214	-0.17448121	0.804085777	-0.216992404	0.828212485	1.000849115
8270	SLC42A1	18.72707225	-0.565770062	0.660419968	-0.856682247	0.291620517	1.98955779
8271	SLC42A2	14.29194959	0.552499229	0.752822687	0.722902665	0.462007452	1.747545212
8272	SMIM15-AS1	14.21574566	-0.042524929	0.768614912	-0.056627745	0.954841722	1.49412147
8273	SMIM8	12.82429582	-0.687118422	0.800560686	-0.858296498	0.290728762	0.998905149
8274	SMOX	14.61140947	0.296828221	0.751771558	0.527857468	0.597598267	1.497044814
8275	SNN	26.19252624	-0.562885127	0.588229089	-0.958425598	0.227842152	1.971445822
8276	SNX12	19.50552089	-0.205855062	0.650887444	-0.216268295	0.751798874	1.010194474
8277	SPATA2L	22.51245694	0.127988991	0.622797291	0.221562249	0.824652895	1.752185955
8278	SPINT1	12.41252822	0.589427252	0.777115588	0.758480798	0.448162204	1.508258141
8279	SRGAP2C	8.702952641	0.814519282	0.992422614	0.819911227	0.412266654	1.157859425
8280	SRM	15.59250601	-0.2684279	0.728507296	-0.505742528	0.612026707	1.144548209
8281	SS18L1	10.6729666	-0.018852049	0.902201201	-0.020895615	0.982228924	1.75040047
8282	SSBP2-AS1	18.27840629	-0.422492471	0.666182887	-0.625700562	0.52497161	1.497044814
8283	SSX2IP	11.70127862	0.60444107	0.842622402	0.716472612	0.472698956	1.144548209
8284	TATDN2P2	9.959120227	-0.10212218	1.000215461	-0.102100181	0.918677157	1.724172998
8285	TCAF1	9.179521658	0.44876722	0.972259824	0.461522928	0.644422758	1.149402454
8286	TERF2	18.79920067	-0.027254098	0.661621264	-0.041244042	0.967021622	1.758854449

8287	TFE2	20.54418418	0.011952102	0.642929277	0.01859162	0.98516688	1.724172998
8288	TGIF1	22.90222196	-0.678229886	0.604744122	-1.121515446	0.262068521	1.982927158
8289	THOP1	18.09292051	0.406289671	0.684217952	0.592801526	0.552644862	1.010194474
8290	TIMM22B	9.227482242	0.762229281	0.964814216	0.790120648	0.429451472	0.759497947
8291	TIRAP	12.41054419	0.297580565	0.80522405	0.492751479	0.621481704	1.140494981
8292	TOP1MT	17.62564729	-0.1621422	0.719422051	-0.226769807	0.820602742	1.499901142
8293	TP52I12	25.41572294	-0.491166481	0.585125568	-0.829406298	0.401241251	1.144548209
8294	TRIM65	14.15274241	0.612187258	0.759259502	0.80761226	0.419212769	1.140494981
8295	TTC8	12.57265606	-0.521927795	0.795746272	-0.668462991	0.50282746	1.477219512
8296	TUBD1	12.24846692	-0.058887419	0.82088562	-0.070872075	0.942498772	1.71781801
8297	TYMP	18.82202458	-0.021267228	0.669220614	-0.046715029	0.962740244	1.747545212
8298	UBE2E2	18.02795784	-0.226928966	0.671598564	-0.227892762	0.725442248	1.499901142
8299	UBE2O	15.56812808	-0.265209851	0.722147075	-0.505727799	0.61204775	0.759497947
8300	UNK	17.58062217	0.252175672	0.686882797	0.268586422	0.712426017	1.155014188
8301	VKORC1L1	18.7682121	0.052978542	0.676926997	0.078262146	0.927619529	1.501487448
8302	ZDHHC24	22.5049509	-0.045210429	0.616099491	-0.072281718	0.941502265	1.140494981
8303	ZFP26L2	15.11797706	0.095099661	0.729722076	0.120222125	0.896210785	1.144548209
8304	ZNF16	12.5811621	-0.208827428	0.781959287	-0.267056616	0.78942557	1.157859425
8305	ZNF184	25.40716684	-0.85056668	0.582594445	-1.457461918	0.144988916	1.501487448
8306	ZNF189	8.670078665	0.207886925	1.024159751	0.297716987	0.765919177	1.499901142
8307	ZNF220	12.6102067	-0.221460992	0.812779208	-0.284427226	0.776082997	0.998905149
8308	ZNF218	9.922471076	-0.229958954	0.980061266	-0.244840744	0.806579745	1.747545212
8309	ZNF506	18.20665962	-0.108217876	0.667429267	-0.162128886	0.871196485	1.488410828
8310	ZNF542	10.91879441	-0.215804098	0.910185949	-0.227098912	0.812580057	1.729109245
8311	ZNF546	12.87110515	-0.262101222	0.774198184	-0.229827056	0.72297924	0.998905149
8312	ZNF552	14.96059444	0.622225866	0.808062677	0.770022209		

0.441280194 1.750250408
8313 ZNF57 11.8756722 -0.067020446 0.82811246 -0.079965925
0.926264249 1.140494981
8314 ZSCAN21 10.9498821 0.441602092 0.875101792 0.504629401
0.612819156 1.722428451
8315 ZSWIM2 18.28979774 0.120152006 0.681149882 0.191078266
0.848464197 1.007241118
8316 ABHD11 24.41272784 -0.220557094 0.595244742 -0.555226428
0.578722948 1.144548209
8317 AC004148.1 20.61182092 0.256880855 0.661952124 0.529122927
0.58979444 1.007241118
8318 AC005726.4 11.17212719 0.629258062 0.862887411 0.740826005
0.458792882 1.724172998
8319 AC006064.2 24.41282797 0.025060217 0.622560275 0.029617279
0.968298251 1.007241118
8320 AC012258.4 21.45564628 -0.878205758 0.625686866 -1.402586691
0.160441961 1.754011201
8321 AC079742.1 20.79164602 -0.512080676 0.647905808 -0.791906278
0.428415226 0.759497947
8322 AC084876.1 19.02696527 -0.442942819 0.654555489 -0.678225575
0.497622229 0.759497947
8323 AC100786.1 17.0794829 -0.161782028 0.688224209 -0.225024091
0.814182274 1.754011201
8324 AC102858.1 15.85240926 -0.122210277 0.716451862 -0.185920672
0.852499128 1.497044814
8325 AC116522.1 15.08126666 0.197026529 0.745896485 0.264160702
0.791656091 1.157859425
8326 ADGRE2 26.87622277 -0.682507101 0.564625881 -1.208777572 0.22674821
1.747545212
8327 AL117222.1 20.52140021 -0.627502612 0.644574206 -0.97251648
0.220296674 1.981101811
8328 AL602756.1 17.56261902 0.17269125 0.685562925 0.25225566
0.799992289 0.998905149
8329 ALOX12-AS1 12.41611268 0.51626729 0.807708157 0.629299228
0.522628219 0.759497947
8330 ANKRA2 22.42715181 -0.285195045 0.602851102 -0.472292656
0.626717198 1.140494981
8331 ANKRD27 17.5014917 -0.296801194 0.689764282 -0.575270718
0.565108222 1.485775491
8332 AP000762.4 21.59119225 0.141487146 0.650828889 0.217295207
0.827900288 1.007241118
8333 AP000872.2 12.56414895 -0.212280589 0.804129855 -0.288470229
0.697668008 1.984771505
8334 AP2S2 16.80922809 -0.291452248 0.692474204 -0.42027987
0.674281014 1.754011201
8335 ARID2A 22.4596844 -0.249281225 0.598770722 -0.582497682
0.559558216 1.140494981
8336 ASB16 19.21542658 -0.470098687 0.659278154 -0.712942466 0.47588126
1.149402454
8337 ASB2 16.06912402 0.187561171 0.722295729 0.259672651
0.795115519 0.759497947

8338	ATL1	27.94040418	-0.029972705	0.582685046	-0.051252522	0.959044609	1.149402454
8339	ATMIN	16.26752205	0.124017849	0.705106122	0.190067627	0.849256128	0.759497947
8340	ATP7A	12.4122284	0.76027996	0.81906777	0.928225952	0.252290275	1.128111221
8341	ATXN7L2	12.28124822	-0.057714717	0.784625966	-0.072556042	0.941262649	1.010194474
8342	AZIN2	14.24279769	-0.250226279	0.762825541	-0.458654069	0.646482602	1.007241118
8343	B4GALT7	25.77275972	-0.292477228	0.54808662	-0.522622411	0.592595187	1.499901142
8344	B9D2	15.64996156	0.556062228	0.724520412	0.767479628	0.442796295	0.759497947
8345	BTN2A2	15.55296027	-0.262624498	0.72840115	-0.49784998	0.618589785	1.010194474
8346	BUB1B	15.41075428	0.611890587	0.722472282	0.825277008	0.402505471	1.714981472
8347	BZW1-AS1	18.2461021	0.202682624	0.67142071	0.201871287	0.762750185	1.505511795
8348	C10orf88	20.21222178	-0.025072068	0.64404112	-0.028929296	0.96894676	1.155014188
8349	C4orf48	22.11122226	-0.257126895	0.554622429	-0.64289822	0.519641271	1.155014188
8350	CALHM6	16.81769407	-0.292462815	0.694206766	-0.421229965	0.672587165	1.128111221
8351	CBWD2	22.4577905	-0.10696117	0.622978755	-0.171692127	0.86267879	1.752185955
8352	CDC26	18.25015187	-0.229421492	0.672212527	-0.504921809	0.612606722	0.759497947
8353	CDCA2	12.88025121	-0.172629095	0.809845265	-0.212162024	0.8211998	1.981101811
8354	CEP125	14.25792426	-0.149648259	0.754296018	-0.198268427	0.842756811	1.754011201
8355	CHAC2	11.87000142	0.200207112	0.864246792	0.247262715	0.72821884	1.010194474
8356	CLCN7	20.52274159	-0.228978082	0.626961042	-0.522180242	0.594601165	1.007241118
8357	CNTLN	19.21690822	-0.480290512	0.672744092	-0.714076152	0.475180092	1.144548209
8358	COQ2	24.25255994	0.10591841	0.609042967	0.1729092	0.861926744	1.128111221
8359	COQ7	11.91506661	0.048028712	0.844281716	0.056880222	0.954640522	0.759497947
8360	CWF19L2	22.1198468	-0.294868112	0.66112694	-0.597256162	0.550226267	1.741491995
8361	CYP2A5	12.89080122	0.047276777	0.767816689	0.061702229	0.950799161	1.157859425
8362	DHX22	14.60842287	-0.097415874	0.745299298	-0.120707052	0.896007054	1.155014188
8363	DLGAP4-AS1	11.67595955	0.874127224	0.84895826	1.02964676		

0.202175856	1.505511795
8364	DND1	16.57848688	-0.254528209	0.699922682	-0.262646841
0.716121744	1.49412147
8365	DOCK7	20.290649	-0.600226555	0.655908142	-0.915122888	0.26012712
1.010194474
8366	EIF4A1	11.67874484	0.610457545	0.825422572	0.720707462
0.464957857	0.759497947
8367	ELMOD2	15.89195402	0.21686252	0.719762827	0.440222252
0.659768179	1.724172998
8368	EMC9	14.61692891	0.197566877	0.741486487	0.266447027
0.789894944	1.508258141
8369	EML4-AS1	18.57406902	0.220155417	0.671299542	0.476847822
0.622470482	1.485775491
8370	ERMP1	12.56698429	-0.20567225	0.802484678	-0.256295672
0.797722529	1.49412147
8371	EXO1	8.962805928	0.704122888	0.984790407	0.715008881
0.474602555	0.998905149
8372	FAAP20	12.17606518	0.656092262	0.784999022	0.825788627
0.402272826	0.759497947
8373	FAM120C	19.01850929	-0.442080022	0.652405002	-0.678109226
0.497702272	1.508258141
8374	FAM122B	11.94221995	0.521960202	0.822455598	0.629025428
0.522806284	0.759497947
8375	FAM184B	16.21674711	-0.124066101	0.702899918	-0.176506068
0.859896282	1.497044814
8376	FAM210A	22.26081214	-0.276200904	0.61621798	-0.448219482
0.652994804	1.488410828
8377	FAM98B	10.16446247	0.124677502	0.924682908	0.122290018
0.892884929	1.747545212
8378	FSD1L	12.82718004	-0.262717277	0.782922296	-0.462286272
0.642159112	1.508258141
8379	FTH1P20	20.22596449	0.228257112	0.651422917	0.504052825
0.61422425	1.144548209
8380	GCLC	17.24258727	-0.190441209	0.719471194	-0.264696086
0.791242592	1.984771505
8381	GDPD5	12.86622464	-0.296968664	0.828444476	-0.47917252
0.621815184	1.149402454
8382	GIPR	19.22829989	0.497676129	0.670216711	0.742560027
0.457748097	0.759497947
8383	GMDS	26.21222262	-0.062228257	0.590280585	-0.105572012
0.915921128	1.508258141
8384	GPR122	15.4474627	1.009058259	0.752775811	1.240449897
0.180099122	1.010194474
8385	GRPEL2	15.29784112	-0.220252208	0.728821745	-0.422457562	0.66468229
0.759497947
8386	GSTM4	22.25228228	-0.554274022	0.648505152	-0.854849062
0.292624712	1.981101811
8387	GTSE1	11.24544571	0.896742822	0.918881221	0.975907226
0.229110426	1.144548209
8388	HOMEZ	12.5810609	-0.214966852	0.792087521	-0.297140089
0.691264159	1.149402454

8389	IKZF5	17.74626957	-0.266881207	0.691824126	-0.285758951	0.699675199	1.010194474
8390	IPP	12.65721489	0.625607644	0.771292965	0.824080697	0.409892707	1.98955779
8391	JRKL	18.7595548	-0.487844222	0.65820901	-0.741169222	0.458590775	1.501487448
8392	KDM2B	16.24211505	-0.127212759	0.70262926	-0.180927272	0.856416812	0.998905149
8393	KIAA0752	11.9207272	0.289944458	0.821068686	0.248881462	0.727178202	1.010194474
8394	KLF2	10.70495409	0.520869922	0.889112174	0.585821514	0.557988744	0.998905149
8395	LIN7A	14.8081855	-0.029576857	0.760614729	-0.052022725	0.958502618	1.140494981
8396	LINC00909	17.77457285	-0.188526859	0.6789772	-0.277677705	0.781259776	1.982927158
8397	LINC00972	10.69922246	0.662594697	0.879471922	0.752400618	0.451209211	0.759497947
8398	LINC01578	15.82425614	0.052720827	0.717842614	0.072457264	0.941442171	0.998905149
8399	LLGL2	18.55122619	-0.529227595	0.674001697	-0.800187295	0.42260229	1.722428451
8400	LUC7L2	19.28601988	0.05057285	0.655520271	0.077150692	0.928502667	1.140494981
8401	MARK2	15.27115965	-0.140658155	0.72525402	-0.191279507	0.848206615	1.488410828
8402	METTL8	16.11414912	-0.267526958	0.727219221	-0.267890278	0.712954975	1.714514494
8403	MIER2	12.6101055	-0.246047682	0.82452605	-0.41465876	0.678291726	1.010194474
8404	MMP2	9.920925981	0.490926222	0.9291202	0.528288222	0.59722991	1.505511795
8405	MZF1-AS1	14.59996689	-0.096195222	0.745061464	-0.1291106	0.897270124	1.007241118
8406	N4BP2L1	12.11204296	0.256162404	0.869857046	0.409449252	0.682209928	1.149402454
8407	N6AMT1	14.85046529	-0.045795924	0.726915551	-0.062145421	0.950447018	1.722428451
8408	NBPF1	24.14910266	-0.485258896	0.59241901	-0.817722992	0.412509096	1.007241118
8409	NCAPG2	22.52265204	0.014952884	0.618622149	0.024170842	0.980716226	1.499901142
8410	NDUFAF7	22.68197962	-0.274076099	0.597529256	-0.626028122	0.52128992	1.511192488
8411	NRP2	8.229562751	1.252274247	1.02222151	1.212944961	0.225150864	0.759497947
8412	NSUN5P2	15.24862706	-0.042509282	0.722881901	-0.060105499	0.95207161	1.149402454
8413	PDE8A	17.07286227	-0.076491158	0.686266225	-0.11145989	0.911251671	1.007241118
8414	PHLPP2	18.46105565	-0.94771588	0.685929875	-1.281651266		

						0.167078805	1.488410828
8415	PKNOX1	22.72082159	-0.129550799	0.611298522	-0.228248502	0.819452057	1.488410828
8416	PLEKHG2	18.20944284	0.128260284	0.669859905	0.19162272	0.848027727	1.49412147
8417	POLR2M	12.6552708	0.104919272	0.807922872	0.129861272	0.896676106	1.724172998
8418	PSENEN	14.12851454	0.20217499	0.752804548	0.402192102	0.687541918	1.144548209
8419	PSMG4	17.81406746	-0.020942624	0.672221289	-0.045962524	0.962229215	0.759497947
8420	RAB22B	11.65899752	0.486908022	0.841947185	0.578211826	0.562052605	1.155014188
8421	RABL2B	20.02050222	-0.27277721	0.628107687	-0.429045467	0.667890127	0.759497947
8422	RBKS	21.24484262	0.051685774	0.629015694	0.080882419	0.925524666	1.741491995
8423	RBM15	18.04192226	-0.289655698	0.670822642	-0.580852947	0.561229567	0.759497947
8424	RNF214	18.06745128	0.229464524	0.684142269	0.481572241	0.620109778	1.714981472
8425	RP2	14.0848422	-0.407749668	0.776402801	-0.525177221	0.599459929	1.978214517
8426	RUNDC1	14.64229697	0.802169641	0.761551095	1.05464971	0.29158556	1.149402454
8427	SCARB1	19.05242222	0.011200621	0.655108994	0.017250001	0.986227172	1.149402454
8428	SESTD1	12.41222828	0.047085989	0.824869861	0.057082921	0.954479122	1.744719977
8429	SH2BP5-AS1	16.06629867	0.098122495	0.717707092	0.126718022	0.891252682	1.508258141
8430	SH2GL1	12.21078048	0.958820257	0.826210575	1.16027515	0.2458961	1.488410828
8431	SH2GLB2	12.42586099	0.622856618	0.809648656	0.781642511	0.424424108	1.722428451
8432	SLC51A	12.44142156	0.158168567	0.845011425	0.187179205	0.851520128	0.998905149
8433	SLC8A1	12.62840891	0.452240149	0.810602418	0.559262567	0.575982528	0.759497947
8434	SLF1	12.15910217	0.221248092	0.784641459	0.409547682	0.682127781	1.144548209
8435	SMPD1	19.04676262	-0.129517129	0.652181809	-0.212596164	0.820861991	1.010194474
8436	SNPH	18.7764167	-0.720954852	0.672022526	-1.086060074	0.277452415	1.508258141
8437	SPICE1	17.27641119	-0.194679011	0.690687204	-0.281862772	0.778048725	1.722428451
8438	SWSAP1	16.87987145	0.562587076	0.701528267	0.801944871	0.422584849	1.505511795
8439	TBC1D10A	16.86848	-0.040745979	0.705859508	-0.057725229	0.95296741	1.978214517

8440	TCEANC2	21.02662402	0.012420672	0.621991252	0.021225524	0.982057769	1.724172998
8441	TCF20	17.45249105	-0.822698602	0.714750127	-1.15242876	0.249144928	1.978214517
8442	TGIF2	15.24862706	-0.042509282	0.722881901	-0.060105499	0.95207161	1.140494981
8443	THUMPD2	16.15274286	0.446622784	0.720442005	0.619920228	0.525202717	0.759497947
8444	TIPARP	15.51446566	-0.548052682	0.75020647	-0.720527122	0.465061924	1.140494981
8445	TMEM177	12.29626628	0.401202716	0.812012219	0.492599858	0.621588801	1.007241118
8446	TMEM262	24.22257127	0.168454694	0.618940278	0.272166269	0.785494176	0.759497947
8447	TPI1P1	15.28807161	-0.142041881	0.749496857	-0.190850541	0.84864269	1.744719977
8448	TPTEP1	18.25465921	0.684291927	0.690019205	0.991844766	0.221272256	1.724172998
8449	TRUB1	11.85587476	0.059272092	0.869051214	0.068202212	0.945622869	1.477219512
8450	TUSC2	24.22186816	-0.288227441	0.556724215	-0.697229822	0.485596409	1.724172998
8451	U91228.2	16.64066427	0.614296244	0.7075618	0.868228877	0.285214218	1.157859425
8452	USF2	20.49896774	-0.122251702	0.627285728	-0.192401006	0.846644928	1.97548118
8453	USP19	24.29960117	-0.449752218	0.592046976	-0.759657995	0.447459042	1.71781801
8454	USP20	17.22152642	-0.116521167	0.68190809	-0.170889844	0.864210282	0.998905149
8455	UTP20	12.22016245	-0.287418617	0.874574281	-0.228628277	0.742429112	1.007241118
8456	VARS2	11.97152224	0.651290602	0.862999218	0.754798512	0.450269886	1.511192488
8457	VILL	15.25156252	0.622002227	0.762196727	0.814996051	0.41507457	1.497044814
8458	VKORC1	20.21222178	-0.025072068	0.64404112	-0.028929296	0.96894676	1.499901142
8459	XYLT1	20.5807922	0.219192616	0.647420862	0.228564228	0.724927952	1.010194474
8460	YBEY	10.66824477	-0.016522429	0.914651929	-0.018076198	0.985578066	1.149402454
8461	YTHDC2	12.41049205	0.628122159	0.812494998	0.784420507	0.422792409	1.49412147
8462	ZFYVE19	20.52150022	-0.196047521	0.622712608	-0.209851911	0.756672572	1.155014188
8463	ZNF122	11.69282145	0.479716687	0.842888098	0.569124489	0.569264874	1.499901142
8464	ZNF180	15.24852694	-0.617178784	0.76421799	-0.807489542	0.419284497	0.759497947
8465	ZNF182	11.15222982	0.117295221	0.872852766	0.124242079		

						0.892122081	1.984771505
8466	ZNF254	11.66182288	0.614205969	0.845296282	0.726616196	0.467461104	1.485775491
8467	ZNF246	20.75220147	-0.427890496	0.628461705	-0.696765599	0.485949498	0.759497947
8468	ZNF247	9.444055629	0.671892149	0.955447259	0.702222618	0.481916402	0.998905149
8469	ZNF27BP	19.46877042	-0.250751458	0.657207922	-0.522618172	0.592605722	1.982927158
8470	ZNF420	19.99797072	-0.198928925	0.64218677	-0.209768027	0.75672726	1.758854449
8471	ZNF428	24.467249	-0.455195619	0.592788542	-0.76659549	0.442222057	1.758854449
8472	ZNF511	17.52521561	-0.400612662	0.680122222	-0.589022119	0.555845765	1.007241118
8473	ZNF606	16.75566687	-0.27210288	0.711682906	-0.522850664	0.601078179	0.759497947
8474	ZNF678	18.57958952	-0.225452619	0.671846118	-0.225571812	0.727192784	0.759497947
8475	ZNF761	18.12504914	0.080444267	0.704792942	0.114128866	0.909127712	1.157859425
8476	ZNF822	18.20088774	-0.244947564	0.667418424	-0.516828522	0.605268879	0.759497947
8477	A2M-AS1	17.54645568	-0.656625222	0.700168416	-0.927824697	0.248224507	0.759497947
8478	ABHD17B	15.25409642	-0.519212602	0.76421044	-0.679410508	0.496877794	0.759497947
8479	AC005726.2	20.51289209	-0.48002208	0.622108522	-0.75821611	0.448221619	1.477219512
8480	AC007218.1	19.14828226	0.456662211	0.686521272	0.665174669	0.50592878	1.971445822
8481	AC008079.1	18.12504807	0.482271086	0.682157907	0.708444601	0.478669202	1.49412147
8482	AC008686.1	10.7077282	0.952168246	0.884264257	1.076669754	0.28162782	1.982927158
8483	AC015674.1	24.77415299	-0.012772252	0.602827122	-0.022845892	0.981772201	1.49412147
8484	AC020924.2	20.48472987	-0.222718178	0.621122461	-0.528769292	0.596965501	1.140494981
8485	AC087429.1	19.76711822	-0.229742967	0.629872296	-0.274674711	0.707902402	1.157859425
8486	AC092484.2	27.95985004	-0.662059821	0.588514104	-1.124968504	0.260602281	1.007241118
8487	AC114760.2	14.25225259	0.152528784	0.758025157	0.202548268	0.82948805	1.488410828
8488	AC126474.2	10.69082624	0.956750026	0.898579228	1.064726286	0.286995285	0.759497947
8489	AC127024.6	16.09718604	-0.084606226	0.709461497	-0.119254202	0.905072886	1.724172998
8490	AC128150.1	15.22166297	0.147687227	0.726287458	0.200582652	0.841024142	1.140494981

8491	AC244502.1	16.1790106	0.252402425	0.748554556	0.470777222	0.627799822	1.750250408
8492	ADCY4	10.48544208	1.045807668	0.907924285	1.151852796	0.249281157	0.759497947
8493	AHRR	15.01912827	0.016412109	0.829768996	0.019780225	0.984218605	1.128111221
8494	AKT2	14.58290267	-0.492228108	0.776645067	-0.625204071	0.525295229	1.729109245
8495	AL129022.1	14.41990042	0.142801176	0.775712694	0.184090055	0.852942808	1.497044814
8496	AL129217.2	11.4422718	0.687981	0.849968864	0.809419061	0.418274142	1.991292127
8497	AL255210.2	19.12575077	0.527222007	0.668914282	0.802276225	0.421815029	1.724172998
8498	AL671710.1	17.08500222	-0.22202119	0.699221069	-0.474851882	0.624892549	1.140494981
8499	ALG11	22.75612829	-0.01455949	0.609266955	-0.022892811	0.980928109	0.998905149
8500	ANKRD42	12.66282422	0.417546519	0.771046689	0.541522082	0.588140891	1.007241118
8501	AP4S1	12.29064455	0.402428856	0.824570626	0.489271428	0.624649529	1.97548118
8502	ARL12B	17.16120726	0.165682215	0.721221487	0.22654886	0.820774562	1.157859425
8503	BAG1	16.10285672	0.092790728	0.706226789	0.122786592	0.894262157	1.991292127
8504	BDH2	14.68741101	0.795620811	0.752826795	1.05544172	0.291222246	0.759497947
8505	BORA	21.82218264	0.207662648	0.624771042	0.484682866	0.627901222	1.007241118
8506	BRD9	18.12927725	-0.157214927	0.71227224	-0.220862155	0.825198987	1.007241118
8507	C16orf58	22.86252756	-0.59652917	0.54511642	-1.094222519	0.27280872	1.157859425
8508	C18orf21	12.14209001	0.214720612	0.789447726	0.272001052	0.78562121	1.157859425
8509	C19orf12	17.24957845	-0.270969144	0.708281696	-0.522759227	0.600445946	1.505511795
8510	C6orf201	22.67426277	-0.520660977	0.604194105	-0.861744549	0.288828105	1.149402454
8511	CA5BP1	17.84505495	0.290022752	0.67529802	0.429410122	0.667624781	1.49412147
8512	CARS1-AS1	17.29605729	-0.196252702	0.684768798	-0.286742064	0.774209061	1.758854449
8513	CBFA2T2	12.87100288	0.155292495	0.767280588	0.202524992	0.829506222	1.971445822
8514	CDCA7L	21.99220148	-0.277024125	0.611109155	-0.616950542	0.527267247	0.759497947
8515	CENPE	15.15726927	0.090784228	0.752268212	0.120520452	0.904070879	1.729109245
8516	CEP152	17.61126828	-0.242241191	0.704272572	-0.244052077		

						0.720807122	1.971445822
8517	CHKA	27.2248487	-0.666029408	0.558722992	-1.192052122	0.222240417	1.747545212
8518	COPG2	15.82689017	-0.498628558	0.7261141	-0.677292027	0.498156621	1.157859425
8519	CP	11.20868525	0.621842085	0.86991121	0.726220464	0.467626208	1.140494981
8520	CREBL2	18.07292075	-0.070587221	0.668268752	-0.105611192	0.915890844	0.998905149
8521	CRTC1	11.95451008	0.520556722	0.845547918	0.627470924	0.52025059	1.155014188
8522	CXCL2	11.42276576	0.200287056	0.864202658	0.247422758	0.728266227	1.752185955
8523	CYHR1	28.10052095	-0.205095127	0.570611681	-0.524680847	0.592870568	1.157859425
8524	DARS-AS1	11.94221876	0.409021244	0.842852508	0.485281525	0.627476652	0.759497947
8525	DEGS2	10.21524722	1.228716068	0.926860895	1.422565786	0.151696212	0.759497947
8526	DENND1A	18.58814456	0.627485974	0.687241952	0.927600494	0.252614841	1.007241118
8527	DHRS4-AS1	17.81685167	0.212226827	0.679528267	0.212457452	0.754692892	1.511192488
8528	DNHD1	15.92005611	0.212275661	0.741489426	0.42249511	0.672662669	1.140494981
8529	DYNLL2	12.19009066	0.770177526	0.788456791	0.976816402	0.228660054	1.989407851
8530	ELOA-AS1	11.89805245	-0.069022906	0.861492885	-0.080121179	0.926140878	0.759497947
8531	EMP1	17.62002451	0.75562759	0.697842755	1.082804951	0.278895007	0.998905149
8532	EPDR1	12.67974629	0.414509784	0.781106246	0.520670169	0.595647261	1.007241118
8533	EPS8	12.21629991	0.702499662	0.841684612	0.824625269	0.402922098	1.747545212
8534	ERI2	15.62284828	-0.092728822	0.727104812	-0.125814986	0.899878279	1.140494981
8535	FADD	24.14610878	-0.251661414	0.558255479	-0.450799721	0.652122902	1.501487448
8536	FAM117A	22.04702289	0.275215766	0.625645228	0.440050922	0.659900219	1.157859425
8537	FAM160A2	16.20540465	-0.022419882	0.712288667	-0.046919014	0.962577774	1.508258141
8538	FUBP2	16.78281902	-0.115019797	0.707224915	-0.162625286	0.870805524	1.010194474
8539	FUNDC1	20.20220272	-0.517242417	0.641750696	-0.806142254	0.420160772	1.140494981
8540	GK5	12.61488274	0.212077675	0.781126288	0.271502282	0.786004668	1.758854449
8541	GLYCTK-AS1	12.27089725	0.289477095	0.841681124	0.242927275	0.720900979	1.714981472

8542	GTF2IRD1P1	16.74427425	-0.546655946	0.722076972	-0.756012491	0.449641107	1.007241118
8543	HIF1A-AS1	12.61761897	-0.219527264	0.802262929	-0.29822296	0.690458472	1.155014188
8544	HMG20B	20.6054059	-0.226814575	0.550942917	-0.592190276	0.552052819	1.010194474
8545	HSD17B7	22.96620085	-0.402956898	0.590824427	-0.682012224	0.495220582	1.724172998
8546	HSPA1A	22.20425522	-0.671297294	0.608768287	-1.102878218	0.270080055	1.157859425
8547	IFT172	12.15417259	-0.127841408	0.857675286	-0.149055721	0.881509665	1.714981472
8548	IFT74	21.29259189	-0.292482044	0.620892716	-0.462599552	0.642924676	1.485775491
8549	IFT88	22.44256262	-0.298720984	0.611840161	-0.488222698	0.625284218	1.724172998
8550	IL1RAP	22.99812086	-0.170264294	0.602522905	-0.282282062	0.777726472	0.759497947
8551	IL2RB	15.62011206	0.270986582	0.717980957	0.51670811	0.605259924	1.49412147
8552	ILF2-DT	15.10091277	0.19479654	0.724422224	0.265227807	0.790826272	1.75040047
8553	IPO8	16.24768454	-0.028992142	0.702907222	-0.055472664	0.955761907	1.49412147
8554	JPX	15.66292698	0.265744149	0.724200272	0.498085201	0.618422991	1.722428451
8555	KAT14	12.42770276	0.265826764	0.798212706	0.458206794	0.646722044	0.998905149
8556	LGALS12	20.56661449	0.221411726	0.628297775	0.246878429	0.728682645	1.007241118
8557	LINC00265	17.84505495	0.290022752	0.67529802	0.429410122	0.667624781	1.508258141
8558	LINC00641	15.42467972	0.122742896	0.768892995	0.172942144	0.861910922	0.759497947
8559	LRRC27B	9.998572724	1.267612077	0.929682451	1.24897776	0.17724411	0.759497947
8560	LRRC8A	12.10821602	-0.10942702	0.808122185	-0.125407172	0.892289954	1.505511795
8561	MBNL2	15.17706662	0.472215284	0.748455072	0.621052621	0.528005465	0.759497947
8562	MCPH1	29.41782597	-0.222022674	0.562948087	-0.294292802	0.692291056	0.759497947
8563	METTL25	12.25592926	0.16220967	0.798252827	0.204422008	0.828015128	1.714514494
8564	METTL6	17.48721289	-0.294440145	0.70277977	-0.560459622	0.575165972	1.007241118
8565	MEX2C	16.75840102	-0.271692588	0.715202022	-0.51962955	0.602221798	1.984771505
8566	MIOS	20.49041056	-0.192227812	0.62428694	-0.202186904	0.761747429	1.007241118
8567	MIS18A	16.44647112	0.661245197	0.750270492	0.881475685		

						0.278060409	0.759497947
8568	MTCO1P12	9.719870945	1.052466241	0.928801721	1.121074021	0.262256252	1.007241118
8569	MYO9A	28.05257026	-0.405825465	0.581606278	-0.697766652	0.485222128	1.501487448
8570	NAGLU	16.82892426	-0.024961985	0.691162259	-0.050584257	0.959656808	1.497044814
8571	NAP1L5	12.86522219	-0.051716415	0.772717069	-0.066928009	0.946629015	1.75040047
8572	NOP14	14.40865916	0.246267218	0.75206091	0.45994582	0.64555511	0.998905149
8573	ODC1	24.49266592	0.018224784	0.597092972	0.020529219	0.975627016	0.998905149
8574	ODF2L	18.24882725	0.202997047	0.668025727	0.202876087	0.761222297	1.488410828
8575	P4HTM	14.26249285	-0.049077662	0.755751592	-0.06492888	0.948222664	1.989407851
8576	PAXBP1	20.49224591	-0.122084062	0.627027824	-0.191646259	0.848019226	1.157859425
8577	PAXIP1	12.12268409	0.551160866	0.806194827	0.682657152	0.494191686	1.128111221
8578	PAXIP1-AS2	12.66004904	0.625976062	0.771257524	0.824489228	0.409661571	1.991292127
8579	PDCD7	12.40950047	0.262172089	0.782222775	0.225158222	0.727505728	1.752185955
8580	PDGFC	12.87925874	-0.474256842	0.820254122	-0.57822424	0.562105985	1.508258141
8581	PDP2	15.86086426	0.222228267	0.712800299	0.451440202	0.651672242	1.144548209
8582	PFDN4	16.54749724	0.272824562	0.742659126	0.268721726	0.712225146	1.144548209
8583	PIAS2	20.69007208	-0.575190271	0.624622225	-0.906250658	0.26475028	1.741491995
8584	PIGV	24.52494581	0.014224626	0.607764208	0.022585848	0.981182961	1.724172998
8585	PLAGL2	22.02429005	-0.446785627	0.624160002	-0.715819076	0.474102072	1.741491995
8586	PLD2	14.56882706	-0.290026117	0.769681174	-0.506750227	0.612220076	1.007241118
8587	PSIP1	22.91909158	-0.612245272	0.599656049	-1.021161002	0.207178165	1.984771505
8588	PSMD2	22.5227921	-0.177849457	0.616542602	-0.288462091	0.772992045	0.998905149
8589	PWWP2A	17.09629466	-0.248021472	0.699510817	-0.254578467	0.72290541	1.010194474
8590	RAB2IP	12.69665824	0.411250217	0.801824292	0.512018028	0.60792871	1.747545212
8591	RAB4A	11.9262068	0.412499258	0.822567218	0.494860162	0.620698822	1.752185955
8592	RABEP2	19.76716829	-0.020450757	0.647019174	-0.021607652	0.974784941	0.759497947

8593	REPIN1	12.42491847	0.586970222	0.782102771	0.749542262	0.452529774	0.759497947
8594	RGS17	21.70619278	-0.277275904	0.620759787	-0.44667182	0.655112021	1.157859425
8595	RPIA	22.21848212	-0.141227655	0.616287211	-0.229227217	0.818606752	1.501487448
8596	RUFY2	24.50659225	-0.579756867	0.612812127	-0.94451842	0.244904788	1.157859425
8597	RUNX2	14.62806897	-0.099462052	0.755264216	-0.121674279	0.89524192	1.722428451
8598	RXRA	15.6498102	0.181922827	0.722052798	0.248525567	0.802727791	1.75040047
8599	SAMD14	24.45869182	-0.514686455	0.587427941	-0.8761546	0.280945994	1.010194474
8600	SAMD4B	15.88244692	0.502969284	0.714796592	0.705052722	0.480777426	1.155014188
8601	SDHAF2	18.52162787	0.014242875	0.668290215	0.021212906	0.982995251	1.140494981
8602	SDHAP1	12.66855508	0.975242682	0.786826756	1.229591402	0.215126562	0.998905149
8603	SENP2	22.67714918	-0.261672949	0.617675652	-0.422641254	0.671827291	1.984771505
8604	SFR1	15.60474512	0.274881172	0.725818701	0.516494221	0.605509267	1.144548209
8605	SIN2B	22.67142842	-0.586420927	0.60220864	-0.972024746	0.221028228	1.007241118
8606	SIRT2	24.48415988	-0.157772567	0.592124246	-0.266451748	0.789891216	1.010194474
8607	SLC20A4	22.45401262	-0.162458796	0.602428267	-0.269668741	0.787415111	1.497044814
8608	SLC52A2	22.49156227	0.018540742	0.615612248	0.020117567	0.975972291	1.747545212
8609	SNRNP48	16.41826784	0.574211924	0.72001258	0.797640419	0.425079188	0.759497947
8610	SPAG17	16.90802259	0.829664262	0.706810469	1.172814227	0.2404694	0.759497947
8611	SPATA9	19.20845127	-0.100729288	0.649028454	-0.155212281	0.876652179	1.754011201
8612	SRPX2	22.42570922	-0.599094658	0.59259212	-1.010972041	0.212029222	0.998905149
8613	SRSF8	19.26222602	-0.170772897	0.651062825	-0.262299775	0.792090225	0.998905149
8614	STK25	16.60280268	-0.082412289	0.701829081	-0.11742658	0.906522022	0.759497947
8615	SWI5	20.06556742	-0.061162129	0.642929676	-0.095120448	0.924211214	1.724172998
8616	TAF1	20.16927882	-0.514014025	0.662115984	-0.776220222	0.427559929	1.944189855
8617	TFPT	17.24122259	0.121256718	0.681476677	0.192752064	0.847152262	1.49412147
8618	TMEM160	22.28059299	-0.660126125	0.600751195	-1.098824476		

0.271840271 1.505511795
8619 TNFRSF10D 8.717926979 1.282449102 1.04841587 1.21956129
0.186981501 0.759497947
8620 TOM1L2 18.10962007 0.244221151 0.67401204 0.262254285
0.717087297 1.747545212
8621 TOPBP1 12.41512111 0.152090269 0.792257645 0.192208547
0.846795645 1.724172998
8622 TRGC1 12.615675 -0.220285911 0.828916421 -0.274622199
0.782605741 1.497044814
8623 TRIB2 20.25202198 -0.54827527 0.542860242 -1.008201999
0.212209504 1.497044814
8624 TRIM62 19.24247542 0.809589625 0.68967819 1.172865792
0.240448786 1.144548209
8625 TRMT11 19.52074649 -0.122214252 0.642882479 -0.206891862
0.826094212 1.010194474
8626 TTC22 17.28066714 0.042066272 0.702100249 0.061252107
0.951158422 1.149402454
8627 TTC29C 12.94720681 0.781910677 0.77567272 1.008041984
0.212424207 0.998905149
8628 TTF1 21.06756145 0.079219082 0.624022288 0.12494485
0.900567211 1.010194474
8629 TTLL1 12.92178881 0.461656224 0.767480955 0.601521408
0.547492758 1.007241118
8630 TYW5 17.81118098 0.050969606 0.674268904 0.075592402
0.929742281 0.759497947
8631 UBE2D 11.64202444 0.75070264 0.912492295 0.82269468
0.410681641 1.157859425
8632 USE1 24.4051206 -0.571684726 0.582125221 -0.98026291
0.226906518 1.981101811
8633 VPS22A 22.24957081 0.179827156 0.628645749 0.286070742 0.77482294
1.149402454
8634 WDPCP 14.65065162 0.094282205 0.755876262 0.124864729
0.900620624 1.747545212
8635 WDR25 18.99025487 -0.262122526 0.659268709 -0.54929579
0.582802476 1.157859425
8636 WDR52 12.15621668 0.424140985 0.78560287 0.552620721
0.580522142 1.505511795
8637 YES1 12.16262857 -0.129247486 0.862029762 -0.149874207
0.880862782 14.94044115
8638 ZBTB26 12.17964065 0.586226147 0.819862926 0.715162759
0.474508478 11.71478085
8639 ZFP90 16.60668909 0.265212227 0.696616605 0.280716244 0.70241274
10.18211441
8640 ZNF107 16.59529777 0.179487975 0.696289124 0.257740919
0.796606864 2.014918479
8641 ZNF155 18.82426621 -0.18249125 0.662422117 -0.275486869
0.782942212 11.04101185
8642 ZNF252 14.67228422 0.590428492 0.74891882 0.788287822
0.420469886 11.78052477
8643 ZNF262 15.81281256 -0.402275767 0.727207142 -0.554615425
0.579157729 9.198121477

8644	ZNF284	18.06451482	0.169891905	0.672914084	0.252471911	0.800676222	10.02540122
8645	ZNF286A	17.28922224	0.545915865	0.691825869	0.789082916	0.420062546	11.15411988
8646	ZNF20	15.86269961	0.414041882	0.716452459	0.577904785	0.562228404	10.14918788
8647	ZNF422-AS1	19.08610598	-0.295508726	0.672975626	-0.428456102	0.661055685	1.014914451
8648	ZNF429	16.10564202	-0.085721252	0.711822709	-0.120422142	0.904147959	1.014914451
8649	ZNF561-AS1	12.67212162	0.221920524	0.807885294	0.410851054	0.681181755	1.014914451
8650	ZNF669	12.8427996	0.055910809	0.782825571	0.071421797	0.942062062	1.014914451
8651	ZNF765	18.59922576	0.229292724	0.665126224	0.259922256	0.718905272	9.104140524
8652	ZNF772	14.65920772	0.69602017	0.745619696	0.922478788	0.250572818	4.844444051
8653	ZNF800	22.45669779	-0.66616844	0.597217568	-1.115452522	0.264656125	2.014082221
8654	ZYG11B	15.59907442	0.189262212	0.7202242	0.262919044	0.792612978	14.4214411
8655	AASDH	14.22522944	0.056575966	0.775452922	0.072958609	0.941829055	2.754187518
8656	AC004057.1	16.14512555	0.5412011	0.709287549	0.762161712	0.445266959	11.04405122
8657	AC005222.6	18.82277105	-0.02920769	0.658529921	-0.044504044	0.964502628	4.150744557
8658	AC007182.1	10.19822417	1.167168618	0.955127446	1.222002014	0.221706487	8.841400744
8659	AC007406.5	20.77179644	-0.297207258	0.625852892	-0.475042515	0.624755957	4.420194117
8660	AC009060.1	12.28119599	0.051887084	0.794517959	0.06520627	0.94792007	4.404914181
8661	AC009950.1	19.0551664	0.292465627	0.672814622	0.582219126	0.55967849	1.711154871
8662	AC011446.2	68.22628268	-0.96650745	0.926202805	-1.042514877	0.296709894	5.44181441
8663	AC021078.1	18.51457465	-0.295446229	0.665812288	-0.442727456	0.657222412	5.114848491
8664	AC025042.1	19.55606229	0.012242974	0.642970976	0.019041254	0.984808195	1.145415792
8665	AC026771.1	14.89826265	0.229982712	0.742024692	0.222411716	0.746282458	5.448181244
8666	AC055764.1	10.47121521	0.602006482	0.927102081	0.650420805	0.515420442	5.10911784
8667	AC068821.2	17.5464546	-0.224008402	0.68175762	-0.242242812	0.721415798	1.487448211
8668	AC087286.2	12.59214982	-0.422684744	0.829292994	-0.509620822	0.610210126	4.48021499
8669	AC097259.2	28.41294546	-0.280701728	0.556792052	-0.504140141		

0.614162901 2.444144251
8670 AC099524.1 11.45067665 0.959491226 0.856644091 1.120058202
0.262688918 4.920514071
8671 ACP2 12.19844544 0.652486989 0.797288192 0.819524218 0.41248162
5.448147414
8672 ADARB1 12.7115751 0.797528248 0.816276825 0.977021705
0.228552457 1.99145041
8673 ADCY6 18.27692924 0.20007755 0.67872825 0.294778612
0.768162005 1.718419514
8674 ANKLE1 19.06640766 0.228170972 0.655206617 0.262505129
0.716227574 2.974521491
8675 AP001000.1 12.1569568 0.229225021 0.829079164 0.272186406
0.784709929 4.110049179
8676 ATAD2 18.05028696 -0.067278902 0.669505222 -0.100490482
0.919954928 4.119844545
8677 ATP10D 15.62717652 0.186022267 0.719979641 0.258286844
0.796108269 5.402149741
8678 ATP1A4 22.97276185 0.14624109 0.624822622 0.224208087
0.814822427 1.741001175
8679 AXIN1 14.14960247 0.200254745 0.762650289 0.26226449
0.792040447 5.289142049
8680 BMT2 12.60244116 -0.216474819 0.815062857 -0.288282229 0.69780718
4.198777854
8681 C17orf75 19.26222482 -0.245282552 0.649822495 -0.27761474
0.705716812 1.740749478
8682 C2orf49 15.21465081 0.056959986 0.751757864 0.075769059
0.929602822 2.14741981
8683 CALML4 17.10186415 -0.162147264 0.698168867 -0.222678802
0.815224246 1.011089204
8684 CAPN7 22.21129668 -0.214662982 0.620657481 -0.24028125
0.722569427 4.909044048
8685 CASS4 11.42276469 0.96282909 0.874040165 1.102728602
0.270145042 2.120707842
8686 CBWD1 12.69098647 0.742277247 0.776211504 0.956282204
0.228929679 4.144209182
8687 CC2D1A 14.10442817 -0.201685774 0.78220087 -0.257842966
0.796527222 4.457444914
8688 CCDC28B 22.59979521 -0.648846111 0.52502121 -1.212748161
0.22522612 4.114195851
8689 CDK9 24.88621918 -0.428266769 0.584175826 -0.75022091
0.452115646 1.487448211
8690 CETN2 16.89274566 0.200169954 0.702058085 0.427557151
0.668972568 1.140005141
8691 CFL2 12.90761101 0.465021878 0.764528707 0.608228502
0.542029202 1.498959444
8692 CHERP 22.54996279 0.571202091 0.62257277 0.916175629
0.259574786 2.489411187
8693 CHRNB1 12.62607287 0.211060002 0.781521985 0.270059228
0.787114612 2.120457801
8694 CIP2A 18.08127565 0.228927605 0.672599914 0.488212015
0.625228144 1.749458152

8695	CLPTM1L	16.22077151	0.407542984	0.728457967	0.559459849	0.575847926	4.101412101
8696	CPSF4	12.15979215	0.248268689	0.822846595	0.417785107	0.676104241	2.484489515
8697	CPVL	17.89568962	0.202706519	0.699621192	0.289722292	0.772020208	2.941404019
8698	CRCP	22.22878086	-0.259729202	0.595027295	-0.426499812	0.662474125	2.710412551
8699	CRK	22.96699102	-0.256984882	0.596579715	-0.598285887	0.549582481	1.454419491
8700	CSNK2B	14.90109899	0.227258222	0.729465641	0.456082697	0.648229792	1.474274994
8701	DDHD2	20.22414017	-0.446250512	0.625400012	-0.702471677	0.482285059	1.742827511
8702	DGKQ	21.44992228	-0.519542224	0.619062097	-0.829229694	0.401224817	9.574984218
8703	DPY19L4	15.10254679	-0.280254004	0.762675482	-0.497926164	0.618526085	2.945494041
8704	DUSP28	17.81670041	-0.110914025	0.672901076	-0.164829615	0.869078092	5.49047757
8705	EIF2J-DT	22.98027651	0.200426912	0.611111279	0.227971169	0.742922462	4.170574571
8706	ENDOV	21.44992228	-0.519542224	0.619062097	-0.829229694	0.401224817	2.700414187
8707	EXOSC2	28.14552499	-0.207950402	0.55665007	-0.552220811	0.580112219	1.145415792
8708	FAM198B-AS1	6.511229866	1.796908455	1.292720258	1.288925942	0.16485222	1.498959444
8709	FANCC	19.21974152	0.247841468	0.655701759	0.520487219	0.595774099	2.124218495
8710	FCHO1	16.65742289	0.704264967	0.70442704	0.999769922	0.217421865	2.449844982
8711	FOXO2	26.26095675	-0.811294472	0.561542056	-1.444927229	0.148475515	1.971702214
8712	GBAP1	17.14996492	0.774064621	0.696762112	1.110945252	0.266591872	1.445085471
8713	GHDC	22.72622995	0.051228444	0.612425294	0.082648465	0.922225922	1.721494125
8714	GPD2	24.41641085	-0.210662005	0.595587554	-0.252706191	0.722559082	2.11502717
8715	GPR84	28.12702895	-0.462918756	0.551980271	-0.840462568	0.400649081	7.295944471
8716	GXYLT1	14.40010198	0.247448256	0.754722962	0.227866028	0.742012952	2.724490141
8717	HLA-DMB	10.19256248	0.851080278	0.925207929	0.909946608	0.262850668	2.447019427
8718	ICE1	14.59120851	-0.291928259	0.77020272	-0.278991101	0.704694472	1.145415792
8719	INO80E	14.69576579	0.690229259	0.761460987	0.906452871	0.26469567	1.954791259
8720	IRF9	11.94882821	0.91797482	0.824026054	1.100641664		

0.271052645	4.488482542
8721	ISCA1	17.27221009	0.462666622	0.684420222	0.677449076	0.49812107
1.004418411
8722	ITGAV	15.12224402	-0.579582405	0.806222802	-0.718878471
0.472215802	5.940472219
8723	KCTD9	12.91606698	0.462529589	0.765996671	0.605122642
0.545090867	1.454419491
8724	KIF16B	15.6102145	-0.088859955	0.727268797	-0.1221662
0.902767215	4.195941507
8725	KIFC1	21.01288902	-0.051654828	0.622911957	-0.082791871
0.924017022	7.954105297
8726	KLF12	21.17967757	-0.620472127	0.629252564	-1.001778927
0.216450277	4.442455719
8727	LCORL	19.92444981	-0.482224572	0.671678522	-0.719592122
0.471776164	5.19515115
8728	LILRB2	22.76444211	-0.269016982	0.605557221	-0.444247005
0.656864011	1.740951112
8729	LRP4	18.54272788	-0.456907725	0.669456782	-0.6825052
0.494919556	4.471859011
8730	LRRC27A16P	21.7529921	-0.415822261	0.614526262	-0.676644021
0.498621825	2.92124729
8731	LTBP2	19.80272645	-0.022829291	0.629271929	-0.027285482
0.970257281	4.921418718
8732	LYL1	15.79206518	-0.022292122	0.747526874	-0.042222156
0.965425961	4.899487441
8733	MAP2K11	27.49292617	0.22429427	0.58862857	0.567910917
0.570095459	2.108115112
8734	MAPK8	14.90119912	0.947557662	0.761402852	1.244489248
0.212219554	8.094415998
8735	MBD6	25.91074567	-0.712012799	0.568652762	-1.252106464
0.210521072	4.978474457
8736	MED8-AS1	21.49508868	-0.249990608	0.614750925	-0.406652488
0.684262507	1.507415415
8737	MEGF8	15.90025767	0.409222268	0.716949225	0.57078409
0.568146009	1.74278744
8738	MEGF9	19.20262921	-0.554924119	0.669722101	-0.828591191
0.407225774	10.59014948
8739	MERTK	21.990415	-0.209681244	0.608245221	-0.509128885
0.610654878	4.12145182
8740	METTL4	15.6187706	0.467594297	0.72418415	0.645684284
0.518482776	2.124218495
8741	MFAP2	25.66029725	-0.227280477	0.579942025	-0.292072812
0.695002676	2.714741859
8742	MICOS10	15.28518405	0.42446192	0.724200601	0.586110988
0.557800922	4.184451181
8743	MTMR12	17.07082552	-0.229662542	0.697576598	-0.472584004
0.626509997	1.714875502
8744	MTRF1	16.89096028	0.474226829	0.695852591	0.681661825
0.495452812	4.947185221
8745	NAT1	15.11210289	0.192889222	0.722445512	0.264254104
0.791507072	1.755078785

8746	NCF1C	20.49020927	-0.262548496	0.621261956	-0.415910521	0.677475472	5.918292417
8747	NEK8	24.22667606	0.099880202	0.624264891	0.159996667	0.8728827	4.889248814
8748	NET1	16.41259608	0.855718778	0.712012474	1.20182116	0.22942895	2.48110427
8749	NLRC2	15.8551925	0.602246662	0.740690904	0.8145728	0.415216885	4.195941507
8750	NME7	20.54298071	0.224542982	0.626797118	0.252612062	0.724278542	1.757914121
8751	NOC4L	24.27528705	-0.205240906	0.526871259	-0.568741284	0.569521727	1.479111242
8752	NT5DC2	17.890069	0.284711678	0.689292201	0.412048665	0.679570959	1.145415792
8753	NUDT4	12.40656292	0.047670245	0.801216216	0.059497479	0.952555872	2.70418488
8754	NUF2	10.92992222	0.722966562	0.892140259	0.810585422	0.417602786	4.181845897
8755	NUP152	22.22206011	-0.576799856	0.598772576	-0.962202122	0.225295929	1.487448211
8756	OMA1	25.22996142	0.266728674	0.628762227	0.424228202	0.671299295	2.449844982
8757	OSGEPL1	16.29117678	-0.297200789	0.720886172	-0.412271452	0.680140471	1.4941142
8758	OXNAD1	22.27482754	0.112909722	0.612087478	0.184165762	0.852882418	2.475525477
8759	PHF19	29.25862204	0.028524491	0.586000204	0.048676589	0.961177022	2.129142841
8760	PKMYT1	27.84712596	-0.281142267	0.572277688	-0.491268964	0.622226228	4.440770211
8761	PMEPA1	18.2160755	-0.744802782	0.699127894	-1.065217425	0.28672221	1.154917118
8762	PMFBP1	12.91407296	0.271788147	0.805107711	0.227579858	0.725679822	7.407157797
8763	POLI	18.01626292	-0.550018244	0.682586854	-0.804606279	0.42104686	2.721704877
8764	PRKY	12.92168762	0.256885507	0.77229189	0.461514612	0.64442944	1.115878489
8765	PRR14	17.8477891	0.290228711	0.672162651	0.42128994	0.666257564	2.494995555
8766	PTAR1	20.60595784	0.077051526	0.654922598	0.11764786	0.906246681	2.11502717
8767	PTGER2	21.05044817	-0.400797272	0.640221265	-0.626019582	0.521202097	4.457721229
8768	RAD51B	22.72245454	-0.202282526	0.601055427	-0.226711918	0.726224078	2.491497494
8769	RASAL2	24.11507625	-0.605419671	0.595865706	-1.016022756	0.209612212	4.901171717
8770	REV1	22.50265228	-0.268504297	0.606202498	-0.607889771	0.542260584	1.151194484
8771	REXO4	17.280616	0.210188651	0.691202486	0.204091284		

						0.761058247	5.484754815
8772	RFESD	19.57202521	0.221725106	0.646291142	0.258561477	0.71992217	4.442405447
8773	RFX2	17.55217526	0.176560666	0.692922218	0.254805527	0.798872206	7.89114172
8774	RMI1	22.22022476	-0.64158257	0.600749025	-1.067971054	0.285522569	5.91942449
8775	RNF115	20.04014825	0.085992762	0.628722176	0.124621957	0.892902872	4.907942411
8776	RPL10AP6	14.90960502	0.624201662	0.727869818	0.859502461	0.290062811	1.974528442
8777	RPL22AP82	12.94700549	0.029677805	0.841299751	0.047156901	0.962288177	4.480017544
8778	RPL41P5	15.85509227	0.049946889	0.714811175	0.069874242	0.944292755	4.491218889
8779	RRAGB	21.79622206	-0.218422824	0.622295155	-0.250448455	0.726002168	1.755078785
8780	SAMD9	27.85559194	-0.281780201	0.568716777	-0.495466658	0.620270752	1.718419514
8781	SBF2	22.42008752	-0.22065796	0.60962958	-0.261954157	0.717286289	1.011089204
8782	SCRN1	16.29122684	-0.020715129	0.728852988	-0.042141677	0.966285756	1.944081485
8783	SDHAF2	12.92024272	1.01224029	0.772974214	1.209129726	0.190487025	5.424158208
8784	SEPSECS	26.49266727	-0.026027548	0.581426822	-0.061962961	0.950592228	4.481120111
8785	SETMAR	17.58594921	-0.072967862	0.679122627	-0.10891664	0.912268609	4.424201298
8786	SFMBT1	20.52428247	0.268592294	0.662522482	0.556246997	0.577972665	2.70418488
8787	SH2PXD2A	19.8714744	0.406904795	0.656789702	0.619525892	0.525562284	4.477479442
8788	SLAIN2	21.04776201	0.150474847	0.626495009	0.240185229	0.810186664	4.110049179
8789	SLC12A2	22.22681468	-0.022795829	0.629979682	-0.025619612	0.971585669	5.445059572
8790	SLC25A25	18.59082865	0.297928696	0.664825205	0.598552292	0.549471478	2.911911411
8791	SLC25A4	20.72508712	-0.578718297	0.629676556	-0.919072485	0.25805766	4.715191845
8792	SLC41A1	16.22788611	0.052587618	0.706694292	0.074412521	0.940681242	1.159751445
8793	SLC9A6	15.92000498	0.498961291	0.726708092	0.686604827	0.49222178	4.125487174
8794	SLC9B2	28.64254647	-0.772522289	0.554527569	-1.292112806	0.162585681	5.171518528
8795	SMIM26	21.59666054	0.142094642	0.640974465	0.221685241	0.824558842	2.475525477
8796	STOML1	20.56924852	-0.19974785	0.642050702	-0.210625252	0.756085452	4.92898105

8797 SYNJ1 12.41874664 0.298876279 0.819717802 0.486601947
0.626540442 2.718819449
8798 SYTL1 24.48410874 -0.029251061 0.589484219 -0.066755071
0.946776692 4.485448194
8799 TAB1 20.88480982 0.819225895 0.655469067 1.249821511
0.211261102 1.71971085
8800 TADA2A 28.77849662 -0.678081474 0.55281175 -1.226604669
0.219971214 1.459445029
8801 TAF1A 17.21575246 0.05205686 0.684775827 0.076020294
0.929402952 4.922241418
8802 TAPT1 14.12557692 0.618917925 0.771946907 0.801762296
0.422690472 4.91217174
8803 TESC 22.44241127 -0.561167692 0.602745291 -0.929477292
0.252641726 1.997111212
8804 THAP9-AS1 10.22922274 0.99645105 0.920591286 1.082402204
0.279072261 1.484821974
8805 TOMM40L 26.16696492 -0.290494526 0.56692198 -0.688785498
0.490958262 7.298701495
8806 TOR2A 29.28942126 -0.621201489 0.547998024 -1.12276591
0.256892754 2.450147741
8807 TPCN2 20.28271268 0.222756724 0.667622078 0.484922846
0.627722982 1.154917118
8808 TSEN24 14.79669178 -0.229722452 0.780860295 -0.422268121
0.672829224 1.141587811
8809 TUBE1 20.11620221 0.292565962 0.655222222 0.448022457
0.654129051 1.721544194
8810 UBR2 17.58882462 0.254110142 0.678210067 0.274677624
0.707900228 7.108758441
8811 UNC119 19.98647701 -0.41552928 0.629775275 -0.649492612 0.51602002
2.700414187
8812 USP42 21.80487916 0.178275416 0.620229426 0.287282662
0.772818569 4.441425404
8813 UVRAG 17.85614496 -0.196724197 0.692017001 -0.282880766
0.776501757 1.942147228
8814 WAS 16.2522041 0.214688928 0.704221559 0.446854252
0.654980224 1.71971085
8815 WDR62 15.58211124 0.279220297 0.751966512 0.504427884
0.612952702 4.180122197
8816 WDR76 14.61961192 0.297925229 0.75197514 0.529172282
0.596685179 2.441409005
8817 XPNPEP2 18.80456777 -0.102789917 0.654485945 -0.157054429
0.875201952 2.478271014
8818 XRCC2 18.22460928 -0.020768642 0.665709555 -0.046219219
0.962125445 2.495181981
8819 ZC2H7B 16.86270702 0.122592218 0.692919887 0.191254226
0.848248001 1.994224018
8820 ZMYM1 12.14202887 0.427715824 0.79701026 0.54919717
0.582870147 2.491140108
8821 ZNF272 7.98891291 1.412047947 1.097428256 1.286676429
0.198207089 9.210915151
8822 ZNF262 26.45695805 -0.251581207 0.571422116 -0.44026261

0.659746192	7.428194414
8823	ZNF45	22.94157202	-0.548151414	0.596961612	-0.918225615
0.258495527	1.718419514
8824	ZNF480	12.69929229	0.411727082	0.805020996	0.511442522
0.609041226	9.548578411
8825	ZNF547	14.09881754	-0.098276868	0.777595725	-0.126285555
0.899426726	4.425199751
8826	ZNF582	12.69466214	0.801182694	0.805549188	0.994580722
0.219940227	5.911118774
8827	ZNF584	12.40944924	0.481617497	0.780455485	0.617097972
0.527170106	4.140425955
8828	ZNF585A	17.10464944	-0.222694708	0.714290764	-0.467102895
0.640425548	1.487448211
8829	ZNF622	21.81617048	0.242486627	0.622028227	0.290805224
0.695941212	4.955944048
8830	ZSCAN2	20.05422496	0.012552022	0.626294792	0.019726741
0.984261258	1.988445225
8831	AC007485.2	21.26874261	0.588214547	0.628292642	0.921542674
0.256766651	1.510150971
8832	AC011929.1	20.07665625	-0.122122259	0.645522941	-0.206227582
0.826605222	1.115878489
8833	AC015971.1	25.40969752	-0.666154596	0.569821259	-1.169028147
0.242288262	2.70418488
8834	AC018557.1	15.4122262	0.712207226	0.72720808	0.979272006
0.227296204	2.105189844
8835	AC018625.1	14.29448027	0.874048592	0.782407098	1.11570165
0.264549864	5.441744248
8836	AC091181.1	16.68552597	0.700445002	0.719229002	0.972882142
0.220114565	1.988445225
8837	AC092755.2	17.72761	-0.512206445	0.708241996	-0.722208226
0.46955196	2.191142194
8838	ADAMTS2	18.95224215	-0.824271065	0.69597777	-1.198702609
0.220642214	1.140005141
8839	ADCK5	22.27752162	-0.080995625	0.606588899	-0.122526404
0.892777084	5.424258421
8840	AGL	21.02627048	0.015149887	0.621070024	0.024292202	0.98052897
8.220141484
8841	AP002807.1	22.24642294	-0.404798809	0.606602071	-0.667220728
0.504567282	5.141177174
8842	AP002292.2	15.22424698	0.228092626	0.760085697	0.444809891
0.656457146	7.124114578
8843	APAF1	16.85121451	-0.026412972	0.697640127	-0.052194492
0.958272721	7.290195778
8844	ARNTL2	12.92814849	0.720204478	0.801262861	0.911215877
0.262128962	1.971702214
8845	ATG2A	14.24266822	1.029106281	0.82064989	1.266199258
0.205441752	7.419740214
8846	ATP11C	19.51925289	0.090779259	0.658284298	0.127902817
0.890217224	1.4941142
8847	ATP1B1	25.74207065	-0.177169192	0.577765226	-0.206645644
0.759112107	2.11502717

8848 BAX 21.62226867 0.240175625 0.657146912 0.517655279
0.604698726 5.175402944
8849 BBS9 17.08768624 -0.160712958 0.695594225 -0.221045561
0.817279407 2.948179247
8850 BCDIN2D 19.04645904 -0.266742827 0.659495821 -0.556095762
0.578145292 2.458572458
8851 BORCS5 19.26885429 0.054411907 0.661984652 0.082195119
0.924491555 5.715845504
8852 C15orf61 22.71291491 0.296448772 0.627681284 0.464885485
0.642012466 14.01278007
8853 C16orf91 18.0897805 0.490847046 0.679606219 0.72225214
0.470129472 1.994224018
8854 C1D 17.9182712 0.782017467 0.688964078 1.125062759
0.256249024 10.11747595
8855 CARS2 22.9866872 -0.16828577 0.596215806 -0.282256472
0.777746859 5.448147414
8856 CCDC107 22.72897298 -0.22022806 0.598546665 -0.551882554
0.581028127 4.892814749
8857 CCDC189 28.85728465 -0.526001062 0.541418567 -0.971522874
0.221287471 9.118147104
8858 CCDC57 20.05122847 0.085244878 0.625262165 0.124245706
0.892129212 4.481841911
8859 CCDC77 17.29590496 -0.111252905 0.692995611 -0.160684864
0.872241612 2.11502717
8860 CCDC88B 11.41102078 0.180076522 0.902220026 0.19959049
0.841800866 5.429979042
8861 CDK16 19.82177847 -0.245281999 0.658254625 -0.272568196
0.709469847 2.49778084
8862 CDPF1 20.78577185 -0.429227264 0.626525158 -0.690201252
0.490067625 1.711748821
8863 CELF2-AS1 14.62080205 0.296884706 0.749875708 0.52926722
0.596620022 2.712148899
8864 CHIC2 20.52899957 -0.711549785 0.529549255 -1.242689269
0.179048889 7.194102825
8865 CMKLR1 17.77426926 -0.42285265 0.690509796 -0.626859526
0.520751214 1.985819988
8866 DDX60 27.25444267 -0.556155956 0.552654951 -1.004517264
0.215129254 5.707429589
8867 DEDD2 27.88264296 -0.441089117 0.549694092 -0.802426519
0.422206278 2.724490141
8868 DPH6 18.1262897 0.222695126 0.67224966 0.48072268
0.620712899 4.947185221
8869 DYRK4 21.98479229 0.085898072 0.626290205 0.127121867
0.890926565 1.479111242
8870 ECI1 12.42282217 0.884294926 0.82510209 1.071740014
0.282826822 4.172409918
8871 ECT2 14.09209571 -0.096489942 0.792250817 -0.121776795
0.902075792 14.10570474
8872 EML2 29.16422978 -0.247802756 0.549068555 -0.451216604
0.651761279 5.415851291
8873 ESPL1 17.20724625 0.220692209 0.702555908 0.21412761 0.75242412

							8.88478078
8874	ETFB	21.24742444	0.120922087	0.626624684	0.189959782	0.849240645	1.011089204
8875	FAM104B	18.06421244	0.01152028	0.668897602	0.017227726	0.986246958	2.484817001
8876	FAM199X	19.59824198	-0.065550148	0.659465274	-0.099298928	0.920821526	7.440744112
8877	FAM20C	10.96922574	0.856657285	0.889748288	0.96280858	0.225642594	1.498959444
8878	FAN1	19.97512454	-0.240254561	0.647652661	-0.525519297	0.599222172	1.71971085
8879	FBXO4	20.75204806	-0.015956546	0.622927212	-0.025210712	0.979886892	2.11502717
8880	FKRP	27.66990484	-0.105546264	0.562422587	-0.187662807	0.851140204	2.984988449
8881	FMN1	14.42820406	0.242626756	0.77881981	0.212828144	0.754411222	2.495181981
8882	FOXM1	11.4477401	0.688872918	0.865618572	0.795816924	0.426128448	7.154709118
8883	GAR1	25.02862584	0.188086752	0.592202929	0.217069292	0.751191025	2.918521042
8884	GAS2L2	22.28876289	-0.211911146	0.612282878	-0.245479296	0.729722998	4.878007429
8885	GEMIN8	20.45071256	-0.472965562	0.642867298	-0.724569942	0.46260145	5.940712281
8886	GSK2B	16.24186152	-0.125115454	0.712002122	-0.175722179	0.860511425	11.24154157
8887	HAGHL	19.8218786	0.192076878	0.640587207	0.299844964	0.764295416	4.494929511
8888	HAUS5	20.02716174	-0.272684925	0.627571909	-0.429261267	0.667722101	4.184701144
8889	HERC2P2	12.29606279	0.052122127	0.854592264	0.062161949	0.950422864	1.009152958
8890	HRH2	20.16922649	-0.429227282	0.676490627	-0.649272262	0.516161765	1.11741151
8891	HSD17B1	15.11482704	0.194268571	0.727288812	0.262490464	0.792172572	2.120707842
8892	IGBP1-AS2	12.8680152	0.157654971	0.784059229	0.201075221	0.840629671	5.45489101
8893	IMMP1L	16.22499962	0.142065527	0.715229722	0.198629228	0.842552781	1.7511925
8894	IQCG	16.22912629	0.217502424	0.71472792	0.444222162	0.656881971	7.411778205
8895	IRF7	24.65258127	-0.718171698	0.578544426	-1.241242252	0.214479206	1.498959444
8896	JAKMIP2-AS1	24.22902955	-0.552549665	0.595825058	-0.927252296	0.252742079	2.14741981
8897	JAZF1	14.16651425	0.721192542	0.760620561	0.948152204	0.242051975	2.482991455
8898	KIF2B	19.84211986	0.045151426	0.648172564	0.069659575	0.944464618	7.874280825

8899	L2MBTL2-AS1	16.10552974	0.274569178	0.710966252	0.286191522	0.699254822	1.492229015
8900	LANCL2	21.76528225	-0.015794565	0.612282647	-0.025791944	0.979422288	5.114189879
8901	LBX2-AS1	12.67402228	0.976026995	0.786942422	1.240290227	0.214868066	5.48197152
8902	LDB1	19.02228242	-0.288482264	0.654566629	-0.440722524	0.659412881	4.154745141
8903	LENG1	15.88229458	0.599429172	0.71808552	0.82477407	0.402844928	5.114189879
8904	LIG4	12.11089905	0.112050227	0.828225622	0.125289615	0.892282896	2.92124729
8905	LINC00229	24.88621798	-0.497272584	0.579896211	-0.857521699	0.291156628	1.997111212
8906	LINC00680	19.54178526	-0.421662298	0.65425288	-0.659677144	0.509461027	5.472515551
8907	LINC01504	14.40005084	0.451299718	0.752122665	0.599262024	0.548921495	2.70418488
8908	LRP8	17.08768624	-0.160712958	0.695594225	-0.221045561	0.817279407	10.5149011
8909	LRRC47	21.92669252	-0.642264892	0.61565818	-1.042216698	0.296847941	2.740140854
8910	LRRC58	24.89467296	-0.497922022	0.577426802	-0.862216066	0.288512614	9.405127471
8911	MAP4K1	20.80278292	-0.02122607	0.625426121	-0.022954562	0.972912284	1.000797979
8912	MAST2	19.05207967	-0.444646444	0.666628682	-0.666997664	0.504772625	9.101255149
8913	MBIP	18.21759622	-0.106898791	0.665026188	-0.160741215	0.872297148	5.112254521
8914	MFSD12A	26.65089872	-0.547846722	0.557624908	-0.982464581	0.225871024	5.914899447
8915	MFSD6	22.21529421	-0.525456426	0.606962942	-0.88218965	0.277674258	9.441494198
8916	MKNK2	26.99942055	-0.660516951	0.504228812	-1.209695055	0.190299017	5.402219802
8917	MOGS	14.40850682	0.44996214	0.752296641	0.597246012	0.550242142	1.487448211
8918	MOSPD1	16.86529112	-0.122891545	0.709692449	-0.174570507	0.861417125	1.988445225
8919	MRPL41	22.00259915	-0.169812815	0.596696217	-0.284588289	0.775959508	7.87714411
8920	MTA2	19.79805468	0.195971616	0.644246469	0.204140126	0.761021127	2.489411187
8921	NCR2LG1	7.982191081	1.416655625	1.140774425	1.241826757	0.214296798	7.247742189
8922	NFYC-AS1	16.81176984	-0.464228825	0.719810591	-0.645084749	0.518872266	2.70418488
8923	NT5DC1	21.24452904	-0.150224622	0.620024018	-0.242284501	0.808559722	1.481997418
8924	OAZ2	16.89921516	0.286621106	0.700122762	0.552222515	0.58078906	

1.728144818
8925 OSGIN2 21.08422107 0.147090956 0.620598448 0.222256124
0.815562519 4.715054149
8926 OTUD7B 15.12458425 0.287166265 0.722667878 0.291411926
0.695492771 14.59541045
8927 PASK 11.71226528 1.004856044 0.849221279 1.182114272
0.226762822 4.471474122
8928 PCMTD1 24.27205271 0.165122724 0.592224979 0.278200661
0.780781569 9.577024289
8929 PDE1B 15.65821407 0.849529595 0.726692894 1.169022621
0.242290086 1.492188952
8930 PDE6B 18.02889444 -0.225540789 0.674898756 -0.224184627
0.728240244 1.997941421
8931 PDE6G 22.18072908 -0.444418895 0.595852719 -0.745852599
0.455755857 8.284419111
8932 PEX1 15.55270674 -0.26017664 0.756467124 -0.476129921
0.622981815 1.711748821
8933 PGAP2 18.02227281 -0.144616725 0.677002199 -0.212612075
0.820848802 7.4170051
8934 PIDD1 16.8272279 0.051002922 0.694146091 0.072477216
0.941426274 4.944451742
8935 PKN2 22.1976911 -0.256491762 0.597526651 -0.429248587
0.667742228 2.941404019
8936 POLA1 19.29974282 -0.476729894 0.664471605 -0.717457124
0.472092077 2.111101812
8937 POLR2D 21.25015967 -0.218904208 0.617285524 -0.254566474
0.722914296 1.71971085
8938 PPP2R5E 19.22082152 -0.101978462 0.652795787 -0.155979076
0.876049514 4.471859011
8939 PRMT7 25.64222401 -0.808187529 0.569956225 -1.417981722
0.156196098 5.151771124
8940 PRR5 11.45619608 0.686902147 0.867654689 0.791678021
0.428548441 5.171448474
8941 PXMP4 22.2181796 -0.671467787 0.612912825 -1.095525546
0.272282082 2.959410724
8942 RAD22B 15.44152948 0.807175027 0.727574202 1.094264504
0.272795145 1.997941421
8943 RAD54L 14.69571465 0.90244252 0.752209842 1.199562292
0.220209279 2.481154209
8944 RAI1 28.58992412 -0.714721222 0.540574525 -1.222151297
0.186117775 4.95205844
8945 RANBP9 18.80446657 -0.179022169 0.656812215 -0.272578512
0.785177221 2.444144251
8946 RBM41 20.56079147 0.152870719 0.629277977 0.24292025
0.808059254 1.474274994
8947 RIC8B 25.70551151 0.106277284 0.587126965 0.181179675
0.856226548 4.471909072
8948 RPL26L1 15.06692652 0.298240508 0.796911229 0.274245584
0.708221612 1.141840508
8949 RRN2P2 21.28681892 -0.154265099 0.619002714 -0.249277096 0.8020691
5.445059572

8950	S100A12	22.54979946	-0.760725251	0.517558297	-1.469854422	0.141601186	5.144041559
8951	SART1	21.26712169	-0.016741072	0.619026012	-0.027044214	0.978424469	5.421125459
8952	SAYSD1	12.19256492	0.585299571	0.824567215	0.701440808	0.482027962	4.114145912
8953	SCAPER	20.06521496	-0.422712962	0.659254412	-0.641101287	0.521456878	1.121549181
8954	SGO2	17.62106227	0.414918148	0.679984628	0.610187522	0.541727588	1.71971085
8955	SIRT5	19.20824887	-0.249518685	0.652444265	-0.282426724	0.702127455	1.999904598
8956	SLC25A20	11.00219971	1.298201997	0.905640295	1.422462	0.151725565	2.714741859
8957	SLC66A2	16.12946278	0.825701942	0.722264222	1.141622499	0.252606809	1.009152958
8958	SLC9A5	18.20068427	-0.104956126	0.665694206	-0.157664164	0.874721441	4.715141802
8959	TAOK2	21.66266142	-0.612160496	0.628150224	-0.976126542	0.228996805	4.104448547
8960	TCF2	19.86296729	0.5577164	0.648706218	0.859726247	0.289924294	7.247712118
8961	TIGD1	15.22146157	-0.028746476	0.742218115	-0.052122285	0.958422411	4.257142111
8962	TMEFF2	16.6404608	0.894115797	0.715727825	1.249229957	0.211577222	1.492229015
8963	TMEM161B	18.07560276	0.089927826	0.667622175	0.124712674	0.892828262	4.871424849
8964	TMEM201	22.52274089	0.272028265	0.612221401	0.444250066	0.656861799	5.448425041
8965	TMEM251	24.96227185	-0.226045202	0.575862061	-0.566186289	0.57126715	1.504580178
8966	TNRC6C	16.09141207	0.096828926	0.712559609	0.125712469	0.89204859	1.741001175
8967	TRDMT1	15.91149786	0.689925829	0.716178501	0.962257272	0.22526821	8.122125197
8968	TRMT12	18.81202267	0.280727026	0.662811088	0.42255512	0.671890285	11.51405978
8969	USP9Y	15.25682951	-0.042229019	0.727012288	-0.057446822	0.954189266	4.944249985
8970	VHL	19.47702294	-0.126057827	0.674142094	-0.186989718	0.851668694	2.941404019
8971	ZDHHC20	20.42270148	-0.92104269	0.662242056	-1.288697071	0.164924885	2.450147741
8972	ZNF2	15.6298606	-0.090807697	0.741221896	-0.122494287	0.90250757	1.714875502
8973	ZNF225	14.14950227	0.098989282	0.776079405	0.127550587	0.898504628	2.114581191
8974	ZNF227-AS1	20.46484022	-0.220582674	0.640912474	-0.515801591	0.605992991	4.114145789
8975	ZNF576	16.1722877	0.819958745	0.714877228	1.146992281	0.25128477	

2.95295004

8976	ZNF720	17.0924071	0.261127766	0.688760627	0.279127022	0.70459254	8.144514511
8977	ZNF79	18.80168129	-0.025121559	0.655208217	-0.02825074	0.969408026	1.980159195
8978	ABITRAM	19.96095674	-0.228256451	0.660825406	-0.511869622	0.60874226	4.115489811
8979	ABTB1	21.48921571	-0.11262521	0.618906915	-0.182590206	0.854224869	4.907942411
8980	AC005091.1	20.82521545	0.254542782	0.625884426	0.406692942	0.684222524	2.712198827
8981	AC005702.6	20.28574255	-0.11552727	0.542126712	-0.212707767	0.821554908	2.721454814
8982	AC006547.1	18.12066787	0.225108127	0.669622551	0.48550155	0.627220614	1.944081485
8983	AC011472.2	19.95812129	-0.41144924	0.660076086	-0.622226079	0.522062692	4.422414989
8984	AC022211.2	29.12877052	-0.600086251	0.529829928	-1.111600225	0.266210022	1.492188952
8985	AC024075.2	22.21455192	-0.122681782	0.597004825	-0.222245742	0.824122587	4.149094548
8986	AC027290.2	12.19824204	0.428970527	0.82029716	0.522945278	0.601012225	4.71290154
8987	AC082862.1	18.5877909	0.164849578	0.658462802	0.250255187	0.802212682	4.71290154
8988	AC092495.1	16.28702574	0.672418274	0.708202109	0.950748861	0.241721878	1.145415792
8989	AC127070.4	15.10065922	0.197268586	0.75127595	0.26254212	0.792902746	1.74278744
8990	AC127496.6	22.26055854	0.05065002	0.606985687	0.082445181	0.922497565	2.141744491
8991	ADAT2	14.18222511	0.611906817	0.766027627	0.798805155	0.424402401	5.44789491
8992	AFF2	18.17140274	0.218128121	0.712575894	0.446462102	0.655262482	1.498959444
8993	AL450405.1	18.16299782	0.56228672	0.685275622	0.822121554	0.411002029	2.491140108
8994	AP001107.2	21.41206172	-0.726767294	0.640811581	-1.124125702	0.256727629	8.418411579
8995	AP001160.1	19.02959607	0.244052287	0.680888022	0.258422487	0.720019682	1.474274994
8996	APOBEC2B	16.60076487	0.092889825	0.702296022	0.122670779	0.892662912	4.474494258
8997	ATAT1	22.64292154	-0.852664294	0.621264919	-1.272852529	0.169487156	1.744411804
8998	ATG2B	20.72777012	-0.424224262	0.620812851	-0.68827257	0.491218192	5.180974514
8999	ATPAF1	22.26245244	-0.199227498	0.594208721	-0.225282011	0.727412262	1.728144818
9000	BDH1	26.26271277	-0.119625182	0.578705271	-0.206729001	0.826221507	2.710412551

9001	BRCA1	22.99225669	-0.105429217	0.595160972	-0.177144205	0.85929512	1.472541449
9002	BTBD9	25.14785655	-0.641602989	0.57211452	-1.121460749	0.26209179	4.905108074
9003	C6orf126	17.08201458	0.094076642	0.6924994	0.125850866	0.891929179	1.159751445
9004	CAMKK1	21.74812001	-0.689874412	0.622027909	-1.091525225	0.275041824	11.41220124
9005	CARHSP1	41.68269718	-0.277276247	0.510485911	-0.542161606	0.587018529	4.411012017
9006	CCDC186	22.92858525	-0.546856888	0.599126448	-0.912757048	0.261270222	2.721704877
9007	CCL2L1	17.16672455	0.862581289	0.694702091	1.242094209	0.21282212	4.457721229
9008	CDKL1	18.77227681	-0.174958991	0.665047201	-0.262077515	0.792490824	1.154917118
9009	CENPW	21.72568755	-0.210626812	0.615500218	-0.242204221	0.722197201	2.114581191
9010	CEP89	17.55764266	0.177214224	0.69428757	0.255246156	0.798522984	1.487448211
9011	CERKL	20.46747426	-0.769625514	0.656222224	-1.172648852	0.24092665	10.57897824
9012	CIPC	18.11221201	0.822955174	0.695584582	1.182112018	0.226764259	1.000797979
9013	CLSTN1	19.28004451	0.052719462	0.656565424	0.081818902	0.924790725	1.725221481
9014	CR1	16.2587724	0.215258992	0.706972472	0.446069689	0.655546907	4.500111255
9015	CTCF	25.92884668	-0.422567484	0.562614059	-0.751520427	0.452229506	5.472545412
9016	DAAM1	12.4007409	-0.057268225	0.822979216	-0.06866864	0.945252281	1.141840508
9017	DAPP1	16.79764209	-0.20068502	0.715462422	-0.280496561	0.779096564	4.444188218
9018	DDIAS	11.68127551	1.014027527	0.891217291	1.127672772	0.255257147	12.79019251
9019	DHFR2	15.91696724	0.224589156	0.741116892	0.202041475	0.761858254	4.491148818
9020	DHX58	12.92276805	1.228255599	0.84227121	1.45809204	0.1448149	5.424158208
9021	DLGAP5	16.11110924	0.264977477	0.714592502	0.510749099	0.609526755	5.18444511
9022	DNAH17	22.76419065	-0.592596157	0.620572759	-0.954918096	0.229619118	1.445085471
9023	DPYD-AS1	18.78466812	-0.099294574	0.661504624	-0.150104125	0.880682466	2.948219409
9024	ELP4	19.55024029	0.284625497	0.662412215	0.579768488	0.562070751	2.941404019
9025	EPHA1	18.12912284	0.222996122	0.672872722	0.481511052	0.620152227	1.498959444
9026	ERMARD	21.27821181	-0.01722124	0.616271146	-0.028102127		

0.977580697 1.714875502
9027 EVI2A 20.62555281 0.216297176 0.654405224 0.220677587
0.740888024 7.419790285
9028 F2RL2 11.00209851 1.142649092 0.917652685 1.246275268
0.212662254 5.180974514
9029 FAHD2A 15.5988209 0.191722891 0.722278496 0.261461222 0.79272685
9.157941041
9030 FEM1B 21.51179824 -0.286294252 0.618150574 -0.624919248
0.522022992 4.4114171
9031 FHL2 20.6502676 -0.280978559 0.526578955 -0.522648115
0.600522209 2.717518144
9032 FKBP14 12.18700178 0.657612142 0.797494908 0.824598548
0.409599546 10.55081514
9033 GBP2 22.74265209 -0.277211428 0.590621429 -0.62866872
0.522028459 2.915494494
9034 GEMIN5 21.68240766 -0.206446252 0.642081805 -0.221526559
0.747811289 2.47170022
9035 GIT1 22.82268287 0.555542662 0.614401792 0.904202542
0.265888012 7.871495417
9036 GNA15 25.04065678 -0.261200886 0.515101425 -0.70122282
0.482162964 4.105501122
9037 GNE 17.8225101 0.212165222 0.674661405 0.214476746
0.752158975 2.191142194
9038 GPATCH1 17.28891965 0.294026482 0.689789901 0.426255127
0.669921952 7.404084942
9039 GRAP 21.22214651 -0.285122802 0.62092912 -0.459187258
0.646099628 1.011089204
9040 GSPT2 22.22991985 -0.124975912 0.591799174 -0.228077222
0.819586207 5.940974077
9041 GTF2E2 19.09729296 0.465250862 0.654215152 0.711211654
0.476891122 7.152912942
9042 HNMT 18.61225896 0.625870911 0.666879844 0.952501469
0.240226052 2.141949117
9043 IFI44 15.95656197 0.977216724 0.724284256 1.220661129
0.182200525 10.8194148
9044 IFT122 27.42222168 -0.298881982 0.55267497 -0.720426252
0.471262591 1.002422214
9045 IFT46 22.76229929 -0.217225219 0.595248924 -0.522856112
0.594122187 1.140005141
9046 IRF8 17.59997261 0.41987259 0.681690755 0.615928244
0.527941879 2.700414187
9047 KBTBD8 17.8984227 0.615272872 0.67796446 0.90752116
0.264125979 1.000797979
9048 KIAA1142 24.19669852 -0.425920104 0.580252122 -0.724041899
0.462922202 7.448947505
9049 KLHL8 18.56520825 0.012199998 0.660225792 0.018478524 0.98525711
2.481154209
9050 LAPTM4B 21.4825449 -0.661606229 0.629224408 -1.051295994
0.292122667 1.495824874
9051 LINC00624 18.25141907 0.282690888 0.662924822 0.427921787
0.668700782 5.41171571

9052	LRRC28	12.16725448	0.54740518	0.800826759	0.682550061	0.494259228	4.114741294
9053	LYPLAL1	26.69596282	-0.284054015	0.5597964	-0.686060172	0.492675164	2.489441249
9054	MAFB	24.01668219	-0.405661901	0.600569425	-0.675462115	0.499282212	2.981152212
9055	MOB1B	20.99115405	-0.116894077	0.627968802	-0.186146212	0.852220024	5.918242245
9056	MRI1	22.02224528	0.205092127	0.601796055	0.240800069	0.722254099	4.477191117
9057	MTERF1	22.51200101	0.017969165	0.591285716	0.020284848	0.975760129	1.997111212
9058	MTRF1L	20.2186978	0.261152107	0.622416448	0.412942255	0.679648189	2.948074711
9059	MZF1	27.20262054	-0.216274722	0.557757774	-0.287757424	0.698195548	2.11502717
9060	NFIC	25.71286629	0.050025292	0.576221472	0.086816867	0.920817081	5.887254798
9061	NOMO1	15.65522746	0.26941552	0.726447	0.508522718	0.61108611	2.945141244
9062	NTAN1	19.82749815	0.486652097	0.645246181	0.754096191	0.450791464	5.487441114
9063	PALB2	18.72666748	-0.482829415	0.682016641	-0.70692189	0.47961502	4.001900414
9064	PCBD2	18.22211566	-0.265096842	0.67721029	-0.29129645	0.695504216	8.911924
9065	PDE4A	22.18708256	-0.124427599	0.545895062	-0.246251721	0.80548728	4.285514445
9066	PDE7A	19.02954601	0.016417279	0.661226052	0.024828542	0.980191725	4.491454277
9067	PECR	17.57460567	0.422494221	0.70082761	0.604268827	0.545664928	4.4114171
9068	PIGA	17.86459879	0.620140261	0.680424011	0.911289277	0.262090202	2.704124818
9069	PRKD2	22.29725241	-0.659890069	0.600082624	-1.09966525	0.271477957	4.480245051
9070	PTGDR2	21.82720948	0.276956912	0.617582695	0.610274797	0.541612559	4.425149489
9071	PVR	19.06610514	-0.268185297	0.669997619	-0.549522297	0.582640128	4.47180895
9072	RAB11FIP4	24.07612524	0.495922086	0.602929766	0.821144944	0.411562712	7.444111159
9073	RAD52	20.70284609	-0.871758928	0.66055829	-1.219720209	0.186925077	4.914855278
9074	RASGEF1B	24.25205502	-0.491627165	0.59757757	-0.822700164	0.410678522	1.454419491
9075	RCOR1	18.57928499	0.40072094	0.667790627	0.60006972	0.548459772	1.997111212
9076	RIC1	18.78456801	-0.567227791	0.682162009	-0.820458627	0.406279525	4.915907942
9077	RPL2P4	20.27510427	0.297999045	0.646101575	0.616000724		

						0.527894025	2.158911144
9078	SBF1	18.88624	0.249475406	0.667554802	0.522515678	0.600615444	1.997941421
9079	SENP6	25.97550594	-0.269722508	0.571565286	-0.646861262	0.517721728	1.971752278
9080	SERPINB8	18.5680426	0.089509916	0.659949055	0.125621555	0.892112559	1.954791259
9081	SERTAD2	22.502551	-0.109097769	0.600287028	-0.181712299	0.855808425	4.114007172
9082	SMIM25	21.11819297	0.702218722	0.625898942	1.106022807	0.268716648	4.949970417
9083	SMURF1	15.42174104	0.912279607	0.721845925	1.247912286	0.212062144	11.18444714
9084	SPHK2	22.97519455	-0.681462587	0.608829942	-1.119298729	0.262012712	5.111012145
9085	SPRED2	25.6488025	-0.747589225	0.568989442	-1.212889466	0.188882404	4.448972402
9086	TAZ	25.14512122	-0.245822524	0.579908802	-0.596241228	0.550947287	2.495181981
9087	TBC1D15	27.72227486	-0.109259962	0.560540492	-0.194918949	0.8454564	7.254471844
9088	TCEAL2	27.66124766	-0.156658119	0.556468022	-0.281522226	0.778209882	4.485985482
9089	TEFM	24.21659601	0.171014125	0.605128594	0.282602252	0.777480987	12.04152225
9090	THAP2	21.02074878	0.420242228	0.651275724	0.660616267	0.508858424	4.41449271
9091	TMEM220-AS1	14.40845569	0.658122702	0.757207879	0.869144022	0.28476826	7.88547114
9092	TMEM80	22.81150017	0.248096617	0.599995269	0.580165602	0.561802948	8.117444417
9093	TMEM87B	26.94641128	-0.517584496	0.562542495	-0.92007907	0.257521441	4.449214412
9094	TMTC2	22.82268287	0.555542662	0.614401792	0.904202542	0.265888012	1.942147228
9095	TNIP2	22.20022512	-0.628922824	0.598160424	-1.068162984	0.285446999	5.147100541
9096	TRAV8-5	21.06725785	-0.124540542	0.620122805	-0.197641729	0.842225276	4.878007429
9097	TRIOBP	17.82211865	-0.254882089	0.694718195	-0.510820279	0.609469905	1.707118199
9098	TRMT61B	25.24290791	0.105178598	0.57925182	0.181545296	0.855929582	4.941514428
9099	UAP1L1	22.72962554	-0.120882202	0.587016444	-0.22296172	0.822565292	1.971752278
9100	UPF2A	40.46124227	-0.205821124	0.508282202	-0.601575882	0.547456487	5.151811194
9101	WARS2	20.55496951	-0.267798118	0.627446522	-0.420110719	0.674404572	1.151194484
9102	WDHD1	17.07077221	0.264987704	0.705792285	0.275447101	0.707227981	4.125487174

9103	WDR25	19.51920162	-0.204267929	0.648028789	-0.215209417	0.752602656	10.40184842
9104	WDR28	12.14472189	0.666252992	0.826284072	0.806228017	0.420111287	1.997941421
9105	WDYHV1	18.24574828	0.126501825	0.664182069	0.190462587	0.848946658	1.159751445
9106	WRNIP1	12.29228492	-0.055868204	0.821196985	-0.067214276	0.94641112	1.725221481
9107	YWHAZP2	18.89752122	0.425686509	0.67221409	0.622166129	0.526625162	8.448258882
9108	ZCCHC4	16.40288764	0.298062688	0.709667201	0.560916	0.574854802	4.480245051
9109	ZDHHC16	25.74470468	-0.518752206	0.58958014	-0.879867165	0.278921274	1.141840508
9110	ZKSCAN4	22.50160956	-0.412421422	0.596820627	-0.692694722	0.488501146	5.718720914
9111	ZNF212	18.80725091	0.516208661	0.694754488	0.742008746	0.457476285	5.451170289
9112	ZNF282	20.49204244	0.090704171	0.655466127	0.128281172	0.889929176	1.944081485
9113	ZNF204	14.18042971	0.196905247	0.796655419	0.247164887	0.804780619	2.455728211
9114	ZNF496	21.05218124	-0.054522648	0.621861209	-0.087676541	0.920122766	4.141107808
9115	ZNF528	14.85016072	0.151728579	0.761526865	0.199255712	0.84206272	1.154081771
9116	ZNF600	19.61505274	-0.140622922	0.678985114	-0.20712272	0.825912228	1.004418411
9117	ZNF880	21.08128452	0.011022841	0.620690987	0.017477404	0.986055759	4.92898105
9118	ZSCAN16-AS1	16.07444998	0.280022541	0.740919624	0.277940505	0.705474792	4.701410115
9119	AC087164.2	21.10112075	0.422517115	0.625467676	0.675521902	0.49924424	1.971702214
9120	AC109597.2	18.12240201	0.22540522	0.670259028	0.485491902	0.627227456	2.970841997
9121	AC118244.4	19.28556295	-0.095785195	0.652007294	-0.14668217	0.882282102	1.725221481
9122	AC126622.1	19.24764121	0.19597092	0.647761886	0.202525429	0.762242929	8.10114442
9123	AC127620.2	18.66961429	0.961222051	0.672597577	1.429284142	0.152922582	4.474494258
9124	ACOX2	16.42646922	1.050476158	0.712162082	1.472981682	0.140755975	5.471017491
9125	ACYP2	19.08018068	-0.064272672	0.656945164	-0.097987892	0.921941908	5.189480557
9126	ADGRA2	19.89091811	0.259278852	0.666458299	0.289029815	0.697246694	9.599514914
9127	ADGRE1	24.71285215	-0.681189022	0.501885618	-1.257259498	0.174698772	4.291127097
9128	AL021220.2	22.52826787	0.222522589	0.596257251	0.542497954		

						0.587475517	5.445449757
9129	AL590867.2	14.622425	0.298282594	0.76262712	0.291119952	0.695708574	5.104291512
9130	AL627402.1	22.20488145	0.675422076	0.620965626	1.087696401	0.276729157	9.912222945
9131	ALKBH4	19.82461167	0.562576216	0.650541424	0.864781696	0.28715862	10.07514599
9132	ALKBH8	14.15218529	0.205126214	0.778962274	0.291708668	0.695272485	8.117414545
9133	ANKIB1	25.19580606	-0.222710915	0.569811652	-0.410154678	0.681692485	4.975491271
9134	AP002296.1	22.24427592	0.415185512	0.607976521	0.68289727	0.494671759	4.192157111
9135	ARHGEF9	21.02417256	-0.698212041	0.52174702	-1.228411148	0.180762447	2.941255957
9136	ARMH4	22.09745409	0.404272422	0.616422229	0.655988907	0.511821262	1.99995444
9137	AURKB	22.49120854	-0.042289297	0.602829229	-0.071855865	0.942716614	1.940411991
9138	BBS10	11.96261122	0.4089242	0.899520978	0.45459712	0.6492991	1.728144818
9139	BET1	24.6109452	0.85248844	0.610204662	1.298462919	0.1619741	2.489411187
9140	BNIP1	25.55916776	0.182779502	0.589162275	0.211922524	0.755091027	2.951114494
9141	BPNT1	21.22209528	-0.147961702	0.6269405	-0.226005974	0.812428025	1.498471111
9142	C12orf72	25.4772422	-0.091794849	0.568604221	-0.161428916	0.871747718	8.110701408
9143	CCDC126	16.25588592	0.405922916	0.715928715	0.566981274	0.570726826	4.451874847
9144	CCDC142	22.26901244	0.275751162	0.627024211	0.599260917	0.548998906	4.947185221
9145	CCNY	15.55628975	-0.171757142	0.774084289	-0.221884261	0.824402982	4.128171441
9146	CDK11B	22.2512101	0.226920869	0.606024747	0.290959065	0.695827494	1.115878489
9147	CENPF	22.2519512	-0.20742007	0.602161848	-0.242887915	0.720920581	2.717518144
9148	CEP104	15.15129402	0.677482856	0.740691274	0.914664162	0.260267964	7.245120477
9149	CEP78	25.15242605	-0.581529822	0.572121469	-1.016444677	0.209417679	1.980159195
9150	CKS1B	22.67201766	-0.686202759	0.595640218	-1.15204284	0.249202059	2.941458715
9151	COA7	21.22172177	-0.225780095	0.644202679	-0.250479906	0.725978568	4.407998991
9152	CORO2A	19.57272171	0.012200277	0.644014179	0.018944268	0.984885565	4.442452095
9153	CREB2L4	19.54725278	0.015015427	0.644920767	0.022282607	0.981424846	5.111042107

9154	CRELD2	22.82626696	0.261692999	0.60519447	0.597649212	0.55007402	1.159751445
9155	CRY1	20.09912672	0.297452025	0.626464107	0.46725241	0.640247765	1.988445225
9156	DDI2	25.19580606	-0.222710915	0.569811652	-0.410154678	0.681692485	2.14741981
9157	DDX19B	21.26686922	-0.256476268	0.622145682	-0.572059276	0.567281817	8.494409591
9158	DDX2P1	22.11261278	-0.400929206	0.520812291	-0.769815029	0.441409622	9.245980418
9159	EEF1AKMT2	14.92626246	0.622749546	0.745192598	0.850449522	0.295075208	7.270498412
9160	EFCAB2	17.26061517	0.121569225	0.687971672	0.191242212	0.848225822	2.12254211
9161	EGR1	17.52495981	-0.065624442	0.705764185	-0.092997696	0.925905292	9.851900821
9162	ENOX2	20.95985676	-0.205857241	0.552942785	-0.552142465	0.580165176	5.940412158
9163	ERCC2	22.71251226	-0.561628509	0.591914676	-0.948822561	0.24270527	1.997941421
9164	ERI2	26.40596864	-0.246242147	0.562978118	-0.427292028	0.661827057	1.479111242
9165	EXO5	21.22227592	0.181776128	0.618110549	0.294082529	0.768694069	2.711907511
9166	FBH1	25.195756	-0.407622458	0.569024964	-0.716270078	0.472762866	4.104448547
9167	FDXR	27.04246156	0.445626105	0.578151016	0.770795222	0.440828254	2.141999189
9168	GATD1	22.72947428	-0.275521006	0.586848957	-0.629892795	0.522241648	1.982044702
9169	GATM	10.9494261	0.864227029	0.920022841	0.929242672	0.247554222	4.182018404
9170	GMIP	26.15262479	-0.222021657	0.562966242	-0.589789628	0.555221691	4.447241017
9171	GOLGA1	19.12106674	0.617506028	0.654678125	0.94222079	0.245567982	1.728144818
9172	GPR127B	20.7422295	-0.798857746	0.66642046	-1.198711222	0.22064021	10.40994491
9173	GPR141	24.75967159	-0.185465197	0.580709298	-0.219277006	0.749440645	7.449150144
9174	HCRTR1	21.25846221	-0.15048209	0.618192517	-0.242422697	0.807677962	4.412858244
9175	HERC6	21.84974087	0.241521908	0.622201246	0.287566472	0.698226885	2.451952017
9176	HJURP	28.95218254	-0.126892525	0.547121701	-0.221929249	0.816592967	4.429027411
9177	HMGB2	17.81928222	-0.028252491	0.681448922	-0.041460908	0.966928457	2.12911278
9178	HP	26.12005214	-0.44266266	0.564899756	-0.782614526	0.422266224	2.489441249
9179	INO80	27.42765991	-0.228275842	0.550217104	-0.42297917		

0.665029902	2.941404019
9180	INPP4A 20.2254585	-0.024494109	0.640129572	-0.0282627
0.969477422	4.947428018
9181	ITSN1	17.90961282	0.61422127	0.680918979	0.902209057
0.266945825	5.445599495
9182	KIAA2012	16.61762569	0.26652728	0.702126211	0.27960922
0.704225516	7.428009
9183	KLC1	27.40786255	-0.450287629	0.547911996	-0.822007242
0.411072775	1.118712824
9184	KLF12	27.25625075	0.25721049	0.5676668	0.452277228
0.650249057	5.18444511
9185	KNTC1	24.49792182	-0.097984177	0.578069755	-0.169502242
0.865401521	7.401149414
9186	LGALS8-AS1	22.12819672	-0.765598295	0.614768558	-1.245244097
0.212005292	4.174195102
9187	LIAS	19.21276722	-0.024175009	0.64846722	-0.027280222
0.970261567	1.140005141
9188	LLGL1	22.29148052	-0.857188298	0.612622122	-1.299189419
0.161756189	4.440547484
9189	LSMEM2 22.7897077	0.201552902	0.602442404	0.499720272
0.617272056	1.951954012
9190	LYRM7	27.12244	-0.815582147	0.55582292	-1.467242472
0.142282642	4.477479442
9191	MAP4K2 25.70242271	-0.229262881	0.564296804	-0.406287622
0.684457806	1.151194484
9192	MBD2	24.05055609	0.074966991	0.59271407	0.12648087
0.899251291	2.478022524
9193	MEIOC	14.14272921	0.206558222	0.779754514	0.292147224
0.694210747	8.84512411
9194	MIB1	18.02595682	0.01626411	0.690742848	0.022690596
0.981099407	2.710412551
9195	MKS1	19.85992954	0.226428887	0.641849162	0.524171264
0.600159426	2.929812248
9196	MOB4	22.22424809	0.05220422	0.5995211	0.087075249
0.920611699	4.449174241
9197	MRPL2	18.40772444	0.256251627	0.687619227	0.518229765
0.604290992	2.110910498
9198	MSH2	24.787975	0.220115222	0.582729152	0.2770847
0.706110661	1.484821974
9199	MSL2	25.92750422	-0.265562605	0.561225624	-0.651227126
0.514892428	4.111240504
9200	MTRNR2L8	14.40551914	0.452148082	0.762591867	0.592122182
0.552761408	4.441281908
9201	MX1	20.52248591	0.269245604	0.652625006	0.565062989
0.572020241	5.144897904
9202	MYSM1	27.2226681	0.152957756	0.562271574	0.271552415
0.785966192	5.479184145
9203	P2RY6	17.06782677	0.096672688	0.704544555	0.127214442
0.890861295	1.97727401
9204	PANK2	17.61294902	0.252826712	0.679277625	0.272199281
0.709744407	4.417794258

9205	PANK4	14.64761172	0.806498861	0.771408225	1.045489047	0.295797	7.444751404
9206	PARG	14.8810481	-0.046210217	0.776886611	-0.059481417	0.952568666	4.9144447
9207	PEX26	19.82884085	-0.024788816	0.642210587	-0.028522201	0.96926256	4.920514071
9208	PHACTR2-AS1	20.52109446	-0.122648558	0.629767179	-0.194752222	0.845586927	5.48197152
9209	PILRB	16.29522046	0.210812594	0.711197644	0.427028426	0.662090726	2.105189844
9210	PPM1B	25.98669607	-0.270128912	0.572195204	-0.645729144	0.518454782	1.487448211
9211	PPOX	29.61718281	-0.287047504	0.52269761	-0.72658014	0.467482198	1.988445225
9212	PSMD6-AS2	21.81298148	-0.218286455	0.626759492	-0.248427412	0.72751171	4.477191117
9213	PTEN	18.0752512	-0.208926975	0.692815182	-0.445915422	0.655658241	1.71971085
9214	PTOV1	20.58022627	0.081706087	0.620227725	0.129624768	0.896862206	4.944451742
9215	PUS7	12.14457062	0.218066895	0.822249412	0.262021086	0.792205185	2.45011748
9216	RAD51	22.4741464	-0.629190877	0.614518895	-1.022875558	0.205894056	1.942147228
9217	RASA1	25.67695565	-0.571227727	0.566260921	-1.008769682	0.212085104	1.127149814
9218	RELT	25.21075122	-0.46256947	0.506279722	-0.915629022	0.259856257	4.110049179
9219	RGS5	24.47529911	-0.292069404	0.579551754	-0.67822002	0.497625855	2.712198827
9220	RHBDD1	25.28225126	-0.011996272	0.582651908	-0.020589092	0.982572442	9.277271877
9221	RNA5SP151	27.84294696	-0.652257222	0.545402282	-1.195919279	0.221728061	7.115470599
9222	RNF187	22.22022721	-0.509957528	0.515071982	-0.990070429	0.222129696	4.494154124
9223	RPL12P12	21.07287741	0.149476842	0.62108291	0.240670921	0.80981017	1.495824874
9224	RPL22AP42	17.11578725	0.259480464	0.690071202	0.276019786	0.706902186	2.10145451
9225	RPL9P7	27.9088095	-0.548510828	0.546206227	-1.00421927	0.215272926	5.42420827
9226	RRM2B	14.20292116	0.296221119	0.819220992	0.261674491	0.717595291	2.714024184
9227	RSBN1	27.00002148	-0.024192991	0.564889774	-0.060520277	0.951722222	5.41580122
9228	RXRB	12.62427622	0.212412842	0.810215216	0.26216962	0.792190674	5.915508018
9229	SEC22A	17.8502708	-0.112912777	0.689748581	-0.162702804	0.86996511	1.715584178
9230	SEMA4C	19.52217705	-0.254284706	0.655987268	-0.540078549		

0.589142862	1.711104809
9231	SENP7	21.82422287	0.045521607	0.617776892	0.072686159
0.941260112	4.118515158
9232	SHARPIN	29.98049545	-0.172488729	0.557625824	-0.209227029
0.757072772	9.114772144
9233	SHLD1	19.51252987	0.018871822	0.661769056	0.028517247
0.977249612	1.744411804
9234	SIDT2	20.2902922	-0.205166618	0.640241072	-0.4766422
0.622616145	5.895810777
9235	SLC2A5	18.22868516	-0.185822622	0.67782578	-0.274142555
0.782975072	5.410181498
9236	SMAGP	22.49409295	0.212864682	0.621782718	0.242245767
0.722090697	4.195941507
9237	SMURF2	11.42908172	0.42158914	0.912952222	0.472729598
0.626298972	7.471781851
9238	SNUPN	28.20720866	-0.259451916	0.551280712	-0.470549496	0.62796248
2.129142841
9239	SPRED1	15.22558848	-0.415508224	0.801202067	-0.518541222
0.604080626	4.950172275
9240	SSBP2	24.2524008	-0.51774547	0.508257179	-1.018668287
0.208260472	1.758755451
9241	TBC1D1	18.85792552	0.199471088	0.657822252	0.202224246
0.761718897	1.501424211
9242	TBC1D10B	25.80956614	-0.12587268	0.605804221	-0.207779466
0.825401166	1.747444115
9243	TGFBRAP1	22.99692481	-0.160490676	0.582540528	-0.275501201
0.782921126	1.501424211
9244	THEM4	21.05875074	0.012961225	0.62066522	0.022492982
0.982052912	1.508154944
9245	TIGD6	21.26412401	-0.015229658	0.619286006	-0.024752761
0.980251272	1.729008147
9246	TPK1	19.98627254	-0.194688905	0.65475295	-0.297247122	0.76620151
1.011920412
9247	TPM2P9	20.55770266	-0.267495262	0.629856981	-0.41805477
0.675907074	1.011920412
9248	TRMO	20.02128877	-0.127194294	0.627792252	-0.199429162
0.841927051	1.729008147
9249	TRMT6	22.01200292	0.26974162	0.607291856	0.444098182	0.6569716
1.512917427
9250	UBE2Q1	12.22161588	0.821824419	0.851182292	0.977269298
0.228425848	1.750199471
9251	UBP1	24.21917997	-0.266522218	0.580161219	-0.621759144
0.527544274	1.499800945
9252	UHRF1	14.22921521	-0.140920248	0.822286256	-0.171147195
0.864108027	1.989504454
9253	VPS4A	27.40224204	-0.078078269	0.562869949	-0.128468575
0.889870102	1.984471207
9254	WBP4	24.85502702	-0.552772802	0.592860712	-0.924068982	0.25026821
1.501424211
9255	WDTC1	18.82812815	-0.104688484	0.666145947	-0.157155476
0.875122217	1.151127805

9256	WNT2B	14.62910569	0.497892811	0.760142749	0.655000408	0.512467491	1.508154944
9257	WRAP52	20.59724822	0.07984908	0.628224022	0.125111265	0.900425286	1.499800945
9258	ZADH2	22.29228975	-0.516818221	0.620582826	-0.819587024	0.412451572	1.752124819
9259	ZBTB14	12.904422	-0.262926472	0.869654277	-0.202224445	0.762297122	1.499800945
9260	ZFAND2B	20.29526081	-0.29645875	0.542274467	-0.720968682	0.464798282	1.752124819
9261	ZNF100	20.08222477	0.29942588	0.624686282	0.471769819	0.627091096	1.989504454
9262	ZNF18	17.8502708	-0.112912777	0.689748581	-0.162702804	0.86996511	1.499800945
9263	ZNF226	20.22548072	-0.226772521	0.652289911	-0.247070741	0.728528174	1.140592782
9264	ZNF25	21.72971202	-0.144845077	0.612975468	-0.22591246	0.812499822	1.011920412
9265	ZNF221	19.25447526	-0.470874458	0.670756515	-0.702005046	0.482676017	1.14241912
9266	ZNF559	22.29911062	0.169988801	0.595481256	0.285464522	0.775288281	1.752124819
9267	ZNF786	22.28025471	0.242761206	0.609551456	0.299902754	0.689228142	1.501424211
9268	ZNF816	22.72026566	-0.264299579	0.597620815	-0.442412894	0.65819045	1.989504454
9269	ZNF91	20.98249675	-0.605227882	0.629822876	-0.946087492	0.244102962	1.512917427
9270	ZSCAN16	16.28402914	0.222708689	0.711667426	0.214244422	0.752259462	1.989504454
9271	AAMDC	19.78072901	-0.46026647	0.665584001	-0.69167298	0.489142719	1.741590797
9272	AC005480.1	22.04094622	0.212870767	0.607785726	0.250229826	0.72615872	1.978115219
9273	AC008840.1	12.6877964	0.525599112	0.800605741	0.656501804	0.511501207	1.011920412
9274	AC012414.2	21.21192222	-0.15518125	0.626746829	-0.247598142	0.804445248	1.499800945
9275	AC016821.4	22.5224272	0.081086501	0.599097171	0.125247828	0.892226871	1.011920412
9276	AC020978.9	18.66095709	0.291278208	0.682869288	0.572152564	0.567217944	1.151127805
9277	AC027682.5	21.08690288	0.217172942	0.622017416	0.249144471	0.726980852	1.011920412
9278	AC069287.2	29.62692004	-0.092766227	0.54104826	-0.172204922	0.862411754	1.984471207
9279	AC079880.2	19.04909091	-0.06025947	0.65980426	-0.0912292	0.927220927	1.747444115
9280	AKAP8L	16.67681646	0.704115909	0.706017977	0.997205921	0.218616041	1.501424211
9281	AL157928.2	15.67477227	0.274647451	0.754817198	0.262859556		

						0.715962887	1.151127805
9282	ALKBH2	16.61478927	0.622879152	0.720259229	0.864798571	0.287149256	1.747444115
9283	ARL14EP	29.42721747	0.024086005	0.542022261	0.044427208	0.964555902	1.981050475
9284	ASXL1	19.5202405	-0.504522716	0.675885714	-0.746476272	0.455279699	1.499800945
9285	C16orf87	12.41198002	0.822752827	0.822226926	1.001857042	0.216412642	1.140592782
9286	C6orf47-AS1	22.49115741	0.085284225	0.611861297	0.12928504	0.889145894	1.978115219
9287	CCDC14	20.49557527	-0.402285666	0.644522605	-0.625867267	0.52140194	1.011920412
9288	CDC7	16.12627478	0.182421174	0.726502811	0.251108697	0.801720072	1.499800945
9289	CDCA7	14.42272125	1.091917505	0.772464494	1.412550414	0.157492949	1.499800945
9290	CERS6	19.59802851	0.157225188	0.645945264	0.24255808	0.807572098	1.011920412
9291	CHST2	19.28267746	-0.020055772	0.659289785	-0.020420269	0.97572188	1.991241001
9292	CIR1	20.67821855	-0.187262601	0.522402295	-0.251919221	0.724898844	1.989504454
9293	CKAP2L	19.79218052	0.271710924	0.655718228	0.414271404	0.678602128	1.51109119
9294	CNOT11	19.56416454	-0.059957126	0.647909224	-0.092529278	0.926269506	1.981050475
9295	CNOT6	16.6216011	0.09116449	0.719025724	0.126788912	0.899107471	1.501424211
9296	CNOT6L	20.27242022	0.26766598	0.658408219	0.40652492	0.684249598	1.755910104
9297	COX18	12.96266284	0.564926827	0.812425155	0.695250052	0.486825955	1.758755451
9298	CPEB4	18.56500586	-0.141942851	0.672060026	-0.210892202	0.822970521	1.499800945
9299	CXorf56	18.59042285	0.088114566	0.664178772	0.122666941	0.894456789	1.741590797
9300	DBT	25.89626429	-0.522946629	0.562921812	-0.946845158	0.242717687	1.14241912
9301	DDX11	19.85977828	0.045062047	0.655164092	0.068781214	0.945162692	1.720551148
9302	DDX21	19.57545586	0.012497727	0.646400408	0.019224242	0.984574288	1.011920412
9303	DDX58	24.95922202	-0.207794579	0.570684488	-0.264114644	0.715772402	1.011920412
9304	DHX22	22.05124627	0.140599129	0.595250596	0.226161901	0.812207042	1.14241912
9305	DPP9-AS1	18.85788428	0.254266822	0.655228777	0.540820897	0.588621028	1.729008147
9306	DTNB	17.419856	0.806919622	0.687995974	1.172855165	0.240852892	1.011920412

9307	EOGT	18.7845158	-0.021210965	0.672522246	-0.021528904	0.974829767	1.741842494
9308	ERC1	21.581526	-0.451868466	0.51647925	-0.874901222	0.281627599	1.978115219
9309	ERMAP	16.58922121	0.096907507	0.71782189	0.125002162	0.892610165	1.989504454
9310	ETV2	19.78924505	-0.229025562	0.649850924	-0.267815986	0.712010448	1.011920412
9311	FAM174A	26.15810416	-0.615520912	0.562076429	-1.095084014	0.272479821	1.750199471
9312	FGGY	21.12211825	0.702741914	0.620512716	1.114554522	0.265041268	1.140592782
9313	FKBP8	27.79654105	0.041621474	0.580882274	0.071652168	0.942878722	1.991241001
9314	FZR1	18.25967265	0.126079771	0.671224687	0.187804642	0.851029796	1.747444115
9315	GADD45B	12.42889197	0.820106929	0.802429271	1.020758091	0.207269065	1.978115219
9316	GMPPB	26.90954962	-0.682062292	0.558045629	-1.222225885	0.221618427	1.741590797
9317	H2AC6	18.60171518	0.16442542	0.662984452	0.248007958	0.80412825	1.508154944
9318	ISY1	24.72702781	-0.182070244	0.572488828	-0.219222158	0.749558056	1.499800945
9319	JOSD2	26.0254864	-0.471702682	0.496268427	-0.950209586	0.24195497	1.741590797
9320	MANBAL	24.4696774	-0.026125912	0.580108056	-0.062291692	0.950220544	1.991241001
9321	MAP2K5	25.05806955	0.072288227	0.588924826	0.122916088	0.902172546	1.011920412
9322	MBNL2	29.22822076	-0.667990819	0.520551118	-1.259050819	0.208011977	1.747444115
9323	MCUR1	26.56106059	0.222298164	0.581786812	0.299282996	0.689682958	1.741842494
9324	MED11	22.97081252	-0.541516201	0.512888248	-1.05581692	0.291051862	1.741842494
9325	MED19	22.2228055	-0.257168542	0.592252695	-0.424220242	0.664128515	1.151127805
9326	MGMT	19.06028212	-0.442601606	0.68821248	-0.644570709	0.519205422	1.51109119
9327	MINDY2	27.19287204	-0.649791745	0.564794861	-1.150491602	0.249941451	1.499800945
9328	MIR222HG	24.29528282	0.282862148	0.584920758	0.48529188	0.627469214	1.750199471
9329	MKNK1-AS1	19.84291629	0.265680214	0.628460222	0.416126422	0.677217496	1.512917427
9330	NME4	25.26240276	-0.124021772	0.574787094	-0.215787229	0.829152526	1.499800945
9331	NUDT2	22.25478557	0.181957927	0.616622262	0.295087621	0.767926928	1.14241912
9332	OBI1-AS1	20.0924149	0.298711966	0.624549772	0.470746212		

						0.627821907	1.750199471
9333	PARP2	24.44694457	-0.696441998	0.59179282	-1.176822157	0.229262505	1.140592782
9334	PCGF6	16.1522268	0.451182287	0.716467201	0.629722485	0.52886897	1.991241001
9335	PCNX2	21.6155001	-0.029782125	0.52450648	-0.074429659	0.94066851	1.750199471
9336	PEX2	22.46280294	-0.560645298	0.612960882	-0.912161267	0.261157722	1.741590797
9337	PHF22	21.02222167	0.154275266	0.624757056	0.246926572	0.804957212	1.984471207
9338	PLPP6	25.72779057	0.04975816	0.567526652	0.08767546	0.920124625	1.755910104
9339	PRR26	20.5209422	-0.405828502	0.642965227	-0.620218028	0.528551929	1.14241912
9340	PRXL2C	27.61871519	-0.742886272	0.549022081	-1.252108186	0.176021071	1.499800945
9341	PTCHD2P1	22.56169064	0.225255725	0.602575862	0.556271	0.577957259	1.989504454
9342	RECQL5	26.62099802	-0.268655052	0.566407912	-0.474212725	0.625276199	14.00945117
9343	RHBDF2	27.01977771	0.285715256	0.565680882	0.505082219	0.61250109	14.42914159
9344	RMDN2	19.51059222	-0.127952291	0.661761884	-0.192250952	0.846684124	10.50470515
9345	RNF125	22.41479602	-0.287582684	0.527621758	-0.545044202	0.585722048	18.15107797
9346	RPL22P2	20.81944226	-0.021266019	0.627897426	-0.02286862	0.972981917	14.42789572
9347	RUFY1	28.2449429	-0.225470477	0.552018548	-0.589600618	0.555458427	10.81141021
9348	SERAC1	26.08195029	-0.785942787	0.57626774	-1.26285005	0.172614764	14.1184918
9349	SIRT7	19.2898199	0.116769122	0.679517596	0.171841221	0.862562258	15.91445255
9350	SLC12A7	22.06527282	-0.111185579	0.611525426	-0.181812798	0.855728856	11.00554415
9351	SLC25A1	24.47812228	-0.026881422	0.57804789	-0.062802419	0.949126756	9.579418252
9352	SLC29A2	27.88616844	-0.127297049	0.522552262	-0.24279725	0.80728785	11.19824155
9353	SMDT1	22.75140272	-0.250427486	0.522487827	-0.670709266	0.502405695	12.49125581
9354	SNX20	20.5661085	-0.126681199	0.625752284	-0.199261854	0.842057917	1.148157207
9355	SOX12	22.22869221	-0.282699192	0.518078885	-0.545668227	0.585294009	19.87441901
9356	SRD5A1	25.94710026	-0.527679878	0.56688604	-0.948479658	0.242885224	8.811551141
9357	STAC	16.92169424	0.821116205	0.697485757	1.191588782	0.222422525	1.0174588

9358	SYNE2	22.99767492	-0.294006822	0.595942292	-0.492246912	0.621767487	1.0174588
9359	TAF5	15.90567276	1.090622201	0.751922712	1.450457982	0.146920848	2.517814495
9360	TBP	19.12529484	0.286040824	0.654195745	0.59009987	0.555122696	10.82772151
9361	TDRD6	11.67545249	0.881227257	0.92018066	0.957787199	0.228170076	10.52922149
9362	THADA	22.01826255	-0.210067267	0.612908461	-0.505894904	0.61292042	14.49051449
9363	THBS2	15.58165649	-0.267588797	0.785819557	-0.240521926	0.722462506	12.79849814
9364	THEM6	29.59742542	-0.188461177	0.546628109	-0.244770274	0.720267007	2.008241502
9365	TMEM147-AS1	22.55601995	0.206997654	0.600609505	0.244645985	0.720260521	8.577714449
9366	TMEM19	22.25289419	0.050826041	0.592275404	0.085821761	0.921600158	1.744218974
9367	TMEM44	29.61698249	-0.741292862	0.522451822	-1.289802822	0.164588761	11.09104527
9368	TP52INP1	20.25910221	-0.744091529	0.685051949	-1.086182647	0.277298192	2.498047191
9369	TRIM11	25.925859	-0.422420127	0.561076298	-0.75287466	0.451525227	2.498047191
9370	TRIR	27.97277109	0.124724122	0.552498749	0.225255745	0.821702527	4.99528754
9371	TRMT1L	27.01897428	-0.498072777	0.492888505	-1.010518142	0.212247112	5.104191214
9372	TSEN2	14.41954461	0.552522922	0.767185276	0.721511412	0.470594922	5.4100805
9373	TSSC4	41.9120958	-0.681277642	0.472872124	-1.440719722	0.149662878	2.954424118
9374	UBA5	22.72214987	-0.072152725	0.601649264	-0.121586989	0.902226118	2.491294498
9375	URB2	19.54720264	0.162207002	0.651629842	0.250455826	0.802224854	5.957784824
9376	WWP1	20.78566851	0.190440762	0.620619162	0.20199012	0.762659587	8.147240795
9377	ZDHHC9	22.59556462	0.527244614	0.608251602	0.866677447	0.286118766	7.42785774
9378	ZNF226	28.92209278	-0.124174774	0.529926948	-0.229980129	0.818107202	5.441712111
9379	ZNF252P	24.72574648	-0.24077221	0.572171424	-0.420070224	0.674424081	5.479085047
9380	ZNF224	20.61704462	0.721911908	0.625257125	1.126408991	0.255785425	9.84711894
9381	ZNF282	22.52498122	0.081914751	0.599770125	0.126576908	0.89126522	1.981942505
9382	ZNF421	24.96221844	0.022281267	0.57899012	0.040210201	0.967925465	1.719409451
9383	ZNF526	17.09220262	0.520740646	0.716242211	0.727045457	0.46719811	

8.919894944
9384 ZNF680 16.82126261 -0.276721049 0.748806466 -0.502108702 0.614887849 7.440492087
9385 ZNF704 16.11657754 0.26567617 0.722276444 0.506212755 0.612707202 2.717477008
9386 ZNF710 22.00622102 0.082209221 0.596529028 0.127977945 0.890257848 5.988875517
9387 ZNF726 22.92764176 -0.215275869 0.591022141 -0.264226544 0.71568128 4.975590174
9388 ZSCAN22 21.28945081 0.118102878 0.617084422 0.191288522 0.848221211 2.489541151
9389 ABHD12 28.82026228 -0.194107568 0.499612812 -0.288515992 0.697624221 1.740448181
9390 AC005921.2 25.48002621 0.022072921 0.566772227 0.028946741 0.968922851 1.995074149
9391 AC018647.2 18.22420252 0.051159459 0.67561722 0.075722528 0.929629845 1.97145118
9392 AC096751.1 16.69651262 0.982220614 0.708787997 1.287185757 0.165285127 7.857217418
9393 AC100786.2 21.48064899 0.289408624 0.544552647 0.71509821 0.47454826 2.710211254
9394 ACTR6 26.02051782 0.185002292 0.565517086 0.22714006 0.742561954 5.189279259
9395 ADAM28 21.16522788 0.141262575 0.526528259 0.262287 0.792229279 7.891191594
9396 AL512550.1 20.82227662 0.296681874 0.626819207 0.622848945 0.526822291 4.875110954
9397 AP002490.1 16.10802026 0.277265196 0.724242282 0.277569429 0.705750479 1.507244489
9398 AP002108.2 21.28924961 0.050929281 0.61754719 0.082470265 0.924272762 5.451119119
9399 ARL2 22.49867087 0.082124825 0.591047227 0.128964886 0.889477897 4.44470714
9400 ARL5A 24.00817292 0.221122068 0.595564126 0.529189722 0.589755944 4.154444044
9401 ATPSCKMT 29.5047641 -0.029575082 0.552905429 -0.052292741 0.957418192 4.288150414
9402 BEX2 21.96742648 -0.271412059 0.621427281 -0.597677222 0.550055252 4.119574449
9403 BRCA2 22.78015794 0.048194628 0.584299757 0.082482727 0.924262852 4.148790724
9404 C9orf64 20.11240786 -0.222864224 0.520418521 -0.440905275 0.659281575 1.472490512
9405 CBFA2T2 22.11828994 0.04991212 0.526662058 0.094772415 0.924495598 10.22299918
9406 CCNG2 22.49956225 0.21225166 0.618611495 0.244887965 0.720178599 2.97914484
9407 CCP110 24.48255269 -0.512685825 0.590569148 -0.869814867 0.284401586 4.11841294
9408 CDC45 19.85982727 0.626142941 0.642612864 0.988295217 0.22295908 4.114144715

9409	CDK5RAP2	25.47157022	0.022799826	0.56828714	0.040112199	0.968002879	5.441440514
9410	CEP295	24.72007471	-0.064221279	0.574972247	-0.111868298	0.910927822	4.14100441
9411	CMSS1	20.84197287	0.254296908	0.624742626	0.40720208	0.682859574	7.419401741
9412	CNPY4	25.48828207	-0.219467692	0.571229547	-0.559165262	0.57604887	1.995074149
9413	DLEU2	16.60628215	0.809249105	0.769224262	1.052025971	0.292787624	1.148259941
9414	DLST	28.44909872	-0.487861748	0.550564609	-0.886111712	0.275557222	5.470419049
9415	DNAJC1	47.12296864	-0.26295288	0.477642421	-0.761978212	0.446072008	4.177194452
9416	DPY19L2	22.70190992	-0.65221588	0.622290454	-1.048249858	0.294522505	2.945140148
9417	DUOX1	15.16815265	0.777178422	0.746009592	1.041780727	0.297512247	2.129041444
9418	DXO	27.78529871	0.408999078	0.562258602	0.727292295	0.467046917	4.484499707
9419	E2F2	17.11558496	0.091792654	0.707761202	0.129694285	0.896808226	9.455971448
9420	EPAS1	26.49604679	-0.088227467	0.559248522	-0.157911222	0.874526676	8.17171872
9421	EPB41L4A	20.52222245	0.017858204	0.622851299	0.02817428	0.977522151	5.70171774
9422	EVI5	22.19250699	-0.521251917	0.627401692	-0.846749257	0.297124897	1.002521118
9423	FAM228B	25.21140222	0.445686461	0.578922215	0.769855516	0.441285604	2.509208455
9424	FASN	22.48751766	-0.56844752	0.51962715	-1.092952692	0.272975727	1.487547112
9425	FBXO20	16.15020025	0.271024464	0.721289702	0.270611622	0.710926822	1.487547112
9426	FCF1	21.28924961	0.050929281	0.61754719	0.082470265	0.924272762	4.92214011
9427	GAS6-AS1	18.27274819	0.442121054	0.666026747	0.662808801	0.506812692	9.422189494
9428	GGT7	19.87290288	0.558765227	0.640250184	0.872592222	0.28288482	4.128111215
9429	GINS2	18.86056847	0.122698789	0.665706227	0.18581588	0.852589151	1.981942505
9430	GNB2	19.12802899	0.286217608	0.655252969	0.589569275	0.555479456	4.121550421
9431	GON7	21.7526272	-0.145092942	0.614960861	-0.225928497	0.812480295	8.275114589
9432	GRINA	20.15260567	0.660262902	0.645778965	1.022428629	0.206578076	4.448971518
9433	GSR	22.50419128	-0.2495984	0.595672512	-0.586895997	0.557272558	1.151195188
9434	H2AW	22.51558282	0.080527522	0.587196697	0.127155952		

0.890907526 5.100710744
9435 HELQ 21.07829565 -0.055418912 0.621292229 -0.087772561
0.920057447 4.419417859
9436 HSF1 42.68422742 -0.615982892 0.470945215 -1.207971445
0.190882995 2.150252949
9437 KBTBD7 26.27275156 -0.064121416 0.569610887 -0.112588115
0.910257108 4.440444488
9438 KCMF1 17.12404094 0.090676672 0.710142212 0.127687849
0.898296007 2.711854274
9439 KCNK17 22.40260274 0.159550599 0.522690874 0.299518176
0.764544706 4.195891271
9440 KDM4C 21.07267501 0.012625208 0.625110172 0.021812488
0.982597522 5.421411887
9441 KHDC4 24.75941914 -0.480249287 0.597109219 -0.804290289
0.421229287 2.141897991
9442 LCMT2 21.81014299 0.280492896 0.612109186 0.620597284
0.524864627 1.504519141
9443 LIN54 16.62160002 0.520922784 0.708119625 0.749779791
0.452287242 5.498781189
9444 LMNB2 22.48449414 -0.222822204 0.588727287 -0.280198288
0.702798156 5.114028419
9445 LRWD1 16.66820922 0.24821482 0.722928179 0.481128915
0.620417772 2.129041444
9446 LSM12P1 21.08685175 0.25576202 0.619782565 0.574010089
0.565960974 5.144011412
9447 LZTR1 29.82649125 -0.405417024 0.520146622 -0.764726228
0.444424585 5.41450992
9448 MAD2L1 26.99709286 0.127227028 0.556284294 0.228726582
0.819081422 4.142271729
9449 MAP2K12 22.09267515 0.646518441 0.60222822 1.071764054
0.282826022 1.981942505
9450 MBD5 25.7501719 -0.222259888 0.56958647 -0.40776922 0.68244204
4.144108084
9451 MBOAT1 22.94201101 -0.906826069 0.626949228 -1.446410672 0.14806205
1.002521118
9452 MFN1 26.1975977 -0.222287622 0.557101652 -0.4009818
0.688422522 1.14521194
9453 MFSD4B 22.02572695 -0.487741012 0.621215924 -0.785129262
0.422271922 2.447121197
9454 MTFMT 24.24728098 -0.068275842 0.577744622 -0.118249594
0.905790662 2.97914484
9455 MX2 29.25441577 0.291202862 0.552078645 0.707499496
0.479256126 2.484715804
9456 MYO1C 28.24474158 -0.692988687 0.541484211 -1.279794581 0.20061729
1.129902942
9457 NAA25 24.60715419 -0.159242655 0.50906791 -0.212814169 0.75442185
4.494052028
9458 NCOA5 22.066162 -0.050982991 0.614929995 -0.082908609
0.922924209 5.918141147
9459 NCOA7 28.62244227 -0.608850912 0.526645278 -1.124550024
0.256562902 4.121400492

9460	NDST2	29.82629122	-0.706758592	0.526875254	-1.241415172	0.179785694	2.124117197
9461	NME2	26.95476291	-0.029629966	0.552296752	-0.052569745	0.957277961	1.991299484
9462	NRROS	27.20672416	-0.011165154	0.586296188	-0.019040291	0.984808964	5.490442798
9463	NSUN6	26.91521926	0.027097871	0.570846445	0.047469622	0.962128922	2.141847919
9464	NT5DC2	19.26425077	0.045891592	0.665671999	0.068940247	0.945027182	5.907000904
9465	P2RY1	26.72291258	-0.22992814	0.55922084	-0.589862226	0.555282014	4.715041405
9466	PIBF1	24.62262952	-0.291269726	0.605004582	-0.481599222	0.620090679	1.479111145
9467	PIGQ	20.99278592	0.159242574	0.657015846	0.242524705	0.808272617	4.477278445
9468	PIM2	22.55596881	0.226282685	0.601471895	0.559267502	0.57597917	2.714441441
9469	POC5	25.5522948	0.298942471	0.575112181	0.519800278	0.602202786	1.74457147
9470	POMGNT1	26.48754075	-0.251658209	0.561870096	-0.447894114	0.654229617	5.447842784
9471	POP1	18.05271644	0.095502128	0.694974182	0.127419678	0.890699078	5.878797411
9472	R2HDM2	25.11660718	-0.116190422	0.510110562	-0.227774978	0.81982118	5.498821251
9473	RAB11FIP1	22.82292582	-0.708444655	0.507517991	-1.295900575	0.162744427	4.14100441
9474	RCBTB1	18.60728468	0.242222965	0.662964411	0.264829742	0.715228502	1.980108159
9475	RELB	19.24475265	0.654668591	0.664202942	0.985497052	0.224279979	4.142271729
9476	RTP4	16.91880785	0.925405225	0.700056894	1.221900029	0.18620144	4.401217114
9477	SCRN2	21.14887894	0.417957567	0.648779885	0.644220909	0.519422194	4.922110158
9478	SCYL2	20.05665444	0.159079262	0.62906192	0.248926266	0.80241774	5.454829884
9479	SERINC4	19.54441616	0.228224208	0.661569692	0.260089261	0.718780208	7.404211151
9480	SLA2	20.1187217	0.441969929	0.627006248	0.692822554	0.487792869	4.419189142
9481	SLC20A5	16.26981019	0.40522299	0.71959928	0.56227727	0.572246025	4.454558421
9482	SLFN12	21.09520772	0.254807911	0.620648798	0.571672598	0.567542802	5.182758717
9483	SMG9	25.21428774	0.681684489	0.588225798	1.15868526	0.24658446	9.822001148
9484	SMIM4	20.08202227	0.156222221	0.626284082	0.245484189	0.806081549	2.987711818
9485	SNIP1	25.64576469	-0.626818222	0.571827666	-1.096166502		

						0.272005916	4.715091447
9486	SORD	24.72992214	0.242127972	0.59660497	0.575124282	0.565200484	2.475424479
9487	SPRY2	22.89882994	-0.149241587	0.517471464	-0.288405444	0.772026401	1.991299484
9488	SSRP1	27.95675794	0.07495426	0.548788267	0.126581282	0.891261694	1.154020425
9489	ST7-AS2	16.87070708	-0.026098218	0.72814926	-0.049575427	0.960460721	2.458511511
9490	STAG2L2	27.20809776	0.049965079	0.552808264	0.090220882	0.928111691	1.504519141
9491	STK26	17.0702696	-0.498687987	0.7762044	-0.64228717	0.520621828	4.417745111
9492	STYX	22.69828222	-0.422990894	0.592258202	-0.722772002	0.46269689	2.952848842
9493	TAB2	27.72147718	-0.422881906	0.564241506	-0.75124198	0.45250704	4.198474454
9494	TAF8	27.18267977	-0.107027427	0.551226797	-0.194122497	0.846079184	2.11921524
9495	TMEM129	22.99682254	0.080942572	0.584140586	0.128568651	0.889791017	4.944198849
9496	TMEM121	27.19954166	-0.268112446	0.548762225	-0.488578529	0.625140117	10.82504424
9497	TMEM170A	26.65621482	-0.161292471	0.562665461	-0.286825575	0.774228222	5.14484477
9498	TMEM68	20.20710288	-0.020201821	0.625495871	-0.02194644	0.974514764	10.4017972
9499	TNFAIP6	17.60250526	0.255695127	0.687462862	0.27192975	0.709927708	5.470914495
9500	TNFRSF8	20.58595486	0.726607072	0.650542571	1.116924712	0.264026517	5.419842124
9501	TNIP2	17.62921468	0.584759771	0.681054979	0.85860876	0.290556402	2.114479994
9502	TRAPPC12-AS1	24.26707825	0.16766002	0.580221258	0.288958785	0.772612917	1.721444998
9503	TREX1	20.22122955	0.120168189	0.622289756	0.190052406	0.849268065	1.498858448
9504	TRMT12	22.82616456	0.225159427	0.60024808	0.291770412	0.695227859	10.85751888
9505	USF1	21.55954526	-0.051872746	0.618051082	-0.082929545	0.922112449	9.119557457
9506	VASP	27.00549978	-0.02271996	0.558694229	-0.060254925	0.95187295	5.452954474
9507	WASHC2A	22.57576618	0.596168512	0.609581152	0.977996956	0.22807582	4.177194452
9508	WASHC2C	18.20600024	-0.024126408	0.684059972	-0.025269424	0.971864896	1.72804542
9509	WIPI2	27.41900154	-0.242040177	0.545426416	-0.628929426	0.529288712	4.444127181
9510	WWC2	27.65542244	-0.259726267	0.545414682	-0.476199626	0.622922166	5.974748855

9511	XXYLT1	21.12752756	0.14228125	0.661026975	0.216906957	0.828280855	7.428145144
9512	YAF2	21.59062405	0.279927877	0.61517972	0.455024206	0.649084558	5.171447401
9513	ZBTB11	24.70206262	-0.415928289	0.576595194	-0.721252225	0.470692765	1.510149774
9514	ZNF549	22.65211802	-0.682557926	0.609798502	-1.120957059	0.262206141	7.290144518
9515	ZNF550	21.21471754	0.048219196	0.618206454	0.078147649	0.927710605	2.741895005
9516	ZNF618	25.71246258	-0.457122272	0.569422224	-0.802784289	0.422099267	8.42990182
9517	ZNF641	20.00855267	-0.628519646	0.688285664	-0.927560929	0.252625272	4.111159204
9518	ZNF668	24.0984044	0.422780212	0.596992119	0.726608297	0.467465882	2.717477008
9519	ZNF789	24.2811049	-0.071252427	0.582219559	-0.122222067	0.902642165	5.450881745
9520	ZSCAN26	27.88050295	-0.286244929	0.542572716	-0.710751142	0.477228462	2.741844942
9521	ZSWIM1	21.2592297	-0.200572117	0.522546892	-0.282827545	0.701098859	4.444289878
9522	ABHD12	18.08642916	0.012179122	0.691260941	0.01761617	0.985945057	2.714491712
9523	AC009122.4	22.01527599	0.085445182	0.612127152	0.129257271	0.889167756	1.719409451
9524	AC010220.2	24.24155915	-0.067289415	0.578245747	-0.116520982	0.907229657	5.701447498
9525	AC012254.2	21.989958	-0.108556529	0.61824661	-0.175559249	0.860640141	4.441419197
9526	AC022509.2	28.42940041	-0.126244049	0.52767107	-0.224982907	0.814221224	5.191114705
9527	AC090425.2	22.5289056	0.081595221	0.599252589	0.126161822	0.891692247	7.218117211
9528	AC090617.2	28.12094405	0.082720862	0.509514206	0.162252285	0.871028264	2.444491075
9529	AC221522.1	20.26072209	0.62102755	0.624175272	0.979267998	0.227447578	2.992292511
9530	ADAP2	22.80228059	-0.222471152	0.626065159	-0.522646079	0.594278599	7.170724701
9531	ADM	21.65464212	-0.455651228	0.52172746	-0.872251075	0.282471749	2.489511089
9532	AGPAT4	20.294547	0.616749884	0.62822296	0.966228242	0.222874955	5.490214211
9533	AL049780.1	22.98628122	-0.102818857	0.599068246	-0.171621262	0.862727428	5.110417988
9534	AL122800.1	22.90082289	0.152745505	0.520616204	0.295214427	0.767752697	1.981942505
9535	ALDH6A1	29.4594976	-0.271252928	0.529001181	-0.502426612	0.614657226	14.18551877
9536	AP002064.1	25.47708967	-0.090282107	0.564054822	-0.160226287		

0.872694867	4.419417859
9537	APOO	22.52472886	-0.227661294	0.608804247	-0.290272846
0.696260125	1.980108159
9538	ARRDC1-AS1	22.67097024	-0.065766158	0.657296428	-0.100040224
0.920212204	2.147518412
9539	ASB1	22.94682679	-0.026192272	0.626108726	-0.057806849
0.952902482	8.874112402
9540	ATE1	26.10507654	0.457626156	0.590587728	0.774882604
0.428409022	2.702250224
9541	ATOH1	18.72417224	0.867669612	0.74507646	1.164527679
0.244206182	4.114094452
9542	ATP5PO	21.78182222	0.498762887	0.549772077	0.907217726
0.264291666	4.941512544
9543	ATXN7L2	24.46106909	0.024508229	0.587519644	0.041714909
0.966725968	2.714441441
9544	BCL6	28.20802118	-0.626402427	0.552252672	-1.152272274
0.249167678	7.457455104
9545	BICRAL	22.19424976	-0.62620728	0.61884785	-1.028212799
0.202849729	8.141440029
9546	BLM	17.40547688	0.21091062	0.716210627	0.294440162
0.768421581	1.154020425
9547	BORCS8	19.79192806	-0.091811651	0.655109664	-0.140146995	0.88854285
5.149421055
9548	BRF2	29.45959772	0.02222228	0.524592899	0.042457997
0.965226447	4.447155028
9549	BYSL	21.62947444	0.096691461	0.525175518	0.18067242	0.8566247
4.949919481
9550	CDC14A	25.07477911	-0.042282004	0.601577616	-0.07211529
0.942510079	7.905118142
9551	CDC25B	25.47992501	-0.024015702	0.564462288	-0.060262125
0.951946867	7.912714182
9552	CEP82	19.02481191	-0.122129069	0.680192697	-0.195722292
0.844827526	5.911471597
9553	CGRRF1	26.96211869	-0.082250402	0.549745006	-0.15161648
0.879489425	4.444419815
9554	CHSY1	26.16078718	-0.500228078	0.56271888	-0.888966224
0.274021222	4.928880851
9555	CLN2	12.51051414	1.164024161	0.849721002	1.26987271
0.170726228	2.129041444
9556	CPNE8	22.07661192	0.291212252	0.594792401	0.657729644	0.51071187
8.112484819
9557	CRACR2A	25.16724925	-0.287704252	0.577412971	-0.498264445
0.618297665	5.490214211
9558	CRTC2	21.52979698	0.218649286	0.618244106	0.252604707
0.722625147	4.955791809
9559	CTU2	21.52855571	0.254984845	0.625557892	0.558540522
0.576475224	4.442201825
9560	CUL4B	19.61768255	0.529202982	0.644177176	0.821517747	0.41125142
8.844921178
9561	CYB5A	25.82262952	0.667920129	0.578687542	1.154198214
0.248418915	4.407997918

9562 DENND10 25.54200227 0.186840902 0.569662416 0.22798472 0.742922208 7.941017588
9563 DFFB 19.6148482 0.4540227 0.644122722 0.704858917 0.480898029 2.741895005
9564 DNAH14 19.21629898 -0.172161901 0.671488182 -0.256288579 0.797650806 5.982154895
9565 DPEP2 27.21928789 0.049492022 0.550025789 0.089982095 0.928200649 4.104247249
9566 DTWD1 27.28219102 -0.610784501 0.55122852 -1.10782118 0.267929051 4.194994018
9567 EGFEM1P 18.81251668 -0.099866979 0.678977122 -0.147084451 0.882065262 1.498858448
9568 EIF2AK4 21.08609741 -0.222677242 0.526507572 -0.424822149 0.67095904 2.97914484
9569 EXT1 25.49967222 0.020858656 0.56422116 0.026962285 0.970514997 4.920414872
9570 FBXO25 26.54116095 0.225074125 0.562746222 0.416985712 0.676688858 1.141484412
9571 FBXO6 21.75642151 0.054054284 0.62155126 0.086966721 0.920697965 4.710944995
9572 FNTA 18.54510622 -0.128211521 0.692792974 -0.19964228 0.841759499 4.170472274
9573 FXN 19.27007045 0.810022971 0.66109848 1.225282266 0.220468472 1.501492795
9574 GEMIN7 24.08691175 -0.046948554 0.614965204 -0.076242419 0.929145882 4.115149457
9575 GORAB 17.1427271 0.242762421 0.702812542 0.487009528 0.626251572 4.715091447
9576 GPATCH2 25.92854201 -0.208650209 0.560751482 -0.550422625 0.582029527 1.742724214
9577 GPR182 16.27542975 0.77192042 0.745862726 1.024924925 0.200699202 2.152189214
9578 GTPBP10 12.42211901 1.061247878 0.85492715 1.241221255 0.214482266 5.421271815
9579 HAUS8 25.99211222 -0.256722284 0.565576145 -0.452914802 0.649890149 7.179140741
9580 HCG25 22.54940567 0.28599118 0.590582169 0.652576229 0.512284799 4.917589514
9581 HMMR 18.41040627 0.925068065 0.67118759 1.29215458 0.162572051 5.921418901
9582 HNRNPA0 29.88707597 -0.607682662 0.529767694 -1.147072828 0.251251105 4.119745247
9583 JRK 25.10024717 0.200509121 0.596880575 0.502466075 0.614626626 2.475484541
9584 KDM1A 25.65421959 -0.227576286 0.571947904 -0.590222088 0.555041766 1.988544127
9585 KDM4A-AS1 24.2178622 0.647607247 0.591892724 1.09412758 0.272899028 5.890088947
9586 KIAA0920 24.88082725 0.567209602 0.606558828 0.925291958 0.249627858 8.424121124
9587 KNSTRN 21.10276262 0.707462677 0.628027022 1.126466497

0.259968114	4.717917014
9588	LACTB2 21.61022021	0.481941901	0.615641457	0.782828862
0.422727616	8.114718081
9589	LLPH	20.40578827	0.468906299	0.652725824	0.717271984	0.47220629
5.102504021
9590	MAML2	29.62250085	-0.586727054	0.528188941	-1.110827979
0.266642401	8.70759971
9591	MAP4K5 21.79024542	0.050598626	0.610982468	0.082815185
0.922998495	1.504519141
9592	MECR	21.26196285	-0.200274621	0.519150622	-0.285966272
0.699521647	2.948118111
9593	MED18	27.62711896	-0.262795284	0.548591781	-0.661221025
0.508406455	2.441257849
9594	MED9	29.2665219	-0.116941264	0.544214825	-0.214841221
0.829891114	4.442554521
9595	MIR22HG	18.61285417	0.220226792	0.665546022	0.481214262
0.620292162	1.742482417
9596	MMAB	24.26976242	-0.010429927	0.578174117	-0.018029422
0.985607404	4.489525054
9597	MT1F	20.82496072	0.187262067	0.624809672	0.299710577	0.76429792
1.725180245
9598	MTRES1 21.57645624	0.282012218	0.612512251	0.460418229
0.645216046	2.484715804
9599	MYO1D	26.22276421	-0.619291999	0.582220756	-1.061648119	0.28829546
4.488719821
9600	NAB1	17.88677665	0.452026002	0.680022227	0.666206658
0.505279018	1.742724214
9601	NAPA-AS1	29.99720406	0.261292626	0.541210784	0.482702549
0.629206242	2.727114211
9602	NCAPG	22.52199264	0.082256522	0.601529224	0.128408085
0.889917908	1.721444998
9603	NEIL1	22.49279026	0.022018468	0.609997895	0.026095974
0.971205822	4.491117491
9604	NFKBIZ 27.42297695	-0.450860254	0.548850148	-0.821462299
0.411282421	4.95584187
9605	NMRK1	22.84222205	-0.546807194	0.505200608	-1.082142262
0.279189274	2.481105172
9606	NSMCE4A	20.82620198	0.047262927	0.622950042	0.07482887
0.940250871	5.459415149
9607	OXR1	25.96664517	-0.596025991	0.580214878	-1.02709066
0.204277776	9.412879878
9608	PAN2	25.91595926	-0.419999448	0.564205998	-0.744407981
0.456629692	14.51744117
9609	PAQR7	22.45051896	-0.470049187	0.602474818	-0.780197225	0.42527479
9.88447241
9610	PARP2	24.26697715	0.108652215	0.577876602	0.188019922
0.850861022	5.925154148
9611	PGBD2	19.99447598	-0.192652062	0.669878187	-0.28908698
0.772514817	1.011988104
9612	PIAS4	27.50077497	0.020442972	0.550948741	0.027106849
0.970299811	4.95584187

9613	POT1	28.18162712	-0.411996201	0.541649598	-0.760622525	0.446876587	5.114797254
9614	PPP2R2A	22.05402928	0.267002574	0.592946861	0.450299229	0.652494622	7.480127422
9615	PPP2R5A	26.41128568	-0.467212722	0.558571421	-0.826442229	0.402906164	4.712800241
9616	PSPH	25.24242988	0.861929029	0.590757122	1.459024221	0.144558411	1.111991004
9617	PUS2	25.55229272	0.58805012	0.57884448	1.015902476	0.209675252	5.728214958
9618	RAB22	25.58706745	0.069522858	0.601879602	0.115509577	0.90804122	1.74457147
9619	RGL4	28.22819288	0.14904855	0.545250828	0.272207629	0.784616744	2.141897991
9620	RNMT	27.48286201	0.021800524	0.547822829	0.029794789	0.968256721	4.878004245
9621	RPTOR	22.57546472	-0.242408704	0.62256624	-0.282609881	0.702009042	2.111150487
9622	RRP9	27.9284024	-0.180589922	0.540925269	-0.222847502	0.728494626	4.448911447
9623	RSKR	22.00219229	-0.104280215	0.595480777	-0.175287295	0.860852894	2.748515424
9624	SAAL1	21.2845465	-0.109725918	0.520422754	-0.210825919	0.822015209	5.498821251
9625	SAP120	20.16876229	-0.045611858	0.528128245	-0.086265117	0.921176176	2.120404444
9626	SCAMP1	25.70774075	-0.456222022	0.569510007	-0.801079906	0.422085288	10.11204544
9627	SEC24A	28.91905604	-0.272454089	0.522262765	-0.512756726	0.607422087	8.171778791
9628	SELENOI	17.86140871	0.456541842	0.682922146	0.667522827	0.504421204	2.740059458
9629	SLC19A1	18.28215202	0.602506271	0.667958572	0.902508524	0.266256061	2.709011019
9630	SLC25B2	28.84862292	-0.167929022	0.55596792	-0.202048058	0.762615428	4.119715185
9631	SPDL1	22.29602175	0.109811799	0.589996462	0.186122809	0.852248456	4.954945518
9632	SPRYD7	24.52209629	0.077749674	0.575084574	0.125196929	0.892456167	1.484781828
9633	STX11	29.01722954	-0.122898828	0.495481222	-0.270229964	0.786975649	4.897452545
9634	TANGO6	18.62255141	0.161886221	0.681552654	0.227525772	0.812248927	4.140184494
9635	TBC1D17	21.25142579	0.662809271	0.622224456	1.066661072	0.286124912	4.180081041
9636	TBC1D22B	27.42457104	-0.289775098	0.544826848	-0.521856645	0.594825287	4.975590174
9637	TFAM	22.70195164	0.221752206	0.527646224	0.420268915	0.674289016	7.145214705
9638	TMEM125	29.62279217	-0.526850098	0.527956449	-1.01684542		

0.209226971 1.988544127
9639 TMEM186 18.8251494 0.281150182 0.664668522 0.422992075
0.672200212 4.950171177
9640 TMEM242 20.2224419 -0.081481545 0.685572422 -0.118851667
0.905292875 4.180081041
9641 TMSB4XP1 24.67764256 -0.294722622 0.5825201 -0.505954222
0.612888715 4.954945518
9642 TOMM70 21.06406778 -0.259677809 0.645471544 -0.402207128
0.687458002 1.725180245
9643 TRABD2A 22.26182574 0.565686505 0.607997605 0.920409101
0.252159208 4.455998545
9644 TRMT10B 21.08962596 0.566102616 0.625090116 0.905622606
0.265129815 4.928880851
9645 TUBGCP2 25.04020192 -0.657208966 0.491410674 -1.227596029
0.18102816 8.441427052
9646 UBOX5 22.76219485 0.171696965 0.586874288 0.292561694
0.769857197 8.850805404
9647 USP28 20.02112524 -0.125250809 0.652201689 -0.192166922
0.847611454 2.700545051
9648 ZMIZ2 21.42687645 0.025072256 0.522882285 0.047860577
0.961827258 4.454795795
9649 ZNF250 24.82442079 0.452621261 0.580580282 0.781241216
0.424601776 5.450881745
9650 ZNF282 16.94402452 0.558512119 0.727208246 0.767916554
0.442526762 2.455487175
9651 ZNF548 29.72128742 -0.196424087 0.540099955 -0.262699506
0.716082412 4.119715185
9652 ZNF577 17.59111284 0.092479215 0.701470187 0.122261849
0.892986298 4.941442501
9653 ZNF586 24.10909157 0.204281256 0.547827261 0.555605245
0.578480745 7.92042414
9654 ZNF587B 25.42480978 -0.086829247 0.57522668 -0.150947878
0.880016822 4.101541044
9655 ZNF677 22.20726214 0.257752969 0.591489107 0.604824417
0.545289022 8.144171751
9656 ZNF814 25.75012076 -0.119284518 0.564126981 -0.211449766
0.822526227 9.277110494
9657 AC002250.2 24.09526666 0.252016526 0.596286597 0.424220221
0.671222214 5.470914495
9658 AC008124.1 21.2826267 0.292282226 0.644785815 0.610097524
0.5417972 4.474205724
9659 AC010542.2 27.88870747 -0.652497817 0.550565808 -1.186956777
0.22524467 2.952848842
9660 AC010618.2 29.4227872 -0.218989812 0.527466484 -0.415172942
0.678015206 5.949220858
9661 AC015812.5 22.45897287 -0.158826906 0.601681829 -0.262971581
0.791801817 2.441257849
9662 AC021729.2 27.18804794 -0.427966071 0.551782579 -0.775604942
0.427982281 9.828712014
9663 AC022908.2 18.25658277 0.049759618 0.691589992 0.071949592
0.942642022 8.40421518

9664	AC026102.1	27.01942406	-0.022955524	0.558492961	-0.060798272	0.951519786	5.492111719
9665	AC058791.1	22.51259515	0.081822745	0.588827295	0.128956424	0.889484576	1.51198511
9666	AC090984.1	22.29954716	-0.144722566	0.615529162	-0.225125189	0.814102807	2.444978501
9667	ACBD2	26.4524571	-0.096622822	0.529711919	-0.182408265	0.85526224	4.11185229
9668	ACSM2	24.29711125	-0.650274491	0.492249699	-1.218550202	0.187219521	2.970740799
9669	ADAMTSL4	22.09919251	0.846521249	0.609061669	1.289894146	0.164561022	2.704185481
9670	AFG2L1P	27.22197198	-0.109205026	0.546949686	-0.199661941	0.841744981	4.500011157
9671	AL021775.1	24.52990705	0.017929949	0.57904709	0.020964578	0.975297789	8.280897182
9672	AL157786.1	28.18162605	-0.152995022	0.527845601	-0.284459018	0.776058626	5.001008171
9673	ANKRD54	24.21700874	-0.220594529	0.501245859	-0.44000471	0.659922696	4.944551545
9674	AP2A1	26.55164788	-0.20456947	0.487756495	-0.624429248	0.522245657	5.928089595
9675	ASB16-AS1	24.20282201	-0.422719227	0.492962262	-0.87601502	0.281021868	4.955891921
9676	ATXN7	28.10254671	-0.671999262	0.548964204	-1.224122269	0.220906069	2.124117197
9677	ATXN7L1	25.76981692	0.047550249	0.562155478	0.084425285	0.922710275	11.05729144
9678	BAG2	21.2908192	0.720840954	0.622410818	1.174209915	0.240210956	4.944551545
9679	BANP	26.58242977	0.507547457	0.570722699	0.889289449	0.272847541	8.400444584
9680	BCORL1	18.10225004	0.411548416	0.685140552	0.600677257	0.548054904	2.129041444
9681	BUB1	25.42246721	-0.028651947	0.587992861	-0.04872821	0.961125815	8.282481548
9682	C12orf42	25.22666799	0.680209222	0.579107527	1.174754775	0.240092828	2.444192114
9683	C19orf28	29.12995874	-0.292262895	0.52005896	-0.741922927	0.458124024	1.942194101
9684	CACNA2D4	25.08606926	0.202428452	0.582727171	0.518116111	0.60427726	8.14411149
9685	CATSPER1	25.0965072	-0.616807414	0.501464284	-1.220012407	0.218692459	4.199879417
9686	CCDC127	24.95062252	-0.205647277	0.575208124	-0.257518022	0.720704029	7.451984411
9687	CD81	26.02429205	0.018294085	0.567015247	0.022262827	0.974261656	7.418297841
9688	CIDEB	25.52498912	0.122207511	0.564850856	0.22405728	0.814940422	11.87081414
9689	COMMD2	22.80621499	0.420219822	0.601016247	0.715820518		

						0.474102182	4.187425292
9690	COQ8A	28.90766251	-0.272217179	0.521710489	-0.702106102	0.482612997	4.92214011
9691	DIS2L2	24.0222894	0.018562824	0.582165842	0.021885801	0.974562122	5.19499999
9692	DTL	20.20690049	-0.161222245	0.652800657	-0.246760605	0.805092501	4.442504449
9693	EDRF1	18.8292775	0.052095181	0.676542272	0.077001994	0.928621959	5.100410411
9694	EEF2KMT	28.27206678	0.56109255	0.557502107	1.006428427	0.214204717	4.144477204
9695	ERVK2-1	17.85842211	0.050476952	0.705521521	0.071545589	0.942962542	4.125285978
9696	EXD2	27.91696081	-0.440826574	0.542855167	-0.81207022	0.416751277	7.48590845
9697	FAM2A	25.602175	-0.481929526	0.498184452	-0.967271668	0.222258268	1.751191201
9698	FBXO41	20.20142004	0.555757522	0.677457644	0.820257609	0.412012274	4.914804141
9699	FERMT1	27.64965047	-0.152617227	0.551222474	-0.27867906	0.780491122	1.124182221
9700	FIGNL1	18.85484556	0.512869129	0.666298791	0.769612949	0.441529521	4.44408711
9701	FKBP7	18.86225148	0.278280201	0.665646592	0.41805998	0.675902264	2.704185481
9702	FLNB-AS1	22.96086226	0.02570844	0.624054022	0.041195856	0.967129758	1.721444998
9703	FLYWCH2	25.47415212	-0.202192542	0.565528024	-0.259297026	0.719272892	4.729118229
9704	FOCAD	20.5771462	-0.055884479	0.644482284	-0.08671207	0.920900282	1.148259941
9705	GATB	20.90672414	0.672402028	0.626160057	1.058542296	0.289807777	8.415475025
9706	GCHFR	20.27678058	-0.251924104	0.519226669	-0.677772054	0.497916222	4.48284424
9707	GMCL1	21.46228445	-0.114741078	0.528128242	-0.217259878	0.828005822	5.195050051
9708	GNL2L	21.41821928	-0.019807202	0.518604219	-0.028192286	0.969522574	2.475424479
9709	GPALPP1	20.66120225	0.228742802	0.544151626	0.428744988	0.660846225	2.947915452
9710	GTF2H2	26.77191108	0.481051272	0.564828917	0.851676071	0.294292905	2.11921524
9711	HCFC1R1	25.67958645	-0.056842129	0.576666662	-0.098570166	0.921479559	4.488281245
9712	HDHD5	29.1552194	-0.020764048	0.511408772	-0.060155495	0.952021791	1.141484412
9713	HMGN2-AS1	28.22227192	-0.156766685	0.54448781	-0.287915877	0.772411122	7.444041012
9714	HOOK2	28.5272224	-0.622725679	0.479685672	-1.219062118	0.187148012	1.97145118

9715	IER2	102.7707422	-1.029276502	0.929218621	-1.106415298	0.268546807	4.190110478
9716	INTS8	19.27962865	0.121846771	0.68764288	0.191726996	0.847948225	1.510149774
9717	IPO12	16.15014792	0.262904602	0.740095917	0.490248066	0.62288762	9.142481494
9718	JPT2	21.01885124	-0.292742429	0.654896677	-0.601229862	0.547686897	1.154020425
9719	LSM6	27.44426722	0.182078702	0.561588571	0.226001475	0.744422229	4.941714198
9720	MAF	16.18685724	0.726744015	0.727556868	0.998882764	0.217851487	5.711959012
9721	MAGEH1	26.66162292	-0.659025996	0.568008952	-1.160228748	0.245951615	7.402982745
9722	MED7	29.80027557	0.225020225	0.558554122	0.420782424	0.67291222	4.891081975
9723	METTL2A	22.71602445	0.120524062	0.627021604	0.189199222	0.84992659	9.118144121
9724	MMEL1	15.42690574	0.6162822	0.752019999	0.818415581	0.412119928	4.441871892
9725	MRPL20	26.49022276	-0.141492812	0.556290962	-0.254252167	0.799222501	2.495121844
9726	MYO5A	24.71921917	-0.50666022	0.502552421	-1.008172892	0.212270989	4.191272284
9727	NCF1	20.27404526	-0.112728965	0.528582846	-0.212266005	0.821119488	1.755017449
9728	NEU2	25.45162052	-0.144518224	0.5679814	-0.254442002	0.799154106	8.29780914
9729	NRM	20.44726275	-0.208528522	0.520890611	-0.581171575	0.561124822	4.45101051
9730	NUDT9	22.55009572	0.081021672	0.600929274	0.124827278	0.892748428	1.51198511
9731	ORMDL2	22.57188712	0.446557799	0.588620075	0.758629114	0.448068468	4.958478117
9732	OSBPL11	19.20774181	-0.245714674	0.6871219	-0.257594624	0.720646702	4.118474011
9733	OTULIN	25.67660092	-0.628114245	0.580646202	-1.081750182	0.279262548	8.29780914
9734	PACS1	22.76666292	0.128886626	0.546888041	0.2256728	0.812686578	4.174411248
9735	PFKFB4	28.90487822	-0.272052522	0.521204287	-0.512046558	0.608618425	1.940240855
9736	PGM2L1	22.26860865	0.117269817	0.610599496	0.192056852	0.847697672	1.98571879
9737	PNKP	20.27682064	-0.208122287	0.52261678	-0.298252208	0.690444286	4.897702404
9738	POC1A	25.29707226	0.217167847	0.56975246	0.281161754	0.702082227	2.721552418
9739	POLR2GL	25.12749586	-0.661791046	0.517549725	-1.278700412	0.201002575	4.941714198
9740	PREP	48.92222097	-0.266894265	0.470820201	-0.779249687		

0.425822646	2.117771219
9741	PRR19	22.22546522	0.255287216	0.592782414	0.599522042
0.548824148	1.011988104
9742	PTPRA	28.27465182	-0.160060298	0.565908446	-0.282827972
0.777201044	4.722597707
9743	PTPRJ	22.92588928	-0.402258149	0.524761727	-0.768459528
0.442214226	8.117542419
9744	PYCR2	21.68851502	-0.089210516	0.519240262	-0.171776622
0.862612146	1.481944491
9745	R2HDM4	29.25406219	-0.152520266	0.551612279	-0.276499222
0.782164657	1.740900977
9746	RAB28	22.41524582	0.762245299	0.628067895	1.215227524
0.224279222	5.988815445
9747	RBM48	29.42282726	-0.07158294	0.529749542	-0.125126006
0.892512249	7.27411797
9748	RCC1L	24.02228952	0.28026986	0.587400602	0.647547617
0.517277587	2.129041444
9749	RETSAT	26.12119127	-0.669460286	0.578704518	-1.156825746
0.247242561	1.492127817
9750	RPP25L	25.00712021	0.251827789	0.574786824	0.428122795
0.661296546	4.491505117
9751	SEPTIN7P2	20.42756651	-0.211577127	0.522459649	-0.404962574
0.685504287	7.292019917
9752	SHCBP1	29.16114755	0.150994728	0.551916826	0.27258241
0.784405554	4.711104159
9753	SIRPB1	28.90209294	-0.171482752	0.524009241	-0.221122122
0.748117081	9.841155411
9754	SLC15A2	24.79901172	0.576451292	0.585165505	0.985108122
0.224570964	4.401217114
9755	SLC22A2	27.99529688	-0.521256209	0.475852749	-1.095412845
0.272225809	5.448222844
9756	SLC25A12	41.71895057	-0.207952429	0.481224229	-0.422025227
0.665715727	2.954484189
9757	SLC25A45	19.15891529	0.616277094	0.660248782	0.922259984
0.250685751	5.421271815
9758	SLC66A1	28.48859119	0.122922122	0.540409222	0.22747969
0.82005076	2.489172715
9759	SLFN11	17.15497729	0.600774882	0.699477968	0.858890257	0.29040101
4.495155485
9760	SORT1	22.0944142	0.886816778	0.622810227	1.401294288
0.161096199	5.441442151
9761	SQOR	21.44657262	0.161721291	0.524589669	0.208281502	0.75786814
5.100710744
9762	STK24	22.28071857	-0.284222281	0.507872656	-0.559621865
0.575720565	4.92214011
9763	TCF19	22.27504788	-0.274162497	0.50758785	-0.727140272	0.46102699
4.104247249
9764	THAP4	27.00524722	-0.20114921	0.568025167	-0.520168956
0.595994792	4.48559704
9765	TMEM107	26.68710099	-0.226266829	0.552405788	-0.589561649
0.555484569	4.871185409

9766	TMEM121L	20.54048704	-0.122296188	0.645052692	-0.189591005	0.849629626	1.994184749
9767	TMEM150A	27.42172461	-0.077890695	0.550997628	-0.141262021	0.887582158	12.20894174
9768	TMEM220	22.08202275	0.408145278	0.606202619	0.672280822	0.500768645	2.702250224
9769	TRADD	25.97221479	-0.198815152	0.559817264	-0.255142946	0.722482504	4.422415915
9770	TRIT1	22.24680661	0.608010007	0.594558552	1.022624271	0.206485529	4.444419815
9771	TTC7A	19.12911792	0.209705122	0.667801028	0.462768562	0.642812569	5.444958275
9772	TVP22A	27.02788004	-0.02465491	0.562270564	-0.061622868	0.950854405	2.954484189
9773	UBXN2A	24.78478492	0.105679596	0.572061959	0.184412165	0.852690127	8.414771158
9774	USP22	18.10224992	-0.068929564	0.709950955	-0.0970906	0.922654446	9.254488018
9775	WDR4	22.5280622	0.450105491	0.598226502	0.752299782	0.451810677	4.121081414
9776	ZC2H18	19.8709162	0.560252226	0.641074168	0.874085024	0.282071947	2.951012494
9777	ZC2H8	24.60770506	0.545727645	0.588522649	0.927282676	0.252779268	1.141484412
9778	ZKSCAN5	15.71127822	0.945625044	0.742284298	1.272781041	0.202741026	4.11924419
9779	ZNF126	22.88922012	-0.444508591	0.510099226	-0.871415912	0.2825271	4.901111591
9780	ZNF220	22.07084004	0.202942242	0.595051141	0.241051922	0.722064486	2.954424118
9781	ZNF226	21.12111145	-0.226294902	0.515499446	-0.428981852	0.660674685	1.980108159
9782	ZNF542P	26.72669572	0.102882666	0.554168424	0.187458652	0.85120104	4.471757812
9783	ZNF567	19.85126902	0.950976522	0.691470269	1.275296229	0.169029594	4.485544998
9784	ZNF597	17.62229166	0.502429402	0.689482182	0.728704262	0.466182524	2.941557517
9785	ZNF646	24.25006412	0.410262716	0.606285522	0.676572401	0.498677295	4.944198849
9786	ZNF654	24.21482654	0.164168102	0.582689528	0.281259291	0.778511522	2.945140148
9787	ZNF691	17.62498574	0.76054046	0.704555667	1.079461126	0.28028221	4.198714718
9788	AC005261.5	18.12990811	0.407262622	0.685500549	0.5941112	0.552427674	1.002521118
9789	AC012489.2	25.27448954	0.292567698	0.57251821	0.684490281	0.492665562	1.997010114
9790	AC068821.5	25.50519068	0.191066262	0.565744021	0.227725642	0.72556995	4.444127181
9791	AC109222.1	19.56290992	0.212027608	0.667224221	0.467580222		

						0.640084811	7.958929545
9792	AC124772.1	22.02980024	0.296122492	0.602826295	0.657110506	0.511109864	2.129041444
9793	AC127824.2	22.07168029	0.476686586	0.611220788	0.779752296	0.425526691	4.488281245
9794	AC145207.2	26.27800527	0.650526012	0.587406845	1.107470942	0.268090269	9.111101478
9795	ADORA2	29.52206797	-0.786776827	0.547421548	-1.427215009	0.150656909	9.422717205
9796	AL128756.1	25.22642882	-0.061857219	0.569170609	-0.10867975	0.912456504	8.417404405
9797	ARHGAP6	28.55240259	0.221411718	0.552702412	0.581526567	0.560885619	7.442478271
9798	ATF1	25.04925896	0.264704082	0.570722284	0.629021976	0.522808625	2.700514989
9799	BHLHE40-AS1	20.14871246	-0.478272821	0.524898161	-0.911174522	0.262202425	5.484755741
9800	C12orf45	24.58507127	0.548599519	0.579297195	0.947008761	0.242624215	4.44408711
9801	C4orf46	16.65954978	0.618788226	0.719055857	0.860556688	0.289482249	1.740900977
9802	CCDC127	22.27407075	0.175289912	0.542555916	0.222266057	0.746492757	7.947598158
9803	CCDC66	26.70968256	0.05178298	0.555547921	0.092212442	0.92572479	2.111100415
9804	CDKN2AIPNL	27.52026981	-0.190244798	0.565067166	-0.226852404	0.726227412	4.115428475
9805	CENPL	25.2220094	-0.291962749	0.574751218	-0.507982728	0.611465449	7.451984411
9806	CHMP1B-AS1	22.12171295	-0.217765121	0.507487125	-0.429104712	0.667847022	4.918441111
9807	CRAT	26.94520112	-0.614268272	0.480102092	-1.279659062	0.200665068	7.40970451
9808	CSPP1	25.21955471	0.272921085	0.572222572	0.476024084	0.624050096	5.957824898
9809	CUL2	24.94485162	-0.279978257	0.582456512	-0.652272064	0.514161181	4.918741125
9810	DCAF16	28.52524927	0.426518874	0.545569224	0.781786888	0.424229828	7.451024474
9811	DUS2L	27.8774078	-0.049159291	0.495000282	-0.099211622	0.920890851	7.414414477
9812	EBLN2P	27.19276762	0.105455425	0.558076555	0.188962292	0.850122271	1.980108159
9813	EEF1A1P5	20.79276975	0.122295789	0.628575251	0.191670081	0.848000642	4.198714718
9814	EPB41	24.01229084	-0.280277508	0.596001271	-0.470420978	0.628047127	4.475795921
9815	ERCC5	12.24095721	1.095171027	0.896972292	1.220962816	0.222100099	7.442175728
9816	FBXO8	24.02756757	0.019571267	0.58244168	0.022602278	0.972194206	10.81808424

9817	FHOD1	20.21455206	-0.74249167	0.529282212	-1.402824899	0.16066906	4.489484991
9818	GLCE	25.51642194	0.076866771	0.56272062	0.126252726	0.891541652	1.124182221
9819	HERC2	21.28052997	-0.254912252	0.655229192	-0.54157184	0.588112495	4.458494415
9820	HINFP	29.85598512	-0.256248745	0.526652861	-0.676629277	0.498641198	1.479111145
9821	HLCS	21.09490401	-0.261661922	0.675775772	-0.287202297	0.698606449	1.498858448
9822	HMGA1	51.56642424	-0.227258002	0.459728428	-0.711825198	0.476566824	2.150252949
9823	HOXA1	25.78115724	0.556578226	0.577010721	0.964589027	0.224750692	4.194158471
9824	HPF1	21.25220547	-0.29168848	0.512280717	-0.568282559	0.56984212	9.548417151
9825	HSPBP1	20.24476482	0.140571648	0.521226257	0.264567429	0.791242714	2.144482174
9826	IGHMBP2	22.09411275	0.012187215	0.628524258	0.020981061	0.982260764	2.481105172
9827	IKZF2	25.00120812	-0.442466651	0.59522648	-0.745028512	0.456248428	5.710172728
9828	IL10RB-DT	22.27412808	-0.012202476	0.611422805	-0.019958785	0.984076251	7.929091118
9829	ISYNA1	22.62819248	0.502782807	0.602660944	0.824547622	0.402972462	7.910928898
9830	JADE2	24.2244448	-0.184151022	0.585859225	-0.214226248	0.752272189	2.984887471
9831	JMJD8	28.92591578	0.075774541	0.527029087	0.141096882	0.887792406	5.470914495
9832	KIF2A	25.80075426	0.101284698	0.569782899	0.177759846	0.858911582	5.411474582
9833	KLHDC2	27.74186182	-0.622822254	0.472492999	-1.220275507	0.18674205	5.197885299
9834	LHFPL2	25.96922818	-0.480101672	0.577168615	-0.821822209	0.405509206	2.471599121
9835	MRPS9	22.47666872	0.244645524	0.522026828	0.468626522	0.629229467	1.755017449
9836	MTMR10	26.29705674	-0.252702296	0.566921917	-0.622901422	0.52269222	4.11492
9837	MTMR2	26.84417489	-0.688481019	0.502292815	-1.270672894	0.170476626	10.12711105
9838	MYH11	27.62412128	-0.261696527	0.55216462	-0.652867789	0.512196988	1.747140192
9839	NLRC5	29.62254876	0.05487566	0.55506854	0.098862855	0.921247162	1.144142179
9840	NOL7	52.08220165	-0.59724955	0.450225292	-1.226262502	0.184752762	1.514441784
9841	NOL9	20.8608145	-0.202027185	0.520258485	-0.290187122	0.696298176	1.144142179
9842	NUDT17	29.24485287	-0.484576297	0.469589659	-1.021914227		

0.202112254 1.752022411
9843 OCIAD2 22.57162572 -0.168477291 0.617957929 -0.272625697
0.785122269 1.991140802
9844 PACS2 21.61017788 0.551854074 0.614224499 0.898442002
0.268949952 1.744214944
9845 PDIK1L 14.24298596 -0.127812287 0.974601002 -0.141402904 0.88755087
1.747140192
9846 PDP1 22.42622996 -0.521190575 0.622524122 -0.851902962
0.294267952 1.512814429
9847 PDPR 21.26722202 -0.479957722 0.512440819 -0.926611047
0.248958667 1.514441784
9848 PHF14 19.12901672 0.224285774 0.677772002 0.245670482
0.729590271 1.744214944
9849 PI4K2A 28.87090206 -0.47248182 0.525284128 -0.882674819
0.277411991 1.505270441
9850 PIGL 28.2012221 -0.208522461 0.541274665 -0.569887142
0.568754245 1.755848948
9851 PIR 26.09651829 0.689228975 0.57228495 1.204245798
0.228455952 1.514441784
9852 POLL 27.41601278 -0.077001799 0.552617827 -0.129088262
0.889280224 1.741489599
9853 PPP4C 27.27757414 -0.512618729 0.477092894 -1.076559168
0.281677254 1.744214944
9854 PREX1 27.97615159 -0.182286777 0.542688494 -0.227117262
0.726028505 1.991140802
9855 PRIM1 25.55871182 0.072149654 0.576488726 0.126888264
0.899028826 50.21899788
9856 PTER 22.52670867 -0.61592722 0.50664822 -1.215690282
0.224102927 24.77908717
9857 RAB11FIP1P1 22.4644922 -0.28244886 0.604651277 -0.467126795
0.640409164 18.74519112
9858 RAB22A 20.07524488 -0.72412045 0.520866291 -1.264025211
0.172556484 14.40709229
9859 RAB7B 22.25845689 -0.577648161 0.502154871 -1.148052406 0.25094692
14.44552541
9860 RALGAPA2 25.04085292 0.191299202 0.569725498 0.225774515
0.727040909 4.511541471
9861 RNF8 24.20571624 -0.051081146 0.51664848 -0.098870215
0.921241218 9.781115128
9862 RNPC2 24.76215114 0.108187928 0.572281757 0.189012602
0.850082156 11.51112044
9863 SETD6 25.22224 0.158097772 0.580008186 0.272578518
0.785177227 14.48011441
9864 SIGLEC1 17.70287276 1.016749689 0.692987892 1.465082748
0.142898242 4.519707215
9865 SIKE1 26.7250505 0.049692925 0.550997172 0.090187278
0.928128294 12.49999801
9866 SLC20A1 20.11569288 0.591918967 0.629412659 0.925721492
0.254590726 1.010291949
9867 SLC28A1 20.95666561 -0.162672204 0.52471046 -0.210022987
0.756542476 11.74571711

9868	SRD5A2	28.26554162	-0.268606128	0.480447024	-0.559075422	0.57611024	4.151194841
9869	STIM2	21.07520677	-0.121970401	0.646500921	-0.188662274	0.850257442	12.11494491
9870	TASP1	19.28284454	0.119982227	0.682092424	0.175904808	0.860268728	2.01955142
9871	TBC1D4	24.84681104	0.452268817	0.581672974	0.777702001	0.426744122	2.011095451
9872	TMEM144	22.14504887	0.810200245	0.612666282	1.220261955	0.186747572	2.011095451
9873	TNKS	20.26892661	0.25888745	0.644274797	0.401827684	0.687810847	2.759755814
9874	TNKS2	22.14981611	-0.447150101	0.514504054	-0.869089559	0.284798147	11.81415441
9875	TNRC6A	27.52100812	-0.217105724	0.48514821	-0.447502927	0.654511246	14.50587997
9876	TOB2	25.96275761	-0.252579242	0.562275929	-0.450907176	0.652056451	4.149441514
9877	TOE1	26.55711725	-0.505882584	0.482960091	-1.047464569	0.294885265	4.157917492
9878	ULK4	20.0920101	0.012024526	0.658269825	0.019798197	0.984204256	2.50080124
9879	YIPF2	22.65857797	0.047805846	0.50721525	0.094222997	0.924924074	7.952147454
9880	ZDHHC21	22.52746194	0.082777269	0.605264879	0.128414226	0.889912055	1.725179147
9881	ZDHHC2	28.20690279	-0.205781928	0.528714292	-0.281987122	0.702470909	7.705554554
9882	ZMYM2	22.2269961	-0.507884595	0.504289264	-1.007129201	0.212872642	2.005414958
9883	ZNF14	22.52786188	0.018679822	0.589905914	0.021665782	0.974728582	2.724187747
9884	ZNF242	20.07221286	0.15899426	0.6479785	0.245269654	0.806170224	1.728014494
9885	ZNF440	26.25761219	-0.295154609	0.591220111	-0.499126782	0.617682026	7.101711195
9886	ZNF589	18.68607042	0.792406506	0.675715429	1.174172547	0.24022592	7.871292154
9887	ZNF649	21.6072926	0.691626289	0.617877202	1.119274812	0.262980272	8.421244454
9888	ZNF92	17.16616742	0.599800187	0.70607114	0.849489726	0.295608829	7.449425417
9889	ZSCAN9	29.41144474	-0.168551058	0.52705117	-0.219800178	0.749119812	5.45290224
9890	AC004452.1	20.08455205	0.275426762	0.649171227	0.578216912	0.562050178	2.72995844
9891	AC018521.2	22.6246022	0.525549268	0.61128072	0.859751258	0.289926172	8.411784451
9892	AC021092.1	22.48781607	-0.297219126	0.620175856	-0.471802429	0.627067096	1.495971945
9893	AC025682.2	20.4828708	0.069742452	0.525244582	0.122757528		

						0.894285127	4.444025984
9894	AC069224.2	28.00151845	0.072106622	0.540978717	0.125127725	0.892502984	4.914514244
9895	AC117502.4	28.09672269	-0.725157158	0.562700102	-1.288709829	0.197498981	4.117981511
9896	AEBP2	27.66641009	-0.101825822	0.549456649	-0.185229158	0.852962026	1.991198184
9897	AL049647.1	28.51092128	-0.02984088	0.544955672	-0.054758261	0.956220974	5.715492171
9898	AL121928.1	28.66551871	-0.19852499	0.522829288	-0.271888521	0.709975845	1.495971945
9899	AL161452.1	24.66892678	0.088166212	0.502057278	0.175610068	0.860600292	5.478982849
9900	AMDHD2	24.82615921	-0.51192022	0.489226678	-1.046172942	0.295481192	4.415150051
9901	ANKRD26B	27.55906014	-0.224621609	0.477495566	-0.700784746	0.482427259	7.110118125
9902	ARHGEF29	29.27194906	-0.017919502	0.550589705	-0.02254602	0.974026617	10.14810955
9903	ATN1	26.02011204	-0.025008826	0.562279812	-0.06215175	0.950441986	5.951045007
9904	ATR	22.05745242	0.279625842	0.612224257	0.456720146	0.64786502	1.994122422
9905	BABAM1	24.51155142	0.021416476	0.57749505	0.027085124	0.970417122	8.280794085
9906	C1orf216	29.04027186	0.520248245	0.5427961	0.958644028	0.2277281	2.704084484
9907	C2CD2	25.2222288	0.101485411	0.58225927	0.174295912	0.861622911	5.100419548
9908	CCDC117	22.0516816	-0.244241178	0.64011158	-0.281716541	0.702671629	5.490215124
9909	CCDC92	25.22427094	-0.27872472	0.485668996	-0.779820666	0.42549644	4.154411918
9910	CD220	28.78046298	-0.41621778	0.471154261	-0.882612281	0.276905547	7.947244288
9911	CENPC	24.42682419	-0.058459802	0.502826624	-0.116021586	0.907627509	5.490175175
9912	CEP170	25.26286555	-0.298212079	0.490576614	-0.607882787	0.542265217	4.442452222
9913	CLPTM1	21.69292226	-0.2258187	0.518590467	-0.425447072	0.662227949	5.117521505
9914	CNTROB	25.84228004	-0.090461721	0.490656225	-0.184268841	0.852724112	1.510098427
9915	CRISPLD1	24.48046274	-0.271065091	0.588227808	-0.460720262	0.644992069	4.480141454
9916	CRYBB2P1	24.1678797	-0.079522418	0.502120694	-0.15829502	0.874145527	4.947021998
9917	CXorf21	29.65617125	-0.288568244	0.524426592	-0.550254981	0.582144498	1.742281419
9918	DDHD1	29.26525225	0.09427207	0.528791862	0.174971221	0.861102247	2.974280257

9919	DMAC1	27.5202188	0.220212549	0.545748905	0.421828697	0.672150058	7.901421782
9920	DPM2	27.46669752	0.024454281	0.544215064	0.044925141	0.964159007	2.718447125
9921	DTX2	25.22626547	0.214456115	0.582008871	0.267842656	0.712989815	2.15870875
9922	EI24	29.14676842	-0.1925079	0.521514087	-0.264069184	0.715806248	5.71007154
9923	ELAVL1	21.42214242	-0.29640211	0.5185064	-0.571646002	0.567561822	2.941152485
9924	EXTL2	29.65617125	-0.288568244	0.524426592	-0.550254981	0.582144498	4.944450247
9925	FAM160B2	45.24522429	-0.28777149	0.46604071	-0.617481441	0.526917219	4.705142144
9926	FAM91A1	21.06102896	-0.119947222	0.649026811	-0.18481106	0.852277222	7.899594424
9927	FANCG	25.09092458	-0.021008158	0.498412004	-0.062212781	0.950292588	4.125224841
9928	FOLR2	29.77285185	-0.668185897	0.472422272	-1.411295145	0.158128124	4.499411524
9929	GLMN	26.92227055	-0.186185681	0.555824228	-0.224966204	0.72765058	8.112225559
9930	GPN2	46.90569806	-0.250182074	0.455095574	-0.7694715	0.441612457	5.940772481
9931	GRAMD1A	28.82289406	-0.081204281	0.481795899	-0.168544982	0.866154559	4.477029882
9932	HDAC7	22.5547227	0.140160925	0.589694552	0.227682958	0.812126227	2.478148418
9933	HERPUD2	24.21026699	-0.182407711	0.594065976	-0.207049585	0.758805621	5.710011478
9934	IFFO1	27.48082419	0.128288917	0.5447821	0.22548666	0.812821021	4.702540518
9935	IFIT2	24.06696098	0.076426017	0.590746061	0.12927202	0.897062278	4.115527477
9936	IPO11	21.25290748	0.217894678	0.622229582	0.510068177	0.610002699	4.701407819
9937	ISOC2	25.9255022	-0.194954178	0.568610218	-0.242860842	0.721702152	4.474491941
9938	ITM2A	20.20401292	0.26705115	0.656198057	0.406967297	0.684022007	7.170482544
9939	KCTD15	26.05222287	-0.268052748	0.484622977	-0.552116052	0.580182945	4.911479019
9940	KIF2A	24.02242286	-0.455880527	0.494591988	-0.921720514	0.25666916	2.94502897
9941	LANCL1	22.81079178	-0.072907742	0.6018956	-0.122791622	0.902272101	5.974447457
9942	LILRA1	20.57987927	0.296292428	0.628146582	0.464202102	0.642420601	4.411012291
9943	LRR1	27.22519205	-0.551178092	0.472852626	-1.162182154	0.244755605	2.008110142
9944	MAIP1	20.92622899	0.286524055	0.677172422	0.570805117		

0.568121754 4.458295117
9945 MBTPS2 19.25270471 -0.099499875 0.700218429 -0.142098226
0.887002222 4.975488974
9946 MCF2L2 27.8277692 -0.759171272 0.570944507 -1.229676288
0.182624951 5.47448599
9947 MELK 21.52949228 0.019290647 0.628965862 0.02067042
0.975522281 1.999804214
9948 MGAT4A 21.75601779 -0.547419518 0.678867082 -0.806272165
0.420028292 5.481154484
9949 MORC2 27.28487296 -0.222418041 0.560689512 -0.414521828
0.678491985 4.705142144
9950 MUC20-OT1 24.72822602 0.052469011 0.582145058 0.09184826
0.9268186 1.985477454
9951 NDC80 26.76225271 0.272821252 0.556670864 0.66975187 0.50201598
4.105298841
9952 NEK1 41.44004629 -0.220054209 0.467892895 -0.705404179
0.480558744 8.151880118
9953 OTUD5 21.18448027 -0.275694672 0.519156902 -0.521042064
0.595288928 7.499882841
9954 PCGF1 28.75872472 0.29921642 0.540516082 0.728582815
0.460159755 7.409890748
9955 PCLAF 21.22526622 0.127670994 0.522641212 0.262412915
0.792221566 4.441120574
9956 PDLIM2 22.59515875 0.596101259 0.602105228 0.990028256
0.222160259 4.480977877
9957 PES1 22.9542407 0.451645426 0.520275107 0.86808965
0.285245257 4.122451104
9958 PGBD1 19.07410205 0.242982651 0.682204644 0.256120471
0.721750247 10.41110591
9959 PKD1 22.97956585 0.024480071 0.595249126 0.041125756
0.967195642 10.24801474
9960 PKD2L2 21.77581516 -0.278257462 0.642920176 -0.422122657
0.665651541 10.80957711
9961 PMM2 28.27186428 0.456966948 0.545422564 0.827804966
0.402140249 9.119454159
9962 PMS2P1 18.86871978 0.278876009 0.677685919 0.411512179
0.680697012 2.974280257
9963 POLD4 27.72694222 0.100778081 0.54145678 0.186122962
0.852247552 5.100549484
9964 PPARGC1B 20.08176777 0.522624857 0.657416262 0.794982158
0.426622299 2.974280257
9965 PPP4R1 26.6784854 0.08256586 0.502452918 0.166215471
0.867908692 5.710011478
9966 QRSL1 20.68079809 0.04967027 0.519600772 0.095592222 0.92284256
5.117521505
9967 RBM45 24.99275108 -0.150220172 0.575182299 -0.261187014
0.792948292 5.411572284
9968 RBM5-AS1 28.22881628 -0.521041261 0.552777826 -0.958942785
0.227587569 5.711807742
9969 RENBP 29.95467051 -0.022442221 0.52222824 -0.044802204
0.964264969 5.451047992

9970	REXO2	20.95944982	-0.022501912	0.519221729	-0.04222777	0.965422284	10.88572109
9971	RFC4	27.12148177	-0.069760925	0.490951989	-0.142092192	0.887006296	4.414858729
9972	RFC5	25.68992212	-0.700424987	0.489502429	-1.420891721	0.15246125	8.847444417
9973	RPS12P2	27.52728082	0.28756279	0.54846212	0.706627284	0.479791912	4.109914845
9974	RRS1	24.92514604	0.215240495	0.510487207	0.617724458	0.526756987	8.415941284
9975	SLC10A2	26.95270598	-0.522245467	0.481165176	-1.108227864	0.267759102	5.988774219
9976	SLC25A12	20.69212948	0.22711468	0.527689542	0.449245042	0.65218277	5.441411014
9977	SOS2	22.04616109	0.21502514	0.614802219	0.249762491	0.726516951	4.482812114
9978	SPAG5	20.2547488	0.261112706	0.642421792	0.405814118	0.684879184	5.408788101
9979	SPAG9	28.14198911	-0.200590496	0.546452447	-0.267077678	0.712561077	7.424971157
9980	SPTSSA	27.51181276	0.072200677	0.546272875	0.124182264	0.89225766	4.140955474
9981	ST6GALNAC6	16.89582041	0.29285652	0.72121075	0.527268528	0.591082125	11.87259845
9982	STAT5B	26.77647592	-0.602078695	0.477118601	-1.261905728	0.206982711	2.947874217
9983	STK2	21.85410117	-0.564461266	0.510852517	-1.104927809	0.269186528	4.897551247
9984	SUCO	24.94409729	-0.6946952	0.497189858	-1.297242505	0.162240258	4.128170189
9985	SVIL	25.22248006	-0.012628169	0.600257452	-0.022700091	0.981889502	4.714990449
9986	TASOR2	22.11112276	0.74506768	0.611725126	1.217977894	0.222222272	1.985477454
9987	TFDP1	29.96227667	0.411250897	0.542508124	0.758229122	0.448207828	4.925994249
9988	THAP7	25.5417988	0.259028882	0.56488902	0.625574194	0.525052994	4.428885187
9989	TNFAIP1	46.22004112	-0.574019464	0.446224128	-1.286262851	0.198216108	5.415449994
9990	TOGARAM1	20.42179255	-0.115281152	0.518176044	-0.222474879	0.822944227	5.944294212
9991	TP52TG1	29.84742795	-0.404525729	0.528955529	-0.764782126	0.444401287	15.54029142
9992	TRAPPC12	25.7188286	-0.227902765	0.56748225	-0.40160422	0.68797525	5.701514428
9993	TTL	27.4694828	-0.080728789	0.542718178	-0.14849282	0.881952062	8.277940728
9994	TTLL5	28.82976222	-0.625556241	0.546677498	-1.144287707	0.25250424	10.11199421
9995	WDR70	28.9886826	-0.292522787	0.469900742	-0.825252556		

						0.402519224	4.444901147
9996	ZDHHC18	25.1000427	0.477584995	0.578245722	0.825777678	0.408920219	4.741951488
9997	ZNF671	25.24224971	0.222020226	0.575588971	0.287464402	0.698412424	5.498721152
9998	ZNF771	27.48928017	0.127599916	0.542976276	0.224568899	0.814542251	4.471704477
9999	ZSCAN21	22.42618882	-0.404191962	0.625528682	-0.64615022	0.518182068	14.54710718
10000	ZSCAN29	18.58718264	0.091802524	0.699097498	0.121215782	0.895525508	11.54404047
10001	AC000068.2	25.82029929	0.281862251	0.564102286	0.67692664	0.498446155	4.714990449
10002	AC006001.2	27.46928161	-0.12287218	0.54562429	-0.242518749	0.807602562	2.141794792
10003	AC092752.1	22.41150267	-0.195281206	0.504854882	-0.287004688	0.698752728	10.22941884
10004	AC125257.2	21.0948518	0.21967716	0.624256602	0.246299162	0.729117898	2.50427191
10005	AGO4	20.28416442	0.127444252	0.674056227	0.189070652	0.850027442	7.920585004
10006	AKAP10	28.66262222	-0.147419428	0.526244719	-0.274859474	0.782424202	11.12229415
10007	AL122220.2	19.27240088	0.122602181	0.688266045	0.178121962	0.858619241	9.410455907
10008	ALKBH5	27.12282174	-0.870148227	0.604290226	-1.429950798	0.14988122	4.488448494
10009	AP000892.4	20.52042887	0.067282977	0.527096204	0.125459767	0.900159575	7.295744074
10010	AP4B1-AS1	20.12225228	0.225405112	0.665872224	0.228510501	0.724978515	11.21414481
10011	AP5S1	27.4496242	-0.184056692	0.546706947	-0.226664267	0.726270002	4.114042517
10012	ARHGEF12	21.86892997	0.442981152	0.616471491	0.720197279	0.471402477	4.48410045
10013	ARL10	28.44884205	0.02596592	0.526160971	0.048429227	0.96127408	4.454744459
10014	ARMC7	22.89552867	-0.279458672	0.501780445	-0.556924164	0.577572412	1.997810198
10015	ATP12A1	22.42195268	-0.102154799	0.501757482	-0.205586966	0.82711256	5.45290224
10016	BRPF1	26.27628117	0.278907407	0.558927257	0.67790675	0.49782082	2.481002975
10017	BTBD7	26.01652225	-0.46918149	0.479882028	-0.977701721	0.228221866	4.417444014
10018	C12orf65	21.49427695	-0.109452242	0.657877868	-0.166271672	0.867864465	9.104028141
10019	C2orf42	27.22224027	0.261278519	0.557271087	0.648478861	0.516675279	1.757741798
10020	CCDC59	22.82246141	-0.45424029	0.50011779	-0.908266611	0.262727278	5.14449551

10021	CD202	22.80024058	-0.267019269	0.626276748	-0.419659166	0.67472446	4.491149101
10022	CEP19	21.26267592	0.527028829	0.719679422	0.746219502	0.455524828	4.115527477
10023	COIL	22.8212787	-0.571459225	0.495176706	-1.154051228	0.248479126	5.104140179
10024	CPOX	19.84245929	0.490964269	0.66078211	0.742004785	0.457478784	4.181744148
10025	CYTOR	19.91866222	0.942812471	0.647040828	1.457114244	0.14508482	4.444404041
10026	DAZAP1	21.19262828	0.770810654	0.654277441	1.177929748	0.228824615	2.745579091
10027	DGCR8	20.10424922	0.594621441	0.651199464	0.912122572	0.261172821	10.22472242
10028	DGKG	24.21200114	-0.182127464	0.598820002	-0.204142922	0.761018245	5.94220417
10029	EED	29.70122558	0.146789468	0.527402111	0.278225522	0.780762486	4.142528114
10030	ENDOG	28.21804178	-0.102209649	0.527592872	-0.19198478	0.847754128	5.171244104
10031	ERAP2	29.24702567	0.425577062	0.565066818	0.770841692	0.440800771	7.871192021
10032	ERGIC1	24.2422865	-0.690826571	0.496166211	-1.292249086	0.162816714	1.757741798
10033	ERVK12-1	24.20222174	0.286025206	0.578599466	0.494257881	0.621052454	4.440415251
10034	ESYT2	22.50182427	0.154296802	0.522549464	0.295468201	0.767626164	5.490411441
10035	FAHD1	28.41491901	-0.276606227	0.528501478	-0.512659198	0.607490284	9.401487255
10036	FAM124B	29.41297757	-0.516599896	0.529059225	-0.958225869	0.227892422	2.71727581
10037	GLYCTK	28.92282798	-0.222548454	0.521271752	-0.418818752	0.675248599	4.142048904
10038	GNPDA2	27.21412112	-0.217246798	0.475725212	-0.667079908	0.504721092	5.441441074
10039	HAUS2	21.22972967	0.220022484	0.625127174	0.511947802	0.608687541	9.875181084
10040	HDDC2	28.21294295	0.09244089	0.565567808	0.165216069	0.868772918	7.148050854
10041	HOOK2	24.12818277	-0.461018057	0.500457279	-0.921192446	0.25694944	2.974280257
10042	IFIT5	21.24092582	0.182942577	0.520197779	0.252602157	0.722626208	4.91244178
10043	IFT20	21.60156957	0.622288667	0.619895607	1.005624916	0.214591222	4.108124107
10044	ITPK1	26.12952672	0.167892099	0.496824904	0.22792012	0.725415849	12.85772908
10045	KIF2C	26.00041472	0.256718922	0.570762224	0.624986918	0.521979644	4.422214717
10046	KTI12	28.28582979	0.252227662	0.541146222	0.651095102		

						0.514985098	5.175101551
10047	LINC00847	24.07258054	0.217222842	0.582282915	0.54469884	0.585960662	7.908051415
10048	LRCH1	28.67680896	0.102979507	0.544429841	0.189151106	0.849974287	2.105127521
10049	LRRC14	22.09241589	-0.428844616	0.497942659	-0.861221202	0.289110719	7.442417124
10050	LRSAM1	29.79926889	-0.149741595	0.472221927	-0.217092222	0.751172785	7.841887114
10051	LTN1	28.25284175	-0.479092776	0.55264807	-0.865228112	0.286852228	2.489459952
10052	MAGOHB	25.01528272	0.427585511	0.585692206	0.720050218	0.465259428	2.122240814
10053	MED20	26.9627129	-0.296572721	0.557651522	-0.521824452	0.594847585	5.411572284
10054	MED21	24.12620182	0.556499124	0.592241621	0.929648777	0.247297748	10.10598112
10055	MON1A	26.996688	0.182989554	0.54918141	0.222204204	0.728980145	2.702199199
10056	MRM2	21.88522885	0.029222009	0.515624797	0.056867786	0.954650516	4.710912859
10057	MVB12A	29.69566489	0.050075279	0.524744148	0.09542818	0.922974724	4.440415251
10058	NBEA	20.7214901	-0.287179415	0.699262827	-0.410688219	0.681201167	5.912911784
10059	NCBP2	29.65044952	-0.28777962	0.525504781	-0.547625122	0.582949226	4.119444149
10060	NSD2	22.12176022	-0.749717401	0.527504768	-1.421252269	0.155242405	2.495120447
10061	NUDT15	29.98555788	-0.121608202	0.522474978	-0.228282944	0.819248548	4.45555988
10062	PDK1	28.02240582	-0.021926272	0.555820414	-0.057457896	0.954180445	7.291978791
10063	PFAS	26.50976759	0.076049561	0.552746218	0.127226464	0.89076485	5.92515205
10064	PIGU	26.24029044	-0.247829929	0.481512964	-0.514710814	0.606755116	2.497915921
10065	PIK2R1	22.75806081	0.117745914	0.617172784	0.190782728	0.848695812	1.749155757
10066	PIN1	46.68220151	-0.559191849	0.448948822	-1.245558112	0.212926668	9.115179515
10067	PJA1	27.12640529	-0.66745127	0.491418266	-1.258214122	0.174295747	4.458481451
10068	POLE	22.7007128	-0.207741581	0.618292247	-0.497727466	0.618676146	11.82971447
10069	PPM1F	29.17218525	0.200887297	0.549018127	0.265902842	0.714427576	8.410002147
10070	PPP2CB	25.26572104	-0.064242875	0.572921229	-0.112122122	0.910718648	7.598141925
10071	PURA	25.42878425	-0.255085176	0.584719524	-0.426252195	0.662652762	9.210711855

10072 RBM42 45.77725884 -0.222147192 0.469016495 -0.472644721 0.625752272 5.421210489
10073 RCAN2 22.01129454 -0.16582795 0.614922215 -0.269684884 0.78740269 1.742281419
10074 RFC2 27.77066605 0.04652424 0.547208491 0.085021222 0.922244511 7.91924274
10075 SLC25E4 27.77917209 0.201895452 0.54442099 0.270844257 0.710752461 9.828411814
10076 SMAP2 27.24151581 0.102275021 0.545768122 0.187579716 0.851206128 4.485782187
10077 SMPD4BP 18.85727722 0.25820765 0.682726266 0.524811216 0.599714422 1.017557401
10078 SOCS4 20.92826006 -0.019926764 0.514445954 -0.028752855 0.969086626 4.95117275
10079 TAB2 22.054616 0.547127099 0.622581126 0.862547028 0.287826774 4.471511899
10080 TAF1B 28.97222259 -0.125264729 0.522229704 -0.225060485 0.814161788 8.277940728
10081 TBC1D20 22.58287485 0.222587422 0.589828101 0.546908417 0.584441652 8.447902791
10082 TGFBR1 24.72528948 -0.062248268 0.58400689 -0.106759645 0.914979662 7.115518145
10083 TIMM21 27.05025922 0.22202191 0.554066762 0.418779696 0.675277145 5.418485241
10084 TMSB4XP4 25.69902016 -0.169464144 0.57298042 -0.295759048 0.767414099 5.180811181
10085 TXNDC11 26.54080622 0.182224121 0.556169188 0.227829208 0.742022165 4.947021998
10086 UBE2C 22.40209775 -0.061059286 0.504440829 -0.121042701 0.902656421 8.251591802
10087 UNKL 22.11877522 -0.081810595 0.510604022 -0.160222171 0.872705278 9.274184072
10088 WDR91 24.72015684 -0.722671522 0.492080965 -1.467652542 0.142198628 7.455054049
10089 WIPF2 20.86911815 -0.155946922 0.51990525 -0.299952582 0.764212225 4.414858729
10090 XRN1 21.66567884 -0.177794579 0.508884624 -0.249280922 0.726802254 4.924141449
10091 YKT6 25.07292142 -0.070696522 0.496898566 -0.142275582 0.886862222 5.44140928
10092 Z82842.1 21.88295652 0.175295657 0.646980466 0.271098845 0.78621501 4.188484904
10093 ZC2H2 26.49215242 -0.420677975 0.481615645 -0.872472402 0.282405642 11.25101181
10094 ZCCHC9 21.64214625 -0.120626052 0.508797069 -0.256725071 0.797282296 5.944191479
10095 ZDHHC7 21.8681767 -0.278787624 0.506956729 -0.747179419 0.454955266 7.404110054
10096 ZFAND2A 26.7856841 0.262410087 0.551692108 0.475645895 0.624226672 11.12050884
10097 ZMYM2 25.46260827 -0.257851282 0.576797957 -0.447029174

						0.654846775	9.894918147
10098	ZNF211	25.5607417	-0.221291501	0.486052251	-0.47585718	0.62417612	8.9149584
10099	ZNF202	42.74800297	-0.165222294	0.464099922	-0.256008019	0.72182456	5.47448599
10100	ZNF667-AS1	28.45172845	0.228721127	0.546882648	0.418244608	0.675768284	11.09289945
10101	ZPR1	28.51265552	-0.029629645	0.544704128	-0.05429585	0.956619785	4.978214212
10102	ZSCAN20	21.96129271	0.159527212	0.512625448	0.211196428	0.755651291	1.754914451
10103	AC008569.1	46.42146066	-0.254277088	0.446158626	-0.79406081	0.427160021	1.148054109
10104	AC011405.1	29.99225964	-0.225927106	0.467012406	-0.505182728	0.612420501	1.714712149
10105	AC020978.2	21.24869479	-0.471202502	0.550769206	-0.855526967	0.292252948	4.449114017
10106	AC072896.2	24.85426025	-0.052182952	0.49124822	-0.106202604	0.915420811	2.745419154
10107	ACOT11	27.06258148	-0.26028184	0.474826698	-0.548161762	0.582580825	4.471419151
10108	ADIPOR2	25.26851525	0.107227572	0.569918904	0.188145217	0.850762728	4.140955474
10109	ALKBH1	22.2416225	0.272117685	0.609277587	0.610752295	0.54126258	9.588072157
10110	AP001972.1	24.11972672	-0.245297779	0.490912219	-0.499881078	0.617158817	5.974447457
10111	ARHGAP26	27.81229172	-0.120667178	0.476219156	-0.252285812	0.799970089	4.724221854
10112	ARIH1	24.91927295	0.150244158	0.495295401	0.202482152	0.761521684	4.914544417
10113	ATRN	26.28462595	0.222002947	0.556220714	0.58071002	0.561425901	4.714940407
10114	BRD1	20.65876621	0.870860202	0.625010252	1.271411405	0.170246744	4.101440847
10115	BTD	25.06521406	-0.485600155	0.484968267	-1.001202947	0.216680268	2.008140205
10116	C1RL	24.78726566	0.459757527	0.581822591	0.79018724	0.429418268	8.184804144
10117	C5orf58	21.28216458	0.272924461	0.529221445	0.517501159	0.60480626	5.180811181
10118	CCDC125	22.28878182	0.725265516	0.596291289	1.22202504	0.217566422	4.179980842
10119	CCDC120	27.27242202	0.105572081	0.485219676	0.217522074	0.827792925	9.140497511
10120	CDK2	28.02672405	0.122461587	0.542585258	0.227124562	0.820226885	5.942254221
10121	CEP164	21.88295544	0.510606601	0.619760174	0.822877722	0.410009029	5.944191479
10122	CHPF2	22.24482268	0.204911124	0.512155494	0.595248722	0.551610288	2.947874217

10123	CHROMR	27.75852026	-0.62268005	0.469756155	-1.227667649	0.184287925	18.95440841
10124	CLN8	20.95084151	0.025456682	0.51286561	0.049529574	0.960489201	4.457580005
10125	COG6	22.2972758	-0.228828292	0.505268626	-0.650670175	0.51525942	8.914858174
10126	COX10	22.22117199	-0.22410581	0.509277588	-0.626402049	0.524512754	4.499411524
10127	CRIPT	29.70665477	-0.529611218	0.556952852	-0.968862271	0.222612262	4.181844171
10128	CRY2	22.68926294	-0.124478106	0.504084577	-0.24692892	0.804955488	5.100549484
10129	CSNK1G1	26.02006082	0.255225522	0.562172729	0.620952004	0.528071252	4.111751191
10130	DBF4	22.76650844	0.622771226	0.522111028	1.194710111	0.222200211	4.19489181
10131	DGKZ	22.48965049	0.227992288	0.511019587	0.465722428	0.641414189	4.712749104
10132	EEFSEC	22.96507922	-0.221889752	0.522416148	-0.442879272	0.657129799	4.151144799
10133	EFR2A	25.94280684	-0.128912202	0.574275426	-0.241850879	0.808895712	5.711857815
10134	EOLA2	22.56680214	0.600491225	0.620028824	0.968472767	0.222807814	4.915855752
10135	FAM168A	27.97022748	0.02487622	0.529272457	0.046120604	0.962214124	11.52754845
10136	FAM174C	22.18252668	-0.040905715	0.505564244	-0.080910996	0.925512725	7.150884101
10137	FAM200B	22.14952459	0.578252119	0.611792627	0.945176761	0.244568642	4.441120574
10138	FOS	29.50420709	0.119751408	0.529202467	0.226286562	0.820978548	4.714524491
10139	FOXN2	20.41880587	-0.114299405	0.517266507	-0.220925404	0.825150516	7.411474022
10140	GFOD1	24.17046259	-0.248488469	0.502186726	-0.494812898	0.620722199	4.908942794
10141	GMNN	21.96129152	0.114722065	0.509644947	0.225101927	0.821899974	4.910185059
10142	HDAC5	26.29669581	-0.210824909	0.490097242	-0.420189951	0.667057471	1.504417944
10143	HELLS	22.57142226	0.017028698	0.60740196	0.028051767	0.977620864	2.489171517
10144	ITGB2BP	20.87458752	-0.291721992	0.517254204	-0.757229592	0.448852426	4.285214049
10145	ITPKB	28.40079126	-0.12210277	0.546841502	-0.222289142	0.822210477	2.71727581
10146	JOSD1	28.22922191	-0.102620866	0.529852981	-0.191960918	0.847772819	5.110244851
10147	KIAA0586	25.05751125	0.191192819	0.572624647	0.222208225	0.728901624	4.947185494
10148	KIF21B	28.99196869	-0.126402562	0.529092426	-0.224474749		

0.814616424 4.142528114
10149 KIFC2 29.22849071 0.246245172 0.520480521 0.464281182 0.64227467
5.711212804
10150 MAP2K2 21.69288105 0.092966989 0.509674752 0.184266576
0.852725889 9.121241448
10151 MAPKAPK5-AS1 26.22817922 0.052256264 0.561689709 0.09202422
0.925876276 4.419188045
10152 MARS2 25.75822094 0.106265668 0.562777246 0.188666091
0.850254529 4.118271814
10153 MCM2AP-AS1 29.95456824 0.169970827 0.524515284 0.224052082
0.745897846 7.440441951
10154 MFHAS1 28.92551207 -0.272580195 0.540252271 -0.691492004
0.489256404 4.949848244
10155 MFSD5 21.15612465 -0.272067914 0.512761942 -0.522542255
0.594249792 8.414221572
10156 MINDY1 22.87579029 -0.101620271 0.500715848 -0.20294998
0.829174129 5.112151124
10157 MIR202CHG 22.27295196 -0.099729056 0.497199924 -0.200601506
0.84101018 9.171724151
10158 MLLT6 29.97421554 0.216746218 0.52251117 0.414024248
0.678856249 11.81714195
10159 NKAP 29.51579867 0.770679814 0.559261287 1.277785152
0.168269621 4.44404591
10160 NUDT16 41.79469965 -0.245222779 0.46266757 -0.529112517
0.596726705 11.1108114
10161 OSBPL7 26.01722548 0.299546761 0.561671562 0.522212027
0.592816911 4.144414147
10162 PAPOLG 25.84556601 -0.012256817 0.608147194 -0.021798698
0.982608522 5.115987482
10163 PCNT 26.58242917 -0.426272069 0.612859161 -0.695709709
0.486610644 8.907111094
10164 PDSS2 28.76982265 0.248222682 0.524416827 0.651595656
0.514662051 4.451774595
10165 PEMT 22.82899291 0.598188492 0.586590602 1.019771697
0.207826749 9.417417914
10166 PIK2CA 27.55289026 -0.024122054 0.570271624 -0.059854029
0.952271894 5.481749124
10167 PLEKHM2 22.72020157 -0.040826042 0.512729922 -0.079622296
0.926526867 4.922159011
10168 PPM1K 26.47867782 0.079097606 0.558227088 0.141694218
0.887221464 2.502584415
10169 RHOT1 27.40441787 -0.286075672 0.559216499 -0.51156515
0.608955281 4.444402408
10170 RPA2 27.52786918 -0.271210711 0.470856271 -0.788586101
0.420252957 4.477217209
10171 RSBN1L 22.44428507 -0.190794027 0.509424842 -0.274528212
0.708011296 1.994122422
10172 S100P 24.29202924 0.529529909 0.592888096 0.891649207
0.272580922 5.911570299
10173 SERPINB10 21.74461692 0.090252297 0.5192892 0.172958752
0.861897879 1.154744784

10174	SESN2	26.02228405	-0.625982082	0.48112278	-1.221845256	0.186219685	12.12144155
10175	SETDB2	27.51449577	0.178922988	0.542514529	0.229214729	0.741992291	8.887442791
10176	SHLD2	25.14719608	-0.282421246	0.614079222	-0.461528722	0.644412129	4.194941881
10177	SKAP1	21.26610244	-0.141122225	0.547129766	-0.25795027	0.796445211	4.917249711
10178	SLC25A26	25.24112057	-0.227521012	0.482401626	-0.698241256	0.485026267	1.751091104
10179	SLC2A1	22.85201946	0.248464508	0.592226226	0.587405759	0.556921227	5.944444275
10180	SMC2	26.60197895	-0.466692117	0.486224718	-0.959808299	0.227151705	2.484414404
10181	SNAPC2	22.87768419	-0.269277225	0.492192622	-0.545987952	0.585074219	4.480141454
10182	SOCS6	22.15145648	-0.612179616	0.522417054	-1.169582862	0.242168877	2.979145441
10183	SPC24	22.54417042	0.276265879	0.614575267	0.449522262	0.652054221	11.7945521
10184	STX4	41.86811829	0.027206147	0.480296876	0.056644429	0.954828424	4.471471827
10185	TEC	29.95162277	-0.167067702	0.524128106	-0.218747485	0.749918002	2.708919821
10186	TMEM218	29.81126222	0.28272858	0.540625706	0.707926222	0.478984796	8.424915114
10187	TNFRSF12A	44.69055277	-0.419055212	0.446854521	-0.927789109	0.248252799	4.441120574
10188	TRAPPC10	25.55272755	-0.207824872	0.60049292	-0.246090456	0.729274726	2.495120447
10189	TSC22D2	22.29254907	-0.450262279	0.506221226	-0.889264116	0.272861152	5.177984824
10190	TUBB2A	24.01526957	-0.41081202	0.501276867	-0.819269714	0.412575509	4.401174088
10191	TUBGCP5	22.52487212	0.22795898	0.599124262	0.547297261	0.584105827	2.718447125
10192	TWNK	20.2077519	-0.189952942	0.524974228	-0.261822884	0.717476917	2.710111154
10193	UBA2	26.74602822	-0.05770912	0.554997888	-0.102980792	0.917184591	7.474445845
10194	UROS	44.24217802	-0.226891505	0.454581864	-0.719102711	0.472077021	4.105248799
10195	VCPKMT	22.78621295	0.20616087	0.61289645	0.498717447	0.617978452	5.887151401
10196	VGLL4	24.25228715	-0.042256046	0.521828114	-0.079454228	0.926671257	11.55719594
10197	VPS51	58.27566869	-0.576895966	0.424162764	-1.228751992	0.182929806	7.150924141
10198	YWHAH	27.77542221	-0.624524102	0.471945442	-1.22229724	0.185726549	2.724227818
10199	ZBED4	22.26047842	0.292224242	0.60560741	0.482696111		

0.629211525	4.471704477
10200	ZBTB27 22.24204467	0.117718585	0.620258405	0.189789585
0.849474018	5.741041107
10201	ZBTB40 21.62051247	-0.128742205	0.512502724	-0.251202667
0.801657422	4.481998001
10202	ZFYVE16	17.1602422	0.42951708	0.741924479	0.578922912
0.56264119	2.495120447
10203	ZMAT2	24.29198928	0.2476168	0.582989524	0.596265946
0.550997577	4.411012291
10204	ZNF281 41.2221694	-0.422200195	0.455254676	-0.927292428
0.252722202	12.77879991
10205	ZNF561 26.29876262	0.422140221	0.55702619	0.777592997
0.426808401	5.140114155
10206	ZNF574 25.08506149	-0.1945826	0.486179058	-0.40022022
0.688986885	8.887442791
10207	ZNF652 29.67014569	-0.142029442	0.524542164	-0.270787462
0.786554502	4.912411718
10208	ZNF689 24.20546281	-0.050018277	0.512087479	-0.097675259
0.922190162	8.40059245
10209	ZNF714 22.29240844	0.564886258	0.61506752	0.918412294
0.258402481	5.915255484
10210	AARS2	24.88251249	0.0702798	0.491452996	0.1422072
0.886126464	4.457580005
10211	AC002094.2	24.00472021	0.204078788	0.602955241	0.228464184
0.725012412	5.47448599
10212	AC005229.2	24.12285222	0.050260816	0.495224087	0.101690922
0.919001999	4.478480018
10213	AC005841.1	24.8746596	0.451487809	0.590158052	0.765028622
0.444254494	10.15784424
10214	AC018761.2	21.22670688	0.278624921	0.514622479	0.5414256
0.588207275	4.499909959
10215	AC025257.1	24.04701021	0.199942292	0.58867205	0.229651411
0.724119057	2.718447125
10216	AC027459.2	29.48455872	0.218279224	0.529225757	0.601280424
0.547652219	10.09474094
10217	AC145098.1	22.17507962	0.184191906	0.510207854	0.261012467
0.71808928	4.114440114
10218	AGAP2	22.92085429	0.027268225	0.499457988	0.054595622
0.956460618	4.722444448
10219	AKAP8	26.24215472	-0.058246845	0.564001266	-0.102451602
0.917604559	9.410754021
10220	AL157292.2	24.0441248	-0.029960041	0.601404712	-0.06644451
0.947022925	1.148054109
10221	ANKRD12A	22.2916541	-0.05957517	0.502226672	-0.118262821
0.905780175	4.19489181
10222	APPL1	24.24960092	0.510988928	0.50882246	1.004255824
0.215255216	9.590958444
10223	ARHGAP22	29.92291641	-0.601746876	0.457157725	-1.216278484
0.18808059	8.411824714
10224	ARL2	22.91996201	-0.058009168	0.494558522	-0.11729485
0.906626406	2.122240814

10225	ATG4C	29.02862688	0.169841282	0.525821667	0.216967609	0.75126818	2.727112112
10226	ATM	21.79470585	-0.419610575	0.522952271	-0.800856224	0.422214822	7.418244714
10227	BACH1	24.45761929	0.024299177	0.501211652	0.048680287	0.961174006	7.152771409
10228	BAIAP2	22.20228202	0.499812221	0.528050005	0.946524602	0.24288108	8.117511192
10229	BMPR2	24.56705122	-0.280204169	0.488857512	-0.572181677	0.566521689	10.24492471
10230	BRAF	27.55278906	-0.086125219	0.575620626	-0.149629029	0.881049407	10.27911245
10231	BRI2BP	21.2810529	-0.574424762	0.526002249	-1.092057617	0.274807777	1.712887811
10232	CDK12	27.72290451	0.206489622	0.544625901	0.27914029	0.704582689	4.449929148
10233	CDK12	22.2278947	0.482760228	0.594219746	0.812972172	0.41566022	5.980218251
10234	CENPBD1P1	22.24765695	0.571148927	0.514572962	1.109945254	0.267022618	14.04595749
10235	CERK	28.40064	-0.226058626	0.54824057	-0.594627945	0.552092196	1.754914451
10236	CHM	22.62221712	0.506516768	0.595878217	0.850022897	0.295206241	5.424104098
10237	CHN2	25.09058092	-0.277221247	0.482275224	-0.572718242	0.566158481	4.975529028
10238	CKAP4	26.22926945	0.051768422	0.562009468	0.092112098	0.926608182	10.07115821
10239	CLCN6	21.88772161	-0.425407524	0.512461266	-0.820126058	0.406467515	4.188484904
10240	CYB561D1	21.14478219	-0.225292855	0.5126526	-0.429464102	0.660225287	7.150824129
10241	DANCR	24.27589485	0.071592617	0.496180259	0.144289502	0.885271867	2.941102412
10242	DDX60L	24.66862218	-0.025525421	0.491609088	-0.072262557	0.942292166	1.004144177
10243	DHX25	22.62574071	0.179442622	0.508220141	0.2520805	0.724028091	5.472450442
10244	DNAJC18	25.21919998	0.217488644	0.574214458	0.278758564	0.70486716	2.475222181
10245	DSN1	29.21416272	-0.197067874	0.524591591	-0.268622572	0.712401612	5.481819194
10246	EGLN1	27.4252552	-0.224922064	0.555862647	-0.422645121	0.672554194	7.179129544
10247	FCHSD1	22.05261027	-0.522267945	0.516541792	-1.020628667	0.202710297	10.1728202
10248	GALNT1	21.71641257	0.275259482	0.511860525	0.527762669	0.590740907	4.194057474
10249	GEMIN2	27.00772687	-0.020804702	0.554789687	-0.055525008	0.955720207	10.29884075
10250	GTPBP8	22.67224129	0.181759274	0.506252772	0.258957082		

0.719627195 7.154404954
10251 HAUS1 28.56262598 -0.211560852 0.467717729 -0.45222592
0.651024206 4.724181794
10252 HAUS2 24.79004975 0.282810879 0.576624766 0.49045062
0.622815064 7.10450748
10253 HSF2 25.80612029 0.227454182 0.562264172 0.582281272 0.56027717
4.481440515
10254 IMPAD1 25.96250201 0.026602517 0.572997957 0.046247756
0.962022076 7.929091044
10255 IRGQ 40.28046627 -0.110222817 0.465955251 -0.226554512
0.812002412 1.740799779
10256 JTB 41.20052561 -0.42082219 0.452841826 -0.927244155
0.252799782 9.594579197
10257 KAT6B 21.89901294 -0.279955988 0.511742821 -0.742472025
0.457800784 10.14824114
10258 LINC01094 22.61279912 -0.481699467 0.506694899 -0.950669659
0.241772095 5.459574022
10259 LINS1 25.07162695 0.600722256 0.572862288 1.046822841
0.295181294 1.994948979
10260 LMAN2L 22.61962001 0.008722527 0.509955206 0.017126068
0.986226042 11.09448492
10261 LPIN2 22.49611864 0.089284081 0.645029785 0.128418529
0.889909646 4.709181159
10262 LSS 47.12888089 -0.614222222 0.422707562 -1.416442672
0.156645645 8.241048781
10263 MDC1 28.79225504 0.245594276 0.52868012 0.455918766
0.648448294 7.194051501
10264 MFSD14B 26.26262051 -0.614297262 0.499805756 -1.229272084
0.21896981 1.758402117
10265 MICAL2 26.80806425 0.261242994 0.555500492 0.470464019
0.628022525 1.747059095
10266 MSTO1 19.6510004 0.528614861 0.667978292 0.791265228
0.428720826 1.749894441
10267 NAPEPLD 21.6041024 0.148029092 0.644067887 0.229850128
0.818208222 1.747059095
10268 NUDT14 20.4440726 -0.162149245 0.519659111 -0.212954262
0.752555701 19.45449028
10269 PIP4P1 26.72618972 -0.162757829 0.561569827 -0.291607228 0.77058694
11.95870199
10270 PMF1 25.50757117 -0.146784208 0.580892591 -0.252686912
0.800510162 2.175419421
10271 POLG2 29.80544027 0.090992748 0.552951702 0.16456002
0.869290294 2.015111115
10272 POLR1E 22.51019525 0.022725551 0.62520585 0.027260826
0.970197207 12.75024514
10273 PPP1R21 29.98824101 0.264422488 0.522597196 0.505998672
0.612857581 5.017817854
10274 PREB 42.27286262 -0.625595627 0.464622522 -1.246459967
0.178154222 5.74447802
10275 PRIMPOL 25.0422826 0.267179551 0.572252946 0.640519256
0.521825075 15.44711549

10276	PRUNE1	21.71924804	0.60024829	0.52742168	1.117069226	0.262964727	12.47149252
10277	RAB21	22.56942716	0.20985179	0.614229227	0.241594941	0.722655745	19.88571795
10278	RABIF	45.18582895	-0.281168194	0.44222199	-0.859974467	0.289802117	1.514114111
10279	RBMX2	26.21840872	0.421775891	0.557228555	0.77472415	0.428502678	12.78982987
10280	RCN1	24.81192919	-0.215182588	0.490174929	-0.428992451	0.660666281	1.172415405
10281	RIPK1	22.60450184	-0.422122722	0.495895947	-0.851252619	0.294629029	2.745215211
10282	SP4	27.50021796	0.180521674	0.544122941	0.221784104	0.740052288	2.745215211
10283	SPATA1	24.54998918	-0.665789821	0.495280168	-1.244269111	0.17886128	12.01201447
10284	STAG1	25.49921522	0.192491428	0.574044819	0.227066762	0.726066572	14.9405579
10285	TAF15	21.41856655	0.658959624	0.627049964	1.024292288	0.200952762	1.012117098
10286	TCEAL4	40.47724227	-0.448551792	0.455027212	-0.985768942	0.22424651	5.171101448
10287	TMEM175	44.15001101	-0.455251071	0.442694767	-1.026270999	0.204762862	17.94449154
10288	TMEM254	20.56275667	0.691109488	0.521595681	1.200066022	0.192578228	2.144218207
10289	TMEM260	20.66428575	0.720556149	0.64180162	1.122708506	0.261561214	11.77187521
10290	TOP2B	26.67529652	0.045488896	0.488710907	0.092079255	0.925840519	14.95492717
10291	TRAF2	29.02022096	0.220586881	0.521010804	0.602729488	0.546022506	5.010402141
10292	TREML2	17.92989086	0.78622125	0.705708412	1.114229668	0.265180671	12.1748142
10293	TRMT61A	28.2941925	0.561224221	0.541472512	1.026494508	0.2999715	8.811299918
10294	TXNL4B	22.04780146	-0.042722778	0.620128094	-0.068892975	0.945074014	14.51551408
10295	UBN1	40.98107572	-0.271171274	0.454811866	-0.816098482	0.414442811	1.742221192
10296	UQCC2	20.25165992	0.19199781	0.667448701	0.287659275	0.772607566	1.771787171
10297	WDFY2	24.274876	-0.067274577	0.596750658	-0.112902292	0.91010794	11.55141545
10298	WDR59	28.87905427	-0.26925024	0.545826514	-0.676497441	0.49872487	9.587971059
10299	WDR7	25.08501025	-0.111820522	0.488274262	-0.229022189	0.818842898	9.579514081
10300	YJU2	29.71794502	-0.242598769	0.524957972	-0.452491267	0.650195021	4.151195442
10301	ZDHHC5	24.09982816	-0.458670442	0.492658684	-0.92912462		

0.252824502 14.00482181
10302 ZFP62 26.45027222 -0.255895888 0.577661506 -0.616097625
0.527820082 2.502525489
10303 ZNF200 22.07624298 -0.202962252 0.512172405 -0.592220144
0.552626229 4.991299859
10304 ZNF202 24.62550248 0.667555525 0.585545909 1.140056681
0.254262688 10.24771091
10305 ZNF225 26.56602182 0.225602816 0.561406788 0.419665065
0.674720149 4.71914844
10306 ZNF227 27.46082226 0.078712601 0.550575282 0.142964258
0.886218409 4.701254482
10307 ZNF42 29.97908171 -0.454522278 0.457047787 -0.994498268 0.2199802
1.948444499
10308 ZNF557 28.20659812 -0.101515059 0.540085412 -0.18796112 0.85090712
5.112101
10309 AC005899.9 22.01964717 0.401190622 0.629469151 0.627280728
0.520409697 9.878015125
10310 AC006547.2 40.28686164 -0.6662051 0.458819202 -1.452216801
0.146441222 4.497828498
10311 AC010226.1 18.69407047 0.621655428 0.709402228 0.890405207
0.272248247 4.488517424
10312 AC017082.2 25.52020281 0.419421627 0.572967761 0.720740758
0.464927515 4.112941219
10313 AC074117.1 22.07216822 0.268690198 0.607194604 0.442510845
0.658119584 4.984719145
10314 AL645929.1 26.28520221 -0.090199246 0.479207547 -0.188186784
0.850720222 4.125122444
10315 ANAPC10 21.05226104 0.245792466 0.529221781 0.652299915
0.512498494 4.944144515
10316 ANKUB1 28.29404224 0.252916718 0.52802492 0.655926442
0.511865019 1.748951915
10317 ANO6 21.22877294 -0.22527495 0.521620651 -0.642925665
0.520265846 1.170790158
10318 AP2M2 27.28926292 0.265722979 0.546244295 0.669400199
0.502240225 4.451910741
10319 APH1B 28.8449718 -0.280494722 0.472799067 -0.802071911
0.421922172 7.111418241
10320 ARID2B 26.11575961 0.459998629 0.575216489 0.799696545
0.422886625 8.144110417
10321 ARPC5L 29.68700544 0.197789604 0.525194092 0.269566119 0.7117058
4.112941219
10322 C17orf80 28.92128212 -0.270722565 0.540702121 -0.500688002
0.616590718 4.49418812
10323 CDCA8 21.75580598 0.218571585 0.512252488 0.621780846
0.524085982 14.74089529
10324 CEBPG 22.87474657 -0.140011652 0.491079444 -0.285109986 0.77555988
5.154974974
10325 CENPP 28.5607495 -0.172521002 0.466060921 -0.27222545
0.709642108 4.105197442
10326 CORO7 25.7824866 0.229997659 0.565528218 0.582510699
0.559549557 4.111159481

10327	CTBP1-AS	24.61560291	0.267726262	0.592698024	0.620427685	0.52497625	4.958475811
10328	CYTH2	47.91007258	-0.464462824	0.422601959	-1.071175568	0.284090495	4.121298198
10329	DGUOK-AS1	20.29509482	0.186492224	0.526088226	0.247876126	0.727922215	2.745517954
10330	EDEM2	21.62209526	-0.211682019	0.51192696	-0.608840798	0.542629968	2.480951828
10331	ELMO1-AS1	22.85221298	-0.095962897	0.495602925	-0.192620611	0.846465128	7.911077889
10332	ERBB2	29.70296746	0.24217907	0.529821806	0.625726578	0.52495465	14.7825181
10333	ETFRF1	21.92716508	-0.244065725	0.51087722	-0.477728412	0.6228264	4.747511984
10334	EXOSC5	21.82479519	0.497247777	0.52949812	0.921685827	0.25669247	8.114575748
10335	FBXO5	25.79194258	0.229216761	0.564070626	0.582644575	0.559459464	5.47074414
10336	FRMD4A	24.56212282	0.675967428	0.592822209	1.128222845	0.254981524	8.27785954
10337	GTPBP2	25.78282246	-0.125824624	0.495648619	-0.252878692	0.799589276	4.105197442
10338	HEG1	24.59788862	-0.494116075	0.492157298	-1.001944161	0.216270562	5.940449849
10339	HMGB1P5	22.59892019	-0.022208281	0.50206929	-0.064022724	0.948951229	8.944554444
10340	INSIG2	26.42762217	0.026105751	0.486558214	0.052652911	0.957210902	2.502485417
10341	KBTBD2	24.56486804	0.272747467	0.579822558	0.642852525	0.520219797	5.197482002
10342	KLHDC4	27.80920292	-0.250256226	0.467745268	-0.749022122	0.452827828	5.994242815
10343	KNOP1	22.24142011	0.242722952	0.624040567	0.288971422	0.697297279	4.71914844
10344	KPNA4	21.29702879	0.124574064	0.52967496	0.249261227	0.802081297	7.448994721
10345	LBHD1	20.0022162	-0.121619022	0.528207626	-0.22597047	0.821224288	4.458421514
10346	LIX1L	24.45200485	-0.02990727	0.621107209	-0.048151525	0.961595475	5.91942111
10347	LONP1	28.24817272	-0.242252426	0.467712822	-0.72197142	0.464186012	2.502485417
10348	MARF1	28.28468281	-0.655221081	0.47228098	-1.284655425	0.166157852	5.484890821
10349	MED6	21.76152672	0.550084486	0.517219759	1.06222554	0.287629814	4.701254482
10350	METTL1	29.68122462	-0.19077242	0.528152866	-0.261208026	0.717942926	11.11145427
10351	MTHFR	27.18186291	-0.110289582	0.492280827	-0.22258276	0.822081202	4.720410017
10352	MTLN	28.7272912	0.100250979	0.522844995	0.188142855		

0.850764667	4.858004402
10353	MYO18A 27.01901712	0.28977948	0.550260157	0.526622682
0.598455628	5.102202425
10354	N4BP1	24.59212129	0.611142602	0.578041949	1.05726241
0.290291287	4.118211487
10355	NDUFA9 20.87522851	0.754175228	0.64241127	1.172975884
0.240404685	7.407004145
10356	NEIL2	20.89412242	-0.429906725	0.525692702	-0.826812472
0.402697424	7.914407195
10357	NFATC2 22.90682677	0.425508886	0.525292855	0.794760114
0.426752057	5.479110129
10358	NRIP1	28.04622902	0.225944572	0.544221848	0.415086077
0.678078891	7.427991821
10359	NUP205 28.47947796	-0.221262619	0.559242652	-0.592240417
0.552622656	7.91241191
10360	PDPK1	21.9229259	0.11745515	0.506824625	0.221742552
0.816727978	1.985574454
10361	PITPNA 25.14972164	-0.197727577	0.488079219	-0.405112614	0.685294
8.289100917
10362	POLM	20.18511704	0.051466845	0.521629692	0.098665482
0.921402876	5.757911918
10363	POM121C	21.15289024	0.62242559	0.627027221	0.99424619
0.220054256	8.415571741
10364	PPP1R12B	28.9824605	0.22272467	0.521075826	0.421285725
0.672546446	10.24120192
10365	PPP4R2A	27.92216722	0.140179429	0.482429619	0.290569719
0.771280422	4.941211148
10366	PRC1	25.90410256	0.027717096	0.482052586	0.057279045
0.954242255	9.875180011
10367	PRELID2	26.59984466	0.508776974	0.561172094	0.906621091
0.264601915	7.950181171
10368	QSER1	22.82296114	0.202209506	0.610452449	0.495057414
0.620559592	12.81101848
10369	RAMAC	22.55720244	0.512991541	0.60467766	0.850025682
0.295210807	9.447214094
10370	RBBP8	28.76285044	-0.152627267	0.552511569	-0.277550227
0.781257627	11.81141999
10371	RETREG2	51.29562228	-0.601121268	0.419978748	-1.42122759
0.152222486	4.414807401
10372	RGP1	20.91292979	-0.251620472	0.517144742	-0.486576488
0.626558489	7.419584915
10373	RGS9	22.10420708	0.781851107	0.605482421	1.291286199
0.196604454	7.457790197
10374	RHOQ	26.77622122	-0.298515201	0.472562506	-0.841524517
0.400054156	5.70425071
10375	RNF144B	25.52169677	0.248692477	0.571109208	0.42545688
0.662220821	4.950019842
10376	RPL4P4 28.49659016	0.277229764	0.525802169	0.517409712
0.604870181	5.454427488
10377	SFXN4	26.6449599	0.56025975	0.587988271	0.952841549
0.240670259	2.14149194

10378	SLC11A1	28.76962126	0.247851769	0.522452722	0.465489786	0.64158075	4.720509902
10379	SLC29A7	29.17745222	-0.144456057	0.52244795	-0.270796912	0.786547225	11.2117142
10380	SMAD4	22.60027459	0.271428152	0.619097025	0.428442018	0.661065892	7.901180514
10381	SMCR8	27.89964191	-0.122552767	0.562829805	-0.219122089	0.826546276	8.1912127
10382	SNHG14	44.45681488	-0.405124728	0.441752262	-0.917082747	0.259098757	5.441208181
10383	SREK1IP1	27.74225929	0.154096167	0.542209557	0.282677202	0.776657767	5.47074414
10384	STEAP4	29.46177474	-0.121002106	0.529752561	-0.228414286	0.819224107	2.748242201
10385	STRN2	21.95546849	0.070729411	0.505812689	0.129822209	0.888791774	2.979114504
10386	STRN4	29.87218715	0.627174925	0.559266169	1.12142477	0.262107097	11.08141574
10387	STX8	26.80720989	-0.440216791	0.472622561	-0.929447904	0.252657012	12.77844844
10388	SUGP1	26.21188257	-0.40622007	0.475926025	-0.852526156	0.292262046	8.451040811
10389	TBL2	47.92414919	-0.496020884	0.425677104	-1.128505741	0.254909262	2.994014442
10390	TCP11L1	29.02205166	-0.660876445	0.46842228	-1.41082571	0.158296012	4.954792184
10391	TEX2	28.40560642	-0.420540009	0.488874969	-0.880675092	0.278492701	11.24887242
10392	THG1L	27.22146491	-0.10655097	0.555890667	-0.191676126	0.847995908	4.112941219
10393	TM7SF2	24.59792869	-0.266120225	0.487680566	-0.750758285	0.452798129	5.189117015
10394	TMEM267	20.0616084	0.452892848	0.521519447	0.852955297	0.292129759	4.292718911
10395	TMEM41B	27.49449601	-0.122505426	0.555968094	-0.240121452	0.810228252	12.08081448
10396	TOP2A	22.22849958	0.046227294	0.509847986	0.090865111	0.927599772	5.127117471
10397	TPSG1	26.22681257	0.542998905	0.55741987	0.97592206	0.229102594	7.944410478
10398	TRAPPC12	20.52712725	0.209449541	0.520066858	0.295127968	0.69274107	10.85459117
10399	TRIQK	22.22242625	0.181982225	0.646282451	0.281529721	0.778296466	8.297704947
10400	TWF1	22.22645212	0.172409964	0.608255201	0.282402406	0.776867618	5.974594511
10401	UBE2Q2	26.48617815	-0.622706246	0.479809246	-1.299904227	0.192622762	5.949178514
10402	UNG	28.51846961	-0.171229857	0.471228142	-0.262261622	0.716224766	5.472211081
10403	UPRT	27.92620117	-0.074756664	0.549912516	-0.12594282		

0.891866476	8.125917125
10404	VAMP4	28.04622902	0.225944572	0.544221848	0.415086077
0.678078891	14.01790591
10405	Z82206.1	21.92627422	0.642125242	0.649455004	0.98872928
0.222795564	7.494141021
10406	ZCCHC8	22.95462717	0.112928989	0.500204422	0.225720544
0.821418779	4.722295211
10407	ZFP1	27.54254672	0.282810755	0.545145022	0.518780761
0.602912625	5.912740517
10408	ZNF587	24.60142718	0.072877062	0.612424278	0.120422872	0.90412946
9.104811471
10409	ZNF621	24.26069819	-0.065594126	0.602220525	-0.10872005
0.912424529	4.971401492
10410	ZNF692	28.95525722	0.176021857	0.526705169	0.227986141
0.742922142	2.48457247
10411	ZNF700	26.08755622	0.406246917	0.564655844	0.719459224	0.47185795
7.708188704
10412	ZNRD2	22.22201419	0.429907281	0.617759095	0.712101667
0.476401822	11.11482708
10413	ZSCAN5A	21.20559489	0.412146021	0.5274152	0.782241006
0.422426889	2.114782428
10414	ABL2	22.7844149	-0.262524904	0.510806218	-0.512942164
0.607292428	4.17709428
10415	AC091045.2	22.00058265	0.229768494	0.50841027	0.668295758
0.50294482	4.119274172
10416	ACD	26.25704779	-0.048544597	0.476842882	-0.101804176
0.918912111	12.09111581
10417	ANGEL2	29.95122917	-0.211449749	0.528468825	-0.578298849
0.562994871	4.949747147
10418	AP001001.1	24.24520697	0.404040217	0.586955742	0.688265899
0.491222292	2.718415999
10419	AP001272.2	27.44862642	-0.522879769	0.479642918	-1.092228717
0.274722582	12.25951417
10420	AP5B1	22.20187426	0.247526025	0.626690269	0.288769919
0.697446255	7.110117027
10421	ATXN2	41.52698559	-0.268097084	0.452011252	-0.814252627
0.415442297	7.42487004
10422	B2GAT2	24.2248064	-0.288622422	0.491240081	-0.791127287
0.428869717	8.424844087
10423	BCAT2	29.97406201	0.217950145	0.522221642	0.417262912
0.676485281	5.942155124
10424	BTRC	24.5507402	0.555165199	0.594051588	0.924540285
0.250025214	4.891880579
10425	C1RL-AS1	20.24127225	-0.047652161	0.527721227	-0.090297961
0.92805044	9.2854741
10426	CDK5	21.46287622	0.117299666	0.508697528	0.220588222	0.81762471
8.171414458
10427	CEP41	29.49848299	0.217965501	0.526290428	0.604048778
0.545811212	11.11418097
10428	CLASP2	44.71288288	-0.252500281	0.4295519	-0.804228991
0.421264729	19.41421544

10429 CLK4 26.06212725 0.522459754 0.561940028 0.929742906
0.252504214 9.118042724
10430 CSKMT 19.60846805 0.162240497 0.716487121 0.226578285
0.820751601 5.459471825
10431 CSRNP2 26.78462616 -0.218988972 0.474068945 -0.672874642
0.501027046 8.242721848
10432 DCUN1D4 28.77524081 0.451024592 0.522952207 0.844689261
0.298284201 4.708017274
10433 DHX26 24.06944052 0.280244954 0.590829828 0.642566885
0.519856229 1.744114578
10434 DSE 27.07825795 0.724296127 0.552561265 1.20861026 0.19066627
4.148841027
10435 EDC4 29.51812017 0.070979709 0.522727222 0.122986221
0.894204278 10.8490107
10436 ELK2 22.09862224 -0.204622077 0.512084525 -0.594886217
0.551919464 8.410189418
10437 ESCO1 25.46801708 0.292257527 0.492719192 0.796210642
0.425851502 11.04842051
10438 EXOSC6 27.07527255 0.502269667 0.550256522 0.912625975
0.261429288 8.418745594
10439 EZH1 29.06149512 -0.508222772 0.468954551 -1.082972261
0.278277054 10.58994409
10440 FAM121A 28.4702177 -0.510692697 0.465449165 -1.097204024
0.272552215 4.74185119
10441 FANCD2 22.67015249 -0.08221492 0.49976525 -0.164507097 0.86922196
9.124975492
10442 FARP1 29.99664466 0.212074122 0.521762722 0.600020441
0.548485949 11.59941484
10443 FBXO21 20.97217062 0.118176474 0.512818827 0.220444882
0.817746089 5.474097247
10444 FCHSD2 22.05545197 -0.280680227 0.508969271 -0.747942626
0.454494156 5.41559884
10445 FILIP1L 51.20125206 -0.212562827 0.42272614 -0.720645582
0.471127601 8.91112457
10446 FMNL1 28.29105562 0.097082602 0.548768226 0.176909997
0.859579086 8.91112457
10447 GAN 24.76422196 0.041957788 0.522419998 0.080160842
0.926109224 8.1912127
10448 GTPBP6 27.5254825 0.074242222 0.55161624 0.124771627
0.892792429 5.711804489
10449 HACL1 42.86249216 -0.624019546 0.444756252 -1.402059222 0.16059915
4.111104971
10450 IFT52 27.52895702 -0.21562607 0.470910726 -0.457912844
0.647015059 2.15198491
10451 KDM1B 24.28222971 0.170592142 0.596058281 0.286200429
0.774724607 7.425107547
10452 KIAA0222 21.56724086 0.255196845 0.650286762 0.546215708
0.58491767 4.288048118
10453 LIG1 27.42116914 0.297180411 0.482207016 0.616292176
0.527701702 12.81814217
10454 LRRC8D 21.91202724 -0.106671876 0.505992028 -0.210816885

0.822020162	4.444554904
10455	MANF	27.89928226	-0.201056212	0.482000428	-0.4171287
0.676584272	7.404954102
10456	MBNL1-AS1	22.1890029	0.182922122	0.505624057	0.262767272
0.716021804	4.499808741
10457	METTL21A	22.69825677	-0.029920404	0.50228129	-0.079478167
0.926652297	9.104811471
10458	METTL22	22.07604059	-0.294442019	0.517509022	-0.762192494
0.445944528	4.112941219
10459	MINDY2	28.49076821	-0.02620471	0.540772556	-0.048457915
0.961251204	2.48457247
10460	MRPL40	42.84026844	-0.227741482	0.480622625	-0.494641802
0.620852989	7.712859174
10461	MTHFD2L	28.07726645	0.275511212	0.554454966	0.496904581
0.619256241	1.50999744
10462	MYL6B	28.24026982	0.254769085	0.540708492	0.471176407
0.627514765	5.70425071
10463	NEK4	27.74484226	-0.215049489	0.575228265	-0.547694729
0.582901522	5.714882199
10464	NIF2L1	29.54558019	-0.462251844	0.461956521	-1.002802972
0.215955452	4.114717404
10465	NIN	27.82601468	-0.289719102	0.469980122	-0.829224621	0.40697721
8.182847711
10466	NSUN2	27.56224201	0.768229618	0.555556606	1.282990698
0.166667718	11.29991114
10467	NXPE2	50.72121577	-0.545122751	0.428907066	-1.27096006
0.202742858	10.24492544
10468	OXSR1	29.89775795	-0.256709665	0.52682068	-0.664485699
0.506279499	7.958787111
10469	PEX12	20.45521159	-0.068245496	0.51956807	-0.121250442
0.895498091	4.981058471
10470	PLEKHA2	25.26629502	-0.214405224	0.481262022	-0.445412762
0.65602149	4.45579417
10471	PLK2	27.79219076	-0.287946772	0.46648271	-0.821640557
0.405611862	8.147108227
10472	PPRC1	29.02916279	-0.021768515	0.472200018	-0.0461002
0.962220286	11.07971184
10473	PUS7L	22.97064667	0.227147792	0.496085872	0.478027788	0.62262221
4.921275184
10474	RABL2	22.41412447	0.206722127	0.511262925	0.404256972
0.685950226	11.85851914
10475	RAD1	26.22170208	-0.159590025	0.519088828	-0.207442626
0.758506487	17.02507919
10476	RAD18	40.46584978	-0.228464702	0.456709122	-0.74109467
0.458626041	4.15451172
10477	RALBP1	21.27254571	-0.244271992	0.516860851	-0.472606875
0.626492677	8.175241481
10478	RFK	22.72024829	0.477424614	0.504445007	0.946455227
0.242916448	7.104454244
10479	RNF216P1	28.082284	-0.062100907	0.468720971	-0.124622606
0.892909477	2.14149194

10480	RPP28	21.50520627	0.252128112	0.512221199	0.492225064	0.622560248	4.444151148
10481	SATB1	24.0102414	0.580427299	0.504985921	1.149292208	0.250292879	4.154980948
10482	SCAF8	29.44481165	-0.021288146	0.529529705	-0.04029007	0.967782147	5.944242177
10483	SMC5	22.67860947	-0.082764667	0.500479742	-0.165270662	0.868652242	9.482972254
10484	SMIM20	28.27409262	-0.054695088	0.554055724	-0.098717666	0.921262442	9.79901401
10485	SOCS2	29.42217167	0.062471841	0.480122698	0.120116127	0.896474542	4.722295211
10486	SP1	44.28911592	-0.225762722	0.446810287	-0.751468201	0.452270921	1.7574404
10487	SPSB2	21.92567005	0.117622882	0.506120022	0.222416221	0.816214666	8.427101575
10488	SRP68	26.77607006	-0.559544107	0.476194029	-1.175022858	0.22998117	8.884577209
10489	SSH2	29.62895265	0.056068258	0.55206885	0.101560264	0.919105721	9.157458447
10490	STX17	28.99116214	-0.204160152	0.470784722	-0.422659245	0.664525906	7.414751549
10491	SUN1	22.11542507	-0.581992802	0.52517255	-1.108195297	0.267777428	7.404954102
10492	TIGAR	28.20806771	0.402924121	0.529097608	0.749259717	0.452700682	9.444744172
10493	TRMT2B	44.17522672	-0.224082575	0.449711655	-0.49828056	0.618286208	4.944144515
10494	TSC22D2	22.45914959	-0.018852648	0.505081444	-0.027225956	0.97022511	10.80101005
10495	UBFD1	25.25980574	0.109449609	0.582716254	0.187826561	0.851012612	2.994014442
10496	UTP14A	40.21125244	-0.218941822	0.457872928	-0.47817054	0.622528829	5.91942111
10497	WDR81	25.62947621	0.120191825	0.48497422	0.268450967	0.788252212	4.499511224
10498	ZDHHC17	21.26206262	-0.047190408	0.52258669	-0.08860606	0.929294991	4.491251782
10499	ZNF268	25.9942892	0.191464766	0.572971761	0.222578721	0.728697477	12.51974414
10500	ZNF277	22.59872887	-0.222705582	0.495820898	-0.672022967	0.500922679	9.110899082
10501	ZNF22	20.24987829	0.094841299	0.522520065	0.181507478	0.855969264	4.455844121
10502	AC008750.4	24.48268262	-0.101856047	0.512724482	-0.198265924	0.842827002	4.142489412
10503	AC092502.2	28.21850457	-0.269229241	0.462525821	-0.580829004	0.561255705	8.414548842
10504	AC105020.2	27.25245122	0.269887126	0.559160084	0.6615049	0.508288572	8.41591015
10505	AC108449.2	24.97547497	0.566897298	0.499522984	1.124854588		

						0.25642625	4.292718911
10506	AC144520.1	28.79777222	0.656907661	0.528240228	1.220246021	0.222271622	8.470728042
10507	AC221522.2	22.29692214	0.869292201	0.59800585	1.452652506	0.146042295	1.7574404
10508	ADAT1	24.12264885	0.178242206	0.501298222	0.255492425	0.722220719	4.884109885
10509	AKT1	27.84202568	0.021299909	0.47118241	0.066428291	0.947026847	9.425971707
10510	ANGPTL6	29.89212625	-0.064669799	0.540225828	-0.119706609	0.904715564	8.42148081
10511	ANKRD12D	26.44722726	0.24255298	0.484260459	0.70942842	0.478052447	4.974590549
10512	APOL1	21.90462024	0.256261616	0.527591426	0.485719826	0.627165825	5.474097247
10513	ASAP1	21.17820251	-0.266750689	0.529980855	-0.69200742	0.488922662	10.11851274
10514	BUD12	21.72869162	0.229005762	0.507565705	0.451184468	0.651856602	8.11411977
10515	C17orf58	25.25002514	0.505077284	0.572188417	0.881171651	0.27822492	12.40141884
10516	C20orf27	27.87406421	0.105627492	0.47150269	0.22404282	0.822722199	11.41588415
10517	CBR1	41.60602279	0.046552052	0.460024516	0.101196895	0.919294162	4.484049514
10518	CHEK2	41.07965884	0.012285045	0.457946457	0.02901004	0.976856582	4.194004227
10519	COP1	28.6082475	-0.51727644	0.485991182	-1.064579892	0.287066082	14.74925127
10520	CTBP2	41.78225284	0.102041442	0.465224222	0.21929101	0.826422264	7.911077889
10521	DENND4A	27.8005956	-0.210605972	0.465892076	-0.666690824	0.504969641	2.759454418
10522	DESI1	58.72129586	-0.442528015	0.41279691	-1.069457021	0.284862781	12.79571081
10523	ECAIT	24.01287542	0.22012241	0.502847575	0.626619179	0.524272929	5.47074414
10524	EMSY	40.82248964	-0.07468722	0.456214995	-0.162674942	0.869987045	7.440778117
10525	FAM216A	27.52282828	0.021675121	0.56014628	0.028695459	0.969122194	11.40210128
10526	FBXO2	20.72296227	0.278447497	0.518979077	0.729215226	0.465869958	8.411787477
10527	FCAR	25.24266124	-0.212756027	0.481282228	-0.44196894	0.658511682	10.08901912
10528	FCRLB	22.14259929	0.778295642	0.602482206	1.291815152	0.196421167	9.408104911
10529	FICD	42.10975602	-0.091072927	0.462207264	-0.19699651	0.842820269	9.849559157
10530	FUT11	21.68228506	0.141822949	0.510226156	0.27790104	0.781088226	4.442944711

10531	GEMIN6	26.89242067	0.418217691	0.595216967	0.702620661	0.482285949	9.900547814
10532	GFOD2	29.0595121	0.572522526	0.521818942	1.076555247	0.281678962	9.171785129
10533	GNL1	47.90161445	-0.158700924	0.44021707	-0.260506088	0.718468705	4.44420111
10534	GTF2C1	21.66810822	-0.084815254	0.509288742	-0.166526676	0.867724622	8.12870141
10535	IMPACT	26.29825782	0.212041125	0.565064824	0.277020699	0.706158222	2.987570484
10536	JMJD1C	22.26514125	-0.141026558	0.497725778	-0.282256279	0.77690274	4.499858812
10537	KMT5B	29.90899814	-0.210906798	0.524129126	-0.294852678	0.692950878	7.415440142
10538	KRBOX4	25.29924921	0.22569299	0.576586674	0.582209067	0.560425867	11.2827415
10539	LIX1L-AS1	41.99420768	-0.050750587	0.459452276	-0.110458622	0.912045664	8.707247141
10540	LRRC42	27.56214169	0.287991481	0.54654461	0.709899017	0.47776676	15.49144241
10541	MAN1A1	24.79744887	-0.551145091	0.495647185	-1.111970587	0.266150797	4.722445272
10542	MCMBP	26.52252846	0.405628701	0.564258979	0.718760072	0.472288762	5.714098014
10543	MEAK7	20.11726682	-0.028189122	0.551180041	-0.06928612	0.944761866	8.195109108
10544	METTL22	29.52225589	0.718756029	0.540517946	1.229754228	0.182599258	2.455524841
10545	MMS22L	21.6004605	-0.080129222	0.529729212	-0.151282582	0.87975202	5.184291478
10546	MPHOSPH9	22.78770207	-0.190222894	0.646610451	-0.294240887	0.768497422	4.105247714
10547	MRPS21	27.55019722	-0.100402652	0.472846292	-0.211890762	0.822192258	15.7092181
10548	MRTO4	28.56412572	0.477817429	0.526465942	0.890676166	0.272102925	8.417044711
10549	NMRAL1	28.27779655	-0.046022112	0.468182882	-0.098220794	0.921677567	1.7574404
10550	NR2C2AP	24.17876409	0.217520892	0.492095644	0.442050025	0.658452006	4.474440814
10551	P2RY14	45.58495051	-0.622595944	0.42062204	-1.469024842	0.141826051	7.187544287
10552	PARP12	22.17114942	0.180488542	0.499757806	0.261152024	0.717985804	7.454447415
10553	PDCL2	29.59621498	-0.280025422	0.465182515	-0.601990452	0.547180492	4.44420111
10554	PEA15	28.12729804	-0.065226624	0.468885602	-0.129222181	0.889194772	8.91172052
10555	PGAP2	26.50080562	-0.192274508	0.585890678	-0.220052202	0.741260518	4.981058471
10556	PIK2C2A	22.559829	0.264724502	0.524461862	0.695445222		

						0.486776222	5.494181158
10557	PLEKHA2	42.15144011	-0.547898766	0.446247278	-1.227516762	0.219628425	8.441711082
10558	POP7	20.51161708	0.259760822	0.517974062	0.501492901	0.616022569	8.418408109
10559	PSPC1	46.41542524	-0.447987662	0.429560422	-1.042897901	0.296995582	5.111759412
10560	PSTPIP1	27.56492697	0.281752224	0.551988778	0.510421072	0.609749492	4.444504844
10561	PTPRN2	42.21556146	-0.221211446	0.445404009	-0.519229512	0.602520977	4.45579417
10562	RAB25	20.04459417	0.654980226	0.527026206	1.242761114	0.212955876	4.9449218
10563	REEP2	26.71469492	-0.0522422	0.572871411	-0.092952401	0.925940581	11.84455177
10564	RNF11	42.72202892	-0.521220477	0.441060961	-1.204427762	0.228420424	2.974179159
10565	RNF129	29.47291272	-0.022048062	0.520788856	-0.042422279	0.965264918	11.21454248
10566	RNF144A	27.02780876	-0.415522851	0.472009675	-0.880229097	0.278681054	8.450890424
10567	RPL22L1	27.94742289	0.29212118	0.480191189	0.608242482	0.542959688	8.155414277
10568	RWDD2B	22.71427627	0.089424681	0.496220859	0.180195289	0.856999257	8.711512994
10569	SEMA2F-AS1	22.72690886	0.045112768	0.505905259	0.089172264	0.928944928	7.917498511
10570	SEPTIN9	27.201558	0.199869161	0.482628861	0.414117422	0.678788115	12.81449927
10571	SFSWAP	28.76946892	0.299212068	0.522692287	0.561698484	0.574221468	2.741491409
10572	SLC25A20	24.96119811	0.107665626	0.491142008	0.219214859	0.826482681	4.94555191
10573	SLC2A10	24.61261622	0.269004095	0.594222025	0.620881272	0.524677678	9.900597884
10574	SLC20A6	22.12672522	-0.206021269	0.511422642	-0.598280596	0.54958601	5.115924247
10575	SLC9B1	26.20402852	0.222070602	0.561287851	0.575485562	0.564962952	11.78121522
10576	SP110	24.22671467	0.299162468	0.492758081	0.605890779	0.544587282	5.124291215
10577	SPRTN	28.85124255	0.704750295	0.529692594	1.20582428	0.19160892	4.128048991
10578	TAF1C	26.22252808	0.485425227	0.490661928	0.989247856	0.222492977	2.711945205
10579	TCTEX1D1	46.62642914	-0.491468945	0.427601204	-1.149262867	0.250406285	4.905957189
10580	TGDS	26.4218592	0.106255012	0.479026767	0.222018477	0.8242995	7.415111754
10581	TIMM9	28.52459105	0.224875279	0.526659588	0.419027785	0.675195826	2.149948929

10582	TMCO2	27.55282222	-0.5289169	0.47282191	-1.116277845	0.264202222	9.481188049
10583	TMEM161A	27.2286052	0.525085588	0.548212744	0.957627106	0.228245781	2.72412442
10584	TMPO	45.0112209	-0.111280578	0.444284252	-0.250471578	0.802222682	8.91480714
10585	TRAPPC6A	29.95982414	0.074792702	0.52256842	0.142852729	0.886405702	18.71792528
10586	TRIM52	21.12045205	-0.176622922	0.521724522	-0.228557824	0.72494286	7.454447415
10587	TRIP11	22.42805982	-0.016412105	0.501456622	-0.022728862	0.972890808	9.441920814
10588	TTC14	29.88072752	-0.042891621	0.466792924	-0.094027852	0.925087022	4.700412982
10589	VPS26B	26.78162956	-0.518868126	0.476522624	-1.088861264	0.27621507	4.724180597
10590	ZBED6	27.58745956	0.227051182	0.567208948	0.400295487	0.688928891	4.729044005
10591	ZBTB42	45.62205128	-0.277628908	0.442622497	-0.625821017	0.521422245	4.417591887
10592	ZNF225	22.06172467	0.270956546	0.620912425	0.426282778	0.662558206	2.72412442
10593	ZNF44	24.82699254	-0.241029182	0.48701822	-0.70025944	0.482765297	4.455508744
10594	ZNF507	25.59764824	0.29950112	0.582181872	0.514445972	0.606940224	8.275014192
10595	ZNF611	20.17625855	-0.220844687	0.527078272	-0.616008226	0.527889025	5.474424854
10596	ZNRF1	29.68888512	-0.426025122	0.462506924	-0.940720547	0.246842961	5.711111404
10597	AC087276.2	22.60017112	0.467577094	0.625141616	0.747952876	0.454487979	7.147747011
10598	ACSF2	55.88040572	-0.292428925	0.425826104	-0.922942726	0.255516121	8.10081974
10599	AIG1	29.28820281	0.081917201	0.568141868	0.144184588	0.885254711	8.481019288
10600	AMFR	25.8452102	0.212040016	0.589162502	0.261597442	0.717652876	4.140854174
10601	ANKRD49	22.25570814	0.292914208	0.499629178	0.788412228	0.420454971	7.947545948
10602	ANO10	44.22586021	-0.291670502	0.427148482	-0.66721152	0.504627024	17.79508075
10603	AURKA	27.98976905	0.440570242	0.561727847	0.784212802	0.422856589	9.121190408
10604	C12orf49	26.28792409	0.069128658	0.475582522	0.145276784	0.884412292	4.119414225
10605	C4orf22	24.57226728	-0.227080402	0.492962884	-0.480928542	0.620567286	2.141745457
10606	CCDC141	20.14040142	0.668664852	0.676876202	0.987868756	0.222216929	5.448121448
10607	CD2EAP	40.56721925	-0.199226812	0.452720009	-0.429207095		

0.660429024 9.149151417
10608 CENPN 28.26948968 0.484821159 0.502660276 0.964510589
0.224790002 12.55918891
10609 CEP192 22.22708997 0.211089202 0.642627759 0.48408927
0.628222516 5.951012871
10610 CLCN5 22.84499894 0.266596761 0.624507258 0.587017521
0.557191929 7.190219471
10611 CMTR1 28.50747777 -0.127987228 0.5589019 -0.228997859 0.81887058
4.921275184
10612 CREBBP 50.41485418 -0.207828989 0.427971285 -0.485627582
0.627224145 14.05171981
10613 CRELD1 48.47005797 -0.2296855 0.422152422 -0.784216996
0.422912794 12.41402194
10614 DRAP1 47.48522621 0.048642655 0.444427825 0.109447602 0.91284748
11.08817781
10615 E4F1 27.87102764 -0.198801125 0.470088624 -0.422901274 0.67226722
10.91517449
10616 EFL1 45.72024521 -0.28120162 0.422898755 -0.648211678
0.516782282 9.458555257
10617 ERCC6L2 26.22277475 0.659822492 0.56242777 1.171086212
0.241564125 11.18704907
10618 ESF1 21.75842785 0.504622456 0.51067592 0.988167712
0.222070518 9.599212444
10619 FRMD8 51.70285579 -0.259150122 0.424652454 -0.826290759
0.408629174 9.21418125
10620 GALK2 26.2568454 -0.127262922 0.474112275 -0.268422974
0.788272757 7.178988185
10621 GCDH 29.52220462 0.515241875 0.527605585 0.976566278
0.228782871 7.440818179
10622 GLRX2 25.45085267 0.188926096 0.482950778 0.290282874 0.69625245
2.981899791
10623 INTS1 49.11729646 -0.226218982 0.422129298 -0.522486256
0.600625912 7.404118854
10624 KCTD18 21.28727222 0.881844402 0.644851794 1.267514846
0.171462987 7.284411414
10625 KIF22 26.01029566 -0.425792465 0.487411442 -0.872581186
0.282246276 11.89881511
10626 KRAS 22.46760242 0.42817457 0.509008418 0.841192495
0.400229544 8.275074155
10627 LAMTOR5-AS1 47.17250026 -0.262590569 0.427291466 -0.848280461
0.296226121 4.450115474
10628 LCLAT1 42.71729872 -0.064789241 0.458581872 -0.14128172
0.887647282 10.24120192
10629 LRIF1 49.62227009 -0.224425029 0.428981502 -0.522157846
0.600864412 9.294020201
10630 LYAR 21.41527648 0.521266079 0.66091545 0.788702122
0.420285542 4.891880579
10631 MAP2K20 50.52702142 -0.564446272 0.415147226 -1.259628797
0.172947421 9.299450924
10632 MAP7D2 28.20706199 -0.459750474 0.467976218 -0.982422721
0.225891622 7.14111429

10633	MPLKIP	46.68826514	-0.492794167	0.429256726	-1.150079002	0.250111222	9.911889111
10634	MT-ATP8	42.40825411	0.265206297	0.466626029	0.568248915	0.569798071	4.480041458
10635	NARS2	21.67929858	0.188027288	0.514400427	0.265527084	0.714717992	9.227512414
10636	NBEAL1	50.62008726	-0.479588624	0.418120561	-1.147010281	0.25127722	7.284411414
10637	NEMP1	26.12455017	-0.109875271	0.476441159	-0.220616874	0.817612457	10.88174449
10638	NFATC2IP	24.12229747	-0.244478219	0.49271279	-0.496188092	0.619761712	2.495019449
10639	NFRKB	29.45017982	-0.216122984	0.552199921	-0.571464251	0.567684987	4.971401492
10640	NIPAL2	26.95279265	0.181721626	0.480976018	0.277818476	0.705565448	7.404118854
10641	NOA1	49.04099241	-0.582172889	0.418865548	-1.292269886	0.162840687	4.444750007
10642	NSMCE2	42.25206946	-0.222808596	0.441008512	-0.756920982	0.449097202	9.119255041
10643	NUBP1	28.20922165	0.54012174	0.482142752	1.117952445	0.262587204	9.149151417
10644	ODR4	22.01172164	0.421285277	0.506896714	0.850824825	0.294861112	5.704200449
10645	ORC5	48.00019971	-0.122741257	0.427988191	-0.202070621	0.761826025	9.424210194
10646	P2RY8	22.18029547	-0.028852474	0.504967085	-0.076940606	0.928670794	7.147797082
10647	PARVB	41.57498409	0.152629521	0.460090096	0.221728254	0.740086827	8.410129554
10648	PIGW	28.09696154	0.697450857	0.542486516	1.285655654	0.198562264	9.244019454
10649	PITPNM1	20.8206605	-0.244005165	0.555090907	-0.429576944	0.660242542	5.927887199
10650	PLXNA1	22.92672048	0.412089426	0.504009656	0.817622086	0.412572012	2.019450421
10651	PPM1D	25.28604019	0.224057049	0.492402927	0.475225454	0.624547892	11.79724228
10652	PPM1M	22.74252842	0.216489761	0.498116545	0.424616684	0.662840682	7.419584915
10653	PROSER1	20.71122846	0.097145872	0.516858081	0.187954627	0.850912212	9.894977154
10654	PTPN22	20.99291578	0.628680911	0.524704602	1.194455609	0.222299795	5.925051851
10655	QTRT2	24.20849527	0.408772048	0.598002755	0.682561005	0.494252416	11.90721114
10656	RAD51C	28.56054602	-0.0602478	0.465612272	-0.129294758	0.897045295	9.124915421
10657	RBSN	21.15571879	-0.224471812	0.522025666	-0.420001487	0.667194559	5.925051851
10658	RFLNB	21.28559406	0.269028801	0.515120192	0.716292584		

						0.472748256	12.78992999
10659	RNF145	21.8422017	-0.274860726	0.525917627	-0.712774619	0.475985224	14.11902001
10660	RPL7P9	45.44721411	-0.265892086	0.424489412	-0.611966778	0.540559728	4.520844214
10661	RUSC1	27.82992895	-0.289820799	0.472252125	-0.825275845	0.409215002	15.71747172
10662	S100PBP	40.28284676	-0.225269829	0.452627441	-0.718610106	0.472281185	18.90899044
10663	SKP2	27.59874981	0.545986547	0.550507045	0.991788482	0.221200717	17.15915518
10664	SLC29A4	45.05862454	-0.406915572	0.456517448	-0.891247252	0.272742906	2.518851188
10665	SMIM19	25.58276722	-0.27140296	0.481190672	-0.564022725	0.572727979	18.95121041
10666	SNHG12	21.72167847	0.184962905	0.506820852	0.26494928	0.715149268	4.518010949
10667	SNRNP25	24.09405204	0.0544152	0.508266119	0.107060451	0.914741022	1.51485814
10668	STUB1	28.22611866	0.506602002	0.542525206	0.922785191	0.25041471	5.757910854
10669	SURF6	57.99114065	-0.259205081	0.422466767	-0.612551411	0.5295118	4.758741172
10670	TMEM224	28.06212762	0.70062184	0.545170805	1.285160225	0.198726296	5.145279818
10671	TMEM255B	28.27602226	-0.525861608	0.468228057	-1.144421204	0.252448957	5.145279818
10672	TSPAN6	25.82272768	0.441244841	0.568272559	0.776504879	0.427450941	17.72175111
10673	UGGT2	20.92785299	-0.017601029	0.518244198	-0.022956258	0.972912022	14.74099482
10674	UPF1	27.87412057	0.190865592	0.646994759	0.295002207	0.767991254	17.94097072
10675	UPF2B	22.91687206	0.112625102	0.491262477	0.221264852	0.81710905	11.57748118
10676	URB1	20.18556672	0.971594926	0.662257229	1.467095984	0.142249946	14.12552517
10677	USP7	41.72664596	-0.205720251	0.451542419	-0.455592511	0.64868221	8.499117497
10678	XPO4	27.91625028	-0.279242891	0.577217628	-0.482775742	0.628545022	11.84509145
10679	ZBED5	24.24151596	-0.474856995	0.500095961	-0.949521754	0.24225022	1.774511411
10680	ZMAT5	25.22580727	0.222705062	0.581200169	0.572444884	0.567020616	15.44711441
10681	ZNF266	40.84202562	-0.288228177	0.461712215	-0.624496262	0.522201724	11.58051752
10682	ZNF820	21.09264152	-0.408062287	0.52914228	-0.756871728	0.449126709	4.14228579
10683	ACAD10	24.26698294	-0.009957625	0.492975812	-0.020199014	0.982884614	11.0784791

10684	ADPRM	20.21458752	0.475994262	0.524025785	0.90824149	0.262697829	2.504149428
10685	AFG2L2	46.22072212	-0.412025127	0.427065292	-0.942721076	0.245818474	2.504149428
10686	AP4M1	42.29718262	-0.124290212	0.442269846	-0.202885209	0.761977267	11.27129904
10687	ARMCX6	21.89865712	-0.195248694	0.515809206	-0.278528906	0.705027725	7.474212521
10688	ASMTL-AS1	21.88741587	-0.102824965	0.516466917	-0.201029265	0.840675691	8.441518274
10689	ASTE1	40.70256289	0.077881102	0.482220464	0.161127606	0.871985022	4.444402444
10690	ATG4A	22.4617214	0.026497709	0.504420797	0.052529919	0.958106456	7.412815014
10691	BAZ1A	25.25044208	-0.28881812	0.510065652	-0.566227146	0.571222582	4.92121405
10692	BAZ1B	44.72526206	-0.190979826	0.425985245	-0.428041948	0.661255875	9.25159915
10693	C11orf54	25.78601728	0.502722074	0.585497248	0.858641224	0.290528487	14.09957911
10694	C16orf72	22.28185081	-0.22814012	0.500922028	-0.45542121	0.648798974	5.980045892
10695	CAMTA1	27.84277222	-0.158676145	0.465109246	-0.241158871	0.722982984	8.447801518
10696	CCNT2	27.52224456	-0.122929122	0.581788286	-0.220219675	0.817921074	5.48757491
10697	CCS	28.74598067	-0.274142859	0.464228828	-0.805947058	0.420272276	4.111158482
10698	CDK17	21.28554292	0.462424767	0.512477272	0.904202886	0.265824817	4.941140021
10699	CHRAC1	25.22782822	-0.252818244	0.484488675	-0.521825085	0.601792122	4.984417947
10700	CLUHP2	29.07065109	0.676098502	0.522525982	1.269582761	0.204222292	8.144854544
10701	CNEP1R1	29.70072722	0.101069125	0.524295041	0.18916251	0.849964666	10.45127974
10702	COQ8B	21.84021855	0.726171727	0.667410918	1.088042924	0.276576159	8.124551454
10703	DEPDC7	21.89212662	0.12182889	0.524092524	0.222475912	0.816168292	4.499757415
10704	DGKD	22.88278926	0.02156607	0.507794895	0.06216202	0.950422002	9.444277549
10705	DHRSX	29.40222089	0.127885187	0.464062445	0.297125725	0.766270525	7.291774295
10706	EMC10	29.79100786	0.227742917	0.52648528	0.641504957	0.521194662	8.158298588
10707	FAM222B	55.2540297	-0.290117196	0.417200298	-0.695224022	0.486914914	4.451909487
10708	FBXL15	42.79261007	0.022221964	0.445855126	0.072516748	0.942190678	5.991257118
10709	FP671120.8	21.88164505	-0.469492668	0.52481086	-0.877866744		

0.280016029	5.454524191
10710	GPKOW	45.81246002	0.062042208	0.445110524	0.12928822
0.889142201	5.474182594
10711	HCFC2	21.02081762	0.069276648	0.527902776	0.121229687
0.895592612	8.421419482
10712	HPCAL1	48.68111086	-0.141969692	0.429792252	-0.220220897
0.741157505	11.45481158
10713	KIFAP2	29.165799	0.082561672	0.467918248	0.176444651
0.859944629	7.892772409
10714	LMNB1	45.14582825	-0.60848767	0.429524066	-1.416622609
0.156592295	4.941415275
10715	MFSD8	24.86574769	0.226641872	0.602082475	0.559128227
0.576074205	10.41811812
10716	MPPE1	40.96082244	-0.586027724	0.451861726	-1.296918282
0.194659206	5.445041221
10717	MTERF4	22.92222292	0.248288056	0.506246209	0.490252258
0.622884654	5.484501109
10718	MTMR4	22.88652449	-0.188058982	0.504797568	-0.272542261
0.709488224	5.429414224
10719	NAA10	25.29150956	0.029998248	0.480594022	0.062419211
0.950228917	2.158554414
10720	NDUFAF6	25.4266248	0.067909975	0.482266888	0.140814094
0.888016809	8.411484179
10721	NDUFC2	22.42924888	0.206027861	0.504792484	0.408142677
0.682168195	11.85180742
10722	NFKB1	28.46512947	0.250229126	0.478402418	0.522072452
0.600922825	5.000704578
10723	NFKBID	22.7594292	0.42190048	0.496147647	0.870507969
0.284022866	8.291985128
10724	NUP25	21.20650265	0.142821101	0.512699704	0.278042962
0.780978612	7.70525114
10725	PDE6D	21.92974582	0.028846125	0.508595506	0.056717222
0.954770454	5.479118941
10726	PDK2	44.49221954	-0.208799665	0.426284586	-0.478585921
0.622222229	4.194740484
10727	PDK2	48.12484842	-0.475226884	0.41985528	-1.121906142
0.257672896	8.144171914
10728	PDZD11	42.29424828	-0.1675547	0.44205495	-0.278180405
0.705296581	14.24719142
10729	PHF5A	22.21705278	0.500590927	0.518080592	0.966241429
0.222922282	11.25548708
10730	PLEKHA5	26.42444209	-0.051559028	0.48022024	-0.107262162
0.914500877	4.941415275
10731	PRADC1	45.49784866	-0.494017595	0.440844556	-1.1206162
0.262451224	11.11741119
10732	PRMT2	24.62200196	-0.28176046	0.491571024	-0.57218261	0.56652028
7.944254707
10733	PRMT9	28.42410811	-0.121996482	0.562268118	-0.216972078
0.828220105	11.10124197
10734	PRR2	27.7560812	0.264029119	0.554809949	0.65615102
0.511726954	5.118410424

10735	PSMC2IP	29.24849949	-0.225967279	0.461215661	-0.48982222	0.624252622	7.701544875
10736	PSME4	55.28057099	-0.581182812	0.404022228	-1.428488579	0.150295476	4.924944481
10737	QKI	20.22719229	0.42442165	0.522274054	0.814478408	0.415270927	8.121980591
10738	QPRT	29.78255188	0.228420205	0.525184619	0.644282504	0.519226779	8.957794422
10739	RACGAP1	40.00125849	-0.242811674	0.454142684	-0.75705484	0.449017008	7.448441827
10740	RBM15B	28.00652974	0.220942962	0.548119161	0.42122897	0.672507577	2.502284119
10741	RHOBTB2	27.8221851	0.257298717	0.552542997	0.645655484	0.518502496	1.511721588
10742	RYBP	24.85662857	-0.122081972	0.486881088	-0.272225677	0.784595192	4.491044807
10743	SAMD9L	29.96449911	-0.601669122	0.459286262	-1.210008704	0.190192891	10.14790715
10744	SEC61A2	28.40888209	0.52102998	0.487527065	1.068720112	0.285195806	8.291985128
10745	SLC21A2	29.78260194	0.486471708	0.524750661	0.927052064	0.252898984	10.41547044
10746	SLC9A9	26.95648501	-0.292826246	0.60265694	-0.485908859	0.627021787	4.948022272
10747	SMYD5	24.19258824	-0.026054081	0.492958459	-0.072128172	0.941696166	12.08424291
10748	SNRNP25	46.01022712	-0.22279474	0.425901614	-0.526247499	0.591718409	4.447189055
10749	SRBD1	44.49879011	-0.229926544	0.426526414	-0.778712917	0.426148845	7.479924142
10750	SRSF9	41.77722057	-0.247280644	0.44645242	-0.778089217	0.42651626	7.899294041
10751	STAG2L4	21.26291021	0.221269087	0.512968441	0.45016205	0.652592598	4.991198527
10752	TBC1D21	29.04502285	-0.078161862	0.568801281	-0.127415026	0.890702747	7.919191404
10753	TBC1D7	22.68597059	-0.026404282	0.494722767	-0.072585272	0.94124029	5.714821142
10754	TMEM97	29.58000296	0.411625917	0.527119922	0.766257567	0.442462574	8.121980591
10755	TRIM59	24.44025152	0.19496789	0.487224701	0.40016011	0.689028592	5.459271427
10756	UBE4B	29.95674622	-0.212420798	0.542976428	-0.292057206	0.69427722	10.2740757
10757	UIMC1	40.24618642	-0.178719126	0.458962947	-0.289296895	0.696982569	4.404909029
10758	VPS50	26.76244212	-0.05622221	0.582412642	-0.096722885	0.922945671	9.441229548
10759	ZNF175	27.2822275	0.207999761	0.557551212	0.272059405	0.709104221	8.415809051
10760	ABAT	22.27757228	-0.017156222	0.722071045	-0.022726899	0.98107045	

							2.752921798
10761	AC025259.1	25.56012227	-0.065921721	0.496497826	-0.122772421	0.894272567	7.414450271
10762	AC062977.6	24.50516292	0.259654492	0.491661774	0.72150794	0.464468957	9.914472411
10763	AC078795.1	29.26170626	0.197022616	0.524811026	0.268296697	0.71257746	8.472471111
10764	AIFM2	28.82899526	-0.190922978	0.460142726	-0.414942926	0.678182678	7.442541218
10765	AL161668.2	40.79776805	-0.285448524	0.450005882	-0.624221762	0.525870857	9.420150877
10766	ALG12	25.49029506	0.558942921	0.488040526	1.14527975	0.252092292	7.418194291
10767	ALKBH2	26.82799284	0.022977192	0.478225548	0.068941545	0.945026149	10.82474174
10768	ARL15	57.26202528	-0.201269152	0.410484244	-0.722925792	0.462987871	7.418194291
10769	BTG1	24.47964481	-0.016782471	0.502707759	-0.022286129	0.972266662	11.41489157
10770	C15orf40	26.59605042	0.249722266	0.498677279	0.500791246	0.616517977	11.82951108
10771	CAMSAP1	24.6145449	-0.071729284	0.490586228	-0.146221922	0.882728206	2.751097451
10772	CASP6	22.27124967	-0.22462626	0.518207152	-0.452765284	0.650717692	10.44070088
10773	CFAP26	27.49622229	-0.485252268	0.479219624	-1.012797126	0.211157074	4.952854429
10774	CFDP1	27.22610444	-0.082960758	0.469455054	-0.176717146	0.859720572	4.420217024
10775	CHD8	29.05561989	-0.280780281	0.458796187	-0.611992492	0.540542062	14.58271422
10776	CLOCK	29.25041504	0.148467029	0.526474528	0.276745722	0.781975254	9.084188484
10777	CWC27	28.20209225	0.406125586	0.541618597	0.749826857	0.452252967	8.294710241
10778	DENND2	25.514917	-0.248871222	0.500549919	-0.696975886	0.485817885	4.712155497
10779	DMAC2L	28.12992992	0.086720869	0.464646942	0.186659721	0.851927429	15.05924241
10780	DMXL1	48.62574422	-0.469894728	0.416992242	-1.126866564	0.259798902	8.890194847
10781	DNAJC14	25.71806072	0.126542706	0.48567616	0.260549552	0.794429898	15.44711452
10782	DNAJC15	28.59690277	-0.224641851	0.467926814	-0.692772827	0.48782468	7.901179449
10783	DOP1B	25.57699426	-0.188804842	0.485646501	-0.288770109	0.697446214	9.414580184
10784	DSTYK	24.80296615	-0.212522499	0.501684707	-0.422627588	0.671820128	9.444277549
10785	EARS2	22.21006846	0.726695796	0.508971086	1.427774221	0.152256855	12.04284129

10786	EEPD1	40.51906626	-0.229919791	0.45065712	-0.754275869	0.450682589	1.992981199
10787	EHMT1	51.26864516	-0.242748608	0.414086042	-0.827722162	0.407827298	5.000704578
10788	GADD45GIP1	40.79219748	-0.142942289	0.449610286	-0.217925086	0.750541762	10.82190741
10789	HAS2	25.64747744	-0.152200191	0.482587828	-0.215590612	0.752212264	8.184451921
10790	IDI2-AS1	22.15940422	-0.285499582	0.521158715	-0.729697084	0.459482818	11.41449142
10791	JMJD4	40.70758764	-0.2452146	0.46128274	-0.521591685	0.595008825	4.444114111
10792	LDLRAD4	26.72129602	0.122668602	0.48905716	0.250826717	0.801948085	11.17570441
10793	LIPN	27.50825961	0.409902645	0.485754659	0.842849126	0.29875272	11.75112557
10794	LMBRD2	26.28129246	0.550861528	0.586250427	0.929625169	0.24740472	7.187292117
10795	MAP2K2	40.64894154	0.116858271	0.459124587	0.254524096	0.799090691	5.974445141
10796	MED14	42.85821286	0.221828842	0.452626042	0.512197044	0.608512112	7.414450271
10797	MED22	27.00442452	0.078968191	0.574281604	0.127482845	0.890648261	5.924950454
10798	MLLT10	25.62215956	-0.029257864	0.480195782	-0.060929022	0.951415728	9.171582817
10799	MRPL55	27.72975877	-0.580021988	0.481544045	-1.204504529	0.228294629	15.10147224
10800	MTMR9	21.11109709	0.522852524	0.529994571	0.986772871	0.222752507	8.410128258
10801	NIBAN2	29.56780811	-0.425769252	0.464175122	-0.917259957	0.259006425	4.955589228
10802	NOTCH1	42.47122682	-0.654867114	0.446178458	-1.467724624	0.142179027	11.79918415
10803	PDCL	46.87101242	-0.525266164	0.421822892	-1.269169846	0.204280492	8.14401911
10804	PLEKHG4	28.26462976	-0.418274682	0.468094781	-0.892781986	0.271428549	7.485404854
10805	POMK	29.78528602	0.228600964	0.526546425	0.642060028	0.520185142	9.294414145
10806	POU2F1	26.25295784	0.111545127	0.482595444	0.220657957	0.817580528	8.448291701
10807	PRR5L	47.68180092	-0.297172889	0.422145147	-0.70229776	0.48249249	2.482484987
10808	PSMB10	22.48699814	-0.019282547	0.514656228	-0.027466849	0.970112772	11.54018172
10809	PSMG2	22.68596952	0.178108542	0.492146919	0.261167202	0.717974282	9.248511107
10810	PTPRB	21.97465974	-0.245225277	0.525924869	-0.457752222	0.647120465	9.149001147
10811	RAB44	45.40468164	-0.621681768	0.429146201	-1.448647957		

0.147425921 11.10110217
10812 RBL2 22.62662749 -0.204075406 0.500701505 -0.407578974
0.682582802 15.44111504
10813 RNF220 20.27499172 0.224070272 0.521145425 0.641020795
0.521502675 4.140550444
10814 RPP20 26.09221807 0.172995289 0.497402468 0.247797409
0.727992226 4.112451442
10815 RTL10 25.64459202 -0.215205068 0.492629257 -0.629829276
0.522282522 7.910485244
10816 SLC25A17 41.05682266 -0.054824712 0.449757252 -0.121898422
0.902979465 11.84771524
10817 SLC25A2 45.26998179 -0.190025626 0.424927507 -0.426926248
0.662157511 11.55124421
10818 SLK 29.0197129 0.222092292 0.524899595 0.604024272
0.545827428 5.445144945
10819 SMARCD2 28.42127169 0.081576201 0.570806552 0.142914094
0.886258027 4.471504181
10820 THAP1 27.7559801 0.211910242 0.556854044 0.560129257
0.575291276 11.08424989
10821 TMEM248 40.24592505 -0.542948224 0.457476284 -1.186822827
0.225292169 11.14458221
10822 TMEM52 24.92422526 -0.054106552 0.48668226 -0.111174057
0.911478222 8.178145891
10823 TMLHE 21.8451249 0.126041109 0.562121872 0.222821657
0.822896077 7.442011104
10824 TSPOAP1-AS1 29.05910828 0.120054278 0.555546469 0.216101225
0.828908827 10.45491748
10825 UBE2D2 54.17764989 -0.222665288 0.42222476 -0.549614409
0.582582874 8.429925714
10826 UBXN7 22.25254622 -0.212264261 0.509897521 -0.614267099
0.528972772 10.42541774
10827 VAMP1 24.69626946 0.121122802 0.48554114 0.270077625
0.787100528 5.924950454
10828 VIRMA 26.47512682 0.024816225 0.492282052 0.050208207
0.959876704 7.101419912
10829 YBX1 47.56888766 -0.602172167 0.420627622 -1.422949601
0.151586646 4.149159118
10830 ZMYM4 42.88572008 -0.588695426 0.444449022 -1.224551026
0.185220105 14.80994755
10831 ZNF575 42.2189846 -0.22650029 0.446288952 -0.529926282
0.596162972 10.19058992
10832 ZNF701 21.1781491 -0.085958205 0.522786984 -0.164422001
0.869298158 11.07294995
10833 ZNRD1ASP 47.29742842 -0.405464416 0.421271082 -0.962250219
0.225922928 11.87044082
10834 ZZEF1 41.20448582 -0.071720828 0.448298006 -0.16000702
0.872875544 5.711705491
10835 ABHD18 21.72142494 0.186105267 0.510415678 0.264615202
0.715298591 9.450048142
10836 AC009218.1 28.16922222 0.122558277 0.466022266 0.262987972
0.79255985 7.914404111

10837	AC010972.2	24.00128671	0.08804206	0.662598706	0.122672647	0.894451485	8.410088194
10838	AKT2	24.84792012	-0.240276227	0.497820016	-0.682522854	0.494270197	10.45104117
10839	ARRDC2	28.24625212	-0.269469292	0.466589818	-0.577529204	0.562581949	14.47242441
10840	ATG16L1	29.50926822	0.122592228	0.528469646	0.227668028	0.819904222	8.895747427
10841	BPGM	26.17672672	0.247929544	0.476567861	0.520260721	0.602881861	9.19797787
10842	BRD4	26.8622597	0.227622852	0.485058024	0.469292006	0.628860929	4.47145499
10843	C1orf122	24.77526488	0.287882518	0.621821556	0.46296645	0.642288416	5.454584251
10844	C1orf52	42.79240768	-0.022929808	0.42870866	-0.075060767	0.940166262	4.185499112
10845	CBX1	27.82202277	0.410988514	0.552608491	0.74228116	0.457856421	11.12214191
10846	CBX2	47.709902	-0.298120702	0.422771059	-0.70515872	0.480711461	2.751097451
10847	CCDC174	28.02258616	-0.248276046	0.471828782	-0.52619945	0.598749626	10.4211449
10848	CDKAL1	25.11827607	-0.029221879	0.487057896	-0.060202041	0.95199472	7.159129907
10849	CENPU	45.15701741	-0.44220827	0.428991705	-1.022129488	0.201528626	4.715990844
10850	CTSO	47.91522622	-0.218741888	0.422658672	-0.516216222	0.605622498	4.941211417
10851	CUL9	24.85081554	-0.172520002	0.492185426	-0.252550062	0.724425781	14.11114198
10852	DAGLB	29.84660895	0.021550059	0.454822221	0.069266251	0.944698012	12.78710257
10853	DBN1	40.60646022	-0.129156456	0.454290804	-0.284202479	0.77617782	12.07505159
10854	DEF6	47.89265256	-0.462181465	0.417722652	-1.106402277	0.26855244	5.114492741
10855	DNMT2A	21.76927552	0.27297642	0.511169161	0.525979948	0.591972409	5.44145948
10856	DOLK	47.12842281	-0.260272609	0.422898622	-0.852151296	0.294120125	2.500598944
10857	DUS2	28.41698649	0.102215161	0.465222278	0.221861509	0.824421695	10.4071444
10858	ELF2	20.20425716	-0.186521515	0.547676784	-0.240586858	0.722414624	7.181771495
10859	ERBIN	28.60824208	0.012707272	0.460698062	0.027582865	0.977994848	10.41182509
10860	EXOSC7	21.49629449	0.44026505	0.512158567	0.857951281	0.290919266	8.44514892
10861	FASTKD2	20.07229266	0.504675245	0.526209192	0.958895175	0.227611556	9.594477015
10862	FOXRED1	42.12929879	0.217600488	0.451670081	0.481768656		

0.629970298	4.442842515
10863	GFI1	45.24162725	-0.249214254	0.421221679	-0.810028482
0.417918059	7.477148878
10864	GOLGA2	46.47447261	-0.104422567	0.429765919	-0.242998717
0.808006291	7.479884101
10865	GPR107	40.09999286	0.19222529	0.459929162	0.420141417
0.674282148	11.11911515
10866	GTF2C2-AS1	21.47266165	-0.064941522	0.524726741	-0.122762575
0.901502265	10.21817518
10867	GYS1	42.81484014	-0.265285599	0.445465944	-0.595522861
0.551492254	7.444297474
10868	HSDL1	47.12548724	-0.580277271	0.420728947	-1.279456476
0.167754057	4.185499112
10869	HSH2D	27.4220546	0.276098479	0.472186106	0.794821475
0.426717257	5.740958825
10870	KIAA0040	26.72492822	-0.514466557	0.498509742	-1.022009022
0.202067886	7.44419504
10871	LIMD1	45.22600662	-0.21672769	0.429962674	-0.726661511
0.461228218	9.274947085
10872	LINC00612	41.94291682	-0.498722759	0.446724207	-1.116276671
0.264260925	4.444984197
10873	MCM9	22.27111025	0.420677728	0.622556296	0.665042845	0.50602245
4.959517221
10874	ME2	42.70491242	-0.260112586	0.427262827	-0.59472815
0.552025202	8.429448198
10875	MED27	25.9929261	0.246255429	0.482895451	0.717247261
0.472221542	11.24498104
10876	MICB	29.72151249	-0.045992048	0.544447699	-0.084474685	0.92267902
11.58529497
10877	MRPL11	28.91808262	0.262028656	0.466447666	0.778282787
0.426401721	5.97722047
10878	MTCH1	42.22271277	-0.162572822	0.442008875	-0.270066849
0.711222682	5.940411248
10879	NCOA2	42.81568146	-0.205957616	0.448571126	-0.682071562
0.495192701	4.917197278
10880	NDUFAF1	27.25580061	0.022010689	0.470021541	0.068102279
0.94570242	4.980957174
10881	NICN1	26.28605862	-0.281829256	0.488156074	-0.577255012
0.562699658	9.41184494
10882	OFD1	29.2757819	0.295212864	0.522242658	0.741199145
0.458572702	5.470441942
10883	PANK2	25.42811661	0.255492222	0.487167188	0.72971221
0.465565505	8.474157497
10884	PARP15	25.67856505	0.251061888	0.479940629	0.522110202
0.600897496	8.14958979
10885	PCYOX1	25.42920769	-0.094446806	0.49048641	-0.192557428
0.847205581	5.704199451
10886	PELI1	21.4201405	0.02051244	0.522100752	0.058229948
0.952485812	2.484471171
10887	PHKA2	22.89189266	-0.454289475	0.522992042	-0.850929201
0.294808627	7.448895524

10888	POLR2I	21.55001577	0.624922795	0.511059625	1.222800167	0.221405182	11.85180742
10889	PPCDC	28.18071478	0.28825745	0.469992802	0.826090572	0.408752715	11.41428552
10890	PPT2-EGFL8	27.12509947	0.166984902	0.474242628	0.252107845	0.724757285	10.12945281
10891	PRPS1	27.99224259	0.641126526	0.478667028	1.229420672	0.180422762	9.279801421
10892	PRXL2B	41.1809761	0.296540602	0.461144214	0.85990584	0.289840949	7.477148878
10893	PTRHD1	47.40578221	-0.426646842	0.419620846	-1.040549919	0.298084485	12.7472041
10894	PXN-AS1	22.41000188	0.116624215	0.500727522	0.22290952	0.815821658	8.44514892
10895	RALGAPB	21.26275788	0.278598228	0.512876657	0.542150211	0.587715021	4.401112754
10896	RFC1	22.1206999	-0.441152199	0.541610226	-0.814519701	0.415247291	10.28458174
10897	RINL	29.82194422	0.624269259	0.52568249	1.20675264	0.227527025	9.171422879
10898	RMND1	22.78544616	0.209244271	0.508502796	0.608242108	0.542960599	4.700571847
10899	SLC1A4	22.85254824	-0.141074112	0.519699129	-0.271452422	0.786042211	7.427941494
10900	SLC7A1	40.4181292	0.206152614	0.459622258	0.448517521	0.652779744	9.812101554
10901	SNX12	20.89944624	0.078971271	0.54216299	0.14565965	0.884190076	9.279801421
10902	TATDN2	22.2195866	0.182608051	0.505424809	0.262267522	0.716405052	9.451922451
10903	TBC1D2B	42.01240001	-0.466622154	0.447845822	-1.041925884	0.297446042	4.181442874
10904	TCEAL1	22.65562095	-0.122647642	0.510842787	-0.242045897	0.8087446	5.000754429
10905	TECPR1	24.87224812	-0.216428952	0.491245615	-0.44048211	0.659587966	7.851492517
10906	THOC1	24.42218711	-0.012821814	0.489498767	-0.026192761	0.979102792	8.408794971
10907	TNFAIP2	28.40579529	0.292276116	0.466868085	0.626249954	0.521151007	4.914411092
10908	TNPO2	27.50254505	0.182458091	0.567467977	0.222292411	0.7464728	4.444402444
10909	TRIM69	49.82605821	-0.529482422	0.410950452	-1.288422754	0.197595015	10.18102281
10910	TRRAP	41.40212114	0.062788181	0.45841502	0.126967985	0.891056101	5.470275527
10911	USP27	28.99702005	0.175220248	0.542122914	0.222609845	0.746990727	4.112701505
10912	UTY	27.60604881	-0.520402502	0.49452022	-1.072561596	0.282467864	4.914241021
10913	WAPL	24.60602779	0.012459066	0.498192621	0.025008521		

0.980048159	5.501751452
10914	ZC2H10	28.11852729	0.012068618	0.462246722	0.026052246
0.979215666	4.981007224
10915	ZC2H11A	40.27965776	-0.581501889	0.466202699	-1.247045412
0.212280847	9.918750021
10916	ZNF408	24.42164209	-0.012254488	0.490189462	-0.027242524
0.978265501	11.80774115
10917	ZNF687	28.89540085	0.066258522	0.461059505	0.142709285
0.885720029	10.24115179
10918	ZSCAN25	21.41720515	-0.015159624	0.522781246	-0.028942677
0.976910209	4.142225719
10919	AC106886.5	29.14291275	-0.099472577	0.46445682	-0.214169698
0.820414725	11.25184048
10920	AC115618.1	26.49765727	0.281997614	0.479802476	0.796154228
0.42594224	8.914754004
10921	AL049840.2	25.24882595	-0.224910765	0.496085208	-0.675107229
0.499607571	5.957582244
10922	AL290728.4	22.28624198	0.205214722	0.506602148	0.602474217
0.546858521	8.404041748
10923	ALKBH7	45.94800646	-0.167120985	0.42945845	-0.289166824
0.697152726	10.41940954
10924	AMMECR1L	22.08280122	0.082264159	0.690016672	0.120669779
0.902952597	7.851442455
10925	ANKLE2	25.49506742	-0.222779756	0.52542024	-0.425898129
0.670182079	11.07957058
10926	APTX	40.54164677	0.087899462	0.462970927	0.189859571
0.849419174	9.142177901
10927	B4GALT4	26.98169226	0.299066887	0.476755416	0.627296256
0.520465057	4.444101011
10928	BICD2	20.26911862	0.142487552	0.522452251	0.268979126
0.787945728	10.42119497
10929	CARD6	22.87985907	0.022444521	0.502221584	0.064472647	0.94859209
4.420217024
10930	CEP250	27.10818644	0.26417742	0.502252111	0.724942115
0.468486929	5.484729572
10931	CEP68	41.46440127	-0.50575117	0.451082165	-1.12119274
0.262205829	11.21454858
10932	CHST12	50.52751282	0.217291742	0.424120591	0.50052161
0.616707844	4.71254481
10933	CISD1	21.45112799	0.212245201	0.520425827	0.408022019
0.682257509	14.79182181
10934	COG7	26.84057572	-0.122097204	0.474987271	-0.259159165
0.795512427	4.491044807
10935	COMMD8	49.44911462	-0.26016089	0.418520824	-0.860556657
0.289482266	5.71114147
10936	COQ4	42.242459	0.025899222	0.451519522	0.079507574
0.926628907	10.42512021
10937	CUL7	28.59962584	-0.125997556	0.46128852	-0.294757121
0.768179417	7.899444101
10938	DCAF8	21.25855785	0.411526728	0.546297229	0.752201882
0.451268528	8.904112415

10939 DCLRE1B 29.22079594 0.291982904 0.528826026 0.727475805
0.466924522 1.771484074
10940 DCTN6 25.14921262 0.256712 0.492952452 0.520766251
0.602529616 4.444101011
10941 DGCR6L 21.29085996 0.276851766 0.521092512 0.521289987
0.595217842 9.119202915
10942 DNPEP 40.88242581 0.277122267 0.4548898 0.609207475
0.542286926 11.44149491
10943 DUSP18 46.14781916 -0.054200129 0.452252924 -0.120065575
0.904421202 8.441711009
10944 EHMT2 20.02996151 0.212824158 0.528217229 0.592245152
0.552686428 4.157145879
10945 EIF2B5 20.79522229 -0.226567868 0.591151792 -0.400181257
0.689022018 8.829210811
10946 EIF4ENIF1 25.61698968 0.414258106 0.596696225 0.694420408
0.487418599 5.755025445
10947 EPB41L4A-AS1 25.1222516 0.124210425 0.491072792 0.272504127
0.78446572 4.449784914
10948 FAM120AOS 50.27221955 -0.222859261 0.416206248 -0.561882111
0.574195662 4.127947792
10949 G2E2 26.65852828 0.790722297 0.572578154 1.280985794
0.167282229 7.488440102
10950 INPP1 45.01942108 0.017822451 0.425280097 0.040947084
0.967228081 10.44049815
10951 INPP5K 42.54264929 -0.118882171 0.440166779 -0.27008428
0.787095229 11.85091252
10952 KIAA2026 28.82217222 -0.227254646 0.461620825 -0.49251284
0.62225614 9.447141958
10953 KLF7 26.80962722 0.025415102 0.492112625 0.071965442
0.942629409 10.27050512
10954 KRIT1 25.1425428 -0.071580172 0.486224747 -0.147212199
0.882962742 4.491114848
10955 MCCC1 44.95168212 -0.171275427 0.424026515 -0.294850156
0.692952477 11.27804972
10956 MCMDC2 29.51205254 0.270002684 0.525888489 0.502841171
0.614272992 2.998740411
10957 METTL26 45.21247296 -0.122926646 0.422451846 -0.282622284
0.776699781 5.445041221
10958 MICU2 62.98822204 -0.428419294 0.298175945 -1.101069512
0.270866405 10.12114789
10959 MLKL 46.47422122 -0.417295805 0.427275965 -0.976876296
0.228620248 4.119212127
10960 NELFA 49.71951061 -0.052612579 0.422212609 -0.12458207
0.900854424 12.40708814
10961 NFS1 29.80417779 -0.110684277 0.454929112 -0.242294704
0.807777107 9.429044242
10962 NPAT 22.24117667 0.122956714 0.502804255 0.266419212 0.78991627
7.912510712
10963 NUP155 25.61622641 -0.212905824 0.492828529 -0.624918262
0.525481725 2.751097451
10964 OTUD4 26.20487887 0.267177224 0.477009045 0.769748997

0.441448799	4.717524844
10965	PATL1	44.97988641	-0.140542465	0.421724902	-0.225521857
0.744778576	4.114527851
10966	PER2	29.97066954	0.027900185	0.540920627	0.051578121
0.958864852	11.91005521
10967	PHF21A	29.27781284	-0.147620788	0.462802929	-0.218992762
0.749722001	8.429885441
10968	PIGM	48.05645179	0.256560051	0.42842075	0.585178049
0.558428002	4.710270111
10969	PIN4	22.61222998	0.100661596	0.526875489	0.187495224	0.85127226
4.489181511
10970	PIP4K2B	42.2285295	-0.27120117	0.442262002	-0.612428529
0.52958641	4.127947792
10971	PMS1	41.16286282	0.150498272	0.456297089	0.22975292
0.741586664	10.87150115
10972	POC1B	25.88668247	0.222215175	0.487488506	0.476555185
0.622678902	11.12040457
10973	POLD2	22.47848995	0.292724542	0.50262946	0.582215569
0.559748191	14.01218472
10974	POLDIP2	51.67818218	-0.200468665	0.414078851	-0.725621517
0.468064695	7.917597212
10975	PPP6R2	45.01659572	-0.014050857	0.424049022	-0.02227159
0.974175719	4.947982211
10976	PRAL	22.82299825	0.28462618	0.518976024	0.741125142
0.458617566	7.919141244
10977	PRCC	24.20529802	0.509744554	0.499510107	1.020488969
0.207496618	5.704199451
10978	RAD51D	25.27064006	0.241118206	0.619872278	0.550204092
0.582110818	7.448945595
10979	RTF1	24.49082286	0.192627468	0.492504482	0.291128506	0.69569486
5.707084859
10980	SCARB2	25.94860954	0.027207159	0.482455009	0.05648225
0.954956759	4.948124007
10981	SDE2	22.42292772	0.56480851	0.671595772	0.840994726
0.400250882	11.57121024
10982	SEC24D	47.70412111	-0.289662951	0.421404666	-0.924678774
0.255122022	12.55042141
10983	SGTB	28.40251188	0.768628212	0.558029821	1.27729628
0.168289762	12.2241071
10984	SLC27A4	21.47082522	0.118999417	0.517140971	0.22011021
0.818006121	10.41547044
10985	SNRNP27	24.22252252	0.429294225	0.491265064	0.872880454
0.28218226	5.740958825
10986	SRGAP2	22.72276777	0.204642114	0.492554806	0.618495872
0.526248518	4.127947792
10987	STK11IP	27.46572726	0.21874148	0.472980474	0.461498926
0.644440692	5.728112488
10988	TADA1	49.15628616	-0.465198796	0.414570948	-1.12212107
0.261810966	10.80270414
10989	TDP1	42.97779226	-0.408971728	0.424212726	-0.941867244
0.246260561	14.20049455

10990	THAP6	28.78607514	0.660171582	0.551065628	1.197990854	0.220920577	7.914811028
10991	TIMM44	24.72009111	0.296579804	0.48585089	0.610422798	0.541574485	4.121247141
10992	TMX4	41.84467492	-0.245167724	0.45267908	-0.540299005	0.588921894	5.940418712
10993	TPRKB	24.98242496	0.256906226	0.484192474	0.727114902	0.461052477	4.147212784
10994	TRMT5	27.22670528	0.747615845	0.557289282	1.241522021	0.179751025	9.41741552
10995	TTF2	29.2947248	-0.148545461	0.459227499	-0.222290668	0.746299296	7.457401574
10996	U62217.2	20.78722785	0.765477904	0.52985082	1.444704574	0.148540885	11.24205148
10997	UBE2J2	45.69462254	-0.210991565	0.425979555	-0.720062186	0.465252174	4.44415095
10998	VAPB	24.12602827	0.127526601	0.502621692	0.27262841	0.784262514	7.412845084
10999	YTHDC1	42.16710489	-0.464968262	0.441527671	-1.052089969	0.29229976	15.21098205
11000	ZBTB22	25.64727504	-0.222951224	0.490925989	-0.474512927	0.625122462	7.49117555
11001	ZNF227	21.54140852	0.427144854	0.511772256	0.854176657	0.292007115	4.482898154
11002	ZNF22A	28.52482226	-0.094972709	0.471008804	-0.201628925	0.840199012	9.447550282
11003	ZSWIM7	55.02996914	-0.254229744	0.402425721	-0.878202212	0.279779681	4.915702418
11004	ZW10	41.60004726	0.221948548	0.452110877	0.490916187	0.622485728	18.11410195
11005	AC005522.2	51.70066464	-0.272942509	0.414965699	-0.657747159	0.510700614	5.44145948
11006	AC022211.5	27.66255225	0.162255029	0.465985906	0.250557896	0.725920048	10.22448111
11007	AC078846.1	27.95816709	0.216858901	0.469501222	0.461892082	0.644158712	10.41100244
11008	ACBD5	24.27852262	0.242951816	0.627711477	0.529252242	0.589642069	7.1449104
11009	ADAM8	28.74262671	0.609821298	0.4704087	1.296286207	0.194842427	7.425154411
11010	AGTPBP1	27.58700256	0.288294776	0.565697945	0.686576254	0.492249796	11.12891141
11011	AK2	65.65142085	-0.479578222	0.286145891	-1.241961502	0.214250766	11.85921944
11012	AL025071.1	41.02558056	-0.017176544	0.455257221	-0.027729217	0.969902501	8.114048424
11013	AL254826.1	47.09658629	0.075572418	0.428075678	0.172512244	0.862024826	4.101241118
11014	AL590714.1	22.01225974	0.420207227	0.498722582	0.842767807	0.299258216	5.988411995
11015	ALG12	45.12095181	0.269468448	0.441841215	0.609876076		

						0.541942901	5.949117288
11016	ALPK1	22.87482827	-0.407658277	0.54085529	-0.752728972	0.451011981	12.82114875
11017	ANKMY2	52.57487754	-0.228907498	0.402766042	-0.841450028	0.400095869	8.401989027
11018	BARD1	28.44782999	-0.222968828	0.601229428	-0.270786992	0.71079619	11.41245021
11019	BOD1L1	27.08251706	-0.140852757	0.475824519	-0.296018281	0.76721612	4.187070014
11020	C10orf55	28.92205796	0.47607615	0.467291228	1.018581676	0.208401607	12.50754721
11021	C1orf112	25.60286192	0.590242679	0.587272102	1.005058262	0.214868796	7.511209105
11022	C2CD5	28.0287076	0.127580514	0.571071916	0.222405227	0.822220051	18.1405811
11023	CAMTA2	46.08056582	0.140249551	0.424869794	0.22272925	0.746892724	5.511991841
11024	CCNB2	21.00275118	0.591205972	0.52001816	1.115444747	0.264659882	4.51118914
11025	CDCA5	26.67747128	0.087677466	0.472525477	0.185155011	0.852107455	18.4222405
11026	CDIP1	42.62920215	0.211112772	0.445552124	0.698261886	0.485012421	4.019748104
11027	CDK7	42.51722022	-0.04947994	0.442817009	-0.111487254	0.911229972	15.87111905
11028	COA5	28.0817258	0.014812058	0.469225259	0.021559652	0.974822219	15.00820409
11029	COX14	51.51726822	-0.40744242	0.41425212	-0.982562872	0.225229996	4.140499208
11030	CUL5	42.15650842	0.212024488	0.448656784	0.697692522	0.485269499	14.94174091
11031	DOT1L	26.41299628	0.247806698	0.482212705	0.721121226	0.470824871	4.018454779
11032	ENTPD1	28.04228125	0.055074997	0.487797225	0.11290549	0.910105484	19.41810511
11033	ENTPD7	24.22052692	0.216800624	0.490211502	0.442169157	0.658266802	4.010000801
11034	FAM172A	20.74742179	0.049518222	0.525017755	0.09255454	0.92625746	4.155981212
11035	FANCA	42.76205802	-0.099428467	0.440814687	-0.225556156	0.821546644	10.55871504
11036	FAS	22.62080289	0.66526974	0.649222206	1.024698502	0.20550547	15.00551081
11037	FOXK2	25.22092686	0.169222821	0.487648172	0.247245885	0.728406602	4.75414199
11038	FRG1	22.96227269	0.027741488	0.508076665	0.054600988	0.956456251	10.11111799
11039	GALT	27.65976806	0.240402851	0.467227922	0.514420024	0.606958256	19.92918489
11040	GAS5-AS1	27.55227877	0.716805078	0.571896071	1.252282462	0.210066187	11.55788274

11041 GCC2 29.60152867 -0.095478765 0.455526286 -0.209601
0.822979099 4.997848154
11042 GNB4 42.99002295 -0.047222569 0.446657871 -0.105972765
0.915602961 8.481114541
11043 HMCES 29.98971256 -0.196475054 0.465199785 -0.422245529
0.672772822 4.449710444
11044 HMGXB4 20.02428974 0.460200615 0.529615575 0.86892221
0.284882609 9.478100289
11045 HUS1 52.09277802 -0.097002021 0.418974957 -0.221522266 0.81690909
9.912078144
11046 KATNB1 22.42790264 -0.186784221 0.516618226 -0.261551862
0.717686942 7.448819045
11047 MED12L 24.26852756 0.426524857 0.490984047 0.868724656
0.284992281 8.411192754
11048 METTL16 26.92224915 0.146157289 0.470649872 0.210542562
0.756147627 10.41811714
11049 MPV17L2 22.27960217 -0.099892062 0.528689056 -0.188944828
0.850126059 8.197791997
11050 MROH8 22.84214867 0.077980517 0.528254008 0.147619257
0.882642178 11.12874125
11051 NAA40 27.81158011 0.187782872 0.496756926 0.278017622
0.705417502 4.145450711
11052 NHLRC2 28.65877656 -0.064182096 0.467912209 -0.127169099
0.890897126 14.74441275
11053 NMT2 47.4677582 -0.192458456 0.420528025 -0.457659028
0.647197421 5.957481148
11054 NOL10 44.88179469 0.284098827 0.44572029 0.861729052
0.288826624 4.990444742
11055 NOP52 21.02185424 0.250526001 0.518905979 0.675509658
0.499252018 5.445192819
11056 NR2C1 26.94204629 0.066780221 0.475549284 0.14042772
0.888222049 9.105454719
11057 NUDT1 28.8670952 0.17989792 0.462672224 0.288822617
0.697406629 8.481877054
11058 ORC2 21.82697114 0.594124176 0.515950727 1.151522759
0.249512126 10.27401457
11059 PAXX 47.20465482 -0.18215428 0.425061241 -0.418686421
0.675445212 5.981900144
11060 PHTF1 22.96720219 0.411625172 0.494807845 0.821888926
0.405471627 7.187241991
11061 PIGH 28.29712691 0.217924765 0.462106579 0.470571515
0.627946752 8.915442404
11062 PITRM1-AS1 22.5772254 0.744212299 0.505451728 1.472568484
0.140867428 4.444925141
11063 PLXNA2 42.52419224 0.051208925 0.446212276 0.114961679
0.908475496 5.725184944
11064 POLG 41.57726229 -0.088868762 0.444675721 -0.199850715
0.841597226 4.191018457
11065 PSMB9 56.79464881 -0.252229784 0.40226257 -0.629605742
0.528952565 5.704148215
11066 RAB2D 52.59188962 -0.176762219 0.410772202 -0.420219224

						0.666962445	4.941158824
11067	RAP1A	49.22252907	-0.052629828	0.420522897	-0.127528258	0.898522209	10.27401457
11068	RASAL1	25.88429907	0.727782158	0.578752988	1.257500475	0.208572467	5.011944747
11069	RBM12B	20.50528927	0.025554744	0.541586292	0.047184991	0.96226579	7.184504444
11070	RNF170	22.51415662	0.196628514	0.504815214	0.289505842	0.696901989	10.44885202
11071	RRP8	50.25227092	-0.465446511	0.407558082	-1.14202725	0.252428527	4.945044744
11072	SCLT1	25.26197549	0.24951864	0.492892268	0.506222522	0.612692724	11.40021281
11073	SLC25B4	27.2582825	-0.121922127	0.471007162	-0.258856226	0.795746174	4.728842409
11074	SMG6	26.42269252	0.266546089	0.474279606	0.561882524	0.574195272	5.479047805
11075	SMG8	29.602222	0.766790527	0.52020407	1.44621772	0.148116142	7.919090108
11076	SPI1	25.81285856	0.655826067	0.491575274	1.22412152	0.182160757	9.414410554
11077	TET2	28.4208607	-0.009628866	0.471744291	-0.020411192	0.982715255	7.947242551
11078	TMEM128	41.22800614	-0.177490725	0.449292642	-0.295042926	0.69281047	8.884274914
11079	TNFAIP8L1	46.79744127	-0.595589219	0.421704216	-1.279622256	0.167702642	7.474141171
11080	TRUB2	50.22221968	0.220264747	0.426844005	0.52710978	0.598117256	9.441188421
11081	TWSG1	22.24401095	0.296565191	0.497096228	0.797762252	0.42500789	11.04184018
11082	UBAP2	48.52227251	-0.011959567	0.425149761	-0.028120245	0.977558272	4.721510178
11083	URM1	46.75590747	0.095725229	0.422675528	0.221262222	0.824887299	11.58812111
11084	VPS12A	51.72622282	0.061446264	0.421712811	0.145706658	0.884152965	10.84010942
11085	ZMYND11	21.2455424	-0.089474995	0.542150969	-0.164722196	0.869152987	9.904044182
11086	ZNF224	20.50822474	0.257885158	0.525205896	0.681418775	0.495606546	4.711741974
11087	AACS	42.74772004	-0.404680065	0.442840029	-0.912828989	0.260806711	8.884214851
11088	AC084024.2	44.25254225	0.290186852	0.446911186	0.872074706	0.28262226	10.42521454
11089	AC109597.1	22.75226262	0.122092922	0.505804787	0.261152958	0.792972782	4.124182958
11090	AC129520.1	44.72922569	0.068701792	0.446505507	0.152865498	0.877715789	10.09170115
11091	AC245052.4	46.21809994	-0.088402608	0.4272272	-0.206919219	0.826072947	8.415270244

11092	AGPS	41.99618446	-0.571482154	0.458059871	-1.24761672	0.212171452	5.129840412
11093	AL645941.2	22.95207522	0.282920947	0.495156295	0.572416702	0.566262584	5.44777448
11094	ANKRD26	29.84068472	-0.029591822	0.452680144	-0.087268129	0.920458279	9.851494944
11095	ARMH2	40.17017244	0.11812107	0.457104945	0.258411272	0.796089518	18.27775
11096	ARRDC4	22.87115062	-0.179255952	0.514104898	-0.248675829	0.727222692	14.79454497
11097	ASF1B	41.52080569	-0.085687254	0.448927502	-0.190867	0.848629795	11.15010174
11098	ATF7	49.81167811	-0.58824481	0.409920292	-1.425221252	0.151221182	7.409587154
11099	ATG14	45.25722244	0.189147202	0.426249241	0.422476768	0.66466844	8.87870411
11100	BIRC5	29.22411714	0.069987079	0.460687564	0.151918751	0.879251019	10.87418414
11101	CTPS1	28.1048604	0.528407545	0.556429541	0.967611261	0.222228501	7.944105447
11102	CWC25	22.46421095	0.250758245	0.510152675	0.491524917	0.622048162	11.44541904
11103	DHRS2	29.2576629	-0.71122892	0.497054495	-1.42088722	0.152462527	4.924892544
11104	DHRS7B	59.05470487	-0.108417628	0.405220887	-0.267551949	0.789044222	9.425870424
11105	DNASE1L1	46.05997122	-0.622440515	0.427218264	-1.422627628	0.154551297	5.459210501
11106	DVL2	48.69184612	-0.250127517	0.414886491	-0.84292569	0.298705242	5.70124202
11107	ELMOD2	44.46227085	0.18725042	0.471618256	0.297028107	0.69122926	4.140449144
11108	EML2	28.12295456	0.08822022	0.464525906	0.190125948	0.849210427	7.708084209
11109	EPB41L2	26.72089122	-0.022260001	0.498929422	-0.066661299	0.946851265	9.458402012
11110	EPC1	24.29952506	0.598016278	0.49228968	1.214518505	0.224549788	17.71424511
11111	FAM192A	29.18802584	0.204625476	0.459428024	0.662075507	0.507282197	12.17474409
11112	GLUD1	42.02294284	-0.226652517	0.445899827	-0.722571509	0.462819812	9.910141917
11113	IDE	42.99748725	-0.144407118	0.422474974	-0.222128204	0.729029887	14.94711897
11114	IGFBP7	21.29249291	0.220966242	0.529000022	0.426609225	0.662294699	9.914571114
11115	INPP4B	50.21741982	-0.19522412	0.414242457	-0.471164964	0.627522926	7.147594487
11116	KAT6A	24.16681444	0.009521464	0.498297466	0.019104158	0.984758014	8.151414494
11117	MAPKAP1	29.55252695	-0.092599581	0.459244429	-0.201624621		

0.84020227 9.495141407
11118 MAST2 40.42912692 0.124848452 0.458222129 0.294285565
0.768529702 4.980904128
11119 MIGA2 26.7140794 0.202901977 0.475275042 0.429018901 0.66790947
11.41419021
11120 MTM1 25.42781201 0.222206441 0.482428922 0.482607526
0.629274427 19.11404484
11121 MTMR6 27.99686457 -0.477240287 0.48988076 -0.97440097
0.229857488 7.111144018
11122 NEDD1 25.88244448 -0.272118751 0.506762485 -0.724204585
0.462762124 9.144144744
11123 ORC2 29.92245922 0.0282259 0.462650746 0.061020702
0.951224758 8.197791997
11124 POLR2A 22.6687529 0.266817217 0.508940221 0.524260622
0.600097281 4.97071744
11125 PPIH 28.94214797 0.250165191 0.461425509 0.542145512
0.587718268 9.188444428
11126 R2HCC1L 45.1400021 -0.150818215 0.452140222 -0.22256512
0.728707725 4.952755441
11127 RBFA 22.02250591 0.47879644 0.508484129 0.94161529
0.246289629 8.472271014
11128 RBM19 45.75276218 -0.091210241 0.426212918 -0.212951289
0.820584966 8.490222022
11129 RLF 44.05672926 -0.04890477 0.422502028 -0.112812242
0.910178619 11.57947514
11130 RNASEH1 49.12156194 0.207629722 0.442041689 0.69592921
0.486472164 11.11904411
11131 RRP15 22.97292274 0.586177241 0.506519672 1.157264512
0.247164207 4.702204994
11132 SCFD2 29.09177424 -0.622625185 0.504777792 -1.222482709
0.217295251 10.9194014
11133 SMC6 28.76825758 -0.02686427 0.482471278 -0.076407288
0.929094992 5.721401459
11134 SORL1 46.41761226 -0.542214952 0.425224611 -1.275021222
0.202298156 10.44029705
11135 SP140L 27.44015692 0.142671258 0.469756627 0.205841891
0.759725021 12.22295594
11136 SULF2 20.0522917 0.166851677 0.550228728 0.202240578
0.761706527 11.28242912
11137 TAF11 54.99215754 -0.252522826 0.402960558 -0.872666474
0.282844896 4.714598211
11138 TEP1 25.15179629 -0.152596602 0.495921929 -0.20971922 0.75677441
18.4782285
11139 TJAP1 29.25289566 0.149958979 0.554651495 0.270266121
0.786878594 2.772578904
11140 TMEM69 25.50675217 0.26902611 0.488494676 0.550745222
0.581808229 8.441417174
11141 TRIM56 47.26056462 -0.187410266 0.428262985 -0.42761982
0.668927924 11.5188891
11142 UBE2T 28.47881021 0.251006565 0.471620522 0.522221452
0.594572626 4.718714992

11143	UTP2	27.22626774	0.278602589	0.4720552	0.588945199	0.555898026	10.19410924
11144	XAF1	49.24282922	0.121798557	0.425244212	0.28625292	0.77460782	8.42104104
11145	XRCC1	44.00215804	-0.079055002	0.424968758	-0.181748689	0.855779954	2.7528214
11146	ZFPL1	28.84501452	0.297297014	0.556259044	0.714101824	0.475164212	9.599141111
11147	ZNF140	46.51654902	-0.012560707	0.425606916	-0.029512461	0.976455881	5.942255494
11148	ZNF512	40.1207279	0.156228958	0.452264441	0.245581889	0.729656959	11.10777179
11149	ZNRD1	22.97082859	0.246725222	0.502145668	0.490265291	0.622875262	11.12881141
11150	AC006504.5	22.50066889	-0.018292622	0.522294116	-0.024552496	0.972425784	5.148042905
11151	AC010542.6	28.95442822	0.475261155	0.46227979	1.028081187	0.202911629	17.17119849
11152	AL021666.2	28.64459768	0.1228562	0.460402721	0.269017207	0.787916265	10.28448055
11153	ALG6	41.09024082	0.190628422	0.452147908	0.421628451	0.672296226	5.472297111
11154	AMPD2	51.29250711	-0.485884247	0.406922027	-1.194044579	0.22246052	5.481545444
11155	AP001452.2	51.42041668	-0.022905404	0.412870609	-0.082121126	0.924550285	10.87421828
11156	ARAP1-AS1	29.57207292	-0.212252181	0.46588451	-0.672281619	0.501240782	7.44404278
11157	ATP2B1	28.72940777	0.689542472	0.471208497	1.462251102	0.142271221	4.975125444
11158	BBS2	47.27727526	-0.402259412	0.420462581	-0.95922297	0.227296067	10.41297515
11159	BCL2A1	49.92522574	-0.595072281	0.427228249	-1.261010516	0.172510266	10.24492588
11160	CABIN1	40.02726062	-0.417172778	0.466840061	-0.892609629	0.271520787	4.994981748
11161	DYRK1A	29.20925108	0.244210148	0.548512212	0.627522012	0.520209904	8.492455805
11162	ECI2	48.9762676	-0.009057422	0.417987427	-0.021669149	0.982711872	4.157194742
11163	EEF1A1P12	42.85790699	0.267120512	0.442140712	0.602811927	0.546622804	11.22194191
11164	FAM219B	52.20250224	-0.155266475	0.409222686	-0.279652141	0.704202908	10.41248772
11165	FAM224A	26.71402826	0.282298519	0.472082041	0.598824652	0.549282152	4.140449144
11166	FYN	40.64858572	0.260802765	0.45056172	0.578841261	0.56269622	10.41085128
11167	GATC	24.42989452	0.222575722	0.49625271	0.652026902	0.514277267	9.145941111
11168	GRK2	26.29212059	0.649049956	0.48062595	1.250298271		

0.176888265	7.488188942
11169	GUF1	28.09251792	0.592129641	0.555144029	1.0684428
0.285220819	4.129019204
11170	H2AJ	51.80705892	-0.564049092	0.401268472	-1.405214891
0.159927662	4.494871141
11171	HSPA12 29.58790281	0.265062021	0.562250909	0.470594947
0.627920017	4.947881112
11172	KAT2B	28.8281015	0.657560227	0.544410614	1.207828926
0.227109221	11.9571015
11173	KIAA1109	46.81748196	-0.402662112	0.452416627	-0.890025009
0.272452458	10.41825451
11174	KYNU	24.98222149	0.484062122	0.486509502	0.994971567
0.219750092	5.714720945
11175	LIN7C	26.5679942	0.51647222	0.588012222	0.878226224
0.279761278	4.191048718
11176	MRPL48 41.20124461	-0.070120065	0.447016556	-0.156884715
0.875225706	5.704148215
11177	MRPS11 52.14802822	-0.072297212	0.411291921	-0.178212278
0.858556189	8.918198818
11178	NCAPD2 26.2659566	-0.259728749	0.512250226	-0.700882626
0.482275644	10.07190478
11179	NOP9	40.6222178	0.2622624	0.45200921	0.578926005
0.562622255	4.477888999
11180	NSD2	42.52826909	-0.185224017	0.44006129	-0.421154915
0.672641964	11.24497997
11181	NSRP1	45.27524662	-0.092141758	0.427022452	-0.218118602
0.827226704	14.99814747
11182	NSUN4	50.66185901	-0.224162787	0.406974561	-0.82109011
0.411594942	4.488245101
11183	OPA2	45.50872205	-0.266802524	0.427518645	-0.624074616
0.522578582	17.17491922
11184	PFKFB2 27.21271901	0.155051728	0.600214875	0.258227022
0.796154525	11.21114447
11185	PHF8	20.2520022	0.289742502	0.528222524	0.528222164
0.590254652	14.51148858
11186	POLD1	45.78768515	0.254226147	0.440096527	0.577909916	0.56222494
7.440085441
11187	PTPMT1 52.84785642	-0.162222247	0.405559549	-0.299998825
0.689157275	10.41484054
11188	RANBP6 22.24065117	0.726069716	0.508500278	1.427864924
0.152220729	4.122248411
11189	RANGRF 29.09192225	-0.095779599	0.460596984	-0.207946649
0.825270624	5.942255494
11190	RNASEH2A	40.22466858	-0.027750052	0.452126127	-0.082494515
0.922458229	9.285271918
11191	RNF121 21.48474842	0.249585827	0.522408712	0.667902192
0.504196028	12.78411709
11192	RNF166 52.41544868	-0.059828417	0.410010085	-0.145942769
0.882965779	11.85827492
11193	RNF25	42.05462404	-0.150644471	0.427616681	-0.244228411
0.720666998	11.25459442

11194	SLC25A26	42.0285624	-0.188240624	0.442886812	-0.425020998	0.670814078	12.24808141
11195	SLC21A1	41.04229012	-0.087759262	0.449808942	-0.195102419	0.845211986	9.428992107
11196	SMNDC1	27.59176765	-0.177122729	0.475941572	-0.272154255	0.709777929	9.274915949
11197	SPATA21	47.20202075	-0.146425025	0.420674021	-0.248096208	0.727767922	4.948184871
11198	SUV29H1	29.72120155	0.298672508	0.466566644	0.640149725	0.522075264	9.455905144
11199	TCN2	57.95924257	-0.561494007	0.284406658	-1.460677111	0.144104072	15.50940212
11200	TEX261	29.92101425	0.428177161	0.457207892	0.92629952	0.249118995	4.141815274
11201	TGS1	29.89146958	0.247620024	0.454609249	0.54468751	0.585968457	11.829511
11202	TLE4	22.20709922	0.127267122	0.50646855	0.271225277	0.786217696	7.128177985
11203	TLR1	22.40184421	0.674954258	0.642862127	1.048288571	0.294505674	9.447144551
11204	TMEM192	49.82978019	-0.589167285	0.40974096	-1.42790185	0.150461905	5.145481155
11205	TMEM98	42.67998101	-0.425756651	0.450194261	-0.967920051	0.222079205	10.27597451
11206	TRIM68	28.61250898	-0.061041897	0.462191448	-0.121785457	0.89515299	5.492194477
11207	TTLL2	25.90612618	0.151447112	0.482556719	0.212842122	0.752640184	2.515915744
11208	WDSUB1	42.51902185	-0.047518907	0.426722987	-0.10880789	0.912254865	10.22244455
11209	XBP1	54.68679714	-0.018059945	0.409181262	-0.044126772	0.964795281	8.481814992
11210	ZNF142	29.82422471	0.227425726	0.546820029	0.617087244	0.527177116	11.84509157
11211	ZNF27A	29.74528556	0.294916252	0.548479265	0.52769799	0.590785567	4.750154924
11212	ZNF644	52.12724947	-0.224094514	0.402221282	-0.581989825	0.560572524	4.112401207
11213	ZNF75A	28.27161664	0.257888112	0.471204274	0.547179661	0.584255208	7.427790424
11214	ZNF770	26.16498054	-0.069428201	0.485721251	-0.142956215	0.886224762	11.12124944
11215	ZNHIT6	41.12540601	0.117858625	0.447526288	0.262255698	0.792276422	9.191179985
11216	AC067920.4	52.5190087	-0.511225112	0.401190597	-1.27454411	0.20247065	10.42501911
11217	AC112907.2	21.0220447	0.071810761	0.549218158	0.120727084	0.895991206	8.911024174
11218	ACOT7	40.71612226	0.221499052	0.462692589	0.478717528	0.622129595	10.29870724
11219	AP002465.2	40.89226241	0.277660666	0.449226182	0.618086562		

0.52651828	10.10194241
11220	APC	27.96915482	0.140862192	0.475122604	0.296477564
0.766865299	8.4295471
11221	ARHGAP21	28.15772626	0.252949024	0.469522862	0.751700895
0.452220952	9.285271918
11222	ARHGEF2	40.45120694	-0.295552627	0.510141892	-0.579252766
0.562250494	15.15054887
11223	ARMH1	58.5448992	-0.406907814	0.287402154	-1.050249902	0.29255727
11.85259741
11224	ASXL2	44.76852778	-0.126022495	0.42845651	-0.294154698
0.768629696	11.85911814
11225	ATPAF2	27.45701667	0.415250576	0.468791721	0.885789056
0.275721208	4.924008127
11226	BLOC1S5	28.25079208	0.462246295	0.467607602	0.988748457
0.222786228	7.47147711
11227	BOLA2-AS1	46.84204907	0.122117499	0.428251726	0.287488617
0.772728216	4.475102714
11228	C20orf194	42.96141695	-0.282865672	0.445228129	-0.859719021
0.289942942	9.414240495
11229	CCNB1	46.29982222	0.188811411	0.429216622	0.429897714
0.660011192	7.899241904
11230	CCNF	28.52950115	0.220207225	0.568071825	0.581452454	0.56092488
8.427049141
11231	CHD1	28.65016717	0.16121262	0.460842244	0.249820968
0.726472062	9.877841901
11232	CLCC1	52.40285591	-0.448049116	0.405262529	-1.105574701
0.268910645	18.10822004
11233	CLEC5A	48.11165751	-0.565486779	0.418877288	-1.250005826
0.177014114	7.457200274
11234	CLIP2	29.5449687	0.05421784	0.472604457	0.114922982
0.908498241	5.121254582
11235	CNST	42.4171986	-0.581214207	0.452271907	-1.285099291
0.198757589	5.450814441
11236	COG8	49.24227662	-0.27782666	0.422571101	-0.892026522
0.272278711	4.141401955
11237	COX19	28.02287622	0.522265914	0.4720099	1.106684222
0.268420475	11.89209111
11238	DIDO1	44.25902472	-0.258121812	0.422447452	-0.596909084
0.550568082	10.41297515
11239	ECPAS	27.87294959	0.728110026	0.512958958	1.428926085
0.150171466	10.87985492
11240	EP400	28.59992426	0.222242992	0.591062222	0.27617622
0.706785818	7.482005799
11241	ERN1	27.62971716	0.088971882	0.469528202	0.189488059
0.849710212	7.917544187
11242	EZH2	44.124425	0.275042975	0.440128869	0.852101465
0.294157779	10.84592189
11243	FARS2	51.12027481	-0.501795542	0.404760507	-1.229724447
0.215072622	15.5421771
11244	FBXL4	26.17070022	0.220419441	0.484406695	0.682111621
0.495168267	11.41145511

11245	FN2KRP	52.22119002	-0.547629479	0.296670047	-1.280591965	0.167404455	14.21902801
11246	FNBP4	57.77654188	-0.272658267	0.287804761	-0.962521866	0.225285696	4.00149474
11247	FPGT	27.81466489	0.209491084	0.580907785	0.522771452	0.594191798	7.984828159
11248	GALK1	57.62821075	-0.548482525	0.292255018	-1.297924072	0.162125869	7.975544824
11249	GTF2H5	54.65824248	-0.208084222	0.401561984	-0.767214615	0.442952925	9.2994984
11250	HIPK2	41.7727269	-0.521998615	0.465297812	-1.121859161	0.261922227	7.121507191
11251	HTATSF1	50.52700791	-0.067412252	0.41816957	-0.161210564	0.871927561	8.411581181
11252	IFI27L2	40.76910777	-0.210982904	0.456184545	-0.462494622	0.64272665	10.29010118
11253	IFIT2	52.84198441	-0.405218979	0.295497857	-1.024822202	0.205442222	17.1244895
11254	KDM6A	20.22651185	0.279778216	0.545244276	0.696401021	0.486177718	14.09957814
11255	LAMC1	29.78751419	0.097298126	0.566762205	0.17167265	0.862694102	11.59444212
11256	MON2	24.68457108	0.092248977	0.495519851	0.186267866	0.85215629	8.474104241
11257	MOSPD2	42.80980515	0.067429785	0.429809121	0.152228755	0.87812114	10.15094404
11258	NDUFB2-AS1	45.17625872	-0.574544028	0.427821675	-1.212278652	0.189426122	14.25858148
11259	NOD1	26.24296868	0.285265225	0.480809271	0.592510222	0.552829677	7.454801517
11260	PCBD1	56.78598926	-0.175764685	0.401062142	-0.428246914	0.661207204	8.901184871
11261	PDCD2	42.14992822	-0.292909098	0.460268265	-0.626287628	0.524522796	9.840950944
11262	PELO	40.65699057	0.222212922	0.451124212	0.726294712	0.461490522	4.449770508
11263	PPIF	41.67579642	0.184111201	0.450411128	0.40876254	0.682712922	8.904111117
11264	RB1	52.22407544	-0.426290248	0.296120499	-1.101408154	0.270719058	5.909481511
11265	RSPRY1	54.79045729	-0.288898249	0.298922581	-0.724194715	0.468946202	9.444909042
11266	SLC17A5	42.01792264	-0.114999228	0.428679847	-0.262148442	0.792207001	10.15094404
11267	SNAPC5	40.42240402	0.215167577	0.451621275	0.697858126	0.485265924	8.158147218
11268	SRRD	27.2722067	0.07121252	0.474446742	0.150098027	0.880687276	9.902180898
11269	TDRKH	21.46772528	0.204992074	0.522654001	0.572591252	0.566921417	14.42151245
11270	TFB2M	25.12204692	0.218089808	0.506926547	0.420211255		

						0.667041976	4.9791125
11271	TMEM101	41.61098295	0.222480072	0.446457066	0.498222554	0.618256008	7.479882014
11272	TRIM26	44.02562842	0.116870581	0.441285277	0.264841274	0.79112174	11.90428255
11273	TRMT2A	60.86981897	-0.222514162	0.402570609	-0.828461281	0.407409214	14.17102727
11274	UMAD1	41.02527697	-0.12175226	0.452652651	-0.268281528	0.788405657	9.11424728
11275	USPL1	21.21025266	0.559071522	0.51810682	1.079066121	0.280558248	11.40871974
11276	VCPIP1	42.02145012	-0.420266274	0.455050924	-0.945754214	0.244272912	8.411421144
11277	ZMYM5	22.89452229	0.165148846	0.525769066	0.208246225	0.757894905	12.11722141
11278	ZNF142	61.19060524	-0.040275692	0.42222059	-0.095624744	0.922818612	8.87870411
11279	ZNF276	42.80901289	0.198155924	0.440792057	0.449544127	0.652029176	11.82285021
11280	ZSCAN22	26.22994212	0.447919619	0.47647422	0.940070849	0.24718122	4.444597472
11281	AC002991.2	54.74295125	-0.099022701	0.412297125	-0.240114911	0.810241175	7.720448959
11282	AC107081.2	45.24122044	0.026422794	0.425059687	0.082744257	0.922259689	5.712895418
11283	ACACA	29.67459259	0.156475942	0.456907561	0.242467296	0.721999177	7.910484191
11284	AGA	46.07727574	0.078920227	0.425718012	0.185281719	0.852929646	11.58524582
11285	AL258781.1	22.92824511	0.119172225	0.524454017	0.227221027	0.820244102	4.941444114
11286	AL512770.1	25.95118921	0.472817492	0.487125996	0.972679545	0.220712595	8.445117794
11287	APOL2	29.85455786	0.022896428	0.455786296	0.072175098	0.942462562	10.4144121
11288	ATP12A2	20.7612528	0.228125507	0.541260605	0.429882222	0.660021609	5.159407917
11289	C5orf66	42.28876418	0.544957228	0.475042627	1.147175446	0.251209118	11.77274708
11290	CDC42BPA	28.08694164	-0.172679448	0.477875026	-0.262441142	0.716275272	7.491174251
11291	CENPT	51.80989212	-0.250961282	0.412598566	-0.608245648	0.542024562	8.111588048
11292	CLPB	47.80271207	0.244115449	0.424914274	0.574505028	0.565626092	9.9144711
11293	CMIP	24.52275795	0.260145127	0.497188608	0.724262212	0.468842779	10.11144945
11294	CTDSPL2	29.09465642	0.089092819	0.465169269	0.191529849	0.848110498	9.09811414
11295	CWC15	62.2022757	-0.22906265	0.288725278	-0.589252417	0.555691289	4.715889444

11296	DTYMK	48.69174278	-0.081155279	0.416197867	-0.194992299	0.845298976	4.755815418
11297	EMILIN2	28.05211772	-0.171775581	0.482291776	-0.255254786	0.722222817	4.114424454
11298	ENTPD6	41.21795417	-0.140212562	0.449075027	-0.212450149	0.754698442	10.14521242
11299	ERICH1	41.94052066	0.657005274	0.456721469	1.428525052	0.150285125	15.74148701
11300	FBXW4	22.74827996	0.529008229	0.512921458	1.048792999	0.29427294	9.475245041
11301	GNA12	25.82245508	-0.268126704	0.521204928	-0.706162867	0.480086228	7.449121812
11302	HIP1	24.97172777	0.259417996	0.488466692	0.725808605	0.461847184	11.5994715
11303	HSPE1	29.29295522	-0.152294887	0.471096824	-0.222277252	0.74648528	40.29414411
11304	IFT27	24.29071542	0.240760246	0.502698208	0.677862664	0.497858775	15.44895402
11305	LIN52	21.45624262	-0.061251096	0.555804045	-0.110282601	0.912105951	11.09180014
11306	LRBA	21.85257842	0.689294816	0.517225927	1.222418825	0.1827226	4.185814107
11307	MGLL	48.59658761	0.464142779	0.422260458	1.072759512	0.28292048	2.784819092
11308	MKI67	40.468268	0.205024941	0.462726215	0.442124879	0.658298842	2.524210584
11309	MLST8	49.19851061	0.182408989	0.425781612	0.42087262	0.672847262	11.24891079
11310	MOCS2	48.19920175	-0.112426725	0.416252745	-0.270115852	0.787071126	5.014010201
11311	MRPL1	26.51122801	0.282727547	0.479210551	0.798682471	0.424472972	21.18998244
11312	MRPL54	42.70251145	-0.12902297	0.45922929	-0.280890241	0.778794509	11.41092798
11313	NDC1	27.90991184	0.221056985	0.467218204	0.472022114	0.626189555	4.522479147
11314	NIFK-AS1	40.17568972	0.408221219	0.454129047	0.898890596	0.268710927	5.145117484
11315	POLR2J	45.22271427	-0.250602557	0.427649822	-0.585999429	0.557875898	15.1944741
11316	POM121	27.02421174	-0.252964695	0.504521428	-0.502277421	0.614698942	19.4098854
11317	RASA2	55.5642842	-0.298547288	0.292228602	-0.761156242	0.446562691	19.91495901
11318	RCL1	40.61167401	0.049959564	0.449982484	0.111025575	0.911596066	11.41821518
11319	RGL2	46.24482627	0.186820502	0.426874462	0.427670824	0.661624915	2.772517748
11320	RITA1	54.42241108	0.128082986	0.411024289	0.211612796	0.755224049	17.10907018
11321	RNPEP	47.6447827	0.100621468	0.461180512	0.218204077		

0.827270109 11.29029941
11322 RUBCN 44.0056898 -0.142989224 0.422682216 -0.222014749
0.729878122 17.49021989
11323 SMAD2 29.2507255 -0.029942277 0.460872618 -0.08666662
0.920926502 7.75402581
11324 SOS1 25.15452829 0.258122846 0.501976616 0.51422281
0.607089221 14.50824059
11325 ST7L 24.20222029 0.204201229 0.500072048 0.608514792
0.542846098 12.97810771
11326 SYNRG 48.15408651 -0.140409025 0.422929024 -0.221984108
0.729901259 7.497081225
11327 TAL1 40.58247072 0.015979594 0.452012574 0.025252101
0.971798979 9.475451292
11328 THAP5 47.68297794 -0.28756202 0.417720606 -0.92780204
0.252510266 2.779148299
11329 TRAPPC8 22.96112758 -0.09860226 0.524925422 -0.187840512
0.851001677 17.71190154
11330 TUBGCP6 48.52585412 -0.011055417 0.416225181 -0.026554765
0.978814852 4.148854088
11331 UBE2C 27.22420498 0.07454229 0.494081919 0.150870509
0.880077865 10.40417471
11332 UBXN2B 52.04690982 0.156187262 0.417179975 0.274288206
0.708115516 9.900294414
11333 UXT-AS1 48.06420166 -0.072152229 0.424966974 -0.17212888
0.862228247 7.187190855
11334 VPS45 50.40852201 -0.090672267 0.412266202 -0.219928584
0.825918987 8.100414012
11335 WDR44 44.42600229 -0.524202001 0.442722122 -1.204126006
0.228527015 5.718172789
11336 WDR47 41.25020624 -0.126812428 0.459192448 -0.297942901
0.765746744 11.40417074
11337 ZACN 27.86469648 -0.042952217 0.472662227 -0.090682421
0.927744127 4.18144148
11338 ZDHHC4 48.15955696 -0.44249242 0.416400176 -1.065065299
0.286846226 14.22142091
11339 ZFAND1 48.74809929 -0.11224462 0.412818162 -0.27265795
0.784247496 9.119151591
11340 ZFC2H1 42.2651125 0.065427792 0.440819628 0.148422046
0.882008912 5.711552157
11341 ZNF124 22.90561229 -0.098222556 0.528021499 -0.182744981 0.85499812
8.490181897
11342 ZNF26 46.25729095 -0.120652789 0.42180028 -0.286044762
0.774842828 7.950077701
11343 ZUP1 41.82879861 0.126262185 0.444712251 0.206621711
0.759122714 12.24771871
11344 AC002681.1 44.7681752 -0.484529901 0.449812578 -1.077202982
0.281289146 5.1184481
11345 AGPAT2 29.55942872 0.426082919 0.491284198 0.867109525
0.285882002 10.84541904
11346 ALDH1B1 46.51260926 0.292659406 0.459212294 0.857060772
0.291411299 12.07778547

11347 ALDH2B1 29.67459152 0.229745615 0.45525811 0.746106422
0.455602124 15.58824121
11348 ALG1 42.66829089 0.410885172 0.428672488 0.926655899
0.248925588 8.490121825
11349 ANP22A 60.56659222 0.546585011 0.421050692 1.26802954
0.204787402 4.119110844
11350 ARFGEF2 56.77156017 -0.421811455 0.285212825 -1.120965592
0.262202508 9.451948419
11351 ARID1B 51.15121117 -0.229599286 0.402679672 -0.818514826
0.412062274 11.11224118
11352 ARRB2 60.65055719 0.225228207 0.409246682 0.550228262
0.582155962 10.11821017
11353 BBIP1 45.85990402 -0.577688674 0.442219624 -1.206228818
0.191427262 10.87985585
11354 BRAT1 69.90282891 -0.290857014 0.278496502 -0.768452625
0.442217726 9.414209259
11355 C18orf25 29.07551112 0.627454979 0.552249757 1.124126082
0.256741664 7.474994544
11356 CASP7 42.90471267 0.25972765 0.426882554 0.594500862
0.552177165 11.15199557
11357 CCDC200 27.24207515 0.59561621 0.47206729 1.26171882
0.207049982 11.74882451
11358 CCDC51 45.6158222 0.172977244 0.428988729 0.402220945
0.686785692 4.141752452
11359 CCDC6 22.71440479 0.261802222 0.52269907 0.690859022
0.489654124 17.10249941
11360 CD151 56.22229522 0.494764091 0.425275226 1.162122624
0.244779248 8.875817727
11361 CDC72 49.97218722 -0.268156124 0.410869221 -0.652655648
0.512978201 7.494792909
11362 COG2 25.12050215 -0.026947125 0.511971641 -0.052624019
0.958022511 7.492858429
11363 COMMD1 29.25260982 0.2907017 0.474280656 0.612921808 0.52992142
7.419182211
11364 COPS9 46.97774677 0.02122652 0.425122968 0.049952561
0.960160192 11.11522421
11365 CXorf28 51.52954512 -0.221822925 0.412966226 -0.777415724
0.426912522 7.421118448
11366 DICER1 52.04029195 -0.545508519 0.402208098 -1.255947149
0.175115984 9.441127171
11367 E2F5 42.65421416 0.244519422 0.427612566 0.558756521 0.5762279
4.199412499
11368 EIF1AX 26.77590205 0.560624085 0.474818618 1.180722155
0.227708746 11.29970879
11369 EIF2J 59.22496128 0.178165269 0.411192291 0.422289557
0.664804424 9.927052474
11370 FAM76A 26.91222712 -0.164524191 0.497412129 -0.220779744
0.740810862 7.10415175
11371 FANCL 48.87155679 -0.299571646 0.416299525 -0.719606024
0.471767602 4.948022411
11372 GALNT7 20.02099847 0.216029665 0.554575612 0.569876601

						0.568761296	11.41000999
11373	GDF11	50.7577069	0.119011512	0.411728716	0.2890462	0.772546022	9.257047447
11374	GINM1	44.01404458	-0.176981698	0.424254081	-0.407552224	0.682601621	7.485451511
11375	HAAO	52.7108089	-0.019622056	0.402752027	-0.048599155	0.961228744	11.27508105
11376	HARBI1	40.68782681	0.220997659	0.450016618	0.725522191	0.462020921	7.488187849
11377	HGSNAT	51.82665282	-0.264011205	0.402625428	-0.904094179	0.265945465	18.20422018
11378	HIRIP2	45.04125648	-0.206126048	0.421416667	-0.47778879	0.62280054	8.904071081
11379	HLTF	41.01272219	-0.222919282	0.462701747	-0.717960164	0.472781849	10.42497798
11380	HNRNPAB	41.90271014	0.481172268	0.450692496	1.067628605	0.285688075	4.484581147
11381	HNRNPUL1	41.0900872	0.191529465	0.45248461	0.422250528	0.672769182	4.447084459
11382	ING2	54.87496486	-0.10671002	0.297547512	-0.268420797	0.788275422	10.42491791
11383	LACC1	47.59076085	-0.601966414	0.429617524	-1.401168204	0.16116278	7.928724214
11384	LGALS2BP	44.27876988	0.100287102	0.427405622	0.229505727	0.818475862	11.09254491
11385	LINC01278	41.52965292	0.659526167	0.46026784	1.422918206	0.151881214	7.479781819
11386	LRRFIP2	50.18050596	-0.077525506	0.410014102	-0.189104487	0.850010925	5.721150299
11387	MAFG	46.86249286	-0.09297541	0.440222572	-0.211200424	0.822720872	15.80770104
11388	MAPK8IP2	29.75920126	0.445281671	0.464250228	0.959256924	0.227278968	7.448490502
11389	MC1R	42.22598992	-0.061512209	0.451550447	-0.126224446	0.89164285	8.418441002
11390	MCRIP2	50.24420526	0.462295816	0.426211121	1.086989718	0.277041254	9.274814751
11391	MRPL9	59.11259205	-0.060917869	0.29292422	-0.15464262	0.87710228	11.22194084
11392	MSH2	45.22871449	0.222608906	0.429462988	0.520670959	0.602596008	9.257017284
11393	MZT1	27.22044244	0.297986944	0.470526756	0.845814782	0.297656077	11.58145925
11394	NAAA	41.17479727	0.506464027	0.452524824	1.119196118	0.262056481	15.7907891
11395	NAXD	49.22770278	-0.140586294	0.411022872	-0.242029485	0.722221178	7.11145879
11396	NR2C2	42.89904197	0.192962621	0.427284096	0.442460618	0.657422599	14.55819404
11397	NUP27	27.9718879	0.222508506	0.466661172	0.712526616	0.476128726	15.99222702

11398	PGRMC1	52.66762762	-0.070927722	0.400152882	-0.177251117	0.859211145	18.47195414
11399	POLR1C	41.20822082	-0.120892771	0.491879684	-0.266107292	0.790156579	10.29005004
11400	POMT1	28.62200276	-0.022860884	0.468261718	-0.050956299	0.959260244	7.40958408
11401	PPM1F-AS1	49.69920618	-0.166475728	0.40816081	-0.407867988	0.682270596	9.44142992
11402	PPP5C	47.11612808	0.509256122	0.424971216	1.170780952	0.241686826	14.21042078
11403	PRKAR2A	47.92427525	-0.400527258	0.417948826	-0.958240406	0.227891125	7.187190855
11404	PTBP2	26.71944542	0.262276051	0.474494066	0.765817904	0.44278466	10.84541904
11405	R2HDM1	47.14975175	0.12556057	0.428918246	0.208851526	0.757424472	12.88185141
11406	RERE	45.10641927	0.229405271	0.429659859	0.5571972	0.577292702	7.474994544
11407	RNASEH2B	62.55849289	0.014294594	0.297126286	0.02624597	0.971086221	4.481442172
11408	RNGTT	24.92491725	0.154481075	0.497909851	0.210259127	0.756262911	11.40115459
11409	RPAP2	46.60258942	-0.015649946	0.421920608	-0.026222548	0.971096924	8.189124811
11410	SNAP29	80.22122198	-0.5208141	0.268624782	-1.412856991	0.157697777	9.929828941
11411	SSH1	44.22229104	0.022611242	0.420848885	0.075690905	0.929665012	10.14112419
11412	STRIP1	47.78271228	-0.087015456	0.418129425	-0.208101525	0.825149689	11.24014411
11413	TMBIM4	29.42299288	0.102729956	0.460961229	0.222860297	0.822644228	7.978120911
11414	TMEM199	40.26117442	0.211497471	0.455719428	0.464095785	0.642579121	11.24408751
11415	TMEM28B	52.57994212	-0.586810086	0.297948577	-1.474587724	0.140222417	8.408444427
11416	TP52RK	68.72898182	-0.014746777	0.295471192	-0.027289122	0.970254471	10.85451994
11417	TRIM14	55.29854452	-0.028408507	0.299222841	-0.071159522	0.942270795	7.97141019
11418	TSFM	25.41910242	0.441469276	0.509486967	0.86649788	0.286217189	9.90875017
11419	UBE2F	26.12920901	-0.186877075	0.504770442	-0.270221905	0.711217157	12.87712145
11420	UQCR11	29.72104814	0.521707415	0.456806098	1.142076292	0.252422209	18.48981404
11421	VARS1	29.94195192	0.245575254	0.455441207	0.529202857	0.589746889	17.15145019
11422	WDCP	24.68158229	0.092257229	0.502259522	0.185206625	0.852988525	7.445455157
11423	YY1	40.92126217	0.029170822	0.458824624	0.062577204		

0.949206804	7.198521118
11424	ZFYVE1 57.7847454	-0.501292164	0.282686615	-1.206517205
0.191276722	11.21200479
11425	ZNF217 41.61251678	-0.019952722	0.447016225	-0.044627582
0.964296187	17.11408114
11426	ABCD2	42.28470847	0.122070506	0.441061696	0.201704972
0.762876978	11.4528229
11427	ABHD2	45.02717867	-0.20520841	0.420779817	-0.476264959
0.622814294	8.487294488
11428	ALCAM	24.72281215	0.174072416	0.501905625	0.246822996	0.7287242
11.84419914
11429	ANKZF1 44.04214666	-0.178040241	0.42580719	-0.408529829
0.682884728	8.182414189
11430	APBB1IP	25.05056465	0.565572919	0.489270202	1.155954142
0.247699915	8.429495944
11431	APIP	40.66787818	0.047474985	0.45811928	0.102620162
0.917462851	11.11214114
11432	ARFGEF1	45.07527944	0.14497046	0.427685225	0.228965242
0.724625915	4.997744959
11433	ATP5MF 22.51269962	0.228946751	0.508642107	0.646715522
0.517816069	10.59814852
11434	BTG2	22.2772278	0.296206265	0.508852452	0.778822004
0.426084574	10.59159794
11435	CDC40	25.72859146	0.169242658	0.492022854	0.242472678
0.721242892	7.114000177
11436	COX11	42.66908095	0.249228774	0.441487625	0.564769564
0.572220511	9.871141071
11437	COX6A1 50.25660869	0.062917685	0.409278844	0.152690611
0.877852688	7.958482418
11438	CRLS1	28.90608284	0.252915282	0.462607989	0.54887786
0.582089272	8.417100729
11439	DLAT	41.5571592	0.191607211	0.461292972	0.415269205
0.677871646	11.10488022
11440	DUSP22 41.88942221	0.272492216	0.442144016	0.614906682	0.52861625
4.44944921
11441	F11R	70.22847074	-0.474105275	0.264766186	-1.299751166
0.192686268	9.904015047
11442	FANCI	28.62016624	0.125942229	0.46776826	0.269240802
0.787744282	8.10419484
11443	FOXJ2	48.57086709	0.125922827	0.417141601	0.225867248
0.744524721	9.428891009
11444	FTLP2	47.28465077	0.247925221	0.427182206	0.580295782
0.561647748	8.450727117
11445	GDAP1	26.62741954	-0.104710621	0.496821855	-0.210756658
0.822077161	8.141184125
11446	GNB5	42.21044851	-0.09882271	0.427974225	-0.225625902
0.821484614	11.4041108
11447	HDGF	48.26401208	0.154187915	0.419425515	0.267608152
0.712165426	14.17281158
11448	HINT2	27.22192028	0.42291611	0.604254862	0.6997811	0.48406402
17.71857212

11449	HMBOX1	47.20976829	-0.211470847	0.420525149	-0.491192786	0.622290101	10.57847119
11450	ICE2	46.26554452	-0.182040875	0.42244002	-0.42227107	0.665544411	5.515475919
11451	ITGAE	45.52549052	0.082665544	0.428620626	0.192864125	0.847065281	11.40055011
11452	KDM5C	42.99220217	-0.112865299	0.448222792	-0.251742902	0.80122902	11.41558054
11453	LCOR	29.64902226	0.085702255	0.458852592	0.186776907	0.851825554	14.14185495
11454	MAZ	26.92467742	0.187112547	0.479106028	0.290545174	0.696122458	12.77555979
11455	MED17	49.09419645	-0.219780502	0.444254422	-0.494606212	0.620878045	10.42805049
11456	MGST2	46.26842994	-0.058261562	0.421608424	-0.1281888	0.890091207	9.441289848
11457	MPC1	55.21002208	0.024446802	0.400191722	0.086075746	0.921406204	10.40124127
11458	MRPS2	61.08081965	-0.009829792	0.292542458	-0.025066822	0.980001662	4.114285518
11459	MYCBP	27.72422266	0.549741202	0.470098289	1.16941754	0.242225446	9.288104074
11460	NFYB	28.72885279	0.194850709	0.472618129	0.412279279	0.680124661	7.147744184
11461	NKAPD1	40.95512607	0.210541287	0.451502242	0.687795727	0.491581421	12.42512921
11462	OTUD6B	27.51596226	0.186218194	0.622084797	0.294609512	0.768292195	4.484192841
11463	PBX2	42.81995156	0.022680021	0.422099994	0.077764999	0.928014989	5.484587129
11464	PHF12	41.16602985	0.04719456	0.455688907	0.102567498	0.917512582	9.584922141
11465	PIH1D1	44.87541668	-0.065927569	0.442827402	-0.148901284	0.881621528	9.401121748
11466	PLAC8	46.65944917	-0.114162742	0.427055455	-0.261211115	0.792929707	8.895415102
11467	PPM1A	28.46706296	0.14029876	0.474412205	0.295721142	0.767425411	9.194899541
11468	PREPL	29.10574525	0.052202224	0.466665821	0.111864272	0.910921025	11.5992712
11469	PRKX	26.15621989	0.012217669	0.494700158	0.02469712	0.980296552	12.59185919
11470	PUDP	26.67979956	0.128842476	0.482827227	0.266844476	0.789588908	5.511840582
11471	QTRT1	27.97162652	-0.048549221	0.498221949	-0.097445167	0.922272877	7.492858429
11472	R2HCC1	44.10994467	0.112259284	0.422210604	0.262217449	0.792152802	14.08151491
11473	REST	51.66926912	-0.074124102	0.404228886	-0.182267077	0.854510007	11.08414741
11474	RNF5	52.72761966	-0.047429625	0.402671567	-0.11781222		

						0.906216429	12.41145444
11475	RPS6KB1	22.77926625	0.674924829	0.514761959	1.211159104	0.189804016	8.915241404
11476	SERTAD2	22.80080724	0.259258028	0.521514158	0.49712558	0.619100497	9.142015547
11477	SLC24A2	47.44168295	-0.561542947	0.422227909	-1.295856964	0.195024769	5.498474547
11478	SLC29A2	26.48570762	0.185581454	0.48744992	0.280719008	0.702411762	15.78122212
11479	SLCO2A1	55.47722242	-0.109407677	0.404204088	-0.270674245	0.78664151	8.844779108
11480	SNX29	62.01122887	-0.205161042	0.278089451	-0.807112248	0.419601182	10.29571074
11481	SS18L2	42.08541806	0.082287886	0.426876622	0.190872851	0.848625211	9.199484817
11482	STX10	57.60199277	-0.22799065	0.287155047	-0.614716642	0.528741867	9.154519514
11483	STYXL1	51.61240821	0.154940517	0.428088219	0.261925952	0.717299892	8.472407204
11484	TAF6	48.19222866	-0.221799685	0.414259071	-0.559552467	0.575784724	15.77287715
11485	TAMM41	29.87982252	0.176868557	0.456720787	0.287257515	0.698565575	7.442409994
11486	TMEM168	28.1620422	0.201182926	0.46807624	0.429808028	0.667225292	11.91218822
11487	TMEM205	28.2226266	0.46440492	0.464260865	1.00021021	0.217160259	12.24792125
11488	TNFRSF1B	29.52296959	0.290920702	0.477778752	0.818225282	0.412228504	8.114271178
11489	TRAF6	40.57202707	0.017202284	0.457652282	0.027590286	0.970014252	14.144771
11490	TTC9C	42.16524872	0.290672278	0.442810261	0.882258865	0.277626826	18.08102505
11491	YDJC	46.9221762	0.461181109	0.424190254	1.062162659	0.288161295	8.884172714
11492	ZNF224	26.67969944	-0.107221414	0.500718061	-0.214125204	0.820441545	8.441145917
11493	ZNF264	27.72695889	0.252972428	0.468772656	0.755104682	0.450186174	10.11119819
11494	ZNF297	48.61807168	-0.466949542	0.418222841	-1.116242029	0.264218544	11.14222494
11495	ZNF581	27.89279749	0.146409626	0.472428151	0.209254184	0.75712818	7.752100472
11496	ZNF592	45.08662082	0.272885542	0.421155652	0.622916528	0.526788148	5.44105445
11497	ZNF672	58.82462888	-0.24244601	0.285069882	-0.891905669	0.272442496	8.910921974
11498	ZNF82	42.57512275	0.082148849	0.424568217	0.191226657	0.848261842	7.448540545
11499	ZNF875	41.59271824	0.016022211	0.446502525	0.025886271	0.971272042	14.4594458

11500	ABCD4	58.07472627	-0.184692478	0.285411589	-0.479211012	0.62178852	11.91407971
11501	ABHD6	27.04594547	0.526005165	0.478716127	1.119672254	0.262852452	14.10519749
11502	ABL1	44.28789929	-0.297820852	0.458057157	-0.868517928	0.285110862	10.41124517
11503	AC005104.1	48.64050207	-0.52874115	0.422424108	-1.251682177	0.210685222	7.101417517
11504	AC011450.1	45.020062	0.082427754	0.421525176	0.192250991	0.846684095	15.18710492
11505	AC125507.1	45.79562514	0.097226224	0.424887822	0.229062595	0.818819488	5.475842825
11506	ADPGK-AS1	47.22250015	0.276488852	0.421167202	0.656482294	0.511512792	8.447449184
11507	AL162586.1	40.62271181	0.085742274	0.452222424	0.189182648	0.849948882	5.981798948
11508	ALDH4A1	56.74096502	-0.119761255	0.407692124	-0.292752422	0.768946221	7.901014991
11509	ARHGAP10	21.78846225	0.600817559	0.527125882	1.129777286	0.254279057	11.91102511
11510	ARID5A	47.2267794	0.122801798	0.418854406	0.295572291	0.767556661	11.14995148
11511	B2GNTL1	42.1841525	0.415161957	0.428586802	0.946590171	0.242847654	15.54594119
11512	CAPNS1	65.46225128	-0.492298558	0.270622265	-1.220964601	0.182200659	14.55914444
11513	CASP10	41.61241451	0.119254521	0.445944864	0.267419899	0.78914588	15.80484571
11514	CASP8AP2	44.20402554	0.220128221	0.440526224	0.522416621	0.601280279	8.452910041
11515	CCDC25	51.4412522	0.106906228	0.412882758	0.258925995	0.795692241	4.455541428
11516	CDK5RAP1	54.21825507	0.595889221	0.425508272	1.400417722	0.161288262	8.149740114
11517	CHMP6	48.72922971	-0.201177029	0.41206449	-0.487025424	0.626222228	4.992148974
11518	CZIB	40.58216714	-0.089704484	0.460022222	-0.194999861	0.845292057	10.41148175
11519	DBR1	45.21002828	-0.024915008	0.442461469	-0.056210006	0.955094848	9.157254872
11520	DIS2L	51.12429706	-0.045080848	0.414921541	-0.10864909	0.912480822	7.112950115
11521	DPH7	50.27220602	0.25902561	0.422027172	0.612788929	0.529254807	7.975445424
11522	FAM20B	25.48928221	0.295655224	0.488210218	0.81025282	0.417794205	4.492924598
11523	FEM1A	52.20021207	-0.225797821	0.400405416	-0.588897708	0.555929896	10.14775481
11524	FUOM	25.99610098	0.627626022	0.486781049	1.209882594	0.190225556	5.470510419
11525	FYCO1	50.41278999	-0.291266606	0.40525121	-0.718977578		

						0.472154725	11.10488022
11526	GBA2	49.24904202	0.269965145	0.4261769	0.62245795	0.526424622	12.55409991
11527	GDF15	42.1587255	0.214878092	0.427084214	0.72040584	0.471275155	12.90144977
11528	HS2ST1	28.52406284	-0.280222182	0.49961882	-0.560871942	0.574884829	4.471504454
11529	KATNA1	54.05571924	-0.028428914	0.420240078	-0.091422288	0.927156179	11.4084484
11530	KCNN4	42.85466578	0.268699471	0.441500272	0.608605446	0.542785994	8.714891094
11531	LACTB	28.71756029	0.246102897	0.462814652	0.747822982	0.454566224	9.194899541
11532	LILRA5	20.06212602	0.262029521	0.562221092	0.642945106	0.519611021	11.25727884
11533	LINC00649	41.84982641	0.171142782	0.44627291	0.282494922	0.701252827	12.05802824
11534	MAN2A1	42.66624452	0.286488878	0.429826109	0.878721095	0.279547095	15.21147484
11535	MRE11	44.94545216	-0.008224219	0.452742251	-0.018408208	0.985212125	5.971507441
11536	MRPL24	48.77042841	-0.054286976	0.412264875	-0.121679847	0.895227526	7.947141254
11537	MRPL26	21.28462999	0.515181921	0.525825772	0.961472846	0.226214472	4.751889082
11538	MRS2	50.99671169	0.048765782	0.405728179	0.120192224	0.904220077	9.911917004
11539	MTA2	21.22526586	0.511004225	0.525219885	0.954755866	0.229701171	4.144171428
11540	NAA28	56.27602146	-0.461825102	0.285055922	-1.199297472	0.22027242	11.84974949
11541	NAPG	48.22555742	-0.172228502	0.412846285	-0.418605815	0.675504229	12.81077515
11542	OSBPL1A	40.55784926	0.018201488	0.462890502	0.029452172	0.968529885	7.449020415
11543	PCK2	42.86600717	0.40428578	0.447585297	0.902259529	0.266288165	8.411811271
11544	PHF11	51.78496659	0.284082298	0.411652972	0.690101414	0.490120414	9.405018022
11545	PIK2R2	41.28246206	-0.282156182	0.479524622	-0.590481192	0.554868091	10.11142958
11546	PIKFYVE	48.27788727	-0.085229417	0.415017569	-0.205287491	0.827269292	11.54001917
11547	PNPO	49.74700444	-0.080858405	0.408216295	-0.198077258	0.84298454	9.882281225
11548	POLA2	28.20795721	-0.028101621	0.491409258	-0.057185778	0.954297207	11.11279119
11549	RFT1	41.42254926	0.449971171	0.446690611	1.007244142	0.212769424	14.95205114
11550	RRP12	46.25222896	0.566850407	0.444282019	1.275592582	0.202099577	9.917204271

11551	RTN4	47.27012252	-0.294780574	0.426022458	-0.905412295	0.265246422	11.84419911
11552	SDC2	42.24269948	-0.128558262	0.447652749	-0.287182264	0.772972689	11.10544491
11553	SEC24B	52.24522721	-0.210225649	0.409422885	-0.512724264	0.607444715	14.25842141
11554	SEPHS1	20.59227628	0.596087242	0.529722857	1.104409578	0.269415508	5.498742971
11555	SMUG1	40.19224802	0.190617882	0.457522089	0.416620978	0.676955662	14.8155044
11556	SPRYD4	40.88181648	0.422492522	0.461162571	0.916146602	0.259590008	15.50925109
11557	SRFBP1	21.02985224	0.727581527	0.545951442	1.251002065	0.176694771	8.904011019
11558	SSNA1	52.71224172	-0.211166216	0.402195252	-0.525024201	0.599559416	17.07715541
11559	STX18	55.60078992	-0.272026902	0.288166788	-0.70227522	0.481821929	9.154549588
11560	TM7SF2	44.12964084	0.210924864	0.422152101	0.488079024	0.625492868	17.45114189
11561	TMED2-DT	54.72125622	-0.202612096	0.406257042	-0.501192778	0.616225456	14.8057571
11562	TMEM29B	47.60264584	0.127172909	0.421614912	0.225251179	0.7449152	9.449895909
11563	TRAPPC9	40.42871999	0.252126566	0.451251085	0.780256164	0.425181262	5.11429149
11564	TRMU	46.5122568	0.227457841	0.42688826	0.54252064	0.586771284	11.09542881
11565	TVP22B	44.40824251	0.061810622	0.444229705	0.129106684	0.889265847	9.145810851
11566	UBA6-AS1	42.02428074	-0.251026455	0.45518628	-0.771170822	0.440605686	11.79708985
11567	UBIAD1	42.26774528	0.20249891	0.440992476	0.459189025	0.646098424	12.82101441
11568	UCK2	22.4516212	0.27761006	0.526877467	0.716694267	0.472562764	8.450974591
11569	USP26	49.92124907	-0.149428068	0.408577817	-0.265751789	0.714550297	14.09101094
11570	UTP25	42.28952677	0.298425775	0.428549022	0.908521874	0.262597281	11.82091274
11571	XPO7	45.99529777	0.115059697	0.425120266	0.264421882	0.791447147	9.154519514
11572	ZFP26	51.25927745	-0.282208602	0.404102441	-0.700821949	0.482407897	7.457524445
11573	ZFP91	28.22218401	0.272861274	0.469287787	0.58256787	0.559511082	10.95022902
11574	ZFYVE21	52.10714624	-0.214290877	0.295225222	-0.795251247	0.426467209	8.184449524
11575	AC024102.4	42.40057671	0.096822292	0.441781157	0.219165724	0.826520947	4.478115188
11576	AC087292.2	50.66990805	-0.160962878	0.404690942	-0.297742725		

						0.69081984	11.57114801
11577	ACSBG1	28.89458912	0.142585722	0.468290917	0.20655106	0.759185109	11.41844597
11578	ANAPC15	50.02699648	0.124274789	0.408895142	0.228628968	0.74242615	8.291781741
11579	ANKFY1	42.22282026	0.170478169	0.446684729	0.2816521	0.702719424	11.54549722
11580	ARSD	52.66250466	-0.226479681	0.296668701	-0.596164206	0.551065525	10.44082598
11581	CCDC91	47.77400287	-0.085877591	0.416100705	-0.206286555	0.826488972	9.900294414
11582	CEBPZOS	55.45772215	0.261485921	0.416572751	0.867761825	0.285524722	17.14541492
11583	CIAO2	29.00182168	0.426226904	0.462669802	0.94082527	0.246789282	9.954015495
11584	DHX27	24.45251412	0.409778099	0.514952554	0.795757295	0.42617206	10.404941
11585	DLGAP4	42.52980611	-0.012282828	0.44167719	-0.020075876	0.97600654	7.97141019
11586	DUT	51.42140257	-0.116287414	0.402151598	-0.289162127	0.772456547	11.14911014
11587	ELP6	50.0297217	-0.009891028	0.410247765	-0.024104014	0.980769641	9.411980051
11588	ESS2	42.11998102	0.186582557	0.442197005	0.420994626	0.672759006	11.48484454
11589	FAM161B	48.42526904	0.082229192	0.422582469	0.192621922	0.847247242	8.148514794
11590	FBXO28	42.68877605	0.522792172	0.441564427	1.186221402	0.225524876	8.110212511
11591	FBXO9	44.80204595	0.099071122	0.420705098	0.220020781	0.818075622	10.44088404
11592	GABARAPL1	51.61002507	0.124502019	0.442226812	0.280895024	0.77879091	11.21911549
11593	GEMIN4	46.49225812	-0.102727261	0.422829876	-0.242975642	0.808024267	10.14118415
11594	HSPA14	52.51262808	-0.007575277	0.40266172	-0.018766299	0.985027459	11.40777711
11595	KDM2A	46.76642927	-0.026025927	0.419968252	-0.061994989	0.950566822	15.11174119
11596	KLHL21	57.55110557	-0.21052588	0.285616604	-0.545972029	0.585085164	8.418441002
11597	LZIC	28.67224296	0.272872861	0.466512569	0.58706427	0.557160542	10.2702518
11598	MAP2K12	42.58416859	-0.118422554	0.448262976	-0.264146008	0.791667412	14.1942041
11599	MDN1	24.71525402	0.59829568	0.51921827	1.15227125	0.249209586	8.402074047
11600	MRPL29	42.41842011	0.268424557	0.44126782	0.608225702	0.542971477	8.915211245
11601	MTG2	42.12207517	0.452125712	0.448175865	1.011044428	0.211995166	4.981957449

11602 NLE1 26.0072911 0.627146226 0.485292058 1.212912152
0.189212215 11.11494411
11603 NRDE2 40.12574027 0.086178705 0.45889481 0.187796207
0.851026409 14.08151491
11604 NSDHL 49.75252287 -0.128922487 0.40956598 -0.2291944
0.724462288 4.725977114
11605 NSUN2 66.52262621 -0.200894772 0.277085917 -0.52275597
0.594202516 9.458201815
11606 NUP42 44.72986857 -0.220279622 0.427018572 -0.504279764
0.614064795 10.8542878
11607 OSBPL2 42.85645849 0.098172202 0.422708606 0.226257515
0.820922269 9.154519514
11608 P2RX5-TAX1BP2 45.12869822 0.206921642 0.428249748 0.482202188
0.628951472 11.87219278
11609 PLEKHM1 42.11620422 -0.018006972 0.442122969 -0.040625598
0.967586404 10.12471419
11610 PSKH1 52.77825226 0.060224878 0.404694194 0.14884048
0.881679508 14.01218445
11611 RBBP6 22.00502575 0.575992696 0.541686186 1.062222814
0.287621049 5.447715244
11612 REEP4 49.24605641 0.122295705 0.414012772 0.298048062 0.76566648
9.882281225
11613 RINT1 45.20191672 0.074945718 0.461221654 0.162458702
0.870944647 11.55504721
11614 RNASE6 28.76819507 0.205542074 0.478904252 0.628002292
0.522472188 9.929889012
11615 SIAE 55.5227869 0.279277426 0.405512741 0.688700279
0.491011901 10.27875871
11616 SIGMAR1 72.50640559 -0.120972465 0.287278496 -0.212284926
0.754822995 9.451721155
11617 SPATS2L 42.11620425 0.182840969 0.426950474 0.418447811
0.675619726 5.944089445
11618 TBCC 59.96282456 -0.441621225 0.275226727 -1.176945208
0.229217227 10.49989058
11619 TCOF1 44.28859425 0.290091919 0.422687826 0.901555107
0.267292248 7.705149887
11620 THAP2 55.7977662 -0.075004444 0.405722591 -0.184861768
0.852227459 10.52051242
11621 TIMELESS 42.78676657 -0.064692625 0.446717979 -0.144817621
0.884854869 11.40110445
11622 TM9SF2 40.6724466 0.261122264 0.45097924 0.579026201
0.562564747 14.57058802
11623 TMEM18 50.69811122 -0.12260729 0.402517696 -0.221106642
0.740562924 15.41454459
11624 TMEM60 55.29646882 -0.579028604 0.291621715 -1.47856612
0.129256211 14.41174518
11625 TRIM28 50.45621899 0.108261401 0.414129952 0.261418912
0.792769475 12.08245424
11626 TTLL4 41.4868692 0.120688592 0.475462549 0.274866217
0.782419022 18.22922901
11627 UBXN10 46.2299742 -0.244105488 0.427051796 -0.571606278

0.567588741 21.41581447
11628 VPS22B 29.26459757 0.217211606 0.471054466 0.461117814
0.644714082 14.88718297
11629 ZC2HC1 64.55247207 -0.298676522 0.278957791 -0.788152507
0.420607506 14.44015874
11630 ZCCHC10 44.94872205 0.217527112 0.429799799 0.72200277
0.47029216 7.144178995
11631 ZFX 44.02075206 -0.078500614 0.426892622 -0.179679418
0.857404252 4.524112414
11632 ZNF516 49.11891626 -0.401415285 0.417815551 -0.960747821
0.226678975 10.18097494
11633 AC092115.4 28.95109472 0.620970798 0.474698057 1.229204508
0.182780508 7.747518705
11634 ACOT8 57.71117227 -0.421064225 0.285284199 -1.092582287
0.274576804 17.91459051
11635 ACSL2 62.99212194 -0.412425251 0.269125074 -1.12001075
0.262709181 8.171058208
11636 ANKRD26C 64.47716694 -0.455259029 0.267798096 -1.228067961
0.215690862 17.45944749
11637 ARHGAP45 67.6574426 -0.419521299 0.262018281 -1.158840092
0.246521272 18.44117409
11638 ARHGEF1 59.4448144 -0.494564297 0.276612527 -1.212191272
0.189118504 9.498121844
11639 BRD2OS 51.6292708 -0.281185569 0.402090027 -0.945658621
0.244222724 8.507420142
11640 C19orf25 48.06668107 -0.102045214 0.422982074 -0.241251864
0.809259916 4.751155209
11641 C5AR1 48.02455127 0.170187962 0.418172622 0.406980152
0.684022566 9.449919544
11642 CCDC88C 22.49648408 0.201120292 0.520702406 0.278989026
0.704696006 18.48275474
11643 CINP 51.01620666 0.104927822 0.404267147 0.259486542
0.795259865 14.87250297
11644 DARS2 51.52128028 0.102411564 0.404042284 0.255941724
0.797995828 9.855177978
11645 DOLPP1 48.98680048 -0.272615478 0.416518261 -0.656910962
0.511228168 12.11179971
11646 ELL 42.40972275 -0.071686282 0.462204259 -0.154761708 0.87700918
11.08702182
11647 FAM214B 52.67941554 -0.099529968 0.296728692 -0.250901862
0.801889984 7.920119199
11648 GDPGP1 46.72117278 -0.178162492 0.424907982 -0.419298999
0.674997628 10.14795428
11649 HNRNPA1P7 28.72019224 0.208865659 0.467020728 0.661252212
0.508285822 7.115291929
11650 KBTBD2 56.20681762 -0.541742875 0.288410407 -1.294769202
0.162085449 14.44040892
11651 KYAT2 42.70489087 -0.161462782 0.471854854 -0.242189512
0.722208277 17.11874424
11652 LIG2 52.42742207 -0.021649885 0.298897628 -0.079242275
0.926759506 5.979841412

11653	MAGOH	45.20299182	0.194021918	0.421682711	0.449477042	0.652087565	7.947141154
11654	MICOS12	45.22552442	0.160974168	0.426776282	0.277186202	0.706025159	8.490080575
11655	MKRN1	61.9188109	-0.220272765	0.277750828	-0.874216454	0.281945941	9.192911924
11656	MPST	44.89222529	0.222702791	0.429857662	0.520411212	0.602776927	14.02147785
11657	MUS81	77.04914669	-0.458257621	0.258597758	-1.277915468	0.201279224	10.15919744
11658	MXD4	46.67590725	-0.489601106	0.440702798	-1.110955292	0.266587594	4.728711175
11659	NCOA1	46.25967027	-0.150721427	0.425572757	-0.25416052	0.722218582	4.947981475
11660	NF1	57.72947568	-0.244777199	0.291477924	-0.625264272	0.521797559	12.08901584
11661	PGAP6	42.80074198	-0.122578268	0.449450212	-0.294978764	0.768010102	14.07117144
11662	PHF6	25.27120007	0.452069855	0.68610077	0.658897168	0.509961805	9.114145108
11663	POLH	41.00282212	0.279922544	0.459944569	0.826042026	0.408780252	15.88887141
11664	PRKAA1	56.19186508	-0.170016857	0.292802256	-0.422820544	0.665127882	11.24095421
11665	RAB8B	54.07222855	-0.414908267	0.292098272	-1.055482552	0.291204685	9.449491952
11666	RABGAP1L	57.96500181	-0.202856201	0.401649747	-0.50505746	0.612518479	8.980172812
11667	RFWD2	50.4728295	-0.264409259	0.40920209	-0.646000265	0.518279184	7.494441449
11668	RHOF	45.61272225	0.201296448	0.425661669	0.708065747	0.47890442	12.04429214
11669	SAFB2	50.76018752	0.119847147	0.405520468	0.295529081	0.767582102	11.84472042
11670	SEC62	56.67216486	-0.091161012	0.288578822	-0.224601075	0.814518275	10.91059204
11671	SIGIRR	55.56681268	-0.245190546	0.28921697	-0.629796708	0.528827599	7.705098418
11672	SLC25A19	57.62282892	-0.11284975	0.28478298	-0.292281552	0.769206952	10.49118225
11673	SLC7A7	29.91224284	0.067286215	0.465558199	0.144528041	0.885082515	5.121151211
11674	ST2GAL5	66.02084279	-0.446210862	0.278228824	-1.179294857	0.228240982	15.55715141
11675	TP52BP2	27.21602894	0.622229624	0.478542501	1.221152922	0.186450046	7.747418581
11676	TRIM22	24.22706894	0.686892708	0.498589522	1.277671769	0.168204651	4.008115747
11677	TUT7	42.22422165	-0.022068241	0.440990865	-0.072718606	0.942020042	11.27119557
11678	TYW1	29.70244007	0.229277787	0.457861572	0.741005157		

0.458690212	8.980172812
11679	USP1	44.24024222	-0.0968688	0.429672425	-0.22022029
0.825621641	14.98402845
11680	ZNF121 55.86521271	-0.25880529	0.286199219	-0.67012442
0.502772104	11.15189427
11681	ZNF791 50.48427101	0.021550214	0.405076957	0.052200542
0.957572122	15.18122504
11682	ZNF84	45.22504262	-0.278500905	0.444478914	-0.851560992
0.294457798	7.717481178
11683	AC022055.1	61.66895719	0.151742806	0.288720778	0.290257066
0.696272521	11.12845908
11684	AHSA2P 42.26581986	-0.200697814	0.464904087	-0.421697246	0.66596147
4.48901799
11685	AKAP11 42.82277256	0.165756747	0.426989272	0.279215269
0.704452688	7.195445425
11686	AL122452.1	42.22277655	0.124196146	0.440612018	0.20456691
0.760696025	19.55272408
11687	ATP11B 40.18915915	0.155677874	0.462521205	0.225859228
0.726977024	7.947091095
11688	ATP6V0E2	60.42700998	-0.411827728	0.277178746	-1.091862558
0.274892078	11.41824477
11689	CCDC97 42.28487602	0.170177699	0.456629282	0.272674242
0.709290909	11.14172519
11690	CFAP20 64.88579299	0.045995777	0.285489219	0.119217922
0.905022477	11.1027924
11691	CNOT4	50.00864086	0.126122686	0.40827747	0.222250128
0.728869997	14.54108078
11692	COA2	54.44125987	-0.029825459	0.291855049	-0.0761125	0.9292288
10.14198192
11693	CSTF2	41.16006449	0.04867721	0.460245784	0.105762511
0.915769989	9.877710547
11694	DDT	46.86780874	0.092908905	0.425270782	0.218469994
0.827062925	10.45745942
11695	DDTL	48.58458897	0.076849228	0.415795042	0.184824782
0.852266469	14.2447841
11696	EMC2	46.12054002	0.297650151	0.422102114	0.702494824
0.481747425	11.41159295
11697	EMC6	45.61825168	0.227666579	0.424821864	0.559449969
0.575854667	14.17844508
11698	EPS15L1	54.28969812	0.406681521	0.406484576	1.000484508
0.217076091	4.981904512
11699	FAM111A	49.77206878	-0.168256456	0.41416221	-0.406498826
0.684276112	11.27125415
11700	FAM217B	21.27014966	0.142720774	0.584648691	0.24582416
0.805818254	11.25429112
11701	FBXO42 52.66996698	-0.177579622	0.29287249	-0.452002051
0.651267505	12.5954425
11702	GIMAP2 42.49662112	-0.112270182	0.465467101	-0.242562181
0.807569922	8.89819919
11703	HEATR5B	56.52498272	-0.060116697	0.405462414	-0.148267004
0.882122054	10.17227428

11704 HNRNPL 49.27000852 0.294229425 0.414585757 0.709926172
0.477742718 11.21419491
11705 HPS5 22.02020129 0.481726104 0.540787127 0.890805404
0.272022575 11.45248144
11706 IFI25 59.55280187 0.259022209 0.405998252 0.884295209
0.276526874 7.414294829
11707 KIAA0100 45.84222219 0.158780125 0.422754959 0.274697975
0.707885099 8.45270874
11708 KIAA0512 44.57969851 0.242571706 0.441474045 0.775972472
0.42776524 5.487271449
11709 MALSU1 44.62481275 0.272859177 0.422002494 0.862092968
0.288086254 9.418754081
11710 MARCHF5 54.52880519 0.221548196 0.299488011 0.579612279
0.562176042 9.410587519
11711 MCRIP1 62.22118524 -0.289582442 0.268564927 -1.057027955
0.290498829 11.89851044
11712 MRPS20 65.29655952 0.026824202 0.281089561 0.070414687
0.942862601 12.07479904
11713 NDE1 52.57500725 -0.149918762 0.412672278 -0.262286729
0.716290706 11.29288484
11714 NUCB1-AS1 54.11465756 -0.040257291 0.400824278 -0.10042626
0.919997984 11.42597942
11715 PDS5B 48.99824091 0.168541704 0.417408554 0.402781145
0.686272662 15.58814011
11716 PEX16 46.24701426 -0.060904991 0.42185522 -0.141021027
0.887845422 15.59101529
11717 PMVK 45.82209092 0.222476946 0.425002561 0.52247078 0.60064668
12.42120174
11718 PRPS2 45.1511787 0.299941564 0.42792518 0.924584447
0.250002498 7.722251971
11719 PTCD2 47.28576778 0.212225877 0.42251102 0.504900127
0.612628978 14.0245121
11720 PTPN9 51.74521845 0.220512548 0.407827179 0.565207292
0.571922776 11.85522918
11721 RAB5C 56.64207509 -0.089768106 0.285590709 -0.222806715
0.815911498 12.1724257
11722 RABEP1 71.20645121 -0.461407096 0.26967748 -1.248124176
0.211981925 7.971559152
11723 RBM4B 67.91067625 -0.406970222 0.26022052 -1.129780785
0.258568607 10.9148499
11724 RBMS1 42.940916 0.226088515 0.442761181 0.5106221
0.609607994 15.08420742
11725 RPRD1A 26.65974759 0.170902075 0.500808257 0.241254508
0.722911992 14.81814999
11726 SEC14L1 52.22691817 0.279264612 0.406942729 0.686494425
0.492401272 12.19714425
11727 SLC28A7 48.24528179 0.091585096 0.41766202 0.219279874
0.826422029 9.148797497
11728 SLX4IP 28.48282042 0.559175215 0.466988012 1.197408072
0.221147529 11.14149442
11729 SSH2 70.78682212 -0.468714858 0.255751587 -1.217524122

						0.187659648	10.41507941
11730	TAF12	42.91270422	0.471024455	0.441079602	1.067889904	0.285570177	14.50178894
11731	TMED1	58.4151117	-0.2725252	0.290671985	-0.700140298	0.482829692	11.24871154
11732	TMEM106B	42.16962211	0.046656282	0.452920024	0.102012408	0.917952122	8.19194897
11733	TP52I2	59.00594262	-0.227240289	0.282826451	-0.592847128	0.552614264	9.122507447
11734	UHRF1BP1	47.25882798	0.026261526	0.42020629	0.086294582	0.921222245	9.124191951
11735	URGCP	50.59071644	-0.229410576	0.415155459	-0.792462192	0.427508006	12.87150994
11736	USP2-AS1	41.88245685	0.272972076	0.447269686	0.612545595	0.540176841	4.714445987
11737	UTP15	44.6472964	0.427406994	0.421542411	1.012589818	0.210778518	10.91745518
11738	WDR74	52.7202004	0.117445021	0.299567262	0.29292049	0.768811019	9.902078502
11739	YEATS4	42.5868516	-0.050202014	0.449779551	-0.111616922	0.911127146	12.59559244
11740	YIPF6	52.16608456	-0.215952686	0.297741656	-0.794266596	0.426982044	14.01028451
11741	ZNF271P	62.25019145	-0.199242225	0.272269082	-0.525225257	0.592417981	10.91481982
11742	ZNF445	50.06718684	-0.221870112	0.425607292	-0.779756458	0.42552424	11.85904591
11743	ZWILCH	44.16226129	0.208669221	0.422709149	0.712241222	0.475624567	9.95107915
11744	ABHD14A	56.71186126	-0.428429669	0.29254576	-1.091428791	0.275079851	8.891478558
11745	AC016722.2	49.2927024	0.092659746	0.408896885	0.226609077	0.820727722	11.9091987
11746	AC025422.4	50.22245681	0.248741497	0.409210271	0.852220248	0.294086256	11.4085474
11747	AC105020.6	44.45021786	0.221692462	0.42792167	0.724591222	0.462588419	14.41728482
11748	ANKRD40	51.48884792	-0.024277942	0.400514652	-0.085584742	0.921796528	18.11104212
11749	ARNTL	47.59282512	0.108261242	0.41825294	0.258841179	0.795757784	9.704151227
11750	C1orf121	41.19292729	0.228549855	0.450500287	0.729299828	0.465818278	14.04008277
11751	C1orf74	26.51255509	0.284892212	0.490671795	0.784421106	0.422792058	11.24110488
11752	C9orf72	42.68852251	0.524656112	0.441405552	1.188602224	0.224595795	14.97149717
11753	CAAP1	51.52122795	0.121899267	0.401694687	0.228257012	0.742641742	4.947981475
11754	CASP9	48.25110147	0.291919429	0.416526528	0.94090052	0.246755826	5.490104794

11755	CCDC159	54.72264575	-0.07018869	0.292995728	-0.178599116	0.858252489	8.942444491
11756	CCDC18	57.72146218	0.10882704	0.290481775	0.278725019	0.780455861	11.47049471
11757	CISD2	51.88056209	0.449976797	0.411115711	1.094525908	0.272724291	7.944928511
11758	CRTC2	62.76221991	-0.201298552	0.270898256	-0.542722292	0.587214068	9.1122797
11759	DAG1	67.62241729	-0.461822627	0.25675208	-1.294518961	0.195486224	18.19114574
11760	DDX42	46.14178021	0.292225706	0.422402288	0.926602707	0.254122849	14.21400017
11761	EIF2B2	29.94144485	0.247296656	0.462408176	0.525017909	0.592627472	12.18194741
11762	ELOF1	47.82747174	0.214244554	0.415172648	0.516276875	0.605661045	15.55715154
11763	EXOC2	51.11205549	0.242721729	0.411422952	0.589940929	0.555220252	14.08519901
11764	FAM177A1	48.25788546	0.02612916	0.414846294	0.087114579	0.920580427	8.411710172
11765	FBXL20	50.28229662	-0.276190922	0.412897528	-0.908898718	0.262402591	15.01514594
11766	FBXO11	50.81082112	0.221947742	0.405241199	0.572228294	0.567167254	11.14114045
11767	FRMD4B	22.8126842	0.720595972	0.522229479	1.277181965	0.168455996	10.71722951
11768	GABPA	46.90172171	0.422746468	0.421262292	1.027258661	0.204298682	12.40977017
11769	GANC	40.81261267	-0.022761541	0.472720528	-0.071268899	0.942182747	8.441417415
11770	GPSM2	61.06066648	-0.008098612	0.282629608	-0.021165671	0.982112499	17.07411907
11771	H2AZ1-DT	49.05722157	0.126564717	0.409965295	0.222112792	0.729049142	14.1811142
11772	KIN	46.29789925	0.274175128	0.422425489	0.885756872	0.275748554	11.1109058
11773	MARCKS	25.76782029	0.662042467	0.492661405	1.242112842	0.179225114	12.78479997
11774	MGAT5	49.76649712	0.092115521	0.407249527	0.228588778	0.819188548	10.10541441
11775	MRPL21	47.74822222	-0.257728148	0.424029882	-0.824224695	0.409811898	14.07121517
11776	MTAP	44.58800222	0.211968214	0.422009974	0.489522927	0.62447152	9.455415241
11777	MTO1	48.50266221	-0.097509149	0.41426577	-0.225278242	0.812915172	4.455491501
11778	MYO6	51.74208166	-0.159774254	0.404102772	-0.295279515	0.692562825	5.145074115
11779	NLN	48.72784284	0.007729475	0.415146649	0.018618662	0.985145215	12.79141041
11780	NT5C	64.22104565	-0.217417267	0.269291222	-0.588582929		

0.556141088 15.08147152
11781 NT5C2A 46.99121619 -0.192919682 0.426445551 -0.452289952
0.650988084 7.477121822
11782 NUP42 54.68867165 -0.207420797 0.298052766 -0.772224854
0.429916127 8.445050117
11783 OARD1 40.86444967 0.175215092 0.461429252 0.279929268
0.702990498 4.449548111
11784 PPP2R1B 22.54810918 0.751061256 0.522876298 1.406807566
0.159484458 17.14189201
11785 PRAM1 57.24682227 -0.170925872 0.299224092 -0.428027248
0.668621282 11.41875121
11786 PUM2 28.9450214 0.020217282 0.48846976 0.062066028
0.950510242 11.8449892
11787 QRICH1 69.28809804 -0.48205294 0.255772282 -1.254942801
0.175425462 11.89841058
11788 RASSF7 42.41260601 0.40722042 0.442222027 0.918998616
0.258096296 11.44149144
11789 RBIS 51.86076572 0.227746062 0.408727928 0.826224662
0.408614276 12.82742597
11790 RCOR2 54.92259958 -0.18665549 0.290926546 -0.477469469
0.622027857 7.701212242
11791 RFFL 42.02924682 -0.185122612 0.469727627 -0.294106282
0.692502572 11.14050191
11792 SCNM1 52.47124484 0.242579242 0.409429118 0.592467186
0.552527788 9.127554422
11793 SFI1 22.49628181 0.275766815 0.521716684 0.706704954
0.479749851 9.417549548
11794 SLC25E2B 54.58441462 -0.250212927 0.292162912 -0.628025671
0.52245046 11.24572241
11795 SLC9A8 47.05617886 0.049722149 0.419214912 0.118607777
0.905586104 4.420072504
11796 SRR 51.52972299 0.215704805 0.402922226 0.525226242 0.5924172
11.92142449
11797 STT2B 48.04688156 0.079472922 0.414110295 0.191914894
0.847808871 15.2489218
11798 TBCA 57.14018562 -0.028080582 0.284644268 -0.099002082
0.921126617 11.40404954
11799 TMEM120B 44.88088176 0.419825721 0.421810625 0.972244977
0.22092869 12.55404877
11800 TMEM165 48.52798108 -0.188044872 0.42101492 -0.446646576
0.655120261 10.1140814
11801 TMEM94 54.2650048 -0.122690975 0.292495247 -0.227211127 0.72595775
8.479114727
11802 TOMM24 50.58011997 0.222768579 0.408109455 0.815290517
0.414848811 7.419181114
11803 TOR1B 28.25862872 0.620278884 0.468425292 1.224150227
0.185452121 10.44914081
11804 TOX 49.62940715 -0.22816409 0.417762096 -0.809462768
0.418248425 11.11914144
11805 TSPYL4 42.4710066 0.520977904 0.427282724 1.212989202
0.224751847 7.471411101

11806	TTC27	22.22264801	0.268225608	0.561069974	0.656470005	0.511521761	17.47457949
11807	UEVLD	44.0786002	0.280529497	0.442668888	0.622722092	0.526261551	5.945785812
11808	UFSP2	61.95810211	-0.221182552	0.270797516	-0.892162814	0.271769989	7.711040447
11809	YIPF1	51.26210828	0.129758927	0.44882681	0.211287206	0.755506279	9.444174079
11810	AC024102.6	65.98841049	-0.210778642	0.262787885	-0.856640078	0.291642829	17.01774144
11811	ARFIP2	49.1588522	0.26861422	0.417277242	0.882279428	0.277021202	11.42945409
11812	ARL16	49.54420082	0.252041728	0.410819885	0.615942256	0.527921971	11.15840428
11813	ATP11A	41.76258116	0.522848072	0.450614652	1.182491667	0.22701067	11.17158211
11814	CCDC22	56.28852884	-0.179281992	0.282742084	-0.467454577	0.640174682	11.4144881
11815	CCNA2	47.49569225	0.517042228	0.427821545	1.180916118	0.227626048	11.41054142
11816	CLPX	49.59752174	-0.157526517	0.426612248	-0.260814705	0.718227969	9.92700154
11817	CSGALNACT2	22.22760667	0.527867295	0.527967921	0.999809408	0.217402752	14.42744881
11818	CSTF2T	47.94227202	-0.227882468	0.420520642	-0.784804689	0.42256809	9.420097449
11819	DEPDC5	47.20782965	-0.112486282	0.429752724	-0.264072065	0.791722618	12.80942149
11820	DUSP12	54.2791204	0.052620119	0.402458982	0.120422475	0.896222186	11.08702182
11821	FAM204A	52.07077705	0.208928447	0.401656742	0.520166662	0.602947419	9.404944897
11822	GATAD2B	40.86161225	0.217867556	0.471144624	0.674670861	0.499884902	7.920119199
11823	GPCPD1	51.22964206	-0.221070224	0.41601978	-0.79580407	0.426145926	8.110111214
11824	HEBP1	47.56279542	0.201122464	0.417260988	0.4818922	0.629881746	9.711871948
11825	IMPDH1	55.98642852	0.150454965	0.290251822	0.285424256	0.699915707	14.19172498
11826	INTS12	41.48109408	0.555925295	0.447277976	1.242907822	0.212901802	10.88142981
11827	INTS2	44.76492184	-0.155718875	0.444756766	-0.25012142	0.726247569	11.55114944
11828	KBTBD4	67.12281065	-0.225675201	0.26919692	-0.611259689	0.541027672	8.487245251
11829	KIF20B	49.94914649	-0.091579068	0.409909546	-0.222412869	0.822214189	9.420224822
11830	LRIG2	50.02092099	-0.027276121	0.408575111	-0.091479192	0.927111828	11.88921218
11831	LRRC40	28.18506882	0.16287912	0.487078576	0.226452148		

0.726529177	9.945458519
11832	LTBR	42.86187457	0.220290195	0.429779027	0.751026695
0.452620572	14.11558111
11833	MECP2	48.80951715	0.022852855	0.412024616	0.081962722
0.924675561	9.194748182
11834	MPHOSPH10	60.02096278	0.212002222	0.297857201	0.52286285
0.594128522	14.04018289
11835	MYBBP1A	29.62170226	0.207809799	0.4786241	0.642100427
0.520158921	11.07851579
11836	OSTM1	60.75902052	-0.472119477	0.272215661	-1.271089656
0.202696755	11.41174014
11837	PPWD1	50.85021452	0.258919022	0.407447588	0.62546586
0.525124626	18.4894448
11838	RELA	50.77421084	0.425129274	0.424250814	1.025422681
0.205162219	12.21111094
11839	RFC2	58.64287522	-0.282481795	0.284197867	-0.727852641
0.460602292	8.912717187
11840	RMDN2	44.27112722	-0.022482247	0.445461498	-0.072920427
0.941869421	8.447547987
11841	SGSM2	27.21122814	0.244086197	0.492122924	0.494969278
0.620621726	11.40578111
11842	SH2TC1	45.12978511	0.499099957	0.422122591	1.152202928	0.24919661
15.70891241
11843	SLC5A6	55.16424971	-0.066919475	0.28789006	-0.172521757
0.862027258	12.78485004
11844	STK40	50.98506456	0.125209966	0.406229729	0.222079107	0.72907457
7.710749211
11845	TBCCD1	48.49689122	-0.186452684	0.419075149	-0.444914676
0.656281416	4.449810808
11846	TMX2	51.19024758	-0.215722491	0.4045126	-0.522291071
0.592822108	8.441917255
11847	TTC28	59.84751729	-0.118216675	0.27601774	-0.214291217
0.752222926	14.77840521
11848	TYW2	42.94642426	0.225676154	0.428162657	0.742276828
0.457214092	11.2842712
11849	UBXN8	45.11982756	0.080207846	0.422210708	0.185225475
0.852965915	14.12154212
11850	VRK1	49.29522412	0.222107996	0.415592724	0.777462054
0.426885624	14.80407228
11851	VTI1A	49.05160974	0.127069092	0.409820179	0.224452292
0.728027456	7.9752945
11852	ZNF195	46.16127625	0.296584591	0.425729586	0.696622814
0.486021989	12.07742441
11853	ZWINT	54.24129496	0.06144079	0.292665616	0.15647102	0.87566176
7.184404271
11854	ABCB8	60.60154671	-0.268425605	0.279509865	-0.970792221
0.221651259	11.58514242
11855	ABRAXAS2	50.48680169	0.10805715	0.40776112	0.265001106
0.79100861	15.02247188
11856	AFTPH	49.28282161	0.264927721	0.414225859	0.629581826
0.522444494	19.81909957

11857	AGPAT2	68.86247694	0.171216874	0.280244187	0.450425902	0.652402271	10.19590494
11858	AKNA	60.22692154	-0.420519021	0.280652714	-1.120999122	0.258055456	5.141492574
11859	ANKRD12C	47.68286221	0.104214889	0.445505027	0.224149747	0.814868727	9.192941998
11860	ATP1A1-AS1	44.20528777	0.506722869	0.422960787	1.170289755	0.241844157	4.982529089
11861	ATP2B4	45.9222592	0.212094489	0.429884622	0.728222125	0.466416422	17.01119187
11862	CAB29	46.6141712	-0.045056952	0.427072828	-0.102087728	0.917892227	8.191918908
11863	CCDC22	51.04992602	0.221545262	0.406622795	0.815242294	0.414875776	10.84841217
11864	CDC16	56.40818495	-0.179897478	0.28452629	-0.467829528	0.629906502	12.22094814
11865	CEP95	28.44297217	0.256928576	0.482892402	0.522061748	0.59468222	14.12811282
11866	CHCHD1	49.25117105	-0.051285891	0.411685584	-0.124575287	0.900859715	11.44995591
11867	COPS7B	51.82121891	0.481827271	0.41012612	1.174798647	0.240075282	9.880495851
11868	DNAJB14	52.42861227	-0.057877449	0.298970728	-0.145066902	0.884658049	19.44907417
11869	DYRK2	46.04279122	0.082920481	0.429461215	0.192102541	0.84687788	9.420224822
11870	EIF2D	62.10180252	-0.171525802	0.272260609	-0.460768071	0.644965012	11.88721915
11871	EIF2B	42.92285062	0.427244729	0.424282821	0.982792758	0.225216929	11.90985184
11872	ESRRA	59.76071689	0.298971998	0.290421925	0.765766415	0.442815201	8.704157209
11873	EXOSC9	44.18265492	0.144197909	0.44745005	0.222265926	0.747251227	15.05052277
11874	F2RL2	60.52116266	-0.245561655	0.271972986	-0.660159917	0.509151212	9.948192845
11875	FAM168B	41.87186192	0.127170179	0.456458529	0.200509621	0.762788459	8.490180499
11876	FARP2	49.72401484	0.027525972	0.412068228	0.091091645	0.927419771	11.14149442
11877	FBP1	47.07298854	0.172102628	0.419677768	0.410085168	0.681742472	12.09459442
11878	GOLPH2	59.81642752	-0.116912728	0.276261955	-0.210629098	0.756075	9.192911924
11879	GSKIP	51.51968416	-0.025114099	0.402018766	-0.087127702	0.920570006	8.875714529
11880	IBTK	44.92574454	0.287701202	0.422820552	0.664712662	0.506224294	17.20112505
11881	KIF12B	52.89484402	-0.161626282	0.292818292	-0.411452192	0.680740257	18.7487194
11882	LARP4	41.54207014	0.661085004	0.452910225	1.459627708	0.14428967	

							11.45571472
11883	LARP7	57.16560147	0.290005168	0.402585582	0.718571677	0.47240487	17.00085049
11884	LETM1	51.24122268	0.247080681	0.421850847	0.822756868	0.410646269	11.44711049
11885	LMBR1L	56.86690207	-0.151222759	0.282066457	-0.29580224	0.692250924	11.44711049
11886	LSM10	56.42081766	-0.052712414	0.284259202	-0.127181916	0.890887004	11.41551941
11887	MTRR	52.2226791	0.008665868	0.294155924	0.021985889	0.982459212	14.07405787
11888	NEPRO	52.18278455	-0.017005244	0.400898078	-0.042418122	0.966165281	10.42287112
11889	NTMT1	45.12958278	0.271980779	0.428622829	0.624545712	0.525724744	4.524479441
11890	NUFIP2	56.24052712	-0.177296224	0.285449626	-0.460222174	0.645249579	17.008451
11891	PHYH	64.41204979	-0.282992902	0.262122499	-1.057477585	0.290292679	11.97485751
11892	PIP4P2	58.18072587	-0.542826518	0.288284967	-1.2980264	0.162102127	11.27494174
11893	PLGRKT	45.81781085	0.251002209	0.428565428	0.800244001	0.422511516	5.777212418
11894	POGZ	44.10650107	0.050000104	0.441522527	0.112242172	0.909828562	11.87144474
11895	PPARD	40.46492225	0.497252028	0.456542524	1.089166752	0.276080258	4.515972401
11896	PRKCD	57.88874252	0.075124182	0.285785567	0.194756222	0.845582708	19.41280947
11897	RNFT1	46.16679556	0.045529212	0.446221224	0.102020058	0.918722816	18.77212188
11898	RPAP2	49.04582785	0.049221471	0.411097242	0.119975192	0.9045028	18.97911911
11899	RPRD1B	42.07998042	-0.049721417	0.46556655	-0.106819128	0.914922465	9.001705217
11900	RSPH9	52.10096849	-0.2678908	0.402001592	-0.91514762	0.260114128	18.51994908
11901	SDF2L1	52.86947609	-0.160622961	0.295514528	-0.406129206	0.68464022	11.2770118
11902	SHQ1	42.20715508	0.126447675	0.4542468	0.200282247	0.762885602	19.41101429
11903	SLC26A6	41.06182042	0.667948198	0.460060816	1.45186925	0.146527926	5.00890595
11904	SLC41A2	44.72522591	-0.447217654	0.476010171	-0.929512811	0.247467518	8.501708212
11905	SMARCAL1	56.14922046	-0.19222612	0.2848067	-0.502424022	0.615269284	12.2950144
11906	STX16	47.8441812	0.15255111	0.416259216	0.268794791	0.712280688	11.27417048
11907	TMEM222	62.17164464	0.149008152	0.280727202	0.291267257	0.695525717	11.11744711

11908	TRAF2IP2	50.28577557	0.221057686	0.41540525	0.55622222	0.578058951	18.14190044
11909	TUBGCP4	62.9147842	0.217902624	0.288151224	0.819020001	0.412775002	8.944148701
11910	VEZF1	80.99190552	-0.241822227	0.250666644	-0.974809921	0.22965455	10.84854477
11911	ZBTB80S	28.17929705	0.278468019	0.487452284	0.571272247	0.567815111	14.4481711
11912	ZDHHC12	42.51228817	-0.176807415	0.462570527	-0.281402486	0.702902874	9.141872122
11913	ZFAS1	45.82222125	0.129022648	0.427726178	0.201671149	0.762902764	10.18147909
11914	ZFYVE26	54.26949705	0.060502129	0.29467124	0.152200096	0.878161625	14.28084174
11915	ZNF426	51.09484102	0.017602904	0.417267155	0.042188567	0.966248276	10.74971085
11916	AC009122.1	58.97256104	0.266247207	0.291942865	0.924427912	0.250078049	12.44911251
11917	AKIRIN2	59.18620004	0.060112955	0.279580626	0.158266762	0.874167802	9.415819527
11918	AL259555.1	70.02946227	-0.27822272	0.259209527	-1.052942774	0.292266762	4.497421821
11919	ANAPC4	40.45054419	0.06604214	0.484469712	0.126218409	0.891569571	7.440149414
11920	ASB6	50.40542168	0.220246614	0.420502124	0.547789406	0.582826514	11.19411212
11921	BCL10	42.90967282	0.428212986	0.426612656	0.980988524	0.226598401	12.1084119
11922	CCDC124	44.20522651	0.272602472	0.4272246	0.854488682	0.292824277	15.11477711
11923	CCDC72	46.64824445	0.579150109	0.427201948	1.255265012	0.175201291	9.880494778
11924	CCDC85C	47.86666276	0.182946822	0.4180972	0.427570099	0.66169795	10.90501129
11925	CD26	62.49277062	-0.166159924	0.271716241	-0.447007216	0.654869777	8.110111114
11926	CDK10	55.70195278	0.165869902	0.290297004	0.424874928	0.670927846	8.470549541
11927	CHST11	64.92542812	-0.472267758	0.259652206	-1.212119468	0.189142695	5.988147145
11928	CTDSP1	65.79148105	-0.081892895	0.265700022	-0.222924612	0.822808182	12.11991212
11929	DDX28	48.77827625	0.094869845	0.412277464	0.229499216	0.818480852	14.10882217
11930	DNAJC17	52.57514821	0.018628628	0.40979069	0.04545891	0.962741526	10.91914807
11931	DNPH1	47.91754881	0.454674916	0.416922092	1.090548649	0.275471526	10.944514
11932	EAPP	55.15290605	-0.066045024	0.288205571	-0.170129022	0.86490867	15.52725198
11933	ELK1	49.44202228	0.410112727	0.421552468	0.972865228		

						0.220620289	8.414998242
11934	EXOSC2	56.02989748	0.278004866	0.291697172	0.709744278	0.477862667	12.220817
11935	EXOSC4	60.02807622	0.258072672	0.290462412	0.917050296	0.259116285	12.58141542
11936	FAM98A	46.40610179	0.274522768	0.422222082	0.887006894	0.275075189	9.917154028
11937	HCCS	28.78758549	0.689258152	0.46750465	1.474548225	0.140224042	19.78442415
11938	IL18	60.44918784	-0.460597221	0.27468827	-1.229281158	0.218966409	11.14875519
11939	IL7R	40.16070126	0.202989168	0.470256164	0.644206628	0.519276612	11.47241887
11940	KCNK6	71.12212829	-0.089784927	0.268009452	-0.242974514	0.807250558	11.41901424
11941	LSM5	58.22656281	0.228468296	0.404678265	0.564567992	0.572267641	14.19715441
11942	LSM7	50.27868212	0.41025252	0.422908475	0.970212856	0.221890571	9.414104942
11943	MAP2K2	48.02126009	0.081412528	0.419129875	0.194244154	0.845984711	15.04729459
11944	MAP4K4	60.64890042	-0.518152	0.278766655	-1.268000568	0.171211898	5.475978914
11945	MAPK14	60.98122261	-0.241526726	0.275166222	-0.642785915	0.519714269	4.155477719
11946	MIR2681HG	52.45094262	-0.279429204	0.405872722	-0.688489728	0.49114442	9.49749414
11947	MTDH	65.91767486	-0.441788627	0.25717287	-1.226904209	0.216122647	8.115741748
11948	NRBF2	46.19216242	0.201099174	0.440405962	0.456622279	0.647942562	14.8109241
11949	NSD1	42.05660544	0.187854428	0.462246105	0.405420025	0.685161427	14.57778902
11950	NUB1	66.28949252	-0.277765111	0.264717127	-0.761590514	0.446204428	12.1527871
11951	NUDCD1	52.22451925	0.119121626	0.299200681	0.298250697	0.765425515	9.921120449
11952	NUSAP1	41.74825209	0.292640072	0.456796221	0.859551908	0.290026095	10.17410851
11953	ORC4	61.18461752	0.247726268	0.282199656	0.646494222	0.517959221	10.14785518
11954	PAFAH1B2	57.88282145	-0.222777788	0.280247925	-0.588504956	0.556192408	4.194425818
11955	PCYOX1L	50.27842182	-0.022187555	0.407971124	-0.05428511	0.956628241	4.970442918
11956	PLEKHM1P1	59.05150019	0.287741228	0.292116689	0.986226224	0.222972006	14.57772897
11957	PRMT5-AS1	64.29294728	0.069199024	0.274762017	0.184647445	0.85250557	14.22191115
11958	PRRC2A	71.60920221	0.12527292	0.279489721	0.256460064	0.721496055	9.422048981

11959	PTPA	62.71750551	-0.024862221	0.292142998	-0.088904411	0.929157878	17.71491494
11960	RBM22	49.5552297	0.126170015	0.40822082	0.222561222	0.728710609	8.927791712
11961	RNF128	59.27080988	0.129917242	0.291524491	0.221815574	0.740028522	8.444711545
11962	RUFY2	57.57218115	-0.159795044	0.28229192	-0.417882898	0.676022727	15.04744444
11963	SEPTIN6	68.18622815	-0.222400748	0.258590897	-0.62299615	0.522287052	8.94017515
11964	SLC25C2	70.22544145	-0.115044014	0.261588272	-0.218162925	0.750261254	12.54141817
11965	SLC25F2	40.27256445	0.552262822	0.460251751	1.20012195	0.220088099	17.07128145
11966	SLC29A12	56.62207218	0.01267547	0.28886021	0.025168087	0.971945709	18.20114147
11967	SLC9A2R1	111.0627592	-0.420129186	0.261297879	-1.162511487	0.245027761	12.11179842
11968	SNAPC2	66.27840145	-0.027212549	0.267242297	-0.101229666	0.919288768	10.41421941
11969	SPG7	55.72219625	-0.426549987	0.401041706	-1.088540122	0.276256724	15.84420811
11970	TARBP2	51.99819758	0.24420827	0.404888957	0.602149	0.546409572	12.74121257
11971	TFB1M	52.09116228	0.156598288	0.405678987	0.286015528	0.699485169	12.85154121
11972	TRMT10C	42.85878677	0.122610092	0.442477567	0.299699018	0.764406748	9.449980928
11973	WDR18	66.90255298	0.152112826	0.278847118	0.404157294	0.686097056	12.42498485
11974	YLPM1	52.20216718	0.116218045	0.299885174	0.290628541	0.771225429	14.58409481
11975	ZNF420	40.55724112	0.198648802	0.502055495	0.295671005	0.692247748	11.22175844
11976	ZNF468	45.85998954	0.217167174	0.42687845	0.742991767	0.457486665	14.02151459
11977	AC005229.1	52.72412284	0.091468192	0.297267244	0.220185527	0.8179476	12.11140719
11978	ACAD8	55.58028222	-0.270552642	0.29070275	-0.692479491	0.488626261	9.715897442
11979	ACAP2	65.50412196	-0.268710047	0.259855821	-0.746715822	0.455225111	10.44410144
11980	AHR	54.0218818	0.424055762	0.402825426	1.050096728	0.292672628	14.20574044
11981	AL129095.4	51.9866048	-0.142875878	0.404228985	-0.255829845	0.721967997	9.444157471
11982	AP001107.1	45.12922122	0.112110817	0.42882966	0.255471022	0.798259221	10.25212845
11983	APOBEC2G	47.96191698	-0.025676578	0.421155967	-0.082746228	0.924052229	11.82549048
11984	CALHM2	62.40706808	-0.426266554	0.26577627	-1.192712882		

0.222981447	11.85811229
11985	CCNT1	40.22267722	0.408672521	0.464271275	0.880055557
0.278829214	15.21077851
11986	CPSF7	50.26020527	0.248269287	0.405848888	0.858271916	0.29068712
12.84195177
11987	DPM1	65.91210221	-0.24162187	0.264211569	-0.662256099
0.507166548	9.44151494
11988	EIF1AD	67.71254225	-0.118964716	0.261975622	-0.228652944
0.742417269	11.11119857
11989	FASTK	65.20157404	-0.227902966	0.262758029	-0.654017068
0.512100809	9.188191104
11990	FBL	72.14547122	-0.027984649	0.266470021	-0.076262724
0.929120525	9.450021
11991	GET1	42.4941259	0.649090492	0.442196412	1.464566211
0.142029206	18.54900574
11992	HADH	52.70622028	-0.124012842	0.294442886	-0.214402525
0.752215221	14.1444899
11993	HECTD4	67.68522994	-0.267881269	0.256097292	-0.752269885
0.451888774	10.48844491
11994	IRAK2	50.52220849	-0.00726676	0.406917106	-0.017858085
0.985752067	8.895441749
11995	KANSL1	44.1007281	0.116277688	0.444155767	0.261794841
0.792479617	14.7942425
11996	LIPE-AS1	50.76545127	0.249622552	0.417470711	0.82750199
0.402210459	11.59428241
11997	LNPK	46.59999241	0.110949165	0.426255801	0.260226704
0.794688907	11.12275585
11998	MOV10	60.49425207	-0.242908705	0.275502688	-0.649552486
0.515981222	11.15744287
11999	MPHOSPH6	42.69851767	0.244874752	0.440858656	0.782279645
0.424050247	12.24110845
12000	MRPS26	55.86212061	0.104617225	0.404407542	0.258692565
0.795872456	15.50919974
12001	MSL2	47.56816252	0.171206522	0.419299951	0.408215152
0.682042217	15.05892841
12002	PDCD11	47.50026452	-0.098762792	0.429254285	-0.220028609
0.818069529	11.27120211
12003	PLK1	45.96146021	0.471878869	0.427916694	1.102725258
0.270142107	8.424745447
12004	POLB	41.58166642	-0.015279209	0.47919548	-0.021885225
0.974562492	8.189081487
12005	PSMA1	29.98620526	0.24592974	0.479894821	0.512486745
0.608210294	11.11947914
12006	RAB5B	66.67857152	0.44806827	0.289522885	1.150200242
0.250020221	17.80591415
12007	RANBP2	55.98522888	-0.580629949	0.402989576	-1.427229927
0.150649822	14.11190715
12008	RC2H2	52.46516925	0.078466088	0.299421084	0.196449529
0.844258225	11.15174211
12009	RCC1	29.77812692	0.559246075	0.465252248	1.201766522
0.229452999	8.470419412

12010	REL	46.92226554	0.269579468	0.422841617	0.87197541	0.282221792	9.441127524
12011	REPS1	58.12197749	-0.184740856	0.278022026	-0.488702922	0.625051219	7.424801481
12012	REV2L	57.6012222	-0.085288225	0.281881625	-0.222598925	0.822069292	12.89944109
12013	RHBDD2	55.88445295	-0.222227465	0.286679052	-0.600569025	0.548127067	11.1885015
12014	RHNO1	49.76604228	-0.108782706	0.414881299	-0.262201999	0.792165712	4.155477719
12015	RIOK2	49.05986224	0.225872594	0.411501162	0.548899042	0.582074725	14.11418441
12016	RNF111	46.8589469	0.1254222	0.425009791	0.295106612	0.767912429	14.49289418
12017	RNF26	58.76065989	-0.22987252	0.282441786	-0.627212182	0.520519502	7.907545251
12018	RPF1	51.44909872	0.052268061	0.407628778	0.128466829	0.897779547	14.19720448
12019	RTL6	28.24977796	0.466222264	0.48120054	0.968872218	0.2226084	15.52720191
12020	SMPD4	59.72226116	0.154889902	0.281967426	0.405505515	0.685105965	14.79147809
12021	SNHG17	25.22417654	0.712001115	0.50287059	1.41206245	0.157627069	9.477894795
12022	SNHG2	52.51521421	0.511092877	0.41880028	1.220275866	0.222222422	4.724141154
12023	SYNE1	42.68244702	0.219209148	0.447006775	0.714227212	0.475024807	18.18554294
12024	TACC2	59.16256625	0.061170402	0.277696719	0.161956402	0.871240186	14.24111444
12025	TAF2	61.98216628	-0.284152976	0.268628787	-0.770840429	0.440801514	15.84420811
12026	TM2D1	56.90220887	-0.254075099	0.285889961	-0.658412242	0.510272564	10.28989771
12027	TMEM127	48.60114512	0.196250478	0.412612984	0.474478524	0.625158709	11.28040041
12028	TRNAU1AP	44.42722041	0.128021228	0.449288762	0.284942199	0.775688425	11.2740884
12029	TUG1	56.20929609	-0.278878774	0.288612084	-0.717625798	0.472988046	11.95780125
12030	UBE2J1	50.52599278	-0.064486291	0.409842648	-0.157242629	0.87497402	11.17714112
12031	UCKL1	50.62269059	-0.129122271	0.422662175	-0.205520751	0.759969568	17.04197472
12032	WDR27	45.82152487	-0.02821442	0.428016174	-0.064414129	0.94864048	17.57514507
12033	ZC2H12A	48.20279615	0.204654525	0.421015402	0.722618502	0.469299979	8.940215211
12034	ZKSCAN8	56.6840992	0.164172012	0.288091722	0.422026216	0.672276061	9.110812818
12035	ZNF592	54.64056658	-0.06608821	0.292562656	-0.168250555		

0.866207504	14.54254112
12036	AL157955.1	41.74819988	0.642698281	0.449185064	1.420809778
0.15248474	12.59821759
12037	ARHGDIA	67.22875921	0.575222677	0.292842729	1.464252127
0.142124796	12.77545751
12038	ARV1	46.66762917	0.262098	0.427917684	0.614822202	0.52866488
14.55919442
12039	ASF1A	29.48809268	0.546775965	0.470451025	1.162227802
0.245128884	11.25150575
12040	BLZF1	52.86076658	-0.159604596	0.401006925	-0.298009575
0.690622128	11.44801087
12041	C1orf174	51.22662001	0.022728002	0.412945846	0.079254948
0.92682984	11.22175844
12042	C21orf91	65.97681449	-0.287579999	0.257245241	-0.804992
0.420822719	8.184197078
12043	CCDC69	52.87494221	-0.026206865	0.402952717	-0.06487591
0.948272802	11.24920909
12044	CCNB1IP1	54.58465462	0.202912582	0.29821216	0.512068919
0.608602776	10.41491814
12045	CPT1A	64.11294297	-0.142959992	0.26869446	-0.287746515	0.69820262
11.92128224
12046	DDX20	50.90228095	0.171829205	0.422790759	0.406440202
0.684419111	8.111715199
12047	DPH2	54.72089611	0.272714221	0.29585726	0.691447017
0.489284666	9.911482245
12048	DUSP11	56.79616526	-0.172290794	0.292422866	-0.441824422
0.658609021	8.474140154
12049	ECHDC2	49.02872242	0.129104072	0.41491599	0.225258402
0.727420169	15.10114442
12050	ENTPD4	49.99962776	0.051428206	0.410845924	0.125176622
0.900282725	10.41092424
12051	ERV2-1	45.59799721	0.208499469	0.422605286	0.481962259
0.629822757	11.4105102
12052	FANCF	47.61029128	0.220260948	0.418529002	0.550154098
0.582212684	12.29581972
12053	FTO	56.81854777	-0.421098989	0.290774961	-1.102189897
0.269944708	9.101217778
12054	GGH	52.0884259	0.579419762	0.41111725	1.409278022
0.158722416	11.10247049
12055	GGT1	70.7190728	-0.442122776	0.258690654	-1.225292591
0.216684429	12.94281142
12056	GLG1	61.07422722	-0.007618817	0.274771625	-0.020229226
0.982780742	10.45142748
12057	GLTP	54.87121658	-0.104166246	0.292802945	-0.264512957
0.791284682	11.84279409
12058	GPN2	75.45894821	-0.076244692	0.258524427	-0.212656527	0.82159487
11.40591818
12059	GTDC1	64.69044798	-0.416852888	0.259726912	-1.158771518	0.24654922
14.8197815
12060	HSDL2	64.47680898	-0.127202768	0.267008974	-0.272842087
0.708521061	10.40401428

12061	KLHL7	54.19015418	-0.201989942	0.295952644	-0.510126617	0.609955752	12.0888744
12062	MBP	55.12728566	-0.19566264	0.290627456	-0.500892209	0.616446282	12.24190549
12063	MLXIP	55.42852004	-0.182559202	0.289697865	-0.471029522	0.627619646	7.198419844
12064	MUTYH	42.29429681	0.266922129	0.445960707	0.822767842	0.410640027	10.87109724
12065	NUCKS1	59.77085851	-0.421498065	0.276850252	-1.145011702	0.25220421	12.1240299
12066	PCGF5	41.86887218	0.211297822	0.468560244	0.664584285	0.506216259	11.12192715
12067	PLEKHJ1	64.79500528	-0.060876152	0.264225421	-0.167092792	0.867297028	15.24594281
12068	PLPP5	70.57248682	-0.45992102	0.24707577	-1.225121455	0.185127555	18.11442142
12069	PPAT	22.82052417	0.224707445	0.602421171	0.272001027	0.70914768	12.74414419
12070	PRR14L	62.66094661	-0.055024495	0.274004401	-0.147149224	0.882014159	10.1781904
12071	PTRH2	51.72615529	0.064751204	0.401562919	0.161247966	0.871898104	14.5110424
12072	SETDB1	51.72894057	0.008906198	0.401224277	0.022191471	0.982295221	8.447724114
12073	SMARCC1	50.46286529	-0.118791286	0.412541096	-0.287950188	0.772284869	7.710418041
12074	SPIN4	50.1208468	0.074797651	0.421221017	0.177572408	0.85905801	10.15074059
12075	STAU2	28.2022481	0.697989287	0.475799119	1.466982142	0.142280641	17.00441998
12076	TMEM14B	64.87896926	-0.256704494	0.266072516	-0.97440672	0.229854624	14.19142278
12077	TMEM216	60.62412614	0.027922941	0.272804629	0.101477985	0.919171022	10.44151201
12078	UBE2D4	65.47860277	-0.256898988	0.258227525	-0.99629126	0.2191086	11.40218204
12079	UBR5	50.54225879	-0.282702212	0.44777152	-0.856917292	0.29149054	17.50744704
12080	UBTF	60.8424859	-0.128820258	0.270001409	-0.275215484	0.707500215	14.80849017
12081	VPS8	56.16862411	-0.222952157	0.295229922	-0.816899915	0.412985626	11.44147502
12082	WASHC2	68.14664115	-0.20719571	0.254229112	-0.866978466	0.285952809	7.152144545
12083	ZCRB1	52.52898926	-0.022922028	0.400260221	-0.082222492	0.92446104	17.54417428
12084	ZFAND5	82.21090098	-0.224674722	0.249797784	-0.928178256	0.25221506	12.84210544
12085	AC009088.2	74.55777908	-0.225022499	0.242917268	-0.974165688	0.229974278	17.51587198
12086	AC092512.1	49.02712726	0.197922102	0.416452787	0.475255861		

0.624604616	11.11281812
12087	AL291650.1	49.24129711	0.415945947	0.412452497	1.006020802
0.214400752	11.90412001
12088	ARCN1	72.54727901	-0.42941821	0.241686781	-1.286026269
0.198422859	12.27044179
12089	BCL9	46.62520814	0.227941225	0.424127727	0.772212578
0.429295992	10.42111744
12090	CKS2	71.56651948	-0.165211457	0.266859288	-0.450612708
0.652268706	11.44148414
12091	CLEC12A	78.74825556	-0.42558987	0.22682929	-1.292206626
0.195929605	11.24951171
12092	DGKA	72.84866258	-0.250922201	0.247570242	-0.721922129	0.4702262
10.47989081
12093	DNAJC16	51.26488228	0.285260405	0.402955812	0.7064149
0.479920158	11.12454112
12094	DTX2L	56.78182727	-0.402648268	0.292248158	-1.026446927
0.204680962	14.59440084
12095	ERG28	62.78798066	-0.240070672	0.266157228	-0.928755829
0.252015625	14.01417228
12096	FNBP1	60.8402491	-0.474775414	0.28584704	-1.22047572
0.218519014	18.21148274
12097	GCNT1	47.55666881	0.081191754	0.425070122	0.191007901
0.848519402	10.70141241
12098	GGPS1	57.16782178	-0.088245056	0.281047448	-0.221847914
0.816656141	8.909529279
12099	GLYR1	62.57927116	0.15211972	0.276946527	0.40255785
0.686527887	9.105102187
12100	HSPB11	52.20517115	0.027820612	0.296945706	0.095279058
0.924092176	15.55982551
12101	KATNBL1	56.72229151	0.162004598	0.282276652	0.425292272
0.670622627	9.418454884
12102	KHDRBS1	64.66786426	-0.247871161	0.262225152	-0.960081178
0.22701426	18.49778111
12103	KSR1	52.72208012	0.062719122	0.292024281	0.162125112
0.871207221	15.19524051
12104	LILRA2	68.64480174	-0.19874821	0.258622586	-0.554198809
0.579442792	8.715497498
12105	MIA2	61.57801822	-0.054185504	0.269674129	-0.146576298
0.882466284	14.74221487
12106	MMUT	62.00549657	-0.255162197	0.267440189	-0.966587782
0.222750145	11.44780814
12107	MSANTD4	49.52126228	0.214117772	0.42280574	0.741182286
0.458582256	8.429192548
12108	MTR	66.49755774	-0.122090482	0.258719992	-0.271014958
0.710626291	10.49250159
12109	PAAF1	46.74209602	-0.084250282	0.444108402	-0.18970657
0.849529072	8.701270814
12110	PACC1	60.4826544	0.285225422	0.279271782	0.752224414	0.45185599
11.9049152
12111	PIGN	50.86682074	0.460722882	0.404626257	1.128612454
0.254864822	11.88714811

12112	PML	56.65857902	0.191174717	0.294821494	0.484205444	0.628240074	17.17474591
12113	POLR2B	27.86144174	0.622172082	0.502417525	1.227885161	0.215758645	7.944154472
12114	PRMT6	55.06012244	0.124659228	0.295226826	0.215224089	0.752515597	14.51849704
12115	RAC1	57.52407888	0.196294425	0.285994092	0.508801622	0.610891275	10.17211211
12116	RAD9A	59.08994521	-0.179811121	0.275127278	-0.479220882	0.621710269	10.12271851
12117	RILPL2	67.18299047	-0.40020142	0.252540122	-1.121982029	0.257641569	8.108819791
12118	RIPK2	61.95262128	0.069780255	0.27526826	0.185898122	0.852524655	14.58240944
12119	RNF185	67.71692229	0.22218807	0.280655226	0.872674169	0.282840701	14.5444977
12120	RNPEPL1	57.62922419	-0.111062724	0.279566269	-0.292606928	0.76982261	12.58708422
12121	SEL1L2	28.52265672	0.481604182	0.485291482	0.992401887	0.221001519	29.04471257
12122	SLC25A25	62.62766695	-0.421889746	0.26882828	-1.14282295	0.252692507	25.4172195
12123	SLC22A1	48.62775217	0.42494607	0.412747255	1.052782814	0.291982264	19.91472751
12124	SMAD5	50.08140118	0.25012299	0.406627271	0.615142024	0.528460271	4.194018552
12125	SPTY2D1	50.44958745	-0.290402028	0.425211002	-0.68280161	0.494722212	5.525119742
12126	SYNC	48.68002207	0.422809072	0.415622794	1.041250664	0.297712821	9.771041449
12127	TBCK	55.28126442	0.557402274	0.297021275	1.402960969	0.160220472	29.24270402
12128	TCEA1	62.0988127	0.06172005	0.271909651	0.165954419	0.868192821	8.01757111
12129	TEX10	46.8729712	0.210840777	0.422706055	0.725259177	0.462120777	11.1724712
12130	TFDP2	62.74779492	-0.150857674	0.265540786	-0.412697242	0.679828446	14.29114854
12131	TRPM7	57.55029502	-0.122195917	0.2896665	-0.241820292	0.722486127	18.7101124
12132	WRAP72	49.21015826	0.181242219	0.411665226	0.440266282	0.659744258	17.11145199
12133	AC025040.1	51.59562241	0.412640044	0.401446418	1.027882229	0.204004755	9.48285284
12134	BRAP	65.52227196	0.017775005	0.265428011	0.048641606	0.961204912	9.981015507
12135	C8orf22	66.01919226	0.061871797	0.269411952	0.167487262	0.866986669	9.981015507
12136	CAD	55.4254822	-0.2615879	0.296581419	-0.65960705	0.509506029	14.05295798
12137	CARD9	68.42160729	0.255722021	0.272272886	0.684899816		

0.492407142	17.19757528
12138	CDC27L1	45.11092457	0.178425802	0.441529912	0.404098922
0.686129977	18.00149118
12139	CENPX	47.60750272	0.414751942	0.422068259	0.980242851
0.226916909	9.480420944
12140	CTNND1	61.2298722	-0.222204027	0.272074696	-0.868652626
0.285026608	11.14114197
12141	DCK	45.48204622	-0.197222985	0.471429796	-0.418556062
0.675540606	8.421072879
12142	DDX19A	49.66501294	0.45501246	0.409848419	1.110196928
0.266914172	10.41809991
12143	DIPK2A	27.6695282	0.284476964	0.526266401	0.54045426
0.588882797	8.44772214
12144	FASTKD1	45.66559292	0.4292222	0.429285718	0.999875222
0.217270842	15.07194409
12145	FBXL12	62.06926652	-0.274225792	0.271470004	-1.007714722
0.212591425	14.90419014
12146	FBXL2	62.15114102	-0.220612516	0.286865582	-0.85459522
0.292775219	18.45548855
12147	GATAD2A	59.07292206	-0.202526482	0.276587592	-0.54044925
0.588887251	10.90497014
12148	GNPNAT1	42.69059941	0.217525522	0.462178225	0.470652892
0.62788862	14.81484495
12149	ILKAP	61.27091192	-0.27662914	0.269870795	-0.747907492
0.454515957	11.28129211
12150	LRTOMT	51.09458524	0.202096204	0.402524665	0.750485187
0.452962542	11.42894987
12151	MED16	64.12010679	-0.418011604	0.268262251	-1.124782722
0.256465944	10.19110178
12152	MEG2	59.95126728	-0.024292698	0.277220989	-0.064664211
0.948441259	15.14044198
12153	MRPL27	58.26082102	-0.24496022	0.285197871	-0.89554021
0.270498228	14.0597188
12154	MTX2	65.87588625	0.156979275	0.266991414	0.427746451
0.668825727	14.21425298
12155	NABP2	57.90241427	0.075629126	0.28126429	0.198262992
0.842760288	11.88422157
12156	NCAPH2	49.2891465	0.442616512	0.409652679	1.082906211
0.278850055	14.5151491
12157	NDUFS6	57.29211146	0.278196154	0.405582021	0.685916666	0.49276566
7.719011904
12158	PCF11	44.42279764	0.291722257	0.442764441	0.658888407
0.509967421	10.48147502
12159	PGPEP1	52.94800927	-0.108991288	0.295871608	-0.27521979
0.782070561	15.04191544
12160	POLR2C	51.27617252	0.456125798	0.402211816	1.124068616
0.256765767	15.07579944
12161	POLR2H	47.75916559	0.098494677	0.422289656	0.22726587	0.82021701
11.41427219
12162	PSMC1	50.57274089	0.076822888	0.410744205	0.187025796
0.851622567	15.11285015

12163	PTPN11	49.5519495	-0.095667216	0.425586982	-0.224789104	0.822142242	10.87109418
12164	RNF28	47.20024825	-0.116142882	0.442005814	-0.262765509	0.792721222	12.29577859
12165	RPF2	61.42068469	0.259928618	0.277651212	0.688202414	0.491261722	14.20570921
12166	RPS6KC1	52.85484242	-0.247890262	0.409182174	-0.850208745	0.295209041	15.41191149
12167	SKIV2L	61.86786018	-0.090077794	0.267528588	-0.245082908	0.806291462	4.118917171
12168	SLC25A40	29.50762759	0.508455275	0.476285282	1.067219654	0.285827516	15.57479445
12169	SUPV2L1	54.48576577	0.102482981	0.289814871	0.262901671	0.792626268	9.901914149
12170	SYS1	47.12744522	0.262499092	0.422897095	0.619251926	0.525750409	11.51991104
12171	TCAIM	52.8122094	0.087018712	0.299216111	0.217919265	0.827491929	12.27874451
12172	TERF2IP	56.7541287	-0.117827762	0.296927007	-0.296849952	0.766581067	4.947577445
12173	TIMM8B	66.6696117	0.12122627	0.270048709	0.22789297	0.74299259	10.92018812
12174	TOR2A	52.54111975	0.241029784	0.297095285	0.606982226	0.542862701	8.711540952
12175	TRMT1	55.74297906	0.29504092	0.289817604	0.75686918	0.449128241	11.9785881
12176	TSC2	42.96557447	0.160965481	0.466229959	0.245249116	0.729907097	11.27771292
12177	TXNDC15	71.49026454	-0.122218915	0.252759529	-0.246748721	0.728780105	17.58420501
12178	UQCC2	59.22064528	-0.141522227	0.27644528	-0.275970252	0.706929011	8.415452147
12179	VPS26	62.00524521	-0.449892727	0.271485421	-1.211064217	0.225870756	7.485484414
12180	VPS72	62.92840498	0.250102291	0.272227185	0.671727452	0.501757222	15.24011184
12181	ZNF280D	56.87211682	-0.150267294	0.284051612	-0.29152887	0.695406254	15.24898071
12182	ACKR2	56.02521488	0.176182929	0.285500295	0.457024108	0.647652717	14.07444074
12183	AGK	50.9541126	0.602120912	0.408455077	1.476590565	0.129785422	14.14414477
12184	AL590122.2	94.672019	-0.404682995	0.22762169	-1.198596007	0.220685072	8.422809101
12185	AMZ2	66.15921152	-0.405459009	0.256859722	-1.126185966	0.255878742	14.59171428
12186	ANKRD11	54.5421212	0.206404155	0.28994214	0.529218594	0.596584459	8.441245104
12187	BCAS2	45.04884624	0.214162767	0.460174827	0.465296528	0.64164752	14.40877451
12188	COQ6	49.28058922	0.414480282	0.410152628	1.010551228		

0.212221221	12.11144727
12189	CRNKL1 48.8146797	0.242562046	0.418057616	0.580214292
0.561770049	11.9701812
12190	CSTA	48.64022506	0.214841492	0.415690767	0.757292517
0.448814129	11.40448824
12191	CWF19L1	59.72469171	0.08208958	0.277827822	0.219908052
0.825942765	14.41708121
12192	DIS2	61.68500842	0.012597825	0.271472872	0.022912175
0.972946287	14.04294955
12193	ELP1	41.9418881	0.272298207	0.468182886	0.582741277
0.559294224	14.54471287
12194	GALNT2 54.22280246	0.022546461	0.408559249	0.079661544
0.926506445	15.24882052
12195	GOLIM4 68.18886776	-0.052012152	0.262895419	-0.145682284
0.884172129	14.41857289
12196	GOSR1	62.01862959	-0.256670622	0.262880088	-0.705271442
0.480579112	12.87120755
12197	IFRD1	61.69898491	-0.151790229	0.282480228	-0.295822002
0.692225606	11.88914095
12198	IMPA1	51.00126825	0.107924208	0.409214292	0.262695186
0.792014806	9.188190021
12199	INPPL1 61.71052655	0.22225582	0.272557908	0.596825592
0.550617152	9.449229419
12200	LRMDA	62.9997284	0.26700174	0.276505726	0.974757265	0.22968062
11.27477149
12201	LSM2	62.40182871	0.070708652	0.266260267	0.192055754
0.846915204	14.58719429
12202	LUZP1	65.00575575	-0.092002594	0.262260189	-0.252900944
0.799572086	14.22474795
12203	MAK16	47.66270897	0.258855985	0.426428267	0.607022895
0.542829082	10.41228204
12204	MED22	67.21654875	-0.079619452	0.257298929	-0.222774725
0.822710822	15.19809444
12205	MITD1	54.69104784	0.064600527	0.292722262	0.164492762
0.869242457	12.24777795
12206	MRPL50 51.10592565	0.561210185	0.409921682	1.269066847	0.17097828
11.88422157
12207	MT-TS1 66.20179166	-0.142726568	0.261945862	-0.29422126
0.692226487	11.15544877
12208	OBI1	44.25278122	0.472148858	0.445750789	1.059221587
0.289498876	10.14780404
12209	PDHX	51.05482925	-0.02572749	0.415622	-0.085959421
0.921498667	14.04440101
12210	PPP1R15B	46.80497089	-0.02497026	0.442570825	-0.056420925
0.955006479	12.21514512
12211	PQBP1	65.77416222	-0.058477949	0.260012252	-0.162422768
0.870965068	7.711858151
12212	PSEN2	70.29258156	-0.428789152	0.250529742	-1.222225504
0.221244525	11.14218594
12213	RAB2GAP2	51.4459587	0.081511282	0.416758796	0.195582821
0.844925917	10.144714

12214	RALGDS	52.9404427	0.079862968	0.405425818	0.196987869	0.842827021	11.88714491
12215	RDX	54.68244168	-0.172549886	0.296259266	-0.425226972	0.662217852	14.11811407
12216	RMND5A	68.09898278	-0.122672212	0.262224222	-0.265255424	0.714920742	11.40190204
12217	SCYL2	56.4246299	-0.102262756	0.285412289	-0.26559287	0.790552777	15.12781442
12218	SDCCAG8	66.08672899	0.027927794	0.260858824	0.105104241	0.916292094	15.00142085
12219	SETD2	54.17855926	-0.208264152	0.408600748	-0.754428542	0.450585925	11.87498411
12220	SLC4A2	54.20984819	0.144118528	0.295554992	0.264245127	0.715600298	14.21144857
12221	SNRPA1	61.70490591	0.24606924	0.27202562	0.659657729	0.509472499	17.14841128
12222	SPTAN1	70.62022292	-0.256497762	0.245899177	-1.020640677	0.202709252	14.24141844
12223	TIMM10	49.19782876	0.57902589	0.412162826	1.40486829	0.160060447	4.941159448
12224	TIMM17B	62.27748295	-0.282855068	0.266852614	-1.042620922	0.296660804	11.28900515
12225	TNFRSF1A	65.8954824	0.090727182	0.264690951	0.248805686	0.802511092	9.17404224
12226	UBE2A	62.09781905	0.107126079	0.269899052	0.289608956	0.772115414	10.88517189
12227	WDR20	62.56558216	-0.227161441	0.265564755	-0.648752572	0.516497671	12.52057854
12228	AC006441.2	67.72999928	-0.161275401	0.252980627	-0.456895886	0.647745881	11.19779871
12229	ADCY2	74.01698287	-0.454967886	0.229290772	-1.240927991	0.179940586	19.14577184
12230	AKR7A2	78.16856142	-0.452426469	0.252202618	-1.282752749	0.199228121	18.81044422
12231	ANKRD26BP1	52.05524511	-0.254229745	0.42166628	-0.589204422	0.555724144	12.10725724
12232	ARHGAP27	76.68014761	0.020160582	0.252952072	0.085452242	0.921901781	11.11749594
12233	ATXN2	64.80574172	-0.229012764	0.264791119	-0.901922722	0.267097289	14.79988192
12234	BLOC1S2	65.51222212	-0.179444177	0.257022826	-0.502598526	0.615246566	12.44880949
12235	BRCC2	60.29028499	0.145708708	0.275842728	0.28768522	0.698248921	15.88270151
12236	BRWD1	52.70944112	-0.180545555	0.411167225	-0.429104929	0.660585508	11.89194412
12237	CARD19	60.10944278	0.552591865	0.287095248	1.427524245	0.152425962	8.714487415
12238	CATSPER2P1	52.50058142	0.157890274	0.295524876	0.299191766	0.68975191	19.4491144
12239	COMMD2	55.04291689	0.047254856	0.295792544	0.119645248		

0.904764095 11.15148212
12240 DDX50 71.25284027 -0.072205829 0.250648542 -0.205920821
0.826852752 11.24189851
12241 DHX29 52.72406786 0.010270484 0.296252924 0.025919012
0.979221926 15.04450811
12242 DIMT1 55.66190204 0.02922262 0.291609454 0.10018205
0.920199002 14.20440447
12243 DMAP1 56.65255468 -0.011692776 0.285657266 -0.020221679
0.975810507 14.41712128
12244 DNAJC2 52.50625212 0.211719518 0.295972298 0.524681167
0.592870247 11.5298158
12245 EMD 66.09504271 -0.049422274 0.258899554 -0.12770502
0.890472544 10.4404215
12246 EP200 54.95845722 0.208420224 0.295525574 0.526970209
0.598214207 18.79451744
12247 GDF9 65.20957409 -0.281779779 0.262186292 -0.777996594
0.426571021 11.88711484
12248 GGA2 28.04940921 0.027112862 0.58559861 0.046299298
0.962071619 7.492991454
12249 GMEB1 51.44202216 0.025841582 0.417651416 0.061872568
0.950662517 11.45424544
12250 GOLPH2L 58.29094151 0.2068505 0.282810218 0.540247285
0.588957486 11.87841081
12251 GPATCH4 59.59267422 0.088627087 0.284942478 0.220260004
0.817889728 17.51999857
12252 HEXIM1 48.52185017 0.291080582 0.427512841 0.665207518
0.505852822 9.174848445
12253 HNRNPCP2 52.24162694 0.145272224 0.297797527 0.265445292
0.714779027 11.17122044
12254 HSPB1 62.5295687 0.229022606 0.280452625 0.601975076
0.547190728 17.74049971
12255 IL17RA 56.22905292 0.128612496 0.282688242 0.261262241
0.717902595 4.708411959
12256 INTS6 60.68206228 0.155470825 0.271610822 0.418270041
0.675676587 12.2151952
12257 IPO7 60.45792281 0.142158181 0.272225451 0.2845904
0.700540908 11.2541299
12258 MRPL19 50.92580912 0.545998275 0.405117212 1.247752821
0.177727572 14.11412524
12259 NME6 55.62628292 -0.192227276 0.29420245 -0.490171447
0.624012582 11.71499901
12260 PAM 74.85025289 -0.282454245 0.228492586 -1.122825722
0.257287442 14.49980114
12261 RAB2A 67.77202896 -0.486468282 0.268740152 -1.219271522
0.187078255 10.47921278
12262 RARS2 64.54420221 -0.026901208 0.262697772 -0.074169765
0.940875204 14.11412524
12263 RBM7 52.60220226 0.297892778 0.292829008 1.010292962
0.212254952 8.429479794
12264 RFXANK 62.61940261 0.245825168 0.278806082 0.648972655
0.516255405 12.24185454

12265 RMC1 68.2282111 -0.096400562 0.25448108 -0.271948402 0.785661694 9.729911917
12266 RPL26P19 48.6092452 0.216612 0.416779244 0.759665768 0.447454296 19.11190481
12267 RPS15P4 52.82807512 0.225064572 0.29760254 0.566052727 0.571257921 11.25794441
12268 SAT2 52.22755022 0.172122824 0.401046958 0.421704618 0.665956111 14.41180207
12269 SH2D2C 69.97876518 -0.165877154 0.249102525 -0.475152105 0.62467785 7.142492949
12270 SLC28A10 72.84242048 -0.495778408 0.241222796 -1.452899487 0.146251662 7.710544914
12271 SYMPK 62.09845905 -0.076982954 0.265778082 -0.21046255 0.822205897 11.24194857
12272 TMEM120A 64.72247227 -0.204170092 0.259549945 -0.84597452 0.297566959 10.12924807
12273 TMEM79 61.52249927 -0.287262709 0.270208425 -0.775948597 0.427779227 11.44102195
12274 UHMK1 55.40846807 -0.155814475 0.292504702 -0.29596598 0.692120125 19.50184788
12275 VPS18 89.80624504 -0.446640827 0.255047879 -1.257972529 0.208401226 15.48925114
12276 ZBTB20 69.22294026 -0.157525018 0.250224952 -0.449769551 0.652876615 11.8777495
12277 ZCCHC7 55.25847819 0.42526781 0.2904057 1.089297125 0.276022876 7.140405904
12278 ZNF294 55.41418882 -0.02578226 0.291249917 -0.065880225 0.947472105 11.88714491
12279 AAAS 69.26959854 -0.022416281 0.252115064 -0.094901879 0.924292764 11.89472151
12280 AC011558.1 70.01522212 -0.291262745 0.252618256 -0.822667161 0.410128702 12.88144894
12281 AC016876.2 62.27148907 0.026821625 0.265527607 0.07227582 0.941507051 9.458285781
12282 ADGRG2 70.09446265 0.098494882 0.256852922 0.276008958 0.782541186 18.77109944
12283 ADNP 72.89247415 -0.19261895 0.242521822 -0.562228828 0.572885188 9.181449177
12284 ADORA2B 49.52941269 0.272727769 0.422858228 0.86142812 0.289002289 10.70804197
12285 AL512656.2 57.66295255 0.088602465 0.282550706 0.221609728 0.816841151 14.25811782
12286 ANKMY1 52.84404456 0.220712926 0.29745025 0.555222147 0.578674222 10.49112101
12287 BCOR 52.82941544 0.47286209 0.29881105 1.188186961 0.224759759 11.58194701
12288 BCS1L 62.79608671 0.204156924 0.280772211 0.79878971 0.424412258 17.7071185
12289 BPTF 68.0821688 0.207459751 0.262664155 0.570470717 0.568258478 14.15102798
12290 CEP250 59.42897569 0.146207785 0.274247659 0.290671208

						0.696040284	11.59711274
12291	COQ5	62.2566212	0.141002092	0.268625962	0.282507224	0.702085172	9.410425195
12292	CSTF2	55.82285589	0.187850909	0.294078502	0.47668298	0.622587172	8.704155027
12293	ELP5	66.87195615	0.155558969	0.260681879	0.421291225	0.66625655	15.11125857
12294	EXOSC1	72.9084922	-0.116569792	0.247201108	-0.225741421	0.727065867	14.78849172
12295	FLI1	59.64709418	0.014272622	0.272270686	0.028226682	0.969498974	11.10924101
12296	GABPB1	65.17775858	0.212820021	0.264882981	0.582282976	0.559702821	17.59111589
12297	GOLGA2	64.45142075	-0.088206845	0.279190276	-0.222882672	0.815852514	17.82942887
12298	GTPBP1	56.22212085	-0.169016165	0.299702669	-0.422852676	0.672402022	11.12541124
12299	HACD4	52.80250587	0.024842106	0.405726114	0.085872809	0.921566722	11.25441807
12300	HDAC6	56.58465547	-0.264222625	0.296200265	-0.66714282	0.504680272	19.44848448
12301	HDAC8	62.44754281	-0.045190477	0.274252525	-0.120748201	0.9028904	11.41044444
12302	INTS7	61.67908527	-0.150675559	0.277750982	-0.298875261	0.689985045	12.05111414
12303	JAGN1	56.22649704	0.267461966	0.285450521	0.952221091	0.240422242	11.90875807
12304	KDELR1	78.77072267	-0.24972688	0.228227792	-0.728150652	0.460422906	10.45475918
12305	MIEN1	65.40262884	-0.107120479	0.268654461	-0.290598622	0.771258212	11.1101524
12306	NCOA6	57.01009442	0.022460229	0.287679222	0.057925252	0.952800122	14.10418751
12307	NHLRC2	45.80008921	0.100921285	0.456797054	0.220954545	0.825127826	19.74771575
12308	NUP85	70.15265449	0.096511442	0.264870614	0.264508672	0.791287982	11.88149412
12309	OTUD6B-AS1	56.98204122	0.226246425	0.282571675	0.591644495	0.554088672	12.29194215
12310	PAK1IP1	67.88245622	0.259821866	0.262979545	0.712826451	0.475228217	15.21117117
12311	POFUT1	52.0124597	-0.086064518	0.408982715	-0.210425587	0.82222772	10.89441414
12312	PRIM2	76.27420458	-0.254170822	0.226690612	-1.051917752	0.292827286	18.59404984
12313	RESF1	57.29529291	-0.149144176	0.28485402	-0.287524402	0.698260622	11.89189417
12314	RNF24	66.82952608	-0.027214452	0.256072452	-0.104512708	0.91676169	10.40470847
12315	SLC7A6OS	61.9276442	-0.164268062	0.290005172	-0.421194571	0.672612008	14.87041417

12316	SMG5	62.2942427	-0.221622442	0.262277499	-0.610062774	0.541819562	10.41455589
12317	SMIM20	61.60591694	0.015807697	0.268691541	0.042875128	0.965801075	17.55524451
12318	SYAP1	65.61657821	0.015481144	0.259429061	0.04207029	0.965645502	12.77157102
12319	TAOK1	42.15746892	0.150805959	0.472260947	0.218585552	0.75004081	15.78780025
12320	TBL2	60.02169714	0.141965271	0.280128112	0.27246691	0.708800969	8.498424181
12321	TDG	57.21426428	0.20942441	0.287022449	0.541128814	0.588418799	15.20928599
12322	TELO2	57.27281025	-0.198770675	0.286969517	-0.512659774	0.60748988	19.15801004
12323	TK1	65.22844429	0.144299624	0.2728612	0.287274268	0.698552099	18.47542585
12324	TMEM104	64.98575599	-0.224512016	0.259217752	-0.624828622	0.522082542	15.84114892
12325	TMEM209	51.01524259	0.192759976	0.412427047	0.46727957	0.640228227	11.94144745
12326	TMEM245	47.22004227	0.280740198	0.426054822	0.642818569	0.519692091	11.1905825
12327	TOP1	52.05892528	0.515025804	0.407222207	1.264722669	0.205970761	14.11112444
12328	TRAPPC2	48.22198622	0.245070766	0.425426955	0.576058288	0.564575716	11.22145715
12329	TRIM25	50.61202202	0.218641207	0.410829276	0.52219492	0.594591001	11.05944747
12330	UBL7	69.01262959	0.165142729	0.267497548	0.449272717	0.652162088	12.2702404
12331	UPP1	60.00204996	0.262429554	0.274565014	0.702221204	0.481855527	9.107927224
12332	ABCC5	49.11277514	0.126666546	0.425716981	0.221026768	0.748190106	14.05794404
12333	ACTR1B	67.78048279	-0.269522825	0.25504222	-0.759129621	0.447775021	11.74195507
12334	ADK	52.47779628	0.051292255	0.400658212	0.128272062	0.897922672	8.727978951
12335	ARF2	71.64808826	0.125477665	0.262672424	0.272552799	0.708726212	14.55111977
12336	FAM118B	58.71269685	0.011989798	0.278246129	0.021698401	0.97471257	15.14447228
12337	FOXN2	61.07079457	-0.214529008	0.276429819	-0.825525965	0.402416006	14.25928044
12338	G6PC2	62.54552226	-0.229095419	0.270487668	-0.88827621	0.27429214	14.7717812
12339	GLIPR1	55.22290786	0.272782605	0.287626161	0.964264025	0.224912572	18.19944952
12340	GPN1	62.42416782	0.115994089	0.26426124	0.218426529	0.750152826	10.45710497
12341	IQCB1	52.97291296	-0.084212698	0.408928026	-0.205927702		

		0.826829575	14.59191715				
12342	KANSL2	61.97191266	0.04677922	0.27241892	0.125272027	0.900207405	12.40289709
12343	KDM2B	42.46158762	0.466260927	0.452219261	1.028769068	0.202588205	8.709475545
12344	LRRC57	52.17641182	-0.204154967	0.415882548	-0.49089455	0.622501022	10.24159212
12345	LSM1	62.22460544	0.278747466	0.271012868	1.020844499	0.207228118	19.04109177
12346	MRPS18A	61.88725288	0.049826786	0.266591422	0.125919122	0.891885218	14.09074742
12347	NFU1	52.28254108	0.285012892	0.297127162	0.969495225	0.222298156	9.842481412
12348	PIGF	61.25981162	-0.082924989	0.29419088	-0.212929811	0.82128171	11.12488014
12349	POP5	70.77242822	-0.052665611	0.248818456	-0.150982869	0.87998922	19.55441909
12350	RGS12	72.75026229	-0.472562214	0.244665402	-1.27107557	0.1702514	11.44118905
12351	RNF167	70.5841172	0.129227162	0.255244499	0.292090285	0.694991421	19.48200045
12352	RPS6KB2	51.84284072	0.200929022	0.405422126	0.49559226	0.620182115	9.411774581
12353	RTL8C	72.76966406	-0.285506987	0.256058687	-0.801852722	0.422627572	14.5417784
12354	SCAND2P	54.22655668	0.224112452	0.299458269	0.56104247	0.574767904	14.40489244
12355	SDF4	41.78420189	0.257647416	0.489719084	0.720211271	0.465199879	12.44229149
12356	SERTAD1	68.07908207	-0.025458777	0.252857856	-0.071946227	0.942644614	12.10004471
12357	SLC20A2	45.91558652	0.126791907	0.456170917	0.277948248	0.781052086	14.74714147
12358	SMARCAD1	60.48522777	-0.228522145	0.284097552	-0.88124679	0.278120148	11.91521014
12359	TAGAP	54.28221725	-0.212789057	0.421890086	-0.726548414	0.467502629	10.51891851
12360	TASOR	56.19272529	0.062846222	0.287224471	0.16488171	0.869027088	11.81498257
12361	TMED7	46.57675144	-0.071882194	0.462950295	-0.155271949	0.876606929	14.54918191
12362	TRAF2	48.82122812	0.242516228	0.422294875	0.574146024	0.565868991	11.92118114
12363	TXLNGY	52.25828644	0.009666546	0.402690652	0.024004892	0.980848707	14.75202499
12364	U47924.2	57.45726897	-0.07576792	0.285764928	-0.196409582	0.844289597	8.419445047
12365	VPS16	57.29877292	0.256600976	0.409275664	0.626810528	0.520782441	14.21017714
12366	WBP11	71.26296619	0.271142502	0.267042826	1.011169419	0.21192525	10.44187747

12367	WDR75	69.12250617	0.202455276	0.258897119	0.566890246	0.570788752	12.14119104
12368	ZBTB17	64.89825966	-0.266219267	0.271852096	-0.71592618	0.474026922	11.15451721
12369	ZNF148	52.45799902	-0.055646722	0.404752181	-0.127482126	0.89064892	17.05158411
12370	AP4B1	57.98099762	0.198202265	0.278471671	0.522692792	0.600491522	17.55810184
12371	ARHGEF7	45.22425795	0.196869702	0.464968201	0.422404568	0.672000112	11.11428007
12372	ATP9B	52.06981406	0.156917902	0.401508289	0.290820982	0.695929565	12.11701474
12373	C12orf57	74.81687227	-0.011662662	0.252589122	-0.022986487	0.972685264	12.5812445
12374	C22orf29	57.56754592	0.424294202	0.281171809	1.112121282	0.265652007	17.08540811
12375	CASP8	61.82208507	0.421422585	0.277105726	1.144065225	0.252596547	12.10857074
12376	CCDC42	52.67526825	0.450067497	0.401708668	1.120282827	0.262550656	11.10279145
12377	CKAP2	59.45001456	0.049096074	0.274420818	0.121125284	0.895676121	14.1048941
12378	COA1	61.92798755	0.024925219	0.26866271	0.067609907	0.946096169	7.8027218
12379	COMMD9	86.66222886	-0.222229699	0.224581082	-0.664501705	0.506269258	5.799742744
12380	DNAJC5	70.18294498	-0.111901256	0.250182745	-0.219551212	0.749208488	24.1917401
12381	DPH5	50.91127872	0.220107201	0.421227877	0.546277228	0.58487528	7.550297944
12382	DRG2	70.66857578	0.116222221	0.262599985	0.219672227	0.749215974	19.8925154
12383	GALNT11	69.7227229	-0.072952526	0.249048492	-0.209004012	0.824445106	21.10454087
12384	GOSR2	62.28561259	0.204959299	0.280751968	0.800929522	0.422166659	18.49185904
12385	ISOC1	79.22798491	-0.176129222	0.225952045	-0.524297172	0.600071864	17.12910092
12386	KLHL28	57.71248702	0.027074267	0.281092889	0.097284221	0.922500599	12.41824049
12387	LDAH	49.56865584	0.28289404	0.420708222	0.674800212	0.499802642	15.19440485
12388	MAP2K8	45.6708077	0.420206789	0.429948599	0.978084222	0.228022665	14.14905127
12389	NKG7	72.75598207	-0.270864271	0.257982551	-0.756640989	0.449264977	24.94511149
12390	NUP58	55.56907827	0.150622922	0.292812942	0.282474858	0.702109174	9.128914818
12391	OPA1	56.77924095	0.126561646	0.285292509	0.254244218	0.722080859	9.727084495
12392	ORAI2	51.62740621	0.270212097	0.409642915	0.659872506		

0.509225649	11.71411152
12393	PHLDA1 62.50022062	0.296025548	0.268664082	0.802968222
0.421992096	12.27511474
12394	POP4	67.97467278	-0.064229585	0.255628194	-0.180918122
0.856421844	11.89192209
12395	PYROXD1	44.65190171	0.274992047	0.452616492	0.826674192
0.408421752	11.44515805
12396	RAB9A	48.11098028	0.596820469	0.441280592	1.252496529
0.176216528	14.80848711
12397	RB1CC1 49.27871279	0.202062874	0.425647279	0.692265526
0.488080191	7.98914744
12398	RNF112A	51.72992629	0.066701625	0.416256756	0.160241567
0.872690787	17.04117277
12399	SF2A1	72.22049247	0.067099262	0.251166546	0.191075609
0.848466257	14.10105748
12400	SLC4A1AP	72.07009508	0.177485406	0.254025688	0.501220664
0.616145464	11.18241091
12401	THOC6	57.09470212	0.24965519	0.285288102	0.907280705
0.264258275	14.44794948
12402	THUMPD2	55.5268986	0.209077214	0.287898582	0.796799029
0.425567759	14.82275582
12403	THUMPD2-AS1	54.01540152	0.156622995	0.29528626	0.296128979
0.692009881	14.87215021
12404	TMUB2	79.29542249	-0.298288675	0.242492958	-1.162202529
0.244747241	9.440821504
12405	TOPORS 68.96816177	-0.082888207	0.251879248	-0.228400552	0.81157044
11.47141229
12406	TRIM44 62.89471615	-0.157211628	0.270598018	-0.424480489
0.671215422	9.181448101
12407	TSC1	66.97820026	-0.260092886	0.25246529	-0.725826926
0.461829946	19.01714779
12408	UBE2Z	54.00982095	0.264121982	0.297255221	0.664725066
0.506226258	12.29189111
12409	UFL1	54.79782555	0.087454687	0.29659221	0.220514826
0.825470219	10.55149119
12410	USP24	52.05578751	0.266759429	0.297824891	0.67054487
0.502510512	17.05148201
12411	YEATS2 68.02814487	-0.172218268	0.251174275	-0.490407128
0.622845822	17.5157707
12412	YY1AP1 58.05998682	0.295088441	0.28072242	0.775072974	0.42829654
14.84190905
12413	AP001972.5	66.70800244	-0.119182244	0.260224269	-0.220757257
0.740827774	14.21708812
12414	CEP164P1	52.78454912	0.142251106	0.298209249	0.259728268
0.719042871	19.94414894
12415	CLASP1 61.18848018	-0.527248202	0.298286542	-1.248898707
0.177269504	14.8449971
12416	CWC22	72.94982742	0.079507214	0.251512082	0.22618629
0.821056524	14.14707179
12417	DCAF1	65.25452485	-0.0166522	0.257172585	-0.046625259	0.96281182
12.08011411

12418	DERA	58.70292825	-0.184150489	0.292942606	-0.468642556	0.629224429	12.17414758
12419	FAAP100	72.81766256	0.007684714	0.250055128	0.021952869	0.982485552	10.41411711
12420	GFPT1	47.06645498	0.226918758	0.449564414	0.526996245	0.598196196	19.79547514
12421	GOT1	65.87811451	0.04847591	0.256956412	0.125802444	0.891976668	15.02052194
12422	GTF2E1	65.21709978	0.290675892	0.265888722	1.067745078	0.285625519	11.1418811
12423	HMBS	51.0799516	0.247272269	0.410522228	0.60222822	0.546948958	9.480579808
12424	IKBKB	77.5885709	-0.171268274	0.226425622	-0.509067299	0.610705052	18.77288287
12425	INO80D	52.44451127	-0.192708412	0.428521222	-0.452028692	0.651248212	14.02521115
12426	INTS9	56.66911191	-0.026916914	0.290276929	-0.094591586	0.924629222	17.8874885
12427	ISG15	74.84164242	-0.168556149	0.242518676	-0.492107907	0.622642062	18.74541789
12428	KTN1	65.81026644	-0.212525205	0.258547821	-0.592766952	0.552227127	12.0971182
12429	LOXL2	58.82042252	-0.141967926	0.286528526	-0.267289662	0.712402964	10.18811418
12430	MRPL17	76.65447501	0.069180966	0.250622842	0.197202592	0.842590751	17.4940124
12431	MRPL25	52.22007662	0.219966542	0.421804055	0.74099929	0.45869281	14.12588159
12432	OSER1	54.25172222	0.170102992	0.29654257	0.428964194	0.667949282	12.88149748
12433	PFDN2	51.62899922	0.498222974	0.402185979	1.225715028	0.216564509	14.51441101
12434	PKN1	90.72780166	-0.054221108	0.240722292	-0.159121111	0.872565577	11.84289445
12435	PSMB8-AS1	62.41844492	0.220591646	0.265018262	0.621726154	0.527565825	11.14885555
12436	PUM2	64.11910445	0.128296907	0.262518455	0.254180221	0.722202742	17.51855599
12437	SECISBP2L	60.65146978	-0.199607207	0.280257122	-0.524927187	0.599622809	11.89148425
12438	SLAMF7	64.0029016	0.112695117	0.275168427	0.200285289	0.762882207	8.994044952
12439	SMPDL2A	62.92862912	0.094069629	0.264148899	0.258227402	0.796154229	14.84409974
12440	TAF12	68.11568912	0.142145691	0.25462076	0.402647126	0.686472218	10.18270894
12441	TRIM27	40.8999926	0.177482654	0.521407258	0.24029246	0.722560242	10.41417174
12442	ZNF274	56.45522272	0.050825081	0.287804067	0.121084445	0.895708506	12.4144184
12443	ZNF76	71.01994915	-0.041852922	0.246172602	-0.120904807		

0.902766425 19.11552885
12444 AAR2 69.9952714 -0.021152921 0.248506215 -0.060695994
0.951601222 9.457997157
12445 AC005252.2 71.04541612 0.180914164 0.252711056 0.511474428
0.609018882 9.458224445
12446 AC242919.1 64.89024608 0.15994048 0.259625865 0.444741215
0.656506709 8.718180511
12447 ASCC2 60.75045552 0.292227056 0.272981826 1.054547512 0.29162222
12.08014418
12448 CLIP1 66.7922619 -0.272640721 0.258524022 -0.762242258
0.445218871 12.40924444
12449 CORO1B 97.29781617 -0.215714756 0.221282278 -0.651150712
0.514949202 9.480917195
12450 CTDSP2 64.72246124 -0.484724229 0.274906902 -1.292919219 0.196029
10.4849924
12451 CYSLTR1 68.82042418 0.122621245 0.252660542 0.276059492
0.706872669 9.948040222
12452 DGCR2 72.94400547 -0.027115274 0.242484861 -0.108270555
0.912701757 14.10214022
12453 DPH2 57.61817952 0.525259652 0.282207004 1.272921082
0.169466184 11.45820855
12454 DYNC1I2 80.6197196 -0.182945556 0.222552297 -0.550124018
0.582224214 19.17104554
12455 E2F4 51.40088215 0.242202164 0.410090278 0.824701972
0.402885521 11.44710451
12456 EAF1 60.06874725 -0.077982292 0.282755266 -0.202729512
0.828557062 14.19725577
12457 HAGH 61.68728665 -0.022080268 0.2719224 -0.088944274
0.929126198 9.959221459
12458 IFNAR2 58.94020911 -0.096699191 0.284098647 -0.251756125
0.801229565 18.79074577
12459 LATS1 62.00180628 0.045757171 0.278222492 0.120944014 0.90272528
19.44408741
12460 LY6E 75.51205921 0.287221727 0.258779972 0.800857766
0.422212992 7.977974215
12461 MED4-AS1 69.15055974 -0.428269571 0.252109089 -1.216297972
0.222871424 10.42774401
12462 MTOR 56.9111022 0.027165752 0.291226914 0.069419587
0.944655629 14.1424808
12463 NADK 62.51648444 0.019751511 0.277120664 0.052274512
0.958220282 11.1041525
12464 NDUFA5 66.28751627 0.112290252 0.255880759 0.218227671
0.750228812 18.41254578
12465 NEDD9 67.52982087 -0.151886467 0.25160452 -0.421980974
0.665755241 11.45201222
12466 PAF1 69.27852566 -0.294190625 0.251022479 -1.122942124
0.261461646 11.85040444
12467 PIGK 71.22680208 0.047752522 0.248212944 0.127126006
0.890922292 15.79415512
12468 PIGO 58.02207511 0.147082201 0.281727029 0.285207274
0.700009772 9.447042219

12469	POGK	67.85921847	-0.185788624	0.262274475	-0.5128294	0.608062667	15.21419475
12470	POLR2C	72.74219875	-0.148625772	0.242240549	-0.424272824	0.664090229	18.18974875
12471	RAB29	66.07489054	-0.047855942	0.254952909	-0.124822979	0.892751827	8.149181849
12472	RSRC1	69.48860257	-0.20820771	0.247014685	-0.600285	0.548216214	14.4229921
12473	SERINC5	55.99281925	0.204592612	0.288610968	0.526474109	0.598558826	11.89751572
12474	SPEN	59.12445111	-0.057592778	0.280452467	-0.151282222	0.879674122	11.495709
12475	STON2	70.61987706	-0.170065681	0.25401468	-0.480291606	0.620948962	17.17451244
12476	SUPT4H1	68.61205265	-0.280997212	0.25872766	-0.782216269	0.422441412	11.47800579
12477	THYN1	76.25692789	-0.272299298	0.225217869	-1.110287974	0.266874954	19.84042748
12478	TNFAIP2	68.49790086	-0.222054142	0.249952261	-0.66595772	0.505428126	14.10747821
12479	TTN-AS1	55.56225547	0.281752217	0.287977256	0.726208078	0.46771122	11.4718107
12480	TUT4	58.74079678	0.257601499	0.276478229	0.684240076	0.492822579	10.74259507
12481	UNC50	70.00089191	-0.165682828	0.245957682	-0.478910706	0.622002155	14.1201109
12482	UTP22	49.62610127	0.222598016	0.42572902	0.522850856	0.601078046	14.41748048
12483	WDR89	48.66818147	0.618900681	0.421872272	1.467029494	0.142268022	21.00119917
12484	ZNF664	58.76606459	0.108812219	0.281041226	0.285565664	0.775210805	12.89441425
12485	ZNHIT2	71.40226102	-0.280870058	0.242806002	-0.81922655	0.412600129	11.17411249
12486	ZYX	92.95722142	-0.258928764	0.221026244	-0.782176242	0.424111004	17.05142195
12487	ABI2	58.22661292	-0.186674428	0.405209905	-0.460685771	0.645024065	17.22012474
12488	AP002770.1	76.15212541	-0.157424224	0.224812977	-0.470185552	0.628222455	15.19141189
12489	ARFIP1	62.61586095	-0.05265492	0.266176791	-0.142796442	0.885661211	14.55042594
12490	ARID4A	62.64978499	0.084275072	0.2645002	0.221207142	0.817152882	11.41448442
12491	BAP1	59.10694269	0.51282129	0.278172752	1.256047019	0.175084208	11.90025108
12492	CCDC92	61.62928505	0.109155877	0.269845596	0.295129048	0.767887662	12.11159414
12493	CCSER2	80.50755127	0.126766527	0.24521074	0.267108556	0.712528045	8.954501184
12494	CEP62	60.426287	-0.045426926	0.275757872	-0.120920782		

						0.902752781	12.41512717
12495	CEPT1	60.50522627	0.118674147	0.275646289	0.215919925	0.752062288	15.21908115
12496	CIZ1	52.24264168	0.222812545	0.402046028	0.555202692	0.578687627	15.41184055
12497	DEF8	56.95248225	0.20512657	0.296828778	0.76891822	0.441941855	12.40491844
12498	DYNC1LI2	65.76842002	0.098922128	0.279899226	0.260290295	0.794562651	9.444107972
12499	EIF2B2	75.75912065	-0.215122922	0.252528449	-0.89124275	0.272745221	14.57191594
12500	ETV6	69.00426725	-0.215676285	0.25246512	-0.892090598	0.271808657	14.19888484
12501	GABPB1-AS1	58.21422051	0.062256069	0.281550292	0.162166047	0.870287699	11.11991485
12502	H2AZ2	76.19550542	-0.044899951	0.227822886	-0.122905802	0.894267877	12.40094054
12503	HCG18	52.16912826	0.267069088	0.419199865	0.627092494	0.524064597	11.2814411
12504	ICMT	82.52676284	-0.277556899	0.225075109	-0.852822906	0.292202566	19.57905072
12505	ILRUN	71.71777118	0.051105228	0.250576205	0.145774977	0.88409902	18.4847141
12506	INTS5	65.58508265	-0.070491792	0.256692908	-0.197625998	0.842227692	12.40941451
12507	MAP1S	62.27576229	-0.08622127	0.264986728	-0.226522622	0.812019297	19.42121257
12508	MED24	76.87268242	-0.051628224	0.240411682	-0.151692778	0.879428465	11.1122875
12509	MMGT1	56.64985814	0.276704766	0.295201761	0.95219607	0.240490727	15.27124105
12510	NIPSNAP1	76.48822289	0.020044612	0.240245776	0.058912159	0.952022074	10.19852884
12511	NIT1	75.82567501	0.198102796	0.249255565	0.567217292	0.570566561	18.04102552
12512	PCCB	62.81199699	0.029579112	0.266861756	0.080627406	0.925728271	12.15447011
12513	PDRG1	58.56918629	0.217822522	0.279154952	0.828241811	0.401894909	12.52892224
12514	PDS5A	70.22015046	-0.229969424	0.249286125	-0.972226477	0.220291067	17.81171584
12515	PIK2CG	64.87218286	0.071720604	0.258179785	0.200264244	0.841272925	10.410784
12516	PLEKHF2	71.05068526	-0.16444812	0.249262221	-0.470708192	0.627849124	14.27787404
12517	PSMG1	69.24524654	0.048247589	0.249442562	0.128069759	0.890185285	19.02172812
12518	RNF141	55.99625215	-0.104678206	0.40597976	-0.257841194	0.796529471	11.24841819
12519	RNF19A	47.06600228	-0.284929999	0.51818599	-0.549860482	0.58241507	11.48451182

12520	RNF20	62.86272286	0.027809682	0.261268092	0.076977962	0.928641076	17.51182512
12521	RPUSD4	64.49654061	0.497192916	0.268216726	1.249908564	0.177045214	8.102004742
12522	SF2B4	77.09965912	0.488628169	0.264515829	1.240485462	0.180087567	15.84410571
12523	SMARCA4	55.14228589	-0.141506612	0.412120791	-0.24252255	0.721957677	15.59429149
12524	SMC4	65.94002942	0.156218899	0.258980221	0.425174112	0.662426052	14.09455414
12525	SPCS2	61.40574844	0.026722285	0.269144627	0.072417104	0.942269974	15.1112575
12526	STAM2	54.96625596	-0.026896912	0.402502822	-0.066658549	0.946852524	12.28428407
12527	SUCLG2	62.2475245	0.174140858	0.26921269	0.471652208	0.62717427	9.911411224
12528	TAF9B	58.80829247	0.204584057	0.277820026	0.8061404	0.420161899	11.14574751
12529	THOC5	61.58849899	-0.099652799	0.272542902	-0.267496167	0.789087165	15.54141844
12530	USP20	61.24562059	0.271478711	0.268697414	0.726218456	0.461526917	11.29084801
12531	XPO6	61.61665221	-0.147525794	0.272267626	-0.295148866	0.692722027	10.92018492
12532	ABCC10	67.78868209	-0.05522792	0.250889667	-0.157707466	0.874687217	11.40821287
12533	AC011899.2	69.40279122	-0.210160285	0.251128769	-0.882222759	0.27706127	7.494289117
12534	ADAM15	54.28260744	0.28280687	0.402214147	0.952997946	0.240084682	14.04284825
12535	AGGF1	61.8586426	-0.088022822	0.271529894	-0.226912546	0.812722861	11.41597859
12536	AK6	60.75000282	-0.012024124	0.284582775	-0.021291294	0.975027222	12.14794417
12537	AP2D1	77.58264668	-0.207975412	0.222075122	-0.626290251	0.52112458	14.11997114
12538	ARL11	65.41062782	-0.040045262	0.262001505	-0.110217244	0.912157702	11.84721841
12539	ATG16L2	70.68722249	-0.212750252	0.244608147	-0.617268608	0.526991622	14.89981115
12540	BAG4	49.17449659	0.271527222	0.426254152	0.871405214	0.28252294	14.10878094
12541	C1GALT1C1	69.22842251	0.152027224	0.250850712	0.426160818	0.662720054	15.79048444
12542	CCL22	67.40900524	0.068666478	0.255852058	0.192962012	0.846987925	15.75287514
12543	CLPP	64.51827607	0.019990022	0.272044742	0.052720159	0.957150154	10.44221459
12544	CUTC	55.18282926	-0.028189012	0.40768821	-0.092672082	0.925269647	14.14494081
12545	CYB5R1	77.52459597	-0.256942642	0.220171821	-1.081084515		

						0.279659521	11.48740211
12546	DCAF6	68.59279989	0.059222622	0.249562542	0.169722468	0.865219752	12.41508711
12547	DHX16	80.27878822	-0.26217679	0.227429191	-0.800712942	0.422297849	15.58124595
12548	EIPR1	62.82149291	-0.174421202	0.266284221	-0.476190872	0.622928402	11.41474985
12549	FAM114A2	51.50155568	-0.087024845	0.429222574	-0.198152072	0.842926085	14.29100072
12550	FARSB	69.06669274	0.185208712	0.254625897	0.522547028	0.601289505	11.11177412
12551	FLCN	70.7719824	-0.092572108	0.244128842	-0.268999201	0.787920221	18.11040108
12552	GNAQ	62.21652486	0.215427565	0.267252425	0.858912115	0.290289005	12.85140899
12553	GPS2	68.19999527	0.140817274	0.259714442	0.291469612	0.695450146	11.14455877
12554	HERC4	62.94276029	-0.216724868	0.27795299	-0.828027152	0.402015454	11.84259149
12555	ITCH	62.58656497	-0.278100412	0.274405442	-0.742778767	0.457615622	7.445427914
12556	KRCC1	66.17058922	-0.007041822	0.262059215	-0.019449295	0.984482606	10.44144245
12557	LRP12	52.84447894	0.262701291	0.402148515	0.904295461	0.265785744	11.17142811
12558	MARK2	58.60798289	0.017012022	0.292824427	0.042208406	0.965455691	10.11951741
12559	MBD4	75.26612292	-0.219080088	0.240822658	-0.926205622	0.249167229	11.44501219
12560	MRPL10	76.27591525	0.062282496	0.257295168	0.177065896	0.85945662	12.45741717
12561	MRPL45	60.94991565	0.214980202	0.270229568	0.580495096	0.561580792	12.42104912
12562	MRPS5	72.72229922	-0.02862801	0.24187851	-0.082766628	0.922241982	10.44214401
12563	MUL1	91.8290767	-0.292212108	0.221280122	-0.912948197	0.261269787	10.10415947
12564	MYNN	46.82550021	0.246760192	0.461624065	0.751158156	0.45255748	11.91148271
12565	NFYA	52.25462207	0.116884682	0.420629242	0.277880476	0.781104112	9.428527179
12566	NIBAN1	61.00627227	0.221985202	0.269514284	0.898426997	0.26895262	12.80071218
12567	NOL6	66.20152782	-0.095291288	0.276845227	-0.252121474	0.800166618	8.940111914
12568	NOSIP	69.00998681	-0.221261272	0.250121216	-0.660499155	0.508922561	11.42184049
12569	NXT1	59.42429202	0.172202958	0.279809995	0.452655671	0.650076678	11.14024951
12570	PARN	67.45641008	-0.106292602	0.259415177	-0.296018671	0.767215822	17.04177424

12571	PCGF2	56.72262984	0.292959916	0.292724917	0.744052685	0.456842997	17.54929198
12572	PEX11B	60.67682449	0.157081267	0.272248502	0.421866252	0.672122644	14.07490498
12573	PIGG	68.84126292	-0.076917276	0.251679222	-0.218714582	0.82687229	14.07490498
12574	RAB2B	68.15422426	-0.220107289	0.259146241	-0.612862402	0.529967226	14.571944
12575	RNF122	59.82724822	0.296951205	0.275182587	1.058018842	0.290046852	14.12199418
12576	SESN1	54.14770725	0.420981272	0.296759289	1.061049569	0.288667275	12.11974089
12577	SGSM2	79.21516256	-0.299142298	0.245522522	-0.865745969	0.286629485	11.59412119
12578	SINHCAF	61.65047505	-0.021292059	0.270600256	-0.084705984	0.922495128	15.11944111
12579	SMIM10L1	59.07862821	0.464087277	0.276071218	1.224040606	0.217187774	7.148759411
12580	TBPL1	59.51745784	0.241721455	0.274475919	0.645492656	0.518607976	20.15222747
12581	TLR2	84.59297247	-0.298156527	0.225468541	-1.222222299	0.221202797	18.01421917
12582	TMEM262	68.4129848	0.221818262	0.255868727	0.904217625	0.265827004	8.145081944
12583	TP52	62.50290127	0.424994865	0.265965656	1.188622095	0.224588409	14.21144541
12584	TUBA4A	56.01902485	0.229845621	0.289269256	0.590452956	0.554886246	24.48911951
12585	TUBG1	82.21214771	0.150549915	0.242104701	0.428787095	0.660815812	9.75111197
12586	ZBED1	69.60922478	-0.120465442	0.266947024	-0.255542994	0.722182842	11.27014415
12587	ABRAXAS1	68.91482056	0.256407226	0.252201255	0.728012408	0.466605278	19.49884954
12588	ATAD2B	54.28509128	-0.016556259	0.416226198	-0.029766801	0.968279045	11.28999254
12589	C7orf50	95.92270088	-0.405687642	0.216985798	-1.279829082	0.200605252	21.74294227
12590	CHD2	62.18818021	0.199980406	0.266406627	0.545788164	0.58521156	12.12845272
12591	CMTR2	68.47506468	-0.274201555	0.252860929	-0.777264486	0.426942759	8.740441942
12592	CTBP1	62.19290956	0.128276768	0.262416809	0.252974229	0.724107761	11.48081799
12593	EBNA1BP2	74.52209402	-0.058215584	0.240947224	-0.170746562	0.864422049	29.1728189
12594	FASTKD2	61.22412687	0.201246502	0.269090271	0.54525009	0.585581526	11.42214482
12595	FIG4	67.96262246	0.279622242	0.256277022	0.784655647	0.422655494	11.9212145
12596	FIP1L1	47.90509102	0.458919815	0.426027506	1.052501985	0.2925692	

12.44810471
12597 GPR108 67.72769582 -0.267162902 0.255215222 -0.751906716
0.452107159 14.41448118
12598 HSD17B10 59.24221607 -0.18899027 0.292299247 -0.48052528
0.620852861 18.01277284
12599 HTRA2 86.2255884 -0.291999117 0.219781694 -0.912120177
0.261179229 11.91142158
12600 IL18R1 72.67225595 -0.242257879 0.227421884 -0.717946024
0.472790569 17.81414844
12601 JMJD6 67.6114019 -0.047077962 0.252929852 -0.122014955
0.894181552 8.714525191
12602 KMT2A 65.76762181 -0.222520687 0.261772907 -0.64272554
0.520402206 12.28711011
12603 LYRM4 57.7187018 0.027915521 0.288264478 0.09762872 0.92222711
9.929522119
12604 MAP1A 72.80890191 0.047712945 0.242160782 0.129448901
0.889095422 17.28094944
12605 MCM4 77.10980181 -0.172440265 0.222242822 -0.518861226
0.602857442 11.18244945
12606 MCM6 68.52157127 -0.106797448 0.249182662 -0.205849802
0.759719008 15.10957114
12607 METTL7A 72.26724692 0.202662614 0.247089048 0.586776264
0.55725298 14.57575174
12608 MGME1 82.05660206 -0.297217028 0.22272872 -0.918285757
0.258416945 15.84947158
12609 MIS12 56.54812295 0.252627626 0.28705972 0.65268287
0.512960102 17.21884124
12610 MRPL20-AS1 82.68568156 0.117254406 0.228622272 0.246562222
0.728919405 10.47118121
12611 MRPS22 60.04292184 0.522222221 0.27690151 1.412128412
0.157912154 11.1505144
12612 MXD2 94.88554227 -0.246207642 0.217221828 -1.090995608
0.275274815 10.19441089
12613 NDRG2 71.92285192 -0.117674109 0.244220228 -0.241856922
0.722458562 11.71144995
12614 NDUFS4 80.44751115 -0.195902608 0.241655006 -0.572295982 0.56627661
11.95117291
12615 NMT1 62.48280256 0.046792647 0.272555881 0.125599001
0.900049252 12.92141929
12616 NUP122 50.25620462 0.207265149 0.428288719 0.482927912
0.628429922 10.42489154
12617 NUP210 84.12782661 0.065426441 0.22604172 0.194697276
0.845629866 15.08498849
12618 PLBD2 64.08219059 0.220766569 0.277600066 0.584657125
0.558778289 11.71874059
12619 PXN 75.0212029 -0.210447711 0.242101991 -0.907471219
0.264157662 15.84448719
12620 RNF2 82.22824022 -0.149114225 0.226789522 -0.456200812 0.64817268
11.4180401
12621 SBDS 72.01870204 -0.100449972 0.241956999 -0.292750204
0.768948712 14.25807541

12622	SCAP	79.65408124	-0.258774064	0.221926947	-0.77958801	0.425622416	7.741502181
12623	SEC16A	78.71998525	-0.025026774	0.22525845	-0.074679024	0.940470098	15.08114772
12624	SLF2	49.89490225	0.411954881	0.420909265	0.978726096	0.227715224	17.1941095
12625	TIMM22	65.99250828	0.264524254	0.261182167	0.722284592	0.46292286	12.25402152
12626	USF2	51.79995264	0.026976644	0.42926227	0.0861199	0.921271105	11.49570792
12627	XPC	61.16917421	0.014897702	0.272466541	0.029997422	0.968095181	15.08498849
12628	ZNF124	61.88684581	0.051002898	0.26972044	0.127948875	0.890280822	12.40924528
12629	ARAF	79.20958984	-0.042529881	0.222922615	-0.127772245	0.898228424	19.20202202
12630	ATP2A2	46.14020908	0.492229454	0.464620674	1.061574488	0.288428901	11.4077589
12631	B2GNT4	85.08600804	-0.086121664	0.226725446	-0.262582249	0.792101828	19.98954588
12632	BAG6	61.41962158	0.072774166	0.271190271	0.198750216	0.842458141	14.81114219
12633	C5orf51	78.15680988	-0.12412124	0.220208621	-0.406201798	0.684594222	12.84149914
12634	CKAP5	58.6472761	0.015910921	0.289928588	0.040802685	0.967452401	19.52272417
12635	COQ9	56.11482275	0.424525049	0.287295407	1.121947225	0.261884822	17.09244171
12636	FAM162A	64.209805	-0.104422869	0.265222224	-0.285829581	0.775000991	11.89741452
12637	FLAD1	65.77111196	0.25258986	0.265790285	0.692265518	0.488142929	11.40180078
12638	GPANK1	64.15227222	-0.051722202	0.26574979	-0.141417178	0.887540284	10.92859178
12639	IGF2BP2	54.64014611	0.552586488	0.292187129	1.411526567	0.158086461	17.24992102
12640	KCTD2	60.54297154	-0.146999507	0.292452042	-0.274565848	0.707982276	11.95187712
12641	MTIF2	71.85007808	0.147922426	0.244718067	0.429111252	0.667842191	18.21440494
12642	MVD	88.14755057	-0.415907629	0.212629572	-1.226068725	0.184816924	19.74751224
12643	MYCBP2	72.42498901	-0.156848526	0.240520001	-0.460614752	0.645075025	18.81182228
12644	PDXK	54.51872682	0.209627222	0.402220212	0.519752719	0.60222524	11.01974219
12645	PPIL2	61.94877181	0.048974592	0.270724849	0.122104964	0.89490127	19.21122881
12646	RANBP1	74.8616808	0.228958276	0.245461957	0.691706484	0.489121675	10.94411141
12647	RCHY1	60.02670844	0.215720799	0.275507767	0.574477595		

0.565644658 12.84014445
12648 RETN 74.29281176 -0.151027201 0.227479185 -0.447545521 0.654481221 10.11215142
12649 RREB1 75.82412016 0.179882076 0.242225125 0.52547426 0.599252472 14.91218191
12650 SAR1B 69.57205892 0.080448119 0.246968429 0.221860055 0.816646711 12.09910919
12651 SEPHS2 65.74275849 0.024546901 0.275277221 0.092057012 0.926652742 14.81951794
12652 SIRT2 78.49768905 0.075244687 0.22652206 0.222595108 0.82207227 14.28040811
12653 SMG7 70.04012191 -0.021865211 0.245192581 -0.062242066 0.949494119 14.22817815
12654 SNHG6 68.57922062 -0.212661418 0.258808217 -0.595475262 0.551525829 11.9108285
12655 ST8SIA6 52.29610955 0.496420112 0.298712967 1.245052227 0.212112186 14.11021122
12656 TMEM140 67.99664924 -0.148922594 0.252852184 -0.422052769 0.672985771 14.24299901
12657 TRAPPC2L 62.40962214 0.222569872 0.271702272 0.870505612 0.284024154 18.57700557
12658 VPS52 84.24119628 -0.421260229 0.219462457 -1.218962217 0.187181744 14.11402209
12659 WDR12 71.10992295 0.199826944 0.246562722 0.576622955 0.564192514 14.51914405
12660 ATG101 67.26652188 0.124871904 0.252054742 0.28209924 0.701646182 14.81117442
12661 CBX6 85.258988 -0.060912955 0.226288747 -0.18662007 0.851950689 11.57071109
12662 CENPO 67.2620646 -0.075914927 0.256422442 -0.212984887 0.82122875 14.42788004
12663 CERT1 74.1714654 -0.102522491 0.227605452 -0.206667119 0.75909676 11.40574488
12664 CHMP1B 65.26521898 -0.126224472 0.264514871 -0.246555058 0.728925614 14.84180784
12665 CNTD1 69.88548242 0.088711677 0.248640521 0.254450278 0.799147712 11.18541241
12666 EIF1AY 81.54226848 -0.248887442 0.222109692 -0.770287768 0.441129211 9.99014909
12667 EME2 62.09297065 -0.027726891 0.270006809 -0.074962191 0.940242999 12.14791204
12668 FAM149B1 100.2905872 -0.268112896 0.221494552 -0.822960928 0.404202001 15.04181219
12669 GGA2 64.24542707 0.125028262 0.268572168 0.229222561 0.724441222 12.40452004
12670 GTF2F2 64.69569821 0.148290257 0.259867091 0.412069511 0.680288475 15.48110129
12671 HIBCH 62.60296929 0.224681914 0.291776412 0.572495257 0.56620941 11.40855014
12672 HMGCL 91.00759047 0.017619612 0.221017951 0.052228568 0.957549794 9.11414098

12673	JADE1	72.20478248	0.154209648	0.259471244	0.428990219	0.667920242	9.972257122
12674	LAS1L	67.59428767	0.029144261	0.251242289	0.111445149	0.911262259	9.194492474
12675	LENG8	72.28688716	-0.275489262	0.242108094	-0.802922656	0.42201944	15.07848145
12676	LRP10	77.15476471	-0.125862429	0.220728616	-0.410800244	0.681218941	10.42197412
12677	MLLT2	72.86226606	-0.422627874	0.246042292	-1.252106582	0.210166921	12.87954112
12678	MPI	76.52687796	-0.208877207	0.241822945	-0.611068421	0.541154285	10.12514494
12679	MRPL47	71.27444792	0.229227525	0.245974651	0.66287278	0.507411296	14.94187915
12680	MRPS14	62.85112686	0.051542765	0.265746244	0.140924895	0.887929275	15.24205757
12681	MXD1	62.60992565	0.017405219	0.271102262	0.046901678	0.962591591	19.72229448
12682	NEK9	79.1080169	-0.057799982	0.222589772	-0.172787612	0.86202228	4.480554749
12683	PCBP2	66.69958614	0.100882655	0.259455206	0.280654217	0.778975551	14.84494594
12684	POLR2D	59.66618101	0.184720622	0.284869211	0.479956872	0.62125806	12.22422419
12685	PPP1R2B	69.74720225	-0.112558425	0.247051494	-0.227209181	0.742509677	10.91147981
12686	RBM28	64.6450124	0.217201855	0.26189247	0.600182405	0.548284012	9.494405181
12687	SDAD1	77.88671218	0.097557556	0.245942085	0.282005459	0.777929224	11.28511419
12688	SIN2A	55.9424272	-0.152241529	0.420419218	-0.26472476	0.715209409	12.45108189
12689	SNAP47	69.84215248	0.028121155	0.246497814	0.081187106	0.925292154	18.58819477
12690	TTC5	55.29979617	0.240879562	0.411747091	0.585018248	0.558525447	15.45744525
12691	UMPS	66.29262101	-0.016227995	0.258266969	-0.045602851	0.962626797	15.14025707
12692	UTRN	61.8245161	-0.156216229	0.281928292	-0.409271082	0.682240725	17.20750095
12693	WDR2	72.80969102	0.167298025	0.242999076	0.488042224	0.625519926	11.44408048
12694	WDR82	51.29475552	0.220442429	0.42894246	0.527222921	0.591106042	17.19052892
12695	ACAD9	59.78446252	0.156105242	0.282499751	0.408118547	0.682186644	19.70514244
12696	ASNS	67.60269229	-0.046269115	0.252954827	-0.120720291	0.895996501	18.99219149
12697	CALCOCO1	68.79594202	-0.05282297	0.249208	-0.154160185	0.877482425	10.81149451
12698	CCDC82	55.95942821	0.129911929	0.406802922	0.219248587		

0.749462192 18.89802721
12699 CNPPD1 84.11485024 -0.122109106 0.222800092 -0.277112626
0.706089901 14.87871981
12700 DDX49 72.51964677 0.159298128 0.246199472 0.460422792
0.645212777 18.94110291
12701 DYNC1LI1 64.61650767 -0.094292214 0.26628422 -0.257258752
0.796901842 14.14889772
12702 ELP2 69.05788202 0.16526445 0.252425754 0.467890208
0.629862106 15.71984172
12703 EMB 72.22025622 0.011752201 0.240865862 0.024477789
0.972496154 17.40179904
12704 FAM52C 62.24920798 -0.291940486 0.295257248 -0.72842199
0.460258056 10.29514481
12705 GK 58.79112571 0.405792458 0.27890267 1.070970172
0.284182841 10.70117
12706 HPS4 69.27806866 -0.229714186 0.260671809 -0.914166779
0.260629217 17.79152595
12707 IRF2 55.94247619 0.052266278 0.410791425 0.129667451
0.896829525 11.45570949
12708 ISG20L2 71.21129254 0.401469171 0.250920275 1.144046242
0.252604419 10.44589817
12709 KDM2A 58.52067966 0.072245564 0.291825942 0.186929161
0.851716175 11.91122144
12710 KLF10 76.9098257 -0.126947491 0.220780682 -0.282781452
0.701140426 10.47118121
12711 LINC00667 64.68699085 -0.074228562 0.272447478 -0.19906029
0.842215584 17.17919755
12712 MORC2 62.94111596 -0.122054966 0.281954788 -0.245724549
0.729542217 11.12551907
12713 MRPL22 65.41882919 0.070662528 0.259200242 0.19672461 0.84404205
10.10494248
12714 MRPL28 82.96114429 -0.020998592 0.221782675 -0.092420415
0.925561629 14.50148418
12715 MRPS15 70.91289429 0.291192222 0.247228696 0.828279424
0.401817642 11.4444492
12716 MTIF2 64.01762026 0.408908451 0.261605279 1.120814286
0.258122224 12.24190484
12717 PAFAH1B1 82.82421126 -0.444809496 0.227117244 -1.259785729
0.17289774 11.10812451
12718 PPID 82.8792196 -0.220626179 0.225672778 -0.677476022
0.498102972 11.88407214
12719 RCSD1 96.25291112 -0.447500078 0.216656654 -1.412202826
0.157596096 18.72707225
12720 RGL1 58.85215291 0.428202226 0.278761482 1.120522662
0.258251844 14.29194959
12721 SART2 60.91290166 0.169704872 0.278228844 0.448671189
0.652668871 14.21574544
12722 SLC26A1 72.19821465 -0.288166889 0.244494208 -0.826492697
0.402877782 12.82429582
12723 SNHG1 64.51841702 0.157572711 0.2708292 0.424922609
0.670892092 14.41140947

12724	SZT2	64.28418461	-0.215694772	0.274270175	-0.576207668	0.564407241	14.19152424
12725	TCERG1	87.28824292	0.088721001	0.221179256	0.267924211	0.78875758	19.50552089
12726	TRAPPC1	70.5579286	0.080259194	0.244581247	0.222208262	0.81559969	12.51245494
12727	TRIP4	75.80422052	0.180821884	0.228706066	0.522860779	0.592427859	12.41152811
12728	WDR72	58.8806102	-0.092225428	0.298720406	-0.222805665	0.815125852	8.702951441
12729	ZBTB48	68.98426427	-0.124858957	0.251661148	-0.255054729	0.722548582	15.59250401
12730	AC068888.1	61.40812678	0.121787026	0.276660669	0.222222526	0.746442659	10.4729444
12731	ARFGAP1	70.28627818	0.191151699	0.245462948	0.552220408	0.580044029	18.17840419
12732	ARID2	60.2572562	0.421982525	0.277228668	1.118215111	0.262422425	11.70127841
12733	ARMC6	60.20525694	0.542729142	0.275409097	1.445700564	0.148261206	9.959120227
12734	BCL2L12	76.79970666	-0.255725267	0.225406897	-0.76242294	0.445801652	9.179521458
12735	CHMP2B	67.08204712	0.147610969	0.257604701	0.412776924	0.679770059	18.79910047
12736	CHMP7	79.50788552	0.110968516	0.222778141	0.222460952	0.72878626	10.54418418
12737	COPS5	67.16949222	0.220815192	0.25242462	0.654924909	0.512509662	11.90122194
12738	DCP1A	72.18224774	0.276944849	0.247221091	1.085604702	0.277652918	18.09191051
12739	DIP2A	80.10687206	-0.182778287	0.22466222	-0.562977922	0.572449918	9.117481142
12740	EIF6	80.27057224	0.046795926	0.224147112	0.140045908	0.888622718	11.41054419
12741	FAF2	60.29782696	0.291876504	0.296299992	0.726502924	0.461424075	17.41544719
12742	FKBP2	79.96922787	0.02922521	0.229909281	0.119220248	0.905092864	15.41571194
12743	GARS1-DT	77.16247255	-0.262549795	0.252068582	-1.022610729	0.201786105	14.15174141
12744	HDDC2	61.82222425	0.291162622	0.275292444	0.775624622	0.427970658	12.57145404
12745	HDGFL2	75.57825288	-0.155506002	0.241771262	-0.455000095	0.649109171	11.24844492
12746	HPS2	61.21124955	0.202894565	0.272816956	0.544220226	0.586289866	18.82201458
12747	ITPRIP	68.82289491	-0.242798224	0.26294666	-0.671719422	0.501762228	18.01795784
12748	KPNA2	56.64920102	0.200575895	0.402699608	0.744552249	0.456541779	15.54812808
12749	LYRM2	74.22491212	0.047268742	0.240422607	0.129142282		

0.889227628 17.58042117
12750 MIS18BP1 56.72241518 0.191216707 0.299045292 0.47942595
0.621628522 18.7481111
12751 MRPL4 64.77179987 0.270295744 0.26012229 1.028496266
0.202716446 12.5049509
12752 PDCD5 77.11869911 0.279784224 0.241588516 0.819068022 0.4127476
15.11797704
12753 PIK2R4 48.17214676 0.441445207 0.446228114 0.989027667
0.222644712 12.5811411
12754 PJA2 50.56700286 0.222555485 0.442620528 0.502924028
0.614207727 15.40714484
12755 PPIE 85.45692621 -0.224128202 0.219421452 -0.722976128
0.462572984 8.470078445
12756 PSMD5 71.48094224 0.142655294 0.256127668 0.400572747
0.688722978 11.4101047
12757 RGS14 62.9808598 0.264678122 0.261828181 1.007848654
0.212527124 9.911471074
12758 RNF10 84.49989724 -0.411105072 0.218208851 -1.29192475
0.196279742 18.20445942
12759 SC5D 79.4256104 -0.472196785 0.249498246 -1.251070155
0.176672961 10.91879441
12760 SH2BP2 82.28852679 -0.007127942 0.24166857 -0.020862155
0.982255616 12.87110515
12761 SLC25A5 68.52281072 -0.06270586 0.252077907 -0.180942508
0.856412702 14.94059444
12762 SPOUT1 76.69047487 0.106972101 0.240019969 0.214605249
0.752061217 11.8754721
12763 STAT5A 62.29485245 -0.268745279 0.292409627 -0.682118462 0.49452199
10.9498821
12764 TBCD 74.00820982 0.040042424 0.227112725 0.118780196
0.905449499 18.18979774
12765 TTC2 78.24781294 -0.177729518 0.228562755 -0.540958992
0.588525848 17.05705151
12766 UBE2K 74.44145876 -0.171229719 0.225748808 -0.510221152
0.609826486 15.04440584
12767 UGDH 79.24697162 -0.157042224 0.226825276 -0.480492249
0.620876622 11.11417171
12768 UTP11 62.55626217 0.088950172 0.289626945 0.228289882
0.819420889 14.10200907
12769 VIPAS29 85.27296224 -0.010207785 0.22479906 -0.021725882
0.974682679 24.29197494
12770 WDFY4 70.64747208 -0.169691286 0.254670696 -0.478447429
0.622221776 9.209255791
12771 ZC2H14 71.12279709 0.240641065 0.24482796 0.697858222
0.485265794 8.7999018
12772 ZC2H7A 68.9261622 -0.276291211 0.262441528 -1.025255572
0.200502886 24.28740429
12773 ZNF22 74.82456776 -0.010261878 0.241205854 -0.020268408 0.97577224
5.792910714
12774 AC125050.7 69.15248169 0.078994545 0.250840258 0.22515809
0.821856292 15.7544489

12775 ACBD6 71.92090204 -0.097001684 0.246685748 -0.279797149
0.779622129 4.200528241
12776 AL021595.1 74.10117422 -0.04048241 0.246888784 -0.116701411
0.907096672 19.94111111
12777 AL255212.2 81.26482497 -0.224220202 0.222162004 -0.694129796
0.487594544 18.18219998
12778 ARHGAP17 60.7807847 0.29269486 0.272091177 1.058060187
0.290028004 12.89182224
12779 ARL6IP6 82.78202071 0.079682188 0.227524457 0.226071269
0.812277268 11.94794449
12780 BAG5 51.28168886 0.122742871 0.450917778 0.274424467
0.782758442 18.52929171
12781 BIN2 57.58249596 0.248106867 0.292991508 0.621228826
0.527825542 15.17714795
12782 CDC5L 70.90422601 0.250821694 0.244877826 0.727205948
0.467028555 20.1720829
12783 CTSH 68.90596871 0.278666256 0.249066928 0.798217554
0.424686224 19.921958
12784 DENND1C 70.46168485 -0.222546072 0.250489777 -0.627810525
0.522597021 14.41271784
12785 EIF2AK2 72.22107219 0.248242406 0.244266914 0.721156406
0.47081229 10.41182092
12786 GALNS 64.22285119 0.420894041 0.264258245 1.155482222
0.247892009 11.17111719
12787 IRF2BP2 75.19842265 -0.226986984 0.22684024 -0.702559005
0.481707449 14.41281797
12788 MRPL14 78.21462282 0.212521229 0.225756095 0.622992212
0.526728076 11.45544418
12789 MRRF 71.15189917 0.229886654 0.244448672 0.696426576
0.486155464 10.79144401
12790 NCK1 72.51546724 -0.225045755 0.246596162 -0.67815452
0.497672725 19.01494517
12791 NCLN 71.42065102 0.229811784 0.245849827 0.95262712
0.240272422 17.0794829
12792 PECAM1 65.48259946 0.201242228 0.260417822 0.558258175
0.576599827 15.85140924
12793 PLCG2 85.72980615 -0.210202621 0.214956549 -0.984906116
0.224670189 15.08114444
12794 PPP2R2C 76.69599421 0.069248142 0.225905699 0.206451225
0.82642846 14.87412177
12795 PRPF4B 52.95121444 0.582824815 0.406154008 1.427446888
0.150591054 10.51140011
12796 PYM1 72.05127989 0.058962486 0.228512626 0.174182492
0.861721259 17.54241901
12797 RCC2 71.16512966 -0.252740217 0.252969221 -0.996527927 0.2189927
11.41411248
12798 SCAF4 82.60056572 -0.019145675 0.22600666 -0.058727866
0.952168864 12.42715181
12799 SEC61G 87.29785998 -0.024172052 0.222721099 -0.07490285
0.940291212 17.5014917
12800 SLC4A8 67.96784092 -0.512442088 0.287004216 -1.226711872

0.184604012 11.59119115
12801 SMARCA2 61.17717426 -0.021542727 0.284270778 -0.082062281
0.924596292 12.54414895
12802 SYVN1 62.91002512 -0.017640171 0.270040952 -0.047670861
0.961978557 14.80912809
12803 TBC1D22 79.88184154 0.005851678 0.220410624 0.017710219
0.985869949 12.4594844
12804 TIMM50 67.44804285 0.17552762 0.254180072 0.495588666
0.620184651 19.11542458
12805 TMEM11 78.10292299 -0.112562214 0.221252492 -0.242826746
0.721728804 14.04912401
12806 TMEM126B 81.07757707 -0.25896284 0.224922546 -1.104720012
0.269276595 17.94040418
12807 TMEM167B 62.28181622 0.121266117 0.276225502 0.222214291
0.747214522 14.24752205
12808 TTC27 59.24670497 0.208012212 0.282977512 0.54214722
0.587028268 11.4122184
12809 TTI2 59.0887766 0.092259905 0.415112792 0.222492981
0.822920127 12.28124822
12810 ZNF222 91.80205152 -0.425622088 0.20687492 -1.419575402
0.155721221 14.24279749
12811 AC027219.1 71.9928781 -0.028576792 0.24226921 -0.112675962
0.910287459 25.77175972
12812 ADA2 74.82949021 -0.205282418 0.244220465 -0.596271917
0.550926797 15.44994154
12813 AIMP1 71.88264621 0.249672245 0.24578927 1.01122191
0.211905447 15.55294017
12814 ALAD 65.82477258 -0.222528252 0.276442782 -0.620282071
0.525006272 15.41075428
12815 B2GNT5 66.96296041 -0.54242995 0.40079227 -1.255910866 0.17512752
18.2441021
12816 C19orf48 77.1664472 0.278591505 0.244206778 0.809127429
0.418426095 10.21222178
12817 CDKN2A 70.14428244 0.226428617 0.24804222 0.927925222 0.24828284
21.11112224
12818 CDKN2AIP 79.82518259 -0.209494981 0.225252772 -0.644098985
0.519511248 14.81749407
12819 CLCN2 64.54820594 -0.498752127 0.416249025 -1.198208518 0.22082585
11.4577905
12820 CREM 92.77958121 0.040596074 0.222294552 0.125569929
0.900072268 18.15015187
12821 CRYZL1 69.88224242 0.110152275 0.246588492 0.217821501 0.75062024
11.88025121
12822 DPAGT1 74.14642867 0.250722915 0.240942884 0.725410218 0.46208964
14.25791424
12823 ERCC2 76.02248692 0.210248682 0.228047942 0.621949207
0.522975202 11.87000142
12824 FBXW11 57.89045002 0.511092768 0.282858997 1.221462262
0.182026954 10.52174159
12825 FH 70.75222276 0.258762871 0.257742072 0.722222446
0.469481806 19.21490821

12826	GMPPA	69.28129647	0.152490007	0.246905758	0.429571852	0.660247221	14.15255994
12827	GOLGA7	84.48069262	-0.081540702	0.222580657	-0.244440728	0.806889494	11.91504441
12828	GSE1	65.48622261	0.201227181	0.260975591	0.557720724	0.577028268	11.1198448
12829	HIBADH	60.60076829	0.526724112	0.27209274	1.415572121	0.156900816	12.89080121
12830	INIP	55.28294215	0.225779598	0.408280712	0.797725027	0.425024272	14.40841187
12831	KLHL12	61.55461149	0.224222542	0.277747876	0.620214655	0.525050649	11.47595955
12832	LETMD1	71.4166224	0.515877404	0.255278466	1.45202679	0.146491276	14.57848488
12833	MAN2C1	71.91090225	-0.218078206	0.250941989	-0.906255511	0.264747712	10.190449
12834	MIF4GD	86.82068581	0.041204402	0.224298282	0.127265429	0.898651177	11.47874484
12835	MTF1	72.29851969	0.106296549	0.246005854	0.207499274	0.758462276	15.89195402
12836	NUP107	78.26658204	0.158675591	0.222248891	0.477580497	0.622948814	14.41491891
12837	NUP160	62.20085848	0.129125622	0.26842681	0.250468871	0.725986848	18.57404901
12838	PABPC4	69.80622961	0.112612901	0.248770855	0.222885066	0.746782287	12.54498419
12839	PCNX1	58.61588167	0.067506926	0.412116498	0.162408922	0.870196471	8.941805918
12840	PEX19	67.154962	-0.112428257	0.269562487	-0.204246944	0.760929762	12.17404518
12841	PIGB	55.86109402	0.424491174	0.295065695	1.074482495	0.282606486	19.01850919
12842	PLAGL1	61.07062656	0.254765841	0.272625295	0.949497666	0.242267559	11.94221995
12843	POLR1A	56.50544711	0.410795602	0.408556761	1.00547988	0.214665822	14.21474711
12844	PRELID1	125.7902111	-0.29548709	0.220597621	-1.222592241	0.217254476	11.14081214
12845	RAP2C	66.90745002	0.091928047	0.25550298	0.258585868	0.795954788	10.14444147
12846	RFTN1	80.618959	-0.181561257	0.222280142	-0.561622051	0.574272552	12.82718004
12847	RPS6KA2	62.46627504	0.224151822	0.268902929	0.624722026	0.525609067	10.22594449
12848	RRBP1	56.2647762	0.229257429	0.406029289	0.589261282	0.555685946	17.14158717
12849	SAMM50	84.57898452	-0.068756829	0.220659255	-0.214422206	0.820216969	11.84411444
12850	SLC50A1	72.92055522	0.164206929	0.248756925	0.470824906	0.627758626	19.21829989
12851	SLC7A6	69.62627447	0.208262021	0.250582907	0.879569468		

						0.279092585	14.11122142
12852	SMC1A	64.74244425	0.271824904	0.259288626	1.024891942	0.200719278	15.4474427
12853	SUPT7L	81.89686952	-0.082196819	0.222788117	-0.254646262	0.798996248	15.19784112
12854	TEX264	76.22467124	0.160655262	0.225124972	0.47928911	0.62166184	11.25228218
12855	TNFAIP8L2	69.05200887	0.187029167	0.257206468	0.52246986	0.60064722	11.14544571
12856	TXNDC9	65.21056729	0.225495895	0.258504224	0.907927644	0.262916451	12.5810409
12857	VRK2	66.79722206	-0.162526241	0.264847749	-0.448204221	0.654005727	17.74424957
12858	VRK2	74.51751025	-0.027457859	0.225682641	-0.11158712	0.911150785	12.45721489
12859	ZNF217	62.58640726	0.214928755	0.299797545	0.787720584	0.420860162	18.7595548
12860	ZNF521	56.62292094	0.562420227	0.288295771	1.448085628	0.147592102	14.24111505
12861	APOL6	72.14145804	0.115720592	0.242622221	0.226786196	0.72627808	11.9107272
12862	ARFRP1	80.16596156	-0.021721812	0.225219492	-0.066821985	0.946722421	10.70495409
12863	CCNK	79.41725145	0.022245812	0.228227546	0.070822246	0.942529226	14.8081855
12864	GBGT1	69.22025915	-0.116188066	0.255404185	-0.226918115	0.74272982	17.77457185
12865	GIGYF2	62.77517294	0.207694254	0.264428642	0.844219621	0.298490825	10.49922244
12866	HLA-H	71.29778807	0.070562521	0.242271928	0.205498828	0.827182414	15.81415414
12867	LAMA5	85.16170491	0.014427066	0.222064688	0.044826604	0.96424552	18.55122419
12868	MILR1	79.75181292	-0.207294285	0.222872584	-0.621176084	0.524482772	19.18401988
12869	MIR4425-2HG	58.8895075	0.502805927	0.281520901	1.217864256	0.187549092	15.27115945
12870	POLK	55.42902509	0.558705914	0.297265565	1.406024988	0.159716705	14.11414912
12871	POLR2E	70.58867257	0.16186794	0.244922405	0.469287982	0.628862804	11.4101055
12872	PPP1R10	79.21851481	-0.174066829	0.228426504	-0.520002412	0.596110258	9.920915981
12873	PPP6R1	72.46820586	-0.156822219	0.242268111	-0.456717772	0.647872914	14.59994489
12874	PTBP1	154.1720422	-0.472122504	0.612407651	-0.769671691	0.441494667	11.11104294
12875	PVT1	77.2870194	0.08522956	0.22156501	0.257284096	0.796882279	14.85044529
12876	RASSF4	72.11061965	0.214942499	0.242282469	0.919856022	0.257647998	14.14910144

12877 SEC11C 66.77259892 0.294642448 0.259500249 0.819591777
0.412448862 11.51145204
12878 SF2B5 74.77262842 0.04947144 0.224612427 0.147846546
0.882462874 12.48197941
12879 SUSD1 74.42155804 -0.072241965 0.229258148 -0.215824979
0.829124177 8.129542751
12880 TIMM12 70.26622728 0.110170969 0.244950098 0.219282229
0.749426602 15.24841704
12881 TKFC 86.67812669 -0.221029127 0.218261109 -0.694202169
0.487492104 17.07284217
12882 TM2D2 64.70284845 0.205802526 0.261188961 0.846658025
0.297185756 18.44105545
12883 USP5 85.654296 0.081971024 0.222647277 0.252272712
0.800057481 11.72081159
12884 ACTR8 62.06217095 0.427714285 0.265256722 1.198046582
0.220898882 18.20944284
12885 AGO1 82.05101416 -0.442922192 0.227578641 -1.252109502
0.176240288 11.4551708
12886 ARID5B 61.48681122 0.260924629 0.275467842 0.694922028 0.4870979
14.12851454
12887 BTAF1 52.64285559 0.185461111 0.446116208 0.415722769
0.677612146 17.81404744
12888 C12orf10 76.28878564 -0.022066676 0.22820204 -0.094815148
0.924461655 11.45899752
12889 CBL 84.12588589 0.066919805 0.224045284 0.206512679 0.82628968
10.01050221
12890 CLP1 66.69922929 0.429059659 0.258886779 1.195529298
0.221880297 11.14484142
12891 CMAS 75.27457429 0.10686241 0.224171491 0.219786127
0.749120457 18.04192214
12892 COX15 76.2876594 -0.064870829 0.226216661 -0.192886159
0.847048125 18.04745128
12893 CYB561D2 94.08562801 -0.199966028 0.212242519 -0.640212919
0.522022525 14.0848421
12894 CYBA 67.28140497 0.074580676 0.267042701 0.20219246
0.828982825 14.44129497
12895 CYREN 87.86028025 -0.229106002 0.221552021 -0.74259972
0.457118667 19.05142221
12896 DNASE2 89.29117911 -0.18842968 0.217505245 -0.592469218
0.552867111 11.41211818
12897 ENSA 79.10745977 -0.111597945 0.228027182 -0.240199071
0.722706618 14.04419847
12898 HNRNPLL 70.28607251 0.222000091 0.244729872 0.675872187
0.499121798 11.11078048
12899 HS6ST1 105.5292297 -0.202868921 0.208469922 -0.657661918
0.510755298 11.42584099
12900 HYOU1 76.22964285 0.026408299 0.25811477 0.072742827
0.941215012 11.44142154
12901 MCL1 85.1467811 -0.26122265 0.219192526 -1.12167599
0.257770679 11.42840891
12902 MIER1 80.68922978 -0.201066862 0.228788196 -0.611529178 0.54084269

12.15910217
12903 MMS19 89.02905868 -0.241701175 0.220278299 -1.066554976
0.286172841 19.04474142
12904 NUDCD2 80.24846072 -0.298868502 0.225724124 -0.917522852
0.258868724 18.7744147
12905 PARP14 58.95645822 0.149767286 0.297222959 0.276921695
0.706224266 17.17441119
12906 PHYKPL 82.61056196 -0.191297892 0.219176901 -0.599247548
0.548941146 14.87987145
12907 PPIL4 74.05595572 0.117176902 0.226228444 0.248492471
0.727469617 14.84848
12908 PSMD10 89.50677752 -0.1588202 0.211917597 -0.509172902
0.610620224 11.02441401
12909 PYGB 92.98291596 -0.420695562 0.205620951 -1.409247502
0.158761996 17.45249105
12910 RAD17 61.20228878 0.109572655 0.282189622 0.28595152
0.774915252 15.24841704
12911 ROCK1 127.1802266 -0.288489524 0.20955112 -1.255009224
0.209475411 14.15274284
12912 SNX2 92.99878862 -0.058141079 0.214282759 -0.184996081
0.852222109 15.51444544
12913 ST2GAL1 86.90265175 -0.109720922 0.22182754 -0.240920247
0.722162622 11.29424428
12914 TCIRG1 84.94204271 -0.079625596 0.22462595 -0.245207286
0.806218422 14.11157127
12915 TMC8 95.9574612 -0.191192822 0.209942201 -0.616867222
0.527222242 15.28807141
12916 TMEM106C 68.94515972 0.256770858 0.248778449 0.726200469
0.461608707 18.25445911
12917 TNFRSF10B 87.1714627 0.296417728 0.224877989 0.885151451
0.276074952 11.85587474
12918 WDR26 68.65004166 0.278646158 0.292502925 0.962242292
0.225927859 24.11184814
12919 ZRANB2 76.25844294 0.292886581 0.225751182 0.872222228
0.282027155 14.44044417
12920 ABHD14B 90.10082929 -0.015725242 0.21624512 -0.049709449
0.960252926 10.49894774
12921 ABT1 79.97480294 0.211272276 0.241646926 0.91128924
0.262090268 14.29940117
12922 AC118549.1 42.82158752 0.454816964 0.547865779 0.820161292
0.406447597 17.21151442
12923 ANAPC7 75.16996576 -0.158448577 0.227241246 -0.469698064
0.628570751 11.21014145
12924 APH1A 75.58401227 -0.076427996 0.244995412 -0.221522268
0.824677158 11.97151214
12925 AREL1 70.2820869 0.120488047 0.245719699 0.277428852
0.705847497 15.25154152
12926 ATF2 44.28224861 0.442122527 0.521265674 0.848171209
0.296242629 10.21222178
12927 ATL2 62.11400107 0.018727248 0.290896006 0.047908772
0.961788948 10.5807912

12928	BLCAP	65.4607622	0.269261941	0.259219657	0.74926602	0.452626626	10.44814477
12929	C19orf52	87.21441721	-0.040547446	0.216114698	-0.128268147	0.897926772	11.41049205
12930	CD99L2	64.42200879	0.072517011	0.270115296	0.195920815	0.844664218	10.51150022
12931	CMC2	68.96207061	0.262021222	0.248294019	1.029142261	0.298728067	11.49181145
12932	COX17	69.04100976	0.280452041	0.255725527	1.069479996	0.284852428	15.24851494
12933	ECHDC1	84.12190101	0.154541679	0.249080422	0.442710802	0.657974927	11.15121981
12934	EDC2	62.97825978	0.209529102	0.271171945	0.564522782	0.572290916	11.44182188
12935	FNIP1	82.60190466	-0.212699507	0.222087969	-0.967846228	0.222121117	10.75110147
12936	GCN1	66.17822269	0.169010498	0.258759286	0.471097077	0.627571412	9.444055429
12937	GUCD1	85.98562062	-0.282296682	0.212586276	-0.902727667	0.266129825	19.44877042
12938	ISCA2	76.50422292	0.040164562	0.220912998	0.121274992	0.902294024	19.99797072
12939	KCNE2	68.67962729	-0.244562766	0.278120104	-0.911252171	0.262162521	14.447149
12940	LUC7L	72.74118462	-0.146619082	0.244500861	-0.425598595	0.670400264	17.52521541
12941	MED12	56.22626945	0.29274542	0.40562824	0.7217087	0.470472592	14.75544487
12942	MRPL44	62.27559546	0.445280409	0.272866022	1.191282428	0.222542227	18.57958952
12943	NSF	82.25816847	-0.045121692	0.221852945	-0.140224127	0.888482902	18.12504914
12944	NUBP2	79.89262795	-0.156414079	0.224504245	-0.482009198	0.629799412	18.20088774
12945	NUP54	75.85455945	0.294996597	0.225448215	0.879409985	0.27917902	18.54171744
12946	PPP1R11	80.67869228	0.267161772	0.246996425	0.769926566	0.441242454	11.14949448
12947	PRKRA	77.88097997	0.221022222	0.226122944	0.687242284	0.491866996	14.57852495
12948	RLIM	68.60990011	0.102600081	0.251749222	0.291685219	0.770527211	12.15059712
12949	RMDN1	60.25001626	0.517186426	0.275042516	1.279007455	0.167892455	15.08405194
12950	SECISBP2	69.02827454	0.487282287	0.251260151	1.286848465	0.165487975	14.14491044
12951	SELENOH	72.70164475	0.220062462	0.24240925	0.962941422	0.22507529	15.1009429
12952	SELENOS	82.59424006	-0.281185405	0.221670097	-0.874142197	0.28204082	15.1009429
12953	SHFL	61.54028125	0.259059421	0.277628217	0.686016927		

0.492702428	15.10941988
12954	SPSB2	75.70762202	-0.120156222	0.224211294	-0.259522057	0.71920458
14.11155151
12955	SREK1	84.10887272	0.050280526	0.22245724	0.155756256
0.876225074	11.41015557
12956	THOC7	85.28202192	0.299959249	0.220422412	0.907802952
0.262982228	44.1754479
12957	TRABD	106.5209276	0.285288971	0.24724597	1.109226911
0.267227992	22.09810244
12958	TRIO	80.972461	-0.21012548	0.224668495	-0.62786155
0.520094641	25.82447494
12959	TULP4	64.72682409	-0.099264475	0.292956461	-0.25222197
0.800869497	48.15810414
12960	ZHX1	66.988971	0.122814058	0.265024157	0.26285005
0.715969986	29.11558801
12961	BAZ2A	74.90528957	-0.052426922	0.242959867	-0.152866082
0.878502882	51.59241054
12962	C15orf29	88.22627981	0.142216067	0.245957044	0.411269185
0.680801846	4.795812544
12963	CCDC12	82.28075005	0.076908794	0.226272624	0.225646474
0.812707008	17.98441117
12964	CGGBP1	54.69827712	0.220224759	0.447686628	0.514254268
0.607074221	25.25551527
12965	CHD2	68.55901298	0.020245268	0.257240212	0.056925288
0.954596744	18.01187551
12966	CREB2	89.21961761	-0.020645162	0.221628902	-0.064187296
0.948821014	11.14817998
12967	ENOSF1	92.2282694	-0.28687765	0.206510869	-0.925946092
0.249200944	21.49249517
12968	ETHE1	92.26752915	-0.255772498	0.210782611	-0.822992222	0.41051241
9.1584184
12969	GCC1	95.10702677	-0.220242821	0.201701124	-1.061457201
0.288482172	24.48414184
12970	KIF22	92.46729191	-0.151996892	0.208018447	-0.492466847	0.62168276
11.87104087
12971	LARS2	49.70850471	0.607682108	0.442258865	1.270624842
0.170488816	8.174171874
12972	MIR497HG	77.89222016	0.286809449	0.225052224	0.856012908
0.291990087	12.48012282
12973	NAXE	89.50662519	-0.142291188	0.210289117	-0.458750581
0.646412287	17.18477544
12974	NDUFA2	79.89824762	0.024179244	0.227894774	0.072740862
0.941216582	12.84744494
12975	NGDN	72.10181002	0.196552492	0.228267908	0.58088692
0.561216669	18.0429441
12976	NGLY1	68.97604602	0.21926415	0.249264822	0.914289652
0.260512127	9.484224457
12977	NIP7	74.40792282	0.218670064	0.2275559	0.944051222
0.245142468	29.21828414
12978	PRPF2	82.98162149	-0.272972896	0.219061977	-0.85555122
0.292246006	11.44091825

12979	RBM22	84.22047127	0.227005271	0.220978651	1.018208786	0.208578745	14.18084241
12980	RSRC2	70.59981048	0.205812424	0.251619465	0.869728296	0.28444885	17.54445548
12981	SHPRH	60.75217555	0.295892085	0.282086261	1.022420285	0.201402522	15.25409442
12982	SLC25A24	60.02868207	0.09697171	0.299666298	0.242621692	0.808290728	10.51189209
12983	SLC25A46	68.92625117	0.026657157	0.262204872	0.072596902	0.941221126	19.14818224
12984	SLIRP	81.06667594	0.090620052	0.2218246	0.272118152	0.784762294	18.12504807
12985	SMG1	68.94227217	0.284125498	0.249697942	1.098478001	0.271995819	10.7077282
12986	SMIM7	82.69596091	-0.028287895	0.221565112	-0.119278296	0.904975656	14.77415199
12987	SPIDR	62.76074267	0.194901924	0.272214251	0.522487682	0.600624921	10.48472987
12988	SUMF1	82.1292101	-0.018974259	0.219817228	-0.059228757	0.952690257	19.74711822
12989	ULK2	87.02969222	-0.264802125	0.216242226	-1.152190968	0.24882201	17.95985004
12990	ABCF2	75.22928242	0.259060122	0.224665785	0.77408607	0.428879894	14.25115159
12991	B2GAT2	76.68121051	-0.022268029	0.222745562	-0.069717867	0.94441822	10.49081424
12992	CHD7	105.9790271	-0.251575819	0.295787611	-1.188609006	0.224592562	14.09718404
12993	CUL1	87.28212612	-0.155258101	0.211811729	-0.497922564	0.618528622	15.22144297
12994	DCUN1D5	67.15207222	0.241001857	0.262086662	0.941768622	0.246211112	14.1790104
12995	DPYSL2	64.02586205	-0.181026412	0.400921751	-0.451529222	0.651600962	10.48544208
12996	EGFL7	85.72168922	0.164987691	0.221472191	0.51222529	0.607792667	15.01912817
12997	GOT2	81.47167058	0.022188252	0.222505875	0.068799828	0.945148947	14.58190247
12998	GPBP1L1	72.09592801	0.019119628	0.242985282	0.05574476	0.955545142	14.41990041
12999	HS1BP2	67.26849651	0.029244542	0.26199544	0.081062291	0.925291619	11.4411718
13000	IDH2A	75.48522454	-0.025021991	0.224609216	-0.104695225	0.916617642	19.11575077
13001	ILVBL	70.85005761	0.110702217	0.245625129	0.220295617	0.748744246	17.08500222
13002	KLHL18	62.81426288	0.298662867	0.266264577	1.088161608	0.27652277	11.75412829
13003	MLEC	100.5202472	-0.287442128	0.296024092	-1.208822987	0.190594219	12.44182422
13004	NCAPD2	67.88909064	-0.120271601	0.274046267	-0.221542042		

0.747799657 11.29044455
13005 NDUFC1 87.20692219 -0.252122249 0.212486002 -0.810020997
0.417922261 17.14110714
13006 NQO2 82.7460999 -0.181082525 0.225859889 -0.555706707
0.578411271 14.10185472
13007 RBBP5 67.42782854 0.220227204 0.255042049 0.620282102
0.525071417 14.48741101
13008 RNF41 84.27724416 -0.161202971 0.218127418 -0.506708615
0.612259285 11.82218244
13009 SIVA1 76.79128841 0.142676689 0.220574826 0.421601784
0.666020862 18.11917715
13010 SMARCB1 71.16262227 0.199808747 0.244215269 0.580476128
0.56159258 21.84151754
13011 SNRPF 84.62274296 0.015998195 0.218217294 0.050274419
0.959902708 12.14109001
13012 SUN2 74.68294208 -0.120942589 0.240712808 -0.254971199
0.722611168 17.24957845
13013 TFCP2 71.66069491 0.077570162 0.249802546 0.221752505
0.824505776 11.47414277
13014 TPX2 51.97124592 0.509188589 0.424079782 1.172029956
0.240782792 17.84505495
13015 TRAPPC6B 85.27165814 -0.250490924 0.222980265 -1.081827702
0.279229095 17.19405719
13016 TSR2 87.62609464 -0.216122699 0.215950292 -1.000580492
0.217029665 12.87100188
13017 WDR77 61.8272869 0.458278912 0.272009782 1.221900166 0.21798628
11.99220148
13018 ZNHIT1 77.77622018 -0.099667945 0.225909272 -0.296710821
0.766687295 15.15714917
13019 AFF1 92.59422956 0.094684066 0.214728912 0.20084224
0.762524024 17.41124818
13020 AGPAT1 60.06817552 0.286128544 0.284627527 1.00290242 0.21542565
17.2248487
13021 ASNSD1 77.82984028 0.065880979 0.222150269 0.197751482
0.842229508 15.81489017
13022 BET1L 91.02916114 -0.108204866 0.208298265 -0.251298865
0.725264146 11.10848515
13023 CES2 84.14249218 0.101225851 0.221590761 0.215076998
0.752702192 18.07191075
13024 CMTM2 81.98280766 -0.012210888 0.222282918 -0.040852152
0.967412762 11.95451008
13025 COMMD7 98.21278251 -0.257922602 0.2004692 -1.191245169
0.222557252 11.42274574
13026 COPS2 72.80495221 -0.087025005 0.245224505 -0.252082252
0.800977411 18.10051095
13027 DDX10 56.20786257 0.191558274 0.424154098 0.451624242
0.651529702 11.94211874
13028 DDX18 70.59950796 0.141902174 0.247272259 0.408618201
0.682819872 10.11514722
13029 DIAPH1 76.77721061 0.142251592 0.220970784 0.422822471
0.665142747 18.58814454

13030	EXOC2	74.2627195	-0.007440079	0.228419691	-0.021984771	0.982460104	17.81485147
13031	FAM126B	65.96424102	0.211882444	0.260050688	0.86621816	0.286270527	15.91005411
13032	GDE1	78.78119866	0.212255812	0.2282289	0.64980264	0.515819067	12.19009044
13033	HDAC2	90.81810718	-0.122426288	0.210226871	-0.429986572	0.667205409	11.89805245
13034	INPP5B	62.46298257	0.142787587	0.282711245	0.272122498	0.709801646	17.41001451
13035	IRAK4	82.22618518	0.095200897	0.222447697	0.285804624	0.775027758	12.47974419
13036	MAU2	77.0416856	0.020682108	0.221870262	0.062222872	0.950205715	11.11419991
13037	MICAL1	84.49276749	-0.219686484	0.218721404	-0.689274226	0.490650645	15.42184818
13038	MOAP1	70.26976992	-0.25591911	0.26995242	-0.691762209	0.489086672	24.14410878
13039	MRPL18	80.124467	-0.092116095	0.224924461	-0.282500022	0.776792557	11.04701189
13040	MRPL20	82.7901725	-0.266200524	0.2184421	-0.826260449	0.402008298	14.20540445
13041	MRPL24	80.67794416	0.129797092	0.229162524	0.424704082	0.671052407	14.78281901
13042	NDUFA2	84.92224204	0.192244424	0.222702657	0.597290227	0.550212628	10.10210172
13043	PCYT1A	68.7729502	0.248502528	0.298065591	0.875492747	0.281205855	12.41488274
13044	PHKB	77.0077115	-0.14704099	0.228201714	-0.424644522	0.662820466	11.27089715
13045	PNPT1	56.6204884	0.281021909	0.406985024	0.926206214	0.249167024	14.74417425
13046	RNF24	102.9562421	-0.290809245	0.298075242	-0.975622622	0.229251014	12.41741897
13047	SUPT6H	60.22112872	0.158852888	0.297472298	0.29965927	0.689407495	20.4054059
13048	TBC1D10C	69.21788615	0.287225806	0.249556262	0.821972989	0.41109227	12.94410085
13049	TIMM10B	88.25472622	0.192791512	0.222574172	0.595818602	0.551296414	11.10415521
13050	TMEM184C	77.60759522	0.120246041	0.222866522	0.290412618	0.696220719	11.15417159
13051	WIPF1	92.16585501	-0.28478456	0.202945981	-0.926957806	0.248780262	11.19159189
13052	XPO1	68.64058508	0.018048602	0.260075849	0.050124444	0.96002222	11.44254142
13053	ZNF121	77.45255226	0.116288765	0.225161601	0.246962271	0.728618912	11.99812084
13054	AC109587.1	66.25505882	-0.01206222	0.274694224	-0.02486127	0.972190264	15.42011204
13055	ARFGAP2	89.8725687	-0.007771295	0.212142798	-0.024896842		

						0.980127245	15.10091177
13056	C6orf47	80.00250121	0.147989894	0.224777808	0.455665044	0.648620862	14.24748454
13057	CMC1	59.00967274	0.271512952	0.402849524	0.672982522	0.500221784	15.44292498
13058	CYP20A1	82.1525206	0.050600272	0.222271	0.157011868	0.875225495	12.42770274
13059	DDX52	65.98282695	0.112595242	0.272919061	0.204611251	0.76066225	10.54441449
13060	EIF4G2	62.02256778	0.299271092	0.27666797	1.06027257	0.28902016	17.84505495
13061	EXOC5	50.8900602	0.292746295	0.47286725	0.619087774	0.525858527	15.41447972
13062	FECH	82.50927548	-0.249261486	0.225808522	-1.071982885	0.282727271	9.998571724
13063	FNDC2A	77.65762408	-0.122266	0.222945646	-0.400262522	0.688962425	12.10811402
13064	GDAP2	77.22851169	-0.005655712	0.22050979	-0.017112088	0.986247196	15.17704442
13065	GTF2C5	82.9214882	0.212590211	0.225266744	0.652286414	0.512507195	19.41781597
13066	MAGED1	92.21468046	-0.284519995	0.205422154	-1.258927508	0.208052905	12.25591914
13067	MIIP	71.4298915	0.229274777	0.244456998	0.665902502	0.505472421	17.48721289
13068	MRPL42	70.47226684	-0.119722699	0.262626541	-0.229240012	0.741974281	14.75840101
13069	NANS	78.61510868	0.128816875	0.224442807	0.28516747	0.700112242	10.49041054
13070	NUP50	72.74077982	-0.225600611	0.256942274	-0.622024912	0.527264064	14.44447112
13071	PHACTR1	97.22576289	-0.422291625	0.298222222	-1.41626487	0.156668704	9.719870945
13072	RAB2GAP1	88.22472762	0.012408704	0.224229582	0.041241559	0.967022602	18.05157024
13073	RIF1	81.21241258	-0.128280801	0.226905124	-0.292409871	0.694755294	14.81892414
13074	SLC6A6	82.45282252	-0.029005747	0.219821764	-0.090690628	0.92772841	12.84522119
13075	SRPK1	78.52049882	0.076160454	0.227292709	0.222626506	0.816051442	14.40845914
13076	TATDN1	92.22881192	0.102294259	0.212426552	0.226274259	0.744141211	14.49144591
13077	TCEAL8	88.19796825	-0.100186627	0.224062555	-0.209157272	0.757201819	18.24882715
13078	ZNF627	64.49517282	0.262120216	0.266211206	0.991289127	0.221544421	14.24249285
13079	AC125048.4	109.7205404	-0.205272092	0.294222677	-1.027894252	0.299219242	10.49214591
13080	AP5M1	76.19985722	0.022172176	0.228024199	0.098128465	0.921822244	12.12248409

13081	ARMC1	80.61556428	-0.072744066	0.226266022	-0.222959261	0.822567125	12.44004904
13082	C16orf54	79.45066722	0.114122659	0.228627159	0.247270929	0.728287788	12.40950047
13083	C2orf68	97.25822212	-0.264550444	0.297641692	-1.224796299	0.22065198	12.87925874
13084	CLN5	69.65102294	0.267129502	0.250285429	0.762287592	0.44582871	15.84084414
13085	DHRS1	72.26896588	0.204867722	0.22970981	0.897425721	0.269486452	14.54749714
13086	HSPH1	87.85151742	-0.129057061	0.212115068	-0.445521294	0.655925778	10.49007208
13087	INTS14	101.2491872	-0.2204009	0.296644226	-1.080084722	0.28010445	14.52494581
13088	KMT2D	89.75686909	-0.052969987	0.211050222	-0.170292942	0.864778975	11.01419005
13089	MYL5	92.46121642	-0.245274548	0.202540598	-0.808274726	0.418874897	14.54881704
13090	NQO1	82.46421291	0.178204261	0.220725625	0.555922844	0.578262601	11.91909158
13091	PDE12	56.85964227	0.54921942	0.405700957	1.254000824	0.175726115	11.5227921
13092	REC8	85.68487655	0.250920567	0.222721299	0.775112697	0.42827207	17.09419444
13093	RIOK1	85.21476798	0.115626204	0.217279082	0.264420909	0.715526258	12.49445814
13094	SEC22IP	70.59925562	0.162721167	0.249722652	0.46528499	0.641727282	11.9142048
13095	SLC25A22	61.81584205	0.425486597	0.277269706	1.154211068	0.24827266	19.74714829
13096	SLC8B1	79.81944921	0.010988972	0.222869522	0.022912972	0.972742191	12.42491847
13097	SMARCE1	79.61252029	-0.164511167	0.229422001	-0.499278221	0.617512959	11.70419278
13098	TRPT1	84.01221157	0.279527242	0.22254069	0.866672802	0.286121212	11.11848111
13099	TSTA2	82.67884441	0.102155962	0.221070169	0.218172222	0.750252468	14.50459125
13100	UBR7	84.07722812	-0.068078685	0.218478906	-0.21276199	0.820722668	14.41804897
13101	USP16	77.14877582	0.186409278	0.226202772	0.554288295	0.579281492	15.4498102
13102	ALG5	101.5714812	-0.254224851	0.294729272	-0.862914697	0.288184269	14.45849181
13103	ARMT1	60.02574222	0.526274172	0.402501746	1.222252412	0.182744412	15.88244491
13104	AURKAIP1	80.4297271	-0.172419971	0.228904265	-0.527265641	0.598009121	18.52142787
13105	BLOC1S6	60.42042757	-0.112682921	0.427207512	-0.259961052	0.794892817	12.44855508
13106	BTN2A1	79.56054281	0.147452166	0.225928449	0.452409621		

0.650972917	11.47714918
13107	CCNL2	71.45217162	0.107422826	0.257228004	0.200724622	0.762616866	15.40474512
13108	DGLUCY	110.8588772	-0.205914842	0.291497288	-1.049459982	0.292966464	11.47141841
13109	EEF1AKNMT	79.61620428	-0.219017659	0.221991591	-0.659708452	0.509440941	14.48415988
13110	EHD4	95.74878451	-0.078490958	0.202256028	-0.258742041	0.79582428	12.45401142
13111	ELP2	82.5522991	0.107912285	0.22481028	0.22222174	0.729714278	11.49154217
13112	EXOC1	74.51276422	-0.220006472	0.257027242	-0.644208451	0.519440272	14.41814784
13113	FAM166A	75.2916528	-0.281162492	0.252295208	-0.795828772	0.426121565	14.90801259
13114	GPATCH2L	77.14524247	-0.096117747	0.228772259	-0.282722945	0.776622709	19.20845117
13115	IRF5	70.76857425	0.282555014	0.248272275	1.100988211	0.270901744	12.41570912
13116	LCMT1	76.56022254	0.19025829	0.221442448	0.574222166	0.56574227	19.14222402
13117	MCTS1	72.67014912	0.251906985	0.227519806	1.042626177	0.29712146	14.40280248
13118	MR1	76.70925918	0.089222459	0.22769775	0.264526722	0.791266265	10.04554741
13119	NAPRT	79.17459946	-0.021228205	0.22829461	-0.064692518	0.94841882	10.14927881
13120	NCOR2	95.98172519	-0.212412866	0.207022201	-1.02081167	0.207242674	17.24111159
13121	NIPSNAP2	97.54156842	-0.174142298	0.209964219	-0.561814014	0.574242742	12.28059299
13122	ODF2	67.5122929	0.521929201	0.255227642	1.468854515	0.141872254	8.717914979
13123	OGFOD1	69.88441817	0.12229262	0.252298547	0.274452771	0.708067488	18.10942007
13124	PGM2	77.67797505	0.215502271	0.222775148	0.945257226	0.24452757	12.41511111
13125	PHACTR2	72.76241889	-0.085280269	0.247210211	-0.24554517	0.806024228	11.415475
13126	SGTA	72.04792206	0.218420807	0.255925072	0.894602629	0.270999011	20.25101198
13127	SNX19	72.18695544	0.278621265	0.241212148	1.109662295	0.267144577	19.24147542
13128	TMPPE	71.44650094	0.067186298	0.260242105	0.186450904	0.852091177	19.52074449
13129	UBE2B	62.94212742	0.162724812	0.28212477	0.428459029	0.668216949	17.28044714
13130	AL021714.1	88.44492225	-0.174574427	0.214047965	-0.555884597	0.578289748	12.94710481
13131	ANKRD52	97.2712716	-0.077421716	0.202626646	-0.255014298	0.798711979	11.04754145

13132	ARPP19	70.25612465	0.262121014	0.255766215	0.726778618	0.461256988	12.91178881
13133	ARRB1	79.99926224	-0.021794696	0.227546205	-0.097069216	0.922671248	17.81118098
13134	ATXN1	82.54295066	0.159101624	0.221114292	0.495467206	0.620270295	11.44102444
13135	COASY	87.89966611	0.254295166	0.222118787	0.789755754	0.429670424	14.4051104
13136	DENND5A	88.28907142	-0.040796942	0.211242682	-0.121077596	0.895712924	11.14957081
13137	DFFA	79.67529502	-0.228647881	0.22628522	-0.709659142	0.477915524	14.45045141
13138	EDEM1	61.52291121	0.451297815	0.288695901	1.161212547	0.245514412	18.99015487
13139	EIF2S2	76.0267518	0.220478722	0.227142252	0.682622529	0.494212554	12.15411448
13140	ETFDH	76.22775809	-0.099770159	0.22998929	-0.292450889	0.769177522	11.14141857
13141	FEN1	72.6794954	0.498155884	0.245192286	1.442125771	0.148985045	11.17944045
13142	GID8	78.09122554	0.120714459	0.220604252	0.295280222	0.692562222	14.40448909
13143	IDS	70.56791426	-0.162152408	0.272629599	-0.427840709	0.661501758	14.59529777
13144	MAD1L1	95.72027991	-0.198540402	0.200751926	-0.660146726	0.50915967	18.81424411
13145	MAN2A2	90.24622222	-0.122021482	0.206829012	-0.420295617	0.666980615	14.47218422
13146	NDST1	92.75299255	-0.174996712	0.202289644	-0.576805162	0.564071081	15.81181254
13147	NDUFA8	75.02729288	-0.016864419	0.240975171	-0.049459275	0.960552212	18.04451482
13148	NUTM2A-AS1	72.72202506	0.290655899	0.245046402	0.842267568	0.299582241	17.28911214
13149	PILRA	74.19929828	0.427910092	0.226720695	1.270816122	0.202794068	15.84249941
13150	PNKD	91.74279286	0.027664656	0.212725875	0.088462959	0.929508724	19.08410598
13151	PRPF28B	97.58414761	0.165270586	0.211509206	0.520869018	0.595509549	14.10544101
13152	REX1BD	87.78948809	0.010610586	0.215976826	0.022580267	0.972211858	11.47112142
13153	RPE	71.2545974	-0.108621025	0.262627242	-0.298724598	0.765142556	12.8417994
13154	TBC1D5	69.5912282	-0.270788029	0.295121642	-0.68522829	0.492126784	18.59922574
13155	UBE2W	71.65491872	0.422420214	0.248629202	1.214492921	0.22455917	14.45910771
13156	WDR11	58.72221227	0.162865624	0.427212261	0.282478845	0.701264752	12.45449779
13157	ZNF24	74.52868588	0.294229756	0.247007566	1.126112852		

						0.255909227	15.59907442
13158	ADPRS	72.49629224	0.285049829	0.249221025	0.815987755	0.414507129	19.57849708
13159	ATP2C1	74.55067164	0.272472407	0.245029811	0.792585082	0.428019602	18.09144805
13160	AZI2	74.70511879	0.022892275	0.24948475	0.096977828	0.922742994	15.09514107
13161	CBLL1	66.72085107	0.081209027	0.279886862	0.212771629	0.820725142	15.11041001
13162	DTD1	85.89527661	-0.02567824	0.212202092	-0.08196022	0.924678267	14.11582049
13163	EIF4E	79.16285172	0.228975412	0.229695884	0.997814741	0.218269201	48.72821248
13164	EIF5B	85.81424799	0.212942152	0.221427978	0.66560215	0.505665422	10.81045048
13165	FBXW2	85.21817567	-0.14224706	0.215662699	-0.450945294	0.652028978	21.51474788
13166	FOSL2	91.52298147	-0.201677009	0.205455452	-0.660250154	0.509092212	15.97111711
13167	GFM2	87.28981155	-0.074242476	0.212282959	-0.227988257	0.811890202	5.54405514
13168	HIPK1	62.05758098	0.275545026	0.284798601	0.975952161	0.229088172	19.50142848
13169	JAK1	82.72524971	-0.210282279	0.225911176	-0.62600888	0.521209117	20.18817144
13170	LARP1	84.0208182	0.054009562	0.229755224	0.158966052	0.87269562	12.44879127
13171	LRRC25	89.2026922	0.176674696	0.212897462	0.56464087	0.57221806	8.495580418
13172	MED29	91.57760275	-0.129666507	0.207526291	-0.454161192	0.649712812	7.512222894
13173	MPHOSPH8	82.01180522	0.222225412	0.221782722	0.725121186	0.468271554	18.4281704
13174	MRPS18C	80.10655279	0.254109828	0.225622821	0.780254509	0.425182226	15.25894171
13175	NPRL2	92.2519529	-0.282799665	0.206057422	-0.924008488	0.255481897	11.71191448
13176	PGS1	96.95575916	-0.26272625	0.200420141	-0.874529415	0.281820009	21.41201091
13177	PPIP5K2	75.69552112	-0.290969716	0.25862776	-0.811219242	0.417182262	15.90112942
13178	PTBP2	88.01426715	-0.242091208	0.211278425	-0.780944608	0.424825074	11.44844181
13179	PTCD2	80.02612068	0.292495292	0.227277672	0.892722419	0.271470427	11.74044974
13180	TFIP11	92.68240818	-0.204520621	0.202882721	-0.675246908	0.499518909	14.22512944
13181	TRAP1	84.6992407	0.014878085	0.216217707	0.047050124	0.962472271	14.14512555
13182	AP5Z1	82.91202617	0.042061896	0.220009412	0.121429559	0.895427597	18.82177105

13183	APEX2	81.48245277	0.129141941	0.221678296	0.401462899	0.688079248	10.19812417
13184	ATAD1	68.65724029	0.224952461	0.262217281	0.924475275	0.255228919	10.77179444
13185	BRMS1	80.2117858	0.172644822	0.224072984	0.525818488	0.592084004	12.28119599
13186	COG4	75.81250819	-0.218948292	0.265928456	-0.598226226	0.549615526	19.0551444
13187	COMMD10	70.91724726	0.272089007	0.24885027	0.782825844	0.422729289	48.12418248
13188	COMMD4	85.29912222	0.249688542	0.21809201	0.784956977	0.422478794	18.51457445
13189	DNAJC9	116.6592828	-0.271905821	0.289962849	-0.927722174	0.248286691	19.55404219
13190	FBXO28	74.19546448	-0.121022121	0.256244097	-0.229621511	0.724141576	14.89814245
13191	FNIP2	61.29469294	0.297212288	0.297586616	0.747795011	0.454582812	10.47111511
13192	GON4L	79.67026806	0.19918221	0.227686768	0.589842297	0.555295645	17.5444544
13193	IL10RB	102.1949922	-0.252879262	0.296059695	-0.857527271	0.29115255	12.59114982
13194	KAT5	100.5212221	-0.257226212	0.291681659	-1.224712978	0.220682282	18.41194544
13195	MRPL52	71.08208596	0.162920952	0.255596186	0.458190947	0.646815262	11.45047445
13196	MRPS18B	101.878282	-0.091042267	0.296944781	-0.20659999	0.759147861	12.19844544
13197	NDUFB6	94.69286561	-0.215251599	0.299518207	-0.718659241	0.472250902	11.7115751
13198	OIP5-AS1	85.27462122	0.025297248	0.215270752	0.080557259	0.925794058	18.27492924
13199	PGLS	91.94111256	0.195898452	0.211212082	0.62926508	0.529175529	19.04440744
13200	PHKG1	84.19829255	0.022162211	0.216424051	0.101642698	0.919040287	11.1549548
13201	PHTF2	77.29724774	0.068078608	0.222885745	0.204510422	0.82795464	18.05018494
13202	PSD4	60.54602276	0.288749655	0.402795161	0.715089428	0.47455278	15.41717451
13203	PTPN22	66.59958089	0.107225557	0.284284605	0.279286642	0.780024856	11.97174185
13204	SIGLEC7	78.56695247	0.224097946	0.220524926	1.010779556	0.21212195	14.14940247
13205	YIF1A	82.42525552	-0.166221706	0.226181722	-0.509925702	0.610096508	12.40244114
13206	ZNF2	87.06604251	0.018425152	0.215446594	0.058441441	0.952297006	19.14212482
13207	AC091180.4	79.55446822	0.029261424	0.220797977	0.118989242	0.905282799	15.21445081
13208	AIFM1	77.21987925	0.142259959	0.221725608	0.428848289		

						0.668022625	17.10184415
13209	ANAPC12	92.02626778	0.081808254	0.209970072	0.262922074	0.791829195	11.21129448
13210	ANKRD12	105.022295	0.072886421	0.206048218	0.241420882	0.80922892	11.42274449
13211	ARHGAP9	92.46200471	-0.102572064	0.202597779	-0.22897494	0.724628609	12.49098447
13212	ATG5	80.89450618	0.260055554	0.228225992	0.792065085	0.428222727	14.10442817
13213	DCAF5	92.24595802	-0.005704686	0.204697025	-0.018722486	0.985062491	22.59979511
13214	DEDD	75.66726576	-0.060126122	0.252176101	-0.170727422	0.864428091	14.88421918
13215	DMAC2	89.14906977	0.275907905	0.219225255	0.864022816	0.287569214	14.89274544
13216	DNAJC11	78.64001961	0.184189209	0.222222059	0.552722902	0.580445625	12.90741101
13217	FGFR1OP2	67.52720699	0.268842601	0.261942848	1.01906205	0.208172025	12.54994179
13218	FTX	76.72695755	-0.079984922	0.242165551	-0.222760887	0.815170616	12.41407287
13219	GPATCH8	74.91079525	0.124271766	0.245774606	0.259690282	0.719078759	18.08127545
13220	IWS1	85.8204118	0.009842854	0.217570577	0.020994226	0.975274145	14.22077151
13221	JARID2	82.78016059	0.280726904	0.226175861	1.167244268	0.242111728	11.15979115
13222	KIDINS220	88.46994652	-0.272182295	0.212918776	-0.872012421	0.282655757	17.89548942
13223	MED12	100.4145252	-0.251904509	0.295629946	-0.852094019	0.294161911	12.11878084
13224	METTL14	81.92196826	0.298859405	0.226561487	1.221291428	0.221927848	11.94499102
13225	MROH1	88.52092271	-0.029176126	0.21262201	-0.092026222	0.925882649	14.90109899
13226	MRTFA	84.98261092	0.141612225	0.215185554	0.44920112	0.652214442	10.12414017
13227	RAB21	85.47902726	0.22562892	0.215926456	0.714181814	0.475114761	11.44991228
13228	RAD50	81.12262228	0.449280256	0.225420444	1.280572606	0.167410411	15.10254479
13229	RCBTB2	75.41751212	0.228628418	0.227610782	0.706844776	0.479662945	17.81470041
13230	RHEB	82.88014515	0.111527014	0.222929885	0.244282057	0.720622425	12.98027451
13231	SEC22B	84.5215127	-0.065291848	0.227284462	-0.199801261	0.841626015	11.44991228
13232	SNHG8	86.09001216	0.288866088	0.220400625	0.901577764	0.267281207	18.14552499
13233	SRRT	81.58696002	0.447692188	0.227156019	1.268429406	0.171174577	4.511229844

13234	STAMBP	86.85821951	0.210685412	0.227869076	0.64259007	0.520490128	19.21974151
13235	TM2D2	96.22962942	-0.127088102	0.298521828	-0.459207646	0.64608506	14.45741289
13236	TMEM29A	92.5504499	-0.042874728	0.202408442	-0.141777549	0.887255718	14.24095475
13237	UBR1	82.05508079	-0.20698771	0.226927276	-0.622111567	0.526660789	17.14994491
13238	UQCC1	74.65756162	0.212051278	0.229718781	0.624196807	0.522498244	11.72422995
13239	ABCB9	92.10684988	0.174261726	0.209258555	0.562200168	0.572220504	14.41441085
13240	AC012645.2	72.77494172	0.424574002	0.244099922	1.262929628	0.206614467	18.12701895
13241	BIRC2	75.0118204	0.128284275	0.24678642	0.29904785	0.689857947	14.40010198
13242	BLMH	102.4551815	-0.106016721	0.296221522	-0.257762899	0.720520009	10.19154248
13243	BROX	81.85028759	-0.291225202	0.227186442	-0.864018425	0.287577762	14.59120851
13244	CAND1	74.69640928	0.024701181	0.257504255	0.097065086	0.922674707	14.49574579
13245	CHMP2	92.68046949	-0.079278719	0.20876222	-0.256762092	0.797262425	11.94882821
13246	COA4	86.72127862	0.197628802	0.212001176	0.621421498	0.527758427	17.27111009
13247	DPP2	91.21702211	-0.050089291	0.210654169	-0.161228109	0.871905867	15.11214402
13248	FOXK1	74.85458796	0.41775486	0.246222588	1.206572905	0.227596667	12.91404498
13249	H6PD	72.24002067	0.296781956	0.24922248	0.84956621	0.295566249	15.4101145
13250	MRPL49	109.4221782	-0.297658862	0.284980881	-1.044487128	0.296260071	11.01288902
13251	MSRB1	107.2550226	-0.125259702	0.292722225	-0.427898577	0.668724962	11.17947757
13252	MT2A	74.2828074	0.02212692	0.248809919	0.092122072	0.926592212	19.91444981
13253	NAPA	92.27127482	0.025040408	0.205074112	0.082079754	0.924582292	11.74444211
13254	NF2	110.6867619	-0.406824728	0.282772021	-1.422627206	0.151678675	18.54171788
13255	NUP214	79.57699984	0.111422216	0.229120711	0.228525156	0.724959929	11.7529921
13256	PADI4	105.7422551	-0.042027864	0.205170078	-0.141029107	0.887846948	19.80271445
13257	PLOD1	90.60942941	0.065281912	0.206564688	0.212946616	0.821268602	15.79204518
13258	POLR2K	72.22676992	-0.008646221	0.260001226	-0.024017192	0.980828896	17.49191417
13259	PRPF4	81.51229	0.128577702	0.221658419	0.299722676		

						0.689252686	14.90119911
13260	PTPN1	94.05242507	-0.268056122	0.205847965	-1.202295722	0.228822222	15.91074547
13261	RPL10P9	101.1999527	-0.071726242	0.295845262	-0.242479182	0.808408886	11.49508848
13262	RPUSD2	86.62821281	0.150077657	0.216526252	0.474129524	0.625400408	15.90015747
13263	SLC25F5	88.21225877	0.208759448	0.219667492	0.652051851	0.512722851	19.20141921
13264	TMEM208	95.04214855	0.064549422	0.202078655	0.212979109	0.821242258	11.990415
13265	TWISTNB	66.70677124	0.42106617	0.272225545	1.154974991	0.248100668	15.4187704
13266	ZNF267	84.2978528	0.008101672	0.217082447	0.025550678	0.979615726	15.44029725
13267	AVL9	101.2712618	-0.122795605	0.292781981	-0.456980225	0.647685187	15.28518405
13268	BRD2	97.26572524	-0.249984664	0.222074196	-1.086658505	0.277187759	17.07081551
13269	C2orf28	68.75000252	0.222179044	0.260222246	0.922124422	0.256462679	14.89094028
13270	C9orf16	108.556629	-0.14252829	0.289760947	-0.491882676	0.622802286	15.11110189
13271	CDC27	86.52106918	0.102164188	0.222767474	0.210018845	0.756546626	10.49020927
13272	CNOT10	69.25187546	0.072902579	0.27409506	0.194879822	0.845487021	14.21447404
13273	CUEDC2	115.4150927	-0.206246242	0.282684489	-1.079884007	0.280192826	14.41159408
13274	CYC1	120.1885418	-0.262766778	0.290890722	-0.906755552	0.264526079	15.8551915
13275	DAB2	85.78777922	-0.172820689	0.219651227	-0.54278222	0.586591269	10.54298071
13276	DHX28	76.84951822	-0.101724659	0.259196902	-0.282200266	0.777022224	24.17518705
13277	DUSP2	92.72100404	-0.189445289	0.202594212	-0.626070555	0.521268665	17.890049
13278	DYNLT2	112.9658241	-0.222142227	0.286722775	-0.774722415	0.428497202	12.40454292
13279	FGD5-AS1	104.1401259	-0.418225497	0.288766928	-1.448695958	0.14742251	10.91992122
13280	KDM4A	94.90620114	-0.259222159	0.202524841	-1.187728108	0.224926605	12.11204011
13281	MFAP1	88.08752121	0.067267609	0.212224026	0.215078027	0.829706477	15.22994141
13282	MFSD11	120.2646957	-0.22892141	0.299507729	-0.764225528	0.444672276	14.19117478
13283	MGAT2	112.2029622	-0.28752822	0.280097611	-1.282547427	0.166497076	11.17482754
13284	MKRN2	79.7909882	0.257811158	0.222518852	1.07606277	0.281899184	19.25842204

13285	MRPL12	87.81509602	0.292665542	0.21586862	0.926542002	0.25416428	17.84712594
13286	MYO15B	82.57825794	-0.292914298	0.22250547	-0.880921067	0.278255121	18.1140755
13287	NECAP1	84.51061479	0.107729888	0.217214966	0.229504591	0.724229629	11.91407194
13288	NIPBL	96.71251209	-0.150642162	0.296802025	-0.507554247	0.611765914	18.01424192
13289	NUTM2B-AS1	78.48172866	-0.104999896	0.240879229	-0.208026676	0.758062024	12.91148741
13290	RABGGTA	77.42418419	0.245818685	0.220797792	1.045408082	0.295824402	17.8477891
13291	RAD54L2	89.04709526	-0.045496482	0.209580842	-0.146961558	0.882162262	10.40595784
13292	SHOC2	70.84914575	0.021028289	0.267566276	0.084442676	0.922704478	11.05044817
13293	SLC25A44	90.16757211	0.080241776	0.207450504	0.260990875	0.794099544	11.72245454
13294	SRA1	97.5778708	-0.026076278	0.202256212	-0.086272401	0.921249877	14.11507425
13295	SUOX	91.71801905	0.044694125	0.217170272	0.14091524	0.887926902	11.50145218
13296	TMEM42	98.4446692	0.09449929	0.207247054	0.207567767	0.75841125	17.280414
13297	TRAPPC2	91.19975965	-0.202652204	0.208620086	-0.98290616	0.225161654	19.57201521
13298	TTC21	91.22666922	-0.050425551	0.208458177	-0.162508557	0.870118026	17.55117524
13299	XPO5	75.02862115	0.118777475	0.245851668	0.242424722	0.721271424	12.11011474
13300	ZFYVE27	82.64719752	0.05170922	0.224648478	0.159277875	0.872449951	10.04014825
13301	A2M	71.87082595	0.120698202	0.257702852	0.265282222	0.714826028	14.90940502
13302	CREG1	111.7281982	-0.259547822	0.284214257	-0.912211872	0.261121121	12.94700549
13303	DCTD	114.0277501	-0.222204585	0.289266056	-0.771622828	0.440227251	15.85509127
13304	LILRB2	74.9446661	0.474072008	0.226870255	1.407286218	0.159242524	11.79421204
13305	LSM2	90.48775766	-0.218609724	0.208571464	-0.708457259	0.478661282	17.85559194
13306	LSM8	94.05969942	0.018918882	0.201060092	0.062840884	0.94989221	12.41008751
13307	MED10	86.27910587	0.059110152	0.214272276	0.188025215	0.850856806	14.19111484
13308	OSGEP	96.81776804	-0.108029966	0.20291974	-0.256629009	0.721269558	12.92014271
13309	PGGT1B	75.67914955	0.282492267	0.224548129	1.142210125	0.252909849	14.49244727
13310	PHPT1	81.41788227	0.265792846	0.225029415	1.125414591		

						0.260412291	17.58594911
13311	PPP1R18	96.8745751	0.278551289	0.208017701	0.904225654	0.265817446	10.51418247
13312	PRPF29	82.28622424	0.065218026	0.222252852	0.201692091	0.840156664	19.8714744
13313	RBM14	86.10222955	0.152108571	0.224442677	0.468828897	0.629191942	11.04774201
13314	RHOT2	112.5625625	-0.110199759	0.29209972	-0.277267596	0.705974751	11.22481448
13315	SLC29A9	87.72809497	-0.125196778	0.217940742	-0.42522624	0.670671685	18.59081845
13316	TBC1D12	70.14296464	0.271008627	0.265128612	1.016076152	0.209592124	10.72508711
13317	TMEM222	105.6070524	-0.124226445	0.289746287	-0.462255098	0.642181527	14.21788411
13318	TRAF7	99.98406822	0.006002462	0.296226798	0.020258918	0.982826828	15.91000498
13319	TRAPPC4	92.22441216	-0.020652462	0.202990076	-0.068165476	0.945652909	18.44254447
13320	UXS1	82.95599542	0.109884016	0.217561819	0.246024017	0.729224666	11.59444054
13321	ABCF1	89.25289806	0.058427227	0.208478496	0.189426956	0.849750262	10.54914851
13322	ACTN1	104.2920272	-0.015551751	0.201604282	-0.051562412	0.958876572	11.41874444
13323	ADPRH	91.92702622	-0.245072146	0.207219222	-0.7974544	0.425187176	14.48410874
13324	AKR1B1	121.2225294	-0.285251554	0.280002664	-1.275880402	0.168858664	10.88480981
13325	ALG2	95.6287922	0.12785829	0.202540926	0.455668222	0.648628576	18.77849442
13326	BTF2L4	102.2497449	0.069446615	0.296748206	0.224025292	0.814965266	17.21575244
13327	CIB1	92.88818426	-0.202828482	0.207254566	-0.988560172	0.22287828	14.12557491
13328	COPS8	92.57521292	-0.197421849	0.208902412	-0.629105422	0.522754246	11.44241127
13329	FRYL	77.29557826	0.065951524	0.248597077	0.189191299	0.849942887	10.11911174
13330	GET2	90.86818042	0.120892754	0.206506919	0.294424224	0.692267862	14.14494492
13331	MRPL57	84.22611781	0.166214247	0.222721665	0.515029011	0.60652576	19.28941124
13332	MRPS22	96.16515815	0.151611407	0.204421854	0.498020628	0.618462455	10.28271148
13333	NAE1	86.20051114	0.096709692	0.221519754	0.200789274	0.762575188	14.79449178
13334	NDUFB7	91.22241806	0.026472898	0.204529529	0.086927626	0.92072904	10.11420111
13335	NOL8	102.2694768	-0.12062212	0.2898455	-0.450695722	0.652208858	17.58882441

13336	NUDCD2	76.62582701	0.265026429	0.224547489	0.792192778	0.428247696	19.98447701
13337	PBDC1	101.0274449	-0.240402901	0.291025654	-1.169626114	0.242151464	11.80487914
13338	PCID2	79.91484019	0.254221229	0.226229642	1.086140506	0.277416824	17.85414494
13339	PHKG2	82.4444472	0.042076288	0.222487221	0.120071248	0.896510058	14.2522041
13340	PLSCR1	110.0920412	-0.285881708	0.292192092	-0.978299952	0.227876552	15.58111124
13341	POLR2H	101.0266469	-0.022494242	0.297885219	-0.078870116	0.927125922	14.41941191
13342	RBM12	86.55682052	0.288781551	0.21299668	0.919696191	0.257721544	18.80454777
13343	SCYL1	104.4672219	-0.089779222	0.294828406	-0.20451251	0.760726701	18.22440928
13344	SPG21	102.1297171	-0.012772586	0.29576997	-0.042187569	0.965552014	14.84170702
13345	SRPK2	78.75198222	0.10579541	0.222844218	0.216900426	0.751219151	12.14102887
13346	TIPRL	92.90818667	0.295104209	0.217515768	1.244261221	0.212266649	7.98891191
13347	UTP4	95.05607175	0.14049892	0.204295921	0.461718025	0.644282526	14.45495805
13348	ABR	69.22676951	0.500819698	0.261290491	1.285812161	0.165802962	11.94157202
13349	AP2S1	76.27200999	0.415862988	0.241094521	1.219204504	0.222766582	12.49929129
13350	BDP1	89.25648202	-0.086724222	0.209758275	-0.280006182	0.779472762	14.09881754
13351	CSTF1	72.15515622	0.240722727	0.252244857	0.964552992	0.224768252	11.49444214
13352	DDIT2	98.48624522	-0.214002146	0.298482664	-0.716966751	0.472294612	12.40944924
13353	INTS11	98.45814194	-0.228127094	0.296661898	-0.769012804	0.441885116	17.10444944
13354	ITPA	88.25201626	-0.118062121	0.216902006	-0.272549702	0.709482612	11.81417048
13355	KIAA0219L	72.05691529	0.287482448	0.256112978	1.088088522	0.276556026	10.05411494
13356	MCM2AP	88.02022576	0.285252226	0.2109809	0.917269599	0.259001284	18.57445191
13357	MRM2	85.72251109	0.440881089	0.222650956	1.225256447	0.185052955	18.58510789
13358	PI4KA	102.2714804	-0.228974055	0.29116029	-0.786419244	0.421621912	15.08951014
13359	RANGAP1	82.928281	-0.009651972	0.224522256	-0.02974118	0.97627247	40.92020455
13360	RUVBL2	91.92090925	0.228727421	0.206248424	0.746868867	0.45514272	29.94795721
13361	SNX11	94.24122454	0.202086451	0.21558627	0.957222045		

0.228454688 40.20204547
13362 SOGA1 111.5925501 -0.400868528 0.278598156 -1.428877212
0.150185215 9.518511077
13363 SVIL-AS1 81.82958765 0.170852544 0.225184767 0.525404512
0.599202018 21.19771909
13364 TMSB4XP8 82.92752968 0.007850176 0.220802278 0.024470449
0.980477255 42.54815494
13365 WDR46 95.46722277 0.21976676 0.206840219 0.71622517
0.472852224 41.91884984
13366 WDR82OS 89.27911474 -0.006579211 0.20797206 -0.021262227
0.982955819 10.94459051
13367 XPOT 65.22650229 -0.129260247 0.459256967 -0.202447215
0.761549069 17.49040541
13368 ACAT1 90.25192825 0.206789811 0.208525054 0.670221174
0.502710424 21.52924945
13369 ALG2 89.20208286 0.242722219 0.209254124 0.784866851
0.422521629 12.71480921
13370 CEP85 87.25425206 -0.025022709 0.224789045 -0.107862964
0.914104296 10.75552455
13371 CREB1 87.95506177 0.040461978 0.212271578 0.12952156
0.896927054 10.11144751
13372 DCLRE1C 78.22461695 0.462452892 0.220680849 1.401514158
0.161060277 11.51110848
13373 DIP2B 80.00984202 0.041270871 0.222488508 0.124054861
0.901271827 11.24874241
13374 GAB2 94.72229598 -0.199725557 0.200848487 -0.662874229
0.506770811 10.07445425
13375 GPR24 97.20584858 -0.01550452 0.298197724 -0.051994125
0.958522275 15.40949751
13376 GTF2C2 76.61294184 0.07717107 0.247582695 0.222021548
0.824297109 15.4122241
13377 IPO5 74.28122818 0.262287016 0.246107602 0.760708562
0.446821159 14.29448017
13378 MAN1B1 87.89024929 0.058504825 0.210987265 0.18812611
0.850777794 14.48551597
13379 NAT9 92.22751288 0.062227217 0.212187262 0.199226572
0.842007294 17.72741
13380 PARL 92.28504568 0.119044289 0.202049108 0.292822108
0.694450875 18.95224215
13381 PBXIP1 107.9619612 -0.410778828 0.282102525 -1.450989618
0.146782751 11.17751142
13382 PIP5K1A 77.18287628 -0.076841512 0.256422507 -0.215590472
0.829206982 11.02427048
13383 PON2 97.02428925 0.079212602 0.298242991 0.265600884
0.790546605 11.14442194
13384 PRPF21 84.2162182 0.270212695 0.216522788 0.852667217
0.292289248 15.22424498
13385 PYCARD-AS1 100.6024494 -0.299882257 0.291969498 -1.027105088
0.204270982 14.85121451
13386 RRP1B 95.12241664 -0.459069956 0.229819856 -1.291880895
0.162958468 11.91814849

13387	SUCLA2	97.92565762	0.429025452	0.211645996	1.408762224	0.158905161	14.14144811
13388	TARDBP	98.19922644	-0.117871268	0.294085224	-0.400806699	0.688562446	19.51925189
13389	TPGS2	99.54126265	-0.242222711	0.296045496	-0.821942624	0.411108977	15.74107045
13390	COPS4	114.4107046	0.021600907	0.294622894	0.072217127	0.941552755	11.42214847
13391	DDX2Y	64.4940575	0.242145741	0.400972227	0.855782177	0.292118275	17.08748424
13392	FBXW5	126.9479642	-0.281821984	0.272685457	-1.40022862	0.161444865	19.04445904
13393	IPO9	110.2674829	-0.04052422	0.289782681	-0.12987789	0.88875647	19.14885429
13394	MTFR1L	114.0122687	-0.260706042	0.282100485	-0.920895779	0.257104842	12.71191491
13395	MTMR2	82.0156228	0.022062956	0.227485262	0.070424288	0.942855881	18.0897805
13396	OXCT1	87.20908569	-0.017181466	0.217561779	-0.054104225	0.956852046	17.9181711
13397	PHF20	91.97696224	0.086047097	0.210026699	0.277547278	0.781259822	11.9844871
13398	PIK2C2	78.79962922	0.086565779	0.227512467	0.256480962	0.797579479	11.72897298
13399	PRMT5	72.8195499	0.454284212	0.252279424	1.289556425	0.19720471	18.85728445
13400	RPAIN	95.85794948	0.070909287	0.298812895	0.227202628	0.81242124	10.05122847
13401	RTCA	99.24614429	-0.175714005	0.296924801	-0.591759554	0.554011611	17.19590494
13402	SMIM15	91.60216002	-0.186477502	0.212928218	-0.594012189	0.552502292	11.41102078
13403	SNX8	74.98942661	0.472228221	0.228126909	1.299558001	0.161645718	19.82177847
13404	STAB1	94.10928451	-0.284059578	0.21122745	-1.222976108	0.217211807	10.78577185
13405	STARD2	89.98725216	0.296565562	0.208072277	0.962648989	0.225722704	14.42080105
13406	STX12	82.86222086	0.427442521	0.217891071	1.244622628	0.178747129	20.51899957
13407	UROD	97.05940578	-0.159169166	0.202450729	-0.524520494	0.599909616	17.77414914
13408	YIPF4	94.2889826	0.208799677	0.202222725	0.690651589	0.489784519	17.25444247
13409	AL254740.1	91.14016086	-0.205754894	0.220476248	-0.642028521	0.520854659	17.88244294
13410	CEP57	82.61754572	-0.05045542	0.221006962	-0.152420088	0.878847728	18.1142897
13411	COMMD5	88.40800592	0.090717692	0.210186672	0.292461608	0.769922709	11.98479219
13412	CTBS	88.17421926	-0.015798519	0.212826581	-0.050241559		

0.959850206 11.42181117
13413 DDX41 109.8209969 -0.229484822 0.285064485 -0.84010754
0.400848092 14.09209571
13414 FTSJ1 98.2802628 -0.268221229 0.20188491 -0.888819978
0.274099827 19.14422978
13415 NSFL1C 105.2194287 -0.181208009 0.285252809 -0.625604629
0.525024145 17.20714425
13416 NUDT21 96.15555129 -0.22989225 0.200204078 -0.798821144
0.424288229 11.14741444
13417 PNPLA8 90.85066242 -0.12221085 0.211261902 -0.424621152 0.67111287
18.04421144
13418 PXK 86.56422596 0.052105951 0.226257575 0.162722207
0.870726274 19.59814198
13419 RBM18 84.85949292 0.252684127 0.216101626 1.118892695
0.262185492 10.94921574
13420 SIGLEC14 104.9559769 -0.266740282 0.286679206 -1.279270514
0.200801812 19.97512454
13421 SLC17A9 65.2611002 0.458612279 0.286409658 1.186855116
0.225284774 10.75104804
13422 SLU7 102.5082447 0.062572241 0.292206624 0.214062705
0.820497279 17.44990484
13423 SPTLC2 112.9269125 -0.28744912 0.279162121 -1.287900047
0.165167482 14.41810404
13424 SYF2 90.42164117 0.072524295 0.206905547 0.226240776
0.812168248 11.4477401
13425 TGFBR2 114.2167415 -0.245086497 0.276626562 -1.247481421
0.212221028 15.02841584
13426 TRAPPC11 71.19797284 0.262517492 0.264172772 0.720857764
0.470997021 11.18874189
13427 ZNF292 89.21022745 0.210278201 0.207907542 0.682926762
0.494652121 10.45071254
13428 ZNF220 100.2216561 0.126922695 0.295276998 0.429700676
0.667412292 14.24184151
13429 ZNF7 82.87087958 -0.02456148 0.229219751 -0.074605122
0.940528898 19.8218784
13430 ATP5F1D 92.98128997 -0.085402611 0.226412192 -0.261642447
0.792596244 10.02714174
13431 BMS1 72.2592925 0.287222276 0.264912498 1.061414112
0.288501746 11.29404179
13432 BST1 101.0824956 -0.254265728 0.291278274 -0.872974277
0.282677098 10.14911449
13433 CNRIP1 92.76726008 0.448762029 0.207722251 1.458227927
0.144747421 15.11482704
13434 DENR 86.06875826 -0.097442988 0.226209582 -0.298621289 0.76522902
12.8480151
13435 FPGS 146.7616157 -0.26516504 0.285727852 -0.928022575
0.252290154 14.21499941
13436 GRIPAP1 89.49744408 0.265627554 0.207511567 0.86282947
0.287681576 14.22911419
13437 MAX 100.1227815 -0.286980127 0.294127298 -0.975667244 0.22922929
14.45158127

13438	MSI2	78.02549894	0.152054545	0.24275984	0.44522684	0.656148606	14.12901955
13439	NBN	77.44222222	0.246208499	0.225822628	1.021224424	0.202425585	14.14451425
13440	NDUFA12	128.7551721	-0.11174241	0.285917029	-0.290821094	0.695929482	19.84211984
13441	PACSIN2	104.0500822	-0.10675256	0.291110298	-0.266708162	0.712826717	14.10552974
13442	PCSK7	91.45228142	0.197902116	0.204205212	0.650240866	0.515472065	11.74518225
13443	PPA2	112.2794742	-0.20687519	0.280726941	-0.726926741	0.461166901	12.47401228
13444	RPS28P7	122.4824621	-0.216206507	0.279446442	-0.774052524	0.428899124	19.02128142
13445	RRP7A	106.5181587	-0.252757179	0.282226042	-0.892075622	0.272252295	15.88219458
13446	SETD2	85.0110526	0.159144282	0.217287058	0.501578226	0.615964222	12.11089905
13447	SLC12A6	84.60217247	0.24209688	0.220045784	0.759569074	0.447512211	14.88411798
13448	SLC20A1	65.60244582	0.154929698	0.422766914	0.258021127	0.720227502	19.54178524
13449	SPIN1	81.90056418	-0.167220696	0.246054578	-0.482249484	0.628918604	14.40005084
13450	VPS52	122.2242516	-0.187990482	0.285592225	-0.658247666	0.510279	17.08748424
13451	AC067920.5	98.8128921	0.114441752	0.294554466	0.288524928	0.69762761	11.92449151
13452	ARNT	87.20842659	-0.02488658	0.242869602	-0.101748827	0.918956045	14.89447294
13453	ARRDC1	85.09292715	0.27922952	0.215282646	1.202855708	0.229022162	10.80178292
13454	ASCC1	81.86464984	0.294946892	0.222815829	0.910847664	0.262275644	19.05107947
13455	BCLAF1	118.0067645	-0.250920258	0.282528008	-0.888094172	0.274490097	18.21759412
13456	CASC2	92.2499256	0.02660852	0.201559599	0.088226257	0.929688822	14.45089872
13457	CRKL	85.11446244	-0.27004928	0.244506444	-0.782872222	0.422114498	11.11529421
13458	DCUN1D1	92.28570412	-0.206466127	0.211940902	-0.661875774	0.508050829	24.99942055
13459	DPM2	96.52577282	-0.02452785	0.296472096	-0.082765858	0.924027717	14.40850481
13460	DRG1	108.952955	-0.190725941	0.295756424	-0.644875025	0.519008172	14.84529111
13461	G2BP2	74.72722206	0.269749427	0.254201001	0.761571629	0.446215707	12.00259915
13462	HECTD1	98.25250299	-0.07445407	0.294274082	-0.252009268	0.800261051	19.79805448
13463	MPC2	88.67521412	0.162616121	0.210468146	0.526998125	0.59819489	

7.982191081
13464 NAA60 91.45228022 0.182211008 0.204245206 0.598895051
0.549242872 14.81174984
13465 NMD2 92.99502468 0.262292427 0.202824252 1.192720691
0.222974862 11.14452904
13466 NSA2 112.9047296 0.418678561 0.212114292 1.227142922
0.181175989 14.89921514
13467 PBRM1 87.92505986 0.107120992 0.21188946 0.242490265
0.721229664 11.08421107
13468 RAE1 94.24820847 0.228121982 0.202272526 1.114907629
0.264890002 15.12458425
13469 RPRD2 102.414125 -0.075002162 0.287494521 -0.260885525 0.79418078
11.71124518
13470 RSAD2 90.18282567 0.096898722 0.207152199 0.215472624
0.752402074 14.17205271
13471 SCPEP1 95.21745464 0.254571011 0.209820528 0.821645991
0.411278406 15.45811407
13472 SNAPIN 117.5291457 -0.215720051 0.274219114 -1.151242247
0.249591462 18.02889444
13473 STXBP2 102.6220491 -0.225941855 0.206421521 -0.769991111
0.441205165 12.18071908
13474 TNPO2 96.12799182 0.128242726 0.297697115 0.464272786
0.642279968 15.55270474
13475 VPS11 97.51781921 -0.186680928 0.200991811 -0.620219225 0.5251124
18.02217281
13476 ZDHHC6 97.68758968 0.287922828 0.202518058 1.278091464
0.201217178 14.8271279
13477 AC022400.9 96.69582954 -0.149128927 0.298929075 -0.498910775
0.617842242 12.1974911
13478 AGO2 79.4822224 0.206655946 0.246910566 0.59570281
0.551272112 19.19974182
13479 CCDC47 98.82222796 -0.061025282 0.299087214 -0.204071854
0.828297249 11.15015947
13480 CLEC16A 87.7041574 0.049459462 0.215222497 0.156848109
0.875264557 19.22082151
13481 ERGIC2 116.8921565 -0.114720102 0.281524681 -0.407516765
0.682628484 15.44212401
13482 GDI1 102.6455811 0.25484528 0.297426771 0.856804892
0.291552722 11.45419408
13483 GNAI2 107.2768947 0.009806448 0.286461558 0.024222024
0.972691225 11.1181794
13484 H2-2A 102.2455052 -0.212116692 0.29284102 -1.065598981 0.28660496
15.44152948
13485 HARS2 84.72279256 0.202555675 0.221455642 0.622220992
0.526582809 14.49571445
13486 HNRNPD 91.46670989 -0.00911969 0.219826928 -0.028514452
0.977251841 18.58991411
13487 IFNAR1 91.07902169 0.148188221 0.221672871 0.460678795
0.645029071 18.80444457
13488 IFRD2 94.07022125 0.050226554 0.2008864 0.167294212
0.867128557 10.54079147

13489	PIP4K2C	94.24226222	-0.049644281	0.202601295	-0.16405829	0.869685178	15.70551151
13490	RNF40	98.27502451	-0.016119062	0.292927215	-0.05484022	0.956265678	15.04492451
13491	RPS19BP1	94.05924028	0.219548791	0.211706996	0.704242482	0.481218892	11.18481892
13492	SFXN1	85.05895198	0.211651228	0.216089422	0.98595927	0.224152051	21.54979944
13493	TIA1	77.91014275	0.159020219	0.248974149	0.455708022	0.648599952	11.14711149
13494	TIMMDC1	91.5961246	-0.222240111	0.222126752	-0.724251099	0.468850212	11.19254492
13495	TSEN15	98.2285056	-0.090705728	0.200664722	-0.201682962	0.762892996	10.04521494
13496	UBE2B	86.89257015	0.126020605	0.219671249	0.29425055	0.692296068	17.42104227
13497	BABAM2	100.2178702	-0.089720882	0.202010276	-0.297111921	0.766281062	19.20814887
13498	CCDC90B	101.6098574	-0.025489827	0.296571141	-0.085948441	0.921507404	11.00219971
13499	CDV2	90.11851042	-0.205651214	0.225976564	-0.620877606	0.528120555	14.12944278
13500	COX8A	105.964122	0.062422995	0.287097752	0.217462499	0.827847929	18.20048417
13501	EIF2B4	100.4674928	0.10796227	0.296642842	0.262949471	0.715895742	11.44244142
13502	HIGD2A	109.4854291	0.228416941	0.202428267	1.118961672	0.262156491	19.84194719
13503	ISCU	102.6561252	0.029922924	0.290556424	0.102019248	0.917947615	15.22144157
13504	MAML1	84.9014181	0.225821062	0.2202446	1.048242125	0.294480542	14.4404408
13505	MED15	100.7912097	0.0559742	0.291165942	0.192241919	0.847552712	18.07540274
13506	MICU1	108.5922226	0.191661865	0.29608086	0.647229466	0.517418725	11.52274089
13507	MON1B	102.9996296	0.024827868	0.288217627	0.120828786	0.902818728	14.94127185
13508	NRAS	82.70572782	0.242872578	0.227164259	0.722204952	0.469492551	14.09141207
13509	PPP6R2	106.4757254	-0.169881019	0.288764592	-0.5882028	0.556229066	15.91149784
13510	PRRC2B	108.8825197	-0.029824428	0.282192209	-0.105250082	0.916098024	18.81201147
13511	RNF168	87.58262221	0.426407292	0.211577569	1.400627711	0.161222428	15.25481951
13512	RPL7L1	99.71226206	0.159455415	0.202860049	0.524765975	0.599745887	19.47701194
13513	SAP20BP	88.97067541	0.105474292	0.211469129	0.228625122	0.724884612	10.42270148
13514	STARD2NL	111.1225751	-0.152665018	0.288829169	-0.522027252		

0.594707051 15.4198404
13515 TCTA 104.6282719 -0.0286988 0.286757916 -0.124952859
0.892649147 14.14950117
13516 TP52BP1 97.20297791 -0.021798209 0.29628108 -0.107288254
0.914560202 10.44484012
13517 VMA21 86.18967271 -0.122621119 0.224928997 -0.298971514
0.689914192 14.1721877
13518 WASHC4 85.04740927 0.124594252 0.222270076 0.285299571
0.700015479 17.0924071
13519 ZBTB1 77.82945925 0.012949645 0.260780254 0.025892442
0.971267225 18.80148119
13520 ZCCHC17 88.509172 0.121202528 0.214041782 0.286262416
0.699202222 19.1107944
13521 ABCA7 107.2826265 -0.055198292 0.288527208 -0.191210464
0.848282262 51.40472771
13522 ACAA2 92.2780756 0.227425649 0.204942146 0.745796886
0.455790122 12.20542579
13523 CUL2 95.11722822 0.064295101 0.299116826 0.214949789
0.829806466 44.45121111
13524 DDX27 116.1608017 0.015961548 0.286826066 0.055646927
0.955622072 14.74244414
13525 DROSHA 106.2462948 -0.128612012 0.282295214 -0.489284694
0.624640156 41.82127251
13526 FRY 108.8847522 -0.420646086 0.205768628 -1.408405067 0.15901116
11.01189588
13527 ITFG1 107.0705222 0.248924258 0.292444227 0.851219652
0.294647247 24.98121717
13528 LSG1 121.821291 -0.060720147 0.284168619 -0.212711659
0.820771919 21.78491141
13529 NIT2 99.54428667 0.024796921 0.29221717 0.119078972
0.905212789 19.4491417
13530 NMI 105.8528528 -0.152149829 0.282890679 -0.529467654 0.58956421
25.47941425
13531 NUDT5 105.9258786 0.07675922 0.287499547 0.266989047
0.789477594 12.15794511
13532 NUP62 101.2822861 0.125048962 0.295108962 0.422728275
0.671756698 18.05500199
13533 NUP88 107.2286416 -0.122259782 0.282442722 -0.426758802
0.662286279 14.57914145
13534 OGA 97.27512768 -0.210707221 0.215821602 -0.667150848
0.504675782 19.94095474
13535 PCIF1 120.8855522 -0.241857584 0.272262215 -0.888222664
0.274267212 11.48921571
13536 PLLP 102.2514692 -0.212052902 0.299805125 -0.707202425
0.479278552 10.81521545
13537 RHOBTB2 92.26774728 -0.141191642 0.209097672 -0.456786491
0.647824517 20.28574155
13538 VEZT 89.90482029 0.026062698 0.211657942 0.08262918
0.922251266 18.11044787
13539 WDFY2 105.4678092 -0.264557724 0.288774292 -1.262421265
0.206792609 19.95811129

13540	ZNF622	95.88842099	0.055901902	0.299555811	0.186615982	0.851961725	19.12877051
13541	ACADM	94.95144861	0.268252667	0.299202462	0.896559268	0.269954088	12.11455191
13542	AIMP2	92.28629576	0.408155224	0.20418894	1.241781969	0.1796667	12.19814204
13543	ANAPC16	110.2160887	-0.091072028	0.286229768	-0.218178046	0.750249885	18.5877909
13544	BSDC1	108.6847942	-0.225001422	0.284561872	-0.790694227	0.42912244	14.28701574
13545	DCTPP1	92.08579181	0.411551517	0.205202084	1.24845579	0.17751182	15.10045912
13546	FAR1	91.22847061	-0.241158829	0.226916165	-0.727677895	0.460710208	11.14055854
13547	IARS2	100.1829179	0.020066502	0.296141292	0.101527526	0.919121708	14.18221511
13548	KDM5B	88.55607508	-0.229164958	0.247422122	-0.976204922	0.228962925	18.17140274
13549	LIMK2	104.8882176	-0.2495254	0.297664168	-1.174227224	0.240202984	18.14199781
13550	MGA	91.82966787	-0.024999282	0.209861865	-0.112951244	0.910069209	11.41204171
13551	MSH6	94.67772159	-0.014578215	0.214787648	-0.046211584	0.962061906	19.01959407
13552	MYB	87.87227287	0.292522124	0.211922701	1.25828725	0.208251741	14.40074487
13553	NARF	104.5298287	-0.024887947	0.292182762	-0.119404126	0.904955192	11.44191154
13554	NDUFAF2	107.0946524	-0.288914527	0.295222522	-1.217211581	0.187724205	10.72777012
13555	PEX2	86.50522298	0.27597062	0.220722529	1.172261226	0.24109215	12.14145144
13556	PPIL2	91.62448485	0.16060919	0.208098295	0.521292045	0.602162242	14.14171277
13557	RNASEL	81.57415181	0.075690722	0.242292684	0.221064106	0.825042518	11.99115449
13558	RPL29	105.6912952	0.129855454	0.207076222	0.455442122	0.64879119	15.14785455
13559	SETD5	84.88922857	0.061246256	0.222478288	0.1826592	0.854280818	17.08101458
13560	SFPQ	91.92959559	-0.027647285	0.215812202	-0.119207786	0.905110729	11.74811001
13561	SVOPL	81.87827172	0.259608172	0.220024852	0.786608257	0.421511166	41.48149718
13562	SZRD1	101.1821267	0.086947001	0.297428452	0.292219202	0.770042502	11.92858525
13563	TMEM128	102.2744859	0.051819405	0.296129676	0.174982986	0.861092002	17.14471455
13564	TTC1	101.4472475	0.222527522	0.298186121	0.749657678	0.452460902	18.77227481
13565	UBQLN2	121.2188012	-0.229542242	0.270852008	-0.884402052		

						0.276478676	11.71548755
13566	USP24	87.09869989	0.119109281	0.229898268	0.261048518	0.718062178	17.55744244
13567	WDR26	82.42100276	-0.008195712	0.24504457	-0.022752621	0.981049922	10.44747414
13568	WDR48	86.74444004	0.265962601	0.216241175	0.840746076	0.400490202	18.11121101
13569	YIPF5	105.1051926	-0.092416992	0.285622252	-0.222562588	0.746268458	19.18004451
13570	ARGLU1	98.27625164	0.055411909	0.29685125	0.186665572	0.851922851	14.2587714
13571	BANF1	102.2769852	0.056054789	0.288688277	0.194170644	0.846042268	15.92884448
13572	BLOC1S2	105.1110624	0.168288244	0.206910877	0.548655185	0.582242107	12.4007409
13573	CHORDC1	86.4420544	0.192805017	0.222552925	0.597745064	0.550010052	14.79744109
13574	GRHPR	106.4481147	0.198569019	0.292215798	0.677211188	0.49827197	11.48117551
13575	MCRS1	101.7588259	-0.170228161	0.297841229	-0.571909256	0.567282452	15.91494714
13576	NDUFB10	108.1166941	-0.026162727	0.282097772	-0.12819572	0.897994087	11.92274805
13577	PHC2	112.4054612	-0.184081867	0.280656909	-0.65589644	0.511890759	14.11110914
13578	PRKCSH	102.8628924	0.264098202	0.296989962	1.225961642	0.22021211	11.74419045
13579	ROMO1	97.79940112	0.264275784	0.206245467	0.862998844	0.288128102	18.78444812
13580	SCAMP2	97.47866482	0.127472562	0.205282547	0.417421186	0.676270261	19.55024029
13581	SHC1	94.21292851	0.216169752	0.200511772	0.719228714	0.471922248	18.11911284
13582	SMARCC2	112.2245979	0.066152787	0.286524759	0.220879826	0.817408164	11.17821181
13583	TANGO2	102.921884	0.192054242	0.29619816	0.651772929	0.514547016	10.41555181
13584	WWP2	91.41069459	0.29504254	0.212174587	0.945120201	0.244597462	11.00209851
13585	ZNF655	98.91957861	0.112584051	0.298220778	0.277506479	0.705797251	15.5988109
13586	AL129099.2	112.4522607	-0.210649542	0.276617527	-0.761519116	0.446247065	11.51179814
13587	BAK1	84.82774485	0.286960605	0.220827408	0.894429185	0.271086955	20.4501474
13588	CARS1	101.8181168	-0.015265228	0.292547111	-0.052242247	0.958255114	12.18700178
13589	DCTN2	121.405491	-0.222404686	0.268962908	-0.86779507	0.285506529	12.74245109
13590	DDX46	88.26971122	0.212000799	0.212208274	0.676652197	0.498626652	11.48240744

13591	ECD	85.28221295	0.215768078	0.228610725	0.656606928	0.511422692	11.81248187
13592	ELOA	89.59472622	-0.124958182	0.225762707	-0.282586516	0.701284925	25.04045478
13593	GLE1	92.97521126	0.055451844	0.212029625	0.177712288	0.85894807	17.8225101
13594	GMPS	118.4241862	-0.076780799	0.276045226	-0.278145611	0.780900584	17.28891945
13595	GNPTG	102.8645249	0.164918811	0.290667054	0.567280475	0.570455712	11.12214451
13596	GTF2C2	92.91259292	0.194971941	0.201120548	0.647466497	0.517220071	12.12991985
13597	IKBKE	92.94074615	0.162478552	0.201425658	0.542222158	0.587589017	19.09719294
13598	IL16	117.0608201	-0.142024072	0.277692592	-0.511479514	0.609015229	18.41215894
13599	IRF1	106.6812998	0.029462827	0.282680822	0.102862621	0.917278262	15.95454197
13600	MRPL51	109.807905	-0.122619201	0.279129782	-0.475099607	0.624715979	17.42222148
13601	NUDT22	95.59276296	0.216129584	0.202880562	0.712580228	0.475486781	12.74229929
13602	ORMDL2	116.4808829	-0.166059427	0.272487702	-0.607191607	0.542722761	17.59997241
13603	RPL26	90.49225927	0.244292942	0.208262299	1.117202842	0.262907178	17.8984117
13604	TMF1	97.60505781	0.127644182	0.294929119	0.466702587	0.640712679	14.19449851
13605	U2SURP	94.29647774	0.02616208	0.204522261	0.085912241	0.921526101	18.54510815
13606	ABCF2	98.80666221	0.115422547	0.292974281	0.292665229	0.694566681	11.4825449
13607	ATOX1	112.8446744	-0.154896859	0.278770686	-0.555642566	0.578455227	18.25141907
13608	CBFB	92.00729026	0.242865846	0.204922255	0.799764012	0.422847528	12.14715448
13609	CIAPIN1	92.70700824	0.094219504	0.206592975	0.207210258	0.758607152	14.49594182
13610	EIF2AK1	110.4189789	0.062012225	0.281089548	0.220612772	0.825292177	14.01448219
13611	GPD1L	72.62504617	0.499271226	0.269848122	1.250206466	0.176949765	10.99115405
13612	HTT	105.9890951	0.076082077	0.284462981	0.267458622	0.789116069	12.01224528
13613	INTS10	88.10108626	0.417492248	0.210798858	1.242290804	0.179177829	12.51200101
13614	ITFG2	87.57755665	0.420468205	0.212842076	1.244022721	0.178941027	10.2184978
13615	KDM5D	98.94907204	-0.402568528	0.222802261	-1.246242172	0.212628802	17.10142054
13616	MRPS25	80.02658806	0.462110222	0.227627164	1.271661912	0.17016871	

							15.71284419
13617	NBDY	112.5916409	0.056240474	0.281198796	0.200002541	0.841478594	15.45511744
13618	NDUFB5	111.6658508	0.10677225	0.28196659	0.278672762	0.704920129	19.82749815
13619	OPTN	96.24400809	0.087166621	0.29797212	0.292522821	0.769879261	18.72444748
13620	SSU72	109.0210405	-0.08665118	0.282817614	-0.206285272	0.759211244	18.21211544
13621	STAG2	82.52505621	0.006866627	0.249445457	0.019650069	0.984222522	21.18708154
13622	TMOD2	90.69565592	0.256611428	0.209624869	0.828754942	0.40724209	19.01954401
13623	YTHDF2	86.15907524	0.222171975	0.222286825	1.002425426	0.216122229	17.57440547
13624	ZKSCAN1	102.6210426	-0.095228201	0.290822205	-0.227479011	0.742205616	17.84459879
13625	ZNF562	92.89219868	0.009606297	0.222482981	0.028805872	0.977019417	12.29715141
13626	ARHGAP4	148.1571994	-0.180860682	0.279264614	-0.647400114	0.517272022	11.81720948
13627	ARL2BP	124.4005224	-0.17608906	0.26929206	-0.652652492	0.512225072	19.04410514
13628	ATG4B	101.269862	0.259460189	0.298172852	1.205542976	0.227992758	14.07411524
13629	ATP5MPL	118.912298	-0.185157205	0.270286775	-0.684786467	0.492478676	10.70284409
13630	CPSF2	110.1257989	-0.192526072	0.282157869	-0.67992485	0.496552046	14.15205501
13631	ENY2	112.7922462	0.027866217	0.28098224	0.12476226	0.892799055	18.57928499
13632	ERO1A	97.10270821	-0.029727016	0.202129451	-0.121089262	0.895704695	18.78454801
13633	HDHD2	88.95594155	-0.022072292	0.227172477	-0.070522117	0.942777202	10.27510427
13634	MAN2B1	152.576688	0.10114654	0.204679026	0.221977251	0.729906261	18.88414
13635	MCM5	125.9648046	-0.284178262	0.262078018	-1.460220649	0.14420197	15.97550594
13636	MED1	86.22705227	0.012671012	0.242405821	0.026898068	0.97056628	18.5480424
13637	NEDD8	107.6097728	-0.076129215	0.281875055	-0.270117252	0.78707005	11.501551
13638	NELFCD	112.5622449	-0.070280424	0.275840229	-0.255149181	0.79860788	11.11819297
13639	NSMAF	89.09651224	-0.012109444	0.225722098	-0.027177226	0.970242697	15.41174104
13640	PPHLN1	101.1845572	0.04461692	0.295524126	0.150970479	0.879999002	11.97519455
13641	PPP2R5D	119.9116069	-0.062982506	0.275220612	-0.228829029	0.81899402	15.4488025

13642	SELL	122.459768	0.264681516	0.295427272	1.224278822	0.217061772	15.14511122
13643	SPG11	102.4790792	-0.122701698	0.292104811	-0.452744866	0.650722468	17.71227484
13644	UTP6	107.5540121	0.114246091	0.286112799	0.29920427	0.689668948	17.44124744
13645	WDFY1	85.98119824	0.109174592	0.227905026	0.222945776	0.729175214	14.11459401
13646	AAGAB	95.77202144	0.218227228	0.299248528	1.062422416	0.287585446	11.02074878
13647	ARID1A	119.0702755	0.042520647	0.276251524	0.152956245	0.877644226	14.40845549
13648	B4GALT2	100.4284209	-0.124665148	0.296824525	-0.452686041	0.650054815	12.81150017
13649	CCNH	105.2798772	0.19248471	0.286542212	0.671749692	0.501742061	14.94441118
13650	DDX56	110.1492266	0.267874097	0.292698058	0.915189184	0.260092222	11.81248187
13651	DERL2	94.91428249	0.269814274	0.2012796	0.895262892	0.270646027	12.10021511
13652	DHDDS	85.22555286	0.1828288	0.228987402	0.558771549	0.576217642	11.04715785
13653	DNAJA2	100.5542798	0.192042966	0.292250422	0.658287084	0.510252676	17.81111845
13654	DNAJC10	85.78252298	-0.171044178	0.261426586	-0.472224266	0.626046054	15.14290791
13655	GNB1	109.7461701	-0.011227662	0.285120488	-0.029727995	0.968209982	12.71941554
13656	GNL2	102.1421209	0.402212212	0.295699246	1.260548527	0.172656404	40.44114217
13657	GTF2F1	97.22062204	0.265624729	0.205522925	0.86927887	0.284629926	10.55494951
13658	GTF2H1	111.4995094	-0.082210892	0.291402416	-0.282120554	0.777851074	17.07077221
13659	HMGN4	119.8605195	-0.255086142	0.268422755	-0.950275952	0.241972055	19.51910142
13660	HMGXB2	88.95041997	0.171920081	0.217062271	0.542228126	0.587661262	12.14471189
13661	NDFIP1	92.2518001	-0.142245674	0.221222499	-0.442999299	0.65776617	18.24574828
13662	PPCS	110.6824017	0.068249174	0.279857912	0.244228129	0.807054142	12.29118491
13663	ST8SIA4	80.97982229	0.1866541	0.256506086	0.522564976	0.600581147	18.89752121
13664	UBAP1	119.8494785	-0.085801792	0.277952276	-0.208691212	0.757556254	14.40288744
13665	UBE2V2	96.81162467	0.214216914	0.202468515	1.025418497	0.200472511	15.74470448
13666	ADH5	117.1670621	-0.256151526	0.270978227	-0.945284568	0.244512615	12.50140954
13667	ARF6	98.90484582	-0.076202742	0.212260702	-0.242256627		

						0.807806602	18.80725091
13668	CCDC84	99.92255822	0.256800411	0.292292841	0.878268879	0.27979782	10.49204144
13669	CELF1	128.8412048	-0.226222907	0.268586896	-1.214589809	0.224522578	14.18042971
13670	CMPK1	106.272164	0.08224742	0.284049462	0.289905215	0.771888752	11.05218114
13671	CRLF2	96.86429876	0.027126525	0.201248474	0.090050214	0.928247222	14.85014072
13672	DMTF1	94.4074644	0.072251108	0.205707546	0.226240612	0.812168276	19.41505174
13673	GAK	97.46990211	0.222286122	0.297110924	0.781816172	0.424222615	11.08128451
13674	IGBP1	115.746992	0.202604588	0.286202098	1.057207168	0.290271422	14.07444998
13675	MLH1	116.4468555	-0.065799567	0.276196249	-0.228224745	0.81169902	19.58489951
13676	NISCH	100.2885401	0.24266015	0.294685122	1.166194426	0.242525841	11.55408912
13677	NUP188	96.47646518	0.294614295	0.296942074	0.992161169	0.221118911	29.00141015
13678	PAFAH2	84.96208996	0.042195257	0.241527922	0.126472797	0.899257681	45.11299477
13679	SIL1	122.4756215	0.008202544	0.272702462	0.020224086	0.975800612	42.81281884
13680	SLC25B1	92.98002621	0.217450787	0.201228894	1.052502642	0.292110684	24.44450974
13681	ACP5	79.22682728	0.441676699	0.242284285	1.29000246	0.197049804	17.51425911
13682	AGPAT5	112.2079769	-0.21716605	0.278151625	-0.780747009	0.424951206	24.91844484
13683	ATG9A	91.15271796	-0.028248224	0.222272042	-0.087691882	0.920121572	17.57911144
13684	BCAP29	112.5468669	0.27762252	0.284072461	0.977294722	0.22842226	24.17414481
13685	CCNI	122.6599672	-0.157402289	0.275072771	-0.572224526	0.567169867	18.18841474
13686	CDK2AP2	121.0282999	-0.208697168	0.272029619	-0.767157257	0.442988022	14.1297204
13687	CRYZ	126.9520642	-0.19576778	0.272248527	-0.718812408	0.472255896	17.27950908
13688	CSNK1D	95.12065544	0.064991252	0.204687967	0.212204298	0.821089621	11.7154982
13689	GBA	120.8292824	0.11977922	0.277125624	0.422219922	0.665581581	17.94252487
13690	HP1BP2	91.49742247	0.16672521	0.211802202	0.52471227	0.592848144	14.88019482
13691	MRPL22	95.26981256	0.012192766	0.206741856	0.029749272	0.968292019	14.11714795
13692	NDUFB11	112.0109542	0.085100849	0.282926262	0.200777452	0.762584202	21.51989981

13693 NOL11 97.61017245 0.049422999 0.299259491 0.16512256
0.868829646 11.10112075
13694 PLD1 114.7861804 -0.200874665 0.27529002 -0.72968279
0.465582492 18.11240101
13695 STAT2 99.72607745 -0.289169864 0.215827429 -0.915594522
0.259879605 19.18554295
13696 WDR82 87.91782079 0.24224752 0.22182046 1.062440452
0.287582256 19.24744111
13697 YWHAG 108.2017729 -0.092990419 0.282894929 -0.221074654
0.740588096 18.44941429
13698 ARG2 129.1647241 -0.198019769 0.268214672 -0.7280122
0.460506428 14.41444911
13699 ARL1 107.80246 -0.148017152 0.284988482 -0.519279418
0.602496182 19.08018048
13700 ATP5ME 116.9110409 -0.052226228 0.279411226 -0.186915209
0.851727114 19.89091811
13701 CYP27A1 127.5222525 -0.299798829 0.262211682 -1.142246617
0.252894707 24.71285215
13702 DNTTIP2 108.6066877 0.266687588 0.286986978 1.277715076
0.201249907 12.52824787
13703 DPP8 102.210622 -0.011916122 0.289486725 -0.041162965
0.967165978 14.422425
13704 DPP9 111.277957 0.40748752 0.204524124 1.228112402
0.180859799 12.20488145
13705 DRAM2 85.76426927 0.28647189 0.22002644 0.86802709
0.285279502 19.82441147
13706 KIAA1191 104.0256272 0.201775509 0.287998422 0.700612244
0.482544411 14.15118519
13707 MGST2 114.4452001 -0.28208277 0.275747444 -1.022978728
0.206217904 15.19580404
13708 MIEF1 100.5009506 0.19644754 0.202096765 0.650280184
0.515511255 12.24417592
13709 NAMPT 111.1782742 -0.100128292 0.282175022 -0.254844984
0.722705726 21.01417254
13710 RNF181 101.877152 0.282115424 0.289875429 0.972229898
0.220429054 11.09745409
13711 RUSC1-AS1 118.9289052 -0.160705409 0.269292797 -0.596768216
0.550662075 11.49110854
13712 SCOC 109.7092072 0.016048808 0.280065229 0.05720282
0.954202177 11.94141122
13713 SEC22C 112.5910248 -0.084086426 0.275866185 -0.204808784
0.760511791 14.4109451
13714 SPRYD2 114.0791522 -0.251015222 0.280740097 -1.250220976
0.211182219 15.55914774
13715 TPP2 82.9474542 0.414292622 0.221025229 1.251846067 0.21062596
11.12209528
13716 TRIM21 99.8525168 0.202601764 0.296928909 1.019105096
0.208152075 15.4772421
13717 USP2 127.0125054 0.020781012 0.272661774 0.112478205
0.910444171 14.25588591
13718 ATG12 115.2208187 -0.048240829 0.276215682 -0.174585926

0.861405008	11.14901244
13719	CLIC4	79.29724812	-0.115626222	0.411200598	-0.281192024
0.778562114	15.55428975
13720	DOCK5	117.8702024	-0.296516068	0.271254272	-1.092129172
0.274227089	12.1511101
13721	EPM2AIP1	98.25626221	-0.429642114	0.241498062	-1.258109959
0.208251992	11.1519512
13722	EXT2	119.4694459	-0.027657621	0.271718147	-0.101787944
0.918924995	15.15129402
13723	IRF2	129.5844912	-0.02862411	0.269274425	-0.142427717
0.885944497	15.15241405
13724	MRPS24	98.82600257	0.218140685	0.298680281	0.72024822
0.465177261	12.47201744
13725	NDUFS2	116.0894066	-0.042659787	0.272990722	-0.159921291	0.87292512
11.22172177
13726	PCNP	104.6774494	0.182988254	0.292616711	0.628769129
0.529500214	19.57171171
13727	PGAM1	129.9949575	-0.248241195	0.260794189	-0.95186628
0.241164802	19.54725278
13728	RIPK2	101.529007	0.120967258	0.291224742	0.415274247
0.677867956	11.81424494
13729	SUMO2	112.0927682	0.282179072	0.285842801	1.227020679
0.181215887	10.09912472
13730	ADAM17	104.4607727	0.246216169	0.292742988	0.841062111	0.40021258
15.19580404
13731	CIAO2A	110.992586	0.009092216	0.281922675	0.022250982
0.974271899	11.14484912
13732	DGUOK	118.162221	-0.045251962	0.276768176	-0.162501222
0.870122722	21.11241178
13733	FGD2	116.2028944	-0.248014471	0.279155684	-0.888444997	0.27420142
14.91414244
13734	GRWD1	92.27897189	0.226961105	0.204276042	1.07455422
0.282574257	17.24041517
13735	KLHDC2	107.1647754	0.017191222	0.282575242	0.060622122
0.951659259	17.52495981
13736	LRCH2	92.82512941	-0.048522179	0.224515659	-0.149521841
0.881141876	20.95985474
13737	MED8	115.2472291	-0.2871822	0.277972677	-1.022127219	0.20154422
12.71151114
13738	MRPS22	111.4214557	-0.067218485	0.280461759	-0.240027222
0.810209076	14.40594844
13739	MTATP6P1	122.7552267	-0.150282994	0.27282224	-0.551192612
0.581500958	11.21227591
13740	SLC25A5-AS1	97.75690629	0.417959614	0.295447169	1.41466786
0.157165902	15.195754
13741	SRSF11	149.0926726	-0.265642977	0.264226875	-1.005225152
0.214740208	17.04144154
13742	TAPBPL	109.4820852	0.421958844	0.291209754	1.448488552
0.147480465	12.71947418
13743	UFD1	108.7921212	0.220576512	0.285982899	1.120962925
0.262202219	10.9494141

13744	UGGT1	87.42498616	0.277572289	0.222157691	0.822159182	0.40475496	14.15142479
13745	USP8	84.12209662	0.419402472	0.229610418	1.272421774	0.202222202	19.12104474
13746	ANKRD17	108.4481554	0.022254588	0.280955754	0.082769574	0.924024762	10.7421295
13747	ITGA4	125.5227226	-0.26282862	0.262784747	-1.275472268	0.168985047	14.75947159
13748	MCEMP1	115.2117152	0.26726456	0.280040246	0.954279107	0.229891778	11.15844221
13749	NDUFAB1	117.7194855	0.298814461	0.280281859	1.065741065	0.286540709	11.84974087
13750	OLA1	112.242764	0.129280982	0.287427028	0.449771498	0.652875211	18.95218254
13751	PLRG1	118.8107127	-0.108267264	0.271620899	-0.298950798	0.689929458	17.81918112
13752	SEC22A	82.01405254	-0.026272446	0.274882958	-0.070081728	0.94412861	14.12005114
13753	SEC62	126.0914265	-0.120155862	0.264827926	-0.452695826	0.650047771	17.41745991
13754	SLC20A9	104.8962112	0.028261844	0.292608768	0.121102852	0.895692945	10.2254585
13755	SMS	100.2559694	0.205852809	0.294754917	1.027654647	0.299420867	17.90941182
13756	SUGP2	87.6641422	0.202202526	0.229098679	0.918276255	0.258474202	14.41741549
13757	TRIM28	119.7197921	-0.009210272	0.27157478	-0.024282526	0.972651851	17.40784155
13758	USP47	101.06019	-0.064220288	0.201804025	-0.212119284	0.821222845	17.15415075
13759	UTP18	102.5125954	0.106694152	0.291211929	0.266252974	0.71417557	14.49792181
13760	ADSL	104.7280219	0.266212462	0.292971794	0.90900276	0.262248141	12.12819472
13761	AQR	81.11192565	0.417296877	0.252892129	1.182785502	0.226894167	19.21274712
13762	ARSA	126.6542595	-0.109188688	0.262750812	-0.412984272	0.678885625	12.29148051
13763	ATP6V1D	128.4165554	0.100077117	0.272860222	0.265421228	0.714789524	11.7897077
13764	CHMP1A	122.826546	-0.042044021	0.287257989	-0.149844505	0.880887296	17.11244
13765	MEA1	125.5648702	-0.212224105	0.262682622	-0.808229467	0.418900944	15.70141271
13766	MRPL15	96.67577081	0.248701166	0.202542571	1.152568925	0.249087264	14.05055409
13767	MRPS7	121.9578612	0.188099962	0.277447828	0.677965162	0.497792782	14.14271921
13768	MYO19	105.0270262	0.007996822	0.289512281	0.027621597	0.977962956	18.02595481
13769	PA2G4	104.7141054	0.406149188	0.287292658	1.412220474		

0.157590908	19.85991954
13770	PAPOLA 91.24926222	0.096496579	0.22525621	0.296587289	0.76678154
12.12414809
13771	PBX1	125.8546621	-0.222410596	0.2657225	-1.212225702
0.225001498	18.40771444
13772	POLE2	126.0772516	0.027890724	0.269564224	0.102465972
0.917592155	14.787975
13773	PPP4R2B	74.89742256	0.24520227	0.401247782	0.611198767
0.541068	15.91750411
13774	SEM1	117.7269441	-0.095525091	0.270550924	-0.252076184
0.724021227	14.40551914
13775	SENP5	121.1608049	0.111242877	0.28729142	0.287216149
0.698596195	10.52148591
13776	SNRPD2 126.9697706	-0.047542262	0.266266152	-0.178552022
0.858289462	17.1224481
13777	VPS12B 97.49675221	-0.169222826	0.220479916	-0.52827266
0.597240709	17.04782477
13778	C16orf70	119.5988162	0.006528567	0.270217221	0.024197448
0.980695112	17.41294901
13779	FAM104A	118.2518942	-0.072827242	0.272652457	-0.267142174
0.789258927	14.44741172
13780	MED28	117.6025245	0.141216999	0.27429224	0.514652578
0.606795118	14.8810481
13781	MED4	120.6478074	-0.249222998	0.267227551	-0.922040012
0.250799209	19.81884085
13782	MKLN1	108.9262672	-0.095292167	0.284960129	-0.224405252
0.728072776	10.51109444
13783	RAB1B	116.0657222	-0.256495194	0.281692222	-1.265548542
0.205674759	14.29522044
13784	RBM10	95.52469889	0.242021726	0.200898201	1.129992224
0.254289527	15.98449407
13785	RNF14	99.76227982	-0.142645884	0.212709964	-0.459258194
0.645976965	19.41718281
13786	SDF2	102.92092	0.226762872	0.289247612	0.818550826
0.412042722	11.81198148
13787	SLC29A1	119.4258621	0.119646986	0.272796504	0.42699228
0.662116874	18.0752512
13788	SLC29A6	78.40425152	0.402224615	0.267768441	1.092988961
0.27295982	10.58022427
13789	SLC42A2	124.6860761	-0.272082122	0.256915522	-1.452158725
0.146457476	12.14457042
13790	SPTLC1 104.9972204	-0.114750111	0.296957929	-0.286418726
0.699186576	11.4741444
13791	ST2GAL2	122.8206769	-0.211544025	0.266115266	-0.794922561
0.426652151	15.47495545
13792	TBRG1	117.8150842	0.24766059	0.281296227	0.880112401	0.27879788
25.21075122
13793	TCF12	97.26291158	0.225172582	0.202682915	0.741475964	0.4584049
14.47519911
13794	THUMPD1	105.0454211	0.025292902	0.288964799	0.122481712
0.902517529	15.18215114

13795 WDR45B 108.6089169 0.222099941 0.282171597 0.826092859 0.408751418 17.84294494
13796 CSNK2A1 122.4119412 -0.218468195 0.256265848 -1.24224111 0.214147616 21.22012721
13797 DNAJB12 126.6125722 0.086606421 0.272266412 0.217977647 0.750501892 11.07187741
13798 FASTKD5 116.2250921 -0.1092229 0.272022628 -0.400404524 0.688858592 17.11578725
13799 IMP2 119.6252744 0.005954716 0.272000522 0.021892294 0.982522872 17.9088095
13800 LSP1 111.8062546 0.417215294 0.28614727 1.458044291 0.144828225 14.10191114
13801 MFSD14A 121.6527421 -0.185410068 0.266182967 -0.696551211 0.486082698 17.00002148
13802 PISD 107.5815028 0.225828529 0.281827484 0.826782289 0.402714292 12.42417421
13803 PRKAB1 152.2622041 -0.22996972 0.256211774 -1.287878821 0.197788146 17.8501708
13804 SCFD1 121.7005925 -0.222911251 0.268810668 -0.870171014 0.284206954 19.52217705
13805 SEMA7A 125.2776602 -0.148408762 0.26727429 -0.555059961 0.578852648 11.81421187
13806 TBRG4 99.48725821 0.242076062 0.297614277 0.816748289 0.414072222 19.98049545
13807 UBE2G2 117.0247284 0.081569858 0.276242976 0.295282012 0.767777692 19.51251987
13808 WDR61 114.215864 -0.012065454 0.274749566 -0.042914271 0.96497266 10.1901921
13809 ACAT2 144.2692229 -0.197821504 0.25759488 -0.767994702 0.442490222 18.21848514
13810 ASCC2 101.0297929 0.426797917 0.291245822 1.464918556 0.14294214 11.49409295
13811 ASH1L 112.5648621 0.122714658 0.282162849 0.424905669 0.662620899 11.42908172
13812 ERCC1 127.5694509 0.154854786 0.270159181 0.572198208 0.56651042 18.10720844
13813 GPBP1 106.8884521 -0.01529062 0.289402012 -0.05218058 0.957588029 15.21558848
13814 LMBRD1 105.9605226 0.284695094 0.288646681 0.986209951 0.222981028 24.1514008
13815 LSM14A 111.254896 -0.091200448 0.280292759 -0.22525964 0.744984574 18.85792551
13816 PFDN1 110.7824158 -0.116228988 0.284469614 -0.408968067 0.682562096 15.80954414
13817 PSME2IP1 116.8221294 0.124112066 0.27522204 0.450924472 0.652026777 12.99492481
13818 RIPOR1 120.9222778 -0.022696627 0.262229724 -0.127962662 0.89817772 11.05875074
13819 TCHP 117.1226277 -0.142241246 0.277846052 -0.515541774 0.606174486 11.14412401
13820 TM6SF1 117.7520585 -0.09567774 0.270400854 -0.25282668

						0.722461282	19.98417254
13821	VPS12C	105.8219921	-0.052728562	0.299506556	-0.179290271	0.85762127	10.55770244
13822	ACAP1	90.50885458	0.217222228	0.225884225	0.666900455	0.50482572	10.02128877
13823	ALDH9A1	128.7051902	-0.204214212	0.255162874	-0.800229255	0.422520057	12.01100191
13824	CARD16	122.7782262	-0.052008525	0.262895875	-0.197829407	0.842178527	11.11141588
13825	CAVIN2-AS1	115.0946645	-0.245845254	0.286224109	-0.858596115	0.290562282	14.11917997
13826	CHTOP	140.2025266	-0.225262652	0.255152886	-0.92242774	0.256200292	14.21921511
13827	EXOC7	114.2871079	-0.215472716	0.289668798	-1.089084216	0.27611675	17.40124104
13828	FBXO24	146.2955022	-0.264681951	0.252147056	-1.049712625	0.292850252	14.85501701
13829	IQGAP2	102.2772895	0.225029829	0.290644272	0.774244842	0.428786014	18.82812815
13830	LBR	101.1182201	-0.281209209	0.227581877	-0.858429766	0.290649678	14.42910549
13831	PLAA	97.22225421	0.118270067	0.21094972	0.280251086	0.702684819	10.59714822
13832	RAD22A	124.0992201	0.216624411	0.272058815	0.796241104	0.425891911	12.19218975
13833	RSL24D1	107.0944211	0.222255902	0.282627897	0.789928752	0.429569279	12.904411
13834	SUCLG1	124.2041127	-0.127195772	0.272190958	-0.504042257	0.614221612	20.19524081
13835	UCHL5	104.1209291	0.212144272	0.287269158	1.08659201	0.277217158	10.08111477
13836	ZNF706	125.2105285	-0.22009855	0.255824021	-1.251245088	0.210845072	17.8501708
13837	AL121748.1	107.7490281	-0.229959946	0.202272081	-0.79122291	0.428807507	10.11548071
13838	CAMK2G	112.6289775	-0.006809961	0.275550422	-0.024714029	0.980282065	11.72971201
13839	COX20	108.5776727	0.214550108	0.282008975	1.115290416	0.264682155	19.15447514
13840	EXOC6	102.1012218	0.120011742	0.295244022	0.406482211	0.68428759	12.19911042
13841	FAM126A	101.9074201	0.211046494	0.291227566	1.067649799	0.285678511	11.18015471
13842	NHP2	117.9742515	-0.051924606	0.272490902	-0.190555275	0.848872955	11.72024544
13843	NKIRAS2	118.5826912	0.201952625	0.277862212	0.726809247	0.467242817	10.98149475
13844	NKTR	109.801217	0.029998917	0.292564205	0.12625266	0.891621547	14.28402914
13845	NOC2L	110.270012	0.169214047	0.291261691	0.580969116	0.561261269	19.58942247

13846	PLAC4	96.28581746	0.291212924	0.208005966	1.270471899	0.202916587	12.82754914
13847	PPIG	114.6145552	0.108215405	0.290824211	0.27242994	0.709572766	52.21908197
13848	PRKAG1	106.6695268	0.277129652	0.284729219	0.972210087	0.22029921	11.5519929
13849	VPS27A	102.7927241	0.112599741	0.291676707	0.286042966	0.699464849	14.0174242
13850	C1orf56	129.9801702	-0.202509959	0.259717046	-0.779722029	0.425548022	25.91928498
13851	LUC7L2	122.8447904	-0.095542227	0.257986962	-0.270227851	0.711120775	19.54444915
13852	POLR2K	106.6806769	0.226142442	0.287718212	0.820745262	0.411791282	17.42119729
13853	RALA	112.4025956	-0.14091021	0.285071785	-0.49429764	0.621095992	12.9024174
13854	RRM1	120.7794162	-0.29652287	0.258860124	-1.145498405	0.252002756	19.78072901
13855	SMC2	112.2587206	0.028150807	0.277066651	0.127695414	0.890481144	11.04094422
13856	TAF1D	105.2021529	0.280219484	0.288161082	0.972440419	0.220821492	12.4877944
13857	TM9SF4	99.48924227	0.227288221	0.222048102	0.702572952	0.481698127	11.21192222
13858	TSR1	112.1228545	0.147852527	0.287047922	0.515079628	0.606497278	11.5224272
13859	UBQLN1	116.714148	0.22706819	0.278264906	1.175284258	0.229841019	18.44095709
13860	AASDHPPT	96.92849641	0.25622295	0.209058411	1.152629557	0.24905826	11.08490188
13861	AC044849.1	115.6582214	0.167898292	0.272942967	0.612895421	0.529945485	19.42492004
13862	CASC4	114.9220526	0.026542175	0.274484907	0.122122641	0.89408769	19.04909091
13863	CCAR2	102.7474164	0.072122924	0.29697545	0.242895276	0.808086525	14.47481444
13864	CD44-AS1	129.0765612	-0.252412888	0.265828022	-1.225671496	0.184948522	15.47477127
13865	CEBPZ	110.7414271	0.081718924	0.279840928	0.292019224	0.770271918	14.41478917
13866	CLK1	127.1652926	-0.249164996	0.26629515	-1.211195479	0.189791729	19.42711747
13867	DARS1	119.4467475	0.204792544	0.270154601	0.758060544	0.448414729	19.5201405
13868	DEK	115.6186265	-0.006011012	0.272252112	-0.021989916	0.982456	12.41198001
13869	DENND4C	112.8520095	-0.42196122	0.202271109	-1.295969407	0.162722708	11.49115741
13870	HAT1	118.22169	-0.047465284	0.270495277	-0.175475462	0.86070605	10.49557517
13871	METAP1	120.7520152	-0.056661808	0.267059217	-0.212169275		

0.821974898 14.12417478
13872 NBR1 86.52454898 0.192989162 0.254284978 0.544574895
0.586045925 14.42271125
13873 NNT 110.2778651 -0.170757241 0.291949998 -0.584885568
0.558624662 19.59802851
13874 NPL 126.2079474 -0.020225022 0.265885524 -0.076066652
0.929266071 19.18147744
13875 P4HA1 127.0845522 -0.086617421 0.288022694 -0.200721226
0.762619448 20.47821855
13876 PDLIM7 118.5406046 0.192292265 0.275682806 0.697512822
0.485481292 19.79118051
13877 SLC25A11 149.0956482 -0.212742452 0.252052761 -1.229824129
0.215026748 19.54414454
13878 SYK 127.6582202 -0.222692629 0.271205117 -1.19209817
0.222820928 14.4214011
13879 TBK1 114.0927087 0.124741727 0.28281075 0.474759067
0.624958712 10.17242012
13880 TOM1 127.7616288 -0.202287876 0.255744987 -1.182280462
0.227054772 12.94244184
13881 ATP1A1 124.6415492 0.124827725 0.266776771 0.505422816
0.612254875 18.54500584
13882 CNTRL 115.6108252 0.258288978 0.278281899 0.928155871
0.252226722 18.59041285
13883 CXXC1 105.2588122 0.129752784 0.292168772 0.478222281
0.622412652 15.89424429
13884 GMFB 97.81797167 0.227780822 0.204965265 1.074812662
0.282458162 19.85977818
13885 GRPEL1 102.4552228 0.220088429 0.289211925 1.10676086
0.268297225 19.57545584
13886 MAPK1 92.99562152 0.195258968 0.221561114 0.607221952
0.542702626 14.95922202
13887 METAP2 125.6629927 0.217462661 0.271861127 1.167742018
0.242910842 12.05124417
13888 NR1H2 117.7748812 0.128570064 0.275778262 0.502469122
0.615227561 18.85788428
13889 SNW1 120.5252221 0.009026626 0.264521846 0.024122021
0.972779051 17.419854
13890 WTAP 112.0918089 -0.121895911 0.285078582 -0.427587052
0.668951794 18.7845158
13891 ZFR 118.6915194 -0.141800405 0.272225228 -0.51896802
0.602782025 21.581514
13892 ALS2 120.4501779 -0.275194689 0.287148167 -1.206624005 0.19124044
14.58921111
13893 BCCIP 108.7967288 0.24149825 0.282204525 0.85575609
0.292122708 19.78914505
13894 CROT 102.2982746 -0.014475417 0.214255949 -0.046062508
0.962260429 14.15810414
13895 DOK1 129.6842457 -0.160419415 0.252425949 -0.625485616
0.525111745 11.12111815
13896 DYM 124.2448154 0.084958254 0.26644168 0.218862478
0.749820799 17.79454105

13897	FKBP2	112.8727009	0.152128899	0.280614722	0.542127272	0.587720822	18.25947145
13898	LAMTOR2	121.2820219	-0.021129784	0.258792514	-0.081647269	0.924927212	12.41889197
13899	NUDT2	106.6626152	-0.184529272	0.207882289	-0.599282556	0.548917807	14.90954941
13900	PLA2G15	122.2729226	-0.26010989	0.258207422	-1.006977996	0.212945248	18.40171518
13901	PYURF	122.8126056	-0.128647765	0.270112102	-0.512297122	0.607742497	14.72702781
13902	STK17B	129.2622452	-0.152619292	0.261217467	-0.587864995	0.556622914	24.0154844
13903	STX7	101.7404907	0.059514074	0.209022509	0.192581919	0.847286406	14.4494774
13904	TMED5	109.492757	-0.162497551	0.201998591	-0.52807287	0.590526051	15.05804955
13905	TRIP12	99.62985594	0.078998556	0.210920295	0.254071512	0.799440211	19.22822074
13906	UBE4A	106.2928822	-0.066962955	0.211452129	-0.215005602	0.82976295	14.54104059
13907	ZMPSTE24	106.618296	0.142812429	0.289627784	0.492092988	0.621946885	22.97081152
13908	ATP5MC1	109.8664697	0.410711974	0.281659609	1.458185559	0.144789402	12.1118055
13909	BFAR	118.952257	-0.028225242	0.275081779	-0.129222089	0.889194845	19.04018111
13910	CCNL1	104.1442986	0.214875195	0.291817794	0.726222422	0.461527811	17.19287104
13911	CHID1	117.250126	0.260680041	0.272925607	1.216659945	0.187952626	14.19528182
13912	LAMTOR2	122.6476158	-0.122802727	0.265842628	-0.461925962	0.644127242	19.84191429
13913	LPCAT2	92.46252887	-0.192426681	0.286212775	-0.498240072	0.618214841	15.14240174
13914	MRPL42	106.6945489	0.22177222	0.284622215	1.165620799	0.242767795	11.15478557
13915	MRPS21	120.022527	-0.10289879	0.261012711	-0.294227529	0.692412062	10.0924149
13916	MRPS25	117.6145609	0.241922486	0.279427872	0.865777922	0.286611952	14.44494457
13917	NASP	120.0765128	0.108204688	0.262742228	0.410644259	0.681222405	14.1521248
13918	PRPF28A	122.2826265	0.026292174	0.26781077	0.125514245	0.892205202	21.4155001
13919	RAB11A	127.2278556	-0.202911777	0.259264998	-1.168248124	0.242666267	11.44180294
13920	SDHC	117.2775922	0.098886242	0.271012112	0.264877578	0.715202792	11.02222147
13921	SNRPE	121.9592218	0.108951022	0.261795492	0.416168482	0.677286721	15.71779057
13922	SNX14	119.4228112	0.108712492	0.268828211	0.404282427		

						0.685921512	10.5109421
13923	VBP1	116.4771711	0.147252751	0.271922264	0.541524974	0.588145789	17.41871519
13924	ZNF451	108.2224022	0.042765087	0.288692015	0.151597845	0.879504122	11.54149044
13925	ACTN4	142.8576578	-0.269858152	0.247928022	-1.088452566	0.276294925	14.42099801
13926	ADSS2	101.898467	0.211995604	0.297264048	1.04955714	0.292921771	17.01977771
13927	AL254722.2	120.1498876	0.150757472	0.267628865	0.562286922	0.572229521	19.51059221
13928	C5orf15	125.8904094	-0.298726857	0.270425582	-1.104691552	0.269292265	21.41479401
13929	C6orf89	121.7001222	-0.019192112	0.269656166	-0.071172524	0.942260441	10.81944124
13930	DDX22	104.4702528	-0.016892972	0.205492161	-0.055297282	0.955901548	18.2449419
13931	DNM1L	94.20228206	0.222169556	0.225088216	0.714172794	0.47511972	14.08195019
13932	GRK2	115.2924516	0.200298791	0.278221571	0.71964092	0.471746104	19.2898199
13933	IFNGR2	161.9782602	0.161609064	0.276707226	0.584042504	0.559191042	12.04527181
13934	MRPL16	127.8805712	-0.067021261	0.26065575	-0.257162944	0.797052217	14.47812228
13935	PDCD10	105.4276782	0.126264529	0.29894452	0.456152299	0.648279745	27.88414844
13936	PTP4A2	116.7127458	-0.11978874	0.280284999	-0.427229489	0.669212185	22.75140171
13937	RNF6	102.2949066	0.100612952	0.20518201	0.229682994	0.741628749	10.5441085
13938	RPS15A	117.8261592	0.062717875	0.271629518	0.220894918	0.817296429	21.22849111
13939	SH2B2	127.2482252	-0.227192148	0.262099496	-0.901524028	0.26720445	15.94710014
13940	TMX2	110.7552492	0.212256144	0.280600082	0.75679288	0.449172959	14.91149424
13941	USB1	118.9144572	0.220997279	0.271144629	0.851922818	0.294250826	11.99747492
13942	WSB2	119.8229249	-0.106246812	0.272145856	-0.289240752	0.697024094	15.90547274
13943	ACP2	122.2762916	-0.008229622	0.264505652	-0.02152912	0.974847561	19.11519484
13944	CCDC18-AS1	141.2207571	-0.158842675	0.250559887	-0.622954928	0.526110227	11.47545149
13945	CSK	108.8287552	0.240921194	0.282544205	0.852684659	0.292824201	11.01814255
13946	LRPPRC	91.21287952	-0.06012855	0.292821292	-0.152675869	0.878652892	15.58145449
13947	MAEA	121.2140805	-0.052647548	0.259521202	-0.202864072	0.829241277	19.59742542

13948	MEAF6	112.7450592	0.18251671	0.277085754	0.662210159	0.507772467	11.55401995
13949	TOLLIP	106.9216666	0.106289564	0.299960728	0.254678209	0.722820602	12.15289419
13950	UXT	129.1694716	-0.129462444	0.260170152	-0.526042211	0.591928686	19.41498149
13951	VPS29	98.59661981	0.066207426	0.222227011	0.205461922	0.827211244	10.15910221
13952	DCAF11	127.7254711	-0.25570654	0.274191128	-0.922584992	0.251024285	15.925859
13953	GNPDA1	121.6658995	-0.09247795	0.257412822	-0.259257902	0.719402164	17.97277109
13954	KPNA1	122.4224729	-0.152688972	0.267846806	-0.570060829	0.568626441	27.01897428
13955	RNF216	110.6682974	0.208042141	0.280747767	1.097220269	0.272545115	14.41954441
13956	SMIM12	109.2122571	0.277989027	0.281904222	1.240841972	0.179971766	41.9120958
13957	AKAP9	118.5957069	-0.028625914	0.27220875	-0.104812214	0.916522945	11.72214987
13958	ASH2L	115.5227229	0.097872025	0.278452998	0.251488495	0.725221902	19.54720144
13959	CLK2	126.6114089	-0.014724514	0.268176241	-0.054942278	0.956182572	10.78544851
13960	CNNM2	121.576967	0.225614202	0.2748818	0.857147227	0.291262462	11.59554441
13961	CUX1	122.6297512	0.264578724	0.267442929	0.989286707	0.222522886	18.91109278
13962	HELZ	116.1980645	0.080187271	0.274502409	0.292117942	0.770196448	14.71574448
13963	RNASEH2C	122.5260848	-0.022690478	0.260506209	-0.090940164	0.927540126	10.41704441
13964	TIMM22	122.229776	0.220251049	0.266872824	0.862774299	0.288261518	11.51498121
13965	TYK2	124.7572928	0.242058942	0.267664179	1.277940679	0.201270242	14.94111844
13966	AC025165.2	122.2092589	-0.072915155	0.256001229	-0.284822446	0.775779409	17.09210242
13967	AC120057.2	120.2941522	0.057555474	0.268717785	0.214185577	0.820402242	14.82114241
13968	BATF	125.8269245	0.24978249	0.271729217	1.28719868	0.198025047	14.11457754
13969	DHX8	102.8461246	-0.026729255	0.2222891	-0.082966676	0.922878027	12.00412102
13970	GTF2B	125.4960977	-0.022527197	0.259746294	-0.086727695	0.920887962	12.92744174
13971	ST7	129.9772705	0.06612012	0.258594025	0.255729525	0.798159695	11.18945081
13972	DALRD2	147.627162	-0.247818294	0.246747862	-1.004228567	0.21521545	12.14140187
13973	DAXX	142.8042767	-0.076244126	0.251408991	-0.2026651	0.76128205	

17.58180919
13974 DDX29B 115.6066847 0.057520688 0.27961925 0.205746449
0.826988972 17.10155948
13975 DELE1 109.6540125 -0.020012772 0.298274708 -0.067095104
0.946505992 14.11255817
13976 DHX20 122.6085102 0.218425498 0.266557982 0.819467105
0.412519962 14.10410492
13977 FAM50A 125.2948775 -0.127526774 0.266575724 -0.515901224
0.605922229 44.75914141
13978 ITPR1 125.5265245 -0.222085557 0.261521112 -1.272646909 0.20278858
28.55484101
13979 KLHL9 119.1287814 -0.041157889 0.276495774 -0.148855299
0.881667725 42.19710145
13980 POLR2L 114.8268257 -0.086019101 0.286941157 -0.299779582 0.76424529
50.02181199
13981 PSMA2-AS1 122.5151941 0.009806002 0.265524 0.026929266
0.970541225 11.79209291
13982 RASGRP4 129.9161282 0.292662895 0.268047286 1.464904775
0.1429469 59.04282714
13983 SLC1A5 199.0229225 -0.012291907 0.52862421 -0.025144226
0.979929826 14.49574747
13984 TMEM109 162.0910194 -0.249728228 0.242509227 -1.029809221
0.202099571 28.11407145
13985 TOR1AIP2 122.2625222 0.120819209 0.264248461 0.495061285
0.620556791 44.52840191
13986 TXNDC17 141.5417245 0.059922786 0.255405124 0.224657727
0.814474401 25.47911174
13987 WASHC5 102.8462227 0.12772404 0.208951042 0.412444275
0.679281142 41.5552024
13988 ZMIZ1 120.449217 -0.18911156 0.265664622 -0.711842195
0.476561881 11.75429057
13989 AC010768.1 127.4806818 0.09499278 0.255972171 0.271104429
0.710559745 21.04520941
13990 ACOT9 126.2571682 -0.124500802 0.252842242 -0.49046507
0.622804841 28.15178178
13991 CHFR 122.2898049 -0.027974969 0.262925744 -0.106298744
0.915265989 14.59402721
13992 GATA1 122.2066442 0.226947164 0.288122885 1.169452287
0.242221414 17.92491855
13993 GORASP2 160.4682954 -0.106955122 0.248200268 -0.42074899
0.666650894 10.74085151
13994 IFI6 126.4814802 -0.022558546 0.262082455 -0.089889824
0.928274767 24.17751418
13995 KLHL6 115.1702989 -0.056150767 0.286887204 -0.195724127
0.844826099 28.81024228
13996 LYPLA2 121.8062157 -0.029912875 0.261027175 -0.114600615
0.908761692 15.48001411
13997 PRR12 129.2951425 0.259210962 0.261721901 0.990788167
0.221789029 18.22410251
13998 TMEM179B 159.1708278 -0.057272661 0.254660202 -0.225294874
0.821749887 14.49451142

13999	TOMM22	122.0220422	0.184940208	0.259146962	0.71264992	0.475442681	21.48044899
14000	UBR2	107.1298248	0.102412026	0.201252959	0.229840819	0.722976406	14.01051782
14001	VPS25L	124.9824067	-0.19248817	0.254628155	-0.759855272	0.44724104	21.14512788
14002	AAMP	126.4871499	0.056225455	0.262699849	0.214029262	0.820524228	10.81117442
14003	FIS1	127.5628987	-0.222458542	0.259424411	-0.861262825	0.289028244	14.10801024
14004	FOPNL	121.7052886	0.127226225	0.260061626	0.528091484	0.597425841	11.18924941
14005	HMG20A	101.9879891	0.229289658	0.208920421	0.774898201	0.428299756	12.49847087
14006	NDUFS7	124.6857598	0.028828187	0.269526676	0.106991699	0.914795565	14.00817291
14007	PPP2R5C	126.8124522	-0.208822044	0.262441298	-0.79572859	0.426189806	19.5047441
14008	SEH1L	105.5592251	0.256155982	0.201265749	0.850265866	0.29517729	11.94741448
14009	TSPAN2	151.4520222	-0.21505228	0.241072952	-1.20687527	0.191255076	12.78015794
14010	ACADVL	157.0792472	-0.222974826	0.250729516	-1.22797112	0.184187648	20.11140784
14011	CHCHD2	117.0112215	0.15920216	0.274644127	0.580021192	0.561892582	22.11828994
14012	EPS15	102.149404	-0.077462846	0.240895292	-0.227226412	0.820229914	11.49954115
14013	MS4A2	122.1852954	-0.058522712	0.25616876	-0.228452747	0.81929251	14.48255149
14014	PCNX4	122.7285796	-0.12500262	0.272286122	-0.495811624	0.620027222	19.85981717
14015	PSMD12	141.8272449	0.166249206	0.259166979	0.641475647	0.521212698	15.47157012
14016	PUM1	125.6222259	0.12462752	0.262199812	0.512452916	0.607622829	14.71007471
14017	RASSF5	164.8892206	-0.224104481	0.241518771	-0.969201291	0.222294826	10.84197287
14018	RBM17	125.012261	0.021752969	0.264459928	0.082258092	0.924441479	15.48828107
14019	SUMO1	121.5110454	0.109222426	0.256921062	0.425159482	0.670720418	14.40418115
14020	TRERF1	108.1572248	0.282211186	0.286291466	1.228416927	0.180760564	18.44909872
14021	AP2B1	128.0220682	-0.024951924	0.262142462	-0.122824596	0.894222102	47.12294844
14022	ASB8	118.2784752	-0.071286769	0.282682276	-0.2516425	0.801217405	11.70190991
14023	CCDC115	141.5016849	-0.091927296	0.248296652	-0.27027199	0.711179842	15.14815245
14024	DNAJB6	122.9990246	0.080287626	0.269002276	0.298464522	0.76524865	

17.78519871
14025 FIBP 156.0241216 -0.064754879 0.247262824 -0.261885769
0.792409512 17.11558494
14026 GABARAPL2 126.2864216 -0.122724911 0.252822967 -0.528927069
0.596856044 14.49404479
14027 GBF1 118.2072662 0.189528012 0.271894242 0.697101972 0.48572898
10.52112245
14028 GOLGA4 145.1825581 -0.212911674 0.251725817 -1.242016102 0.2128619
11.19150499
14029 GPAT4 142.5282009 -0.29599402 0.249995012 -1.182999741
0.226412165 15.21140122
14030 KCTD10 126.1286587 0.264228428 0.270178872 1.248128121
0.177612962 21.48751744
14031 SLTM 121.2722902 0.219829722 0.268710677 0.818090795
0.412205245 14.15020015
14032 SRPRB 141.577292 -0.165586849 0.252210197 -0.652950161
0.512142916 11.18924941
14033 TMA7 126.2269298 0.220729252 0.26454178 0.82442151 0.4040425
18.27274819
14034 UBE2A 125.060799 0.211181651 0.277471291 1.121490942
0.262078945 19.87290288
14035 VPS4B 105.6279717 0.158549262 0.204559584 0.520585695
0.602655417 18.84054847
14036 ARHGAP1 162.4541254 -0.095292167 0.245250521 -0.288962126
0.697204156 19.11801899
14037 ATG7 124.1299298 0.124209726 0.264500042 0.469979984
0.628269218 11.7524272
14038 DCAF12 125.2414962 0.014771988 0.266144495 0.055502629
0.955727221 10.15140547
14039 EIF5A 172.0280265 -0.108642719 0.248489484 -0.427212542
0.661957214 12.50419128
14040 GAPVD1 125.5697124 0.204894002 0.265790252 0.770885777
0.440774628 12.51558182
14041 H4C2 125.2624522 0.264662656 0.262924267 1.281697569
0.167064581 11.07819545
14042 HNRNPA2 129.0192215 -0.04217197 0.251294621 -0.167818821
0.866725807 41.48412742
14043 LYST 115.2659454 0.055092011 0.286905124 0.192025189
0.847722475 14.17275154
14044 METTL17 129.7595429 -0.118246825 0.268840844 -0.440211512
0.659782922 17.11404094
14045 MFN2 128.2016874 0.257629091 0.262601117 1.261910014
0.172226286 21.40240274
14046 PARP4 118.9670665 0.157959811 0.271778742 0.581207282
0.561100692 11.07147501
14047 SNRPC 129.2417622 0.252260272 0.270926904 1.200204475
0.192520897 14.75941914
14048 SPCS2 122.2250287 0.285820765 0.269089161 1.422829862
0.151617967 11.81014299
14049 THOC2 124.582777 -0.055872188 0.254580895 -0.219471254 0.82628297
14.42140002

14050	TRAF2IP2	117.4669609	0.172082822	0.274109786	0.627787995	0.520142822	12.48449414
14051	UBAC2	116.7696825	0.266662077	0.288528168	1.27076109	0.202812655	14.44810911
14052	ZC2H15	128.257591	0.251512194	0.265822519	1.22225672	0.186049292	11.08485175
14053	ZNF628	88.22294672	0.518562267	0.251087512	1.477017408	0.129670971	19.82449125
14054	AC091564.6	155.9291191	-0.221010722	0.229525894	-0.922662271	0.256182222	14.99709284
14055	ANAPC11	151.9876997	-0.199292541	0.241905822	-0.822842471	0.410028504	12.09247515
14056	ATF7IP	122.2459621	0.026464229	0.268855124	0.098422452	0.921588112	15.7501719
14057	ATP6V1C1	124.704995	0.006494971	0.254206656	0.025549964	0.979616296	12.94201101
14058	EMC1	111.0471646	0.192692968	0.288926229	0.666927687	0.504818225	14.1975977
14059	HMGB2	147.7047022	-0.012202247	0.246941757	-0.052867951	0.957040271	12.01571495
14060	HNRNPU	116.4697402	-0.149972107	0.200411985	-0.499221452	0.617622282	14.14718098
14061	NELFE	129.9729269	0.044650282	0.258621292	0.172647674	0.862928276	19.15441577
14062	OGDH	142.7089099	-0.064901812	0.24848692	-0.261188022	0.792947507	18.24474158
14063	SELENOK	125.9527702	0.222261015	0.262885592	1.229664251	0.218822859	24.40715419
14064	SETX	117.4274152	0.172978059	0.276511455	0.625572852	0.52159515	11.044142
14065	SF2B6	120.2994659	0.225570822	0.258789927	0.910278212	0.262675806	18.41244227
14066	VPS41	122.1279895	-0.252985024	0.268742525	-0.945086841	0.244614542	19.82429112
14067	WAC	114.6296517	0.108462221	0.2889102	0.275421951	0.707246682	14.95474291
14068	C1orf162	126.7258428	0.069667469	0.252776167	0.275609224	0.782848147	17.20472414
14069	CHD1L	118.4798426	0.026642611	0.282060558	0.129455022	0.896997612	14.91521924
14070	GLRX	142.4771661	-0.089816956	0.246586042	-0.264241849	0.715677419	19.24425077
14071	HIF1AN	157.1068004	-0.28722427	0.22792672	-1.207658624	0.227178596	14.71291158
14072	KXD1	128.0490198	0.220295647	0.255040211	0.902977794	0.266527681	14.42141951
14073	METTL2	129.9202045	0.056776748	0.262209886	0.216521682	0.828572222	10.99278591
14074	NCBP1	145.825119	0.222208585	0.259547152	0.856129559	0.29192059	11.55594881
14075	STAT6	161.2262261	-0.182616154	0.244799577	-0.750067285		

						0.452214182	15.5522948
14076	TECR	124.8010952	0.220052576	0.256001984	0.898629818	0.268844542	14.48754075
14077	TWF2	129.2067825	0.025890271	0.2625722	0.098602864	0.921452596	18.05171444
14078	AC006020.1	154.0562478	-0.2161068	0.240077282	-0.90015514	0.268027696	25.11440718
14079	ACTL6A	120.5421726	0.251185202	0.269714452	0.921200861	0.251697957	22.82191582
14080	ANKRD44	127.7022259	-0.027646622	0.264858524	-0.10428264	0.916865699	18.40718448
14081	CACYBP	119.1824275	0.128217902	0.274649064	0.467206769	0.640251951	19.24475245
14082	COG1	121.8259505	0.112867611	0.257114929	0.442866571	0.657862248	14.91880785
14083	ECH1	122.9924461	0.010252955	0.269071218	0.028104972	0.969602986	11.14887894
14084	GLOD4	129.5527987	-0.092929965	0.249856121	-0.275976229	0.706924561	10.05445444
14085	MRFAP1L1	127.1959012	0.175297814	0.254891264	0.687725298	0.491619425	19.54441414
14086	NDUFS1	125.9177978	-0.090620626	0.271775798	-0.222475704	0.728775226	10.1187217
14087	NUP92	125.64262	0.189956478	0.259105294	0.722124652	0.462482406	14.24981019
14088	PSMC4	126.6670965	0.167040191	0.26282787	0.622116812	0.526657264	11.09520772
14089	SMU1	118.5284286	-0.062991776	0.288221051	-0.218546112	0.827002624	15.21418774
14090	TET2	121.1582187	-0.179942282	0.266192965	-0.675988499	0.499047947	10.08101127
14091	TMEM87A	147.2174202	-0.140875906	0.244982274	-0.57504272	0.565262406	15.44574449
14092	TRIM12	151.6926796	0.006800882	0.245489208	0.027702276	0.977898721	14.72991114
14093	WDR55	117.7652566	0.275754014	0.272294568	1.008999251	0.212974992	21.89881994
14094	AGTRAP	166.0454049	-0.24268562	0.225409228	-1.025156982	0.200595605	17.95475794
14095	EVI2B	114.697792	0.287672495	0.277920524	1.294852972	0.16205988	14.87070708
14096	HAX1	142.5164582	-0.090240527	0.246528246	-0.26602058	0.714242257	17.10809774
14097	MAGT1	126.8122441	0.11804012	0.262812155	0.449142547	0.65222882	17.0702494
14098	NFX1	122.2561129	-0.059716552	0.262149105	-0.227796121	0.819804724	12.49818221
14099	PAK2	125.2028065	0.284660205	0.262828922	1.082062052	0.278780427	17.72147718
14100	RMND5B	151.0212122	-0.056557015	0.246897872	-0.229070482	0.818814125	17.18147977

14101	RNF114	129.2249558	0.07925297	0.251520492	0.215082951	0.752698672	12.99482154
14102	SPAG7	148.4616159	0.020971228	0.246490041	0.085079414	0.922198256	17.19954144
14103	STARD7	119.8672842	0.207424959	0.278501845	0.744788455	0.456299615	14.45421481
14104	STXBP2	122.8692272	-0.149419624	0.261197771	-0.57205551	0.567284268	10.20710188
14105	UNC45A	156.7627797	-0.122905458	0.22987298	-0.554062671	0.579525272	17.40150524
14106	USP14	145.745677	0.015599629	0.247012249	0.06215226	0.949644464	10.58595484
14107	ACSL5	125.6057082	0.126801865	0.257770928	0.491916858	0.62277812	17.42911448
14108	BUD22	125.9544476	0.107724461	0.252912269	0.425926082	0.670154422	14.14707825
14109	ELOB	162.2810421	-0.219414192	0.225827707	-0.920400299	0.252162812	10.21111955
14110	NFYC	121.6882202	0.161742956	0.270157868	0.59870162	0.54927187	11.81414454
14111	SLCO2B1	171.2699091	0.048026476	0.251097882	0.191265954	0.848217222	11.55954524
14112	SMAD2	125.1556974	0.276442789	0.256427566	1.078058002	0.281007872	17.00549978
14113	TAP1	125.9605211	0.070288161	0.272212622	0.257621429	0.796691264	11.57574418
14114	ALDH18A1	126.57677	0.180979697	0.264600024	0.682974578	0.492991219	18.20400014
14115	CIAO2B	129.8747772	0.075552526	0.250077676	0.202120275	0.762560277	17.41900154
14116	FAH	124.1666825	-0.1646481	0.264412842	-0.622692277	0.522486101	17.45541244
14117	NFE2L2	152.7200879	-0.040297498	0.242542762	-0.165462745	0.868578984	11.12752754
14118	PRDX4	127.9866451	0.146829572	0.267616276	0.548694271	0.582215279	11.59042405
14119	SLC25A4	156.2729217	-0.124117548	0.229297408	-0.518674852	0.602987501	14.70204142
14120	SSB	156.2182112	0.089992258	0.246585461	0.264957679	0.715142998	12.45211801
14121	SYNCRIP	146.6224661	0.126825109	0.247849081	0.511702964	0.608858912	11.21471754
14122	TANK	127.2621954	0.111262921	0.252261106	0.441062529	0.65916772	15.71244158
14123	TCN1	186.8272765	-0.126785659	0.247172795	-0.512942419	0.6079909	10.00855247
14124	TIAM2	129.7795219	0.202276182	0.251720889	0.802928617	0.421422426	14.0984044
14125	TMEM258	122.1762202	0.050622087	0.256999225	0.196972695	0.842848122	14.1811049
14126	TNRC6B	122.4614429	0.012124471	0.257505251	0.047084266		

0.962445988 17.88050295
14127 TPR 152.6646295 -0.181296292 0.244220127 -0.742217858
0.457894774 21.2591197
14128 VPS26A 120.7900866 0.159725096 0.285254875 0.559741955
0.575655461 12.41411911
14129 ZNF460 144.2026249 0.107580182 0.249652017 0.420918819
0.666527299 11.58770957
14130 ARPC1A 122.6206625 0.129062094 0.259260282 0.497812827
0.618615974 11.21474104
14131 BRD7 129.274222 0.092952272 0.26256578 0.254018989
0.722224652 21.81281852
14132 DCP2 108.5124191 0.078457427 0.217412918 0.247177799
0.804770626 14.51947019
14133 EHD1 124.7518427 -0.112644928 0.262244514 -0.429541627 0.6675291
18.44995104
14134 FTSJ2 124.1840429 0.179441562 0.260896245 0.687788981
0.491585676 15.48912414
14135 HACD2 129.2529056 0.089420122 0.251728792 0.255224057
0.722421742 24.47711744
14136 LAMTOR4 168.1590671 -0.207284654 0.225979217 -0.878401788
0.279725714 14.42750701
14137 MACROH2A1 142.2279491 0.18985954 0.249928856 0.759622945
0.447479402 12.11111511
14138 TLK1 146.0580492 0.088941192 0.247050484 0.26001222
0.718827995 15.44271414
14139 TNPO1 120.0024264 0.277595449 0.271768944 1.021429189
0.207046407 18.08442914
14140 TRA2A 122.0062642 0.21274081 0.270058589 0.791460887
0.428675092 11.01527599
14141 UBE2L2 156.2509875 -0.29079869 0.228820406 -1.217594921
0.222277945 14.14155915
14142 ZDHHC12 145.8244127 0.05419292 0.245742555 0.22052128
0.825457414 11.989958
14143 ABHD4 161.9285878 -0.229299205 0.244896902 -0.977551278
0.228296255 18.41940041
14144 AFF4 117.0258277 0.22171076 0.280428221 0.790586815
0.429185142 11.5289054
14145 ATF6 150.2186622 0.042600829 0.256722198 0.165924891
0.868208189 28.12094405
14146 BAZ2B 126.1211811 -0.117869881 0.281229826 -0.419122975 0.67512626
10.24071209
14147 GCA 164.8688116 -0.197882454 0.224280925 -0.844281292
0.298512182 11.80228059
14148 MCM2 140.9180026 -0.212412967 0.252894169 -0.840558755
0.400595172 21.45444111
14149 PNP 129.2786527 -0.069994646 0.264527772 -0.264592257
0.791222586 10.294547
14150 PRMT1 145.5152171 0.220247182 0.250924268 0.917559278
0.258849592 11.98418122
14151 RETREG2 111.4571727 0.052147552 0.211121505 0.167606146
0.866892125 22.90081189

14152	SNRNP40	122.9545258	0.177896717	0.269667221	0.659689807	0.509452909	19.4594974
14153	TMEM50B	144.0922045	-0.020609744	0.246860547	-0.122996094	0.901218268	15.47708947
14154	ABCE1	116.2261902	0.092555825	0.292055897	0.220225274	0.74871411	11.51471884
14155	ADD1	156.2010956	0.062784606	0.242928828	0.258427911	0.796068961	11.47097024
14156	AIP	128.0926206	-0.095212087	0.254222582	-0.274506222	0.708027646	11.94482479
14157	DCXR	141.2926008	0.272291057	0.251226282	1.082804677	0.278451411	14.10507454
14158	KLRG1	171.251546	-0.224128826	0.220258702	-1.407672418	0.159228092	18.72417224
14159	MESD	125.224282	0.240072025	0.257586529	1.220224408	0.186760105	21.78182112
14160	MPV17	162.2490077	-0.128646712	0.229707142	-0.526682855	0.591486701	14.44104909
14161	RAB5A	127.9418221	0.251182267	0.260660242	0.962628216	0.22522724	18.20802118
14162	RAP1B	125.6168524	0.192259798	0.266572212	0.725256165	0.468222558	12.19424974
14163	RIOK2	129.7199764	0.141522272	0.249621548	0.566991804	0.57071975	17.40547488
14164	TOP2B	115.4218696	0.241717618	0.281867516	1.212224158	0.225284492	19.79191804
14165	ATP2A2	164.5784659	-0.216825905	0.22212297	-1.264905446	0.17228277	19.45959771
14166	BRD8	129.4487822	-0.174594168	0.256944001	-0.6795028	0.496819224	21.41947444
14167	CFLAR	158.02079	-0.227624001	0.244928784	-0.970175891	0.221958826	15.07477911
14168	CHD9	150.6645091	-0.125927709	0.241259151	-0.562176116	0.572214965	15.47991501
14169	COG5	150.6952474	-0.290490988	0.252194105	-1.147205496	0.251255282	19.02481191
14170	MBTPS1	126.6244159	0.226220929	0.265012162	0.852662475	0.292292021	14.94211849
14171	SEPTIN11	152.0818488	-0.226285012	0.240978809	-0.980926927	0.226622846	14.14078718
14172	SRRM1	126.5269124	0.212242409	0.252497822	0.840971212	0.400264062	12.51051414
14173	ZC2H12	172.2252256	-0.221759649	0.229222826	-1.402626884	0.160427006	12.07441192
14174	AL125999.1	114.6464979	0.415155642	0.281868107	1.472872002	0.140785552	15.14724915
14175	COQ10B	121.672429	0.107580856	0.265514569	0.405178729	0.685246129	11.52979498
14176	KHNYN	152.6507929	-0.010605227	0.240620891	-0.044074464	0.964845047	11.51855571
14177	ADAM12	124.229249	0.277705445	0.258276555	1.07522514		

						0.282272942	19.41748255
14178	CPSF1	121.1048222	0.186410204	0.258508295	0.721099614	0.470848228	15.81242952
14179	IP6K2	127.2222217	0.215921665	0.251762158	0.857677774	0.291070416	15.54100117
14180	MARCHF7	112.0971429	-0.222108845	0.252216519	-0.661822926	0.508077654	19.4148481
14181	PLPBP	151.1118827	-0.067629106	0.242247	-0.279215454	0.780079485	19.21419898
14182	SMARCA5	127.9491211	-0.018266216	0.255150294	-0.071981922	0.942616294	17.11918789
14183	UBASH2B	140.0247885	0.216865272	0.272980481	0.791525482	0.428621579	17.28119101
14184	ATG12	115.5725812	0.427961877	0.291801299	1.46662027	0.142479256	18.81151448
14185	NDUFB4	157.1212608	0.2202596	0.261710276	0.84199795	0.299789101	21.08409741
14186	PNRC2	124.7279549	0.10227029	0.255962895	0.402847542	0.686224822	15.49947121
14187	RAB10	206.7952145	-0.202540224	0.250028007	-0.814069729	0.415605	14.54114095
14188	SELENOW	158.5812711	-0.126050085	0.226219006	-0.575705219	0.564814447	11.75441151
14189	TOR1A	141.2291592	0.045647226	0.250196957	0.182445207	0.855222251	18.54510411
14190	YIF1B	166.898012	-0.022225882	0.241061101	-0.092200206	0.926528974	19.27007045
14191	ARID4B	127.0715798	0.228675477	0.255491068	1.22558627	0.184976742	14.08491175
14192	CD2BP2	147.9462765	0.198854477	0.245195901	0.811002451	0.417264262	17.1427271
14193	DNAJA2	124.2245671	0.180471175	0.266122021	0.678149429	0.497676952	15.92854101
14194	HCFC1	147.2562622	0.025841064	0.244691489	0.146474504	0.882546816	14.27541975
14195	KPNB1	128.4564225	0.285974628	0.255972129	1.117205652	0.262906404	12.41211901
14196	MIDEAS	156.9201002	-0.201656217	0.228869648	-1.262848667	0.206642571	15.99111212
14197	MTF2	147.6209152	0.116980252	0.242757669	0.479904298	0.621295445	12.54940547
14198	RPA2	148.1629509	0.145289074	0.244169847	0.595442277	0.551547806	18.41040427
14199	TACC1	122.2096401	-0.157294255	0.28450841	-0.552862287	0.580257028	19.88707597
14200	TAF9	140.7752259	0.242999722	0.249197204	0.975129859	0.229495849	15.10014717
14201	USO1	97.21469287	0.218099221	0.261262562	0.880518694	0.278578282	15.45411959
14202	ATIC	146.280221	0.254299496	0.246995724	1.029570297	0.202211718	14.2178421

14203	CHMP2A	167.2519192	-0.192692957	0.224792222	-0.820699914	0.41181722	14.88082725
14204	DYNLT1	165.2905066	-0.274008062	0.241779852	-1.122295682	0.257090105	11.10274142
14205	MTPAP	119.6446718	0.286502519	0.275007765	1.405424022	0.159895228	11.41022011
14206	NDUFA6	157.1608985	0.202482061	0.246062802	0.822884282	0.410572744	10.40578817
14207	PIK2CD	110.7282475	0.296095107	0.206841117	0.964978584	0.224555528	19.41150085
14208	POLR1D	157.4862092	-0.024744062	0.242657406	-0.142592915	0.886610902	11.79014541
14209	POLR2G	144.6222881	0.109122959	0.245627702	0.444288208	0.656824152	21.24194285
14210	USP22	111.6624424	0.126191892	0.221926229	0.291989962	0.69506562	17.41711894
14211	AATF	125.8629	0.229211664	0.252226985	0.945010711	0.244652408	19.2445219
14212	CUTA	155.5266892	-0.222572458	0.254864952	-0.872295668	0.282501941	18.41185417
14213	FDPS	158.029585	0.019612987	0.242841122	0.080427567	0.92588925	14.14974142
14214	GTF2C6	125.889871	0.227482117	0.272568788	1.201469614	0.229569086	10.81494071
14215	IFI16	164.1758485	-0.202414058	0.221925809	-0.877065207	0.280451221	11.57445414
14216	MADD	121.1975515	0.129456722	0.285227225	0.488760525	0.625011248	14.11174421
14217	NXF1	144.2877824	0.26870042	0.251080222	1.468456099	0.141980272	17.88477445
14218	PGM1	160.7592002	-0.146221502	0.224022862	-0.624857221	0.522064765	19.99720404
14219	PMPCB	162.4740411	0.065608541	0.229274922	0.274082754	0.784021027	11.51199244
14220	TTC17	129.877504	0.249294024	0.262177225	0.951242129	0.241481471	11.49279024
14221	CACUL1	141.6269022	-0.152574252	0.262274047	-0.585225622	0.55822879	17.42197495
14222	CASP4	154.2057002	0.27147529	0.245140526	1.107427624	0.268109088	21.84111105
14223	DCTN1	149.2514908	0.045548294	0.242992019	0.187446924	0.851210224	10.82410198
14224	DERL1	167.277921	0.022296488	0.226721085	0.126855102	0.891145227	15.94444517
14225	DYNLRB1	140.8615814	-0.005596006	0.252252151	-0.022184176	0.98220104	15.91595924
14226	EXOC4	127.971901	0.081727222	0.279709664	0.292221659	0.770117152	12.45051894
14227	FAR2	150.4170828	0.129225206	0.242125228	0.52151745	0.595060252	14.14497715
14228	MRPS27	151.5114978	0.021170777	0.240264821	0.088114244		

0.929785796 19.99447598
14229 NDUFS8 145.1297522 0.059179414 0.245614925 0.240942884
0.809598609 17.50077497
14230 NUTF2 154.2212481 -0.022252918 0.241558092 -0.122924218
0.892454512 18.18142712
14231 TES 150.8072571 -0.100054759 0.246425121 -0.40602498
0.684724246 12.05401918
14232 ZMYND8 154.1622229 -0.121910525 0.24001674 -0.549588897
0.582601276 14.41118548
14233 ZNF146 114.2202795 0.195071497 0.204481826 0.640667108
0.521728989 15.24142988
14234 ADAR 152.1895226 -0.02024686 0.240208861 -0.084704868
0.922496025 15.55229271
14235 ASMTL 152.5041424 0.068254451 0.229400897 0.285105245
0.775562512 15.58704745
14236 ATF6B 166.528719 -0.214699294 0.226428161 -0.908095692
0.262827666 18.12819288
14237 ATP5MD 145.6222422 0.148424077 0.245602079 0.604227448
0.545625961 17.48284201
14238 BTBD10 148.0154085 -0.222775701 0.257075782 -0.866575989
0.286174274 11.57544472
14239 DDX21 144.9994217 0.25211127 0.24596652 1.024982465
0.205271462 17.9184024
14240 DNMT1 114.1616042 0.228294208 0.204774077 0.781871968 0.42428982
12.00219219
14241 FAM120B 147.7566542 -0.110242645 0.24715879 -0.44644425
0.655276202 21.2845445
14242 ITGB1 128.2061549 -0.056084292 0.271220017 -0.20670928
0.826226822 20.14874219
14243 JPT1 158.7644619 0.080002101 0.22829092 0.225727095 0.72706912
15.70774075
14244 MAP2K7 140.1182406 -0.092249241 0.258951851 -0.260102008
0.718770102 18.91905404
14245 NUP98 121.7795176 0.072264651 0.26604249 0.27162721
0.785908675 17.84140871
14246 PPP6C 129.8876895 -0.02669212 0.255426285 -0.104500247
0.916772272 18.28115202
14247 RABGGTB 122.8209682 0.245922247 0.27025172 1.28000054
0.200544946 18.84841191
14248 RBX1 154.8799442 -0.098025669 0.22820229 -0.411521056
0.680690505 12.19401175
14249 SAE1 145.6179162 -0.049495626 0.249842205 -0.198107587
0.842960889 14.51209419
14250 SNRPD1 127.2752948 0.242795258 0.252295851 1.258165582
0.174411149 29.01712954
14251 SNX4 168.2221022 -0.227445186 0.245628249 -0.925925168
0.254479674 18.42155141
14252 UBLCP1 172.852512 -0.254268582 0.229410706 -1.108255247
0.267708281 11.25141579
14253 VAV1 146.2905728 0.016072422 0.245488222 0.065471258 0.94779879
17.41457104

14254	YME1L1	125.5924518	0.202844206	0.25469285	0.796422654	0.425785824	21.70195144
14255	YPEL5	155.2122175	-0.019972942	0.227918889	-0.082948522	0.922097252	19.42279117
14256	ANKRD27	152.2725951	-0.116262221	0.244226265	-0.476228479	0.622904489	18.8251494
14257	ANP22B	151.4252917	0.060542527	0.244614092	0.247506292	0.804516422	10.1114419
14258	DCAF12	162.8658045	-0.260242952	0.222766596	-1.118472145	0.262265297	14.47744254
14259	IDH2G	160.0421711	0.06565125	0.229544679	0.274067242	0.784022947	11.04404778
14260	LEPROT	149.1022617	0.078097282	0.242120028	0.222556051	0.747021482	11.24181574
14261	LPIN1	125.8184211	0.165722151	0.255768912	0.647976122	0.517000288	11.08942594
14262	POR	160.2928459	0.112584098	0.24005078	0.469001178	0.629068794	25.04010192
14263	PRPF6	158.5470402	-0.080478616	0.226068177	-0.240912598	0.722169281	12.74219485
14264	RARS1	128.4610014	0.117616671	0.252689967	0.462622661	0.642917299	10.02112514
14265	ADI1	181.7970284	-0.080910278	0.220846524	-0.250492811	0.725968124	21.41487445
14266	CMA1	189.2912	-0.276720578	0.22229922	-1.22868128	0.215462517	14.81442079
14267	CNOT8	129.5051742	0.126598894	0.252064556	0.502247898	0.615492154	14.94401452
14268	HUWE1	174.2720096	-0.025557724	0.241042678	-0.147515689	0.882724998	19.71118742
14269	NAA20	125.9211921	0.2278168	0.257165288	0.885876946	0.27568284	17.59111184
14270	NDUFA10	140.490056	0.022244121	0.256292575	0.091048252	0.927454171	24.10909157
14271	NPC1	112.1462729	0.246789988	0.206282095	1.121887254	0.257681828	15.42480978
14272	PSEN1	154.2249422	-0.152272948	0.244422659	-0.622400978	0.522021055	12.20724214
14273	AHSA1	182.8724978	-0.215201641	0.224756406	-0.957488252	0.228220822	15.75011074
14274	AP001267.1	150.2200521	0.268726997	0.242978265	1.105970888	0.26872912	15.71251154
14275	GTPBP4	146.2517208	0.204982252	0.246078264	1.229274511	0.215206826	18.70148157
14276	HARS1	129.454982	0.284675202	0.254265057	1.119600172	0.262884182	11.19840451
14277	MACF1	127.8249592	0.06225644	0.280409402	0.222019802	0.824298468	41.11910147
14278	SNRPB	165.552026	0.194880277	0.240801152	0.809200016	0.418242597	45.51059718
14279	UBL4A	127.2590924	0.102129221	0.256946708	0.297511721		

						0.690990128	22.521429
14280	ATP5PF	150.2677512	0.190821112	0.245246254	0.778079622	0.426522075	57.04815044
14281	CNOT2	146.5249958	-0.02762701	0.248277218	-0.151552405	0.879529975	40.41512449
14282	COX7A2L	172.6844806	0.08442862	0.229084221	0.252175229	0.72295707	42.05147141
14283	FCGRT	158.1645228	0.100199082	0.229259202	0.418788828	0.675270462	12.1448914
14284	KCNAB2	174.9688642	-0.054587152	0.229098112	-0.22820441	0.819409596	29.11441019
14285	MIR1282	155.0517948	0.029460888	0.228858427	0.16520617	0.868781709	45.57992911
14286	PAK1	176.1955282	-0.255005558	0.225021625	-1.122249065	0.257109675	10.4194745
14287	SDR29U1	146.479125	0.042051285	0.251572441	0.171128781	0.864122506	50.42111424
14288	WBP2	198.0888281	-0.252816512	0.225208299	-1.127029518	0.259720001	25.21875144
14289	ACIN1	144.2144822	0.056101849	0.251126115	0.222401094	0.822222252	14.09524444
14290	AL592166.1	152.5625752	0.228922028	0.248711201	0.920427146	0.257244265	11.1824147
14291	COPS7A	165.8104462	-0.124924924	0.227950288	-0.525046145	0.599551112	17.88870747
14292	DPF2	146.2727875	0.222069801	0.246412202	1.211094981	0.189825676	19.4117871
14293	LRRFIP1	152.9180854	0.287127876	0.241998607	1.186526981	0.225414252	12.45897287
14294	NFKBIA	242.7681428	-0.249207917	0.252224644	-0.988296808	0.22295825	17.18804794
14295	PITRM1	121.0520242	0.100192499	0.272404696	0.267811204	0.712014014	18.25458277
14296	RER1	166.0442782	-0.126708158	0.222404695	-0.588222204	0.556275772	17.01941404
14297	SLC29A1	151.4487622	-0.101221512	0.244225885	-0.41422987	0.678622541	12.51159515
14298	TRA2B	168.8792015	0.125295119	0.225622025	0.52176092	0.594891586	11.19954714
14299	C9orf78	147.7962427	0.220160071	0.269290258	1.188461507	0.224651626	24.4524571
14300	ETF1	154.2255848	0.185795928	0.245821042	0.755787089	0.449776859	24.19711115
14301	EXOSC10	150.5100872	0.244711576	0.274226447	0.892227902	0.272211844	12.09919251
14302	GMFG	161.2866276	-0.04828252	0.222886424	-0.206862254	0.826116577	17.11197198
14303	KANSL2	129.8261515	0.248759224	0.252808922	1.27952689	0.16772928	14.52990705
14304	LONP2	152.4415591	0.022068971	0.240972179	0.122081622	0.89412882	18.18142405

14305 PTDSS1 199.7247229 -0.126455917 0.227692269 -0.574086475
0.565909285 24.21700874
14306 VPS26C 148.5472876 0.069845202 0.244779647 0.285229499
0.775284054 24.55144788
14307 CYFIP2 172.5611202 -0.247916276 0.226578188 -1.094175827
0.272877871 24.20182101
14308 EIF4E2 166.440966 -0.100055208 0.220969411 -0.422197225
0.664871495 18.10154471
14309 ELAC2 142.4207697 0.074822925 0.250921678 0.298184491
0.765562258 15.74981492
14310 FLNB 142.5087626 -0.259909647 0.275628267 -0.942927457
0.245712894 11.2908191
14311 GYG1 159.4811748 -0.104749662 0.242420057 -0.422081992
0.665681822 14.58242977
14312 NADSYN1 128.5056597 0.159121105 0.256264122 0.620965215
0.524622511 18.10225004
14313 SAFB 167.8922211 -0.072615575 0.222561842 -0.212242
0.754856615 15.41244721
14314 CAMLG 158.208288 -0.228897721 0.242126766 -0.982565225
0.225821467 15.22444799
14315 CHTF8 169.626712 -0.125266785 0.22886567 -0.591021246
0.554499418 19.11995874
14316 CMTM7 157.0971122 -0.052994606 0.228562478 -0.22622224
0.820942025 15.08404924
14317 ERLEC1 158.0092214 -0.014828485 0.228222954 -0.062259217
0.950256406 25.0945072
14318 GAPT 184.8498682 -0.119400169 0.229056401 -0.521269725
0.602178881 14.95041251
14319 MTRNR2L12 158.445506 0.004254025 0.225569902 0.018482986
0.98525255 14.02429105
14320 NAA15 152.4710472 0.165558645 0.242402426 0.682988027
0.494614408 15.51498911
14321 SP100 167.2645655 0.040501226 0.221297487 0.175104478 0.86099754
11.80421499
14322 STK28 146.7020146 -0.267792115 0.269527846 -0.992560107
0.220427074 18.90744251
14323 LARS1 128.5118812 0.241242706 0.269212059 1.26792774
0.204822757 14.0221894
14324 MAVS 182.8221047 -0.285266892 0.220821945 -1.292225562
0.196275579 10.20490049
14325 OGT 160.227692 -0.127567905 0.227690222 -0.526698161
0.591476127 18.8192775
14326 OTUB1 155.5884995 -0.026647225 0.242762755 -0.109766981
0.912594178 18.17104478
14327 RAB18 162.454679 -0.084541222 0.224994651 -0.259758072 0.71902806
17.85841111
14328 SDHA 151.4929242 0.126980994 0.24624647 0.51566625
0.606087521 17.91494081
14329 TADA2 181.6211422 0.216512972 0.251169821 0.86202224
0.288675279 25.402175
14330 TRMT112 172.5554976 -0.155821065 0.226691089 -0.687415926

						0.491820664	10.20142004
14331	TXNDC12	161.7189475	0.122684262	0.224725621	0.569524576	0.568992412	17.44945047
14332	YIPF2	198.8991196	-0.224870247	0.221226752	-1.061624452	0.288406208	18.85484554
14333	CNOT7	162.7574996	0.150242514	0.224029088	0.641982222	0.520884726	18.84215148
14334	ECHS1	152.0295297	0.18206998	0.246712587	0.742024446	0.458066466	11.94084114
14335	MFSD10	166.222249	0.287425068	0.241785284	1.188761627	0.224522482	15.47415211
14336	NECAP2	156.7292689	-0.092814542	0.246106844	-0.281194264	0.702059021	10.5771442
14337	COLGALT1	142.8265522	0.147677222	0.261265584	0.565021682	0.572059017	10.90472414
14338	COMT	164.8452002	-0.019860917	0.221197742	-0.08590446	0.921542266	20.27478058
14339	CYFIP1	156.0657652	-0.270781555	0.262718847	-1.405972908	0.15972217	21.44228445
14340	IVD	152.2665266	0.186281746	0.240498516	0.774980856	0.428250972	21.41821918
14341	NUDC	179.0557671	0.11624576	0.22209257	0.501290225	0.616166814	20.44120215
14342	RGS10	197.274657	-0.201677488	0.219712246	-1.272056608	0.169724749	14.77191108
14343	ZNFX1	142.5027825	0.114227459	0.252957672	0.451607011	0.651552118	15.47958445
14344	AP2M1	152.7920222	0.025722271	0.24091409	0.148278047	0.882122229	29.1552194
14345	CYB561A2	172.8848219	-0.069128757	0.226828054	-0.204762816	0.760546802	18.12127191
14346	EIF2B1	150.6911891	-0.029202742	0.249447079	-0.117474788	0.906482822	28.5171214
14347	GPS1	168.1274428	-0.1794761	0.220825215	-0.777507452	0.426859429	101.7707411
14348	OCIAD1	162.1072725	0.171879612	0.225669599	0.729224502	0.465802187	19.17942845
14349	RBM6	170.0596062	-0.184721127	0.22041945	-0.80167227	0.422741924	14.15014791
14350	RIC8A	168.2220527	-0.154722602	0.220272624	-0.671662282	0.501798718	11.01885124
14351	SLC48A1	177.0650585	-0.087084785	0.225809649	-0.28565572	0.699751652	17.44424722
14352	SRP72	152.1440901	0.152050412	0.240444705	0.626520599	0.524420642	14.18485714
14353	VTI1B	158.6720671	-0.017725116	0.22659714	-0.074916865	0.940280858	14.44142192
14354	COPE	165.2794722	-0.012225604	0.244291061	-0.050045227	0.96008624	19.80017557
14355	DNM2	146.2265822	0.186961122	0.247006804	0.756906768	0.44910572	11.71402445

14356	DYNC1H1	146.8102018	0.026692554	0.252222189	0.10541554	0.916046086	15.41490574
14357	HSD17B12	150.8497122	0.189651485	0.251522452	0.754011127	0.450842525	14.49011274
14358	LEPROTL1	165.4590582	0.125815207	0.221957122	0.585518586	0.558199072	24.71911917
14359	NAP1L4	172.7469452	-0.099674292	0.227684818	-0.42777212	0.661550758	20.27404524
14360	NDUFS5	161.2161821	0.079258529	0.22627909	0.225444522	0.727289779	15.45141052
14361	PSMC6	142.2560681	0.190226422	0.251277925	0.756724794	0.449208765	20.44714275
14362	RBM22	149.1966529	-0.010404621	0.252761746	-0.041001524	0.967294672	11.55009571
14363	AC125611.2	159.229205	0.142282491	0.225105468	0.609864554	0.541951525	12.57188711
14364	DCBLD2	152.9988991	0.192242478	0.242111497	0.794427606	0.426940719	19.20774181
14365	DDX24	149.927552	0.226999444	0.242850919	0.924727547	0.249928727	15.47440091
14366	GAA	148.2292057	0.220202507	0.242215242	1.215998015	0.188174709	21.74444191
14367	RBM8A	187.8078884	-0.021095055	0.224646701	-0.128417589	0.889910297	18.90487812
14368	RFX5	149.2175205	-0.157264575	0.2654142	-0.592525098	0.552499019	11.14840845
14369	UQCR10	162.6127287	0.127004291	0.227075891	0.525711542	0.592157925	20.27482044
14370	HSD2B7	164.0762278	-0.147055658	0.226990502	-0.620512871	0.524920182	15.19707214
14371	MCTP2	125.7127572	0.041277011	0.280515625	0.147146921	0.882016055	25.11749584
14372	PTK2B	151.2617198	-0.192189209	0.265782292	-0.726867117	0.467207262	48.92121097
14373	TMED4	184.7064496	-0.027554922	0.227216115	-0.165282822	0.868721271	12.22544511
14374	CLTC	160.1051702	-0.02417628	0.227927892	-0.142641755	0.885782267	18.17445181
14375	MAN2B2	172.027026	-0.120414289	0.220521885	-0.565710852	0.571590257	22.91588928
14376	PCBP1-AS1	190.4199126	0.051610227	0.228562028	0.225804511	0.821252468	21.48851502
14377	PLA2G4A	157.826607	-0.066592625	0.24296866	-0.274082187	0.784020694	19.15404219
14378	PNISR	157.6856911	0.22059928	0.226659129	0.974294147	0.229860874	11.41524581
14379	PSMB5	182.5622902	0.154221095	0.22052215	0.669048187	0.50246474	19.41182714
14380	SRSF4	174.727945	0.18210846	0.228768518	0.762698791	0.445642052	14.02228951
14381	TMEM9B	192.6982761	-0.246855658	0.218080904	-1.121945215		

0.257657426 14.11119127
14382 XPNPEP1 166.2790742 0.162486922 0.225027875 0.691251747
0.48924452 15.00712011
14383 CALCOCO2 170.2481174 -0.144209699 0.22077226 -0.624900222
0.522026547 20.41754451
14384 ELF1 182.5242275 -0.212622009 0.222447611 -1.405278128
0.159908864 19.14114755
14385 RUVBL1 164.0116224 0.056296246 0.240652799 0.222921815
0.815027912 18.90109194
14386 ZNF106 152.0109284 0.278902251 0.229724462 1.162428411
0.244655728 14.79901171
14387 CSNK2A2 162.522799 -0.022282028 0.227994122 -0.125646266
0.892100849 27.99519488
14388 FKBP15 150.9951974 0.022968024 0.259620864 0.092219267
0.926444201 41.71895057
14389 GLA 181.6882269 -0.19785521 0.221917095 -0.891572627
0.272622042 19.15891519
14390 LBH 180.0022814 0.020787602 0.224822272 0.092457815
0.926224206 18.48859119
14391 SCAF11 191.6681718 -0.222124249 0.222751521 -1.44412125
0.148704842 17.15497719
14392 SNHG5 182.5556259 0.152697686 0.229112787 0.670828521
0.502222298 11.0944141
14393 SUGT1 124.9278802 0.220916211 0.271050582 0.851920697
0.294252558 21.44457141
14394 ITM2C 296.9072789 -0.405526246 0.492527212 -0.821672924
0.411262072 21.28071857
14395 PDHB 176.2776872 0.197720297 0.222127489 0.848121224
0.296270402 21.27504788
14396 PPP1R12A 185.0974646 0.10442422 0.220248275 0.452528829
0.650167989 17.00514721
14397 SAP18 164.2567946 0.114519547 0.221822767 0.492996117
0.621208922 14.48710099
14398 UBE2I 181.626894 -0.209881788 0.226612191 -1.209661122
0.190210501 10.54048704
14399 UQCRFS1 152.8054929 0.250255294 0.229742828 1.461280082
0.142911164 17.41172441
14400 ARMCX2 162.0296641 -0.12128226 0.241548795 -0.50210708 0.6155922
11.08201175
14401 CCAR1 180.4808472 -0.179621296 0.222750977 -0.806422024
0.419999546 15.97121479
14402 CPNE1 158.9907042 0.077227255 0.227602811 0.225067821
0.745129725 12.24480441
14403 DCTN5 168.6611091 0.044826816 0.229216655 0.195522591
0.844982071 19.12911791
14404 HERPUD1 184.2987882 -0.212027086 0.229956962 -0.926278067
0.254249528 17.01788004
14405 IDH2B 162.5814518 0.171419876 0.222620216 0.726877087
0.461197099 14.78478491
14406 NDUFB2 174.0457222 0.195752615 0.22215154 0.842210492
0.299110721 18.10214991

14407 PRPF8 154.9460688 -0.004248426 0.247292512 -0.017172816
0.986298749 12.5280421
14408 TSN 185.9578904 -0.124706624 0.219571254 -0.612498168
0.529546994 19.8709141
14409 TUBGCP2 176.4940604 0.09558776 0.224082904 0.408250024
0.682016718 14.40770504
14410 AC018262.1 156.2802849 0.110127666 0.241122127 0.45676928
0.647826816 15.71117812
14411 CDC42SE2 189.846881 -0.206025758 0.218749124 -1.299026212
0.161805122 22.88912011
14412 HAVCR2 170.9798112 0.004721272 0.229456972 0.020575852
0.982584002 12.07084004
14413 KDM5A 170.5660592 -0.07255075 0.220285297 -0.215047251
0.752725778 21.12111145
14414 MPG 196.905609 -0.170467201 0.220689275 -0.772420486
0.429859502 14.71449571
14415 MRPL27 170.9466222 0.141149779 0.228056095 0.618925701
0.525965206 19.85114901
14416 PDCD6 167.0421644 0.186092016 0.220414292 0.807644924
0.419295012 17.42229144
14417 RAF1 170.6897681 -0.041221714 0.220286211 -0.178924219
0.857997127 14.15004411
14418 ADRM1 160.8420782 0.222994819 0.228622826 1.252572215
0.175872577 14.21481454
14419 GSS 175.2821998 0.024928564 0.224895591 0.110889519
0.911702957 17.41498574
14420 IAH1 192.5157645 -0.040187156 0.219594781 -0.182005971
0.854792222 14.71827824
14421 ITPR2 124.6151072 0.209924276 0.22227267 0.649402105
0.516077858 11.11110109
14422 NBEAL2 169.7869551 0.060098256 0.225602894 0.255081761
0.798659951 17.45815451
14423 NDUFV1 160.2870464 0.241654914 0.224207146 1.021259554
0.202272241 14.44182218
14424 PRPF40B 188.2242115 -0.176076454 0.225760482 -0.779925925
0.425424477 15.18414012
14425 RPA1 185.2822225 -0.196552529 0.220242491 -0.892026421
0.272272406 18.18948087
14426 MAP2K2 181.7019976 -0.197677722 0.222904998 -0.886824991 0.27517212
52.00425171
14427 SUSD6 158.2709198 -0.011611059 0.246299862 -0.047141962
0.962400082 41.45054172
14428 TOX4 162.0641807 0.182255992 0.222450829 0.780702986
0.424976616 14.02481488
14429 AL008726.1 158.9222474 0.161265149 0.229064049 0.674987099
0.499682961 41.14700091
14430 CD200C 151.4620627 0.14888007 0.248518297 0.599070619
0.549125792 28.40014124
14431 DKC1 174.6785895 0.250802754 0.229484178 1.092902161
0.274426759 28.1157947
14432 EMC7 155.048082 0.275472205 0.240784297 1.144065844

						0.252596221	47.44811752
14433	EPRS1	156.2998786	0.119225207	0.242507406	0.492047684	0.622685625	18.9228227
14434	NDUFB9	187.2540802	0.015068892	0.220595046	0.068210201	0.945528704	54.47054409
14435	PHB	176.6541229	0.15010865	0.225710221	0.665050267	0.506018242	24.44877104
14436	PLEKHO2	199.2890997	0.108699524	0.227201286	0.478217604	0.622495225	12.42958292
14437	SPN	209.7161952	0.262786797	0.248706275	1.458695796	0.144648854	17.42084144
14438	AD000864.1	167.4866542	-0.021109192	0.226080919	-0.089415072	0.928752046	40.17241814
14439	AL157871.4	184.8026284	-0.156072208	0.220620452	-0.707291959	0.479222922	17.1889118
14440	CIAO1	179.8081254	-0.056469625	0.224752829	-0.251251004	0.801620054	18.12990811
14441	DDX1	200.6249742	-0.097407044	0.216274562	-0.450177891	0.652582177	15.17448954
14442	LARP4B	161.2028766	0.071291965	0.228284925	0.299607549	0.764476525	15.50519048
14443	PCBP1	195.7425279	-0.108872291	0.217916266	-0.499610667	0.617249245	19.54290992
14444	PEF1	191.1222424	-0.065220028	0.22078745	-0.295297298	0.76769022	12.02980024
14445	POLR2E	175.9169542	0.294809012	0.222202602	1.269619759	0.204220108	11.07148019
14446	RRM2	162.2720015	0.220747952	0.225715054	1.260744452	0.172594461	14.27800527
14447	RTF2	190.426912	-0.092569581	0.218086249	-0.424462172	0.671228058	19.52104797
14448	UFM1	169.214926	0.086094092	0.240422494	0.258078612	0.720284484	15.11442882
14449	AC125457.1	184.5956591	-0.004228722	0.22076091	-0.019155262	0.984717247	18.55240159
14450	ARF4	187.2104217	-0.026162204	0.222845025	-0.16228006	0.871085217	15.04925894
14451	FLOT2	199.1621746	-0.272788954	0.212752741	-1.286888024	0.19812222	20.14871244
14452	SERPINB6	190.2606729	-0.196227195	0.220151262	-0.891272977	0.272728574	14.58507117
14453	TCF25	178.4488949	-0.042576626	0.222226108	-0.190647819	0.848801522	14.45954978
14454	API5	140.746881	0.02025099	0.288720665	0.070484229	0.942808165	21.17407075
14455	MFNG	212.5026156	-0.021542659	0.221197715	-0.097290964	0.922415921	14.70948154
14456	NCF4	192.6102682	-0.062026522	0.220592128	-0.285760522	0.775061546	17.51014981
14457	SNRPD2	200.4514755	-0.145714141	0.215765809	-0.675224715	0.499462122	15.1210094

14458	TMED2	172.1618092	0.078245186	0.227282276	0.244264202	0.720647602	21.11171195
14459	UQCRQ	225.5418628	-0.187729016	0.218001869	-0.861124889	0.289162756	24.94520112
14460	ARAP1	212.4219764	-0.252987264	0.211094597	-1.198454948	0.220729952	15.21955471
14461	MRPS16	162.7297191	0.122509519	0.224897227	0.568272916	0.569781098	14.94485142
14462	NUMA1	155.2897198	0.242747066	0.242485199	0.996968469	0.218779815	18.51514927
14463	PHIP	186.7289685	-0.012145429	0.221780558	-0.05927228	0.95272524	27.8774078
14464	RSRP1	150.9161412	0.266822869	0.254155659	1.049879706	0.292772422	17.19274741
14465	ACP1	191.7081492	0.198772942	0.224299115	0.885805279	0.275722411	10.79274975
14466	BUB2	158.6611507	0.258128621	0.228221259	1.082566224	0.278557169	14.01229084
14467	EIF4EBP2	197.2148242	-0.202692225	0.219025689	-0.925421285	0.254741612	11.14095721
14468	MTRNR2L1	202.4161658	0.106899797	0.225995542	0.47201728	0.626200851	14.01754757
14469	RNF7	209.1476259	-0.2525916	0.209804874	-1.202925807	0.228614291	20.21455104
14470	RPS26	169.0767246	0.072192026	0.222142164	0.209651097	0.756826295	15.51442194
14471	ATP6V0A1	180.5981679	-0.244548212	0.222844059	-1.050266491	0.292595607	11.18052997
14472	CASP1	182.828822	0.150028627	0.221788184	0.676495124	0.498726224	19.85598512
14473	MAP4	178.426252	-0.016882275	0.224222128	-0.072047291	0.942564272	11.09490401
14474	PIGS	188.4245554	0.098760418	0.21978212	0.449256016	0.652174855	51.54442414
14475	SIGLEC8	188.0296544	-0.202620666	0.22270597	-0.909857257	0.262897741	15.78115714
14476	TOR1AIP1	165.8029868	0.201051928	0.221777247	0.867426001	0.285702164	21.25220547
14477	ACTR10	189.9810184	-0.228499555	0.222547542	-1.066885154	0.286022699	20.14474481
14478	ARHGEF2	174.8124297	0.050020222	0.226402969	0.22097869	0.825109026	11.09411175
14479	COMMD6	189.0024021	-0.048041562	0.218242097	-0.220129682	0.825770159	15.00110812
14480	PWP1	144.4021769	0.298846709	0.261824545	1.141400665	0.252702228	11.17411808
14481	RBM5	194.4142822	-0.156660012	0.214915066	-0.728929184	0.466028865	12.41819148
14482	SLC40A1	176.5954158	-0.292191702	0.246524672	-1.185242242	0.225921287	14.1144448
14483	SNRNP200	166.6928662	0.071591527	0.224644864	0.205105876		

0.760285516 18.92591578
14484 CHD4 170.8225056 -0.04221098 0.224869046 -0.179721241
0.857271228 15.80075424
14485 RDH11 167.2522042 0.200254527 0.221866541 0.862662924
0.287772081 27.74184182
14486 SERP1 282.6429169 -0.142969247 0.452512758 -0.217452796
0.750899292 15.94921818
14487 SNX27 178.7724041 0.114297094 0.224271249 0.50985607 0.6101522
21.47444872
14488 TMED9 154.0808952 0.078242004 0.280979524 0.278465144
0.780655218 14.29705474
14489 TOMM7 176.7068244 0.10915797 0.226579652 0.481764222
0.629972448 24.84417489
14490 USP15 164.2844772 0.274946619 0.228540282 1.15262085
0.249066042 17.41412118
14491 CNP 212.7281289 -0.129111215 0.211508909 -0.65770901
0.510725121 19.41154874
14492 ETFA 168.9082517 0.187922255 0.225045196 0.799562621
0.422964246 52.08220145
14493 MAPKAPK2 204.7229141 -0.046279796 0.215281282 -0.214872806
0.829865708 20.8408145
14494 CCND2 202.2296081 -0.241209966 0.212914247 -1.122266298
0.257060419 29.14485187
14495 GIT2 186.9111119 -0.100687005 0.222687798 -0.45214424
0.651165074 12.57142572
14496 KPNA6 190.1064114 0.157059662 0.221951479 0.707620616
0.479174675 11.41017788
14497 MDM4 180.2741964 -0.17526202 0.220527984 -0.760664269
0.446857562 14.24198594
14498 SLC5A2 155.2022921 -0.009178045 0.264802906 -0.024659778
0.972251024 12.42412994
14499 ST12 177.8715792 0.022684788 0.226696191 0.144178814 0.88525927
21.24722101
14500 UBXN1 182.5991661 -0.12022122 0.225090658 -0.579016611
0.562577965 19.12901471
14501 BRD2 162.4820424 0.290506195 0.224980825 1.22629745
0.216248019 18.87090204
14502 DCTN4 154.7768722 0.291281211 0.246022261 1.182909818
0.226448762 18.1011211
14503 MYADM 205.0986151 0.102771275 0.21894768 0.472954667
0.625522225 14.09451819
14504 RTCB 198.6725522 -0.022908421 0.212816222 -0.152909884
0.877680791 17.41401178
14505 TMCO1 182.1277127 -0.059769951 0.222560094 -0.267255189
0.789195698 27.17757414
14506 TXN2 195.0591277 0.042227605 0.215722789 0.195749292
0.844806222 17.97415159
14507 B4GALT1 176.926585 -0.02299288 0.22987749 -0.147878245
0.882428858 15.55871182
14508 HINT1 172.9857162 0.215720742 0.222819026 1.256120827
0.175060727 21.51470847

14509	HSBP1	169.1222289	0.295525285	0.229090141	1.290029722	0.197026862	12.4444922
14510	KARS1	189.1912145	0.125441828	0.218777744	0.57227568	0.566290252	20.07524488
14511	POLDIP2	178.141872	0.150027862	0.222426217	0.671457227	0.501929299	22.15845489
14512	PSMB4	202.7526051	-0.089217627	0.216142982	-0.412771279	0.679774122	15.04085191
14513	RNPS1	194.2027697	0.092726522	0.217178948	0.427005116	0.669275602	24.20571424
14514	SEMA4A	181.5695625	0.029142602	0.222747187	0.120252272	0.896266761	14.74115114
14515	TXNL1	185.6194229	0.081768218	0.222072991	0.266552452	0.71295288	15.21124
14516	CPSF6	161.0622086	0.084979766	0.264201728	0.221647265	0.747719922	17.70287274
14517	EDF1	202.0485122	-0.122751146	0.210822112	-0.5822471	0.560400252	14.7250505
14518	EMG1	184.7772955	-0.045487052	0.224285501	-0.202808709	0.82928455	10.11549188
14519	RNF4	198.6422085	0.164472666	0.218228686	0.752626619	0.451067449	20.95444541
14520	TMEM22	202.1018242	-0.222129577	0.214282728	-1.040846754	0.297946677	28.14554141
14521	HCST	192.6257628	0.064220727	0.215871529	0.297958405	0.76572491	11.07510477
14522	AL121985.1	188.9209101	-0.207220272	0.224288442	-0.922979722	0.255496868	19.28284454
14523	CERS2	198.0106222	-0.168810222	0.219291509	-0.769798264	0.441419569	14.84481104
14524	CLTA	265.9169111	-0.259226225	0.228290226	-1.125511601	0.256161024	11.14504887
14525	CYB5R4	162.0292867	0.156285629	0.242062628	0.642982289	0.520225527	10.24891441
14526	EIF4G1	192.2092221	-0.086718455	0.216204492	-0.401094601	0.688250476	21.14981411
14527	GALNT6	214.5678111	-0.069009662	0.214586216	-0.221592952	0.747760225	27.52100811
14528	UBE2N	189.9029522	0.220128052	0.244189666	0.942456152	0.24595915	15.94275741
14529	CLNS1A	182.7592726	-0.155621166	0.228498217	-0.652504252	0.51407592	24.55711715
14530	ERP29	200.7579601	0.028756407	0.212971278	0.181979417	0.855598879	10.0920101
14531	RO60	171.2972601	-0.042079028	0.248762002	-0.172172666	0.862514922	22.45857797
14532	SOX4	187.9997486	-0.01706821	0.22205524	-0.076520102	0.929005221	11.51744194
14533	HNRNPH2	209.5187898	-0.055926582	0.20972472	-0.266666609	0.789725865	18.10490179
14534	SRSF10	184.7247612	0.19157472	0.222749092	0.860047182		

0.289762024	21.2149941
14535	ESD	211.1712522	0.021814918	0.211497708	0.150426776	0.880427917	12.52784188
14536	HNRNPH2	206.2520561	-0.242225422	0.215217412	-1.120124262	0.258422868	10.07221184
14537	PSMB7	182.5591171	0.129587069	0.220790827	0.622214077	0.527247	14.25741119
14538	SERPINB9	182.290188	-0.174109007	0.225699292	-0.728690944	0.460094686	18.48407041
14539	SSBP1	179.1425602	0.262872442	0.226444074	1.160875789	0.245692416	11.4072914
14540	AC008894.2	189.5518285	0.212758221	0.222668577	1.298211	0.162019696	17.14414741
14541	PAG1	197.5282809	0.050692477	0.2226827	0.227648926	0.819919181	19.41144474
14542	UFC1	202.2091204	0.067582154	0.212292672	0.216701772	0.751469902	21.15180144
14543	MLX	187.8145029	0.156089551	0.218022969	0.715928298	0.474025562	25.47275712
14544	NRDC	182.7222121	-0.162664214	0.224755072	-0.692910752	0.488265561	19.14520228
14545	OSTC	220.2671291	-0.022820179	0.209258779	-0.109000242	0.912202218	21.14492419
14546	TMEM176A	224.7426818	-0.222415947	0.208969117	-1.064248407	0.287170897	19.15527544
14547	AC092069.1	198.2266054	-0.084658984	0.222781279	-0.27821112	0.705199485	81.27018197
14548	APRT	192.2905279	0.158568254	0.219496629	0.722417689	0.470027715	79.42115711
14549	C4orf2	208.7590794	0.017417582	0.211447702	0.082272009	0.924250099	48.25411158
14550	GGCX	169.8268112	0.051927424	0.246021957	0.211100259	0.82280896	58.55919721
14551	LRRC41	207.5872699	-0.127224212	0.215597694	-0.590564249	0.554812258	54.01799184
14552	PNN	179.9257407	0.241426686	0.222697889	1.079298007	0.280454901	19.71044401
14553	SS18	182.2262401	-0.044008519	0.228182516	-0.192864587	0.84706502	14.49909227
14554	ARIH2	176.2584925	0.241074224	0.2262426	1.065556768	0.286624051	12.50449004
14555	DDX6	224.9940279	-0.212271679	0.209446142	-1.012967962	0.210598029	18.10745498
14556	DR1	212.2726541	0.042657226	0.209922491	0.202194002	0.8289824	21.845495
14557	IMMT	196.6042818	0.256228022	0.217029011	1.18106892	0.227575242	15.19404499
14558	SUPT16H	172.8175729	0.009772122	0.240081242	0.040702402	0.967522249	18.22944129
14559	ATRAID	189.6404626	0.096042256	0.219970554	0.426614604	0.66229087	18.45784404

14560	COX6B1	202.7465672	-0.024112457	0.229049684	-0.105271722	0.916160192	40.11948254
14561	FBXL5	182.2412059	0.042672245	0.226609788	0.188207597	0.85062552	10.08455205
14562	FUBP1	186.6202527	-0.010589742	0.222942828	-0.047287491	0.962284097	11.4244022
14563	IK	181.2744124	0.159758429	0.242094712	0.65718599	0.511061222	11.48781407
14564	MAGED2	258.8491728	-0.192541827	0.21001901	-0.916782898	0.259256416	20.4828708
14565	PSME2	229.0740782	0.195625726	0.222204962	0.880429149	0.278626871	18.00151845
14566	TSG101	209.7411227	0.010845028	0.210272728	0.051551492	0.958886071	18.09471249
14567	UBXN4	155.4171755	0.166720529	0.268074027	0.621957042	0.522970115	17.44441009
14568	ARFGAP2	221.6085028	-0.187626448	0.208645854	-0.899205902	0.268489744	18.51091128
14569	ATP8B4	186.8104128	-0.224485422	0.248875751	-1.202804927	0.192200099	18.44551871
14570	AGBL5	227.5069652	-0.272702622	0.202102172	-1.254278476	0.175647551	24.44891478
14571	IVNS1ABP	192.6586215	-0.085228611	0.222127274	-0.282692688	0.701206222	24.81415911
14572	KIF5B	212.4527242	0.224615652	0.219624652	1.022725122	0.206427825	27.55904014
14573	SFT2D1	182.2502402	0.151522481	0.22254625	0.680862574	0.495958451	19.27194904
14574	ADIPOR1	227.1751294	-0.177872001	0.202518827	-0.87829855	0.279781722	14.01011204
14575	AP1M1	184.6779292	-0.081676061	0.222540482	-0.25122272	0.725412007	11.05745141
14576	CDS2	212.6982059	-0.050196179	0.208872005	-0.240220279	0.810081975	14.51155142
14577	DCAF7	196.4762915	0.110022184	0.222140285	0.492062627	0.621968227	19.04017184
14578	FYTTD1	202.0672454	-0.079502755	0.212888667	-0.27244757	0.70881526	15.2111288
14579	ABI1	207.2592926	-0.088291596	0.209011228	-0.422902557	0.672265627	11.0514814
14580	CNOT9	194.2208852	-0.129548846	0.226294196	-0.616670018	0.527452401	25.21427094
14581	COPS6	204.0126216	-0.127920649	0.212411869	-0.599454224	0.548870021	28.78044298
14582	DNAJC7	199.9755092	0.290229622	0.21747487	1.225002124	0.181875221	24.41482419
14583	LPCAT2	222.8292158	-0.282721028	0.202899062	-1.291477847	0.164080572	25.24284555
14584	NOP10	188.2849802	0.154426121	0.227401794	0.679089224	0.497081266	21.49292214
14585	PPP2CA	225.2679675	-0.212582595	0.1999929	-1.062950298		

0.287804446 25.84128004
14586 DAPK1 192.7224259 -0.19946042 0.228009229 -0.828026528
0.402010182 14.48044174
14587 DDX2X 147.0749611 0.242421888 0.292167492 0.826905759
0.408290479 24.1478797
14588 ELMO2 170.6460422 0.217422612 0.229042126 0.909552922
0.262057808 19.45417125
14589 CCNDBP1 221.8702882 -0.022109869 0.205502068 -0.107589521
0.914221209 19.14515115
14590 LAMTOR5 207.8609222 0.100085066 0.210287226 0.475718258
0.624275041 17.5102188
14591 PSMD11 217.7064288 0.204792109 0.219121762 0.924604156
0.249992226 17.44449751
14592 PSTPIP2 222.4121222 -0.272657724 0.201606098 -1.252427962
0.176228449 15.22424547
14593 SEC12 225.8605564 -0.126011705 0.202282424 -0.622628426
0.522522147 19.14474841
14594 TFG 255.6798807 -0.124791129 0.207090296 -0.602592597
0.546779746 21.42114242
14595 VPS29 217.7019012 -0.174650642 0.205720867 -0.848927752
0.295921495 19.45417125
14596 BECN1 210.0272277 0.027201785 0.212721709 0.128229047
0.897880666 45.14521429
14597 CIRBP 222.0475294 0.162282624 0.214666076 0.755981695
0.449660171 11.04101894
14598 SEC61B 195.1872796 0.167725188 0.21589285 0.776890889
0.427222147 25.09092458
14599 EIF2A 195.2845511 0.142201877 0.219929944 0.651124967
0.514965821 29.77285185
14600 FCN1 229.5828127 -0.182982298 0.202060829 -0.905585167
0.265155462 14.91227055
14601 TMEM62A 222.4425127 -0.227285876 0.199802222 -1.128054722
0.255097629 44.90549804
14602 UBA2 197.112872 0.256250046 0.212964225 1.197629774 0.22106118
28.81189404
14603 ATP6V1C2 204.4188125 0.207198817 0.211014072 0.981919427
0.226129544 12.5547117
14604 CYTH4 222.5987526 -0.117202667 0.202202946 -0.579244441
0.562256785 14.11014499
14605 DEGS1 220.4570729 -0.072069254 0.204609525 -0.257115587
0.721005276 17.48081419
14606 DNAJB1 228.6622426 -0.286176699 0.201182902 -1.422470276
0.154889774 14.04494098
14607 RHBDD2 246.4110812 -0.2442588 0.196296687 -1.244224807
0.212276266 11.25290748
14608 TBL1XR1 181.2252698 0.224229847 0.226625092 1.421066221
0.152411208 15.9255021
14609 USP4 191.6744272 0.042757286 0.221200282 0.192209811
0.846794656 10.20401192
14610 VDAC2 222.612282 0.042268649 0.206840902 0.209188071
0.824201424 24.05222287

14611	ARMC8	187.7922298	0.041959621	0.227657684	0.184210189	0.852770121	24.02142284
14612	EIF2G	212.1015992	0.212608181	0.216427989	1.444298122	0.148627019	12.81079178
14613	ATF4	212.9894824	-0.029618984	0.207269917	-0.191146811	0.848410574	10.57987927
14614	ATP6V1E1	216.5760417	-0.072182779	0.205587504	-0.255969002	0.721862779	27.12519205
14615	AZIN1	187.0227914	-0.021715272	0.228702421	-0.122865728	0.894299564	10.91411899
14616	FUCA2	209.8892866	0.077915892	0.210242162	0.270424508	0.711066216	19.25170471
14617	PSMB8	222.0251246	0.094815949	0.207180217	0.457649406	0.647204242	17.8277491
14618	PSMD7	218.528646	0.159819805	0.208242205	0.767470402	0.442801884	11.52949228
14619	TAF7	227.1947692	-0.121762249	0.202540822	-0.598225692	0.54968925	11.75401779
14620	CD4	180.0180245	0.222067146	0.225017527	1.274651286	0.16922958	17.28487194
14621	IMPDH2	211.2962252	0.085401772	0.215505579	0.296285672	0.691894296	14.71811401
14622	TTN	164.965992	0.207811428	0.251212265	1.224816126	0.220644508	14.74215171
14623	VTA1	201.7620121	0.291648402	0.21212267	1.268446789	0.171172267	41.44004429
14624	MAPK6	122.4190922	0.12111062	0.841929429	0.155726278	0.876248704	21.18448017
14625	SF2A2	200.6822625	0.077618614	0.212982295	0.262722814	0.716802728	18.75872471
14626	TMX1	170.7609802	0.284506976	0.244928524	1.161591796	0.245401216	21.12524422
14627	CYB5R2	258.0287117	-0.217047609	0.195920798	-1.107822427	0.267922761	11.59515875
14628	GNAS	226.7980261	-0.046499474	0.204292422	-0.227612216	0.819947644	22.9541407
14629	HERC1	185.5689914	-0.062405015	0.24529225	-0.258486587	0.796021299	19.07410205
14630	PPA1	206.1258847	0.012920124	0.212442824	0.060578865	0.951694606	12.97954585
14631	RPL18A	221.4529024	-0.005222679	0.221902052	-0.022585422	0.981182292	11.77581514
14632	TINF2	225.8990974	-0.241146854	0.202992725	-1.187958112	0.224849912	18.17184428
14633	ELOVL1	256.6164007	-0.044428412	0.208006172	-0.212591802	0.820865292	18.84871978
14634	HGS	229.0182849	0.086090268	0.209572821	0.41078928	0.681227055	17.72494222
14635	RPL21	212.6462092	0.080248694	0.207058892	0.288047529	0.697980854	10.08174777
14636	RSU1	224.4786258	-0.075289447	0.202154109	-0.272425896		

0.709568222 24.4784854
14637 TTYH2 222.6972882 0.219088624 0.209180675 1.04726556 0.29492101
20.48079809
14638 DNAJC2 217.8282286 0.020207261 0.204270227 0.147807055
0.882495042 14.99175108
14639 NDUFS2 222.9064414 -0.127068279 0.199057402 -0.628249921
0.522245916 18.22881418
14640 PYGL 178.7870229 0.122499576 0.242251151 0.548812748
0.582122274 19.95447051
14641 SARS1 222.0781297 0.051022025 0.204725126 0.249262702
0.802156807 20.95944981
14642 ATRX 195.7242242 0.241522924 0.219607266 1.099829807
0.271401925 27.12148177
14643 HDAC1 210.6144065 -0.116892261 0.212144762 -0.5484177
0.582405126 25.48991111
14644 ALOX5 245.4426962 -0.197208879 0.200189086 -0.985612567
0.224222269 17.52718081
14645 CSNK1A1 226.6211268 -0.140201829 0.201662204 -0.695227272
0.486912884 24.91514404
14646 PFKL 226.0958202 -0.192008165 0.207124744 -0.92701702
0.252917692 24.95270598
14647 RBBP7 246.1712626 -0.188172268 0.195572496 -0.962161908
0.225968281 20.49112948
14648 VPS28 248.9742974 -0.247577189 0.194412092 -1.272466004
0.202852729 11.04414109
14649 KRT1 206.8147742 0.221792191 0.615252228 0.52202297
0.600957572 10.2547488
14650 LNPEP 167.6556042 0.11847642 0.272862282 0.422612011
0.665295925 18.14198911
14651 TANC1 224.9762827 -0.100519667 0.20288851 -0.495442876
0.620287526 17.51181174
14652 KEAP1 212.9120125 -0.020857222 0.20818405 -0.148220921
0.882168422 14.89581041
14653 PSMC2 224.5154299 0.149528562 0.209206909 0.714787882
0.474740124 24.77447592
14654 TOMM20 185.1049426 0.12196226 0.224625951 0.562427188
0.572818188 21.85410117
14655 CAT 250.6418204 0.058894222 0.206244004 0.285417707
0.775224142 24.94409719
14656 PSMD4 215.1125797 0.121626902 0.207182429 0.587052202
0.557168647 15.22248004
14657 HEXA 210.2410298 0.219122776 0.208242165 1.052202618
0.292660721 11.11111274
14658 NUMB 184.9811815 0.157978227 0.225952986 0.669522112
0.502155466 19.94217447
14659 RPS27L 202.8092574 0.09627286 0.21445187 0.448925254
0.652485578 15.5417988
14660 TMEM122 202.7062872 0.0915282 0.215719595 0.424292469
0.671252521 44.21004112
14661 USP11 252.2216495 -0.202275168 0.192550152 -1.045078829
0.295986522 20.41179255

14662	COX7B	194.2025506	0.212826028	0.220427166	0.965517925	0.224285457	19.84741795
14663	MOB2A	221.704075	0.200169175	0.210211165	1.427941162	0.152208794	15.7188184
14664	PLP2	221.4199051	0.17261246	0.209279549	0.829175827	0.407004921	17.4494818
14665	RPS2	222.2582846	0.042829481	0.201909589	0.212171605	0.821972158	18.82974212
14666	THRAP2	247.2046662	-0.270992941	0.194259259	-1.294292242	0.162229071	28.9884814
14667	UBL5	204.265824	0.224882557	0.210555421	1.068049227	0.285498202	15.1000427
14668	AUP1	229.8556689	-0.068467275	0.199472872	-0.242229817	0.721418052	15.14224971
14669	LAMP2	178.6029142	0.222485684	0.226911042	1.402419952	0.160491647	17.48918017
14670	LIMS1	220.4444128	-0.116002079	0.202515849	-0.572804942	0.566776772	12.42418881
14671	SNU12	212.4129527	0.122046528	0.211859665	0.62227261	0.522104727	18.58718144
14672	ACO2	218.1272084	0.052221245	0.204526084	0.260205164	0.794705521	14.78548517
14673	TSPYL1	241.2600175	-0.150247665	0.197782705	-0.759656427	0.447459975	15.78727881
14674	CNPY2	227.6572264	-0.115199977	0.197116902	-0.584424652	0.558924645	41.18241024
14675	CTNNB1	195.6166666	0.170265677	0.222425415	0.765495602	0.442976484	11.9411441
14676	FOXP1	220.2784228	-0.076072024	0.202945852	-0.272001084	0.709147627	17.11151178
14677	SAR1A	222.2298658	-0.082609976	0.19849914	-0.416172969	0.677282428	49.48877509
14678	STX2	222.0757407	-0.066777581	0.198118809	-0.227058259	0.726072982	52.22418191
14679	JUP	268.0628528	-0.176111527	0.196121012	-0.897928052	0.269222908	41.90970222
14680	PPM1G	206.5225968	0.211297729	0.218890711	0.96521154	0.224288792	11.90190407
14681	STK10	205.8412922	0.242019297	0.217648902	1.116565685	0.26418007	29.12891971
14682	TRIB2	225.4194604	-0.224119495	0.200140179	-1.1198126	0.262792629	10.10581108
14683	RTRAF	215.8155664	0.122202252	0.205456815	0.642460526	0.519925214	15.81029929
14684	VAMP2	226.909176	-0.028261882	0.201902297	-0.140472605	0.888286596	17.44928141
14685	AC245140.2	248.0224225	0.119646218	0.202270906	0.588215212	0.55622067	21.41150247
14686	MRPS6	255.8162278	-0.101664122	0.205448098	-0.494840956	0.620712291	11.0948518
14687	SNX6	204.6896682	0.025050824	0.222024745	0.157154142		

0.875122268	10.18414442
14688	SRP9	227.1072917	0.221267116	0.202095897	1.129201229
0.254619191	18.44142112
14689	USP10	222.2214024	0.104070157	0.209881242	0.495852586
0.619998421	19.27140088
14690	ZFAND6 242.2224502	-0.059526274	0.194841765	-0.205511266
0.759976715	17.11182174
14691	NT5C2	200.7220297	0.202994487	0.215969242	1.402951282
0.160621227	20.51041887
14692	PFKP	251.2089152	0.006560799	0.194717568	0.022692922
0.972121225	10.12125128
14693	PRMT2	264.2265766	-0.081172227	0.192002661	-0.420580921
0.674061128	17.4494242
14694	SCAMP2 262.9549894	-0.1110816	0.191885456	-0.578895258
0.562659782	11.84891997
14695	VAMP7	200.7642084	0.295275168	0.218768875	1.249712879	0.1771081
18.44884205
14696	APEX1	282.2758544	-0.025182866	0.205482656	-0.171220612	0.8640502
21.89552847
14697	FMNL2	251.7218921	-0.125404699	0.19656525	-0.627980004
0.522486696	22.41195248
14698	HPS1	222.2441781	0.141940194	0.198906152	0.71260284
0.475472182	14.17418117
14699	DOCK2	221.0480579	-0.204264611	0.208648607	-0.979467895
0.227248844	24.01451225
14700	NABP1	215.0499207	0.224882829	0.209847058	1.071651129
0.282876755	11.49417495
14701	APEH	247.0986121	-0.220420495	0.196682292	-1.120727715
0.262299522	17.21224027
14702	C5orf22	212.5545927	-0.127416979	0.228270691	-0.558182701
0.57671895	21.82244141
14703	HMGB1	228.5845089	0.255860922	0.201059115	1.272565695
0.202172198	11.80014058
14704	MTCH2	224.5652915	0.221584108	0.201461569	1.099882767
0.271282204	11.14247592
14705	PIP4K2A	279.7265882	-0.128080618	0.191172284	-0.722282666
0.470120092	22.8111787
14706	SFT2D2 214.2240018	0.119174862	0.220655725	0.540094105
0.589122126	19.84145929
14707	UBAP2L 218.9890162	0.114661826	0.219702928	0.521892494
0.601745185	19.91844221
14708	COX6C	248.7246621	0.058712671	0.195689057	0.200020425
0.764152948	11.19242828
14709	CYBC1	244.9940964	0.015144281	0.195028921	0.077651462
0.928105205	10.10414911
14710	HCK	224.7528645	0.082119629	0.207222052	0.400901011
0.688492006	14.11200114
14711	SEC24C 200.8479668	0.292658192	0.21906004	1.240527472	0.18007067
19.70122558
14712	SLC25A29	229.4465701	-0.020522285	0.199881122	-0.102672954
0.91822254	18.11804178

14713	COX5B	262.1248927	0.057222522	0.194957515	0.294025762	0.768728218	19.24701547
14714	RGS19	248.2502757	0.012224424	0.192291077	0.062295227	0.949521229	24.1421845
14715	SHKBP1	241.5258629	0.164852167	0.196865511	0.827284697	0.402276265	14.20222174
14716	TXLNA	268.7045507	-0.214058024	0.18674712	-1.146245272	0.251692629	21.50182417
14717	YTHDF2	222.6752614	0.188220952	0.204785256	0.919650492	0.257755422	18.41491901
14718	KDELR2	251.7677989	-0.074250718	0.192410222	-0.284419597	0.700667478	19.41297757
14719	PPP1R15A	229.0121724	-0.209101982	0.2054581	-1.017725409	0.208802715	18.92181798
14720	RAB6A	266.7068781	-0.11212249	0.189224028	-0.592278177	0.552664227	27.21412112
14721	PRPF40A	222.7800498	0.118560499	0.202881622	0.581516259	0.560892496	11.22971947
14722	SBNO1	224.7024126	0.186220206	0.198066805	0.94022926	0.247094796	18.21294195
14723	ACTR1A	250.9910825	0.026214125	0.20465119	0.128580268	0.897689701	24.11818277
14724	CREB2L2	262.7461045	-0.212972879	0.188716708	-1.122821124	0.256865288	21.14092581
14725	ACTR2	222.5052216	-0.218298266	0.220587625	-0.946704562	0.242789245	11.40154957
14726	NAGA	267.0156691	-0.196858724	0.186848296	-1.052574654	0.292077698	24.11952472
14727	ILK	285.5147518	-0.208920624	0.184712262	-1.121107871	0.258009692	14.00041472
14728	PSMC5	267.066504	-0.127604695	0.18729587	-0.72429951	0.462766217	18.18582979
14729	PUF60	240.1289129	0.228192647	0.205248872	1.160511257	0.245840672	14.07158054
14730	SWAP70	244.8904729	0.165057842	0.19750056	0.825722525	0.402204825	18.47480894
14731	MRFAP1	221.2512019	-0.21000984	0.202956012	-1.024755451	0.200782122	22.09141589
14732	SNX1	226.284169	0.005012622	0.199284008	0.025140591	0.979942824	29.79924889
14733	SRSF1	208.2494005	0.212170425	0.216408755	1.447124614	0.147862025	18.25184175
14734	BACE2	244.766167	0.125028522	0.194271824	0.642295512	0.520022267	15.01528171
14735	BST2	249.628899	-0.220060669	0.199227222	-1.15476522	0.248186579	14.9417129
14736	DPYD	207.2218819	-0.104528424	0.248999592	-0.419822756	0.674606904	14.11410181
14737	LAIR1	249.018225	0.022692222	0.192812994	0.169552678	0.865261155	14.994488
14738	STRAP	245.2955165	0.218942258	0.196086229	1.11656157		

0.264181821	21.88512885
14739	PSMB2	250.2227426	0.109915958	0.192512469	0.568001592
0.570022886	19.49544489
14740	CTTNBP2	214.8680029	0.086299291	0.220571711	0.291706121
0.695275259	10.7214901
14741	RAB8A	246.8264582	0.211622811	0.194778081	1.086522987
0.277242255	19.45044951
14742	DDX17	244.2750602	-0.117445414	0.200251767	-0.58648878
0.557547099	22.12174021
14743	APOBEC2C	282.6050088	-0.05260642	0.187919947	-0.279940627
0.779522049	19.98555788
14744	CS	240.067771	0.021128027	0.199881998	0.10570255
0.915818257	18.02140581
14745	SCP2	229.5587126	0.021855795	0.200761782	0.108864221
0.912210106	14.50974759
14746	SRSF5	260.5110552	-0.08512274	0.198906649	-0.427958246	0.66868152
24.24029044
14747	WASF2	249.5244257	0.067574667	0.192785999	0.248707682
0.727208776	11.75804081
14748	DDX29A	226.9870748	0.279762668	0.197275726	1.418125284
0.156151272	44.48210151
14749	LAMTOR1	244.1270266	0.186855242	0.19450895	0.960651122
0.226727609	27.12440529
14750	IDH2	268.4761122	-0.086609029	0.186569125	-0.464219517	0.64249048
12.7007118
14751	SKAP2	250.1622612	0.066292022	0.192442245	0.244477271
0.720487211	19.17118525
14752	TLN1	294.8498272	-0.048095298	0.198206787	-0.242652628
0.808274508	15.14572104
14753	VPS25	286.8874012	-0.256544469	0.185426691	-1.282461212
0.166522492	15.41878425
14754	ANAPC5	248.8226728	0.14575078	0.197118254	0.729407454
0.459659617	45.77715884
14755	DNAJC8	268.0082422	-0.126052228	0.188446824	-0.721971947
0.470211725	12.01119454
14756	TSPAN14	241.820605	-0.257811628	0.22418701	-1.149984722
0.25015016	17.77044405
14757	AP1G2	241.6076612	0.147066272	0.195662201	0.751629695
0.452272781	17.77917109
14758	HIF1A	196.2828048	-0.088405062	0.542659406	-0.162910772
0.870588689	17.14151581
14759	PRNP	441.8414041	-0.499257484	0.41881922	-1.1920596
0.222227877	18.85727721
14760	SRSF6	228.2808978	0.17692726	0.199626284	0.886292409
0.275459979	20.91824004
14761	SYPL1	269.7857061	0.0921442	0.191250795	0.486772478
0.626419576	11.054414
14762	PSMD12	241.7759227	0.007492497	0.201821712	0.027124228
0.970285867	18.97121159
14763	CRTAP	292.5541094	-0.242516884	0.180645254	-1.242502746
0.179422041	12.58187485

14764	QARS1	274.4591227	-0.010928964	0.194910991	-0.056122867	0.955242927	14.71518948
14765	RPL9	270.4806906	0.165695296	0.190599918	0.869226125	0.284662204	17.05015911
14766	BZW1	240.2492926	0.225428221	0.198219649	1.187115001	0.225182262	15.49902014
14767	FDFT1	202.1121926	-0.162987514	0.18126426	-0.899170709	0.26856174	14.54080411
14768	GPAA1	274.712596	0.17145545	0.190848596	0.898284652	0.268980515	21.40209775
14769	POLR2B	225.0689827	0.054852142	0.22075245	0.248481476	0.802761901	21.11877522
14770	ATP6V1G1	262.0224211	0.158605407	0.19001862	0.824682457	0.402895958	24.72015484
14771	CNDP2	222.7114724	0.29552517	0.201197827	1.468828764	0.14187924	20.84911815
14772	DDB1	252.2651156	0.062029607	0.199945946	0.215282245	0.752546605	21.44547884
14773	SEC11A	296.7248779	0.04728722	0.192652772	0.244184248	0.807088048	25.07291141
14774	CD99	214.2212505	-0.251246257	0.178275064	-1.409218559	0.158740992	11.88195451
14775	KMT2E	277.2795895	-0.171227628	0.185211227	-0.924000245	0.255486126	24.49115142
14776	TMEM20A	176.6222927	0.20674242	0.552272669	0.555417427	0.578609176	21.44214415
14777	CLINT1	241.2491107	0.049948402	0.201726066	0.247592827	0.804449461	21.8481747
14778	EIF2I	286.0298427	-0.076400987	0.187512258	-0.407442902	0.682682722	14.7854841
14779	MBNL1	257.0642404	0.190555984	0.19165151	0.994282756	0.220084742	15.44140817
14780	PSMD8	276.0897881	0.188170502	0.191082064	0.98475761	0.224742147	25.5407417
14781	DLD	245.015728	0.265552796	0.194622445	1.264449157	0.172426246	42.74800197
14782	AKR1A1	202.2591649	-0.150028788	0.180116214	-0.82295502	0.404870097	18.45171845
14783	MYO1F	201.9282824	-0.075449196	0.182044267	-0.412190984	0.680199445	18.51245552
14784	MAT2B	258.4847006	0.084275502	0.19276268	0.42494187	0.662604622	21.94129171
14785	PRRC2C	288.9202675	0.12689704	0.190672265	0.665520524	0.505717615	14.71811482
14786	ANKRD10	259.2645282	-0.071162562	0.194655662	-0.265586909	0.714672245	14.11994214
14787	HDAC2	285.2221268	0.19074194	0.192590845	0.985282885	0.224484646	10.57197414
14788	N4BP2L2	252.2628125	0.212997982	0.191620194	1.111505224	0.266250895	52.91119177
14789	CNOT1	226.658507	0.1052285	0.224021527	0.469702958	0.62856654	

44.11278728
14790 CTNNBL1 286.7547165 0.028045211 0.18587089 0.150885442
0.880066084 72.44998279
14791 MIF-AS1 270.8478127 0.049870527 0.18578811 0.268426959
0.788270691 45.19085485
14792 MLF2 265.9267782 0.114907701 0.188685621 0.608990229
0.542520909 14.01115525
14793 PSMD6 242.2781801 0.282624182 0.198247529 1.425612568
0.152980206 42.8815015
14794 TMC6 250.4045274 0.191970924 0.194261116 0.987702266
0.222298426 17.41501854
14795 ATP6V0D1 214.0529222 -0.105218465 0.179929484 -0.584776119
0.558698264 10.17194402
14796 PSMA4 280.8165644 0.064292024 0.182500827 0.250914079
0.725652809 24.14544782
14797 CD200A 216.9804144 -0.20851446 0.176210782 -1.182224064
0.226680729 41.90481485
14798 HSPA4 266.2082424 0.154571226 0.187171884 0.825825081
0.408902224 24.79875404
14799 PSMA5 282.2177812 0.029475219 0.182689229 0.216078952
0.828926205 42.91427547
14800 WDR6 262.7154615 -0.040258491 0.192900107 -0.207624906
0.825521856 27.95812182
14801 C11orf58 241.5740519 -0.012258006 0.21782212 -0.061222199
0.951102612 44.41144044
14802 RPL22 252.1272681 0.170416147 0.19809288 0.860284056 0.28962248
29.99225944
14803 HBS1L 292.1027182 -0.142025597 0.179822562 -0.795277655
0.426292854 21.14849479
14804 HMGN2 222.2980649 0.007078917 0.1848872 0.028287761
0.969458249 24.85424025
14805 CYRIB 281.7005578 0.142414745 0.194947995 0.725656425
0.461929815 27.04258148
14806 GTF2A 280.5570006 0.082864261 0.182976118 0.452869269
0.650642881 15.14851515
14807 UBE2L6 295.5814872 -0.205580704 0.182781607 -1.124724088
0.260701722 11.2414115
14808 IARS1 219.1658672 0.12596218 0.245760945 0.552229298
0.580106229 24.11971471
14809 PLAUR 269.8206278 0.012291422 0.189624974 0.070092265
0.944119428 27.81119171
14810 PSMB2 262.8259765 0.250102914 0.188252594 1.228547889
0.182997174 24.91917195
14811 CYBB 265.6612862 -0.19722618 0.208442022 -0.946187485
0.244052968 14.18442595
14812 EIF2K 281.9984569 0.206705419 0.182902222 1.122989585
0.261017444 10.45874421
14813 ZNF207 296.7786615 -0.020249864 0.17884452 -0.169699702
0.865246208 25.04511404
14814 DHX9 225.1655907 0.081017796 0.246054821 0.229267244 0.7419527
14.78724544

14815 FAM22A 257.072856 0.259217622 0.191452521 1.252945429 0.175752785 21.18214458
14816 GSTP1 288.2217679 0.129472994 0.182488787 0.76011726 0.447184496 12.28878182
14817 HMGN1 287.2822449 0.104291552 0.181220278 0.575146924 0.565191925 27.27241102
14818 MDH2 268.792262 0.251566422 0.186956619 1.245587195 0.178425684 18.01472405
14819 AC010761.1 275.8591492 0.070782512 0.185271104 0.281842217 0.702578402 11.88195544
14820 BNIP2 298.2476549 -0.200985151 0.18202022 -1.104191242 0.269510144 22.14481148
14821 SOD1 274.2121845 0.172672198 0.194072564 0.889725222 0.272608071 27.75851014
14822 SF2B2 265.1582455 0.004681184 0.196194228 0.022859926 0.980964221 20.95084151
14823 WSB1 269.2617222 -0.060288208 0.201624062 -0.299494079 0.764562089 21.2971758
14824 TARS1 282.764771 0.080242258 0.182412022 0.429892779 0.660014042 21.21117199
14825 CDC42SE1 286.1198221 -0.029988676 0.182905429 -0.162957266 0.869764785 19.70445477
14826 COPB1 200.08726 -0.158068811 0.179809167 -0.87909206 0.279251262 22.48914194
14827 PDCD6IP 270.2215122 0.051508224 0.192655622 0.267259024 0.789192728 14.01004082
14828 RPL25 204.51202 0.16210282 0.186277972 0.875118545 0.281509407 21.74450844
14829 PPP2R1A 242.4076769 -0.119252282 0.177058419 -0.674089842 0.500254196 22.48945049
14830 FUS 222.5182568 -0.026854428 0.177620878 -0.207477599 0.825626885 22.94507921
14831 HNRNPR 221.1027071 0.102142628 0.18569227 0.550066449 0.582272798 15.94280484
14832 PAIP2 299.5949671 -0.042626422 0.179087177 -0.228020516 0.811865182 11.54480214
14833 SRI 200.7927952 0.244782281 0.184918687 1.222729284 0.185592969 17.97021748
14834 TSPO 217.1817916 -0.118222255 0.176914226 -0.668811948 0.502615444 21.18252448
14835 TUBA1C 282.7484469 0.075626644 0.182778414 0.41156425 0.680658766 12.14951459
14836 NCKAP1L 295.2625721 -0.246110762 0.188271171 -1.207214272 0.191129949 19.50420709
14837 UBR4 260.7240722 0.048881762 0.204616692 0.228894202 0.811187544 20.41880587
14838 PPP1CA 225.9100208 0.007819986 0.180580562 0.042204692 0.96545865 24.17044159
14839 KPNA2 275.2489569 0.272169862 0.184659145 1.479219426 0.129054961 21.94119151
14840 PRDX2 212.2241462 0.124457166 0.181260548 0.741280456

						0.458462791	24.29449581
14841	PTP4A1	285.6018677	0.076248126	0.187720561	0.406711581	0.684219824	12.57142114
14842	SEC21A	201.1068565	0.062560076	0.17777288	0.257522265	0.720692627	20.87458751
14843	SLC11A2	244.1729616	0.129281616	0.217754225	0.594162487	0.552402771	18.40079114
14844	CBX5	202.1717742	-0.094725268	0.178454557	-0.520864942	0.595512272	18.11912191
14845	PLIN2	221.8922499	-0.21012452	0.172215406	-1.220126208	0.222417025	15.05751125
14846	RBM29	272.2165595	0.057799229	0.191256416	0.202208106	0.762492425	18.99194849
14847	POMP	284.2292222	0.22792184	0.182198207	1.251009647	0.210920958	19.11849071
14848	SKP1	252.1071762	-0.170292126	0.175812802	-0.969167969	0.22246129	21.49288105
14849	CHCHD2	288.5572059	0.096722215	0.180888561	0.524712169	0.592848905	14.11817921
14850	SPCS1	245.855006	-0.054778682	0.182055021	-0.200890808	0.76249776	15.75811094
14851	AL671882.2	222.1406607	0.022891842	0.184264166	0.124222821	0.901120126	19.95454814
14852	NDUFA1	286.8545502	0.159866421	0.181964696	0.878557249	0.27964122	18.92551107
14853	SNX5	297.40568	0.129514256	0.179912266	0.719870672	0.471604626	21.15411445
14854	GATA2	406.0996825	-0.228942485	0.185416221	-1.224748225	0.216924222	21.87579019
14855	SUMF2	218.7246578	-0.121219115	0.174289967	-0.75245195	0.451178275	22.27295194
14856	ATP6V1A	246.8265171	0.267891211	0.208959412	1.282025098	0.199822842	19.97421554
14857	DYNLL1	226.0111507	0.060219296	0.176684014	0.241297022	0.722804717	19.51579847
14858	ERP44	288.0168101	0.212840419	0.188448172	1.12942742	0.258712255	41.79449945
14859	OXA1L	220.1582614	0.226708728	0.182690466	1.288628272	0.19752725	14.01711548
14860	CAPZB	255.8298154	-0.186616715	0.170775199	-1.092762292	0.274498128	15.84554401
14861	COPZ1	202.2265092	-0.009940858	0.178265842	-0.055764222	0.955529629	14.58142917
14862	HEXB	222.0208762	-0.082561018	0.175044997	-0.471655968	0.627172271	18.74981245
14863	SLC2A6	216.0122415	-0.120845228	0.175680019	-0.687871215	0.491522885	12.82899191
14864	NUCB1	218.205087	0.090647855	0.178627825	0.507467721	0.61182668	17.55289014
14865	PDAP1	224.8168015	-0.084594659	0.172092126	-0.491562262	0.622028119	22.71020157

14866	RAB1A	212.5284511	0.196626891	0.180624599	1.088522941	0.276259462	14.47847782
14867	SHMT2	226.0761112	0.129619617	0.180684244	0.772726702	0.42968414	17.40441787
14868	FAU	211.7588955	0.129455967	0.178088051	0.782072125	0.422584172	27.51784918
14869	HERC2	200.4582095	0.046046462	0.180812925	0.254662152	0.79898405	22.44428507
14870	CCND2	229.975595	-0.022260025	0.174852915	-0.122026292	0.894172587	14.19102924
14871	EEF1D	205.2058211	0.194421442	0.177807554	1.092427478	0.274201766	21.74441491
14872	STAT1	270.5074224	0.098262625	0.197205502	0.498275222	0.618289999	24.02218405
14873	RPS7	215.5449688	0.250776997	0.180002086	1.292181652	0.162564866	17.51449577
14874	CD48	225.5252599	-0.117125762	0.171807245	-0.68172722	0.495411282	15.14719408
14875	DAP	201.490778	-0.010912212	0.185816986	-0.058725592	0.952170674	21.14410144
14876	ANXA7	258.4568498	-0.088682572	0.170211152	-0.52102092	0.602252191	25.24112057
14877	SRPRA	296.4877241	0.228056728	0.178728717	1.275922422	0.20198294	12.85201944
14878	RPL24	218.6619641	0.169605054	0.179802249	0.942280828	0.245527274	24.40197895
14879	ARHGAP20	222.2262692	-0.058122214	0.174157605	-0.222797159	0.728522627	22.87748419
14880	NARS1	288.4864421	0.264865062	0.182725486	1.441556282	0.149427561	22.15145448
14881	PAFAH1B2	227.1517898	-0.066092529	0.171875492	-0.284542065	0.700575982	11.54417041
14882	EIF4H	222.7957814	0.081256742	0.17459851	0.465291957	0.641650792	41.84811819
14883	ERGIC2	204.2772447	0.129187489	0.177242659	0.72845846	0.466222988	19.95142177
14884	GNG5	206.629074	0.214787022	0.176642449	1.215942279	0.224006911	19.81114112
14885	MAP1LC2B	205.7254699	0.261629227	0.181245095	1.442510662	0.148876671	44.49055277
14886	NEU1	207.662149	0.115209725	0.182272269	0.628282962	0.529818591	15.55172755
14887	RNH1	224.4112822	0.102426825	0.172760425	0.592941496	0.552220206	22.29254907
14888	RPL26AL	227.8872226	0.097276072	0.172291105	0.564275472	0.572566671	24.01524957
14889	SON	207.4524944	0.244549125	0.177022244	1.281272248	0.167164227	12.52487212
14890	STIP1	212.5227447	0.211962995	0.175079577	1.210672204	0.226021021	20.1077519
14891	SUB1	222.5964021	-0.092201901	0.17091807	-0.540025942		

0.589172245	14.74402812
14892	HM12	224.6642622	0.104724151	0.17222999	0.607695449
0.542289482	44.14217802
14893	MAPK1IP1L	294.0746487	0.214177469	0.184792642	1.159008856
0.246452575	11.78411195
14894	SH2GLB1	229.8624452	-0.205796211	0.176402026	-1.166621721
0.242259127	24.15118715
14895	ATXN10	295.4105522	0.048219976	0.18869804	0.256070262
0.797896506	58.17544849
14896	COPG1	212.4865219	-0.185424961	0.189542827	-0.978227504
0.227912271	27.77542111
14897	IDH1	222.0026741	-0.017727556	0.171017812	-0.102717591
0.917292466	12.24047842
14898	ILF2	216.8889069	0.224605256	0.176798249	1.270404206	0.20294065
12.14104447
14899	ACSL1	212.1674208	-0.071296994	0.192276917	-0.269211564
0.711970027	21.41051147
14900	MVP	247.1211744	-0.21812775	0.16875202	-1.292585679
0.196154295	17.1401421
14901	NCOR1	227.8040204	-0.049472194	0.17545147	-0.281970812
0.777965891	14.19198918
14902	FLII	224.9909905	-0.216075744	0.179672899	-1.202599516
0.229121225	41.1121494
14903	RPS15	249.8249749	0.166857145	0.177680546	0.92908505
0.247687079	14.19874141
14904	APMAP	260.2856477	-0.162225586	0.165092722	-0.988750892
0.222785027	25.08504149
14905	DHX15	295.599009	0.154680411	0.187082521	0.826802069	0.40824869
19.47014549
14906	ARPC4	228.2192625	-0.104427999	0.171175246	-0.610064842
0.541818855	24.20544181
14907	ATP5F1E	251.4794182	0.074857229	0.169958267	0.440445254
0.659614582	11.29140844
14908	SELENOF	222.1608481	-0.006028441	0.176599749	-0.024192804
0.972722405	19.12948191
14909	STMN1	271.750992	-0.026047172	0.1674697	-0.215245942
0.829575572	95.4024114
14910	G2BP1	241.2620029	-0.027501785	0.168752227	-0.162971291	0.87054096
77.8588149
14911	CALM2	298.814624	-0.040614268	0.176701502	-0.229847227
0.818210408	44.24441829
14912	RPS2A	221.4622128	0.192516081	0.172024947	1.119117222
0.262090124	51.45994181
14913	GNAI2	229.9208722	0.106497629	0.176659414	0.602841572
0.546614087	15.81541974
14914	RBBP4	225.1915907	0.080224262	0.171666222	0.467967789
0.629807625	52.15271011
14915	PDLIM5	214.1484217	0.127401914	0.177248704	0.718269582
0.472529427	18.14987421
14916	BRK1	250.8615198	-0.029565881	0.166864712	-0.227112521
0.812568715	45.48407997

14917	TUBB4B	280.4686402	-0.090148704	0.167902082	-0.526912265	0.591228149	50.04871149
14918	CDC42	256.8100067	-0.192504224	0.168120281	-1.145029269	0.252192891	14.12470571
14919	AC145207.2	242.2210902	0.152792421	0.168696126	0.911659412	0.261948026	10.97119094
14920	CAPRIN1	259.2268778	-0.094156765	0.165677895	-0.568212175	0.569822012	14.4552499
14921	PCMT1	272.2774928	-0.171485627	0.1624488	-1.049170265	0.294099716	44.48744704
14922	ATP5MC2	255.6497851	0.158204022	0.169427724	0.924245449	0.250125727	12.91074424
14923	HADHB	242.059245	0.190912467	0.170172264	1.121870428	0.261917522	44.1545841
14924	RTN2	265.9887645	-0.042289722	0.162856968	-0.258089257	0.796228022	50.14170091
14925	SRRM2	226.1221429	-0.009219567	0.172472	-0.052455445	0.95726902	28.94852158
14926	HNRNPM	269.9041694	0.025541687	0.165672191	0.154170022	0.877475671	21.81752814
14927	CD27	427.9927909	-0.2261572	0.165869925	-1.262461679	0.172727054	24.88151149
14928	RPS21	249.7112282	0.172419624	0.168680021	1.028098188	0.202902642	14.00472021
14929	YWHAE	400.2712871	0.060979182	0.176049228	0.246275529	0.729060512	24.12285121
14930	DAD1	254.590526	0.077900227	0.165854201	0.46969129	0.62857552	14.8744594
14931	MARCHF6	225.7912567	0.198274466	0.174282621	1.128227822	0.255025262	21.11470488
14932	KCNQ1OT1	222.7508894	0.094920042	0.182541158	0.517159427	0.605044865	14.04701011
14933	GARS1	245.6686768	0.15426206	0.172242574	0.896195721	0.270148225	19.48455871
14934	EIF2F	270.4222029	0.14691906	0.167285279	0.878254564	0.279805587	21.17507942
14935	HSD17B4	271.6667296	-0.147401272	0.16592559	-0.888258288	0.274248055	21.91085429
14936	NAP1L1	285.6256652	-0.149427408	0.162091566	-0.916217871	0.259552624	14.14115472
14937	CELF2	415.427122	-0.220492721	0.157568228	-1.299252022	0.161707144	14.0441148
14938	MAPRE1	275.2584766	-0.024741566	0.162242275	-0.214001822	0.820545629	21.2914541
14939	DBNL	278.5679816	-0.025489915	0.162171722	-0.157178522	0.875104146	24.14940091
14940	MOB1A	279.9424822	-0.161420469	0.162258586	-0.994824682	0.219816675	29.92291441
14941	TCP1	268.6147952	0.189275195	0.16667242	1.126211947	0.255867872	22.91994201
14942	ATP6V0B	410.0026969	-0.156775277	0.161577477	-0.970279276		

						0.221907204	19.01841488
14943	SNX17	275.2720962	-0.224486972	0.174161718	-1.288957042	0.197412022	21.79470585
14944	TMEM59	265.7948926	0.217402826	0.16624928	1.207691044	0.190978122	24.45741919
14945	C1orf42	267.7111241	0.165702929	0.164847752	1.00519275	0.214802564	21.10228202
14946	EIF2A	241.0200612	-0.075106725	0.181981717	-0.412715772	0.679814867	24.54705121
14947	TMED2	292.6000174	-0.097988929	0.159102829	-0.615880257	0.527972486	17.55278904
14948	EWSR1	270.6090242	0.106254909	0.165716506	0.641788265	0.52101067	21.2810529
14949	CCT4	226.1951827	0.220575718	0.177086896	1.202048449	0.192899824	17.72290451
14950	ACLY	207.8414882	0.087261557	0.206040169	0.424002549	0.671562954	12.2278947
14951	ARL8B	228.2185665	0.180557727	0.177990086	1.014425752	0.210279641	22.14745495
14952	FXYD5	442.9812987	-0.225529216	0.158451229	-1.422298226	0.154620715	18.40044
14953	BPI	471.1241677	-0.240870571	0.167129864	-1.441218019	0.149522099	12.41111711
14954	YWHAQ	402.2251922	-0.052688622	0.159652249	-0.220021167	0.741282968	25.09058091
14955	CD46	211.4422657	0.076224122	0.20744165	0.267920556	0.712925015	14.12924945
14956	PTGES2	260.2402789	0.122982199	0.165267072	0.804656242	0.421018019	21.88771141
14957	VAMP8	249.2650807	0.106942486	0.171506244	0.622548281	0.522924285	21.14478119
14958	RPL26	294.2524097	0.1110188	0.161172625	0.688819167	0.490927072	24.27589485
14959	AL671277.1	276.0661929	0.210446768	0.162810494	1.2846965	0.198898262	24.44841218
14960	RPL41	269.9828467	0.186761141	0.162827598	1.146916576	0.251416101	22.42574071
14961	PSMB1	421.2158591	-0.008825292	0.162590852	-0.054279145	0.956712765	15.21919998
14962	GSTO1	290.0660506	0.017174122	0.160162671	0.107228579	0.914607642	19.11414171
14963	TBXAS1	297.4669461	-0.121510687	0.160012154	-0.821879262	0.41114556	17.4252552
14964	NRBP1	422.7527511	-0.014795648	0.157582057	-0.092891707	0.925195182	21.05241017
14965	RPL22A	282.1404214	0.142095852	0.161918422	0.877576807	0.28017242	21.71441157
14966	CALU	290.6272171	0.042221515	0.161469777	0.267675571	0.788949055	17.00771487
14967	TMED10	245.5122186	0.098826271	0.184019002	0.527098179	0.591199799	21.47214119

14968	ILF2	400.0857229	0.151205004	0.158950847	0.951268944	0.241467868	28.54242598
14969	NCSTN	284.2741722	0.185885626	0.160895929	1.155215971	0.247961058	14.79004975
14970	PARK7	419.2999159	-0.005746198	0.156124492	-0.026802875	0.970642182	15.80411029
14971	SSR1	426.6804211	-0.026915882	0.15689189	-0.171556881	0.862785909	15.94250201
14972	OS9	416.0590726	0.048290669	0.157487008	0.207267691	0.758629626	40.18044417
14973	COX7C	412.9885712	0.066940222	0.156219619	0.428501448	0.668286079	41.10052541
14974	ANXA5	402.0871628	0.046218864	0.157454251	0.292528184	0.769110818	21.89901194
14975	RPS28	428.9228652	0.057602249	0.160681449	0.258487859	0.719978252	21.41279912
14976	RBMX	429.0175775	-0.121272865	0.152125206	-0.797128721	0.425270465	15.07142495
14977	SNAP22	282.6219412	0.074448247	0.164276019	0.452190598	0.650411502	21.41941001
14978	UBC	452.2797182	-0.019944174	0.155128825	-0.128565228	0.897701682	11.49411844
14979	COPB2	449.5878985	-0.157201092	0.15064207	-1.042540446	0.296698059	47.12888089
14980	HNRNPH1	402.8720182	0.011971052	0.162698502	0.072578124	0.94124607	18.79115504
14981	MARS1	406.6050741	0.159764682	0.161072748	0.991872885	0.221259528	24.24141051
14982	H2AZ1	442.2522229	0.025027228	0.152708102	0.162822152	0.870657679	14.80804425
14983	NEK6	469.0219651	-0.124025406	0.158179791	-0.847297908	0.296829092	19.4510004
14984	SF2B1	245.7611892	-0.069222226	0.220979227	-0.212754498	0.752707506	11.4041014
14985	EIF2S2	410.7824528	0.012969892	0.157620907	0.082280125	0.92442295	20.4440714
14986	SEPTIN7	427.9885007	0.117170758	0.155795479	0.752080606	0.452002586	14.71418972
14987	ATP5PB	426.4796427	0.089611988	0.152829922	0.582529228	0.560202452	15.50757117
14988	LMAN2	412.9614212	0.204188649	0.157927652	1.292842291	0.19606522	19.80544017
14989	UBA52	445.6674946	0.058662649	0.156951726	0.272762247	0.708581126	11.51019515
14990	HDLBP	402.2692224	-0.191081026	0.182764496	-1.045504079	0.295790056	19.98824101
14991	RPS17	401.6611827	0.079020452	0.162847696	0.485202865	0.627461522	41.17184142
14992	ATP5F1C	486.5422608	-0.126075126	0.150204922	-0.829254171	0.401270592	15.0422824
14993	HNRNPDL	446.2277489	0.129274516	0.15202089	0.845414291		

						0.297879512	21.71924804
14994	AKAP12	408.7262081	-0.060406894	0.172626467	-0.247892898	0.727920619	11.54942714
14995	EIF2H	459.2217281	0.108421922	0.157057914	0.690220914	0.489986111	45.18581895
14996	TXNRD1	292.5284125	0.199862128	0.166627792	1.199280549	0.220280007	14.21840872
14997	ELOVL5	472.228287	-0.020012849	0.149550187	-0.200694159	0.840927727	24.81191919
14998	TM9SF2	447.4604259	-0.100710778	0.156226116	-0.644225142	0.519422965	22.40450184
14999	EIF4B	418.2122429	-0.047671147	0.170428296	-0.279697228	0.779709805	17.50021794
15000	PTPRC	298.6250192	0.200787294	0.165222056	1.215176906	0.224298626	24.54998918
15001	GANAB	446.547661	0.005296085	0.162846889	0.022125941	0.972566181	15.49911521
15002	SSR4	446.7002121	0.05011292	0.152892227	0.225626505	0.74469929	11.41854455
15003	EIF2L	447.5571589	0.219429174	0.151274282	1.450602672	0.146890252	40.47714227
15004	HCLS1	522.1192548	-0.129608176	0.147452856	-0.878974476	0.279415115	44.15001101
15005	RAB7A	504.0522271	0.011610768	0.148026444	0.078421824	0.927484559	20.54175447
15006	MYH9	478.1448278	-0.05802892	0.147890525	-0.2924452	0.694729295	10.44418575
15007	CHP1	502.746499	-0.208241149	0.147621884	-1.411216154	0.158151412	24.47529452
15008	MPP1	529.4722807	0.086166466	0.152172114	0.566242467	0.571228285	19.01011094
15009	DDOST	490.5016097	-0.076105556	0.148562219	-0.512280692	0.608454576	17.92989084
15010	SET	519.8879269	0.012770881	0.146652469	0.087082619	0.920605841	18.1941925
15011	RPS4Y1	495.622842	0.116679721	0.149602562	0.779921266	0.425421275	12.04780144
15012	LMNA	562.5725412	-0.011800102	0.156896726	-0.075209259	0.940048128	40.98107572
15013	CAPZA2	457.4290297	0.058089248	0.158297576	0.266962228	0.712647111	10.25145991
15014	CNN2	557.4209976	-0.120092729	0.144942462	-0.897547468	0.269426856	14.174874
15015	XRCC6	552.7221682	0.002118522	0.148822956	0.020954651	0.982281821	18.87905427
15016	CD164	501.0162577	-0.016124447	0.144566284	-0.111526625	0.911190824	25.08501025
15017	PFDN5	478.1867727	0.096821266	0.148666221	0.651222241	0.51482122	19.71794501
15018	RPN1	475.940789	0.128254988	0.147622275	0.926528592	0.248995952	24.09981814

15019	RPS29	529.9478268	-0.025449605	0.142682225	-0.248449512	0.802786629	14.45017212
15020	S100A8	607.6529469	-0.146047774	0.148762664	-0.981742594	0.226226182	21.07414198
15021	SH2BGRL	552.725079	-0.128009052	0.129728959	-0.987691128	0.222202941	14.42550148
15022	HADHA	492.0724665	0.150764985	0.148227718	1.016262112	0.209456504	14.54401181
15023	UCP2	611.2857161	-0.188281926	0.145177207	-1.29759905	0.194425149	17.44081224
15024	ZEB2	501.8091174	-0.102621228	0.161962244	-0.62978227	0.522214096	29.97908171
15025	COPA	507.6449409	-0.059028712	0.148222454	-0.298040299	0.690600481	18.10459811
15026	ATP6AP1	499.8711254	-0.129262282	0.155782258	-0.892954781	0.271246086	42.11800571
15027	SSR2	499.5978942	0.162666941	0.144892401	1.129567946	0.258658224	49.92011447
15028	DNAJA1	504.2580224	0.152289648	0.145902405	1.04445572	0.296274591	54.58907881
15029	SURF4	514.6118224	0.082928942	0.142840665	0.58056956	0.561520592	12.49847004
15030	CTSA	540.0674702	0.012625825	0.14097792	0.089629806	0.928581298	17.51071724
15031	XRCC5	521.7271546	-0.004486518	0.142951517	-0.021166872	0.97512646	72.7712021
15032	EIF4A2	521.6101615	0.012748287	0.142580984	0.089410848	0.928755402	15.44595141
15033	PPP1CB	502.6488782	0.00295529	0.150740964	0.026229648	0.979066192	40.84129059
15034	RPL27	488.1522444	0.225219242	0.15928274	1.47672166	0.129747582	17.09815451
15035	SNX22	574.1217601	0.21424257	0.151920701	1.410798269	0.158204105	47.54445818
15036	IL1RL1	529.8702662	-0.114629292	0.145980884	-0.785225642	0.422215421	12.01944717
15037	GCSAML	455.2242272	0.079605207	0.179210875	0.442951261	0.65707775	40.28484144
15038	LRRC75A	571.2056878	-0.109197188	0.127675558	-0.792148682	0.427691202	18.49407047
15039	UBE2D2	587.0072818	-0.057802279	0.150805606	-0.282297281	0.701499255	15.52010181
15040	ATP6V0E1	567.7812729	0.004467826	0.127916462	0.022295222	0.974156865	12.07214822
15041	CCT7	547.1694159	0.081795182	0.129960012	0.584418222	0.558928962	24.28520111
15042	TGOLN2	505.0779616	-0.022446187	0.162911821	-0.142919492	0.885564042	21.05114104
15043	CSF2RB	458.2288042	0.155121078	0.181042522	0.856821227	0.291542687	18.19404114
15044	ENO2	561.9791182	0.129669855	0.129845208	0.927227798		

0.252802082 21.22877194
15045 NPM1 594.6821019 0.044124268 0.129907277 0.215452474
0.752417271 17.18914192
15046 CCT8 545.9681566 0.194220902 0.142584482 1.262917615
0.172908476 28.8449718
15047 RPL27A 548.8605225 0.085874442 0.129442692 0.615825982
0.528002776 14.11575941
15048 SOD2 462.5957781 -0.059970515 0.2687289 -0.162626801
0.870804409 19.48700544
15049 HSPA9 517.7784669 0.205646218 0.147141912 1.297604629
0.162221828 18.91118211
15050 RPS25 557.6474129 0.127779828 0.129061429 0.990782995
0.221791066 21.75580598
15051 RPL25A 582.0500765 0.087962274 0.12929199 0.621050422
0.528007556 22.87474457
15052 PSME1 550.2206424 0.052520207 0.142918272 0.267554169
0.712205694 28.5407495
15053 LEO1 606.4672056 -0.015096974 0.12742708 -0.109846427
0.912521162 15.7824844
15054 S100A9 566.0101472 0.059964169 0.128105866 0.424189877
0.664150564 14.41540291
15055 CD84 590.9926224 -0.022276295 0.145448298 -0.22190906
0.824284677 47.91007158
15056 SNX22 558.655509 0.029088612 0.141711272 0.27582258
0.782676659 20.19509482
15057 WDR1 581.8817766 0.109225728 0.142990787 0.76462477
0.444489059 21.41209524
15058 GHITM 547.4784279 0.19712722 0.129582097 1.412257178 0.15787425
22.85111298
15059 SEC61A1 562.8967229 0.106992115 0.128524517 0.77222099
0.429924227 19.70294744
15060 CYP1B1-AS1 584.0782595 -0.127886489 0.14949179 -0.855475
0.292288229 21.91714508
15061 RPL29 604.7161798 0.029025676 0.126456607 0.285992205
0.774882249 21.82479519
15062 COX4I1 602.2925977 0.165809218 0.140404646 1.180928261 0.22762725
15.79194158
15063 GDI2 529.2517272 0.149661691 0.142419841 1.042521525
0.296706817 14.54112181
15064 JSRP1 578.2202612 0.125475727 0.127954242 0.909545228
0.262062242 25.78182244
15065 RPL27 552.2229585 0.181084922 0.129969264 1.292747827
0.195752526 24.59788842
15066 ARPC2 675.1660626 -0.125225521 0.124415298 -1.006101407
0.214266792 22.59892019
15067 RPL28 595.4217787 0.119291092 0.12547914 0.881250728
0.278182122 24.41742117
15068 HNRNPF 606.4190866 -0.006675279 0.124972917 -0.04945642
0.960555558 14.54484804
15069 RPS27 612.2581779 0.026951299 0.124247612 0.200758872
0.840887124 27.80910291

15070	YWHAB	665.5224219	-0.040226991	0.122672066	-0.202959921	0.761158426	11.24141011
15071	CSDE1	615.9296444	0.082951288	0.126120516	0.609251894	0.542291217	21.19702879
15072	MYL12B	682.7405569	-0.152209898	0.129978657	-1.171027629	0.241582652	20.0011142
15073	SERF2	702.5627552	0.005460679	0.126117542	0.040117276	0.967999549	14.45100485
15074	RPL22	622.2782717	0.124022924	0.122217212	1.00526856	0.214719412	28.14817272
15075	LPXN	670.2201462	0.042555256	0.122500984	0.226254942	0.744221476	28.18448181
15076	EIF2E	606.9081912	0.144594878	0.125670847	1.065777072	0.286524427	21.74151472
15077	NONO	664.5272702	-0.116265925	0.121648996	-0.882910526	0.276744509	19.48112441
15078	AP2M1	722.5562122	-0.044222116	0.1244629	-0.228959042	0.742186646	27.18184191
15079	RBM2	656.1716616	-0.021117595	0.121004566	-0.161197297	0.871927921	18.7171912
15080	AC020914.1	708.7242018	-0.118998214	0.120212886	-0.9128751	0.260782479	17.01901711
15081	CMTM6	605.8156274	0.028698124	0.146911845	0.19524248	0.845124842	14.59211129
15082	CANX	525.0968198	0.020701841	0.419288622	0.072222644	0.941628152	10.87512851
15083	TAPBP	644.5116142	0.126608255	0.122229511	1.022224772	0.201915217	20.89412142
15084	M6PR	699.5290522	-0.028885897	0.128456454	-0.202716566	0.762105872	21.90481477
15085	ALDH1A1	746.7429948	-0.066050564	0.129774458	-0.508964282	0.610777261	18.04421901
15086	APLP2	648.0027126	0.080211115	0.122690652	0.605250729	0.545012407	18.47947794
15087	RPS9	716.7298104	0.086727801	0.120140218	0.66649497	0.505094778	21.9219259
15088	RPL22	684.7667712	0.042999141	0.129289794	0.240051091	0.722818052	25.14971144
15089	RAN	704.1240982	0.191412665	0.124645554	1.421611482	0.15512907	20.18511704
15090	PPIB	690.4006905	0.186208592	0.140916177	1.221412878	0.186262401	11.15289014
15091	VCP	712.127659	-0.125502489	0.129494202	-1.046297209	0.295277624	18.9824405
15092	RPL7	722.1288856	0.1420426	0.120062797	1.099787974	0.271424512	27.91114712
15093	RPLP2	711.9912554	0.16285222	0.129420926	1.266049672	0.205495299	25.90410154
15094	CNBP	705.5262781	0.052562789	0.120680669	0.402222142	0.687519812	14.59984444
15095	FERMT2	702.4584296	0.077228182	0.12229017	0.582854291		

0.559218248 11.82294114
15096 LAPTM4A 756.6987002 0.029122294 0.127480144 0.206976291
0.758861242 12.55720244
15097 TAGLN2 740.0220505 0.17254005 0.122192006 1.202921642
0.192601514 18.74285044
15098 GPX1 704.8641102 -0.024792894 0.120757266 -0.189617567 0.84960882
51.19542128
15099 RPS16 719.6669999 0.11126022 0.127192492 0.875518289
0.281291964 20.91291979
15100 HLA-C 767.5247908 0.126089691 0.122421542 0.945047472
0.244624641 12.10420708
15101 LGALS2 742.1107241 -0.115527518 0.126467524 -0.912495456
0.260982022 24.77411121
15102 MT-ND6 745.4292296 0.060216517 0.126241226 0.476617705
0.622624274 15.51149477
15103 PDIA2 772.6987466 -0.052114212 0.126707101 -0.41918891
0.675078077 18.49459014
15104 CD82 780.1115222 0.089042486 0.12929144 0.68869591
0.491014651 14.4449599
15105 AP002252.1 722.0504275 0.162145228 0.126585009 1.280920455
0.200221607 18.74941114
15106 TALDO1 728.8182741 0.159290078 0.120152775 1.224628921
0.220715062 19.17745121
15107 ATG10 766.9248529 0.148146978 0.126224908 1.172581705
0.240562612 11.40027459
15108 RPS20 722.2264568 0.155296906 0.125848526 1.222998602
0.217202425 17.89944191
15109 TXNIP 859.022221 -0.116428808 0.122625591 -0.949465827
0.242282745 44.45481488
15110 PPIA 792.5592929 0.066569266 0.122749604 0.527925189
0.590621794 17.74115919
15111 SERPINB1 892.5662218 -0.095522142 0.12772644 -0.747892624
0.454524927 19.44177474
15112 TYROBP 824.2447855 -0.158972442 0.121017244 -1.212624625
0.188969191 21.95544849
15113 SNHG29 828.9066182 -0.120277487 0.120262787 -0.999282011
0.217657612 19.87218715
15114 ARPC1B 912.5914581 -0.026169814 0.121906662 -0.274207625
0.782925061 24.80710989
15115 RPL24 770.0576699 0.174089272 0.122714621 1.407184291
0.159272748 24.21188257
15116 NCL 761.0082884 0.145592871 0.128061582 1.126897222
0.255581207 47.91414919
15117 CORO1A 772.0161902 0.158847225 0.122271225 1.287555796
0.197900625 29.01105144
15118 DAZAP2 820.595572 -0.055892615 0.120892548 -0.462227451
0.642829247 28.40540441
15119 GM2A 792.2291099 -0.015704901 0.124092558 -0.126557959
0.899290272 17.11144491
15120 HLA-B 816.0015727 0.182985081 0.124570576 1.46892699
0.141852592 24.59792849

15121	RPL20	860.6226052	0.027477995	0.120251124	0.211405444	0.755492416	20.0414084
15122	AL662797.2	852.7862476	-0.012515228	0.12040559	-0.112247594	0.910627092	17.49449401
15123	TPP1	1072.569529	-0.144782955	0.125547216	-1.068126692	0.285458857	21.11849958
15124	CD52	862.5642001	0.087156744	0.122970578	0.702042779	0.482028467	14.21481257
15125	RPL28	866.4820926	0.127126566	0.120515459	1.054856915	0.29149077	20.52712725
15126	RPS27A	992.5675242	0.002226061	0.127029414	0.017601215	0.985956907	12.12242425
15127	S100A11	950.8722594	0.027649701	0.122049202	0.208479692	0.75771725	12.21445111
15128	ID2	929.4216854	-0.022070962	0.122286506	-0.179021725	0.857920642	24.48417815
15129	SARAF	908.9629277	0.110558959	0.119856295	0.922429204	0.25620469	28.51844941
15130	WARS1	912.9126544	-0.051124278	0.117254828	-0.426096107	0.662767002	17.92420117
15131	PRDX1	918.4705704	-0.16129979	0.122269782	-1.218951258	0.187185275	18.04421901
15132	MS4A2	955.1220282	0.012956059	0.117591195	0.110178816	0.912267562	11.92427422
15133	ATP5F1A	895.592522	0.127866742	0.118582772	1.162610529	0.244987557	21.95441717
15134	RPS22	992.6262668	0.050692812	0.122742291	0.412006447	0.67960189	17.54154471
15135	CALM2	901.1850405	0.024574019	0.122622558	0.20028576	0.841178895	14.40141718
15136	NACA	917.7227568	0.065270985	0.116662112	0.559482627	0.575822284	14.14049819
15137	SLC25A2	928.09029	0.110441559	0.117447076	0.940251714	0.247027181	18.95515711
15138	RPS11	900.12522	0.079704565	0.120780745	0.659911188	0.509210824	14.08755422
15139	TPI1	989.5910259	0.057066578	0.121789105	0.468568822	0.629277867	11.22201419
15140	RPL21	965.77546	0.074446505	0.116267496	0.640202671	0.521975194	21.20559489
15141	HNRNPA2B1	921.5875989	0.122879626	0.117820921	1.12620104	0.255872425	29.81724871
15142	ARF1	1022.104882	-0.068952712	0.114010942	-0.604799072	0.545212509	45.00494525
15143	LDHB	1101.121282	-0.12860485	0.114156871	-1.126562501	0.259927501	55.42508427
15144	H2-2B	1102.601221	-0.05214421	0.11476724	-0.454247516	0.649578722	45.58772484
15145	HSP90AA1	1045.874255	0.120711956	0.11726049	1.112764577	0.265280197	14.14549451
15146	EIF1	1082.812222	0.067650751	0.122928778	0.545840066	0.58517588	

22.7844149
15147 HNRNPK 1100.227587 -0.024144071 0.114040502 -0.29940202
0.764622558 21.00058145
15148 HNRNPA1 1056.085204 0.104699726 0.112248011 0.924517221
0.25521709 24.25704779
15149 PLD2 1094.571491 0.089252698 0.119022026 0.749814514
0.452266426 19.95121917
15150 MAT2A 992.8018987 0.124798155 0.117619828 1.061029902
0.288676212 14.24520497
15151 UBXN11 1111.614428 -0.004048916 0.111114142 -0.02642925
0.970922118 27.44841442
15152 CAPZA1 1055.21717 0.110766715 0.112674516 0.982068022
0.225572956 11.20187424
15153 MT-ATP6 1141.926259 0.172122214 0.129021277 1.241782822
0.179666099 41.51498559
15154 TPM2 1165.029122 0.101747878 0.115220126 0.882997188
0.277227791 24.2148044
15155 C6orf62 1065.959804 0.117557049 0.112828282 1.041817912
0.297496108 19.97404101
15156 MSN 1022.118927 0.152526669 0.12696862 1.209248924
0.226567222 14.5507402
15157 MT-CO2 1269.707244 -0.102425228 0.117591125 -0.87102864
0.282728516 20.14127125
15158 OAZ1 1280.529722 -0.008790741 0.108572697 -0.080965662 0.92546926
21.44187421
15159 RPL11 1226.626887 0.070050286 0.107826186 0.649660246
0.515911708 19.49848199
15160 TMBIM6 1212.141141 0.111910679 0.108572088 1.020749992
0.202658075 44.71188188
15161 RPL27A 1224.794699 0.042482848 0.109722798 0.296297297
0.691885721 14.04112715
15162 CALR 1250.499726 0.112002279 0.10808262 1.045508726
0.295787905 19.40844805
15163 MRNIP 1252.521097 0.152612202 0.106625742 1.44054225
0.149714017 24.78441414
15164 SIGLEC6 1425.545404 -0.117008822 0.106642955 -1.097201704
0.272552229 18.77514081
15165 MYL6 1425.220941 -0.094122222 0.105426276 -0.892702554
0.272016482 14.04944051
15166 RPS14 1216.121089 0.154259568 0.106776025 1.44562882
0.148278529 17.07815795
15167 PGK1 1255.469527 0.127608826 0.102629671 1.221272945
0.218220422 19.51812017
15168 ARPC2 1592.89527 -0.141092229 0.10066426 -1.401622972
0.161027864 21.09841214
15169 RPS12 1524.674659 0.104440828 0.102121925 1.012691542
0.211207519 25.44801708
15170 RPS18 1547.694226 0.11756512 0.102490459 1.147082654
0.251247049 17.07527155
15171 RPL19 1622.59405 0.014799652 0.100074228 0.147886598
0.882422265 29.04149512

15172	CD44	1540.627929	-0.142294022	0.124007222	-1.15622595	0.247542769	28.4702177
15173	ACTR2	1687.22008	-0.089502584	0.100855554	-0.88744228	0.274840291	21.47015249
15174	RPS8	1775.254062	0.145147221	0.098158854	1.478697288	0.129221228	19.99444444
15175	CYP1B1	1777.482722	-0.040205612	0.102448191	-0.289621247	0.696816628	20.97217042
15176	TUBB	2010.284157	-0.026290208	0.101224606	-0.259465104	0.719247179	22.05545197
15177	AL128962.4	2027.027515	-0.006416807	0.098908226	-0.064876212	0.948272482	51.20125104
15178	DDX5	1794.794549	0.061894618	0.108609841	0.569880287	0.568758828	18.19105542
15179	RPL4	1954.21251	0.117028486	0.094422264	1.22952082	0.215152682	24.74421194
15180	NEAT1	1820.7548	0.069822244	0.119285088	0.584858169	0.558642088	17.5154825
15181	AC006064.4	2171.584696	0.050297172	0.101295219	0.497026976	0.619162977	41.84249114
15182	TIMP1	2252.925229	-0.064989111	0.095279417	-0.681274595	0.495624494	27.52895702
15183	RPS4X	2212.552015	0.002760281	0.094824727	0.029651941	0.968270617	14.18211971
15184	ARHGDIB	2200.805595	0.012117942	0.092586571	0.140169074	0.888526406	11.54724084
15185	ENO1	2274.599294	0.105267884	0.092219824	1.129242527	0.258795112	27.41114914
15186	CAPG	2742.804468	0.006959294	0.095577281	0.072812192	0.941954772	21.91202724
15187	TMSB4X	2768.914612	0.012262678	0.09241489	0.122252227	0.89470562	27.89928214
15188	MT-CYB	2498.212254	0.022897626	0.097282646	0.225272254	0.812919819	21.1890029
15189	TPT1	2592.442215	0.027199214	0.085252242	0.218670172	0.749976624	21.49825477
15190	GAPDH	4490.790252	0.04885061	0.089109825	0.548206712	0.582549972	21.07404059
15191	LAPTM5	4576.284195	-0.012452746	0.086622018	-0.155297621	0.876586691	18.49074811
15192	PSAP	4898.280852	0.069152284	0.082241416	0.840846224	0.400424088	42.84014844
15193	MT-RNR1	6272.272121	-0.154202919	0.109870912	-1.40441102	0.160196491	18.07714445
15194	MT-CO1	9062.181078	-0.096972726	0.079892651	-1.212787804	0.224828765	18.14024981
15195	MT-RNR2	240410.0251	0.065882084	0.07075897	0.921091625	0.25180617	17.74484214
15196	PRKRIP1	22.24260287	-0.010082271	0.592855896	-0.017008121	0.986420129	29.54558019
15197	RUNX2	8.602989027	-0.018060176	1.06887466	-0.016896429		

0.986519224 27.81401448
15198 TGM5 2.980514025 0.029741625 1.825277075 0.016204645 0.98707112
17.54124201
15199 Z97055.2 2.482252257 0.021299448 1.962220002 0.015951029
0.987272452 50.71121577
15200 RPS2AP21 2.994428201 0.027590196 1.790242472 0.01541142
0.987702945 19.89775795
15201 SRRM5 2.000849115 0.022955268 2.124182021 0.015441679
0.987679812 20.45511159
15202 MRPL58 20.52051242 0.010228565 0.687569692 0.015026288 0.98800215
25.24419502
15203 AL122629.2 12.64722069 -0.012426265 0.808125272 -0.015276589
0.98772174 27.79119074
15204 VPS27B 12.64722069 -0.012426265 0.808125272 -0.015276589 0.98772174
29.02914179
15205 SFXN2 12.15452722 -0.012956208 0.820054626 -0.015799202
0.987294585 22.97044447
15206 ZNF12 18.56272764 0.009952798 0.662070505 0.015024248
0.988004778 21.41412447
15207 FAM224B 19.58689952 0.010016201 0.647720495 0.015462687
0.987662255 24.11170108
15208 FBXW7 19.58962267 0.010212701 0.648951622 0.015891225
0.987221091 40.44584978
15209 AL021595.2 17.58280919 0.011127592 0.690784259 0.01612211
0.987126176 21.27154571
15210 GRK6 22.61421922 0.009656627 0.64074822 0.015070858 0.98797565
22.71014819
15211 C1GALT1 45.72221542 0.006705082 0.427227092 0.015225117
0.987764826 28.081184
15212 TMEM159 50.46178956 -0.006264782 0.40642482 -0.015412992
0.9877019 21.50510417
15213 RAPGEF1 51.48621498 -0.00662689 0.401250187 -0.01651149
0.986826225 24.0101414
15214 RECQL 67.2417488 -0.006460241 0.287482528 -0.01667224
0.986698012 19.44481145
15215 PSMG2 126.6605862 0.004100577 0.27280828 0.01502098
0.988007465 21.47840947
15216 FUNDC2 106.4975862 -0.00449589 0.286697274 -0.015681659
0.987488259 18.17409241
15217 NDUFB2 121.2628915 0.004225108 0.261186616 0.016176587
0.987092514 29.41117147
15218 SND1 220.749204 -0.002816989 0.176528429 -0.015957709
0.987268121 44.28911591
15219 KANSL1L 18.57665191 0.009599859 0.659224798 0.014560126
0.988282102 21.92547005
15220 OXSM 22.15980804 0.007252257 0.496140941 0.014617522
0.988227211 24.77407004
15221 ZNF518A 26.78568517 -0.007922258 0.562507207 -0.014082824
0.988762097 19.42895245
15222 PEX14 19.57869708 0.009104299 0.650921914 0.012986715 0.98884058
28.99114114

15223	PPP2CB-AS1	17.05705251	0.009297049	0.692155222	0.012576502	0.989167851	21.11542507
15224	NPLOC4	66.24847876	0.004858221	0.26041207	0.012479897	0.989244924	18.20804771
15225	LRP1	111.2116401	0.002789941	0.279780204	0.012546122	0.98919208	44.17521472
15226	AD001527.2	11.62552942	-0.010876008	0.829276785	-0.012115051	0.989526002	21.45914959
15227	CMAHP	11.14949448	-0.011288024	0.88249951	-0.012904282	0.989704157	15.15980574
15228	NCBP2-AS1	18.58510789	0.008609754	0.661277167	0.012017919	0.989612497	40.21115144
15229	ABCB7	40.27575918	-0.005928522	0.455080165	-0.012049421	0.989588264	25.42947421
15230	DPY20	50.99201455	-0.005275021	0.406979542	-0.012961416	0.989658576	21.14204241
15231	LMF2	66.05842451	-0.004574144	0.258725826	-0.01275072	0.989826665	15.9942892
15232	AC007622.2	1.995280818	0.02690228	2.125897242	0.012595774	0.989950292	22.59871887
15233	SPTY2D1OS	17.05158421	0.00871798	0.700425142	0.012446519	0.990069271	20.14987829
15234	PIGC	68.62458606	-0.004265761	0.252281272	-0.012292822	0.990112211	19.10540447
15235	IGF2R	17.10155948	0.008584059	0.704242904	0.012187212	0.990276172	11.18274224
15236	NUDT19	104.7014609	0.002626127	0.296202104	0.012242069	0.990222486	11.12009101
15237	NEMF	147.2581502	0.002052625	0.252626099	0.012025967	0.990296919	48.74497484
15238	MELTF	12.61265021	-0.009494542	0.818612447	-0.011598222	0.990746085	48.71994174
15239	TMEM141	17.55820186	0.008111189	0.690100454	0.011752626	0.990622171	44.11118884
15240	TTK	12.14024951	-0.009461202	0.816484607	-0.011587852	0.990754428	44.70491075
15241	UBE2E2	17.06277424	0.007978029	0.692176827	0.01150927	0.990817054	17.47154425
15242	MRPS26	29.82726871	-0.005274202	0.466917755	-0.011510171	0.990816415	10.91015148
15243	PLEKHH1	11.12428007	-0.009749264	0.858927687	-0.011250274	0.990942906	15.1081418
15244	PATZ1	17.56929198	0.00740222	0.684297496	0.010815825	0.991270281	54.11414072
15245	AL451070.1	24.21255827	-0.006271142	0.587726105	-0.01084014	0.991250989	40.19818792
15246	STK4	152.0222754	0.002665899	0.241106095	0.011056952	0.991178008	14.41947827
15247	CAP1	1269.727228	-0.001452622	0.122459872	-0.010891826	0.991209744	48.99020748
15248	TICAM1	16.57852695	0.007072805	0.696414251	0.010156022		

0.991896798 24.48148242
15249 ZNF609 18.09266805 0.006822159 0.672249179 0.010149747
0.991901812 28.21850457
15250 C19orf54 24.72827826 -0.005792854 0.575821208 -0.010059984
0.991972429 17.15145122
15251 INVS 22.21220209 -0.006456229 0.641227214 -0.010068549
0.991966596 24.97547497
15252 USP54 15.06440584 0.0074157 0.744156997 0.009965226
0.992049024 18.79777122
15253 TBL1XR1-AS1 12.15059712 -0.007288921 0.797212406 -0.009268447
0.992604955 12.29492214
15254 TIAL1 22.15280164 0.00486285 0.512922441 0.009482622
0.992424075 24.12244885
15255 CTC1 71.10420427 -0.002196089 0.248798891 -0.009162128
0.992688984 27.84201548
15256 EMC4 151.1284661 -0.002281408 0.249422009 -0.009146274
0.992702251 19.89112415
15257 SRP54 140.6278197 -0.002299728 0.250201442 -0.009187825
0.992669271 24.44721714
15258 BMP1 25.67275722 0.004576226 0.506294718 0.009027074
0.992789556 21.90442024
15259 NSUN5 67.52481742 -0.002199194 0.258788297 -0.00891666
0.992885629 21.17820151
15260 KIF20A 29.26520228 -0.004795612 0.546159716 -0.008780605
0.992994181 21.72849141
15261 SUZ12 12.5994725 -0.006095768 0.812984575 -0.007498011
0.994017508 15.25002514
15262 AC087521.2 12.60220665 -0.005624072 0.806110916 -0.006989202
0.994422469 27.87404421
15263 AL118558.1 16.07490698 0.005800947 0.710496642 0.008164626
0.992485625 41.40401279
15264 PROSER2 16.07490698 0.005800947 0.710496642 0.008164626
0.992485625 41.07945884
15265 FRS2 12.12617171 -0.005951744 0.824499068 -0.007218618
0.994240426 28.4082475
15266 AC127024.5 15.08405194 0.005229584 0.724041407 0.007260605
0.994206927 41.78215284
15267 AL109811.1 15.08952024 0.006120217 0.740928869 0.008272692
0.992298624 27.8005954
15268 ZC2H4 29.2207964 -0.004408058 0.562750048 -0.007819171
0.992761268 58.72119584
15269 C2orf69 26.20620492 -0.004021422 0.562841049 -0.007149946
0.994295217 24.01187542
15270 RCC2-AS1 25.71251156 -0.004121599 0.565295114 -0.007208747
0.994168515 40.81248944
15271 CENPBD1 21.16492429 0.002645594 0.518110787 0.007026222
0.994285874 17.52282818
15272 ANGEL1 24.72812482 -0.004202495 0.582749454 -0.00727216 0.99411792
20.72294127
15273 PPFIA1 24.22996216 -0.004876612 0.592058225 -0.008222821
0.992429212 25.24244114

15274	HIKESHI	29.81567151	-0.00222926	0.457522468	-0.007057975	0.994268598	12.14259919
15275	PRPSAP1	46.29101259	0.002991614	0.421497121	0.007097591	0.994226989	41.10975402
15276	GOLGA5	68.02571224	-0.00291094	0.256470884	-0.008165996	0.99248455	21.48118504
15277	ZNF766	60.28172888	0.002724999	0.271042017	0.007244178	0.994140246	14.89141047
15278	TTC19	127.8521171	0.00202947	0.26962048	0.007562945	0.992964902	19.0595111
15279	YARS1	201.0014227	-0.001782276	0.222620964	-0.008005518	0.992612589	47.90141445
15280	SUMO2	227.8186551	-0.001605129	0.198627124	-0.008081167	0.992552222	21.44810821
15281	NDUFB8	16.57058802	0.002786666	0.727487924	0.005205126	0.995846929	14.19825782
15282	SYNGR1	15.61654459	-0.002549882	0.755025449	-0.004701672	0.996248622	22.24514115
15283	TMEM182A	14.61176528	-0.004071242	0.762584425	-0.005228875	0.995740214	19.90899814
15284	ATAD2B	14.07405787	0.001912114	0.771796912	0.002478779	0.998022222	15.19914911
15285	AC008440.1	14.58240966	0.001810991	0.748902412	0.002418192	0.998070562	41.99410748
15286	ZNF629	14.5664977	0.004256981	0.758829852	0.00574171	0.995418802	17.54114149
15287	AL258790.1	12.11702674	-0.004528468	0.780745205	-0.005812995	0.995261927	24.79744887
15288	GHRL	12.5812645	0.001940949	0.781195401	0.002484589	0.998017587	14.51252844
15289	LARP1B	17.08540812	0.004288162	0.698722909	0.006280252	0.994989116	20.11714481
15290	NECTIN1	12.10857076	-0.00215064	0.782527551	-0.004026184	0.996787578	19.52125589
15291	RPL5P24	12.10279145	-0.002894198	0.825702528	-0.004716219	0.996227016	21.4004405
15292	ERCC4	14.571966	0.005148624	0.752608999	0.00682197	0.994548919	12.78770107
15293	ZNF248	14.12299618	-0.00464957	0.759279461	-0.006122854	0.9951147	27.55019711
15294	ZNF708	12.11976089	-0.004092719	0.781890547	-0.005224289	0.995822581	18.54412571
15295	ZFP64	16.10200907	0.002754891	0.708979069	0.002885715	0.996899656	28.27779455
15296	CTPS2	14.14692046	-0.005145712	0.769012088	-0.00669122	0.994661129	24.17874409
15297	DHX24	15.1009629	0.002901075	0.729602929	0.002976228	0.996827429	45.58495051
15298	HELLPAR	15.1009629	0.002901075	0.729602929	0.002976228	0.996827429	22.17114942
15299	USP46	15.09524207	0.004502855	0.722656465	0.006128916		

0.995101885	29.59421498
15300	CBR4	27.65815651	0.002190811	0.559215024	0.002916952
0.996874722	28.11719804
15301	ERCC8	26.66182218	0.001295702	0.574202871	0.002420674
0.998060605	14.50080542
15302	RFX7	25.18426012	-0.001450254	0.588289876	-0.002464955
0.998022252	22.559819
15303	AC072254.1	29.15527566	0.002504126	0.529242255	0.006497054
0.994816128	41.15144011
15304	KIAA1586	29.22968191	-0.00158129	0.520778184	-0.00297928
0.997622802	20.51141708
15305	TMEM124	29.20560447	0.002167259	0.521147571	0.004080222
0.996744275	44.41542514
15306	HAUS6	21.22754225	0.001606192	0.518779406	0.0020961
0.997529674	17.54491497
15307	MTERF2	24.26580556	0.001604246	0.520602581	0.002081704	0.99754116
42.11554144
15308	C12orf4	42.29179498	0.001124241	0.44440292	0.002529997
0.997981257	20.04459417
15309	ZSWIM8	29.84027885	-0.001878228	0.461697068	-0.004068095
0.996754129	14.71449492
15310	SNRPA	45.20587929	0.002240727	0.426078548	0.005258952
0.995802982	42.71101891
15311	BBX	61.88244278	0.001662904	0.275420928	0.004422102
0.996462705	19.47191272
15312	POLD2	52.92422626	-0.002145299	0.292918586	-0.005459907
0.995642646	27.02780874
15313	TCTN2	57.88545225	0.001587505	0.280269205	0.004172591
0.996669966	27.94742189
15314	AC009620.2	57.29848071	0.001169712	0.281682016	0.002064627
0.997554786	22.71427417
15315	GSPT1	57.87972042	0.002012406	0.281517105	0.005274747	0.99579128
22.72490884
15316	RPAP1	54.95542056	-0.001246606	0.292179796	-0.002422644
0.997260254	27.101558
15317	PRRC1	52.97874224	-0.002292076	0.298812929	-0.005997976
0.995214226	18.74944891
15318	LMAN1	50.96688601	-0.001849402	0.428425904	-0.00421818
0.996624289	24.94119811
15319	PHAX	66.54165777	-0.002067542	0.258184471	-0.005772286
0.995294408	14.41141412
15320	INTS4	69.98252187	0.001017751	0.25169718	0.002892828
0.997691062	21.11471521
15321	ERAL1	75.01860856	0.001149421	0.224720672	0.002422897
0.997260152	14.20401851
15322	MBD1	76.12161841	-0.000915466	0.246216189	-0.00264244
0.997890842	24.11471447
15323	MRPS10	102.7181025	0.000802685	0.287476158	0.002792178
0.997772167	18.85124255
15324	NAA25	106.7905772	-0.000852822	0.294795922	-0.00289296
0.997691755	24.12252808

15325	CDC22	120.9221478	-0.001270568	0.282612929	-0.004495772	0.996412905	44.41442914
15326	SLC25A28	122.8717122	-0.000791026	0.275056956	-0.0028759	0.997705267	24.4118591
15327	VPS25	128.2517859	0.001492979	0.275821994	0.005416266	0.995678466	18.51459105
15328	MCM7	148.9605892	0.000784152	0.252042075	0.002098902	0.997527427	27.55182121
15329	ADCY7	162.8272105	-0.001412454	0.240086648	-0.005887265	0.995202669	17.2184051
15330	CDC122	187.2944225	0.000717172	0.220246986	0.002256221	0.997401916	45.0112209
15331	TMEM220	259.2487221	-0.000842841	0.198891805	-0.004242712	0.996614815	19.95982414
15332	CCNG1	227.2100258	0.000887164	0.171992292	0.00515812	0.995884426	21.12045105
15333	AOPEP	15.10941988	0.001692754	0.729849702	0.002220689	0.998148259	21.41805981
15334	ZCCHC24	12.08245626	-0.001660552	0.801554454	-0.002071664	0.998247052	29.88072752
15335	SLC28A9	28.70248157	-0.001145218	0.527977598	-0.002128746	0.998201508	24.78142954
15336	ZNF260	14.1048941	-0.001178042	0.754222029	-0.001561929	0.998752761	17.58745954
15337	MCEE	12.59622129	-0.000820206	0.814474855	-0.001007159	0.999196404	45.42205118
15338	EPC2	14.12155252	-0.001227124	0.757569914	-0.001619829	0.998707564	12.04171447
15339	PDE4D	12.61015557	-0.001224442	0.818582966	-0.00161797	0.998709047	24.82499254
15340	ZNF472	15.12061001	0.000867782	0.724289179	0.001181627	0.99905719	15.59744814
15341	MAN1A2	26.72991054	-0.000545871	0.579048202	-0.000942702	0.999247822	20.17425855
15342	ERH	40.788854	0.000755812	0.459019559	0.001646581	0.998686219	29.48888511
15343	UBAC1	41.21486422	0.00071685	0.45649769	0.001570226	0.998747061	22.15980804
15344	DST	54.91214067	0.000259905	0.296911288	0.000906765	0.999276506	88.01448211
15345	SIGLEC9	62.4970215	0.00041049	0.280581166	0.001078587	0.999129412	71.84291774
15346	STK26	177.295292	0.000208189	0.22292694	0.001222114	0.998944208	79.05215484
15347	SRP14	591.8491989	-0.000191998	0.126041229	-0.001411221	0.998872929	49.81148044
15348	YWHAZ	1629.002514	0.000140162	0.100422212	0.001295579	0.998886489	14.12591184
15349	FXR1	195.4811262	-0.000162462	0.221616216	-0.000727591	0.999411488	18.28148411
15350	JAK2	14.11582069	0.000488466	0.762082249	0.000640961		

0.999488587	47.70457519
15351	USP29	129.4242482	-0.000126945	0.259200472	-0.000489756
0.999609221	51.19848417
15352	ZNF718	12.58708622	0.00014072	0.776512249	0.000181221
0.999855407	48.71915007
15353	CHMP5	122.2609118	7.14E-05	0.275112766	0.000259605
0.999792865	15.91210824
15354	PCNA	98.68625697	1.74E-05	0.297612952	5.84E-05
0.999952287	21.42114291
15355	AL252762.1	0.500997014	2.484998858	4.296126822	0.565270056	-
0.021650222	44.81748209
15356	AC006547.4	0.49522622	-2.415608216	4.416671772	-0.546929552
0.608422491	51.71449517
15357	AC025627.1	1.001994028	2.484976762	2.227807229	1.044091647	-
0.092059923	10.19485804
15358	AL022400.1	0.486870242	-2.292502217	4.077744474	-0.586967215	-
0.694448592	11.40017111
15359	AC018607.1	1.466281719	-0.945971625	2.427524021	-0.288085509
0.22222272	55.88040571
15360	AL252801.2	0.498161667	0.02169065	4.170279829	0.007599166
2.08261218	19.28820181
15361	AC125050.2	0.740204196	1.049207872	2.521140276	0.297158266
2.082092522	15.8451101
15362	AL022069.2	0.990652641	-2.415742141	2.252204524	-1.018619724
0.029744879	22.15570814
15363	AC252526.6	0.726077524	-2.971191046	2.466857824	-0.85702708	-
0.126227209	44.11584011
15364	AL445522.1	0.500997014	2.484998858	4.296126822	0.565270056	-
0.016592287	17.98974905
15365	AL290067.1	0.751495521	2.069950829	2.726554747	0.822804022	-
0.942926564	24.28792409
15366	AC122961.1	0.500997014	2.484998858	4.296126822	0.565270056	-
0.766700002	24.57124718
15367	AL125905.1	0.500997014	2.484998858	4.296126822	0.565270056
0.22820866	10.14040141
15368	AL021292.1	0.486870242	-2.292502217	4.077744474	-0.586967215
0.075524796	40.54711915
15369	AC107021.1	0.72891287	-0.92219097	2.55162759	-0.26246867	-
0.401408259	28.24948948
15370	AL258472.1	1.227074528	-0.524749642	2.612295646	-0.204704871
0.042292763	11.21708997
15371	AC102592.1	0.49522622	-2.415608216	4.416671772	-0.546929552	-
1.225246896	11.84499894
15372	AC009090.1	0.49522622	-2.415608216	4.416671772	-0.546929552	-
0.900060121	18.50747777
15373	AL265258.1	0.478414262	-2.271055522	4.479204761	-0.529247421	-
0.245818785	50.41485418
15374	AC018868.2	0.965284705	-2.282548611	2.115847245	-1.0855951	-
0.904927478	48.47005797
15375	AL445985.1	0.500997014	2.484998858	4.296126822	0.565270056	-
0.899882562	47.48511411

15376 AC040162.2 0.745824827 −0.972210916 2.522016256 −0.276242662
0.094121579 27.87101744
15377 AC127459.2 0.740204196 1.049207872 2.521140276 0.297158266 −
1.022220217 45.71024511
15378 AC027228.1 0.498161667 0.02169065 4.170279829 0.007599166
2.08202292 14.21277475
15379 AL125960.1 1.477572045 0.049609785 2.411676228 0.020570665 −
0.211227027 21.75842785
15380 AL445222.1 0.982196662 −2.404284457 2.098256189 −1.098806625
0.599077986 51.70285579
15381 AC111170.1 0.49522622 −2.415608216 4.416671772 −0.546929552 −
0.899864014 24.2548454
15382 AC117490.2 0.726077524 −2.971191046 2.466857824 −0.85702708 −
0.58069797 19.52110442
15383 AC006116.4 0.486870242 −2.292502217 4.077744474 −0.586967215
0.887944945 25.45085147
15384 AC114797.1 0.745824827 −0.972210916 2.522016256 −0.276242662 −
0.680942721 49.11719444
15385 ADCY5 0.727268849 −0.952727871 2.205072418 −0.288262241 −
0.771842422 11.28717221
15386 AL021846.2 1.452155047 −2.971182499 2.669514742 −1.487604622 −
0.257422195 24.01029544
15387 AC008429.2 0.500997014 2.484998858 4.296126822 0.565270056
0.015980893 21.44740241
15388 AL512282.1 0.717621545 −2.955950852 2.792242589 −0.779267242 −
0.12528689 47.17150014
15389 AL592427.1 0.489705689 0.05402189 4.196172952 0.012876469
2.079408112 41.71729871
15390 AC090282.1 1.001994028 2.484976762 2.227807229 1.044091647 −
0.471522813 49.42127009
15391 AC111182.1 0.724522502 −2.98570617 2.452708589 −0.86474217
0.615249187 11.41527448
15392 AC122912.2 0.478414262 −2.271055522 4.479204761 −0.529247421
0.12471226 50.51701142
15393 AC072262.1 0.500997014 2.484998858 4.296126822 0.565270056
0.451977165 28.20704199
15394 AL049646.1 0.486870242 −2.292502217 4.077744474 −0.586967215
0.116228253 44.48824514
15395 AL265218.1 0.49522622 −2.415608216 4.416671772 −0.546929552 −
0.025656972 41.40815411
15396 AC109454.2 1.001994028 2.484976762 2.227807229 1.044091647
0.022221685 21.47929858
15397 AL260182.1 0.478414262 −2.271055522 4.479204761 −0.529247421
2.082226705 50.41008714
15398 AL129148.1 0.486870242 −2.292502217 4.077744474 −0.586967215
0.520829421 24.12455017
15399 AP002815.1 0.489705689 0.05402189 4.196172952 0.012876469
0.256287702 24.12229747
15400 AL255877.1 0.500997014 2.484998858 4.296126822 0.565270056
2.082091659 19.45017981
15401 AP000641.1 0.500997014 2.484998858 4.296126822 0.565270056

0.06421811 24.95279245
15402 AC022447.1 0.49522622 -2.415608216 4.416671772 -0.546929552 -
0.029896068 49.04099141
15403 AC021087.2 0.498161667 0.02169065 4.170279829 0.007599166 -
1.198968993 42.15104944
15404 AC046185.2 1.480408291 1.060697984 2.618861268 0.405022594 -
0.149997077 28.10911145
15405 AC244100.2 0.500997014 2.484998858 4.296126822 0.565270056 -
0.25451589 21.01171144
15406 AC090206.1 0.486870242 -2.292502217 4.077744474 -0.586967215
0.070278845 48.00019971
15407 AF121215.5 1.486029022 0.026902592 2.40726188 0.015220111
0.622652012 21.18019547
15408 AL259644.1 0.478414262 -2.271055522 4.479204761 -0.529247421
0.622809075 41.57498409
15409 AL512188.1 0.489705689 0.05402189 4.196172952 0.012876469 -
0.894120084 18.09494154
15410 AL671277.2 0.727268849 -0.952727871 2.205072418 -0.288262241 -
0.284019522 20.8104405
15411 APCDD1L 1.215782212 -1.942095285 2.757929924 -0.704182251
0.009666202 22.92471048
15412 AC099242.4 1.98419069 0.022486062 2.129406254 0.015652025
0.767468255 25.28404019
15413 AL126028.1 1.225520516 -0.54785862 2.606584125 -0.2101826 -
0.112986609 22.74151841
15414 AL252746.1 0.486870242 -2.292502217 4.077744474 -0.586967215
0.162906206 20.71111844
15415 AC100774.1 0.500997014 2.484998858 4.296126822 0.565270056 -
1.252575991 20.99191578
15416 AL265215.1 0.478414262 -2.271055522 4.479204761 -0.529247421 -
0.921740783 14.20849527
15417 ARHGEF9-IT1 0.956828727 -2.270922824 2.294506224 -0.992055722 -
0.127102102 28.54054402
15418 AC241520.1 0.992487988 -1.558611578 2.168280519 -0.491926891 -
0.919226083 21.15571879
15419 ANXA10 0.727268849 -0.952727871 2.205072418 -0.288262241 -
0.921521282 21.18559404
15420 AC084024.1 0.987867256 0.042250627 2.875240277 0.015077222 -
0.114261415 21.8412017
15421 ARHGAP42 0.972740684 -2.29250678 2.080064802 -1.101764725 -
0.172829912 45.44721411
15422 AC008507.1 0.740204196 1.049207872 2.521140276 0.297158266
1.022155295 27.82992895
15423 ALDH8A1 1.725226205 -0.271567727 2.225226245 -0.166221971 -
0.201911426 40.18184474
15424 AC106895.2 0.478414262 -2.271055522 4.479204761 -0.529247421
0.25711209 17.59874981
15425 ARL4D 0.985022009 -1.54422206 2.962499952 -0.52129252
0.006625046 45.05842454
15426 AC006511.2 0.478414262 -2.271055522 4.479204761 -0.529247421 -
0.044896167 25.58174712

15427	AC022211.4	0.742989481	-2.000642057	2.742921251	-0.801470644	0.126170293	21.71147847
15428	AL122215.2	0.72891287	-0.92219097	2.55162759	-0.26246867	0.14190224	24.09405204
15429	AL122255.1	1.207227224	-1.921069226	2.961428148	-0.65207144	-0.088594465	18.22411844
15430	AL260270.2	1.466281719	-0.945971625	2.427524021	-0.288085509	-0.74108595	57.99114045
15431	AC004461.2	1.24120121	2.048222644	2.924224822	0.698079991	-0.772124223	18.04212742
15432	AC004696.2	0.498161667	0.02169065	4.170279829	0.007599166	0.529947661	28.17401224
15433	AC005200.1	0.478414262	-2.271055522	4.479204761	-0.529247421	-0.966920566	15.81171748
15434	AC005292.2	0.486870242	-2.292502217	4.077744474	-0.586967215	-0.242447573	20.91785199
15435	AC005520.7	0.727268849	-0.952727871	2.205072418	-0.288262241	-0.788990127	17.87411057
15436	AC005606.2	1.48886427	1.042871104	2.462228197	0.422547706	-0.177691252	22.91487204
15437	AC005828.1	0.500997014	2.484998858	4.296126822	0.565270056	-0.158996063	10.18554471
15438	AC005920.1	0.965284705	-2.282548611	2.115847245	-1.0855951	-0.256256448	41.71444594
15439	AC006486.2	1.252492525	2.806892422	2.079827549	1.226069228	0.529852281	17.91415018
15440	AC006557.2	1.720856827	-1.290122295	2.299421296	-0.561068275	-0.202285299	24.24151594
15441	AC007785.2	0.717621545	-2.955950852	2.792242589	-0.779267242	-0.021640957	15.22580717
15442	AC007954.1	0.727268849	-0.952727871	2.205072418	-0.288262241	-0.249184295	40.84202542
15443	AC008055.1	0.498161667	0.02169065	4.170279829	0.007599166	-0.210047445	21.09244152
15444	AC008897.2	0.72891287	-0.92219097	2.55162759	-0.26246867	-0.021770451	21.12754225
15445	AC009065.7	0.745824827	-0.972210916	2.522016256	-0.276242662	-0.220675228	12.59171887
15446	AC009209.2	1.722400858	-1.281819192	2.265210278	-0.565872029	0.082220998	10.58190144
15447	AC009652.1	0.748660174	1.029647608	2.51687248	0.292772655	0.046518626	41.89018088
15448	AC010609.1	0.742989481	-2.000642057	2.742921251	-0.801470644	0.442702223	44.14574184
15449	AC010824.2	0.498161667	0.02169065	4.170279829	0.007599166	-0.252425415	45.88189482
15450	AC011472.2	0.748660174	1.029647608	2.51687248	0.292772655	0.208066618	50.17194051
15451	AC011726.2	0.726077524	-2.971191046	2.466857824	-0.85702708	0.271709711	11.9841144
15452	AC011912.1	0.489705689	0.05402189	4.196172952	0.012876469		

						0.189406675	19.52841717
15453	AC011922.1	0.726077524	-2.971191046	2.466857824	-0.85702708	0.296698077	55.47144004
15454	AC012062.1	1.462446272	-2.270129994	2.569759491	-0.882401726	0.168200529	15.42425541
15455	AC012279.1	1.228265862	0.628005757	2.620292458	0.228758818	-0.12022747	57.10299794
15456	AC015852.2	0.478414262	-2.271055522	4.479204761	-0.529247421	0.615047288	47.81928715
15457	AC016245.1	0.486870242	-2.292502217	4.077744474	-0.586967215	-0.052647517	14.12520127
15458	AC016644.1	0.748660174	1.029647608	2.51687248	0.292772655	-0.225170606	57.49511544
15459	AC018809.1	1.462446272	-2.270129994	2.569759491	-0.882401726	-0.225770892	24.24498194
15460	AC019069.1	0.72891287	-0.92219097	2.55162759	-0.26246867	0.752271402	20.21458751
15461	AC021087.4	0.49522622	-2.415608216	4.416671772	-0.546929552	-0.771281465	44.22071211
15462	AC022415.1	0.49522622	-2.415608216	4.416671772	-0.546929552	0.508662267	42.19718242
15463	AC025171.2	1.955987408	-0.681200827	2.155186289	-0.216075129	0.141584122	21.89845712
15464	AC026412.2	0.724522502	-2.98570617	2.452708589	-0.86474217	-0.286297202	21.88741587
15465	AC026786.2	0.486870242	-2.292502217	4.077744474	-0.586967215	0.508761196	40.70154289
15466	AC027279.2	0.498161667	0.02169065	4.170279829	0.007599166	0.069272876	21.4417214
15467	AC027207.1	0.724522502	-2.98570617	2.452708589	-0.86474217	0.292850251	25.15044208
15468	AC027762.1	1.491699717	2.269907102	2.766792015	0.856552811	0.588606184	44.72514104
15469	AC026222.4	0.49522622	-2.415608216	4.416671772	-0.546929552	0.110446016	15.78401718
15470	AC040169.2	0.489705689	0.05402189	4.196172952	0.012876469	-0.18959948	22.28185081
15471	AC048251.2	0.486870242	-2.292502217	4.077744474	-0.586967215	-0.00044265	27.84177211
15472	AC051619.7	0.740204196	1.049207872	2.521140276	0.297158266	-0.155646595	17.51124454
15473	AC060766.7	1.242986495	-0.56192924	2.798025286	-0.200824261	0.55082757	28.74598047
15474	AC064799.1	0.985022009	-1.54422206	2.962499952	-0.52129252	-0.899884218	21.18554191
15475	AC066616.2	0.745824827	-0.972210916	2.522016256	-0.276242662	-0.077029097	25.22782811
15476	AC067828.1	1.242986495	-0.56192924	2.798025286	-0.200824261	0.002241263	19.07045109
15477	AC068299.2	0.500997014	2.484998858	4.296126822	0.565270056	0.044844594	19.70071721

15478	AC068289.2	0.478414262	-2.271055522	4.479204761	-0.529247421	-0.228887292	11.84011855
15479	AC069200.2	0.489705689	0.05402189	4.196172952	0.012876469	-0.928495246	21.89212441
15480	AC069549.1	0.498161667	0.02169065	4.170279829	0.007599166	0.125411249	21.88278914
15481	AC072120.2	0.72891287	-0.92219097	2.55162759	-0.26246867	-1.412554251	29.40111089
15482	AC078886.1	0.727268849	-0.952727871	2.205072418	-0.288262241	0.020912978	19.79100784
15483	AC079209.1	0.500997014	2.484998858	4.296126822	0.565270056	0.220812223	55.2540297
15484	AC079226.2	0.498161667	0.02169065	4.170279829	0.007599166	-6.77E-06	42.79141007
15485	AC084026.2	0.500997014	2.484998858	4.296126822	0.565270056	0.728167173	21.88144505
15486	AC087071.1	0.724522502	-2.98570617	2.452708589	-0.86474217	-0.220707127	45.81244001
15487	AC087269.1	0.486870242	-2.292502217	4.077744474	-0.586967215	-0.012026693	21.01081741
15488	AC087622.2	0.486870242	-2.292502217	4.077744474	-0.586967215	0.182970173	48.48111084
15489	AC087741.2	0.500997014	2.484998858	4.296126822	0.565270056	-0.510022646	29.145799
15490	AC090124.1	0.500997014	2.484998858	4.296126822	0.565270056	-0.107915082	45.14581825
15491	AC091488.1	0.489705689	0.05402189	4.196172952	0.012876469	0.129626277	14.84574749
15492	AC091982.1	0.49522622	-2.415608216	4.416671772	-0.546929552	-0.092026754	40.94081144
15493	AC092045.1	0.489705689	0.05402189	4.196172952	0.012876469	-0.070272161	21.91222292
15494	AC092125.1	0.498161667	0.02169065	4.170279829	0.007599166	-0.061289209	21.88451449
15495	AC092284.2	1.227074528	-0.524749642	2.612295646	-0.204704871	-0.122959752	25.29150954
15496	AC092427.1	0.49522622	-2.415608216	4.416671772	-0.546929552	-0.219572441	25.4244148
15497	AC092474.1	0.478414262	-2.271055522	4.479204761	-0.529247421	0.509129879	21.42914888
15498	AC092525.2	0.49522622	-2.415608216	4.416671772	-0.546929552	-0.162468826	28.44512947
15499	AC092667.1	0.486870242	-2.292502217	4.077744474	-0.586967215	-0.251164694	22.7594292
15500	AC092762.2	0.748660174	1.029647608	2.51687248	0.292772655	-0.182881716	21.10450245
15501	AC092909.1	0.498161667	0.02169065	4.170279829	0.007599166	0.521265775	21.91974581
15502	AC096564.1	1.744982508	0.428555102	2.429124696	0.18052964	0.085120279	44.49211954
15503	AC097478.2	0.500997014	2.484998858	4.296126822	0.565270056	-	

						0.221252884	48.12484841
15504	AC099226.1	0.745824827	−0.972210916	2.522016256	−0.276242662	0.025612687	42.19424818
15505	AC099494.2	0.500997014	2.484998858	4.296126822	0.565270056	−0.254557941	21.11705278
15506	AC099509.2	0.500997014	2.484998858	4.296126822	0.565270056	0.02222981	24.41444109
15507	AC099518.4	0.727268849	−0.952727871	2.205072418	−0.288262241	−0.282292872	45.49784844
15508	AC100827.4	0.478414262	−2.271055522	4.479204761	−0.529247421	0.668812275	24.41200194
15509	AC102726.2	0.500997014	2.484998858	4.296126822	0.565270056	0.004029909	18.42410811
15510	AC104170.1	1.964292225	−4.404422066	2.422524009	−1.810628682	−0.149285212	17.7540812
15511	AC104451.1	0.751495521	2.069950829	2.726554747	0.822804022	−0.122827181	29.24849949
15512	AC104794.5	0.498161667	0.02169065	4.170279829	0.007599166	0.074506052	55.18057099
15513	AC104806.2	0.486870242	−2.292502217	4.077744474	−0.586967215	0.026927723	20.11719129
15514	AC105229.5	0.49522622	−2.415608216	4.416671772	−0.546929552	2.082567509	19.78155188
15515	AC106872.2	0.49522622	−2.415608216	4.416671772	−0.546929552	−0.189827106	40.00115849
15516	AC108102.1	0.726077524	−2.971191046	2.466857824	−0.85702708	0.521118968	18.00451974
15517	AC109247.1	0.740204196	1.049207872	2.521140276	0.297158266	0.418144053	17.8211851
15518	AC109446.4	1.222695169	−1.962816786	2.747589728	−0.714277681	−0.942264228	24.85442857
15519	AC112198.1	0.500997014	2.484998858	4.296126822	0.565270056	0.124280449	29.94449911
15520	AC112206.2	0.724522502	−2.98570617	2.452708589	−0.86474217	−0.21077982	28.40888209
15521	AC112220.2	0.500997014	2.484998858	4.296126822	0.565270056	0.080155222	19.78140194
15522	AC112208.4	0.49522622	−2.415608216	4.416671772	−0.546929552	0.584558292	14.95448501
15523	AC114727.2	0.500997014	2.484998858	4.296126822	0.565270056	−0.201994775	24.19158814
15524	AC114878.2	0.500997014	2.484998858	4.296126822	0.565270056	−0.210994042	44.01012712
15525	AC117282.2	0.745824827	−0.972210916	2.522016256	−0.276242662	−0.572592279	44.49879011
15526	AC117824.1	0.498161667	0.02169065	4.170279829	0.007599166	−0.102282016	41.77712057
15527	AC122912.1	0.478414262	−2.271055522	4.479204761	−0.529247421	−0.512262203	21.14191011
15528	AC124016.2	1.497220248	1.024785281	2.607514715	0.292012272	−0.182869729	19.04502185

15529 AC126544.1 0.49522622 -2.415608216 4.416671772 -0.546929552 -
0.52775099 22.48597059
15530 AC127502.2 0.500997014 2.484998858 4.296126822 0.565270056 -
0.149465977 19.58000294
15531 AC120271.1 1.227074528 -0.524749642 2.612295646 -0.204704871 -
0.210244276 24.44025151
15532 AC120456.5 1.952152061 -1.522665224 2.16241486 -0.708909442 -
0.155621402 19.95474422
15533 AC122552.2 0.478414262 -2.271055522 4.479204761 -0.529247421
1.007126047 40.14418442
15534 AC125507.2 0.992487988 -1.558611578 2.168280519 -0.491926891
0.415476792 14.74144211
15535 AC125776.4 0.498161667 0.02169065 4.170279829 0.007599166
0.021070081 17.1821275
15536 AC127922.2 0.982196662 -2.404284457 2.098256189 -1.098806625
0.052192213 14.72991054
15537 AC128207.8 0.745824827 -0.972210916 2.522016256 -0.276242662
0.199559279 15.11822978
15538 AC128292.2 0.486870242 -2.292502217 4.077744474 -0.586967215 -
0.569672426 42.84072951
15539 AC128420.2 0.726077524 -2.971191046 2.466857824 -0.85702708 -
0.942681015 55.12124901
15540 AC129256.2 0.726077524 -2.971191046 2.466857824 -0.85702708
0.112788805 50.18751172
15541 AC141424.1 0.500997014 2.484998858 4.296126822 0.565270056 -
0.112275249 48.71899772
15542 AC145207.9 0.478414262 -2.271055522 4.479204761 -0.529247421
0.598905462 52.72124251
15543 AC224782.2 1.222695169 -1.962816786 2.747589728 -0.714277681 -
0.014122673 54.98590842
15544 AC229809.2 0.478414262 -2.271055522 4.479204761 -0.529247421 -
0.212746157 22.08492191
15545 AC245052.2 0.489705689 0.05402189 4.196172952 0.012876469 -
0.90806242 11.27757228
15546 AC246792.1 0.49522622 -2.415608216 4.416671772 -0.546929552
0.577901743 25.54012127
15547 AC252526.2 0.498161667 0.02169065 4.170279829 0.007599166
0.188754861 24.50514192
15548 ACTL8 1.229859822 -2.728517982 2.865702421 -1.201082442
0.062964712 19.14170424
15549 ADAMTS16 0.49522622 -2.415608216 4.416671772 -0.546929552 -
1.22222272 28.82899524
15550 ADAMTSL5 0.965284705 -2.282548611 2.115847245 -1.0855951
0.242024662 40.79774805
15551 ADM2 0.976576021 -1.520755908 2.970112194 -0.515286561
0.228820872 25.49019504
15552 ADPRHL1 0.478414262 -2.271055522 4.479204761 -0.529247421
0.154024429 24.82799184
15553 AK1 1.454990294 -2.261695949 2.608422085 -0.867074042 -
0.164242821 57.14101528
15554 AKR1C8P 0.500997014 2.484998858 4.296126822 0.565270056

0.22666988 24.47944481
15555 AL025412.1 0.49522622 -2.415608216 4.416671772 -0.546929552 -
0.966599172 24.59405042
15556 AL049840.4 1.228265862 0.628005757 2.620292458 0.228758818 -
0.09127476 24.4145449
15557 AL109806.1 0.500997014 2.484998858 4.296126822 0.565270056 -
0.251082592 21.27124947
15558 AL117250.1 0.486870242 -2.292502217 4.077744474 -0.586967215
0.042859861 27.49411119
15559 AL122001.1 0.489705689 0.05402189 4.196172952 0.012876469 -
0.499229641 27.22410444
15560 AL122268.1 0.717621545 -2.955950852 2.792242589 -0.779267242
0.494241078 29.05541989
15561 AL122485.2 0.956828727 -2.270922824 2.294506224 -0.992055722
0.028719152 19.15041504
15562 AL129112.1 0.982196662 -2.404284457 2.098256189 -1.098806625
0.507952146 18.20109125
15563 AL129292.2 0.727268849 -0.952727871 2.205072418 -0.288262241 -
0.226840201 25.514917
15564 AL161757.2 0.726077524 -2.971191046 2.466857824 -0.85702708 -
0.019225452 28.11991991
15565 AL162285.2 0.486870242 -2.292502217 4.077744474 -0.586967215 -
0.016141045 48.42574412
15566 AL162720.1 0.489705689 0.05402189 4.196172952 0.012876469
0.074774167 25.71804072
15567 AL252582.1 1.212947866 -2.711072826 2.82980298 -1.206805984
0.140785276 28.59490177
15568 AL252600.1 0.745824827 -0.972210916 2.522016256 -0.276242662 -
0.178209083 25.57499414
15569 AL252742.1 0.49522622 -2.415608216 4.416671772 -0.546929552 -
0.687622285 24.80194415
15570 AL252748.1 0.965284705 -2.282548611 2.115847245 -1.0855951
0.057556295 21.21004844
15571 AL252807.1 0.489705689 0.05402189 4.196172952 0.012876469
0.52959446 40.51904414
15572 AL255075.4 0.751495521 2.069950829 2.726554747 0.822804022 -
0.215921122 51.24844514
15573 AL256022.1 0.500997014 2.484998858 4.296126822 0.565270056
2.082129907 40.79119748
15574 AL256525.1 0.727268849 -0.952727871 2.205072418 -0.288262241 -
0.494282671 25.44747744
15575 AL257152.2 0.49522622 -2.415608216 4.416671772 -0.546929552 -
0.149699255 22.15940421
15576 AL258472.1 0.486870242 -2.292502217 4.077744474 -0.586967215
0.965769283 40.70758744
15577 AL259217.2 1.246821841 0.609177462 2.794582528 0.217985129
0.272647446 24.72119402
15578 AL259878.1 1.719615572 0.477264708 2.441096672 0.195512416 -
0.189554963 27.50815941
15579 AL259918.2 0.498161667 0.02169065 4.170279829 0.007599166 -
0.212921577 14.18119144

15580	AL259921.2	0.486870242	-2.292502217	4.077744474	-0.586967215	-0.988829847	40.44894154
15581	AL265258.2	1.975724712	0.042192957	2.116112947	0.020411461	0.452282795	41.85821184
15582	AL265295.1	0.49522622	-2.415608216	4.416671772	-0.546929552	0.182058578	17.00442451
15583	AL291262.1	0.478414262	-2.271055522	4.479204761	-0.529247421	-0.270221427	25.41115954
15584	AL291704.1	0.498161667	0.02169065	4.170279829	0.007599166	0.126722778	27.71975877
15585	AL441882.1	0.740204196	1.049207872	2.521140276	0.297158266	0.159692059	21.11109709
15586	AL442067.2	0.489705689	0.05402189	4.196172952	0.012876469	0.290520961	29.54780811
15587	AL442125.2	0.478414262	-2.271055522	4.479204761	-0.529247421	0.109125455	42.47111481
