# Supplementary material for: TAFA4-IL-10 axis potentiate immunotherapy for airway allergy by induction of specific regulatory T cells
Source: NPJ Vaccines. 2022 Oct 31;7:133. doi: 10.1038/s41541-022-00559-w (PMC9622679; doi:10.1038/s41541-022-00559-w)

Supplementary materials

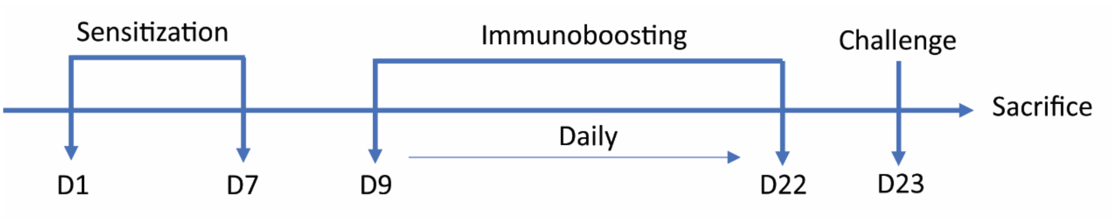

Supplementary Figure 1. A schematic of a murine AR model development. Sensitization: Mice were sensitized by subcutaneous injection of ovalbumin (OVA, 100 µg/mouse, mixed in 0.1 ml Alum) in the back skin on day 1 and day 7, respectively. Immunoboosting: Mice were boosted by nasal instillation (20 µl/nostril, 5 mg/ml) daily from day 9 to day 22. Challenge: On day 23, mice were challenged with the specific antigen (OVA) by nasal instillation (20 µl/nostril, 50 mg/ml). Sacrifice: Mice were sacrificed by the cervical dislocation.

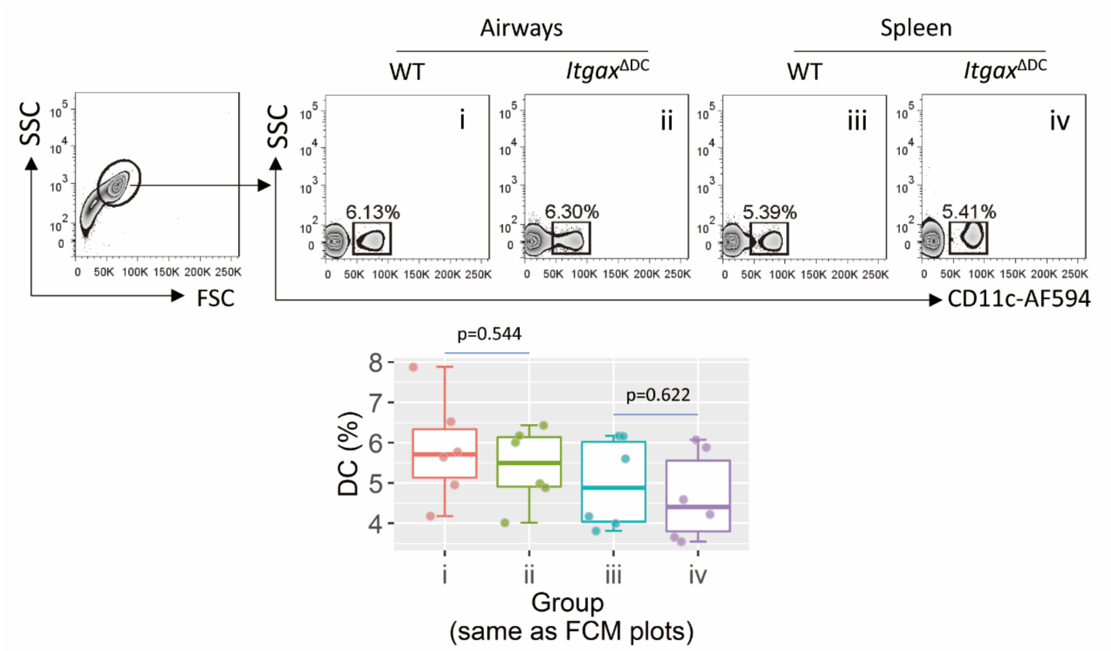

Supplementary Figure 2. Assessment of DCs in the airways and spleen of *Itgax*$^{\Delta DC}$ mice. AMCs and spleen cells were prepared from *Itgax*$^{\Delta DC}$ mice and WT mice. Cells were analyzed by FCM. Gated FCM plots show DC counts. Boxplots show DC frequency of 6 mice per group. The data of boxplot are presented as median (middle line inside box), 75% value (upper bound of box), 25% value (lower bound of box), max (upper whisker), and min (lower whisker). Each dot in boxplot presents data obtained from one sample.

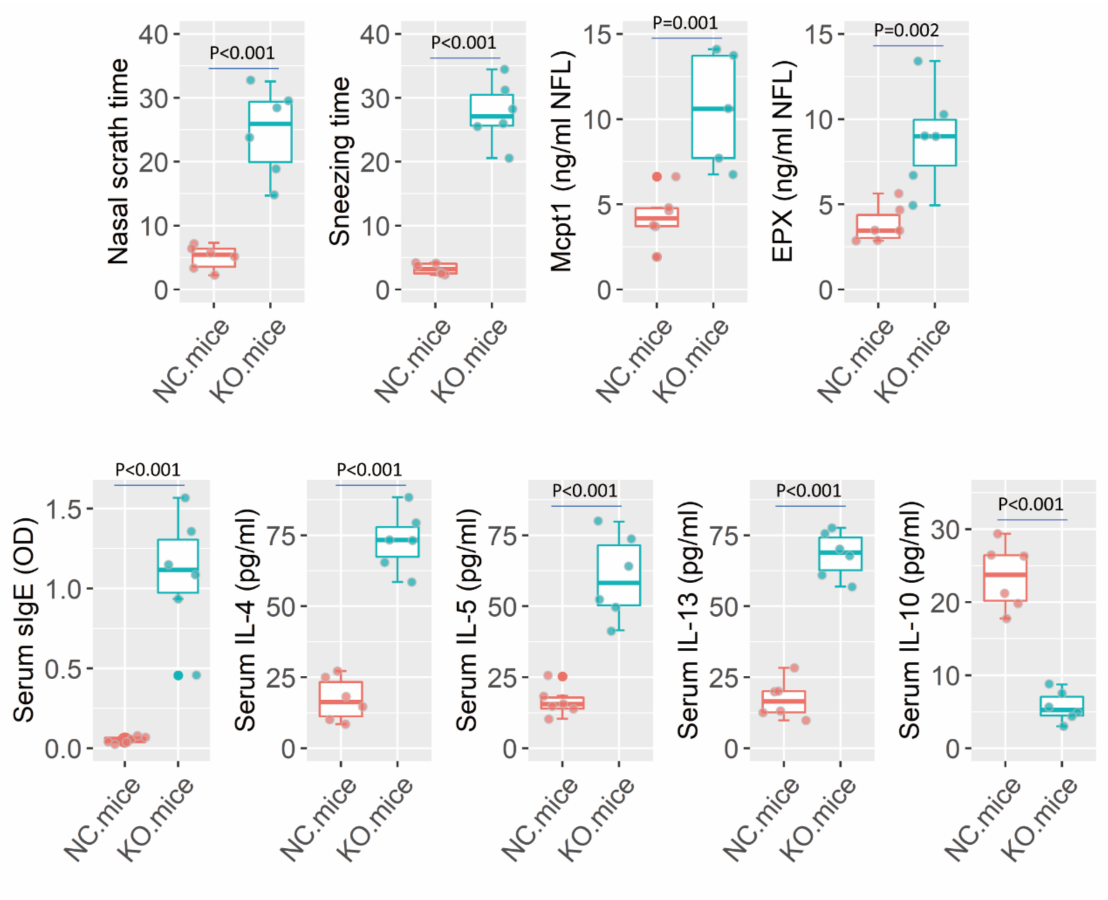

Supplementary Figure 3. Assessment of AR response in *$Fpr1^{\Delta DC}$* mice. An AR mouse model was developed with WT mice (the NC group in figure) and *$Fpr1^{\Delta DC}$* mice (KO mice) were sensitized to OVA as indicated in Fig. S1. Boxplots show the AR response, including AR-like clinical response records (nasal itch and sneezing), allergic mediators (Mcpt1 and EPX) in NLF, serum sIgE levels, serum Th2 cytokine and IL-10 levels. Mcpt1: Mast cell protease-1. EPX: Eosinophil peroxidase. NLF: Nasal lavage fluids. The data of boxplot are presented as median (middle line inside box), 75% value (upper bound of box), 25% value (lower bound of box), max (upper whisker), and min (lower whisker). Each dot in boxplot presents data obtained from one sample. Each group consists of 6 mice.

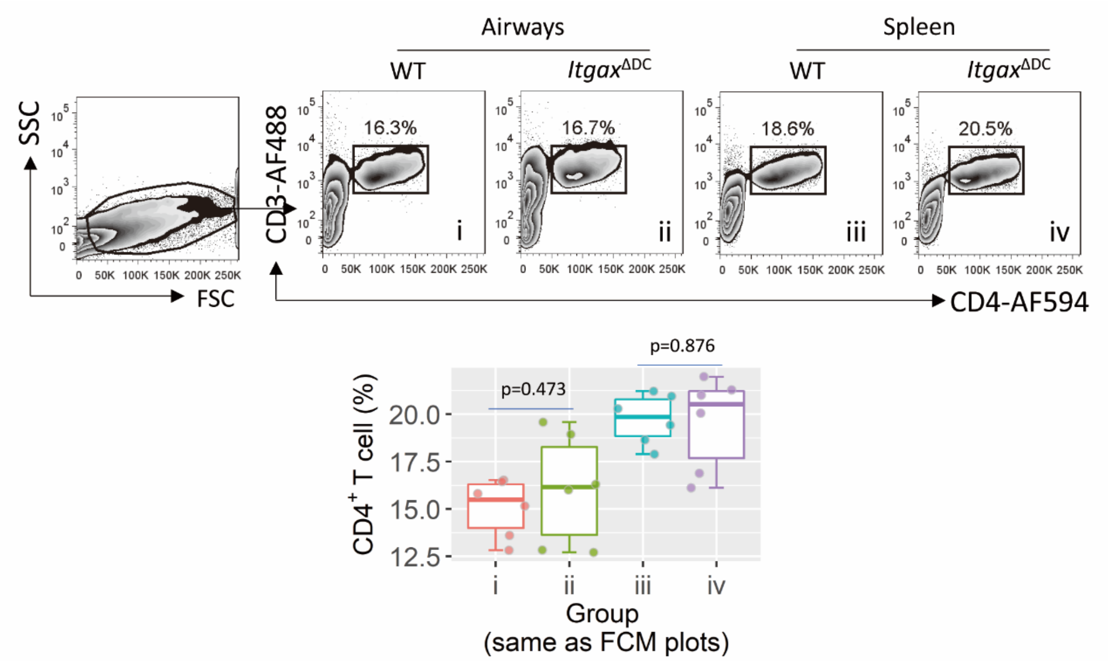

Supplementary Figure 4. Assessment of CD4⁺ T cells in the airways and spleen of *Il10rb*$^{\Delta Cd4tc}$ mice. AMCs and spleen cells were prepared from *Il10rb*$^{\Delta Cd4tc}$ mice and WT mice. Cells were analyzed by FCM. Gated FCM plots show $CD4^+$ T cell counts. Boxplots show $CD4^+$ T cell frequency of 6 mice per group. The data of boxplot are presented as median (middle line inside box), 75% value (upper bound of box), 25% value (lower bound of box), max (upper whisker), and min (lower whisker). Each dot in boxplot presents data obtained from one sample.

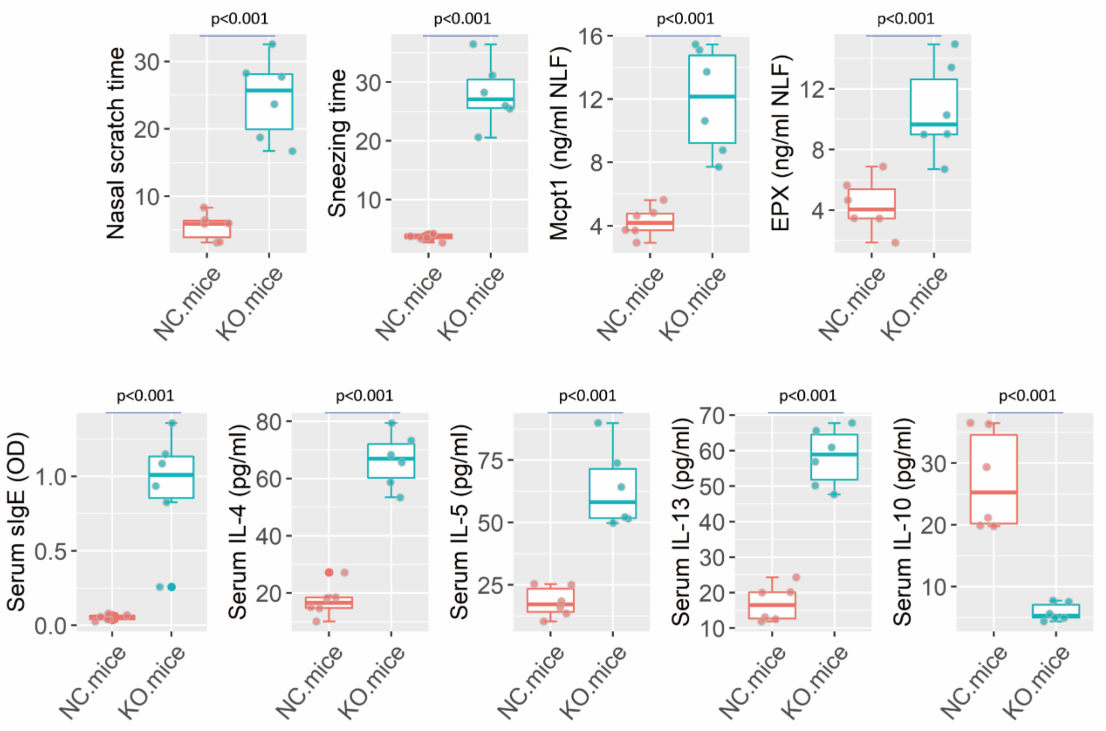

Supplementary Figure 5. Assessment of AR response in *Il10rb*$^{\Delta Cd4tc}$ mice. An AR mouse model was developed with WT mice (the NC group in figure) and *Il10rb*$^{\Delta Cd4tc}$

mice (KO mice) were sensitized to OVA as indicated in Fig. S1. Boxplots show the AR response, including AR-like clinical response records (nasal itch and sneezing), allergic mediators (Mcpt1 and EPX) in NLF, serum sIgE levels, serum Th2 cytokine and IL-10 levels. Mcpt1: Mast cell protease-1. EPX: Eosinophil peroxidase. NLF: Nasal lavage fluids. The data of boxplot are presented as median (middle line inside box), 75% value (upper bound of box), 25% value (lower bound of box), max (upper whisker), and min (lower whisker). Each dot in boxplot presents data obtained from one sample. Each group consists of 6 mice.

Raw data of immunoblots

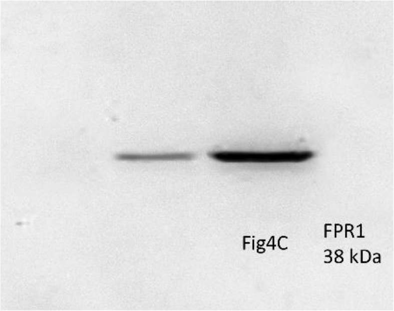

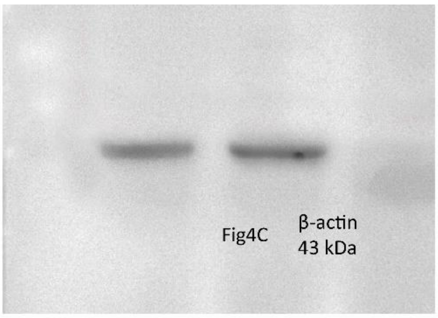

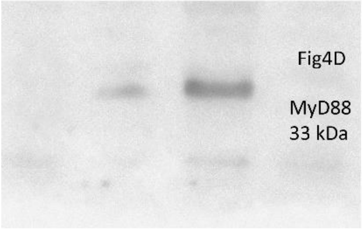

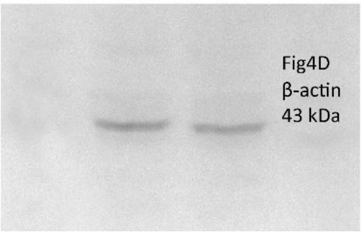

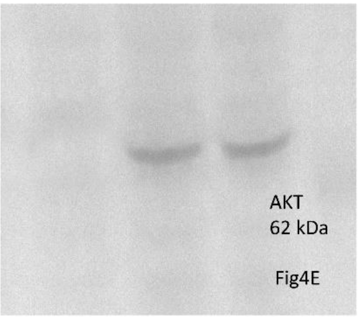

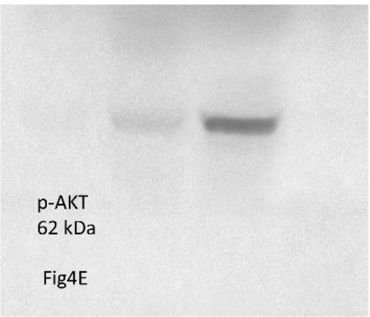

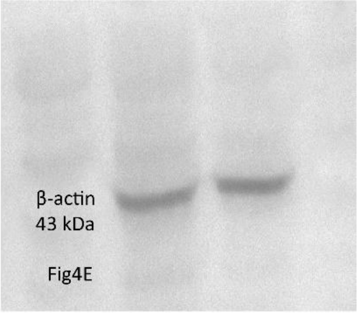

Supplement: Supplementary file 2 — Supplemental materials [file 41541_2022_559_MOESM2_ESM.pdf]
